# Therapeutic efficacy of cell-based therapy in vitiligo: a research letter systematically reviewed using meta-analysis

**DOI:** 10.1007/s00403-024-02920-6

**Published:** 2024-05-22

**Authors:** Xue Wang, Hai Ci, Cheng Chen, Songen Chen, Hanluo Li, Bin Xie, Simin Li, Yongsheng Li, Weishan Wang

**Affiliations:** 1 Dermatology Department, First Affiliated Hospital of Shihezi University, Xinjiang, 832008 China; 2 Department of Burn and Plastic Surgery, First Affiliated Hospital of Shihezi University, Xinjiang, 832008 China; 3 National “111” Center for Cellular Regulation and Molecular Pharmaceutics, Cooperative Innovation Center of Industrial Fermentation (Ministry of Education and Hubei Province), Hubei University of Technology, Wuhan, 430068 China; 4 Zhejiang Lab, Kechuang Avenue, Zhongtai Sub-District, Yuhang District, Hangzhou, 311121 Zhejiang China; 5 Stomatological Hospital, School of Stomatology, Southern Medical University, 366 Jiangnan South Avenue, Haizhu District, Guangzhou, 510280 Guangdong China; 6 School of Chemistry and Chemical Engineering, Pharmacy School, State Key Laboratory Incubation Base for Green Processing of Chemical Engineering, Shihezi University, Xinjiang, 832003 China; 7 Department of Orthopedics Center, First Affiliated Hospital of Shihezi University, Xinjiang, 832008 China

Dear Editors,

Vitiligo is an auto-immune intractable disorder characterized by the loss of functioning epidermal melanocytes, which is treated either conservatively or surgically. When vitiligo becomes refractory to medical treatment and stable, surgical techniques using cell therapies become an important alternative [1]. The rational basis of surgical methods is the transfer of melanocytes from uninvolved skin to the stable vitiligo patch in the form of either a tissue graft or a cellular graft, including epidermal blister grafts, split-thickness grafts, and full-thickness punch grafts [2]. Cellular grafting includes transplantation of cultured pure melanocytes, co-cultured melanocyte–keratinocyte cell suspensions, cultured epidermal cell suspensions, Non-Cultured Epidermis-derived Cell (NCEC) suspensions and Non-Cultured Extracted Hair Follicle Outer Root Sheath Cell Suspensions (NCFCS) [3].

To estimate the treatment outcome of various types of cellular transfer therapy (epidermal cell suspension, melanocyte suspension, extracted hair follicle root sheath cell suspension) in stable vitiligo, we are systematically meta-analysis the treatments to resolve discrepancies of these results. We searched 4 electronic databases (PubMed, EMBASE, Cochrane, and NCBI) with reports of randomized- and non-randomized controlled trials (RCT and non-RCT) in cellular transfer for the treatment of stable vitiligo (Fig. 1). The meta-analysis included 17 RCT studies, 7 non-RCTs, and 4 comparisons.

**Fig. 1:**
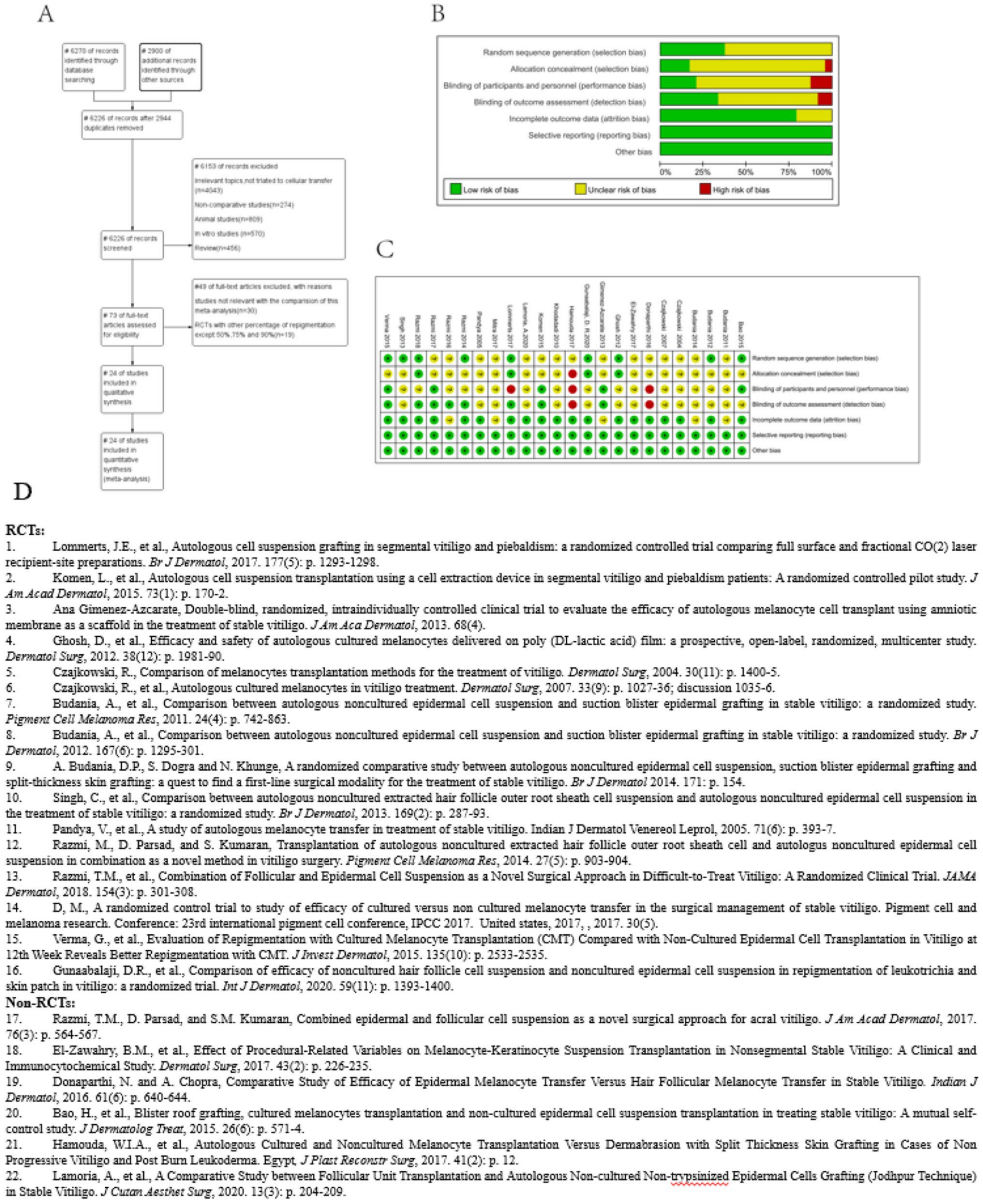
Meta-analysis of 22 studies involving 592 unique patients including 16 RCTs and 6 non-RCTs. **A** Flow diagram of the meta-analysis study selection process. **B** Risk of bias summary of all included RCTs. **C** Risk-of-bias graph of all included RCTs: comparison of epidermis-derived cellular transfer and placebo/no treatment control. **D** List of 22 studies with 16 RCTs and 6 non-RCTs

It was found that there was no significant difference between epidermal tissue grafting(ETG) and NCES in the outcomes of ≥ 75% repigmentation (RR = 1.18, 95% CI 0.93, 1.49) and ≥ 90% repigmentation (RR = 1.06, 95% CI 0.92, 1.23) (Fig. 2). Epidermal melanocyte transplantation is not an inferior alternative to the conventional ETG, which is more suitable for treatments of the larger depigmented areas. No significant difference was demonstrated between cultured epidermis-derived cells and NCEC suspension transfer in the outcome of ≥ 50% repigmentation (RR = 2.23, 95% CI 0.61, 8.08) and ≥ 90% repigmentation (RR = 0.98, 95% CI 0.80, 1.21). Therefore, for the small lesion area, NCEC suspension treatment was recommended. No significant difference was found between NCES and NCFCS in the outcome of ≥ 75% repigmentation (RR = 1.26, 95% CI 0.97, 1.64) and ≥ 90% repigmentation (RR = 1.43, 95% CI 0.91, 2.26); but NCEC is better than NCFSC in repigmentation and healing time (*P* = 0.02). Cultured hair follicle root sheath cell suspension seems to be a promising technique to replace conventional epidermal cellular transfer. Combining the two together (NCES + NCFCS) displayed better efficacy than NCES alone in the outcome of ≥ 75% repigmentation (RR = 1.34, 95% CI 1.09, 1.65) and ≥ 90% repigmentation (RR = 1.99, 95% CI 1.32, 2.99), shown that FCS combined with NCEC is significantly superior to the NCEC alone [4].

**Fig. 2:**
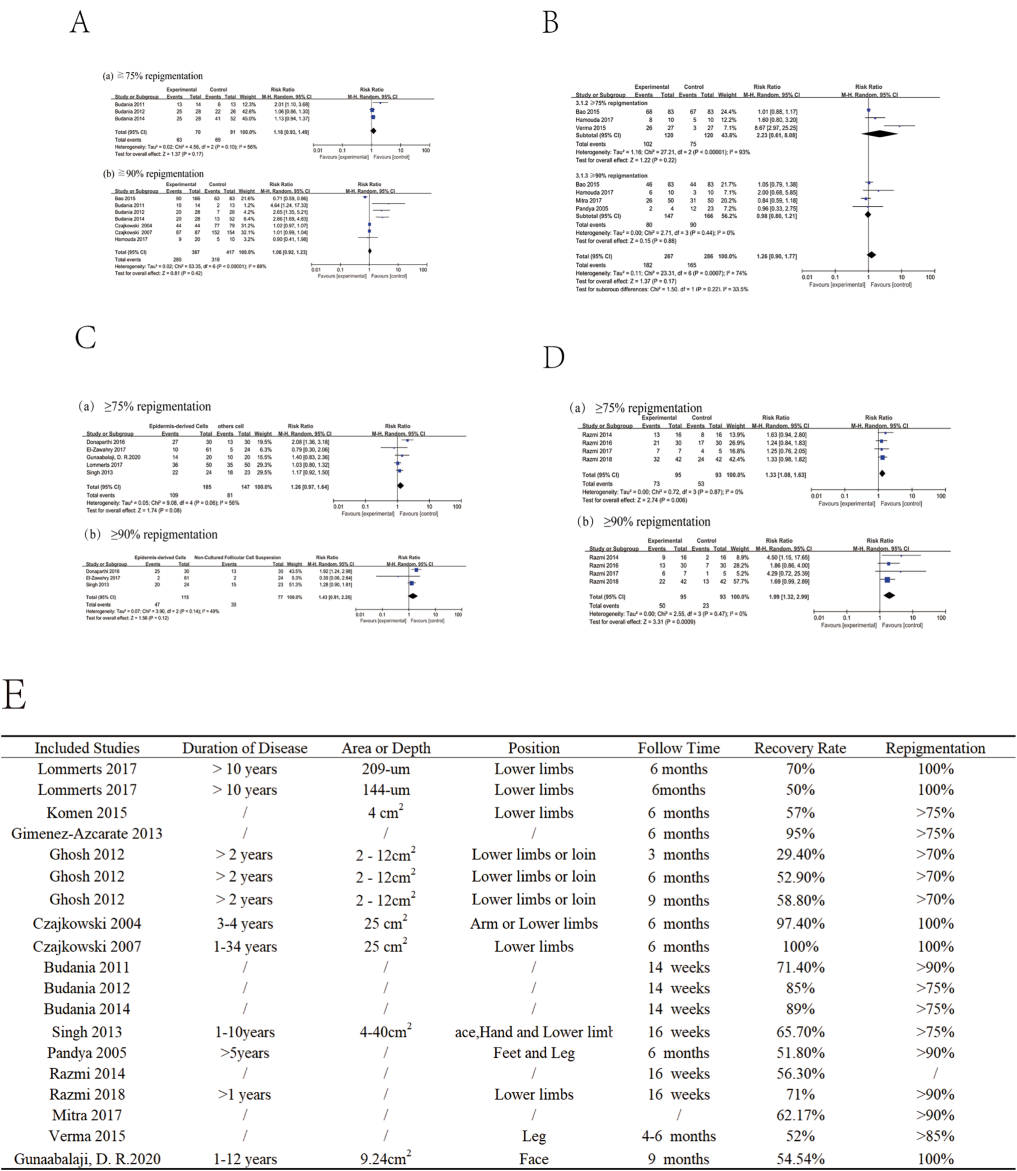
Meta-analysis of forest plots using various cellular transplantation with clinical effects over 75% repigmentation and over 90% repigmentation. **A** Comparison of ETG and NCEC. **B** Comparison of Cultured Epidermis-derived Cells and NCEC transfer. **C** Comparison of NCEC suspension and NCFCS. **D** Comparison between NCFCS + NCEC and NCES alone. **E** Table of the vitiligo patient information including duration of disease and anatomical sites, etc. With “/” representing no dates

In conclusion, cellular transfer could be an alternative method to epidermal tissue grating. More RCTs with high-quality, large sample size, longer follow-up, and consistent repigmentation scoring system should be performed, and an agreed scaling system of outcome measures for repigmentation should be established in the future [5]. The combination of epidermis-derived cells and follicular cells could be a promising trend for treating stable vitiligo.

### Supplementary Information

Below is the link to the electronic supplementary material.

Supplementary file1 (ZIP 24195 KB)

## Data Availability

The data supporting this study are available on request from the corresponding author, WS Wang.
