# Supplementary material for: Therapeutic efficacy of cell-based therapy in vitiligo: a research letter systematically reviewed using meta-analysis
Source: Arch Dermatol Res. 2024 May 22;316(5):198. doi: 10.1007/s00403-024-02920-6 (PMC11111487; doi:10.1007/s00403-024-02920-6)
Supplement: Supplementary file 1 — Supplementary file1 (ZIP 24195 KB) [file 403_2024_2920_MOESM1_ESM.zip › Studies were included/Bao 2015.pdf]

This article was downloaded by: [University of Manitoba Libraries]
On: 03 September 2015, At: 02:52
Publisher: Taylor & Francis
Informa Ltd Registered in England and Wales Registered Number: 1072954 Registered office: 5 Howick Place, London, SW1P 1WG

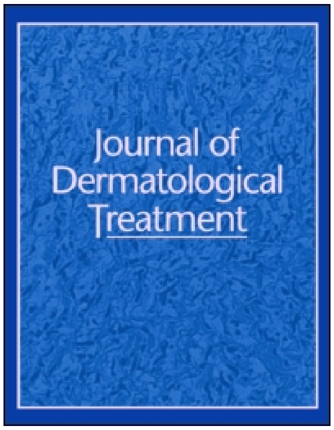

Click for updates

Journal of Dermatological Treatment
Publication details, including instructions for authors and subscription information:
http://www.tandfonline.com/loi/ijdt20

Blister roof grafting, cultured melanocytes transplantation and non-cultured epidermal cell suspension transplantation in treating stable vitiligo: A mutual self-control study

Huaye Bao[a], Weisong Hong[a], Lifang Fu[a], Xiaodong Wei[a], Guopei Qian[a] & Aie Xu[a]
[a] Department of Dermatology, Third People's Hospital of Hangzhou, Hangzhou Institute of Dermatology and Venereology, Hangzhou, Zhejiang, China
Published online: 06 Aug 2015.

To cite this article: Huaye Bao, Weisong Hong, Lifang Fu, Xiaodong Wei, Guopei Qian & Aie Xu (2015): Blister roof grafting, cultured melanocytes transplantation and non-cultured epidermal cell suspension transplantation in treating stable vitiligo: A mutual self-control study, Journal of Dermatological Treatment

To link to this article: http://dx.doi.org/10.3109/09546634.2015.1034068

PLEASE SCROLL DOWN FOR ARTICLE

Taylor & Francis makes every effort to ensure the accuracy of all the information (the "Content") contained in the publications on our platform. However, Taylor & Francis, our agents, and our licensors make no representations or warranties whatsoever as to the accuracy, completeness, or suitability for any purpose of the Content. Any opinions and views expressed in this publication are the opinions and views of the authors, and are not the views of or endorsed by Taylor & Francis. The accuracy of the Content should not be relied upon and should be independently verified with primary sources of information. Taylor and Francis shall not be liable for any losses, actions, claims, proceedings, demands, costs, expenses, damages, and other liabilities whatsoever or howsoever caused arising directly or indirectly in connection with, in relation to or arising out of the use of the Content.

This article may be used for research, teaching, and private study purposes. Any substantial or systematic reproduction, redistribution, reselling, loan, sub-licensing, systematic supply, or distribution in any form to anyone is expressly forbidden. Terms & Conditions of access and use can be found at http://www.tandfonline.com/page/terms-and-conditions

Journal of Dermatological Treatment

http://informahealthcare.com/jdt
ISSN: 0954-6634 (print), 1471-1753 (electronic)

J Dermatolog Treat, Early Online: 1–4
© 2015 Taylor & Francis. DOI: 10.3109/09546634.2015.1034068

Taylor & Francis
Taylor & Francis Group

Downloaded by [University of Manitoba Libraries] at 02:52 03 September 2015

ORIGINAL ARTICLE

Blister roof grafting, cultured melanocytes transplantation and non-cultured epidermal cell suspension transplantation in treating stable vitiligo: A mutual self-control study

Huaye Bao, Weisong Hong, Lifang Fu, Xiaodong Wei, Guopei Qian, and Aie Xu

Department of Dermatology, Third People's Hospital of Hangzhou, Hangzhou Institute of Dermatology and Venereology, Hangzhou, Zhejiang, China

Abstract

Objectives: To compare the efficacy of blister roof grafting (BG), cultured melanocytes transplantation (CMT) and non-cultured epidermal cell suspension transplantation (NCES) in the treatment of stable vitiligo. *Methods*: In each person of 83 vitiligo patients one vitiligo macule was selected and divided in three areas for separate treatment with BG, CMT and NCES in the same session. The results were evaluated 12-month post-surgery for the extent of repigmentation and color match. *Results*: A satisfactory result (>50% repigmentation) was achieved in 92%, 82% and 81% of the 83 patients with the BG, CMT and NCES methods, respectively. Significant differences between the BG and CMT groups ($p = 0.038$), and between BG and NCES groups ($p = 0.017$) were observed, but not between the CMT and NCES groups ($p = 0.986$). The extent of repigmentation on the head neck and trunk was superior to that of the extremities by all the three methods. A difference in the time of onset of repigmentation was observed, with repigmentation first appearing after 10 days, 20–30 days and >30 days in the BG, CMT and NCES groups, respectively. *Conclusions*: All the three methods are safe and effective to treat vitiligo. Future studies with larger groups are warranted to confirm our results.

Keywords

BG, CMT, NCES, vitiligo

History

Received 28 February 2014
Revised 8 March 2015
Accepted 8 March 2015
Published online 6 August 2015

Introduction

Vitiligo is an acquired progressive disorder characterized by white macules resulting from a loss of functioning melanocytes (1). There are several therapeutic alternatives for vitiligo, including topical and oral agents, phototherapy, laser and surgical procedures (2). In stable refractory vitiligo, surgical treatments are often satisfactory to improve repigmentation. These can be divided into two groups, cellular and tissue grafting. Cellular grafting includes transplantation of non-cultured epidermal cell suspension (NCES) and transplantation of cultured pure melanocytes among others, whereas tissue grafting includes punch, split-thickness and blister roof grafting (BG) (3).

Herein, we report on 83 vitiligo patients treated with BG, cultured melanocytes transplantation (CMT) and NCES at the same time and area.

Patients and methods

The study was approved by the Institute's Ethics Committee, and all the patients provided written consent.

Correspondence: Aie Xu, Department of Dermatology, Third People's Hospital of Hangzhou, Hangzhou Institute of Dermatology and Venereology, 38 Xi Hu Da Dao, 310009 Hangzhou, Zhejiang, China. Tel: +86 571 87827535. Fax: +86 571 87827535. E-mail: xuaiehz@msn.com

Patients

Patients attending the clinic of the Department of Dermatology, the Third People's Hospital of Hangzhou between March 2011 and March 2013, with a clinical diagnosis of vitiligo that had been stable for at least 1 year, were eligible for inclusion in the study. Patients with a history of bleeding diathesis or koebnerization, and pregnant women were excluded. The size of the treated areas ranged between 2–10 cm^2 (mean ± SD 5.49 ± 1.79), 40–80 cm^2 (mean ± SD 59.77 ± 12.96) and 20–40 cm^2 (mean ± SD 31.72 ± 5.21) for the BG, cultured melanocytes and NCES methods, respectively. The demographic and clinical data of all the participants are presented in Table 1.

Procedure

Donor sites

Donor skin was obtained from normally pigmented areas of the patients' abdomen. Blisters (8 mm in diameter) were produced with a vacuum of 40 kPa for 60–90 min. The roofs of the blisters were cut and placed in a vial with calcium-free Hanks' solution.

Cell culture

All the culture medium, supplements and methods for isolation and cultivation of cells from the specimens have been previously described (4). After the cell number had met the requirement for transplantation, the melanocytes were dissociated, centrifuged and resuspended.

Downloaded by [University of Manitoba Libraries] at 02:52 03 September 2015

Recipient site

The recipient sites were allocated to three areas: one for each treatment. By consulting previous publications (5,6), we defined the donor site to recipient skin size ratio (DR ratio) as 1:1, 1:20 and 1:5 for BG, CMT and NCES methods, respectively.

Lidocaine cream 5% was applied 2–3 h pre-transplantation. The recipient areas were cleaned with 70% alcohol and treated with an ultrapulse CO_2 laser (pulse rate 30–50 Hz; energy level, 225 mJ/pulse) to remove the epidermis.

Blister roof grafting

The roofs of the blisters at the donor sites were cut and transferred to the recipient areas.

Cultured melanocytes transplantation

The melanocyte suspension was applied to the recipient areas at a density of 600–1000 cells mm^{-2}.

Non-cultured epidermal cell suspension transplantation

The roofs of the blisters were collected at the donor sites. Methods for the isolation and cultivation of cells from the specimens have been previously described (6). The NCES was carefully transferred to the recipient site.

Post-surgery care

The transplant areas were covered with Vaseline gauze and gauze soaked with F12 medium and secured with gauze and surgical tape. The dressings were removed 10-day post-surgery.

Table 1. The demographic and clinical data of all the participants.

Number of patients	83
Age (years), mean ± SD	25.2 ± 10.5
Sex (male/female)	38/45
Type of vitiligo (segmental/non-segmental)	40/43
Period of stability (months), mean ± SD	45.2 ± 50.3
Body surface area involved, mean ± SD	4.6 ± 2.2
Location: Head Neck/Trunk/Extremities	33/26/24

Data are mean ± SD unless otherwise stated.

Evaluation of the treatment efficacy

Two separate certified dermatologists were responsible for the clinical evaluation and follow-up of the patients. Evaluation of repigmentation was performed 2-, 3-, 6- and 12-month post-transplantation. Photographs were taken before and 12-month post-transplantation. Repigmentation was assessed subjectively by digital photography as follows: excellent, ≥90%; good, 50–89%; fair, 20–49% and poor, <20% (7).

Statistics

SPSS version 17.0 (SPSS Inc., Chicago, IL) was used for all the analyses. Comparisons of the different groups were performed using the chi-squared tests. A p value <0.05 was considered significant.

Results

Treatment efficiency

Excellent repigmentation was observed in 76%, 55% and 53% of patients treated with the BG, CMT and NCES methods, respectively. Of all the patients, 92%, 82% and 81% obtained a satisfactory result (>50% repigmentation) with the BG, CMT and NCES methods, respectively (Figure 1). Differences were observed between the BG and CMT methods ($p = 0.038$), and the BG and NCES methods ($p = 0.017$), but not between the CMT and NCES methods ($p = 0.986$) (Table 2 and Figure 2).

Treatment efficacy on different locations of vitiligo lesions

The extent of repigmentation in the head neck and trunk was better than that in the extremities with all the three transplantations methods. In the BG group, excellent repigmentation was achieved in 30/33 patients (91%) with vitiligo on the head and neck, compared with 21/26 patients (81%) with vitiligo on the trunk, and 12/24 patients (50%) with vitiligo on the extremities. The percentage of repigmentation differed between the extremities and the head/neck ($p = 0.003$), and the extremities and trunk ($p = 0.037$), but not between the head/neck and the trunk ($p = 0.284$) in this group. Differences were observed between the extremities and the head/neck, and between the extremities and trunk, but not between the head/neck and trunk in the CMT and NCES groups (Table 3).

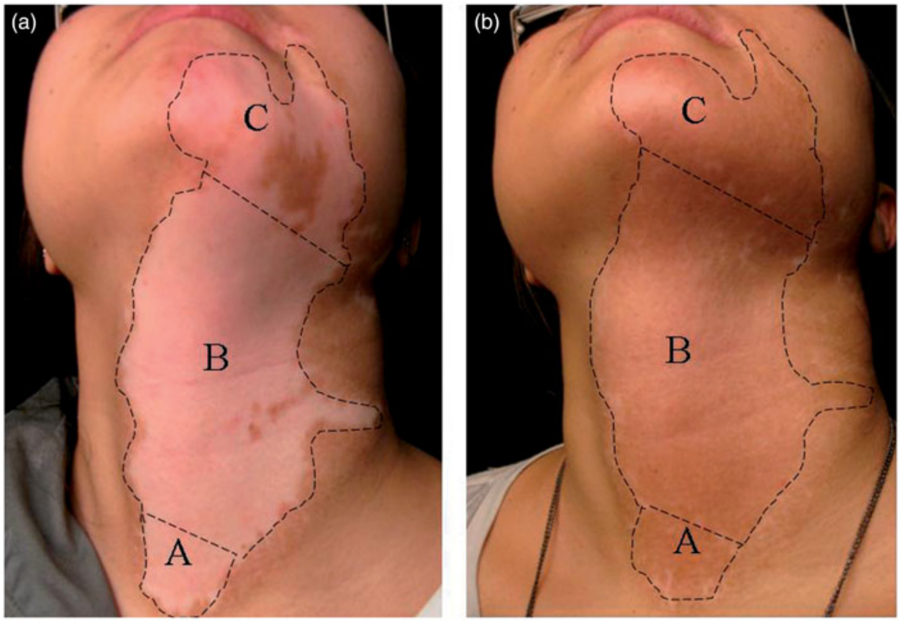

Figure 1. Intra-patient comparison of the results of treatment with three transplantation methods of a vitiligo area situated at the neck and the chin. (A) is for BG, (B) is for cultured melanocytes transplant and (C) is for NCES. (a) Before surgery and (b) 12 months after surgery showing >90% repigmentation.

No differences in the acral lesions were observed in any group: 74%, 58% and 58% had a satisfactory result (>50% repigmentation) with the BG, CMT and NCES methods, respectively (CMT versus NCES $p = 0.977$; BG versus CMT $p = 0.350$; BG versus NCES $p = 0.330$).

Color match of the recipient site

Difference in the time of onset of repigmentation were observed among these groups, with repigmentation first appearing after 10, 20–30 and >30 days in the BG, CMT and NCES groups, respectively. Nearly uniform color was achieved in the recipient sites by the CMT and NCES methods. However, by the BG method, we observed 22 cases with hyperpigmentation, which required >6 months to fade, in order to match the normal skin color.

Adverse effects

None of the patients developed infection, milia or visible scarring at any donor or recipient site.

Table 2. Outcome of three different transplantation methods.

Results	BG[a] *n* (%)	CMT[b] *n* (%)	NCES[c] *n* (%)
Excellent	63 (76%)	46 (55%)	44 (53%)
Good	14 (16%)	22 (27%)	23 (28%)
Fair	3 (4%)	9 (11%)	9 (11%)
Poor	3 (4%)	6 (7%)	7 (8%)

p Value: $p$ab = 0.038, $p$bc = 0.986, $p$ac = 0.017. Significant p value <0.05.

Discussion

Vitiligo represents a common cosmetic problem, and current non-surgical modalities for vitiligo are not always satisfactory (8). Accordingly, surgery is indicated for all the types of stable vitiligo that do not respond well to medical therapy.

We here evaluated the efficacy of three transplantation methods: BG, CMT and NCES. All the three transplantation methods were performed on each patient at the same time and area. Of the 83 patients, 92%, 82% and 81% obtained a satisfactory result (>50% repigmentation) with the BG, CMT and NCES methods, respectively. Moreover, the recipient site had a considerable influence on the repigmentation results, with repigmentation on the head/neck and trunk being better than that of the extremities. Different repigmentation rates were observed in different locations by different methods. Lesions on the extremities are usually more resistant to medical treatment, possibly due to the minimal density of the hair follicles acting as a melanocyte reservoir, the relatively low melanocyte density or the enhanced risk of repeated friction or trauma to the extremities compared to the head, neck or trunk (9).

BG is useful for treating small lesions (10). Gupta et al. (11) treated 10 patients with BG and found that 13 of the 15 patches in 8 of 10 patients showed >75% repigmentation. In our study, BG was superior to the other two transplantation methods. Many comparison studies on grafting techniques have shown that maximum repigmentation is achieved with either split-thickness grafting or suction blister grafting (12). However, in these techniques, the DR ratio is 1:1. Hence, only small areas can be treated per session, and we therefore recommend treating large vitiliginous areas with cellular transplantation.

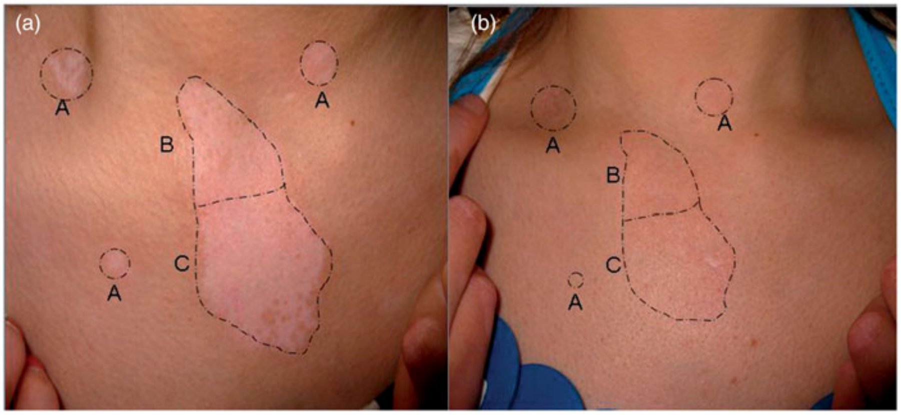

Figure 2. Intra-patient comparison of the results of treatment with three transplantation methods of a vitiligo area situated at the chest. (A) is for BG, (B) is for cultured melanocytes transplant and (C) is for NCES. (a) Before surgery and (b) 12 months after surgery showing >90% repigmentation.

Table 3. Outcome after 12 months of three different transplantation methods at different body sites.

Position	Excellent *n* (%)	Good *n* (%)	Fair *n* (%)	Poor *n* (%)	*p* Value
BG					
Head Neck[a]	30 (91%)	3 (9%)	0	0	$p$ab=0.284
Trunk[b]	21 (81%)	5 (19%)	0	0	$p$bc=0.037
Extremities[c]	12 (50%)	6 (24%)	3 (13%)	3 (13%)	$p$ac=0.003
CMT					
Head Neck[a]	24 (73%)	6 (18%)	2 (6%)	1 (3%)	$p$ab=0.555
Trunk[b]	16 (62%)	8 (31%)	2 (7%)	0	$p$bc=0.013
Extremities[c]	6 (25%)	8 (33%)	5 (21%)	5 (21%)	$p$ac=0.003
NCES					
Head Neck[a]	22 (67%)	10 (30%)	1 (3%)	0	$p$ab=0.199
Trunk[b]	16 (62%)	5 (19%)	4 (15%)	1 (4%)	$p$bc=0.033
Extremities[c]	6 (25%)	8 (33%)	4 (17%)	6 (25%)	$p$ac=0.001

Significant p value <0.05.

Downloaded by [University of Manitoba Libraries] at 02:52 03 September 2015

Downloaded by [University of Manitoba Libraries] at 02:52 03 September 2015

Transplantation of cultured melanocytes is a well-established procedure to treat vitiligo (13,14). In a study by Hong et al., 102 patients with stable vitiligo were divided into low (DR ratio <1:10) and high DR ratio groups (DR ratio 1:10–1:60) and treated by this method. However, no difference was observed between two groups in terms of repigmentation (2). In our study, there is no significant difference between two CMT and NCES.

NCES, which was first introduced by Gauthier and Surleve-Bazeille (15), has been demonstrated to represent an effective treatment for vitiligo. NCES allows transplantation to a recipient area larger than the donor area, with the reported DR ratios ranging from 1:3 to 1:10 (16,17). This method requires a well-equipped laboratory and trained laboratory personnel, and is more complicated than skin grafting, but still relatively simple and inexpensive, compared to CMT.

Thus, we here chose the different transplantation methods depending on the sizes of the lesions: the BG, NCES and CMT methods are recommended for small ($\leq 10\,cm^2$), intermediate ($10–30\,cm^2$) and large ($>30\,cm^2$) vitiliginous areas, respectively.

In conclusion, we found that all the methods were safe and effective. Further large-scale, double-blinded studies on vitiligo transplantation methods are needed in order to determine the most efficacious and safe techniques.

Declaration of interest

The authors report no conflicts of interest. The authors alone are responsible for the content and writing of the article.

References

1. Guerra L, Dellambra E, Brescia S, Raskovic D. Vitiligo: pathogenetic hypotheses and targets for current therapies. Curr Drug Metab. 2010;11:451–67.
2. Ramos MG, Ramos DG, Gontijo G, et al. Non-cultured melanocyte/keratinocyte transplantation for the treatment of stable vitiligo on the face: report of two cases. An Bras Dermatol. 2013;88:811–13.
3. Mulekar SV, Isedeh P. Surgical interventions for vitiligo: an evidence-based review. Br J Dermatol. 2013;169:57–66.
4. Hong WS, Hu DN, Qian GP, et al. Ratio of size of recipient and donor areas in treatment of vitiligo by autologous cultured melanocyte transplantation. Br J Dermatol. 2011;165:520–5.
5. Flabella R, Barona MI. Update on skin repigmentation therapies in vitiligo. Pigment Cell Melanoma Res. 2009;22:42–65.
6. Holla AP, Kumar R, Parsad D, Kanwar AJ. Modified procedure of noncultured epidermal suspension transplantation: changes are the core of vitiligo surgery. J Cutan Aesthet Surg. 2011;4:44–5.
7. Chen YF, Yang PY, Hu DN, et al. Treatment of vitiligo by transplantation of cultured pure melanocyte suspension: analysis of 120 cases. J Am Acad Dermatol. 2004;51:68–74.
8. Njoo MD, Westerhof W, Bos JD, et al. The development of guidelines for the treatment of vitiligo. Arch Dermatol. 1999;135:1514–21.
9. Holla AP, Parsad D. Vitiligo surgery: its evolution as a definite treatment in the stable vitiligo. G Ital Dermatol Venereol. 2010;145:79–88.
10. Ko WC, Chen YF. Suction blister epidermal grafts combined with CO_2 laser superficial ablation as a good method for treating smallsized vitiligo. Dermatol Surg. 2009;35:601–6.
11. Gupta S, Kumar B. Epidermal grafting for vitiligo in adolescents. Pediatr Dermatol. 2002;19:159–62.
12. Njoo MD, Westerhof W, Bos JD, Bossuyt PM. A systematic review of autologous transplantation methods in vitiligo. Arch Dermatol. 1998;134:1543–9.
13. Olsson MJ, Juhlin L. Transplantation of melanocytes in vitiligo. Br J Dermatol. 1995;132:587–91.
14. Olsson MJ, Juhlin L. Melanocyte transplantation in vitiligo. Lancet 1992;340:981.
15. Gauthier Y, Surleve-Bazeille J. Autologous grafting with noncultured melanocytes: a simplified method for treatment of depigmented lesions. J Am Acad Dermatol. 1992;26:191–4.
16. Mulekar SV. Long-term follow-up study of segmental and focal vitiligo treated by autologous, noncultured melanocyte-keratinocyte cell transplantation. Arch Dermatol. 2004;140:1211–15.
17. Mulekar SV, Al Eisa A, Delvi MB, et al. Childhood vitiligo: a longterm study of localized vitiligo treated by noncultured cellular grafting. Pediatr Dermatol. 2010;27:132–6.
