# Supplementary material for: Therapeutic efficacy of cell-based therapy in vitiligo: a research letter systematically reviewed using meta-analysis
Source: Arch Dermatol Res. 2024 May 22;316(5):198. doi: 10.1007/s00403-024-02920-6 (PMC11111487; doi:10.1007/s00403-024-02920-6)
Supplement: Supplementary file 1 — Supplementary file1 (ZIP 24195 KB) [file 403_2024_2920_MOESM1_ESM.zip › Studies were included/Budania 2011.pdf]

Pigment Cell Melanoma Res. **24**; 742–863

doi: 10.1111/j.1755-148x.2011.00885.x

XXIst International Pigment Cell Conference (IPCC) "Skin and Other Pigment Cells: Bridging Clinical Medicine and Science" Organized by the European Society for Pigment Cell Research and the Department of Dermatology and Pediatric Dermatology, University of Bordeaux

20–24 September 2011
Palais des Congrès, Bordeaux, France

Organizing Committee

Honorary Presidents:
Yvon Gauthier, Jean-Paul Ortonne

President:
Alain Taïeb

Vice-Presidents:
Lionel Larue, Mauro Picardo, Lluís Montoliu

Local Secretariat:
Muriel Cario-André, Khaled Ezzedine, Thomas Jouary, Fréderic Mazurier, Hamid Rezvani

Local Advisory Committee:
Benoït Arveiler, Didier Lacombe, Djavad Mossalayi, François Tison, Béatrice Vergier, Hubert de Verneuil

ESPCR Advisory Committee:
Dorothy Bennett, Markus Böhm, Jose Carlos Garcia-Borron, Colin Goding, Marco d'Ischia

National Committee:
Marie-Françoise Avril, Robert Ballotti, Nicole Basset-Seguin, Brigitte Dreno, Heather Etchevers, Marie-Dominique Galibert, Jean-Jacques Grob, Bernard Guillot, Jean-Philippe Lacour, Celeste Lebbé, Alain Mauviel, Thierry Passeron, Philippe Saiag, Alain Sarasin, Nadem Soufir, Luc Thomas

International Programme Committee:
Zalfa Abdel Malek, Nawaf Al-Mutairi, Heintz Arnheiter, Greg Barsh, David Fisher, Claus Garbe, Nicholas Hayward, Vincent Hearing, Meenhard Herlyn, Soshuke Ito, Prasad Kumarasinghe, Takahiro Kunisada, Mu-Hyoung Lee, Caroline Le Poole, Glen Merlino, Frank Meyskens, Shin Ishi Nishikawa, David Norris, Kyoung-Chan Park, Davinder Parsad, John Pawelek, Ze'ev Ronai, John Simon, Shigeki Shibahara, Andrzej Slominski, Richard Spritz, Yasushi Tomita, Hiroaki Yamamoto

Melanoma Day Committee:
Boris Bastian, David Fisher, Claus Garbe, Ghanem Ghanem, Nicholas Hayward, Lionel Larue

Travel Awards Committee:
Marie-Dominique Galibert, Prasad Kumarasinghe, Takahiro Kunisada, Caroline Le Poole,

Poster Prize Committee:
Anja Bosserhoff, Chikako Nishigori, Davinder Parsad, Andrzej Slominski

Sponsored by (**as of 30 June 2011**)
Premium Sponsors
Astellas
Avène eau thermale
Clarins

© 2011 The Authors. Journal Compilation © 2011 John Wiley & Sons A/S

Galderma
L'Oréal
Pierre Fabre Dermo-cosmétique
Giuliani

Major Sponsors
Janssen Pharmaceutica
Leo Pharma
Mavig
Noreva · LED
Procter & Gamble

Others Sponsors
Bioderma
Bristol-Myers-Squibb
Clinuvel
Deka
Expanscience
Intendis
Johnson & Johnson
LVMH recherche
Mene&Moy
Pfizer
Quantel
Université Bordeaux Segalen
Wiley

Invited Patients Support Groups
Association française du Vitiligo, Genespoir (Albinisms), Association les Enfants de la Lune (Xeroderma pigmentosum), Naevus 2000 (Giant Congenital Nevus)

SCIENTIFIC PROGRAMME
Tuesday 20th, September 2011

**13.00–17.00**	**Special Interest Groups Meetings**<br>**Room D Eumelanet Workshop: Standardization of melanin chemistry**<br>Chair: M. d'Ischia<br>**Room E Vitiligo Global Issues Consensus Conference**<br>Chairs: Y. Gauthier – A. Taïeb – M. Picardo<br>**Room F IFPCS Development Group**<br>Chairs: B. Pavan – R. Kelsh – H. Yamamoto – L. Kos
**17:00–18:00**	**Regional Councils meetings ASPCR (Room D), ESPCR (Room G), JSPCR (Room H), PASPCR (Room E).**

IFPCS first Council Meeting Room F 18.00–20.00

Wednesday 21st September 2011
Opening addresses 08:30–09:00 HALL A
Addresses from President of Bordeaux University, IFPCS President, ESPCR President and Honorary Presidents

Plenary session I: Opening lectures 09:10–10:30 HALL A
Chairs: M. Picardo – P. Kumarasinghe – JP. Lacour

9.10–9.30	**SL1 IPCC Special Lecture:** J. Borovansky, P. Riley, G. Ghanem (Czech Republic, UK, Belgium): Twenty-five years of ESPCR
09:30–10.00	**SL2 Presidential Lecture** S. Shibahara (Japan): Pigment production for maintaining epidermal homeostasis: lessons from keratinocytes and melanocytes
10:00–10:30	**SL3 Seiji Memorial Lecture** R. Spritz (USA): Thirty years of vitiligo genetics

Coffee break 10:30–11:00

Plenary session II: Tracking the precursors/Developmental biology 11:00–11:30 HALL A
Chairs: D. Bennett – T. Kunisada – B. Werhle-Haller

11.00–11.30	**Guest lecture 1 (GL1)** Tatjana Sauka-Spengler (UK): Deciphering gene regulatory interactions controlling neural crest formation

Concurrent sessions 1–3 11.30–13.00 HALLS A, B, C

CS1: Developmental biology (continues in HALL A)
Chairs: D. Bennett, T. Kunisada – B. Werhle-Haller

11.30–11.50	**IL1** B. Werhle-Haller: (Switzerland): Melanocyte development: the journey to unknown territory
11.50–12.10	**IL2** T. Kunisada: (Japan): Cellular origin of melanocytes: newly resolved routes to melanocyte cell lineages
12.10–12.22	**C1** A. Saldana-Caboverde, L. Kos (USA): Ets1 interacts with Sox10 during murine melanocyte development.
12.22–12.34	**C2** Y. Takahashi, H. Murai, K.-I. Zakai, R. Tadokoro (Japan): Live imaging of melanosome transfer in the developing skin

© 2011 The Authors. Journal Compilation © 2011 John Wiley & Sons A/S

12.34–12.46	**C3** E. Reyes-Gomez, N. da Silva, S. Gadin-Czerw, J.-J. Panthier, G. Aubin Houzelstein (France): Does overexpression of the Strawberry Notch homolog 2 gene in Dopachrome tautomerase expressing cells trigger a defect in melanoblast specification?
12.46–12.58	**C4** E. Van Otterloo, G. Lai, R. Weigel, R. Cornell (USA): Transcription factor Activator Protein 2 directly activates Sox10 to induce melanoblasts and co-operates with MITF to promote melanocyte differentiation

CS2: Chemistry and biophysics of melanins HALL C

Chairs: A Napolitano – J. Menter-P Riley

11.30–11.50	**IL3** T. Sarna (Poland): Physicochemical changes of retinal pigment epithelium melanin with aging and photoaging monitored by advanced EPR techniques
11.50–12.10	**IL4** K. Wakamatsu: (Japan): Elucidation of biogenic pathway of a dark brown pigment neuromelanin in the substantia nigra of human brain
12.10–12.22	**C5** L. Panzella, G. Greco, L. Verotta, M. d'Ischia, A. Napolitano (Italy): Discovery of Isoquinoline-Containing Dimers as the Fundamental Building Blocks of Human Red Hair Pheomelanin.
12.22–12.34	**C6** K. Glass, R. Rengifo, J. D. Simon (USA): Probing the Melanosome Surface using Molecular Rulers
12.34–12.46	**C7**. P. Meredith, B. Mostert, I. R. Gentle, G. Hanson, K. Tandy, E. Namdas, F. Pratt, B. J. Powell (Australia): Is melanin a semiconductor: the mysteries of electrical conduction and melanin bioelectronics?
12.46–12.58	**C8**. A. Brocas, V. Shynkar, M. Mahet, E. Tham, Y. Abidine, P. Guitera, F. Amblard (France) Rare melanoma cell detection by thermal emission imaging

CS3: Difficult to classify hyperpigmentations, clinically-oriented HALL B

Chairs: D. Parsad – Y. Gauthier – B. K. Goh

11.30–11.50	**IL5** B. K. Goh (Singapore): Difficult Hyperpigmentary Disorders : An Asian Perspective
11.50–12.10	**IL6** Y. Gauthier (France): Pathogenesis of melasma: new insights
12.10–12.22	**C9** R. Dhurat, S. Mishra, C. Nayak, D. Deshpande (India) Diagnostic utility of dermatoscopy in hydroquinone induced exogenous ochronosis.
12.22–12.34	**C10** N. Sarma (India) A study on clinico-histological evaluation of 155 cases of Periorbital melanosis
12.34–12.46	**C11** S. Tambe, H. Jeranjani, S. Ghate (India) Epidemiological, clinical & histopathological profile and patch test results in patients of Primary Localized Cutaneous Amyloidosis.
12.46–12.58	**C12** L. Benzekri (Morocco) How to differentiate melasma from facial postinflammatory hyperpigmentation (PIH)?

Lunch break and poster viewing 13:00–14:30

Concurrent sessions 4–6 14:30–16:00 HALLS A, B, C

CS4: Mouse models: pigment cell biology & melanoma HALL B

Chairs: L. Montoliu – W. Pavan – E. Nishimura

14.30–14.50	**IL7** L. Montoliu (Spain): Mouse models for studying pigment cell biology and pigmentary diseases
14.50–15.10	**IL8** B. Pavan (USA): A novel Sox10 modifier locus identified in a sensitized ENU mutagenesis screen
15.10–15.22	**C13** A. Eychène (France): Mouse models for Raf signaling in melanocyte and melanoma development
15.22–15.34	**C14** Y. Kotobuki, A. Tanemura, S. Serada, M. Fujimoto, T. Naka, I. Katayama (Japan): Periostin Promotes Tumor Growth and Progression in Cutaneous Malignant Melanoma
15.34–15.46	**C15** E. Steingrimsson, H. Arnheiter, K. Bergsteinsdottir, K. Bismuth, B. K. Gisladottir, A. G. Hansdottir, L. Crawford, H. Murakami, M. Gasper, J. Debbache, A. Parekh, S. Skuntz, D. A. Swing, J. H. Hallsson, D. E. Fisher, N. G. Copeland, N. A. Jenkins (Iceland): An induced suppressor mutation at the microphthalmia locus in the mouse reveals novel insights into bHLHZip transcription factor function.
15.46–15.58	**C16** Y. Funasaka, S. Oyama, S. Okazaki, S. Kawana, C. Nishigori (Japan:) Ultraviolet B, but not ultraviolet A initiates and promotes melanoma formation in metabotropic glutamate receptor 1 transgenic mouse

CS5: Chemistry of melanins: standardization workshop roundtable HALL C

Chairs: M d'Ischia – S. Ito – JM. Garcia Borron – J. Simon

**C17**	M. d'Ischia, J.-C. Garcia-Borron Martinez, S. Ito, J. D. Simon (Italy, Spain, Japan, USA) Methods in Melanin Research.

 © 2011 The Authors. Journal Compilation © 2011 John Wiley & Sons A/S

**C18**	S. Ito, Y. Nakanishi, K. Wakamatsu (Japan) Evaluation of alkaline hydrogen peroxide oxidation to analyze eumelanin and pheomelanin

CS6: Human skin color and its evolution HALL A

Chairs: C. Le Poole – R. Sturm – E. Healy

14.30–14.50	**IL9** R. Sturm (Australia): Human pigmentation genes and population polymorphism
14.50–15.02	**C19** C. Delevoye, G. Van Niel, S. Simoes, I. Hurbain, M. Romao, D. Tenza, M. Marks, G Raposo (France): Cellular and molecular mechanisms underlying the biogenesis of melanosomes
15.02–15.14	**C20** S. Commo, K. Wakamatsu, B. A. Bernard, S. Ito (France): Human hair pigmentation characteristics revealed by melanin determination in human eumelanic hairs of various ethnic origins
15.14–15.26	**C21** E Mauger, K. Ezzedine, R. Jdid, J. Latreille, D. Malvy, F. Gruber, P. Galan, S. Hercberg, E. Tschachler, C. Guinot (France): Phenotypic, genetic and lifestyle risk factors for solar lentigines in adult Caucasian women
15.26–15.38	**C22** T. Motokawa, M. Ito, K. Tashiro, K. Yokoyama (Japan): Detection of new factors involved in melanogenesis
15.38 15.50	**C23** P. Tanner, S. Leachman (USA): Vitiligo Color Measurements for Formulating the Base Color Pigment Quantities for Human Integument Phantoms and Replicating Human Integument with Prosthetic Materials
15.50–16.00	Discussion

Coffee Break and poster viewing 16:00–16:30

Concurrent sessions 7–9 16:30–18:00

CS7: Non cutaneous melanocytes HALL C

Chairs: L. Kos – H. Yamamoto – T. Sarna

16.30–16.50	**IL10** L. Kos (USA): The other ones: non cutaneous melanocytes
16.50–17.10	**IL11** H. Yamamoto and S. Uehara (Japan): A role for the inner ear melanocytes in anti-stress responses
17.10–17.22	**C24** A. Shinomiya, K. Kinoshita, M. Mizutani, T. Namikawa, Y. Matsuda, Y. Kayashima, T. Akiyama (Japan): Gene duplication linked to Fm locus is closely correlated to hyperpigmentation of internal organs in Silky chicken
17.22–17.34	**C25** S. Murillo-Cuesta, J. Contreras, M. Cantero, R. Cediel, R. Martínez-Vega, E. Zurita, A. Fernández, I. Varela-Nieto, L. Montoliu (Spain): Albino and pheomelanic mice are more susceptible and present a poorer recovery after noise-induced hearing loss compared to eumelanic mice
17.34–17.46	**C26** X. Ma, L. Pan, H. Li, B. Wen, J. Wang, X. Jin, X. Dai, J. Yang, Y. Chen, Z. Su, L. Hou (China): MITF regulates growth factor expression and cell migration in RPE cells
17.46–17.58	**C27** S. Devi, Y. Markandeya, N. Maddodi, K. Wakamatsu, S. Ito, R. Balijepalli, V. Setaluri (USA): A novel pathway for regulation of pigmentation by glutamate receptor mGluR6 through its action on TRPM1

CS8: Update on physiology of cutaneous pigmentation HALL A

Chairs: Z. Abdel-Malek – S. Moretti – G. Imokawa

16.30–16.50	**IL12** Z. Abdel-Malek (USA) The melanocyte living on the edge, sustained by its neighboring keratinocytes and fibroblasts
16.50–17.10	**IL13** G. Imokawa: (Japan) Endothelin-1/stem cell factor signalling blockade in melanocytes and pigmentation in human epidermal equivalents
17.10–17.22	**C28** D. Kovacs, E. Flori, V. Maresca, M. Ottaviani, N. Aspite, L. Panzella, A. Napolitano, M. d'Ischia, M. Picardo (Italy) The eumelanin intermediate 5,6-dihydroxyindole-2-carboxylic acid (DHICA) promotes differentiation and protection in epidermal cells: an additional role of melanogenesis
17.22–17.34	**C29** S. G. Coelho, S. Ito, K. Wakamatsu, S. A. Miller, J. Z. Beer, V. J. Hearing (USA) Distribution patterns of eumelanin and pheomelanin in human skin
17.34–17.46	**C30** M. Cario-Andre, K. Ezzedine, C. Pain, V. Guyonnet-Dupérat, A. Bibeyran, A. Taïeb (France) Fibroblasts regulate both physiological and pathological pigmentation of skin in vitro and in vivo
17.46–17.58	**C31** C. Duval, C. Cohen-Dellarre, C. Chagnoleau, F. Bernerd (France) Essential role of dermal components in regulating the pigmentation in a full thickness reconstructed skin model

© 2011 The Authors. Journal Compilation © 2011 John Wiley & Sons A/S

CS9: Depigmentation update, clinically-oriented HALL B

Chairs: K. C. Park – JP. Ortonne – H. S. Yu

16.30–16.50	**IL14** JP. Ortonne (France): Depigmentation update: treatment of melasma
16.50–17.10	**IL15** K. C. Park (Korea): Treatment of hyperpigmentary disorders in Asian skin
17.10–17.22	**C32** J. Latreille, K. Ezzedine, E. Kesse-Guyot, R. Jdid, D. Malvy, P. Galan, E. Tschachler, C. Guinot, S. Hercberg (France) Association between severity of solar lentigines and fatty acid intakes in adult Caucasian women
17.22–17.34	**C33** J. Y. Kim, J. Y. Shin, M. R. Kim, S.H. Oh (Korea) The Role of DKK1 in the Development of Vitiligo
17.34–17.46	**C34** M. C. Costa, L. S. Abraham, M. Ardigò, M. Picardo, P. L. Araújo, L. Azulay-Abulafia, J. M. Piñeiro-Maceira (Brazil) Hairs presence and pigmentation in vitiligo lesions: the usefulness of dermatoscopy in prognosis and treatment response evaluation
17.46–17.58	**C35** A.-Y. Lee, N.-H. Kim, J. M. Kim, K. A. Cheong (Korea) H19RNA downregulation in melasma

Welcome Reception, 18:00–19:30
Thursday 22nd, September 2011

Plenary session III: Stem Cells: facts, fancy, fiction? 08.00 – 10.30 HALL A
PASPCR Aaron Lerner Lecture

Chairs: A. Slominski – R. Boissy – J. Pawelek

SL4 C. Goding (UK): Signalling and transcription in melanoma stem-like cells

Chairs: C. Goding – L. Sommer – R Halaban

8.30–9.00	**Guest lecture 2 (GL2**): R. M. Hoffman (USA): Hair follicle pluripotent stem (hfPS) cells for regenerative medicine an advantageous alternative to ES and iPS cells
9.00–9.20	**IL16** E. Nishimura (Japan): Stem Cell Regulation by Stem Cells
9.20–9.40	**IL17** L. Sommer: (Switzerland): Neural crest stem cells and melanoma formation: a likely connection
9.40–9.52	**C36** H. Aoki, T. Kunisada (Japan) Protective effect of Kit signaling for melanocyte stem cells against radiation-induced genotoxic stress
9.52–10.04	**C37** S. Tabone-Eglinger, M. Wehrle-Haller, N. Aebischer, M.-C. Jacquier, D. Boettiger, B. Wehrle-Haller (Switzerland) Dual function of membrane-bound KitL and potential role to anchor melanocyte stem cells in their niche
10.04–10.16	**C38** L. Latif, I. E. Tribe, B. Babakinejad, S. A. Kamali, R. H. Patel, Y. A. Negulaev, Y. E. Korchev, E. V. Sviderskaya (UK) Characterisation of pluripotent immortal postnatal mouse neural crest-like stem cells
10.16–10.28	**C39** B. Ferguson, T. Kunisada, G. Walker (Australia) In vivo responses of melanocyte stem cells and other immature melanocytic cells to ultraviolet radiation-induced damage.

Coffee break and poster viewing 10.30–11.00

Plenary session IV: Photoprotection and beyond: from melanosomes to melanins 11:00–13:00 HALL A

Chairs: M. d'Ischia – K. Wakamatsu – T. Passeron

11.00–11.30	**Guest lecture 3 (GL3**)V. Sundstrom (Sweden): Photochemistry and excited state dynamics of eumelanin building blocks
11.30–12.00	**Guest lecture 4: (GL4**) E. Sprecher (Israel): Keratin disorders associated with abnormal pigmentation: clinical and molecular insights
12.00–12.12	**C40** J. D. Simon, D. N. Peles (USA) Ultraviolet Absorption Properties of Melanosomes Measured by Photoemission Electron Microscopy
12.12–12.24	**C41** A. Napolitano, G. Greco, L. Panzella, G. Gentile, M. E. Errico, M. d'Ischia (Italy) Pheomelanin is a prooxidant promoting DOPA conversion to a eumelanin coating: discovery of a non-enzymatic mimic of the natural casing process of melanosome assembly
12.24–12.36	**C42** C. Kaelin, L. Hong, X. Xu, V. David, A. Schmidt-Kuentzel, S. O'Brien, M. Menotti-Raymond, G. Barsh (USA) Genomics of pattern: from Akitas to Zebras
12.36–12.48	**C43** T. Wiesner, A. C. Obenauf, R. Murali, I. Fried, P. Ulz, S. Loy, W. Wackernagel, C. Windpassinger, I. Wolf, K. G. Griewank, A. Viale, A. E. Lash, M. Pirun, N. D. Socci, A. Ruetten, G. Palmedo, D. Abramson, K. Offit, A. Ott, J. C. Becker, L. Cerroni, H. Kutzner, M. R. Speicher, B. C. Bastian (USA) Germline mutations in BAP1 predispose to melanocytic tumors

© 2011 The Authors. Journal Compilation © 2011 John Wiley & Sons A/S

2nd IFPCS Council Meeting 13:00 to 14:00 Room E

Lunch break and poster viewing 13:00 to 14:30

Concurrent sessions 10–12 14:30–16:00

CS10: Genetics of pigmentation and molecular biology of melanoma, clinically-oriented HALL B
Chairs: R. Spritz – N. Soufir – D. Lacombe

14.30–14.50	**IL18** N. Soufir (France): Pigmentation genes and melanoma: where are we now ?
14.50–15.10	**IL19** L Larue: (France) Murine models: coat color and melanoma
15.10–15.22	**C44** A. Mogha, D. Gilot, A. Primot, J. Debbache, F. Journe, D. C. Bennett, B. Dreno, A. Napolitano, G. Ghanem, M.-D. Galibert (France) Uncovered role of Tyrosinase-related Protein 1 (TYRP1) in melanoma cells aggressiveness
15.22–15.34	**C45** F. Morice-Picard, E. Lasseaux, C. Rooryck-Thambo, A. Rouault, D. Cailley, C. Plaisant, D. Lacombe, A. Taïeb, B. Arveiler (France) Molecular analysis of 246 patients with oculocutaneous albinism – the Bordeaux experience.
15.34–15.46	**C46** M. Sakaguchi, M. Oka, T. Iwasaki, Y. Fukami, C. Nishigori (Japan) Ser727 phosphorylation in STAT3 plays a crucial role in nuclear translocation of STAT3 and growth in human melanoma cells and melanocytes
15.46–15.58	**C47** R. Lazova, G. LaBerge, E. Duvall, N. Spoelstra, M. Sznol, D. Cooper, R. Spritz, J. Pawelek (USA) First evidence for tumor cell-leukocyte fusion in human cancer: a melanoma brain metastasis with a donor-patient hybrid genome following allogeneic BMT.

CS11: Vitiligo: basic science & medical, clinically-oriented HALL A
Chairs: S. K. Hann – T. Anbar – P. Manga

14.30–14.50	**IL20** S. Moretti (Italy): Vitiligo 2011 update
14.50–15.10	**IL21** R. Attili (India): Acrofacial and genital depigmentation is a new pattern disease with features of both vitiligo and vitiligoid lichen sclerosus
15.10–15.22	**C48** S. K. Attili, V. R. Attili (UK & India) Histopathological staging of vitiligo lesions – Implications for treatment
15.22–15.34	**C49** J. Mosenson, A. Zloza, J. Klarquist, S. Mehrotra, M. Nishimura, J. A. Guevara-Patino, I. C. Le Poole (USA) A hot finding: mutant HSP70i to treat vitiligo
15.34–15.46	**C50** R. Kumar, D. Parsad, A. J. Kanwar (India) LXR-α as molecular switch that initiate transition from vitiligo lesional skin to repigmented skin?
15.46–15.58	**C51** R. Yu, M. Su, A. Xu, X. Zhang, Y. Zhou (Canada) ''Bad soil'' – defective local microenvironment for melanocytes in vitiligo

CS12: Stress responses HALL C
Chairs: LF. Xiang – ML. dell'Anna – A. Mauviel

14.30–14.50	**IL22** M Picardo (Italy): Stress responses, a bridge between physiological and pathological processes in melanocytes
14.50–15.10	**IL23** MD. Galibert: (France) How does solar UV radiation initiate specific cellular responses?
15.10–15.22	**C52** L. Marrot, J.-P. Belaïdi, L. Denat, D. Duche, C. Jones, P. Perez, J. Sœur, R. Rani, R. S. Gokhale, J.-R. Meunier (France) Antioxydant defenses in human epidermal melanocytes and keratinocytes suggests that nrf2 plays a peculiar role in epidermis: implication in vitiligo lesions.
15.22–15.34	**C53** E. Flori, A. Mastrofrancesco, D. Kovacs, Y. Ramot, S. Briganti, R. Paus, M. Picardo (Italy) The parrodiene derivative, 2,4,6-octatrienoic acid, acts as a novel promoter of melanogenesis and antioxidant defence in normal human melanocytes in situ and in vitro via PPARγ activation
15.34–15.46	**C54** J. Menter, C. Nokkaew, A. Sprewell, D. Eatman, S. Harris-Hooker (USA) Pigment Melanin Mediates a Redox Reaction between Adsorbed Nitric Oxide and O2 in vitro.
15.46–15.58	**C55** H. Y. Handoko, M. Rodero, G. Walker, K. Khosrotehrani (Australia) The immune response influences melanocyte proliferation after ultraviolet radiation exposure

Coffee Break and Poster Viewing 16:00–16:30

Concurrent sessions 13–15 16:30–18:00

CS13: Neuroendocrinology of pigmentation and MC1R HALL A
Chairs: A. Slominski – D. Tobin – M. Böhm

16.30–16.50	**IL24** A. Slominski: (USA): Introduction to the neuroendocrinology of the pigmentary system
16.50–17.10	**IL25** M. Böhm (Germany)Modulatory effects of a small peptide derivative of alpha-MSH, KdPT, on melanocyte responses to oxidative stress

© 2011 The Authors. Journal Compilation © 2011 John Wiley & Sons A/S

17.10–17.22	**C56** C. Skobowiat, J. C. Dowdy, R. M. Sayre, R. C. Tuckey, A. Slominski (USA) Ultraviolet radiation A and B regulate the neuroendocrine stress response system in melanocyte/keratinocyte co-cultures
17.22–17.34	**C57** A. Belen, P. Oliva, C. Olivares, M. Abrisqueta, C. Jimenez-Cervantes, J. C. Garcia-Borron (Spain) Regulation of human melanocortin 1 receptor (MC1R) signalling by β-arrestins.
17.34–17.46	**C58** A. Kokot, T. A. Luger, M. Böhm (Germany) Tropisetron, a serotonin antagonist, modulates the inflammatory cell response of human epidermal melanocytes and keratinocytes after exposure of UVB light or TNF-alpha
17.46–17.58	**C59** A. L. Kadekaro, V. Maresca, E. Flori, D. Kovacs, G. Cardinali, J. Chen, S. Chen, M. Picardo (USA & Italy) Impact of MC1R variants on the antioxidant responses of melanocytes and implications on human skin homeostasis

CS14: Vitiligo: surgical-instrumental, clinically-oriented HALL B
Chairs: N. Rabobee – S. Mulekar – E. Lan

16.30–16.50	**IL26** E. Lan (Taiwan): Monochromatic light for treatment of vitiligo
16.50–17.10	**IL27** S. Mulekar (Saudi Arabia): Experience of surgical procedures in childhood vitiligo
17.10–17.22	**C60** P. Araujo (Brazil): Surgical management of vitiligo
17.22–17.34	**C61** D. Ghia, C. Nayak (India): To trypsinise, or not to trypsinise, that is the question
17.34–17.46	**C62** S. Awasthi, A. J. Kanwar, D. Parsad (India): Comparing the effect on the outcome of cold trypsinisation v/s warm trypsinisation in transplantation of autologous non cultured epidermal cell suspension in stable vitiligo – a prospective randomized study
17.46–17.58	**C63** G. Garg, U. S. Khopkar (India): A comparative study of efficacy and safety of Modified Dermabrasion followed by NB-UVB therapy with Suction Blister Epidermal Grafting followed by NB-UVB therapy in stable vitiligo patients.

CS15: Non Mouse animal models and in vitro human models HALL C
Chairs: G. Erf – R. Kelsh – MD. Galibert

16.30–16.50	**IL28** R. Kelsh (UK): Pigmentation in non-mouse models – fishing for insight, not just horsing around?
16.50–17.10	**IL29** G. Erf: (USA) Chicken models for vitiligo and other spontaneous autoimmune/autoinflammatory disorders
17.10–17.22	**C64** K. Taylor, J. Richardson, R. Kelsh, I. Jackson, J. Lister, E. E. Patton (UK) Mitf mutations promote differentiated cell division and melanoma in zebrafish
17.22–17.34	**C65** M. Gillard, C. De Brito, J. Abadie, B. Vergier, A.-S. Guillory, E. Cadieu, P. Devauchelle, F. Galibert, B. Hédan, C. André (France) Canine melanoma : promising spontaneous models for genetics and therapies of human melanoma
17.34–17.46	**C66** C. Talari, K. Gledhill, D. J. Tobin (UK) Do epidermal melanocytes contribute to the erythema response in human skin post-UVB irradiation?
17.46–17.58	**C67** T. Biedermann, G. Kiowski, D. S. Widmer, G. Civenni, C. Burger, R. Dummer, M. Meuli, L. Sommer, E. Reichmann (Switzerland) Engineering melanoma progression in a humanized environment in vivo

Regional Societies Assemblies 18:00 to 19:00 ASPCR (HALL A), ESPCR (Room HALL B), JSPCR (Room HALL C), PASPCR (Room E),

Speaker's Dinner (Pierre Fabre) 20:00 to 22:00

Friday 23rd, September 2011

Plenary session V. Fundamental aspects of the initiation and progression of melanoma (1) 08:30–10:30 HALL A
Chairs: R. Luiten – N. Hayward – R. Ballotti

8.30–9.00	**Guest lecture 5 (GL5)**: Y. Hayashizaki (Japan): Availability of transcriptional regulatory network analysis by next-generation sequencer
9.00–9.20	**IL30** D. Fisher (USA) Roles for MITF in melanomagenesis
9.20–9.42	**C68** D. J. Coleman, S. Hyter, H. S. Jang, X. Liang, L. Larue, G. Indra, A. K. Indra (USA) Novel role of melanocytic RXR alpha/RXR beta in UV irradiation induced melanocyte homeostasis
9.42–9.54	**C69** F. P. Noonan, M. R. Zaidi, A. Wolnicka-Glubisz, M. R. Anver, J. Bahn, A. Wielgus, J. Cadet, T. Douki, S. Mouret, A. Popratiloff, G. Merlino, E. C. De Fabo (USA) Two UV pathways to melanoma
9.54–10.06	**C70** V. Pogenberg, M. H. Ogmundsdottir, K. Bergsteinsdottir, M. Milewski, V. Deineko, B. Phung, A. Schepsky, M. Wilmanns, E. Steingrimsson (Iceland) The Mitf structure unravels DNA binding and dimerization specificities

 © 2011 The Authors. Journal Compilation © 2011 John Wiley & Sons A/S

10.06–10.18 **C71** Y. Cheli, S. Giuliano, N. Fenouille, M. Allegra, V. Hofman, P. Hofman, P. Bahadoran, J.-P. Lacour, S. Tartare-Deckert, C. Bertolotto, R. Ballotti (France) Hypoxia and MITF control metastatic behaviour in mouse and human melanoma cells.
10.18–10.30 Discussion

Coffee Break and Poster Viewing 10:30–11:00

Plenary session VI Fundamental aspects of the initiation and progression of melanoma (2) 11:00–12:30 HALL A
Chairs: D. Fisher – Z. Ronai – A. Spatz
11.00–11.20 **IL31** M. Davies (USA): Regulation and Function of the PI3K Pathway in Advanced Melanoma
11.20–11.40 **IL32** B. Bastian (USA): Oncogenic signaling downstream of Gq/11
11.40–12.00 **IL33** R. Marais (UK): RAS and RAF signalling in melanoma: translating biology into therapies
12.00–12.12 **C72** A. Marquette, J. Andrée, M. Bagot, A. Bensussan, N. Dumaz (France) When CRAF takes over from BRAF in melanoma using ERK and PDE4
12.12–12.24 **C73** M. Smith, J. Ferguson, I. Arozarena, C. Wellbrock (UK)A novel link between TGFbeta and MAP kinase signalling is involved in resistance to MEK inhibition in melanoma

Lunch Break and Poster Walk 12:30–14:00

Concurrent sessions 16–18 14:00–15:30

CS16: Preclinical and clinical advances in melanoma management (SMR-IFPCS) HALL A
Chairs: B. Bastian, L. Larue, B. Guillot
14.00–14.20 **IL34** C. Garbe: (Germany): New developments in melanoma therapy – ASCO update
14.20–14.32 **C74** J. Aruri, R. Kapadia, H. Mehr, H. Ho, M. A. White, A. K. Ganesan (USA:) Synthetic Lethal RNAi-screening Uncovers a Novel Role for Rho Family GTPases in Controlling Cell Fate and Chemoresistance.
14.32–14.44 **C75** R. W. Dellinger, H. H. Matundan, F. L. Meyskens (USA): Functional Role for the UDP-Glucuronosyltransferases (UGTs) in Melanoma Drug Resistance
14.44–14.56 **C76** K. Jimbow, A. Yoneda, Y. Tamura, Y. Osai, M. Sato, A. Sato, T. Kamiya, J. Kato, A. Takada, T. Yamashita, A. Miyamoto, A. Ito, H. Honda, K. Wakamatsu, S. Ito, K. Murase, S. Nohara, E. Nakayama, T. Kobayashi (Japan): Introduction of melanoma in situ peptide vaccine by chemothermotherapy through exploitation of melanogenesis substrate, NPrCAP, and its conjugation with magnetite nanoparticles
14.56–15.08 **C77** F. Journe, M. Wiedig, R. Morandini, F. Sales, A. Awada, G. Ghanem (Belgium): cKIT expression level and NRAS/BRAF mutation status predict the response to the tyrosine kinase inhibitor dasatinib in melanoma cell lines
15.08–15.20 **C78** D. Lang, J. B. Mascarenhas, D. Wolfgeher, J. D. Kubic (USA): Promotion of melanoma growth and survival through Glycogen Synthase Kinase-3 protein activity
15.20–15.30 Discussion

CS17: New pathomechanisms in melanoma HALL C
Chairs: F. Meyskens, M. Soengas, N. Basset-Seguin
14.00–14.20 **IL35** F. Meyskens USA): Chemoprevention of melanoma progression mediated by NO/neural NO synthase (nNOS)/NO, an accelerator of the transformation process
14.20–14.40 **IL36** M. Soengas (Spain): Endolysosomal pathways in melanoma maintenance and drug response
14.40–15.00 **IL37** D. Bennett (UK): Impacts of p16 deficiency on melanocyte gene expression and biology: relation to early melanoma.
15.00–15.12 **C79** J. C. Valencia, S. G. Coelho, L. Yin, W. D. Vieira, V. J. Hearing (USA) Fighting Proliferation with Differentiation: How Pmel17/gp100 binding to FHL2 leads the charge in melanoma.
15.12–15.24 **C80** M. Ohanna, R. Ballotti, C. Bertolotto (France) Senescent cells develop a secretome

CS18: From vitiligo to melanoma HALL B
Chairs: D. Norris – W. Westerhof – C. Lebbé
14.00–14.20 **IL38** R. Luiten (The Netherlands): Monobenzone increases the immunogenicity of melanoma cells, and is effective as melanoma immunotherapy
14.20–14.32 **C81** M. W. Kroon, G. Krebbers, R. Thijssen, W. Douwenga, J. D. Bos, J. P. W. Van Der Veen, M. A. Middelkamp Hup, R. M. Luiten (The Netherlands) Effect of UVB therapy on the lymphocytic infiltrate in the skin of vitiligo patients
14.32–14.44 **C82** J. Klarquist, M. Li, D. A. Wainwright, R. M. Luiten, M. I. Nishimura, I. C. Le Poole (USA) Functional cloning of a gp100-reactive TCR from depigmenting vitiligo skin

© 2011 The Authors. Journal Compilation © 2011 John Wiley & Sons A/S

14.44–15.56	**C83** S. Yang, Z. I. Zheng, B. Misner, R. Chamberlin, F. L. Meyskens. (USA) APE/Ref-1, a druggable target for the therapy of human melanoma
15.56–15.08	**C84** M.-J. Pierrat, V. Marsaud, C. Bertolotto, A. Mauviel, D. Javelaud (France) Involvement of TGF-beta signalling and the GLI2 transcription factor in M-MITF regulation and pigmentation in melanoma cells
15.08–15.20	**C85** N. Chin, J. C. Gallegos, R. Cruz, L. Kos (USA) Edn3 promotes metastasis and alters tumour heterogeneity in a mouse model of melanoma
15.20–15.30	Discussion

Concurrent sessions 19–21 15:30–17:00

CS19: Preclinical & Clinical advances in melanoma management (SMR-IPCC) HALL A

Chairs: P. Chapman – G. Ghanem – P. Saiag

15.30–15.50	**IL39** R. Carvajal (USA): KIT Aberrations in Melanoma and Therapeutic Implications
15.50–16.10	**IL40** G. Ghanem (Belgium): TYRP1, a missing link between melanogenesis and melanoma progression ?
16.10–16.22	**C86** M. Hossain, W. H. Chong, A. M. Ross, E. V. Sviderskaya, D. C. Bennett (UK): Markers of telomeric crisis and immortalization in melanoma progression.
16.22–16.34	**C87** D. S. Widmer, O. M. Eichhoff, M. C. Zipser, P. F. Cheng, R. Dummer, K. S. Hoek (Switzerland): The role of hypoxia in melanoma phenotype switching
16.34–16.46	**C88** M. Tichet, N. Fenouille, P. Abbe, S. Rocchi, M. Allegra, J.-C. Chambard, M. Vivinus, J.-P. Lacour, R. Ballotti, M. Deckert, S. Tartare-Deckert (France): SPARC acts as a VCAM-1 ligand to mediate melanoma extravasation and distant metastasis
16.46–16.58	**C89** A. P. Benaduce, D. Lahiri, A. Agarwal, L. Kos (USA): Melanocytes and melanoma cells present different mechanical properties that can be modulated by Endothelin 3.

CS20: Congenital nevus, clinically-oriented HALL B

Chairs: H. Etchevers – V. Kinsler – B. Vergier

15.30–15.50	**IL41** V. Kinsler: (UK): Congenital Melanocytic Naevus Syndrome – clinical and genetic aspects
15.50–16.10	**IL42** H. Etchevers (France): Genomics and the molecular etiologies of congenital nevus formation
16.10–16.22	**C90** F. Luciani, D. Champeval, A. Herbette, L. Denat, B. Aylaj, S. Martinozzi, R. Ballotti, R. Kemler, C. R. Goding, F. De Vuyst, L. Larue, V. Delmas (France) Understanding Melanocyte Development : Biological analysis associated with mathematical modeling
16.22–16.34	**C91** C. M. Salgado, A. Davis, A. Heider, D. Basu, A. Rebbaa, M. Reyes-Múgica (USA) Cellular Dynamics In Congenital Melanocytic Nevi: Is ''Maturation With Depth'' A One-Way Road?
16.34–16.46	**C92** D. Basu, L. Schmitt, C. Gallati, M. Reyes-Mugica, A. Rebbaa (USA) Epithelial to mesenchymal-like transition is an earlier cellular response to stress than senescence: potential role as a target for cancer prevention
16.46–16.58	**C93** O. M. Eichhoff, A. Weeraratna, M. C. Zipser, D. S. Widmer, L. Kriegl, L. Larue, R. Dummer, K. S. Hoek (Switzerland) Differential LEF1 and TCF4 expression is involved in melanoma cell phenotype switching.

CS21: DNA repair and melanoma molecular biology HALL C

Chairs: A. Sarasin – H. de Verneuil-T Jouary

15.30–15.50	**IL43** A. Sarasin (France) The xeroderma pigmentosum syndrome: clinical, genetic and gene therapy issues
15.50–16.10	**IL44** H. Rezvani (Iran) Xeroderma pigmentosum: clues to understanding cancer initiation
16.10–16.22	**C94** H. H. Hu, V. Descamps, A. Bourillon, N. B. Seguin, A. Riffault, K. Ezzedine, C. Lebbe, M. Bagot, A. Bensussan, P. Saiag, B. Grandchamp, N. Soufir (France): A large French case-control study assessing the association of MC1R with melanoma: the unexpected role of non-RHC and rare MC1R variants
16.22–16.34	**C95** S. Corre, Y. Baron, N. Mouchet, A. Bouafia, S. Vaulont, S. Prince, M.-D. Galibert (France): USF1 is critical for the regulation of ner genes essential for early recognition of UV induced DNA-photolesions
16.34–16.46	**C96** C. Leikam, A. Hufnagel, S. Walz, S. Kneitz, M. Eilers, M. Schartl, S. Meierjohann (Germany): Evasion of ROS-dependent pigment cell senescence
16.46–16.58	**C97** V. B. Swope, C. Alexander, A. L. Kadekaro, S. Schwemberger, G. Babcock, Z. A. Abdel-Malek (USA): Induction of γ-H2AX by UV and α-melanocyte stimulating hormone, and implications on DNA repair in human melanocytes

 © 2011 The Authors. Journal Compilation © 2011 John Wiley & Sons A/S

IFPCS General Assembly 17:00 to 18:00 Hall A

Gala Dinner at Chateau Giscours Departure of Buses from 18.30

- Seiji Memorial Award (IFPCS),
- Myron Gordon Award (IFPCS),
- Takeuchi medal (JSPCR),
- Raper medal (ESPCR),
- Thomas B. Fitzpatrick Award,
- Poster Prizes

Saturday 24th, September 2011

Plenary session VII Translational research/vitiligo 08:30–10:30 HALL A
Chairs: G. Barsh – Y. Tomita – MF. Avril

8.30–9.00	**Guest lecture 6: (GL6**) JM Egly (France) Xeroderma pigmentosum and trichothiodystrophy: understanding cancer and non cancer phenotypes
9.00–9.20	**IL45** D. Tobin (UK): The human hair-bulb melanocyte: a model aging system for both our gray hair and our gray matter?''
9.20–9.32	**C98** H. E. Teulings, M. Overkamp, E. Ceylan, L. Nieuweboer-Krobotova, T. Nijsten, J. D. Bos, A. W. Wolkerstorfer, R. M. Luiten, J. P. W. van der Veen (The Netherlands): Retrospective analysis of melanoma and non-melanoma skin cancer incidence in a large vitiligo patient cohort
9.32–9.44	**C99** J. E. Harris, T. H. Harris, W. Weninger, E. J. Wherry, C. A. Hunter, L. A. Turka (USA): A new mouse model of vitiligo with epidermal depigmentation reveals a critical role for IFN-gamma in autoreactive T cell homing to the skin
9.44–9.56	**C100** J. Klarquist, J. Eby, B. J. Longley, M. I. Nishimura, S. Mehrotra, I. C. Le Poole (USA): Spontaneous epidermal depigmentation in mice – a model for vitiligo
9.56–10.08	**C101** S. Itoi, A. Tanemura, Y. Kotobuki, M. Wataya-Kaneda, I. Katayama (Japan): Descriptive Assessment on Dynamic Change of Dendritic Cell Distribution Both in Epidermis and Dermis of the Lesional Skin in Generalized Vitiligo Vulgaris: Link between Cellular Autoimmune Response and Melanocyte Disappearance
10.08–10.20	**C102** A. Tanemura, Y. Kotobuki, L. Yang, M. Wataya-Kaneda, H. Murota, M. Fujimoto, S. Serada, T. Naka, I. Katayama (Japan): Dysregulation of Melanocyte Function and Survival Induced by Th17-Related Cytokines and Their Involvement in the Pathogenesis for Vitiligo Vulgaris
10.20–10.30	Discussion

Coffee Break 10:30–11:00

Plenary session VIII Translational research/Miscellaneous (2) 11:00–13:00 HALL A
Chairs: H. Arnheiter – D. Gawkrodger – F. Tison

11.00–11.30	**Guest lecture 7 (GL7**) : T. Luger (Germany): Alpha melanocyte stimulating hormone: a major component of the skin immune system with a therapeutic potential
11.30–12.00	**Guest lecture 8 (GL8**): L. Zecca (Italy): Neuromelanins in brain aging and Parkinson's disease
12.00–12.12	**C103** C. Grill, C. Praetorius, A. Schepsky, E. Steingrímsson (Iceland): The role of Interferon Regulatory Factor 4 (IRF4) in pigmentation
12.12–12.24	**C104** R. L. Mort, M. Moffat, L. Hay, K. J. Painter, I. J. Jackson (UK): Live imaging and mathematical modeling of the role of Kit/Kitl in melanoblast behaviour
12.24–12.36	**C105** T. Cheng, S. J. Orlow, P. Manga (USA): Melanocyte adaptation to ER stress and activation of the unfolded protein response in Oca2-null melanocytes
12.36.12.48	**C106** R. Lazova, R. Camp, V. Klump, S. Siddiqui, R. Amaravadi, J. M. Pawelek (USA): LC3B punctate expression, a marker for autophagosomes, is a common feature of melanomas and breast carcinomas and associated with proliferation, metastasis, high nuclear grade and poor outcome.
12.48–13.00	Discussion

Lunch break 13:00–14:30

Concurrent sessions 22–24 14:30–16:00

CS22: Vitiligo: Report on Global Issues Consensus Conference and selected papers, clinically-oriented HALL A
Chairs: I. Katayama – H. Lim – C. de Castro

© 2011 The Authors. Journal Compilation © 2011 John Wiley & Sons A/S

14.30–14.50	**IL46**: A. Taïeb (France) on behalf of VGICC panelists: VGICC: Objectives and priorities for international consensus
14.50–15.10	**IL47** DY Lee (Korea) Treatment guideline in segmental vitiligo
15.10–15.22	**C107** B. Bellei, A. Pitisci, M. Ottaviani, M. Ludovici, C. Cota, M. L. Dell'Anna, M. Picardo (Italy) Alterations of cellular redox-sensitive pathways regulation in vitiligo melanocytes converge to stress-activated cellular senescence phenotype.
15.22–15.34	**C108** V. Eleftheriadou, K. Thomas, J. Batchelor, J. Ravenscroft, M. Whitton (UK) Systematic review of outcome measurements for the treatment of vitiligo
5.34–15.46	**C109** T. S. Anbar, D. Kendall, H. Abdel-Raouf, T. S. El-Ammawi, M. Barakat, A. T. Abdel-Rahman, R. Torky, A. Fawzy, M. Hanna (Egypt)The paradox of the effects of PGF2alpha on pigmentation in vivo and in vitro
15.46–15.58	**C110** A. Ramaiah, H. K. Kar, V. K. Garg, N. Bajaj, A. S. Madhava (India) Double blind randomized clinical trial on bFGF related deca peptide to treat vitiligo

CS23: Albinism: Basic science and patient-oriented session, clinically-oriented HALL C

Chairs: T. Suzuki – R. Aquaron – B. Arveiler

14.30–14.50	**IL48** B. Arveiler (France): The genetics of oculocutaneous albinism
14.50–15.10	**IL49** T. Suzuki:(Japan) What's new in albinism among Japanese 2011
15.10–15.22	**C111** A. Wei, Y. Wang, W. Li (China)An optimized strategy for genetic testing of the Chinese patients with oculocutaneous albinism
15.22–15.34	**C112** E. Moltó, A. Fernández, C. Phillips, M. Torres, O. Maronas, B. Arveiler, F. Morice-Picard, A. Taïeb, R. Aquaron, V. Schiaffino, M. Hayashi, T. Suzuki, M. Martínez, M. J. Trujillo, C. Ayuso, Á. Carracedo, L. Montoliu (Spain) Albinochip: a universal genetic diagnosis for all known mutations associated to albinism
5.34–15.46	**C113** R. Aquaron, C. Badens, J. Kaplan, E. Lasseaux, F. Morice-Picard, C. Rooryck-Thambo, B. Arveiler (France) Oculocutaneous albinism type I (OCAI) in the Jewish populations of mediterranean sea countries (Algeria, Morocco, Tunisia): A story of the p.Gly47Asp mutation on the tyrosinase gene and personal data about 5 cases
15.46–15.58	**C114** A. R. Cullinane, J. A. Curry, C. Carmona-Rivera, G. Golas, C. G. Summers, C. Ciccone, N. D. Cardillo, H. Dorward, R. A. Hess, J. G. White, D. Adams, M. Huizing, W. A. Gahl(USA) BLOC-1 mutation screening in Hermansky-Pudlak Syndrome reveals a new HPS subtype, HPS-9, associated with mutations in PLDN (pallidin) and a novel BLOS3 (HPS-8) mutation.

CS24: Skin depigmenting/repigmenting agents, from basic mechanisms to application HALL B

Chairs: K. Al Ghamdi – N. Al Mutairi – MH. Lee

14.30–14.50	**IL50** HY. Kang (Korea) Overview of skin lightening agents and melasma
14.50–15.02	**C115** W. Choi, L. Kolbe, V. J. Hearing (USA) Characterization of the bioactive motif of neuregulin-1, a fibroblast-derived paracrine factor that regulates constitutive color and melanocyte function in human skin
15.02–15.14	**C116** Y. Huang, H. Lui, Y. Zhou (Canada) Active vitiligo lesions are more responsive to combination therapy with narrow band ultraviolet light B and topical tacrolimus
15.14–15.26	**C117** K. M. Al Ghamdi (Saudi Arabia) Depigmentation therapies for normal skin in vitiligo universalis.
15.26–15.38	**C118** M. Tanaka, M. Wataya-Kaneda, E. Kiyohara, A. Tanemura, A. Nakamura, S. Matsumoto, I. Katayama (Japan) Topical rapamycin therapy is effective for hypomelanotic macules arising in tuberous sclerosis complex
15.38–16.00	Discussion

Coffee Break 16:00–16:30

Plenary Session IX: Close of IPCC 2011 16:30 to 17:15 HALL A

Chairs: K. Jimbow – JP. Ortonne – JC Valencia

16.30–17.00	**Fitzpatrick Lecture SL5** T. Kunisada (Japan): Functionally distinct melanocyte populations revealed in mice: noncutaneous and dermal melanocytes versus epidermal melanocytes

Closing remarks and announcements 17:00: M Picardo, BK Goh, A Taieb

Posters

P1–P9: Melanins

**P1**	J. M. Belitsky (USA) Melanin Molecular Recognition
**P2**	S. Cardillo, G. Miotto, M. Massironi, C. A. Pallaoro, I. Meyer, F. Vianello (Germany) New index as melanin-pigmented skin marker
**P3**	T.-C. Lei (China) Measurements of hydroxyl free radical-scavenging capacities of melanin-binding hydroxychloroquine using an electron spin resonance spectroscopy

© 2011 The Authors. Journal Compilation © 2011 John Wiley & Sons A/S

P4 R. Cransberg, K. Munyard (Australia) No evidence of the eumelanic brown phenotype in alpaca (Vicugna pacos)

P5 M. Rachkova, B. Dimitrov (USA) Phenol Oxidase-Tyrosinase Pathway metabolites-possible connection with melanotransferrin MTf,p97 in Melanoma and Alzheimer Disease

P6 A. Pezzella, P. Manini, L. Capelli, A. Napolitano, M. d'Ischia (Italy) Synthetic routes to 5,6-Dihydroxyindole oligomers: a tool for the bottom-up approach to eumelanin stucture

P7 M. J. Simpson, J. Wilson, T. Matthews, S. Degan, W. Warren (USA) Imaging the Distributions of Eumelanin and Pheomelanin in Human Tissue

P8 Y. Niki,T. Hirobe, K. Wakamatsu, H. Ando, M. Yoshida, M. Ichihashi, S. Ito (Japan) Slaty mutation inverses the ratio of DHI to DHICA content of eumelanin in both mouse melanocytes and hair

P9 H. Okuda, T. Sota, K. Koike, K. Monda, T. Nakamura, K. Wakamatsu, S. Ito (Japan) Quantum chemical study of 5,6-dihydroxyindole tetramers as eumelanin model molecules

P10–P13 Non cutaneous melanocytes

P10 S. Uehara, H. Yamamoto (Japan) Characteristic roles for cochlear melanocytes in anti-oxidant responses related to and not related to theirmelanogenesis

P11 S. Julien, A. Biesemeier, D. Kokkinou, O. Eibl, U. Schraermeyer (Germany) Zinc free diet induced a release of melanosomes from choroidal melanocytes and an increase of lipofuscin in the retinal pigment epithelium of rats

P12 D. Nishihara, A. Kawasaki-Nishihara, N. Tsukiji, H. Nakamura, H. Yamamoto (Japan) Involvement of Mitf in the development of retinal pigment epithelium and its possible regulators

P13 M. Sarna, M. Olchawa, A. Pilat, G. Szewczyk, K. Burda, T. Sarna (Poland) Atomic force microscopy analysis of retinal pigment epithelium cells subjected to photodynamic stress

P15–P40 Genetics and Development/Skin colour and UV: in vivo and in vitro studies

P14 B. Sarode, U. Koch, K. Schouwey, L. Larue, V. Delmas, F. Beermann, F. Radtke. (Switzerland) Notch signaling is dispensable for mature melanocytes but essential for melanocyte stem cells

P15 I. Berlin, L. Denat, A.-L. Steunou, I. Puig, D. Champeval, S. Colombo, K. Roberts, E. Bonvin, Y. Bourgeois, V. Delmas, I. Davidson, L. Nieto, C. R. Goding, L. Larue(France) BRN2 phosphorylation regulates melanoblast migration and proliferation through PAX3 and MITF-M

P16 T. Akiyama, A. Shinomiya, K. Kinoshita, M. Mizutani, T. Namikawa, S. Ito, Y. Matsuda (Japan) Endothelin receptor B2 mutation induces the suppression of proliferation and migration of melanoblasts from early embryogenesis in quail and chickens

P17 D. Champeval, S. Colombo, F. Rambow, L. Larue (France) Gene expression profile of murine melanoblasts

P18 K. Menaria (India) Flux balance analysis of melanogenesis pathway

P19 L. Vibert, M. Nikkaido, E. R. Greenhill, R. N. Kelsh (UK) A systems biology approach to in vivo dissection of the gene regulatory network (GRN) underlying melanocyte differentiation in zebrafish

P20 T. Kuramoto, M. Yokoe, T. Serikawa (Japan) Genetic mapping of a rat dominant ventral spotting gene, downunder (Du), to chromosome 3

P21 C. B. Kaelin, X. Xu, L. Z. Hong, V. A. David, K. A. McGowan, G. S. Barsh, M. Menotti-Raymond (USA) Coat pattern genetics in cats

P22 K. McGowan, C. Park, S. Mendrysa, I. Weissman, G. Barsh (USA) Titration of p53 in dark skin mouse mutants causes a spectrum of pigmentary and hematologic phenotypes

P23 Zhang R (China) Transmission electron microscope of fetal scalp melanocytes

P24 Y. Abe, H. Yutaka, T. Gen, T. Suzuki (Japan) Association of the melanogenesis genes with Japanese skin color

P25 E. Mauger, J. Latreille, A. Porcheron, C. Guinot, E. Tschachler, F. Morizot (France) Diversity of skin colour in Indian women

P26 S. Wilson, T. Dadd, F. L. Lim, R. Ginger, M. R. Green (UK) Confirmation that knock down of NCKX5, a gene that regulates natural variation in human skin colour, perturbs lipid and sterol gene expression in human melanocytes

© 2011 The Authors. Journal Compilation © 2011 John Wiley & Sons A/S

P27 T.-K. Kim, Z. Janjetovic, D. L. Peacock, S. N. Tolkachjov, R. M. Slominski, W. Li, T. N. Seagroves, A. T. Slominski (USA) Melanogenesis stimulates HIF-1α expression and accumulation of downstream genes mRNA with attendant changes in cellular metabolism

P28 E. Hacker, Z. Boyce, M. Kimlin, S. Vaartjes, N. Hayward, D. Whiteman (Australia) The proliferative response of melanocytes to sunlight

P29 U. Panich, S. Limsaengurai, T. Onkoksoong, P. Akarasereenont (Thailand) UVA radiation induces melanogenesis through modulation of phase II antioxidant enzymes: The protective effect of gallic acid

P30 C. B. Lin, N. Chen, D. Rossetti, Y. Hu, J. Zhang, P. Bargo, F. Liebel, T. Chen, M. Seiberg (USA) The Effects of visible light and UV exposure on skin pigmentation in vitro

P31 K. Lazoul, A. Soleyan, R. Kurfurst, J.-H. Cauchard (France) Microarray analysis of microRNA modulation in UVB stimulated human melanocytes

P32 H. Ait El Madani, F. Girard, A. Black, L. Gauchet, A. Krief, C. Gomes, H. Nocairi, F. Leroy, P. Sextius, A. Colonna (France) Multiphoton microscopy of pigmented reconstructed epidermis: assessment of 3D pigmentation modulation

P33 C. Herraiz, C. Jiménez-Cervantes, J. C. García-Borrón. (Spain) Role of N-glycosylation in human melanocortin 1 receptor trafficking and function

P34 H. Ando, Y. Niki, M. Ito, K. Akiyama, M. S. Matsui, D. B. Yarosh, M. Ichihashi (Japan) Melanocyte dendrites penetrate through a microporous membrane filter and generate large pigment globules containing multiple melanosomes which transfer to keratinocytes below

P35 T. Strub, D. Koludrovic, I. Davidson (France) Identification and characterisation of the MITF-interactome

P36 J. Debbache, J. Pickel, H. Arnheiter (USA) In vivo role of serine-73 phosphorylation of the transcription factor MITF: Effects on coat color in mice with targeted mutations

P37 S. Ishiwatari, T. Fujita, A. Enomoto, S. Matsukuma (Japan) Melanogenesis mediated by the preservative-induced release of the macrophage migration inhibitory factor in a 3D epidermal model

P38 H. R. Kim, J. Y. Lee, S. Y. Park, H. Y. Kang (Korea)Wnt inhibitory factor (WIF)-1 promotes melanogenesis in normal human melanocytes

P39 S. K. Singh, W. A. Abbas, D. J. Tobin (UK) Bone morphogenetic protein-6 induces melanogenesis and melanin transfer in human skin cells

P40 J. Soong, Y. Chen, E. Terushkin, G. Scott (USA) Sema4D, the ligand for Plexin B1, is a proliferation and survival factor for normal human melanocytes, and down-regulates the activity of c-Met

P41–P47 Albinisms and related

P41 T. Kondo, V. J. Hearing (USA) Subcellular localization of the P protein in human melanocytes

P42 H. Nakajima, S. Koga, T. Nagata, G. Imokawa (Japan) The intracellular trafficking of tyrosinase and tyrosinase-related protein-1 to melanosomes is disrupted independent of the trafficking of dopachrome tautomerase and Pmel17 in reduced glutathione-induced amelanotic B-16 melanoma cells: A model for oculocutaneous albinism type 2

P43 M. Kono, T. Kondo, S. Ito, T. Suzuki, K. Wakamatsu, S. Ito, Y. Tomita (Japan) Oculocutaneous albinism 1 minimal pigment type; a case report on the analysis of genotype-phenotype correlation

P44 A. Rouault, E. Lasseaux, F. Morice-Picard, C. Rooryck-Thambo, D. Cailley, C. Castaing, D. Lacombe, A. Taïeb, B. Arveiler (France) Molecular analysis of the OA1 gene in patients with ocular albinism

P45 E. Lasseaux, F. Morice-Picard, C. Rooryck-Thambo, A. Rouault, C. Plaisant, P. Fergelot, D. Lacombe, B. Arveiler (France) Bioinformatics tools to predict splicing mutation effect in genetic diagnosis of oculocutaneous albinism

P46 V. Baral, B. Duriez, Y. Watanabe, M. Goossens, T. Attie-Bitach, V. Pingault, N. Bondurand (France) Screening of SOX10 and MITF regulatory regions in Waardenburg syndrome

P47 S. Léger, X. Balguerie, A. Goldenberg, V. Drouin-Garraud, A. Cabot, I. Amstutz-Montadert, P. Young, P. Joly, M. Goossens, V. Pingault (France) A novel non-truncating mutation of the MITF basic domain in an atypical form of type II Waardenburg syndrome

P48–P86 Vitiligo and related

P48 D. N. W. Liyanage (Sri Lanka) Clinical and Epidemiological study of Vitiligo

P49 J.-H. Park, J.-H. Lee, D.-Y. Lee (Republic of Korea) Clinical course of segmental vitiligo: a retrospective study of 88 patients

© 2011 The Authors. Journal Compilation © 2011 John Wiley & Sons A/S

P50 C. Muteba Baseke (Congo) Epidemiology of vitiligo in university of Kinshasa Hospital (C.U.K)/D.R.Congo

P51 S. Shan-Yi Ng, L. Hwee-Ying Teo (Singapore) Pseudoleukoderma Angiospasticum: 2 cases

P52 S. G. Krishna, M. Ramam, M. Mehta, V. Sreenivas, V. K. Sharma, S. Khandpur (India) A study of burden of vitiligo in Indian patients using a new and specific rating scale

P53 S. Kumar, T. Kaur, B. B. Mahajan, R. Singh (India) Vitiligo with raised and Inflammatory borders – A rare case report from North India

P54 M. Arunachalam, R. Colucci, R. Conti, S. Berti, F. Lotti, S. Pallanti, T. Lotti, S. Moretti (Italy) Autoimmune signals in vitiligo patients appear correlated with obsession and phobia

P55 N. Oiso, K. Fukai, T. Narita, K. Kabashima, A. Kawada, T. Suzuki (Japan) Generalized vitiligo and related autoimmune disorders in Japanese patients and their families

P56 B. K. Khaitan, D. Seshadri, S. Kathuria, N. Gupta, M. Ramam, V. K. Sharma (India) Prevalence of co-existent organ-specific (TPO) and non-organ specific (ANA) autoantibodies in patients with segmental vitiligo vs non-segmental vitiligo: a case-control study

P57 M. Abdallah, R. Lotfi, W. Osman, R. Galal (Egypt) Assessment of Tissue FoxP3+, CD4+ and CD8+ T-cells in Active and Stable Non-Segmental Vitiligo

P58 R. Speeckaert, N. Van Geel, R. Luiten, M. Van Gele, M. Speeckaert, J. Lambert, K. Vermaelen, E. Tjin, L. Brochez (Belgium) Evidence for a local and systemic immune reaction against melanocyte differentiation antigens in a patient with regressing nevi without halo

P59 N. Van Geel, R. Speeckaert, I. Mollet, S. De Schepper, E. Tjin, R. Luiten, L. Brochez, J. Lambert (Belgium) New in vivo vitiligo induction and therapy model: proof of concept

P60 S. Kerje, Weronika Ek, A.-S. Sahlqvist, O. Ekwall, G. Erf, Ö. Carlborg, L. Andersson, O. Kämpe (Sweden) Genetic mapping of loci underlying vitiligo in the Smyth Line chicken model

P61 R. Conti, R. Colucci, M. Arunachalam, S. Berti, S. Moretti (Italy) Vitiligo: there is more than meets the eye

P62 M. Ivaniciuc (Romania) Coexistence of vitiligo and psoriasis-report of three cases

P63 V. Eleftheriadou, K. Thomas, M. Whitton (UK) What outcomes are important to patients and clinicians: survey results

P64 L. Benzekri (Morocco) A simple index of potential repigmentation in vitiligo

P65 L. S. Abraham, M. C. Costa, A. Pacifico, G. Leone, M. Picardo, M. Ardigò (Brazil) New reflectance confocal microscopy features in vitiligo: beyond the papillary rings

P66 T. Shibata, A. Sasase, C. Hihiro Honda, K. Hayashibe (Japan) The evaluation of our recent therapies of vitiligo vulgaris

P67 M. Phiske, B. Patil, Z. Bharda, H. Jerajani (India) Tacrolimus versus pimecrolimus in localised stable vitiligo

P68 P. Araujo, M. Fabrini (Brazil) Surgery and laser treatment of vitiligo

P69 A. Rao, S. Gupta, V. K. Sharma (India) Determinants of success of melanocyte transplantation in vitiligo: Role of cytotoxic CD8 Tcells

P70 A. P. Holla, D. Parsad, A. J. Kanwar, S. D. Mehta (India) Melanocyte transplantation outcome (metro) scoring to assess the outcome of non cultured epidermal suspension transplantation in vitiligo

P71 A. Budania, D. Parsad, A. J. Kanwar, S. Dogra (India) Comparison between autologous non-cultured epidermal cell suspension and suction blister epidermal grafting in stable vitiligo: a randomized study

P72 R. Batra (India) To compare the outcome of minipunch grafting & suction blister epidermal grafting alongwith postsurgical application of clobetasol propionate 0.05% cream in patients of stable vitiligo

P73 B. S. Daniel, S. S. Venugopal, L. K. Martin, A. L. Agero, L. M. Rhodes, J. W. Frew, R. Wittal, J. Le Guay, D. F. Murrell (Australia) A Randomised Controlled Trial assessing the effectiveness of Minigrafting Vs ReCell in Stable Vitiligo: preliminary results

P74 M. Pascal, L. Valente (France) Treatment of vitiligo hands by ReCell system associated with Excimer lamp

P75 A. P. Holla, R. Kumar, D. Parsad, A. J. Kanwar, S. D. Mehta (India) Role of wound bed nutrition in non cultured epidermal suspension transplantation in vitiligo

P76 H.-K. Lim, M.-K. Shin, M.-H. Lee (Korea) Clinical application of platelet-rich plasma in vitiligo: a pilot study

P77 E. Y. Gan, L. Y. T. Chiam, N. Van Geel, B. K. Goh (Singapore) Repigmentation of leukotrichia in vitiligo using non-cultured cellular grafting

P78 S. Singh, S. Khandpur, V. K. Sharma, M. Ramam (India) Comparison of efficay and side effect profile of oral PUVA versus oral PUVA sol in the treatment of vitiligo: a 36 week prospective study

© 2011 The Authors. Journal Compilation © 2011 John Wiley & Sons A/S

P79 K. Kikuchi, K. Wakamatsu, Y. Tada, S. Ito (Japan) Serum 5-S-cysteinyldopa levels in psoriasis and vitiligo patients undergoing narrowband ultraviolet B phototherapy

P80 D. Keswell, L. M. Davids, S. H. Kidson (South Africa) Novel aspects of melanocyte – keratinocyte interactions in vitro as a clue towards repigmentation in vitiligo

P81 V. Mendiratta, J. Mal (India) Study of oxidative stress in vitiligo

P82 J. Y. Shin, J. Y. Kim, J. E. Do, M. R. Kim, S. H. Oh (Korea) Decreased isocitrate dehydrogenase expression renders melanocytes more vulnerable to oxidative stress

P83 E. Jung, S. Kim, M. Kim, S. Shin, J. Lee, D. Park (Korea) BSP-1 protects melanocytes against oxidative stress-induced cell death and hypopigmentation through MITF upregulation

P84 A.-S. Ricard, D. El Hajj Diab, C. Pain, A. Daubos, K. Ezzedine, A. Bibeyran, V. Guyonnet-Dupérat, A. Taïeb, M. Cario-André (France) Study of CCN3 (Nov) expression in normal melanocytes and vitiligo skin

P85 K Ezzedine, J Marie, D Kovacs, T Jouary, M Picardo, A Taieb, M Cario-André (France & Italy): Inflammasome activation and nonsegmental vitiligo progression

P86 L. Benzekri (Morocco) Is there a clinicopathologic correlation between clustered T8 lymphocytes infiltrate of the perilesional margin and the clinical aspect of vitiligo patches?

P87–P90 Hair and premature graying

P87 M. Giesen, T. Goerlach, S. Gruedl, G. Fuhrmann, M. Briese, G. Scheel, R. Paus, D. Petersohn, T. Förster (Germany) Plucked Hair Follicles as a powerful tool to monitor pigmentation markers

P88 C. Gondran, A. Perrin, C. Meyrignac, C. Dal Farra, N. Domloge (France) A new approach to preserve melanin content in the hair follicle

P89 E. Reyes-Gomez, N. da Silva, S. Gadin-Czerw, J.-J. Panthier, G. Aubin-Houzelstein (France) Characterization of progressive hair whitening and roan coat colour phenotypes associated with overexpression of Strawberry Notch homolog 2 gene in the melanocyte lineage

P90 T.-C. Lei (China) Reduced scavenging abilities of premature graying hair bulbs against hydroxyl free radicals: Direct evidence from an electron spin resonance (ESR) study

P91–P119 Focal hyperpigmentations and depigmenting agents

P91 A. Porcheron, J. Latreille, R. Jdid, C. Guinot, E. Tschachler, F. Morizot (France) Different contributions of pigmented spots in age and attractiveness perception: a cross-cultural approach

P92 R. Jdid, E. Mauger, A. Porcheron, C. Guinot, E. Tschachler, F. Morizot (France) Solar lentigines: an earlier occurrence in Japanese women than in Chinese and Korean women

P93 E. Noblesse, P. Schaeffer, R. Kurfurst, C. Nizard, S. Schnebert, E. Perrier (France) Alteration of epidermis junctions in human Solar Lentigo

P94 S. Socha, N. Pauloski, J. Huertas, B. Potterf, W. Lathrop, C. Bosko, H. Meldrum (USA) Insights into the etiology of solar lentigines through its microRNA and mRNA profile

P95 S. Thang, H. Ranu, A. Burger, B. K. Goh, C. L. Goh (Singapore) Periorbital hyperpigmentation amongst the Singaporean population: a proposed classification and epidemiological review

P96 G. Smita (India) Study of pigmented cosmetic dermatitis

P97 A. Dandale, S. Chavan, R. Dhurat (India) Patch test in facial melanosis

P98 A. Salhi, F. Siebenhaar, M. Maurer, A. Taïeb (Algeria) Idiopathic eruptive macular pigmentation in a 9-year-old girl

P99 J. Nakayama, T. Mori, S.-I. Imafuku (Japan) Narrow-band UVB may improve pigmented spots in patients with neurofibromatosis 1

P100 L. Larribère, X. Nissan, M. Saidani, C. Baldeschi, M. Pechanski (France) Neurofibromatosis type I in vitro model using human embryonic stem cells

P101 C. J. Park, H. J. Lee, H. S. Kim, J. Y. Lee, H. O. Kim, Y. M. Park (Korea) Linear and Whorled Nevoid Hypermelanosis and Progressive Cribriform and Zosteriform Hyperpigmentation in Korea

P102 N. Saedi, A. Ganesan (USA) Treating Hyperpigmentation in Dark Skinned Patients

P103 A. J. Kanwar, D. Parsad (India) Colchicine in the treatment of Lichen Planus Pigmentosus

P104 H. S. Park, H. H. Cho, S. Cho, J. H. Lee (Korea) Oral tranexamic acid with laser treatments in melasma patients

 © 2011 The Authors. Journal Compilation © 2011 John Wiley & Sons A/S

P105 A. W.-M. Tan, P. Sen, S.-H. Chua, B.-K. Goh (Singapore) Oral Tranexamic Acid in the Treatment of Melasma Refractory to Topical Therapy

P106 N. Puri (India) Comparative study of 15% TCA peel versus 35% glycolic acid peel for the treatment of melasma

P107 R. Sarkar, R. K. Jain (India) Salicylic acid peels in the treatment of melasma

P108 G. Sunil, D. Ghate, S. T. Amladi, H. R. Jerajani (India) Utility of glycolic acid peels in melasma & comparison of 55% glycolic and with hydoquinone (4%) cream in melasma

P109 K. Godse (India) Comparative efficacy and safety of mometasone based triple creams v/s fluocinolone based triple creams in melasma in Indian patients

P110 J. W. Shin, S. Y. Choi, K. C. Park (Korea) The ratio of lesional/non-lesional melanin index; a sensitive parameter for the evaluation of skin lightening agents

P111 M. Son, D. Jung, W.-Y. Choi, E. Kim (Korea) Inhibition of Mitf-E box binding and its effect on pigmentation in Melan-a cells

P112 M. Okura, K. Hagiwara, T. Hida, A. Yoneda, K. Yanagisawa, Y. Horio, T. Yamashita (Japan) Effects of a low-molecular-weight polyphenol (oligonol) on the growth and melanogenesis of primary melanocytes and melanoma cells

P113 M. Y. Kim, J. H. Lee, J. Yoon, K. H. Kim, J. S. Hwang, J.-H. Lee, T.-J. Yoon (Korea) Depigmentation by keratinocyte-derived, Wnt inhibitor sFRP2

P114 H.-S. Jeong, S. Y. Kim, H. Li, H.-Y. Yun, K. J. Baek, N. S. Kwon, K.-C. Park, D.-S. Kim (Korea) Sphingosylphosphorylcholine modulates melanin synthesis via various signal transduction pathways

P115 B. Choi, O. Makoto, A. Kanamoto, T. Fujiwara, H. Nakajima, G. Imokawa (Japan) Arenarol isolated from marine sponge abrogates endothelin-1-stimulated expression of tyrosinase by interrupting intracellular MAPK signaling pathway in normal human melanocyte

P116 J. S. Hwang, H. Y. Lee, T.-Y. Lim, T.-J. Yoon, K-Y Nam (Korea) Inhibitory effects of NAG on pigmentation

P117 T. Niwano, H. Nakajima, Y. Wakabayashi, G. Imokawa (Japan) Paracrine interaction interaction between UVB-exposed human keratinocytes and human melanocytes leading to an increased expression of tyrosinase and its blockade by Wetherferin A

P118 T. Kato, H. Nakajima, Y. Wakabayashi, G. Imokawa (Japan) Glucosamine, an asparagin-linked carbohydrate core synthesis inhibitor attenuates endothelin-1+stem cell factor-stimulated expression of melanocyte-specific proteins by down-regulating CREB activation in human melanocytes

P119 M. Cario-André, Y. Gauthier, S. Lepreux, C. Pain, A. Taïeb (France) Influence of estrogens on melanosome distribution in keratinocytes: An ultrastructural study on irradiated skin organ culture

P120–P125 Photoprotection/antioxidants/others

P120 A. Bouafia, S. Corre, N. Mouchet, M. D. Galibert (France) USF1 modulates in vivo skin cell proliferation arrest and DNA damage repair in response to UVB

P121 J. Lee, K.-B. Roh, J. Lee, D. Park (Korea) Protective Effects of BSP-2 on UVB-induced Senescence in Human Keratinocytes

P122 H. Song, H. Kim, G. Choi, J. Shin (Korea) Repeated ultraviolet exposure induces TLR4 expression of neonatal human melanocytes

P123 W. Merdekawati, A. B. Susanto (Indonesia) Antioxydant activity of seaweed biopigments and the potency for human skin protector

P124 A. Von Koschembahr, R. Starner, J. Jameson, V. Swope, Z. Abdel-Malek (USA) A-Melanocyte stimulating hormone enhances nucleotide excision repair in human melanocytes by activating the transcription factor ATF2

P125 K.-B. Roh, J. Lee, J. Lee, D. Park (Korea) Inhibition of eotaxin-1/CCL11 expression by novel compound in mouse embryonic fibroblast

P126–P 171 Melanoma & related

P126 M. Loot, P. Vergnes, A. Taïeb (France) Treatment of giant congenital melanocytic nevus : pediatric skin expansion in infants

P127 S. Rice, A. Fityan, M. Carpenter, L. Vearncomb, J. Baird, E. Healy (UK) Vitamin D levels and ultraviolet radiation exposure; upon what basis do we increase melanoma risk?

© 2011 The Authors. Journal Compilation © 2011 John Wiley & Sons A/S

P128 A. Capper (UK) Genetic variation in zebrafish melanoma

P129 M. Ibarrola-Villava, M. Peña-Chilet, M. Mayor, C. Gomez-Fernandez, B. Casado, M. Martin-Gonzalez, A. Lluch, G. Ribas (Spain) GSTs genes and genetic susceptibility to melanoma

P130 M. Peña-Chilet, M. Ibarrola-Villava, M. Martin-Gonzalez, C. Gomez-Fernadez, B. Casado, M. Mayor, A. Lluch, G. Ribas (Spain) Role of GC transporter and VitD receptor genes on melanoma susceptibility

P131 J. Corominas, J. Estellé, Y. Ramayo-Caldas, M. Lathrop, F. Demenais, C. Rogel-Gaillard, S. Vincent-Naulleau, J.-M. Folch, E. Bourneuf (France) Detection of CNVs throughout the genome of a porcine melanoma model

P132 A. Manjare, P. Pund, S. Tambe, S. Ghate, R. Dhurat (India) Acral lentiginous melanoma

P133 S. Norrenberg, V. Del Marmol, M. Candaele, M. Abramowicz, A. Daubos, C. Ged (Belgium) Xeroderma Pigmentosum type C: report of a case with multiple melanomas

P134 A. Bonthuys, G. Todd, G. Govender, S. H. Kidson (South Africa) The Molecular Phenotype of Acquired Melanocytic Naevi

P135 K. Meissl, K. Terlaak, D. S. Peeper (The Netherlands) Genome-wide shRNA screen for tumor suppressors mediating oncogene-induced senescence

P136 T. Hoashi, S. Sato, Y. Yamaguchi, T. Passeron, K. Tamaki, V. J. Hearing (Japan-USA) Glycoprotein nonmetastatic melanoma protein b (GPNMB) is a melanosome-specific cell marker and is proteolytically released by ectodomain shedding

P137 H. Fujiwara, M. Ito (Japan) Analysis of global 5-hydroxymethylcytosine in malignant melanoma and acquired melanocytic nevi

P138 I. Vaisnoriene, J. Venius, J. Didziapetriene, R. Rotomskis, K. P. Valuckas (Lithuania) Atypia grading in nevi by reflectance confocal microscopy

P139 R. Nkosi, S. Meyer, N. Martin, R. Barnhill, M. Battistella, F. Bernex, G. Houzelstein, A. Janin, X. Sastre-Garau, J.-J. Panthier, C. Lebbé, G. Egidy (France) Diagnostic interest of RACK1 in melanoma

P140 C. Campagne, S. Julé, S. Bibi, C. Koenen, E. Reyes-Gomez, F. Bernex, S. Pons, U. Maskos, J. J. Panthier, G. Aubin-Houzelstein, G. Egidy (France) RACK1 is a marker and a catalyst in melanoma development

P141 R. Hsieh, N. Mms, M. Buim, L. Sv (Brazil) Study of mapk pathway components – Ras, Braf, Mek 1/2 and Erk 1/2 in series of 35 cases primary oral mucosal melanoma

P142 N. Yamazaki, A. Tsutsumida, K. Namikawa, Y. Kiyohara (Japan) The significance of micrometastases in sentinel nodes in Japanese melanoma patients: a retrospective analysis of 450 cases

P143 M. Ziman, M. Millward, S. Medic, A. Reid, J. Freeman, R. Pearce, M. Lee, P. Heenan, A. Ireland, P. Kumarasinghe (Australia) Detection, Quantification and Characterisation of PAX3 across the Spectrum from Melanocytes to Melanoma and in Circulating Melanoma Cells Relative to Disease Stage

P144 K. Yoshio, O. Dai, N. Michiko (Japan) Real-time Tissue Elastography is useful for detecting lymph-node metastases in melanoma

P145 A. Yoneta, Y. Tamura, S. Nohara, A. Ito, H. Honda, K. Wakamatsu, S. Ito, T. Yamashita, K. Jimbow (Japan) Development and evaluation of antitumor effect of novel NPrCAP-magnetite nanoparticles for chemo-thermo-immunotherapy in malignant melanoma

P146 T. Z. Xiao, N. Bhatia, R. Urrutia, G. A. Lomberk, A. Simpson, B. Jack (USA) MAGE Proteins Are Master Regulators of KAP1 and KRAB Domain Zinc Finger Transcription Factor Mediated Gene Suppression

P147 Y. Ishii-Osai, T. Yamashita, M. Okura, Y. Tamura, N. Sato, A. Ito, H. Honda, K. Wakamatsu, S. Ito, K. Jimbow (Japan) N-Propionyl-4-S-Cysteaminylphenol Generates Reactive Oxygen Species and Mediates Apoptosis in Pigmented Melanoma Cells

P148 F. Liu, A. Garcia, F. L. Meyskens (USA) Expression of NADPH Oxidase 1 in Melanoma Cells and its effect on Invasion via induction of Matrix Metalloproteinase-2

P149 Z. S. Pavicevic, R. I. Krutilina, A. R. Chatterjee, C. D. Duntsch, T. N. Ignatova, V. G. Kukekov (USA) MDA MB 435, SKMEL Pigmented and Nonpigmented Melanoma Cell Lines and MDA MB 231 Cancer Cell Line – Derived Cancer Stem Cells (CSC) Show Differential Expression of Green Fluorescent Protein Driven by Oct4 Promoter in Non-Green vs Green Populations Determined by FACS

P150 Z. Janjetovic, A. A. Brozyna, R. C. Tuckey, M. N. Nguyen, S. R. Pfeffer, L. M. Pfeffer, W. Jozwicki, A. T. Slominski (USA) Active forms of vitamin D3 inhibit melanoma growth and are dependant on the activity of NF-kB pathway

P151 L. Xie, F. Liu, A. Garcia, F. Meyskens (USA) Aurora Kinases Play a Critical Role in Hexavalent Chromium-induced Aneuploidy in Immortalized Human Melanocytes

© 2011 The Authors. Journal Compilation © 2011 John Wiley & Sons A/S

P152 D. Koludrovic, T. Strub, I. Davidson (France) Identification of MITF regulated genes involved in melanoma proliferation, migration and invasion

P153 T.-K. Kim, J. Wang, J. Chen, R. C. Tuckey, M. N. Nguyen, D. Miller, W. Li, A. T. Slominski (USA) Correlation between secosteroid induced VDR activity in melanoma cells with computer modeling based on the receptor crystal structure

P154 M. B. Weiss, A. E. Aplin (USA) TWIST1, a B-RAF effector, promotes invasion of melanoma cells

P155 M. Nihal, C. K. Singh, M. Ndiaye, G. S. Wood, N. Ahmad (USA) SIRT1 histone deacetylase is a potential therapeutic target for human melanoma

P156 B. Belloni, P. Cheng, D. Widmer, N. Schönewolf, K. S. Hoek, R. Dummer, O. Eichhoff (Switzerland) Phenotype-specific response of melanoma cells to HDAC inhibition

P157 T. Nishizaka (Japan) Re-expression of epigenetically silenced miRNAs is associated with anti-tumor effects on melanoma cells

P158 Y. Arroyo-Berdugo, P. Cheng, S. Alonso, K. S. Hoek, M. D. Boyano (Spain) CpG island methylation and gene silencing in melanoma

P159 P. Cheng, D. Widmer, O. Eichhoff, B. Belloni, R. Dummer, K. S. Hoek (Switzerland) DNA Methylation Patterns in Melanoma Phenotype Switching

P160 P. Zanna, I. Maida, C. Grieco, S. Guida, N. Cassano, G. A. Vena, A. Naspi, P. Londei (Italia) Eukaryotic initiation factor eIF2-alpha in melanoma

P161 K. Ivanova, P. Eiermann, W. Tsiockas, I. Block, R. Hemmersbach, R. Gerzer (Germany) Cyclic GMP-Signaling Associated Gene Expression in Human Melanoma Cells in Altered Gravity: Down-Regulation in SimulatedWeightlessness

P162 F. Silvy, D. Lombardo, P. Verrando (France) Activity of organic anion transporting polypeptides (OATP) in melanoma cells generates a trans-resistance signal to cisplatin-induced cell death through glutathione and protein kinase C (PKC)-linked mechanisms

P163 D. Zingg, O. Shakhova, L. Sommer (Switzerland) Mechanisms controlling melanoma initiation and progression

P164 M. L. Fontsa, M. Wiedig, R. Morandini, F. Sales, A. Awada, G. Ghanem, F. Journe (Belgium) Pre-treatment of melanoma cells with a first protein kinase inhibitor sensitizes cells to a second protein kinase inhibitor: a rationale to combine targeted drugs

P165 E. Alonso-Tejerina, F. Nicolau-Galmés, Y. Arroyo-Berdugo, G. Pérez-Yarza, A. Asumendi, M. D. Boyano (Spain) Involvement of autophagy in the apoptosis induced by Terfenadine, an H1 histamine receptor antagonist, in human melanoma cells

P166 N. Weiß, A. Kokot, T. A. Luger, C. Weishaupt, M. Böhm (Germany) Subtilisin-kexin isoenzyme-1-a novel player in melanoma biology

P167 A. Marzia, I. Pshenichnaya, A. Trumpp, L. Larue, S. Gallagher, F. Beermann, F. Radtke (Switzerland) Role of myc in melanoma

P168 M. Krayem, M. Berehab, M. Wiedig, R. Morandini, F. Sales, A. Awada, F. Journe, G. Ghanem (Belgium) MAPK inhibitors may reverse the senescence-like phenotype associated with a low proliferation index of melanoma cells bearing the V600EBRAF mutation

P169 I. Ortega-Martínez, J. Gardeazabal, R. Fernandez-Suarez, E. Alonso-Tejerina, J. M. Careaga, J. L. Díaz-Ramón, R. Izu, A. Asumen, M. D. Boyano (Spain) Serum Amyloid A, Clusterin and Apolipoprotein A-I serum levels related to metastatic progression in melanoma patients

P170 J. Wangari-Talbot, B. A. Wall, J. Goydos, S. Chen (USA) GRM1: A Therapeutic Target in Melanoma

P171 M. Böhm, A. Mastrofrancesco, N. Weiss, B. Kemper, G. Von Bally, M. Picardo, T. A. Luger, C. Weishaupt (Germany) Paired basic Amino-acid-Cleaving Enzyme 4 (PACE4) increases metabolic activity, proliferation, migration and collagenase expression of human melanoma cells in vitro and confers increased subcutaneous tumor growth in vivo

P172 L. M. Davids, K. V. Sharma (South Africa) Inhibition of melanogenesis increases the efficacy of photodynamic therapy-induced cell death in melanoma cells

© 2011 The Authors. Journal Compilation © 2011 John Wiley & Sons A/S

Abstracts of Special and Named Lectures

SL1/Special Lecture
Twenty-five years of the ESPCR

J. Borovanský, P. A. Riley, G. Ghanem
Charles University, 1st Faculty of Medicine, Institute of Biochemistry and Exptl. Oncology, Prague, Czech Republic

Unlike the USA, where International Pigment Cell Conferences were regularly organized since 1946, reaching Europe only in 1965 (Sofia), the European scientists engaged in pigment cell and melanin research lacked a specialized forum to present and discuss their results and were limited to morphological, zoological, veterinary, dermatological and oncological meetings. The hunger for specialized interdisciplinary meetings induced the organization of regular European Workshops on Melanin Pigmentation (1976 Lyon, 1979 London, 1981 Prague, 1982 Edinburgh, 1984 Marseille and 1985 Murcia). The success of those meeting (due to the interdisciplinary character, and the friendly mood of the wide-ranging discussions) attracting not only European but also many non-European participants and led naturally to the proposal that a European Society for Pigment Cell Research should be formed. At the Murcia European Workshop meeting a formal session was scheduled at which the pros and cons of setting up a European Society were discussed and the formation of the ESPCR was approved. The initial Constitution was duly sworn before a Notary Public in Naples on 11th December 1985, which can be considered the birthday of the ESPCR. The first Council, called the Steering Committee at that time, undertook the first call for membership and was charged with running the society until official elections, which took place in Sorrento in 1987. The first Council was composed of a President: Giuseppe Prota (Naples), a Secretary and Treasurer: Patrick Riley (London), and Members: Fritz Anders (Giessen), Natale Cascinelli (Milan), Ferdy Lejeune (Brussels), José Antonio Lozano (Murcia), and Hans Rorsman (Lund). The initial letter of invitation to become a member stated that the purpose of the Society was 'to promote interdisciplinary knowledge in the fields of physics, chemistry, biology and medicine concerned with pigment cells, pigmentation and associated processes in man and other living organisms'. This broad aim remains true today. Regular European scientific meetings have been organized ever since the inception of the ESPCR. In the last century there were meetings in Sorrento (1987), Uppsala (1989), Amsterdam (1991), Berlin (1992), Vienna (1994), Lausanne (1995), Bordeaux (1997), Prague (1998), and Ulm (2000). The special lecture will touch on important personages and historically significant topics popular in the last century, and will include brief recollections of the events associated with the official foundation of the ESPCR, the history of the ESPCR Bulletin (launched in March 1987), and the initiation of the Society's website. Supported by IGA MZ NT 11229-3.

SL2/IFPCS Presidential Lecture
Pigment production for maintaining epidermal homeostasis: lessons from keratinocytes and melanocytes

S. Shibahara, K. Takeda
Department of Molecular Biology and Applied Physiology, Tohoku University School of Medicine, Sendai, Japan

The skin is a unique organ that is covered with the stratum corneum and is continuously exposed to external stressful environments, such as atmospheric oxygen and solar radiations. These environmental factors may influence the homeostasis of keratinocytes and melanocytes by changing the production of oxidative stress-protective substances, such as bilirubin and melanin. Heme oxygenase-1 (HO-1) is an essential enzyme in heme catabolism and cleaves heme to form biliverdin, iron, and carbon monoxide. Biliverdin is immediately reduced to bilirubin, a physiological radical scavenger. HO-1 is expressed in all types of skin cells, including keratinocytes and melanocytes. In fact, bilirubin is accumulated in the stratum corneum in normal human skin, indicating the degradation of heme in keratinocytes. Keratinocytes migrate toward the surface of the skin and gradually lose their nuclei to form the stratum corneum. It is therefore conceivable that keratinocytes may contribute to the preservation of iron. On the other hand, lipocalin-type prostaglandin D synthase (L-PGDS) is the enzyme that catalyzes the synthesis of prostaglandin D2 (PGD2) and is expressed in melanocytes, but not in keratinocytes. PGD2 and its metabolites are known to induce HO-1 expression. Importantly, L-PGDS is actively secreted to the extracellular space and also functions as a transporter for lipophilic ligands, including bilirubin and all-trans retinoic acid (RA). We have provided evidence that L-PGDS expression may regulate the proliferation activity of the melanocyte-lineage cells through RA. The human skin therefore contains the regulatory network for production of melanin and bilirubin, thereby maintaining epidermal homeostasis.

SL3/Seiji Memorial Lecture
Thirty years of vitiligo genetics

R. Spritz
University of Colorado School of Medicine, Aurora, CO, USA

Generalized vitiligo (GV) is a common multifactorial, polygenic autoimmune disease in which melanocyte loss results in patchy depigmentation of skin and hair, very often associated with concomitant occurrence of other autoimmune diseases. Over the past three decades, a series of evolving approaches has been undertaken to identify GV susceptibility genes, with the goal of elucidating the underlying pathobiology. The earliest approach was testing of biological candidate genes for genetic association or genetic linkage, which resulted in some successes but principally false-positives. The subsequent approach applied genomewide linkage studies of multiplex families, which identified several additional loci. Most recently, genomewide association studies (GWAS) of GV have been carried out in several different ethnic groups, resulting in the identification or confirmation of at least 16 different GV susceptibility loci: HLA class I, HLA class II, PTPN22, RERE, LPP, FOXP1, TSLP, CCR6, IL2RA, TYR, GZMB, NLRP1, UBASH3A, and C1QTNF6, XBP1, and FOXP3. All of these except TYR encode immunoregulatory proteins, and many are also genetically associated with other autoimmune diseases. TYR encodes tyrosinase, the key enzyme of melanogenesis and a major vitiligo autoantigen, and is the gene defective in classical oculocutaneous albinism, type 1 (OCA1). Even TYR appears to contribute to GV by mediating recognition of melanocytes by the immune system, via antigenic presentation by HLA-A2. Moreover, with respect to TYR, susceptibility to GV and to malignant melanoma appear to be opposite, suggesting that GV may represent dysregulation of immune surveillance of melanoma. Some GV susceptibility loci contribute to disease in multiple populations, whereas others appear to be population-specific. While it is likely that additional GV susceptibility loci remain to be discovered, together these findings clearly establish that GV is an autoimmune disease, increase our understanding of disease pathogenesis, and perhaps provide clues that may result in new or better approaches to diagnosis and treatment of the disease.

 © 2011 The Authors. Journal Compilation © 2011 John Wiley & Sons A/S

SL4/PASPCR Aaron Lerner Lecture

Signalling and transcription in melanoma stem-like cells

C. R. Goding
Ludwig Institute for Cancer Research Oxford University, Oxford, UK

Tumours comprise multiple phenotypically distinct subpopulations of cells, some of which are proposed to possess stem cell-like properties, being able to self-renew, seed and maintain tumours, and provide a reservoir of therapeutically-resistant cells. Here we use melanoma as a model to explore the validity of the cancer stem cell hypothesis in the light of accumulating evidence that melanoma progression may be driven by phenotype-switching triggered by genetic lesions that impose an increased sensitivity to changes in the tumour microenvironment. Although at any given moment cells within a tumour may exhibit differentiated, proliferative or invasive phenotypes, an ability to switch phenotypes implies that most cells will have the potential to adopt a stem cell-like identity. Insights into the molecular events underpinning phenotype-switching in melanoma highlight the close relationship between signalling pathways that generate, maintain and activate melanocyte stem cells with those underpinning melanoma stem-like cells, and the inverse correlation between proliferation and invasive potentials. An understanding of phenotype-switching in melanoma, and in particular the signalling events that regulate the expression and activity of the Microphthalmia-associated transcription factor Mitf, points to new therapeutic opportunities aimed at eradicating therapeutically resistant stem cell-like melanoma cells.

SL5/Fitzpatrick Lecture

Functionally distinct melanocyte populations revealed in mice: noncutaneous and dermal melanocytes versus epidermal melanocytes

T. Kunisada
Gifu University Graduate School of Medicine, Gifu, Japan

Unlike the thoroughly investigated melanocyte population in the hair follicle of the epidermis, the growth and differentiation requirements of the melanocytes in the eye, harderian gland and inner ear – the so-called non-cutaneous melanocytes – remain unclear. By using hair follicle reconstitution analysis, we confirmed the inability of dermal or non-cutaneous melanocytes to be involved as follicular melanocytes to regenerating hair follicles during hair reconstitution assay. To investigate a molecular basis of the functional difference, we investigated the in vitro and in vivo effects of the factors that regulate melanocyte development on the stem cells or the precursors of these non-cutaneous melanocytes. Melanocytes in the eye, ear and harderian gland were revealed to be less sensitive to KIT signaling than cutaneous melanocytes. Instead, melanocytes in the eye and harderian gland were stimulated more effectively by endothelin 3 (ET3) or hepatocyte growth factor (HGF) signals than by KIT signaling, and the precursors of these melanocytes expressed the lowest amount of KIT. In transgenic mice induced to express ET3 or HGF in their skin and epithelial tissues from human cytokeratin 14 promoters, the survival and differentiation of non-cutaneous and dermal melanocytes, but not epidermal melanocytes, were enhanced, apparently irrespective of KIT signaling. Thus revealed clear discrimination between non-cutaneous or dermal melanocytes and epidermal melanocytes might be important in the pathogenesis of melanocyte-related diseases and melanomas.

Abstracts of Guest Lectures (GL)

GL1

Deciphering gene regulatory interactions controlling neural crest formation

T. Sauka-Spengler
Weatherall Institute of Molecular Medicine, University of Oxford, Oxford, UK

The neural crest is a multipotent stem cell-like population that gives rise to a wide range of derivatives in vertebrate embryo, including elements of craniofacial skeleton and peripheral nervous system, as well as melanocytes. Neural crest cells form in a series of regulatory steps that include induction and specification of the prospective neural crest territory at neural plate border, followed by specification of bona fide neural crest progenitors and their subsequent differentiation into diverse derivatives. These individual processes during neural crest ontogeny are orchestrated by a hierarchical gene regulatory network (GRN) comprised of a series of transcriptional circuits. I will present an overview of the current representation of neural crest GRN, inferred from gene perturbation studies performed in several vertebrate model organisms. Additional resolution is provided by interrogating direct regulatory interactions via cis-regulatory analyses. In addition to increasing the resolution of the architectural circuitry within the network, our analyses have permitted us to address the contribution/co-operation of key chromatin remodeling events to the regulatory process controlling early neural crest formation.

GL2

Human hair follicle pluripotent stem (hfPS) cells for regenerative medicine: an advantageous alternative to ES and iPS cells

R. M. Hoffman
AntiCancer, Inc. & University of California, San Diego, CA, USA

The optimal source of stem cells for regenerative medicine is a major question. Embryonic stem (ES) cells have shown promise for pluripotency but have ethical issues and potential to form teratomas. Pluripotent stem cells have been produced from skin cells by either viral-, plasmid- or transposon-mediated gene transfer. These stem cells have been termed induced pluripotent stem cells or iPS cells. iPS cells may also have malignant potential and are inefficiently produced. Embryonic stem cells may not be suited for individualized therapy, since they can undergo immunologic rejection. To address these fundamental problems, our group is developing hair follicle pluripotent stem (hfPS) cells. We have shown that mouse hfPS cells can differentiate to neurons, glial cells in vitro, and other cell types, and can promote nerve and spinal cord regeneration in vivo. hfPS cells are located above the hair follicle bulge in what we have termed the hfPS cell area (hfPSA) and are nestin positive and keratin 15 (K-15) negative. Human hfPS cells can also differentiate into neurons, glia, keratinocytes, smooth muscle cells, and melanocytes in vitro. Human hfPS cells were transplanted in the severed sciatic nerve of the mouse where they differentiated into glial fibrillary-acidic-protein (GFAP)-positive Schwann cells and promoted the recovery of pre-existing axons, leading to nerve generation. The regenerated nerve recovered function and, upon electrical stimulation, contracted the gastrocnemius muscle. The hfPS cells can be readily isolated from the human scalp, thereby providing an accessible, autologous and safe source of stem cells for regenerative medicine that have important advantages over ES or iPS cells.

© 2011 The Authors. Journal Compilation © 2011 John Wiley & Sons A/S

GL3

Photochemistry and excited state dynamics of eumelanin building blocks

V. Sundström
Department of Chemical Physics, Lund University, Sweden

During the last decade significant work has been devoted to the nature of UV-induced processes in eumelanin. All this work has been carried out on the whole pigment-complex. Assigning the observed phenomena to specific UV-induced processes is however complicated because of the large variety of oligomers present in the in-vivo pigment complex. The complexity of such analysis has motivated us to initiate a systematic study of smaller building blocks with a well-defined structure. Using time resolved spectroscopy we have succeeded in identifying several of the excited state processes of DHICA, DHI as well as of oligomers and polymers of these units. Thus, we have found that excited state Intramolecular Proton Transfer is the mechanism of excited state energy dissipation in DHICA monomers. Oligomers of DHICA have extremely efficient excited state dissipation, reminiscent of full melanin pigment. The mechanism of this very efficient energy dissipation is probably excited state inter-unit proton transfer within an oligomer. The two building blocks of melanin DHICA and DHI have very different photochemistry, which may be related to their suggested photoprotective and phototoxic properties, respectively.

GL4

Keratin disorders associated with abnormal pigmentation: clinical and molecular insights

E. Sprecher
Department of Dermatology, Tel Aviv Sourasky Medical Center, Tel Aviv, Israel

Keratin disorders have been traditionally considered to manifest mainly with abnormal cornification and/or skin blistering. Over the past years, a growing number of inherited clinical entities featuring prominent pigmentation abnormalities have been shown to be caused by mutations in keratin genes. Among these disorders are worth mentioning Epidermolysis with Mottled Pigmentation, Dowling-Degos disease and Naegeli–Franceschetti–Jadassohn syndrome. The identification of the molecular basis of these various genodermatoses has revealed that the role of keratins in the epidermis extends to various and critical non-mechanical functions.

GL5

Availability of transcriptional regulatory network analysis by next-generation sequencer

Y. Hayashizaki
Omics Science Center Riken Yokohama Institute, Yokohama, Japan

The emergence of high-throughput techniques has led to so-called data-driven biology, in which data obtained doesn't need a hypothesis about the biology, as opposed to when a hypothesis is tested within the framework of a pre-existing theory. We take a hybrid approach, in which high-throughput biology is driven by computational predictions of hypothetical genes. In FANTOM (Functional Annotation of Mammalian Genome), an international consortium we initiated, we recently have shown transcriptional control in the human monocytic cell line THP-1 throughout a differentiation time course. Using deepCAGE (a new deep sequencing application) we measured the dynamics of genome-wide transcription start site (TSS) usage over time for the first time and used comparative genomic regulatory site predictions in the regions by deepCAGE to identify the key transcription factors driving differentiation, their time-dependent activities, and their target genes. Using systematic siRNA knockdown of key transcription factors we have confirmed the role of individual factors in the differentiation process and mapped a suite of transcription factors required to maintain the undifferentiated state as well asto change from proliferation to differentiation. Our analysis of growth arrest and differentiation goes against the concept of a single 'master regulator'. Instead we emphasize that cellular states are constrained by complex networks involving substantial numbers of both positive and negative regulators. We have found 29 857 active promoters during differentiation of monoblast to monocyte (with 99.99% accuracy). In the FANTOM collaboration we have created a map of transcription regulation using the concept of motif activity and the degree of activity. If you introduce these key factors to the cell you can control its phenotype, for example, from fibroblast to monocyte (precursor cell to mature or highly differentiated cell). Such knowledge is very useful in applications of regenerative medicine and most importantly the information can be used to examine the potential for oncogenicity and monitor the safety of 'destination' (the target) cell.

GL6

Xeroderma pigmentosum and trichothiodystrophy: understanding cancer and non cancer phenotypes

J. M. Egly
IGBMC, Strasbourg, France

In devoting our research to the elucidation of the molecular mechanisms that control, at the transcriptional level, the expression of protein-coding genes (our real fundamental project), we met the fantastic world of DNA repair. This started once we isolated TFIIH, a remarkable multi-subunit complex that was not only essential for several key steps in the transcription process, but also turned out to play an equally pivotal role in DNA repair and more precisely in Nucleotide Excision Repair (NER). To document its role, we systematically dissected and reconstructed the key molecular machines of TFIIH and tested their in vitro (biochemical) and in vivo (physiological) functions using a panel of assays that were designed up in our laboratory after having completely reconstituted and produced the recombinant multi-subunit TFIIH complex. Knowing that mutations in XPB, XPD, and p8/TTDA, three subunits of TFIIH led to xeroderma pigmentosum (XP), trichothiodystrophy (TTD) and Cockayne syndrome (CS), we with the help of clinicians had tried to explain the molecular defects resulting from the various phenotypes e.g. photosensitivity, neuro-degeneration, lack of adipose tissue, ichtyosis, among others. Having realized that there might be a connection between DNA repair and transcription documented by the dual function of TFIIH, we next were surprised to find that RNA polymerase II transcription machinery assembles sequentially with the Nucleotide Excision Repair (NER) factors at the promoter of activated genes and this in absence of exogenous genotoxic attack. We presently document the role of these repair proteins at the promoter of activated genes in contributing to gene expression through their involvement in DNA de-methylation and/or histone post-translational modifications. Moreover we have extended our work by investigating the function of the Mediator that connect the DNA binding proteins to the basal transcription machinery. Mutations in the Mediator subunits result in several genetic disorders that share some clinical features with XP, TTD. Our work contributed not only to a better understanding of the gene expression regulation and the maintaining of genome integrity but also had explained some of the phenotypes of several genetic disorders, the hormonal regulation defects and the mechanism of anti-tumorigenic drugs. Such approaches were beneficial in understanding not only the

 © 2011 The Authors. Journal Compilation © 2011 John Wiley & Sons A/S

function of TFIIH in both transcription and DNA repair, but also of all the other NER factors.

GL7

Alpha melanocyte stimulating hormone: a major component of the skin immune system with a therapeutic potential

T. A. Luger
Department of Dermatology, University of Münster, Münster, Germany

Several components of the neuroendocrine system such as neuropeptides and hormones have been recognized to exert cytokine like effects via regulating innate as well as adaptive immunity. Among these neuromodulators α-melanocyte-stimulating hormone (αMSH) derived from the proopiomelanocortin was found to exhibit marked immunoregulating and anti-inflammatory activities. The effects of αMSH are mediated via direct effects on cells of the immune system as well as indirectly via affecting the function of resident non-immune cells. In order αMSH can exert these effects the expression of specific melanocortin receptors (MC-R) in particular MC-1R on both immunocompetent as well as non-immune cells is required. However, there is increasing evidence that αMSH can also penetrate independently of the expression of specific receptors in the cell and display its activity. αMSH affects several pathways implicated in regulation of inflammatory responses such as NF-κB activation, expression of adhesion molecules and chemokine receptors, production of proinflammatory cytokines and other mediators. Thus αMSH may modulate inflammatory cell proliferation, activity, and migration. Moreover, αMSH prevents the maturation of dendritic cells (DC) and thereby triggers the generation of a subset of regulatory T-cells. The anti-inflammatory and immunomodulatory effects of αMSH have been confirmed by means of animal models of inflammation such as irritant and allergic contact dermatitis, cutaneous vasculitis, psoriasis, inflammatory bowel disease as well as rheumatoid arthritis. Whereas the melanocyte stimulating activity of αMSH requires binding of the core tetrapeptide to MC-1R, there is accumulating evidence that the anti-inflammatory activities of αMSH can be attributed to its C-terminal tripeptide KPV and do not depend on the expression of a functional MC-1R. K(D)PT, a derivative of KPV corresponding to the amino acid 193–195 of IL-1β, is currently emerging as another tripeptide with potent anti-inflammatory effects. The anti-inflammatory potential together with the favourable physiochemical properties most likely will allow these agents to be developed for the treatment of inflammatory skin, joint, and bowel diseases.

GL8

Neuromelanins in brain aging and Parkinson's disease

L. Zecca, W. Zhang, V. Jackson-Lewis, S. Przedborski, J. S. Hong, D. Sulzer, C. Bellei, L. Casella, N. J. Turro, M. Eisner, P. R. Crippa, S. Ito, K. Wakamatsu, J. D. Simon, F. A. Zucca
Institute of Biomedical Technologies-CNR Milan, Italy

Neuromelanins are a family of compounds occurring in all brain regions of human brain. These pigments consist of granules 30 nm in size, contained in organelles together with lipid droplets, and they accumulate in aging, reaching concentrations as high as 1.5–2.6 μg/mg tissue in major brain regions. Neuromelanins, contain melanic, lipid, and peptide components. The melanic component is aromatic in structure, contains a stable free radical, and is synthesized from the precursor molecule cysteinyl-3,4-dihydroxyphenylalanine. This is different in neuromelanin of the substantia nigra, where the melanic precursor is cysteinyl-dopamine. These neuronal pigments have some structural similarities to the melanin found in skin. The precursors of lipid components of the neuromelanins are the polyunsaturated lipids which were characterized as dolichols and are present in the surrounding organelles. All neuromelanins have the same spacing in their stacking of structure components in the range 4.65–4.72 Å. They have a threshold potential of 4.6 ± 0.2 eV, which is reflective of eumelanin on the surface that is quite low and so the surface of intact pigments is not thermodynamically positioned to trigger oxidative damage in neurons. The synthesis of neuromelanins in the various regions of the human brain is an important protective process because the melanic component is generated through the removal of reactive/toxic quinones that would otherwise cause neurotoxicity. Furthermore, the resulting melanic component serves an additional protective role through its ability to chelate and accumulate metals, including environmentally toxic metals such as mercury and lead. Other metals like iron, zinc, aluminum, chromium and molybdenum are also accumulated by neuromelanins. However neuromelanin can play also a toxic role in Parkinson's disease when it is released by dying neurons of substantia nigra. Extracellular neuromelanin particles induce microglial activation and ensuing production of superoxide, nitric oxide, hydrogen peroxide and pro-inflammatory factors. Furthermore, neuromelanin produces, in a microglia-depended manner, neurodegeneration in primary ventral midbrain cultures. Neurodegeneration is effectively attenuated with microglia derived from mice deficient in macrophage antigen complex-1, a microglial integrin receptor involved in the initiation of phagocytosis. Neuronal loss is also attenuated with microglia derived from mice deficient in phagocytic oxidase, a subunit of NADPH oxidase, that is responsible for superoxide and hydrogen peroxide production, or apocynin, a NADPH oxidase inhibitor. In vivo, neuromelanin injected into rat substantia nigra produces microgliosis and a loss of tyrosine hydroxylase neurons. Thus, these results show that extracellular neuromelanin can activate microglia, which in turn may induce dopaminergic neurodegeneration in Parkinson's disease.

Abstracts of Invited Lectures (IL)

IL1

Melanocyte development: the journey to unknown territory

B. Werhle-Haller
Department of Cell Physiology and Metabolism, Centre Medical Universitaire, University of Geneva, Geneva, Switzerland

'To boldly go where no other cell has gone before'. This is the task to which neural crest cells have committed them self when segregating from the dorsal neural tube. They have been instructed by transcription factors such as Pax3 and Snail-2, providing them with the capacity to detach from the mother-tissue, in a process called epithelial to mesenchymal transition. In order to forge their identity they reside in a temporary 'staging area' where they are influenced by morphogenetic factors originating from the notochord or dorsal neural tube such as Wnt or TGF-β-like molecules. Only after expressing specific cell surface receptors such as EndrB and c-kit, which are maintained in a positive feedback with transcription factors such as MITF, Sox10, Brn2, predetermined melanocyte precursors will go onto their journey to colonize the unknown periphery of their universe. Not much is know about the mechanisms and signaling pathways that allows them to lodge into their epidermal niches, where they provide pigmentation and sun-protection or alternatively reside in a dormant state, waiting to be awoken from their hibernation by extrinsic cues. Although their genetic equipment is perfectly evolved, mistakes in the board computer, such as

© 2011 The Authors. Journal Compilation © 2011 John Wiley & Sons A/S

B-RAF may cause the loss of the crew, ship or even the organism in which they are residing. Eventually, understanding the journey of melanocytes will allow us to face our sun and that of other solar systems without fear and hesitation.

IL2

Cellular origin of melanocytes: newly resolved routes to melanocyte cell lineages

T. Kunisada, H. Aoki, N. Yoshimura, T. Motohashi
Gifu University Graduate School of Medicine, Gifu, Japan

A migratory cell population originated from the anterior neural tube was reported to differentiate exclusively into pigment cells in nonvertebrate urochordates. In vertebrates, multipotential migratory cells were delaminated from the neural tube were named as neural crest cells and only those migrating along the dorsolateral pathway were thought to be the main source of melanocytes in the skin. However this concept was challenged by the fact that Schwann cell precursors are the major cellular source of skin melanocytes. In addition, we recently observed that presumptive neural crest cell population not derived from dorsal neuroepithelial cells differentiate into significant portion of skin melanocytes. Later, they clearly segregate from those originated from dorsal neuroepithelial cell derived neural crest cells in the mouse body skin. These findings provide more complex views on the cellular origin of melanocytes, possibly lead to the functional discrimination of mlanocytes. Functionally distinct melanocyte populations revealed in mice: noncutaneous and dermal melanocytes versus epidermal melanocytes.

IL3

Physicochemical changes of retinal pigment epithelium melanin with aging and photoaging monitored by advanced EPR techniques

G. Szewczyk, A. Pilat, M. Zareba, J. M. Burke, T. Sarna
Jagiellonian University, Krakow, Poland

Melanin in the human retinal pigment epithelium (RPE) is formed early in fetal development and thereafter shows little metabolic turnover. Being exposed to intense visible light from focal irradiation and high oxygen tension RPE melanin may undergo oxidative modifications that can alter its physicochemical properties and biological functions. In this study, we analyzed changes in paramagnetic, redox and metal-ion binding properties of human RPE melanin with aging and bovine RPE melanosomes subjected to experimental photoaging. RPE cells, obtained from human donors of different age, and purified porcine or bovine RPE melanosomes, with selected degree of photo-bleaching, where analyzed by 95 GHz (W-band) continuous wave (CW) and saturation recovery (SR) EPR spectroscopy at different pH. Redox properties of human RPE melanin and animal RPE melanosomes were tested by monitoring photo-reduction of an appropriate nitroxide spin probe, and the ability of melanin to form complexes with multivalent metal ions was determined by X-band CW and SR EPR spectroscopy. Our results indicate distinct changes in paramagnetic properties of RPE melanin with aging and RPE melanosomes with photoaging. Partially photobleached bovine and porcine RPE melanosomes, in comparison with untreated melanosomes, exhibit higher efficiency to photoreduce nitroxide spin probe. Aged human RPE melanin and experimentally photo-aged animal RPE melanosomes bind multivalent metal ions less tightly than young human RPE and untreated bovine and porcine RPE melanosomes. The data suggest substantial changes in key physiochemical properties of RPE melanin that occur with aging, which, to some degree, can be modeled by photo-bleaching of animal RPE melanosomes, and are monitored by advanced EPR spectroscopy. Supported by Poland Ministry of Science and Higher Education (grant: 2661/B/P01/2010/39) and NIH (grants: R01EY013722, R01EY019664).

IL4

Chemistry and biophysics of melanins

K. Wakamatsu, T. Murase, F. A. Zucca, L. Zecca, S. Ito
School of Health Sciences, Fujita Health University, Toyoake, Aichi, Japan

Neuromelanin (NM) is a brown insoluble pigment abundant in catecholaminergic neurons of the substantia nigra and locus caeruleus in brains of human and mammalian species. Despite of its possible role in the etiology of Parkinson's disease, biosynthesis and biodegradation of NM is not well elucidated.

In order to elucidate the biosynthetic and biodegradative pathways (aging process) of NM, we synthesized four standard compounds, 5-*S*-cysteinyldopamine (CDA), dihydrobenzothiazine-1 (DHBT-1), 3-oxo-dihydrobenzothiazine-1 (ODHBT-1) and benzothiazoleamine-1 (BZ-1), which are considered as intermediates in NM synthesis. We next prepared model compounds of NM by tyrosinase oxidation at various ratios of dopamine (DA) and cysteine (Cys) under the physiological conditions (pH 7.4, 37°C). We then followed biodegradation (aging process) of NM under various heating conditions: 100°C for 24 h, 60°C for 24 days or 37°C for 120 days. To elucidate the process of NM synthesis and aging process in detail, chemical degradation methods of alkaline hydrogen peroxide oxidation and reductive hydrolysis with hydroiodic acid were applied to the reaction mixtures.

Biosynthetic experiments show that DA is oxidized in a sequence of CDA, DHBT-1, and then ODHBT-1/BZ-1. Gradual conversion of benzothiazine moiety to benzothiazole is noted by following the degradation markers, 4-amino-3-hydroxyphenylethylamine (4-AHPEA, a benzothiazine marker) and thiazole-2,4,5-tricarboxylic acid (TTCA, a benzothiazole marker). To simulate aging process of NM in vivo, biodegradative experiments were performed by heating the synthetic NM prepared from various ratios of DA and Cys. The results suggest that the benzothiazine moiety in NM is converted to the benzothiazole. By comparing ratios of the above degradation markers, it was found that natural NM is close to that prepared by heating synthetic NM from a 2:1 molar ratio of DA and Cys either at 100°C for 8 h, 60°C for 8 days or 37°C for 120 days.

Natural NM was found to be degraded during aging process with the benzothiazine moiety being converted to the benzothiazole. By mimicking this process, it has now become possible to prepare synthetic NM that has structural features of natural NM.

IL5

Difficult hyperpigmentary disorders: an Asian perspective

B.-K. Goh
National Skin Centre, Singapore, Singapore

Hyperpigmentation, a common presenting complaint among Asians, can due to increased melanogenesis, melanocytosis, as well as delayed breakdown and removal of melanin. Although hyperpigmentary disorders can be broadly categorised into congenital/genetic or acquired causes and present as diffuse or localised forms, certain hyperpigmentary dermatoses peculiar to Asians are difficult to classify. This can arise because of paucity of description in the literature, inadequate elucidation of the clinical spectrum of the disorder, as well as overlapping or non-specific pathological findings – making accurate diagnosis difficult. In this presentation, hyperpigmentary disorders such as

 © 2011 The Authors. Journal Compilation © 2011 John Wiley & Sons A/S

lichen planus pigmentosus, erythema dyschromicum perstans, idiopathic eruptive macular pigmentation, amyloidosis cutis dyschromica and 'mixed' melasma will be highlighted and the controversies surrounding their diagnosis and classification discussed.

IL6

Pathogenesis of melasma: new insights

Y. Gauthier, M. Cario-André, S. Lepreux
Hôpital St-André, Bordeaux, France

Melasma is a common acquired light to dark brown hypermelanosis occuring in the sun-exposed-areas of the face and the neck. Melasma lesional skin is characterized by an epidermal melanin deposition in all skin layers including horny layer, sometimes associated with dermal melanin deposits. Under light and confocal microscopy, the melanocytes are larger and more dendritic in melasma skin. In many cases there is no difference in melanocyte number between areas. Immunohistochemistry with anti aMSH antibodies showed a significant labeling of melasma epidermis. More recently it has been reported an altered expression of Wnt pathway, lipid metabolism and melanogenesis-related genes in melasma. Surprisingly a histochemical and immunohistochemical study in melasma revealed evidence of damage to basal membrane which could facilitate the fall and migration of melanocytes and melanin in the dermis. As previously reported by Konrad, electron microscopy revealed a greater number of mature and non aggregated melanosomes in keratinocytes of all epidermal layers. Increased expression of VEGF in keratinocytes has been suggested as the major angiogenic factor for altered vessels in melasma. The major etiologic factors include genetic influence, exposure to ultraviolet radiations and sex hormones. The influence of estrogen and progesterone on human cutaneous pigmentation is largely unknown, despite the presence of estrogen and progesterone receptors on human melanocytes. Recently we have studied by electron microscopy the effects of oestrogens (from 10 to 100 nM) on melanosome distribution in UVB irradiated organ culture. We have reproduced with our experimental model the same non aggregated distribution of melanosomes reported in melasma skin samples. Our data suggest that sex hormones combined with UVB irradiation could be implicated in the modification of melanosomes distribution explaining possibly the chronicity of this hypermelanosis.

IL7

Mouse models for studying pigment cell biology and pigmentary diseases

E. Zurita, A. Fernandez, C. Vicente, E. Moltó, D. Seruggia, M. Cantero, L. Montoliu
CNB-CSIC Campus de Cantoblanco, Madrid, Spain

Almost four hundred loci have been directly or indirectly already associated with the biology of pigment cells. Most of these loci have been detected, primarily, as mouse coat color mutants and about half of them remain to be cloned. These mouse mutants represent useful animal models for the understanding of the corresponding pigmentary diseases. This is the case of albinism, where mutations in up to 14 genes have been associated to the different types of albinism. These genes include obvious candidates, directly involved with melanin biosynthesis (Tyr, Tyrp1, Tyrp2, Pmel, Slc45a2,...). A number of additional genes appear related to the mechanisms of proliferation, differentiation and/or migration of melanocytes (Mitf, Edn3, Ednrb, Kit, Kitl, Sox10,...) and their mutations usually result in 'white spotting' phenotype. Other proteins are fundamental for the biology of melanosomes. When the genes encoding these proteins are mutated a complex phenotype emerges affecting pigment cells and other cell types, where the biogenesis of lysosomal-related organelles is altered. These include all Hermansky–Pudlak syndrome-related genes (Ap3b1, Bloc1s3, Hps1, Hps3, Hps4, Hps5, Hps6...), the Chediak–Higashi syndrome locus (Lyst) and two cases of albinism (Oca2, Gpr143), among other loci. Some examples of mutations altering melanosomal intracellular movements include Myo5a and Rab27a, mutated in the Griscelli Syndrome. Other loci control the ratio between eumelanin versus pheomelanin, such as the locus agouti (A), or its receptor Mc1R, whose mutations are commonly associated to red-hair phenotype and increased risk to develop melanoma. Mutations in some genes may also display some alterations in pigmentations, however these are normally secondary to an underlying more severe defect, such as Atp7a and Atp7b loci, causing systemic effects and mutated in Menkes and Wilson diseases, respectively. The 'Coat Color Mutants' web page (www.espcr.org/micemut) has an updated list of all known pigmentary-related genes, and their corresponding mouse models. Finally, mice are specially suited animal models for studying pigmentary diseases, due to the mouse genetic toolbox enabling researchers to produce genetically-modified mice (transgenic and knockout) with specific mutations reproducing those found in humans. This talk will discuss a number of examples of spontaneous and targeted mouse mutations that have been instrumental for our current understanding of human pigmentary diseases.

IL8

Sox10 modifier loci identified in a sensitized ENU mutagenesis screen

D. Watkins-Chow, K. Leeds, R. Mullen, A. Incao, C. Rivas, W. Pavan
National Institutes of Health Bethesda, MD, USA

Melanocytes are specialized, neural crest-derived cells responsible for pigment production in the skin. Disruption in neural crest development can present as altered pigmentation in skin and/or hair and can be associated with debilitating diseases including deafness, blindness, cleft lip, congenital megacolon, and albinism. Collectively, these diseases are referred to as neurocristopathies. Because genetic background is known to affect the severity of neurocristopathies in both humans and mice, we previously established an enhancer screen to identify mutations that increase the phenotypic severity of Sox10 haploinsufficient mice (Sox10LacZ/+), a well-characterized mouse model of human neurocristopathies. This goal of this mutagenesis screen is to identify previously uncharacterized pathways affecting melanocyte development and to generate models relevant for dissecting human disease etiology. From analysis of 600 pedigrees, we identified five dominant modifiers of the Sox10 phenotype (Mos1-5). Mos3 exhibits a unique, Sox10-dependent, semi-dominant phenotype. Mos3/Mos3 homozygotes exhibit embryonic lethality, but Mos3/+ heterozygotes appear indistinguishable from their wild type littermates. On a Sox10LacZ/+ background, heterozygosity for the Mos3 mutation causes white head spotting that is never observed in Sox10LacZ/+ mice. Embryonic analysis shows that Mos3 causes this synergistic reduction in cranial crest-derived melanoblasts by embryonic day 12.5, before a synergistic reduction in trunk melanoblasts is observed. This suggests that compared to other spotting mutants, Mos3 more severely affects the cranial crest and may reveal a novel pathway affecting melanoblast development. Further comparative analysis of phenotypes identified in our screen will contribute to our understanding of genome function and provide additional disease models for human neurocristopathies and melanoma progression.

© 2011 The Authors. Journal Compilation © 2011 John Wiley & Sons A/S

IL9

Human pigmentation genes and population polymorphism

R. A. Sturm
Institute for Molecular Bioscience, Brisbane, Australia

The physical appearance of skin, hair and eye colour can vary dramatically among geographically isolated human populations. It has long been speculated that this is due to adaptive changes, but the genetic causes for this degree of phenotypic variation have remained largely unknown. It is apparent that a large number of genes impacting melanosome biogenesis or the melanin biosynthetic pathway will influence the diversity seen in human pigmentation. The discovery and characterisation of human pigmentation gene polymorphism within and between human populations, combined with functional studies, have provided a framework to understand normal variation in this physical trait. Major candidate genes include the enzymes encoded by tyrosinase, tyrosinase-related protein-1 and dopachrome tautomerase (TYR, TYRP1 and DCT), the P-protein (OCA2) and the melanocortin-1 receptor (MC1R). Variant alleles of the MC1R gene resulting from a range of amino acid substitutions have been associated with red hair, fair skin, a high degree of freckling as well as increased incidence of melanoma. A single SNP located in a regulatory region upstream of the OCA2 locus that determines blue-brown eye colour inheritance in Europeans. Other population studies have revealed specific polymorphisms within the MATP (SLC45A2) and NCKX5 (SLC24A5) protein coding regions associated with the degree of skin pigmentation. Our studies take advantage of cultures of human primary melanocytes derived from donor skin tissue and selected based on pigmentation genotype and/or phenotype. Direct testing of a range of clonal melanocyte cultures characterised for three causal SNPs within SLC45A2, SLC24A5 and OCA2 have assessed their impact on melanin content and tyrosinase enzyme activity.

IL10

The other ones: non-cutaneous melanocytes

F. C. Brito, N. Fernandez, K. Balani, A. Agarwal, L. Kos
Department of Biological Sciences, Florida International University, Miami, FL, USA

Melanocytes are generally associated with pigment producing cells found in the skin. However, they can be found in organs and tissues as diverse as the eye, inner ear, brain, heart and fat. Except for those found in the retinal pigment epithelium of the eye that originate directly from the neural ectoderm, all others are derived from neural crest precursors. Melanocytes found in the skin exert protective functions against the damaging effects of ultraviolet radiation. The exact function of the other melanocytes have yet to be fully determined but may include detoxification and protection against oxidative stress due to the capacity of melanin to scavenge reactive oxygen radicals. In the heart, melanocytes are specifically located in the pulmonary veins, atrial walls, and atrioventricular (AV) valves. The melanocytes found in the pulmonary veins and atrial walls have adrenergic and muscarinic receptors and have been linked to atrial fibrillation. We have focused on those melanocytes that reside in the AV valves. They reach the heart at a time when major remodeling events are taking place in the endocardial cushion that are necessary for the proper formation of the AV valve leaflets. The resulting arrangement of organized layers of extracellular matrix (ECM) and the biomechanical properties of the leaflets are required for the valves to regulate blood flow direction. We hypothesized that melanocytes influence the distribution of ECM components in the AV valves and as a consequence may affect their biomechanical characteristics. We used a modified Movat's pentachrome stain to reveal the arrangement of ECM components in AV valve leaflets with wild type numbers of melanocytes, no melanocytes (EdnrBs-l/s-l mutants) and large numbers of melanocytes (K5-Edn3 transgenics). Our results indicated that there was an increase in the amount of collagen in the leaflets with no melanocytes while there was an increase in the amount of proteoglycans and elastin in the leaflets with excessive numbers of melanocytes. To test for the leaflets' biomechanical properties, a quasi-static and nanodynamic (Nano-DMA) mechanical analysis was evaluated using a Hysitron's TriboIndenter®. The quasi-static nanoindentation of the wild type leaflet indicated its viscoelastic property. Nano-DMA measurements of the storage modulus of the wild type leaflet showed and average stiffness of approximately 7.5 GPa. The storage modulus of the leaflets with no melanocytes (approximately 3.5 GPa) was much smaller when compared to the wild type whereas that for the leaflets with extra numbers of melanocytes was much higher (approximately 11.5 GPa). Our results suggest that the presence of melanocytes in the AV valves affect their ECM patterning resulting in changes in their stiffness.

IL11

A role for inner ear melanocytes in anti-stress responses

S. Uehara, H. Yamamoto
Faculty of Bioscience, Nagahama Institute of Bio-Science and Technology, Nagahama, Japan

Melanocyte precursors differentiate from the neural crest (NC), which is now considered a fourth germ layer (Hall, 2009), then they migrate and target a wide variety of tissues and organs. During the migration, melanophores/melanocytes and the cell/tissue microenvironment interact continuously. Therefore, it is not difficult to suppose that the developmental and localizing processes of these cells makes each of their local populations different in functions while retaining common lineage signature(s) such as melanin pigment synthesis and expression of other pigment cell-specific genes. It is well known that a group of melanocyte populations localized in the stria vascularis of the mammalian inner ear cochleae are essential for hearing ability, but peculiarly enough, they do not necessarily need to produce melanin pigment. Marcus et al. (2002) reported that knockout of the Kcnj10 gene, which encodes the inwardly rectifying K+ channel Kir4.1 and is expressed in intermediate cells (melanocytes) of the cochleae, abolishes the endolymphatic potential and reduces K+ concentration in the endolymph, resulting in deafness. Is this the only role for cochlear melanocytes? Gratton and Wright (1992) reported that noise stimulates melanin synthesis in inner ear melanocytes. We recently found that cochlear melanocytes in the stria vascularis (but not in hair follicles) specifically express Gsta4, which is deeply involved in anti-stress responses (Uehara et al., 2009). Considering the facts that melanin works both as an energy transducer and as a radical scavenger, we now hypothesize that melanocytes have evolved to execute a variety of functions depending on their localization but still have common signature functions in which melanin synthesis could essentially contribute. We will discuss studies on the functions of such interesting pigmented cells, especially of the non-classical [non-cutaneous (Yajima and Larue, 2008)] melanocytes scattered across the body.

 © 2011 The Authors. Journal Compilation © 2011 John Wiley & Sons A/S

IL12

The melanocyte living on the edge, surrounded by its neighboring keratinocytes and fibroblasts

Z. A. Abdel-Malek
University of Cincinnati, Cincinnati, OH, USA

In human skin, melanocytes (MC) reside on the epidermal-dermal junction, and receive biochemical cues from epidermal keratinocytes (KC) as well as dermal fibroblasts (FB). Melanocytes play a pivotal role in photoprotection, and melanin is also thought to bind and detoxify metals and toxic chemicals. Unlike KC and FB, MC have poor proliferation and self-renewal capacities. Given the significance of MC in the skin, it is important to maintain their survival and genomic stability. It is unequivocal that a paracrine/autocrine network exists in human skin and regulates the response of MC to various environmental factors, particularly UV. Our objective is to review the role various paracrine/autocrine factors and their participation in the UV response of MC. Exposure to UV induces the expression of the primary cytokines IL-1 and TNF-α by KC. Treatment of MC with either cytokine reduces their proliferation and tyrosinase activity. IL-1 stimulates the synthesis of endothelin-1 (ET-1) by KC and POMC by KC and MC. ET-1 and α-MSH interact synergistically to stimulate MC proliferation and melanogenesis, reduce UV-induced apoptosis and generation of reactive oxygen species (ROS), and enhance repair of DNA photoproducts. The effects of α-MSH are mediated by binding and activating the melanocortin 1 receptor (MC1R), and are absent in MC that naturally express loss-of-function MC1R variants that are strongly associated with red hair phenotype and increased risk for melanoma. The mechanisms by which α-MSH enhances repair of DNA photoproducts involve the activation of p53 as well as the transcription factors ATF2 and Nrf2. Treatment of MC with α-MSH decreases oxidative DNA damage by immediately reducing the generation of ROS, and activating catalase and subsequently phase II detoxifying enzymes, such as hemeoxygenase-1. The effects of ET-1 are mediated by the ET B receptor that is predominantly expressed by MC. Interestingly, treatment of MC with ET-1 increased the expression of MC1R, thus allowing for increased and/or sustained response to α-MSH. A new physiological antagonist for the human MC1R is human beta defensin 3 (HBD3), which is synthesized by keratinocytes, and inhibits the stimulatory effects of α-MSH on cAMP formation, proliferation and melanogenesis of MC. Nerve growth factor produced by KC increases MC dendricity, migration and survival following UV exposure. Similarly, stem cell factor and neurotrophin 3, both of which are synthesized by KC and FB, increase the survival of MC. 1, 25(OH)2 vitamin D3, which is synthesized by KC upon UV exposure, inhibits UV-induced apoptosis and increase repair of DNA photoproducts in MC and KC. Interestingly, 1,25 (OH)2 vitamin D3 interacts synergistically with α-MSH to reduce UV-induced apoptosis. Neuregulin-1 is a novel paracrine factor that is expressed at higher levels by FB derived from individuals with dark skin that by FB from individuals with light skin, suggesting an important role for this factor in determining constitutive pigmentation, which in turn affects the UV response of the skin. The paracrine/autocrine network in the skin consists of stimulatory as well as inhibitory factors that function synchronously to maintain homeostasis and preserve survival and genomic stability of MC when challenged with environmental insults, mainly UV.

IL13

Endothelin-1/stem cell factor signaling blockade in melanocytes and pigmentation in human epidermal equivalents

G. Imokawa
School of Bioscience and Biotechnology, Tokyo University of Technology, Japan

UVB exposure of the skin causes keratinocytes to produce and secrete several major melanogenic cytokines such as endothelin-1 (EDN1) and stem cell factor (SCF) which stimulate melanocytes by activating intercellular signaling cascades consisting of TYK/PKC/cAMP-PKA/MAPK/RSK. The PKA and/or RSK activation elicits CREB activation through its dual phosphorylation, which results in increased gene and protein expression of the MITF. Increased MITF function in turn leads to up-regulated gene and protein expression levels of several melanocyte-specific proteins which contribute to the UVB-stimulation of epidermal pigmentation. Whereas in cultured human melanocytes, blockade for the activation of specific signaling pathways distinctly leads to down-regulation of melanocyte-specific proteins accompanied by a deficiency of MITF expression, it remains unclear whether the same blockade is associated with the loss of epidermal hyperpigmentation in the skin in vivo because of little information on how the expression levels of MITF protein/its phosphorylated one as well as their combination as a convergent point of several signaling linkages contribute to melanization within melanosomes and its transfer to keratinocytes. We have recently established reconstituted human epidermal equivalents in which the visible epidermal pigmentation can be stimulated by the addition of major melanogenic cytokines, EDN1 and/or SCF, which was accompanied by up-regulated expression of all melanocyte-specific proteins including MITF at gene and protein levels. To further elucidate role of intracellular signaling within human melanocytes during cutaneous hyperpigmentation, in this study, we used specific signaling inhibitors, chemicals or herb extracts known to interrupt different sites of melanogenic intracellular signaling linkages, to compare the interrupting effect on the activation of melanogenic signaling pathways in cultured human melanocytes with the suppressive effects on the stimulated pigmentation in the human epidermal equivalents after the treatment with EDN1 and/or SCF. A MEK inhibitor, PB98059 distinctly suppressed the stimulated pigmentation in the human epidermal equivalents treated with END1 or SCF, whereas a PKC inhibitor, Gö6983 distinctly attenuated the stimulated pigmentation in the human epidermal equivalents treated with END1 but not with SCF.

IL14

Depigmentation update: treatment of melasma

J.-P. Ortonne
Department of Dermatology Archet 2 Hospital, Nice, France

Although many treatment modalities have been used for melasma. There is no cure for this disorder. A recent study has demonstrated that microphthalmia-associated transcription factor-siRNA (MITF-siR)-silenced MITF gene expression effectively induced a significant reduction in tyrosinase, tyrosinase-related protein-1, and melanocortin-1-receptor levels. Using a novel type of transdermal peptide, these investigators developed the formulation of an MITF-siR cream. This cream significantly lightens brown facial hypermelanosis and lightens the normal skin and also the melasma skin in Asian individuals. Laser therapy has been used to improve melasma, but caution must be exercised when treating this disease or PIH may occur after treatment. QS RL (694 nm), QSNd:YAG (1064 nm), QS AL

© 2011 The Authors. Journal Compilation © 2011 John Wiley & Sons A/S

(755 nm), CO_2 (10600 nm) and Er:YAG (2940 nm) lasers. In the 1550 nm fractionated erbium-doped fiber laser, and the fractional CO_2 laser have all been studied in the treatment of melasma. A stabilized formulation of Kligman's preparation showed significant improvements in the treatment of melasma. A pilot study was performed to evaluate the interest of associating the fixed triple combination cream with PDL in the treatment of melasma. The used of PDL in association with a bleaching cream appears beneficial in treating melasma in patients with phototypes II and III. Because melasma may be present for many years and relapse after is common, development of a maintenance regimen after initial improvement would help in the management of this disorder. An open label-trial has demonstrated that half of patients were able to begin maintenance therapy twice per week after twelve weeks. However, relapses occurred in most of these patients requiring resumption of daily-therapy. In a second study, two maintenance regimens of the triple combination cream were compared (triple combination regiment twice weekly versus tapering regimen). The time for relapse was similar for both regimens. From these results, maintenance is necessary and should be customized in patients with melasma. The treatments of pigmented lesions including melasma continue to evolve as new technologies develop.

IL15

Treatment of hyperpigmentary disorders in Asian skin

K.-C. Park, H.-R. Choi
Department of Dermatology, Seoul National University Bundang Hospital, Seongnam-si, Korea

Hyperpigmentation is a manifestation of melasma, senile lentigo, freckle, and nevus so on. Melasma will be a typical example of pigmentary disorders. It is difficult to treat and is often refractory. Conventional treatments include sunscreens, bleaching creams, azelaic acid, topical retinoids, and facial peels where an acid solution is used to remove outer layers of the skin. Vitamin C iontophoresis can be another choice. Some treatment incorporates a combination approach including triple-combination cream (hydroquinone, tretinoin, and steroids). However, there is no gold standard in the treatment of melasma. Among topical agents, triple-combination cream is effective when compared to hydroquinone alone or dual-combination cream. However, adverse responses are quite common. Recently, QS 1064 nm Nd-YAG laser has increasingly been performed as 'laser toning', or 'laser facing', for melasma in Asian countries. Clinical effects seemed to be promising but the depigmentation can appear. In addition, it is also reported that fractional photothermolysis is a safe and effective treatment for refractory melasma. However, it is also reported that non-ablative fractional laser therapy is not recommended because of the high rate of postinflammatory hyperpigmentation. Thus, it is very confusing to find out the best way for the treatment of melasma. In addition, oral agents such as tranexamic acid are also reported to be effective. In addition to melasma, there are various kinds of pigmentary disorders. Solar lentigo is correlated directly with increasing age and UV. Thus, solar lentigo is common in individuals who sunburn easily and do not tan, But, Asian women also have high incidence of solar lentigo. They usually appear as mottled pigmentation. For the treatment of lentigo, Nd-Yag laser are used. IPL can be another modality which is commonly used effectively and safely for the treatment of pigmentation. However, rejuvenation treatment is necessary using fractional laser or related technology for the extensive treatment or prevention. In this presentation, choices of treatment modality and brief underlying pathogenesis will be discussed.

IL16

Stem cell regulation by stem cells

E. K. Nishimura
Medical Research Institute, Tokyo Medical and Dental University, Japan

In most stem cell systems, the organization of the stem cell niche and the anchoring matrix required for stem cell maintenance are largely unknown. Melanocyte stem cells (MelSC) and hair follicle stem cells (HFSC), which are originally derived from a completely different developmental origin, are located in the bulge area of mammalian hair follicles. Our previous studies indicated that the niche plays dominant role in MELSC fate determination, while the underlying mechanisms and the correlation with HFSCs remain unclear. We recently found that collagen XVII (COL17A1/BP180/BPAG2), a hemidesmosomal transmembrane collagen, is highly expressed in HFSCs and is required for the maintenance not only of HFSCs but also of MelSCs, which do not express Col17a1 but directly adhere to HFSCs. Mice lacking Col17a1 show premature hair graying and hair loss. Analysis of Col17a1 null mice revealed that COL17A1 is critical for the self-renewal of HFSCs through maintaining their quiescence and immaturity, potentially explaining the mechanism underlying hair loss in human COL17A1 deficiency. Interestingly, Col17a1 null mice show defective TGF-β production by HFSCs and targeted TGF-b type II receptor (Tgfbr2) deficiency in the melanocyte lineage causes incomplete maintenance of melanocyte stem cell immaturity and results in premature hair graying. These data demonstrate that the TGF-b signaling pathway is the critical niche factor that regulate melanocyte stem cell immaturity and quiescence. Finally, forced expression of COL17A1 in basal keratinocytes, including HFSCs, in Col17a1 null mice rescues MelSCs from premature differentiation and restores TGF-β signaling, demonstrating that HFSCs function as a critical regulatory component of the MelSC niche.

IL17

Neural crest stem cells and melanoma formation: a likely connection

O. Shakhova, G. Civenni, F. Beermann, L. Sommer
University of Zurich, Switzerland

Human melanoma is composed of distinct cell types reminiscent of neural crest derivatives. We have recently shown that this heterogeneity is established by multipotent cells expressing the neural crest stem cell (NCSC) marker CD271. When isolated from solid tumors using a method that leaves intact cell surface epitopes, these NCSC-like cells, but not CD271-negative cells, form tumors upon xenotransplantation that mirror the heterogeneity of the parental melanoma. Moreover, even upon transplantation into fully immunocompromized mice, presence of CD271-positive cells is required for long-term tumor expansion in vivo. These data indicate a role of stem cell-like cells in tumorigenesis. To further address this issue, we made use of a mouse melanoma model, in which NrasQ61K-oncogene expression in the melanocytic lineage consistently leads to formation of melanoma. Similar to human melanoma, these tumors are composed of various cell types expressing markers of neural crest derivatives. Importantly, tumor initiation in these mice is associated with melanocyte stem cell expansion and emergence of melanoblasts from the stem cell niche, resulting in skin hyperpigmentation and tumorigenesis. Thus, oncogene-mediated activation of melanocyte stem cells is a crucial event in melanoma formation.

 © 2011 The Authors. Journal Compilation © 2011 John Wiley & Sons A/S

IL18

Pigmentation genes and melanoma: where are we now?

N. Soufir
Laboratoire de Biochimie Hormonale et Génétique, Hôpital Bichat Claude Bernard; & Inserm U976, Hôpital Saint Louis, Paris, France

Cutaneous melanoma (CM) is a malignant tumor arising from melanocytes that causes 75% of deaths related to skin cancer. Since the mid-1960s, melanoma incidence has increased by 3–8% per year in Caucasian populations. Nevi count, atypical nevi, and UVR exposure are important and well recognized melanoma risk factors. Incidence is also higher in individuals with fair skin than those with dark skin, suggesting that skin colour, which is known to be related to the degree of protection against UVR, is also important. Therefore, genes involved in the determination of skin color and tanning response are potentially implicated in MM predisposition, and may be useful predictors of MM risk in the general population. Although more than 120 genes involved in the process of pigmentation, such as maturation, transport, and distribution of melanosomes, have been identified through animal models, only several genes have been identified as containing common genetic variants associated with human pigmentation. Multifactorial genetic susceptibility to CM has been linked to polymorphisms in low-penetrance genes that interact with environmental factors – mainly ultraviolet exposure – to modulate CM risk. In this area, the best-studied gene is the melanocortin 1 receptor (MC1R), the presence of Red Hair Colour (RHC) MC1R variants being associated to CM risk in an additive manner. However, variants in other pigmentation genes have recently been shown to modulate the risk of melanoma, such as those coding for the membrane-associated transporter protein (SLC45A2/MATP), the agouti signaling protein (ASIP), the tyrosinase enzyme (TYR), the tyrosinase-related protein 1 (TYRP1), the OCA2 protein, and the endothelin receptor B (EDNRB). In a recent work, we investigated the involvement of 338 candidate SNPs in genetic predisposition to melanoma by using a dedicated chip to 110 pigmentation genes as part of a French case-control study (1069 melanoma patients, 925 controls), and found new biomarkers associated with CM risk belonging to genes involved in melanocyte differentiation, melanosome biogenesis, or melanogenesis, all of them having an additive effect on CM risk. Therefore, genetic variation in the pigmentation pathway seems to be crucial in controlling melanoma susceptibility.

IL19

Murine models: coat color and melanoma

L. Larue
Institut Curie, Orsay, France

Melanocytes are derived from neural crest cells. Genetic defaults may lead to coat color abnormality, early graying and melanoma. Melanoma is a very aggressive tumor and is responsible of the death of 80% of the patients having a cancer of the skin. Incidence in western countries continues to rise. In order to better understand initiation and progression of melanoma, our research based on normal and pathological development of melanocytes combine (i) molecular approach based on a better understanding on cellular signalization associated with b-catenin involving Wnt, PI3K, Map-kinase and cadherins; (ii) cellular approach based on our understanding on the establishment, maintenance and alteration (transformation and hypo/hyperpigmentation) of this lineage and (iii) a pathophysiological approach, based on murine models. We will present original mouse murine models presenting coat color abnormalities and affecting BRAF, NRAS, INK4A, CTNNB1, PTEN and CDH1, which allowed a better understanding in proliferation, immortalization and invasion.

IL20

Vitiligo 2011 update

S. Moretti
Department of Dermatology, University of Florence, Florence, Italy

Latest research efforts have improved our current knowledge of vitiligo and vitiligo patients. From the clinical standpoint, the Vitiligo European Task Force presented a consensus definition of the disease and a method to assess treatment outcome using a system which combines analysis of disease extension, stage of disease (staging), and disease progression (spreading), which is now routinely applied in several vitiligo centres. Although mixed forms have recently described, segmental and non segmental vitiligo (NSV) were clearly demonstrated to be different entities because the features of inflammation and autoimmunity were strongly linked only to NSV, together with a familial background of vitiligo and autoimmunity. New attention has been turned toward subjective symptoms and discomfort of patients, and a strict collaboration between doctors and vitiligo patients' associations led to the identification of top research areas for the treatment of this pigmentation disorder, including systemic immunosuppressants, topical treatments, light therapy, melanocyte-stimulating hormone analogues, gene therapy, and the impact of psychological interventions on the quality of life of patients with vitiligo. In addition, the importance of Koebner phenomenon has been recently highlighted by the Vitiligo European Task Force, proposing a new scoring system to allow an evaluation of Koebner phenomenon in daily practice or in experimental studies. Concerning pathobiology, vitiligo is still a disease not well understood. There is a strong body of biological evidence supporting an autoimmune basis for most cases of generalized vitiligo, although a clear definition of autoimmune vitiligo is still lacking. Genetic studies pointed out that generalized vitiligo behaves as a complex trait, being a polygenic, multifactorial disease, and some new vitiligo susceptibility genes have been identified in the last years; some of which are associated with other autoimmune diseases. Recent studies have suggested that the modifications of lipid membrane components in vitiligo cells may be the biochemical basis for the mitochondrial impairment and the subsequent production of intracellular reactive oxygen species following exposure to a mild stress. Additionally, new possible repair mechanisms for DNA damage have been proposed in vitiligo.

IL21

Acrofacial and genital depigmentation is a new pattern disease with features of both vitiligo and vitiligoid lichen sclerosus – a clinical and histopathological review of 58 cases

V. R. Attili, S. K. Attili
Visakha Institute of Skin & Allergy Visakhapatnam-Andhra Pradesh, India

Vitiligoid lichen sclerosus (VLS) is a superficial variant of lichen sclerosus (LS). Clinical lesions mimic vitiligo due to subtle textural changes which may not be obvious on clinical examination. It has a predilection for oral/genital and acral parts of the body similar to that of acrofacial/lip-tip vitiligo, considered as a limited form of generalized vitiligo. Our objective was to differentiate acrofacial vitiligo from VLS by histopathological assessment.

© 2011 The Authors. Journal Compilation © 2011 John Wiley & Sons A/S

In a prospective study over 5 yr, 58 patients that presented with concurrent acrofacial vitiligoid depigmentation were subjected to clinical and histopathological assessment. VLS was diagnosed when histological features were consistent with LS.

Genital depigmentation was associated with acrofacial depigmentation in 40 of 58 patients. Atypical clinical lesions were observed with symmetrical acral punctate or guttate lesions merging to form larger vitiligoid lesions in 29 and sclerotic/atrophic clinical lesions in 23 cases. Among 78 biopsies screened, LS was diagnosed in 22 of 24 (92%) genital, 22 of 30 (73%) orofacial and 13 of 24(54%) acral lesions.

A high incidence of LS/VLS mandates a review of acrofacial and mucosal depigmentation as specific forms of vitiligo. An association of vitiligo and LS is unlikely since generalized vitiligo has not been reported to be associated with LS. Since stereotypical LS/VLS is also not known to show symmetrical acral vitiligoid depigmentation, this pattern which combines clinical and histological features of both vitiligo and LS appears to be unique disease. It should be recognized as 'Acral vitiligoid lichen sclerosus'.

IL22

Stress responses, a bridge between physiological and pathological processes in melanocytes

M. Picardo
San Gallicano Dermatologic Institute Rome, Italy

Stress responses render cells prone to counteract dangerous stimuli saving them from destruction or irreversibie damage. Under physiological conditions melanocytes differentiation program involves activation of MAP kinase and antioxidant stimulation. Moreover the degree of pigmentation of cultured melanocytes is correlated with the antioxidant activities. Membrane associated signalling kinases and nuclear receptor-dependent transcription factor activation, considered as stress adaptators, also play a central role in controlling melanocyte proliferation, differentiation and pigmentation. UV induced melanogenesis correlates with p38 and ERK phosphorilation and stimulation of transcription factors controlling intracellular detoxifying enzymes (Nrf2). Catalase over-expression and its association with melanosome have been demonstrated following alfa-MSH stimulation suggesting that melanosomal transfer to keratinocytes upgrade both the physical and biochemical defences toward the dangerous effects of UV. In addition intermediates of melanogenesis are capable of inducing keratinocyte differentiation and reduction of UVA induced apoptosis. On the contrary melanocyte degeneration occuring in vitiligo is associated with an increased intracellular ROS production, alteration of the antioxidant pattern, modification of the intracellular trasduction pathways and increased sensitivity to external pro-oxidant stimuli. In adittion the continuos UV irradiation of the skin inducing fibroblast photoaging leads to an umbalce of the antioxidant status which is associated to an overexpression of growth factors able to mantain hyperpigmentary lesions. All these data confirm, at biological level, that pigmentation is a stress response phenomenon of the skin to different dangerous stimuli.

IL23

How does solar UV radiation initiate specific cellular responses?

M.-D. Galibert
CNRS-UMR6061, Institut de Génétique et Développement, Universite de Rennes 1, France

The sun is required and responsible for the development and continued existence of life on Earth, although it triggers deleterious side effects. The skin the most exposed body organ is constantly challenged by ultraviolet rays that comprise UVA (400–320 nm), UVB (320–290 nm), and in particular circonstances UVC (290–200 nm). UV rays monitor specific skin responses, based on the wavelength, with shorter wavelength being more photobiologically active, the UV-dose and the duration of the exposure (acute versus chronic). Skin exposure to solar radiation will thus initiates complex and specific molecular processes. These include the protective tanning response, local inflammation, immune suppression, and DNA damage that can lead to skin carcinogenesis. Understanding how transcription factors interpret the UV output from signal transduction pathways to drive distinct programs of gene expression is a key issue that is still under investigation and will lead to decipher disease development.

IL24

Introduction to the neuroendocrinology of the pigmentary system

A. Slominski
University of Tennessee HSC, Memphis, TN, USA

The field of the neuroendocrinology of the pigmentary system was funded with proposals that L-tyrosine and L-DOPA can act as hormone-like bioregulators with melanocytes acting as targets and producers of these molecules (*J. Theor. Biol.* 1990: 143, 123–138, *Mol. Cell Endocrinol.* 1994: 99, C7–11), and that melanocytes are sensory and regulatory cells of the epidermis with computing capability, that transform external and/or internal signals/energy into organized regulatory networks for the maintenance of the cutaneous homeostasis (*J. Theor. Biol.* 1993: 164, 103–120). Due to their exposed location in the skin, the body's largest as well as most directly and constantly environmentally threatened organ, melanocytes primarily transduce environmental cues (*Physiol. Rev.* 2004: 84, 1155–1228, *Exp. Dermatol.* 2009: 19, 760–76) that include but are not limited to the UVR as well as numerous intraepithelial and systemic signals into complex auto-regulatory local networks of the skin neuroendocrine system (*Endocrine Rev.* 2000: 21, 457–487; *Drug Discovery Today*: Dis Mech 2008: 5, e137–e144) that has established in the periphery for the maintenance of cutaneous homeostasis (*J. Clin. Invest.* 2007: 117, 3166–3169). Melanogenesis and melanin transfer via melanosomes into keratinocytes is a particularly prominent response pattern of melanocytes with a capability of inducing several local and perhaps systemic bioregulatory responses due to the intrinsic nature of melanosomes, melanin and intermediates of melanogenesis (*J. Theor. Biol.* 1993: 164, 103–120, Anticancer Res. 1998: 18, 3709–3716, Physiol. Rev. 2004: 84, 1155–1228; Exp. Dermatol. 2009: 19, 760–763). Consistent with their neural crest-origin, melanocytes have preserved sensory (expression of wide range of receptors), regulatory (production of classical stress neurotransmitters, neuropeptides and neurohormones (Endocrine Rev. 2000: 21, 457–487, Physiol. Rev. 2000: 80, 979–1020, FASEB J. 2001: 15, 1678–1693, Front Biosci. 2006: 11, 2230–2248, FASEB J. 2005: 19, 176–194, Trend End. Metab. 2008: 19, 17–24) and computing properties that allow them to serve as primitive 'neurons of the skin' (Exp Dermatol. 2009: 19, 760–76). These neuroendocrine activities of melanocytes are profoundly regulated by environmental stimuli including UVR and biological and chemical insults and the production of the signaling molecules is hierarchical and displays typical negative or positive feedback loops, following the algorithms of classical neuroendocrine axes (e.g. hypothalamic pituitary adrenal axis (Mol. Cell Endocrinol. 2007: 265–266, 143–149), hypothalamo-pituitary-thyroid axis (J. Invest. Dermatol. 2002: 119, 1449–1455, J. Clin. Endocrinol. Metab. 2008: 93, 4381–4388) serotoninergic/melatoninergic (FASEB J. 2005: 19, 176–194, Trend. End. Metab. 2008: 19, 17–24), catecholaminer-

 © 2011 The Authors. Journal Compilation © 2011 John Wiley & Sons A/S

gic (Exp. Dermatol. 2008: 17, 395–404) and cholinergic systems (J. Invest. Dermatol. 2006: 126, 1948–1965). Melanocyte-derived signals may also activate cutaneous sensory nerve endings to alert the central nervous system (Endocrine Rev. 2000: 21, 457–487, Exp. Dermatol. 2009: 19, 760–76). Also melanocytes might contain receptors for visible and UV spectra of solar radiation, as originally proposed by M. Lerner and J. Pawelek, respectively, a subject deserving future careful studies. These remarkable capabilities of melanocytes indicate that they are far more than simple pigment producing cells but represent an important element of the local neuroendocrine system defining them as sensory and regulatory neuroendocrine computing systems that have co-evolved during the evolution of stress response systems in vertebrates and perhaps non-vertebrates.

IL25

Modulatory effects of a small peptide derivative of α-MSH, KdPT, on melanocyte responses to oxidative stress

M. Böhm, M. Apel, T. A. Luger, A. Kokot
Department of Dermatology, Münster, Germany

It is well known that α-MSH has pigment-inducing and cytoprotective effects in human melanocytes. This natural peptide has also an ever increasing number of anti-inflammatory properties that may be useful for the treatment of immune-mediated inflammatory diseases. However, topical therapy with such a peptide has never been realized in routine dermatology. We have thus started to explore the functional properties of Lys-d-Pro-Thr (KdPT), a tripeptide derivative of the C-terminal domain of α-MSH. This small peptide has a MW of <400 Da and may thus be suitable for topical therapy in dermatology. We could recently demonstrate that KdPT neither binds to Mc1r expressing B16.F1 melanoma cells nor affects melanogenesis in these cells as well as in normal human melanocytes. Since KdPT reduces IL-1β-induced signaling, accumulation of reactive oxygen species and proinflammatory cytokine expression in non-melanocytic cells, we wondered if this agent has any salutary effects in normal human melanocytes. Using 4-tertiary butyl phenol (4-TBP) as an established pro-oxidative cellular stressor we could demonstrate that 4-TBP-induced apoptosis is dose-dependently reduced by KdPT. Interestingly, KdPT per se moderately but significantly increased the expression of the transcription factor nuclear factor erythroid 2-related factor (Nrf2) and heme oxygenase-1 (HO-1) at mRNA and protein level pointing towards an indirect antioxidative effect of the peptide. Interestingly, when normal human melanocytes were preincubated with KdPT, 4-TBP-induced expression of Nrf2 and nrf2-dependent genes (HO-1, γ-GCS, GSTPi and NQO1) was significantly attenuated. In summary, our findings provide first evidence for a modulatory effect of KdPT in normal human melanocytes. The data further demonstrate that this small peptide has some protective effects against 4-TBP-induced apoptosis presumably by modulating the phase II detoxification enzymatic machinery. It will be very interesting to further assess the protective role of KdPT in adequate in vivo models of vitiligo.

IL26

Monochromatic light for treatment of vitiligo

C.-C. E. Lan
Department of Dermatology, National Cheng Kung University College of Medicine and Hospital, Kaohsiung, Taiwan

Vitiligo is an acquired depigmentary dermatosis affecting at least 1% of the world population. Treatment for vitiligo is considered difficult and often results in unsatisfactory outcome. Phototherapy is an important treatment option for vitiligo, and currently, there is no consensus on how to optimally utilize the armamentaria available. Our laboratory has established several platforms to recapitulate the repigmentation processes of vitiligo in vitro. Combining our clinical experiences with laboratory results, this presentation focuses on how to optimally use monochromic light (308 and 632.8 nm) for treatment of vitiligo. Clinically, excimer light has been observed to induce vitiligo repigmentation more rapidly than other UVB emitting devices. In our in vitro studies, we demonstrated that at similar doses, excimer light imparted different biological effects on primitive pigment cells as compared to its NBUVB counterpart. Both nuclear DNA and cytoplasmic tryptophan are recognized photoacceptors for UVB treatment. After irradiation at comparable doses, excimer light was able to induce primitive pigment cell differentiation through efficient activation of cytoplasmic pathway that involved formation of tryptophan derivate to activate aryl hydrocarbon receptor-related cascade while NBUVB failed to do so. Low-energy Helium-Neon laser is another monochromatic light source used to treat vitiligo especially for pediatric patients and anatomical regions not suitable for UV treatment. We showed that He-Ne laser is able to induce primitive pigment cell differentiation via mitochondrial-retrograde signaling. In addition, we also established treatment response predictive assay to select patients suitable for low-energy visible light treatment. Therefore, visible light may be considered as treatment of choice in certain clinical settings. In summary, monochromatic lights are important therapeutic modality for treating vitiligo. By elucidating the mechanisms involved in how lights at different wavelengths induce vitiligo repigmentation, physicians will be able to provide individualized phototherapy tailored to patients' specific conditions.

IL27

Experience of surgical procedures in childhood vitiligo

S. V. Mulekar
National Center for Vitiligo & Psoriasis, Riyadh, Saudi Arabia

Most cases of vitiligo are acquired early in life with majority of patients showing the first symptoms of the disease before the age of 20 yr. The prevalence of segmental vitiligo has been found to be higher in children compared to adults. The psychological impact in children can be devastating and affects their self esteem, and competency. Phototherapy and topical steroids, which are most commonly used for adult vitiligo, are less useful for childhood vitiligo. Surgical techniques like skin grafting may be preferred but are not recommended for the technical difficulties like prolonged immobility and difficult to treat anatomical sites. Lack of expertise in techniques such as non cultured cell transplantation may be a limiting factor to recommend surgical treatment. We have an experience of treating 122 children with vitiligo, two with nevus depigmentosus and one post burn leukoderma. Of fifty six patients with unilateral vitiligo (segmental 39, focal 17) treated with noncultured melanocyte transplantation 36 had good to excellent repigmentation. We do not have follow up of seven patients due to recent surgeries. Repigmentation in patients with nevus depigmentosus is not of high cosmetic quality though the extent of pigmentation may be excellent. One child with post burn leukoderma pigmented almost completely. The follow up period ranged from 9 to 54 months. All the procedures were performed under intravenous anaesthesia using Propaphol and Sufrantenyl. The anaesthesia was uneventful and we did not experience any untoward effect except nausea in some patients. All the patients were co-operative and retained the dressing for the required duration.

© 2011 The Authors. Journal Compilation © 2011 John Wiley & Sons A/S

IL28

Pigmentation in non-mouse models – fishing for insight, not just horsing around?

R. N. Kelsh
Department of Biology & Biochemistry and Centre for Regenerative Medicine, University of Bath, UK

Pigmentation is a prominent feature of almost all vertebrates and consequently numerous species have been used in its study. Whilst the mouse, with its superb genetics and long history of collecting mutants, is the dominant model system, other models offer specific advantages. In this talk I will be briefly outline the roles played by some of these complementary systems. Whereas mammals have just melanocytes and so are depleted in their complement of pigment cell-types, poikilothermic vertebrates show the full diversity, with up to seven different types. Amphibians, and more recently fish, have provided excellent opportunities to characterise these different cell-types and their origin from neural crest. Given the excellent genetics of fish and the genetic and embryological accessibility of their embryos, it is understandable that fish models, particularly zebrafish and medaka, have been the source of substantial insight into pigment cell development. These studies have begun to characterise the intermediate steps in the development of pigment cells from the neural crest, and have identified numerous genes required for their development. These systems allow fine dissection of the processes of pigment cell specification, survival and differentiation, and are now being combined with mathematical modeling to allow a more rigorous understanding of the gene regulatory networks of pigment cell development. Both Xenopus and fish embryos are highly amenable to small molecule screening in vivo, and have identified many compounds with specific effects on pigment cell development; where such compounds affect melanocytes, they can often be applied to study of mammalian cells too. Fish models of melanoma have a long history and will continue to provide excellent opportunities for dissection of the mechanisms of cancer formation and progression and for drug development. One novel application of fish pigmentation concerns the recent design of an iridophore-based assay for receptor tyrosine kinase inhibitors. The fascinating topics of pigment pattern formation and pigment pattern evolution have been dependent upon identification of tractable case studies, with Danio fish, dogs, mice and diverse birds being most important to date. Finally, domestic animals, especially dog, chicken and horses, provide excellent case studies for analysing the comparative genetics of pigmentation. Despite the dominance of the mouse (and human) systems, other mammalian and non-mammalian species have made major contributions and will continue to do so.

IL29

Chicken models for vitiligo and other spontaneous autoimmune/autoinflammatory disorders

G. Erf
University of Arkansas, Fayetteville, AR, USA

The immune system has developed many effective ways to protect an individual from environmental insults and disease. However, responses that lead to specific attack of self components or to uncontrolled destructive immune activities can occur, resulting in autoimmune disease. Autoimmune diseases generally are complex diseases controlled by multiple risk factors that evolve and interact together. Knowledge of the interrelationship between genetic susceptibility and environmental risk factors in the etiology of these immune-mediated diseases is important for disease prevention and treatment. Genetic susceptibility may be manifested in an inherent target cell defect as well as in aberrant immune activity at various levels. The role of environmental factors in the development of autoimmune diseases is also multifaceted. Microbial infections, tissue injury, exposure to chemicals, pollutants and other stress factors have been associated with triggering the expression of autoimmune disease in susceptible individuals. Unfortunately, the relative contribution of genetic, environmental, and immune system factors to the development of autoimmune disease cannot be easily dissected. In order to understand the initial etiologic and pathogenic events, appropriate animal models are required. In this respect, models that spontaneously develop the disorder reflect the complex nature of the disorder much more closely than models with experimentally-induced autoimmune disease. For autoimmune diseases there are several well established chicken models, including the Obese strain chicken which develops Hashimoto's thyroiditis, the UCD 200/206 lines of chicken which develop a scleroderma-like condition, and the Smyth line chicken which develops autoimmune vitiligo. Other non-communicable disorders in poultry suspected to be autoimmune include peripheral neuropathy, ovarian autoimmune disease, and idiopathic pulmonary arterial hypertension. With the successful completion of the sequencing of the chicken genome in 2004 and the growing availability of research reagents for avian species, interest by the biomedical research community in the unique features and possibilities provided by the avian disease models has been renewed. Current research in the Smyth line chicken model for autoimmune vitiligo has incorporated whole animal research with cellular and molecular approaches that have led to important insights into the complex nature of this polygenic multifactorial disorder.

IL30

Roles for MITF in melanomagenesis

D.E. Fisher
Mass General Hospital, Harvard Medical School, Boston, MA, USA

'Abstract not available'

IL31

Regulation and Function of the PI3K Pathway in Advanced Melanoma

M. Davies
The University of Texas MD Anderson Cancer Center, Houston, TX, USA

'Abstract not available'

IL32

Oncogenic signaling downstream of Gq/11

B. Bastian
Department of Pathology, Memorial Sloan-Kettering Cancer Center, New York, NY, USA

'Abstract not available'

IL33

RAS and RAF signalling in melanoma: translating biology into therapies

R. Marais
Division of Cancer Biology, Institute of Cancer Research, London, UK

'Abstract not available'

 © 2011 The Authors. Journal Compilation © 2011 John Wiley & Sons A/S

IL34

New developments in melanoma therapy – ASCO update

C. Garbe
Universität Tübingen, Hautklinik, Sektion Dermatologische – Onkologie, Tübingen, Germany

Two exciting developments in melanoma therapy were reported at ASCO 2011 as plenary lectures and simultaneously published as NEJM articles. Vemurafenib (PLX4032) was found to be highly active in the BRIM3 trial in patients with BRAF V600E mutation and achieved a hazard ratio of 0.37 for melanoma specific deaths in comparison to dacarbazine treatment, and the hazard ratio for tumor progressen was 0.26. Median follow-up at the reported interim analysis was only 3.8 months for patients in the vemurafenib group and 2.3 months for those in the dacarbazine group. In spite of this very short follow-up time the results were very impressing. Results from the BRIM2 trial with a median follow-up of 10 months in 132 patients treated with vemurafenib showed more elaborated survival data. While median overall survival time was not yet reached, the 12 months survival rate was 58% and the objective response rate was 53%. The median progression-free survival time was 6.7 months. A first report on the combination of the specific BRAF inhibitor GSK'436 with the MEK inhibitor GSK'212 showed ongoing responses in 83% of treated patients after a median follow-up of 5 months. Ipilimumab, a fully human, IgG1 monoclonal antibody, blocks cytotoxic T-lymphocyte–associated antigen 4 (CTLA-4), a negative regulator of T cells, and thereby augments T-cell activation and proliferation. A randomized trial in 502 patients with previously untreated metastatic melanoma was reported, comparing treatment with ipilimumab (10 mg/kg) plus dacarbazine to dacarbazine plus placebo. Overall survival was significantly longer in the group receiving ipilimumab plus dacarbazine than in the group receiving dacarbazine plus placebo (11.2 months versus 9.1 months), with higher survival rates in the ipilimumab–dacarbazine group at 1 yr (47.3% versus 36.3%), 2 yr (28.5% versus 17.9%), and 3 yr (20.8% versus 12.2%). Toxicity was moderate and manageable. These new treatment modalities are presently available in early access programs.

IL35

Chemoprevention of melanoma progression mediated by NO/neural NO synthase (nNOS)/NO, an accelerator of the transformation process

F. L. Meyskens, S. Yang, T. Poulos, H. Ji, R. B. Silverman
Chao Family Comprehensive Cancer Center, Department of Medicine, Irvine, CA, USA

Our laboratory has been extensively involved in the study of abnormal redox status and redox-sensitive signalings such as AP-1, NF-κB and more recently APE/Ref-1. Our overall goal is to identify key factors underlying melanoma development that can be exploited for therapeutic intent. It has been well-documented that UVR exposure, especially sunburn at a young age, is particularly linked to melanoma incidence. As an important environmental carcinogen, UVR not only generates ROS, but also produces a large amount of nitric oxide (NO) in human skin. Utilizing a NO-donor (DETA) to mimic NO stress, we demonstrated that melanoma proliferation and invasion potential were significantly stimulated by and associated with induction of many proteins involved in cell growth (c-Jun, JunD), anti-apoptosis (Bcl-2, APE/Ref-1) and metastasis signalings (MMP-1, Snail). Notably, long-term exposure of DETA/NO stress resulted in over-growth of primary normal human melanocytes with formation of foci in in vitro culture, indicating gain of additional vertical growth potential. As melanocytes originate from the neural crest, we propose that neural NO synthase (nNOS) plays an important role in generating NO and mediating NO stress in human melanoma. Both our in vitro cell culture (Immunoblotting) and in vivo human biopsy (Immunohistochemistry) studies demonstrated marked elevation of nNOS expressions in melanoma cells. More interestingly, induction of nNOS and elevated NO levels were evident after UV-B radiation or incubation with bFGF. Knockdown of nNOS with siRNA efficiently reduced the DETA-induced melanoma proliferation and invasion potential, with marked reduction of c-Jun, Bcl-2 and MMP-1. A novel synthesized nNOS inhibitor (JI-11) was utilized to inhibit nNOS activity; our data showed that co-treatment of JI-11 (1 μM) significantly attenuated the alterations induced by UV-B radiation and DETA/NO treatment. We have tested a series of novel synthesized nNOS inhibitors representing a varied range of structures and distinct potentials and enzyme selectivity. The nNOS selectivity of Ki/nNOS values (but not Ki/iNOS or Ki/eNOS) were statistically significantly correlated with their observed anti-melanoma potency and anti-invasion activity, indicating that the inhibition of nNOS is crucial for reducing melanoma invasion. Through extensive structure-activity analysis and chemical modification, we are improving the bioavailability and anti-melanoma potential of nNOS inhibitors. Based on our studies, we propose that targeting nNOS with application of specific synthetic inhibitors to diminish NO stress represents an innovative and promising strategy for the chemoprevention of cutaneous melanoma.

IL36

Endolysosomal pathways in melanoma maintenance and drug response

D. Alonso-Curbelo, D. Olmeda, T. G. Calvo, E. Pérez-Guijarro, M. S. Soengas
Centro Nacional de Investigaciones Oncológicas, Madrid, Spain

Malignant melanoma remains a paradigm of aggressive cancers of poor prognosis. Large efforts aimed at the identification of melanoma-associated oncogenic events have identified a variety of genetic and epigenetic defects in cell cycle and cell death modulators. Still, very little is known about the role of endo/lysosomal compartments in melanoma maintenance. This is relevant because mounting evidence suggests that deregulation of membrane dynamics may favor tumor cell division, cell proliferation, metastasis and survival. Moreover, lysosomes are obligate executors of autophagy programs, which may both facilitate or inhibit tumor development. Here we have functionally characterized RAB GTPases regulating late-endocytosis, lysosomal degradation and macro-autophagy. These proteins were found to display differential expression levels in melanoma cells, compared to normal melanocytes and to other non-melanocytic tumor cell lines. RNA interference revealed new roles of RAB GTPases in melanoma proliferation, cellular morphology, and migration/invasion dynamics. Moreover, melanoma xenografts in murine models unveiled an unexpected RAB-dependent remodeling of tumor microenvironment in vivo. Altogether, our results support new roles of endo/lysosomal RAB-dependent vesicle trafficking in melanoma maintenance and progression.

IL37

Impacts of p16 deficiency on melanocyte gene expression and biology: relation to early melanoma

D. M. Kallenberg, J. K. Soo, E. V. Sviderskaya, D. C. Bennett
St George's University of London, UK

Single oncogenic mutations in melanocytes can produce naevi (moles), growth-arrested via senescence, mediated partly by p16

© 2011 The Authors. Journal Compilation © 2011 John Wiley & Sons A/S

(product of melanoma locus CDKN2A). Melanoma growth requires evasion of senescence: most or all melanomas have defects in the p16 pathway. Human melanomas undergo a radial growth phase (RGP: thin and largely within the epidermis) and/or a vertical growth phase (more invasive). The radial shape may involve keratinocyte-dependence, since low-passage RGP cultures without keratinocytes show poor growth and frequent apoptosis, suppressed by keratinocyte-derived factors (stem cell factor+ endothelin 1: SCF + EDN1). This may arise from p16 loss, since cultured p16-null human and mouse melanocytes likewise grow poorly, but much better with keratinocytes or SCF + EDN1. We have been analyzing the mechanisms of the keratinocyte-dependence in two p16-null human melanocyte strains, using TUNEL and caspase 3/7 assays, growth curves and BrdU assays. Immortalization was by retroviral transduction of TERT. Nonimmortal p16-null melanocytes grown without keratinocytes showed low proliferation rates as well as frequent apoptosis, both alleviated by SCF + EDN1. Inhibition of p53 activity using pifithrin-alpha led to incomplete suppression of the apoptosis, indicating a contribution by other pathways. TERT-immortalized sublines of the p16-null strains showed some markers of cellular transformation: reduced growth-factor dependence, increased saturation densities and reduced anchorage-dependence. The findings are consistent with melanoma invasion out of the epidermis being inhibited by p16/RB deficiency but promoted by immortalization via TERT.

IL38

Monobenzome increases the immunogenicity of melanoma cells, and is effective as melanoma immunotherapy

R. M. Luiten
Academic Medical Center, University of Amsterdam, Amsterdam, The Netherlands

Based on its immunogenicity, immunotherapy of melanoma is extensively studied as a new type of treatment. Since treatment-related skin depigmentation is considered a favourable prognostic sign during melanoma intervention, we here aimed at the reverse approach of inducing vitiligo to achieve effective anti-melanoma immunity. The phenolic compound monobenzone induces progressive skin depigmentation that is clinically and histologically indistinguishable from vitiligo vulgaris. Here, we present a comprehensive study of how monobenzone increases the immunogenicity of melanoma cells, leading to anti-melanoma immunity and melanoma growth inhibition. We showed that monobenzone inactivates tyrosinase enzyme in melanoma cells, leading to decreased cellular melanin synthesis, as well as the formation of quinone metabolites that bind to cysteine residues in proteins, thereby forming quinone haptens to melanosomal proteins. Moreover, monobenzone-tyrosinase interaction induced increased levels of reactive oxygen species, and the release of tyrosinase- and MART-1-containing CD63+ exosomes from monobenzone-exposed melanoma cells. We also found that monobenzone induces ubiquitination of tyrosinase as well as melanosome autophagy; a lysosomal degradation process leading to the targeting of tyrosinase to MHC class-II compartments. Monobenzone-exposed melanoma cells activated human dendritic cells, thereby generating a signal for immune activation, leading to the activation of melanoma-reactive human CD8+ T cells, in contrast to unexposed melanoma cells. Together, these data show that monobenzone, by its specific effects on the enzyme tyrosinase, induces potent CD8+ T cell immunity against auto-antigens expressed by melanocytes and melanoma cells. Monobenzone induced a melanoma antigen-specific immune response in vivo, incorporating NK-, B- and T cells, which abolished subcutaneous B16.F10 melanoma growth in up to 85% of C57BL/6 mice, when combined with immune-stimulatory imiquimod cream and CpG oligodeoxynucleotide injections (MIC therapy). Importantly, this regimen induced over 100 days of tumor-free survival in up to 60% of the mice, and forcefully suppressed tumor growth upon re-challenge after MIC treatment cessation. Based on these results, we have started a clinical trial in melanoma patients.

IL39

KIT aberrations in melanoma and therapeutic implications

R. D. Carvajal
Memorial Sloan-Kettering Cancer Center, New York, NY, USA

The identification of distinct molecular subgroups of melanoma is becoming of increasing therapeutic relevance. The most common melanoma subtype in the US arises from non-chronically sun-damaged (non-CSD) skin and often harbors activating mutations in BRAF. Melanoma arising from mucosal, acral, and CSD sites infrequently have BRAF mutations, but commonly have amplifications or activating mutations of KIT. The importance of KIT in normal melanocyte development is well-established; however, its role as an oncogene and therapeutic target in melanoma has only recently become clear. While KIT is expressed in some melanomas, loss of expression is observed with progression of disease from superficial to invasive to metastatic stages, suggesting that KIT possesses tumor suppressive functions. Furthermore, three phase II studies of metastatic melanoma treated with imatinib mesylate (IM), an orally available ATP-competitive inhibitor of several tyrosine kinases including KIT, did not demonstrate clinical activity. These trials accrued before the discovery of activating mutations of KIT in melanoma and did not select patients based upon the presence of KIT mutations or amplification. KIT is an established therapeutic target in cancers with activating mutations of KIT such as gastrointestinal stromal tumors (GIST), and significant benefit is achieved with various small molecule inhibitors of KIT including IM. Several melanoma cell lines with KIT mutations are highly sensitive to IM. Furthermore, several patients with melanoma harboring KIT alterations, including a K642E mutation as well as a seven-codon duplication of exon 11, have been reported to achieve major durable responses to IM. Given the preclinical and anecdotal clinical activity of IM observed in KIT mutant melanoma, we and others have conducted clinical trials of IM and other inhibitors of KIT to test the hypothesis that inhibition of KIT in a molecularly selected subgroup of patients with melanomas harboring mutations or amplification of KIT will result in objective regression and disease control. In our multicenter phase II study of IM in melanoma harboring somatic alterations of KIT, we screened tumor samples from 295 patients with melanoma for the presence of KIT mutations or amplifications and identified 51 cases with such alterations. KIT mutations or amplifications were identified in 23.8% of acral melanomas, 24.7% of mucosal melanomas, and 18.8% of melanomas arising from skin with histopathologically signs of chronic sun-induced damage. Twenty-eight of the 51 patients whose tumors harbored KIT mutations or amplification were treated with IM: 13 (46%) with mucosal melanoma, 10 (36%) with acral melanoma, and 5 (18%) with melanoma arising from CSD sites (three arising from the head and neck and two arising from the trunk). Twenty-five were evaluable for the primary endpoint of response. Treated patients received IM 400 mg twice daily. We observed two complete responses lasting 94 (ongoing) and 95 weeks, two durable partial responses lasting 53 and 89 (ongoing) weeks, and two transient partial responses lasting 12 and 18 weeks amongst the 25 evaluable patients. The overall durable response rate was 16% (95% confidence interval, 2–30%), with a median

 © 2011 The Authors. Journal Compilation © 2011 John Wiley & Sons A/S

time to progression of 12 weeks (interquartile range, 6–18 weeks; 95% confidence interval, 11–18 weeks), and a median overall survival of 46.3 weeks (interquartile range, 28 weeks-not achieved; 95% confidence interval, 28 weeks-not achieved). To separate bona fide driver from passenger alterations of KIT, we sought evidence for tumor selection of specific mutations. We observed that IM has greater activity in tumors harboring recurrent KIT mutations found in melanoma or GIST, as well as in tumors with a mutant KIT allele in greater abundance than the wild-type allele. Response rate was superior in cases with mutations affecting recurrent hotspots or with a mutant to wild-type allelic ratio greater than one (40% versus 0%, P = 0.05) indicating positive selection for the mutated allele. When combining those whose tumors have an allelic ratio greater than 1 with those whose tumors harbor recurrent primary mutations found in GIST or melanoma, we observed a superior RR (40% versus 0%, P = 0.05), TTP (16 weeks versus 8.3 weeks; 95% CI, 11–35.6 versus 3.6–11.7 weeks; P = 0.02) and survival (80.5 weeks versus 28.3 weeks; 95% CI, 28.3-not reached versus 14.4–61.3 weeks; P = 0.04) when compared with other cases. We conclude that, in patients with advanced melanoma harboring KIT alterations, treatment with IM results in significant clinical responses in a subset of patients. Responses may be limited to tumors harboring KIT alterations of proven functional relevance.

IL40

TYRP1, a missing link between melanogenesis and melanoma progression?

G. Ghanem, F. Journé, A. Mogha, L. Van Kempen, M.-D. Galibert
Inst J Bordet, U Rennes 1 & Université de Liège, Belgium

Melanoma prognosis is based on specific pathological features of the primary lesion mainly Breslow thickness, mitotic rate, ulceration status and extent of lymph node involvement now included in the American Joint Committee on Cancer (AJCC) melanoma staging system. Excision of the primary lesion and sentinel node surveillance are quite effective in the management of early stages of the disease. In metastatic patients, the extent of lymph node involvement is also an important prognosis indicator. Many progression markers both in tissues and serum, including circulating tumor cells, have been studied but none is being used in the daily practice. There is an urgent need for reliable molecular prognostic markers to discriminate between clinical stages and predict disease progression especially with the recent very encouraging results with the anti-mutated BRAF and anti-CTLA4 strategies. Melanosomal proteins have been a focus for many studies and many has been proposed as markers and targets for therapy. One of these, is tyrosinase related protein 1 also known as gp75 glycoprotein (Tyrp1/gp75) is quite intriguing. It is the most abundant glycoprotein within both the normal and transformed melanocyte and yet has no obvious key functions. However, several observations made over the past 30 yr raise numerous questions: It shares structural homology with tyrosinase and even possess a catalytic copper-containing site but without obvious enzymatic function. By contrast, mutations in its related gene (brown locus) confers OCA3 type albinism. A part of the explanation can reside in its reported stabilizing effect on tyrosinase and an increased cell sensibility to oxidative stress in general. This may be in agreement with the reported TYRP1 gene polymorphisms associated with a higher risk for melanomas. A few in vitro and animal studies reported relationships between Tyrp1 protein expression and melanoma cell proliferation and invasion. However, our group showed a discrepancy between TYRP1 gene and protein expression, and demonstrated absence of a causal relationship between Tyrp1 protein expression and pigmentation. Furthermore, although Tyrp1 is regarded a melanoma differentiation marker, our data indicate that TYRP1 mRNA expression in metastatic tissue strongly correlates with poor survival. These data question its function in pigment synthesis and underscore our lack of knowledge of its mechanism of action. Nevertheless, in addition to previous Tyrp1-based vaccines, a clinical trial has been launched recently using a human anti-Tyrp1 antibody for melanoma immunotherapy based on positive preclinical in vitro and animal studies. This antibody may thus not target pigmented melanoma cells specifically, but could as well be used for clinically more aggressive amelanotic melanomas. A thorough understanding of Tyrp1 function is critical to correctly evaluate the outcome of such trials as well as its use as a target for therapy.

IL41

Congenital melanocytic naevus syndrome – clinical and genetic aspects

V. Kinsler
Paediatric Dermatology Department, Great Ormond St Hospital for Children, and Clinical Medical Genetics Unit Institute of Child Health, London, UK

The study of rare congenital diseases frequently illuminates the mechanisms governing normal fetal development, as well as commoner acquired diseases. The study of large/multiple congenital melanocytic naevi (CMN) has the potential to contribute to both areas of knowledge, acting as an example of abnormal neural crest development and as a predisposition to malignant melanoma. Ongoing clinical characterisation of associated features of CMN has recently resulted in the proposal of the term CMN syndrome, and the underlying genetics are currently being investigated using a large cohort of patients from Great Ormond Street Hospital.

IL42

Genomics and the molecular etiologies of congenital nevus formation

H. Etchevers
INSERM U910, Faculté de Médecine Université de la Méditerranée, Marseille, France

Both isolated and syndromic forms of congenital melanocytic nevi involve anomalies in the development, growth or differentiation of derivatives of the neural crest cell population. The primary, but not exclusive, cell type involved in this group of pathologies is the melanocyte. The time course of, and molecules involved in, its normal development within cutaneous and extracutaneous sites are incompletely understood. Recent technological advances in genomic analyses have enabled the elucidation of the molecular bases of a number of extremely rare and sporadic malformations that would have remained inaccessible for the tools of traditional molecular genetics. The large or giant congenital melanocytic nevus is such a rare condition. It can occur as an isolated hamartomous overgrowth of the melanocyte population or in association with melanocytosis of the central nervous system by way of the meninges, proliferative nodules and tumors and/or segmental disorganization of the overlying epidermal annexes, or underlying dermal, hypodermal and muscular layers. These clinical signs can orient hypotheses as to molecular etiologies. This presentation will broach the techniques used to test certain hypotheses currently under investigation using cohorts drawn from French and American patient populations, as well as the use of innovative animal models to address the result of perturbing specific cell signaling pathways during embryonic and fetal cutaneous development.

© 2011 The Authors. Journal Compilation © 2011 John Wiley & Sons A/S

IL43

The xeroderma pigmentosum syndrome: clinical, genetic and gene therapy issues

A. Sarasin
CNRS-UMR8200, Institut Gustave Roussy, Villejuif, France

Xeroderma pigmentosum (XP) is a rare, genetic DNA repair-deficient disease associated with profound sensitivity to sunlight and early onset of skin cancers. Various skin manifestations, ocular abnormalities and sometimes neurologic symptoms are found in severe forms of the disease. One of the seven genes (XPA to XPG) is mutated in classical XP, which is deficient in nucleotide excision repair (NER), while the POLH gene is mutated in the variant form of XP. The most common form in the world is the XP-C group. The XPC protein is involved in the early recognition of bulky DNA adducts on the non-transcribed genome. Germinal mutations on the XPC gene, with founder effect, have been found in North Africa and in the black population of Mayotte, a French island in the Indian Ocean, where we found the highest world prevalence of XP-C patients. The age of these mutations have been calculated to be, at least, as old as 1200 or 800 yr, respectively. Comparison between white-skinned Caucasian and black-skinned XP-C patients reveals major differences in terms of prevalence of skin cancers and ocular manifestations, confirming the major protection effect of melanin toward skin cancers, even in DNA repair-deficient patients. No treatment does exist for XP, except a full protection against UV-exposure. Genetic correction of XP-C skin cells (fibroblasts and keratinocytes) has been successfully obtained by using retroviruses expressing the wild type XPC gene or by using homologous recombination with plasmid DNA coding part of wild type XPC sequences. These complemented cells could be used in the future to produce small patches of corrected XP-C skin in vitro with the project of gene therapy. The NER pathway is able to remove a wide spectrum of bulky DNA lesions, including most damage induced by antitumoral drugs. Sensitivity or resistance of numerous human cancers to various chemotherapies, such as cisplatin or doxorubicin, is partially linked to the efficacy of NER. Genetic polymorphisms or mutations on the NER genes could be associated to therapy response and patient survival. It is important for clinicians to remember that non-tumoral cells of XP patients are very sensitive to antitumoral drugs and that cancer therapy should be adjusted for these patients when they are treated against tumors.

IL44

Xeroderma pigmentosum: clues to understand cancer initiation

H. R. Rezvani
INSERM U1035, Department of Dermatology and Pediatric Dermatology, CHU de Bordeaux, France

The works developed in the last decades on patients and cells of rare disorders of photoprotection indicates that these are very useful models to understand complex diseases such as cancer. As DNA damage and metabolism remodeling are two well-known hallmarks of cancer cells, we took advantage of the intrinsic genomic instability arising in type C xeroderma pigmentosum (XPC) to understand the inter-relationships between these two factors. We showed that lentivirus-mediated knockdown of XPC (XPCKD) in keratinocytes reduces mitochondrial oxidative phosphorylation and increases glycolysis, a hallmark observed in most cancer cells as shown by PET scans in the clinic. The critical step in this process is the activation of bypass repair systems which helps cells with unrepaired DNA escape senescence and death. In fact, activation of DNA-dependent protein kinase results in upregulation of AKT and NADPH oxidase-1 (NOX1) with a concomitant increase in reactive oxygen species (ROS) production, associated with specific deletions in mitochondrial DNA (mtDNA). The XPCKD containing mtDNA deletions are capable of forming squamous cell carcinomas (SCCs) when implanted into immunodeficient mice. Impairment of AKT or NOX activation in XPCKD cells blocks the formation of ROS, mtDNA deletions, and neoplastic transformation. The knowledge gained by studying XPC silencing-mediated tumoral transformation of normal human keratinocytes has given us a greater insight into the contribution of metabolism alteration and ROS accumulation to skin cancer. Further elucidation of the molecular mechanisms involved in skin cancer formation may ultimately lead to implement new strategies for the prevention of skin but of also of other cancers.

IL45

The human hair-bulb melanocyte: a model aging system for both our gray hair and our gray matter?

D. J. Tobin
Centre for Skin Sciences, School of Life Sciences, University of Bradford, Bradford, UK

Nature re-uses protective and defensive systems in complex organisms at both local and systemic levels, and often with remarkable self-similarity. Perhaps this is not too surprising where benefit is proven first at the local level/periphery, and where similar components of these systems are then reused during evolution in phylogenetically more highly-developed organisms with a central nervous system. A case in point is the co-utilization of similar melanocortin and opioid systems by central neurons and their peripheral neuro-ectodermal cousins – the melanocytes. We recently described in some detail this self-similarity for corticotrophin-releasing factor, pro-opiomelanocortin (POMC)-derived melanocortins (α-melanocyte stimulating hormone and adrenocorticotropic hormone), and the POMC-derived opioid β-endorphin in the hair follicle pigmentary unit. As long-living postmitotic neural crest-derived cells, epidermal melanocytes share several features with neurons such as dendritic morphology, utilization of tyrosine (melanins in epidermal melanocytes and catecholamines in neurons), expression of neuropeptides, neurohormones, neurotransmitters and a receptor profile that includes melanocortin receptors, adrenoreceptors, and neurotrophin receptors. Within this context, we are keen to understand how age-associated pigment cell decline and cognitive decline may be ‘physiologic’ when due to mild functional deficit or pathologic when associated with more severe (neuro)degenerative change. The former could involve subtle effects (e.g., synaptic alterations in neurons or modulation of melanocyte-keratinocyte crosstalk), while the latter involves significant neuronal or melanocyte loss through for example apoptosis. Although clearly trivial by comparison with neurodegeneration, aging individuals also exhibit ‘senile’ white hair or canities. Despite our increasing insights of the (co)regulation of melanocyte and epithelial stem cells in the hair follicle from murine studies, we know little about how death signaling in neural crest-derived cells is triggered ‘physiologically’ (eg. catagen or hair follicle regression) and how this differs from dysregulated or pathologic states (e.g. neurodegenerative disorders, canities, vitilgo). Given the relatedness of neurons and melanocytes, this author proposes that the hair follicle pigmentary unit is uniquely placed to unravel some of the mechanisms of neuronal cell aging and degeneration. Despite their common cutaneous origin hair follicle melanocytes appear to be more sensitive to aging influences than melanocytes in the epidermis, as evidenced by the slow loss of pigment tone with age in the epidermis contrasting with the marked dilution of hair color in canities. This is likely to reflect significant differences in the epidermal and follicular melanocyte microenvironments. Not only is there tight coupling of follicular pigmentation to the hair growth cycle, but

© 2011 The Authors. Journal Compilation © 2011 John Wiley & Sons A/S

there are also differences in stem cell niche capacity, niche exposure, and perhaps in the overriding effects of a dominant inheritance. The hair follicle may provide richer information in this context, as the life-histories of its various sub-populations of follicular melanocytes is very diverse. Pre-proliferative, proliferative, differentiated, terminally-differentiated and 'senescent' melanocytes all co-exist in the same growing hair follicle. Thus, there may be several roads that can lead to canities in the human (compared perhaps to mouse) hair follicle, especially when loss of pigmentation occurs within the same single anagen VI sub-stage of the hair growth cycle. Such scenarios could involve a plethora of potential aggressors for melanocytes, which may be similar to those experienced by other neural crest-derived cells. Damage may result due to variable capacity to accumulate, withstand, inactivate (oxidative) stress associated with either endogenous [e.g., 'physiologically-cytotoxic' (neuro) melanogenesis and aging] or exogenous [e.g., (geno)toxic stresses] insults. The availability of human hair follicle melanocyte culture methods has provided some impetus to dissecting the regulation of melanogenesis in the human hair follicle. However, neuronal models for research in neurogenerative disease have significant limitations as a result of their non-human origin and/or transformed state. Previously, Gilchrest and Yaar proposed the human epidermal melanocyte from neonatal foreskin as a model system for Alzheimer's disease research. These researchers found that pigment cells underwent apoptosis (like neurons) in the presence of β-amyloid. Moreover, they found that β-amyloid is a ligand for the 75-kDa transmembrane neurotrophin receptor (a member of the family of apoptotic receptors). We have extended these studies to show that amyloidogenic isoforms of amyloid precursor protein and β-amyloid1–40 can be detected in adult human skin melanocytes obtained from elderly donors. Incubation of these cells with aggregated β-amyloid1–40 peptide caused a concentration-dependent reduction in their viability, whereas age-matched dermal fibroblasts remained unaffected. Here we propose that the aging hair follicle pigmentary unit of scalp hair follicles could provide a most useful surrogate for studying age-associated neural cell decline, both of melanocytes and neural cells in general, including those with mechanisms involving amyloidogenic isoforms of amyloid precursor protein and the β-amyloid1–40 peptide.

IL46

VGICC: Objectives and priorities for international consensus on vitiligo clinical research

A. Taïeb, on behalf of Vililigo Global Issues Consensus Conference panelists
University Bordeaux Segalen & Bordeaux University Hospitals, France

The need for better standards in the field of vitiligo research has been underlined on several occasions. Based on previous group work among the Vitiligo European Task Force, and contacts with other international colleagues, it has been decided to draft an agenda focusing on the most pressing issues of consensus for clinical research. The following have been selected (i) Classification of vitiligo; (ii) Definition of stable disease; (iii) Definition of Koebner phenomenon; and (iv) Definition of 'autoimmune vitiligo'. A first round of discussion was organized in Seoul on 24 May 2011 during the 22nd World Congress of Dermatology, with representatives of all continents, with the generous help of the Korean Vitiligo Society. A draft of the consensus paper based on the contributions of all groups (Europe, Japon/Taiwan; Continental Asia/Singapour; Pacific; Africa; Middle East; North America; Central and South America) discussed in Seoul will be discussed in Bordeaux, and submitted for publication after the meeting. Already other important points need to be envisaged after the Bordeaux IPCC at the international level, such as the standardization of outcome measures.

IL47

Treatment guideline in segmental vitiligo

D.-Y. Lee
Department of Dermatology Samsung Medical Center
Sungkyunkwan University, Seoul, Korea

Vitiligo is classified into segmental and nonsegmental type. Each type has a different clinical feature and natural history. Segmental vitiligo often starts early in life. It is almost always limited to one segment of the body and does not go over the opposite side of the body. It tends to spread rapidly and be stabilized in a few years. Thus, the course of segmental vitiligo is predictable while that of nonsegmental vitiligo is unpredictable. White hairs seem to be always associated with segmental vitiligo. The preferential management for segmental vitiligo includes topical corticosteroids and immunomodulators, and phototherapy such as narrow-band UVB and excimer laser. If medical treatments are unsatisfactory, surgical treatment such as epidermal grafting can be considered. Recently, the treatment guideline for vitiligo was reported, but it has some limitations. Here, based on the previous reports and our experiences we propose a treatment guideline in segmental vitiligo. Recently we reported that medical treatments were not helpful in long-duration segmental vitiligo, demonstrating that disease duration is very important for the responsiveness of medical treatment. Thus, as a first line we recommend combination of topical therapy and phototherapy as soon as possible. In addition, we found that phototherapy was not helpful in the patients with segmental vitiligo who had the majority of white hairs in the lesional skin. Therefore, we recommend that if the majority of hairs are white in the lesional skin, as a first line surgical therapy is indicated to avoid unnecessary treatment and a waste of time instead of medical treatments. We expect that this guideline will provide definite decision for the treatment of segmental vitiligo.

IL48

The genetics of oculocutaneous albinism

B. Arveiler
Laboratoire Maladies Rares – Génétique et Métabolisme, Bordeaux, France

Oculocutaneous albinism (OCA) is a rare genetic disease characterized by generalized hypopigmentation of the skin, hair and eye, and by ophthalmologic abnormalities, caused by a deficiency in melanin biosynthesis. The definition of OCA subtypes formerly based upon clinical phenotype has moved towards a molecular classification based upon the identification of the causative genes. OCA is a genetically heterogeneous group of autosomal recessive disorders. There are four types of non-syndromic OCA: OCA1 caused by mutations in the TYR gene in 11q14.3, OCA2 caused by mutations in the OCA2 gene (formerly called P) in 15q11.2-q12, OCA3 caused by mutations in the TYRP1 gene in 9p23 and OCA4 caused by mutations in the SCL45A2 gene (formerly called MATP) in 5p13.3. In addition to OCA, X-linked ocular albinism (OA1) is due to mutations of the GPR143 gene. Syndromic forms of albinism include Piebaldism, Waardenburg syndrome (WS1–4), Hermansky-Pudlak syndrome (HPS1–7), Chediak-Higashi syndrome (CHS), and the very rare Griscelli syndrome (GS1–3). The molecular diagnosis of OCA relies mainly on the analysis of the coding regions of the OCA1–4 genes, searching for point mutations and intragenic microdeletions and microduplications. Although a few population-specific mutations have been found, the relative frequency of the different forms of OCA can be roughly estimated as follows:

© 2011 The Authors. Journal Compilation © 2011 John Wiley & Sons A/S

OCA1 50%, OCA2 30%, OCA3 3%, OCA4 17%. After the analysis of these four major genes, and, in some laboratories, of other genes such as GPR143, those responsible for some of the syndromic forms, and other candidate genes (TYRP2, SLC24A5, SILV, …), about 20% of patients remain without diagnosis, since either only one mutation or no mutation at all is identified. This suggests that other albinism genes remain to be discovered. In addition, it is worth noting that pigmentation is a complex process involving a large number of genes which may act as modifiers of OCA patients' phenotype.

IL49

What's new in albinism among Japanese 2011

T. Suzuki
School of Medicine, Yamagata University, Japan

Patients with oculocutaneous albinism (OCA) are characterized by reduced skin and hair pigmentation, and by reduced visual acuity and nystagmus. OCA can be classified into two groups. One is the non-syndromic type, which is usually classified into four resposible genes. Another type is the syndromic type, which reveals not only OCA but also other symptoms, e.g. bleeding tendency, immunodeficiency. The product of the responsible gene for the syndromic type works at the physiological level in the synthesis and trafficking of lysosome-related organelles. Thus, mutations only in one gene cause apparent unrelated multi-symptoms. Here, we summarize the characteristics of OCA found in Japanese population, and reveal some topics found in our study, including a patient recently diagnosed as OCA3 which is very rare in non-African populations.

IL50

Overview: skin lightening agents and melasma

H. Y. Kang
Department of Dermatology, Ajou University School of Medicine, Suwon, Korea

Melasma is a common hyperpigmentary disorder of the face. Increased epidermal pigmentation is the main target for melasma treatment. Hydroquinone, well known tyrosinase inhibitor, has been used as the gold standard for the treatment of melasma. The hydroquinone has been studied in combination with other agents such as corticosteroids or retinoic acid to provide greater therapeutic success and faster response than hydroquinone alone. The most widely used combination for treating melasma is hydroquinone, retinoic acid and corticosteroid, namely the so-called Kligman formula. There are evidences to support the use of fixed triple combination cream in the treatment of melasma. Glycolic acid has been used as adjuvant peeling agent in the melasma treatment. Recently, the therapeutic effects of oral or intradermal injection of tranexamic acid, plasmin inhibitor, in the treatment of melasma have been reported. Tranexamic acid has been found in vitro to inhibit melanin synthesis. Further work would be needed to understand how tranexamic acid improves melasma.

Abstracts of Oral Communications C1-C118
CS1: Developmental biology

C1

Ets1 interacts with Sox10 during murine melanocyte development

A. Saldana-Caboverde, L. Kos
Florida International University, Miami, FL, USA

Melanocytes are derived from pluripotent neural crest (NC) cells, which arise from the dorsal aspect of the neural tube. Several genes required for the specification of melanocytes from the NC have been identified via the study of mouse pigmentation mutants. Recently, the deletion of the transcription factor Ets1 was shown to cause hypopigmentation in mice; however, the role of Ets1 in melanocyte development is unknown. Ets1 is a helix-turn-helix transcription factor that is expressed in various developing organs and tissues in the mouse embryo, including the NC. Our goal is to determine the temporal requirement and mechanism of action of Ets1 in melanocyte development. To this end, embryos from crosses between Ets1+/- and Dct-LacZ transgenic mice, in which LacZ expression is driven to melanoblasts under the control of the Dopachrome tautomerase (Dct) promoter, were harvested between embryonic days (E) 11.5–15.5 and LacZ staining was performed. Ets1-/- embryos have fewer melanoblasts compared to Ets1+/- and wild type littermates. Additionally, cell survival and proliferation assays suggest that the lack of Ets1 does not result in increased melanoblast cell death between E11.5–12.5 or in decreased melanoblast proliferation at E11.5. It is possible that Ets1 affects trunk NC cells prior to melanocytic fate specification at E10.5. To determine if this is the case, E9.5 embryos from crosses between Ets1+/- and Sox10+/LacZ mice, in which the LacZ gene was inserted in the Sox10 locus, are currently being harvested and the number and position of Sox10+ cells are being examined via LacZ staining. To further examine a putative relationship between Ets1 and Sox10, Ets1+/- mice were crossed to Sox10+/LacZ mice and the hypopigmentation phenotypes of the double heterozygous progeny were compared to that of the single heterozygotes. The incidence of ventral hypopigmentation in Ets1+/-::Sox10+/LacZ (n = 17) mice was significantly higher than that of Ets1+/- (n = 19) and Sox10+/LacZ (n = 17) mice (P = 0.001). Additionally, the area of hypopigmentation of Ets1+/-::Sox10+/LacZ mice was significantly greater than the sum of the areas of hypopigmentation of Ets1+/- and Sox10+/LacZ mice (P = 0.006). Together, our results suggest that Ets1 is required for melanocyte development on or before E11.5 but does not appear to regulate melanoblast survival or proliferation at this age. Our results also indicate that Ets1 interacts synergistically with Sox10 during melanocyte development.

C2

Live imaging of melanosome transfer in the developing skin

Y. Takahashi, H. Murai, K.-I. Zakai, R. Tadokoro
Department of Biological Sciences, Nara Institute of Science and Technology, Ikoma, Japan

Skin pigmentation in mammals and birds is achieved by a series of processes including melanin synthesis and transport within the melanocyte, a melanosome release from melanocytes, and melanosome uptake by neighboring keratinocytes. Although several distinct (sometime controversial) models for the release and uptake of melanosomes have been proposed, no direct evidence has been available. We have recently succeeded in direct visualization of melanosome transfer by high-resolution live imaging analyses using chicken embryonic skin cultured ex vivo. Developing melanocytes are stably labeled with Tol2-EGFP by electroporating cDNA into their precursors, neural crest cells (E2). A skin tissue (E9–E12) is subsequently peeled off from the embryo, and subjected to time-lapse live imaging analyses using confocal microscopy. Early differentiating melanocytes actively extend and retract numerous dendrites. Subsequently, blebs appear on the plasma membrane of dendrites. Remarkably, these blebs become detached from a melanocyte as melanosome-containing vesicles (0.5–1 μm in diameter), which are ultimately incorporated into adjacent keratinocytes. This is the first demonstration of the direct visualization of melanin transfer

 © 2011 The Authors. Journal Compilation © 2011 John Wiley & Sons A/S

in the live skin, revealing that melanin granules are transferred via membrane vesicles in the skin of chicken embryo. We will also discuss possible roles of dendrite elongation and membrane blebbing of melanocytes in the release of membrane vesicles, and how these events are triggered by intercellular signaling between melanocytes and keratinocytes.

C3

Does overexpression of the Strawberry Notch homolog 2 gene in Dopachrome tautomerase expressing cells trigger a defect in melanoblast specification?

E. Reyes-Gomez, N. da Silva, S. Gadin-Czerw, J.-J. Panthier, G. Aubin-Houzelstein
INRA-ENVA UMR955 Genetique fonctionnelle et medicale, Maisons-Alfort, France

Strawberry Notch homolog 2 (Sbno2) is a mouse homolog of the sno gene, a regulator of the Notch pathway in Drosophila. Sbno2 was identified as a candidate gene for the patchwork (pwk) coat colour mutation. Mapped in the patchwork critical interval, Sbno2 was found overexpressed in the skin of patchwork embryos just before patchwork phenotype was first apparent. To test whether Sbno2 overexpression is sufficient for mimicking the patchwork phenotype, we produced mice overexpressing Sbno2 using the Dct promoter [Tg (Dct::Sbno2) mice]. At birth, the transgenics have a white tail tip, belly spot and feet suggesting an alteration in melanocyte lineage development during embryogenesis. We attempted to determine the origin of the phenotype observed in newborn Tg (Dct::Sbno2) mice. We studied the development of the melanocyte lineage in Tg (Dct::Sbno2) embryos between E10.5 and E18.5 using the Tg (Dct::lacZ) reporter gene, in toto X-Gal staining and immunohistochemistry. The density of melanoblasts was drastically reduced in Tg (Dct::Sbno2) embryos from E10.5 to E12.5. It increased gradually in the subsequent stages to become subnormal at E18.5. In addition, the Tg (Dct::lacZ) reporter gene was strongly expressed in several regions of Tg (Dct::Sbno2) embryos including the dorsal root ganglia (DRG) and spinal nerves all along the rostro-caudal axis from E10.5 onwards, cranial ganglia V and VII to XII from E10.5 to E12.5 and the cutaneous nerves from E12.5 to E18.5. Whether the number of nerves is increased in the transgenics is under investigation. Interestingly, the transgenics overexpressed Foxd3 in DRG and to a lesser extent, in the skin. The transcription factor FOXD3 is a marker of neural crest cells and Schwann cell precursors and is required for the maintenance of neural crest progenitors. FOXD3 controls the lineage choice between neural/glial and pigment cells by repressing Mitf in neural crest cells. Our current hypothesis is that, in Tg (Dct::Sbno2) embryos, overexpression of Sbno2 in DCT-positive cells may upregulate FOXD3 that in turn, would downregulate Mitf and suppress the melanoblast fate. The FOXD3-positive, MITF-negative cells would adopt a neural/glial fate while keeping on the Tg (Dct::lacZ) reporter gene. Our results suggest that in the mouse embryo overexpression of Sbno2 in DCT-positive cells leads to a cell-fate transition from the melanoblast to the neural/glial fate.

C4

Transcription factor activator protein 2 directly activates Sox10 to induce melanoblasts and co-operates with MITF to promote melanocyte differentiation

E. Van Otterloo, G. Lai, R. Weigel, R. Cornell
University of Iowa, Iowa City, IA, USA

The genetic regulatory network governing expression of melanin synthesis enzymes is central to the sun-tanning response. It is also relevant to the pathogenesis of vitiligo, because Tyrosinase is an auto-antigen, and manipulation of this network has been proposed as a means to combat chemoresistance of melanoma. However this network remains incompletely understood. Microphthalmia associated transcription factor (MITF) is a master regulator of transcription of genes encoding melanin synthesis enzymes but it does not work alone: we found that inhibition of two homologous transcription factors, Tfap2a and Tfap2e, strongly reduced melanization in zebrafish melanophores without affecting expression of MITF. To identify the direct transcriptional targets of Tfap2 that effect its roles in melanocyte development. We developed an assay for genes directly regulated by Tfap2 by depleting embryos of Tfap2a and Tfap2c, which eliminates the neural crest, adding cycloheximide to block protein translation, and then stimulating activity of an inducible variant of Tfap2. Genes induced in this paradigm are directly stimulated by Tfap2a. We also conducted anti-TFAP2 chromatin immunoprecipitation experiments in zebrafish embryos, and anti TFAP2A ChIP SEQ in human melanocytes, and anti TFAP2C ChIP-SEQ in a melanoma cell lines. We found evidence that Sox10 is directly regulated by TFAP2. In addition we found that forcing expression of Sox10 in embryos depleted of Tfap2a and Tfap2c, which otherwise utterly lack melanoblasts, results in the appearance of melanized cells. This implies Sox10 is a key effector of Tfap2 in early stages of melanocyte development. We will report the findings of our TFAP2 ChIP-SEQ analyses. These findings, which are still preliminary, support the view that Tfap2 operates in parallel with MITF to regulate expression of key enzymes involved in melanocyte differentiation. They imply that drugs that modulate Tfap2 activity may be effective tools to manipulate expression of melanin synthesis enzymes.

CS2: Chemistry and biophysics of melanins

C5

Discovery of isoquinoline-containing dimers as the fundamental building blocks of human red hair pheomelanin

L. Panzella, G. Greco, L. Verotta, M. d'Ischia, A. Napolitano
University of Naples Federico II, Naples, Italy

Persistence of major gaps in our knowledge of human pheomelanin structure has hindered a detailed understanding of the mechanisms of UV-induced sunburn and skin cancer in red haired individuals. The development of meaningful structural descriptions of this class of photoactive pigments remains therefore an important goal. The pheomelanin precursor 5-*S*-cysteinyldopa was oxidized under biomimetic conditions in the presence of zinc ions. The main product was isolated and characterized by spectroscopic analysis as a benzothiazolylthiazinodihydroisoquinoline (BT-TIQ). This compound afforded on chemical degradation a thiazolylpyridinecarboxylic acid (TPCA), which was formed also in high yields by chemical degradation of red hair pheomelanin. TPCA quantitation against two standard pheomelanin markers (BTCA and AHP) indicated a remarkably high proportion of the BT-TIQ-related units in the red hair pigment. Moreover, BT-TIQ revealed a distinct acid-dependent absorption band around 400 nm, resembling a characteristic feature of the red hair pheomelanin chromophore that could not be attributed to any of the previously known benzothiazine and benzothiazole species. The disclosure of isoquinoline subunits fill a most important gap in the current knowledge of the structure and origin of human red hair pheomelanin. BT-TIQ reproduces the main chemical properties and spectral features of human pheomelanin like no other low molecular weight intermediate,

© 2011 The Authors. Journal Compilation © 2011 John Wiley & Sons A/S

including the trichochromes, and may therefore serve as a 'minimal pheomelanin model' in future studies.

C6

Probing the melanosome surface using molecular rulers

K. Glass, R. Rengifo, J. D. Simon
Duke University, Durham, NC, USA

Little is known about the molecular organization of the melanosome surface. There are carboxylic acids present on the melanonsome surface, but the functional importance of these moieties – how they participate in binding of metal ions and transport into the melanosome interior – remains elusive. We have developed an approach for studying the distance between neighboring carboxylic acid groups, where we take advantage of the heat released upon ion pairing between carboxylate and ammonium groups. Specifically, the binding of diamines, which have expanding linear aliphatic carbon chain lengths separating the charged amine groups, are used as molecular rulers to gauge the distance between the carboxylic acid groups on the surface of sepia and choroid melanosomes using isothermal titration calorimetry (ITC). At pH 5.8, the carboxyl groups on the surface of the melanosome are deprotonated, and both amine groups on the diamines are protonated. ITC measurement of the equilibrium constants and capacity for binding as a function of the distance between the two amine groups provides a unique and novel insight into the distances between neighboring carboxylic acid sites on the melanosome surface. In the case of Sepia, the binding of +H3N-(CH2)m-NH3+ (m = 1–4) exhibit binding constants of 1.89×10^3, 1.14×10^4, 1.71×10^3, and 7.79×10^2. The lowest energy conformation occurs when the two nitrogens of the diamines are 22.7, 37.8, 44.0, and 58.8 nm apart. These results suggest a narrow distribution of distances between nearest neighboring carboxylic acid groups, on the order of ~38 nm.

C7

Is melanin a semiconductor: the mysteries of electrical conduction and melanin bioelectronics?

P. Meredith, B. Mostert, I. R. Gentle, G. Hanson, K. Tandy, E. Namdas, F. Pratt, B. J. Powell
School of Mathematics and Physics, University of Queensland, Brisbane, Queensland, Australia

Melanins have been known to conduct and photo-conduct electricity for more than four decades [1]. Renewed interest in melanins as advanced functional materials has emerged more recently, particularly in the context of a biological electrical interface material [2]. Since the early 70's the standard model for melanin in the solid-state has been as an amorphous semiconductor as per the Mott-Davis formalism [3]. This assertion was derived primarily from observations of electrical switching between high and low resistive states. Indeed, it has been argued that melanin constituted the first demonstrated electrically active organic device and organic semiconductor [4]. However, it is by no means clear that this is the appropriate or correct description and multiple observations of more exotic phenomena such as apparent ambipolar behavior and humidity dependent electrical conductivity have very much cast doubt on amorphous semiconductivity being the necessary and sufficient model [5]. In our paper we will describe recent work focused on unraveling this difficult and seemingly intractable problem. We have used a combination of techniques including muon-spin relaxation (μSR), electron paramagnetic resonance and conductivity measurements and find that melanin has characteristics of a hybrid ion (proton)-electron conductor. Its electronic biophysics is dominated by ionic behavior and we show that this originates from the so-called comproportionation equilibrium whereby protons are released in a hydroquinone-to-quinone reaction. We also demonstrate how this exotic behavior can be used in an all-solid-state organic electrochemical transistor to affect ion-to-electron transduction – a key element in bioelectronic interfacing. A full understanding of melanin's electrical properties will not only allow its potential as a bioelectronic material to be realized but could have major implications for advancing our knowledge of its biological role and function

References:

[1] Meredith et al., Soft Matter, 2006, 2, 37–44.
[2] Bothma et al., Adv. Mater., 2008, 20, 3539–3542.
[3] McGinnes et al., Science, 1974, 183, 853–855.
[4] The device is now housed in the Smithsonian Institute Chip Collection (http://smithsonianchips.si.edu/proctor/index.htm).
[5] Meredith and Sarna, PCR, 2006, 19(6), 572–594.

C8

Rare melanoma cell detection by thermal emission imaging

A. Brocas, V. Shynkar, M. Mahet, E. Tham, Y. Abidine, P. Guitera, F. Amblard
Institut Curie, Paris, France

The outstanding ability of melanin, as a solid state structure, to convert absorbed light into heat, is the very basis of its photoprotective activity. One consequence is that melanin detection by light excitation can hardly be based on its very low florescence, but rather on absorption or reflexion contrast. We proposed here to use a well know phenomena as a source of imaging contrast: thermal radiation. We demonstrated quantitatively that melanosome can be heated non-destructively up to temperatures that are high enough to make melanosomes and pigmented cells detectable by appropriate imaging means, either in the visible or the near infrared range. The strong linearity of the thermal response ensures high signal to noise ratios, and permit the selective and sensitive detection of rare pigmented cells in tissues and blood samples. We will describe the basic physics of this novel mode of imaging, and show results obtained for the detection of circulating melanoma cells.

CS3: Difficult to classify hyperpigmentations, clinically-oriented

C9

Diagnostic utility of dermatoscopy in hydroquinone induced exogenous ochronosis

R. Dhurat, S. Mishra, C. Nayak, D. Deshpande
Department of Dermatology, Lokmanya Tilak Municipal Medical College and General Hospital, Sion, Mumbai, India

Hydroquinone is the preferred topical bleaching agent used in the treatment of melasma. The adverse effects of its chronic use are confetti-like depigmentation and exogenous ochronosis. Exogenous ochronosis manifests clinically with gray-brown or blue-black hyperpigmentation, as well as pinpoint hyperchromic caviar like papules over the malar region. Dermatoscopic findings of ochronosis are unique and point towards a clue for its diagnosis. We report three cases of hydroquinone-induced exogenous ochronosis while treating melasma. Dermatoscopy performed in patients on the areas of caviar like hyperpigmentation revealed accentuation of the normal pseudo-rete of the facial skin with amorphous densely pigmented structures obliterating some follicular opening and multiple thin, short arciform structures. On histopathological examination, curved ochre-colored struc-

 © 2011 The Authors. Journal Compilation © 2011 John Wiley & Sons A/S

tures, 'banana-shaped' fibers, were seen in the dermis of all patients. Exogenous ochronosis is an avoidable dermatosis and is difficult to treat. Dermatologists should be able to differentiate it from melasma and immediately discontinue hydroquinone. Exogenous ochronosis can be easily diagnosed on dermatoscopy which obviates the need of an invasive procedure of cutaneous biopsy.

C10

A study on clinico-histological evaluation of 155 cases of periorbital melanosis

N. Sarma
Howrah, India

Pigmentation around eyes is known as periorbital pigmentation (POM). This condition is common world-wide but is particularly common in India and many Asian countries. Despite various hypotheses, this condition has remained idiopathic and even its mode of transmission is inconclusive. The present study is directed towards evaluation of detail clinical parameters like onset, transmission pattern, shape, orientation and distribution, factors associated with aggravation and remission and histological alterations. Adult patients with POM were randomly included for the study. Demographic profile, onset and progression, any associated illness was recorded. Examination of local skin, other body surfaces as well as relevant systemic organs were undertaken. Pigmentary alterations of other form were searched. However all forms of post traumatic and post inflammatory pigmentation were excluded. Punch biopsy (3 mm) and histological examination was done from both pigmented and control site. Total 155 consecutive adult patients with well formed POM were selected. Age ranged from 27 to 45 yr, females were predominant (F – 96, M – 59). Family history was noted in 34%. Melasma (n = 89), macular amyloidosis (n = 76) and acanthosis nigricans (n = 21) were commonly associated. Comparative histological findings between pigmented and normal facial skin were noted. In addition to examine the clinical details, this study was particularly focused to evaluate the histological alterations. The findings will surely have great impact on understanding the pathogenesis and will help in searching the correct mode of therapy.

C11

Epidemiological, clinical and histopathological profile and patch test results in patients of primary localized cutaneous amyloidosis (PLCA)

S. Tambe, H. Jeranjani, S. Ghate
Lokmany Tilak Municipal Medical College & General Hospital, Sion, Mumbai, India

We carried this project to (i) study epidemiological and clinical profile of PLCA; (ii) to compare findings of histopathology, Thioflavin-T & DIF; and (iii) to evaluate the photopatch test results in these patients. Patients of clinical diagnosis of PLCA were included in the study. History and clinical examination was followed by histopathological evaluation of skin biopsies and then patch testing with antigens of universal and cosmetic series. Amyloid deposits along with features like dilated dermal papillae, pigmentary incontinence and increased melanin in basal layers for H&E, Yellowish green deposits of amyloid in papillary dermis for Thioflavin-T & fluorescent deposits in papillary dermis for DIF were considered diagnostic of PLCA. Mean age of presentation was 38.76 yr with female preponderance. Diabetes was seen in 20% of patients. History of Itching and friction was present in 78.46 and 76.92% of patients respectively. Macular type (84.61%) was present on the back and arms. Lichen (50%) on the legs and biphasic (6%) at combined sites. Amyloid was demonstrated by H&E in (63.07%), Thioflavin-T in (64.61%) and DIF in (44.61%) of cases with best results on the legs. Patch test positivity was found in six patients. PLCA is a common pigmentary dermatosis in Indian population with friction and itching as inciting factors. H&E stain was found equally effective in demonstration of amyloid compared to newer stains.

C12

How to differenciate melasma from facial postinflammatory hyperpigmentation (PIH)?

L. Benzekri
Department of Dermatology, Ibn Sina University Hospital, Mohammed V Souissi University Rabat, Morocco

The most common facial pigmentations are melasma and PIH. Melasma has a predilection for sun exposed area. In PIH the patches are characteristically limited to the site of the preceeding inflammation and have indistinct, feathered borders. According invasive techniques distinguished features have been described. In melasma there is an epidermal melanin deposition (non aggregated melanosomes) in all the layers including horny layer associated in some cases with dermal melanin deposition. In PIH the basement membrane is frequently disrupted and melanin falls into the dermis and resides within melanophages. To develop a non invasive technique for the detection of melanin in the horny layer useful to differenciate melasma from PIH. Based on the detection of melanin in the horny layer. The adhesive coated surface of one slide is briefly pressed to the pigmentation with a rocking motion. If applied directly to human skin, the slide provides a specimen of a monolayer of thousands of cells. Staining of the melanin granules is achieved with Masson's ammoniacal silver nitrate. Under oil immersion the number of melanin granules can be easily counted. In some horny cells large clusters of granules were noticed particularly in the center of the cells. In melasma, many isolated or aggregated melanin granules are observed in all the corneocytes whereas in PIH very rare granules are found in some corneocytes. It is very important to differenciate melasma from PIH because they have not the same prognosis and their management is quite different.

CS4: Mouse models: pigment cell biology & melanoma

C13

Mouse models for Raf signaling in melanocyte and melanoma development

A. Eychène
INSERM U1021 – CNRS-UMR3347, Institut Curie, Orsay, France

The RAS/RAF/MEK/ERK pathway plays a key role in melanoma, with BRAF and NRAS mutations in about 50 and 15% of cases, respectively. While the discovery of compounds specifically targeting mutated V600EBRAF raises new hopes, such compounds cannot be used for the treatment of half of melanoma patients, including those mutated on NRAS. The contribution of BRAF and its closely related kinase CRAF downstream of RAS during tumoral progression appears complex. We developed mouse models in which single or compound ablation of BRAF and CRAF is achieved upon conditional Cre expression in the melanocyte lineage. These models allow us investigating the role of both kinases during normal development of the melanocyte lineage. In addition we are currently using these models to assess the role of RAF kinases in NRAS-induced melanoma at each step of tumoral progression, from initiation (benign nevi formation) to metastasis.

© 2011 The Authors. Journal Compilation © 2011 John Wiley & Sons A/S

C14

Periostin promotes tumor growth and progression in cutaneous malignant melanoma

Y. Kotobuki, A. Tanemura, S. Serada, M. Fujimoto, T. Naka, I. Katayama
Department of Dermatology, Osaka University, Suita City, Osaka, Japan

The detection of proteins responsible for tumor development and regulation is of great value. In this study, we aimed to identify novel functional proteins associated with tumor progression in malignant melanoma. The proteins extracted from malignant melanoma and normal skin tissue were labelled with iTRAQ reagent and processed for a mass spectrometry using nano LC-MS/MS system. The detected protein expression and localization in cutaneous malignant melanoma tissue was confirmed by immunohistochemistry. Human melanoma cell line (Mewo) was injected subcutaneously into Rag2-/- mice and POSTN-/-Rag2-/- mice, thereafter implanted tumor growth was compared. Among 1314 identified proteins, 118 proteins were increased more than 5.0-fold and 37 proteins were decreased <0.5-fold in malignant melanoma compared to normal tissue. In these proteins, we found significant upregulation of an extracellular matrix protein, periostin (POSTN). POSTN expression was robustly induced in fibroblast cultured with Mewo and evident on the stroma of advanced melanoma tissue, suggesting the importance of interaction of these cells. As an assessment of functional implication in POSTN in malignant melanoma, we observed significant increase in melanoma cell number in the treatment with recombinant POSTN. Moreover, tumor growth in POSTN-/-Rag2-/- mice was significantly suppressed compared with that in Rag2-/- mice. We newly identified upregulation of POSTN expression in malignant melanoma tissue. POSTN was induced in the co-culture of fibroblast with melanoma cell and showed the growth promoting effect. These results indicate that periostin might be a potential therapeutic target in malignant melanoma.

C15

An induced suppressor mutation at the microphthalmia locus in the mouse reveals novel insights into bHLHZip transcription factor function

E. Steingrimsson, H. Arnheiter, K. Bergsteinsdottir, K. Bismuth, B. K. Gisladottir, A. G. Hansdottir, L. Crawford, H. Murakami, M. Gasper, J. Debbache, A. Parekh, S. Skuntz, D. A. Swing, J. H. Hallsson, D. E. Fisher, N. G. Copeland, N. A. Jenkins
Department of Biochemistry and Molecular Biology, Faculty of Medicine, University of Iceland, Reykjavik, Iceland

Suppressor mutations which compensate other mutations have revealed novel insights into gene function, gene interactions and genetic pathways in bacteria, in baker's yeast and in multicellular organisms such as Drosophila and C. elegans. Although few suppressor screens have been performed in the mouse, spontaneous suppressor mutations, such as dilute-suppressor (dsu), suggest that such screens can lead to important biological insights. Here we report the isolation of an intragenic mutation in Mitfmi-sp which largely corrects the phenotypic enhancement associated with the original Mitfmi-sp allele, including enhancement of the Mitfmi-vga9 loss-of-function mutation. This novel mutation (termed spotless or Mitfmi-sl) introduces a stop codon at amino acid 316 of MITF, leading to premature termination. As it was induced on the Mitfmi-sp chromosome it also lacks the alternative 6 amino acids due this mutation. We have compared the genetic, biochemical and developmental behaviour of Mitfmi-sl to that of Mitfmi-sp. Developmental studies show that the onset of pigmentation is delayed in Mitfmi-sl homozygotes whereas no differences were seen in effects on proliferation. We found no differences in DNA binding ability or protein stability. However, competition assays suggest that the suppressor mutation affects dimerization of the protein and reporter assays show promoter-specific differences. Our data indicate that the carboxyl-end of MITF is not essential for normal function in melanocytes and appears to have negative function in the wild type context. We conclude that searching for suppressor mutations in the mouse is feasible and provides a powerful tool for dissecting gene function.

C16

Ultraviolet B, but not ultraviolet A initiates and promotes melanoma formation in metabotropic glutamate receptor 1 transgenic mouse

Y. Funasaka, S. Oyama, S. Okazaki, S. Kawana, C. Nishigori
Department of Dermatology Nippon Medical School, Tokyo, Japan

Metabotropic glutamate receptor subtype 1 (mGluR1) is a G protein coupled receptor activated by glutamate, and is functionally expressed in the central nervous system. We have previously shown that ectopic expression of mGluR1 in melanocytes is essential for both development and in vivo growth of melanoma in double transgenic mice, NSE-tTA/TRE-mGluR1. Using these transgenic mice, we irradiated once at day 3 and three times a week for 2 months from day 30 by ultraviolet B (UVB) at 1.5 J/cm^2 or UVA at 14 J/cm^2. Single irradiation by UVB at day 3 induced melanoma formation earlier than non-irradiated group, and repetitive irradiation for 2 months promoted melanoma growth. In case of UVA irradiation, this induction or promotion of melanoma formation was not observed. To see whether melanin species and contents affect UV-induced melanoma formation, we created yellow mice which dominantly contain pheomelanin by crossing transgenic mice with recessive yellow mice. In yellow mice, UVB but not UVA induced and promoted melanoma formation. Initiation of melanoma occurrence was earlier in yellow mice compared with that in black mice, however melanoma promotion rate was not significantly changed between yellow and black mice. These results indicate that initiation and promotion of melanoma can be induced by UVB, but not UVA irradiation and pheomelanin might be involved in initiation of melanoma formation.

CS5: Chemistry of melanins: standardization workshop roundtable

C17

Methods in melanin research

M. d'Ischia, J.-C. Garcia-Borron Martinez, S. Ito, J. D. Simon
University of Naples, Department Organic Chemistry, Naples, Italy

Despite intense research efforts, melanins can be still regarded as the most enigmatic pigments/biopolymers found in Nature. This state of affairs is due to the adverse physicochemical properties; the instability to acids, alkali, oxidizing agents and UV radiation; the lack of standardized materials and protocols to be used for investigative purposes; reliance on assumptions and speculations that have never been proven on experimental grounds; incomplete biochemical characterization of the melanogenic pathway(s). It is thus difficult to compare data on the same pigment or melanogenic protein obtained in different laboratories, and it is unclear whether the range of synthetic systems studied are appropriate to investigate the structure and function of natural pigments or if various mammalian and

© 2011 The Authors. Journal Compilation © 2011 John Wiley & Sons A/S

invertebrate melanins are accurate models for the human pigment. Furthermore, there is a lack of consensus on melanin 'structure', and several unwarranted models are still found in scientific literature. This situation has engendered much disagreement in the pigment cell community and contributed to some extent to slow down progress in the various fields of melanin research. The objective of this Concurrent Session is to discuss and eventually propose a consensus document addressing crucial issues in melanin research (i) Definition, classification and terminology (e.g. should all black insoluble pigments of plant origin be classified as melanins?) (ii) Recommended protocols for preparation, isolation, purification, spectral and chemical analysis of both natural and synthetic melanins. (iii) Recommended protocols for utilization of enzymes for studies of melanin properties in cell cultures and other in vitro systems. (iv) Preparation and/or assessment of standard compounds for melanin research, including commercially available pigments and enzymes. These objectives will be pursued through: (i) survey of literature and definition of criteria that should guide classification and terminology; (ii) critical discussion of current methods and identification of best protocols, protocols to avoid, effects of experimental approaches on melanin structure and properties, best criteria for determination of purity, selection of 'best practices' for sample preparation and spectral analysis, scope and limitations of chemical degradation methods for analysis and quantitation. Realization of these goals should enable the creation of a set of recommended procedures for investigative.

C18

Evaluation of alkaline hydrogen peroxide oxidation to analyze eumelanin and pheomelanin

S. Ito, Y. Nakanishi, K. Wakamatsu
Fujita Health University School of Health Sciences, Toyoake, Aichi, Japan

Natural melanins are mixtures of eumelanin and pheomelanin. The color of hair, skin, and eyes is mainly determined by the quantity and ratio of eumelanin and pheomelanin produced in melanocytes. Furthermore, it is generally accepted that eumelanin is photoprotective, while pheomelanin is phototoxic to tissues. Eumelanin and pheomelanin in tissue samples can be specifically measured as the markers pyrrole-2,3,5-tricarboxylic acid (PTCA) and 4-amino-3-hydroxyphenylalanine (4-AHP) after acidic permanganate oxidation and reductive hydrolysis with hydroiodic acid, respectively. However, those degradative methods are not easily performed in most laboratories that do not have significant chemical expertise. We evaluated alkaline H_2O_2 oxidation in 1 M K_2CO_3 that produces, in addition to PTCA, the marker for 5,6-dihydroxyindole-2-carboxylic acid-derived eumelanin, pyrrole-2,3-dicarboxylic acid (PDCA) as a marker for 5,6-dihydroxyindole (DHI)-derived eumelanin, and thiazole-2,4,5-tricarboxylic acid (TTCA) and thiazole-4,5-dicarboxylic acid (TDCA) as markers for pheomelanin. Those four degradation products can be easily separated by HPLC and analyzed with ultraviolet detection. The alkaline H_2O_2 oxidation method is simple, reproducible and applicable to various pigmented samples such as synthetic melanins, hair, melanocytes, and skin. The ratios of TTCA/PTCA, PDCA/PTCA, and TTCA/4-AHP can be used to estimate relative contents of pheomelanin in melanin, DHI units in eumelanin, and benzothiazole units in pheomelanin, respectively. The measurement of PTCA, TTCA, PDCA, and TDCA in a single chromatographic analysis provides valuable information for characterizing mixed melanogenesis. Its application to characterize melanin in human hair shows that PTCA and TTCA serve as specific markers for eumelanin and pheomelanin, respectively, although some caution is needed regarding the artificial production of TTCA from eumelanic tissue proteins.

CS6: Human skin color and its evolution

C19

Cellular and molecular mechanisms underlying the biogenesis of melanosomes

C. Delevoye, G. Van Niel, S. Simoes, I. Hurbain, M. Romao, D. Tenza, M. Marks, G Raposo
CNRS-UMR144, Institut Curie Paris, France

Studies on the melanosome, the lysosome-related organelle of pigment cells has provided insights into mammalian endosomal membrane dynamics by revealing how specific trafficking events are exploited to generate these tissue-specific organelles. Our recent studies have started to unravel how the endosomal system specializes to generate first unpigmented fibrillar melanosomes and secondly pigmented, mature melanosomes that can be transfered to keratinocytes. During early melanogenesis, sorting of the protein Pmel17 to intraluminal vesicles of multivesicular bodies precursors of melanosomes is concomitant with its cleavage and consequent formation of Pmel17-driven amyloid-like fibrils. Sorting of Pmel17 is independent of ubiquitylation and of the ESCRT (endosomal sorting complex required for transport) machinery. Our recent studies highlight a role for tetraspanins in endosomal sorting of the PMEL luminal domain and consequently on the generation of amyloid-like fibrillar sheets in vitro and in vivo. Still ESCRT complexes operate for disposal of the PMEL C terminal fragment revealing a tight interplay within the endosomal membrane. Late melanogenesis requires the transfer of melanogenic enzymes from early endosomes products mutated in different forms of albinism (such as the Hermansky Pudlak syndrome) encode proteins that regulate late melanogenesis (AP-3, BLOC complexes). Our recent studies have brought further knowledge on how these novel trafficking regulators operate in concert with additional adaptors, cytoskeletal motors and Rab GTPases to specialize endosomal sorting, endosome localization and positioning facilitating endosome-melanosome crosstalks required for the biogenesis of functional organelles. Our current studies aim to shed light on how the specialized trafficking events can be regulated within the integrated epidermal-melanin unit upon establishment of the pigmentation synapse.

C20

Human hair pigmentation characteristics revealed by melanin determination in human eumelanic hairs of various ethnic origins

S. Commo, K. Wakamatsu, B. A. Bernard, S. Ito
L'Oréal Recherche et Innovation, Clichy, France

Studies carried out in human hair follicle revealed intriguing findings with regard to melanogenic enzyme expression in relation to eumelanin and pheomelanin synthesis, and pointed out unexpected hair-type particularities. Changes with age in human hair have also been revealed. More specifically, the loss of hair pigment leading to the visual greying of hair stems from a gradual and specific depletion of the pool of melanocyte progenitors hosted in each hair follicle. From a chemical point of view, however, data remains scarce regarding human hair pigmentation characteristics. Systematic melanin determination in human hair should give insights for understanding molecular controls of melanin synthesis and melanocyte phenotype within human hair bulbs. In an attempt to bring clues to this objective, we examined the quantity and quality of melanin in eumelanic hair in humans in relation to gender, to ancestry origins, and to

© 2011 The Authors. Journal Compilation © 2011 John Wiley & Sons A/S

age, in 168 individuals among African-American, East Asian, and Caucasian. We evidenced differences in melanin composition and content in relation to ethnic origins. Furthermore, our results gave evidence of an age-dependent increase in the pyrrole-2,3-dicarboxylic acid (PDCA)/pyrrole-2,3,5-tricarboxylic acid (PTCA) ratio in African-American and Caucasian hairs, suggesting a chronological evolution of hair follicle melanocyte phenotype (e.g. decrease in dopachrome tautomerase expression with age). In conclusion, we show, for the first time, clear evidence of age-dependent changes in the quantity and quality of eumelanin in pigmented human hairs. Furthermore, our results reveal differences in melanin composition in relation to ethnic origins in eumelanic human hairs.

C21

Phenotypic, genetic and lifestyle risk factors for solar lentigines in adult Caucasian women

E. Mauger, K. Ezzedine, R. Jdid, J. Latreille, D. Malvy, F. Gruber, P. Galan, S. Hercberg, E. Tschachler, C. Guinot
CERIES, Neuilly sur Seine, France

Very scarce epidemiological studies have been conducted in Caucasian on the relationships between solar lentigines, and behavioural and constitutional host factors. Therefore, a cross-sectional study was conducted on a sub-sample of 443 French middle-aged Caucasian women from the SU.VI.MAX cohort. The severity of solar lentigines (SL) was assessed on forehead and cheeks by a dermatologist using a specific 6-grade scale with photographic illustrations. To be used as an outcome variable a score of SL was calculated, and its distribution was dichotomized according to the median value: low SL score versus high SL score. In addition, the women have been assigned into a 6-type sun behaviour typology. Possible risk factors for SL were examined using logistic regression models. The risk of SL was found significantly linked with age [Adjusted Odd Ratio (95% Confidence Interval): 1.12 (1.08–1.17)], history of facial freckles [3.95 (2.46–6.34)], dark skin colour [2.12 (1.18–3.80)], dark and very dark suntan intensity [1.99 (1.21–3.29)], the two most risky behaviours regarding sun exposure [2.11 (1.12–3.99), 3.21 (1.46–7.08)], and current intake of combined oral contraceptives or progestogen treatments [3.68 (1.49–9.07)]. Moreover, a significant relationship was found with the presence of R160W variant of MC1R gene [2.58 (1.00–6.66)]. This study highlights complex links between genotypic background and its phenotypic expression, sun exposure behaviour and sun protection habits. Besides, our results suggest that the 6-type sun behaviour typology could be easily used to identify and target risk groups for intervention or epidemiological studies, and/or information campaigns of public health.

C22

Detection of new factors involved in melanogenesis

T. Motokawa, M. Ito, K. Tashiro, K. Yokoyama
POLA Chemical Industries, inc., Yokohama, Japan

Paracrine factors such as alpha-melanocyte-stimulating hormone (MSH) and endothelin have been demonstrated to play pivotal roles in human skin pigmentation. The discovery of new factors can have impacts on wide areas of pigmentation studies, given the potential contributions to not only the development of basic research, but also etiological research, since the factor itself or its receptor may show associations with pigment disorders or characteristic phenotypes. The objective of this study was to detect new factors involved in melanogenesis using comprehensive analysis. A co-culture system was constructed, in which melanocytes are stimulated to produce melanin under ultraviolet (UV) irradiation. DNA microarray studies were performed to detect up-regulated genes after UV irradiation. We then validated the significance of the detected genes on UV-induced melanogenesis using a small interfering RNA study. We also directly examined the importance of candidate genes by testing whether recombinant peptides can induce melanogenesis. Examination of over 40 000 candidate genes using microarray techniques demonstrated the possibility of the existence of new factors. We are currently verifying the functions of these factors in terms of melanogenesis. In this presentation, we present the latest results of our research.

C23

Vitiligo color measurements for formulating the base color pigment quantities for human integument phantoms and replicating human integument with prosthetic materials

P. Tanner, S. Leachman
University of Utah, Huntsman Cancer Institute, Salt Lak City, UT, USA

The purpose of this study was to test the hypothesis that regardless of skin color type, amelanocytic human integument is indistinguishable with respect to remitted electromagnetic radiation. Thirty-two patients seen at the University of Utah for medical treatment of leukoderma (vitiligo) lesions from 2004 to 2008 consented to participate in our study. Twenty-seven people participated in a patient history and demographic questionnaire and 28 people voluntarily participated in integument color measurements using Ocean Optics USB-2000 photospectrometer. Only 23 people participated in both measurements and the survey. A minimal data set was obtained for 19 of these study participants. Polydimethylsiloxane (PDMS) samples were fabricated in an assay-like grid using SiliClone Studio's Human Coloration System colorants, creating a range of non-pigmented to heavily pigmented samples in addition to a range of red and blue flocking concentrations. Upon cure, 55 samples were measured with the Ocean Optics USB-2000 photospectrometer. The spectral curves of patients with leukoderma were analyzed with the spectral curves of the silicone samples and a range of pigment concentrations dispersed in PDMS was determined. Contrary to our hypothesis, turbidity of human integument varies among phototypes. A larger data set will validate this conclusion. When assessing overall skin color, a colorimeter must be used to verify color acuity. Afterwards, a photospectrometer can be used to measure the turbidity of the PDMS.

CS7: Non cutaneous melanocytes

C24

Gene duplication linked to Fm locus is closely correlated to hyperpigmentation of internal organs in Silky chicken

A. Shinomiya, K. Kinoshita, M. Mizutani, T. Namikawa, Y. Matsuda, Y. Kayashima, T. Akiyama
Keio University Yokohama, Japan

Silky chicken displays heavy melanization in their internal organs and the trait is designated as Fibromelanosis (Fm). In our previous analysis, the segregation rate of Fm and fm (wild type) in F2 progeny between Silky (Fm) and Black Minorca or Fayoumi (fm) was closely to 3:1, showing that Fm was controlled by a single dominant gene. The purpose of this study is to map the gene responsible for Fm. We performed genetic linkage analysis using backcross progeny (BC) from crossing between Black Minorca and (Silky X Black Minorca) F1, and found one microsatellite marker linked to Fm (93%). We developed new

 © 2011 The Authors. Journal Compilation © 2011 John Wiley & Sons A/S

sequence tagged site markers around the microsatellite marker using the information of the chicken draft genome sequence, and mapped the Fm locus in the extent of 1.46 Mb on the chicken chromosome 20 by genotyping of the BC progeny (n = 87). Furthermore, we found a DNA segment, of which copy number in Silky was twice higher than that in Black Minorca, within the Fm region by quantitative real-time PCR assay, and linkage analysis of the gene duplication and Fm confirmed that the duplicated copy was also located in the Fm region. Further analyses demonstrated that the duplicated segment was the extent of approximately 130 kb and that the gene duplication was not observed in other fm lines (Fayoumi and Red jungle fowl). In the 130 kb-region, five genes were located and the expression levels of four out of the five genes in Silky were higher than those in Black Minorca. Present results demonstrate close correlation between the Fm phenotype and the gene duplication. We will discuss the mechanism of hyperpigmentation in Silky chicken.

C25

Albino and pheomelanic mice are more susceptible and present a poorer recovery after noise-induced hearing loss compared to eumelanic mice

S. Murillo-Cuesta, J. Contreras, M. Cantero, R. Cediel, R. Martínez-Vega, E. Zurita, A. Fernández, I. Varela-Nieto, L. Montoliu
CNB-CSIC, Madrid, Spain

Neural crest-derived melanocytes found in the cochlea are required for normal hearing. Albinism has been associated with hearing deficits in several species, although melanin appears to be dispensable for normal auditory function. Recently, we have analyzed the auditory brainstem responses (ABR) of pigmented tyrosinase-transgenic mice, using albino outbred mice (Murillo-Cuesta et al. 2010 Pigment Cell & Melanoma Res.). We showed that 2–4 months-old albino mice present a higher prevalence of profound sensorineural deafness and a poorer recovery of auditory thresholds after noise-exposure than pigmented mice. Similarly, phenotypically albino transgenic mice producing melanin-precursor metabolites, such as L-DOPA, behaved as pigmented mice, thus suggesting that the observed hearing loss was associated with the absence of cochlear melanin or its precursor metabolites. Our results concluded that albino outbred mice showed impaired hearing responses during ageing and after noise damage when compared to pigmented or melanin-precursor-producing transgenic mice. We have extended this analysis to inbred or congenic mouse strains, derived from C57BL/6J (Zurita et al. 2011 Transgenic Res.), and to explore the differences in hearing capacities associated with eumelanin and pheomelanin. We have analysed the basal ABR thresholds and hearing responses after noise damage using albino coisogenic (‘albino’ B6(Cg)-Tyrc-2j/J), pheomelanic congenic [dominant ‘yellow’ (Aya), B6.Cg-Ay/J] and eumelanic inbred [nonagouti ‘black’ (aa) C57BL/6J] mice. No statistically significant differences were found in basal ABR thresholds in response to click stimulus among the three considered mouse strains. Likewise, frequency audiograms and latencies of the central auditory pathway were comparable in all three cases. However, both albino and yellow strains presented statistically significantly higher threshold shifts, after noise exposure and a poorer recovery to noise-induced hearing loss, as compared to black mice. These results confirm in a coisogenic albino mouse strain those previously obtained with albino outbred mice. Furthermore, these results indicate a higher susceptibility to noise-induced hearing loss in both albino and pheomelanic mouse strains, as compared to the eumelanic mouse strain, thus suggesting a better protective role of presumptive eumelanic versus pheomelanic compounds of the mammalian cochlea after noise exposure.

C26

MITF regulates growth factor expression and cell migration in RPE cells

X. Ma, L. Pan, H. Li, B. Wen, J. Wang, X. Jin, X. Dai, J. Yang, Y. Chen, Z. Su, L. Hou
Developmental Cell Biology and Disease Program, Wenzhou Medical College, Wenzhou, China

MITF plays critical roles in the development of many lineages including RPE (retinal pigment epithelium) and melanocytes. Mutations in MITF are associated with Waardenburg syndrome type 2 in human that show heterochromia iridis, pigmentary disturbance and deafness. During eye development, MITF expresses at the RPE region and plays critical roles in RPE development. Mutations in Mitf such as Mitfmi-vga9 not only cause deafness and loss of pigmentation in the skin, but also exhibit microphthalmia, RPE developmental defect and retina degeneration. Although RPE dysfunctional changes in growth factors expression and abnormal migration are associated with retinal diseases, how these growth factors and cellular events are regulated in RPE cells remain largely unknown. Here we used lentiviral system to overexpress Mitf in RPE cells and performed gene expression profiling using a microarray analysis. We found that MITF regulates the expression of many growth factors in RPE cells. As a result, the upregulation of MITF impaired microtubule assembly and thus inhibits RPE cell migration. We further demonstrated that the addition of one of the upregulated growth factors into the RPE cell cultures inhibits the cell migration in a dose dependent manner. On the other hand, the specific knockdown of the gene rescued the microtubule assembly impairment and cell migration proceeds in MITF overexpressing stable cells. Taken together, the results suggest that MITF plays functional roles in regulating the expression of growth factors and cell migration in RPE.

C27

A novel pathway for regulation of pigmentation by glutamate receptor mGluR6 through its action on TRPM1

S. Devi, Y. Markandeya, N. Maddodi, K. Wakamatsu, S. Ito, R. Balijepalli, V. Setaluri
University of Wisconsin, Madison, WI, USA

Transient receptor potential, Melastatin 1 (TRPM1), a calcium channel expressed in epidermal melanocytes and retinal ON bipolar cells, has been shown to play a role, respectively, in melanin pigmentation and vision. However, TRPM1 mutations that cause congenital night blindness in humans seem to have no effect on skin pigmentation or skin photosensitivity, although TRPM1 mutations in Appaloosa horses are associated with both a coat color phenotype and night blindness. Recent studies have shown that in ON bipolar cells, activity of TRPM1 channel during activation of visual pathway is negatively coupled to glutamate and metabotropic glutamate receptor 6 (mGluR6) through Gα0. To understand the reasons for lack of skin pigmentation phenotype in individuals with TRPM1 mutations, we investigated whether mGluR6 and Gα0 are expressed in melanocytes and TRPM1 channel is functionally coupled to mGluR6. In cultured neonatal foreskin epidermal melanocytes, while mGluR6 protein is readily detected, Gα0 expression is undetectable by western blotting. Interestingly, treatment of melanocytes with glutamate receptor agonists L-glutamate or a group III specific agonist L-AP4 caused a dose dependent increase in calcium uptake, instead of a decrease, in ratiometric calcium imaging studies and

© 2011 The Authors. Journal Compilation © 2011 John Wiley & Sons A/S

an increase in inward current in whole-cell patch clamping studies. This increase is TRPM1-dependent since it was abolished by knockdown of TRPM1 by shRNA. These data indicate that TRPM1 activity is positively coupled to glutamate binding to its receptors. Treatment with glutamate or L-AP4 also caused a decrease in cell survival, increase in melanin content, a decrease in dendritic length and an increase in dendritic width. The effect of L-AP4, a mGluR6-selective agonist, was more pronounced than L-glutamate, supporting a role for mGluR6 in melanocyte biology. Knockdown of mGluR6 using shRNAs targeting both the 3′ and 5′ ends showed that the increased calcium uptake and inward current upon addition of glutamate agonists was mGluR6 dependent. These data show a functional link between glutamate receptor and TRPM1 in melanocytes and a novel regulatory pathway of calcium homeostasis involving glutamate receptors and TRPM1 implicating a role for neuronal glutamate receptor 6 in melanocyte biology and melanin pigmentation.

CS8: Update on physiology of cutaneous pigmentation

C28

The eumelanin intermediate 5,6-dihydroxyindole-2-carboxylic acid DHICA promotes differentiation and protection in epidermal cells: an additional role of melanogenesis

D. Kovacs, E. Flori, V. Maresca, M. Ottaviani, N. Aspite, L. Panzella, A. Napolitano, M. d'Ischia, M. Picardo
San Gallicano Dermatologic Institute, Rome, Italy

The melanocyte-keratinocyte interaction is regulated by a complex paracrine network of hormones and cytokines, released from all skin cell population in response to Ultraviolet Radiations (UV), which can promote several responses including differentiation and survival. In response to UV, keratinocytes and fibroblasts are known to produce several hormones and cytokines whereas only few factors are described to be released by melanocytes themselves. The objective of the present study was to analyze the possible contribution of DHICA in mediating cell protection mechanisms not only in melanocytes but also in the surrounding keratinocytes acting as a diffusible chemical messenger in the context of the paracrine interactions between epidermal cells, which regulate skin homeostasis and defence. This study was performed on primary cultures of human keratinocytes employing molecular (Real Time-RT-PCR; Western Blot); cellular/morphological (Immunofluorescence; FACS analysis) and biochemical (GC-MS; enzymatic assays by spectrophotometry) approaches. At micromolar concentrations, DHICA induced: (i) time- and dose-dependent cell growth arrest, as evidenced by the decrease of Ki67-positive nuclei, without concomitant toxicity; (ii) up-modulation of early (spinous keratins K1 and K10) and late (loricrin and filaggrin) differentiation markers; (iii) increased activities and expression of superoxide dismutase and catalase; (iv) decreased cell damage and apoptosis following UVA exposure. The hitherto unrecognized role of DHICA as a protective, antiapoptotic and antiproliferative endogenous cell messenger points to a reappraisal of the biological functions of melanocytes and TRP2 in skin homeostasis and photoprotection beyond the mere provision of melanin pigments.

C29

Distribution patterns of eumelanin and pheomelanin in human skin

S. G. Coelho, S. Ito, K. Wakamatsu, S. A. Miller, J. Z. Beer, V. J. Hearing
Laboratory of Cell Biology, National Cancer Institute, NIH, Bethesda, MD, USA

In humans, the gradient from light to dark skin color can be seen throughout the world's population. These differences in pigmentation can be attributed to the quantity, size and types of melanins (eumelanin and/or pheomelanin) that are produced by epidermal melanocytes in response to a variety of melanogenic factors, environmental stresses, etc. The identification of melanins in tissue sections using the standard Fontana–Masson silver stain is not very sensitive and does not provide any specificity as to the type of melanin(s) being visualized. In order to characterize the pigmentary patterns in human skin, we generated several antibodies to melanins based on synthetic melanin preparations of Pheo-melanin (DOPA + CYS-melanin), 5,6-dihydroxyindole (DHI)-melanin, 5,6-dihydroxyindole-2-carboxylic acid (DHICA)-melanin and DHI/DHICA-melanin. These antibodies were initially tested in vitro using a dot-blot assay to evaluate their specificities and sensitivities against each type of melanin. We then used them to investigate the eumelanogenic and pheomelanogenic distribution patterns in normal human melanocytes and in human skin using immunocytochemistry and immunohistochemistry, respectively. We report the characterization of several distinct polyclonal rabbit antibodies generated against several types of synthetic melanins. Analysis by confocal microscopy indicates that the antibodies raised against Pheo-melanin, DHI-melanin and DHICA-melanin are valuable for studying melanin distribution in melanocytes in vitro and in situ. In addition, those antibodies show differences in melanin distribution among melanocytes and keratinocytes in epidermal skin layers of different phototypes with or without UV exposure. Based on these results, we propose that the improved sensitivity and specificity of the eumelanin and pheomelanin identification in tissue sections will allow the characterization of specific differences in melanin distribution patterns in human skin of varying skin color.

C30

Fibroblasts regulate both physiological and pathological pigmentation of skin in vitro and in vivo

M. Cario-Andre, K. Ezzedine, C. Pain, V. Guyonnet-Dupérat, A. Bibeyran, A. Taïeb
INSERM U1035, Department of Dermatology and Pediatric Dermatology, CHU de Bordeaux, France

We have previously described that white human skin or white human epidermal reconstructs xenografted onto nude mice could become black or totally white. The increase in human epidermal pigmentation was shown to be associated with an increase in melanin content and melanosome transfer whereas the white pattern was associated with a disappearance of melanocytes, like in vitiligo. These modifications of pigmentary pattern were associated with changes in densities of dermal fibroblasts. In vitro, we have compared reconstructs made on dead de-epidermized dermis colonized or not with various concentrations of fibroblasts or treated with fresh or frozen media conditioned by various densities of human or murine fibroblasts and we have reproduced this phenomenon of increase or decrease of pigmentation. We have noticed that FGF2 stimulates melanin secretion, and that cytokeratin 5, which is associated with melanosome transfer, was overexpressed in

 © 2011 The Authors. Journal Compilation © 2011 John Wiley & Sons A/S

epidermis of hyperpigmented samples. In vitro treatment of reconstructed epidermis without fibroblasts with FGF2 increased cytokeratin 5 expression. Using our model of reconstructed epidermis with fibroblasts of systemic scleroderma (SSc) patients, which in addition to fibrosis have skin pigmentary changes, we were able to demonstrate that SSc fibroblasts can either stimulate or inhibit pigmentation according to patients. To better understand the dermal influence on pigmentation we compared lesional and perilesional skin of a patient with a pronounced depigmentation following erythema multiforme for TRP-1, MelanA, Vimentin, K5, FGF2, expression. We found that the number of melanocytes was decreased in lesional skin and that the remaining melanocytes were non functional (TRP-1 negative) which may explain the depigmentation. Interestingly, FGF2 was overexpressed in lesional area whereas densities of fibroblasts and K5 expression were similar in both areas. Since all these data point out the key role of FGF2, we are using our model of reconstructs with fibroblasts overexpressing FGF2 to detect changes in pigmentation. In conclusion, our data associated with clinical observations of pigmentary disorders support the hypothesis that fibroblasts modulate pigmentation by paracrine factors especially FGF2.

C31

Essential role of dermal components in regulating the pigmentation in a full thickness reconstructed skin model

C. Duval, C. Cohen-Dellarre, C. Chagnoleau, F. Bernerd
L'Oréal, Clichy, France

Essential role of dermal components in regulating the pigmentation in a full thickness reconstructed skin model. 3-D pigmented skin models are useful tools for research purposes in skin pigmentation as well as for the evaluation of the efficacy and safety of pro- or depigmenting agents. We recently developed a highly physiological model composed of a fully stratified and well differentiated pigmented epidermis onto a dermal compartment populated by fibroblasts. Constitutive and inducible pigmentation could be obtained in this model demonstrating its functionality. In the present study, we investigated the effects of dermal compartment modifications on the skin model pigmentation. In a first approach, reconstructed pigmented skin samples produced normally or without viable fibroblasts in the collagen matrix were compared. The absence of fibroblasts induced a drastic hyperpigmentation thus showing the important role of these cells in regulating the pigment synthesis. Then, reconstructed skin models were made with dermal fibroblasts of different skin origin, especially taken from donor of different age and from photoprotected or photoexposed sites. Interestingly, our results indicate that the degree of the constitutive pigmentation solely depends on the characteristic of the fibroblast strains. In a second approach, the effect on the pigmentation level of Pro-Xylane™, a molecule known to modulate extracellular matrix components, was studied. We first showed that in the absence of fibroblasts within the collagen matrix, Pro-Xylane did not modify the pigmentation level of reconstructed skin. In contrast, we found that in presence of dermal fibroblasts, Pro-Xylane significantly reduced the pigmentation level without affecting the melanocytes pool. Altogether, these results validate the fact that the dermal compartment, especially the nature of the fibroblasts, play a regulatory role in skin pigmentation and illustrate that pigmentation can be efficiently modulated by a molecule such as Pro-Xylane acting on dermal matrix.

CS9: Depigmentation update, clinically-oriented

C32

Association between severity of solar lentigines and faity acid intakes in adult Caucasian women

J. Latreille, K. Ezzedine, E. Kesse-Guyot, R. Jdid, D. Malvy, P. Galan, E. Tschachler, C. Guinot, S. Hercberg
CERIES Neuilly sur Seine, France

Topical application of fatty acids has been proposed for the treatment of melasma patients. However, data on the relationship between fatty acids diet and the presence of skin hyperpigmentation is scarce. The aim of this study was to investigate the links between daily fatty acid intakes and the severity of solar lentigines. In a cross-sectional study conducted on a sub-sample of women of the SU.VI.MAX cohort, the solar lentigines were assessed on forehead and cheeks by a dermatologist using a specific ordinal scale with photographic illustrations. Then, a score of lentigines (0–10) was calculated. Besides, the fat diet was determined using at least ten 24-h dietary record questionnaires completed during a period of 2.5 yr for 229 women. Total lipid, saturated fatty acids, monounsaturated fatty acids (MUFA), and polyunsaturated fatty acids: linoleic (LA; 18:2n-6), α-linolenic (ALA; 18:3n-3), arachidonic (AA; 20:4n-6), eicosapentaenoic (EPA; 20:5n-3), n-3 docosapentaenoic (DPA; 22:5n-3), and docosahexaenoic (DHA; 22:6n-3) acids were estimated. After adjustment on possible confounders, a higher intake of AA was found to be significantly linked with a lower severity of solar lentigines and a higher intake of monounsaturated fatty acid tended also to be linked with a lower severity of solar lentigines. Our findings suggest that dietary fatty acids could play a role in hyperpigmentation disorders such as solar lentigines.

C33

The Role of DKK1 in the development of vitiligo

J. Y. Kim, J. Y. Shin, M. R. Kim, S.H. Oh
Department of Dermatology and Cutaneous Biology Research Institute, Seoul, Korea

Vitiligo, an acquired depigmentary disorder of the skin, is caused by melanocyte destruction by an autoimmune mechanism and decreased levels of mitogens for melanocytes. Dickkopf 1 (DKK1) secreted by fibroblasts is responsible for thickened and hypopigmented palmoplantar epidermis. We examined the expression of DKK1 between vitiligo lesion and non-vitiligo lesion in the protein and mRNA levels.

Methods: Vitiligo lesional and non-lesional skins were taken from fifteen Korean patients with vitiligo. Immunohistochemical stainings for DKK1, PAR-2 and β-catenin were performed, and their expressions were compared between lesional and non-lesional skins. In addition, real-time RT-PCR was performed to compare expression of DKK1 and β-catenin between lesional and non-lesional skins. Vitiligo lesions showed significantly lower expression of PAR-2 in immunohistochemical staining compared to non-vitiligo lesions. Expression of DKK1 in vitiligo lesions was much higher than that in non-vitiligo lesions in both immunohistochemistry and real-time RT-PCR. In immunofluorescence staining for β-catenin, expression of β-catenin in vitiligo lesions was somewhat lower than that in non-lesional skins without significance. However, expression of β-catenin mRNA in depigmented lesions were significantly lower compared with the normally pigmented skins. High expression of DKK1 in vitiligo lesions might affect the survival of melanocytes by suppressing PAR-2 expression or Wnt signaling. DKK1 secreted by fibroblasts might play a role in the development or maintenance of vitiligo.

© 2011 The Authors. Journal Compilation © 2011 John Wiley & Sons A/S

C34

Hairs presence and pigmentation in vitiligo lesions: the usefulness of dermatoscopy in prognosis and treatment response evaluation

M. C. Costa, L. S. Abraham, M. Ardigò, M. Picardo, P. L. Araújo, L. Azulay-Abulafia, J. M. Piñeiro-Maceira
Instituto de Dermatologia Prof. Azulay, Rio de Janeiro &Brasilia, Brazil

It is difficult to predict topical or systemic therapy response in vitiligo. Moreover, and, not rarely, in the same lesion, different responses to one treatment can be found. This is often related to hair follicles presence and their pigmentation. Residual perifollicular islets are considered to be melanocytes reservoirs and this is the reason they exert considerable influence on vitiligo prognosis. Dermoscopy, a noninvasive, handheld and low cost toll, widespread used by dermatologists for melanocytic lesions assessment, has shown great usefulness in analyzing vitiligo; in particular, diminished hair presence and its pigmentation, which can help in both prognostic and treatment response evaluations. In our daily practice, we largely use dermoscopy to evaluate vitiligo lesions and have observed a close correlation to hair presence and pigmentation with vitiligo prognosis. In order to verify this hypothesis, we selected 12 patients presenting vitiligo that underwent clinical and dermoscopic evaluation prior and after phototherapy. Seen by the naked eyes, some lesions contained both dark and white hairs inside, while others, especially when located on face and extremities, apparently contained no hair. However, using a manual dermatoscope, we could easily notice the presence of hair follicles inside and if they were pigmented or not. In detail, we could additionally observe areas with very thin hair, some pigmented and some white, that previously, without dermoscopy, were misjudged as areas of hair absence. After 3 months of phototherapy (UVB-NB or PUVA), we detected that in the areas presenting less or no repigmentation, most of the hair follicles were white; whereas in the ones presenting more repigmentation, it occurred surrounding pigmented hair follicles. By using a handheld device with a 10× image magnification, we were able to observe a strong correlation between pigmented and nonpigmented hair and the repigmentation phenomenon. Consequently, for a better clinical assessment of vitiligo lesions, we consider the use of dermoscopy as useful and it should be performed routinely, especially when hair shafts are too diminished or thin to be seen with the naked eye, like in women and children or in facial areas. Thereby, dermoscopy can be considered an easily handling and helpful device not only to vitiligo prognosis but also to its therapy response evaluation.

C35

H19RNA downregulation in melasma

A.-Y. Lee, N.-H. Kim, J. M. Kim, K. A. Cheong
Dongguk University Graduate School of Medicine, Gyeonggi-do, Korea

Recently, we reported that H19 downregulation might involve in melasma development, although the role in tumorigenesis but not in melanogenesis has been reported. Downregulation of the H19 gene was detected on microarray analysis of hyperpigmented and normally pigmented skin from melasma patients, and significant clinical correlations were identified. The H19 knockdown in melanocyte monoculture did not result in obvious tyrosinase overexpression, whereas the knockdown in a mixed normal human cell culture system, composed of H19 siRNA transfected keratinocytes and non-transfected melanocytes, did induce both a tyrosinase overexpression and an increase of melanosome transfer. Moreover, estrogen treatment of the H19 RNA knockdown in the mixed cell culture was more than an additive effect on the tyrosinase overexpression, whereas UV irradiation was not. Since H19RNA is a noncoding RNA, the importance could be determined with respect to the control of gene expression. Although the H19 consists of an imprinted cluster with IGF2, no reciprocal changes was detected between H19 and IGF2 RNA expression in the melasma. It has been reported that a 23-nucleotide microRNA, miR-675, derived from H19 is endogenously expressed in human keratinocytes. We found SUN3 as a target gene for miR-675. The SUN-3 expression was increased in the hyperpigmented compared to normally pigmented skin from melasma patients. The H19 knockdown using siRNA also increased the SUN3 expression, which involved in melanosome transfer. These findings supported the role H19 downregulation in the melasma development.

Plenary session III: Stem Cells: facts, fancy, fiction?

C36

Protective effect of Kit signaling for melanocyte stem cells against radiation-induced genotoxic stress

H. Aoki, T. Kunisada
Gifu University Graduate School of Medicine, Gifu, Japan

Radiation-induced hair graying is caused by irreversible defects in the self-renewal and/or development of follicular melanocyte stem cells (MSCs) in the hair follicles. X-ray damaged melanocyte stem cells seemed to take the fate of ectopically pigmented melanocytes in the bulge regions of hair follicles in vivo. Kit signaling is an essential growth and differentiation signaling pathway for various cell lineages including melanocytes and its radioprotective effects have been shown in hematopoietic cells. To investigate whether Kit signaling exerts a radioprotective effect for melanocytes, we investigated the radioprotective function of Kit signaling in melanocytes in vivo. According to the telogen-hair plucking method (Potten, 1970), one day after plucking the hair on the dorsal skin, the mice were irradiated at 7–8 weeks of age (Argyris and Chase, 1960). Whole-body X-ray radiation (IR) was performed using a Hitachi MBR-1520 (Hitachi Medical) operating at 50 kV or 150 kV, 20 mA with a 2.0 mm Al filter and a dose rate of 0.4 Gy/min as previously reported (Inomata et al., 2009). 6, 24 h or 1 week after irradiation, the skins were analyzed by immunohistochemistry. Various loss-of-function mutations of Kit facilitate radiation-induced hair graying. The extreme possibility that changes in the numbers of MSCs in vivo were the cause of the radioprotective effect of Kit signaling observed was excluded by demonstrating the nearly constant numbers of MSCs in the mice used in our experiments. The X-ray doses used did not show a systemic lethal effect, indicating that the in vivo radiosensitivity of Kit mutants is mainly caused by the damaged MSC population. In contrast, transgenic mice expressing the ligand for Kit (Kitl) in the epidermis have significantly reduced levels of radiation-induced hair graying. We also observed a reduced frequency of double strand breaks measured by phosphorylated H2AX staining which corresponded to the increased Kit signaling level. The prominent radioresistance of MSCs observed in hk14-Kitl transgenic animals. Endothelin 3, another growth and differentiation factor for melanocytes, showed a lesser radioprotective effect compared with Kitl. By comparing hair graying at various Kit signaling levels following exposure to IR, we demonstrate a significant in vivo radioprotective role of Kit signaling in the regeneration of melanocytes in hair follicles.

 © 2011 The Authors. Journal Compilation © 2011 John Wiley & Sons A/S

C37

Dual function of membrane-bound KitL and potential role to anchor melanocyte stem cells in their niche

S. Tabone-Eglinger, M. Wehrle-Haller, N. Aebischer, M.-C. Jacquier, D. Boettiger, B. Wehrle-Haller
Centre Médical Universitaire – Dept of Cellular Physiology and Metabolism – Lab 6218, Genève, Switzerland

Kit Ligand (KitL) is a growth factor crucial for cells of the melanocytic, hematopoietic and germ cell lineages. As a result of differential splicing and sensitivity to proteolytic shedding, KitL is expressed either as a stably membrane-bound (mb-KitL), or releasable form (s-KitL). KitL was reported to maintain hematopoietic stem cells (HSC) in their niche, while its proteolytic shedding induced the release of HSC from the bone marrow, suggesting that mb-KitL might not only convey survival signals but also provide a mechanical anchor for stem cells in their niche. Fluorescently tagged KitL (GFP) or ckit (cherry) proteins were stably (MDCK epithelial cells) or transiently (COS cells) expressed, to study their respective mechanical (spinning disc, FRAP) and signaling (P-tyr staining) interaction. FRAP and the spinning disc device (Garcia et al. 1997) was used to measure the dynamics of the KitL/c-kit interaction and its respective resistance to shear stress between c-kit (MC9 cells) and mb-KitL expressing cells (COS, MDCK), or KitL coated surfaces. When co-cultured, both c-kit as well as KitL accumulated in clusters at the interface of adjacent cells, which were highly tyrosine phosphorylated. These clusters were mobile, demonstrating fusion and fission events. Compared to non-clustered mb-KitL, fluorescence recovery after photobleaching (FRAP) was very slow, demonstrating a high stability of the KitL/c-kit complex. In addition, spinning disc experiments revealed resistance to mechanical forces between MC9 mast cells and membrane-bound or substrate immobilized KitL, with strength ressembling shear stresses in small vessels and capillaries. Interestingly, this mechanical link was independent of cKIT kinase activity (inhibited with imatinib mesylate). In contrast, surfaces with immobilized cKIT, were not able to induce force resistance of mb-KitL expressing cells. This suggested that the cytoplasmic part of cKIT, establishes a physical connection to the cytoskeleton, independent of its kinase activity. Our data are consistent with a dual function of mb-KitL providing mechanical anchoring and signaling to cKIT expressing cells, giving new mechanistic insight into the role of mb-KitL/c-kit interaction in the hematopoietic and melanocytic niche. In addition, it highlights the important role of the epidermal niche in controlling melanocyte behavior by altering KitL presentation.

C38

Characterisation of pluripotent immortal postnatal mouse neural crest-like stem cells

L. Latif, I. E. Tribe, B. Babakinejad, S. A. Kamali, R. H. Patel, Y. A. Negulaev, Y. E. Korchev, E. V. Sviderskaya
St George's University of London London, UK

We have established a novel type of pluripotent neural crest-like stem cells from neonatal mouse epidermis (Sviderskaya et al., 2009, FASEB J.). These cells isolated as three independent immortal lines resemble neural crest stem cells in their apparent capacity to differentiate into several cell types normally derived from the neural crest. Using alternative regulatory factors, they could be converted to either melanoblasts and melanocytes, chondrocytes, Schwann precursor cells, or functional sensory neurons. Here we investigated whether neural crest-like stem cells can be induced to produce other cell types. Firstly, we examined whether neuronal cells can be produced in the conditions previously shown to induce production of pre-Schwann cells. We examined the effect of transforming growth factor β1 (TGFβ1) and neuregulin β1 (NRG1-β1) on these cells growing in RPMI medium with foetal calf serum (FCS).
These factors significantly increased cell proliferation in 7 days with the highest effects resulting from treatment with both factors and NRG1-β1-treatment along (25.5% higher level of proliferation compared to TGFβ1-treatment) with increase of bi- and tri-polar cells. These cells showed immunoreactivity for a neuronal marker, neurofilament. About 14% of all cells showed positive staining with both growth factors after 7 days of treatment. The percentage of stained cells increased after longer treatments. Secondly, we confirmed that smooth muscle cells, another mesenchymal cell type and product of normal neural crest cells, could be produced by neural crest-like stem cells grown in the presence of BMP-2, TGFβ1 and FGF2 in DMEM medium with FCS. These factors increased cell proliferation with the effect of FGF2 being the highest and similar to that of all three factors combined. These factors also affected cell morphology. Immunostaining for phalloidin confirmed the appearance of triangular, stellate and spindle-shaped cells. They were immunoreactive for α-smooth muscle actin with the highest number of positive cells in BMP-2-treated cultures. Thus our immortal neural crest-like cells produce neuronal cells in the conditions previously described to induce production of Schwann precursor cells, and two types of mesenchymal cells, chondrocytes and smooth muscle cells, and thus can differentiate into six cell lineages. These lines provide a new and valuable model for large-scale studies of the biology and gene expression of neural crest.

C39

In vivo responses of melanocyte stem cells and other immature melanocytic cells to ultraviolet radiation-induced damage

B. Ferguson, T. Kunisada, G. Walker
Queensland Institute of Medical Research, Herston, Australia

The induction of melanoma in mice with neonatal UVR is accompanied by a strong melanocyte (MC) proliferative response. In neonates MCs are in the epidermis but migrating downwards in contact with keratinocytes that are forming the developing hair follicle. After UVR MCs migrate upwards out of the follicular bulge, the location of MC stem cells (MCSCs), again appearing in the interfollicular epidermis a few days later. In adult mice MCSCs are only activated during early anagen. As it is unknown how MCSCs respond to UVR-induced damage, we assessed how they, and other immature bulge MCs, respond by differentiating, proliferating, and migrating to the burned area of skin. Neonatal or adult mice were given a single burning UVB exposure and sacrificed at multiple time points afterwards. Skin sections were stained with MC-specific antibodies. We assessed the response of MCSCs to UVR by 'isolating' them in vivo in neonates using antibody-mediated depletion of all other MCs, or by exposing adult skin with hair follicles synchronized in telogen. After neonatal UVR MCs increased in number in the epidermis, and in the upper outer root sheath (ORS) of hair follicles (bulge and infundibulum). At least three distinct populations are observed; Kit -ve/Trp1 -ve MCSCs, Kit +ve/Trp1-ve transit amplifying (TA) cells, and fully differentiated Kit +ve/Trp1+ve MCs. After MC depletion with a Kit-blocking antibody, which removes all MCs except MCSCs, we saw no UVR-induced activation of MCs in the bulge and none in the epidermis. We further tested the response of MCSCs to UVR in adult mice after synchronizing the hair cycle in telogen or anagen, but we could not elicit MC proliferation in the bulge or ORS (at least with a single exposure). UVR treatment of K14-Kitl mice, which have

© 2011 The Authors. Journal Compilation © 2011 John Wiley & Sons A/S

epidermal MCs throughout life, also revealed major differences in UVR responses of neonatal and adult MCs even in the epidermal setting. A single UVR-induced insult to the skin is insufficient to induce MCSC activation in either neonatal or adult mice. Alternatively, in neonates MCSCs may respond indirectly, requiring the presence of UVR-activated 'TA' cells which in turn signal MCSC activation. As the MC proliferative response does not occur in adult follicles, even in anagen, neonatal MCs must be exquisitely sensitive to this response. Contact between MCs and keratinocytes is critical to the MC migration.

Plenary session IV: Photoprotection and beyond: from melanosomes to melanins

C40

Ultraviolet absorption properties of melanosomes measured by photoemission electron microscopy

J. D. Simon, D. N. Peles
Department of Chemistry, Durham, USA

Central to understanding the photochemical properties of melanosomes is a direct measurement of their absorption cross-sections. The objective of this study is to determine the absorption spectra of intact melanosomes of varying melanin compositions over the spectral range from 240 to 310 nm. A novel approach to photoemission electron microscopy is used to obtain the first direct measurement of the absorption cross-sections from intact melanosomes isolated from tissues of bovine and human eyes. There is significant heterogeneity among the absorption spectra for individual melanosomes; however, the majority of the melanosomes examined share a common set of features.

1 The absorption spectra of eumelanosomes differs significantly from that of the precursor molecules, DHI and DHICA, to eumelanin, the UV-absorbing pigment contained in the melanosome. This difference is most notable in the UV-A region and indicates that polymerization of DHICA into the melanin pigment disrupts the electronic structure of these monomeric building blocks.

2 The absorption spectra of melanosomes containing a mixture of eumelanin and pheomelanin do not significantly differ from those containing pure eumelanin, arguing that the two pigments have similar absorption properties on a per volume basis.

3 The absorption cross-section of bovine RPE melanosomes is greater than that for eumelanosomes isolated from bovine choroid or iris tissues, suggesting that either the pigment is present in greater density or that there is an underlying difference in molecular structure. In summary, the results suggest that there is little variation in the spectral properties of melanosomes with varying DHI/DHICA ratios and eumelanin/pheomelanin ratios.

C41

Pheomelanin is a prooxidant promoting DOPA conversion to a eumelanin coating: discovery of a non-enzymatic mimic of the natural casing process of melanosome assembly

A. Napolitano, G. Greco, L. Panzella, G. Gentile, M. E. Errico, M. d'Ischia
University of Naples Federico II, Dept. Organic Chemistry and Biochemistry, Naples, Italy

According to a currently accepted kinetic model, assembly of pigment-containing organelles, the melanosomes, occurs by a casing process in which a pheomelanin core is initially formed and is then encapsulated into a eumelanin coating. Photoemission electron microscopy imaging studies of iridial melanosomes and neuromelanin granules showed eumelanin-type surface properties despite a large pheomelanin content. The present study aimed at probing the oxidation reactivity of pheomelanin to address at chemical level the mechanisms underlying the growth of eumelanin shell onto the pheomelanin core. Oxidation of dopa and other eumelanin precursors at 1 mM concentration in air-equilibrated phosphate buffer at pH 7.4 in the presence of 20% (w/w) 5-S-cysteinyldopa melanin (CD-melanin) is followed by HPLC analysis. The resulting dark pigment is analyzed by scanning electron microscopy (SEM) and chemical degradation. The rate of aerial oxidation of a series of catecholamines including DOPA is markedly increased in the presence of CD-melanin, leading to dark insoluble eumelanin-like polymers. SEM analysis indicated a close similarity of the morphology of the resulting pigment with that of a pure DOPA-melanin sample suggesting encasing of the CD-melanin component into the DOPA-melanin coating. Chemical degradation experiments indicated that while most of the CD-melanin was readily solubilized and released from the filter by alkaline washings, no CD-melanin was detected in the washings of the DOPA melanin-coated sample, confirming the presence of an insoluble DOPA-melanin coating preventing solubilization of the pheomelanin pigment during the washing steps. In conclusion, synthetic pheomelanin from CD is capable of accelerating the aerial polymerization of DOPA to give eumelanin-like deposits that encapsulate the pheomelanic core and gradually quench the redox centers. The oxidative polymerization process is likely mediated by key benzothiazine units through a redox interaction mechanism occurring on the surface of the finely suspended pro-oxidant polymer. These results disclose a non-enzymatic process mimicking the natural casing model of melanin synthesis which may be relevant to the origin of the casing architecture in the neuromelanin of human substantia nigra which apparently lacks enzymatic systems for catecholamine oxidation and deposition onto the pheomelanin-like core.

C42

Genomics of pattern: from Akitas to Zebras

C. Kaelin, L. Hong, X. Xu, V. David, A. Schmidt-Kuentzel, S. O'Brien, M. Menotti-Raymond, G. Barsh
HudsonAlpha Institute, Huntsville, AL, USA

Stripes and spots in mammals are a fascinating character thought to be important for camouflage and species recognition, but the genetic basis for pattern formation and pattern variation remains unknown. In nature, the remarkable diversity of color patterns is exemplified by the variation displayed among the 36 extant wild cat species. We demonstrate the utility of a forward genetics approach in domestic cats that takes advantage of emergent genomic resources, genomic partitioning, and next generation sequencing to fine map and to clone pigmentation patterning genes. Using this approach, we find that at least one variation in pattern type is caused by recessively inherited loss-of-function in a gene conserved among all vertebrates, and whose identity is consistent with a theoretical reaction-diffusion system first proposed by Alan Turing more than 50 yr ago. A mutation in the same gene causes a characteristic wild felid phenotype. We also developed a highly sensitive and robust methodology—EcoP15I-tagged Detection of Gene Expression, or EDGE—that is suitable for detecting and comparing gene expression among tissues from animals for whom fully assembled and annotated genomes do not yet exist. We carried out an EDGE analysis of patterned skin from dogs (yellow and black brindled stripes), cheetahs (yellow and black spots), and zebras (white and black stripes). In brindled dogs, the striping pattern is caused by a segmental duplication that leads to gene silencing,

 © 2011 The Authors. Journal Compilation © 2011 John Wiley & Sons A/S

and our results suggest that epigenetic alterations in gene expression are confined to the duplicated segment. In zebras, the striping pattern is limited to hair rather than skin, and our results indicate that alterations in hair color are accompanied by alterations in hair structure.

C43

Germline mutations in BAP1 predispose to melanocytic tumors

T. Wiesner, A. C. Obenauf, R. Murali, I. Fried, P. Ulz, S. Loy, W. Wackernagel, C. Windpassinger, I. Wolf, K. G. Griewank, A. Viale, A. E. Lash, M. Pirun, N. D. Socci, A. Ruetten, G. Palmedo, D. Abramson, K. Offit, A. Ott, J. C. Becker, L. Cerroni, H. Kutzner, M. R. Speicher, B. C. Bastian
Department of Pathology, Memorial Sloan-Kettering Cancer Center, New York, NY, USA

We report a new autosomal dominant syndrome characterized by multiple skin-colored, elevated melanocytic tumors. The melanocytic neoplasms ranged histopathologically from epithelioid nevi to atypical melanocytic proliferations that showed overlapping features with melanoma. Some affected patients developed uveal or cutaneous melanomas. Segregating with this phenotype, we found inactivating germline mutations of the BAP1 gene. The majority of melanocytic neoplasms lost the remaining wild-type allele of BAP1 by various somatic alterations. Our findings identify BAP1 as a novel susceptibility gene for melanocytic neoplasia and characterize a form of epithelioid melanocytic nevus that was previously subsumed under the category of spitzoid lesions.

CS10: Genetics of pigmentation and molecular biology of melanoma clinically-oriented

C44

Uncovered role of Tyrosinase-related Protein 1 TYRP1 in melanoma cells aggressiveness

A. Mogha, D. Gilot, A. Primot, J. Debbache, F. Journe, D. C. Bennett, B. Dreno, A. Napolitano, G. Ghanem, M.-D. Galibert
CNRS-UMR6061 French National Center of Scientific Investigation, Rennes, France

TYRP1 is the most abundant glycoprotein in melanocytic cells and has been involved in pigmentation. While the murine protein has been reported as a DHICA-oxidase enzyme in the eumelanin pathway, the exact role of human TYRP1 remains unclear. We quantified TYRP1 transcript levels by RT-qPCR in a cohort of 62 metastatic melanoma biopsies, what led us to distinguish two subgroups according to high and low expression levels of TYRP1. These results were consistent with recent data obtained by G. Ghanem's group from J Bordet Institute (Belgium): they demonstrated that metastatic melanomas with high levels of TYRP1 mRNA were correlated to overall survivals lower than 30 months. Thus, to address the question of a potential link between TYRP1 expression levels and melanoma outcome, we adopted an RNA-interference strategy using lentivirus encoding short hairpin RNA (shRNA) to knock-down TYRP1 expression in three melanoma cell lines derived from vertical-growth phase (VGP) and metastatic tumors. TYRP1 depletion resulted in dramatic morphological changes and cytoskeleton remodeling. Moreover, shTYRP1 cells displayed decreased proliferation, migration and invasion abilities, but no cell death. Such consequences were not observed when TYRP1-negative melanoma cells were infected with shTYRP1-lentivirus, indicating that this phenotype is specific to TYRP1 knock-down. Altogether, these results show that TYRP1 modulates the aggressiveness of melanoma cells and raise the question of another function for this protein, what is currently under deep investigation.

C45

Molecular analysis of 246 patients with oculocutaneous albinism – the Bordeaux experience

F. Morice-Picard, E. Lasseaux, C. Rooryck-Thambo, A. Rouault, D. Cailley, C. Plaisant, D. Lacombe, A. Taïeb, B. Arveiler
Laboratoire Maladies Rares – Génétique et Métabolisme, Bordeaux, France

Oculocutaneous albinism (OCA) is a rare autosomal recessive disease affecting 1/20 000 person in the general population. OCA is caused by mutations in the TYR (OCA1), P (OCA2), TYRP1 (OCA3), and SLC45A2 (OCA4) genes. Mutations of GPR143 are responsible for X-linked ocular albinism (OA1). Due to extensive phenotypic heterogeneity and lack of genotype-phenotype correlations, molecular investigations are necessary to establish the diagnosis and to evaluate the relative frequency of the different forms of albinism. We analysed 246 patients from various origins (mainly Europe). Point mutations were analysed by DHPLC/HRM and sequencing of exons, intron-exon junctions and promoter regions. Gene dosage anomalies were investigated by semi-quantitative PCR (QMF-PCR) and, more recently, by high resolution (100 bp) array-CGH using a custom array covering the OCA1–4 and OA1 genes. The molecular analysis identified 141 point mutations (missense, nonsense, splice site) in TYR, 88 in P, eight in TYRP1, 25 in SLC45A2 and 11 in OA1. We identified three deletions in TYR, 28 deletions and two duplications in P, and two deletions in SLC45A2. The relative frequencies of OCA1–4 were: 46.4% OCA1, 35% OCA2, 2.4%OCA3, and 16.2% OCA4. Despite the extensive analysis of the OCA1–4 and OA1 genes, a single heterozygous mutation was found in 28 patients (11.4%), and no mutation was found in 40 patients (16.3%). The thorough analysis of the OCA1–4 and OA1 genes in 246 patients allowed us to establish a diagnosis in 178 patients (72.3%). Intragenic microrearrangements accounted for 10.5% of the OCA1–4 alleles. It is worth noting that microrearrangements represented as many as 25.4% of all OCA2 alleles (including the common exon 7 deletion). This shows that microrearrangements should be searched for systematically in a diagnostic set up. Despite the thorough analysis performed, 27.7% of the patients remained undiagnosed. Mutations may therefore hide in unexplored regions of the genes (deep in introns, or in regulatory elements), or in other genes, which are either involved in syndromic forms of albinism or which still have to be discovered.

C46

Ser727 phosphorylation in STAT3 plays a crucial role in nuclear translocation of STAT3 and growth in human melanoma cells and melanocytes

M. Sakaguchi, M. Oka, T. Iwasaki, Y. Fukami, C. Nishigori
Division of Dermatology, Department of Internal Related, Kobe University Graduate School of Medicine, Kobe, Japan

The transcription factor signal transducer and activator of transcription 3 (STAT3) is constitutively activated through Tyr705 phosphorylation in most melanoma cells. In this study, the role and regulation of Ser727 phosphorylation in STAT3, another critical phosphorylation event, in melanoma cells and melanocytes were examined. Ser727 was constitutively phosphorylated in all of seven melanoma cell lines examined, whereas Tyr705 phosphorylation was absent in two cell lines. Constitutive Ser727 phosphorylation was partially attenuated by U0126, an inhibitor of extracellular-regulated kinase (ERK) kinase (MEK). In WM39 cells

© 2011 The Authors. Journal Compilation © 2011 John Wiley & Sons A/S

containing STAT3 phosphorylated on Ser727 (pS-STAT3) but not STAT3 phosphorylated on Tyr705 (pY-STAT3), pS-STAT3 was localized mainly in the nucleus and U0126 treatment resulted in a decrease in nuclear pS-STAT3 and total nuclear STAT3 concomitant with an increase in total cytosolic STAT3. Blockade of STAT3 activity in WM39 cells by small interfering RNAs suppressed the growth of the cells. Melanocytes did not express pY-STAT3 but expressed low levels of pS-STAT3 in the nucleus. Upon treatment with 12-O-tetradecanoylphorbol-ester (TPA), a growth stimulator for melanocytes, nuclear pS-STAT3 and total nuclear STAT3 were increased, without affecting Tyr705 phosphorylation, concomitant with enhanced proliferative activity. These results indicate that Ser727 phosphorylation in melanoma cells is mediated in part by the MEK–ERK1/2 pathway and that it plays a crucial role in the nuclear translocation of STAT3 and growth in melanoma cells and melanocytes. Furthermore, immunohistochemical studies on specimens of primary lesions of acral lentiginous melanoma (ALM) revealed that Ser727 phosphorylation frequently precedes Tyr705 phosphorylation in the early stages of ALM progression.

C47

First evidence for tumor cell-leukocyte fusion in human cancer: a melanoma brain metastasis with a donor-patient hybrid genome following allogeneic BMT

R. Lazova, G. LaBerge, E. Duvall, N. Spoelstra, M. Sznol, D. Cooper, R. Spritz, J. Pawelek
Yale University School of Medicine, New Haven, CT, USA

Tumor cell fusion with motile bone marrow-derived cells (BMDCs) has long been proposed as a mechanism for metastasis, but while such fusion has been demonstrated in animal melanoma models little is known of this in human cancer. Here we report the first genetic evidence for BMDC-tumor fusion and genomic hybridization in a human melanoma brain metastasis from a patient with a prior allogeneic bone marrow transplant (male-male, sibling). Forensic STR analyses of DNA from melanoma cells revealed the presence of both donor and patient alleles, with common aberrant allelic patterns throughout the tumor at multiple chromosomal loci indicating a clonal origin of the tumor. Donor and patient DNAs were obtained from pre-transplant frozen lymphocytes. Formalin-fixed, paraffin-embedded tumor specimens were stained by immunohistochemistry with leucocyte common antigen (LCA) in order to avoid leucocytes during microdissection of tumor cells. LCA-negative tumor cells were isolated with an Arturus XT laser dissection microscope system. DNA was extracted and analyzed for donor and patient alleles using forensic STR analyses at 14 chromosomal loci and amelogenin for the X and Y. Tissue sections were analyzed from five regions throughout the tumor. DNA from donor and patient lymphocytes showed balanced diploidy at all loci and the allelic patterns were consistent with the sibling relationship. On the other hand, DNA from tumor cells contained a mixture of donor and host alleles at some loci and only donor or patient alleles at others. There was massive aneuploidy with donor and patient specific alleles present at eight of 15 loci and numerous allelic imbalances throughout involving 70% of the genome. Aberrant genetic profiles seen in one tumor region were seen in all, demonstrating a clonal origin of the tumor. The results provide the first genetic proof of leucocyte-tumor fusion and genomic hybridization in human cancer and indicate that the tumor was clonal and generated from a single fusion event. While this report concerns only one case, we demonstrate that forensic genetic analyses of tumors arising as secondary malignancies represents a fruitful area for research in tumor cell fusion in human cancer.

CS11: Vitiligo: basic science & medical clinically-oriented

C48

Histopathological staging of vitiligo lesions – Implications for treatment

S. K. Attili, V. R. Attili
NHS Grampian, Dundee, UK

Vitiligo is largely perceived as an invisible dermatosis with no significant histopathological findings. Basing on our experience of studying vitiligoid lesions over the last 8 yr, we propose that histopathology of vitiligo has an important role in the diagnosis as well as its treatment. Histopathological features of vitiligo lesions in routine haematoxylin and eosin stained sections from 280 patients were analyzed. Three primary patterns were identified common to focal, segmental as well as the generalized disease: (i) lichenoid inflammatory lesions (51%) or post-inflammatory changes characterized by a disarrayed dermo-epidermal interface with loss of melanocytes and mild perivascular lymphocytic infiltrates with melanophages in papillary dermis; (ii) stable depigmentation (42%) with absence of melanocytes as well as cellular infiltrates with minimal structural alterations; and (iii) long standing lesions with mild atrophy/sclerosis (7%) associated with gross structural alterations. These three patterns are proposed as evolutionary stages in the histopathogenesis of vitiligo which can also be used for selecting appropriate treatment protocols. Microinflammatory lesions are likely to respond to oral steroids/immunosupressants by arresting the disease progression. Stable lesions can respond better to treatments aimed at migration of dermal melanocytes to the depleted epidermis. Absence of cellular infiltrates in a biopsy can be an objective method to identify stability for vitiligo surgery as against an unreliable patient history. In longstanding lesions with adnexal destruction, melanocyte migration from the depleted dermal reservoir is unlikely and various surgical grafting techniques can be considered.

C49

A hot finding: mutant HSP70i to treat vitiligo

J. Mosenson, A. Zloza, J. Klarquist, S. Mehrotra, M. Nishimura, J. A. Guevara-Patino, I. C. Le Poole
Loyola University Chicago, Maywood, IL, USA

Vitiligo is a T cell mediated autoimmune disease of the skin that results in progressive depigmentation. We have previously shown that inducible heat shock protein 70 (HSP70i) is required to drive autoimmune depigmentation, with crucial involvement of amino acid residues 435–445. Here we evaluated depigmentation in vitiligo-prone mice vaccinated with HSP70i with and without a base pair mutation in this dendritic cell (DC) activating region. Wild-type C57BL/6 mice and vitiligo-prone strains transgenic for melanocyte-reactive T cell receptors: Pmel-1 (depigmentation initiating >6 months of age) and h3TA2 (progressive vitiligo by 4 weeks of age) were gene gun vaccinated with HSP70iQ435A, wild-type HSP70i, or empty vector DNA, and evaluated for depigmentation of the pelage. DCs were phenotyped by FACS analysis. Vitiligo-prone Pmel-1 mice displayed significantly increased depigmentation in response to wild-type HSP70i alone. Importantly, mutant HSP70iQ435A fully abrogated the depigmentation response, supporting the prophylactic potential of mutant HSP70i. In actively depigmenting h3TA2 mice, further depigmentation was visible within 4 weeks after HSP70i vaccination. In animals vaccinated with HSP70iQ435A however, a fully pigmented pelage returned. FACS analysis of non-lymphocyte splenocytes harvested from Pmel-1 mice

 © 2011 The Authors. Journal Compilation © 2011 John Wiley & Sons A/S

6 months after vaccination revealed distinct monocyte derivative cell populations with differing levels of CD11c and CD11b expression. The abundance of cells with an immunosuppressive phenotype significantly increased after vaccination with mutant HSP70i, whereas an opposite trend was observed for the inflammatory DC population. Together, these data support sustained effects of HSP70iQ435A on dendritic cells in vitiligo-prone mice and highlight the treatment potential of HSP70i with a single base pair mutation for progressive vitiligo.

C50

LXR-α as molecular switch that initiate transition from vitiligo lesional skin to repigmented skin?

R. Kumar, D. Parsad, A. J. Kanwar
Department of Dermatology, PIGMER, Chandigarh, India

The LXR is a member of the nuclear receptor superfamily of transcription factors. The liver X receptors (LXRs) in skin physiology and pathology has evolved rapidly in recent years. Liver X receptors (LXRs) modulate epidermal proliferation, carcinogenesis, differentiation, and permeability barrier function, which identifies them as promising drug targets for the treatment of skin disease. Vitiligo vulgaris patients with no ongoing treatment for last 8 weeks were selected. A biopsy was taken from clinically active perilesional skin before narrow band UVB and repigmented skin after narrow band UVB from 15 vitiligo vulgaris patients and a normal skin biopsy from six controls with their written informed consent and this part of the study was approved by the Ethics Committee at PGIMER, Chandigarh. Melanocytes were isolated and cultured from perilesional skin, repigmented skin of vitiligo patients and control. The mRNA expression was checked by RT-PCR analysis. Adhesion of melanocytes to collagen type IV and laminin5 was checked by adhesion assay. LXRα expression was compared in the perilesional skin of vitiligo vulgaris patients and controls, the expression was significantly more in perilesional melanocytes as compared to control. Whereas the adhesion of perilesional melanocytes to collagen type IV and laminin5 was significantly less as compared to control melanocytes. After treatment with narrow band UVB, we found that expression of LXRα significantly decrease whereas the adhesion of melanocytes increases. Our results demonstrate that treatment of control melanocytes with LXR α agonist 22-hydroxycholesterol significantly decreases the adhesion of melanocytes. Our present data revealed that the expression of LXRα at both mRNA and protein level was significantly higher and adhesion was significantly less in perilesional skin as compared control. It can be hypothesized that, higher LXRα expression might decrease the melanocyte adhesion in perilesional vitiligo skin which might lead to the melanocytorrhagy. There was significant decrease in LXRα expression and adhesion increased in repigmented skin after succseeful treatment with narrow band UVB. Therefore, we can hypothesis that LXR-α may act as molecular switch that initiate transition from vitiligo lesional skin to repigmented skin.

C51

'Bad soil' – defective local microenvironment for melanocytes in vitiligo

R. Yu, M. Su, A. Xu, X. Zhang, Y. Zhou
Department of Dermatology and Skin Science, University of British Columbia, Vancouver, Canada

Vitiligo is a common skin depigmentation disorder with unknown pathogenesis. The objective of this study is to systematically investigate the local microenvironment of melanocytes in the skin of patients with vitiligo in order to provide additional clues on vitiligo pathogenesis. Full thickness skin biopsies were obtained from (i) vitiligo lesional skin; (ii) vitiligo non-lesional skin; and (iii) control skin from healthy volunteers, and used for genomic transcription profiling analysis. Gene expression abnormalities were confirmed with immunohistochemistry (IHC), immune fluorescence microscopy (IF), explant skin culture and in vitro melanocyte survival experiments. Of 41 000 genes screened, 17 genes were defective in vitiligo skin compared with normal control skin, including one neuroenic factor that normally inhibits the innate immunity. Conversely, 13 genes were up-regulated, including eight genes that encode proteins and peptides involved in the activation of the innate immunity, especially the natural killer cells. The IHC, IF and cell culturing results confirmed these findings. The local environment of vitiligo melanocytes is non-supportive for their survival due to inadequate inhibitors and excessive activation of the innate immune response.

CS12: Stress responses

C52

Antioxydant defenses in human epidermal melanocytes and keratinocytes suggests that Nrf2 plays a peculiar role in epidermis: implication in vitiligo lesions

L. Marrot, J.-P. Belaïdi, L. Denat, D. Duche, C. Jones, P. Perez, J. Sœur, R. Rani, R. S. Gokhale, J.-R. Meunier
L'Oréal, Aulnay sous Bois, France

Melanin and its chemical intermediates can both generate and scavenge reactive species. Melanocyte have thus to manage a specific situation towards oxidative stress, especially when exposed to sunlight. For instance oxidative stress induced by UV radiation from a solar simulator (SSUV: 300–400 nm or UVA: 320–400 nm) in cultured human melanocytes was stronger when melanogenesis was stimulated. By comparing antioxidant status in human melanocytes and keratinocytes from same donors, we could show that: (i) reduced glutathione content was higher in keratinocytes; (ii) basal expression of NQO1 (mRNA and protein) was higher in melanocytes; (iii) when Nrf2 was stimulated (by sulforaphane, lipoïc acid or by silencing of Keap1), HO1 (mRNA and protein) and modulatory subunit of γ-glutamyl-cysteine-ligase (GCLm, mRNA) were mainly induced in melanocytes whereas catalytic subunit of GCL (GCLc) was over expressed in keratinocytes; and (iv) in a microarray assay, HO1, ferritin, catalase as well as genes from NQO family or GST family displayed a stronger basal expression in melanocytes whereas genes from GPX family were mainly expressed in keratinocytes. Moreover, Nrf2, NQO1, GCLm and GCLc were up regulated in lesional epidermal skin of subjects with vitiligo, suggesting that redox balance is impaired in this disease. These data are of importance in order to better understand how Nrf2 is involved in skin in order to cope with environmental oxidative stress.

C53

The parrodiene derivative, 2,4,6-octatrienoic acid, acts as a novel promoter of melanogenesis and antioxidant defence in normal human melanocytes in situ and in vitro via PPARγ activation

E. Flori, A. Mastrofrancesco, D. Kovacs, Y. Ramot, S. Briganti, R. Paus, M. Picardo
San Gallicano Dermatologic Institute, Rome, Italy

Melanogenesis is controlled by the activation of several intracellular transduction pathways. Increasing evidence suggest an important role for nuclear receptors (NRs)-dependent pathways, including those mediated by carotenoids, retinoids and peroxisome proliferator-activated receptor (PPAR) ligands. In particular,

© 2011 The Authors. Journal Compilation © 2011 John Wiley & Sons A/S

the PPARγ isoform induces melanocytes differentiation and antioxidant properties. Since parrodienes share some structural features with carotenoids and retinoids, stimulate antioxidant defence and counteract senescence phenomena in fibroblasts, we explored whether the parrodiene-derivative, 2,4,6-octatrienoic acid (Octa), alters key parameters of melanogenesis and antioxidant defence. Further, we checked whether any observed Octa effects are related to PPARγ signaling. Pigmentary and antioxidant Octa effects were studied in organ-cultured human skin and/or in primary human melanocytes (NHMs), employing molecular (transfection, Real Time-RT-PCR, Western Blot), biochemical (enzymatic assays and spectrophotometry) and morphological approaches (Immunofluorescence and histochemistry). Octa promoted melanogenesis by up-regulating MITF protein levels as well as tyrosinase expression and activity. This correlated with an increase of melanin content in both, human epidermis in situ and NHMs. Moreover, Octa increased the biological antioxidant potential content, and the expression and activity of catalase in NHM in vitro. These effects appeared to be largely mediated by PPARγ activation, since PPARγ silencing was able to abrogated the observed effects. Since optimal photoprotection is provided by a combined pigmentary response and activation of physiological systems able to reduce UV damage, our results strongly encourage one to explore Octa as a promising novel candidate for promoting human skin photo-protection.

C54

Pigment melanin mediates a redox reaction between adsorbed nitric oxide and O_2 in vitro

J. Menter, C. Nokkaew, A. Sprewell, D. Eatman, S. Harris-Hooker
Morehouse School of Medicine, Atlanta, GA, USA

Pigment melanin can adsorb molecular O_2, scavenge nitric oxide (NO) and thereby couple a redox reaction between them. In this work, we show formation of peroxynitrite (ONOO-) in the presence but not in the absence of melanin. NO generated by DEA/NO or SNAP was dialyzed into membranes containing purified sepia melanin in 0.1 M phosphate buffer, pH 7.4 or control buffer alone. NO was measured as nitrite and nitrate via the Greiss methodology and by the DAF fluorescence assay. Peroxynitrite was detected by selective scavenging with 3.3 μM MCP or via detection of nitrotyrosine in cultured fibroblasts. H_2O_2 was monitored by the scopoletin/peroxidase assay. Appropriate controls were used. Dialyzate NO concentrations were significantly lower in the test dialyzates than in controls. In the test systems in vitro we detected significant amounts of peroxynitrite but little or no hydrogen peroxide. No significant amounts of either of these were detected in the absence of melanin. In cultured fibroblasts, we observed positive staining for nitrotyrosine in the presence, but not in the absence of melanin. Sepia melanin can couple the redox reaction between adsorbed NO and O_2 to afford ONOO – via a superoxide intermediate. Superoxide can undergo 'pseudodismutation' to H_2O_2 and O_2 by melanin or reaction with NO. Peroxide is scavenged by melanin, and is not detected in significant amounts. Supported in Part by MBRS Grant #GM 08248, RCMI Grant #RR 03034 and DOD Grant # W911NF – 10 – 1 – 0448.

C55

The immune response influences melanocyte proliferation after ultraviolet radiation exposure

H. Y. Handoko, M. Rodero, G. Walker, Ki. Khosrotehrani
Queensland Institute of Medical Research, Brisbane, Australia

Sunburn results from the release of many inflammatory molecules within the skin. We have found that melanoma initiation in mice by using a single burning dose of ultraviolet B radiation (UVR) is accompanied by a strong melanocyte (MC) proliferative response. Recently, it has been shown that an important driver of this MC response is interferon-γ (IFNg) released from inflammatory CCR2-positive macrophages that infiltrate the skin after UVR. Defects in the neonatal inflammatory response – leading to an environment tolerant for the survival of aberrant cells – is thought to play a role in murine melanoma initiation by UVR. However, neither the nature of this response, nor the source and homing path of the critical monocytes, is well characterized. Neonatal mice were exposed to a single burning dose of UVB and sacrificed at multiple time points subsequently. Skin sections were either fixed and stained with MC-specific various antibodies, or digested for flow cytometry analysis using a panel of antibodies that differentiate the various myeloid lineages. After neonatal UVR, MCs significantly increased in number in the interfollicular epidermis and the infundibulum – bulge region of hair follicles. UVR induced a complex inflammatory infiltrate in the skin, including neutrophils defined as CD11b-high/Ly6G-high. However, the infiltrate was mostly composed of monocytes/macrophages, with specific subpopulations activated at different times. The D1 infiltrate was dominated by inflammatory Ly6C-hi/MHC class II-lo cells that later disappeared in favour of a Ly6C-lo/MHC-hi cells that peaked at D4 after UVR and disappeared by D7. Importantly, depletion of infiltrating macrophages using clodronate-filled liposomes significantly reduced MC activation after UVR. The liposomes depleted mostly Ly6C-lo/MHC-hi cells, suggesting that they are responsible for MC activation. Of interest, UVR in IFNg-null pups resulted in MC response comparable to wild type. Our results indicate a prominent role for a specific population of MHC class II-hi/Ly6C-lo monocytes in inducing a MC proliferation after neonatal UVR. Most melanomas do not seem to fit a classical UVR carcinogenic mechanism of repeated DNA damage due to chronic sun exposure and are more associated with intermittent sunburns. Further dissection of the inflammatory response to UVB might offer clues to new paradigms of melanomagenesis.

CS13: Neuroendocrinology of pigmentation and MC1R

C56

Ultraviolet radiation A and B regulate the neuroendocrine stress response system in melanocyte/keratinocyte co-cultures

C. Skobowiat, J. C. Dowdy, R. M. Sayre, R. C. Tuckey, A. Slominski
University of Tennessee Health Science Center, Memphis, TN, USA

Mammalian skin expresses a local equivalent of the hypothalamic-pituitary-adrenal (HPA) axis which modulates internal homeostasis against the external environment. Epidermal cells expresses corticotropin releasing hormone (CRH) and proopiomelanocortin (POMC) which is further processed by proconvertase 1/3 (PC1/3) and 2 (PC2) to β-endorphin (β-END), adrenocorticotropin (ACTH) and melanocyte stimulating hormone (MSH), similar to the central axis. Since ultraviolet (UV) radiation (UVR) is a tissue-specific stressor for the skin, we investigated the steps involved in the induction of the CRH $\rightarrow$ CRH-R1 $\rightarrow$ POMC $\rightarrow$ β-END/ACTH $\rightarrow$ MC2R $\rightarrow$ glucocorticoids (GC) responses to UVR. In our comparative studies we used UVA and UVB wavelengths and different doses of exposure. Experiments were performed with co-cultured keratinocytes/melanocytes, a reliable epidermal equivalent for which the method is well established in our laboratory.

 © 2011 The Authors. Journal Compilation © 2011 John Wiley & Sons A/S

We have also employed real time PCR (RT-PCR), ELISA, Western blotting and immunohistochemistry (IHC) in our analyses. We found enhanced expression of the 'upper arm' of the HPA axis (CRH, POMC-derived β-END and ACTH) after UVA and UVB exposure.

However, the lower arm of the HPA axis (MC2R, CYP11A1, CYP11B1, HSD11B1 and cortisol) was stimulated only by UVB, with no marked effects from UVA radiation. The enhanced production of cortisol after UVB was followed by decreased expression of the GC receptor (GR), unlike for UVA where the nuclei of epidermal cells exhibited a strong immunofluorescent signal to GR. This study shows that UVR can stimulate the 'local equivalent' of the HPA axis in the epidermis of the human skin in a dose and wavelength dependent way. The subsequent decrease in GR expressions may indicate an epidermal mechanism to attenuate the long-term immunosuppressive effects of cortisol by down-regulating the expression of its receptor, in order to restore the biological barrier.

C57

Regulation of human melanocortin 1 receptor (MC1R) signalling by β-arrestins

A. Belen, P. Oliva, C. Olivares, M. Abrisqueta,
C. Jimenez-Cervantes, J. C. Garcia-Borron
Department of Biochemistry and Molecular Biology, School of Medicine, University of Murcia, Murcia, Spain

G protein coupled receptor (GPCR) signalling is typically controlled by the concerted action of dedicated GPCR kinases (GRKs) and members of the β-arrestin (ARRB) family of cytosolic proteins in a process known as desensitization. Current models state that phosphorylation by GRKs of active, agonist-occupied GPCRs triggers ARRB binding thereby uncoupling receptors from G proteins and initiating endocytosis of GPCR-agonist complexes. Two ubiquitous non visual ARRBs have been described (ARRB1 and ARRB2), with several splice variants, and HBL human melanoma cells express three ARRB isoforms: ARRB1 isoform 201 (ARRB1.201), and ARRB2 isoforms 201 (ARRB2.201) and 001 (ARRB2.001). The melanocortin 1 receptor (MC1R) expressed in melanocytes belongs to the GPCR superfamily, and is regulated by GRK2 or GRK6-dependent phosphorylation. However, there are no data on the involvement of ARRB, and the possibility of a differential regulation of MC1R desensitization by ARRB isoforms has not been investigated. We analyzed MC1R-ARRB interactions in basal conditions and following stimulation with agonist by coimmunoprecipitation, gel filtration chromatography and functional assays performed in heterologous cells co-expressing MC1R and ARRB isoforms, or in human melanoma cells. We show the occurrence of agonist-independent and competitive interactions of ARRB1 and ARRB2 with MC1R. Formation of MC1R-ARRB complexes in basal conditions was independent on the phosphorylation of C-terminal Ser/Thr residues in MC1R. The functional effects of ARRB isoforms on agonist-stimulated MC1R function were different. ARRB2 variants stimulated markedly MC1R internalization and inhibited significantly agonist-dependent cAMP production. Upon internalization, the association of MC1R-agonist complexes and ARRB2 was stable and did not result in receptor degradation. Conversely, ARRB1 had no effect on the rate of internalization of MC1R-agonist complexes or on their functional coupling to the cAMP pathway. Our results show that MC1R is a class B GPCR that binds ARRB1 and ARRB2 with comparably high affinity and forms stable complexes with both arrestins under basal conditions. Upon stimulation with agonists, MC1R undergoes internalization and prolonged co-localization with ARRB2 in endocytic vesicles, and is slowly recycled to the cell surface. These data highlight the possibility of a novel mechanism of MC1R functional regulation based on the levels of expression of ARRB isoforms.

C58

Tropisetron, a serotonin antagonist, modulates the inflammatory cell response of human epidermal melanocytes and keratinocytes after exposure of UVB light or TNF-α

A. Kokot, T. A. Luger, M. Böhm
Department of Dermatology, Münster, Germany

Ultraviolet (UV) light has a key role in skin carcinogenesis. Proinflammatory cytokines such as tumor necrosis factor-alpha (TNF-α) mediate some of the inflammatory responses of epidermal cells after UVB treatment. There is increasing evidence for a modulatory role of serotonin (5-hydroxytryptamine, 5-HT)-mediated pathways in the control of inflammatory responses in various organs of the human body. However, the role of antiserotoninergic strategies in the inflammatory UVB response remains largely unexplored. Using tropisetron, a 5-HT-receptor (5-HT-R) antagonist approved as an antiemetic drug, we investigated the effect of this agent on UVB- and TNF-α-mediated induction of proinflammatory mediators such as interleukin (IL)-6, IL-8 and cyclooxygenase-2 (COX-2) in human epidermal melanocytes (NHM) and keratinocytes (NHK). Tropisetron at doses from 10 ng/ml to 10 μg/ml attenuated UVB- and TNF-α-induced IL-6, IL-8 and COX-2 expression in both cell types. Importantly, this effect of tropisetron occurred in absence of exogenous serotonin and the amounts of endogenously produced serotonin by NHK and NHM were below 15 ng/ml. The kinetics of the attenuating effect of tropisetron differed among NHK and NHM. Accordingly, NHK were in general more sensitive than NHM towards the drug. Mechanistically, tropisetron reduced TNF-α-mediated nuclear translocation of p65 NF-κB in NHK but neither affected p38-signaling nor IκBα-degradation. In support of a serotonin-receptor-independent action of the drug, 5-HT3-R the putative tropisetron receptor, was undetectable in both cell types in our hands suggesting alternative signaling pathways, e. g. via the closely related alpha7 nicotinic acetylcholine receptors that have previously been detected in human epidermis. In summary, our results highlight an anti-inflammatory potential of tropisetron in epidermal cells and create a basis for further research into neuroendocrinology of the skin.

C59

Impact of MC1R variants on the antioxidant responses of melanocytes and implications on human skin homeostasis

A. L. Kadekaro[1,2], V. Maresca[1,2], E. Flori[1,2], D. Kovacs[1,2], G. Cardinali[1,2], J. Chen[1,2], S. Chen[1,2], M. Picardo[1,2]
[1]Department of Dermatology, University of Cincinnati College of Medicine, Cincinnati, USA;[2]San Gallicano Institute, Rome, Italy

Substantial evidence implies oxidative stress generated by increased reactive oxygen species (ROS) as an etiological factor for different types of cancer, including melanoma. We have previously demonstrated that melanocytes (MCs) expressing R/R (disruptive allelic variants) of the melanocortin 1 receptor (MC1R) sustained increased ROS and oxidative DNA damage compared to MCs expressing functional receptor. Our goal in this study was to determine the mechanisms by which α-MSH/MC1R contribute to the detoxification of ROS in human skin. Co-cultures of MCs and keratinocytes (KCs) or single culture of MCs expressing mutated MC1R (R160W/D294H) or wild type gene were used to determine the expression and distribution of antioxidant enzymes by

immunofluorescence and Western Blot analysis post UVR challenge. The presence of 8-oxodG, a common form of oxidative DNA damage, was determined as a biomarker of oxidative stress. Our results demonstrated that α-MSH increases the activity and/or expression of key antioxidant and DNA repair enzymes in MCs expressing functional receptor, but not in MCs expressing non-functional MC1R. As a result, the later MCs sustained more oxidative DNA damage. Comparing the stress responses of MCs normally expressing and silenced for p53, we found that the protective effects of α-MSH were dependent on the activation of the tumor suppressor. Besides its role on human pigmentation α-MSH/MC1R mediate important non-pigmentary effects. Our studies revealed that α-MSH/MC1R are involved in ROS detoxification in MCs and possibly in KCs. The protein p53, the major sensor of cellular stress mediated α-MSH responses in MCs. These findings revealed protective mechanisms that constitute a unique adaptive response of skin to UVR.

CS14: Vitiligo: surgical-instrumental clinically-oriented

C60

Surgical management of vitiligo

P. Araujo
Private clinic and Universitary Hospital Belo Horizonte, MG, Brazil

Both non surgical and surgical therapies have been employed in the management of vitiligo.
However, there are no uniformly agreed guidelines or recommendations available for surgical management of vitiligo, based on topography of involvement. According to the literature, succion blister epidermal grafts are recommended for eyelids, for nipples and mammary areolas. The elbows, however, are difficult to treat because of their mobility. We describe cases of excellent response when needling is associated with Excimer laser 308 nm.

C61

To trypsinise, or not to trypsinise, that is the question

D. Ghia, C. Nayak
T. N. Medical College & B. Y. L. Nair Hospital, Mumbai, India

Several tissue grafting techniques are available for treatment of stable vitiligo but very few comparative studies are available. Our objective was to compare efficacy of autologous non-cultured trypsinised melanocyte-keratinocyte transfer versus autologous non-cultured non-trypsinised melanocyte-keratinocyte transfer in stable vitiligo patches. Patients having anatomically comparable multiple patches of vitiligo stable for at least 1 yr were enrolled for the study. After taking written informed consent, a patch was treated with autologous trypsinised melanocyte keratinocyte suspension (Standard technique) and its anatomically comparable counterpart was treated with autologous non-trypsinised melanocyte keratinocyte suspension (Jodhpur technique). Patch was assessed at 1 week for infection and at 1 month, 3 month and finally at 6 month for pigmentation. The results of the surgery of both techniques at the end of 6 months were evaluated by scoring system for autologous transplantation methods for vitiligo by Gupta S. et al. Nine patients with 42 patches of stable vitiligo were treated, of which 21 were treated by Standard technique and the anatomically comparable 21 patches were treated by Jodhpur technique. The 21 patches treated with Standard method were graded as excellent: 6, good: 2, fair: 6, poor: 7 and anatomically comparable 21 patches treated with Jodhpur technique were graded as excellent: 5, good: 2, fair: 1, poor: 13 by the scoring system for autologous transplantation in vitiligo. On statistical evaluation of the individual scores of standard technique (Mean 11.05 ± 7.00) and Jodhpur technique (Mean = 7.5 ± 7.63) the P value = 0.0064 by the paired t test which was statistically significant. On comparison of the two techniques standard technique was found to be more efficacious than the Jodhpur technique.

C62

Comparing the effect on the outcome of cold trypsinisation versus warm trypsinisation in transplantation of autologous non cultured epidermal cell suspension in stable vitiligo – a prospective randomized study

S. Awasthi, A. J. Kanwar, D. Parsad
PGIMER, Chandigarh and Jaipur, India

Vitiligo is a major psychosocial problem which can lead to embarrassment, psychologic turmoil and cosmetic disfigurement in those afflicted. Surgical modalities appear to be the method of choice in recalcitrant stable cases, which comprise approximately 5% of all vitiligo patients. Noncultured autologous epidermal cell suspension transplant is a simple, cheap and effective surgical modality for treating vitiligo; however the outcome of cold trypsinisation versus warm trypsinisation used for preparing the suspension has never been compared. Our objective was to compare the effect on the outcome of cold trypsinisation versus warm trypsinisation in transplantation of autologous non cultured epidermal cell suspension in stable vitiligo. Patients with lesions of stable vitiligo (no new lesions in past 1 yr) were recruited from pigmentary clinic and treated with non cultured melanocyte transplantation. The lesions to be treated were randomly distributed into two groups – Group I (using cold trypsinisation method for preparing non cultured epidermal cell suspension) and Group II (using warm trypsinisation method for preparing non cultured epidermal cell suspension) to compare the results of the transplantation procedure. Patients were asked to follow up at the clinic at day 8, week 4, 8 and 16 after the transplantation procedure. The extent and pattern of repigmentation, colour matching of repigmented skin and DLQI were assessed. Excellent repigmentation (>90%) at 16 weeks post surgery, was seen in 86.3% of lesions in Group I (15 patients and 22 lesions) compared to only 55% of lesions in Group II (15 patients and 20 lesions) (P = 0.025). Greater than 75% repigmentation was seen in 90.9% of patients in Group I compared to 80% in Group II. There was also a significant (P = 0.0001) decline in DLQI in both the groups, but difference amongst the two groups was not significant. Colour match was excellent in 81.8 and 75% of lesions in Groups I and II respectively. The most common pattern of pigmentation was the diffuse pattern. In addition, a significant improvement in leukotrichia was noticed in all lesions showing depigmented hair. Adverse effects were minimal and not of much concern to the patients. Results of non cultured epidermal cell transplantation are significantly improved by using cold trypsinisation method for preparing non-cultured epidermal cell suspension compared to warm trypsinisation method.

 © 2011 The Authors. Journal Compilation © 2011 John Wiley & Sons A/S

C63

A comparative study of efficacy and safety of modified dermabrasion followed by NB-UVB therapy with suction blister epidermal grafting followed by NB-UVB therapy in stable vitiligo patients

G. Garg, U. S. Khopkar
Departement of Dermatology, Seth GS Medical College, Indian Association of Dermatology Venerology and Leprology, Mumbai, India

Advance technologies for surgical correction of residual vitiligo lesions, are not available or expensive at most of the treatment centers making them inaccessible to most patients. Hence any attempt at innovation of current techniques without the help of sophisticated technologies is welcomed. Such a technique is modified dermabrasion (MD) in which we apply dermabraded material collected from normal skin over dermabraded vitiligo area. Our objectives were (i) to evaluate the efficacy and safety of modified dermabrasion (MD) followed by NB-UVB therapy with suction blister epidermal grafting (SBEG) followed by NB-UVB therapy in stable vitiligo patients; (ii) Time duration required for repigmentation. After proper written consent, thirty five stable vitiligo patients fulfilling inclusion and exclusion criteria were included in the study. In MD and SBEG, 17 and 18 patients were included respectively. Vitiligo area was calculated with the help of transparent sheet and graph paper. Vitiligo patches were graded according to degree of depigmentation. Patients were evaluated for extent of pigmentation and side effects monthly for 6 months post-operatively. In MD, donor to recipient ratio was 1:3 while in SBEG it was 1:1. After 6 months of follow up, 45% patches in SBEG and 30% in MD showed >90% repigmentation. However, 70% patches in MD and 77% patches in SBEG showed >50% repigmentation. Color matching was good to excellent in 85 and 82% patches of MD and SBEG respectively. Repigmentation was early in SBEG (after 4 months, 45% patches of MD and 64% patches of SBEG showed 50% repigmentation). MD is easier to perform and less time consuming than SBEG. Also it requires less donor area than SBEG. However, repigmentation by MD takes longer time in comparison to SBEG. Hyperpigmentation and peripheral halo are not the side effects in MD.

CS15: Non Mouse animal models and in vitro human models

C64

Mitf mutations promote differentiated cell division and melanoma in zebrafish

K. Taylor, J. Richardson, R. Kelsh, I. Jackson, J. Lister, E. E. Patton
Institute for Genetics and Molecular Medicine, MRC Human Genetics Unit, Edinburgh, UK

The coordination of cell proliferation with differentiation is important for both developmental and cancer biology. In development and in the hair follicle, pigmented melanocytes are thought to be derived from undifferentiated precursor cells or stem cells. However, differentiated melanocytes may also have proliferative capacity in animals, and the potential for differentiated melanocyte cell division in development and regeneration remains largely unexplored. Melanomas often consist of differentiated cells, but little is known about the origin of melanoma cells or how melanoma is maintained. We have used time-lapse imaging of the developing zebrafish to show that while most melanocytes arise from undifferentiated precursor cells, an unexpected subpopulation of differentiated melanocytes arises via cell division. Chemical or genetic depletion of the melanocyte population triggers a regeneration phase in which pigmented melanocyte cell division is significantly enhanced, particularly in young differentiated melanocytes.

This process is regulated by Mitf because we find a mitf hypomorphic mutation causes successive differentiated cell divisions in the developing embryo. These results reveal a novel pathway of differentiated melanocyte division that acts in parallel to de novo melanocyte development to rapidly re-establish skin pigmentation in zebrafish. The division potential of differentiated melanocytes may be relevant for stem-cell and differentiation based therapy in melanoma, and in melanocyte regeneration in vitiligo. Mitf mutations have been identified in melanoma and we are now addressing the role of the mutations and differentiated cell division in a zebrafish model of BRAFV600E mitf melanoma.

C65

Canine melanoma: promising spontaneous models for genetics and therapies of human melanoma

M. Gillard, C. De Brito, J. Abadie, B. Vergier, A.-S. Guillory, E. Cadieu, P. Devauchelle, F. Galibert, B. Hédan, C. André
CNRS-UMR6061, Institut de Génétique et Développement, Universite de Rennes 1, France

Melanoma spontaneously affect dogs on the same localizations than human: cutaneous, oral and ocular, with striking breed predispositions and we thus propose that dog breeds with high risk of melanoma constitute unique models to unravel the genetics of melanoma and prove useful for clinical trials. Indeed, melanoma diagnosed in several dog breeds, present clinical, histological and treatment response similarities with human melanoma. In addition, the dog population is structured in more than 350 breeds, which are rather heterogeneous between them, but strongly homogenous within breeds, thus resembling human isolated groups. Canine breeds spontaneously develop melanoma in a breed and tissue specific manner, with high risks (example: 3–5% of poodles develop melanoma, 99% of them of oral localization). Moreover, dog and human share similar environmental exposures and the relationship between melanoma and phototype can be largely explored with the variety of coat colours! Due to a strong homology in the physiopathology and the response to treatments between dogs and humans, the discovery of new canine genes is expected to be transferred to human in a very promising way. These characteristics make the canine model ideal to identify predisposing and tumor progression genes in melanoma and shows an interesting potential for clinical trials. Our work consists in: (i) characterisation of the homology between dog and human melanoma types; (ii) case/control genetic analyses to identify predisposing genes in high risk breeds; and (iii) CGH and RNA sequencing analyses to identify tumour progression alterations (1). A total of 150 clinical questionnaires of all localisation have been collected and analysed in order to characterize epidemiology and clinical data of dog melanoma compared to human. An histological confrontation between dog and human cases have been performed on 150 dog melanoma cases defining a canine classification and homologies to human. Moreover cDNA sequencing of a set of genes in dogs frequently altered in human melanoma has been undertook (Braf, Nras, Ckit, CDKN2A, Pten, MC1R and CDK4) (2). We have selected the poodle to undergo a Whole Genome Association Study with 100 cases and 100 healthy controls to search for loci predisposing to oral melanoma (3). In the same time, tissue samples of tumour of different location and healthy tissue samples (100 tumour/control pairs have been collected so far) will be used for RNA sequencing and CGH analyses of oral and cutaneous dog melanoma. All together, these genetic analyses are thought to

© 2011 The Authors. Journal Compilation © 2011 John Wiley & Sons A/S

identify identical or novel genes and/or pathways in the different melanoma types to be able to transfer to human for a better understanding of the physiopathology and genetics of these tumours, and anticipate clinical trials on dogs for the most human homologous melanoma types.

C66

Do epidermal melanocytes contribute to the erythema response in human skin post-UVB irradiation?

C. Talari, K. Gledhill, D. J. Tobin
Centre for Skin Sciences, School of Life Sciences, University of Bradford, UK

Ultraviolet B (UVB)-induced erythema is mediated in-part by prostaglandin E2 (PGE2) (Rhodes et al., 2001). In UVB-irradiated skin the sole source of this pro-inflammatory mediator was thought to be the keratinocyte (KC), however, we have recently shown that irradiated epidermal melanocytes (EM) may also make and release PGE2 (Gledhill et al., 2010). Consequently, EM may have the capacity to contribute to erythema. It is therefore important to examine the production of PGE2 by EM and KC in response to UVB, so that the potential contribution of each cell type to the erythema response can be spatio-temporally delineated. Here we assessed the production of PGE2 by ELISA in primary EM and KC derived from healthy individuals under UVB-stimulated (73 mJ/cm^2) conditions. We also investigated by RT-PCR, qPCR and Western blotting the likely route for PGE2 production by examining the expression of the cyclooxygenase (COX) and prostaglandin E synthase (PGES) enzymes. Results suggested differential production of PGE2 by primary mono-cultures of EM and KC in response to UVB. Interestingly, EM responded to UVB by increasing COX-2 mRNA expression ≈80 fold at 1hr post-irradiation (returning to basal levels by ≈6 h post-UVB) whereas matched KC responded by increasing COX-2 mRNA expression ≈80 fold at 3 h post-irradiation (returning to basal levels by ≈12 h post-UVB). However, in response to UVB a statistically-significant increase in PGE2 production was observed at 1 h post-stimulation in both primary cultures. These results may suggest that PGE2 production immediately following UVB exposure is governed by levels of pre-existing COX-1 in EM and KC. Moreover, EM-derived PGE2 may be of more consequence to erythema following subsequent UVB exposures. These results may highlight EM as a novel therapeutic target that could modify the human skin response to UVB in cases of sunburn.

C67

Engineering melanoma progression in a humanized environment in vivo

T. Biedermann, G. Kiowski, D. S. Widmer, G. Civenni, C. Burger, R. Dummer, M. Meuli, L. Sommer, E. Reichmann
Tissue Biology Research Unit, Childrens Hospital Zurich, Switzerland

To overcome the lack of effective therapeutics for aggressive melanoma, new research models closely resembling the human disease are required. Here we report the development of a fully orthotopic, humanized in vivo model for melanoma exactly recapitulating human disease initiation and progression. Human melanoma cells were seeded together with human keratinocytes onto collagen type I hydrogels, previously populated with human dermal fibroblasts. The engineered human dermo-epidermal skin substitutes were transplanted onto the back of immuno-compromized rats. The transplantations consistently resulted in the development of melanoma displaying the hallmarks of their parental tumors. Importantly, all initial steps of disease progression were recapitulated, including the incorporation of tumor cells into their physiological microenvironment, transition of radial to vertical growth, and the establishment of highly vascularized, aggressive tumors with dermal involvement. Because all cellular components can be individually accessed using this approach, it allows manipulation of tumor cells as well as of keratinocyte and stromal cell populations. This is a model of tissue engineered human skin that allows melanoma formation and investigation of local tumor progression as well as of those mechanisms leading to metastasis.

Plenary session V. Fundamental aspects of the initiation and progression of melanoma (1)

C68

Novel role of melanocytic RXR alpha/RXR beta in UV irradiation induced melanocyte homeostasis

D. J. Coleman, S. Hyter, H. S. Jang, X. Liang, L. Larue, G. Indra, A. K. Indra
Department of Pharmaceutical Sc, OSU/OHSU, Department of Dermatology, OHSU, Corvallis, OR, USA

Retinoid-X-Receptors (RXRs) α, β, and γ are members of the nuclear hormone receptor (NR) superfamily, and act as central coordinators of cell signal transduction through heterodimerization with several other NRs. In malignant human melanoma samples, loss of RXRα expression has been previously observed both in the melanoma cells themselves (Chakravarti et al., 2007) and in adjacent epidermal keratinocytes (Hyter et al., 2010). Previously, epidermal-specific ablation of RXRα in a mouse model has been shown to promote increased melanocyte proliferation after UV radiation (Wang et al., 2010) and increased susceptibility to malignant melanomas (Hyter et al., 2010). We recently found that Cre-LoxP mediated selective ablation of RXRα and RXRβ specifically in melanocytes of skin results in an increase in apoptosis in non-melanocytic cells in dermal layer following UV irradiation. UV induced interferon-y production, preferably by macrophages, controls melanocyte activation and has protective effects on survival of melanoma cells in vivo (Zaidi et al., 2010). Interestingly, expression of interferon-γ was found to be downregulated in dermal layer following UV radiation, suggesting possible defects in activation/recruitment of macrophages and other inflammatory cells. Additionally, melanocytic ablation of RXRα/RXRβ resulted in a decreased percentage of apoptotic melanocytes following UV radiation, suggesting an increased survival of cells following DNA damage. That reduced apoptosis can contribute to increased incorporation of mutation into the melanocytes and increased susceptibility of UV induced melanoma formation in the long-term.

Chakravarti, N., Lotan, R., Diwan, A.H., Warneke, C.L., Johnson, M.M., Prieto, V.G. (2007). Clin Cancer Res. 13, 4817–24.
Hyter, S., Bajaj, G., Liang, X, Barbacid, M., Indra G., Indra, A.K. (2010). Pigment Cell and Melanoma Research. Oct: 23 (5), 635–648.
Wang, Z., Coleman, D.J., Bajaj, G., Liang X., Ganguli-Indra, G., Indra, A.K. (2011). JID (NPG). Jan;131(1), 177–87. Epub 2010 Oct 14.
Zaidi, M.R., Davis, S., Noonan, F.P., Graff-Cherry, C., Hawley, T.S., Walker, R.L., Feigenbaum, L., Fuchs, E., Lyakh, L., Young, H.A., Hornyak, T.J., Arnheiter, H., Trinchieri, G., Meltzer, P.S., De Fabo, E.C., Merlino, G. (2011) Nature 469(7331), 548–53.

© 2011 The Authors. Journal Compilation © 2011 John Wiley & Sons A/S

C69

Two UV pathways to melanoma

F. P. Noonan, M. R. Zaidi, A. Wolnicka-Glubisz, M. R. Anver, J. Bahn, A. Wielgus, J. Cadet, T. Douki, S. Mouret, A. Popratiloff, G. Merlino, E. C. De Fabo
The George Washington University, Washington, DC, USA

The role(s) of UVB (280–320 nm) and UVA (320–400 nm) in melanoma are unclear but can be addressed in experimental animal models. The hepatocyte growth factor transgenic (HGF/SF) mouse has extrafollicular ectopic melanocytes which are absent in wild-type mice and develops melanomas recapitulating human disease after neonatal UV exposure. We used specialized optical sources to deliver precisely defined UVA or UVB radiation to albino and pigmented HGF/SF transgenic mice and compared melanoma formation. Unexpectedly, the presence of melanin pigment exacerbated melanoma. In pigmented HGF/SF transgenics, melanin was largely confined to melanocytes and protective epidermal melanin was sparse, enabling direct exposure of melanocytes to UV radiation. UVB initiated melanoma and produced cyclobutane pyrimidine dimers (CPDs) and pyrimidine (6-4) pyrimidone photoproducts (6-4 PPs) quantified by HPLC-MS/MS in neonatal skin independent of pigment status. UVA initiation of CMM, however, was completely dependent on the presence of melanin pigment. UVA irradiation produced only TT-CPDs but at 10-fold lower levels than UVB and melanin did not increase their formation. UVA-induced oxidative DNA damage, however, quantified as nuclear 8-oxo-7,8-dihydro-2′-deoxyguanosine (8-oxodGuo), was found at significantly higher levels in melanocytes in the presence of melanin, both in vivo and in vitro, supporting a photooxidative role for melanin and/or its precursors in UVA melanomagenesis. Our findings have identified two wavelength-dependent pathways to UV-induced melanoma and demonstrate a novel and important role for melanin/melanin precursors in melanoma. These studies also provide experimental support for the epidemiologic associations between solar UV exposure, or use of UVA-emitting tanning lamps and increased melanoma risk.

C70

The Mitf structure unravels DNA binding and dimerization specificities

V. Pogenberg, M. H. Ogmundsdottir, K. Bergsteinsdottir, M. Milewski, V. Deineko, B. Phung, A. Schepsky, M. Wilmanns, E. Steingrimsson
Department of Biochemistry and Molecular Biology, Faculty of Medicine, Reykjavik, Iceland

The microphthalmia-associated transcription factor (Mitf) is essential for normal melanocyte development and has also been shown to play an important role in melanoma where it acts as a lineage survival oncogene. Mitf plays multiple roles in melanocytes and melanoma cells by regulating the expression of genes involved in various different processes including survival, proliferation, migration and differentiation. It is, however, not clear how it performs these different tasks in the same cell type, although signalling and DNA-binding specificity have been proposed to play an important role. We have analysed the structure of the MITF protein using X-ray crystallography and classical DNA binding studies of both wild type and mutant MITF proteins. The structures reveal how MITF discriminates between its target sequences. In addition, they show an unusual mode of dimerization that may explain how MITF selects its dimerization partner. This has provided new insights into the mechanisms of Mitf-mediated transcription regulation.

C71

Hypoxia and MITF control metastatic behaviour in mouse and human melanoma cells

Y. Cheli, S. Giuliano, N. Fenouille, M. Allegra, V. Hofman, P. Hofman, P. Bahadoran, J.-P. Lacour, S. Tartare-Deckert, C. Bertolotto, R. Ballotti
Université de Nice-Sophia Antipolis – INSERM U 597 Nice, France

Melanomas are very aggressive neoplasms with notorious resistance to therapeutics. It was recently proposed that the remarkable phenotypic plasticity of melanoma cells allows for the rapid development of both resistance to chemotherapeutic drugs and invasive properties. Indeed, the capacity of melanoma cells to form distant metastases is the main cause of mortality in melanoma patients. Therefore, the identification of the mechanism controlling melanoma phenotype is of paramount importance. We previously identified MITF, the master regulator of melanocyte differentiation, and p27, a CDK inhibitor, as the key molecular switches that control the transition between melanoma-initiating cells and their differentiated progeny. In the present report, we show that deletion of MITF, the master gene in melanocyte differentiation, is sufficient to increase the metastatic potential of mouse and human melanoma cells. MITF silencing also increases fibronectin and Snail, two mesenchymal markers that might explain the increased invasiveness in vitro and in vivo. Furthermore, ablation of this population by forskolin-induced differentiation or MITF forced expression dramatically decreases tumour formation, indicating that eradication of low-MITF cells is an appealing strategy to cure melanoma. Moreover, we demonstrate that a hypoxic micro-environment decreases MITF expression through an indirect, HIF1α dependent transcriptional mechanism and increases the tumorigenic and metastatic properties of melanoma cells. Our results reveal a hypoxia-HIF1α-MITF cascade controlling the phenotypic plasticity in melanoma cells and favouring metastasis development. Targeting this pathway might be helpful in the design of new anti-melanoma therapies.

Plenary session VI Fundamental aspects of the initiation and progression of melanoma (2)

C72

When CRAF takes over from BRAF in melanoma using ERK and PDE4

A. Marquette, J. Andrée, M. Bagot, A. Bensussan, N. Dumaz
INSERM UMR-S976 Paris, France

In melanocytes, the MAPK pathway is activated through BRAF because CRAF is inhibited by the cAMP pathway in these cells. On the contrary, in melanoma harboring Ras mutations the MAPK pathway is activated through CRAF and the cAMP pathway is inhibited. We now provide insight into the molecular mechanism of this Raf isoform switching and cAMP pathway disruption, which happens during melanocyte transformation. We show that mutant Ras drives ERK-mediated phosphorylation of BRAF on Ser151. This phosphorylation inhibits BRAF activity by blocking its ability to interact with RAS. Consequently, in mutant RAS melanoma cells, MAPK is activated through CRAF. Moreover, cAMP phosphodiesterase activity is elevated inhibiting cAMP signaling to allow CRAF reactivation in melanoma cells. Using PDE inhibitors and RNA interference in melanoma cells harboring mutant RAS, we identified PDE4B and PDE4D as the main suppressors of cAMP signaling. Inhibition of PDE4 during α-MSH stimulation resulted in reactivation of the cAMP pathway. Interestingly, we show that small hairpin RNA–mediated inhibition of

© 2011 The Authors. Journal Compilation © 2011 John Wiley & Sons A/S

PDE4 is sufficient to inhibit transformation of normal melanocytes by oncogenic Ras. Moreover, inhibition of PDE4 by inhibitors or RNA interference can induce cell death in melanoma cells, but not in melanocytes, highlighting a novel therapeutic approach to treat melanoma-harboring Ras mutations.

C73

A novel link between TGFβ and MAP kinase signalling is involved in resistance to MEK inhibition in melanoma

M. Smith, J. Ferguson, I. Arozarena, C. Wellbrock
University of Manchester, UK

The MAP-kinase pathway consisting of RAF, MEK and ERK is deregulated in over 90% of melanoma skin cancers. Last year a novel inhibitor (PLX4032) targeting the oncogenic RAF kinase V600E-BRAF in melanoma patients demonstrated an unprecedented 80% anti-tumour response. However, acquired resistance occurs rapidly through various and partially unknown mechanisms. Moreover due to the complex molecular mechanisms of RAF-kinase regulation, BRAF selective inhibitors can cause tumour-promoting effects in non-mutant BRAF patients. Therefore, targeting BRAF downstream effectors i.e. the kinase MEK can provide a more potent and specific way of treating melanoma. In addition, this also allows treating patients with NRAS mutations, which are found in up to 20% of melanomas and lead to MEK activation. Potent inhibitors of MEK are currently tested in the clinic and show promising results. However, recent findings demonstrate the existence of mechanisms that can also provide resistance against MEK inhibition. In summary, there is need for a better understanding of the mechanisms underlying resistance to MAP-kinase pathway inhibition. With this knowledge we can identify other 'druggable' proteins that can be targeted in combination with MAP-kinase pathway inhibitors. We have discovered a novel mechanism of resistance to MAP-kinase pathway inhibition involving a link between the BRAF/MEK/ERK cascade and TGFβ signalling. The analysis of the regulatory link between TGFβ and MAPkinase signalling revealed the involvement of the SMAD specific E3-ligase SMURF1. Exploiting the cross-talk between SMURF1 and the MAP-kinase-kinase MEK, we have identified a synthetic lethal approach that efficiently kills melanoma cells, thereby overcoming resistance. Acquired resistance to mono-therapy targeting the MAP-kinase pathway in melanoma is an increasingly apparent dilemma. However, our findings open new avenues for the treatment of melanoma based on combining agents with synergistic activity on the MAP-kinase pathway in order to prevent the development of resistance.

CS16: Preclinical and clinical advances in melanoma management (SMR-IFPCS)

C74

Synthetic lethal RNAi-screening uncovers a novel role for rho family GTPases in controlling cell fate and chemoresistance

J. Aruri, R. Kapadia, H. Mehr, H. Ho, M. A. White, A. K. Ganesan
Department of Dermatology, Irvine, CA, USA

Melanoma is resistant to conventional chemotherapy, immunotherapy, and molecularly-directed therapies via unknown mechanisms. In this study, we utilized a systems-level functional genomics approach to identify novel pathways that modulate melanoma chemoresponsiveness. In addition to isolating fragile nodes in the DNA repair network, this analysis identified two Rho family GTPases (RhoJ, Rnd2) as pathological suppressors of melanoma cell sensitivity to DNA damage. Mechanistic studies revealed that RhoJ supports melanoma migration and invasion, and is necessary and sufficient to drive the expression of Sox10, a central regulator of melanocyte lineage specification. Sox10 is overexpressed in melanoma and required for melanoma cell survival. These observations uncover a novel role for Rho family G-proteins in cell fate specification and implicate melanocytic lineage specification as an intrinsic chemoresistance determinant.

C75

Functional role for the UDP-glucuronosyltransferases (UGTs) in melanoma drug resistance

R. W. Dellinger, H. H. Matundan, F. L. Meyskens
University of California, Irvine, Orange, CA, USA

The UGT family of enzymes catalyzes the glucuronidation and clearance of several anti-cancer agents. UGTs are expressed primarily in the liver, but are also expressed in several extra-hepatic tissues including prostate, breast, colon, lung, brain, kidney, bladder and ovary. However, there have been no reports of UGTs in melanocytes or melanoma. Our objective was to ascertain the role of UGTs in melanocytes and melanoma. We screened several primary melanocyte cultures as well as several melanoma cell lines for UGT expression by RT-PCR. TaqMan Real time PCR assays were used to determine UGT expression in melanoma cells before and after drug treatment. MTT assays were employed to determine IC50 values and UGT-Glo assay was used to measure UGT activity. Three UGT family members, UGT2B7, UGT2B10 and UGT2B15, were found to be expressed in melanocytes. Interestingly, of all the melanoma cell lines screened only WM115, a primary melanoma, had UGT expression. Again, only UGT2B7, UGT2B10 and UGT2B15 were detected. UGT expression was not detected in another primary melanoma cell line, WM3211, or in any metastatic melanoma cell line assayed indicating that loss of UGT expression occurs during melanoma progression. Treatment of melanoma cells lacking UGT expression (WM3211, SKmel28 and A375) with an anti-cancer agent (temozolomide, adriamycin, or epirubicin) induced expression of UGT2B7, UGT2B10 and UGT2B15 in these cells. Furthermore, UGT activity assays show increased glucuronidation in melanoma cells following treatment with an anti-cancer agent. Finally, knockdown of UGT2B7 in WM115 cells sensitized these cells to epirubicin and adriamycin treatment, but not temozolomide treatment. UGTs are expressed in melanocytes and their expression can be induced in melanoma cells. Since the UGTs are Phase II drug metabolism enzymes, we hypothesize that the upregulation of the UGTs in melanoma cells in response to anti-cancer agents is a potential mechanism for the well documented drug resistance observed in melanoma. Our observed results that knockdown of UGT2B7 sensitizes melanoma to epirubicin and adriamycin, but not temozolomide treatment supports our hypothesis since both epirubicin and adriamycin are substrates for UGT2B7 while temozolomide is not. We conclude that future anti-cancer drug strategies for melanoma will have to account for tumor cell mediated glucuronidation.

 © 2011 The Authors. Journal Compilation © 2011 John Wiley & Sons A/S

C76

Introduction of melanoma in situ peptide vaccine by chemothermotherapy through exploitation of melanogenesis substrate, NPrCAP, and its conjugation with magnetite nanoparticles

K. Jimbow, A. Yoneda, Y. Tamura, Y. Osai, M. Sato, A. Sato, T. Kamiya, J. Kato, A. Takada, T. Yamashita, A. Miyamoto, A. Ito, H. Honda, K. Wakamatsu, S. Ito, K. Murase, S. Nohara, E. Nakayama, T. Kobayashi
Institute of Dermatology & Cutaneous sciences, Sapporo Medical University, Sapporo, Japan

Our goal is to develop melanoma-targeted vaccine therapy using peptides introduced in the in situ melanoma lesions by chemothermotherapy. Melanogenesis substrates were exploited and N-propionyl cysteaminylphenol (NPrCAP) was synthesized to develop a unique melanoma-targeted therapeutic drug because of its selective incorporation into melanoma cells and production of highly reactive free radicals, which result in apoptotic cell death and melanoma antigen production, possibly through generation of heat shock protein (HSP) upon exposure to tyrosinase. Magnetite nanoparticles were conjugated with NPrCAP (NPrCAP/M for animals and NPrCAP/PEG/M for humans) to introduce thermotherapy because of non-apoptotic cell death and HSP generation upon exposure to alternating magnetic field (AMF). We examined the feasibility of this 'in situ chemothermotherapy introduced peptide (vaccine) for melanoma immunotherapy' by experimental study using in vivo and in vitro B16 melanoma cells and preliminary clinical study to advanced melanoma patients. We found (i) that a tumor-specific drug delivery and cell death were achieved by NPrCAP itself and exposure to AMF, (ii) that NPrCAP/M with AMF (and without AMF to lesser extent) not only inhibited the growth of primary transplant, but also prevented the growth of the secondary, re-challenge transplant and increased life span of the host mice, (iii) that HSP70 production at the site of primary transplant and CD8+T cell infiltration at MHC I expressing sites of the re-challenge melanoma transplant were seen, (iv) that prevention of re-challenge transplant was not seen in those mice with pre-administration of anti-CD8 antibody and finally (v) that our preliminary clinical trial to stage III/IV patients showed PR and CR. CD8+ T cells were seen predominantly at NPrCAP/PEG/M sites. Our study is the first report indicating that successful utililization of melanogenesis substrate, NPrCAP, an amine derivative of sulfur homologue of tyrosine, which is the good substrate of tyrosinase, can be exploited for the development of chemothermotherapy-introduced in situ vaccine therapy, which is least toxic and safely and effectively applicable to advanced-stage melanoma patients.

C77

cKIT expression level and NRAS/BRAF mutation status predict the response to the tyrosine kinase inhibitor dasatinib in melanoma cell lines

F. Journe, M. Wiedig, R. Morandini, F. Sales, A. Awada, G. Ghanem
LOCE, Institut Bordet, Université Libre de Bruxelles, Brussels, Belgium

Patients with advanced melanoma have limited effective therapy. New targeted drugs have to be evaluated in melanoma lines with regards to mutation status and active signalling pathways. We evaluated the cytotoxic effects of the SRC tyrosine kinase inhibitor dasatinib (BMS-354825) in a panel of melanoma cell lines, we examined the molecular targets of the drug in sensitive cells, and we proposed markers to select melanoma patients who would benefit from dasatinib therapy. We assessed the effects of dasatinib on melanoma cell proliferation (crystal violet staining) and apoptosis (annexin-V-PE detection) in relation with cKIT, NRAS and BRAF mutation status (PCR/sequencing) and key proteins involved in signalling pathways (Western blotting). We examined 33 melanoma cell lines and found that eight lines were highly sensitive to dasatinib (IC50 < 10^{-9} M), 13 were moderately sensitive (IC50 from 10^{-8} to 10^{-6} M) and 12 were resistant (IC50 > 10^{-5} M). All highly sensitive lines expressed high cKIT levels, whereas others had undetectable cKIT expression. Then, we checked for the most common mutation in cKIT (L576P), NRAS (Q61L/R) and BRAF (V600E/K), and showed that the highly sensitive cells did not bear any of these mutations, while 60% of the moderately sensitive and 75% of the resistant lines had NRAS or BRAF mutations. We also found that, in the sensitive cells, dasatinib induced apoptosis in time and concentration-dependent manner. Interestingly, we showed that the combination of a cKIT inhibitor (10^{-10} M sunitinib) and a SRC inhibitor (10^{-8} M PP2) resulted in a synergistic effect, suggesting that dasatinib is most effective at low concentrations by targeting cKIT and SRC simultaneously. To confirm this, we showed that, in the sensitive cells, 10^{-12} M dasatinib completely inhibited the phosphorylation of cKIT, SRC, ERK and AKT, while in the resistant cells, at least 10^{-4} M concentration was needed to inhibit ERK phosphorylation but without any effect on AKT phosphorylation. Dasatinib appears as a promising agent for the treatment of a selected group of melanoma patients with tumor tissues harbouring cKIT overexpression and no mutations of cKIT, NRAS or BRAF. By targeting wildtype BRAF tumors, dasatinib use might be complementary to the recent unprecedented encouraging therapeutic responses obtained with RG7204 (PLX4032), a specific inhibitor of V600EB-RAF activating mutation.

C78

Promotion of melanoma growth and survival through Glycogen Synthase Kinase-3 protein activity

D. Lang, J. B. Mascarenhas, D. Wolfgeher, J. D. Kubic
University of Chicago, USA

Glycogen Synthase Kinase-3 (GSK-3) is a serine/threonine kinase involved in a diverse range of cellular processes. GSK-3 exists in two isoforms, GSK-3 α and GSK-3 β, which possess some functional redundancy but also play distinct roles depending on developmental and cellular context. Improper activity and expression of GSK-3 has been linked to the survival and proliferation of several cancer types including melanoma through an unknown mechanism. In this report, we demonstrate that GSK-3 α and GSK-3 β are phosphorylated at Ser 21 and Ser 9 in many melanoma cell lines, respectively. Phosphorylation of these epitopes is known to inhibit some, but not all, kinase functions of GSK-3 proteins. In melanoma cells, GSK-3 actively promotes cell growth and survival, and blocking this activity, with small molecule inhibitor SB216763 or gene-specific siRNA, results in a decrease in proliferation, an increase in apoptosis and cellular morphological changes. These alterations coincide with a loss of PAX3, a transcription factor implicated in proliferation, survival and migration of developing melanoblasts and melanoma. While knockdown of either GSK-3 α and GSK-3 β leads to a moderate phenotype, only inhibition of both isoforms causes significant phenotypic consequences and a decline in PAX3 protein levels, suggesting possible functional redundancy. We further show that GSK-3 β directly interacts with and phosphorylates PAX3 at Ser205 and either Ser197 or Ser201 in vitro. These data support a model wherein GSK-3 regulates proliferation and survival of melanoma through phosphorylation and stabilization of PAX3.

© 2011 The Authors. Journal Compilation © 2011 John Wiley & Sons A/S

CS17: New pathomechanisms in melanoma

C79

Fighting Proliferation with Differentiation: How Pmel17/gp100 binding to FHL2 leads the charge in melanoma

J. C. Valencia, S. G. Coelho, L. Yin, W. D. Vieira, V. J. Hearing
PCBS, LCB, CCR, NCI, Bethesda, USA

During tumor progression and as a result of several mechanisms, melanoma cells proliferate in an uncontrolled manner and irreversibly lose expression of their differentiation antigens, including Pmel17/gp100 (hereafter Pmel17). Besides the classic role of Pmel17 in determining the fibrillar structure of melanosomes, several non-structural properties are also attributed to Pmel17. Along this line, clinical studies have shown that overexpression of Pmel17 induces a transitory tumor regression without improving immune recognition of tumor cells.

Objective: Evaluate whether restoration of Pmel17 expression restrains cell proliferation in advanced melanomas. We used yeast-two hybrid assays, specific gene expression profiling, proteomics, microarray analysis, and in vivo (nude mice) and ex vivo (human reconstructed skin models; hRSM) tumor progression models. Restoration of Pmel17 function significantly reduced proliferation and invasion of A375 melanoma cells compared to untreated controls. Such an event was followed by the stabilized expression of the adaptor protein four and half LIM domain-2 (FHL2), a protein known to form multimeric complexes. Using yeast-two hybrid assays, we found that Pmel17 binds FHL2, and co-immunoprecipitation assays confirmed that interaction. Confocal microscopic analysis showed the colocalization of Pmel17 and FHL2 and the decreased presence of FHL2 in focal adhesion points after ectopic expression of Pmel17. Indeed, our earlier proteomic analysis had detected Pmel17 and FHL2 in premelanosomes of pigmented melanoma cells. In the absence of Pmel17, we confirmed that gene silencing of FHL2 by two short-hairpin RNA significantly reduced melanoma tumor formation in in vivo and ex vivo models compared to controls. Thus, when taken together, these results support the view that melanoma cell proliferation in the absence of Pmel17 expression requires FHL2. We conclude that one of the non-structural properties of Pmel17 is to exert its novel anti-tumor effect through its binding to FHL2 in melanoma cells.

C80

Senescent cells develop a secretome

M. Ohanna, R. Ballotti, C. Bertolotto
INSERM U895, Centre Méditerranéen de Médecine Moléculaire (C3M), Nice, France

Melanoma cells can enter the process of senescence but whether they express a secretory phenotype, as reported for other cells, is undetermined. This is of paramount importance, because this secretome can alter the tumor microenvironment and the response to chemotherapeutic drugs. More generally, the molecular events involved in formation of the senescence-associated secretome have yet to be determined. We reveal here that melanoma cells experiencing senescence, in response to diverse stimuli produce a secretory profile endowed with pro-tumoral and pro-metastatic properties. We further provide a molecular relationship between senescence induction and secretome formation by deciphering some of the molecular mechanisms which drive formation of the secretome. Our findings also point out to the existence of such regulatory pathway in non-melanoma cells. Most importantly, inhibition of the signaling cascade involved in secretome formation prevents the pro-invasive properties of the secretome. Collectively, the results gathered in this report opens new avenues for therapeutic intervention against cancers.

CS18: From vitiligo to melanoma

C81

Effect of UVB therapy on the lymphocytic infiltrate in the skin of vitiligo patients

M. W. Kroon, G. Krebbers, R. Thijssen, W. Douwenga, J. D. Bos, J. P. W. Van Der Veen, M. A. Middelkamp Hup, R. M. Luiten
Deptartment of Dermatology and The Netherlands Institute for Pigment Disorders, University of Amsterdam, The Netherlands

Vitiligo is characterized by the formation of depigmented lesions of the skin, mediated by anti-melanocyte immunity. In vitiligo patients treated at the Netherlands Institute for Pigment Disorders, we found that exposure of vitiligo skin to narrowband (311 nm) ultraviolet B (UVB) radiation induces repigmentation of skin lesions. The immunosuppressive effect of UVB radiation has been described in murine models of contact hypersensitivity and skin carcinogenesis. These studies demonstrated that UVB radiation activates regulatory T cells, which suppress immune responses in an antigen-specific fashion. Activation of regulatory T cells is thought to be mediated by UV-damaged epidermal Langerhans cells migrated to local lymph nodes. We investigated the changes in infiltration of immune cells in the vitiligo skin upon UVB treatment. Twenty-two vitiligo patients, of whom 14 with actively spreading vitiligo, were treated with narrow band UVB (311 nm) twice weekly for 26 weeks. Skin biopsies of nonlesional (NL), lesional and perilesional (PL) skin were taken before and after UVB therapy. Lymphocytic infiltrate in these biopsies was analyzed by immunohistochemistry and correlated to the level of clinical repigmentation. We found an increased lymphocytic infiltrate upon UVB treatment in dermal skin of all biopsies of repigmenting patients, whereas infiltration was reduced in the epidermis. Analysis of CD3, CD4, CD8 and CD25 showed an increase in T cell infiltrate in lesional skin for all markers. The increase in CD4+ T cells was also found in PL en NL skin. Only low levels of cells expressing regulatory T cell marker Foxp3 were found, which was not affected or decreased by UVB. Further analyses are ongoing to identify the character of the CD4+ cells that infiltrate upon UVB treatment. This study shows that UVB therapy affects the lymhocytic infiltrate in vitiligo skin, predominantly by increasing the dermal influx of CD4+ cells. These cells may suppress ongoing anti-melanocyte immunity in vitiligo skin, thereby facilitating repigmentation.

C82

Functional cloning of a gp100-reactive TCR from depigmenting vitiligo skin

J. Klarquist, M. Li, D. A. Wainwright, R. M. Luiten, M. I. Nishimura, I. C. Le Poole
Loyola University Chicago, Maywood, IL, USA

In vitiligo, progressive melanocyte loss is accompanied by skin infiltrating T cells. As T cells effectively eliminate melanocytes in vitiligo but fail to clear melanoma tumors, TCRs specific for vitiligo antigens may offer superior therapeutic efficacy for clearance of melanoma. Thus, we proposed isolating, cloning and characterizing T cells infiltrating vitiligo skin. T cells were sorted using peptide-pulsed dimers, reactivity towards melanoma cells was compared with TIL, and cells were cloned by limiting dilution. Peptide reactivity was confirmed by ELISPOT analysis. Clones were amplified to $>1 \times 10^6$ cells and subjected to RNA isolation. Rearranged TCR genes were identified by 5′RACE and subsequent DNA sequencing of cloned variable

 © 2011 The Authors. Journal Compilation © 2011 John Wiley & Sons A/S

subunit fragments. T cells amplified from depigmenting skin of an HLA-A*0201+ patient were >16% gp100-209-217-reactive and demonstrated superior avidity compared with melanoma-derived T cells. TCR subunit analysis revealed a TCRα8, TCRβ17 and a TCRα21, TCRα17 expressing clone. The former was linked by a P2A slippage sequence, cloned into the SAMEN construct under the CMV promoter, transfected into Phoenix packaging cells and transduced into Jurkat cells. Resulting Jurkat-SILv44 cells produced 20 pg/ml IL-2 in response to peptide-pulsed T2 cells, even in the absence of phorbol ester stimulation. These studies represent the first cloning of a vitiligo skin-derived, HLA-A2+, gp100-reactive TCR. Whereas previous studies have implicated the α subunit in defining peptide binding specificity of MART-1-reactive T cells, both our gp100-reactive clones share a common β subunit with gp100-reactive, melanoma-derived T4H2, suggesting the defining subunit may be peptide-dependent. Importantly, SILv44 Jurkat cells display high TCR affinity as demonstrated by successful peptide-mediated activation without the need for CD8 co-stimulation.

C83

APE/Ref-1, a druggable target for the therapy of human melanoma

S. Yang, Z. I. Zheng, B. Misner, R. Chamberlin, F. L. Meyskens
University of California, Irvine, Orange, CA, USA

Human malignant melanoma exhibits impaired redox status and abnormal redox-regulated signal pathways. Induced as an adaptive response to reactive oxygen species (ROS) and reactive nitrogen species (RNS), a multi-functional protein called APE/Ref-1 serves as a redox chaperone and modulator of many nuclear transcription factors and for maintaining intracellular redox status. Our previous studies showed that knockdown of APE/Ref-1 significantly sensitized melanoma cells to chemo-treatment and reduced metastatic potential markedly. In this study, we further characterized the role of APE/Ref-1 in the invasive properties of human melanoma. Two function-deficient Ref-1 constructs were stably transfected into melanoma cells; further studies with Scratch Migration and Matrigel assays showed that both ΔNLS-Ref-1 and RedoxD-Ref-1 markedly decreased migration and invasive c-apacity. Matrix metalloproteinase (MMP)-1 mRNA levels were also significantly reduced in transfectants, which was reversed by APE/Ref-1 cDNA overexpression.

In addition, nitric oxide (NO) stress induced by DETA (NO donor) treatment was associated with enhanced invasion potential of melanoma cells, which was significantly reversed by APE/Ref-1 depletion. These results suggest that specific and potent inhibitors targeting APE/Ref-1 should be explored for therapeutic potential. Accordingly, through 3-D modeling and virtual docking, we successfully screened compounds from 35 chemical vendors (total number of compounds is more than 7 millions) and synthesized a specific APE/Ref-1 inhibitor (#598-21) with IC50 below 1 μM, which also significantly reduced the invasion of metastatic melanoma Lu1205 cells. Taken this molecule as a lead compound, we are screening and synthesizing more potent inhibitors with enhanced anti-melanoma activities.

C84

Involvement of TGF-β signaling and the GLI2 transcription factor in M-MITF regulation and pigmentation in melanoma cells

M.-J. Pierrat, V. Marsaud, C. Bertolotto, A. Mauviel, D. Javelaud
Institut Curie Orsay, France

The melanocyte-specific transcription factor M-MITF is involved in numerous aspects of melanoblast lineage biology including pigmentation, survival, and migration. It also plays complex roles at all stages of melanoma progression and metastasis. We have previously shown that expression of M-MITF in human melanoma cell lines is highly variable and inversely correlated with that of GLI2, a Hedgehog mediator identified as a TGF-β gene target. In this study, we demonstrated that GLI2 overexpression in highly pigmented melanoma cells reduced the expression of M-MITF and that of its gene targets TYR, TYRP1 and DCT, accompanied with a decrease of melanin synthesis. Transient cell transfection experiments with 5′-end M-MITF promoter deletion constructs identified the -317/-280 region as critical for GLI2-driven transcriptional repression. Chromatin immunoprecipitation (ChIP), identified GLI2 bound to the -395/-228 region of the M-MITF promoter. Since GLI2 is a transcriptional target of TGF-β pathway, we investigated whether the TGF-β signaling can repress M-MITF expression in melanoma cells. We showed that M-MITF expression was decreased by TGF-β treatment independently from proteasomal degradation. Inversely, the TbRI (TGF-β Receptor I) inhibitor SB431542 increased M-MITF expression. TGF-β induced a strong inhibition of M-MITF promoter activity. ChIP experiments indicated that TGF-β induces GLI2 binding to the -395/-228 region of the M-MITF promoter in melanoma cells. Yet, TGF-β still repressed M-MITF promoter activity after GLI2 knockdown, suggesting that other mechanisms are involved to drive TGF-β effect. Remarkably, TGF-β inhibited both CREB phosphorylation and PKA activity in melanoma cells and mutation of the cAMP Responsive Element (CRE) located at position -147/-140 of the M-MITF promoter abolished the inhibitory effect exerted by TGF-β. Taken together, these results suggest that TGF-β represses M-MITF expression and transcription by two distinct and concomitant means implicating GLI2 induction and inhibition of the cAMP/PKA pathway.

C85

Edn3 promotes metastasis and alters tumor heterogeneity in a mouse model of melanoma

N. Chin, J. C. Gallegos, R. Cruz, L. Kos
Florida International University, Miami, FL, USA

Melanoma is highly metastatic and resistant to current therapies. Accumulating evidence suggests that particular populations of tumor cells with a more undifferentiated phenotype may be responsible for these characteristics. Melanoma progression and metastasis is not only a result of these cells' intrinsic properties but is also determined by a number of complex tumor-host interactions. Cytokines released by cells in the tumor and in the microenvironment alter a variety of processes that result in tumor progression and malignancy. Tumor cells with a more undifferentiated phenotype may be more sensitive to the effects of these secreted factors and acquire a highly metastatic potential. The cytokine Endothelin 3 (Edn3) has been implicated in melanoma progression based on in vitro data showing that Edn3 alters the expression of cell adhesion proteins and metalloproteinases in melanoma cell lines. In this study we investigated the effects of Edn3 over-expression in melanoma progression and heterogeneity in vivo. We crossed Dct-Grm1 mice whose expression of the metabotropic glutamate receptor 1 (Grm1) under the Dopachrome tautomerase (Dct) promoter produce spontaneous melanocytic lesions in the ears and tails that do not metastasize, with transgenic mice that over express Edn3 under the keratin 5 promoter (K5-Edn3). Tumors appeared 2–3 months earlier and grew at a rate 3–4 times faster in Dct-Grm1/K5-Edn3 mice when compared to the Dct-Grm1 control mice. Ninety-five percent of Dct-Grm1/K5-Edn3 mice had pigmented lesions in distant organs such as the lung, spleen and salivary glands. These results indicate that Edn3 paracrine signaling alters the kinetics of melanocytic tumors' progression

© 2011 The Authors. Journal Compilation © 2011 John Wiley & Sons A/S

and can lead them to a fully malignant state. Dct-Grm1 tail tumors present heterogeneity with cells at different levels of differentiation as shown by non-overlapping populations of cells expressing the stem cell marker Nestin and the melanocyte-specific marker Tyrosinase related protein 1 (Trp1). There was a dramatic increase in the number of Nestin positive cells in the tail tumors of Dct-Grm1/K5-Edn3 when compared to Dct-Grm1control tumors indicating that Edn3 affects the size of the undifferentiated cell population. Together these results suggest that Edn3 may promote metastasis by regulating tumor cellular heterogeneity.

CS19: Preclinical and Clinical advances in melanoma management (SMR-IFPCS)

C86

Markers of telomeric crisis and immortalization in melanoma progression

M. Hossain, W. H. Chong, A. M. Ross, E. V. Sviderskaya, D. C. Bennett
St. George's, University of London, UK

Abnormal mitoses are characteristic of cancer cells. A common cause of these appears to be telomeric crisis. Crisis occurs in cells that have bypassed cellular senescence (irreversible cell cycle arrest after extended division, mediated by p16 and p53 pathways), and proliferated further. This leads to very short, dysfunctional telomeres, which can be ligated giving dicentric chromosomes. Consequences include large-scale chromosomal rearrangements, anaphase bridges, tripolar and other abnormal mitoses, and apoptosis. Rare cells may overcome crisis and become immortal by re-expressing TERT, a subunit of telomerase, required to maintain telomeres. Primary melanomas appear to have evaded senescence but possibly not crisis, as they often fail to yield immortal cells when explanted. Here we aimed to test whether and when crisis occurs in melanoma progression, and whether immortalization is associated with metastasis. Routine paraffin sections of archival lesions from St. George's Healthcare Trust were used, including approximately 20 each of radial growth phase (RGP), vertical growth phase (VGP), and metastatic melanomas (from lymph nodes and skin). Haematoxylin and eosin-stained sections were viewed for scoring of abnormal mitoses, with emphasis on anaphase bridges (relatively specific for crisis). Also noted were giant and multinucleate cells, and later nuclear blebs and chromatin bridges between nuclei. Currently, unstained sections are being immunostained for TERT, and for additional crisis markers. RGP melanomas showed very few mitoses, with no anaphase bridges, but some multinucleate cells and chromatin bridges. One abnormal mitosis only was observed in one RGP melanoma. In VGP melanomas, multinucleate cells, tripolar mitoses, nuclear blebs and chromatin bridges all appeared common, and anaphase bridges were seen in up to 40% of anaphases. Surprisingly, similar features were seen in most metastatic lesions, with similar percentages of anaphase bridges. Some RGP melanomas may be entering crisis. VGP melanomas have extensive features of crisis, consistent with the rarity of immortality in culture. Even some metastases show features of crisis, suggesting that melanoma cells may metastasize before full immortalization. Marker expression data should clarify these findings.

C87

The role of hypoxia in melanoma phenotype switching

D. S. Widmer, O. M. Eichhoff, M. C. Zipser, P. F. Cheng, R. Dummer, K. S. Hoek
University Hospital of Zürich, Switzerland

Our group reported a model for melanoma progression which involves melanoma cell switching between phenotypes of invasion and proliferation. In this study we examine the possible role of hypoxia as one of the microenvironmental influences driving metastatic progression in that it may promote the shift from a proliferative to an invasive phenotype. Microarray experiments were performed on proliferative phenotype melanoma cells exposed to hypoxic conditions in vitro. These experiments showed an up-regulation of invasive-phenotype-specific genes as well as a down-regulation of proliferative-phenotype-specific genes. In vitro invasion assays were performed to show that a hypoxic environment increased the invasiveness of proliferative melanoma cell cultures.
Extended periods of hypoxia increased invasive potential in proliferative phenotype cells in a dose-dependent fashion. Importantly, invasiveness was still increased when cells were returned to normoxia for 48 h. In contrast, invasive phenotype melanoma cells showed no increase in invasive potential upon exposure to hypoxia. Additionally, we stained Clark's level IV primary human cutaneous melanoma biopsies for key markers concerning melanocytic function, hypoxia, proliferation and vascularisation. Hypoxic regions correlate with a loss of melanocytic marker expression, supporting our in vitro data. These results suggest that exposure to a hypoxic microenvironment leads to the reprogramming of proliferative phenotype melanoma cells, down-regulating melanocytic marker expression and increasing their invasive potential. Separately, using microarray data we developed a protocol to mathematically assess the similarity of the gene expression data from individual samples to proliferative and invasive phenotype meta-standards. This tool allows us to make predictions concerning both phenotype and phenotype switching in different experimental contexts.

C88

SPARC acts as a VCAM-1 ligand to mediate melanoma extravasation and distant metastasis

M. Tichet, N. Fenouille, P. Abbe, S. Rocchi, M. Allegra, J.-C. Chambard, M. Vivinus, J.-P. Lacour, R. Ballotti, M. Deckert, S. Tartare-Deckert
INSERM U895, Centre Méditerranéen de Médecine Moléculaire (C3M) Team 1, Nice, France

A hallmark feature of melanoma is its metastatic propensity. Metastasis occurs by a complex process whereby tumor cells dissociate from their primary site of growth, invade surrounding tissues, intravasate into a blood vessel, adhere to and extravasate from the vessel, and form a new tumor at secondary site. SPARC is a secreted matricellular protein, which is highly produced by melanoma cells. Clinical studies have reported that elevated SPARC is associated with melanoma patients with poor survival and occurrence of distant metastases. Mechanistically, tumor cell-autonomous SPARC signaling promotes invasive abilities and favors melanoma growth by inhibiting p53 tumor suppression function. Although SPARC has a significant role in invasion and cell survival, its contribution to later stages of the metastatic cascade such as tumor-endothelial cell interactions and extravasation remains unexplored. By modeling in vitro the extravasation process between metastatic melanoma from short-term cultures or cell lines and primary vascular endothelial

 © 2011 The Authors. Journal Compilation © 2011 John Wiley & Sons A/S

cells, we show that melanoma cell–derived SPARC is dispensable for adhesion of tumor cells to TNF-alpha-activated endothelial cell monolayers but required for subsequent transendothelial migration. SPARC-induced transendothelial migration is partially dependent on its binding to vascular adhesion molecule (VCAM-1) expressed on inflamed endothelial cells. Melanoma cell SPARC activates Src family kinases and p38 MAPK in endothelial cells and promotes intercellular gaps permissive for tumor cell diapedesis. Importantly, SPARC-mediated melanoma transmigration does not require the p53 regulatory function of SPARC. Using fluorescence and bioluminescence imaging, we also show that knockdown of SPARC leads to a dramatic decrease in short-term and long-term colonization of the lungs by melanoma cells. Together, our studies reveal that tumor cell SPARC is emerging as a multifunctional mediator of metastatic development contributing both to local invasion and lung extravasation, which provide a rationale and mechanistic basis for targeting SPARC to inhibit melanoma metastases.

C89

Melanocytes and melanoma cells present different mechanical properties that can be modulated by Endothelin 3

A. P. Benaduce, D. Lahiri, A. Agarwal, L. Kos
Florida International University, Miami, USA

The transformation of melanocytes into melanoma cells and the processes of local invasion and metastasis are accompanied by several molecular changes. Although it is clear that these events also require that cells undergo changes in shape and deformation, little has been done to describe the cellular mechanical properties that may underlie these changes and how they may be regulated by signals from the microenvironment. In this study, we evaluated if changes in mechanical properties can be used to distinguish between normal and transformed melanocytes. We adapted a technique generally used in materials engineering, nanoindentation, to compare the elastic storage modulus and membrane rupture load of human primary melanocytes and human melanoma cells cultured on plastic chambers. We found that primary melanocytes are 2.5 times stiffer and 3.5 times harder than melanoma cells. Furthermore, the rupture strength of the cell membrane in primary melanocytes was 2.5 times higher than melanoma cells. These results indicate that melanoma cells are significantly more elastic than normal cells. This difference in elasticity may facilitate the migration and invasion of cancerous cells during metastasis. Previous studies have suggested that the activation of Endothelin receptor b by Endothelin 3 (Edn3) may be involved in melanoma progression by altering the expression of cell adhesion molecules. Therefore, we evaluated the effects of Edn3 on the mechanical properties of primary melanocytes and melanoma cells. Cells were exposed to 1 nM of Edn3 for the period of 1, 3 and 5 days. Primary melanocytes showed a gradual decrease in hardness and stiffness as the Edn3 exposure period increased, reaching values similar to those of melanoma cells. Edn3 treated melanoma cells did not show any significant difference in hardness and only a moderate gradual increase in stiffness as the Edn3 exposure period increased. There was no significant difference between the rupture strength of the cell membrane for both types of cells after Edn3 exposure. These results demonstrate that primary melanocytes and melanoma cells modulate their biomechanical properties differentially upon Edn3 exposure. This is the first study to validate the use of nanoindentation as a valuable tool to analyze the differences in mechanical properties of normal and cancerous cells. It also supports the use of cellular biomechanical properties as potential markers of cellular transformation.

CS20: Congenital nevus and melanoma, clinically-oriented

C90

Understanding melanocyte development: biological analysis associated with mathematical modeling

F. Luciani, D. Champeval, A. Herbette, L. Denat, B. Aylaj, S. Martinozzi, R. Ballotti, R. Kemler, C. R. Goding, F. De Vuyst, L. Larue, V. Delmas
CNRS-UMR3347, Institut Curie, INSERM U1021, Orsay, France

Mice with a defined background display a similar uniform coat color. This indicates that there is no major variation in melanocyte production during development throughout the entire body or between individuals. Therefore, the mechanisms of melanocyte production must be tightly regulated to provide a defined number of cells: initially there are a very limited number of progenitors and subsequently thousands of cells. Mice with a non-uniform pigmentation pattern are the result of mutations in genes controlling the melanoblast developmental program: the affected genes may be involved in determination, proliferation, migration, differentiation or other processes. In this study, we aim to evaluate environmental and genetic effects on the expansion/proliferation of melanoblasts during development. The better understanding of these processes will help to characterize abnormal melanoblast development leading to pigmentation disorder. We showed that melanoblast expansion is tightly controlled both spatially and temporally, with little variation between embryos. We established a mathematical model reflecting the main cellular mechanisms involved in melanoblast expansion, including proliferation and migration from the dermis to epidermis. The model allows the calculation of doubling times for melanoblasts, revealing that dermal and epidermal melanoblasts have short but different doubling times. Moreover, the number of trunk founder melanoblasts at E8.5 was estimated to be 16, a population impossible to count by classical biological approaches. We also assessed the importance of the genetic background by studying gain- and loss-of-function β-catenin mutants in the melanocyte lineage. We found that any alteration of β-catenin activity, whether positive or negative, substantially reduced both dermal and epidermal melanoblast proliferation. Finally, we determined that the pool of dermal melanoblasts remains constant in wt and mutant embryos during development, implying that specific control mechanisms associated with cell division ensure half of the cells at each cell division to migrate from the dermis to the epidermis. Modeling melanoblast expansion revealed novel links between cell division, cell localization within the embryo, and appropriate feedback control through β-catenin. In consequence, the disruption of this feedback control may result in abnormal dermal and/or epidermal melanoblast number and distribution leading to pigmentation disorder.

C91

Cellular dynamics in congenital melanocytic nevi: is 'maturation with depth' a one-way road?

C. M. Salgado, A. Davis, A. Heider, D. Basu, A. Rebbaa, M. Reyes-Múgica
Children's Hospital of Pittsburgh, University of Pittsburgh Medical Center, Pittsburgh, PA, USA

Giant congenital melanocytic nevi (GCMN) are malformative neurocristopathies composed of early transformed melanocytes, i.e. nevus cells (NVCs) whose precursors derive from neural crest cells (NCs) and reach the skin migrating through anatomical pathways shared with other elements such as skin nerves. NVCs of GCMN, being present at or around birth, had no time to be

© 2011 The Authors. Journal Compilation © 2011 John Wiley & Sons A/S

exposed to usual environmental stressors (i.e. UV light) linked to adult nevus development. Therefore, the pathogeneses of adult-age and congenital nevi are most likely different. Furthermore, clinical repigmentation is frequently seen in GCMN treated by peeling. Despite this, current histological criteria to evaluate GCMN are based on long-held views of an assumed downward maturation cellular dynamics, possibly applicable to acquired but unlikely in congenital nevi. Our aim is to assess the progressive maturation of NVCs in GCMN using markers of temporal/stage differentiation. Immunophenotyping of GCMN formalin-fixed paraffin-embedded tissue using markers present in NCs and/or NVCs: CD56, DCT, Tyrosinase, MITF, c-Kit, HMB45, Mart1. Semiquantitative evaluation at four levels of deepness: dermo-epidermal junction, papillary/reticular dermis, and subcutaneous tissue. Correlation between morphology and Immunoreactivity. SPSS® Kriskal–Wallis statistical analysis. Thirty-two GCMN, 23 females, age range 3 months to 13 yr. Head and neck: 10; trunk: 15; Limbs: 5; unspecified: 2. Earlier markers like CD56 and DCT tend to stain deeper portions and to diminish or disappear in superficial layers ($P < 0.001$). More mature markers such as HMB45 are stronger in superficial layers ($P < 0.001$). MITF stains equally all layers. NVCs begin expressing earlier markers of NC cells towards melanocyte differentiation predominantly in deeper layers. This expression is conserved and intensifies in superficial layers. More mature markers predominate in superficial layers of nevi. Developmental biology concepts are in keeping with different pathogeneses and cell dynamics for GCMN and adult-age nevi. In congenital nevi, the traditionally held approach of histological analysis may require revision.

C92

Epithelial to mesenchymal – like transition is an earlier cellular response to stress than senescence: potential role as a target for cancer prevention

D. Basu, L. Schmitt, C. Gallati, M. Reyes-Mugica, A. Rebbaa
University of Pittsburgh, Pittsburgh, USA

Epithelial to mesenchymal transition (EMT) is a phenotypic reprogramming thought to enhance cellular invasiveness. In cancer, this process is considered as a late event responsible for the orchestration of metastasis, however its role in tumor initiation is not yet understood. Here we sought to determine whether EMT occurs in premalignant nevi and thus, could be amenable to targeting for the prevention of melanoma. We hypothesized that if EMT plays a role in the formation of premalignant tumors, it must occur before the senescence state that characterizes these lesions. In vitro experiments were carried out to study the temporal relationship between these two processes in response to oncogenes and other stressors. Expression of EMT and senescence markers was analyzed in melanoma cell lines and also in nevi specimens. The role of GSK3 beta as a potential target to inhibit EMT was validated using genetic and pharmacologic approaches. The data indicated that regardless of the stress applied, EMT was induced first, then senescence. Immunohistological analysis indicated that melanocytes within benign nevi expressed mesenchymal markers, suggesting that an EMT-like process may play a role in the biology of these lesions. GSK3 beta was found to facilitate the degradation of Zeb1, a key player in EMT, and inhibit cellular proliferation as well as motility. In addition, activators of GSK3 beta exerted anti-proliferative and anti-EMT activities. Findings from this study suggest that: (i) EMT is an earlier cellular response to stress than senescence; (ii) an EMT-like process is likely to occur in premalignant tumors and (iii) GSK3 beta may represent a compelling target to disrupt EMT pathways in premalignant and malignant melanoma.

C93

Differential LEF1 and TCF4 expression is involved in melanoma cell phenotype switching

O. M. Eichhoff, A. Weeraratna, M. C. Zipser, D. S. Widmer, L. Kriegl, L. Larue, R. Dummer, K. S. Hoek
Department of Dermatology, Universtiy Hospital Zürich, Switzerland

The phenotype switching model for melanoma progression holds that melanoma cells, in response to microenvironmentally-regulated changes in signalling, switch back-and-forth between states of proliferation and invasion to drive metastatic spread. Proliferative phenotype melanoma cells show up-regulation of melanocytic marker genes, while the invasive phenotype cells show up-regulation of factors involved in modifying the extra-cellular environment. Furthermore, proliferative and invasive expression signatures correlate with specific characteristics of in vitro behaviour. Since canonical Wnt signaling has been shown to be important for melanocytic gene expression, survival and proliferation, phenotype switching has been linked to changes in Wnt signalling. Therefore we looked for cell phenotype-specific differences in the expression levels and activity of beta-catenin and its LEF/TCF co-factors. This showed that beta-catenin nuclear distribution and activity is not phenotype-specific. Conversely, we found the expression of LEF1 and TCF4 to be both phenotype-specific and anti-correlated. LEF1 is preferentially expressed by proliferative phenotype cells and TCF4 by invasive phenotype cells. Knock-down experiments confirmed that these co-factors are important for the phenotype-specific expression and behaviour, and that LEF1 suppresses TCF4 expression independently of beta-catenin. Our data shows that melanoma cell phenotype switching behaviour is regulated by differential LEF1/TCF4 activity.

CS21: DNA repair and melanoma molecular biology

C94

A large French case–control study assessing the association of MC1R with melanoma: the unexpected role of non-RHC and rare MC1R variants

H. H. Hu, V. Descamps, A. Bourillon, N. B. Seguin, A. Riffault, K. Ezzedine, C. Lebbe, M. Bagot, A. Bensussan, P. Saiag, B. Grandchamp, N. Soufir
INSERM U976, Centre de Recherche sur la Peau, Hôpital Saint-Louis, Paris, France

Melanocortin 1 receptor (MC1R), a Gs protein coupled receptor, regulates the melanin synthesis and skin pigmentation. The MC1R gene is highly polymorphic. Several alleles are associated with red hair and fair skin phenotypes and contribute to melanoma risk. Here, we focused on the role of the various classes of MC1R alleles, especially non-RHC alleles and rare MC1R variants, as their role on melanoma risk is still debated. The entire coding region of MC1R was sequenced in 1131 cutaneous melanoma patients and 869 skin cancer free Caucasian controls. MC1R variants were classified in two main categories: R (RHC), and r (non-RHC). The functionality of rare variants was assessed according to the type of mutation and/or the predicted functional effect by in silico prediction tools (SIFT, SNPs3D and PolyPhen), and these were thereafter classified in two subgroups, functional (F) or non-functional (nF). Statistical analysis was performed by PASW software. The effect of R, r, F, and nF variants was assessed by Fisher's exact test and Odds Ratio calculation with 95% confidence interval. All known frequent MC1R variants (including R and r alleles) were found

© 2011 The Authors. Journal Compilation © 2011 John Wiley & Sons A/S

in this study. In all, we identified 70 r variants, in which 30 were exclusively found in cases and 24 in controls. Of these, there were 46 missense, two non-sense, three frame shift and 19 silent mutations. Thirty-nine missense mutations were predicted to be functional. R alleles were strongly associated with melanoma (P = 1.09E-33; OR = 3.295). Interestingly, r alleles were also associated, although less strongly, with melanoma (P = 1.1E-15; OR = 1.839). Of these, the most frequent V60L and V92M were clearly associated with melanoma (ORs = 3.22 and 3.9). In addition, rare F variants were strongly associated with melanoma, as high as R variants (P = 3.2E-5; OR = 2.933). Oppositely, rare nF variants had no effect on melanoma risk (P = 0.82). Furthermore, multivariate analysis showed that both R and r variants associated with melanoma risk. R, but not r variants also associated with familial and multiple melanoma risks (P = 0.019 and P = 0.0018). In this large study, we confirmed the role of R variants on melanoma risk. In addition, we clearly showed that r variants also associate with melanoma, and define a novel class of F variants that are also strong melanoma risk factors. These findings may have important consequences in defining high-risk melanoma factor subgroups and/or may help to the genetic council in melanoma families.

C95

USF1 is critical for the regulation of ner genes essential for early recognition of UV induced DNA-Photolesions

S. Corre, Y. Baron, N. Mouchet, A. Bouafia, S. Vaulont, S. Prince, M.-D. Galibert
CNRS-UMR6061, Institut de Génétique et Développement, Universite de Rennes 1, Rennes, France

The skin is the first body organ that is exposed to various physical, chemical and biological hazards that can alter DNA structure. To maintain the integrity of the genome, cells are equipped with specific defense machinery, which targets different types of alterations of the DNA. NER is one of the most versatile DNA repair systems that can eliminate a wide variety of DNA lesions. It can be divided into at least two sub-pathways depending on the localization of the distorted DNA: Global Genome Repair (GGR) and Transcription Coupled Repair (TCR). Both pathways involve the cooperation between a wide number of factors, required for lesion recognition through to DNA repair. The lack of these proteins lead to defects in DNA repair processes and are associated with severe genetic disorders, including xeroderma pigmentosum (XP), Cockayne syndrome (CS), Fanconi anemia. Here, we show for the first time that the expression of CSA and HR23A, two factors involved in TCR and GGR respectively, is up-regulated after UV induced DNA damage in the skin. Using a combination of in vivo and in vitro assays we demonstrate that in response to multiple UV-mediated DNA damage there is a specific and coordinated regulation of CSA and HR23A genes and their protein levels. We show that up-regulation of both HR23A and CSA is driven by a common p53 independent mechanism involving the stress responsive Upstream Stimulating factor 1. USF1 is an evolutionary conserved transcription factor of the bHLH-LZ protein family, which regulates its target genes through specific cis regulatory elements called E-boxes. The current study therefore extends the repertoire of USF target genes mediating skin protection against UV. CSA and HR23A are particularly important in the global regulation of the NER and are responsible for both removing DNA lesions and for the resumption of cell cycle progression and transcription.
Importantly, we show using a genetic approach with USF1 knock-out mice, that the regulation of CSA and HR23A by USF1 in response to UV-induced DNA damage is physiologically significant. Results from this study provide compelling evidence that USF1 plays a key role in DNA-repair and in maintaining genome integrity.

C96

Evasion of ROS-dependent pigment cell senescence

C. Leikam, A. Hufnagel, S. Walz, S. Kneitz, M. Eilers, M. Schartl, S. Meierjohann
Department of Physiological Chemistry I, University of Wurzburg, Wurzburg, Germany

The development of malignant melanoma is a highly complex process which is only partly understood. A majority of human melanomas are found to express a handful of oncogenic proteins, such as mutant c-KIT, RAS, BRAF and GNAQ variants. However, most of these oncogenes are also found in nevi, and it is now a well-accepted fact that their expression alone leads to senescence. This renders the understanding of senescence escape mechanisms an important point in order to be able to understand tumor development. Here, we describe the ability of the transcription factor MYC to drive the evasion of melanocyte senescence. Conversely, MIZ1, the growth suppressing interaction partner of MYC, is involved in mediating melanocyte senescence. MYC overexpression and Miz1 knockdown led to a strong reduction of endogenous reactive oxygen species (ROS), DNA damage and senescence. We identified the cystathionase (CTH) gene product as mediator of the ROS-related MYC and MIZ1 effects. Blocking CTH enzymatic activity in MYC-overexpressing and Miz1 knockdown cells increased intracellular stress and senescence. Importantly, pharmacological inhibition of cystathionase in human melanoma cells also reconstituted senescence in many cell lines, and CTH knockdown enhanced senescence and reduced proliferation and soft agar growth. Thus, we identified cystathionase as new MYC target gene with an important function in MYC-mediated senescence evasion.

C97

Induction of γ-H2AX by UV and α-melanocyte stimulating hormone, and implications on DNA repair in human melanocytes

V. B. Swope, C. Alexander, A. L. Kadekaro, S. Schwemberger, G. Babcock, Z. A. Abdel-Malek
Department of Dermatology, University of Cincinnati, Cincinnati, USA

Activation of the melanocortin 1 receptor (MC1R) by α-melanocyte stimulating hormone (MSH) enhances nucleotide excision repair, reduces oxidative DNA damage, and stimulates melanogenesis in UV-irradiated human melanocytes (HM). To investigate the regulation of γ-H2AX (the phosphorylated form of H2AX) in UV-irradiated HM ± MSH pretreatment, and the correlation of γ-H2AX with repair of DNA damage. Western blotting and immunostaining followed by flow cytometry analysis were performed to detect γ-H2AX, ChK1 and CHK2; immunocytochemistry was conducted to detect γ-H2AX in HM irradiated with UV ± MSH pretreatment. UV resulted in a dose- and time-dependent induction of γ-H2AX, which peaked 6–8 h post irradiation. Pre-treatment with 10 nM MSH or 1 μM forskolin increased UV-induced γ-H2AX, suggesting that this effect was cAMP-dependent. The response to MSH required the expression of functional MC1R. Treatment with the antioxidant N-acetyl cysteine did not alter the UV-induced γ-H2AX, suggesting that γ-H2AX was mainly due to DNA photoproducts or their DNA repair intermediates. The kinetics of γ-H2AX correlated with the kinetics of repair of pyrimidine 6–4, pyrimidone photoproducts. The UV-induced γ-H2AX ± MSH was reduced by caffeine, an

© 2011 The Authors. Journal Compilation © 2011 John Wiley & Sons A/S

inhibitor of ATR, ATM and DNA- PK, and KU 55933, an inhibitor of ATM. MSH increased the phosphorylation of CHK1 within 1 h, and of CHK2 within 2 h after UV exposure. CHK2 phosphorylation had similar kinetics as γ-H2AX. These results demonstrate enhancement of UV-induced γ-H2AX by MSH, and suggest a role of γ-H2AX in repair of DNA photoproducts in UV-irradiated HM.

Plenary session VII Translational research/vitiligo

C98

Retrospective analysis of melanoma and non-melanoma skin cancer incidence in a large vitiligo patient cohort

H. E. Teulings, M. Overkamp, E. Ceylan, L. Nieuweboer-Krobotova, T. Nijsten, J. D. Bos, A. W. Wolkerstorfer, R. M. Luiten, J. P. W. van der Veen
University of Amsterdam, The Netherlands

Vitiligo development in melanoma patients is associated with effective anti-melanoma immunity and consequent favourable clinical outcome. Anti-melanoma immunity can mediate vitiligo by targeting both melanoma cells and melanocytes. This suggests that vitiligo patients, having active anti-melanocyte immunity, have a decreased risk of developing melanoma. Little is known about the incidence of melanoma in vitiligo patients. We evaluated the lifelong incidence of melanoma in a large vitiligo patient cohort and in a matched healthy control group. Vitiligo patients lack pigmentation and are therefore expected to have a higher risk of developing non-melanoma-skin-cancer (NMSC), we therefore also evaluated the lifelong incidence of NMSC. We performed a retrospective survey in patients of the SNIP aged 50 yr or older and diagnosed with vitiligo vulgaris. The questions in this survey concerned patient demographics, skin type, onset of vitiligo and treatment history, sun exposure/ behaviour and history of skin cancer. All patients were asked to have a control questionnaire filled in by their (non-vitiligo) partner or non-blood line related family member or friend. All skin cancers reported by patients or controls were validated by the official pathology report. Statistical significant differences in outcome between both groups as well as external factors contributing to melanoma or NMSC incidence were analysed. Until now we have collected 1200 vitiligo – and 800 matched controls' questionnaires. Preliminary results indicate that melanoma occurs less frequently in vitiligo patients compared to healthy controls. NMSC incidence is also lower, despite a history of NB-UVB/PUVA therapy in most vitiligo patients. Results of other analyses will be discussed.

C99

A new mouse model of vitiligo with epidermal depigmentation reveals a critical role for IFN-gamma in autoreactive T cell homing to the skin

J. E. Harris, T. H. Harris, W. Weninger, E. J. Wherry, C. A. Hunter, L. A. Turka
Division of Dermatology, University of Massachusetts Medical School, Worcester, MA, USA

Vitiligo is a devastating, disfiguring disease of the skin that results from melanocyte destruction and subsequent patchy depigmentation of the epidermis. Both intrinsic melanocyte abnormalities and autoimmune mechanisms contribute to disease pathogenesis, and an essential autoimmune component is the direct destruction of melanocytes by CD8+ T cells, which migrate from the blood into the skin. Preliminary data from our laboratory reveal that the expression of IFN-gamma (IFNg) and IFNg target genes is increased within affected skin of patients with vitiligo, while the expression pattern of other inflammatory pathways is normal. Existing mouse models of vitiligo consist primarily of depigmentation of the hair, rather than skin. Our objective was to develop a mouse model of vitiligo with epidermal depigmentation, and to test our hypothesis that IFNg is required for disease pathogenesis. We developed the first mouse model of vitiligo to display prominent depigmentation of the skin, which is the hallmark of human disease. In this model, melanocyte-specific CD8+ T cells were adoptively transferred to hosts with high melanocyte density in the epidermis, which results in black skin as well as black hair. Gene expression was quantified by quantitative PCR, and T cell accumulation in the skin was measured through microscopy and flow cytometry. Five weeks after transfer, hosts develop patchy depigmentation of exposed epidermal surfaces, including the ears, nose, feet, and tail. Histology of depigmenting skin reveals a patchy mononuclear infiltrate at the dermoepidermal junction, single-cell infiltration of the epidermis, and melanocyte loss, all features of active human vitiligo. Depigmentation is accompanied by accumulation of autoreactive CD8+ T cells in the skin, quantifiable loss of tyrosinase expression, and local IFNg production. Neutralization of IFNg results in defective T cell accumulation within the skin and abrogates disease, demonstrating a critical role for the cytokine in driving pathogenesis. We have established a new mouse model of vitiligo that closely mimics human disease through clinical appearance, histology, and IFNg expression. Our data implicate IFNg as a critical cytokine in vitiligo pathogenesis, and reveal its role in autoreactive T cell migration to the skin. We propose that targeting IFNg signaling is a promising strategy for the development of new treatments.

C100

Spontaneous epidermal depigmentation in mice – a model for vitiligo

J. Klarquist, J. Eby, B. J. Longley, M. I. Nishimura, S. Mehrotra, I. C. Le Poole
Loyola University Chicago, Maywood, IL, USA

A HLA-A2 restricted, tyrosinase reactive T cell receptor derived from CD4+ TIL was cloned, characterized and transgenically expressed in HLA-A2 transgenic C57BL6 mice under the TCR promoter. Resulting h3TA2 animals were crossed with k14-SCF mice that carry epidermal melanocytes. As the 1383I TCR functions in a coreceptor independent manner on human T cells, resulting mice can reveal whether the TCR can be functionally expressed without coreceptor involvement in mice, and whether resulting T cells are reactive with tyrosinase expressing target cells in the epidermis. Surprisingly, transgenic T cells are found in the periphery of h3TA2 mice and spontaneous depigmentation of the pelage is observed starting from 4 weeks of age, increasing to 42% depigmentation regardless of gender by 25 weeks of age and 60% depigmentation by week 28. Depigmentation is accompanied by loss of melanocytes and T cell infiltration to the skin. TCR Tg expression is found primarily on T cells expressing a CD4–CD8– phenotype, specifically activated in response to HLA-matched human and mouse melanocytes. A five-fold reduction in infiltrating Treg was observed in skin homogenates but not splenocytes from h3TA2 mice, similar to reduced Treg abundance selectively in human vitiligo skin. On a k14-SCF background, we observed depigmentation of the skin and pelage amounting to 75% depigmentation by 25 weeks of age and 80% depigmentation by week 28. Curiously, depigmenting mice developed nevus-like skin lesions at around 3–4 weeks old, which increased in pigmentation level over time until stabilizing at 7–8 weeks. The NeVi (Nevus-Vitiligo) mouse faithfully reproduces the effector phase of autoimmune reactivity

 © 2011 The Authors. Journal Compilation © 2011 John Wiley & Sons A/S

in vitiligo and can be used to assess efficacy of new treatment modalities for the disease.

C101

Descriptive assessment on dynamic change of dendritic cell distribution both in epidermis and dermis of the lesional skin in generalized vitiligo vulgaris: link between cellular autoimmune response and melanocyte disappearance

S. Itoi, A. Tanemura, Y. Kotobuki, M. Wataya-Kaneda, I. Katayama
Graduate School of Medicine, Osaka University, Suita city, Japan

Vitiligo vulgaris is an acquired depigmentation disease with well-circumscribed depigmented spots as its character and gradually expanding, increasing, and fusing partly due to local immune condition. For the cause of disease, the autoimmune theory is the most widely-held consideration cited for the existence of HLA-DR+CLA+CD8+ cell and anti-melanocyte antibody in vitiligo vulgaris patient. Since this theory does not fully explain the pathogenesis for autoimmune vitiligo, we examined whether there might be a certain change in inflammatory cells distribution in vitiligo skin compared to normal skin. In the present study, we found apparent difference of langerhans cells number and distribution between lesional and nonlesional skin. Thereafter, to investigate the comprehensive cellular immunity involved in vitiligo in addition to CD8+ cell infiltration, we performed immunohistochemical analysis for the detection of immune competent cells with the following markers; CD3, CD4, CD8, Foxp3, IL-17A, HLA-DR, AHR in an affected area by utilizing the skin tissue of 14 cases from generalized vitiligo patients.
Moreover, the presence of dendritic cells (DC), key modulator of an innate immune system in the skin, was also assessed by DC markers including CD11c, CD123 and langerin. The change in those distribution patterns was paneled with clinical characteristics including disease duration. Based on these results, we would like to show a comprehensive infiltration pattern of immune competent cells in vitiligo skin and propose a pathological cellular contribution to autoimmune vitligo.

C102

Dysregulation of melanocyte function and survival induced by Th17-related cytokines and their involvement in the pathogenesis for vitiligo vulgaris

A. Tanemura, Y. Kotobuki, L. Yang, M. Wataya-Kaneda, H. Murota, M. Fujimoto, S. Serada, T. Naka, I. Katayama
Department of Dermatology, Osaka University Graduate School of Medicine, Osaka, Japan

Although the cellular immunoresponse, mainly of cytotoxic T cells targeting melanocyte-specific proteins has been shown to destroy functional melanocytes in autoimmune vitiligo, this does not provide a full explanation for the etiology of vitiligo, suggesting the presence of other cellular immunity. Recent reports showed increase in inflammatory cytokines expression and their regulation of melanocyte activity in vitiligo. In this study, we investigated whether Th17 cells infiltrated into vitiligo skin as they do in cases of psoriasis, and whether the proinflammatory cytokines produced by Th17 cells and/or skin resident cells influenced in melanocyte function and survival. We observed a significant number of Th17 cells in 16 out of 18 of the vitiliginous skin, while there was sparse infiltration in the normal skin. There was no significant correlation between the number of infiltrating Th17 cells and the clinical type or with disease parameters such as disease duration, age or gender. In vitro analysis, exogenous IL-1β, IL-6, and IL-17A downregulated the expression of MITF and its downstream melanogenic proteins, TYR, TRP-1 and -2 in dose-dependent manner. In addition, melanin production was significantly decreased after the treatment with those cytokines. IL-17A exponentially increased the production of IL-1β, IL-6 and TNF-α in skin resident cells such as keratinocyte and fibroblast. These results suggest that Th17 cell infiltration in vitiligo skin may be important for the dysregulation of melanocyte function and survival and that local IL-17A may trigger the production of proinflammatory cytokines and formulate the cytokines loop involving Th17 cell recruitment.

Plenary session VIII Translational research/Miscellaneous (2)

C103

The role of Interferon Regulatory Factor 4 IRF4 in pigmentation

C. Grill, C. Praetorius, A. Schepsky, E. Steingrímsson
University of Iceland, Reykjavík, Iceland

In a recent gene-expression analysis we identified the Interferon Regulatory Factor 4 (Irf4) gene as a potential target of Mitf in melanoma cells. The Irf4 gene encodes a transcription factor which acts as a lineage survival oncogene in multiple myeloma. Genome-wide association studies in humans have shown that polymorphisms in the IRF4 gene are associated with fair skin and light/blue eye color, suggesting that the gene plays an important role in melanocytes. Effects of Mitf on Irf4 gene expression were investigated using Mitf mutant mice and siRNA studies in melanocyte and melanoma cell lines. ChIP studies were used to investigate presence of the MITF protein on the Irf4 promoter and reporter gene assays to investigate effects of MITF on the Irf4 promoter and the synergistic effects of MITF and IRF4 on Tyrosinase expression. Irf4 gene expression is dependent on MITF. This is shown both by the absence of Irf4 expression in Mitf mutant mice and by significant reduction of Irf4 gene expression upon Mitf reduction using siRNA. ChIP studies show that the MITF protein binds regulatory sequences in IRF4 intron 4, near a polymorphism that has been associated with skin and eye color. Importantly, the IRF4 and MITF proteins synergistically regulate the expression of the pigmentation gene Tyrosinase but not of several other melanocyte specific genes. Mutating the IRF4 binding sites in Tyrosinase eliminates this synergistic effect. We have shown that Irf4 expression in pigment cells depends on Mitf and that together, the MITF and IRF4 proteins affect the regulation of the Tyrosinase promoter. This may explain the pigmentation phenotype in humans.

C104

Live imaging and mathematical modeling of the role of Kit/Kitl in melanoblast behaviour

R. L. Mort, M. Moffat, L. Hay, K. J. Painter, I. J. Jackson
MRC Human Genetics Unit, Edinburgh, UK

Melanoblasts, the embryonic precursors of melanocytes, are derived from the neural crest and migrate along the dorsolateral pathway. They populate the epidermis around embryonic day 12 (E12) and localise to embryonic hair follicles between E13.5 and E16.5. Partial failure of this developmental process leads to white spotting phenotypes often associated with mutations in the tyrosine kinase Kit or its receptor Kitl. To investigate this process we fluorescently labeled the melanoblast lineage by crossing transgenic mice that express Cre recombinase under the control of the tyrosinase promoter with R26EYFP reporter mice. We cultured embryonic skin in an ex vivo system and for the first time were able to perform live imaging of melanoblast migration. Analysis of time lapse sequences reveals that mela-

© 2011 The Authors. Journal Compilation © 2011 John Wiley & Sons A/S

noblasts are highly motile and allows measurement of the dynamics of their migration. Preliminary evidence confirms a chemokinetic role for Kitl in melanoblast motility and localisation to developing hair follicles. We have begun to construct mathematical models from this data to allow us to explain how individual cellular properties, tissue expansion and signalling through Kit/Kitl can contribute to the net dispersal of the melanoblast population and its subsequent localisation to the developing hair follicles.

C105

Melanocyte adaptation to ER stress and activation of the unfolded protein response in Oca2-null melanocytes

T. Cheng, S. J. Orlow, P. Manga
Department of Dermatology, NYU School of Medicine, New York, NY, USA

Stress induced by buildup of misfolded proteins in the endoplasmic reticulum (ER) triggers the unfolded protein response (UPR). Failure by the UPR to restore homeostasis can trigger apoptosis. We have shown chronic UPR activation in tyrosinase and Tyrp1 mutant melanocytes, where tyrosinase is retained in the ER, without concomitant loss of viability, suggesting that melanocytes adapt to ER stress. Identifying adaptive mechanisms has implications for treatment of melanocyte disorders. For example, UPR activation may permit adaptation to hypoxia and play a role in melanomagenesis. Thus melanoma therapies targeting the UPR are being tested. The UPR may also play a role in vitiligo. Xbp1 (a key UPR transcription factor) polymorphisms are associated with increased risk of vitiligo, while ER dilation in perilesional melanocytes suggests ER stress. The UPR consists of three pathways regulated by IRE1, ATF6 and PERK. Prolonged IRE1 signaling in stressed cells promotes survival, while sustained PERK activity promotes apoptosis. OCA2 mutations result in hypopigmentation due, in part, to ER retention of tyrosinase, but do not result in loss of viability. We therefore investigated the UPR in Oca2-(null) melanocytes. Wildtype and Oca2-melanocytes were treated with ER stressors thapsigargin or tunicamycin and UPR activation was monitored by RT-PCR and Western blot analysis. Ire1 expression was increased in Oca2-melanocytes compared to wildtype cells. However, downstream signaling was not activated, with no splicing of Ire1 targeted Xbp1. Expression of Perk and its downstream effector Atf4 were decreased in Oca2-melanocytes. Upon ER stress, Ire1 expression and Xbp1 splicing increased in both cell lines indicating UPR activation. Typically, UPR activation leads to Perk-mediated phosphorylation of eIF2α. Remarkably, levels of p-eIF2α were markedly diminished in stressed Oca2-cells. Expression of pro-apoptotic CHOP was still induced in Oca2-melanocytes, but did not result in significant cell death. Our data suggest that Oca2-melanocytes adapt to persistent ER stress by UPR dysregulation. Xbp1 is not spliced despite increased Ire1 expression, while additional ER stress leads to disruption of pro-apoptosis Perk signaling. Delineating the mechanisms by which melanocytes adapt to sustained ER stress and UPR activation will be key to identifying determinants of viability and provide targets for therapies for vitiligo and melanoma.

C106

LC3B punctate expression, a marker for autophagosomes, is a common feature of melanomas and breast carcinomas and associated with proliferation, metastasis, high nuclear grade and poor outcome

R. Lazova, R. Camp, V. Klump, S. Siddiqui, R. Amaravadi, J. M. Pawelek
Dermatopathology, New Haven, CT, USA

There is rapidly growing interest in autophagy and cancer, however the full extent and impact of autophagy throughout human malignancies is as yet unknown. We recently showed that the well-known presence of coarse melanin in pigmented melanomas is due to autophagosomes filled with melanized melanosomes. Further, using the autophagosomal modulating protein, LC3B (microtubule-associated protein 1, light chain 3, isoform B) as a marker, 31/31 invasive malignant melanomas expressed moderate to high levels of autophagosomes regardless of the presence of pigment. However, correlation of autophagy with pathological or clinical outcomes in cancer has been limited. Here using punctate LC3B as an autophagosome marker we analyzed hundreds of melanomas and breast carcinomas in tumor tissue microarrays (TMAs) with a focus on correlations between LC3B staining and clinical outcomes. TMAs consisted of primary and metastatic melanomas (n = ~1200) and clinically annotated primary breast carcinomas (n = ~600). IHC protocols were developed for automated quantitative analysis (AQUA) using fluorescence-tagged antibodies against LC3B and the proliferation marker Ki-67. An AQUA program was designed to quantitate LC3B distribution in punctate and diffuse cellular compartments. LC3B staining was moderate to high in approximately 90% of invasive and metastatic melanomas and approximately 75% of primary breast cancers. In both, the cytoplasmic area occupied by punctate LC3B was elevated three to five-fold in tumors with the highest versus lowest levels of total LC3B expression. LC3B and Ki-67 expression showed strong correlations ($P < 0.0001$) and mitotic figures were most frequently detected in high versus low LC3B-expressing tumors, together indicating an association of autophagy with proliferation. In melanoma, LC3B total expression was elevated in nodal metastases versus primaries, and in breast carcinoma was elevated in node-positive versus node-negative primaries and correlated with increased nuclear grade and shortened disease specific survival. High levels of LC3B expression were associated with increased punctate LC3B, proliferation, metastasis, high nuclear grade and worse patient outcome. The results indicate a surprisingly common occurrence of LC3B expression in these cancers and strongly support previous evidence that autophagy plays a role in cancer progression. It is thus becoming apparent that autophagy presents an important new target of vulnerability in solid tumors.

CS22: Vitiligo: Report on Global Issues Consensus Conference and selected papers clinically-oriented

C107

Alterations of cellular redox-sensitive pathways regulation in vitiligo melanocytes converge to stress-activated cellular senescence phenotype

B. Bellei, A. Pitisci, M. Ottaviani, M. Ludovici, C. Cota, M. L. Dell'Anna, M. Picardo
IFO San Gallicano, Rome, Italy

Several in vitro and in vivo studies evidenced an alterated redox status and an increased susceptibility of vitiligo cells to pro-oxidant agents supporting the idea that imbalance of the cellular redox status might be the pathogenic clue in vitiligo. In order to study

 © 2011 The Authors. Journal Compilation © 2011 John Wiley & Sons A/S

intracellular pathways crucially involved in stress-dependent vitiligo cells damage, we focused on cAMP/PKA/CREB and MAPK-dependent signal transduction. We found a full correlation between ROS production, constitutive stimulation of antioxidant enzymes expression (Nrf2, HO-1, NQO1, SOD2 and catalase), activation of the stress activated protein kinase p38 MAPK, p53 nuclear localization, hyperphosphorylation of CREB and increased membrane cholesterol level. Notably, all these long-term effects of subcytotoxic oxidative stress are also 'biomarkers' of premature cellular senescence. Consistent with the hypothesis that vitiligo melanocytes are inclined to acquire senescent phenotype, we demonstrated that vitiligo cells in vitro presented a significant increase in p16 and p21 expression that did not correlate with donor chronologic age. Chronic stimulation of mitogenic-activated signaling and downstream inhibition of cyclin-dependent kinases in presence of elevated level of cyclinD1 suggests that vitiligo melanocytes are prone to undergo to a hypermitogenic cell cycle arrest. Moreover, as predicted by the hypermitogenic arrest vitiligo cells showed attenuated responses to growth factors. In fact, despite the normal induction of cyclinD1 gene expression, cell proliferation remained largely inhibited for vitiligo melanocytes treated with α-MSH in minimal medium. We report for the first time robust evidences demonstrating that vitiligo melanocytes present alteration of redox homeostasis, membrane lipid composition, transduction pathways, and changes at mRNA and protein level of genes coding for cell-cycle regulators that altogether argue for a pathologic predisposition to stress-induced premature senescence.

C108

Systematic review of outcome measurements for the treatment of vitiligo

V. Eleftheriadou, K. Thomas, J. Batchelor, J. Ravenscroft, M. Whitton
Centre of Evicence Based Dermatology, Nottingham, UK

Vitiligo is a chronic depigmentation disorder affecting around 0.5% of the world population. Despite previous attempts, there is still a lack of consensus as to the definition, methods of assessment and outcome measurements of this disorder. The updated Cochrane systematic review concluded that the majority of studies differ greatly in the ways in which vitiligo is measured. The pre-specified outcomes were based mainly on empirical evidence and specialist consensus, therefore more research is needed to address the definition of effectiveness and success of interventions from clinicians' and patients' perspective. The objective of this review is to describe the heterogeneity in outcome measurements currently used in randomised controlled trials for vitiligo. A systematic review of outcome measures used in randomised controlled trials published in English and included in the updated Cochrane systematic review was performed. Two independent researchers extracted outcomes data from eligible randomised controlled trials. Fifty-four randomised controlled trials were evaluated. Three were excluded as published English translations were not available. Only 22% (12/54) of trials had clearly stated primary outcome measures. Repigmentation was reported as an outcome in 98% (53/54) of trials and was measured using a great variety of scales including grades (e.g. 0–4), categories (e.g. poor to excellent, partial to complete), percentage of repigmentation, quartiles (e.g. 0–24, 25–50, 51–74, 75–100) percentage grades (e.g. 0–40, 40–60, 60–100, 0–30, 31–50, 51–75, 75–100), reduction of surface areas in %, mean difference in lesion size in millimeter. The definition of excellent repigmentation (or the top grade repigmentation) varied from trial to trial and included values from 50 to 100% repigmentation of vitiliginous lesions. Repigmentation was assessed mainly by clinicians. Only 1% (2/54) of trials included patients as assessors. Pattern of repigmentation was reported in 4% (7/54) of trials. Colour match of newly regimented vitiliginous lesions was assessed in 2% (4/54) of trials. Patients' opinions on colour match were only included in one of them. Three percent (6/54) of trials assessed quality of life of patients. Five percent (9/54) measured cessation of spreading of the disease; only 1.5% (3/54) clearly stated the scale used. Only 2% (4/54) of studies reported patients' opinions regarding treatment efficacy and degree of satisfaction including cosmetic acceptability of the results. Other outcomes such as side effects, cumulative dose, mean number of sessions to reach repigmentation, blood parameters were reported. The importance of incorporating patients' and clinicians' views into clinical research is now being increasingly recognised. We believe that more emphasis should be placed on patients' views with regard to assessing outcome measurements in future vitiligo trials and that consensus should be reached as to the most informative ways of reporting percentage of repigmentation.

C109

The paradox of the effects of PGF2alpha on pigmentation in vivo and in vitro

T. S. Anbar, D. Kendall, H. Abdel-Raouf, T. S. El-Ammawi, M. Barakat, A. T. Abdel-Rahman, R. Torky, A. Fawzy, M. Hanna
Andrology and STDs, Al-Minya University, Al-Minya, Egypt

In a laboratory study we used cultured immortalized human melanocytes (Hermes-2B) to study the effects of prostaglandin (PG)E2 and PGF2alpha on melanin content and ERK1/2 activation, which is belongs to MAPK family responsible for proliferation, differentiation, cell survival and apoptosis, using Western immune-blotting. Since cAMP is known to be involved in melanin synthesis, the acute effects of the drugs on cAMP formation in the cells were assessed using a [3H]-adenine pre-labelling assay. It was found that both prostaglandins induced a significant activation of the ERK1/2, however only PGE2, but not PGF2alpha, increased the melanin content. Neither PGE2 nor PGF2alpha increased intracellular cAMP accumulation. On the other hand, in an animal study using 18 female adult wild Guinea pigs we evaluated the effects of three PGF2alpha analogues on skin pigmentation in comparison to NB-UVB both clinically and histopathologically. All three PGF2α analogues were found to increase skin pigmentation at the site of application to a degree comparable with that of NB-UVB. Moreover, in a recent study we evaluated the efficacy of topical Latanoprost, PGF2alpha analogue, in induction of skin re-pigmentation in 22 stable non-segmental vitiligo patients and compared its potency with NB-UVB. It was found that Latanoprost induced a significantly better skin re-pigmentation compared with placebo. This pigmentation was similar to that obtained after NB-UVB exposure. From all the above obtained results, we are wondering if the enhancement of dendricity and maturation induced by PG F2alpha in vitro is enough to explain its effect on induction of pigmentation, in animal model and vitiligo patients, or there are other mechanisms related to the nearby keratinocytes or growth factors that may play a role, a question waiting to be answered.

C110

Double blind randomized clinical trial on bFGF related deca peptide to treat vitiligo

A. Ramaiah, H. K. Kar, V. K. Garg, N. Bajaj, A. S. Madhava
Independent Higher Education Professional, Hyderabad, India

Combination of Topical application of bFGF related deca peptide with sun/UVA exposure was found effective in the earlier conducted open ended clinical trials to treat Non-segmental, segmental, PUV-A resistant and fast spreading vitiligo either by

© 2011 The Authors. Journal Compilation © 2011 John Wiley & Sons A/S

itself or in combination with PUV_A, or with oral steroids. The deca peptide lotion is marketed in India since 2004 to treat vitiligo. A multicentric randomized double blind phase 1V clinical trial on bFGF related deca peptide to treat vitiligo was conducted in Delhi during 2009 to evaluate the efficacy and safety of topically applied deca peptide alone and in combination with Narrow band ultra violet light (NB-UVB) in patients with non segmental vitiligo. The chosen vitiligo macules were non-sun exposed areas on patients with stable non segmental vitiligo. The trial was conducted for 3 months on 30 volunteer patients with NB-UVB alone or plus peptide group and an equal number with deca peptide applied group with peptide alone or vehicle alone. This research was conducted in accordance with the principles enunciated in the Declaration of Helsinki (Ethical Principles for Medical Research involving Human subjects revised in 2008. The pigmentation of the macules before and after treatment were measured objectively by Canfield Inc proprietary soft ware. The change from base line macule area over time for macules treated with Peptide and NB-UVB indicated an earlier and more dramatic response than control. Peptide and NB-UVB act synergistically and repigmented the vitiligo macules far better than either of them alone with statistical significance by Chi square statistical method. It was concluded that the results with NB-UVB plus deca peptide is far superior to NB-UVb alone and that it may be further improved by application of the deca peptide many hours after the NB-UVB radiation rather than soon after the radiation treatment as was done in the present trial. The peptide and NB-UVB treatment was well tolerated and safe.

CS23: Albinism: Basic science and patient-oriented session clinically-oriented

C111

An optimized strategy for genetic testing of the Chinese patients with oculocutaneous albinism

A. Wei, Y. Wang, W. Li
Institute of Genetics and Developmental Biology, Chinese Academy of Sciences, Beijing, China

Oculocutaneous albinism (OCA) is a relatively common inherited disorder in all populations worldwide. Currently, at least 16 genes have been identified as causative genes for OCA. The mutational spectra of OCA are population-specific. We aim to develop an optimized strategy for the genetic testing of Chinese OCA patients based on the spectra of the mutational genes and alleles. Genomic DNA was extracted from the blood samples of 179 clinically diagnosed OCA patients and 100 unaffected subjects. The amplified DNA segments from the axons and the adjacent intronic regions were screened for mutations of TYR, OCA2, TYRP1, SLC45A2, HPS1 and HPS4 by Sanger sequencing. To exclude the previously unidentified alleles (PUAs) from polymorphisms, samples from 100 unaffected controls were sequenced for the same regions of variations. Among the 179 OCA patients, 115 (64.3%) were found mutations on TYR gene, 21 (11.7%) on OCA2, 28 (15.6%) on SLC45A2, 4 (2.2%) on HPS1, and 11 (6.2%) patients uncharacterized. We have not found any mutations on the TYRP1 and HPS4 gene. We identified 61 PUAs in these patients, 21 in TYR, 21 in OCA2, 17 in SLC45A2, and two in HPS1. Common alleles have been revealed in the TYR and SLC45A2 gene in the Chinese OCA patients. The unidentified 11 samples may be due to: (i) unamplified regions of the screened OCA genes; (ii) mutations on the unscreened rare OCA genes and (iii) mutations on the unidentified genes. To exclude the later two possibilities, we developed a family-based exome sequencing to identify the causative genes. An optimized method to screen the OCA mutations is efficiently implemented in the routine genetic testing of Chinese OCA patients accompanied with genetic counseling.

C112

Albinochip: a universal genetic diagnosis for all known mutations associated to albinism

E. Moltó, A. Fernández, C. Phillips, M. Torres, O. Maronas, B. Arveiler, F. Morice-Picard, A. Taïeb, R. Aquaron, V. Schiaffino, M. Hayashi, T. Suzuki, M. Martínez, M. J. Trujillo, C. Ayuso, Á. Carracedo, L. Montoliu
CNB-CSIC, Campus de Cantoblanco, Madrid, Spain

Albinism is a rare disease affecting 1:17 000 Europeans. There are several types of albinism, globally characterised by hypopigmentation, affecting skin, hair and eyes (in oculocutaneous albinism, OCA) or mainly eyes (in ocular albinism, OA), and by a profound visual impairment, for which they are primarily handicapped. Severe and rarer forms of albinism show additional symptoms, including increased infections, bleeding problems and bruising (Hermansky–Pudlak syndrome, HPS, and Chediak-Higashi syndrome, CHS). At least 14 genes have been identified whose mutations are associated with albinism, namely: TYR (OCA1), OCA2 (OCA2), TYRP1 (OCA3), SLC45A2/MATP (OCA4), GPR143 (OA1), LYST (CHS1), HPS1 (HPS1), AP3B1 (HPS2), HPS3 (HPS3), HPS4 (HPS4), HPS5 (HPS5), HPS6 (HPS6), DTNBP1 (HPS7) and BLOC1S3 (HPS8). All these genes encode proteins that are involved in melanin synthesis, melanosome function or lysosome-related organelle biogenesis. In persons with albinism, besides the characteristic hypopigmented phenotype, the main handicapping feature is their common severe visual deficiencies, leading to legal blindness in most cases. More than 500 mutations have been reported in those 14 loci, causing albinism, as obtained from the literature and human gene mutation databases. Currently, very few institutions in the world offer the genetic diagnosis of albinism for some of the known 14 loci involved. As a result, the vast majority of persons with albinism remain not diagnosed and hence, early detection of this rare genetic condition is prevented, and the establishment of the recommended care and helping measures is unfortunately delayed or missed, thus affecting the life-quality of the patients. In this regard, from the Biomedical Network Research Centre on Rare Diseases (CIBERER) in Spain, in collaboration with experts in albinism from around the world, we have developed a new technological strategy aiming to a universal genetic diagnosis of all types of albinism. This approach uses iPlex methodology, by Sequenom, which combines automated array processing of human subject's DNA samples with mass spectrometry (MALDI-TOF) and can be applied to discriminate, simultaneously, up to 1000 different alleles with known mutations, from a given DNA sample. Therefore the 'albinochip' proposal aims to define a universal genetic test for all known forms of albinism using massive genomic approaches with the aim of detecting any known mutation in any of the affected genes. Moreover, additional new complementary genomic approaches will be designed to reveal new mutations in these and other loci. About 20% of the patients with albinism remain undiagnosed or not fully diagnosed, because either no mutation or only one mutation is found. The mutation search must therefore be improved using novel genomic approaches, including exome sequencing for detecting new mutations and/or new albinism genes. Members of ALBA (www.albinismo.es), the Spanish association in support of persons with albinism, have contributed extensively to this project. Updated results of this collaborative project will be reported. Grant Support: CIBERER INTRA/07/704.1

 © 2011 The Authors. Journal Compilation © 2011 John Wiley & Sons A/S

C113

Oculocutaneous albinism type I (OCAI) in the Jewish populations of Mediterranean Sea countries (Algeria, Morocco, Tunisia): a story of the p.Gly47Asp mutation on the tyrosinase gene and personal data about five cases

R. Aquaron, C. Badens, J. Kaplan, E. Lasseaux, F. Morice-Picard, C. Rooryck-Thambo, B. Arveiler
School of Medicine, Marseilles, France

Oculocutaneous albinism type I (OCAI) is a rare autosomic recessive disorder. In classic, type IA (i.e. tyrosinase-negative) OCA, tyrosinase activity and melanin biosynthesis are entirely absent, whereas in type IB (i.e. 'yellow') OCA tyrosinase activity and melanin biosynthesis are greatly reduced, thus giving rise to two different phenotypes. Both types result from homozygosity or compound heterozygosity for different mutations in the tyrosinase gene. The p.Gly47Asp (p.G47D) missense mutation, located in exon 1, has been first identified by Oetting et al. (1991), in one Caucasian family from USA and 3 yr later by the same group in 12 unrelated subjects from Hispanic descent originating from the Canary Islands and Puerto Rico, all with the OCAIA phenotype. This mutation has also been found by Gershoni-Baruch (1994), in nine Morroccan Jews in the homozygous state and in five Jews patients from Morocco and Tunisia in the heterozygous state with OCAIA and IB phenotypes. This mutation appears to be associated with the same RFLP haplotype in Moroccan Jews, in patients from the Canary Islands, Puerto Rico and the United States.
We report examination of five OCAI patients from Morocco, Algeria and Tunisia in Marseille (3), Paris (1) and Bordeaux (1). All five exons and exon-intron boundaries of the tyrosinase gene were PCR ampified and sequenced according to standard methodologies. We found the p.G47D mutation at homozygous state in two Morroccan subjects with the OCA1A phenotype and at heterozygous state in three other patients. The other mutation, found in two subjects from Algeria and Tunisia with the OCA1B phenotype, was near the 3′ end of the second intervening sequence (IVS2), a T>A transition 7 bp upstream of the 3′ splice (IVS2-7A). This mutation interfering with normal splicing resulted in a frameshift. The other mutation in the second Tunisian patient was unknown. The history of the Sephardic (Spanish) and Moroccan Jewish populations may provide clues to the origin and spread of this mutation. The close geographic proximity and political relationship of Spain to Morocco and the Canary Islands, the exploration of Puerto Rico by Spanish sailors, and the historical population migration from the Canary Islands to Puerto Rico suggest that the p.G47D mutation in all of these populations derives from a common origin. The very high prevalence of this mutant allele among Sephardic Jewish population should facilitate carrier status

C114

BLOC-1 mutation screening in Hermansky–Pudlak syndrome reveals a new HPS subtype, HPS-9, associated with mutations in PLDN (pallidin) and a novel BLOS3 (HPS-8) mutation.

A. R. Cullinane, J. A. Curry, C. Carmona-Rivera, G. Golas, C. G. Summers, C. Ciccone, N. D. Cardillo, H. Dorward, R. A. Hess, J. G. White, D. Adams, M. Huizing, W. A. Gahl
Medical Genetics Branch, Bethesda, USA

Hermansky–Pudlak Syndrome (HPS) is an autosomal recessive condition characterized by oculocutaneous albinism and a bleeding diathesis due to absent platelet delta granules. HPS, a genetically heterogeneous disorder of intracellular vesicle biogenesis, and has eight known subtypes, seven of which are associated with genes encoding different Biogenesis of Lysosome-related Organelles Complex (BLOC) proteins. We screened patients with HPS-like symptoms for mutations in HPS1-6 and found no functional mutations in 38 individuals. We then examined these individuals for all eight genes encoding the BLOC-1 proteins. We identified homozygous nonsense mutations in PLDN, encoding the BLOC-1 subunit, pallidin, in a single individual with characteristic features of HPS. No PLDN mutations have previously been described in humans, although Pldn is mutated in the HPS mouse model pallid. The pallidin protein interacts with the early endosomal t-SNARE syntaxin-13. We could not detect any full-length pallidin protein in our HPS-9 patient's fibroblasts or melanocytes despite normal mRNA expression of the mutant transcript. An alternative transcript was detected in the patient that would skip the exon that harbors the mutation; however, we demonstrate that, if this transcript were translated into protein, it would not interact with syntaxin-13 although it correctly localizes to early endosomes. We also identified only the second mutation to date in BLOS3, causing HPS-8. In this infant's melanocytes, BLOS3 mRNA expression was significantly reduced compared to control, suggesting nonsense-mediated decay was occurring for this mutation. Absence of either PLDN or BLOS3 causes instability of the BLOC-1 protein complex, and in both HPS-9 and HPS-8 melanocytes, the melanogenic protein TYRP1 showed aberrant localization, increased plasma-membrane trafficking, and failure to reach melanosomes. These results help explain the patients' severe albinism and establish a common cellular defect within patients having BLOC-1 gene mutations.

CS24: Skin depigmenting/repigmenting agents, from basic mechanisms to application

C115

Characterization of the bioactive motif of neuregulin-1, a fibroblast-derived paracrine factor that regulates constitutive color and melanocyte function in human skin

W. Choi, L. Kolbe, V. J. Hearing
NCI, NIH, Bethesda, MD, USA

Interactions between melanocytes and neighboring cells in the skin are important in regulating skin color in humans. We previously demonstrated that the less pigmented and thicker skin on the palms and soles is regulated by underlying fibroblasts in those areas, specifically via a secreted factor (DKK1) that modulates Wnt signaling (J. Cell. Biol., 2004). Similarly, fibroblasts in the dermis also play a role in regulating constitutive skin color of individuals ranging from very light to very dark via secretion of neuroregulin 1 (NRG-1) (J. Cell. Sci., 2010).
Both of those factors cause their effects by interacting with specific receptors expressed on the surface of melanocytes, and the signaling pathways affected then regulate melanocyte differentiation. We have now begun mapping the bioactive domain in NRG-1 that is involved in regulating pigmentation and are identifying the minimal bioactive motif within the EGF domain of NRG-1 that is sufficient to increase the pigmentation of normal human melanocytes. We show that overlapping 8-mers within the EFG domain are able to mimic the stimulatory activity of the complete protein, and our goal is to identify even smaller peptides that are bioactive. This information will be useful for the future in vivo application of NRG-1 to regulate human skin pigmentation.

© 2011 The Authors. Journal Compilation © 2011 John Wiley & Sons A/S

C116

Active vitiligo lesions are more responsive to combination therapy with narrowband ultraviolet light B and topical tacrolimus

Y. Huang, H. Lui, Y. Zhou
Yuanshen Huang, Vancouver, Canada

At present, narrow band UVB is the most commonly used treatment worldwide for regaining pigment in patients suffering from vitiligo. Recent results support improved efficacy by addition of topical tacrolimus. The objective for the current study is to evaluate if the clinical factors such as lesional activity and lesional molecular markers are correlated with therapeutic outcomes. Fifty-nine patients with non-segmental vitiligo were recruited for this study. A target lesion is selected for each vitiligo patient. The lesional activity is deemed 'active' if one of the following two conditions is met: (i) its onset is within 6 month; or (ii) for lesions lasting longer than 6 month, they show signs of expansion the past 6 months. The target lesion was then biopsied, together with non-lesional skin that matches the target lesional skin in anatomic characteristics. As controls, torso skin from healthy volunteers was also obtained. The vitiligo patients then underwent repigmentation therapy that combines narrow-band UVB phototherapy (2×/week) and topical tacrolimus (twice per day on non-phototherapy days). The therapeutic response is defined as significant if there is >30% repigmentation at 6 months. In addition, the gene expression profiles of the skin biopsies were analyzed using DNA microarrays containing 41 K human gene probes. The results were verified using RT-PCR. The 'active' vitiligo lesions are much more likely to respond to combined NBUVB plus topical tacrolimus therapy than older or stable lesions. Further, compared with lesions that show no or little repigmentation response, the responsive lesions showed much higher residual markers of melanogenesis, such as TYR and TRP1. Recent onset vitiligo lesions are much more responsive to the repigmentation therapy, possibility due to higher numbers of residual melanocytes. Therefore, early initiation of therapy is an important therapeutic strategy for regaining lost pigmentation.

C117

Depigmentation therapies for normal skin in vitiligo universalis

K. M. Al Ghamdi
King Saud University, Riyadh, Saudi Arabia

If vitiligo involves most of the body, it might be easier to depigment the normal remaining skin rather than to attempt repigmentation. We reviewed the literature to date regarding available therapies for depigmenting the normal skin in vitiligo universalis. Our review revealed that the threshold regarding what percentage of body surface area qualifies as depigmentation is variable among practitioners. Monobenzyl ether of hydroquinone (MBEH) is the most widely used depigmenting agent and has few side-effects. Tretinoin in combination with MBEH is able to speed depigmentation of the skin. Monomethylether of hydroquinone has also been used successfully for depigmentation. Eighty-eight per cent phenol is also effective in depigmenting the skin but its application on large areas is toxic for liver and kidney. Different types of lasers are also available to destruct the melanocytes selectively, but this technique can be painful and expensive. Cryotherapy is a cheap depigmenting therapy but, because of scarring risk, it should only be used by experienced dermatologists. No trials have compared the efficacy of the above-mentioned well-established depigmentation agents/techniques. Certain drugs such as imatinib, imiquimod and diphencyprone, which are used to treat other diseases, caused depigmentation as a side-effect. Some depigmentation agents used for branding cattle can also serve as topical depigmentation agents. In conclusion, comparative clinical trials are needed to compare the efficacy of various depigmentation agents/techniques. In particular, topical imatinib, imiquimod and diphencyprone may be considered as potential depigmenting agents, which require further investigation. This review revealed that MBEH is safe and effective depigmenting agent.

C118

Topical rapamycin therapy is effective for hypomelanotic macules arising in tuberous sclerosis complex

M. Tanaka, M. Wataya-Kaneda, E. Kiyohara, A. Tanemura, A. Nakamura, S. Matsumoto, I. Katayama
Department of Dermatology Course of Integrated Medicine Graduate School of Medicine, Osaka University, Suita-shi, Osaka, Japan

Tuberous sclerosis complex (TSC) is an autosomal dominant disorder characterized by the development of hamartomas in multiple organs. TSC has various characteristic features besides hamartomas, and hypomelanotic macule is one of them. TSC is caused by mutations in either TSC1 gene encoding hamartin or TSC2 gene encoding tuberin. The complex of hamartin with tuberin downregulates mammalian target of rapamycin (mTOR). Dysfunction of this complex results in constitutive activation of mTOR, which induces cell proliferation and tumorigenesis in TSC. Therefore, mTOR inhibitors such as rapamycin are effective for tumors arising in TSC. Although many functions of mTOR in TSC are known, there have been no reports of the involvement of mTOR in hypomelanotic macules arising in TSC. We aimed to examine the efficacy of rapamycin treatment on hypomelanotic macules in TSC and the influence of rapamycin for melanogenesis. Hypomelanotic macules on the faces of two patients diagnosed with definite TSC were treated topically with 0.2% rapamycin ointment twice a day for 12 weeks. In addition, the expression of microphthalmia-associated transcription factor (MITF) in melanoma cells and melanocytes treated with rapamycin was examined by RT-PCR. Hypomelanotic macules were disappeared in both patients after 12 weeks topical treatment without obvious side effects. Treatment with rapamycin influenced the expression of MITF in melanoma cells and melanocytes. Topical rapamycin therapy is effective and safe for hypomelanotic macules due to TSC. These results indicate that activation of mTOR participates in hypomelanotic macules arising in TSC.

Abstracts of Posters P1–P171

P1

Melanin molecular recognition

J. M. Belitsky
Department of Chemistry and Biochemistry, Oberlin College, Oberlin, OH, USA

There are many opportunities at the intersection of the fields of molecular recognition and melanin biochemistry. Self-assembly appears to play a large role in melanin structure, thus there is the problem of how the components recognize each other. Melanins bind a wide variety of metal ions and organic compounds, a property which can be exploited for environmental applications. Binding interactions can also be useful for the inhibition of melanin formation. An iterative synthetic strategy centered on the Suzuki reaction (one of the reactions highlighted in this year's Nobel Prize in Chemistry) has been used to generate well-

 © 2011 The Authors. Journal Compilation © 2011 John Wiley & Sons A/S

defined simple indole oligomers. Extension of this strategy to DHI analogs will allow to address many questions in eumelanin self-assembly, structure, and function. We are using polymerization chemistry methods to generate eumelanin-like materials with potential as water purification agents, including coatings that change color upon binding lead and other heavy metals. We have identified a class of compounds known as boronic acids, a wide number of which inhibit in vitro melanin polymerizations, both at the L-dopa/tyrosinase stage and at later stages of synthetic melanin formation. While our own synthetic chemistry efforts are at an early stage, this is promising approach to the complex problem of melanin structure. The selective binding of heavy metals may impact melanin biochemistry, and, in addition to probing these interactions, our synthetic materials may be useful as filtration agents and sensors. Boronic acids inhibit multiple stage of melanin polymerization in vitro, and our studies suggest design principles for further development of melanogenesis inhibitors.

P2

New index as melanin-pigmented skin marker

S. Cardillo, G. Miotto, M. Massironi, C. A. Pallaoro, I. Meyer, F. Vianello
Symrise AG, Holzminden, Germany

Melanin is a natural pigment produced within organelles, the melanosomes, located in melanocytes. Biological functions of melanosomes are often attributed to the unique chemical properties of the melanins they contain; however, the molecular structure of melanins, the mechanism by which the pigment is produced, and how the pigment is organized within the melanosome remains to be fully understood. Most natural melanins are mixtures of eumelanin and pheomelanin, and their formation is a complex pathway that starts with the oxidation of tyrosine (or DOPA, 3,4-dihydroxy-phenylalanine) by auto-oxidation or by enzymes, like tyrosinase or peroxidase, possibly in the presence of cysteine, which results in the production of 5-S-cysteinyldopa and its isomers. A procedure for the analysis of melanin-pigmented skin samples, based on alkaline hydrogen peroxide degradation coupled with high-performance liquid chromatography (HPLC) and ultraviolet determination of pyrrole-2,3,5-tricarboxylic acid (PTCA) for eumelanin and 6-(2-amino-2-carboxyethyl)-2-carboxy-4-hydroxybenzothiazole (TTCA) for pheomelanin was developed. Advantages related to the degradation conditions and sample handling were coupled to an improved chromatographic methodology for simultaneous determination of PTCA and TTCA as representative markers of eumelanin and pheomelanin, respectively, based on the use of an octadecylsilane column (Phenomenex Hidro-RP 80A, 250 × 4 mm) with 16 mM potassium phosphate, 4 mM tetrabutylammonium chloride, and 10% acetonitrile at pH 6, as eluent. The method requires conventional HPLC equipment and results in good peak shapes and resolution. Advantageous is a 3D (dimensional) plot representation of analytical data of skin samples (PTCA content, PTCA/TTCA ratio, and skin phototype), that gives a valuable graphical parameter for reliable identification of the structural skin markers, and a normalization of melanins to the skin surface by calculating an index as melanin-pigmented skin marker. The method can be applied to various eumelanin and pheomelanin pigmented skin tissues and is suitable to be employed in population screening studies. The presented methodology is susceptible to be further improved and refined in order to explore specific issues related to cosmetic and therapeutic applications.

P3

Measurements of hydroxyl free radical-scavenging capacities of melanin-binding hydroxychloroquine using an electron spin resonance spectroscopy

T.-C. Lei
Department of Dermatology, Renmin Hospital of Wuhan University, Wuhan, China

To determine the scavenging effects of melanin-binding hydroxychloroquine (HCQ) on transient hydroxyl free radical generated via the Fenton reaction, it is helpful in better understanding the mechanisms of antimalarials behind anti-inflammation and anti-photosensitivity. Water-soluble bacteria-derived melanin was prepared from a tyrosinase-producing bacterium (Pseudomonas maltophilia AT 18). Transient hydroxyl free radical was generated via the Fenton reaction and trapped by DMPO. DMPO (400 mM), ferrous sulfate (0.4 mM), H_2O_2 (0.1%) and varying concentration of melanin-binding hydroxychloroquine were mixed and incubated, then sucked into a quartz capillary for 30 s and measured by ESR. The relative amount of the radicals was estimated from the peak height of the second peak of the DMPO-OH signals in typical ESR spectra. The bacteria-derived melanin (b-melanin) ranging from 20 to 100 mg/l exhibited a potent scavenging action for hydroxyl free radical in a dose-dependent manner. One hundred milligram per litre of b-melanin could afford a maximum scavenging activity (92.5%) of against hydroxyl free radical. HCQ showed a limited and dose-irrelevant scavenging activity toward hydroxyl radical. Fifty micromolar of HCQ had the highest scavenging rate (43.72%) against hydroxyl free radicals. The melanin bound with 10 μM HCQ, corresponding to the mean blood concentration of HCQ in SLE patients receiving 400 mg of HCQ daily, demonstrated an increased scavenging activity against the radicals to compare with that of HCQ alone ($P < 0.05$). Elevated concentration of HCQ to bind with the melanin didn't increase their scavenging rate of hydroxyl free radical. The melanin achieves an enhanced scavenging activity on hydroxyl free radical by binding with HCQ at the physiological concentration (10 μM HCQ), by which protects the skin cells from oxidative damages and likely contributes to anti-photosensitivity of antimalarials in the treatment of cutaneous lupus erythematosus.

P4

No evidence of the eumelanic brown phenotype in alpaca (Vicugna pacos)

R. Cransberg, K. Munyard
Alpaca Fibre, Bentley, Australia

The diversity of alpaca fibre colour is one of its main selling advantages. The aim of this study was to increase understanding of what physical properties of the fibre are contributing to colour. Samples of the most common solid alpaca fibre colours were collected from Australian Alpaca Fleece Limited (AAFL) and from the 2009 Western Australian Winter Alpaca Show. The samples were grouped into 11 different colour categories as defined by AAFL. These were assayed using the spectrophotometric method of Ozeki et al. (1996) to identify the relative levels of total melanin and ratio of eumelanin to pheomelanin. There was no significant difference ($P = 0.209$) between white and light fawn animals in total melanin content (A500 nm). There was similarly no significant difference in the eumelanin: pheomelanin ratio (A650 nm/A500 nm) between white ($\mu = 0.144$) and light fawn ($\mu = 0.158$, $P = 0.238$), fawn ($\mu = 0.146$, $P = 0.428$), brown ($\mu = 0.155$ $P = 0.208$), and red brown samples ($\mu = 0.155$, $P = 0.232$), all of which were predominantly pheomelanic. Black and grey animals displayed higher levels of total melanin

© 2011 The Authors. Journal Compilation © 2011 John Wiley & Sons A/S

(Average A500/mg hair were 0.413 and 0.289 respectively) and fibre containing more eumelanin (Average A650/A500 were 0.329 and 0.395 respectively) when compared to lighter coloured animals. It is probable that the eumelanic brown phenotype is not present in alpacas, because brown alpaca fibres contained predominantly pheomelanic melanin, rather than brown eumelanin. Furthermore, using this method, there is no difference in total melanin content or ratio between animals that are classified as white and light fawn.

P5

Phenol Oxidase-Tyrosinase Pathway metabolites – possible connection with melanotransferrin MTf, p97 in melanoma and Alzheimer disease (AD)

M. Rachkova, B. Dimitrov
Michael Reese Hospital, Chicago, IL, USA

Melanotransferrin as a melanoma antigen and Alzheimer's disease (AD) biological marker has been implicated in diverse physiological processes such a plasminogen activation, angiogenesis, cell migration, but does not play essential role in Fe metabolism in vivo under normal physiological or Fe overloaded conditions. (Rahmanto *et al.*, 2007). We hypothesize on crosslink between the two disease based on common biochemical markers and metabolites thus elucidating further underlying mechanisms. In our previous work we speculated about immunological and biochemical crosslink between melanoma and AD based on common metabolites like phenoloxidase (PO), Tyrosinase (Rachkova and Dimitrov, 2008), where PO is marker of defense system Schmidt (1988), found in salivary gland, liver, eosinophyls, connective tissue. In addition MTf has been found in wide variety of human tissues: endothelium and reactive microglia of brains of AD patients (Jefferies et al., 1996; Yamaha et al., 1999). The highest MTf mRNA were found in the salivary gland (Richardson, 2000). Mtf is a membrane bound via Glucosyl phosphatidyl inositol (GPI) anchor. As a member of super family of transferrin (Tf), Mtf share 37–39% sequence homology with human serum Tf, human lactoferrin (Sekyere and Richardson, 2000). Lactoferrin (LF) is a component of first line of host defense. Its expression is up regulated in response to inflammatory stimuli and has anti inflammatory and immune modulating properties (Conneely, 2001). Lactoferrin is associated with oxidated stress in serum of patients with AD (Thome et al., 1997). We postulate the hypothesis of Mtf expression in melanoma and AD as a result of reactive oxygen species (ROS) and foreign toxins. Mtf may function as a immunomodulating factor via GPI anchor. Since melanocytes express high level of sAPP-beta amyloid precursor protein (Quast et al., 2003) we would like to discuss possible therapeutic intervention of phenolic compounds which prevents amyloid beta aggregation in AD (Hamaguchi et al., 2009) and are well known inhibitors of tyrosinase and pigment synthesis (Smith et al., 2009).

P6

Synthetic routes to 5,6-Dihydroxyindole oligomers: a tool for the bottom-up approach to eumelanin stucture

A. Pezzella, P. Manini, L. Capelli, A. Napolitano, M. d'Ischia
University of Naples Federico II, Italy

5,6-Dihydroxyindole (DHI) derived oligomers provide a most powerful investigative tool to elucidate the structure and physical/chemical properties of eumelanin and to unravel the complex process of supramolecular aggregation via a bottom up approach.[1–4] Until a few years ago access to DHI oligomers was based almost exclusively on controlled oxidative polymerization processes. However, several limitations inherent to the diverse bonding patterns in DHI coupling and the ensuing complexity of the reaction mixtures, prevented this approach from reaching a synthetic status Herein we report a convenient synthetic entry to DHI oligomers as the acetyl derivatives, including dimers (1-Ac, 2-Ac, 3-Ac),[5] a trimer (4-Ac)[6] and a tetramer (5-Ac). The approach is based on a unified procedure involving ortho-ethynylanilines as key intermediates.

References

1. Meredith P., Sarna T. (2006) Pigment Cell Melanoma Res. 19: 572–94.
2. d'Ischia M., Napolitano A., Pezzella A, Meredith P., Sarna T., (2009) Angew. Chem. Int. Ed., 48: 3914–21.
3. Pezzella A., Iadonisi A., Valerio S., Panzella L., Napolitano A., Adinolfi M., d'Ischia M. (2009) J. Am. Chem. Soc. 131: 15270–75.
4. Bothma J. P., de Boor J., Divakar U., Schwenn P. E., Meredith P. (2008) Adv. Mat. 20: 3539–42.
5. Capelli L., Manini P., Pezzella A., Napolitano A., d'Ischia M. (2009) J. Org. Chem. 74: 7191–4.
6. Capelli L., Manini P., Pezzella A., d'Ischia M. (2010) Org. Biomol. Chem. 8: 4243–45.

P7

Imaging the distributions of eumelanin and pheomelanin in human tissue

M. J. Simpson, J. Wilson, T. Matthews, S. Degan, W. Warren
Duke University, Durham, NC, USA

Melanoma diagnosis poses tremendous challenges in dermatopathology; recent studies show discordance rates in pathology as high as one in seven [1] and the severe consequences of missing a melanoma diagnosis has increased the number of biopsies taken and lowered the threshold for diagnosis of early melanoma [2]. This leads to unknown but likely significant societal costs and morbidity from associated unnecessary treatments. Clinicians need better technology to increase the specificity of current diagnostic techniques. We have previously reported a technique that resolves eumelanin and pheomelanin using nonlinear optics, specifically two-color pump-probe microscopy [3]. This technique, which is compatible with standard pathology procedure, has been used to image the microscopic morphology of the distribution of eumelanin and pheomelanin in human tissue slides. It has revealed that melanomas tend to have an inhomogeneous distribution of melanins and higher fractional eumelanin content compared to other lesions. Here we report on a variety of extensions of that work. Studies on the dependence of laser parameters (pump and probe wavelengths, polarization) reveal the effects of the increased ground state depletion signal in pheomelanin relative to eumelanin. We have quantified eumelanin and pheomelanin morphology using wavelet analysis and other image processing methods to further improve specificity. High resolution imaging has identified microstructure with variable eumelanin/pheomelanin ratios in individual melanosomes. We have demonstrated epi-mode detection and imaging in vivo (of lesions induced in human skin grafted to nude mice). Finally, we have demonstrated three-dimensional sectioning capabilities. As a result, it can be used for imaging thick slices of tissue (to a depth of around 120 μm), allowing a clinician to examine a large section of a lesion all at once. This has been demonstrated with a fresh, excised human mole, on which the rete ridges can be seen using melanin as a source of contrast. Taken together, these results provide new insights on the chemical morphology and biochemical heterogeneity of normal and diseased tissue, and provide valuable diagnostic information.

 © 2011 The Authors. Journal Compilation © 2011 John Wiley & Sons A/S

References

1. Shoo B. A. et al. (2010) J. Am. Acad. Dermatol. 62: 751.
2. Glusac E. J. (2011) J. Cutan. Pathol. 38: 264.
3. Matthews T. E. et al. (2011) Sci. Transl. Med. 3: 71ra15.

P8

Slaty mutation inverses the ratio of DHI to DHICA content of eumelanin in both mouse melanocytes and hair

Y. Niki, T. Hirobe, K. Wakamatsu, H. Ando, M. Yoshida, M. Ichihashi, S. Ito
Kobe Skin Research Institute, Kobe, Japan

Tyrosinase-related protein-2 (TRP2; slaty) is a DOPAchrome tautomerase (Dct) which is believed to determine the ratio of 5,6-dihydroxyindole-2-carboxylic acid (DHICA)- to 5,6-dihydroxyindole (DHI)-derived units in eumelanin but how the slaty mutation can impact the DHI/DHICA ratio remains unclear. In this study, both the quality and quantity of eumelanin in melanocytes and hairs from wild type (black) and Dct mutated (slaty) mice were analyzed after alkaline hydrogen peroxide oxidation, which leads to the formation of pyrrole-2,3,5-tricaboxylic acid (PTCA) and pyrrole-2,3-dicarboxylic acid (PDCA) as markers for DHICA and DHI respectively. Total melanin (TM) was determined by spectrophotometric measurement. We used primary melanocytes from dorsal epidermis of 0.5-day-old newborn mice after 14 days of cultivation and dorsal hairs from 5-weeks-old mice. DHICA-derived levels in eumelanin were estimated from PTCA/TM and PDCA/PTCA ratios in comparison to chemical synthetic eumelanins which were synthesized from various ratios of DHICA and DHI by tyrosinase oxidation. In both melanocytes and hair samples from slaty mice, TM and PTCA were decreased while PDCA was increased compared to wild type. Estimated DHICA-levels based on the PDCA/PTCA ratio was 72 and 24% in black and slaty melanocytes and 82 and 20% in the corresponding hairs. Similar results were obtained with PTCA/TM. These results demonstrate for the first time that mouse slaty mutation not only leads to a decrease in DHICA-melanin formation but also inverses the ratio of DHI- to DHICA-melanin in both dorsal melanocytes and hair samples.

P9

Quantum chemical study of 5,6-dihydroxyindole tetramers as eumelanin model molecules

H. Okuda, T. Sota, K. Koike, K. Monda, T. Nakamura, K. Wakamatsu, S. Ito
Fujita Health University, Toyoake, Aichi, Japan

It is widely known that eumelanin is a photoprotective brown-to-black macromolecule built produced by the oxidation of 5,6-dihydroxyindole (DHI) and/or other precursors. However, its three-dimensional (3D) structure as a macromolecule is less well understood. In this study, as a first step towards describing the macromolecular structure of eumelanin, we have carried out quantum chemical calculations for DHI tetramers as its oligomeric precursors. All quantum chemical calculations were carried out using Gaussian 03. The molecular structures have been optimized using Hartree–Fock theory with the 6-31G(d) basis set, and thermochemical effects have been calculated to estimate the molecular stability using the Gibbs free energies at 300 K. The electronic energies for the corresponding molecular structures have been estimated at the B3LYP/6-31++G(d,p) level of theory. All tetramers considered here are those with 2,4′- or 2,7′-homo linkages. Supposing that tetramers are formed via dimer–dimer couplings, we have to consider two types of 3D structure for the same semi-structural formula. This is because the dihedral angle may change depending on how dimers bond with each other. We call the two possibilities α-type and β-type here. Multiple oxidation states are considered by changing the number of electrons in each molecule. Our calculations have demonstrated the following. Among tetramers with 2,4′- (2,7′-)homo linkages, α- (β-)type molecules are more stable. The 2,4′-linked α-type is the most stable, followed by (in order) the 2,4′-linked β-type, 2,7′-linked β-type, and 2,7′-linked α-type. Each α- (β-)type molecule looks like a part of a helix (zigzag). For α-type molecules, each tetramer is just one lap of a helix, and the length of C4 (C7)–C2′′′ for 2,4′- (2,7′-)linked tetramer, which may be considered to determine the pitch of the helix, changes depending on its oxidation state.

P10

Characteristic roles for cochlear melanocytes in anti-oxidant responses related to and not related to their melanogenesis

S. Uehara, H. Yamamoto
Laboratory for DNA Data Analysis, Center for Information Biology (CIB)/DNA Data Bank of Japan (DDBJ), National Institute of Genetics, Mishima, Japan

Mammalian melanocytes are derived from neural crest cells, migrate not only to the skin but also to other tissues, such as outer layers of eyes, heart valves, and the inner ears. The functions of those non-cutaneous melanocytes are not clearly understood. Among non-cutaneous melanocytes, the inner ear melanocytes localized in the cochlear stria vascularis are known to be essential for the maintenance of hearing acuity. However, melanin synthesis (pigmentation) in the cochlea is less associated with auditory function. Thus, the functions of non-cutaneous melanocytes seem not to be identical to those of cutaneous melanocytes. We compared the expression patterns and enzymatic activities of melanocytes in the cochleae and on the skin of three types of mice, normally pigmented wild-type, albino and melanocyte-lacking mutants. Cochlear melanocytes have important roles in anti-oxidant responses in the normal (wild-type) cochleae that are correlated with the expression of glutathione S-transferase (GST) alpha (Gsta), which is lost in melanocyte-lacking cochleae. Concerning albino cochleae, which have melanocytes without melanogenesis, no differences were detected in anti-oxidant responses and Gsta expression compared with the wild-type. However, the expression patterns of another GST (Gstp) in albino cochleae were different from those of wild-type. GSTA was not detected in cutaneous melanocytes even in normally pigmented skin, but Gstp was expressed both in cochlear and in cutaneous melanocytes. Cochlear melanocytes have functions against oxidative stress. Both the existence and pigmentation of cochlear melanocytes are closely associated with the expression of different GST isoforms in the cochlea. Gsta expression has an essential function against oxidative stress in the cochlea, related to the existence of cochlear melanocytes, and is a specific function of cochlear melanocytes. Gstp expression is related to the pigmentation of cochlear melanocytes, and is a common function in cutaneous and cochlear melanocytes.

P11

Zinc free diet induced a release of melanosomes from choroidal melanocytes and an increase of lipofuscin in the retinal pigment epithelium of rats

S. Julien, A. Biesemeier, D. Kokkinou, O. Eibl, U. Schraermeyer
Institute for Ophthalmic Research, Section of Experimental Vitreoretinal Surgery, Tuebingen, Germany

Age-related macular degeneration (AMD), the leading cause of blindness in the Western world, is associated with lipofuscin

© 2011 The Authors. Journal Compilation © 2011 John Wiley & Sons A/S

accumulation whereas the melanosome content declines. Melanosomes are the main storage of zinc in the pigmented tissues. Since the elderly population, the most affected group for AMD, is prone to zinc deficit, we investigated the chemical and ultrastructural effects of zinc deficiency in pigmented rat eyes after a six-month zinc penury diet. Adult Long Evans (LE) rats were used. The control animals (eight LE eyes) were fed with a normal alimentation whereas the zinc-deficiency rats (eight ZD-LE eyes) were fed with a zinc-free diet for 6 months. Quantitative Electron Dispersive X-ray (EDX) micro-analysis yielded the zinc mole fraction of Retinal Pigment Epithelial (RPE) melanosomes. Light, fluorescence and electron microscopy, as well as immunohistochemistry were performed. The numbers of photoreceptor nuclei, of lipofuscin granules as well as of the cells found in the choroid of ZD-LE rats were quantified. EDX microanalysis showed that RPE melanosomes of untreated LE rats contained 0.04 ± 0.02 at % zinc. In ZD-LE rats, the zinc mole fractions were always at or below the minimum detectable mole fraction of 0.02 at % and therefore significantly lower compared to controls. In contrast, the zinc mole fraction of choroidal melanosomes did not change with respect to controls. The number of photoreceptor nuclei of ZD-LE rats was significantly decreased and the number of the lipofuscin granules was found to be significantly increased in ZD-LE when compared to control rats. Moreover, pigmented ED1-positive cells were detected in the choroid of ZD-LE animals. In pigmented animals, the zinc deficiency yields an accumulation of lipofuscin, pigmented macrophages in their choroids and a degeneration of photoreceptors. Moreover, we showed that zinc deficiency reduces the zinc mole fraction of melanosomes in the RPE.

P12

Involvement of Mitf in the development of retinal pigment epithelium and its possible regulators

D. Nishihara, A. Kawasaki-Nishihara, N. Tsukiji, H. Nakamura, H. Yamamoto
Graduate School of Life Sciences, Tohoku University, Nagahama, Japan

The retinal pigment epithelium (RPE), one component of the vertebrate eye, consists of a monolayer of melanin-pigment cells. Although the RPE is known to be indispensable for adult visual function, little is known about the molecular mechanisms underlying its development. In the early optic cup (OC) stage, the outer layer of the OC starts differentiating into the RPE. As eye development proceeds, the characteristic phenotypes of the RPE, such as melanin pigmentation and a monolayered structure, are gradually formed in a specific spatio-temporal pattern. Although some transcription factors, such as Mitf, are thought to be essential for these processes, the detailed underlying mechanisms for the regionalization and characterization of the RPE remain to be elucidated. Mice mutant for transcription factors known to contribute to normal eye development frequently have severe eye abnormalities. In such cases, with so many complicated phenotypes, analyses focusing on RPE development are not easily carried out. To elucidate how eye-related transcription factors function during RPE development in detail, we conducted gene transfection into the chick OC by electroporation. Using that method, the transfected areas could be limited (not the whole developing eye affected) and easily controlled. The resultant eye phenotypes are not too disrupted to analyze how RPE development is affected with the transfected molecules in specific areas. Here we show that several transcription factors are able to regulate RPE regionalization and characterization by controlling the expression and function of Mitf, also a key player for RPE development.

P13

Atomic force microscopy analysis of retinal pigment epithelium cells subjected to photodynamic stress

M. Sarna, M. Olchawa, A. Pilat, G. Szewczyk, K. Burda, T. Sarna
Department of Medical Physics and Biophysics, Faculty of Physics, AGH University of Science and Technology, Krakow, Poland

Retinal pigment epithelium (RPE) provides key metabolic support for the entire retina and is involved in biological renewal of photoreceptor outer segment (POS) membranes. Periodic phagocytosis of POS by RPE is critically important for proper function and survival of photoreceptor cells. Although melanin in normal human RPE contributes to visual acuity by absorption of light that could otherwise be reflected from the fundus and lead to spurious signals, the pigment may also play photoprotective and antioxidant functions. In this study, we analyzed inhibitory effects of oxidative stress, induced in cultured human ARPE-19 cells by photodynamic treatment, on the cell phagocytic activity and correlated the results with changes in morphology and nanomechanical properties of the cells examined with atomic force microscopy (AFM). Preliminary results of our study show that significant inhibition of phagocytic activity of ARPE-19 cells, induced by sub-lethal oxidative stress, is accompanied by dramatic changes in morphology of the cells. The modulatory effect of melanin in ARPE-19 cells, exposed to photodynamic stress, was analyzed by comparing survival of cells and their phagocytic activity before and after loading the cells with purified bovine RPE melanosomes. Our data indicate that cells containing phagocytized melanosomes were more resistant to photoinduced stress than cells containing control particles. This study has demonstrated that AFM is a method of choice for monitoring changes in morphology and nanomechanical properties of living RPE cells, under physiological conditions in vitro, in response to sub-lethal oxidative stress.

P14

Notch signaling is dispensable for mature melanocytes but essential for melanocyte stem cells

B. Sarode, U. Koch, K. Schouwey, L. Larue, V. Delmas, F. Beermann, F. Radtke
Ecole Polytechnique Fédérale de Lausanne (EPFL), Swiss Institute for Experimental Cancer Research (ISREC), Lausanne, Switzerland

Notch signaling is essential for cell growth, survival and differentiation. We previously showed that signaling through Notch1 (N1) and Notch2 (N2) receptors contributes to the maintenance of melanoblasts and melanocyte stem cells, and that is essential for proper hair pigmentation. The deletion of both receptors results in dose dependent hair graying, due to an elimination of melanocytes and melanocyte stem cells. Investigate the molecular mechanisms involved in N1 and N2 signaling which contribute to melanoblast maintenance and are involved in mature melanocytes. Using a genetic Cre-lox approach we deleted N1 and N2 in melanocytes and melanoblasts (Tyr-Cre::N1lox/lox, N2lox/lox). Subsequently, melanoblasts deficient for both N1 and N2 were isolated by FACS sorting from E16.5 embryos. Melanoblasts, identified as CD117+CD45− cell population, showed a significant reduction in the absolute numbers of cells isolated from N1N2 deficient embryos as compared to littermate control embryos. So far, the downstream target genes of Notch signaling involved in melanoblasts maintenance have not been identified. Therefore, we performed microarray analysis on N1N2 deficient melanoblasts and wild type (WT) controls. The array results are currently validated using qRT-PCR. Since Notch

 © 2011 The Authors. Journal Compilation © 2011 John Wiley & Sons A/S

deficient immature melanocytes (P1 to P3) are not viable we utilized an established in vitro assay to generate mature melanocytes carrying N1lox/loxN2lox/lox alleles. Using a lentiviral approach to transduce the mature N1lox/loxN2lox/lox melanocytes with CreERT2 construct, we will be able to identify and evaluate the molecular mechanism involved in Notch signaling in these cells. The deletion of both N1 and N2 was confirmed by Southern blot analysis and deletion PCR. In addition, qRT-PCR results showed a significant downregulation of Hes1. Our results revealed that there is no change in the proliferation rate in both WT and Notch deficient mature melanocytes. We also analyzed WT and Notch deficient melanocytes for changes in cell cycle progression and apoptosis and could not detect a significant difference between the two cell populations assessed. In addition, the cell adhesion properties between WT and N1N2 deficient mature melanocytes were not affected. Overall our results show that although Notch signaling is dispensable for the survival of mature melanocytes but it is required for melanocytes during embryonic stages.

P15

BRN2 phosphorylation regulates melanoblast migration and proliferation through PAX3 and MITF-M

I. Berlin, L. Denat, A.-L. Steunou, I. Puig, D. Champeval, S. Colombo, K. Roberts, E. Bonvin, Y. Bourgeois, V. Delmas, I. Davidson, L. Nieto, C. R. Goding, L. Larue
Institut Curie, Orsay, France

BRN2 is a POU domain transcription factor overproduced in human melanoma cell lines and involved in melanocyte proliferation and migration. The T361 and S362 residues of BRN2, located within the POU domain, are conserved throughout the POU proteins family. We investigated the importance of phosphorylation of these residues for migration and proliferation by mutation, replacing T361 and S362 in the wild-type form of BRN2 (BRN2TS) by two alanine residues (BRN2AA). BRN2TS was phosphorylated by PKA whereas BRN2AA was not. The T361 and S362 residues are the most important targets of this kinase. In vitro, BRN2TS overproduction induced proliferation and slightly repressed migration in murine melanocytes whereas BRN2AA overproduction repressed both proliferation and migration. We investigated the role of BRN2TS and BRN2AA in vivo, after generating transgenic mice overproducing either BRN2TS (Tyr::BRN2TS) or BRN2AA (Tyr::BRN2AA) specifically in the melanocyte lineage, using tyrosinase promoter. Tyr::BRN2TS mice showed a slight hyperpigmentation, whereas Tyr::BRN2AA mice had a white spot on the belly. These phenotypes were associated with changes in migration an dproliferation during establishment of the melanocyte lineage. Indeed, on day 13.5 of embryonic development (E13.5), Tyr::BRN2TS embryos had slightly more melanoblasts than wild-type embryos, which had more melanoblasts than Tyr::BRN2AA embryos strongly suggesting that T361 and S362 residues are involved in the proliferation of melanoblasts and melanocytes in vivo. BRN2 is known to regulate Pax3, Gadd45 and NotchL positively and Mitf-M negatively. Moreover, Pax3 and Mitf-M are implicated in migration and proliferation of the melanocyte lineage.

We thus investigated the regulation of Pax3 and Mitf-M by BRN2TS and BRN2AA at the molecular and genetic levels. Mitf-M transcription is repressed similarly by BRN2TS and BRN2AA whereas Pax3 transcription is induced by BRN2TS but repressed by BRN2AA. These results suggest that the regulation of proliferation and migration mediated by BRN2 involves Pax3 and Mitf-M and that BRN2 phosphorylation controls the regulation of proliferation mediated by Pax3.

P16

Endothelin receptor B2 mutation induces the suppression of proliferation and migration of melanoblasts from early embryogenesis in quail and chickens

T. Akiyama, A. Shinomiya, K. Kinoshita, M. Mizutani, T. Namikawa, S. Ito, Y. Matsuda
Department of Biology, Keio University, Yokohama, Japan

A signal transduction system of endothelins (EDNs) and their receptors (EDNRs) is known to be one of the strong factors to affect melanization in vertebrates. Aves have EDNRB2 in addition to EDNRB, as a paralog of it. The purpose of this study is to clarify the function of EDNRB2 in Aves. The Japanese quail line, 'panda' (s/s), with EDNRB2 mutation that was reported previously showed white plumage as a main feature. Recently, several chickens with white-plumage mutation were found in Japanese native colored lines. These white individuals were crossed with the 'albino' (recessive albino; ca/ca) and linkage mapping analysis was performed using F2 progenies having black eyes. We determined two types of the mutations in EDNRB2. The first type was identified from 'Minohiki' and 'Uzurao' lines and the mutation site was Cys244Phe. The second was isolated from 'Ehime-jidori' and 'Uzurao' with Thr8Ile, Thr15Ale and Arg332His substitutions in EDNRB2. The plumage colors were complete white in the former and partial white with mottling pattern in the latter. When we observed melanoblast proliferation during development, all of these EDNRB2 mutants showed clear suppression of the melanization in the integument and feather buds at the early developmental stage. Also, the organ culture of neural crests of these mutants showed that the quite less number of melanoblasts appeared from tissues than those of wild types and these cells didn't grow well even addition with EDN3. These results clearly demonstrated that EDNRB2 is an indispensable factor in melanoblast development and migration to the final locations in Aves. In conclusion, EDNRB and -B2 in Aves are seemed to share the roles of EDBRB in mammals and then EDNRB2 may play a role specifically on proliferation and migration of melanoblasts in their bodies.

P17

Gene expression profile of murine melanoblasts

D. Champeval, S. Colombo, F. Rambow, L. Larue
Institut Curie, Orsay, France

Studying development of melanoblasts, precursors of melanocytes, is challenging owing to their scarcity and dispersion in the skin embryo. However, this is an important subject because diverse diseases are associated with defective melanoblast development. Consequently, characterizing patterns of expression in melanoblasts during normal development is an important issue. This requires isolating enough melanoblasts during embryonic development to obtain sufficient RNA to study their transcriptome. ZEG reporter mouse line crossed with Tyr::Cre mouse line were used to label melanoblasts by EGFP autofluorescence. We isolated melanoblasts by FACS from the skin of E14.5-E16.5 embryos, and obtained sufficient cells for large-scale transcriptomic analysis after RNA isolation and amplification. We confirmed our array-based data for various genes of interest by standard Q-RT-PCR. We demonstrated that PTEN was expressed in melanoblasts but BRN2 was not although both are involved in melanomagenesis. We also revealed the potential contribution of genes not previously implicated in any function in melanocytes or even in neural crest derivatives. Finally, the Schwann cell markers, PLP1 and FABP7, were significantly expressed in melanoblasts, melanocytes and melanoma. This

© 2011 The Authors. Journal Compilation © 2011 John Wiley & Sons A/S

study demonstrate the feasibility of the transcriptomic analysis of purified melanoblasts at different embryonic stages and reveals the involvement of novel genes in melanoblast development.

P18

Flux balance analysis of melanogenesis pathway

K. Menaria
Department of Bioinformatics, MANIT, Bhopal, India

Proteins play the most important role in the world of Biology. These are macro molecules that influence every possible reaction in any living organism. It is a widely known fact that these biomolecules are made up of a combination of amino acids arranged in their most stable states of confirmation to produce a chain of polypeptides that fold in interesting ways to form the functional unit of living organisms. These proteins however are numerous in number, although they are formed by the combination of only twenty one amino acids. This copious number leads rise to difficulty in understanding their folding mechanism, functional properties, structural variance and other fundamentals. A large variety of techniques are in vogue lately and the techniques of Systems Biology stand out amongst them. Systems Biology is the systematic study of various levels of biomolecules like nucleotides (Genes and RNA molecules), polypeptides (proteins and enzymes) and organs. Systems Biology incorporates some of the best mathematical and modeling techniques to date to analyze the biological data. High throughput techniques like Micro-array analysis, SAGE, mass spectrometry, etc. produce humongous data to be analyzed every day. Systems Biology involves mathematical techniques that can be used to analyze these enormous biological data sets to predict significant information. The process of biological network reconstruction, followed by the synthesis of in-silico models describing their functionalities, is the essence of systems biology. Systems biology is not necessarily focused on the components themselves, but on the nature of the links that connect them and the functional states of the networks that result from the assembly of all such links. Metabalomics is the study of all the metabolic networks involved in a part or whole of an organism. It is an important branch of study which forms a part of Systems Biology. Flux Balance Analysis is an interesting modeling technique that is used to balance the flow of flux in a metabolic circuit. The functions of reconstructed networks are defined by the interconnections of their parts. Since these connections involve chemical reactions, they can be described by a stoichiometric relationship. The stoichiometric matrix, which contains all such relationships in a network, is thus a concise mathematical representation of reconstructed networks. This matrix comprises integers that represent time- and condition-invariant properties of a network. The FBA revolves around this stoichiometric matrix to optimize the output of a metabolic network. The current project deals with balancing the flow of flux (Flux Balance Analysis) of the biosynthesis of melanin (melanogenesis pathway). We strive to understand the optimized flow of flux in the melanin biosynthesis to enable the future researchers pursue the study of importance of various genes involved in the synthesis of melanin. We sincerely believe that this would be the first step in eliminating the ill effects of melanin production in human body, especially in diseases like cancer.

P19

A systems biology approach to in vivo dissection of the gene regulatory network (GRN) underlying melanocyte differentiation in zebrafish

L. Vibert, M. Nikkaido, E. R. Greenhill, R. N. Kelsh
University of Bath, UK

Neural crest cells can differentiate into an extensive range of derivatives. Understanding the factors driving fate specification of multipotent cells and maintaining the differentiated phenotype are key challenges in developmental biology. We are focusing on melanocyte differentiation in zebrafish. Two transcription factors, Sox10 and microphthalmia-associated transcription factor (Mitfa), are crucial for this process (Dutton et al., 2001; Elworthy et al., 2003). Fate specification of melanocytes depends upon Sox10 and Wnt signalling, to mediate regulation of Mitfa transcription. In contrast, the mechanism resulting in stable melanocyte differentiation remains unclear. In order to better define the GRN underlying melanocyte differentiation, we used a systems biology approach consisting of iterative cycles of genetic testing and mathematical modeling. This work suggested that Sox10 forms part of a Feed-Forward Loop repressing melanocyte differentiation that is relieved by Mitfa-dependent repression of Sox10. Our modelling also predicted an unknown factor, Factor Y, required for maintaining Mitfa expression. Comparison of mitfa expression in wild-type and mitfa mutant zebrafish provides biological evidence of this mechanism. Furthermore, overexpression studies provide an indication that Mitfa itself may contribute directly to this feedback. Wnt signalling has been shown to regulate mitfa expression during fate specification in mouse and zebrafish (Dorsky et al., 2000) and studies in mouse show that Wnt regulation might also contribute to maintenance of mitfa expression in differentiated cells. To test the hypothesis of Wnt signalling as Factor Y, a limiting factor regulating mitfa expression in differentiated melanocytes, we first show that Wnt signalling remains activate in melanocytes until 72hpf. We assess the effects of LiCL and BIO, two GSK3b inhibitors, on zebrafish embryos, showing by RT-qPCR that boosted Wnt signalling drives elevated mitfa expression. Treated embryos show increased melanocyte dendricity and altered melanocyte pattern, similar to recent observations in Xenopus (Kawasaki et al., 2005). Our data suggests that Wnt signalling is not limiting for melanisation, but that it contributes to mitfa expression and to melanocyte morphology. We conclude with a new definition of Factor Y as an ensemble of factors, likely to include both Mitfa itself and Wnt signalling, that maintain Mitfa expression in differentiated melanocytes.

P20

Genetic mapping of a rat dominant ventral spotting gene, downunder (Du), to chromosome 3

T. Kuramoto, M. Yokoe, T. Serikawa
Institute of Laboratory Animals, Graduate School of Medicine, Kyoto, Japan

The downunder (Du) mutation has been discovered in an Australian fancy stock and it displays ventral spotting [Pigment Cell Res. (2004) 17:451]. Inheritance of the ventral spotting is dominant, but it is not known whether the Du shows homozygous lethality, or on which chromosome the Du is mapped. Here, we examine lethality among embryos produced from intercross between Du/+ female and Du/+ male rats, and carry out genetic linkage mapping of the Du locus. We maintain the Du mutation using F344.Cg-Du congenic rats. To examine the embryonic lethality, we performed cesarean section of 12 Du/+ females mated with Du/+ males at P15 (n = 6) and P20 (n = 6). To map

 © 2011 The Authors. Journal Compilation © 2011 John Wiley & Sons A/S

the Du, we produced 98 F344 × (F344 × F344.Cg-Du)F1 backcross progeny. Twenty-six embryos were dead among 102 embryos harvested from 12 females. The rate of lethality is 25.5%, which suggests homozygous lethality occurs in Du/Du embryos. Du is mapped approximately 7.9-Mb between D3Got19 (20.8 Mb) and D3Rat190 (28.7 Mb), where 13 genes have already been mapped, but any gene has not been known to be associated with coat color in rats yet. The Du locus corresponds to the mouse chromosome 2 (40–48 Mb), where no mouse coat color mutation has been mapped neither Du is a novel spotting gene in rats as well as mice. Identification of Du will provide us valuable information about melanocyte differentiation and development.

P21

Coat pattern genetics in cats

C. B. Kaelin, X. Xu, L. Z. Hong, V. A. David, K. A. McGowan, G. S. Barsh, M. Menotti-Raymond
School of Medicine, Stanford University, Stanford, USA

Mammalian coat color patterns display a remarkable variety of form but share a common feature that color markings are spaced at non-random intervals. Differences in coat patterns among species present abundant opportunities to explore the processes driving morphological diversity. On the other hand, the periodic nature of coat patterns hints at a conserved mechanism that embodies a fundamental and largely unexplored question in developmental biology – how do characteristic, non-random structures form from a substrate without pattern? The remarkable morphological diversity of coat patterns is exemplified by variation displayed among the 36 extant wild cat species. Domestic cats also have a variety of characteristic coat patterns under the control of at least four genetic loci. Therefore, domestic cats provide a good model for identifying genetic components of pigmentation patterning and platform for interrogating the genetic basis of natural pattern variation in a wide range of other felid species. We demonstrate the utility of a forward genetics approach in domestic cats that takes advantage of emergent genomic resources, genomic partitioning, and next generation sequencing to identify pigmentation patterning genes in the domestic cat.

P22

Titration of p53 in dark skin mouse mutants causes a spectrum of pigmentary and hematologic phenotypes

K. McGowan, C. Park, S. Mendrysa, I. Weissman, G. Barsh
Stanford University, Stanford, USA

During the course of a large-scale mutagenesis project in mice with defects in skin color, we identified missense mutations in Ribosomal protein S19 (Rps19) and Ribosomal protein S20 (Rps20) in two dark skin mutants. Mutations in a third ribosomal protein, Ribosomal protein S6 (Rps6), produced a similar, albeit more severe, dark skin phenotype. Genetic and molecular studies show that the transcription factor p53 is both necessary and sufficient for the pigmentation phenotype, and that skin darkness correlates with the degree of p53 activation in the skin. Using dark skin as an entry point, we found that the ribosomal protein mouse mutants also exhibit a spectrum of hematologic phenotypes that depend upon the underlying ribosomal protein mutation and genetic background. More importantly, like skin color, blood parameters correlate with the degree of p53 activation in bone marrow precursors. Additional work in mice based on mutations of Mdm2, a negative regulator of p53, suggests that stabilization of p53, is indeed, sufficient to induce changes in skin color and bone marrow maturation. Thus, titration of p53 may explain the pleiotropy and spectrum of phenotypic severity observed in human patients with ribosomal protein mutations, and supports an emerging paradigm in which activation of p53 gives rise to a variety of developmental or disease-related phenotypes depending on the amount and tissue-specific context in which activation occurs.

P23

Transmission electron microscope of fetal scalp melanocytes

R. Zhang
Department of Dermatology, First Affiliated Hospital, Nanjing Medical University, Nanjing, Bengbu, China

To observe the melanocytes in fetal scalp, their dendrites, and the ultrastructure of the melanin granules found in these cells under transmission electron microscope Human fetal skin was obtained from induced abortion of 140–150 day estimated gestational age. The specimen was washed in PBS and fixed in 2.5% glutaraldehyde and 0.1 M phosphate buffer (pH 7.2) for at least 2 h. The postfixation was in 1% osmium tetroxide and same buffer for 1 h followed by staining for 30 min in 1% uranyl acetate and 50% ethanol. The specimen was dehydrated using serial alcohol and acetone incubations and then embedded in spurr resin. A Sorvall MT-2B ultramicrotome was used to cut the scalp to 80 nm. Sections were stained with uranyl acetate and lead citrate. Grids were viewed in a Hitachi H-7000 transmission electron microscope at an accelerating voltage of 80 kV. The melanocytes located in the epidermis of the fetal scalp presente as clear cytoplasm and heterochromatic nuclei with various stages of melanosomes, most of them were immature. These cells consisted of abundant mitochondrion, endocytoplasmic reticulum and ribosome. Melanosomes transferred to neighbouring keratinocytes were singly distributed. The melanocytes in the hair follicles were rare, only parts of two melanocytes were observed, which contained most II–III stages of melanosomes. In this study, we also observed the melanosomes which presenting as degradative conglomerates within phagocytic vacuoles of the keratinocytes. Neither individual premelanosomes nor melanosomes occurring free in the keratinocytes were observed. The ultrastructure of melanocytes in fetal hair follicles is different from that in epidermis. The degradative conglomerates within phagocytic vacuoles in keratinocytes in dermal papilla maybe suggest the fate of hair bulb melanocytes during hair cycle.

P24

Association of the melanogenesis genes with Japanese skin color

Y. Abe, H. Yutaka, T. Gen, T. Suzuki
Yamagata University School of Medicine, Yamagata, Japan

There is a high degree of variation in color and skin type apparent within Japanese population with normal range of skin color. However little is understood about the genetic and molecular basis of light skin color in Japanese population. We aimed to determine the genetic variants of skin color variation in Japanese population. A total of 456 unselected Japanese females. The subjects have lived in Yamagata prefecture and were without pigmented disorders. The skin color (melanin index) was measured using a portable spectrophotometer with an analysis program (CM-2600d and CM-SA, Konica Minolta Sensing Inc.). As a new unit containing the spectrometer and the program has been developed for measurement of melanin content in skin. A total of four candidate genes were selected which are reported to play a role in pigmentation through melanin biosynthesis and

© 2011 The Authors. Journal Compilation © 2011 John Wiley & Sons A/S

can be responsible genes for oculocutaneous albinism (OCA), including TYR, OCA2, SLC45A2, MC1R. The choice of variants were refined by looking at frequency in the Japanese population. Analyses of association between variants and melanin index was investigated by multiple regression analysis with genotype, age used as independent variables. We found that the non-synonymous variant rs74653330 (Ala481Thr) and rs1800414 (His615Arg) located within the OCA2 gene, was significantly associated with melanin index in our sample. Our results suggest that genetic variations of the responsible genes for the OCA are important determinants for skin color in Japanese population.

P25

Diversity of skin colour in Indian women

E. Mauger, J. Latreille, A. Porcheron, C. Guinot, E. Tschachler, F. Morizot
CERIES, Neuilly sur Seine, France

India, the 2nd most populated country in the world after China, exhibits a wide geographical and cultural diversity. The aim of this study was to investigate skin colour variations on 804 Indian women living in Mumbai (20–70 yr old) with phototype ranging from II to V. Skin colour was measured on four skin sites (forehead, cheek, forearm, and inner upper arm), using a spectrocolorimeter CM 2600d (Minolta, Japan). The results were expressed in the CIELab colour system, and afterwards Individual Typological Angle values (ITA) were calculated. In addition, spectral reflectance measurements across the visible waveband (400–700 nm, 10 nm interval) were also recorded. The range of ITA on each skin site was very large: minimum and maximum values on the cheek were −54.3° and +51.1° respectively, which highlights the diversity of skin colour in Indian women. Moreover, in all phototypes, the forehead was found to be the darkest site, which may be attributed to various factors such as chronic sun-exposure and hyperpigmentation disorders (melasma, mechanical-induced hyperpigmentation…). Finally, as expected, an age-related increase in facultative pigmentation was found that occurs earlier in phototypes II–IV than in phototype V (20–30 versus 50–59 yr), whereas a decrease in constitutive pigmentation occurs in the same class of age in all phototypes (i.e. 40–49 yr). Our results highlight the uniqueness of Indian population groups to perform genome wide association study to explain variation in pigmentary traits.

P26

Confirmation that knock down of NCKX5, a gene that regulates natural variation in human skin colour, perturbs lipid and sterol gene expression in human melanocytes

S. Wilson, T. Dadd, F. L. Lim, R. Ginger, M. R. Green
Unilever, Sharnbrook, UK

Natural variation in human skin colour is determined by genetic variation in a very few genes. Variation of a non-synonymous single nucleotide polymorphism (pA111T, rs1426654) in one of these genes (SLC24A5) is associated with marked differences in constitutive skin colour in peoples from South Asia. NCKX5 is expressed in melanocytes and functions as a potassium-dependent sodium-calcium exchanger. Using NCKX5-specific antibodies alongside trans-Golgi-network (TGN) disrupting treatments we have confirmed that NCKX5 is a TGN resident protein and can find no evidence of its expression in melanosomes. When heterologously expressed the 111Thr variant of NCKX5 confers significantly lower ion-exchange activity than the Ala111 variant, a change which is proposed to regulate the lower melanogenic activity in individuals of lighter skin colour. Using genome wide microarrays following knockdown of SLC24A5 we surprisingly found that the expression of genes associated with melanogenesis were not altered with the notable exception of MC1R. Unexpectedly the expression of a number of sterol and cholesterol homeostatic genes were changed and the total cholesterol content of NHM was increased. Using quantitative pcr we now confirm the perturbation of genes involved in sterol and cholesterol metabolism, MC1R and also of the related exchanger protein NCKX4. Cholesterol has previously been identified as a potential melanogenic regulator and is known to be essential for vesicular budding at the TGN. Our data imply that SLC24A5 affects natural variation in skin pigmentation through a TGN resident ion exchange mechanism and a mechanistic route regulating intracellular sterol/cholesterol levels in melanocytes.

P27

Melanogenesis stimulates HIF-1α expression and accumulation of downstream genes mRNA with attendant changes in cellular metabolism

T.-K. Kim, Z. Janjetovic, D. L. Peacock, S. N. Tolkachjov, R. M. Slominski, W. Li, T. N. Seagroves, A. T. Slominski
University of Tennessee Health Science Center, Memphis, USA

To study the effect of melanogenesis on HIF-1α expression and attendant pathways, we used two stable melanoma lines (Bomirski hamster Abc1 and human SK-Mel188) in which the amelanotic versus melanotic phenotypes are dependent upon the concentration of melanogenesis precursors in the culture media. To induce melanin pigmentation in amelanotic (white) cells, Ham's F10 medium (low in tyrosine) was supplemented with 400 μM of L-tyrosine and then changed to DMEM medium (high in tyrosine, phenylalanine and tryptophan) after 3 or 4 days, respectively. The cells were harvested and processed for western blot (WB) and real-time PCR (rtPCR) or intact (live) cells were analyzed by high resolution magic angle spinning (HRMAS) nuclear magnetic resonance (NMR). The induction of melanin pigmentation led to significant up-regulation of HIF-1α, but not HIF-2α, in heavily melanized cells of both lines. As controls for HIF-1α expression, HIF-1α wild type (WT) or knockout (KO) cell extracts were included that were derived from the polyoma virus middle T transgenic mouse (PyMT). HRMAS-NMR showed that these effects were associated with changes of glucose and sodium acetate metabolism in intact cells. To further investigate this phenomenon we measured HIF-1α dependent gene expression in human melanoma cells. RtPCR showed significant upregulation of mRNA for pyruvate dehydrogenase kinase 1 (PDK1), sodium hydrogen exchanger (NHEI), BLCL2/adenovirus E1B 19 kDa interacting protein 3 (BNIP3), aldolase A (ALDOA), lactate dehydrogenase A (LDHA), hexokinase 2 (HK2), glucose transporter type 1 (GLUT-1), and vascular endothelial growth factor A (VEGFA), while having no effect on monocarboxylate transporter 4 (MCT4). Several of these genes are known to be regulated directly by HIF-1. The most significant stimulation was observed for BNIP3, NHEI and PDK1. In conclusion, induction of the melanogenic pathway leads to robust changes in genes controlling glucose and sodium acetate metabolism concomitant with a dramatic increase of HIF-1α protein expression, leading to up-regulation of many downstream genes (except MCT4) with relative most significant stimulation seen for NHEI, BNIP3 and PDK1.

 © 2011 The Authors. Journal Compilation © 2011 John Wiley & Sons A/S

P28

The proliferative response of melanocytes to sunlight

E. Hacker, Z. Boyce, M. Kimlin, S. Vaartjes, N. Hayward
Institute for Molecular Bioscience Brisbane, Australia

Over 434 000 non melanoma and 10 000 melanoma skin cancers are removed in Australia each year. That is approximately 1200 people every day having a skin cancer removed. This costs the Australian health system $300 million annually. Survival rates from melanoma are high if the disease is detected early, however once the disease has progressed to metastatic melanoma, it is usually fatal. Melanocytes are the precursor cells to melanoma and sunlight is the principal environmental causal factor for this group of cancers, although there is increasing evidence that the effect of sunlight on melanocytes is not the same for all people. The objective of this study was to measure the response of melanocytes to solar simulated ultraviolet radiation and to test whether these responses are modified by constitutional genotype, host phenotype or sunscreen. We recruited 57 healthy volunteers and exposed several small areas of their lower back to a mildly burning dose of solar simulated ultraviolet radiation with sunscreen applied to one site for comparison. Biopsies were taken from these sites at time-points following ultraviolet radiation exposure and immunohistochemistry was used to assess the level of melanocyte proliferation and cellular migration. This study was designed to improve our understanding of the interplay between sun exposure, genetic susceptibility and melanoma risk. We will present our preliminary findings and look to shed some light on what happens to melanocytes 'in vivo' following exposure to ultraviolet radiation, and address whether phenotypic factors or the use of sunscreen, modify these effects.

P29

UVA radiation induces melanogenesis through modulation of phase II antioxidant enzymes: the protective effect of gallic acid

U. Panich, S. Limsaengurai, T. Onkoksoong, P. Akarasereenont
Department of Pharmacology, Faculty of Medicine Siriraj Hospital, Bangkok, Thailand

Ultraviolet A (UVA) has been recognized as a major factor for skin hyperpigmentation through increased melanogenesis. While melanin exhibits photoprotective properties, its overproduction could cause genotoxicity probably leading to melanomagenesis, especially in lightly pigmented skin. UVA-induced oxidative stress may play a role in melanogenesis and thus improving antioxidant defenses may be useful in inhibition of abnormal melanin synthesis. Phase II antioxidant enzymes including γ-glutamate cysteine ligase (γ-GCL), the rate-limiting enzyme for GSH synthesis, and glutathione S-transferase (GST) are essential for the skin in protecting against photooxidative stress. Therefore, attempts have been made to study the roles of antioxidants including gallic acid, a phenolic present in various botanicals, in protecting against UVR-induced melanogenesis in order to develop effective depigmenting agents. This study aimed to investigate antimelanogenic effects of gallic acid (up to 60 μM) on lightly pigmented human melanoma (G361) cells irradiated with UVA with respect to modulation of γ-GCL and GST activity and mRNA. Melanin synthesis, tyrosinase activity, GSH content and GST activity were determined using spectrofluorophotometric methods as well as γ-GCL and GST mRNA levels were assessed using real-time RTPCR. Gallic acid was capable of inhibiting UVA-induced melanin production and tyrosinase activity in relation to restoration of GSH levels and GST activity as well as upregulation of γ-GCL and GST mRNA in G361 cells. However, UVA radiation exhibited time-dependent effect on γ-GCL and GST mRNA expression as downregulation of γ-GCL and GST mRNA levels was observed at 2-h post-irradiation but recovery of the mRNA levels was achieved by 4 h. In conclusion, upregulation of phase II enzymes including γ-GCL and GST could be a possible mechanism by which gallic acid provides protection against UVA-mediated melanogenesis. This work was supported by Faculty of Medicine Siriraj Hospital, Mahidol University, Thailand.

P30

The Effects of visible light and UV exposure on skin pigmentation in vitro

C. B. Lin, N. Chen, D. Rossetti, Y. Hu, J. Zhang, P. Bargo, F. Liebel, T. Chen, M. Seiberg
Johnson & Johnson, Skillman, USA

UV irradiation is a major environmental factor that affects the skin, leading to increased skin pigmentation (tanning), reduced extracellular matrix quality (wrinkles, sagging), skin carcinogenesis and immuno-modulation. The darkening effects of different UV wavelengths are heavily documented, however little is known about the effects of visible light on skin pigmentation. Visible light induced darkening of human skin, which was most noticeable in darkly pigmented skins. Therefore, we examined the possible effects of visible light on pigmentation production and deposition using in vitro model systems, and compared the relative efficiency and kinetics of UVA, UVB and visible light in this process. Exposure to UVA, UVB or visible light induces visible darkening and increases melanin deposition in pigmented epidermal equivalents. The induction of melanin deposition by visible light and UVA was more evident at earlier time points, while the increased melanin deposition induced by UVB was more significant at later time points. Similar pigment induction patterns by UVA, UVB and visible light were reproducible in human skin explants. UVA, UVB and visible light induced comparable levels of melanin deposition in skin explants at earlier time points. However, UVB stimulated the highest level of melanin production and deposition at late time points. The observed different kinetics of melanin induction by UVA, UVB and visible light may reflect their possible different mechanisms of action in regulating skin pigmentation. The induced pigment production observed in our studies was skin color dependent, with the induction of melanin more evident in darkly-pigmented versus lightly pigmented skins.

It was also observed that UVA induced tissue damages in lightly pigmented skin, but not in darkly pigmented skin, suggesting that darker pigment better protects skin from UVA damage. QPCR analyses demonstrated a similar correlation in the induction of tyrosinase and melanocortin-1 receptor expression. Topical pretreatment of human skin explants with 2.5% non-denatured soybean extracts reduced both basal, UV and visible light-induced melanin levels. These results shed lights on the mechanism of sunlight-induced tanning, and demonstrated the contribution of visible light (in addition to UVA) to the early sign of tanning.

P31

Microarray analysis of microRNA modulation in UVB stimulated human melanocytes

K. Lazoul, A. Soleyan, R. Kurfurst, J.-H. Cauchard
LVMH Recherche, St Jean de Braye, France

MicroRNAs are single-stranded regulatory RNAs of 18–25 nucleotides length generated from endogenous transcripts that form local hairpin structures. miRNAs occur naturally within cells to regulate gene expression at the post-transcriptional level. Evidence indicates that miRNAs play essential roles in embryo-

© 2011 The Authors. Journal Compilation © 2011 John Wiley & Sons A/S

genesis, cell differentiation, skin aging and pigmentation control. In this study, we have investigated miRNAs expression profiles in human normal melanocytes in response to UVB exposure. The miRNAs profiling was carried out with TaqMan miRNA assay from Applied Biosystems. We have found that more than 150 miRNA were expressed in melanocytes in basal conditions. In a 24 h time-course analysis after UV exposure, 50% of miRNA showed a variation in their expression levels. Among the miRNA modulated by UVB induction, 35 are implicated in control of tyrosinase activation cascade and along them 11 in tyrosinase (TYR) or tyrosinase-related protein (TRP1) gene expression control. Further analysis revealed that in a global manner, TYR and TRP1 miRNAs expression levels are increased in UVB stimulated melanocytes in comparison with sham control. By qRT-PCR analysis we confirmed that upregulation of tyrosinase miRNAs, in response to UVB, is correlated with downregulation of tyrosinase gene expression. In conclusion, dynamic changes in miRNAs expression occurred during melanocytes UVB response, with an overall increase in TYR and TRP1 specific miRNAs levels indicate than the use of gene silencing effectors such as TYR and TRP1 miRNAs could provide a powerful strategy for melanogenesis regulation control.

P32

Multiphoton microscopy of pigmented reconstructed epidermis: assessment of 3D pigmentation modulation

H. Ait El Madani, F. Girard, A. Black, L. Gauchet, A. Krief, C. Gomes, H. Nocairi, F. Leroy, P. Sextius, A. Colonna
L'Oréal, Aulnay Sous Bois, France

In situ imaging methods based on Multiphoton Microscopy (MMP) were developed to assess the effects of molecules used to modulate epidermal melanin content. MMP is a non invasive optical imaging technique that allows the three dimensional (3 D) structures of skin to be investigated, even at a sub-cellular resolution. It provides complementary information namely two-photon excited fluorescence (2PEF) and Second Harmonic Generation (SHG). 2PEF signals are emitted by skin endogenous chromophores such as NADP(H), flavins, keratin, melanin or elastin, whereas SHG signals are specific to dense and ordered macromolecular structures such as fibrillar collagen. Here, MMP allowed a non-invasive analysis of the 3 D distribution of melanin at different levels in epidermis, thanks to melanin endogenous fluorescence. First, use of different pigmented reconstructed epidermises showed that MMP could discriminate 'in vitro phototypes', i.e. phototypes II, IV and VI. Second, the efficacy of a whitening compound was tested on pigmented reconstructed epidermises and recorded MMP images were analyzed with specifically-developed software combined with statistical analysis. A significant decrease in epidermal melanin was demonstrated in samples treated with the whitening compound. In this study, MMP appeared to be a relevant methodology to visualize and quantify the 3 D distribution of melanin in epidermis which makes it a promising new diagnostic tool to characterize and evaluate whitening compounds.

P33

Role of N-glycosylation in human melanocortin 1 receptor trafficking and function

C. Herraiz, C. Jiménez-Cervantes, J. C. García-Borrón
Department of Biochemistry and Molecular Biology, School of Medicine, University of Murcia, Espinardo, Murcia, Spain

The melanocortin 1 receptor (MC1R), a major determinant of skin pigmentation and phototype, is a Gs protein-coupled receptor that mediates the actions of a-melanocyte-stimulating hormone on melanocytes. MC1R signaling through the cAMP and ERK pathways is critical for melanocyte proliferation and differentiation. MC1R has two N-glycosylation targets, Asn15 and Asn29 in its extracellular N-terminus. MC1R is a glycoprotein with an unusual sensitivity to endoglycosidase H (EndoH) digestion. However, the occupancy and functional importance of each glycosylation sequon is unknown. To analyze the role of glycosylation in MC1R function, we prepared glycosylation-deficient mutants by site-directed mutagenesis of either Asn15 or Asn29 to Gln and we analyzed their ligand binding, functional coupling and intracellular trafficking properties. We also compared the functional properties of wild type (WT) MC1R expressed in heterologous cell lines defective in specific steps of the N-glycosylation pathway. Finally, we analyzed the effects of pharmacologic interference with glycan chain addition or trimming on the cell surface expression and function of endogenous MC1R in human melanoma cells. We demonstrate that MC1R is N-glycosylated at both Asn15 and Asn29, with structurally different endoglycosidase H-sensitive glycans of high-mannose or hybrid types. The functional effects of occupancy of each glycosylation site are also different. N-glycosylation is not necessary for high affinity agonist binding or functional coupling but has a strong effect on the availability of MC1R molecules on the plasma membrane, most likely by a combination of improved forward trafficking and decreased internalization. However, whereas glycosylation of the 15NST17 sequon is dispensable, occupancy of the 29NQT31 site is required for normal plasma membrane density and full signalling through the cAMP. Surprisingly, coupling to the mitogen-activated kinases ERK1 and ERK2 was similar for glycosylation mutants and WT receptor. MC1R variants exhibit different degrees of glycosylation without a simple correlation with their functional status or intracellular trafficking, but their sensitivity to EndoH is comparable with WT. Thus EndoH cannot be used to assess trafficking of MC1R variants. Our data underscore exceptions for major rules concerning the effects of Pro residues on N-glycosylation and the resistance to EndoH of mature glycoproteins.

P34

Melanocyte dendrites penetrate through a microporous membrane filter and generate large pigment globules containing multiple melanosomes which transfer to keratinocytes below

H. Ando, Y. Niki, M. Ito, K. Akiyama, M. S. Matsui, D. B. Yarosh, M. Ichihashi
Okayama University of Science, Okayama, Japan

We recently reported that co-cultures of normal human melanocytes and keratinocytes separated by a microporous membrane filter demonstrated melanosome transfer. Here we show a model in which melanocyte dendrites penetrating through a membrane filter generate pigment globules containing multiple melanosomes along the surface of the dendrites. Many pigment globules having a diameter larger than the pore size of the filter were observed by electron microscopy. The pigment globules were connected to the filopodia of melanocyte dendrites, previously shown to be conduits for melanosomes, and appeared to have been grown layer-by-layer in the filopodia. The pigment globules were then released into the extracellular space from the dendrites, captured by the microvilli of normal human keratinocytes which incorporated them, and then the melanosomes within the pigment globules were distributed primarily in the perinuclear area of the keratinocytes. These results suggest that the pigment globules containing large number of melanosomes play a pivotal role in melanosome transfer from normal human melanocyte to keratinocyte.

 © 2011 The Authors. Journal Compilation © 2011 John Wiley & Sons A/S

P35

Identification and characterisation of the MITF-interactome

T. Strub, D. Koludrovic, I. Davidson
IGBMC, Illkirch, France

The transcription factor MITF plays a pivotal role in melanoma. We previously performed profiling of MITF genomic occupancy by ChIP-seq in human 501Mel cells and identified MITF regulated genes by RNA-seq following siRNA-mediated MITF knockdown. This approach showed that loss of MITF leads to both down- and up-regulation of target genes involved in DNA replication, repair and mitosis, invasion and metastasis, indicating that it acts as a transcriptional activator or repressor in a promoter-specific manner. The identification of MITF partners proteins, and characterisation of their role in MITF-mediated gene regulatory networks may explain how it exerts this 'dual function'. We have generated 501 mel cells expressing Flag-HA tagged MITF and performed tandem affinity purification followed by mass-spectrometry of both the soluble nuclear and chromatin associated MITF fractions. Using this approach, we identified many of known partners (for example b-catenin and subunits of the BRG1 chromatin remodeling complex). We also show that MITF forms heterodimers with TFE3, TFEB and TFEC in melanoma cells suggesting that MITF does not occupy all of its genomic sites as a homodimer. In addition, we identify a set of other factors that may potentially act as transcription co-activators and repressors. Interestingly, we identified novel potential MITF-interactors that are directly involved in the DNA replication and repair processes. This suggests that MITF may regulate DNA repair and genome stability not only through its ability to regulate the expression of genes involved in this processes as we previously reported, but also in a much more direct manner.

P36

In vivo role of serine-73 phosphorylation of the transcription factor MITF: effects on coat color in mice with targeted mutations

J. Debbache, J. Pickel, H. Arnheiter
Mammalian Development Section (NIH/NINDS), Bethesda, MD, USA

The transcription factor MITF is critical for melanocyte proliferation, differentiation, survival and maintenance and for formation and metastasis of melanoma. MITF proteins constitute a family of isoforms generated by alternative promoter choice, alternative splicing and post translational modifications including phosphorylation at serine-73 which affects MITF activity in vitro. Nevertheless, targeted mutations in mice have not so far been able to delineate the role of serine-73 phosphorylation because changes in the codon for serine-73 resulted in efficient exclusion of exon 2B which harbors this codon. Therefore, we re-engineered knock-in mice in which exon 2B exclusion is prevented by targeted mutations in the alternative exon 2B splice donor and which carried either a wild type serine, an alanine, or a phosphomimetic aspartate at position 73. Mice homozygous for these mutations express MITF mRNA levels at about 70% of those encountered in wild type mice when measured in the heart (an organ not overtly affected by Mitf mutations). MITF protein levels, on the other hand, vary with the mutations at position 73. Furthermore, the mutations affect pigmentation: homozygosity for alanine at position 73 is associated with normal pigmentation, homozygosity for aspartate with a small white belly spot, and, interestingly, homozygosity for wild-type serine with a large white belly spot. When combined in compound heterozygotes with the extant semi-dominant MitfMi-wh mutation, the alanine mutation creates a darker coat than the aspartate mutation, darker even than the coat of MitfMi-wh/Mitf+ heterozygotes. In combinations with the semi-dominant allele Mitfmi or the null allele Mitfmi-vga9, the alanine mutation creates minor white spotting, the wild type serine completely white animals, and the aspartate mutation an intermediate phenotype. These genetic results indicate that the forced incorporation of exon 2B affects melanogenesis depending on the amino acid at position-73 and hence support previous in vitro findings showing that exon 2B and residue 73 regulate cell proliferation and differentiation. To substantiate the genetic findings further, we currently investigate whether the new mutations affect melanocyte proliferation or differentiation, both in vivo and in vitro in neural crest cell cultures and in cells transfected with constructs allowing tamoxifen-regulatable activation of the mutant proteins.

P37

Melanogenesis mediated by the preservative-induced release of the macrophage migration inhibitory factor in a 3D epidermal model

S. Ishiwatari, T. Fujita, A. Enomoto, S. Matsukuma
FANCL Corporation, FANCL Research Institute, Yokohama, Japan

Melanogenesis in the skin is stimulated in response to various environmental factors such as UV radiation, environmental pollutants, and chemical compounds. Among these factors, our focus is on agents that are absorbed into the skin through daily topical application. We previously reported that certain preservatives increased the melanin content in a reconstituted 3D human epidermal model (MelanoDermTM). In this study, to investigate the possible mechanism by which such preservatives enhance melanin synthesis, we examined the effects of methyl lparaben (MP) on the protein profile in a 3D epidermal model by performing 2D electrophoresis 12 days after MP exposure. The results revealed that approximately 31 proteins probably underwent changes due to MP exposure. These candidate proteins and proteins, which are reported to have an association with melanogenesis, in normal human keratinocytes (NHEKs), normal human melanocytes (NHEMs), and a 3D epidermal model were analyzed by ELISA or immunoblotting after MP exposure. One of the markedly altered proteins was macrophage migration inhibitory factor (MIF). MIF secretion into the culture medium increased notably in the NHEKs, NHEMs, and 3D epidermal model after MP exposure. According to a recent study, MIF mediates melanogenesis mainly by activating protease-activated receptor-2 (PAR-2) in keratinocytes following exposure to UVB radiation. PAR-2 is a key regulatory factor in keratinocytes, as it mediates melanosome transfer. Therefore, PAR-2 expression in keratinocytes was analyzed after MP exposure, and it was observed that MP enhanced PAR-2 expression in keratinocytes. Furthermore, induction of tyrosinase and tyrosinase-related protein 1 was confirmed in the NHEMs and 3D epidermal model. These data suggest that MP affects both keratinocytes and melanocytes and may stimulate melanogenesis via the MIF pathway.

P38

Wnt inhibitory factor (WIF)-1 promotes melanogenesis in normal human melanocytes

H. R. Kim, J. Y. Lee, S. Y. Park, H. Y. Kang
Ajou University School of Medicine, Suwon, Korea

Wnt signaling has been implicated in melanocyte biology. Recently, we found that Wnt inhibitory factor-1 (WIF-1) gene was significantly up-regulated in melasma skin. We investigated the effect of WIF-1 on melanogenesis of human melanocytes.

© 2011 The Authors. Journal Compilation © 2011 John Wiley & Sons A/S

Human melanocytes were treated with recombinant WIF-1. Cell proliferation and pigmentation were assessed. To further confirm the WIF-1 functions on melanocytes, cells were transfected with a WIF1 expressing vector. WIF-1 treatment inhibited cell proliferation and increased melanin contents and tyrosinase activity in human melanocytes. WIF-1 transfection also induced melanogenesis in melanocytes. WIF-1 remarkably increased the expressions of tyrosinase and MITF protein. WIF-1 stimulates melanogenesis in normal human melanocytes.

P39

Bone morphogenetic protein-6 induces melanogenesis and melanin transfer in human skin cells

S. K. Singh, W. A. Abbas, D. J. Tobin
Centre for Skin Sciences, School of Life Sciences, University of Bradford, Bradford, UK

Bone morphogenetic proteins (BMPs) represent a large family of multi-functional secreted signaling molecules. It has previously been reported that BMP-2 and-4 skin pigmentation by down-regulating the expression and activity of tyrosinase, to reduce melanogenesis in epidermal melanocytes (MC). However, the role of other BMPs (and their antagonists) in melanogenesis and melanin transfer has not yet been explored. We evaluated the role of BMP-6 in melanogenesis and melanin transfer using assays for melanin determination, tyrosinase activity, SEM, RT-PCR, siRNA knockdown, Western blotting, and in situ double immunofluorescence. BMP-6, and antagonist Sclerostin were variably detected in both MC and keratinocytes (KC) in human epidermis. BMP-6 markedly stimulated melanogenesis in MC by up-regulating tyrosinase protein expression and activity. BMP-6 increased melanosome transfer from MC to KC via a dose-dependent stimulation of Myosin-X expression and associated MC filopodia This was confirmed using siRNA knockdown of BMP receptor (BMPR1A/1B), as well as by incubation of MC/KC co-culture in the presence of Sclerostin. To understand the signalling involved in these BMP-6-mediated effect, BMP-6 was found to use the p38 MAPK pathway to regulate melanogenesis in human MC independent of Smad pathway. By contrast, the p38 MAPK and Smad pathways were both involved in BMP6-mediated melanin transfer. In summary, these data indicate complex regulation of MC by different members of the BMP family, including in the regulation of MC melanogenesis and melanin transfer to KC. Further differential control may be exerted by the presence and relative concentration of different BMPs, their respective antagonists, and Smad inhibitors.

P40

Sema4D, the ligand for Plexin B1, is a proliferation and survival factor for normal human melanocytes, and down-regulates the activity of c-Met

J. Soong, Y. Chen, E. Terushkin, G. Scott
University of Rochester School of Medicine, Rochester, USA

Semaphorins are membrane bound or secreted proteins that bind to Plexin receptors, and are implicated in tumor progression in breast, kidney, ovarian and prostate cancer. We recently showed that Plexin B1, the receptor for semaphorin 4D (Sema4D) is a tumor suppression protein for melanoma, in part through its' ability to suppress activation of the oncogenic c-Met receptor by its ligand, hepatocyte growth factor (HGF). In this report we examined the signaling pathways of Plexin B1 in primary human melanocytes, the expression of Sema4D in the skin, regulation of Plexin B1 by UVR, and effects of Sema4D on c-Met activation. Treatment of melanocytes with recombinant Sema4D induced rapid phosphorylation of Akt and Erk, which was abrogated in Plexin B1-silenced cells. Functional analysis of proliferation (CLICK-IT assay) and apoptosis (TUNEL) in Plexin B1 knockdowns showed inmarkedly increased apoptosis and decreased proliferation, supporting a role for Plexin B1 in melanocyte survival and proliferation. Treatment of melanocytes with Sema4D, which we show is expressed by epidermal keratinocytes in vivio, abrogated UV-dependent apoptosis of melanocytes by 50%, shown by Western blotting for caspase-3. c-Met stimulates melanocyte migration, in part, through down-regulation of E-cadherin expression. Treatment of melanocytes with HGF, in the presence of Sema4D, partially blocked c-Met activation and abrogated the effects of c-Met on E-cadherin expression. Further, Sema4D blocked the stimulatory effects of c-Met on melanocyte migration. Finally, we show that Plexin B1 expression is markedly suppressed by UVB at the mRNA and protein level. These results show that Plexin B1 and its ligand Sema4D are important for melanocyte survival and proliferation, and suggest that Sema4D plays a role in moderating the effects of HGF on c-Met activity in melanocytes. Down-regulation of Plexin B1 by UVB may release inhibitory pressure on the c-Met receptor, promoting melanoma itiation.

P41

Subcellular localization of the P protein in human melanocytes

T. Kondo, V. J. Hearing
NIH, Bethesda, USA

The P gene is the pathogenic gene of Oculocutaneous albinism type 2 (OCA2) and encodes a pigment cell-specific, 12-transmembrane domain protein with homology to ion transporters. The P protein has been suggested to be involved in regulating melanosomal pH and/or substrate transport, but its function(s) remains unclear at this time, and its subcellular localization is still under debate. The goal of this project was to resolve both of those issues and determine the mechanism(s) underlying OCA2. We generated rabbit antibodies against the human P protein. We confirmed their specificities and sensitivities with western blots. To elucidate the subcellular localization of the P protein, we used confocal microscopy with antibodies specific for various subcellular organelles. Analysis of the specificities and sensitivities of those antibodies showed that only 1 of the rabbits had a high titer and was completely specific. Using this P antibody and other antibodies specific for various subcellular organelles allowed the subcellular localization of the P protein to be identified by confocal microscopy. The P protein was not localized in the endoplasmic reticulum although some of it was localised in lysosomes and in lysosome-related organelles.
Further identification of the processing and subcellular localization of the P protein using this new antibody should provide valuable information regarding its function and mechanism of action. This study should eventually clarify the role of the P protein in OCA2.

P42

The intracellular trafficking of tyrosinase and tyrosinase-related protein-1 to melanosomes is disrupted independent of the trafficking of dopachrome tautomerase and Pmel17 in reduced glutathione-induced amelanotic B-16 melanoma cells: a model for oculocutaneous albinism type 2

H. Nakajima, S. Koga, T. Nagata, G. Imokawa
Tokyo University of Technology, Hachioji, Japan

We have previously reported that reduced glutathione (GSH) abolishes the intracellular trafficking of tyrosinase (TYR) to

 © 2011 The Authors. Journal Compilation © 2011 John Wiley & Sons A/S

melanosomes, which results in completely unpigmented B-16 melanoma cells during the glucosamine (GlcN)-depleted recovery of melanization in GlcN-treated B-16 amelanotic melanoma cells. To characterize the trafficking of other melanocyte-specific (melanosomal) proteins, such as TYR-related proteins (Tyrp1), dopachrome tautomerase (Dct), Pmel17, MART-1 and Rabs27A/B in amelanotic GSH-induced B-16 (GB-16) cells, we used sucrose density-gradient ultracentrifugation, western blotting and confocal laser microscopy (CFLM) to examine differences in intracellular distribution of those melanosomal proteins compared with melanotic control non-GSH-treated B-16 (NonGB-16) cells. In sucrose density-gradient purified melanosome- or premelanosome fractions, the amelanotic GB-16 cells had a distinctly diminished protein level of TYR and Tyrp1 compared with the melanotic NonGB-16 cells, whereas there was substantially no difference in the distribution of Dct, Pmel17 or Rab27A/B proteins, which suggested that melanosomes or premelanosomes in the amelanotic GB-16 cells contain those melanosomal proteins except for TYR and Tyrp1. Analysis of merged images obtained by CFLM revealed that whereas all melanosomal proteins studied (TYR, Tyrp1, Dct, Pmel17 (HMB45), MART-1 and Rab27A/B) co-localized with each other in the vicinity of the nuclei of the melanotic NonGB-16 cells, those proteins, except for TYR and Tyrp1, also co-localized with each other in the amelanotic GB-16 cells. These results suggest that the intracellular trafficking of TYR and Tyrp1 to melanosomes is selectively disrupted independent of the trafficking of Dct, MART-1 and Pmel17 in the amelanotic GB-16 cells, providing a model similar to oculocutaneous albinism type 2.

P43

Oculocutaneous albinism 1 minimal pigment type; a case report on the analysis of genotype-phenotype correlation

M. Kono, T. Kondo, S. Ito, T. Suzuki, K. Wakamatsu, S. Ito, Y. Tomita
Department of Dermatology, Nagoya University, Nagoya, Japan

Tyrosinase gene-related oculocutaneous albinism (OCA1) is clinically divided into three types of A, B and temperature-sensitive (TS) according to the amount of melanin produced by the mutated tyrosinase, and the correlation between their genotypes and phenotypes have been clarified. King et al. proposed the fourth type, OCA1 minimal pigment (MP) characterized by having only minimal pigment, but the molecular basis of the disease has been unclear. To report a case of OCA1MP and to clarify the genotype and the function of the tyrosinase gene

A 37-yr-old woman with white skin, ivory white hair and faint brown irises had several brown freckles like solar lentigo that developed on the nape of the neck and the backs of the upper arms after grown-up. Tyrosinase gene of the patient was sequenced and its functional analysis was carried out by evaluating the activity of melanin production in the melan-c melanocytes transfected with the mutated tyrosinase cDNA. The pheomelanin and eumelanin contents in the patient's hair and in the transfected cells were evaluated by direct chemical analyses. The patient had two missense mutations of p.R77Q and p.D383N of the tyrosinase gene. Functional analyses of the two mutant TYR proteins produced in the melanocyte lines revealed no significant melanin production. The pheomelanin and eumelanin contents in mg hair were 155 and 86 ng in the OCA1MP patient, around 148 and 14 640 ng in normally-pigmented volunteers and 70 and 78 ng in a OCA1A patient, respectively. Our study demonstrated for the first time the genotype-phenotype correlation of OCA1MP. The eumelanin content of OCA1MP patient's hair was as low as that in the OCA1A patient. However, the hair pheomelanin content of the patient was almost the same as that in normal volunteers, the amount of which was higher than that of OCA1A patient. The activity of mutated tyrosinase must be too low to be detected, and only pheomelanogenesis could occur by extremely low enzyme activity.

P44

Molecular analysis of the OA1 gene in patients with ocular albinism

A. Rouault, E. Lasseaux, F. Morice-Picard, C. Rooryck-Thambo, D. Cailley, C. Castaing, D. Lacombe, A. Taïeb, B. Arveiler
Laboratoire de Génétique Moléculaire, CHU Pellegrin, Bordeaux, France

Mutations in the OA1 gene cause the most common form of ocular albinism (OA), an X-linked disorder mainly characterized by congenital nystagmus, a severe reduction of visual acuity, hypopigmentation of the retina and foveal hypoplasia. Male OA patients showing ocular albinism symptoms and X-linked inheritance were screened for mutations in the OA1 gene. An ophthalmologic exam and a genetic testing were also performed in mothers when possible. All nine exons and exon-intron boundaries of the OA1 gene, as well as the 5′ and 3′ untranslated regions, were screened for point mutations by direct sequencing of PCR amplified exons, and for genomic deletions by a high-resolution OA1-specific array-CGH. Thirteen male index patients from 11 families were screened for OA1 mutations. We found five different missense mutations, three different nonsense mutations, two duplications and one deletion. Seven of these mutations were previously not described and one was de novo. No large gene deletion was identified. We identified heterozygous mutations in six of seven mothers tested. The association of OA1 direct sequencing and array-CGH in the diagnostic strategy of OA enables us to screen at once point mutations and large gene deletions. We identified seven novel mutations. This study validates our strategy in which male patients with typical OA and with an X-linked familial history are tested for OA1 in a first instance. It should be noted that a mutation was found in two patients who did not present with an ocular albinism and family history typical of OA1, and who were initially thought to have OCA. The identification of a mutation in the OA1 gene provides useful information for the genetic counselling and allows prenatal diagnosis for families.

P45

Bioinformatics tools to predict splicing mutation effect in genetic diagnosis of oculocutaneous albinism

E. Lasseaux, F. Morice-Picard, C. Rooryck-Thambo, A. Rouault, C. Plaisant, P. Fergelot, D. Lacombe, B. Arveiler
Laboratoire de Génétique Moléculaire, CHU Pellegrin, Bordeaux, France

Oculocutaneous albinism (OCA) is an autosomal recessive disease of skin, hair and eye hypopigmentation caused by a deficiency in melanin biosynthesis. Four genes respectively responsible for the four types (1–4) of OCA have been identified: TYR, OCA2, TYRP1 and SLC45A2. Molecular analysis is essential for precise diagnostic of the different forms of OCA. We found mutations affecting splicing in the different genes leading to OCA. We sought to highlight the major role of bioinformatics resources in helping to evaluate the consequence of variants on splicing. We analyzed the whole coding region of the four OCA genes, including intron-exon boundaries and searched for deletions/duplications. Intronic variants were analysed using the

© 2011 The Authors. Journal Compilation © 2011 John Wiley & Sons A/S

Alamut 2.0 software (Interactive Biosoftware, Rouen, France) which gives access to several algorithms like Human splicing Finder and Max Ent Scan. We identified three new intronic variants in TYR, eight in OCA2, one in SLC45A2 and one in TYRP1 potentially affecting splicing. The splicing variant c.1037-7T>A in the TYR gene was already known as a frequent mutation.

We found several variants which occurred in the splicing consensus region of the acceptor site (at position -1). As expected all of these variants were predicted to disrupt splice site signalling. Then we analysed the deleterious effect of the variants located at position between +3 and +7 from the donor splice site, and –3 to –10 and beyond from the acceptor splice site for which these algorithms are most useful to predict pathogenic effect. For some of the tested variants, the algorithms provided splice scores indicating that splicing was altered. Bioinformatics resources play a major role in helping diagnostic laboratories to evaluate the consequence of mutations on splicing (exon skipping, partial exonic deletion, intronic retention) especially because most genetic tests use DNA samples and not RNA samples. RNA studies in albino patients are rendered difficult by the fact that expression of the OCA genes is restricted to specific cells such as melanocytes. Results of analyses specifically focused on splice sites should however be taken with caution, since features other than splice sites are also involved in the splicing process.

P46

Screening of SOX10 and MITF regulatory regions in Waardenburg syndrome

V. Baral, B. Duriez, Y. Watanabe, M. Goossens, T. Attie-Bitach, V. Pingault, N. Bondurand
INSERM U955, Creteil, France

Waardenburg syndrome (WS) is a rare auditory-pigmentary disorder that exhibits varying combinations of sensorineural hearing loss and pigmentation defects. Four subtypes are clinically defined based on the presence or absence of additional symptoms. WS2, which is defined by the absence of additional features, results from mutations within the SOX10 and MITF genes. However, 70% of WS2 remain unexplained, suggesting that other genes could be involved and/or that mutations within the known genes escape screening performed so far. The crucial role of SOX10 and MITF during melanocyte development and the identification of several of their regulatory regions prompted us to search for mutations within some of these regions in 30 WS2 unexplained cases. In case of MITF, we tested the involvement of the well described melanocyte-specific (M) promoter region and a distal regulatory region (MDE) located 14.5 kb upstream from exon 1M. In case of SOX10, sequence conservation analysis led to the identification of at least five segments directing expression in overlapping populations of neural crest derivatives. Previous functional studies revealed the role of at least two of them (U1 and U3) during melanocyte development. No deletion was identified upon QMF-PCR analysis of the MITF promoter and the five SOX10 regulatory regions. So far, sequencing of U1, U3 and the two MITF regions led to the identification of two variations not previously reported in dbSNP: one in close proximity to the MDE sequence, and one within U1. Their functional consequences should be tested in the near future.

P47

A novel non-truncating mutation of the MITF basic domain in an atypical form of type II Waardenburg syndrome

S. Léger, X. Balguerie, A. Goldenberg, V. Drouin-Garraud, A. Cabot, I. Amstutz-Montadert, P. Young, P. Joly, M. Goossens, V. Pingault
Clinique Dermatologique, Rouen, France

The microphthalmia-associated transcription factor (MITF) is a basic helix-loop-helix (bHLH) leucine zipper transcription factor which regulates melanocyte development and biosynthetic melanin pathway. The basic domain of bHLH factors is their DNA binding domain, necessary to recognize and bind their transcriptional targets. A noteworthy relationship has been described between non-truncating mutations of the MITF basic domain and Tietz syndrome, which is characterized by an severe hearing loss and an albinoid-like hypopigmentation of the skin and hair, rather than the patchy depigmentation seen in Waardenburg syndrome (WS). Six family members were affected on three generations with relative similar phenotype. They lived in France and had both Vietnamese and Martinique origins. The proband was a 9-yr-old boy who consulted for marked premature greying affecting eyelashes and eyebrows. He also had blue irides and generalized hypopigmentation of the skin, in contrast with familial dark phototype, associated with patchy depigmentation macules, freckles in sun-exposed regions, lentigines, cafe-au-lait macules. His auditory function was normal. A nucleotide substitution in the MITF gene, c.635T>G, that predicts a missense variation at the protein level (p.Ile212Ser) was found in all the affected members of this family. According to 3D models of other bHLH factors, the mutated amino-acid is expected to be on the side of the basic domain α-helix that is localized in contact with the DNA groove. This family strongly differs from the other cases of mutations located in the basic domain by the lack of congenital hearing loss over several generations. This precludes its classification as Tietz syndrome despite the uniform dilution of skin pigmentation. We also observed a striking high number of freckles as well as lentigines and café-au-lait macules. These pigmentary features are not considered as part of WS and therefore might be underestimated.

P48

Clinical and epidemiological study of vitiligo

D. N. W. Liyanage
Sri Lanka College of Dermatologist, Bandarawela, Sri Lanka

This study was conducted to analyze clinical and epidemiological profile of vitiligo in Sri Lanka. A descriptive study carried out over 2 yr. Two hundred and fifty-five patients were intervieved and examined to find out epidemiological profile, clinical features, beliefs and impact of the disease. There were 140 (54.9%) females and 115 (45.1%) males. Mean age at onset was 18.8 yr and 62.3% got vitiligo before 30 yr. 54 (21.17%) had segmental vitiligo and 201 (71.83%) had non segmental vitiligo. Vitiligo vulgaris was the commonest type, 85 (42.2%) among the non segmental vitiligo.

Koebner phenomena was found in 120 (47.3%). 117 (48.8%) believed rat bite and 54 (21.9%) thought vitamine deficiency was the cause for vitiligo. 210 (82.3%) had their personal life affected by the disease and 160 (62.7%) had their social lfe affected. Marital life and occupational lives were affected in 22 (7.8%). Associated autuimmune or endocrine disorders were found in 18 (7%). Vitiligo is the commonest pigmentary disorder in Sri Lanka with a grate personal and social impact. The clinical and

 © 2011 The Authors. Journal Compilation © 2011 John Wiley & Sons A/S

epidemiological profile is similer to studies done in other countries.

P49

Clinical course of segmental vitiligo: a retrospective study of 88 patients

J.-H. Park, J.-H. Lee, D.-Y. Lee
Department of Dermatology Samsung Medical Center, Seoul, Korea

Segmental vitiligo (SV) has distinct clinical feature and natural history. SV is almost always limited to one segment of the body and does not cross the midline of the body. It has been reported that SV usually shows an early onset of age and a stable course after its initial rapid spreading phase. Thus, unlike nonsegmental vitiligo the course of SV may be predictable. However, there has been very little study about the clinical course of SV. Thus, in this study we evaluated about disease progression in SV. The clinical course of 88 patients with SV was retrospectively examined through the chart review and comparison of serial photographs. More than 50% of SV patients showed a stable course within a few years after disease onset. However, about 20% of SV patients showed disease progression 4 yr after disease onset. Our result suggests that contrary to the previous report some SV patients may not show a stable course over a long time. This information about the clinical course of SV seems to have an implication for the treatment and prognosis.

P50

Epidemiology of vitiligo in university of Kinshasa Hospital (C.U.K)/D.R. Congo

C. Muteba Baseke
University Hospital Kinshasa, R.D. Congo

Vitiligo is the most common hypomelanosis, it affects 0.5% of world population, with no predominance of sex or ethnicity. It can occur at any age, 50% of people with vitiligo develop it before age 25. To survey the data on patients referred to University of Kinshasa Hospital for vitiligo and to determine prevalence, age, sex, location of choice and possible association with other diseases in our midst. Retrospective study conducted at the dermatology department of the university of kinshasa hospital from January 2000 to January 2010. Data were entered and analyzed using Epi Info version 3.5.1, we used the following statistical tests: the prevalence, percentage and average. Spread over 10 yr, 204 patients suffering from vitiligo were selected, however, 14 195 patients with all kinds of skin diseases have been received during the same period, representing a frequency of 1.4%. The female is most affected with 61%, Sex-ratio F/M is 1.5. Age groups before 35 yr are most affected for both sex with 67%. The most generalized form is found to be 47%. One patient developed vitiligo during melanoma evolution, two patients were diabetic and seven patients had hyperthyroidism. The present data indicate in our community that Vitiligo affects preferably female with 61%, it starts well before the age of 35 yr for both sex with 67% and the generalized form is most prevalent with 47% of cases.

P51

Pseudoleukoderma angiospasticum: two cases

S. Shan-Yi Ng, L. Hwee-Ying Teo
Ministry of Health Holdings, Singapore

Pseudoleukoderma angiospasticum is a rare condition that has not been widely reported in the literature. Central arteriolar spasm with peripheral vasodilatation leads to a presentation of white-checkered patches over the palms, soles, buttocks and flexural areas of arms and legs. It is more noticeable in fairer skin types. We report two Chinese males presenting with this condition, one of whom was treated initially as a fungal infection.

P52

A study of burden of vitiligo in Indian patients using a new and specific rating scale

S. G. Krishna, M. Ramam, M. Mehta, V. Sreenivas, V. K. Sharma, S. Khandpur
Academics and Research, New Delhi, India

Vitiligo is an enigmatic disease. Though asymptomatic, it carries a huge burden on patients' lives. It is considered a social stigma particularly in colored skin populations. Till date, the burden of vitiligo has not been measured using a specific vitiligo questionnaire or instrument. To measure the burden of vitiligo using a questionnaire specific for vitiligo – the Vitiligo Impact Scale (VIS). The VIS was administered to vitiligo patients above 18 yr of age. Along with VIS the patients also answered the DLQI and Skindex-16. Detailed clinical examination was done. The instrument was administered to 100 consecutive patients of vitiligo. There were 57 males and 43 females. Forty-eight patients were married and 52 were unmarried forty-six patients were working and 54 patients were either unemployed, housewives, students or retired. Acrofacial vitiligo was the most common type of vitiligo. Scores ranged from 5 to 64 (mean (SD) 26.57 (12.13), median: 26). Nearly half of the patients had moderate to severe burden. Females were found to have a higher burden. But there was no significant difference in burden of patients in repect to the surface area involved, marital status, employment status, age or duration of vitiligo. Many patients had suicidal tendencies. A significant number had marital problems. The study proves that vitiligo carries a huge burden on patients' lives. The results obtained confirmed the observations of some previous studies. Use of a vitiligo specific rating scale gave more authentication to the data.

P53

Vitiligo with raised and inflammatory borders – a rare case report from North India

S. Kumar, T. Kaur, B. B. Mahajan, R. Singh
Guru Gobind Singh Medical College and Hospital, Faridkot, Punjab, India

Vitiligo is the most common hypomelanosis. Inflammatory vitiligo, a rare variant of vitiligo, has been classically described as an erythematous rim at the periphery of a patch of hypopigmented skin characterized histologically by superficial perivascular infiltrate of mononuclear cells. We here report a case of a 50 yr old female from Faridkot (India), who presented with hypopigmented macules on left shoulder region and both shins, surrounded by red raised margins. Histopathological examination confirmed the diagnosis of inflammatory vitiligo. Immunological disturbance involving keratinocytes, melanocytes and Langerhans cells by T cells, is implicated. The case is being reported for its rarity as slightly more than 20 cases have been reported in literature till now and to the best of our knowledge this might be from a few case reports from India.

P54

Autoimmune signals in vitiligo patients appear correlated with obsession and phobia

M. Arunachalam, R. Colucci, R. Conti, S. Berti, F. Lotti, S. Pallanti, T. Lotti, S. Moretti
University of Florence, Italy

Current studies have treated a limited portion of the subjective aspects of vitiligo patients and have yet to elucidate possible

© 2011 The Authors. Journal Compilation © 2011 John Wiley & Sons A/S

psychological differences between those with autoimmune signs respect to those without autoimmune indicators. Thus, we performed an 1:1 age and sex matched case–control study, comparing non segmental vitiligo patients having autoimmune features versus those not showing autoimmune features in regards to psychiatric features, psychosomatic aspects, and social parameters. One hundred and sixteen non segmental vitiligo patients have been examined at the Florence University dermatology outpatient service (2nd dermatology unit). Vitiligo with an autoimmune background was identified according to the presence of autoimmune antibodies and/or autoimmune diseases. Psychiatric screening was performed by dermatologists using the modified Middlesex Healthcare Questionnaire (MHQ); psychosomatic aspects and social impact were analyzed with a standardized, Florentine questionnaire. Upon performing a multivariate analysis with a conditional regression model, age, phobia, and obsession were significantly predictive of the presence of AIS and total MHQ score was significantly predictive of NAIS. With univariate analysis using McNemar's test, we found significant differences in: identifiable stress related to the onset of vitiligo, vitiligo triggered by stress, and modified interpersonal relationships related to vitiligo, which were associated with the subgroup containing autoimmunity signals. We found a higher prevalence of age, obsession, and phobia among vitiligo patients with autoimmune signals as compared to vitiligo patients without autoimmune signals. Therefore, in the presence of demonstrated autoimmunity, screening for particular psychiatric aspects may be useful in clinical practice of vitiligo.

P55

Generalized vitiligo and related autoimmune disorders in Japanese patients and their families

N. Oiso, K. Fukai, T. Narita, K. Kabashima, A. Kawada, T. Suzuki
Department of Dermatology, Kinki University Faculty of Medicine, Osaka-Sayama, Japan

Generalized vitiligo is an acquired disorder in which depigmented macules result from the autoimmune loss of melanocytes from the involved skin. It is frequently associated with other autoimmune diseases, particularly autoimmune thyroid diseases (Hashimoto's thyroiditis and Graves' disease), rheumatoid arthritis, type 1 diabetes, psoriasis, pernicious anemia, systemic lupus erythematosus, and Addison's disease. One hundred and thirty-three Japanese individuals with generalized vitiligo were enrolled for investigating the occurrence of autoimmune diseases in patients with generalized vitiligo and their families. Twenty-seven of the patients with generalized vitiligo (20.3%) had autoimmune diseases, particularly autoimmune thyroid disease (16 patients, 12%) and alopecia areata (seven patients, 5.3%). Thirty-five patients (26.3%) had a family history of generalized vitiligo and/or other autoimmune diseases. Familial generalized vitiligo was present in 15 (11.3%), including four families with members with autoimmune disorders. Twenty (15.0%) had one or more family members with only autoimmune disorders. Among Japanese vitiligo patients, there is a subgroup with strong evidence of genetically determined susceptibility to not only vitiligo, but also to autoimmune thyroid disease and other autoimmune disorders.

P56

Prevalence of co-existent organ-specific (TPO) and non-organ specific (ANA) autoantibodies in patients with segmental vitiligo versus non-segmental vitiligo: a case–control study

B. K. Khaitan, D. Seshadri, S. Kathuria, N. Gupta, M. Ramam, V. K. Sharma
Department of Dermatology and Venereology, All India institute of Medical sciences, New Delhi, India

To study the levels of organ-specific (thyroid peroxidase, TPO) and non-organ specific (anti-nuclear antibodies, ANA) autoantibodies in patients of segmental vitiligo versus non-segmental vitiligo. After ethical clearance, a cross-sectional study was carried out where consenting consecutive patients of segmental (cases) and nonsegmental (control) vitiligo presenting to Dermatology Outpatient Department, AIIMS, New Delhi were recruited. All patients were subjected to detailed history and complete skin examination with charting of lesions. A venous blood sample was drawn from each case/control after informed consent and tested for anti thyroid peroxidase antibody (TPO) and antinuclear antibody (ANA) levels using chemiluminescence and immunoflourescent microscopy respectively. A total of 101 patients were recruited including 45 with segmental vitiligo, six with mixed vitiligo (segmental with non-segmental vitiligo) and 50 with non-segmental vitiligo. ANA testing was done in all 101 patients while 76 underwent TPO testing (including 39 from non-segmental group, 35 from segmental group and two from the mixed group). Overall, ANA was positive in 10/101 (9.9%) and TPO in 9/76 (11.8%). In non-segmental vitiligo patients, ANA was positive in 8/50 (16%) and TPO in 7/39 (17.9%), while in patients with segmental and mixed type vitiligo, ANA was positive in 2/51 (3.9%) and TPO was positive in 2/37 (5.4%). The difference in ANA positivity between non-segmental and segmental tended towards statistical significance (two-tailed Fischer's Exact test $P = 0.051$). However, the apparent difference in TPO positivity was not statistically significant. Antinuclear antibodies are significantly more common in patients with non-segmental vitiligo as compared to those with segmental vitiligo. TPO antibodies are more frequent in nonsegmental vitiligo, however, the difference is not statistically significant.

P57

Assessment of tissue FoxP3+, CD4+ and CD8+ T-cells in active and stable non-segmental vitiligo

M. Abdallah, R. Lotfi, W. Osman, R. Galal
Ain Shams University Hospital, Cairo, Egypt, Cairo, Egypt

FoxP3+ T regulatory cells (T-regs) play a role in inhibition of autoimmune responses, but their definite role in active versus stable non-segmental vitiligo (NSV) was not assessed. To assess tissue CD4+, CD8+ T cells and FoxP3+ T-regs in active and stable NSV. Immunohistochemichal double staining for CD4 and CD8 in addition to FoxP3 were done in lesional (L), perilesional (PL), and non-lesional (NL) skin. Significant increase in CD4+ and CD8+ T cells in PL skin in comparison to L and NL skin in both stable and active NSV. Both cell types were significantly increased in active cases in PL, L and NL skin. Highly significant reduction in the number of Foxp3+ T-regs was observed in PL in comparison with L and NL in both active and stable cases. Active NSV showed more reduction in T-regs in PL skin. At and near the dermo-epidermal junction (DEJ) of PL and L skin, CD8+ T cells were markedly increased in comparison to CD4+ cells. T-regs were more abundant at the DEJ of PL skin. Elevation in the number of CD4+ and CD8+ T cells in PL and L in active vitiligo and increase the number of CD8+ T cells at the DEJ implies their

 © 2011 The Authors. Journal Compilation © 2011 John Wiley & Sons A/S

involvement in melanocyte destruction. The reduction in the number of T-regs in PL in active cases suggests that this is the site where regulatory activity is lacking to suppress the activity of helper and cytotoxic T cells that are actively contributing to depigmentation.

P58

Evidence for a local and systemic immune reaction against melanocyte differentiation antigens in a patient with regressing nevi without halo

R. Speeckaert, N. Van Geel, R. Luiten, M. Van Gele, M. Speeckaert, J. Lambert, K. Vermaelen, E. Tjin, L. Brochez
Gent University, Gent, Belgium

Regressing nevi are considered an example of an efficient early antitumoral immune response preventing the development of neoplasia. The underlying mechanism has not been elucidated although an immune-based destruction of melanocytes is supposed. The aim of this study was to provide evidence of an effective immunosurveillance of pigment lesions in a patient at high risk of melanoma. A patient with the dysplastic nevus syndrome (>100 nevi) and a history of melanoma (Breslow < 1 mm) in 1997 was included in this study. Since 2003, a marked regression of almost all nevi was observed without halo formation. Standard immunohistochemistry (CD3, CD4, CD8, CD1a, Foxp3) was performed on the regressing nevi. Flow cytometry with HLA-A2-peptide tetramers specific for Mart-1 (26–35), gp100 (280–288), gp100 (209–217) and tyrosinase (369–377) was performed on peripheral blood mononuclear lymphocytes and lymphocytes isolated from a regressing nevus. A peptide-specific stimulation experiment with melanocyte antigen-specific peptides was carried out. Immunohistochemistry of the regressing nevi showed a strong infiltrate of CD3+, CD4+, CD8+ and CD1a+ cells with a low frequency of Foxp3+ cells. Flow cytometric analyses demonstrated the presence of a CD8 lymphocyte reaction against gp100 (280–288) and Mart-1 (26–35) both in peripheral blood and lesional lymphocytes. A moderate increase in proinflammatory cytokine production (TNF-γ, IFN-α, IL-4) after melanocyte specific peptide stimulation was observed. These findings indicate that an effective systemic immune reaction against melanocyte differentiation antigens can target specifically nevi without signs of vitiligo and suggests that boosting the anti-melanocyte immune response in patients at high risk for melanoma may prevent tumoral development at an early stage.

P59

New in vivo vitiligo induction and therapy model: proof of concept

N. Van Geel, R. Speeckaert, I. Mollet, S. De Schepper, E. Tjin, R. Luiten, L. Brochez, J. Lambert
Ghent University Hospital, Gent, Belgium

Tissue damage can cause new lesions in vitiligo (= Koebner's phenomenon). The aim of our study was to develop an in vivo vitiligo induction model to explore the underlying mechanisms leading to Koebner's phenomenon and to evaluate the effect of therapeutic strategies on this process. Twelve pigmented test regions (2.5 × 2.5 cm) on the back of three generalized vitiligo patients were exposed to three different Koebner induction methods: cryotherapy, 755 nm laser therapy and CO2-laser abrasion. In addition, four cream treatments (pimecrolimus, tacrolimus, local steroid and placebo) were randomly applied (day 0, 3 and 6). Surface measurement of skin depigmentation per test region was performed at different time points using ImageJ software. Skin biopsies were taken for immunohistochemical stainings (CD3, CD4, CD8, CD1a, Foxp3). In one patient lesional lymphocytes were isolated and stained with HLA-A2 tetramers for MART-1, gp100 and tyrosinase. Koebnerisation was efficiently induced in all three patients, resulting in patterns of depigmentation which differed between the patients according to the induction method. Strong significantly higher percentages of depigmentation were observed at placebo treated sites compared to therapeutic test regions ($P < 0.001$). The therapeutic methods showed in all patients reproducible results and pointed to tacrolimus and local steroids as significantly better inhibitors of Koebner's process ($P < 0.05$) compared to pimecrolimus. The most pronounced lymphocytic infiltrate and the highest percentage of gp100 and MART-1 CD8 cells were observed at placebo treated sites. This is a very informative model to investigate vitiligo induction. This proof of concept confirms the efficient comparison of head to head therapeutic strategies intra-individually in a standardized, specific and better timed way.

P60

Genetic mapping of loci underlying vitiligo in the Smyth Line chicken model

S. Kerje, W. Ek, A.-S. Sahlqvist, O. Ekwall, G. Erf, Ö. Carlborg, L. Andersson, O. Kämpe
Uppsala University, Uppsala, Sweden

Vitiligo is an autoimmune disease characterized by loss of hair and skin pigmentation due to a destruction of the pigment producing melanocytes. Here, we study the Smyth Line chicken, which displays several features similar to the disease characteristics in humans. An F2 intercross was generated from Smyth Line chickens and a line of chickens with a low incidence of vitiligo. All F2 individuals were scored as either affected or unaffected with vitiligo. A genome-wide scan for quantitative trait loci (QTL) was performed to identify loci underlying vitiligo. Two different single QTL models and one model for pairwise epistatic QTL were used. Twenty percent of the F1 chickens and 26–32% of the birds in the F2 population (variation between batches) were affected by vitiligo, suggesting a polygenic inheritance of disease. Sex ratio was slightly, but not significantly higher in males then in females. One novel QTL was identified on chromosome 12 (OR = 2.72). The estimated effect of this locus on vitiligo is nearly twice as large as those previously reported for vitiligo in human GWAS studies, indicating that it could potentially be a key locus in determination of the disease.

P61

Vitiligo: there is more than meets the eye

R. Conti, R. Colucci, M. Arunachalam, S. Berti, S. Moretti
Santa Chiara Hospital, Florence, Italy

Vitiligo macules, at first glance, might be considered only an aesthetic problem. However, they may represent a sign of very complicated internal diseases, such as Vogt Koyanagi Harada syndrome (VKH). We further report the first association of VKH with psoriasis, common variable immunodeficiency (CVID), celiac disease, and thyroiditis, all in the same patient. Herein we describe the case of a 39 yr old female that referred to our dermatology outpatient clinic with a 20 yr history of non segmental vitiligo associated with VKH syndrome, psoriasis, CVID, celiac disease, and thyroiditis. Thyroid echography revealed chronic thyroiditis, intestinal histology was diagnostic of celiac disease, and opthalmological examination showed late features of a VKH uveitis. Blood analysis showed normal platelets, ferritin and transferrin values, but low hemoglobin and iron levels. IgA and IgM were notably inferior to normal values but IgG levels were within normal range. IgA antiendomysial, anti-transglutaminase, anti-thyroglobulin, anti-thyroid peroxidase, anti-platelets and anti-red blood cells antibodies

© 2011 The Authors. Journal Compilation © 2011 John Wiley & Sons A/S

were normal. No alteration of thyroid hormones and TSH levels were observed. We report the first association of VKH with the above-mentioned diseases all in the same patient, demonstrating that meticulous observation and clinical examination in vitiligo patients is necessary.

P62

Coexistence of vitiligo and psoriasis – report of three cases

M. Ivaniciuc
Dermatology, Suceava County Polyclinic, Suceava, Romania

Vitiligo is a chronic aquired hypomelanotic autoimune disorder affecting from 0.5 to 2% of the general population. Psoriasis is one of the most common T-cell mediated autoimune disease in humans, with prevalence ranging between 1 and 3% in the population worldwide. There have been several reports of the concurrence of these two diseases in the literature. I report three cases of the coexistence of vitiligo and psoriasis (two male and a female). After careful anamnesis, a clinical exam was performed for each patient and screening for autoimmune diseases was recommended. The onset of psoriasis occured before that of vitiligo in two patients (male and female) and after vitiligo in one case (male). Two patients had a positive family history: (i) one male's parents with vitiligo (mother) and psoriasis (father) and (ii) one female's relatives (sister, maternal aunt and niece) with psoriasis. On clinical examination the lesions of psoriasis and vitiligo were partly coincidental in the male patients, while the woman had separate distribution. The patients were screened for autoimmune diseases and found negative. The family history for autoimmunity was also negative. The concurrence of vitiligo and psoriasis in the same patients could indicate a common pathogenesis. Both diseases have a genetical component suggested in our cases by the positive family history. However the relationship of vitiligo and psoriasis needs to be further investigated.

P63

What outcomes are important to patients and clinicians: survey results

V. Eleftheriadou, K. Thomas, M. Whitton
Centre of Evidence Based Dermatology, Nottingham, UK

Vitiligo is the most common chronic depigmentation disorder, which has a major impact on the quality of life of its patients. The authors of the recently updated Cochrane Systematic review of interventions for vitiligo concluded that there was insufficient robust evidence to support the efficacy of the treatments assessed. One of the most important reasons was that it was not possible to pool data due to the wide variations in outcome measurements for this disorder reported in the trials. It is increasingly recognised that both patients and clinicians have a key role to play in driving the research. It has also been reported that patient-centred outcomes should be incorporated into the design of future studies for vitiligo. Therefore it is essential to incorporate patients' and clinicians' views in the choice of outcome measurements. The objective of this survey is to find out what outcome measurements are important to both patients and clinicians. A survey of patients (Vitiligo Society) and clinicians (UK Dermatology Clinical Trials Network and British Association of Dermatologists) was conducted, as part of the Vitiligo Priority Setting Partnership (PSP). Participants in the PSP were asked in an open-ended questionnaire to submit their opinions on what should be measured in vitiligo trials. Results: 86% (399/461) of PSP participants suggested at least one outcome measurement. In total 412 suggestions were made. Seven percent (30/412) of suggested outcomes were excluded as non-relevant. Two thirds of respondents were patients and 1/3 were clinicians. More women responded that men and most were 30–60 yr old. Sixty-nine percent (277/399) of participants stated that repigmentation is the most desirable outcome measure for the treatment of vitiligo but consensus over how this should be expressed was mixed. Patients tended to focus on 'cosmetically acceptable' and 'permanent repigmentation', rather than the simple proportion of repigmented skin. Fifteen percent (61/399) of participants suggested that the cessation of spreading of vitiligo should be measured. Stabilising the disease is seen as a realistic outcome measure at least until repigmentation occurs. Nine percent (37/399) of participants consider Quality of Life important. Reduction in stress, embarrassment and improvement in support and self confidence were mentioned. In conclusion, it is well known that valid and reliable outcome measurements are a prerequisite of evidence based medicine, and this study constitutes a first step.

P64

A simple index of potential repigmentation in vitiligo

L. Benzekri
Department of Dermatology, Ibn Sina University Hospital, Mohammed V Souissi University Rabat, Rabat, Morocco

Repigmentation of vitiligo depends on available melanocytes remaining in the melanocyte reservoirs. Vitiligo recovery depends on the existence of viable melanocyte reservoirs. The repigmentation is possible when pigment cells are stimulated. The potential for repigmentation is an important determinant for the management of vitiligo. To develop after a careful examination a simple index of potential repigmentation in vitiligo. In a given patient with vitiligo, all the lesions are carefully examined clinically and with the help of Wood's light. Firstly the total number of vitiligo lesions is assessed. Secondly each lesion is classified according the following classification: Type A: Hypochromic lesion, Type B: Achromic lesion with dark hairs, Type C: Achromic lesion with white hairs, Type D: Achromic lesion on glabrous skin. Usually type A and type B respond well to the medical treatment while type C and D are refractory. At the end of the examination the exact percentage of each type is easily evaluated in a given patient. The index of potential repigmentation is calculated as follows by dividing the addition of (percentage of type A lesion + percentage of Type B lesion) by the addition of (percentage of type C lesions + percentage of type D lesions). The index is ranging from 0 to 9. We estimate that an index superior to 5 is a good index with a reasonable hope of repigmentation. In the opposite an index inferior to 5 corresponds to a refractory status. The assessment of this simple index could be useful to make a reliable prognosis in vitiligo patient.

P65

New reflectance confocal microscopy features in vitiligo: beyond the papillary rings

L. S. Abraham, M. C. Costa, A. Pacifico, G. Leone, M. Picardo, M. Ardigò
Instituto de Dermatologista Professor Rubem David Azulay, Rio de Janeiro, Brazil

Reflectance confocal microscopy (RCM) is a spreading technology for noninvasive evaluation of the skin up to papillary dermis. This technique provides real-time en face images with a resolution close to histopathological examination. Since melanin represents one of the main targets of RCM, it seems to be an interesting device in pigmentary disorders assessment.

The preliminary studies about RCM in vitiligo presented absence of papillary bright rings at the dermo-epidermal junction in achromic lesions and their abnormal bright distribution in appar-

 © 2011 The Authors. Journal Compilation © 2011 John Wiley & Sons A/S

ently normal skin. Nonetheless, there are still other RCM findings in vitiligo that need to be described and better understood. By evaluating lesional, perilesional and non lesional skin of vitiligo patients, presenting varied stages of the disease, we observed, in different amounts and distribuition, inflammatory cells, melanophages and dermal deposit of pigment.

Regarding disease stage, we found more inflammatory cells and melanophages in achromic lesions and perilesional skin of active vitiligo when comparing to the skin of stable vitiligo, in which they were very few or absent. In repigmented spots, dendritic and activated melanocytes were noted thanks to their highly brightness. Due to its noninvasiveness, serial examinations could be performed in the same spot in order to evaluated therapy response at a cellular level. RCM examination in vitiligo should not be faced as a diagnostic method for vitiligo, analyzing only the presence or absence of the papillary rings. In fact, RCM has shown to be probably a useful tool to define the activity of the disease by the detection of inflammatory cells and melanophages. In this preliminary report, we describe unpublished RCM features with possible direct clinical implication on lesion staging, therapeutic response evaluation and management of vitiligo.

P66

The evaluation of our recent therapies of vitiligo vulgaris

T. Shibata, A. Sasase, C. Hihiro Honda, K. Hayashibe
Shibata Clinic of Dermatology, Osaka, Japan

We examined about 7000 patients of vitiligo vulgaris every year in our clinic. We will present results of recent therapies for about 450 patients of vitiligo vulgaris who visited our clinics in the last 6 yr including methods, risks and effects of narrow-band UVB (DERMARAY-400), excimer lamp (VTRAC) therapies. Furthermore, the effect of various ointments, vitamin D3, tretinointocoferil, tacrolimus, prostaglandin and ViTiX will be reported. The number of valid patients was 449. We found 91.5% patients (411/449) who were treated in our clinics could get repigmentation and that 13.6% patients (61/449) had >50% repigmentation. The effectiveness depends upon not age but site (ex. face) and date of onset. The four conditions of better prognosis for vitiligo vulgaris are Face, Child, Early phase and Smaller lesions (FACES).

P67

Tacrolimus versus pimecrolimus in localised stable vitiligo

M. Phiske, B. Patil, Z. Bharda, H. Jerajani
Navi-Mumbai, India

To compare efficacy and safety of 0.1% Tacrolimus ointment versus 1% Pimecrolimus cream in localised stable vitiligo. Fifty-two cases of localised stable vitiligo were randomly divided to receive 0.1% Tacrolimus ointment or 1% Pimecrolimus cream and followed up monthly for 6 months to assess repigmentation based on 7-point ordinal scale. Overall response was graded as- Poor (1–25%), Moderate (26–50%), Good (51–75%), Excellent (>76%). Mean age of cases was 19 yr, F/M ratio – 3:2. In 54% cases, vitiligo was <6 months duration. Maximum repigmentation in Tacrolimus group was seen in age group 11–20 yr (45%) while in Pimecrolimus group it was 21–30 yr (22%). Repigmentation in both groups was more in males (50 and 27%) than females (26 and 12%). Vitiligo of duration <1 yr responded maximally (50% in Tacrolimus and 21% in Pimecrolimus group). Maximum repigmentation was seen on trunk (62%) in Tacrolimus group, while on lower extremity (33%) in Pimecrolimus group. Moderate to excellent response was seen in 39% cases in Tacrolimus against 8% in Pimecrolimus group. Burning and pruritus were present in 6% cases in both groups. Tacrolimus is more efficacious than Pimecrolimus in localised stable vitiligo when treated and monitored for 6 months.

P68

Surgery and laser treatment of vitiligo

P. Araujo, M. Fabrini
Private clinic and City Hospital, Belo Horizonte, MG, Brazil

The treatment of vitiligo is a great challenge, due to the erratic evolution of this disease and to its enigmatic pathophysiology. The present study shows some types of treatments which can be used in order to obtain a pigmentation, although a definitive cure is still not possible. A group of 30 patients with stable vitiligo was treated using various techniques including phototherapy, excimer laser, dermoabrasion, mosaic surgery, skin grafts ormicropigmentation, isolated or in combination. The results were obtained after a 3–24 months follow-up. The results are variable, but they retrospectively allow to select the best indication for vitiligo treatment on an individual basis.

P69

Determinants of success of melanocyte transplantation in vitiligo: role of cytotoxic CD8 Tcells

A. Rao, S. Gupta, V. K. Sharma
All India Institute of Medical Sciences, New Delhi, India

The role of cytotoxic T cells in vitiligo has long been recognised but the relationship of cytotoxic T cells with success of melanocyte transplant is unknown. To assess the relationship of cytotoxic T cells with the success of melanocyte transplantation 15 patients with generalized vitiligo with body surface area involved <10% were included in the study. Patients were divided equally into three stability groups: Group 1 with clinical stability >3 months but <1 yr, Group 2 with clinical stability ≥1 yr but <2 yr, Group 3 with clinical stability ≥2 yr. A single vitiliginous patch was chosen for melanocyte transplantation using suction blister grafting. A 3 mm punch biopsy was taken from the margin of the patch. Similar punch biopsy taken from normal skin of four patients served as control. Immunohistochemistry was done using antibodies for CD8 on all the biopsied samples. Positive cells were counted and expresseed as percentage of total lymphocytes. Repigmentation was assessed after 6 months. Those with >75% repigmentation were labelled as responders and those with <75% repigmentation were labelled as non responders. None of the patients in group 1 responded to treatment while all five patients in group three responded to the treatment. In group 2, two patients (40%) responded to treatment. The mean CD8% were highest in group 1 (4.2 ± 1.643), comparatively low in group 2 (1.6 ± 1.34) and lowest in group 3 (0.4 ± 0.54). The results were statistically significant (P = 0.007). Also, the mean cytotoxic T cell percentage was significantly higher in the non responders (3 ± 2.07) than in responders (1 ± 1.41) (P = 0.035) while there was no difference with relationship to the controls. A higher percentage of cytotoxic CD8 cells is associated with failure of melanocyte transplantation.

P70

Melanocyte transplantation outcome (METRO) scoring to assess the outcome of non cultured epidermal suspension transplantation in vitiligo

A. P. Holla, D. Parsad, A. J. Kanwar, S. D. Mehta
Vitiligo Institute for Care and Research, New Delhi, India

There is urgent need for universally acceptable measurement tool to assess the efficacy of vitiligosurgery, which should

© 2011 The Authors. Journal Compilation © 2011 John Wiley & Sons A/S

include quality of life assessment because percentage of regimentation may not always be a good indicator of patient satisfaction. To design an uniformly acceptable, more objective, reproducible and easy to use measurement tool to assess the outcome of the non cultured epidermal suspension transplantation. Melanocyte Transplantation Outcome (METRO) Scoring has following parameters and scoring pattern. A. Pigmentation extent (at 6 month) – <25, 26–50, 51–75, 76–90 and >90%; scored 1–5. B. Colour match (6 month) – somewhat lighter/darker and normal; scored 1or 2. C. Predominant type of pigmentation (1 month) – marginal, perifollicular or diffuse and combination; scored 1–3. D. Improvement of leukotrichia (6 month) – not present, no improvement and improvement; scored 0, –1 and 1. E. Complications (During 6 month follow up) – no complication and complication at recipient site or donor site; scored 1 or –1. F. Dermatology Life Quality Index change (Before versus At 6 month) – no change, mild to moderate change (<75% decrease) and significant change (>75% decrease); scored 0–2. METRO SCORE = A + B + C + D + E + F. Score ranges 0–14. Poor outcome if score is 0–4, good outcome if 5–9 and excellent if 10–14. It could become a valid measurement tool for prospective or retrospective assessment of the outcome of vitiligosurgery in general and non cultured epidermal suspension transplantation in particular. This scoring system considers patient's perspective and it can be used to compare the outcome of two different procedures.

P71

Comparison between autologous non-cultured epidermal cell suspension and suction blister epidermal grafting in stable vitiligo: a randomized study

A. Budania, D. Parsad, A. J. Kanwar, S. Dogra
PIGMER, Chandigarh, India

Vitiligo is an acquired disorder of pigmentation due to loss of epidermal melanocytes. Various modalities of treatment (both surgical and medical) are available. Amongst the surgical modalities, autologous non-cultured epidermal cell suspension (ANCES; a cellular grafting technique) and suction blister epidermal grafting (SBEG; a tissue grafting technique) gives promising results. We compared the two techniques, ANCES and SBEG in producing repigmentation in stable vitiligo patients, which, to best of our knowledge, is the first study in the literature. We randomized 20 patients with 27 stable vitiligo lesions into two groups. Patients in group one were treated with ANCES, while in group two with SBEG. They were evaluated 16 weeks post surgery for the extent of repigmentation, colour match, change in DLQI and patient satisfaction. Repigmentation results were excellent (showing >90% repigmentation) in 71.4% of lesions in ANCES group while 15.4% of lesions in SBEG group ($P = 0.003$). 92.9% lesions in ANCES group and 46.2% lesions in SBEG group showed >75% repigmentation ($P = 0.008$). There was a significant decline in DLQI in both the groups, also the mean decline amongst groups differed significantly ($P = 0.019$); the mean decline in DLQI was 7.8 and 4.3 in ANCES and SBEG groups respectively. No significant difference was seen in colour match. The diffuse pigmentation was the most common pattern of repigmentation. Adverse effects were minimal. ANCES is significantly much better than SBEG and should be preferred in the treatment of stable vitiligo patients.

P72

To compare the outcome of minipunch grafting and suction blister epidermal grafting alongwith post-surgical application of clobetasol propionate 0.05% cream in patients of stable vitiligo

R. Batra
Department of Dermatology, Sir Ganga Ram Hospital, New Delhi, India

To compare the outcome of minipunch grafting and suction blister epidermal grafting alongwith postsurgical application of clobetasol propionate 0.05% cream in patients of stable vitiligo. One hundred and eighty patients with vitiligo ($n1 = 60$ for minipunch grafting and $n2 = 120$ for suction blister epidermal grafting), for more than 1 yr were recruited. Minipunch grafting was performed by using 2.5 mm minipunch for the donor site and 2 mm minipunch for creating the recipient well. Suction blister epidermal grafting was carried out by using 10 and 20 cm^3 syringes while a 50 cm^3 syringe was used to create the negative pressure for suction.
Various body sites including lips, genitals, arms, legs and neck were taken for the procedures.
After complete healing of the wound in both the cases patient was prescribed clobetasol propionate (0.05%) cream and regular follow ups were carried out. Mean age of our study population was 24.7 yr (range 10–46 yr). The stability of vitiligo lesions ranged from 1 to 21 yr. The mean age of stability of lesions was 4.46 yr in Suction blister epidermal grafting, 5.34 yr in minipunch grafting. Generalized vitiligo was present in 48 (26.6%) patients, segmental in 42 (23.3%) and focal in 90 (50%) patients. No significant difference between these types of vitiligo was noticed in context to repigmentation and complications. More than 75% of the patients performed with suction blister epidermal grafting showed more than 50% repigmentation at the end of 4 weeks while it took 8–10 weeks to show complete results. Maximum achievable results were seen by the end of 10 weeks. We found that both the procedures are simple, safe, effective and inexpensive; and can be performed as OPD procedures. However, cosmetically better end results was seen in a significantly higher number of patients in the Suction blister epidermal grafting group due to absence of cobblestone appearance and hypertrophic scarring.

P73

A randomised controlled trial assessing the effectiveness of minigrafting versus ReCell in stable vitiligo: preliminary results

B. S. Daniel, S. S. Venugopal, L. K. Martin, A. L. Agero, L. M. Rhodes, J. W. Frew, R. Wittal, J. Le Guay, D. F. Murrell
St George Hospital, Sydney, Australia

Non-cultured Epidermal Suspension (NCES) involves preparing a split thickness skin graft which is processed into an epidermal cell suspension with the ReCell device and transplanted onto dermabraded skin affected with vitiligo. The role of ReCell as a surgical treatment in vitiligo has been published as case reports and this study seeks to determine if repigmentation differs between NCES and minigrafting. We are currently conducting a prospective intra-patient single centre randomised comparison trial for patients with chronic stable vitiligo. Participants have both minigrafting and NCES performed on randomised anatomically paired sites. Pre and post-procedure pictures of donor and recipient sites are used to evaluate the percentage of repigmentation which is assessed by an independent blinded investigator. The primary outcome of the trial is the percentage repigmentation at 12 months of NCES as compared with the conventional

 © 2011 The Authors. Journal Compilation © 2011 John Wiley & Sons A/S

minigrafting. Secondary outcomes include percentage repigmentation at 3 and 6 months and cosmetic outcome of recipient and donor sites assessed by investigators and subjects. Interim results of 14 patients: ReCell was associated with 27 and 15% repigmentation at 3 and 12 months compared to 11 and 12% with minigrafting, respectively. Both subjects and investigators thought ReCell provided a better cosmetic outcome at the donor site but most subjects preferred the speckled pigmentation at recipient sites of the minigrafting. Our results require more power for statistical comparison but suggest a potential beneficial use of the ReCell device in the treatment of stable vitiligo.

P74

Treatment of vitiligo hands by ReCell system associated with excimer lamp

M. Pascal, L. Valente
Centre Laser Espace Saint-Honoré, Paris, France

Vitiligo hands is a common localisation but most of the time, medical treatment (UVB TL01, excimer lamp or laser, tacrolimus or corticosteroids) is not efficient. Many surgeons recommend surgical procedures such as punch grafting, suction blister grafting, split-thickness skin grafting, cultured melanocytes or noncultured melanocytes transplantation. A preliminary report has been published in 2010 August about Recell system (non cultured autologous epidermal suspension) for the treatment of one case with a stable vitiligo hands by Cervelli and collegues (Department of Plastic and Reconstructive Surgery, Roma University, Italy). An excellent repigmentation has been noticed in this case. The authors consider this procedure as a simple and safe method. The goal of this study was to find a easier technic to separate melanocytes from epidermis before grafting and to avoid big loss of melanocytes after classic skin dermabrasion. One patient with a vitiligo hands resistant to uvb TL01 has been selected. All the vitiligo areas are treated by liquid nitrogen 24 h before the session. A thin skin sample is taken from thigh and treated by ReCell. Keratinocytes and melanocytes suspension is injected in blisters. After healing the skin is exposed to excimer lamp (QUANTEL) two times a week during 6 months. A repigmentation of 70% of treated area has been observed. No hypertrophic or cheloïd have been noticed. This procedure of melanocytes obtained by ReCell system injected in blisters seems to be a good and safe method for treating difficult areas as vitiligo hands.

P75

Role of wound bed nutrition in non cultured epidermal suspension transplantation in vitiligo

A. P. Holla, R. Kumar, D. Parsad, A. J. Kanwar, S. D. Mehta
Vitiligo Institute for Care and Research, New Delhi, India

It is usual practice to use special solutions in non cultured epidermal suspension transplantation procedure to achieve better outcome. Oozing serum at wound bed can play a significant role by giving nutrition to transplanted cells and having trypsin inhibitor action in the efficacy of procedure. To evaluate the role of wound bed nutrition in effectiveness of non cultured epidermal suspension transplantation where special solutions and special dressing are not used. Thirty-eight stable vitiligo patients (28 females, 10 males; age range 8–40 yr) included and 70 lesions treated. Non cultured epidermal suspension obtained from split thickness skin graft were suspended in phosphate buffered saline (PBS). Trypsin inhibitor and melanocyte medium were not used to prepare the suspension. This suspension was transplanted to dermabraded recipient area with a modified procedure using chlorhexidine gauze, PBS soaked gauze and Tegaderm and collagen dressing was not used in the procedure. During 6 months follow up results were assessed based on extent of repigmentation, colour match and adverse events. Repigmentation was successful (repigmentation >75%) in 63/70 lesions (90%). Color match at sixth month was excellent in 61/70 lesions (87.14%). No significant adverse events were reported. This study shows that there is no need of special solutions like melanocyte medium or trypsin inhibitor and collagen dressing to achieve better results in non cultured epidermal suspension transplantation where cells are ex vivo for short duration. Relying on wound bed nutrition due to oozing serum is equally effective, more safe and cheaper option.

P76

Clinical application of platelet-rich plasma in vitiligo: a pilot study

H.-K. Lim, M.-K. Shin, M.-H. Lee
Department of Dermatology, School of Medicine, Kyung Hee University, Seoul, Korea

Platelet-rich plasma (PRP) contains several growth factors and has been used as an effective treatment in various surgical and medical fields. Vitiligo is an acquired depigmenting disorder with no entirely satisfactory treatment. It was recently reported that not only melanocytes but also keratinocytes and fibroblasts are involved in pathogenesis of vitiligo in some ways. We conducted a pilot study to evaluated the efficacy and the safety of PRP for the treatment of vitiligo. A total of 19 patients with vitiligo were enrolled for a prospective open pilot study of 10 weeks. In each patients, symmetrically located and similar sized (3×3 cm^2) depigmented patches were selected for testing. Lesion on the right side of the body was treated with PRP, and lesion on the left side of the body was treated with normal saline. PRP and normal saline were injected intradermally into lesions spacing 0.5 cm between each injection (0.05 ml/each injection) by using a 1 ml syringe with a 26-gauge needle. This was repeated weekly for 10 weeks. We evaluated clinical outcome and the self assessment of patients every week, and checked possible side effects of the treatment. Sixteen patients completed the trial. A significant improvement was observed in two patients. No patient showed worsening of the disease. Side effects were minimal except for pain during injection. The results suggest that PRP does not effectively induce repigmentation in vitiligo. A few patients respond to this treatment. Considering our result and infinite potentiality of PRP, it is possible to apply as an alternative treatment modality for some vitiligo patients. Further in vitro studies are required to assess direct and indirect effect of PRP on melanocyte in microenvironment. A larger clinical trial can be helpful to determine the efficacy of PRP in treatment of vitiligo.

P77

Repigmentation of leukotrichia in vitiligo using non-cultured cellular grafting

E. Y. Gan, L. Y. T. Chiam, N. Van Geel, B. K. Goh
National Skin Centre, Singapore, Singapore

Vitiligo in hair-bearing areas is often associated with leukotrichia. Repigmentation of leukotrichia has been reported mainly with tissue and follicular hair grafts. This study aims to evaluate the repigmentation response of leukotrichia after non-cultured cellular grafting. We retrospectively reviewed patients with stable generalised and segmental vitiligo who underwent non-cultured cellular grafting from March 2008 to November 2010 in areas with leukotrichia. Based on clinical and photographic evaluation, leukotrichia repigmentation was graded at 3–12 months after grafting as 'poor', 'fair', 'good' or 'excellent', corresponding to a scale of 0–100% repigmentation with respective intervals of 25%. Eighty-four patients with vitiligo underwent cellular graft-

© 2011 The Authors. Journal Compilation © 2011 John Wiley & Sons A/S

ing, out of which 13 had grafting in areas with leukotrichia. Twelve patients (92%) had poor repigmentation of leukotrichia 3 months after transplant. One patient defaulted follow-up after this period. At 6-month, fair repigmentation was achieved in eight out of 12 remaining patients (67%). After 9–12 months, however, 91% (n = 10) of patients achieved good or excellent repigmentation. Only one patient has not reached the 9-month follow-up period. Leukotrichia of eyebrows yielded excellent repigmentation in eight out of nine patients whereas hairs on the limbs and scalp showed good repigmentation in two of two patients at 9–12 months. Good to excellent repigmentation of leukotrichia can be achieved with non-cultured cellular grafting, obviating the need for hair transplantation. We postulate that this occurs via retrograde migration of transplanted melanocytes or epidermal stem cells to the hair follicle bulb.

P78

Comparison of efficacy and side effect profile of oral PUVA versus oral PUVA sol in the treatment of vitiligo: a 36 week prospective study

S. Singh, S. Khandpur, V. K. Sharma, M. Ramam
Department of Dermatology, AIIMS, New Delhi, India

Both Oral PUVA and PUVA sol have been successfully used in vitiligo treatment. However, there is paucity of studies comparing the two therapies, especially under conditions of abundant sunlight where PUVA sol is more feasible. To compare the efficacy and side effects of oral PUVA versus oral PUVA sol therapy in generalized vitiligo. Comparative prospective clinical trial conducted on consecutive patients of generalized vitiligo allocated to either oral PUVA or PUVA sol groups treated for period of 36 weeks. Response to treatment was assessed using change in Lund & Browder (L & B) score for assessment of reduction in body surface area of involvement, patient global assessment (PGA) of improvement in vitiligo, investigator's global assessment (IGA) of extent of repigmentation, and quality of life (QOL) assessment using Tjioe et al. questionnaire. Thirty-five patients were recruited – 18 in PUVA and 17 in PUVA sol group. Mean percentage change in L & B score at 36 weeks was 46.4% in PUVA and 26.1% in PUVA sol group ($P = 0.06$), mean PGA score in PUVA was 4.58 and in PUVA sol group was 6 ($P = 0.13$), mean IGA score was 3.08 in PUVA and 1.79 in PUVA sol group ($P = 0.11$). QOL scores were significantly higher in PUVA group as compared to the PUVA sol group ($P = 0.04$). Side effects were comparable in two groups except for phototoxicity which was significantly more in PUVA group. PUVA is more efficacious than PUVA sol and also provides greater psychological benefit in treatment of generalized vitiligo but is associated with more phototoxic adverse effects.

P79

Serum 5-S-cysteinyldopa levels in psoriasis and vitiligo patients undergoing narrowband ultraviolet B phototherapy

K. Kikuchi, K. Wakamatsu, Y. Tada, S. Ito
Division of Dematology Tokyo, Japan

Narrowband UV-B (NBUVB), a new light source, is effective for treating generalized psoriasis and vitiligo without the use of psoralens. First, we measured the time course of changes in serum levels of the melanin-related metabolite 5-S-cysteinyldopa (5-S-CD) in psoriasis patients undergoing NBUVB phototherapy. Eleven Japanese patients with generalized psoriasis vulgaris received NBUVB treatment five times per week, with an initial dose of 0.1 J/cm^2. The dose was increased by 10–20% per treatment for more than 20 treatments. Serum samples were taken before, and 3, 7, 10, 14 and 28 days after the phototherapy. After 4 weeks of NBUVB treatment, nine of 11 patients were in remission. Two patients were dropped from the investigation before day 28 because of other complications. The mean level of 5-S-CD in serum was significantly elevated on days 7, 10 14, and 28 compared with that before the phototherapy. The serum 5-S-CD level peaked on day 10.
We also treated vitiligo outpatients with NBUVB twice a week. We measured serum 5-S-CD of seven vitiligo patients undergoing NBUVB phototherapy. Serum 5-S-CD level of vitiligo patients was significantly lower than that of normal controls or psoriasis patients (3.13 ± 0.24 nM versus 4.78 ± 0.80 nM, $P < 0.05$). Also serum 5-S-CD level of psoriasis patients was significantly higher than that of normal controls. Serum 5-S-CD level of vitiligo patients was significantly lower than that of normal controls. One patient who received NBUVB therapy five times per week showed sustained increased 5-S-CD levels. Even outpatient who received NBUVB therapy twice per week showed elevated 5-S-CD level up to 38.9 nM. Frequent sun-exposure of psoriasis patients as home-remedy might explain these differences.

P80

Novel aspects of melanocyte – keratinocyte interactions in vitro as a clue towards repigmentation in vitiligo

D. Keswell, L. M. Davids, S. H. Kidson
University of Cape Town, Cape Town, South Africa

Repigmentation of skin hypopigmentary disorders such as vitiligo is thought to involve the activation and migration of melanocyte precursors resident in the epidermal or hair follicle niches, into the denuded areas. The mechanism and mode of this migration is not well understood and may occur trans-epidermally, and/or along the basement membrane and/or in the dermis. In order to explore possible mechanisms of human melanocyte migration, primary human melanocytes and keratinocytes were co-cultured and the migration of melanocytes into the keratinocytes using lateral migration and transmembrane assays. Melanocytes were visualised by immunofluorescent detection and scanning electron microscopy and gene expression changes were quantified using qPCR. These assays demonstrated that human melanocytes were stimulated to actively migrate through intercellular spaces between keratinocytes both laterally and from the basolateral surfaces, suggesting that reciprocal signals stimulated both melanocyte migration and de-adhesion of intercellular connections between keratinocytes. Immunofluorescent and scanning electron microscopy analyses demonstrated that the melanocyte dendrites play a pivotal role by extending ahead of the melanocyte nucleus to explore the substrate and act as anchorage points. In addition, melanocyte migration through a 0.45 μm Millipore membrane demonstrated that the melanocyte nucleus is a highly malleable structure that aids in its migration through the intercellular spaces between the keratinocytes. Analysis of gene expression in these migrating cells using qRT-PCR indicated that c-Kit expression was increased in migrating melanocytes, suggesting that SCF/c-Kit signalling provided chemotactic and chemokinetic signals for melanocyte migration. This study provides further insight into the mechanism of melanocyte migration and potentially contributes towards improving therapies for hypopigmentary disorders such as vitiligo as well as providing further clues towards healing skin wounds.

 © 2011 The Authors. Journal Compilation © 2011 John Wiley & Sons A/S

P81

Study of oxidative stress in vitiligo

V. Mendiratta, J. Mal.
Department of Dermatology and STD, Lady Hardinge Medical College and Associated Hospitals, Delhi, India

Recent studies bring to light putative role of oxidative stress generated by reactive oxygen species in causing destruction of melanocytes in vitiligo. Estimation of serum levels of superoxide dismutase (SOD) and Glutathione peroxidase (GPx) in vitiligo. Cross sectional, investigative, case control study. One hundred subjects (>15 yr) were recruited for the study. Group 1 had 50 untreated patients with active vitiligo. Group 2 had 25 patients of stable vitiligo and 25 healthy controls who fulfilled the inclusion criteria. All were subjected to detailed history, blood investigations, thyroid function test, antinuclear antibody and rheumatoid factor assay. Estimation of superoxide dismutase activity, Glutathione peroxidase (PAGLIA and Valentine method) was performed in all. Vitiligo affected (16–25 yr) age group in 54%. M:F ratio was 1:2.13. V. vulgaris was the commonest (52%). Onset was at 11–20 yr in 44%. Family history was noted in 10%. Raised TSH levels were noted in 10% of the active vitiligo cases only. Mean value of (SOD) was 367 U/ml in active vitiligo, followed by 300 U/ml in vitiligo controls and lowest (237 U/ml) in healthy controls. Mean value for Glutathione peroxidase was (4011 U/ml) in active vitiligo, (3990 U/ml) in vitiligo controls and (3945 U/ml) in healthy controls. Level of superoxide dismutase (SOD) were significantly higher in active vitilgo as compared to controls. The present study reflects a fascilitatory role of oxidative stress in activity of vitiligo. Glutathione peroxidase levels showed an inconsistent pattern.

P82

Decreased isocitrate dehydrogenase expression renders melanocytes more vulnerable to oxidative stress

J. Y. Shin, J. Y. Kim, J. E. Do, M. R. Kim, S. H. Oh
Severance hospital, Seoul, Korea

Oxidative stress and a variety of antioxidant mechanisms have been implicated in the pathogenesis of vitiligo. In our previous study, NADP-dependent isocitrate dehydrogenase (ICDH) was found out as one of melanocyte antigens reacting to IgM antibodies in the sera of vitiligo patients through proteomics and 2-dimensional western blotting. ICDH has an antioxidant effect by supplying cytosolic NADPH, which acts as a cofactor for the production of glutathione, a well-known antioxidant. We tried to examine whether the decreased expression of ICDH is one of the reasons why melanocytes are especially vulnerable to oxidative stress. After confirming the expression of ICDH in both primary human melanocyte and immortalized mouse melanocyte cell line Melan-A, ICDH short interfering RNA (siRNA) was used to downregulate ICDH expression in Melan-A. Compared with the control group, the proportion of apoptotic and necrotic cells increased after ICDH was downregulated by ICDH siRNA and higher rate of apoptosis and necrosis was observed in melanocytes when the cells were exposed to H_2O_2. In addition, ICDH-silenced melanocytes showed a greater intensity of DCF fluorescence than scrambled cells. The ratio of glutathione disulfide (GSSG)/total glutathione in ICDH-knockdown melanocytes was much higher than that of the control. ICDH might be crucial to protect melanocytes against oxidative stress through efficient glutathione recycling. And this study suggests that ICDH can be associated with the pathogenesis of vitiligo.

P83

BSP-1 protects melanocytes against oxidative stress-induced cell death and hypopigmentation through MITF upregulation

E. Jung, S. Kim, M. Kim, S. Shin, J. Lee, D. Park
Biospectrum Life Science Institute (BLSI) Seongnam-Si, Gyeonggi-Do, Korea

The occurrence of oxidative stress has been proposed as a pathogenetic mechanism for melanocyte degeneration in vitiligo. Vitiligo is an acquired condition characterized by depigmented, cutaneous lesions that result from the death of pigment-producing cells, melanocytes. In the epidermis from subjects with active vitiligo, an increased production of H_2O_2 has been reported and is associated with reduced expression and activity of the antioxidant enzymes. In addition, oxidative stress induces hypopigmentation through downregulation of a microphthalmia (MITF) and MITF dependent-melanogenic enzymes which play important roles not only in the control of differentiation, but also in melanocyte survival. Recently, prostaglandin analogues have been reported to be effective on pigmentation of vitiligo lesions. Additionally, combination of antioxidants and melanogenic inducers was suggested to improve therapeutic efficiency for vitiligo. BSP-1 is kaempferol glycosides and isolated from the Cornus macrophylla Wall. BSP-1 has been reported to have inhibitory activity against 3-hydroxy-3-methylglutaryl-coenzyme A (HMG-CoA) reductase and quinone reductase 2 (QR2). The effects of BSP-1 on skin have remained unknown. This study was aimed to investigate protective effects of BSP-1 against oxidative stress and its mechanism in human epidermal melanocytes. In order to understand the cytoprotective role of BSP-1 on melanocytes, we performed cell viability test using MTT analysis and annexin-V/PI staining assay. Signaling pathways related to cell damage and pigmentation was investigated by measurement of ROS production, lipid peroxidation and expression of antioxidant enzymes and melanogenesis-related genes. We found that BSP-1 significantly inhibited oxidative stress-induced cell death, cellular ROS production and lipid peroxidation in melanocytes. In addition, BSP-1 increased expression of antioxidant enzymes including nrf2, HO-1, catalase and SOD. Furthermore, expression of MITF and its downstream melanogenic enzymes such as tyrosinase and trp-1 was increased by BSP-1. We found that expression of MITF-siRNA significantly reduced protective effect of BSP-1 on oxidative stress-induced cell death. The data support a role of BSP-1 in acute protection of cells to oxidative stress that precedes MITF activation. In this report, we present the novel use of BSP-1 for the prevention of oxidative stress and damage in melanocyt

P84

Study of CCN3 (Nov) expression in normal melanocytes and vitiligo skin

A.-S. Ricard, D. El Hajj Diab, C. Pain, A. Daubos, K. Ezzedine, A. Bibeyran, V. Guyonnet-Dupérat, A. Taïeb, M. Cario-André
INSERM U1035, Department of Dermatology, CHU de Bordeaux, Bordeaux, France

We have hypothesized that melanocytes disappear in vitiligo because they are weakly attached to basal membrane (melanocytorhagy). In the epidermis, the attachment of melanocytes to basal lamina is in part due to DDR1, which is under the control of CCN3 (Nov). DDR1 genetic variants have been associated with vitiligo in patients of different ethnic origin. Funukaga-Kalabis et al. (2006) have observed that inhibition of Nov induces the detachment of melanocytes. We have decided to study in parallel the expression of Nov and DDR1 in lesional and non-lesional skin of vitiligo patients and the impact of the inhibition of

© 2011 The Authors. Journal Compilation © 2011 John Wiley & Sons A/S

Nov. and DDR1 in melanocytes on their behaviour in reconstructed epidermis. In normal skin, we have observed that Nov is highly expressed in melanocytes as compared to keratinocytes. Expression of Nov in keratinocytes of lesional versus perilesional/non lesional skin of vitiligo patients was markedly different. In five of eight patients, expression of Nov in keratinocytes was decreased in peri lesional skin. Melanocytes in peri-lesional and non lesional vitiligo skin expressed Nov as in normal skin. In contrast with normal skin where Nov highly expressing cells were melanocytes, in vitiligo skin another subset of cells, mostly dermal, had a high Nov expression. These cells are probably lymphocytes T since we observed lymphocyte T infiltration in dermis and basal layer in most vitiligo samples. DDR1 expression seemed not modified in lesional skin as compared to perilesional/non-lesional skin. To inhibit DDR1 and Nov we have constructed vectors expressing both shRNA and GFP. In culture, we found that inhibition of Nov in melanocytes induced the inhibition of DDR1 whereas DDR1 inhibition had no effect on Nov expression. Using adhesion assays, we measured a significant inhibition of adhesion to collagen IV with sh Nov melanocytes. Melanocytes detachment was absent/limited when reconstructs were made with melanocytes transduced 1 day before reconstruction, whereas a significant melanocyte detachment was noted in case of shNov transduction 5 days prior reconstruction. However, we could not detect GFP in detached melanocytes but this result may be due to the fact that shNov and GFP were not under the same promoter. Thus, we have decided to reproduce these experiments using puromycin instead of GFP. In conclusion, in vitro and in vivo data suggest that Nov is implicated in vitiligo etiology.

P85

Inflammasome activation and nonsegmental vitiligo progression

K. Ezzedine, J. Marie, D Kovacs, T. Jouary, M. Picardo, A. Taieb, M. Cario-André
INSERM U1035, Department of Dermatology, CHU de Bordeaux, Bordeaux, France

Non segmental vitiligo is a chronic cutaneous disease characterized by bilateral patchy depigmentation. Data have suggested the central role of cytotoxic T lymphocytes in melanocytes disappearance. Recently a genome-wide analysis study has underlined the possible implication of NALP-1, a key protein of the inflammasome complex, in the pathogenesis of vitiligo. In fact, genomic variants of NALP-1 have been linked to increased susceptibility to autoimmune diseases and to vitiligo. However, the exact role of NALP-1 is the early phase of depigmentation as well as its relation with the T lymphocytes infiltration is still to be elucidated. In this context, we setup a study to evaluate the presence of NALP-1 in lesional and perilesional skin of non-segmental vitiligo patients and to search for a correlation between NALP-1, lymphocytic infiltrates and disease activity. Fourteen patients were enrolled after having given their written informed consent between June 2009 and September 2010. Biopies were taken from lesional and perilesional skin and disease activity was evaluated according to spreading as defined in the VETF questionnaire. The importance of NALP-1 immunostaining, CD4 and CD8 was evaluated as follows Grade 1: negative or absence of infiltrate Grade 2: mild staining/sparse infiltrate Grade 3: positive staining and/or infiltrate along the basal layer Grade 4: positive staining in all layers. NALP-1 was almost absent in lesional skin of most patients whether the disease was active or not whereas NALP-1 was strongly positive in five patients (grade 3 and 4) with active disease (spreading evaluated from 3 to 5). In addition, we noted a marked inflammatory infiltrate made of CD8 and CD4 T cells in peri-lesional skin of patients with active disease (grade 3 and 4 in patients with spreading ≥2), and to a lesser extent in lesional skin of active disease. On the contrary there was no infiltrate in most patients with limited disease activity. Our result suggests that NALP1 expression in non-segmental vitiligo is strongly linked to disease activity. In addition, NALP1 seems to be active at the border of lesions and may play an important role in disease progression.

P86

Is there a clinicopathologic correlation between clustered T8 lymphocytes infiltrate of the perilesional margin and the clinical aspect of vitiligo patches?

L. Benzekri
Department of Dermatology, Ibn Sina University Hospital, Mohammed V Souissi University Rabat, Morocco

Immune infiltrates in the skin of vitiligo patients with active disease were considered as a rare phenomenon which clinically determines an erythema in inflammatory vitiligo. Recently this infiltrate was detected in some non segmental vitiligo (NSV) without any local cutaneous inflammation. In NSV, two histopathological findings were reported according to the evolutivity of the disease: (i) In some spreading vitiligo macules 'microinflammatory'changes could be found spaning the margin (with dermal melanophages and clustered T8 lymphocytes in the epidermis in close apposition with melanocytes) and (iii) In stable vitiligo lesions, inflammatory infiltrate cannot be demonstrated. Our purpose was to investigate if these two histopathological findings could correspond to two different clinical aspects of the vitiligo macules. Forty-eight skin biopsies of the perilesional skin were obtained from 48 patients with NSV. Pictures were taken prior to the biopsy of vitiligo lesion. A routine staining of the section and an immunostaining of melanocytes and of T8 lymphocytes were performed for each skin samples. A clinicopathologic correlation was obtained in 84.8%. (i) Clustered T8 lymphocytes at the margin were found in extensive vitiligo macules frequently hypochromic or trichrome with not well delimited borders. (ii) Rare or few non clustered T8 lymphocytes were found in stable really achromic macules with well delimited borders. Many authors considered this clustered inflammatory infiltrate of the margin as a marker of local autoimmunity of vitiligo. We propose that, with only a careful clinical examination, we could determine with a correct reliability, the probability of this local immune process in view of melanocytes grafting.

P87

Plucked Hair Follicles as a powerful tool to monitor pigmentation markers

M. Giesen, T. Goerlach, S. Gruedl, G. Fuhrmann, M. Briese, G. Scheel, R. Paus, D. Petersohn, T. Förster
Henkel AG & Co KGaA, R&D Cosmetics, Duesseldorf, Germany

The human hair follicle is a highly specialized skin appendage continuously renewing itself including the whole machinery of the pigment producing unit to create the visible, pigmented hair shaft. The complex biological mechanisms controlling pigmentation and also the greying process are still under evaluation and are addressed in a plurality of research activities. However, the analysis and monitoring of human follicular pigmentation markers in vivo to elucidate molecular pathways or control follicular response on certain treatments remains difficult, if invasive techniques such as taking biopsies should be avoided. Providing a simple and rapid method to monitor the profile of pigmentation markers in the follicular pigmentary unit of pigmented and grey hairs. To evaluate the profile of pigmentation markers in the human hair follicle pigmented and grey hairs of 28 individuals have been extracted and analysed using RT-PCR and immuno-

 © 2011 The Authors. Journal Compilation © 2011 John Wiley & Sons A/S

histochemistry. We showed that plucked human hair follicles still comprise parts of the pigmentary unit, demonstrating the presence respectively the reduction or absence of several melanogenetic parameters in pigmented and grey hair follicles. Parameters evaluated include SCF, ckit, POMC, MCR1, MART 1, TRP1 and gp100 using RT-PCR. To confirm correct protein expression gp100, MART1 and TRP1 has been evaluated via specific antibody staining. Furthermore, enzymatic tyrosinase activity has been proven in sections of plucked hair follicles. The results demonstrate that plucked hair follicles are a powerful tool to monitor pigmentation markers in vivo and might help to elucidate the molecular events underlying the complex interactions of the melanogenetic process, its disturbance and follicular response to bioactive treatment.

P88

A new approach to preserve melanin content in the hair follicle

C. Gondran, A. Perrin, C. Meyrignac, C. Dal Farra, N. Domloge
ISP Vincience, Sophia-Antipolis, France

Hair graying (canities) is a natural process accompanying human aging, which results in the production of less and less melanin pigment by follicular melanocytes. The aim of the present study was to investigate the modulation of melanogenesis pathway in cultured NHEM (normal human epidermal melanocytes) and ex vivo human scalp skin biopsies, containing hair follicles. NHEM were treated with a biofunctional compound, designed to modulate melanin synthesis. After 48 h treatment, the following markers of melanogenesis were examined by immunostaining: MITF, tyrosinase, TRP-1, Pmel17 and the receptor of stem cell factor, c-kit. Melanin content in the hair follicle was studied using Fontana–Masson staining. Our results showed that the key markers of the melanogenic pathway were increased following application of the biofunctional compound in cultured NHEM. Regarding MITF expression, we observed an increase both in the cytoplasm and in the nucleus of treated cells. Treated NHEM cells also showed a higher level of c-kit, whose enhancement has been related to the level of hair pigmentation. Moreover, treated hair follicles showed an increased level of tyrosinase staining and melanin content in the hair bulb. In conclusion, our results introduce an interesting way to maintain melanin content in the hair follicle.

P89

Characterization of progressive hair whitening and roan coat colour phenotypes associated with overexpression of Strawberry Notch homolog 2 gene in the melanocyte lineage

E. Reyes-Gomez, N. da Silva, S. Gadin-Czerw, J.-J. Panthier, G. Aubin-Houzelstein
UMR955 INRA-ENVA Génétique Fonctionnelle et MédicaleMaisons Alfort, France

Strawberry Notch homolog 2 (Sbno2) is the mouse homolog of the sno gene, a Notch modifier in Drosophila. It was identified as a candidate gene for patchwork mutation. (pwk/pwk) in which mice display a unique roan coat colour characterized by a mixture of white and black hairs without gray hairs. We found that Sbno2 was overexpressed in the skin of patchwork embryos at the end of embryogenesis. Our objective was to test whether Sbno2 overexpression was causative of the patchwork phenotype. We generated Tg(Dct::Sbno2) mice that overexpress Sbno2 in Dopachrome tautomerase (DCT) -expressing cells. The transgenics were backcrossed on the C57BL/6J background for 10 generations then crossed with mice carrying either the Tg(Dct::lacZ) or the Pax3GFP reporter genes. Hair follicles from double mutants were isolated at different ages ranging from 8 days to 1 yr and the distribution of ß-galactosidase (ßgal)-positive and GFP-positive cells was studied. At birth, Tg(Dct::Sbno2) mice had a wild-type coat except for their white tail tip, belly spot and feet. They started whitening at 3 months of age, exhibiting a roan phenotype that persisted throughout life. However the coat never turned entirely white. In Tg(Dct::Sbno2); Tg(Dct::lacZ) hair follicles, the number of βgal-positive cells was reduced compared to that in controls at every time point studied. In particular, the number of βgal-positive cells in the bulge decreased with every hair cycle, suggesting a defect in the maintenance of melanocyte stem cells. Surprisingly, in Tg(Dct::Sbno2) mice, we repeatedly observed a prominent perifollicular network resembling cutaneous nerve plexuses that expressed both the Tg(Dct::lacZ) and the Pax3GFP reporter genes. To determine whether the network originated from pigment cells, we crossed the transgenics with MitfVga9/Vga9 mice that lack pigment cells in their coat. The network was present around Tg(Dct::Sbno2); Tg(Dct::lacZ); MitfVga9/Vga9 hair follicles, suggesting that it does not derive from pigment cells. Our data suggest that overexpression of Sbno2 in DCT-expressing cells is causative of the roan phenotype and leads to a hair whitening phenotype probably linked to a disruption in melanocyte stem cells maintenance. The presence of a nerve-like perifollicular network in Tg(Dct::Sbno2) mice either having or devoid of pigment cells suggests a disruption in the differentiation of a DCT-positive precursor not belonging to the melanocytes.

P90

Reduced scavenging abilities of premature graying hair bulbs against hydroxyl free radicals: direct evidence from an electron spin resonance (ESR) study

T.-C. Lei
Department of Dermatology, Renmin Hospital of Wuhan University, Wuhan, China

The purpose of this study is to investigate whether the premature graying hair bulb has intrinsic deficiency to scavenge oxidative stress and whether MCs and its precursor are still present in the graying hair bulbs and the bulge regions. (i) The occipital or/temporal scalp samples were obtained from of patients undergoing plastic surgery with informed consent. Single whole anagen hair follicle was isolated by microdissection, and then its bulb and the middle part in where the arrector pili muscle inserts into the bulge region were precisely excised under a binocular steromicroscope. These isolated folliculе segments were used for further H_2O_2-scarvenging assay and total RNA extraction, respectively. (ii) Transient hydroxyl free radical was generated via a Fenton reaction and trapped by DMPO, same aliquots of the hair bulb or/bulge segment was added in this reaction system to measure the DMPO-OH signals using an ESR spectrometer. (iii) Thirty hair bulbs or/bulge segments were pooled in an RNase-free tube with 400 μl Trizol reagent for the extraction of total RNA. Standard RT-PCR technique was carried out to semi-quantitatively analyze the mRNA level of catalase, mitf, tyrosiase, tyrp1 and tyrp2.

Results: (i) Loss of pigment in both hair bulb and bulge segments was seen in graying hair follicles; (ii) The relative DMPO-OH signals were dramatically increased in graying hair bulb, no change in graying bulge segment, comparing with pigmented ones. (iii) The mRNA level of catalase was decreased in graying hair bulb, but no change in graying bulge segment. The transcripts of melanogenic proteins (mitf, tyrosiase, and tyrp2) were undetectable in both graying segments. Our findings

© 2011 The Authors. Journal Compilation © 2011 John Wiley & Sons A/S

indicate that graying hair bulb has a decreased scavenging activity on hydroxyl free radical, thereby causing H_2O_2 accumulation in the graying bulbs and even ultimately inducing apoptosis of the oxidative stress-sensitive MCs. This study was supported by a research grant of the CMA-Loreal hair foundation 2010 and by NSFC Grant 81071308.

P91

Different contributions of pigmented spots in age and attractiveness perception: a cross-cultural approach

A. Porcheron, J. Latreille, R. Jdid, C. Guinot, E. Tschachler, F. Morizot
CE.R.I.E.S, Neuilly sur Seine, France

Comparisons of skin ageing in Chinese and Caucasian women show differences between the two populations. Chinese women are affected by wrinkles later than Caucasian women, whereas a higher pigmented-spot occurrence is observed in Chinese women. Interestingly, facial age-related features have been found to have a strongest correlation with perceived age than with chronological age in both populations. Two studies were conducted to investigate the respective contribution of pigmented spots, wrinkles and skin sagging on age perception in Caucasian and Chinese women using virtual manipulation of facial images. In the first study, French female participants were asked to estimate the age difference between each manipulated Caucasian face and the original face. In the second study, Chinese female participants had to estimate the age difference between the manipulated faces and the original ones in both sets of Caucasian and Chinese faces. In addition, Chinese participants were asked to rate attractiveness. For French participants, the highest age difference was observed after the reduction of skin sagging of Caucasian faces, whereas they were equally sensitive to the reduction of wrinkles and pigmented spots. Interestingly, the Chinese participants judged the Caucasian faces younger after wrinkles/sagging reduction than after pigmented spots reduction, whereas they were much more influenced by the correction of age spots of Chinese faces. Unlike for age estimation, Chinese participants judged faces with reduction of pigmented spots the most attractive whatever the origin of the faces. Our findings highlight a different contribution of the pigmented spots in age and attractiveness perception according to the faces' origin and the observers' origin.

P92

Solar lentigines: an earlier occurence in Japanese women than in Chinese and Korean women

R. Jdid, E. Mauger, A. Porcheron, C. Guinot, E. Tschachler, F. Morizot
CERIES, Neuilly sur Seine, France

Pigmented irregularities appear to be prominent features of photoaging in Asian populations. The objective of our study was to perform a detailed comparison using a strict definition of solar lentigines in Japanese, Chinese, Korean and French women. Investigators graded the severity of pigmentation irregularities from high resolution facial images of 281 French, 256 Japanese, 301 Korean and 301 Chinese women using photographic reference scales. The four groups did not differ significantly with regard to age. Furthermore, no major differences of skin color were found, except that fair to very fair skin were only found in French women. Self-reported lifetime sun exposure was found to be higher in Chinese women; and skin dryness was more frequently reported in Chinese and Korean women. An early onset of pigmented irregularities was revealed in Japanese women: 46% among the 35–49 age group who had already pigmented spots of grade 2 or more. By contrast, lentigines were found to appear later in French Chinese and Korean women than in Japanese women. In older age groups, the severity of age spots was found higher in the three Asian populations than in the French one. Our results demonstrate distinct differences in the age of occurrence of pigmented irregularities in age-matched groups of women from Japan, Korea, China and France, which can be explained by exposure to different environmental and cultural factors.

P93

Alteration of epidermis junctions in human solar Lentigo

E. Noblesse, P. Schaeffer, R. Kurfurst, C. Nizard, S. Schnebert, E. Perrier
LVMH Recherche, St Jean de Braye, France

Solar lentigo is a common component of photoaged skin. It is characterized by hyperpigmented aging spot which appear in chronically irradiated skin mostly after age 50. Previous studies showed that keratinocytes junctions contribute to the maintenance of epithelial barrier functions, proliferation/differentiation equilibrium and epithelial cell polarity, process which are linked with the natural process of melanin evacuation. A comparison between lesional, perilesional and normal skin from biopsies of 15 female patients was performed using histology, transmission electron microscopy and immunofluorescence of DSG1, ZO-1 and E.cadherin proteins. The ZO-1, E.cadherin and DSG1 expression are evaluated by immunostaining and image analysis to explore the structural aspect of tight junctions (TJs), adherens junctions and desmosomes. Ultrastructure study shows important modifications of lesional skin in comparison with adjacent normal skin: a hyperpigmented basal layer with melanin accumulation, an elongation of the epidermis rete ridges and a disorganization and disruption of dermal epidermal junction associated with an increases of keratinocyte basal microvillosity The study of keratinocytes junctions ZO-1, E.cadherin and DSG1 expression shows a significant decrease in the epidermis granular layers, gradually from perilesional (–40, –44 and –20% respectively) to lesional area (–68, –75, –68% respectively) in comparison with the adjacent normal skin. Structural analysis of TJs network shows that in the non lesional epidermis, ZO-1 forms a continuous network at the stratum granulosum. When it progresses to the spot area, this network is gradually disorganised in perilesional skin (–28%) and tended to fully disappear in some patients in lesional skin (–51%). In a polarized system such as normal human skin, disruption of the epidermis junctions affects normal turn-over of the living epidermis. Necessary for the normal process of keratinocytes differentiation, cell movements are disrupted and lead to a decrease in evacuation of the melanin overproduction which is associated to lentigo formation. Taking together, these results open a new strategy to reduce and prevent solar lentigo in human skin.

P94

Insights into the etiology of solar lentigines through its microRNA and mRNA profile

S. Socha, N. Pauloski, J. Huertas, B. Potterf, W. Lathrop, C. Bosko, H. Meldrum
Unilever R&D, Trumbull, USA

Solar lentigines are hyperpigmented lesions on photodamaged surfaces of the skin. They have been characterized histopathologically as having a hyperpigmented basal layer and elongated rete ridges. Here we examined the expression profile of microRNAs in lesional and non-lesional areas of skin to gain more insight into the etiology of these lesions. MicroRNAs

 © 2011 The Authors. Journal Compilation © 2011 John Wiley & Sons A/S

(miRNAs) are small endogenous RNA molecules that play an important role in the regulation of gene expression. miRNAs have recently been shown to play a pivotal a role in diverse developmental and cellular processes and have been linked to a variety of skin diseases. Disruption of the miRNA expression has been shown to be involved in wound healing and inflammatory skin conditions. Through the analysis of over 160 samples via microRNA and mRNA arrays we have identified characteristic expression profiles in solar lentigines, distinct from that of photo-exposed skin. The microRNA data highlights the importance of the immune/inflammatory and cellular communication systems in the development and maintenance of solar lentigines. Target genes of differentially expressed microRNAs were confirmed by mRNA arrays and qPCR. Our mRNA array data confirms that ETBR, SILV and tyrosinase, which have been previously reported to be up-regulated in solar lentigines, are differentially expressed in lesions versus sun-exposed (peri-lesion) skin. We have also confirmed that some melanosome transfer genes and FGF-7 and FGFR2 are differentially expressed in perilesional skin compared to sun-protected skin. However, these are not differentially expressed between solar lentigines and photoexposed skin. This data suggests that FGF7 and FGFR2 are up-regulated in response to UV exposure but do not play a pivotal role in solar lentigines development or maintenance. Through this approach we have identified novel genes that are involved in the etiology of solar lentigo.

P95

Periorbital hyperpigmentation amongst the Singaporean population: a proposed classification and epidemiological review

S. Thang, H. Ranu, A. Burger, B. K. Goh, C. L. Goh
Department of Dermatology, Singapore

Periorbital hyperpigmentation refers to a condition that presents with a dark area on the lower and occasionally on the upper eyelids. It is an ill-defined condition. Numerous attempts have been made to classify the condition but the pathogenesis of this condition remains unknown. This study was conducted to assess the prevalence and characteristics of periorbital hyperpigmentation in patients attending a tertiary dermatology referral centre in Singapore. We also propose a classification for periorbital hyperpigmentation. One thousand consecutive patients aged 18–70 yr old, attending the tertiary dermatology referral centre in Singapore were enrolled into the study to assess for periorbital hyperpigmentation. Two hundred consecutive patients who reported having periorbital hyperpigmentation were examined and investigated to define the causes of their periorbital hyperpigmentation. Of the 200, 16 cases were excluded from the study because of refusal to be photographed or absence of periorbital hyperpigmentation visible on examination. The causes of periorbital hyperpigmentation were determined by taking a detailed history, clinical examination and assessment of photographs by all three authors. The mexameter was used to measure the degree of melanin and erythema on the affected parts of the eyelids. The commonest type of periorbital hyperpigmentation observed was the 'vascular' type (41.8%) followed by the 'constitutional' type (38.6%), 'post inflammatory hyperpigmentation' type (12%) and lastly by 'shadow effects' (11.4%). Subgroup analysis of the 'vascular' type showed that 66.2% were due to 'capillaries', 15.6% were due to prominent underlying blue 'veins' and 18.2% were due to a combination of prominent capillaries and veins. The 'vascular' type of periorbital hyperpigmentation was predominantly seen in the lighter skin type I–IV e.g. the Chinese, where-as the constitutional type was most commonly seen in the darker skin type V–VI e.g. the Indians and Malays. These findings were statistically tested using Tukey's multiple comparisons test among proportions and found to be statistically significant. In conclusion, with this study we propose a classification for periorbital hyperpigmentation. This is a preliminary study to identify the predominant type of periorbital hyperpigmentation in the local population.

P96

Study of pigmented cosmetic dermatitis

G. Smita
LTMMC-MGH, Mumbai, India

Pigmented cosmetic dermatitis is a term proposed by Nakayama. It is a variant of pigmented contact dermatitis where cosmetics ingredients are the causative allergen and face is predominant site. Clinically, it is characterized by diffuse or patchy brown pigmentation on the face. Here, we present 56 cases with pigmentation on face. To study the common allergens in cases of pigmented cosmetic dermatitis. Patients with pigmentation and mild dermatitis on face were included. History of cosmetic use was present in all cases. Patients with severe dermatitis were excluded.

Consent and detailed history was taken. Patients were patch tested with universal and cosmetic series and as is wherever possible. The readings were taken on day 2, 4, 7 according to ICDRG grading. Fifty-six patients fulfilled the criteria. Females (n = 38, 67.85%) outnumbered the males (n = 18, 32.14%). The median age for males (38.83) and females (33.13) were same. The occupation of maximum people was homemaker. The duration of complaints ranged from 1 month to 15 yr. The contact of cosmetics was in form of talcum powder, fair & lovely, kumkum, shaving cream, beauty creams, perfumes, hair dye. The face, cheek and forehead were maximally affected. The positive results were seen in 25 (44.64%). Female (n = 18.72%) outnumbered males (n = 7.28%). The most frequently affected age group was 20–40 yr. The number of sensitization were 60, sensitization index was 2.4. The allergens were fragrance (16.66%), thiomersal (8.33%), emulsifiers (18.33%). In pigmented cosmetic dermatitis not only fragrances but emulsifiers and preservatives also play a role.

P97

Patch test in facial melanosis

A. Dandale, S. Chavan, R. Dhurat
LTMMC & LTM Hospital, Sion, Mumbai, Mumbai, India

Facial melanosis is a common manifestation of many dermatoses, commonly allergic contact dermatitis caused by allergens such as fragrances, nickel, preservatives, PPD, photocontact dermatitis, sesquiterpine lactone containing plants, grooming tools like cosmetics, and rubber products. To define epidemiological characteristics of patients with facial melanosis and to determine the prevalence of common allergens by Patch test. A retrospective study of 81 patients presenting with facial melanosis over a period of 4 yr was conducted. Patients were patch tested with Universal, cosmetic series and relevant products brought by the patients, with further photopatch testing. They were evaluated on day 3 and 7 as per ICDRG score and the results were documented. It was observed that the maximum number of patients were female in middle aged group having complaints for more than 6 months of duration. The most common diagnosis was allergic contact dermatitis (hair dye, cosmetics, bindi, kumkum, topical medications, plants), photocontact dermatitis, atopic dermatitis, seborrheic dermatitis, lichen planus pigmentosus. Patch test were positive in 35 patients (43.2%) out of which nickel was most common allergen seen in eight patients (22.9%) followed by fragrance mix (11.4%), thiomersal (11.4%), potassium dichromate (8.6%),

© 2011 The Authors. Journal Compilation © 2011 John Wiley & Sons A/S

PPD (8.6%), nail paint (5.7%), colophony (5.7%), disperse orange (5.7%). Photopatch tests was negative in all patients. Thus we conclude that allergic contact dermatitis is a common cause of facial melanosis and nickel is the commoest allergen causing it.

P98

Idiopathic eruptive macular pigmentation in a 9 yr old girl

A. Salhi, F. Siebenhaar, M. Maurer, A. Taïeb
University of Algiers, Alger, Algérie

Idiopathic eruptive macular pigmentation is a distinct clinicopathologic and histologic entity first described by Sakae in 1941. It is caracterised by asymptomatic pigmented macules of trunk limbs and neck appearing spontaneously. We present the case of a 9 yr old girl. She presented numérous pigmented macules on the trunk, limbs and neck. The face, soles and palms were spared. She has been burnt on the dorsum of her foot with boild water 15 days before the eruption appears. There was no general sign nor pruritus. A cutaneous biopsy for histologic analysis showed elongated rete ridges with basal hyperpigmentation and some melanocytes. The dermis was unaffected. The Giemsa stain showed no accumulation or increased numbers of mast cells. So a cutaneous mastocytosis could be excluded. The patient did not receive any treatment. The lesions now present for 1 yr are still present with a slight tendency to regression. Treatment of this asymptomatic condition is unnecessary because spontaneous resolution of the lesions is to be expected within several months to a few years.

P99

Narrow-band UVB may improve pigmented spots in patients with neurofibromatosis 1

J. Nakayama, T. Mori, S.-I. Imafuku
Department of Dermatology, Fukuoka University Faculty of Medicine, Fukuoka, Japan

Café-au-lait spots or ephelides-like spots are one of the major problems of patients with NF1. Recently, we observed slight improvement of the spots after irradiation of NB-UVB because of the concomitant pruritus of the skin of the patients. All of the patients irradiated with NB-UVB are satisfied with the improvement of the pigmented spots by questionnaire. The mechanism of the improvement by NB-UVB is not clear at present, but it may be either due to the induction of activated vitamin D3 in the skin of the patients or suppression of mast cells infiltrating to the pigmented lesions. We have previously found that a vitamin D3 analogue inhibited melanocyte growth in vitro, and topical application of the vitamin D3 ointment improved café-au lait spots in vivo. Therefore, it is now in progress of investigating whether NB-UVB induces activated vitamin D3 in the keratinocytes or other cells in vitro and also whether NB-UVB increased activated vitamin D3 in the periphery blood of the irradiated patients in vivo.

P100

Neurofibromatosis type I in vitro model using human embryonic stem cells

L. Larribère, X. Nissan, M. Saidani, C. Baldeschi, M. Pechanski
STEM, Evry, France

Neurofibromatosis type I (NF1) or Von Recklinghausen's disease is one of the most frequent genetic disease (1/3000) for which no specifique treatment exists. Clinicial manifestations are numerous and variable. In particular, cutaneous manifestations comprise neurofibromas, pigmented 'café au lait' macules (CALM), axillary and inguinal lentigines, and a general diffuse hyperpigmentation. The origin of this pathology comes from NF1 mutations leading to a non-functional neurofibromin, a RAS negative regulator. To date, the molecular mechanisms linking NF1 mutations to those skin lesions are poorly understood and murine models cannot bring pertinent informations due to the skin anatomic differencies with humans. In addition, patient skin biopsies are difficult to access and do not provide enough material to conduct deep studies on NF1-mutated human skin cells and melanocytes in particular. Here, we are bringing an original and unique in vitro NF1 model based on the use of human embryonic stem cells (HESC). Indeed, we were able to differentiate NF1-mutated HESC (obtained from pre-implantation genetic diagnosis) towards the skin lineages and to generate at will a theorically unlimited NF1-mutated human melanocyte population. This model allows us to study the molecular mechanisms involved in NF1 ethiopathology and to better understand how NF1 mutations can lead to benign cutaneous lesions. In particular, we are looking at the activation status of the signalling pathways downstream RAS: MAPK and AKT/mTOR.

P101

Linear and whorled nevoid hypermelanosis and progressive cribriform and zosteriform hyperpigmentation in Korea

C. J. Park, H. J. Lee, H. S. Kim, J. Y. Lee, H. O. Kim, Y. M. Park
Department of Dermatology, Seoul St Mary's Hospital, Seoul, Korea

Linear and whorled nevoid hypermelanosis (LWNH) is a rare skin condition characterized by swirls and whorls of hyperpigmented macules in a streaky configuration along the Lines of Blaschko, without preceding inflammation or atrophy. LWNH was first described as a distinctive entity in 1988 by Kalter. Histologically, it showed hyperpigmentation of basal layer without pigmentary incontinence or increase in dermal melanophage. Progressive cribriform and zosteriform hyperpigmentation (PCZH) was first described in 1978 by Rower and his colleagues as following clinical characteristics: a single area of uniformly-tan, cribriform, macular pigmentation in a zosteriform distribution, histologically a mild increase of melanin pigment in the basal layer and complete absence of nevus cells. LWNH has been used to encompass as wide-spectrum of clinical entities. PCZH is thought to be a variant of LWNH. Recently, we reviewed clinical and histological features of three cases of LWNH and 10 cases of PCZH from our hospital records for the past 5 yr. Additionaly, we further analyzed 12 cases of PCZH and LWNH from the Korean Dermatological Literature. We herein analyzed Korean cases of both diseases and reviewed the literature. In the PCZH, the age at onset ranged from birth to 64 yr and in the LWNH, the age at onset ranged from birth to 14 yr. Associated abnormalities including congenital hemi-hypertrophy, neuro-developmental delay and Becker's nevus were reported.

P102

Treating hyperpigmentation in dark skinned patients

N. Saedi, A. Ganesan
Department of Dermatology, University of California, Irvine, Dana Point, USA

The treatment of hyperpigmentation in darker skinned patients (Fitzpatrick Type III–VI) has remained challenging for dermatologists. To date, there are no studies conducted on hyperpigmentation under the eyes, axilla, and neck in darker skinned patients. This survey was designed to assess current treatments of hyperpigmentation in these areas.

 © 2011 The Authors. Journal Compilation © 2011 John Wiley & Sons A/S

With approval from the IRB at UC Irvine, an electronic survey was sent to practicing dermatologists. The survey contained 18 questions regarding the approach to evaluating and treating hyperpigmentation under the eyes, in the axilla, and along the neck. Fifty dermatologists completed the survey, and 46 (92%) reported treating patients with darker skin. The ethnic groups treated were Latino (97.8%), African American (97.8%), Middle Eastern (77.6%), and Asian (88.9%). Thirty-six reported treating patients with hyperpigmentation under the eyes, and 22 (61.1%) thought the hyperpigmentation was a result of idiopathic increase in melanin deposition. Hydroquinone (84.4%) and a combination of hydroquinone and tretinoin (69.7%) were the most commonly used treatments. Forty-two responded to treating hyperpigmentation in the axilla and most thought it was related to acanthosis nigricans (69.0%) or contact dermatitis (59.5%). Forty responded to treating hyperpigmentation on the neck, and most treated the condition with hydroquinone (66%). Treatments for these three areas were not found to be effective Hyperpigmentation under the eyes, under the arms, or on the neck is a significant problem in darker skinned patients which is refractory to currently available treatments, highlighting the necessity of developing treatment approaches directed towards this population. Two cases of hyperpigmentation on the neck are presented, describing a new entity that primarily affects dark skinned individuals.

P103

Colchicine in the treatment of lichen planus pigmentosus

A. J. Kanwar, D. Parsad
Department of Dermatology, PIGMER, Chandigarh, India

Lichen planus pigmentosus is a common pigmentary disorder in Indian population. It is a cause of cosmetic disfigurement. The available treatment modalities for this cosmetically disfiguring disorder are very limited and not uniformly effective. We tried to find out the effectiveness and safety of colchicine in patients with lichen planus pigmentosus. A total of 37 patients diagnosed clinically as lichen pigmentosus attending the pigmentary clinic of department of dermatology, venereology and leprology at Postgraduate Institute of Medical Education and Research, Chandigarh. All the patients included in the study were started on colchicine 0.5 mg twice daily initially for 2 weeks and then the dose was increased to maximum of 0.5 mg thrice daily. Response to therapy was recorded at each visit. Pre and post treatment photographs was taken in few selected patients. Adverse effects related to the drug were noted if any. Out of the total of 37 patients one patient withdrew because of gastrointestinal side effects while six patients were lost to follow up were thus excluded from the analysis. Out of the remaining 30 patients 22 were males and eight females. Duration of treatment ranged from 2 months to 1 yr. Improvement was more marked and noticeable in patients who had taken colchicine for longer period of time (>4 months).

Thirty to eighty percent reduction in the intensity of pigmentation was noted in 20 patients. No improvement was seen in five patients. To conclude colchicine appears to be a safe and effective drug for otherwise difficult to treat condition like LPP.

P104

Oral tranexamic acid with laser treatments in melasma patients

H. S. Park, H. H. Cho, S. Cho, J. H. Lee
Department of Dermatology, Seoul National University Boramae Hospital, Seoul, Korea

A plasmin inhibitor and lysine analog, trans-4-aminomethylcyclohexanecarboxylicacid (TA) has been reported to prevent ultraviolet light induced pigmentation when topically applied in guina pig. Topical or intradermally injected TA has been uwidely used in melasma treatment. However, no controlled study showing the clinical effects of oral tranexamic acid on melasma has been performed until now. Authors investigated the clinical effects of oral TA on melasma patients. Patients with mixed type melasma were divided into two treatment groups. In one group, patients received oral TA (500 mg daily) and intense pulsed light with low fluence QS Nd:YAG laser and patients in the other group were treated with only IPL and QS Nd:YAG laser. Photograph documentation was done. Modified MASI score were checked and melanin index were measured at each visit. All patients completed the treatments of one time IPL and subsequent four times of low fluence QS Nd: YAG lasers. Oral TA group showed dramatic decrease in melanin index after IPL compared with laser only group. The comparison of modified MASI score in each group did not show any significant differences. However, patients with facial erythema and chronic sun damage signs reported better results when they were on oral TA during treatments. Oral TA can be a good supplementation in treatment of melasma patients especially who have more sundamaged skin and facial erythema.

P105

Oral tranexamic acid in the treatment of melasma refractory to topical therapy

A. W.-M. Tan, P. Sen, S.-H. Chua, B.-K. Goh
National Skin Centre, Singapore

Melasma is a common acquired hyperpigmentary disorder, particularly among Asians and Hispanics. Although genetic influence plays an important role, the exact pathomechanism of melasma remains incompletely understood. Aberrations in the lesional melanocytes are not the only aetiological factors in this hyperpigmentary disorder, as abnormalities in the surrounding keratinocytes, fibroblasts and endothelial cells have been observed. Tranexamic acid or trans-4 aminomethylcyclohexanecarboxylic acid, is an antifibrinolytic agent that inhibits plasminogen activator, through the reversible blockade of lysine-binding sites on plasminogen molecules. Tranexamic acid has been found to inhibit melanin synthesis in melanocytes by interfering with the interaction of melanocytes and keratinocytes through inhibition of the plasminogen/plasmin system.

Our objective was to evaluate the therapeutic effects of oral tranexamic acid in the treatment of melasma refractory to topical skin lightening agents. This study is a retrospective analysis of patients with melasma, recruited from a tertiary dermatological centre in Singapore between 1st August 2009 and 31st April 2011. The patients chosen had refractory melasma who were treated with oral tranexamic acid 250 mg twice daily in addition to pre-existing combination topical therapy. Patients who had a history of severe renal failure, thrombophilic disorders or thromboembolic disease, or hypersensitivity to tranexamic acid were excluded. Objective assessment using the Physician's Global Assessment (PGA) and Melasma Area and Severity Index (MASI) scores were performed based on post-hoc analysis of photographic records by three independent physicians. A paired t-test was used to evaluate the changes in the MASI scores pre- and post-treatment. Statistical significance was defined as P-value <0.05. Twenty-five patients were treated with tranexamic acid for a mean period of 3.7 ± 0.33 months, in addition to combination topical therapy. Their mean age was 47.2 ± 1.61 yr. The mean MASI scores after tranexamic acid treatment (2.6 ± 0.43) were significantly lower (P < 0.01) than those prior to treatment (10.1 ± 1.13). The mean improvement in scores was 70.1 ± 4.24%. The follow-up period was up to 6 months. Two (8%) patients relapsed after stopping tranexamic acid. Low dose oral tranexamic acid can serve as a safe and useful adjunct in the treatment of refractory melasma.

© 2011 The Authors. Journal Compilation © 2011 John Wiley & Sons A/S

P106

Comparative study of 15% TCA peel versus 35% glycolic acid peel for the treatment of melasma

N. Puri
Ludhiana, India

Chemical peels are a mainstay in the cosmetic practitioner's armamentarium because they can be used to treat some skin disorders and can provide an aesthetic benefit. Chemical peels are used to create an injury of a specific skin depth with the goal of stimulating new skin growth and improving surface texture and appearance. The exfoliative effect of chemical peels stimulates new epidermal growth and collagen with more evenly distributed melanin. Chemical peels are classified by the depth of action into superficial, medium, and deep peels. We selected 30 patients aged between 20 and 50 yr from the dermatology outpatient department for the study. In group I patients, 15% TCA peel was used and in group II patients 35% Glycolic acid was used. The pattern of melanosis was of malar type in 40% patients, mixed in 40% patients and centrofacial in 20% patients. It was seen that the commonest cause of melasma in our study was after pregnancy in 40% patients, oral contraceptive use was associated with melasma in 30% patients and outdoor occupation was the cause of melasma in 30% patients. Subjective response as graded by the patient showed good or very good response in 70% patients in glycolic acid group and 64% in the TCA group which was statistically insignificant. The commonest side effect after peels was burning which was seen in 26.6% patients after TCA peel, where as in glycolic acid peel burning sensation was seen only in 6.6% patients.

P107

Salicylic acid peels in the treatment of melasma

R. Sarkar, R. K. Jain
FAAD, New Delhi, India

Melasma, a common disorder of hyperpigmentation is rather recalcitrant to therapy. Although salicylic acid peels have been tried for melasma, there is a dearth of studies in dark skinned patients.

Twenty patients of epidermal melasma as ascertained by Wood's light, were inducted into the study. They were primed with broad spectrum sunscreens and 2% hydroquinonone/ 0.025% tretinoin prior to treatment. Six serial 20–30% salicylic acid peels were done two weekly followed by the 2% hydroquinone/0.025% tretinoin in between peels. A subjective assessment was carried by the patient and MASI index and photographic evaluation were done by the investigators both before and after treatment. Subjectively, the result was graded as very good (33–66%) by seven patients and excellent in three patients. There was a decrease in the MASI index after treatment which was found to be significant ($P < 0.05$). Salicylic acid could be an adjuvant in the management of melasma. But is effective only in combination with topical agents.

P108

Utility of glycolic acid peels in melasma and comparison of 55% glycolic and with hydoquinone (4%) cream in melasma

G. Sunil, D. Ghate, S. T. Amladi, H. R. Jerajani
Skin and VD Department, Rajawadi Municipal General Hospital, Ghatkopar, Mumbai, India

Melasma is a commonest type of facial hypermelanosis. It is difficult to treat. Although, Hydroquinones have been in use since decades, the ideal agent still eludes us. Glycolic acid is a recent introduction to our armamentarium. We evaluated various concentrations (30, 40 and 50%) of glycolic acid in melasma and then compared the 55% glycolic acid with hydroquinone (4%) in treatment of melasma. Twenty patients each were treated with glycolic acid peeing (30, 40 and 50%) once in 2 weeks for a period of 3 months. All were advised photoprotection. We then compared 55% glycolic acid on the right side of the face and Hydroquinone (4%) cream applied daily on the left side in 30 patients for a duration of 3 months. The clinical assessment was done with MASI score. The first part of the study to compare various concentrations of glycolic acid concluded that as the concentration of glycolic acid was increased, the response to treatment increased. Following were the observations in the comparative study. The average age was 32 yr. The average duration of pigmentation was 4 yr. There were 27 women and three men. There was similar family history in six patients and nine women gave history of aggravation during pregnancy. Malar type of pigmentation was seen in 22 and nine had cetrofacial type of pigmentation. Epidermal type of pigmentation was seen in 26, three had mixed type while one had dermal type. The MASI score remained comparable on both sides of face at the end of study. Both glycolic acid 55% and hydroquinone (4%) improved melasma by 33%. Hydroquinone side had mild irritation. Thus, to conclude, glycolic acid 55% is not superior to hydroquinone 4%.

P109

Comparative efficacy and safety of mometasone based triple creams versus fluocinolone based triple creams in melasma in Indian patients

K. Godse
Shree Skin Centre, Navi-Mumbai, India

Melasma (from the Greek word, 'melas' meaning black) is a common, acquired, circumscribed hypermelanosis of sun-exposed skin. It presents as symmetric, hyperpigmented macules having irregular, serrated, and geographic borders. The most common locations are the cheeks, upper lips, the chin, and the forehead, but other sun-exposed areas may also occasionally be involved. Melasma occurs in all skin types and in people of all racial and ethnic groups, but is more common in those with darker complexions living in areas of intense UV radiation, such as Latin Americans, Asians and blacks. Melasma is the most common pigmentary disorder among Indians. In India over the counter medications are often tried by patients before approaching a dermatologist. In 2004 a triple combination of hydroquinone (2%) with tretinoin (0.025%) and mometasone furoate (0.1%) was launched in India. This combination has a potent steroid mometasone furoate (0.1%) which induces local side effects like thinning of skin, persistent erythema and telangiectasia within few weeks of application. Patient keeps on applying this combination as it can be purchased without prescription in India. Now triple combination cream with fluocinolone acetonide (0.01%) as steroid is available in India. We tried these two combinations in 20 Indian patients (age group 18–40 yr, mean age 23.4 yr) with mild to moderate melasma with glycolic acid peels and sunscreens. First group of 10 patients (eight females and two males) were advised to use mometasone based triple creams along with physical sunscreen zinc oxide. Second group of 10 patients (seven females and three males) were advised to use fluocinolone based triple cream in the night along with sunscreen in the morning and glycolic acid peels. Those with a history of recurrent herpes, and nursing and pregnant women were not included in the study. All the patients were informed about the procedure and their consent was taken. All patients with melasma were put on a maintenance regimen of triple combination to be applied at night. The patients were instructed to apply medication all over the face. In the morning, physical

 © 2011 The Authors. Journal Compilation © 2011 John Wiley & Sons A/S

sunscreen of SPF 19 (Micronized zinc oxide 15%) was used. Serial Glycolic acid peels were used at three weekly intervals, for 2–6 min, depending on tolerance and erythema. Glycolic acid of 57% with free acid of 55% and pH of 2.3 was used in gradually increasing duration of application on the face.

P110

The ratio of lesional/non-lesional melanin index; a sensitive parameter for the evaluation of skin lightening agents

J. W. Shin, S. Y. Choi, K. C. Park
Department of Dermatology, Seoul National University Bundang Hospital, Seongnam-si, Korea

The efficacy of cosmetic formulations is usually minimal and it may therefore be difficult to determine whether these formulations are really effective for pigmented skin lesions. Especially in the season of increasing sunlight, evaluation is more difficult as general skin tone becomes dark. In the present study, we tried to evaluate the efficacy of skin lightening products mainly composed of tranexamic acid by calculating the ratio of lesional/non-lesional melanin index (MI)

A total 60 Korean females with melasma were enrolled in the present study which was performed for 8 weeks, from March through July, season of increasing sunlight. The subjects were randomly divided into two groups (combination group: patients who used skin lightening cosmetics with oral medication, topical group: patients who used skin lightening cosmetics with placebo). Lesional and non-lesional MI were measured and the ratio of lesional/non-lesional MI was calculated. In addition, we reviewed the results of other clinical trials which had been conducted in our clinic previously. When changes of lesional MI were analyzed, combination group only showed significant decrease compared to topical group. Non-lesional MI increased in both groups. Thus, the ratio of lesional/non-lesional MI significantly decreased in both groups. However, combination group showed more significant results compared to topical group. Analysis of previous studies also showed similar results. The ratio of lesional/non-lesional MI could be a simple and reliable indicator for evaluating efficacy of skin lightening cosmetics.

P111

Inhibition of Mitf-E box binding and its effect on pigmentation in melan-a cells

M. Son, D. Jung, W.-Y. Choi, E. Kim
Biological Engineering, Inha University, Inchon, Korea

Microphthalmia associated transcription factor (Mitf) is a key regulatory transcriptional factor of pigmentation-related genes including tyrosinase. Inhibition of tyrosinase transcription by blocking the binding of Mitf with its promoter E-box DNA can control the pigmentation. However, no such chemicals were reported so far. To discover and evaluate the small molecule inhibitors of Mitf-E-box DNA, candidate chemicals were screened by virtual screening from pharmacophore data followed by Mitf E-box DNA protein chip. After selecting the chemical, its inhibitory activity on binding interaction between Mitf and E-box DNA, electrophoretic mobility shift assay (EMSA) was performed. To evaluate the depigmenting activity of Compound no. 17, cellular melanin assay, and Western blot were performed in melan-a cells. Among 27 chemicals selected from a pharmacophore data by virtual screening, Compound no. 17 was screened which showed the most potent inhibitory activity against Mitf-E-box DNA binding in protein chip. EMSA results confirmed the specific inhibition of Compound no. 17 on Mitf-E-box DNA binding. In melan-a cells, Compound no. 17 reduced tyrosinase expression and melanin synthesis (62.5% with 25 μM). The results show that Compound no. 17 is the first small molecule inhibitor of Mitf-E-box DNA binding with depigmenting activity.

P112

Effects of a low-molecular-weight polyphenol (oligonol) on the growth and melanogenesis of primary melanocytes and melanoma cells

M. Okura, K. Hagiwara, T. Hida, A. Yoneda, K. Yanagisawa, Y. Horio, T. Yamashita
Sapporo Medical University School of Medicine, Sapporo, Japan

Oligonol is a low-molecular-weight polyphenol prepared and purified from lychee fruit (Amino Up Chemical Co., Ltd., Japan), which has been reported to exhibit various biological effects similar to polyphenol. To elucidate its biochemical effects on the melanogenesis, we studied its activities on the oxidative stress, tyrosinase activity and melanin production in cultured human primary melanocytes and melanoma cells. To examine the anti-oxidative stress of oligonol, DCF fluorescent assay was carried out. Similar to resveratrol, oligonol suppressed antimycin A-mediated ROS (reactive oxygen species) generation in melanocytes and SK-mel-24 melanoma cells. However, different from resveratrol, suppression of ROS generation by oligonol was not affected by nicotinamide, an inhibitor of SIRT1. Both the DOPA activity of cell-free extract prepared from oligonol-treated melanocytes and in vitro dopa oxidase activity of mushroom tyrosinase were decreased in the presence of oligonol in a dose-dependent manner. Amounts of melanin were also reduced in the cell extract cultured in the oligonol-containing medium. Finally, Western blot analysis revealed that oligonol, as well as resveratrol, down-regulates tyrosinase in melanocytes and tyrosinase-expressing SK-mel-24 cells. These results suggest that oligonol suppresses ROS-mediated cytotoxicity by a mechanism different from resveratrol, and possibly exerts a skin-lightening effect through suppression of melanogenesis.

P113

Depigmentation by keratinocyte-derived, Wnt inhibitor sFRP2

M. Y. Kim, J. H. Lee, J. Yoon, K. H. Kim, J. S. Hwang, J.-H. Lee, T.-J. Yoon
Department of Dermatology, Gyeongsang National University Hospital, Jinju, Korea

A link between Wnt/β-catenin signaling and melanocyte differentiation has been revealed by the finding that β-catenin forms a complex with lymphocyte enhancer factor-1 to up-regulate expression of the MITF gene. Also, β-catenin directly interacts with the MITF protein itself and then activates MITF-specific target genes. Conversely, the inhibitor for Wnt/β-catenin such as DKK1 is known to inhibit melanocyte growth and function. In this study, we found that another Wnt/β-catenin inhibitor sFRP2 was increased in epidermis of palmoplantar skin. To investigate the effect of sFRP2, we set the co-culture model in which HaCaT keratinocytes and melanocytes were incubated using Boyden's chamber. After transduction of HaCaT with the recombinant adenovirus expressing sFRP2, cells were loaded into upper chamber, and melanocytes were seeded in lower chamber.

Results: Interestinlgy, the β-catenin level was significantly decreased in melanocytes by co-culture with sFRP2 overexpressing HaCaT cells. In addition, the protein levels for pigmentation-related molecules including MITF and TYR were also down-regulated by co-culture with sFRP2 overexpressing HaCaT. These results suggest that increase of sFRP2 in epidermal keratinocytes of palmoplantar skin comparing to trunk (or non-

© 2011 The Authors. Journal Compilation © 2011 John Wiley & Sons A/S

palmoplantar) one may decrease the melanogenic potential of melanocytes, contributing the depigmentation and relate clinically with the whitening of the palmoplantar areas.

P114

Sphingosylphosphorylcholine modulates melanin synthesis via various signal transduction pathways

H.-S. Jeong, S. Y. Kim, H. Li, H.-Y. Yun, K. J. Baek, N. S. Kwon, K.-C. Park, D.-S. Kim
Department of Biochemistry, Chung-Ang University College of Medicine, Seoul, Korea

Sphingosylphosphorylcholine (SPC) acts as a potent lipid mediator and signaling molecule in various cell types. In the present study, we investigated the effects of SPC on melanogenesis and SPC-modulated signaling pathways related to melanin synthesis. Melanin production was measured in Mel-Ab cells. A luciferase assay was used to detect transcriptional activity of the MITF promoter. To examine SPC-induced signaling pathways, Western blot analysis was performed. SPC had significant hypopigmentation effects in a dose-dependent manner. It was found that SPC induced not only activation of Akt but also stimulation of mTOR, a downstream mediator of the Akt signaling pathway. Moreover, SPC decreased the levels of LC3 II which is known to be regulated by mTOR. Treatment with the mTOR inhibitor rapamycin abolished decreases in melanin and LC3II levels by SPC. Furthermore, we found that the Akt inhibitor LY294002 restored SPC-mediated downregulation of LC3 II and inhibited the activation of mTOR by SPC. Taken together, our data suggest that the mTOR signaling pathway is involved in SPC-modulated melanin synthesis.

P115

Arenarol isolated from marine sponge abrogates endothelin-1-stimulated expression of tyrosinase by interrupting intracellular MAPK signaling pathway in normal human melanocyte

B. Choi, O. Makoto, A. Kanamoto, T. Fujiwara, H. Nakajima, G. Imokawa
Nagoya University, Kasugaisi, Japan

Endothelin-1 (EDN1)–stimulated MAPK cascade in normal human melanocytes (NHM) is a major intracellular signaling pathway leading to up-regulated expression of tyrosinase in UVB-melanosis and lentigo senilis. Therefore, it is of interest to identify chemicals capable of attenuating the EDN1-stimulated MAPK linkage, which results in abolishing up-regulated expression of tyrosinase. In the search of such anti-pigmenting agents, we used marine sponge collected around Okinawan islands to purify 'arenarol' as an active chemical by partition methods combined with reverse-phase HPLC. When NHM were cultured for 3 h in the presence of arenarol at concentrations ranging from 0.3 to 3 μM and then stimulated with EDN1 (10 nM), a marked increase at the activity and protein levels of tyrosinase was markedly abrogated at 24–72 h post-EDN1 stimulation up to the non-stimulation level, which was not accompanied by any cytotoxic effect evaluated by MTT assay. Signaling analysis showed that the EDN-1-stimulated phosphorylation of MEK, ERK, MITF and CREB were distinctly abolished at 15 min post-EDN1 stimulation by arenarol at 3 μM. These findings indicate that arenarol attenuates EDN1-stimulated tyrosinase expression via interruption of MAPK pathway in NHM.

P116

Inhibitory effects of NAG on pigmentation

J. S. Hwang[1,2,3], H. Y. Lee[1,2,3], T.-Y. Lim[1,2,3], T.-J. Yoon[1,2,3], K-Y Nam[1,2,3]
[1]Department of Genetic Engineering, Kyung Hee University, Yongin;[2]Department of Dermatology and Institute of Health Sciences, School of Medicine, Gyeongsang National University, Jinju;[3]Bioinformatics and Molecular Design Research Center Yonsei Engineering Research Complex, Yonsei Univ. Seoul, Korea

Tyrosinase, an important enzyme for melanin production, is synthesized and glycosylated in the endoplasmic reticulum (ER) and Golgi. The enzyme is subsequently transported to melanosome where it participates in melanogenesis. N-acetyl-glucosamine (NAG) inhibits the glycosylation of tyrosinase accounting for its pigment-reducing abilities Previous studies have shown that NAG effectively reduces the appearance of hyperpigmented spots and the production of melanin in skin equivalent cultures. In this study, we demonstrated that NAG decreases the pigmentation in human melanoma cell, brown guinea pig and human skin. NAG inhibits the α-glucosidase activity with dose-dependent manner and disrupts tyrosinase glycosylation in HM3KO human melanoma cell, showing similar results with well-known glycosylation inhibitor, deoxynojirimycin (DNJ). The color of cell pellet and melanin contents were also reduced by various concentrations of NAG and the tyrosinase activity was slightly inhibited in HM3KO melanoma cell. To investigate the in vivo effects of NAG, we applied topically the NAG solution (5% in propylene glycol:ethanol:water = 5:3:2) twice daily for 4 weeks in the dorsal skin of brown guinea pigs and for 8 weeks in the forearm of humans tanned by UV irradiation. As expected, the lightening effects were observed in the skin of both brown guinea pigs and humans in terms of L value and histological analysis.

P117

Paracrine interaction interaction between UVB-exposed human keratinocytes and human melanocytes leading to an increased expression of tyrosinase and its blockade by Wetherferin A

T. Niwano, H. Nakajima, Y. Wakabayashi, G. Imokawa
Tokyo University of Technology, Tokyo, Japan

In this study, using membrane-separated co-culture system with cell culture insert, we examined paracrine interaction between UVB-exposed human epidermal keratinocytes (HEK) (in insert) and normal human melanocytes (NHM) (in well plate) leading to an increased expression of tyrosinase. The exposure of cultured HEK to UVB radiation significantly stimulated in a dose-dependent manner the activity of tyrosinase at 72 h post-irradiation in cultured NHM. Since endothelin-1 (EDN1) is well known to be keratinocyte-derived and membrane-permeable soluble melanogenic cytokine, we examined the effects of neutralizing antibodies to EDN1 on the up-regulated activity of tyrosinase. The addition of anti-EDN1 significantly abolished the increased activity of tyrosinase at 24 h but not at 0 and 12 h post-irradiation addition timing, probably reflecting onset of EDN1 secretion around 24 h post-irradiation. We used Whetherferin A (WFA) to examine the blocking effect on the paracrine interaction leading to up-regulation of tyrosinase activity. The addition of WFA (12.5–50 μM) at 0 h post-irradiation to the co-culture system significantly abolished the UVB-induced up-regulation of tyrosinase activity after 72 h of culture. Signaling analysis using ALM melanoma cells revealed that WFA (at 12.5, 25 or 50 μM) significantly abolished EDN1-stimulated phosphorylation of Raf-1, MEK, ERK, MITF and CREB although intracellular calcium mobilization by EDN1 stimulation in NHM was not interrupted by

 © 2011 The Authors. Journal Compilation © 2011 John Wiley & Sons A/S

WFA (50 μM). Taken together, these findings suggest that the co-culture system is a useful tool for selecting blockers for the melanogenic paracrine interaction and it is anticipated that WFA is a potent signaling-interruptible anti-melanogenic agent.

P118

Glucosamine, an asparagin-linked carbohydrate core synthesis inhibitor attenuates endothelin-1 + stem cell factor-stimulated expression of melanocyte-specific proteins by down-regulating CREB activation in human melanocytes

T. Kato, H. Nakajima, Y. Wakabayashi, G. Imokawa
Tokyo University of Technology, Tokyo, Japan

Although an asparagin-linked carbohydrate core synthesis inhibitor glucosamine (GlcN) is implicated to interrupt tyrosinase (TYR) trafficking to melanosomes, resulting in yielding amelanotic B-16 cells, it remains unclarified as to whether they are also effective in preventing stimulated melanogenesis in normal human melanocytes (NHM). In this study, we evaluated effects of GlcN on endothelin-1 (EDN1) + stem cell factor (SCF) stimulated melanogenesis in NHM and analyzed its biological mechanisms. When NHM were cultured for 24–72 h in the presence of GlcN (1 mg/ml) and then treated with EDN1 (10 nM) + SCF (5 nM), there was significant reduction in EDN1 + SCF stimulated expression of melanocyte-specific proteins (MSP) including tyrosinase compared with non-GlcN treated NHM. The decreased expression of MSP was accompanied by a significantly down-regulated gene and protein expression of MITF, suggesting an impairment in intracellular signaling upstream of the gene expression of MITF. Western blotting for intracellular signaling revealed that in the GlcN-treated NHM, there is a marked deficiency in ET-1 + SCF stimulated phosphorylation of CREB due to decreased level of CREB protein but not of ERK1/2 compared with the control non-treated NHM. These findings indicate that GlcN attenuates ET-1 + SCF-stimulated melanogenesis by down-regulating preferentially CREB activation in NHM. These findings indicate that the carbohydrate synthesis inhibitor has a distinct capability to inhibit the stimulated pigmentation in the epidermis equivalent. Since the depigmenting effect was accompanied by significantly suppressed expression of melanogenic genes and proteins, it is likely that the depigmentation is mediated not by interrupted tyrosinase trafficking to melanosomes within melanocytes, but possibly by dysfunction of ET-1/SCF receptors with carbohydrate moieties, ETBR or cKIT, leading to suppressed expression of tyrosinase or other melanogenic molecules.

P119

Influence of estrogens on melanosome distribution in keratinocytes: an ultrastructural study on irradiated skin organ culture

M. Cario-André, Y. Gauthier, S. Lepreux, C. Pain, A. Taïeb
INSERM U1035, Department of Dermatology, CHU de Bordeaux, Bordeaux, France

Melasma occurs mainly in women during pregnancy, taking oral contaceptives or hormone replacement therapy. By histological examinations, epidermal melanin deposition is found in all skin layers including horny layer, sometimes associated with dermal melanin deposition. Konrad and Wolff (1973) have previously observed in melasma skin samples by electronic microscopy a non-aggregated distribution of melanosomes in keratinocytes. The mechanisms of action of estrogen and progesterone in human cutaneous pigmentation are largely unknown. Despite the presence of estrogen and progesterone receptors in human skin, physiological and pregnant levels of estrogen and progesterone do not induce in vitro significant effects on proliferation of human melanocytes and on tyrosinase activity. We have previously treated pigmented epidermal reconstructs with estrogen without detecting any change in pigmentation and melanosome distribution. To study with electron microscopy the effects of estrogens on melanosome distribution in irradiated skin organ culture we have treated phototype III skin samples with 2, 10 and 100 nM of 17 b-estradiol for 3 days before a single irradiation (UVB 50 mJ/cm^2). Skin samples were fixed for optical and electronic microscopy 3 days after irradiation to allow stimulation of melanogenesis. By Fontana–Masson staining, we could not detect visible changes in pigmentation in our irradiated, treated samples as compared to irradiated controls. However, our preliminary data suggested that the addition of 10 mM 17 b-estradiol in culture medium prior to UVB irradiation induced a modification of melanosome distribution with non aggregated melanosomes within keratinocytes. We have reproduced with our experimental model of irradiated skin organ culture pretreated with estrogen the same non aggregated distribution of melanosomes reported in melasma skin samples. The combination UV and estrogen may be implicated in the modifications of melanosome distribution observed in melasma explaining why melasma is observed in photoexposed skin.

P120

USF1 modulates in vivo skin cell proliferation arrest and DNA damage repair in response to UVB

A. Bouafia, S. Corre, N. Mouchet, M. D. Galibert
CNRS-UMR6061, Institut de Génétique et Dévelopement, Universite de Rennes 1, Equipe 'Gene Expression and Oncogenesis' Rennes, France

UV radiation is the major risk factor in developing melanoma and non-melanoma skin cancers. Skin cells normally prevent genetic mutations caused by UV induced DNA lesions by employing a DNA repair machinery coupled to cell cycle control mechanisms. An early response to UV irradiation, oxidative stress and bacteriological insults is the activation of the stress signaling p38 MAPK pathway. Once the p38α is active, the ubiquitously expressed transcription factor Upstream Stimulating Factor 1 (USF1) is phosphorylated by p38α kinase and acts as a stress sensor to modulate UV dependent melanogenesis (Galibert et al., 2001) and genes implicated in melanocyte survival (Corre et al., 2009). Importantly, USF1 has previously been described to regulate transcription of cell cycle regulators and genes involved in DNA repair (Davis et al., 1999, Goueli and Janknecht, 2003; Reisman and Rotter, 1993). Currently, there are no in vivo data implicating USF1 in cell cycle regulation or in the DNA damage of skin tissue irradiated by UV. To determine the involvement of USF1 in the response of skin tissue to UVB, we tested whether the skin of USF1 KO mice was still able to modulate epidermal proliferation and to repair DNA lesions after UVB irradiation. Briefly, we irradiated the dorsal skin of KO mice with UVB both in vivo and in an organotypic culture system and performed histological, protein and transcriptomic analysis. Staining of histological skin sections with an antibody to the proliferation marker KI-67 and western blot analysis show that knocking out USF1 inhibited a normally occurring proliferation arrest. Additionally, ELISA analysis demonstrated that USF1 KO mice are significantly less efficient at removing cyclo-pyrimidine dimers lesions. Comparative transcriptomic analysis confirmed the deregulation of genes crucial for cell proliferation and the DNA damage response in the KO mice exposed to UVB. Interestingly, USF1 target genes identified in this study have also been shown to play a role in the response to solar simulated radiation in humans as previously described by our group (Mouchet et al, 2010). This raises the exciting possibility that USF1 could be

© 2011 The Authors. Journal Compilation © 2011 John Wiley & Sons A/S

responsible for regulating these genes in both mice and human skin in response to UV. Our results suggest that USF1 plays a critical role in skin UVB response by modulating the expression of cell proliferation and DNA damage repair genes needed to prevent the onset of skin cancer.

P121

Protective effects of BSP-2 on UVB-induced senescence in human keratinocytes

J. Lee, K.-B. Roh, J. Lee, D. Park
Skin Disease Research Team, Biospectrum Life Science Institute (BLSI), Seongnam-Si, Gyeonggi-Do, Korea

Ultraviolet (UV) irradiation is a major inducer of DNA damage in the epidermis and is the most important environmental mutagen and carcinogen of epidermal cells. To cope with the frequent exposure to carcinogenic UVB wavelengths found in sunlight, keratinocytes have acquired extensive protective measures to handle UVB-induced DNA damage. Ataxia telangiectasia mutated (ATM) plays a critical role in the cellular response to DNA damage. Upon activation, ATM phosphorylates a number of substrates including targets that initiate cell cycle arrest, DNA repair, and apoptosis. We investigated here the protective mechanism of BSP-2 (derivatives of glutaric acid) against UVB-induced DNA damage in human keratinocytes. To understand the biological consequence of UVB-irradiated skin, we have used an in vitro model system to probe the response of human keratinocytes to UVB exposure (30 mJ/cm^2). At the indicated times after UVB irradiation, keratinocytes were harvested and assayed for the phosphorylation of ATM (pS1981), Chk2, p53 (pS15), p21, H2A.X, and MAPK via immunoblot analysis and analyzed the amount of FITC fluorescence of ATM and senescence-associated beta-galactosidase (SA-b-gal) via FACS analysis. Also, TNF-α, IL-1b, and IL-6 levels in the supernatant of keratinocytes were measured by ELISA assay. ATM-dependent signaling pathways (ATM, Chk2, p53, and H2A.X), MAPKs (SAPK/JNK and p38), PI3K (AKT1) and ribosomal protein S6 (S6) were rapidly phosphorylated and pro-inflammatory cytokines such as TNF-α, IL-1b, and IL-6 were significantly released in response to UVB irradiation in keratinocytes. Also, SA-b-gal accumulation was observed in keratinocytes at the indicated times after UVB irradiation. We found that BSP-2 meaningfully decreased UVB-induced phosphorylation of ATM, Chk2, p53, JNK, p38, and S6. Our results indicate that BSP-2 suppresses the UVB effects by regulating UVB-induced ATM activation in human keratinocytes. Therefore, our results suggest that BSP-2 may be introduced as a putative agent to suppress UVB-induced senescence in epidermal cells.

P122

Repeated ultraviolet exposure induces TLR4 expression of neonatal human melanocytes

H. Song, H. Kim, G. Choi, J. Shin
Department of Dermatology, College of Medicine, Inha University, Incheon, Korea

Toll-like receptors (TLRs) play a critical role in cutaneous innate immunity. In invertebrates, a major aspect of the innate immune defense system against invading pathogens involves melanin. Recent studies showed that human melanocytes expressed TLR1, 2, 3, 4, 7 and 9, though their function is not yet established. TLR4 is thought to regulate UV-induced cutaneous immunosuppression. Studies have reported that in Langerhans cells, TLR4 is upregulated upon exposure to ultraviolet (UV) light. However, this phenomenon has not been studied in melanocytes. In the present study, we examine the effect of single or repeated UV exposure on TLR4 expression in human melanocytes and its relationship to pigmentation. Primary cultured neonatal human melanocytes were exposed to either a single dose of UVA (3 J/cm^2) or UVB (300 mJ/cm^2), or daily low dose UVA (1 J/cm^2) or UVB (100 mJ/cm^2) for five consecutive days. TLR4 expression was evaluated by flow cytometry and reverse transcription–polymerase chain reaction (RT-PCR), and pigmentation was determined by melanin content assay. We found that TLR4 was not expressed in the resting state of neonatal human melanocytes. LPS stimulation increased both TLR4 and melanin content. A single dose of either UVA or UVB increased melanin content only. However, repeated exposure to either UVA or UVB increased both TLR4 and melanin content. The level of TLR4 expression from UV was lower than that of LPS. Our results suggest TLR4 is not expressed in resting neonatal human melanocytes, and its levels can be upregulated by repeated exposure to UV light. These findings differ from previous studies in that TLR4 is expressed in basal state adult human melanocytes. This study suggests human melanocytes may participate actively in UV-induced immune modulation, and UV-induced melanogenesis is not dependent on TLR4 expression.

P123

Antioxydant activity of seaweed biopigments and the potency for human skin protector

W. Merdekawati, A. B. Susanto
Brawijaya University Solo, Indonesia

Sargassum sp. is a species of brown seaweed which is potential as a nutritional and medical sources. It has a variety of pigment composition. *Sargassum* sp. has been known for its highest antioxidant activity among the other brown seaweed species. The aim of this research is to determine the pigment composition of *Sargassum* sp., the antioxidant activity of chlorophyll a, beta-carotene and crude extract of *Sargassum* sp. and also to explore its potency for human skin protection. Pigment identification was conducted by Thin Layer Chromatography (TLC), Spectrophotometer UV-V is and High Performance Liquid Chromatography (HPLC). The contents were conducted by HPLC with photodiode-array detector (PDA). The antioxidant activity of chlorophyll a and beta-carotene were measured by diphenylpicryl-hydrazil (DPPH) method. Results show that *Sargassum* sp. consist of chlorophyll a, chlorophyll c, derivatives of chlorophyll, βbeta-carotene, fucoxantin, and xantophyll. The contents of each pigment is 52.82; 1.05; 15.23; 1.49; 20.95 and 8.46%. Antioxidant activity based on IC50 value, resulted 350.63 ± 0.03 ppm of chlorophyll a, 351.64 ± 0.05 ppm of beta-carotene, and 888.41 ± 0.08 ppm of crude extract of *Sargassum* sp. Research result showed that the chlorophyll a in *Sargassum* sp. has a strong potential as antioxidant. Carotenoids and chlorophyll also contribute via several mechanisms to protect skin from UV radiation.

P124

α-Melanocyte stimulating hormone enhances nucleotide excision repair in human melanocytes by activating the transcription factor ATF2

A. Von Koschembahr, R. Starner, J. Jameson, V. Swope, Z. Abdel-Malek
Department of Dermatology, University of Cincinnati, Cincinnati, OH, USA

The human melanocortin 1 receptor (MC1R) is a major determinant of human pigmentation and is a melanoma susceptibility gene. Binding of α-melanocyte stimulating hormone (α-MSH) to the MC1R expressed on cultured human melanocytes (HM) enhances nucleotide excision repair (NER) and reduces oxidative DNA damage. These effects are expected to inhibit the transformation of HM to malignant melanoma. ATF-2 is a transcription factor that is activated by cellular stressors, such as UV, and upregulates

 © 2011 The Authors. Journal Compilation © 2011 John Wiley & Sons A/S

expression of genes involved in NER, including XPC, XPA and ERCC1. To investigate the hypothesis that α-MSH enhances NER in HM expressing functional MC1R by activation of the MAP kinase p38, the downstream transcription factor ATF-2 and upregulation of the ATF-2 targets XPA, XPC, ERCC1. HM were irradiated with UV ± 1 or 10 nM α-MSH. Western blotting was performed to detect phospho- and total p38, phospho- and total ATF-2, XPC, XPA, and ERCC1. Immunofluorescent staining was carried out to determine the localization of XPC, XPA, and ERCC1. UV resulted in rapid phosphorylation of p38, and ATF-2 within 15 min, and α-MSH augmented these effects but only in HM expressing functional MC1R. Exposure to UV resulted in reduction, while α-MSH increased the protein levels of XPC within 8 h. No change was observed in the expression of either XPA or ERCC1. XPC was localized in the nucleus, while XPA localized in the cytoplasm and nucleus. α-MSH enhances NER by further activating p38, ATF-2 and increasing the expression of XPC in UV-irradiated HM.

P125

Inhibition of eotaxin-1/CCL11 expression by novel compound in mouse embryonic fibroblast

K.-B. Roh, J. Lee, J. Lee, D. Park
Biospectrum Life Science Institute Seongnam, Korea

The chemokines are a large family of small proteins involved in the activation and recruitment of specific cell populations during the course of disease. CCL11, a CC chemokine, is a potent chemoattractant and an activator of eosinophils, basophils, and Th2 lymphocytes. CCL11 expression was found to be restricted to a few cell types, including eosinophils, bronchial epithelial cells, and dermal fibroblast cells. In asthmatics, the expression of CCL11 has been found to be enhanced in these types of cells, and increased expression is associated with disease severity. Additionally, CCL11 expression in epithelial cells was found to be increased in atopic dermatitis, as well as in other inflammatory conditions. This study was aimed to investigate effects of BSP-3 on expression of CCL11 induced by TNF-α and its action mechanisms in fibroblasts. In order to determine the effects of BSP-3 on TNF-α -induced expression of CCL11, we performed enzyme-linked immunosorbent assay and quantitative RT-PCR for CCL11 and a western blot assay for activation of MAPKs. The transient luciferase reporter was used to examine activation of transcription factors. In our NF-kB luciferase reporter system, TNF-alpha-induced NF-kB activation was observed to be reduced by BSP-3 (a dicarboxylic acid). In accordance with this result, BSP-3 also inhibited TNF-alpha-induced phosphorylation and degradation of IkB-a as well as nuclear translocation of NF-kB heterodimer induced by TNF-alpha. This suggests that BSP-3 downregulates the expression of CCL11 via the inhibition of NF-kB activation signaling. Using the NF-kB luciferase reporter system, Western blot analysis, and IKK-beta activity assay, we determined that BSP-3 inhibits IKK-beta activity in NF-kB signaling, which upregulates the expression of CCL11. Our results show that BSP-3 inhibits the expression of CCL11 by suppressing the IKK-beta activity in NF-kB activation signaling.

P126

Treatment of giant congenital melanocytic nevus: pediatric skin expansion in infants

M. Loot, P. Vergnes, A. Taïeb
Departments of Pediatric Surgery and Pediatric Dermatology, Bordeaux, France

Complete excision of the involved skin is the treatment of choice of giant congenital melanocytic nevi. Among surgical techniques, skin expansion provides a good quality replacement of the skin defect. For the last 23 yr we have used this technique in the first months of life with overall good results. We retrospectively included 52 consecutive patients who underwent tissue expansion before the age of one. Clinical follow-up by dermatologist and surgeon allowed to record early and late complications, and to assess aesthetic results. Thirty-six infants underwent a single procedure and 16 had repeated skin expansion. The median age of first treatment was 5 months. The early complication rate was 25% at any stage of treatment. On average 95% of the nevus could be removed on the head and 90% on the trunk. In 15 children, long term assessment revealed later growth-related complications like widening of scars and morphological deformities. Early treatment of giant nevus improves social and aesthetic prognostic. The complication rate of skin expansion in infants is comparable to that of older children. This technique allows an excellent excision rate, even for giant nevi of the trunk. However, one must consider the possibility of late growth-related complications. Overall, considering the poor results of other treatments, we consider early skin expansion before the age of 1 yr as the best option for giant melanocytic nevi.

P127

Vitamin D levels and ultraviolet radiation exposure; upon what basis do we increase melanoma risk?

S. Rice, A. Fityan, M. Carpenter, L. Vearncomb, J. Baird, E. Healy
University of Southampton, Southampton, UK

The World Health Organisation estimates that 200 000 cases of cutaneous melanoma and 65 000 melanoma-associated deaths occur each year worldwide. Although exposure to ultraviolet radiation (UVR) is the primary cause of melanoma, public health guidelines frequently encourage UVR exposure for maintenance of serum vitamin D levels. However, despite studies documenting that exposure to UVR increases vitamin D concentrations, extrapolating from these to suggest that UVR is necessary for maintenance of adequate vitamin D levels is flawed. In addition, it has recently been reported that 10–20 min of sun exposure (often promoted in public health statements) is inadequate to increase serum vitamin D and that sufficient sun exposure to generate meaningful benefit would compromise skin health. Unfortunately, 'experts' advocating UVR exposure frequently do not provide the original research study evidence upon which they base their views, therefore, to address whether UVR exposure is necessary for maintenance of adequate vitamin D levels, we undertook a detailed systematic review according to the Centre for Reviews and Dissemination's guidance and standards. Twenty-one literature databases (from 1948 to present day) were searched for original studies using synonyms of vitamin D, diet, supplementation and UVR. A total of 40 150 articles which met the inclusion criteria were screened by two independent reviewers, resulting in 356 interventional and observational studies which reported on effects of UVR on serum vitamin D levels. Extraction of data from these studies identified 53 data sets from 41 cohorts which provided information relevant to UVR exposure and adequate vitamin D levels. In 12 of 25 data sets which recorded winter levels of vitamin D in areas of limited UVR exposure, over 50% of individuals had adequate levels of vitamin D. These results demonstrate that many subjects can maintain adequate vitamin D levels despite negligible UVR exposure, and suggest that policies advocating UVR exposure for vitamin D production are misplaced and inappropriate.

© 2011 The Authors. Journal Compilation © 2011 John Wiley & Sons A/S

P128

Genetic variation in zebrafish melanoma

A. Capper
MRC Human Genetics Unit, Edinburgh, UK

Melanoma is the most lethal form of skin cancer and with its incidence rising rapidly it is important to determine the role of genetic components of this disease and develop specific and effective therapeutics. The recent discovery of recurrent mutations in the melanoma genome has provided greater understanding of how melanomas develop and progress, as well as giving an opportunity for the development of new targeted drugs. The most common mutation identified in melanoma is in the BRAF proto-oncogene, which is found in 50–60% of malignant melanomas. Targeted therapies including specific BRAF inhibitors have been developed in light of this discovery, but as yet there is no effective single-agent drug to treat metastatic melanoma. It is known that other mutations, such as p53 and PTEN, co-operate with BRAF mutations in progression of melanoma from the initial benign nevus stage. If future therapies are to be effective, drug development must take these co-operating mutations into account to improve current treatment of melanoma patients. Our group has generated a model of melanoma in zebrafish that is based on the most frequently mutated gene in melanoma, BRAF. We are using this model to identify new melanoma genes and investigate the role of these genes in melanoma initiation and progression. The aim of my project is to identify the genetic differences between zebrafish melanomas with varying driver mutations; BRAFV600E with p53, PTEN or MITF. I am carrying out both exome and transcriptome sequencing of these melanomas in the hope that it will provide a novel insight into the genetics of melanoma formation and may identify new genes that affect melanoma progression.

P129

GSTs genes and genetic susceptibility to melanoma

M. Ibarrola-Villava, M. Peña-Chilet, M. Mayor, C. Gomez-Fernandez, B. Casado, M. Martin-Gonzalez, A. Lluch, G. Ribas
Fundacion Investigacion Hospital Clinico Valencia-INCLIVA, Valencia, Spain

The aetiology of melanoma is complex and likely involves multiple low penetrance susceptibility genes, interactions among these genes, the influences of environmental exposures and the interaction of genotypes and environments. The melanocortin-1 receptor (MC1R) low penetrance gene is responsible for constitutive pigment variation in humans and also appears as a risk factor for melanoma. Moreover, the key environmental risk factor is exposure to the ultraviolet (UV) component in sunlight, which causes various kinds of DNA damage, including bulky lesions and oxidative damage. Glutathione S-Transferases (GSTs) are a family of multifunctional enzymes involved in detoxification of reactive species produced during melanin synthesis and are important players in the cellular defence systems against oxidative stress, including the oxidative stress caused by UV radiation. Therefore, GSTs, either alone or in several combinations between them and with MC1R may play a role in protection against development of melanoma. GSTM1 and GSTT1 have copy number polymorphisms represented by gene delections. Genotypes were established by quantitative RT-PCR. GSTP1 (Ile105Val and Ala114Val) polymorphisms were genotyped using TaqMan. MC1R was analysed by sequencing. We present a case–control study including 563 Spanish MM patients and 337 control subjects. We examined the effect of GSTs genotypes on melanoma risk in presence of MC1R gene variants, sun exposure and host-related phenotypes. Associations with melanoma and pigmentary characteristics such as hair, skin and eye colour will be discussed. These results will confirm the contribution of GSTs to genetic susceptibility to melanoma in Spain.

P130

Role of GC transporter and VitD receptor genes on melanoma susceptibility

M. Peña-Chilet, M. Ibarrola-Villava, M. Martin-Gonzalez, C. Gomez-Fernadez, B. Casado, M. Mayor, A. Lluch, G. Ribas
Fundacion Investigacion Hospital Clinico Valencia-INCLIVA, Valencia, Spain

Currently, vitamin D (VitD) has been recognized as a prohormone with multiple functions to maintain optimal health. Vitamin D3, also known as cholecalciferol, is synthesized in skin exposed to sunlight. Cholecalciferol is converted in the liver in 25-hydroxyvitamin D, which is the most abundant in serum. Several studies have detected insufficient levels of VitD widely in apparently healthy populations worldwide. In addition to its known role in maintaining bone homeostasis, VitD sufficiency, along with controlled solar exposure, has been linked to lower risk of many cancers, including melanoma and various complex diseases. Polymorphisms on this pathway gene, cholecalciferol transporter gene GC and VitD receptor gene VDR might modulate VitD levels as well as melanoma risk. We present a case–control study in Spanish population including 519 melanoma patients and 330 control subjects frequency-matched for sex and age. Phenotypic information was collected using a standardised questionnaire. All studied subjects gave informed consent. Functional (from coding and regulatory regions) SNPs with MAF <0.5 were selected using HapMap database. Eleven SNPs in the GC gene and nine in the VDR gene, belonging to the VitD synthesis pathway were finally selected. The genetic variability was analyzed by Kaspar and Taqman technology. Genotyping of the GC and VDR genes, LD and haplotype studies will be performed. Associations with melanoma and pigmentary characteristics such as hair, skin and eye colour will be discussed. These results will confirm the melanoma susceptibility role of the GC and VDR genes in Spanish population.

P131

Detection of CNVs throughout the genome of a porcine melanoma model

J. Corominas, J. Estellé, Y. Ramayo-Caldas, M. Lathrop, F. Demenais, C. Rogel-Gaillard, S. Vincent-Naulleau, J.-M. Folch, E. Bourneuf
CEA, Jouy-en-Josas, France

Structural variation of genomes as copy number variants (CNV) or insertion-deletions of several kilobases is a subject of growing interest in biomedical research. Indeed, CNVs are suspected to have a great influence on the outbreak of several complex diseases and could explain a part of the missing heritability phenomenon observed in genome-wide association studies. The objective of this study was to detect CNVs in a porcine model of melanoma, the MeLiM pig, which develops spontaneous cutaneous melanomas at birth or within the first months of life, before undergoing a complete tumor regression. We took advantage of high-throughput SNP genotyping realized on a backcross pedigree MeLiM × Duroc and used the raw intensity data from more than 60 000 markers to detect potential CNV events with the PennCNV software. We observed CNVs specific to MeLiM and to Duroc breeds, and corresponding to deletions as well as segmental duplications. Thanks to the family information of the pedigree, a filter to remove spurious CNVs was performed. We are now selecting and annotating each CNV specific to the MeLiM pigs. A subset of relevant CNVs will be validated with quantitative PCR in both MeLiM purebreds and

 © 2011 The Authors. Journal Compilation © 2011 John Wiley & Sons A/S

MeLiM × Duroc animals and association studies with melanoma-related traits will be performed.

P132

Acral lentiginous melanoma

A. Manjare, P. Pund, S. Tambe, S. Ghate, R. Dhurat
L.T.M.M.C and G.H. Sion, Mumbai, Navi-Mumbai, India

Although acral lentiginous melanoma is a common subtype in Asian population (<0.5 per 10 00 000), the overall incidence of melanoma is very low compared to white population (17.5 per 10 00 000). Three male patients aged 65, 50 and 46 yr presented with painful black lesions associated with discharge over the left sole in first two and right big toe in the third case since 3 months, 9 yr, one and half years respectively. Cutaneous examination revealed multiple dark nodules associated with patch with uneven pigmentation and irregular border in the first case. The other two cases showed blackish plaque on left sole and right great toe respectively. Inguinal Lymphadenopathy was present in first case with evidence of metastasis on FNAC of inguinal lymph node. Histopathology of all the tumors revealed atypical melanocytic cells in lentiginous pattern with uniform cellular atypia suggesting the diagnosis of acral lentiginous melanoma. In all three patients the tumor was excised with reconstruction surgery in only two cases. Acral lentigenous melanoma, a rare variant of malignant melanoma is the most common type in Asian population. Early detection, surgical intervention is desirable.

P133

Xeroderma pigmentosum type C: report of a case with multiple melanomas.

S. Norrenberg, V. Del Marmol, M. Candaele, M. Abramowicz, A. Daubos, C. Ged
Hopital Erasme ULB, Bruxelles, Belgium and INSERM 1035, Bordeaux, France

Xeroderma pigmentosum (XP) is a rare, autosomal recessive genodermatosis, characterized by DNA repair deficiency, after UV-induced damage, caused by mutations in genes involved in nucleotide excision repair. XP patients have severe photosensitivity and >1000 fold increased incidence of UV-induced cancers at an early age (<10 yr), which are mostly non melanoma skin cancers. Seven complementation groups, XP-A through XP-G, and a variant form (XP-V) have been described. XP type C (XP-C) is caused by biallelic inactivating mutations in the XPC gene involved in global genome repair (GGR). We report the case of a 25-yr-old Caucasian male, who presented typical cutaneous features of XP during infancy, and developed multiple skin cancers from age 6. Numerous basal and squamous cell carcinomas developed during childhood, followed from age 23, by multiple melanomas including in situ and invasive profiles. DNA repair analysis, performed at age 15, showed 15% residual activity. XPC gene sequencing evidenced compound heterozygosity for two novel mutations: a nonsense and a missense. The XPC protein, analysed by western blot, was barely detectable. The influence of additional mutations interesting melanoma susceptibility genes is under investigation. The singularities of the present case will be discussed in view of previously published observations: Norgauer J., Idzko M., Panther E., Hellstern O., Herouy Y. (2003) Xeroderma pigmentosum. Eur. J. Dermatol. 13:4–9. Blankenburg S., König I. R., Moessner R., Laspe P., Thoms K.M., Krueger U., Khan S.G., Westphal G., Berking C., Volkenandt M., Reich K., Neumann C., Ziegler A., Kraemer K.H., Emmert S. (2005) Assessment of three xeroderma pigmentosum group C gene polymorphisms and risk of cutaneous melanoma: a case–control study. Carcinogenesis 26:1085–90. Jacobelli S., Soufir N., Lacapere J.J., Regnier S., Bourillon A., Grandchamp B., Hétet G., Pham D., Palangie A., Avril M.F., Dupin N., Sarasin A., Gorin I. (2008) Xeroderma pigmentosum group C in a French Caucasian patient with multiple melanoma and unusual long-term survival. Br. J. Dermatol. 159:968–73.

P134

The Molecular Phenotype of Acquired Melanocytic Naevi

A. Bonthuys, G. Todd, G. Govender, S. H. Kidson
Faculty of Health Sciences, University of Cape Town, Cape Town, South Africa

Understanding of the pathogenesis of cutaneous melanoma remains a major challenge. The cancer stem cell hypothesis is gaining widespread recognition as it challenges the widely accepted model of cancer development. This model further opens the question of the relationship between melanocytic naevi and melanoma. To examine the hypothesis that melanocytic naevi may originate from a pluripotent or neural crest-like stem cell and not via de-differentiation from a mature epidermal melanocyte, and to characterise, in detail, the phenotype of a 'naevus cell', we are systematically re-evaluating a series of formalin-fixed, paraffin embedded naevus biopsy samples, as well as cultured naevus cells. Compound, junctional, intradermal, blue and dysplastic naevus subtypes are being analysed using combined techniques of immunofluorescence, real time PCR and protein expression analysis. Melanocyte markers Melan A (Mart-1) and S100, as well as the Schwann cell markers S100 and Protein 0 are being utilised to evaluate the phenotype of naevus cells. To determine the presence and/or contribution of pluripotent or precursor cells within naevus tissue, we are using antibodies to pluripotency markers OCT 4 and NANOG; and to neural crest precursor marker NGFR P75. Lastly, to determine the presence and/or location of possible proliferating naevus cells, we are using the proliferation marker Ki-67 in combination with the above selected markers. Preliminary results show high levels of Melan A and S100 expression in the epidermal and superficial dermal component of naevi, however, there is a loss of Melan A staining, with a persistence of S100 staining in the deeper dermis. In some cases, nests of naevus cells appear to express high levels of OCT 4, while only a few – yet some, express NANOG and NGFR P75. These preliminary results support the previous finding of a decrease in melanocyte specific markers in deeper dermal nests with a possible Schwann cell phenotype of these deeper naevus cells. The presence of stem cell markers in naevus tissue supports the hypothesis that at least some naevus cells may arise from stem cells, and not from differentiated melanocytes.

P135

Genome-wide shRNA screen for tumor suppressors mediating oncogene-induced senescence

K. Meissl, K. Terlaak, D. S. Peeper
Netherlands Cancer Institute, Amsterdam, The Netherlands

Oncogene-induced senescence (OIS) is a tumor-suppressive mechanism that restrains the outgrowth of neoplastic cells in vivo. We have shown previously that OIS occurs in oncogene-expressing melanocytic nevi in humans, confining the progression of these benign lesions to melanoma. Such malignant transformation events are relatively rare, however, suggesting that additional (epi)genetic alterations are required. Animal models with melanocyte-specific oncogene expression support these findings. To date, only few mediators of OIS have been identified. We therefore performed a genome-wide shRNA OIS bypass screen to identify mediators of RAS-induced senescence.

© 2011 The Authors. Journal Compilation © 2011 John Wiley & Sons A/S

We made use of a genome-wide shRNA (TRC) library, that was transduced into a cell system that allows for conditional induction of RAS-induced senescence. We screened for shRNAs that were enriched in cells bypassing OIS, by quantitative high throughput sequencing (Illumina). The analysis of technical replicates, used to determine the quality of PCR amplification and sequencing, revealed that we could reproducibly detect shRNAs with a high abundance. Gratifyingly, shRNAs targeting genes that are known to play a role in OIS (Cdkn2a, p53) were among the top outliers. We are currently validating our top 25 hits. By identifying critical mediators of OIS we aim to gain further insight into the molecular mechanism of this tumor suppressive mechanism and to identify signaling pathways that contribute to malignant transformation.

P136

Glycoprotein nonmetastatic melanoma protein b (GPNMB) is a melanosome-specific cell marker and is proteolytically released by ectodomain shedding

T. Hoashi, S. Sato, Y. Yamaguchi, T. Passeron, K. Tamaki, V. J. Hearing
Sanraku Hospital, Tokyo, Japan

Melanosomes are organelles specialized for the production of melanin pigment and are specific to melanocytic cells. More than 150 pigmentation-related genes that associated with pigmentation have been identified to date, including glycoprotein nonmetastatic melanoma protein b (GPNMB).
A recent proteomics analysis revealed that GPNMB is localized in melanosomes, and GPNMB is a membrane-bound glycoprotein which shows high homology with a well-known melanosomal structural protein, Pmel17/gp100. In this study, we show that GPNMB is predominantly expressed in melanocytes of normal human skin as well as in human melanoma cells, even in amelanotic melanoma cells. GPNMB is heavily glycosylated and is enriched in Stage III and IV melanosomes.
We also demonstrate that a secreted form of GPNMB is released by ectodomain shedding from the largely Golgi-modified form of GPNMB and that a phorbol ester or a calmodulin inhibitor induces the shedding. We conclude that GPNMB is a melanosomal protein that can be released by regulated proteolytic ectodomain shedding.

P137

Analysis of global 5-hydroxymethylcytosine in malignant melanoma and acquired melanocytic nevi

H. Fujiwara, M. Ito
Institute of Medicine and Dentistry, Niigata University, Niigata, Japan

Epigenetic regulation, e.g. cytosine methylation, histone code, miRNA, plays a significant role in oncogenesis. Cytosine methylation in promoter regions suppresses gene expression in general, and the expression of several tumor suppressor genes, e.g. p16, CDH1, were down-regulated by cytosine methyltion in malignant melanoma. In 2009, another modification of cytosine, 5-hydroxymethylcytosine (5-hmC) was discovered. Although the role of 5-hmC in gene expression remains to be elucidated to date, its different function from 5-methylcytosine (5-mC) was speculated. We developed a novel method to measure 5-hmC, NICEA for 5-hmC, based on the global methylation analysis method, non-isotopic cytosine extension assay, and analyzed the 5-hmC in malignant melanoma and acquired melanocytic nevi. Genomic DNA was extracted from malignant melanoma (n = 9) and acquired melanocytic nevi (n = 10). The extracted DNA was treated with the combination of T4-beta-glucosyltransferase and restriction enzymes MspI or HpaII, resulted in differentially created overhang at the recognition site CCGG. Incorporating biotinylated cytosine at the overhang, the biotinylated DNA was visualized on the nylon membrane, and underwent densitometry. The relative amount of unmodified cytosine (5-C) was identical in melanoma and nevi. The amount of 5-mC was significantly smaller and that of 5-hmC was larger in melanoma (P = 0.01). We developed the novel method, NICEA for 5-hmC, for analyzing global 5-hmC, and reported the difference of 5-hmC between melanoma and nevi. Although the function of 5-hmC is yet to be determined, our data suggest the importance of 5-hmC in developing malignant melanoma.

P138

Atypia grading in nevi by reflectance confocal microscopy

I. Vaisnoriene, J. Venius, J. Didziapetriene, R. Rotomskis, K. P. Valuckas
Vilnius University Oncology Institute, Vilnius, Lithuania

Dysplastic nevi are important clinically because they are associated with a higher risk of developing malignant melanoma (MM). Dysplasia/atypia gradation in three degrees (mild/moderate/severe) is based on histological evaluation of architectural and cytological features. In vivo reflectance confocal microscopy (RCM) enables the visualization of superficial skin layers at cellular-level resolution. The algorithm is developed for distinguishing between benign melanocytic lesions and MMs using RCM. RCM allows express diagnostics without invasion in vivo. The purpose of the research was to develop an algorithm and prove existing criteria of dysplastic nevi for in vivo distinguising between mild, moderate and severe degrees of atypia in nevi by RCM. Total of 34 benign and malignant melanocytic skin lesions were investigated by RCM before excision. We analyzed RCM features, evaluated atypia grade and and compared with histological grade of atypia. RCM evaluation criteria were following: (i) mild/moderate/severe distortion of the dermo-epidermal junction (DEJ) architecture (irregular shape dermal papillae); (ii) mild/moderate/severe degree of atypia in nevomelanocytes (heterogenous in brightness, size and shape) and (iii) mild/moderate/severe disarray of cell clusters. Normal epidermal honeycomb and cobblestone patterns were considered features for mild/moderate atypia. Severe atypia in nevi was distinguished by (i) disruption of the DEJ and focal loss of the cell-cell keratynocytic boundaries (edged and non-edged dermal papillae) and (ii) atypical keratinocytes or 'pegetoid' melanocytes within epidermis. Common nevus was distinguished from atypical nevus and MM by (i) normal architecture of DEJ and (ii) no cellular atypia. Malignant melanoma was distinguished from atypical nevi by six criteria based algorithm (cell atypia at the DEJ, non-edged dermal papillae, roundish pegetoid cells widespread infiltration, cerebriform nests, nucleated cells within upper dermis). Exact RCM atypia grade evaluation correlation with histopathology diagnosis was achieved in four of five cases in common nevi, in two of six cases in mild atypia, in one of four cases in moderate atypia, in seven of eight cases in severe atypia, in 11 of 11 cases in MM. In conclusion, preliminary results show that grading of atypia in nevi by general RCM criteria for dysplastic nevi allows 73.5% correlation with histopathology diagnosis. However, severe atypia and MM was diagnosed with 94.7% accurancy. For even more accurate diagnosis of dysplastic nevi we need to look for more specific criteria in RCM.

 © 2011 The Authors. Journal Compilation © 2011 John Wiley & Sons A/S

P139

Diagnostic interest of RACK1 in melanoma

R. Nkosi, S. Meyer, N. Martin, R. Barnhill, M. Battistella, F. Bernex, G. Houzelstein, A. Janin, X. Sastre-Garau, J.-J. Panthier, C. Lebbé, G. Egidy
INRA-ENVA, Maisons-Alfort, France

The term melanoma comprises a family of malignant tumors developing from melanocytes of the skin, mucous membranes, eyes and internal organs. Cutaneous melanoma is the deadliest form of skin cancer. Ocular melanoma is the most common type of cancer affecting the eye. Although routine anatomopathological analysis of lesions is quite precise, histological features are not always sufficient to determine tumour aggressiveness. An earlier detection of primary melanoma would help improve prognosis. Most melanocyte markers are based on pigment synthesis, hence shared by benign and malignant lesions. Markers identifying malignant lesions are needed. We have recently identified the detection of RACK1 on tissue sections as a possible marker of malignancy of human melanocytic proliferations. We wished to obtain the tangible proof of RACK1 detection utility as a marker of melanocytic malignancy by testing a series of early melanoma stages. A collection of samples has been constituted consisting of 30 different samples of stage I and II cutaneous melanoma, blue naevi and uveal melanoma of good prognosis, as well as control tissues consisting of common naevi, stage III and IV melanoma and bad prognosis uveal melanoma. Detection of RACK1 and MITF by double immunofluorescence has been performed on sections. RACK1 was abundantly detected in most malignant samples from stage I onwards. In contrast, RACK1 was not detected in normal epidermal melanocytes and was poorly detected in nevi. Confirmation of RACK1 detection as malignancy marker in melanoma will be further exploited looking for partners in the tissue collection.

P140

RACK1 is a marker and a catalyst in melanoma development

C. Campagne, S. Julé, S. Bibi, C. Koenen, E. Reyes-Gomez, F. Bernex, S. Pons, U. Maskos, J. J. Panthier, G. Aubin-Houzelstein, G. Egidy
UMR955 Functional and Medical Genetics, Maisons-Alfort, France

Cutaneous melanoma is the deadliest skin cancer. Severe prognosis results from its high metastatic capacity with radio- and chemoresistance. Earlier histological characterization of the primary malignant melanoma would help improve prognosis. We have shown that RACK1 (Receptor for Activated C Kinase-1) overexpression, detected in situ, characterized human cutaneous melanoma and its metastasis: RACK1 could be a marker of malignancy in melanoma. As a scaffold protein RACK1 could allow cross-talk of several pathways involved in melanoma development. We focus on understanding RACK1 function in melanoma development in vivo with a genetic approach using the spontaneous melanoma bearing mice Tyr::NRasQ61K; Ink4a–/–. To test whether RACK1 has any relevance in melanoma development we produced Tyr::Rack1-HA transgenic mice. We crossed them with the melanoma model mice. We evaluated the therapeutic potential of targeting RACK1 in melanoma cells using RNA interference. We analysed RACK1 status in situ in healthy melanocytes from predisposed mammals to test its predisposition potential. Tyr::Rack1-HA mice did not develop melanoma. Nonetheless, when crossed with melanoma model mice, tumours developed earlier. This suggests that RACK1 could be a catalyst in melanoma development. In vitro RACK1 knock-down reduced tumorigenic potential of mouse melanoma cells. We showed that RACK1 is present in healthy skin melanocytes from several predisposed melanoma models. RACK1 seems to be an actor in melanoma development. We are currently identifying RACK1 partners and the biological pathways in which it is involved. Eventually, this should allow us to identify new therapeutic targets for melanoma treatment.

P141

Study of MAPk pathway components – Ras, Braf, Mek 1/2 and Erk 1/2 in series of 35 cases primary oral mucosal melanoma

R. Hsieh, M. Buim, S. V. Lourenço
Medical School, University of Sao Paulo, Sao Paulo, Brazil

Primary oral mucosal melanomas are rare, accounting for approximately 2% of all melanomas, with an incidence of 1.2 cases per 10 million per year. They are considered more aggressive and have a poorer prognosis than their cutaneous counterparts. According to the literature, there are well-established factors participating in the development and evolution of cutaneous melanomas, such as familial and environmental factors. However, the aetiology and pathogenesis of oral mucosal melanomas are poorly understood. Recent studies have reported somatic genetic alterations affecting the mitogen-activated protein kinase (MAPK) signalling pathway in the pathogenesis of cutaneous melanomas. This pathway comprises the Ras-Raf-MEK-ERK cascade and is considered an important regulator of melanoma cell proliferation, survival and differentiation. To assess the status of the MAPK pathway in primary oral mucosal melanomas pathogenesis, our study examined the key components of Ras-Raf-MEK-ERK cascade by immunohistochemistry in a series of 35 primary oral melanomas organized in tissue microarray (TMA). Patients' ages ranged from 9 to 91 yr and the average age at time of diagnosis was 61 yr old. There were 18 males (51.43%) and 17 (48.57%) females and the main prevalence of melanomas were amongst whites (21 patients or 75%) against non-whites (25%) (five Black/Afro-Brazilian, two Asian). The majority of the patients (71.42%) had palate lesions and invasive histopathological aspect (grade III) was observed in 80% of the specimens. Long distance metastasis was found in 60% of the cases. Expression of MAP kinase cascade proteins was altered in most cases. Ras was positive in 28.57% of the cases studied; Braf and ERK2 were overexpressed in most cases (82.85 and 74.28%, respectively); MEK2 and ERK1 were downregulated (48.57 and 80% of cases were negative, respectively) and MEK1 was not expressed in all cases. Downregulation of Ras and ERK1 and positivity for Braf and ERK2 were correlated with higher histological grade, vascular invasion and metastasis. Additionally, expression of MEK2 was statistically correlated with vascular invasion ($P = 0.043$). These results point to important alterations of Ras-Raf-MEK-ERK cascade and those are related to histological indicators of poor prognosis in primary oral mucosal melanomas, independent of the UV-exposition.

P142

The significance of micrometastases in sentinel nodes in Japanese melanoma patients: a retrospective analysis of 450 cases

N. Yamazaki, A. Tsutsumida, K. Namikawa, Y. Kiyohara
National Cancer Center Hospital, Dermatologic Oncology, Tokyo, Japan

Malignant melanoma is relatively rare in Japan. Patients with a positive sentinel node (SN) have conventionally undergone completion lymphadenectomy (CLND) of the involved basin.

© 2011 The Authors. Journal Compilation © 2011 John Wiley & Sons A/S

Trends in modern surgery have favored procedures that are less invasive. We try to identify criteria that might be used to be more restrictive in selecting those who should undergo CLND. Four hundred and fifty Japanese patients with melanoma underwent a sentinel node biopsy (SNB) procedure in Japan Melanoma Study Group. SNs were identified by the standard triple technique. The SN was step-sectioned and examined by H&E and immunohistochemical staining. We selected the following three microscopic classifications of tumor lesion in SN; maximum diameter, invasion depth and microanatomic location. One hundred and forty-nine patients (33.1%) had positive SNs. 38.3% (57/149) of SN positive patients have further non-SNs that contain tumor deposits in CLND specimen. 0% of additional lymph node positivity was for a maximum diameter of the tumor of <0.1 mm. Five year overall survival rate was 85.5% for cases without SN metastasis. The prognosis was poorer for all the parameters included in the microscopic classifications of metastatic SN compared with that in the SN metastasis-negative cases. For the overall survival, significant differences were demonstrated by multivariate analysis between the microanatomic location category of 'extensive' and the four categories ('subcapsular', 'combined', 'parenchymal', or 'multifocal') other than 'extensive'. The results of SNB provide important prognostic information for Japanese patients with melanoma, but it is not easy to determine in which subgroup CLND could safely be avoided.

P143

Detection, quantification and characterisation of PAX3 across the spectrum from melanocytes to melanoma and in circulating melanoma cells relative to disease stage

M. Ziman, M. Millward, S. Medic, A. Reid, J. Freeman, R. Pearce, M. Lee, P. Heenan, A. Ireland, P. Kumarasinghe Melanoma Research Foundation, Edith Cowan University, Perth, Australia

The aim of this research was to assess PAX3 expression across the spectrum from melanocyte to melanoma and determine its role in tumour progression. The transcription factor PAX3 regulates melanocyte specification from neural crest cells during development but expression in differentiated melanocytes has not been detailed to date. In this study we analysed PAX3 expression in normal skin melanocytes, in melanoma cells of primary and metastatic tissue and in circulating melanoma cells. We then quantified and characterised circulating melanoma cells using a variety of markers including PAX3. PAX3 expression was assessed in normal skin melanocytes and melanoma cells, using immunofluorescence and qRT-PCR. qRT-PCR was also used to quantify PAX3 levels relative to other melanoma markers, in patient blood of 300 melanoma patients and 100 healthy volunteers. Frequency and level of expression of melanoma markers in patient blood were correlated with Breslow thickness of the primary tumour and to presence of metastases and results were statistically analysed. Immunomagnetic bead capture and flow cytometry were used to isolate, quantify and characterise circulating cells from patient blood samples. Here we demonstrate PAX3 in normal skin melanocytes as well as in melanoma cells of primary melanoma and metastatic melanoma, and in circulating melanoma cells. Furthermore, circulating melanoma cells were identified in 79% of patients with stage III and IV disease whilst these markers were observed in only 20–30% of early stage patients. Surprisingly, melanoma cells are found in peripheral blood of patients with no clinical evidence of metastatic disease, including patients with early stage disease and patients from whom tumours were removed several years previously. Flow cytometry and immunomagnetic bead capture confirmed the presence and quantity of circulating melanoma cells relative to disease stage. PAX3 is a useful marker of melanocytes and melanoma cells, including circulating melanoma cells. As such, PAX3 can be utilised as one of several melanoma markers for quantification and characterisation of circulating melanoma cells and may be a valuable clinical indicator of disease progression and treatment efficacy. This research is funded by grants from the NHMRC of Australia and the Cancer and Palliative Care Research and Evaluation Unit.

P144

Real-time tissue elastography is useful for detecting lymph-node metastases in melanoma

K. Yoshio, O. Dai, N. Michiko
Shizuoka Cancer Center Hospital Dermatology, Sunto-gun, Shizuoka, Japan

We have previously reported the improvement of diagnostic accuracy by combined use of Color Doppler Imaging (CDI), Pulse Doppler Imaging (PDI) and Real-time Tissue Elastography (RTE) for primary skin cancer. Recently, we have expanded application of this method to lymph-node metastasis and found its feasibility. Especially, small metastasis of lymph-node is more important for patients with melanoma than those with other types of skin cancer (e.g. squamous cell carcinoma). In case of melanoma, microscopic metastasis occurs even in early phase, so it is difficult to manage metastasis, resulting very poor prognosis. We performed lymph-node RTE for eight melanoma patients, and examined 14 lymph-nodes histologicaly. Sensitivity of this method was 88%, and specificity was 100%. In almost all cases, RTE demonstrated lymph node metastasis better than qualitative assessment with PET-CT or MRI. In conclusion, RTE is useful for detection of lymph node metastasis in melanoma patient.

P145

Development and evaluation of antitumor effect of novel NPrCAP-magnetite nanoparticles for chemo-thermo-immunotherapy in malignant melanoma

A. Yoneta, Y. Tamura, S. Nohara, A. Ito, H. Honda, K. Wakamatsu, S. Ito, T. Yamashita, K. Jimbow
Department of Dermatology, Sapporo Medical University, Sapporo, Japan

N-Propionyl-4-S-cysteaminylphenol (NPrCAP) is a melanogenesis substrate, specifically taken up by melanoma cells and inhibits their growth by producing cytotoxic free radicals. By taking advantage of this unique chemical agent, we have established melanoma targeting intracellular hyperthermia by conjugating NPrCAP with magnetite nanoparticles (NPrCAP/M) which generate thermal heat upon exposure to alternating magnetic fields (AMF). NPrCAP/M with AMF to B16 melanoma grown in C57BL mice inhibited the growth of not only primary transplant of melanoma cells (chemotherapeutic effect) but also re-challenged, secondary melanoma transplant on to the opposite side of the body (immunotherapeutic effect). This treatment strategy also showed the melanoma necrosis and immune reactions generating cytotoxic T cells with regression of distant skin metastases in human clinical trials. These particles (NPrCAP/PEG/M) were, however, found to be unstable at room temperature and aggregated at the site of subcutaneous administration around/or within melanoma tissues, hence it being necessary to develop a new formula of NPrCAP-magnetite particles that will be stable, and more efficiently and diffusely incorporated into melanoma tissues. We have successfully made new particles (NPrCAP/PEG/DNM) in which maleimide-PEG5000-Carboxyl-NHS is used to combine NPrCAP and dextran magnetite

 © 2011 The Authors. Journal Compilation © 2011 John Wiley & Sons A/S

(DNM). These NPrCAP-magnetite particles are 50–60 nm in diameter, much smaller than that of the previous one (350–500 nm), and generate heat efficiently upon exposure to AMF. They did not aggregate around/or within melanoma tissues, and were more diffusely and uniformly distributed. Administration of these new NPrCAP nanoparticles with/or without AMF exposure resulted in marked growth inhibition of primary and secondary melanoma transplants. Novel chemo-thermo-immunotherapy to malignant melanoma can be established by employment of the newly developed NPrCAP/PEG/DNM that will be selectively, diffusely and efficiently incorporated into melanoma cells through melanogenesis cascade.

P146

MAGE proteins are master regulators of KAP1 and KRAB domain zinc finger transcription factor mediated gene suppression

T. Z. Xiao, N. Bhatia, R. Urrutia, G. A. Lomberk, A. Simpson, B. Jack
Department of Dermatology, University of Wisconsin, Madison, Wisconsin, USA

Class I MAGE proteins are normally expressed only in developing germ cells but are aberrantly expressed in melanoma and many other cancers, making them ideal therapeutic targets. In primary melanomas, MAGE expression is a strong prognostic biomarker, negatively correlated with relapse free survival independent of thickness, ulceration, and mitoses. We have previously shown that MAGE proteins promote melanoma survival in vitro and in vivo by binding to the KAP1 scaffolding protein and enhancing KAP1 suppression of p53, but MAGE functions and the underlying mechanisms have not been fully elucidated. The KRAB domain zinc finger transcription factors (KZNFs) are the largest group of vertebrate transcription factors. KZNFs also bind to KAP1 and direct it to specific DNA sequences where it represses gene expression by inducing localized heterochromatin characterized by histone t lysine 9 trimethylation (H3me3K9). Our objective was to test the hypothesis that MAGE proteins can affect KZNF KAP1 interactions and function we used loss of function mutants, ChIP, ChIP-seq, RT-qPCR and immunoblotting to determine effects of MAGE expression on KAP1 binding, chromatin structure, and gene repression. We found that MAGE expression relieves repression of a reporter gene by ZNF382, a KZNF with tumor suppressor activity. ChIP of MAGE (–) HEK293T cells showed KAP1 and H3me3K9 are normally bound to the ID1 oncogene, a target of ZNF382, but that binding is greatly reduced in the presence of MAGE proteins. MAGE expression relieves KAP1 mediated ID1 suppression, causing increased expression of ID1 mRNA and ID1 chromatin relaxation characterized by loss of H3me3K9. MAGE binding also induces KAP1 mediated ZNF382 poly-ubiquitination and degradation, consistent with loss of ZNF382 leading to decreased KAP1 binding to ID1. In contrast, MAGE expression caused increased KAP1 binding to other genes with increased H3me3K9 and decreased mRNA expression. Chip-seq showed MAGE affects KAP1 binding to large numbers of genes including oncogenes and tumor suppressors. Since KZNFs are required to direct KAP1 to specific genes, we conclude that MAGE proteins can differentially regulate members of the KZNF family and KAP1 mediated gene repression. Global analysis suggests that MAGE may be master regulators of transcription, affecting genes that involved in neoplastic transformation, tumor suppression, cellular proliferation, and apoptosis.

P147

N-Propionyl-4-S-Cysteaminylphenol generates reactive oxygen species and mediates apoptosis in pigmented melanoma cells

Y. Ishii-Osai, T. Yamashita, M. Okura, Y. Tamura, N. Sato, A. Ito, H. Honda, K. Wakamatsu, S. Ito, K. Jimbow
Sapporo Medical University School of Medicine, Sapporo, Japan

N-propionyl-4-S-cysteaminylphenol (NPr-4-S-CAP) is selectively incorporated into melanoma cells as a substrate of the tyrosinase and degrades them. In this study, we aimed to elucidate the mechanism of the cell death induced by NPr-4-S-CAP and its antimelanoma effect. We first conducted cell proliferation assay, flow cytometric analysis and caspase assay to assess the growth-inhibitory activity and elucidation of the mechanism of cell death induced by NPr-4-S-CAP in vitro.
We also examined the relation between the NPr-4-S-CAP-mediated ROS generation and cytotoxicity in pigmented and non-pigmented melanoma cells. Results indicated that NPr-4-S-CAP, but not inactive NPr-2-S-CAP, suppressed growth of B16F1 mice melanoma cells in a dose-depending manner. NPr-4-S-CAP-treated B16F1 cells showed degradation accompanied with an activation of caspase 3 and DNA fragmentation. Pigmented melanoma cell lines produced larger amounts of intracellular ROS and underwent apoptosis more significantly than non-pigmented cell lines. This suggests that NPr-4-S-CAP selectively induces apoptosis in pigmented melanoma cells and ROS generation plays an important role in the process.

P148

Expression of NADPH oxidase 1 in melanoma cells and its effect on invasion via induction of matrix metalloproteinase-2

F. Liu, A. Garcia, F. L. Meyskens
University of California, Irvine, Orange, CA, USA

NADPH oxidase (EC 1.6.3.) is a family of enzymes that catalyze transfer of an electron from NAD(P)H to molecule oxygen to generate superoxide or hydrogen peroxide. It consists of seven members, represented by their catalytic subunits: Nox1, Nox2 (gp91phox), Nox3, Nox4, Nox5, Duox1 and Duox2. Among these members Nox4 was previously demonstrated to play a role in melanoma invasion down-stream of Akt pathway. Expression of Nox1 was not reported in melanoma cells. RT-PCR and Western blot analysis was used to analyze expression of Nox1, Noxo1 and p22phox in normal melanocytes and melanoma cells; over-expression, knockdown or inhibition of Nox1 was achieved in radial growth phase Wm3211 melanoma cells, cell invasion was assayed using Matri-gel coated transwells. Expression of MMP-2 was analyzed by qRT-PCR and western blot in these cells. Promoter reporter plasmid of human MMP-2 is being constructed; promoter activity will be analyzed in cells clones with varied Nox1 expression levels. Nox1 was expressed in normal melanocytes and all melanoma cell lines examined, as in contrast to Nox4 which was not expressed in normal melanocytes but only expressed in a subset of metastatic melanoma cell lines. Nox1 subunits Noxo1 and p22phox were also expressed in normal melanocytes and all melanoma cell lines. Over-expression of GFP-fused Nox1 protein in Wm3211 cells increased cell invasion rate by four to six-fold, while knocking down Nox1 decreased invasion rate by two to three-fold. Inhibiting Nox1 by Diphenyliodonium (DPI) also inhibited invasion rate. We further found that secreted MMP-2 increased in cells over-expressing Nox1, and decreased in cells with Nox1 knockdown. Quantitative RT-PCR analysis demonstrated the regulation of MMP-2 may occur at transcription level as mRNA of MMP-2 increased about 10 to 11 fold in cell clones over-expressing Nox1. Our data

© 2011 The Authors. Journal Compilation © 2011 John Wiley & Sons A/S

shows that Nox1 is over-expressed in all melanoma cell lines examined; it contributes to enhanced cell invasion by activating MMP-2 at transcriptional level in melanoma cells.

P149

MDA MB 435, SKMEL pigmented and nonpigmented melanoma cell lines and MDA MB 231 cancer cell line – derived cancer stem cells (CSC) show differential expression of green fluorescent protein driven by Oct4 promoter in non-green versus green populations determined by FACS

Z. S. Pavicevic, R. I. Krutilina, A. R. Chatterjee, C. D. Duntsch, T. N. Ignatova, V. G. Kukekov
UTHSC, CRB, Memphis, USA

We have created a method for identification, sorting, and stable maintenance in culture of cancer stem-like cells based on their ability to maintain the expression of green fluorescent protein (GFP) driven by the POU5f/Oct4 promoter. We established a culture system which maintains isolated GFP-enriched MDA MB 435, SKMEL, and MDA MB 231 subpopulation in purity through serial passages and non-green GFP-depleted subpopulation, respectively. We created an orthotopic model in which GFP-enriched melanoma MDA MB 435 and MDA MB 231 cells were injected into to the mammary fat pad of nude mice and demonstrated that GFP- enriched MDA MB 435 and MDA MB 231 cells have higher tumorigenic and metastatic potential. Whereas GFP-depleted cells do not. Cancer stem cells are resistant to the numerous conventional therapies. When we studied tumor derived GFP-enriched MDA MB 231 cells derived from orthotopic and metastatic tumors by Fluorescence Activated Cell Sorting (FACS) we found that FACS method gives contradictory data on the GFP- expression status of cell populations isolated from the tumors. This is a very important finding since GFP is a widely used biomarker that can label Cancer Stem Cell fraction within the tumor

P150

Active forms of vitamin D3 inhibit melanoma growth and are dependant on the activity of NF-kB pathway

Z. Janjetovic, A. A. Brozyna, R. C. Tuckey, M. N. Nguyen, S. R. Pfeffer, L. M. Pfeffer, W. Jozwicki, A. T. Slominski
School of Medicine, University of Banjaluka, Bosnia and Herzegowina and UTHSC, Memphis, USA

Melanoma is highly resistant to current modalities of therapy, with pigmentation playing an important role in therapeutic resistance. NF-kB is constitutively activated in melanoma and can serve as a molecular target for cancer therapy and steroid/secosteroid action. Cultured melanoma cells were used for mechanistic studies on NF-kB activity, utilizing immunofluorescence, western blotting, EMSA, ELISA, gene reporter and proliferation assays. Formalin-fixed, paraffin-embedded tissue samples from melanoma patients were used for immunohistochemical analysis of NF-kB activity in situ. Novel 20-hydroxyvitamin and 1a,25-dihydroxyvitamin D3 inhibited melanoma cell proliferation. The NF-kB inhibition by active forms of vitamin D was confirmed by NF-kB DNA binding and NF-kB-dependent reporter assays in nonpigmented cells, while having little effect on pigmented cells, due to lower expression of VDR in melanized cells. Treatment of nonpigmented cells with vitamin D3 derivatives resulted in inhibition of the nuclear translocation of the p65 NF-kB subunit and its accumulation in the cytoplasm. Translocation of NF-kB into the cell nuclei was accompanied by higher proliferation activity assessed using both Ki67 immunostaining and mitotic index in melanoma tissue, as well as VDR expression. Also, analysis of clinical samples from melanoma patients showed that nonpigmented and slightly pigmented melanomas displayed higher nuclear NF-kB expression and higher percentage of Ki67-positive cells than highly pigmented melanomas. Therefore, hydroxy-vitamin D3 derivatives, 20(OH)D3 and 1,25(OH)2D3, can target NF-kB and inhibit melanoma progression. Furthermore, strong melanin pigmentation is associated with the resistance of melanomas to 20-hydroxyvitamin D3 and 1a,25-dihydroxyvitamin D3 treatment.

P151

Aurora kinases play a critical role in hexavalent chromium-induced aneuploidy in immortalized human melanocytes

L. Xie, F. Liu, A. Garcia, F. Meyskens
The Chao Family Comprehensive Cancer Center, University of California, Irvine, Santa Anna, USA

Hexavalent chromium [Cr(VI)] has been widely used in industries throughout the world. Epidemiological studies demonstrate a strong association between Cr(VI) and cutaneous melanoma (CM). A large meta-analysis also demonstrates an increase of CM in metal-on-metal hip athroplasties that is time dependent. However, the carcinogenic mechanism for chromium is poorly understood although genomic instability is a prominent feature. The Aurora kinase family of serine/threonine kinases comprises three members A, B, and C. Auroras A and B are important regulators of mitosis that are frequently over-expressed in human cancers and have been implicated in the development of chromosomal instability in cancer cells. The goal of this study was to determine the importance of Auroras A and B in Cr(VI)-induced aneuploidy in immortalized human melanocytes as previous work from our laboratory showed that Cr(VI)-treatment caused aneuploidy and foci formation of cultured human melanocytes. In this study, we show that Cr(VI)-treatment increased Aurora A and decreased Aurora B expression and activity. Cr(VI)-induced Aurora A over-expression can be blocked by c-Myc inhibitor (10058-F4, 100 μM), suggesting the induction of Aurora A is mainly through Cr(VI)-induced Myc activation. Cell cycle analysis showed increased proportions of G2/M and polyploidy cells in Cr(VI)-treated cells in comparison to untreated cells; this induction can be decreased by a specific Aurora A inhibitor (MLN8237, 1 μM). In conclusion, the imbalance between Auroras A and B might be a novel mechanism for chromium induced chromosomal instability which may present the opportunity to develop novel prevention and therapeutic compounds as Aurora kinase inhibitors are available.

P152

Identification of MITF regulated genes involved in melanoma proliferation, migration and invasion

D. Koludrovic, T. Strub, I. Davidson
IGBMC, Illkirch, France

Melanoma is highly aggressive form of skin cancer, particularly due to very low survival rate of the patients once the cancer becomes metastatic. Transcription factor MITF is a major regulator of cell differentiation of melanocytes, and is involved in regulation of proliferation, migration and invasive properties in melanoma. The level of MITF in the melanoma cells determines the transcriptional profile of particular melanoma cell lineage. High levels of MITF are characteristic for proliferative cells, while low levels of MITF are associated with slow growing cells, prone to senescence with increased migratory and invasive properties. To better elucidate the role of MITF in melanoma, a Chip-sequencing was performed to determine MITF binding sites and RNA-sequencing was used to determine the gene expression

 © 2011 The Authors. Journal Compilation © 2011 John Wiley & Sons A/S

profile upon MITF knock-down. MITF silencing down-regulated a subset of genes involved in DNA replication and repair, and up-regulated subset of genes involved in migration and invasion properties of melanoma. SHC4 (also known as RaLP) is a signaling adaptor protein previously shown to be involved in melanoma migration. MITF binds to several sites in the SHC4 locus and represses its expression which is strongly up-regulated upon MITF silencing. Transient siRNA-mediated SHC4 silencing has been shown to affect migration in vitro and tumor formation in vivo, but had no effect on proliferation. In contrast, we show that stable SHC4 silencing using a shRNA approach leads to reduced proliferation showing that the basal level of SHC4 expression is important for normal melanoma cell proliferation. A possible signaling pathway underlying this phenotype will be discussed. MITF bind to and represses the expression of several other genes that are strongly induced upon MITF silencing. These include are matricellular proteins of extracellular matrix that have already been shown to be involved in melanoma cell adhesion properties, chemostasis, angiogenesis and survival. MITF also represses the expression of several tyrosine kinase receptors and transmembrane signaling adaptors. Many of these genes are upregulated in invasive cells isolated from tumors and several have been shown to be involved in angiogenesis and migration. Their role in melanoma development will be further investigated.

P153

Correlation between secosteroid induced VDR activity in melanoma cells with computer modeling based on the receptor crystal structure

T.-K. Kim, J. Wang, J. Chen, R. C. Tuckey, M. N. Nguyen, D. Miller, W. Li, A. T. Slominski
University of Tennessee Health Science Center, Memphis, USA

Our studies have identified new family of vitamin D derivatives (secosteroids) produced by action of P450scc, which fulfill definition of natural products. They are biologically active and regulate the behavior of a number of cell types including the inhibition of proliferation of normal and malignant melanocytes. To define their interaction with vitamin D receptor (VDR) we used human melanoma cell line overexpressing VDR fused with EGFP, e.g. stably transduced with pLenti-CMV-VDR-EGFP-pgk-puro construct (Am. J. Physiol: Cell Physiol (2011) 300:C526–41). In this study, we tested the ligand induced translocation of VDR from the cytoplasm to the nucleus using wide range of secosteroids including hydroxyderivatives (OH) of vitamin D3 with full (D3) and shortened side chain (pD). All compounds stimulated VDR translocation, however, with different potency. Their EC50 values are 6.1×10^{9}, 3.6×10^{10}, 5.1×10^{11} and 1.8×10^{9} M for 20(OH)D3, 20,23(OH)2D3, 1,20(OH)2D3 and 1,20,23(OH)3D3, respectively, which were comparable with that of 1,25(OH)2D3. In contrast, 22(OH)D3, 20,22(OH)2D3 and 17,20,23(OH)3D3 had lower activity with EC50 values of 2.9×10^{8}, 5.6×10^{9} and 1.4×10^{8} M, respectively. pD analogues were less potent. The ability of secosteroids to bind to the VDR was investigated by molecular modeling based on the crystal structure of human VDR, which showed that binding of hydroxylated vitamin D3 metabolites overlap well with the native ligand (1,25(OH)2D3). D3 compounds have better chance to form hydrogen bond to the VDR binding site than pD compounds based on this docking studies. The docking score ranged from −11.3 to −14.2 for hydroxylated D3 and of −10.7 to −11.3 for pD analogues, most likely due to the important lipophilic interactions between the sidechain and VDR. Also, better score was seen if more OH groups were added with a preference for position 1, indicating its large contributions for strong binding to the VDR. In conclusion, the number of OH groups added, their position and length of the side chain affect the activation of VDR in human melanoma cells.

P154

TWIST1, a B-RAF effector, promotes invasion of melanoma cells

M. B. Weiss, A. E. Aplin
Thomas Jefferson University, Philadelphia, PA, USA

The TWIST1 protein is up-regulated in melanoma and its expression correlates strongly with poor clinical prognosis. We tested the role of high TWIST1 expression in traits associated with the progression of melanoma, as well as sought to delineate the mechanism underlying TWIST1 up-regulation. Our data shows that TWIST1 protein levels are higher in melanoma cells, especially invasive lines, compared to normal melanocytes. To evaluate the biological relevance of TWIST1 expression, we generated invasive melanoma cell lines depleted of TWIST1 by shRNA and non-invasive melanoma cell lines which constitutively overexpress TWIST1. First, we utilized these cell lines in Matrigel invasion assays through boyden chambers. Depletion of TWIST1 in several invasive melanoma cell lines significantly reduces Matrigel invasion. The inverse phenotype is apparent when TWIST1 is overexpressed in low TWIST1-expressing, non-invasive melanoma cell lines. Additionally, we tested the effect of altered Twist expression in 3D collagen spheroid outgrowth assays, which mimic both tumor architecture and the in vivo collagen-rich dermal layer. We observed significant reduction in spheroid outgrowth when invasive cell lines are depleted for TWIST1 as well as increases in outgrowth when non-invasive cells overexpress TWIST1. Alterations to spheroid outgrowth were not as a result of apoptotic changes or proliferative rate. However, we have found that TWIST1 positively regulates a number of matrix metalloproteinases which may contribute to invasion phenotypes. Importantly, the mechanisms regulating high TWIST1 expression in melanoma are unclear. A comparison between wild-type and mutant B-RAF melanoma cell lines demonstrates that TWIST1 is more highly expressed in mutant B-RAF cells. We have found that disruption of B-RAF/MEK signaling, through siRNA/shRNA knockdown or pharmacological inhibition, strongly represses TWIST1 at the mRNA and protein level. The data generated from these studies will allow for a greater understanding of the role and regulation of TWIST1 during the progression of melanoma towards metastasis. In addition, it may highlight TWIST1 as an attractive molecule/pathway for novel targeted therapies for advanced melanoma.

P155

SIRT1 histone deacetylase is a potential therapeutic target for human melanoma

M. Nihal, C. K. Singh, M. Ndiaye, G. S. Wood, N. Ahmad
University of Wisconsin, Madison, USA

Melanoma is one of the most aggressive skin cancers and is potentially lethal, if not diagnosed and treated early. The existing therapeutic approaches have shown little success in melanoma management. This warrants the search for new therapeutic targets to combat melanoma. Therefore, understanding of molecular mechanisms and genetic control of melanoma development and progression is critical. The objective of this study was to assess the role of SIRT1 histone deacetylase in melanoma. SIRT1, a nicotinamide adenine dinucleotide NAD(+)-dependent class III histone deacetylase, has been shown to be critically involved in cellular metabolism, stress response and aging via deacetylating a variety of substrates including p53, forkhead-box transcription

© 2011 The Authors. Journal Compilation © 2011 John Wiley & Sons A/S

factors (FoxO), nuclear factor NF-kB, Ku70 and histones. As a first step, we determined the expression of SIRT1 in multiple melanoma cell lines (A375, Hs294T and G361) and normal skin cells (normal human keratinocytes and normal human melanocytes) as well as in clinical human melanoma tissues and normal skin. Our data demonstrated that compared to normal skin cells, SIRT1 was significantly overexpressed at protein as well as mRNA levels in all the melanoma cell lines tested. Further, we found that SIRT1 was overexpressed in melanoma samples compared to normal skin tissues. Next, to understand the functional significance of SIRT1 in human melanoma, we determined the effects of SIRT1 inhibition on melanoma cells. SIRT1 inhibition via small molecule inhibitors (at the activity level) sirtinol and tenovin-1 as well as via short hairpin RNA-mediated RNA interference (at the genetic level) resulted in a significant (i) decrease in the growth and viability, (ii) induction of apoptosis and (iii) inhibition of colony formation ability, in human melanoma cells. Taken together, our data suggest that SIRT1 may be a contributing factor in melanoma progression. We suggest that SIRT1 could serve as a potential target towards developing novel strategies for the management of melanoma. However, future studies in appropriate in vitro and in vivo systems are required to substantiate our findings and to define the mechanism(s) by which SIRT1 imparts a growth advantage to melanoma.

P156

Phenotype-specific response of melanoma cells to HDAC inhibition

B. Belloni, P. Cheng, D. Widmer, N. Schönewolf, K. S. Hoek, R. Dummer, O. Eichhoff
Universiy Hospital Zürich, Switzerland

Lysine-rich N-termini of Histones are subject to posttranslational modification by acetylation which leads to reduced DNA-binding properties and transcriptional activation. In turn, increased Histone deacetylase (HDAC) activity has been shown to repress transcription of genes related to differentiation, cell cycle arrest and tumor suppression in various cancer cells. There have been no detailed studies investigating the effects of HDAC inhibition on melanoma cells. We investigated the effect of the HDAC inhibitor vorinostat on different melanoma primary cultures in the context of the phenotype switching model for disease progression. Cell cultures of different phenotypes (proliferative or invasive) were treated with vorinostat in increasing concentrations (0.001–10 μM). After 72 h a decrease in cell viability was detected only in proliferative phenotype cells which also showed apoptosis and cell cycle arrest. Furthermore, we measured the expression of cell cycle regulating genes (p15, p16, p21 and p27) in proliferative phenotype cells. After vorinostat treatment we found up-regulation of some of these tumor suppressors, suggesting that HDAC inhibitor treatment counteracts loss of cell cycle control in melanoma cells and may therefore offer a therapeutic strategy in melanoma treatment.

P157

Re-expression of epigenetically silenced miRNAs is associated with anti-tumor effects on melanoma cells

T. Nishizaka
Sapporo Medical University School of Medicine, Sapporo, Japan

Melanoma is an extremely high-grade skin cancer and resistant to many chemotherapeutic agents. MicroRNAs (miRNAs) are small non-coding RNAs that regulate gene expression at the posttranscriptional level. Recently, a number of studies have revealed altered expression of miRNAs in human malignancies, although their roles in melanoma are not yet fully understood. The aim of this study are to examine one is about the combination treatment effect of DNA demethylating agent 5-aza-2′-deoxycitidine (5-aza-dC) with interferon (IFN)-beta in melanoma cells and the other is about involvement in the anti-tumor effect. The combination of 5-aza-dC and IFN-β synergistically suppressed proliferation of multiple melanoma cell lines. TaqMan Low Density Array analysis revealed that the drug treatment induced upregulation of 23 miRNAs (greater than five-fold) in melanoma cell line TXM18. Six out of these miRNAs exhibited CpG islands in their proximal upstream. Through methylation-specific PCR (MSP) and bisulfite pyrosequencing, we observed that the miR-596 CpG island is frequently methylated in multiple melanoma cell lines, including TXM18 and primary melanoma tissues, whereas the methylation was limited in normal melanocytes. Ectopic expression of miR-596 inhibited the proliferation of melanoma cell lines. Our data suggest that miR-596 may be involved in the melanoma development and that re-expression of the silenced miRNAs could be an effective therapeutic strategy.

P158

CpG island methylation and gene silencing in melanoma

Y. Arroyo-Berdugo, P. Cheng, S. Alonso, K. S. Hoek, M. D. Boyano
University of the Basque Country, Leioa, Spain

Melanocytes can give rise to a variety of benign and malignant neoplasms that differ in their genomic and proteomic profiles. The identification of suitable biomarkers would be useful to improve clinical diagnosis and treatment options for melanoma patients. We examined differences in protein expression between melanoma and melanocytes by 2D-PAGE, studied mRNA transcriptional levels by RT-PCR and used bisulfite sequencing to assess whether CpG island methylation states correlated with observed expression differences. We found highly significant differences in the expression of some proteins between melanomas and melanocytes. For most of these mRNA and protein levels were correlated indicating that down-regulation occurs at the RNA level. We identified differential CpG island methylation but this was not always associated with down-regulation, nor did down-regulation necessarily correlated with CpG methylation. The proteins identified to be down-regulated in melanoma have known roles in survival, proliferation and apoptosis. While CpG island methylation of promoters may contribute to transcriptional deregulation of some gene, our results indicate that there are likely to be other factors contributing to gene silencing.

P159

DNA methylation patterns in melanoma phenotype switching

P. Cheng, D. Widmer, O. Eichhoff, B. Belloni, R. Dummer, K. S. Hoek
University Zurich Hospital, Zürich, Switzerland

Our group recently described the phenotype switching model for melanoma progression, where we postulate that melanoma switches between proliferative and invasive phenotypes depending upon microenvironmental pressures. These two phenotypes are distinguishable by gene expression and phenotypic characteristics. However the molecular mechanisms of regulating their molecular plasticity are currently unknown. To investigate the role of DNA methylation on melanoma cell plasticity, we performed DNA methylation profiling of ten melanoma cell cultures established from seven patient melanoma biopsies by methylated DNA immunoprecipitation microarray analysis using a Nimblegen CpG promoter array. The DNA methylation profile of

 © 2011 The Authors. Journal Compilation © 2011 John Wiley & Sons A/S

the ten melanoma cell cultures could be distinguished into two distinct cohorts. Preliminary analysis of this dataset identified a number of genes involved in the WNT signaling pathway such as WNT5A, TCF4, and FZD2 to be differentially methylated between the two cohorts. WNT5A, TCF4, and FZD2 expression levels of these genes correlated with the methylation status of the promoter and are also differentially expressed between the proliferative and invasive phenotypes. These results suggest that DNA methylation might be a mechanism which regulates WNT signaling in melanoma phenotype switching.

P160

Eukaryotic initiation factor eIF2-alpha in melanoma

P. Zanna, I. Maida, C. Grieco, S. Guida, N. Cassano, G. A. Vena, A. Naspi, P. Londei
Università degli Studi di Bari, Bari, Italy

The differentiation, proliferation and survival of melanocytes and melanoma cells are controlled at various levels. Recently, the importance of translational regulation in promoting and sustaining tumorigenesis is being increasingly recognized. The expression of certain translational factors transiently increases in normal cells in response to growth factors and is constitutively upregulated in tumor cells. Moreover, post-translational modifications such as phosphorylation play a crucial role in modulating protein synthesis. The initiation factor IF2 is a central regulator of translation: phosphorylation of IF2 on its alpha subunit inhibits protein synthesis. Activation of eIF2 kinases in response to stress or tumoral transformation has been reported; increased phosphorylation of eIF2 alpha has been correlated with a metastatic phenotype in some kinds of tumors. Phosphorylated ERK levels can be used as marker of cell growth in the actively proliferating cells. The aim of this study is to investigate whether peIF2-alpha and pERK levels are reliable markers of tumorigenic potential in different melanoma cell lines, and to get insight into the mechanisms whereby eIF2-alpha phosphorylation may modulate cellular transformation. Cell lines were obtained from melanoma surgical specimens. For Western blot analyses the antieIF2-alpha and peIF2-alpha, were purchased from Cell Signalling Technologies; the anti ERK and pERK were purchased from Santa Cruz. Confocal analysis was performed as described in Zanna et al., 2008. Our melanoma cell lines, derived from metastatic or primary cutaneous melanoma lesions, showed different levels of phosphorylated eIF2alpha and phosphorylated ERK, which were higher in the cell lines derived from metastatic tumours as compared to primary melanoma cell lines. We show that after treatment with PD98059, a MEK inhibitor, ERK phosphorylation was inhibited while eIF2-alpha phosphorylation was enhanced in all melanoma cell lines. Moreover Western blotting and confocal analyses revealed that phosphorylated eIF2-alpha was localized in the nucleus of melanoma cell lines. Our results support a possible relationship between phosphorylation levels and subcellular localization of phosphorylated eIF2-alpha and cell proliferation in malignant melanoma. These studies could suggest a novel mechanism through which cells couple the regulation of protein synthesis with cell proliferation.

P161

Cyclic GMP-signaling associated gene expression in human melanoma cells in altered gravity: down-regulation in simulated weightlessness

K. Ivanova, P. Eiermann, W. Tsiockas, I. Block, R. Hemmersbach, R. Gerzer
DLR, Institute of Aerospace Medicine, Cologne, Germany

Cell functions are known to be influenced by gravity alterations (hyper- and microgravity). In a previous study we have reported that hypergravity created by horizontal centrifugal accelerations (up to 5 g for 24 h) may stimulate guanosine 3′,5′-cyclic monophosphate (cyclic GMP or cGMP) efflux and pigmentation in melanocytes and non-metastatic melanoma cells, but not in metastatic phenotypes under conditions of limited degradation (phosphodiesterase inhibitors) or stimulated cGMP synthesis (nitric oxide, NO). The cGMP synthesis is catalyzed by different guanylyl cyclase (GC) isoforms. For metastatic melanoma cells, we have found absence of NO-sensitive soluble guanylyl cyclase (sGC), a heterodimeric (alpha/beta) hemeprotein, whereas activities of the membrane-bound isoforms (GC-A and GC-B) were up-regulated. Moreover, we recently have shown that simulated microgravity down-regulates GC-A and GC-B mRNA levels in these cells. Here we report that the hypergravity-induced cGMP efflux, which is related to an increase in the mRNA levels of the multidrug resistance proteins 4 and 5 (MRP4/5) as selective cGMP exporters, depends on the presence of NO-sensitive sGC. In contrast, the exposure of the melanoma cells to simulated microgravity using a fast-rotating clinostat with one rotation axis (up to 0.0121 g for 24 h) induced down-regulation in the mRNA levels of NO synthases, MRP5, and tyrosinase between 20 and 50% in comparison to 1 g. The down-regulation of the mRNA levels was partly abrogated in cells transfected with siRNA for sGC-beta1 subunit, suggesting a role of sGC-cGMP pathway in gravity-dependent signaling. Our results support the finding of inverse effects of hypergravity and simulated microgravity on cells. Finally, the down-regulation of expression of cGMP-signaling associated genes, which have been implicated in cancer, may indicate alterations in the metastatic behavior of cells in real microgravity.

P162

Activity of organic anion transporting polypeptides (OATP) in melanoma cells generates a trans-resistance signal to cisplatin-induced cell death through glutathione and protein kinase C (PKC)-linked mechanisms

F. Silvy, D. Lombardo, P. Verrando
INSERM UMR 911, School of medicine Timone, University of Méditerranée, Marseilles, France

Many studies underscore the role of ABC efflux transporters in melanoma cell (MC) resistance to anti-neoplastic drugs, while very little is known concerning the contribution modalities of other transporters, such as the influx solute carriers belonging to the OATP/SLCO family. Since we characterized first the presence of OATPs in several MC, our aim was to search for their biologic roles. We found that they are involved in a trans-resistance signal to CisPt-induced (apoptotic) cell death. Cell cultures, apoptosis related assays, GSHi quantitation, OA uptake assay, immunoblots and signal transduction protocols. The trans-resistance signal to CisPt toxicity generated by organic anions (OA) triggering OATP activities coincides with a fast (2–4 h) depletion (~50%) of the intracellular reduced glutathione (GSHi) and does not involve the OAT/SLC22A transporters. It can be impaired by challenging the cells with a GSH permeant analogue. CisPt alone lowers GSHi levels only by 24 h, as a consequence of cell death signal, not similar to that one from OATPs. Inhibition of γ-glutamyl-cysteine synthase results in a slow decrease of GSHi level, excluding the enzyme as a mediator of OATP signal. Inhibition of ABCC/MRP transporters does not impair the protection signal. This suggests that they are not involved in GSHi exclusion and indicates indirectly that OATPs may be the GSHi exchangers. ERK-, p38- and PI3-kinase inhibition as well as a stimulation of adenylate cyclase do not affect the OATP signal, contrary to a stimulation of PKC. This finding originally discloses

© 2011 The Authors. Journal Compilation © 2011 John Wiley & Sons A/S

PKC as a mediator of OATP-induced trans-resistance to CisPt toxicity. OATP activity generates a trans-survival signal to CisPt-induced cell death that coincides with a fast depletion of GSHi (possibly through OATP exchange activity) and that involves PKC. Conclusively, beside the role played in cis by the ABC carriers, resistance of MMC to CisPt can be also explained by OATP trans-activities which should be considered in pharmacologic treatment strategies.

P163

Mechanisms controlling melanoma initiation and progression

D. Zingg, O. Shakhova, L. Sommer
Institute of Anatomy, University of Zurich, Zürich, Switzerland

During embryonic development, neural crest cells (NCC) originate at the margins of the neural tube and, after a phase of migration, settle down in different parts of the body to contribute to the formation of a plethora of cell types, including melanocytes in the skin. In mice, NC-derived stem cells persist into adulthood in many of their post-migratory targets. A prominent example are melanocyte stem cells (MSC) that localize to the bulge, the stem cell niche of the hair follicle, where they self-renew and give rise to mature melanocytes that transfer pigment to the growing hair. Previously, we have described molecular pathways that are of great importance for the development and survival of embryonic NCCs. It has become evident that these molecular key players also function in homeostasis of mature NC-derived tissues and might play a role in disease of NC-derived tissue. Accordingly, molecular pathways regulating embryonic NC development are conceivably involved in the formation of melanoma, a cancer that arises from the melanocytic lineage. To address this issue, we make use of the Tyr::N-RasQ61K INK4a–/– mouse that develops melanoma within 6 month. In this melanoma model, we study tumor initiation and progression in vivo upon conditional knockout of genes described before to play a role in embryonic NC and the melanocytic lineage. To complement our in vivo studies, we established FACS-isolation of melanocytic cells from Tyr::N-RasQ61K INK4a–/– mice, which allows us to investigate cellular and molecular mechanisms underlying the in vivo phenotype. With these studies we intend to gain knowledge about the processes that control melanoma initiation and progression.

P164

Pre-treatment of melanoma cells with a first protein kinase inhibitor sensitizes cells to a second protein kinase inhibitor: a rationale to combine targeted drugs

M. L. Fontsa, M. Wiedig, R. Morandini, F. Sales, A. Awada, G. Ghanem, F. Journe
Institut Bordet, Brussels, Belgium

Targeted therapy (e.g. RG7204/PLX4032) has provided evidences of effectiveness in melanoma. However, after a period of efficacy, resistance may appear, probably due to crosstalks between different signalling pathways. It becomes therefore evident that more effective treatment needs concomitant inhibition multiple pathways. The aim of our study is to evaluate the possible additive and/or synergistic effect on cell proliferation of various inhibitor combinations targeting important signalling pathways in melanoma. Our model comprises three particular cell lines with V600EBRAF (MM074), Q61LNRAS (MM057) and WTBRAF/WTNRAS (HBL). We exposed each of these lines to doubling concentrations of specific protein kinase inhibitors targeting SRC (PP2), MEK (U0126), PI3K (LY294002) and JAK (pyridine 6) according to the three following conditions: (i) cells were exposed for 3 days to each of the inhibitors; (ii) cells were exposed for 3 days to a combination of 2 different inhibitors and (iii) cells were exposed for 7 days to each of the inhibitors and, while maintaining this pre-treatment, cells were incubated for three additional days with each of the other inhibitors. We used cell proliferation and phosphorylation levels of key proteins as end points. By comparing IC50s, cell exposure to a single inhibitor (condition 1) showed that both WTBRAF cells were 15 fold more sensitive to a SRC inhibition while V600EBRAF cells were 30 fold more sensitive to a MEK inhibitor, confirming that the latter cells are largely dependent on the MAPK pathway for growth. Concomitant treatment (condition 2) revealed antagonism between SRC and JAK inhibitors as compared to each inhibitor alone in all three cell lines. This can be explained as both SRC and JAK may stimulate the STAT pathway. By contrast, pre-treatment (condition 3) with MEK or PI3K inhibitors showed additive/synergistic effects when combined to a SRC inhibitor in both WTBRAF cells, suggesting that MEK or PI3K inhibition could activate the SRC/STAT compensatory signalling pathway. Pre-treatment with a single targeted inhibitor affecting a crucial signalling pathway in melanoma cells may render them more dependent on alternative pathways that should subsequently be inhibited to potentiate efficacy. By contrast, simultaneous combinations of inhibitors failed to produce such effect, further supporting the importance of both duration of cell exposure and their sequential use

P165

Involvement of autophagy in the apoptosis induced by Terfenadine, an H1 histamine receptor antagonist, in human melanoma cells

E. Alonso-Tejerina, F. Nicolau-Galmés, Y. Arroyo-Berdugo, G. Pérez-Yarza, A. Asumendi, M. D. Boyano
University of the Basque Country, Leioa, Spain

Previously we demonstrated that terfenadine acts as a potent apoptosis inducer in melanoma cells. Deeping on the apoptotic mechanisms activated by terfenadine we found that terfenadine induced autophagy. The aim of this study was to know the role of the autophagy in the cell death induced by terfenadine. Cells treated with terfenadine for 8 h were observed by Electron microscopy. LC3B-II protein expression, wich is localized on the autophagic vacuoles, was analyzed by Western Blot. Intracellular ROS levels were measured by flow cytometry after labeling cells with carboxi-H2DCFDA. Finally, to investigate how autophagy inhibition could affect terfenadine-induced cell death, we down-regulated Atg-7 by siRNA transfection assays. Seventy two hours after transfection, cell lysates were obtained to verify the Atg7 protein knockdown by Western Blotting. The viability experiments in the presence of terfenadine were performed by XTT assay after 48 h of transfection. Electron microscopy examination revealed a massive vacuolization of the cytoplasm and autophagic vacuoles of multiple membranes. Western Blotting analysis revealed that terfenadine could rapidly elicit accumulation of LC3B-II. ROS production assays demonstrated that autophagy triggered by terfenadine in a ROS-dependent or ROS-independent manner according to the culture conditions. Moreover, Atg 7 down-regulated using siRNA assays partially prevented cell death induced by terfenadine at initial times of treatment, in both A375 and HT144 cells, but not when terfenadine had finally killed most of the cells. These results indicate that autophagy acts by promoting cell death in terfenadine-treated melanoma cells. This study has been supported by grants from the University of the Basque Country (GIC 07/25-IT-423-07 and the Healthy Department of the Basque Country).

 © 2011 The Authors. Journal Compilation © 2011 John Wiley & Sons A/S

Erika Alonso-Tejerina was supported by a research fellowship from the Gangoiti Foundation.

P166

Subtilisin-kexin isoenzyme-1-a novel player in melanoma biology

N. Weiß, A. Kokot, T. A. Luger, C. Weishaupt, M. Böhm
Hautklinik Münster, Germany

Prohormone convertases (PCs) are Ca2+-dependent serine proteases which do not only process prohormones into biologically active peptides but also activate cancer-related proteins such as growth factors, growth factor receptors, adhesion molecules and matrix metalloproteases. Although it has previously been reported that some PCs are overexpressed in a number of solid tumors, their function in human pigment cells is poorly investigated. In order to clarify if PCs are involved in the pathogenesis of melanoma we focused on subtilisin-kexin isoenzyme-1 (SKI-1), one of the most recently identified PC members. In vitro expression of SKI-1 in normal and transformed human melanocytes (NHM) as well as in nine human melanoma cell lines was determined by quantitative real-time RT-PCR and Western immunoblotting. Regulation of SKI-1 mRNA was examined in NHM exposed to a panel of established natural and artificial growth factors, extracellular and intracellular stressors. Regulation of SKI-1 was further investigated by in silico promoter analysis. Finally, the biological function of SKI-1 was tested by treating normal and transformed melanocytes with decanoyl (dec)-RRLL-chloromethylketone (CMK), a cell-permeable pharmacological SKI-1 inhibitor. Metabolic activity, cell survival and apoptosis were assessed by XTT test, crystal violet test and various apoptosis read-outs. SKI-1 was found to be constitutively expressed at mRNA and protein level in both normal and transformed melanocytes. In silico promoter analysis revealed several transcription factor binding sites for transcription factors that are typically activated by melanocytes mitogens as well as by extracellular and intracellular stressors. However, among several growth factors and stimuli tested, only phorbol-12-myristate-13-acetate and tunicamycin affected SKI-1 expression in NHM. Interestingly, dec-RRLL-CMK led to a dose-dependent inhibition of the metabolic activity and proliferation of normal and transformed melanoma cells in vitro. In general, melanoma cells appeared to be more sensitive towards the SKI-1 inhibitor. Moreover, dec-RRLL-CMK dose-dependently led to apoptosis of melanoma cells as shown by cell death detection assay, Annexin-V staining and processing of poly-adenosine diphosphate-ribose polymerase 1/2. This effect was associated with suppression of caveolin-1 and glucose-regulated-protein 78, two prototypical SKI-1 target genes implicated in melanoma growth and progress.

P167

Role of myc in melanoma

A. Marzia, I. Pshenichnaya, A. Trumpp, L. Larue, S. Gallagher, F. Beermann, F. Radtke
EPFL, Lausanne, Lausanne

Malignant melanoma is one of the most highly invasive and metastatic tumors. Over the past years significant advances in understanding the cancer biology of melanoma have been achieved, but the molecular mechanisms involved in its development are still incompletely established. c-myc is an oncogenic transcription factor that is frequently upregulated in human malignancies including melanoma. We recently addressed the role of c-myc in the melanocyte differentiation during normal development. Mice conditionally deleted for c-myc in pigmented cells (Tyr::Cre, c-myc flox/flox) display a hair graying phenotype due to a reduction in melanocyte/melanoblast number during embryonic development. Interestingly, c-myc inactivation does not affect melanocytes differentiation and/or function. We therefore decided to address the role of c-myc in melanoma formation and maintenance. We made use of lentiviral constructs to block c-myc expression in the B16F1 established melanoma cell line and in primary melanoma cells isolated from Tyr::NrasQ61K mice. In both cases, shRNA-mediated inhibition of c-myc resulted in growth arrest and induction of differentiation. We then crossed conditional c-myc knockouts (Tyr::Cre, c-myc flox/flox) with mice spontaneously developing melanoma (Tyr::NrasQ61K, p16–/–). Preliminary analysis revealed proper melanoblast migration and/or survival but severe hair-graying and impaired melanocyte differentiation in Tyr::N-RasQ61K, p16–/–, Tyr::Cre, cMycflox/flox mice. We are currently monitoring these animals for spontaneous melanoma formation. Moreover, we plan to evaluate the effect of c-myc deletion on melanoma formation in the context of chemically-induced carcinogenesis. Finally, we want to generate an inducible c-myc conditional knockout melanoma mouse (Tyr::N-RasQ61K, p16–/–, Tyr::Cre-ERT2, cMycflox/flox) to study the in-vivo role of c-myc in melanoma maintenance and survival.

P168

MAPK inhibitors may reverse the senescence-like phenotype associated with a low proliferation index of melanoma cells bearing the V600EBRAF mutation

M. Krayem, M. Berehab, M. Wiedig, R. Morandini, F. Sales, A. Awada, F. Journe, G. Ghanem
Institut Jules Bordet, Brussels, Belgium

The mutations of BRAF are described in about 50% of melanoma tumors with 90% of these mutations occurring at a single site, leading to the V600E substitution. This mutation leads to the constitutive activation of the MAPK pathway which is critical for cancer cell survival. We evaluated the impact of V600EBRAF mutation on melanoma cell proliferation and response to various MAPK inhibitors. We compared three V600EBRAF-mutated (MM032, MM043, MM074) with three wildtype (WT) melanoma cell lines (HBL, LND1, MM079) addressing: (i). ERK phosphorylation (Western blotting); (ii) cell proliferation (crystal violet staining); (iii) apoptosis (annexin V); (iv) senescence (beta-galactosidase activity) and (v) response to a MEK inhibitor (U0126) in terms of proliferation and senescence. First, we confirm that mutated cells exhibited significantly higher levels of ERK1/2 phosphorylation. But, surprisingly, these cells had a 2.5 fold lower proliferation index (d3/d1 ratio) as compared to the group of WT cells. We also found no significant difference in apoptotic cells between the two groups, but mutated cells expressed a higher level of the cell cycle inhibitor p21WAF, indicating that the lower growth rate of these cells was rather due to a reduced cell cycling and not to an increased cell death. Moreover, we observed that beta-galactosidase activity was extremely high in mutated cells along with significant increase both in cell size (two-fold) and in p53 expression, supporting that constitutive MAPK hyper-activation induces a senescence-like phenotype. Furthermore, we interfered with the MAPK signaling pathway by long-term exposure of mutated cells to non toxic but effective concentrations of various inhibitors, and we found, e.g. with a MEK inhibitor (U0126), that both beta-galactosidase activity and cell volume significant decrease together with an increase of the proliferation index. We confirm that in V600EBRAF melanoma cells, the hyper-activation of ERK leads to a senescence-like phenotype associated with a low proliferation. We also report that long-term (from 10 to 17 days in our study) inhibition of MAPK signaling pathway may reverse the senescence leading to a stimulation of cell proliferation. Therefore, the

© 2011 The Authors. Journal Compilation © 2011 John Wiley & Sons A/S

MAPK pathway inhibitors currently tested for the treatment of melanoma might promote proliferation in cells with high MAPK activity, especially in V600EBRAF mutated ones.

P169

Serum Amyloid A, Clusterin and Apolipoprotein A-I serum levels related to metastatic progression in melanoma patients

I. Ortega-Martínez, J. Gardeazabal, R. Fernandez-Suarez, E. Alonso-Tejerina, J. M. Careaga, J. L. Díaz-Ramón, R. Izu, A. Asumen, M. D. Boyano
University of the Basque Country, Leioa, Spain

Biomarkers are important tools for cancer detection and monitoring. The identification of novel biomarkers in the early phases of the cutaneous melanoma will be useful for the prognosis and the understanding of the malignant progression of melanoma. We compared the proteomic profiles of sera obtained from melanoma patients with good prognosis (10 yr disease-free after surgery of the primary tumor) and patients who developed metastasis during the first 2 yr after surgery. Serum samples were obtained from peripheral blood of patients and were stored at −80°C until use. We applied 2D electrophoresis analysis, after enrichment of low-abundance serum proteins and reduction of high-abundance proteins in serum samples by ProteoMiner kit. Gels were stained with SYPRO and analyzed with Progenesis SameSpots software. In the image analysis we found 21 spots differentially expressed ($P < 0.05$) with a change of volume bigger than 60%. A total of 44 proteins were identified by HPLC-MS/MS. These proteins are implicated in different biologic processes mainly immune and inflammatory response and minor proportion in cell adhesion, metabolism, apoptosis and signal transduction. Among the differentially expressed proteins, three apolipoproteins as Serum Amyloid A (SAA), Clusterin (CLU) and Apolipoprotein A-I (APO A-I) have been selected for validation by ELISA assay in 101 healthy controls and 334 melanoma patients dividing in a group including patients that remained disease free during 10 yr of follow-up and a group of patients that developed metastasis. Despite the fact that the melanoma patients were in an early state of disease at diagnosis moment (I, II and III stages according to the AJCC), 83 developed metastasis during follow-up. Mean values of SAA, CLU and APO A1 in melanoma patients were significantly higher than in controls.

P170

GRM1: a therapeutic target in melanoma

J. Wangari-Talbot, B. A. Wall, J. Goydos, S. Chen
Rutgers University, Piscataway, USA

Malignant melanoma accounts for approximately 5% of skin cancer incidences but accounts for >80% of skin cancer related deaths. Metabotropic glutamate receptor 1 (Grm1) is one of the genes implicated in melanoma. Ectopic expression of Grm1 in mouse melanocytes has been shown to induce melanocytic lesions in transgenic mice and tumorigenicity in-vivo in mouse allografts. Additionally, GRM1 expression has been demonstrated in human melanoma cell lines and biopsy samples. Here we investigate whether GRM1 is essential in the maintenance of a melanoma phenotype and whether combination therapies that include a GRM1 signaling inhibitor are viable. We investigated si-RNA mediated down-regulation of GRM1 in a malignant melanoma cell line. Utilizing a Ponasterone-A inducible gene expression system, we showed in western immunoblots suppressed GRM1 protein expression, reduced activation of the MAPK signaling pathway, reduction in in-vitro cell proliferation, reduction in in-vivo tumor progression and induction of apoptosis in a xenograft model. We have identified Riluzole, a glutamate release inhibitor as a drug that has cytotoxicity against GRM1 positive melanomas.
Given that a majority of melanomas harbor an activating BRAF V600E mutation, we investigate the utility of Sorafenib and PLX 4720 combined with Riluzole in-vitro and in-vivo. Our results show that the combination of Riluzole and Sorafenib has synergistic or additive anti-proliferative activity against a variety of GRM1 positive melanoma cell lines and has potential clinical translatability. Our results support ongoing therapeutic targeting of GRM1 in melanoma patients.

P171

Paired basic Amino-acid-Cleaving Enzyme 4 (PACE4) increases metabolic activity, proliferation, migration and collagenase expression of human melanoma cells in vitro and confers increased subcutaneous tumor growth in vivo

M. Böhm, A. Mastrofrancesco, N. Weiss, B. Kemper, G. Von Bally, M. Picardo, T. A. Luger, C. Weishaupt
Department of Dermatology, Münster, Germany

Proprotein/prohormone convertases (PCs) are mostly known in cutaneus and pigment cell biology for their important role in processing of proopiomelanocortin to melanocortin peptides such as alpha-melanocyte-stimulating hormone. There is increasing evidence however that PCs are also involved in the pathogenesis of some solid tumors. We investigated the in vitro and in vivo effects of selective overexpression of the PC member Paired basic Amino-acid-Cleaving Enzyme 4 (PACE4) in human melanoma cells in vitro and in vivo. SK-Mel-30 melnaoma cells expressing low endogenous levels of PACE4 were stably transfected with rPACE4. The PACE4 transfectants secreted high amounts of rPACE4 in vitro, had elevated protein levels of matrix metalloproteinase 1 and 2, displayed increased cell motility as shown by digital holographic microscopy in collagen as well as enhanced invasiveness demonstrated in Matrigel migration assays. Moreover, PACE4 transfectants but not vector-alone transfected melanoma cells exhibited increased metabolic activity and cell proliferation but did not show any change in melanin content. In vivo, subcutaneous injection of PACE4 transfectants into immunodeficient SCID Hairless Outbred mice resulted in significantly increased local tumor growth compared with injected control cells. However, ectopic expression did not enable transfectants to metastasize. To assess the relevance of these findings we examined the endogenous expression of PACE4 in human melanoma cell lines derived from different stages of disease as well as in human melanoma specimens ex vivo. In six out of nine melanoma cell lines, mRNA expression levels were significantly elevated compared with normal human melanocytes. Immunohistochemical analysis further revealed that PACE4 expression is detectable within melanoma cells in 89% of 47 tumor samples with the highest immunoreactivity in primary melanomas. Our findings highlight PACE4 as a novel player in melanoma biology and point towards novel future strategies for treatment of melanoma. As local invasion properties are enhanced by PACE4 expression we attribute its function to the early phase of melanoma progression. Further studies have to evaluate in what extent inhibition of PACE4 can reduce tumor growth and invasion.

 © 2011 The Authors. Journal Compilation © 2011 John Wiley & Sons A/S

P172

Inhibition of melanogenesis increases the efficacy of photodynamic therapy-induced cell death in melanoma cells

L. M. Davids, K. V. Sharma
Department of Human Biology, University of Cape Town Medical School, Cape Town, South Africa

Melanoma is the main cause of death in skin cancers. Despite early detection, resection and post-operative therapy, combating melanoma remains ineffective and investigations into other forms of adjuvant therapy such as photodynamic therapy (PDT) are prudent. This study proposes that depigmentation i.e. the removal of the free radical scavenging pigment, melanin, in melanotic melanoma cells increases their susceptibility to PDT-induced cell death. Two human melanoma cell lines: one pigmented (Mel-1) and one amelanotic (A375) (non-pigmented control) cell lines were used. Kojic acid (KA), a tyrosinase-specific inhibitor, was shown to inhibit melanin synthesis after a 3-day exposure. Cells were then treated with the photosensitizer hypericin (HYP) followed by PDT and cell viability measured. Intracellular reactive oxygen species (ROS) was also measured. Apoptosis cell death was measured by caspase 3/7 activity after 4 h of PDT activation. PDT on KA-treated depigmented cells resulted in a 3.82 fold increase in ROS production which correlated to 11% increase in cell death susceptibility over pigmented control cells. Moreover, cells allowed to regain their pigment failed to return to normal even after 72 h, proving the sustained effectiveness of PDT. Using a DPPH* free radical assay, the results confirmed the scavenging properties of melanin (IC50 = 18.30 μg/ml) proving that this pigment may be one of the reasons for melanoma chemoresistance. There was no significant difference observed in the caspase 3/7 activity in KA treated melanoma after PDT which suggests that the HYP-PDT treatment induced a caspase-independent cell death mechanism. Overall this study shows that melanin can act as a radical scavenger in melanoma cells to neutralise treatments which produce ROS. Moreover, it highlights that removal of pigment or inhibition of melanogenesis in melanoma increases the efficacy of adjunctive PDT treatment.

© 2011 The Authors. Journal Compilation © 2011 John Wiley & Sons A/S
