# Supplementary material for: Therapeutic efficacy of cell-based therapy in vitiligo: a research letter systematically reviewed using meta-analysis
Source: Arch Dermatol Res. 2024 May 22;316(5):198. doi: 10.1007/s00403-024-02920-6 (PMC11111487; doi:10.1007/s00403-024-02920-6)
Supplement: Supplementary file 1 — Supplementary file1 (ZIP 24195 KB) [file 403_2024_2920_MOESM1_ESM.zip › Studies were included/Budania 2014.pdf]

Commonwealth Session Orals

CS01
Childhood leprosy in the postleprosy elimination era: a retrospective analysis of epidemiological and clinical characteristics of disease over 11 years from a tertiary care hospital in North India

S. Dogra,[1] T. Narang,[2] G. Khullar,[1] R. Kumar[1] and U. Saikia[2]

[1]*Department of Dermatology and* [2]*Department of Histopathology, Post Graduate Institute of Medical Education and Research, Chandigarh, India*

Childhood leprosy reflects disease transmission in the community as well as the efficiency of ongoing disease control programmes (Dayal R, Hashmi NA, Mathur PP, Prasad R. Leprosy in childhood. *Indian Pediatr* 1990; **27**: 170–80). The aim of this study was to analyse the epidemiological and clinical trends of childhood cases of leprosy at our tertiary care hospital during 2001–11. A retrospective study was undertaken analysing the clinic records of children with leprosy aged ≤ 18 years registered at the leprosy clinic of our institute over an 11-year period. Demographic and disease characteristics including age, sex, history of contact, duration of disease, clinical pattern, bacteriological and histopathological parameters, reactions and deformities were noted from a predesigned format. A total of 1225 cases of leprosy were registered during this period, of whom 59 (4.8%) were children. The mean age of the patients was 10.06 ± 3.35 years, with a male preponderance (3.9 : 1). History of close contact with a leprosy case was present in 15 (25%) of these children. The mean duration of illness before diagnosis was 18.5 months (range 1–70). Borderline tuberculoid was the most common clinical type of leprosy, in 40 children (68%), followed by lepromatous in seven (12%), borderline lepromatous in six (10%), pure neuritic in two (3%), and tuberculoid, midborderline, histoid and indeterminate in one (2%) each. Lesions were located over the upper extremity in 32 patients (54%), lower extremity in 29 (49%), face in 27 (46%) and trunk in 26 (44%). A single lesion was observed in 23 patients (39%), two to five lesions in 12 (20%), more than five lesions in 22 (37%) and only nerve involvement in two (3%). Slit-skin smear was positive in 17 patients (29%). Lepra reactions were observed in 20 patients (34%), of whom 14 (70%) had type 1 and six (30%) had type 2 reaction. Thickened peripheral nerve trunks were present in 48 children (81%). Neuritis occurred in nine patients (15%) and deformities (both grade 1 and 2) at the time of diagnosis were noted in 24 (41%). Overall 10% of children defaulted from treatment, and three cases (5%) of relapse were observed. Childhood leprosy and its complications continue to present in alarming numbers, suggesting possible gaps in the national programmes aimed at leprosy elimination. We stress the importance of continuous and sustained efforts for early case detection in the community in general, and close follow-up of susceptible children in household contact with leprosy cases in the postleprosy elimination era.

CS02
Do the active edge, centre or adjacent normal skin have better histological yield in the diagnosis of tuberculoid leprosy?

F. Srisaravanapavananthan,[1] K. Satgurunathan[2] and J. Fernando[2]

[1]*Teaching Hospital Jaffna, Jaffna, Sri Lanka and* [2]*National Hospital of Sri Lanka, Colombo, Sri Lanka*

Leprosy is still a burden to our healthcare system. Prevention of disability requires early detection and treatment. Clinical diagnosis of early forms of leprosy is challenging, as they require histopathological confirmation. The accuracy of the biopsy depends mainly on the site from where the biopsies are taken, among different sites in a lesion (Sina B, Kao GF, Deng AC, Gaspari AA. Skin biopsy for inflammatory and common neoplastic skin diseases: optimum time, best location and preferred techniques. A critical review. *J Cutan Pathol* 2009; **36**: 505–10). The aim of this study was to identify the optimum site of biopsy for a better diagnostic yield in early forms of tuberculoid leprosy. A hospital-based descriptive cross-sectional study was carried out in the dermatological clinic at a tertiary care hospital from September 2011 to January 2012. In total, 83 patients with clinical evidence of early tuberculoid lesions were enrolled. An interviewer-administered questionnaire was completed; monofilament and hot-water-filled test tubes were used for sensory examination; and punch biopsies were obtained at the lesion centre and active edge, and adjacent normal skin. Data were entered and monovariate and bivariate analyses were carried out using SPSS 17. Among 83 patients, 72 and 11 were clinically suspected as having tuberculoid leprosy (TT) and indeterminate leprosy (IL), respectively, and 76 (92%) were histologically confirmed. Among these 76 cases, 64 and 12 were confirmed as having TT and IL, respectively. Rates of histological diagnosis at the active edge ($n = 68$) were 56/64 (88%) for TT and 12/12 (100%) for IL. Similarly, TT and IL were diagnosed histologically at the centre ($n = 53$) in 46/64 (72%) and seven of 12 patients (58%), respectively. The observed differences between the proportions of histological diagnosis at the active edge and centre are statistically significant for TT and IL ($P < 0.05$). The sensitivities of histology for the diagnosis of TT at the active edge and centre were 88% and 72%, respectively. Among all the patients with TT ($n = 64$), 17 biopsies (27%)

© 2014 The Authors

were positive only at the active edge and seven (11%) only at the centre. A single biopsy was histologically positive in the adjacent normal skin. Although biopsy at the active edge is superior to that at the centre, considering the detrimental effect of missing the diagnosis of leprosy, a greater yield could be achieved by taking biopsies from both sites. If we were to take a single biopsy, the best site would be the active edge.

CS03
A prospective case series evaluating the efficacy and safety of itraconazole and potassium iodide in rhinofacial conidiobolomycosis

T. Narang,[1] M. Gupta,[2] R. Kaur[2] and A. Manhas[2]
[1]*Post Graduate Institute of Medical Education and Research, Chandigarh, India and* [2]*Gian Sagar Medical College & Hospital Ramnagar, Banur Dist., Patiala, Punjab, India*

Rhinofacial conidiobolomycosis (RFC), or rhinofacial zygomycosis or conidiobolomycosis, is an uncommon subcutaneous fungal infection of the tissues of the face and neck, producing painless swelling with grotesque deformity of the face. Although there are case reports and small case series from different parts of the world, there are very few prospective studies evaluating treatment and long-term follow-up from Northern India. In this prospective study we set out to evaluate the safety and efficacy of itraconazole (200 mg twice daily) and a saturated solution of potassium iodide (SSKI) in patients with RFC. Ten patients of RFC were studied over a span of 5 years in a tertiary care hospital in North India. Biopsy and microbiological studies were helpful in making the final diagnosis. *Conidiobolus* was cultured in four cases, and in the other six the histopathology was suggestive of rhinofacial conidiobolomycosis, although no organism could be cultured. These patients were started on itraconazole (200 mg twice daily) and SSKI, and were followed up for a minimum of 1 year after stopping treatment. The patients were aged 24–65 years, with a mean age of 38.7 years and a mean duration of disease of 1.78 years. They were predominantly male (9 : 1). Almost all of the cases had been to various specialists, and a few had also undergone nasal and sinus surgery. Seven patients responded to treatment with itraconazole and SSKI. Of these five had complete resolution and two had significant improvement (> 75%); however, in two patients the response was partial (< 25%) with regression of the swelling, and one patient did not show any response after 1 year of combination therapy. Rhinofacial conidiobolomycosis can present to both dermatologists and ear–nose–throat specialists; clinical awareness and histopathological/diagnostic mycological facilities are required to ascertain an early diagnosis. Long-term treatment with itraconazole and potassium iodide in adequate doses is helpful in the majority of the patients. However, some patients may require other treatments such as amphotericin-B or voriconazole. Surgery should be reserved for management of deformities.

CS04
Amyloidosis cutis dyschromica in two siblings and a review of the epidemiology, clinical features and management

C. Mahon, F. Oliver, D. Purvis and K. Agnew
Dermatology Department, Greenlane Clinical Centre, Auckland, New Zealand

Amyloidosis cutis dyschromica (ACD) is a rare form of primary cutaneous amyloidosis. Both familial and sporadic cases have been reported. There is a paucity of information in the dermatology literature about this condition to guide diagnosis and treatment. We present two new cases of ACD in adolescent siblings and summarize the epidemiology, clinical findings, natural history and treatments in 41 cases of ACD from the medical literature, in order to define this pigmentary disorder better. ACD is reported predominantly in patients of East Asian or South East Asian ethnicity (71% of all reported cases). Male and female patients are equally affected. Thirty-two familial and 11 sporadic cases of ACD were reviewed. The mean age of onset was 10 years (range 4–22) in the familial group, and 23 years (range 9–35) in the sporadic group. The average time between onset and diagnosis was 20 years in the familial group and 11 years in the sporadic group. The epidemiological and clinical characteristics of ACD in sporadic and familial cases do not otherwise differ substantially. There was no reported case with systemic involvement. Improvement in the dyschromica was noted in five of the seven cases reporting a response to acitretin. To our knowledge, this is the largest review of published cases and the first complete description of ACD as an entity. We describe both epidemiological and clinical features as well as the natural history of ACD, aspects of this disorder that have not previously been reported. Given the similarity of clinical features in familial and sporadic cases it is unlikely that these groups represent distinct disorders, a conclusion that may in the future be confirmed by the identification of a specific genetic abnormality for this condition. In ACD, routine investigation for systemic involvement is unnecessary. Acitretin may be a treatment option, although this requires further study.

CS05
Fibroscan monitoring for liver complications of methotrexate use in dermatology patients

A. Chandran, M. Rademaker, R. Vyas, A. Yung, E. Johns, R. Jurawan, J. Irwin and F. Weilert
Waikato District Health Board, Hamilton, New Zealand

Fibroscan is a noninvasive and validated technique used to assess liver fibrosis. The aim of this study was to assess its value in patients taking long-term methotrexate for a variety of dermatological indications. Baseline body mass index, waist circumference, serial procollagen-3 N-terminal peptide (P3NP), liver function tests, and current and cumulative methotrexate doses were recorded. Fibroscan measurement or transient elastography (TE), was graded as normal (F0/F1, TE 0–7), mild fibrosis (F1/F2, TE 7.1–9), moderate fibrosis (F2/F3, TE 9.1–12) or severe fibrosis/cirrhosis (F4, TE > 12). In total 133 patients taking methotrexate had fibroscans

© 2014 The Authors

performed; 33 were excluded due to inadequate imaging (mostly due to obesity), 56% were female and the mean age was 54 years. Indications for methotrexate were psoriasis (63), eczema (24), nodular prurigo (five), lichen planus (four) and one each of chronic discoid lupus erythematosus, localized scleroderma, chronic actinic dermatitis and Behçet syndrome. The mean weekly dose of methotrexate was 14.1 mg, the mean cumulative dose was 1.95 g and the mean duration of therapy was 28.6 months. Overall 80% of patients had normal fibroscans (F0/F1); 13% had mild fibrosis (F1/F2), 3% moderate fibrosis (F2/F3) and 2% had severe fibrosis/cirrhosis (F4). Fifteen of the 80 patients (19%) who were F0/F1, five of 13 (38%) of the F1/F2 patients and 100% of F2/F3/F4 patients had abnormal P3NP levels. One patient with severe fibrosis (F4, TE 14.3) went on to have a liver biopsy that showed features of fibrosis likely due to methotrexate. In conclusion, although the number of abnormal fibroscans was low, fibroscan with P3NP appears to be a useful risk stratification tool for liver damage in dermatology patients on long-term methotrexate.

CS06
A prospective study of childhood leprosy from 2001 to 2014

J. Seneviratne, J. Akarawita, N. Madarasinghe and C. Siriwardana

Lady Ridgeway Hospital for Children, Colombo, Sri Lanka

Leprosy is a chronic infection caused by *Mycobacterium leprae*, which affects children only rarely. Childhood cases indicate ongoing transmission in the population. A dearth of prospective studies on childhood leprosy made us look at clinical features, treatment outcomes, reactions, impact of multidrug therapy (MDT) on transmission, and screening of contacts. All children with leprosy attending the skin clinic of a premier hospital for children were included over the study period. Out of 209 524 new visits, 443 patients (0.21%) were diagnosed as having leprosy. The number of new cases per year was 22–45, without a tendency to decline over the years. Overall 51% were girls, 16% were aged 2–5 years and 84% were aged 6–14 years. Some 67% had one lesion, while 5% had > 10 lesions at presentation. Only 1% had visible deformities, while 50% of patients presented within 6 months of first noticing the lesions, and 73% within 1 year. A significant 13% presented after > 2 years. Based on the Ridley–Joplin classification, 68% of patients were tuberculoid and 9% were borderline tuberculoid; 7% were lepromatous. Out of 85 subjected to biopsy, 80% showed good clinicopathological correlation. In total 86% of patients received paucibacillary treatment and 14% received multibacillary treatment, as per the World Health Organization guidelines. While on treatment 2% developed type I reaction and 1.1% developed type II reaction; 2% developed drug reactions, half of which were due to dapsone hypersensitivity. In 2011, 47 patients who completed treatment were reviewed for outcome. Lesions had completely resolved in 74% patients while 2% had active disease. There was residual numbness/weakness in 19%, while 4% had atrophic scaring. On questioning, 5% of patients had a known leprosy contact. This is in contrast to screening of contacts. A total of 311 contacts of 100 index cases were screened from January 2007 onwards; 51 had features of leprosy. Some 33% of index cases had positive contact within the household, and 11% had more than one family member affected; 83% of positive contacts were of tuberculoid type, and 21% of contacts were < 15 years old. This study highlights a number of interesting features. The majority of children affected are schoolchildren. A significant number were late to present. Screening of contacts, especially family members, is a useful method of identifying undetected patients. Although rare, dapsone hypersensitivity remains the main drug side-effect. Compared with adults, both type I and type II reactions are rare in children. Even after 32 years since introducing MDT treatment, the number of new cases detected per year remains the same. This is possibly due to the long incubation period of the disease.

CS07
Leprosy controversies: in therapy and its elimination

K. Bhushan

Post Graduate Institute of Medical Education and Research, Chandigarh, India

Introduction of effective antimicrobial treatment of leprosy with dapsone in 1940 was a major scientific development of the 20th century. Monotherapy had two distinct disadvantages: dapsone resistance and associated relapse, and continued morbidity and development of deformity due to prolonged activity of the disease. The problem was soon settled by introduction of multidrug therapy (MDT). Another landmark was classification of the disease into paucibacillary (PB) and multibacillary (MB). Subsequently, with an increasing number of patients requiring very long treatment to achieve smear negativity, and the logistics involved in drug delivery and repeated slit-skin smears (SSSs), a fixed duration of therapy of 24 months was recommended. In 1998 the World Health Organization (WHO) recommended reducing MB therapy to 1 year. Although relapses after 2 years of MDT were few, some workers had reported an incidence of up to 20%. In 2002 the WHO suggested further reduction of treatment to 6 months (three drugs for both PB and MB for 6 months), which fortunately did not find favour with the majority of specialists. Simultaneously with changing drug schedules, a change in the classification of the disease spectrum also occurred. In 1982 the classification of PB and MB disease was based on the degree of SSS positivity; in 1988 all smear-positive cases became MB. By 1995 the classification was based on the number of lesions. The number of nerves involved or the extent and distribution of disease was not considered. This was to obviate the need for SSS and skin biopsies. The ultimate criterion of success of any therapeutic regimen lies in the prevention of relapse, residual activity in the lesions at the end of therapy, and a fall in bacteriological index, disability and reactions. Because of the unchanged occurrence of cases (230 000 cases yearly worldwide) the focus is now on very short-course chemotherapy combining two or more bactericidal

© 2014 The Authors

drugs given over 28 days. Advantages of the shortened regimen need to be balanced against the risk of relapse. The 44th world assembly in 1991 declared that leprosy would be eliminated by 2000 as a public health problem (< 1 case per 10 000 population). To achieve this deadline at the national level, political pressure was enormous, and by 2004 elimination in 122 countries was claimed. However, this claim may be real or injudicious, because it resulted from redefinition of leprosy cases, cleaning of leprosy registers, leprosy classification and shortened duration of MB treatment. Reduction of MB therapy from 2 to 1 year diminished the number of cases by half, and further reduction in duration to 6 months again halved this number. A patient given single- or full-dose therapy was considered as treated/cured, so struck off the register. The ideal combination of drugs, their dose and duration for MB disease, and chemo-/immunoprophylaxis are still not well known. Our primary responsibility is to treat leprosy and not to attain numerical goals: we know that targets disrupt the focus of services.

CS08

A randomized comparative study between autologous noncultured epidermal cell suspension, suction blister epidermal grafting and split-thickness skin grafting: a quest to find a first-line surgical modality for the treatment of stable vitiligo

A. Budania,[1] D. Parsad,[2] S. Dogra[2] and N. Khunger[1]

[1]Vardhman Mahavir Medical College and Safdarjung Hospital, New Delhi, India and [2]Post Graduate Institute of Medical Education and Research, Chandigarh, India

Vitiligo is an acquired disorder of pigmentation caused by loss of epidermal melanocytes. Autologous noncultured epidermal cell suspension (NCES; a cellular grafting technique), suction blister epidermal grafting (SBEG) and split-thickness skin grafting (STSG; both tissue grafting techniques) are important established surgical modalities for the treatment of stable vitiligo (Njoo MD, Westerhof W, Bos JD, Bossuyt PM. A systematic review of autologous transplantation methods in vitiligo. *Arch Dermatol* 1998; **134**: 1543–9). We compared the three techniques for producing repigmentation in stable vitiligo lesions. To the best of our knowledge, this is the first study in the literature to establish the hierarchy of surgical modalities for stable vitiligo. We randomized 63 patients with 80 stable vitiligo lesions into three groups. Patients in group 1 were treated with NCES, group 2 with SBEG and group 3 with STSG. They were all evaluated 16 weeks after surgery for the extent of repigmentation, colour match, change in Dermatology Life Quality Index (DLQI) and patient satisfaction. The statistical analysis was carried out using SPSS version 17. Means were compared using Student's t-test for outcome. Qualitative data were compared using the χ^2-test or Fisher's exact test. The extent of repigmentation was excellent (> 90% repigmentation) in 71% of lesions in the NCES group, 27% of lesions in the SBEG group and 23% of lesions in the STSG group. So, in terms of producing excellent repigmentation, NCES was found to be significantly superior to SBEG ($P = 0.002$) and STSG ($P = 0.001$). Good repigmentation (> 75%) was seen in 89% of lesions in the NCES group, 85% in the SBEG group and 73% in the STSG group. There was a significant decrease (improvement) in DLQI scores in all of the groups, also the mean decrease among groups differed significantly. No significant difference was seen in colour match and pattern of repigmentation. Adverse effects were minimal. NCES (a cellular grafting technique) was found to have an edge over SBEG and STSG (both tissue grafting techniques) in terms of excellent repigmentation and patient satisfaction. NCES should be preferred over tissue grafting techniques and can be chosen as the first-line surgical modality for the treatment of stable vitiligo.

CS09

Herpes zoster in Sri Lanka

D. Bandara, M.T. Wickramasinghe, R. Weerasekara, D. Dissanayake and W. Ranaraja

General Hospital, Kegalle, Sri Lanka

The General Hospital, Kegalle, Sri Lanka, is a provincial teaching hospital serving mainly a farming community. Between 2002 and 2007 herpes zoster was very common among dermatology patients, with at least two admitted with this condition every day. This study was performed on 97 consecutive patients with herpes zoster admitted for treatment with aciclovir, between 30 December 2003 and 24 May 2005. All patients were treated in isolation for 1 week, with aciclovir 800 mg five times daily. Most were direct admissions from the clinic, along with some referrals from other departments. Diagnosis was performed clinically and a dermatomal chart was used to locate the dermatomes. All patients were subjected to blood screening on admission. If there was secondary infection, antibiotics were given together with analgesics. If there were no complications patients were discharged after 1 week and followed up at 1 and 3 months and 1 year. Forty-eight patients were male and 49 female; the age range was 9–85 years. Dermatoses were thoracic in 33/97 patients, in the trigeminal nerve in 31, cervical in 18 and sacral in one. The first division of the trigeminal nerve was the most commonly involved cranial nerve. A single dermatome was affected in 56 patients and multiple (adjacent) dermatomes were affected in 41 patients. Five patients presented with facial palsy and Ramsay–Hunt syndrome. Concurrent illnesses were found in 25 patients, mostly diabetes mellitus, hypertension, bronchial asthma and hyperlipidaemia. Two patients had psoriasis, one patient gave a history of pulmonary tuberculosis (treated), one patient was found to have severe anaemia subsequent to bleeding per rectum and another had haematuria. Interestingly no patient was found to have malignancy. Sixty-three patients had a definitive history of chicken-pox, but no patient had a history of herpes zoster. Seventy-two patients gave a history of neuralgic pain along the affected dermatome prior to the eruption. Thirty-six patients had pain at the time of discharge, 24 had pain after 3 months and 16 (mostly aged > 40 years) continued to have pain after 1 year. At the 1-month follow-up 76 patients presented; there were 67 at

© 2014 The Authors

3 months and only 47 after 1 year, perhaps because others were asymptomatic. Postherpetic neuralgia was managed with amitriptyline, carbamazepine and sodium valproate. Of the five patients with Ramsay–Hunt syndrome, one was male and four were female. Their age range was 27–64 years. Aciclovir and physiotherapy were given to these patients; two patients recovered completely and two improved their paralysis but continued to have tearing from their eye. One patient continued to have severe disability due to facial paralysis.

Posters

CS10
The challenge of managing pityriasis rubra pilaris: success at last with ustekinumab?

S.H. Foo, A. Rowe, M.B. Maheshwari and A. Abdullah

Sandwell and West Birmingham Hospitals NHS Trust, West Midlands, U.K.

Pityriasis rubra pilaris (PRP) is a rare papulosquamous disorder with unknown aetiology, which has traditionally been considered a variant of psoriasis. It was recognized as a separate entity only in 1857, by Devergie, and the term 'pityriasis rubra pilaris' was introduced in 1889 [Wood GS, Reizner GT. Other papulosquamous disorders. In: *Dermatology* (Bolognia JL, Jorizzo JL, Schaffer JV, eds), 3rd edn, Vol. 1. Philadelphia: Elsevier Saunders, 2012; chapter 9]. Therefore it is not uncommon for PRP to be misdiagnosed as psoriasis, especially in its early evolution. A 72-year-old man presented with sub-erythrodermic psoriasis not responding to topical treatment. He was initially treated with methotrexate, as ciclosporin was contraindicated due to his hypertension. He did not tolerate methotrexate. Despite an increased dose of acitretin and combination therapy of acitretin with etanercept, the patient continued to have generalized erythroderma with minimal improvement. An incisional skin biopsy was arranged to revisit the diagnosis. It showed alternating orthokeratosis and parakeratosis in a checkerboard pattern. The epidermis was acanthotic with broad rete ridges. No features of psoriasis were visible, confirming a diagnosis of PRP. The patient was then started on ustekinumab as he was extremely symptomatic and had had a poor response to previous multiple systemic treatments. Four weeks following his first injection of ustekinumab, his skin condition improved by 50% and his Dermatology Life Quality Index (DLQI) decreased from 21 to 9. By 12 months of therapy, he achieved near complete clearance of his skin condition, including improvement of his nail dystrophy. Therapeutic options for PRP mirror those for psoriasis; thus retinoids, methotrexate, phototherapy and antitumour necrosis factor therapy have been reported with variable success. Two case reports in the literature supported ustekinumab as a successful treatment for PRP. Resolution of PRP using ustekinumab as first-line therapy has been reported (Ruiz Villaverde R, Sánchez Cano, D. Successful treatment of type 1 pityriasis rubra pilaris with ustekinumab therapy. *Eur J Dermatol* 2010; **20**: 630–1), while Wohlrab and Kreft reported a patient with PRP who had previously been therapy resistant and was finally successfully treated with ustekinumab monotherapy (Wohlrab J, Kreft B. Treatment of pityriasis rubra pilaris with ustekinumab. *Br J Dermatol* 2010; **163**: 655–6). PRP is a challenging condition to manage, and despite its predicted clinical course of spontaneous resolution within 3–5 years, it still poses a significant impact on patients' quality of life during its active course. Our case highlights the successful option of using ustekinumab to manage therapy-resistant PRP with high patient satisfaction.

CS11
Psoriasis audit: secondary care vs. intermediate care for the assessment and management of psoriasis

R. Atkar and E. Derrick

Brighton General Hospital, Brighton, U.K.

The National Institute for Health and Care Excellence (NICE) and British Association of Dermatologists (BAD) publish guidelines and standards of care for the assessment and management of patients with psoriasis. The main objective of our audit was to compare adherence to the standards between dermatologists in secondary care with general practitioners with a special interest, working in intermediate care. We compared the results that were collected nationally for secondary care by the BAD against the results that were collected locally for intermediate care for psoriasis. We recruited 44 patients with a mean age of 49 years; 55% were male and 45% female. The national audit recruited 1092 patients. Our patients had the following conditions: chronic plaque psoriasis (91% national, 77% local); localized pustular psoriasis (1.6% national, 23% local); generalized pustular psoriasis (0.6% national only); and erythroderma (1.7% national only). In secondary care the main treatments given to each of the patients were classified as 'topical treatment alone' (11%), 'topical therapy with phototherapy' (10%), 'systemic nonbiologic with/without phototherapy' (51%), 'biologic with/without nonbiologic systemic therapy' (26%) and other types of therapy (1%). In intermediate care, the main treatment was either 'topical therapy alone' (64%) or 'topical therapy plus phototherapy' (36%), although the phototherapy was subsequently performed in secondary care. According to the recording assessments of the NICE standards, Physician's Global Assessment was recorded in 78% of patients in secondary care and 64% in intermediate care. Psoriasis Area and Severity Index (PASI) was recorded in 60% of patients in secondary care and 7% in intermediate care (a statistically significant difference). Dermatology Life Quality Index was recorded in 57% of patients in secondary care and 46% in intermediate care. Regarding the recording of clinical information, involvement of nails, high-impact and difficult-to-treat sites was recorded in 71% of patients in secondary care and 64% in intermediate care. Psoriatic arthritis was documented in 62% of patients in

© 2014 The Authors

secondary care and 8.2% in intermediate care (a statistically significant difference). Documentation of the Psoriasis Epidemiological Screening Tool was very poor in both secondary and intermediate care, with a median of 0%. In conclusion, this was a useful exercise as it demonstrates that there is no real difference in adherence to the standards when comparing secondary and intermediate care for the assessment and management of psoriasis. The only exceptions include documenting the PASI score and identifying psoriatic arthritis. Recommendations that were made from the national audit were presented to the intermediate care team to bridge these differences.

CS12
Pemphigus and associated comorbidities: a Canadian population-based study

K. Heelan,[1] A. Maher,[2] S. Walsh[1] and N.H. Shear[1]

[1]*Division of Dermatology, Department of Medicine, Sunnybrook Health Sciences Centre, Toronto, Ontario, Canada and* [2]*Department of Public Health Sciences, Queen's University, Kingston, Ontario, Canada*

Pemphigus is a rare autoimmune blistering disease, which has been reported to be associated with other coexisting disorders, autoimmune diseases and rare entities. We aimed to identify and describe patients with pemphigus who have a diagnosed comorbidity, and to calculate standardized prevalence ratios (SPRs) to quantify the risk of additional comorbidities. This was a cross-sectional study of patients diagnosed with pemphigus treated at a tertiary referral centre between September 2012 and April 2013. Prevalence rates of 15 comorbid diseases present at the time of the clinic visit (e.g. anxiety, diabetes, thyroid conditions) were calculated. Age-standardized SPRs and their 95% confidence interval (CIs) were calculated in comparison with prevalence rates of each disease in the general Canadian population using data from the Canadian Community Health Survey. The data were analysed using SAS 9.2. In total, 295 patients with pemphigus were identified. The median age was 56.0 years (range 21–90), and 175 patients (59%) were female. The most frequently occurring comorbidities included diabetes, hypertension, hypothyroidism, solid organ malignancy, heart disease and asthma. We observed an increased risk of hypothyroidism (n = 38, SPR 1.53, 95% CI 1.08–2.10), an increased risk of inflammatory bowel disease (SPR 1.48, 95% CI 0.40–3.80) and a twofold increase in diabetes risk (SPR 2.20, 95% CI 1.64–2.87). There was a lower prevalence in the pemphigus group of anxiety, asthma, solid organ malignancy, cataracts, chronic bronchitis, cardiovascular accident, fibromyalgia, glaucoma, heart disease, hypertension, migraines and rheumatoid arthritis than in the general population. Patients with pemphigus have a higher incidence of some comorbidities compared with the general population. As part of pemphigus work-up and surveillance, screening for thyroid disease and inflammatory bowel disease may be considered.

CS13
Invasive granulomatous aspergillosis mimicking cervicofacial actinomycosis in an immunocompetent host

S. Kumaran and S. Dogra

Post Graduate Institute of Medical Education and Research, Chandigarh, India

The incidence of deep fungal infections is on the rise owing to the background of immunosuppression (HIV). Cutaneous aspergillosis is a common systemic mycosis affecting immunosuppressed patients. Very few reports are available of *Aspergillus* affecting immunocompetent patients {Abir B, Abouchadi A, Hamama J *et al.* [Invasive aspergillosis of the maxillary sinus in an immunocompetent patient]. *Rev Stomatol Chir Maxillofac* 2012; **113**: 127–30 (in French); Alrajhi AA, Enani M, Mahasin Z, Al-Omran K. Chronic invasive aspergillosis of the paranasal sinuses in immunocompetent hosts from Saudi Arabia. *Am J Trop Med Hyg* 2001; **65**: 83–6}. We recently treated a young immunocompetent women who presented to us with a novel morphological type of cutaneous aspergillosis as a long-standing history of multiple nodules and discharging sinuses over the left side of the face The lesions clinically mimicked cervicofacial actinomycosis. Histological examination of skin biopsy revealed a granulomatous inflammation with presence of septate fungal hyphae, with acute angled branching morphologically resembling *Aspergillus*. On fungal culture using Sabouraud agar this was confirmed as *Aspergillus flavus*. On radiological examination, contrast-enhanced computed tomography of the peripheral nervous system (PNS) revealed abnormal soft tissue in the left maxillary sinus. There were hyperdense foci within it consistent with fungal sinusitis. Based on the clinical and investigational findings, a diagnosis of granulomatous invasive aspergillosis of the PNS with contiguous cutaneous aspergillosis and pulmonary aspergillosis in an immunocompetent patient was made. The patient was started on oral itraconazole 200 mg twice a day with marked clinical improvement in 8 weeks. The oral antifungal was continued for 4 months with complete closure of the palatal perforation and healing of facial sinuses.

CS14
Skin disease and infective endocarditis: a review of cases in New Zealand children, 1994–2013

C. Mahon,[1] H. Cheng,[1] R. Webb,[2] D. Purvis[1] and K. Agnew[1]

[1]*Dermatology Department, Greenlane Clinical Centre, Auckland, New Zealand and* [2]*Department of Paediatric Infectious Diseases, Starship Children's Hospital, Auckland, New Zealand*

Infective endocarditis is a life-threatening infection of cardiac valves and endothelium. *Staphylococcus aureus* is the most frequent cause of severe, complicated infective endocarditis. Established risk factors include congenital and rheumatic heart diseases as well as immune compromise. New Zealand is a developed country with high rates of invasive bacterial infections and postinfectious sequelae, in particular rheumatic heart disease. The association between skin disease and infective endocarditis is unclear, and in children is described only in a few case

© 2014 The Authors

reports. There is increasing interest in eczema as a potential risk factor for infective endocarditis, in which *S. aureus* colonization and recurrent infection is common. We sought to describe the clinical and microbiological features of New Zealand children with a recognized cutaneous dermatosis who were treated for infective endocarditis between 1994 and 2013. Cases of infective endocarditis in children aged under 16 years were retrospectively identified from hospital records. There were 69 cases of definite endocarditis (defined by modified Duke criteria) identified between 1994 and 2013. We observed six cases of infective endocarditis in children with a concurrent skin disorder. There were five cases with eczema and one case with Netherton syndrome. Two cases with eczema also contracted primary varicella zoster virus infection, which preceded the diagnosis of infective endocarditis. Congenital cardiac anomalies were identified in two children and another child had rheumatic heart disease. *S. aureus* was the causative organism in five cases and *Streptococcus pyogenes* in one case. This is the first series describing skin disease and infective endocarditis in a paediatric population. In addition, the association between varicella zoster virus infection and infective endocarditis has not previously been reported in the dermatological literature. Potential cutaneous risk factors should be considered in children with underlying cardiac abnormalities. In New Zealand, where varicella vaccination is not routine, children with eczema may be at particular risk during primary varicella infection.

CS15
An uncommon fungal infection of the skin associated with granulocytic sarcoma: a diagnostic dilemma

K. Mallawaarachchi,[1] S. Mendis[1] and S. Ratnayake[2]

[1]*Colombo North Teaching Hospital, Ragama, Sri Lanka and* [2]*Faculty of Medicine, University of Ragama, Ragama, Sri Lanka*

Granulocytic sarcoma (myeloid sarcoma) is a tumour of myeloid origin in an extramedullary site. It usually occurs concurrently with acute myeloid leukaemia or myeloproliferative disorder. However, it may develop *de novo* and precede the manifestation of haematological disease. Among other sites, skin is a known site for this neoplasm. Granulocytic sarcoma can pose diagnostic difficulties, especially when bone marrow and peripheral blood are not involved (Markoc F, Bozdogon N, Yükrük FA *et al.* Granulocytic sarcoma: difficulties in diagnosis. *Tumori* 2010; **96**: 149–153). Even in such cases, haematological disease tends to manifest within a few months. While it is rare for patients to remain free of systemic disease for several years, cutaneous lesions demonstrating gradual resolution are extremely unusual. *Curvularia lunata* is a ubiquitous soil fungus in temperate areas, and causes human infections only rarely. While *Curvularia* spp. are well known to cause mycetoma and onychomycosis, ulcerative and nodular infiltrated lesions are only rarely reported. Because *Curvularia* spp. are dematiaceous fungi, the resulting infections are generally known as cutaneous phaeohyphomycosis (Moody MN, Tchen J, Mesko M. Cutaneous *Curvularia* infection of the forearm. *Cutis* 2012; **89**: 65–8). Usual histological findings in *Curvularia* infection are those of a neutrophilic and granulomatous reaction. Herein, we report the case of a 68-year-old woman who presented with two nodular and ulcerated skin lesions adjacent to her left knee for 3 years. Repeated skin biopsies from different sites within the lesions yielded pure growths of *Curvularia lunata*, suggesting an infection rather than colonization. However, histopathological and immunohistochemical studies, including positive myeloperoxidase and leucocyte common antigen, favoured a diagnosis of granulocytic sarcoma. Interestingly, extensive investigations failed to reveal peripheral blood, bone marrow or solid organ involvement. Meanwhile, the patient did not consent to oncological management. Considering this fact and the evidence of fungal infections, she was started on antifungal therapy. Her lesion demonstrated signs of healing within the first month of treatment. After a 6-month course of oral itraconazole therapy, $> 90\%$ of the lesion healed without evidence of any local recurrence. Throughout this period, peripheral blood investigations remained normal. This clinical picture is unusual for both granulocytic sarcoma and for this type of fungal infection. Further follow-up is needed to exclude any recurrence and to confirm complete recovery.

CS16
Autologous serum skin test vs. autologous plasma skin test in patients with chronic spontaneous urticaria: is there a response correlation?

S. Kumaran, S. Manal and D. Parsad

Post Graduate Institute of Medical Education and Research, Chandigarh, India

Autologous serum skin test (ASST) and autologous plasma skin test (APST) are simple tests for demonstrating functional properties of antibodies in chronic spontaneous urticaria (CSU). However, concerns have been raised regarding their interpretation and specificity. The objective of this study was to assess differences in response of ASST and APST, and to correlate further the intensity of positive response with clinicoepidemiological and laboratory parameters. This was a prospective, case-controlled study of 110 patients with CSU, who had a duration of illness > 3 months; 100 sex- and age-matched healthy controls were included. Routine laboratory investigations including thyroid function tests (TFTs), measurement of antithyroid peroxidase (anti-TPO) antibodies, IgE levels and antinuclear antibodies, and ASST and APST. Disease severity was assessed using the Urticaria Activity Score (UAS). Among the 110 patients analysed, 78 and 90 patients were ASST+ and APST+, respectively, with 68 having a positive response to both. Ten ASST+ patients were APST−, whereas all 22 ASST− patients were APST+. ASST and APST positivity were significantly higher in the patient group compared with controls (both $P < 0.0001$). ASST positivity was observed in 84% of patients with normal IgE levels ($\kappa = -0.141$, $P = 0.012$), and 92.9% of patients with normal TFTs were APST+ ($\kappa = -0.062$, $P = 0.029$). The majority of patients with involvement of the neck, flexures, and interdigital and retroauricular areas were ASST− but APST+. No statistically signifi-

© 2014 The Authors

cant correlations were observed between the intensity of positive response with regards to age, sex, angio-oedema, duration of disease, UAS and anti-TPO antibodies. APST appears to be better than ASST in many aspects for investigation of patients with CSU.

CS17
Deep vein thrombosis following thalidomide therapy in a patient with erythema nodosum leprosum receiving multibacillary multidrug therapy and prednisolone

A. Budania[1] and H.K. Kar[2]
[1]*Vardhman Mahavir Medical College and Safdarjung Hospital, New Delhi, India and* [2]*Post Graduate Institute of Medical Education and Research, and Dr Ram Manohar Lohia Hospital, New Delhi, India*

Thalidomide is an important part of treatment for multiple myeloma and is also used for treatment of severe resistant type 2 leprosy reaction. It has been found to suppress all clinical manifestations of type 2 reaction, including erythema nodosum leprosum (ENL), within 48–72 h. As well as its known and dreaded adverse effects, including teratogenicity and neuropathy, thalidomide has been associated with deep vein thrombosis (DVT) in patients with multiple myeloma, with incidence rates of around 1% when given alone and 20–30% when combined with dexamethasone (Weber D, Rankin K, Gavino M *et al*. Thalidomide alone or with dexamethasone for previously untreated multiple myeloma. *J Clin Oncol* 2003; **21**: 16–19). Rarely, DVT has been reported with thalidomide administered for ENL (Vetrichevvel TP, Pise GA, Thappa DM. A case report of venous thrombosis in a leprosy patient treated with corticosteroid and thalidomide. *Lepr Rev* 2008; **79**: 193–5). Here we report DVT in a patient with recurrent ENL on multibacillary multidrug therapy (MDT-MB) with thalidomide and prednisolone. A 66-year-old man was under treatment for lepromatous leprosy with MDT-MB at our urban leprosy centre. After about 1 month of MDT-MB therapy, the patient developed ENL (diagnosed clinically and on histopathology). After no response with prednisolone, thalidomide 400 mg daily was added to his regimen. After 3 months of combined treatment with prednisolone and thalidomide, the patient developed pain and swelling in his right lower limb, which was diagnosed as DVT by clinical and radiological examination. Thalidomide was stopped immediately, while oral prednisolone continued at 40 mg daily. The patient was started on enoxaparin 60 mg in 0.6 mL subcutaneously twice a day. Warfarin 5 mg was added on the next day. Combined enoxaparin and warfarin therapy was continued for 5 days, after which enoxaparin was stopped. The prothrombin time was maintained between international normalized ratio 2.5 and 3.5. After about 2 weeks of therapy, the patient improved symptomatically and all the swelling and pain reduced. To conclude, combined thalidomide–prednisolone therapy could be a rare risk factor for DVT – whether it is for multiple myeloma or ENL. Prophylactic anticoagulation in the form of low-dose warfarin or low-molecular-weight heparin can be recommended during inpatient management of ENL with thalidomide and prednisolone.

CS18
Clinical and investigational profile of herpes genitalis in a tertiary care centre

A. Itty, B. Joy and R. Sridharan
Academy of Medical Sciences, Pariyaram, Kannur, Kerala, India

Our aim was to study the clinical and investigational profiles of patients with herpes genitalis reporting to the sexually transmitted disease (STD) clinic of a tertiary care centre during a period of 2 years. This was a retrospective study carried out among patients attending the STD clinic between May 2011 and April 2013. History, clinical examination, Tzanck smear and serology were evaluated in all patients with herpes genitalis. Herpes genitalis was present in 36% of patients (male-to-female ratio 0.46) attending our STD clinic in the study period. Among the patients with a first episode of disease (45%), erosion constituted the major sign (48%). Superficial ulcer (26%) was the major sign in recurrent cases (55%). Tzanck smear was positive in 60% of patients. Herpes simplex virus (HSV) serology was positive in 62% of patients, with specific HSV-2 antibodies positive in 38% of cases (Lafferty WE, Downey L, Celum C. Herpes simplex virus type 1 as a cause of genital herpes: impact on surveillance and prevention. *J Infect Dis* 2000; **181**: 1454–7). From this study we infer that primary genital herpes rarely presents with classical vesicles. Increasing prevalence of HSV-2 suggests transmission by a large number of asymptomatic cases and the occurrence of asymptomatic shedding (Dhawan J, Khandpur S. Emerging trends in viral sexually transmitted infections in India. *Indian J Dermatol Venereol Leprol* 2009; **75**: 561–5). A greater prevalence of the disease in the female population points to the need for immunoprophylaxis to prevent transmission to the next generation.

CS19
A survey of dermatological conditions in northern India: are we seeing changes reflecting urbanization?

F. Latheef[1] and R. Strauss[2]
[1]*Leeds Centre for Dermatology, Chapel Allerton Hospital, Leeds, U.K. and* [2]*Dermatology Department, Harrogate and District NHS Foundation Trust, Harrogate, U.K.*

There is limited information on the epidemiology of skin disease in India, with only a small number of cross-sectional studies reporting mostly on population subgroups such as children or immunocompromised patients. Some of these studies suggest a preponderance of conditions of infective aetiology attributed to the rural population being studied (Negi KS, Kandpal SD, Parsad D. Pattern of skin disease in children in the Garhwal Region of Uttar Pradesh. *Indian Pediatr* 2001; **38**: 77–80). In order to gain further insight into the spectrum of skin disease prevalent in this area, we conducted a rudimentary survey of dermatological conditions presenting to a

medical camp held in the Jalandhar province in the Punjab area of northern India. All patients attending the camp during a 3-day period were initially triaged by an accident and emergency consultant. Those identified with skin disease were subsequently examined by a British-trained consultant dermatologist. Access to the camp was free, and awareness had been previously raised by adverts in the local newspapers, radio and television stations and through posters in the area. Information regarding age, sex and presenting skin complaints were registered. Diagnoses were made based on characteristic clinical features alone and grouped into broader categories for ease of analysis. In total 135 patients with 41 skin conditions were seen over the 3-day period; 44% were male and 56% female, and the mean age was 34 years (range 2–80). Overall 113 patients (83.7%) presented with one skin condition, and 22 patients (16.3%) attended with two skin complaints. Acne vulgaris was the most common presenting condition (15%) followed closely by various presentations of eczema/dermatitis (13.7%). Other conditions commonly seen were pigmentary disorders (11.8%), hyperkeratotic conditions (10%), ichthyosis (6.9%) and benign tumours (6.3%). Infections, both fungal (5%) and bacterial (2.5%), featured relatively less frequently. Limitations of the study include possible selection bias, as expected with an annual camp, which may have skewed the range of conditions from the acute to the chronic side, lack of diagnostic tests and a small sample size. Despite this, the disease spectrum presented, especially with dermatitis featuring higher than infection, is similar to that observed in the Western world, possibly reflecting a change in epidemiology of skin disease as reported with urbanization elsewhere (Schram ME, Tedja AM, Spijker R *et al*. Is there a rural/urban gradient in the prevalence of eczema? A systematic review. *Br J Dermatol* 2010; **162**: 964–73).

CS20
Moxifloxacin-based regimens in leprosy: interim observations on occurrence of reactions and bacterial decline

V.V. Pai

Bombay Leprosy Project, Bombay, India

Clinical trials in leprosy using moxifloxacin-based regimens were reported for first time in 2009 {Ganapati R, Pai VV, Khanolkar SA, Shinde M. [Clinical trials with treatments based on moxifloxacin: preliminary communication]. *Revista de Leprologia* 2009; **27**: 49–55 (in Spanish)}. Moxifloxacin, a fluoroquinolone, has proven to be a powerful bactericidal agent against *Mycobacterium leprae*. Observations pertaining to clinical aspects, bacterial index (BI) decline and reactions were reported in 2013 (Pai VV, Halwai V, Nanda A, Raja D. Moxifloxacin based regimens in leprosy – observations on occurrence of reactions and bacterial decline. Presented at the *18th International Leprosy Congress*, Brussels, Belgium, 16–19 September 2013; abstr. O239). We now report further observations based on this regimen on a larger sample of patients. The aim of the study was to make observations on a selected sample of 284 patients. Of these, 95 were smear positive (81 male and 11 female) and receiving moxifloxacin 400 mg, rifampicin 600 mg and minocycline 200 mg (MRM); 94 formed the smear-negative paucibacillary (PB) group and received MRM; and 95 were smear-positive (77 male and 18 female) and receiving MRM plus clofazimine (MRMC; to judge the anti-inflammatory properties of clofazimine in preventing reactions). The patients were part of an ongoing comparative clinical trial where the drugs were dosed at monthly intervals for 12 months. Patients available for a follow-up period of 3 years were analysed. The bacteriological status of all patients, as measured by BI at months 12 and 24, was assessed in both groups. It was observed that a high proportion of patients underwent reactions: 32 (34%) in the MRM group, 25 (26%) in the MRMC group and six (6%) in the PB group. The reactions were well controlled with conventional treatment. Addition of clofazimine had no particular influence on occurrence of reactions, although they were less common in the MRMC group. Long-term observations are in progress. During the period of study, the average BI of all patients in the smear-positive groups with initial mean BI > 3.0 showed a steady decrease. In conclusion, interim observations in both the MRM and MRMC groups are encouraging and comparable with standard multidrug therapy. As the study is in progress, long-term observations on the rate of decrease of BI on a larger sample of patients after stoppage of treatment, as well as relapses, will be reported.

© 2014 The Authors
