# Supplementary material for: Therapeutic efficacy of cell-based therapy in vitiligo: a research letter systematically reviewed using meta-analysis
Source: Arch Dermatol Res. 2024 May 22;316(5):198. doi: 10.1007/s00403-024-02920-6 (PMC11111487; doi:10.1007/s00403-024-02920-6)
Supplement: Supplementary file 1 — Supplementary file1 (ZIP 24195 KB) [file 403_2024_2920_MOESM1_ESM.zip › Studies were included/Ebadi 2015.pdf]

ORIGINAL ARTICLE

# The additive effect of excimer laser on non-cultured melanocyte–keratinocyte transplantation for the treatment of vitiligo: a clinical trial in an Iranian population

A. Ebadi,[1] M.M. Rad,[1] S. Nazari,[1] R.J. Fesharaki,[1] F. Ghalamkarpour,[1]*, S. Younespour[2]

[1]Skin Research Center, Shahid Beheshti University of Medical Sciences, Shohada-e-tajrish Hospital, Tehran, Iran
[2]Department of Epidemiology and Biostatistics, School of Public Health, Tehran University of Medical Sciences, Tehran, Iran
*Correspondence: F. Ghalamkarpour. E-mail: fghalamkarpour@yahoo.com

## Abstract

**Background** Non-cultured cellular grafting is specified for stable vitiligo treatment as a novel surgical technique, however, the additive effect of excimer laser on melanocytes–keratinocytes transplantation (MKT) have not experienced yet.

**Objective** To assay the additive effect of excimer laser on MKT for the treatment of vitiligo in an Iranian population.

**Methods and materials** In this non-randomized clinical trial, 39 patches were evaluated. Nine patches treated by MKT alone, 10 patches treated by MKT and excimer laser, 10 patches treated with excimer alone and 10 patches left without any treatments as control patches.

**Results** In the patches treated with excimer, the percentage reduction of depigmented area from baseline varied, ranging from 0 to 43.9%. In the nine patches treated with non-cultured MKT, the median percentage reduction of depigmented area from baseline was 15.9%. The median percentage reduction of depigmented area in excimer + MKT was 41.9% .In the untreated patches, the median percentage reduction of depigmented area was 0.1%. After controlling for the effect of 'depigmented area at baseline', significant reductions were observed in depigmented area of the patches treated with combination therapy of excimer and non-cultured MKT in comparison with the other therapies and untreated patches.

**Conclusion** Although pigmentation in our patients was lower than previous reports from Iran and other countries, however, we signified adding excimer to MKT increased the pigmentation rate in treated patches. Further investigations are recommended with longer follow-up and larger series to validate the findings reported here.

Received: 11 February 2014; Accepted: 17 June 2014

## Conflicts of interest

None declared.

## Funding source

None declared.

## Introduction

Vitiligo is a common acquired skin pigmentary disorder characterized by white patches on the skin resulting from progressive loss of melanocytes.[1] The worldwide incidence of vitiligo is about 1% to 2% and there is a frail female predominance (2–3 : 1 ratio).[2] The aetiology of vitiligo remains unknown, however, genetic investigations support non-Mendelian inheritance signifying that vitiligo is a polygenic and multifactorial skin disorder.[3] The autoimmune theory is well recognized and previous investigations have indicated the association of vitiligo with some autoimmune disease such as thyroid disease, adrenal insufficiency, gonadal dysfunction, etc.[4] There is no cure for vitiligo; however, there are diverse classes of treatment options such as corticosteroids, calcineurin inhibitor derivatives, phototherapy and surgery that improve the patient's condition. The main therapeutic principle for treating vitiligo is to restore pigmentation in the depigmented area and improve the patient's appearance.[3]

Tissue grafts including full-thickness punch grafts, split-thickness grafts and suction blister grafts, cultured autologous epithelial grafts, cultured melanocytes and non-cultured melanocytes–keratinocytes transplantation (MKT) are the surgical techniques for vitiligo treatment.[5] The non-cultured MKT technique has applied previously and showed an easy economic technique, which may be applied in resistant areas of stable vitiligo.[6,7]

 © 2014 European Academy of Dermatology and Venereology

It was presented by Gauthier and Surleve-Bazeille[8] in 1992. In their experience, to prepare a cellular suspension, they superficially shaved the scalp and achieved a skin donor and treated it, using trypsin 0.25% for 18 h for dermoepidermal separation. Then they inoculated suspension at the recipient region into blisters raised with liquid nitrogen.

Olsson and Juhlin[9] in 1998 carried out a similar experience, but they obtained donor sample from gluteal area and reduced trypsinization to 50 min; furthermore, to carry out the procedure on a day, they used cellular suspension directly on to a dermabraded vitiligo lesion. Moreover, Pianigiani *et al.* in a study evaluated the possibility of treating vitiligo by autologous grafting of epidermal cells and narrow-band ultraviolet B they showed this treatment can be regarded for the stable vitiligo treatment. However, the additive effect of excimer laser on MKT have not experienced yet, hence we steered this study to assay the additive effect of excimer laser on MKT for the treatment of vitiligo in an Iranian population.

## Methods and materials

This study was the second experience in Shohada Tajrish Hospital and the technique was the same and in line with Toosi *et al.* study. However, we added the excimer laser on non-cultured melanocyte–keratinocyte transplantation for the treatment of vitiligo as an adjunct therapy.

In this non-randomized clinical trial we included 16 patients but six of them were excluded (five had organ specific antibody, one patient gave up the study due to car accident and fracture of the femur after 4 session of laser therapy) so, at last 10 patients (four men and six women) aged between 21 and 48 years with stable generalize vitiligo were recruited in this study between March 2001 and October 2012. The ethical committee of Shaheed Beheshti University of medical sciences approved the study protocol (registration number M/T/P 88/1076), moreover, the study procedure was explained for all the patients and informed written consents were taken.

In this study 39 patches were dermabraded, nine patches treated by MKT alone, 10 patches treated by MKT & excimer laser, 10 patches treated with excimer alone and 10 patches left without any treatments as control patches .

The percentage of depigmented area of the treated regions was calculated before and after the procedures using AutoCAD (Autodesk Inc., San Rafael, CA, USA) software.

The repigmentation grading was as follow; 95% to 100% excellent, 65% to 94% good, 25% to 64% fair and 0–24% poor repigmentation.

To conduct this trial, we applied the modified Olsson and Juhlin technique that formerly was modified by Mulkar.[1]

The clinical profile of the patients and a description of the lesions treated are detailed in Table 1.

Because vitiliginous patches located on hands and feet usually poorly response to different treatment modality, we tried to select the patches from trunk or face preferably, but only in one patient the patches were located only on joints, but all MKT and MKT + excimer patches were from same site, except for one patient due to limited number of vitiligo patches, the MKT patch choose was on trunk and MKT + excimer patch choose was located on upper part of the thigh. The locations of patches in each patient are included in Table 1.

### Inclusion and exclusion criteria

The patients were recruited if they had clinically stable lesions (patients whose vitiligo patches had not demonstrated progression or who had not reported any new lesions in the past 12 months).

Exclusion criteria were as follows: body surface involvement >30%; receiving any concomitant medical treatment in the last 12 months; aspirin, NSAIDS and vitamin E in the last 10 days; NBUVB in last two weeks; age <12 years and abnormal quantities of organ specific antibodies (ANA, Anti-parietal cell antibody, anti-mitochondrial antibody, anti-microsomal antibody, anti-thyroglobulin and anti-TPO), moreover, an abnormal haemogram (CBC/diff, ESR, RF, CRP, BUN, Creatinine, ALT, AST, A1kaline Phosphatase, FBS) and abnormal urinalysis.

### Preparation of the patients

Two dermatologists and a dermatology resident evaluated the patients using digital photographs taken before and 2 weeks after the end of the excimer laser treatment (Digital camera; Canon 40D, SLR, Japan, lens canon 18–200).

Then to estimate the recipient area before procedure the lesions were measured under a wood's lamp.

One night before to 7 days after operation cephalexin 500 mg/TDS were given to the patients, moreover, acetaminophen 500 mg and Xanax 0.5 mg 30 min before operation were given. In addition, Acyclovir 400 mg TDS for 7 days were given if there was a history of herpes simplex.

### Donor site biopsy and cell separation

To rule out a non-visible or undetected vitiligo region, we marked a donor region of 1/5–1/10 of the recipient area on the lateral aspect of the upper third of the thigh under the wood's lamp.

One hour before the biopsy, we applied a thick layer of cream (EMLA 5%; Astrazeneca, Istanbul, Turkey) to the donor and recipient areas and covered by Tegaderm (3M Healthcare, St. Paul, MN, USA) semipermeable dressing.

Then one hour later, we cleaned the donor site using alcohol 70% and anaesthetized using lidocaine 2%. The skin was stretched by the surgical aide and a very superficial sample with a small Silver Dermatome (Aesculap, Tuttlingen, Germany) was harvested and the skin layers were removed down to the papillary dermis where pinpoint bleeding was observed. Then we covered the superficial wound with gentamicin ointment, sterile

 © 2014 European Academy of Dermatology and Venereology

**Table 1** The percentage change of depigmented area (from baseline) in the treated and untreated patches of patients with stable generalized vitiligo

| Patients | Age | Sex | Duration of disease (years) | Percentage change from baseline* | | | |
|---|---|---|---|---|---|---|---|
| | | | | Excimer | Non-cultured MKT | Non-cultured MKT + excimer | Control |
| 1<br>Locations | 25 | Male | 5 | −11.75<br>Face | −51.21<br>Trunk | −98.73<br>Trunk | 0.47<br>Face |
| 2<br>Locations | 22 | Female | 8 | −5.84<br>Trunk | −40.58<br>Trunk | −84.13<br>Trunk | 0.78<br>Trunk |
| 3<br>Locations | 28 | Male | 4 | −0.95<br>Face | −15.91<br>Trunk | −40.14<br>Trunk | −0.59<br>Face |
| 4<br>Locations | 48 | Female | 9 | 0.20<br>Face | −8.66<br>Trunk | −40.42<br>Trunk | −0.08<br>Trunk |
| 5<br>Locations | 21 | Female | 3 | −0.75<br>Face | 0.62<br>Trunk | −10.43<br>Trunk | −0.18<br>Trunk |
| 6<br>Locations | 32 | Female | 17 | −43.91<br>Breast | −76.70<br>Breast | −98.73<br>Breast | −0.41<br>Breast |
| 7<br>Locations | 44 | Male | 4 | −6.61<br>Trunk | −12.25<br>Trunk | −28.74<br>Trunk | −0.11<br>Face |
| 8<br>Locations | 30 | Female | 3 | −3.58<br>Ankle | −9.03<br>Knee | −14.30<br>Knee | 0.05<br>Face |
| 9<br>Locations | 37 | Male | 5 | −17.96<br>Face | . | −43.46<br>Trunk | −1.10<br>Face |
| 10<br>Locations | 31 | Female | 3 | 0.0<br>Face | −59.27<br>Breast | −88.60<br>Breast | 0.84<br>Trunk |

*Percentage change from baseline ($cm^2$): (depigmented area at end point − depigmented area at baseline)/depigmented area at baseline × 100.

Vaseline gauze and the semipermeable lucent dressing (Tegaderm). The thin skin sample was transferred to a 15 mL tube containing 4 mL of trypsin-EDTA solution and then the sample was properly soaked in the solution.

*Cell separation and control of cell viability before transplantation* In a laminar flow bench kept in the laboratory beside the operation room we performed all of the following procedures.

The obtained sample was incubated at 37°C for 20 min and then the tube was removed from the incubator and trypsin solution was aspirated using a Pasteur pipette. Moreover, to remove the remaining trypsin the sample was washed.

Then to remove the remaining of trypsin, the sample was washed using Dulbecco's Modified Eagle's Medium F-12 (DMEMF-12).

We used DMEMF-12 for various stages of the cell separation procedure and the solution was not supplemented with any kind of antibiotic or growth factor throughout the cell suspension preparation stages.

By a pair of forceps, the epidermis was separated and broken into small pieces using a disposable surgical blade then transferred to a test tube containing DMEMF-12 and mixed for 15 s. Then to prepare a suspension, the epidermal pieces were centrifuged at 251 g for 3 min.

The epidermal pieces were discarded and the cell pellet was suspended in DMEMF-12 medium and hyaluronic acid gel (R-fine), (to increase the viscosity of the suspension and to lessen suspension movement) in a 1 mL syringe with a detachable needle, and then one drop of the cell suspension was stained with trypan blue and observed under an inverted microscope to check the viability of the cells.

## Preparation of recipient area

Using an alcohol soaked cotton ball the recipient and control regions were cleaned and sterilized using povidone iodine and ethanol 70%, then these regions anaesthetized with 2% lidocaine and a ring block was performed down to the dermoepidermal junction. Recipient and control regions were abraded using a high-speed (more than 20 000 T/min) dermabrader (Strong, Korea) fitted with a diamond fraise wheel and the procedure was continued to appear the pinpoint bleeding spots (Fig. 1).

Subsequently, the mentioned areas were covered with gauze pieces which were moistened with normal saline solution, until the cell suspension was applied on the recipient areas. Both recipient and control sites were covered with dry collagen (Surgicoll, Neustadt-Glewe, Germany) to help the transplanted cells to remain in place in the MKT group and held in place with Tegaderm transparent semipermeable dressing.

Finally, the prepared regions were covered with gauze pieces which were moistened with normal saline solution, until the cell suspension was applied on the recipient areas. To help the transplanted cells to remain in place (in the MKT group) and held in


© 2014 European Academy of Dermatology and Venereology

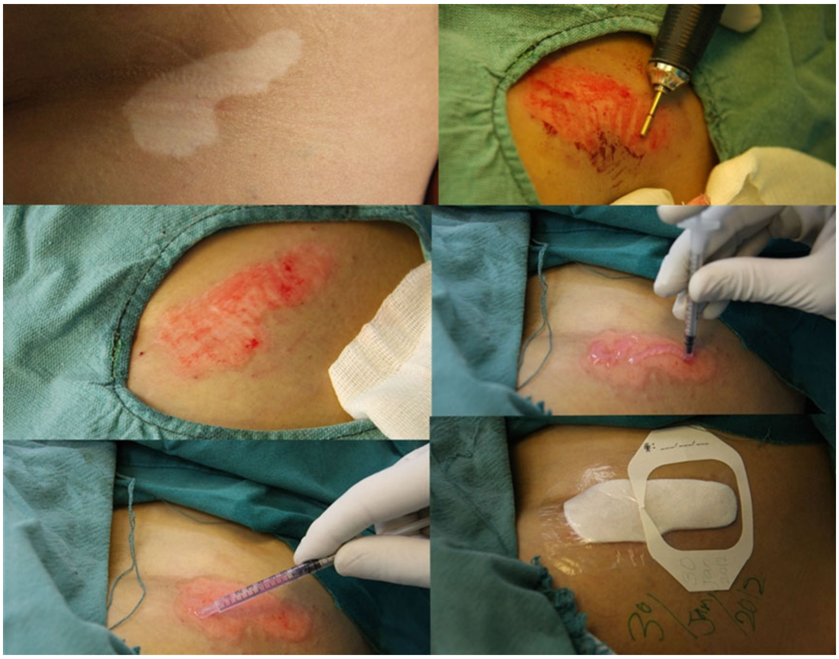

**Figure 1** The steps of operation in patients.

place (with Tegaderm transparent semipermeable dressing), recipient and control regions were covered with dry collagen (Surgicoll)

After 1 week, dressing was removed and a control photograph was taken. For removing the dressing, the recipient and control areas were soaked in 0.9% normal saline solution to separate the dressing from the skin spontaneously and without any pulling forces.

Four hours after dressing, the patients were allowed to go home and recommended to avoid vigorous activity for 1 week and cover the operated regions with a dark cloth to protect it from visible and ultraviolet light.

One week later, the wounds were undressed and control photograph was taken and excimer laser got started 1 week after dressing removal.

*Laser protocol* We applied 308-nm excimer laser with a self-contained gas system of Xe-Cl (DEKA excimer laser, Excillite, Italy). If there was no erythema or inflammations 2 weeks after grafting, excimer laser (308 nm) therapy got started in all patients for 24 sessions, 2-3 times/week.

Two patches including the grafted + excimer patches and the excimer patches were irradiated by excimer laser.

Furthermore, the control and other grafted patches were protected by a window glass and fixed in place by thick gauze dressing to prevent unwanted irradiation on these patches.

The fluence was begun by 200 $mJ/cm^2$, and the following dose was determined: if there was no erythema after the initial treatment 100 $mJ/cm^2$ increment, if there was erythema less than 24 h, 50 $mJ/cm^2$ increment, and no increment if there was erythema for 24 h or more. If symptomatic erythema such as burning and pain or blistering developed, treatment was withdrawn until resolution, and then the dose was decreased by 50 $mJ/cm^2$.

### Statistical analysis

Missing responses in a randomized complete block design make the usual ANOVA calculations inappropriate. However, using the regression approach in the case of missing responses is ordinarily still appropriate for testing factor effects. In this randomized complete block design, there was a missing response for one of the patches treated with non-cultured MKT because a photo of one session was lost due to camera memory card error. Thus, the regression approach for testing factor effects was used. To control the effect of the depigmented area at baseline on the depigmented area at end point, this variable was considered as a covariate in the regression analysis. Pair-wise comparisons with Bonferroni corrections were applied to assess the differences in repigmentation scores between different therapies. All tests applied were two-sided and a significance level of 0.05 was considered significant. All statistical analysis were performed with statistical software SPSS 16.0.0. (SPSS Inc. Chicago, IL, USA).

## Result

Totally we evaluated 10 patients with stable generalized vitiligo (six women and four men). The mean age of patients was 31.8 (SD 8.9) with median of 30.5 years (range 21–48 years). The median duration of disease was 4.5 years (range 3–17 years). Two patients had family history of vitiligo. Table 1 shows the percentage change of depigmented area (from baseline) in

 © 2014 European Academy of Dermatology and Venereology

treated and untreated patches of patients with stable generalized vitiligo.

All patients were treated three times/week, but two patients were treated two times/week because of they were very busy .One of them showed good response to treatment and one fair (Table 1). Moreover, all patches placed on trunk and face but patches in one patient placed on joints (patient 8) that showed good response to treatment.

The frequency distribution of patients with vitiligo, classified according to the percentage change of depigmented area from baseline is shown in Table 2.

In the patches treated with excimer, the percentage reduction of depigmented area from baseline varied, ranging from 0 to 43.9%. The median percentage reduction of depigmented area was 4.7% (interquartile range, 0.6–13.3%).

In the nine patches treated with non-cultured MKT, the median percentage reduction of depigmented area from baseline was 15.9% (range, 0.0–76.7%; interquartile range, 8.8–55.2%).

With the combination therapy of excimer and non-cultured MKT, good and excellent responses were observed in four patients (Table 2). The median percentage reduction of depigmented area was 41.9% (interquartile range, 25.1–91.1%).

In the untreated patches, the median percentage reduction of depigmented area was 0.1% (with the maximum reduction of 1.1%).

In this randomized complete block design, there was a missing response for one of the patches treated with non-cultured MKT. Using regression approach for testing the therapies' effects, after controlling for the variable 'depigmented area at baseline', the mean depigmented area for the four therapies differed ($P < 0.001$, Table 3). After controlling for the effect of 'depigmented area at baseline', significant reductions were observed in depigmented area of the patches treated with combination therapy of excimer and non-cultured MKT in comparison with the other therapies and untreated patches (Table 4).

**Table 3** Summary results of a multiple regression analysis applied to the data

| Source of variation | Type III sum of squares | df | Mean square | *F* | Sig. |
|---|---|---|---|---|---|
| Subjects (block) | 2562.463 | 9 | 284.72 | 46.72 | <0.001 |
| Treatments | 197.268 | 3 | 65.756 | 10.79 | <0.001 |
| Depigmented area at baseline | 1446.996 | 1 | 1446.996 | 237.45 | <0.001 |
| Error | 152.340 | 25 | 6.094 | | |
| Total | 4359.067 | 38 | | | |

## Discussion

Using autologous melanocytes in the form of skin cell suspension of melanocytes and keratinocytes to treat vitiligo is not to cure the disease but to influence pigmentation by replacing non-functional or deficient melanocytes in the skin.[8]

In this study, we evaluated additive effect of excimer laser on non-cultured MKT for the treatment of vitiligo. Since the first experience by Gauthier and Surleve-Bazeille in 1992, several studies followed, including some modifications and more simplifications.[8–13] In this survey we followed the Toosi *et al.* Method, but we added the excimer laser as an adjunct therapy. Totally 10 patients (39 patches) were evaluated. In excimer and control groups, the excellent and good repigmentation (64–100%) did not occur in any patches, in MKT group good repigmentation occurred in one patches (11%), but in MKT plus excimer group two patches showed good and two patches excellent repigmentation, in other word four of 10 patches (40%) showed repigmentation more than 65%.

Prior studies did not use MKT plus excimer and to our knowledge this is the first study in this filed, but in comparison to previous reports that used MKT method, repigmentation rate in our experience is lower than these surveys. However, we emphasize that this was a controlled trial that lower pigmentation rate did not affect our principal objective was the additive effect of excimer on MKT.

In previous experience in Shohada Tajrish hospital Toosi *et al.*[7] indicated repigmentation more than 65% occurred in five of eight lesions (62%); furthermore, van Geel *et al.* [11] in another trial signified 71% of treated patients obtained a repigmentation of 75%. Moreover, they noted in 93% of patients repigmentation

**Table 2** Frequency distribution of patients with vitiligo, classified according to the percentage change of depigmented area from baseline

| Repigmentation percentage* | Excimer (10 patches) | Non-cultured MKT (9 patches) | Non-cultured MKT + excimer (10 patches) | Control (10 patches) |
|---|---|---|---|---|
| No pigmentation or becoming worse | 2 | 1 | 0 | 4 |
| Poor | 7 | 4 | 2 | 6 |
| Fair | 1 | 3 | 4 | 0 |
| Good | 0 | 1 | 2 | 0 |
| Excellent | 0 | 0 | 2 | 0 |

Repigmentation was graded as excellent with 95–100% repigmentation, good with 65–94%, fair with 25–64%, poor with 1–24% repigmentation of the treated area.
*Based on percentage change of depigmented area from baseline.


© 2014 European Academy of Dermatology and Venereology

**Table 4** Pairwise comparisons of therapies

| (I) group | (J) group | Mean difference (I–J) | Std. error | *P*-value* | 95% CI for difference* | |
|---|---|---|---|---|---|---|
| | | | | | Lower bound | Upper bound |
| Excimer | Non-cultured MKT | 2.084 | 1.158 | 0.08 | −1.23 | 5.40 |
| Excimer | Non-cultured MKT + excimer | 5.384 | 1.107 | <0.001† | 2.21 | 8.55 |
| Excimer | Control | −0.111 | 1.140 | 0.92 | −3.38 | 3.15 |
| Non-cultured MKT | Non-cultured MKT + excimer | 3.300 | 1.148 | 0.01† | 0.01 | 6.59 |
| Non-cultured MKT | Control | −2.195 | 1.149 | 0.07 | −5.49 | 1.10 |
| Non-cultured MKT + excimer | Control | −5.495 | 1.123 | <0.001† | −8.71 | −2.28 |

Based on estimated marginal means.
*Adjustment for multiple comparisons: Bonferroni.
†The mean difference of depigmented area (after controlling the effect of the depigmented area at baseline) is significant at the 0.05 level.

at the grafted area was retained during the follow-up period. The repigmentation rate in our experience also was lower than Mulekar *et al.*[12] study that in a 6-year follow-up experience on 142 patients with non-cultured MKT signified 56% excellent and 11% good repigmentation. Furthermore, Mulekar *et al.*[13] in another study compared focal and segmental vitiligo treated by MKT and showed higher repigmentation than our study (excellent results in 73% and 84% respectively).

Apart from expertise of the operating surgeon and method used, several factors impact the repigmentation after transplantation, such as segmental or general vitiligo (lower repigmentation in generalized than in segmental disease),[14] disease stability (lower repigmentation in active disease than in stable),[15] and site of the vitiligo patch (due to mobility of the part skin over joints, acral parts is difficult to treat surgically).[16]

In line with this guideline, all patients in our experience had stable (more than three years) generalized vitiligo disease, moreover, only three patches were on the skin over joints (knee & ankle). Hence, the reason for such a discrepancy between our experience and previous reports, despite clinical stability and normal values of organ specific antibodies, is not clear, but it might be related to active disease and technical errors.

In this study, seven patches (one in MKT, two in excimer and four in control group) did not show pigmentation or becoming worse after treatment that was in contrast with former reported long-term results that indicated loss of pigmentation occurred only in on patients with generalized vitiligo.[11–17] Earlier experiences signified the satisfaction in patients with generalized vitiligo is less than patients with segmental vitiligo. In addition, a poor outcome of treatment was also related to Koebner phenomenon, disease activity or lesions on the joint areas.[13,18] However, in a recent experience, Mulekar *et al.*[19] showed good results with epidermal cellular grafting on ‘more difficult-to-treat sites’ although several treatment sessions were necessary.

Our experience was a placebo control study and a group of patients only were under dermabrasion, the results in these patients showed poor repigmentation, so, dermabrasion was not confirmed to be productive for repigmentation, as was revealed by the absence of improvement in the control regions. This finding was in agreement with Kachhawa *et al.*[20] investigation in 2008, however, Toossi *et al.*[7] claimed some patients who had hyperpigmentation of the recipient and control sites, may benefit from dermabrasion as a treatment modality.

We started excimer 2 weeks after graft healing, based on previous studies that started PUVA therapy 3 weeks after autologous non-cultured epidermal cell transplantation.[21]

Melanocyte–keratinocyte transplantation technique for the treatment of vitiligo remains at best uncertain and inconclusive. Some clinical results seem controversial because the heterogeneity in patient samples, treatment procedures and trial design. Moreover, the literatures registered a great variability regarding equipment, experimental designs and techniques used in surgical treatment of vitiligo, and close attention should be paid to therapy parameters when reviewing and comparing these studies. Moreover, the most studies in this filed suffer from small sample size and does not allow us to draw definitive conclusions of melanocyte–keratinocyte transplantation technique.

Our study had some limitation as small sample size and short duration of follow-up that limit the ability to generalize the result of our survey. Further investigations are recommended with longer follow-up and larger series to validate the findings reported here.

Conclusion; although pigmentation in our patients was lower than previous reports from Iran and other countries. However, we signified adding excimer to MKT increased the pigmentation rate in treated patches.

## Acknowledgements

We thank the nursing, administrative and secretarial staff of the dermatology department and clinic at our hospital for their contribution to the maintenance of our patient record without which this project would have been impossible.

 © 2014 European Academy of Dermatology and Venereology

## References


1 Mulekar SV. Melanocyte-keratinocyte cell transplantation for stable vitiligo. *Int J Dermatol* 2003; **42**: 132–136.

2 Handa S, Dogra S. Epidemiology of childhood vitiligo: a study of 625 patients from North India. *Pediatr Dermatol* 2003; **20**: 207–210.

3 Lee B.W, Schwartz RA, Hercogová J, Valle Y, Lotti T.M. Vitiligo road map. *Dermatologic Therap* 2012; **25**: S44–S56.

4 Le Poole IC, Wan˜kowicz-Kalin˜ska A, van den Wijngaard RM *et al.* Autoimmune aspects of depigmentation in vitiligo. *J Investig Dermatol Symp Proc* 2004; **9**: 68–72.

5 Bahadoran P, Ortonne J-P. Classification of surgical therapies for vitiligo. In Gupta S, Olsson M, Kanwar A, Ortonne J-P, eds. Surgical Management of Vitiligo. Blackwell Publishing, Oxford, 2007: 59–68.

6 El-Zawahry BM, Zaki NS, Bassiouny DA, Sobhi RM, Zaghloul A. Autologous melanocyte–keratinocyte suspension in the treatment of vitiligo. *JEADV* 2011; **25**: 215–220.

7 Toossi P, Shahidi-Dadras M, Mahmoudi Rad M, Fesharaki RJ. Non-cultured melanocyte-keratinocyte transplantation for the treatment of vitiligo: a clinical trial in an Iranian population. *JEADV* 2011; **25**: 1182–1186.

8 Gauthier Y, Surleve-Bazeille JE. Autologous grafting with noncultured melanocytes: a simplified method for treatment of depigmented lesions. *J Am Acad Dermatol* 1992; **26**: 191–194.

9 Olsson MJ, Juhlin L. Leucoderma treated by transplantation of a basal cell layer enriched suspension. *Br J Dermatol* 1998; **138**: 644–648.

10 Pianigiani E, Risulo M, Andreassi A, Taddeucci P, Ierardi F, Andreassi L. Autologous epidermal cultures and narrow-band ultraviolet B in the surgical treatment of vitiligo. *Dermatol Surg* 2005; **31**: 155–159.

11 van Geel N, Wallaeys E, Goh BK, De Mil M, Lambert J. Long-term results of noncultured epidermal cellulargrafting in vitiligo, halo naevi, piebaldism and naevus depigmentosus. *British Association of Dermatologists* 2010; **163**: 1186–1193.

12 Mulekar SV. Long-term follow-up study of 142 patients with vitiligo vulgaris treated by autologous, non-cultured melanocyte– keratinocyte cell transplantation. *Int J Dermatol* 2005; **44**: 841–845.

13 Mulekar SV. Long-term follow-up study of segmental and focal vitiligo treated by autologous, noncultured melanocyte–keratinocyte cell transplantation. *Arch Dermatol* 2004; **140**: 1211–1215.

14 Gupta S, Kumar B. Epidermal grafting in vitiligo: influence of age, site of lesion, and type of disease on outcome. *J Am Acad Dermatol* 2003; **49**: 99–104.

15 Gupta S. Stability in vitiligo: why such a hullabaloo? *J Cutan Aesthet Surg* 2009; **2**: 41–42.

16 Parsad D, Gupta S. Standard guidelines of care for vitiligo surgery. *Indian J Dermatol Venereol Leprol* 2008; **74**(Suppl.): 37–45.

17 Olsson MJ, Juhlin L. Long-term follow-up of leucoderma patients treated with transplants of autologous cultured melanocytes, ultrathin epidermal sheets and basal cell layer suspension. *Br J Dermatol* 2002; **147**: 893–904.

18 van Geel N, Ongenae K, Vander Haeghen Y *et al.* Subjective and objective evaluation of noncultured epidermal cellular grafting for repigmenting vitiligo. *Dermatology* 2006; **213**: 23–29.

19 Mulekar SV, Al Issa A, Al Eisa A. Treatment of vitiligo on difficultto-treat sites using autologous noncultured cellular grafting. *Dermatol Surg* 2009; **35**: 66–71.

20 Kachhawa D, Kalla G. Keratinocyte-melanocyte graft technique followed by PUVA therapy for stable vitiligo. *Indian J Dermatol Venereol Leprol* 2008; **74**: 622–624.

21 van Geel N, Ongenae K, De Mil M, Naeyaert JM. Modified technique of autologous noncultured epidermal cell transplantation for repigmenting vitiligo: a pilot study. *Dermatol Surg* 2001; **27**: 873–876.


 © 2014 European Academy of Dermatology and Venereology
