# Supplementary material for: Therapeutic efficacy of cell-based therapy in vitiligo: a research letter systematically reviewed using meta-analysis
Source: Arch Dermatol Res. 2024 May 22;316(5):198. doi: 10.1007/s00403-024-02920-6 (PMC11111487; doi:10.1007/s00403-024-02920-6)
Supplement: Supplementary file 1 — Supplementary file1 (ZIP 24195 KB) [file 403_2024_2920_MOESM1_ESM.zip › Studies were included/El-Zawahry 2017.pdf]

Effect of Procedural-Related Variables on Melanocyte–Keratinocyte Suspension Transplantation in Nonsegmental Stable Vitiligo: A Clinical and Immunocytochemical Study

Bakr Mohamed El-Zawahry, MD,* Samia Esmat, MD,* Dalia Bassiouny, MD,* Naglaa Sameh Zaki, MD,* Rehab Sobhi, MD,* Marwah A. Saleh, MD,* Dalia Abdel-Halim, MD,* Rehab Hegazy, MD,* Heba Gawdat, MD,* Nesrin Samir, MD,* Marwa El-Hawary, MD,* Zeinab El Maadawi, MD,† Heba Gouda, MD,‡ and Mervat Khorshied, MD‡

BACKGROUND Melanocyte–keratinocyte suspension (M–K susp) is gaining popularity for vitiligo treatment. Few studies have addressed procedure-related variables.

OBJECTIVE To assess the effect of different M–K susp procedure-related variables on the clinical outcome in stable vitiligo.

METHODS This prospective multicenter comparative study included 40 cases with nonsegmental stable vitiligo. Donor site was either a skin graft in noncultured epidermal cell suspension (NCECS) or hair follicle units in outer root sheath hair follicle suspension (ORSHFS). Recipient site was prepared by either cryoblebbing or CO_2 laser resurfacing. Cell counts and viability were recorded in the cell suspensions. Tissue melanocytes and keratinocytes were examined by melan-A and cytokeratin, respectively. Assessment of repigmentation was performed 18 months after the procedure.

RESULTS Thirty-seven subjects completed the study. Cell count was significantly lower in the ORSHFS compared with NCECS with no significant difference in the repigmentation outcome. On comparing techniques of recipient site preparation, homogenicity was better in the CO_2 group. Elbows and knees responded better to CO_2 resurfacing, whereas distal fingers responded better to combination of cryoblebbing with NCECS.

CONCLUSION Using different techniques in M–K susp produces comparable results. However, the distal fingers showed better results using combination of donor NCECS and recipient cryoblebs.

The authors have indicated no significant interest with commercial supporters.

Noncultured melanocyte–keratinocyte suspension (M–K susp), first introduced by Gauthier and Surleve-Bazeille[1] and later modified by Olsson and Juhlin, in 2002[2,3] and Mulekar in 2004,[4] is commonly used in stable vitiligo not responding to medical treatment.

The donor tissue in noncultured M-K susp is either a skin graft in noncultured epidermal cell suspension (NCECS)[4] or hair follicle unit in outer root sheath hair follicle suspension (ORSHFS).[5] The recipient site can be prepared by several procedures including cryotherapy,[6] dermabrasion,[4] or carbon dioxide laser resurfacing.[7]

Several studies addressed the effect of patient-related variables on M–K susp transplantation in stable vitiligo.[4,8,9] In this study, the objectives were to assess the effects of procedural-related variables on repigmentation, namely donor tissue, its cytological composition, as well as recipient site preparation technique.

*Departments of *Dermatology, †Histology, and ‡Clinical Pathology, Kasr El-Ainy Teaching Hospital, Faculty of Medicine, Cairo University, Cario, Egypt*

© 2017 *by the American Society for Dermatologic Surgery, Inc. Published by Wolters Kluwer Health, Inc. All rights reserved.*
ISSN: 1076-0512 • Dermatol Surg 2017;43:226–235 • DOI: 10.1097/DSS.0000000000000962

© 2017 by the American Society for Dermatologic Surgery, Inc. Published by Wolters Kluwer Health, Inc. Unauthorized reproduction of this article is prohibited.

Methods

This prospective multicenter comparative study included 40 nonsegmental stable vitiligo cases treated surgically by noncultured M–K suspension. Patient recruitment was performed from the Dermatology Outpatient Clinic, Kasr El-Ainy Hospital, Cairo University and El-Zawahry Dermatology Clinic, Cairo during the period from January 2008 till January 2013. Patients were allocated to different study groups by 2 senior investigators. Surgical treatment was performed, and patient follow-up continued until June 2014. Inclusion criteria were as follows: stability for at least 1 year and resistance to medical therapy for a minimum of 6 months. Exclusion criteria were as follows: disease activity or keloidal tendency. Detailed history taking and clinical assessment were performed using vitiligo European task force (VETF),[10] vitiligo area and severity index (VASI),[11] and vitiligo disease activity (VIDA) scores.[12] Areas of the lesions to be treated were calculated by point counting technique.[13] Approval of the Dermatology Research Ethical Committee was obtained. All patients (or guardians of minors) signed informed written consents before surgical treatment. Patients were divided into 3 groups (Figure 1) to evaluate the effect of procedural-related variables on repigmentation (extent and homogenicity) as follows: (1) Comparison of NCECS and ORSHFS in donor area. Cell count, viability, and immunocytochemical staining for melanocytes and keratinocytes were also compared in both suspensions. (2) Comparison of cryoblebbing and CO_2 laser resurfacing in recipient area.

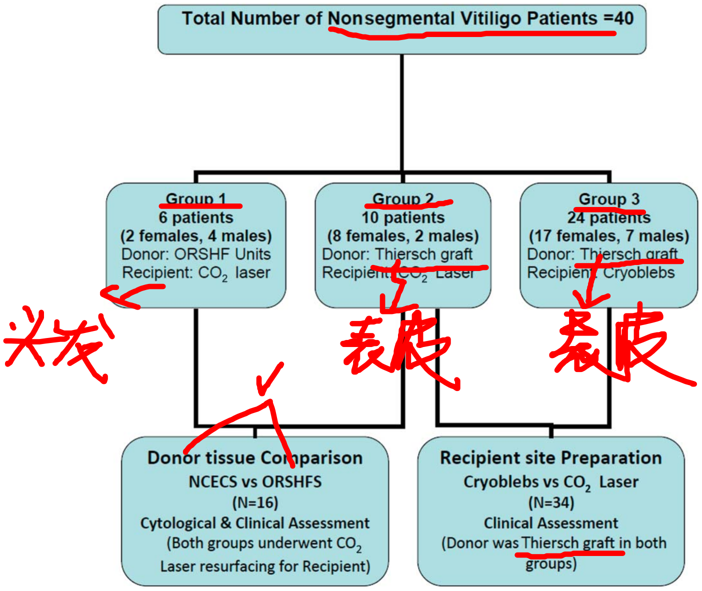

Figure 1. Study groups and design. A total of 40 nonsegmental stable vitiligo patients were divided into 3 groups according to M–K suspension transplantation techniques. CO_2, carbon dioxide. NCECS, noncultured epidermal cell suspension; ORSHFS, outer root sheath hair follicle suspension.

Donor Site

Thiersch Graft

An area 1/5 the area of the recipient site was shaved and excised using a hand dermatome from the patient's gluteal area or front of thigh. Local anesthesia by intralesional Mepecaine L (carpule: mepivacaine HCL 2% [NeüSkin, New Delhi, India] and levonordefrin 1: 20,000 [Alexandria Co. for Pharmaceuticals & Chemical Industries, Alexandria, Egypt]) was used. The donor site was covered by sterile petrolatum jelly gauze and adhesive tape for 1 week.

Hair Follicle Unit Harvesting

One pigmented HF was extracted per square centimeter of recipient skin (maximum number extracted was 50 HF) and collected in saline according to the technique described by Mohanty and colleagues.[5]

Preparation of Noncultured Autologous M–K Suspension

Noncultured Epidermal Cell Suspension

The skin graft was washed using saline and immersed in 0.25% trypsin-EDTA (GIBCO) solution for 40 minutes at 37°C. The sample and trypsin were poured into a petri dish then neutralized using 1% fetal bovine serum. The epidermis was separated from the dermis which was discarded. Then the epidermis was cut into tiny pieces, transferred to sterile falcon tubes and centrifuged for 20 minutes at 1,000 rpm.[14]

Outer Root Sheath Hair Follicle Suspension

The follicles were incubated with 0.25% trypsin-EDTA (GIBCO) at 37°C for 60 minutes divided into 3 intervals. The first interval lasted 30 minutes after which the supernatant fluid containing separated

© 2017 by the American Society for Dermatologic Surgery, Inc. Published by Wolters Kluwer Health, Inc. Unauthorized reproduction of this article is prohibited.

cells was poured into a new falcon tube and neutralized by 1% fetal bovine serum. Fresh trypsin was then added to hair follicles and reincubated for 15 minutes twice. Finally, thin keratinous hair shafts were left, which were discarded. Cell suspensions of all 3 stages were centrifuged for 15 minutes at 1,000 rpm to obtain a cell pellet. The reduction in the second and third trypsinization intervals are a modification of the original technique described by Mohanty and colleagues.[5]

Preparation of Suspension Before Application

The NCECS and ORSHFS cell pellets were suspended in a medium modified after Pandya and colleagues[15] and Olsson and Juhlin.[2] The volume of enriched medium depended on the method of donor site preparation.

Laser Resurfacing Cases. The cell pellet was resuspended in 1 to 2 mL of medium (1 mL/20 cm^2 recipient skin).

Cryobleb Cases. The cell pellet was resuspended in medium to which hyaluronic acid (Hyalift) was added in a ratio of 6:1 to get a homogenous viscid cellular suspension. Each bleb was injected by 0.1 mL of the mixture.[16]

Recipient Site Preparation and Transplantation

Cryoblebbing

Spraying liquid nitrogen for 5 to 8 seconds was performed 24 hours before transplantation using a plastic shield with 8-mm diameter circular opening. This created equal sized 10-mm diameter cryoblebs spaced 0.5 cm apart covering the whole area treated. The intact blebs were emptied by aspiration of the fluid inside followed by injection of M–K susp mixture.

CO_2 Laser Resurfacing

Using dot mode off resurfacing was performed at a power of 12 W, dwell time of 600 milliseconds for the face, trunk, wrists, or limbs and 20 W, dwell time 1,000 milliseconds for the dorsum of the hands and feet, knees, and fingers (DEKA, Florence, Italy). One to three passes were performed until the epidermis was removed uniformly. The suspension was then applied by a pipette and covered immediately by collagen sheets (NeüSkin, New Delhi, India).

Postprocedural Care

The recipient area was covered by sterile petrolatum jelly gauze, thick gauze soaked in medium of culture, and adhesive tape for 7 days. Patients were instructed to lie flat for 20 minutes to allow successful attachment of cells. Oral broad spectrum antibiotic was given for 10 days. The dressings were repeated for an additional week in cryoblebbing patients if the blebs did not heal.

Cytological and Immunocytochemical Assessment

Cell Count and Viability

Cells were counted manually by the hemocytometer. Viability was assessed by trypan blue dye exclusion test.

Immunocytochemical Staining

Pre-prepared cytospin slides with acetone-fixed cells were stained by ready-to-use mouse monoclonal anti-melan-A for melanocytes and mouse monoclonal anti-cytokeratin for keratinocytes (Genemed, CA). Universal Dako labeled Streptavidin-Biotin 2 system, Horseradish Peroxidase (LSAB2 System, HRP) (Dako, Carpinteria, CA) was used as a secondary antibody detection system. Mayer's hematoxylin was used for counter staining of nuclei. Negative control was concurrently included in which the primary antibody was omitted. Slides were examined by Olympus light microscope with digital camera (BX51; Olympus, Tokyo, Japan). Both antibodies showed cytoplasmic staining pattern. Positive cells were counted in 10 randomly selected nonoverlapping fields using ×1,000 magnification (~200 μL) (Figure 2).

Phototherapy

All cases started twice weekly narrow band ultraviolet B (NB-UVB) therapy 3 weeks after the procedure (UV1000L; Waldmann GmbH, Villingen-

© 2017 by the American Society for Dermatologic Surgery, Inc. Published by Wolters Kluwer Health, Inc. Unauthorized reproduction of this article is prohibited.

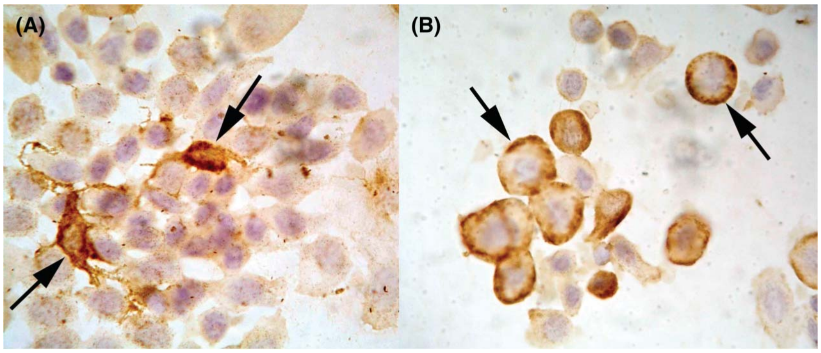

Figure 2. (A) Photomicrograph with arrows pointing at positive cytoplasmic-stained melanocytes with multiple dendritic cytoplasmic processes (melan-A immunocytochemistry ×1,000). (B) Photomicrograph with arrows pointing at positive cytoplasmic-stained epithelioid-shaped keratinocytes (cytokeratin immunocytochemistry ×1,000).

Schwenningen, Germany). Two cabins were used (placed opposite each other to cover patient's whole body) with 26 lamps.

Clinical Evaluation and Follow-up

Patients were followed up monthly for first 3 months then every 3 months for a total duration of 18 months. Clinical examination and digital photography was performed at each visit.

Primary outcome was assessment of the effects of procedural-related variables on repigmentation of each treated lesion, using a reversed VASI scoring[11] (0%: uniform depigmentation, 10%: specks of pigmentation, 25%: depigmented area > pigmentation achieved, 50%: pigmented area equaled the residual depigmented area, 75%: pigmentation achieved > residual depigmentation, 90%: few depigmented specks left and 100%: full repigmentation). The color match, homogenicity, and the onset of repigmentation were also noted. In addition, the overall repigmentation in each patient according to the repigmentation of the largest treated lesion was assessed. Cases with pigmentation ≥75% were considered responders. Secondary outcomes were patient satisfaction (graded as high, moderate, or poor), duration of wound healing, and complications such as scars or infection.

Statistical Methods

Data were statistically described in terms of mean ± SD, median and range, or frequencies and percentages when appropriate. Comparison of numerical variables between the study groups was performed using Mann–Whitney *U* test for independent samples. For comparing categorical data, Chi-square (χ^2) test was performed. Fisher exact test was used instead when the expected frequency is less than 5. Correlation between various variables was performed using Spearman rank correlation equation. *P* values less than 0.05 were considered statistically significant. All statistical calculations were performed using the computer program SPSS (Statistical Package for the Social Sciences; SPSS Inc., Chicago, IL) release 15 for Microsoft Windows (2006).

Results

The demographic data of the patients are summarized in Table 1. The VIDA score ranged from 0 to −1. A total of 182 vitiligo lesions were treated, 24 over the distal fingers, 93 in acral skin (dorsum of hands and feet, wrist, ankle, and proximal fingers), 41 over the joints (elbows, knees), 6 over the arms and legs, 12 over the trunk (including the breast), and 6 over the face with areas ranging from 0.5 to 55 cm^2. Of the 40 patients treated, 37 cases with 174 lesions returned for follow-up and were included in the analysis and 3 cases dropped out from Group 3 because of personal causes. Thirteen patients (35%) showed ≥75% repigmentation, 9 cases (24%) 50% repigmentation, 6 (16%) 25% repigmentation, 4 (11%) 10% repigmentation, and 5 (14%) cases showed no repigmentation at all.

© 2017 by the American Society for Dermatologic Surgery, Inc. Published by Wolters Kluwer Health, Inc. Unauthorized reproduction of this article is prohibited.

TABLE 1. Demographic and Clinical Data in Patient Groups

	Group 1, n = 6 Donor: ORSHFS Recipient: CO_2 Laser Resurfacing	Group 2, n = 10 Donor: Thiersch Graft Recipient: CO_2 Laser Resurfacing	Group 3, n = 24 Donor: Thiersch Graft Recipient: Cryoblebs	p
Age (mean ± SD)	28.3 ± 15.8	24.1 ± 8.1	22.4 ± 7.5	.786*
				.444†
Male	4 (67%)	2 (20%)	7 (29%)	.118*
Female	2 (33%)	8 (80%)	17 (71%)	.686†
Disease duration, median (range), yr	5 (3–10)	6 (2–23)	4 (2–8)	.581*
				.078†
Stability median (range), yr	2 (1–9)	1 (1–2)	1 (1–3)	.057*
				.481†
VETF area score	1.9 (0.5–13)	7.75 (0.5–37)	9 (0.5–37)	.175*
				.906†
VETF stage score median (range)	3 (2–6)	4 (1–7)	2 (1–6)	.781*
				.332†
VASI score median (range)	1.9 (0–7.8)	4.1 (0.3–10.3)	4.25 (0–14)	.278*
				.969†
No. of lesions treated median (range)	4.5 (1–6)	6.5 (1–12)	4 (1–8)	.227*
				.091†
Area treated, + median (range), cm^2	35 (5–50)	30 (12–160)	42 (5–127)	.514*
				.543†

p-value < .05 is statistically significant.
**p*-value between Groups 1 and 2.
†*p*-value between Groups 2 and 3.
ORSHFS, outer root sheath hair follicle suspension; SD, standard deviation; VASI, vitiligo area and severity index; VETF, vitiligo European task force.

Cytological and Immunocytochemical Composition of Noncultured Epidermal Cell Suspension Versus Outer Root Sheath Hair Follicle Suspension

Viability was comparable in both suspensions, whereas cell count was significantly higher in NCECS. The M–K ratio was also comparable in both preparations (Table 2).

Comparison of Effect of Donor Tissue Variation (Groups 1 and 2; Noncultured Epidermal Cell Suspension vs Outer Root Sheath Hair Follicle Suspension)

There was no significant difference in number of responders with ≥75% repigmentation (Table 3). All patients started to repigment homogenously within 3 months of the procedure. A good color match was observed in all 6 cases of ORSHFS and in 6/8 in the NCECS group. Donor site in the scalp healed faster with no apparent scars, whereas Thiersch graft site produced transient dyspigmentation in all cases and apparent scars in 4/10 cases.

Comparison of Effect of Recipient Site Preparation (Groups 2 and 3; CO_2 Resurfacing vs Cryoblebbing)

There was no significant difference in number of patients achieving ≥75% overall repigmentation. Only 3 patients reached 90% to 100% repigmentation and they were all in Group 3 (cryoblebbing) (Table 4). When the degree of response was assessed according to the site of lesion, there was a significant difference in response of distal finger lesions ($p \leq .0001$) (Table 5). Patients in both groups started to repigment homogenously within 3 months of the procedure. The repigmentation was color matched in both groups, but as expected was restricted to the area of the cryobleb in Group 3 cases which in large vitiliginous patches left

© 2017 by the American Society for Dermatologic Surgery, Inc. Published by Wolters Kluwer Health, Inc. Unauthorized reproduction of this article is prohibited.

TABLE 2. Cytological and Immunocytochemical Characteristics of NCECS Versus ORSHFS

	NCECS, N = 10	*ORSHFS, N = 5*	*p*
Percentage cell viability (mean ± SD)	74 ± 23.5	81 ± 17.4	.583
Total cell count $\times 10^3$ median (range)	2,537 (375–10,250)	130 (80–200)	.002*
Cell yield $\times 10^3/cm^2$ vs HFU donor tissue (mean ± SD)	364 ± 237	3.7 ± 1.4	.002*
Cells $\times 10^3/cm^2$ recipient area (mean ± SD)	95 ± 62	7 ± 6.5	.003*
Melanocytic count (melan-A)/10 fields	5 ± 3	4 ± 2	.742
Keratinocytic count (cytokeratin)/10 fields	25 ± 3	28 ± 3	.187
M:K ratio	0.2 ± 0.09	0.1 ± 0.05	.515

*p-value < .05 is statistically significant.
NCECS, noncultured epidermal cell suspension; ORSHFS, outer root sheath hair follicle suspension; SD, standard deviation.

white lines across the lesions (Figure 3). Scar at the donor site occurred in 8/21 cases of Group 3. Compared to CO_2 resurfacing, the cryoblebs took more time to heal and were more commonly infected. However, infected cases showed better repigmentation (Table 4).

Correlation of Percentage Repigmentation With Clinical Variables

There was a positive correlation between duration of stability and percentage repigmentation of the lesions in all groups which was significant in Groups 1 and 3 ($r = 1, p \le .0001$; $r = 0.427, p \le .0001$, respectively). A significant positive correlation was found between rate of pigmentation and disease duration only in Group 2 cases ($r = 0.469, p \le .001$). A negative correlation was found between VASI, and VETF area and stage scores and percentage repigmentation in all groups denoting that the larger the area of vitiligo the less favorable the response to surgery. This was significant as regards VASI score in Group 1 ($r = -0.567, p = .004$) and VETF area score in Group 3 ($r = -0.445, p \le .0001$). A significant correlation between the VETF area score

TABLE 3. Effect of Donor Tissue Variation on Clinical Outcome (Groups 1 and 2)

Clinical Outcome	*NCECS (Patient, n = 10; Lesion, n = 61)*	*ORSHFS (Patient, n = 6; Lesion, n = 24)*	*p*
Overall repigmentation in patients (%)			
90%–100%	0 (0)	1 (16.7)	.604*
75%	2 (20)	1 (16.7)	
50%	5 (50)	1 (16.7)	
25%	1 (10)	2 (33.2)	
10%	0 (0)	1 (16.7)	
0%	2 (20)	0 (0)	
Total no. lesions ≥ 75% repigmentation			
90%–100%	2	2	.753
75%	8	3	
Patient satisfaction			
High	2	1	.441
Moderate	2	3	
Poor	6	2	
Healing time median (range), wks	1.5 (1–2)	1 (1–2)	.529

p-value < .05 is statistically significant.
*p-value comparing patients with ≥75% repigmentation.
NCECS, noncultured epidermal cell suspension; ORSHFS, outer root sheath hair follicle suspension.

© 2017 by the American Society for Dermatologic Surgery, Inc. Published by Wolters Kluwer Health, Inc. Unauthorized reproduction of this article is prohibited.

TABLE 4. Effect of Recipient Site Preparation on Clinical Outcome (Groups 2 and 3)

Clinical Outcome	*CO_2 Laser (Patient, n = 10; Lesion, n = 61)*	*Cryoblebbing (Patient, n = 21; Lesion, n = 89)*	*p*
Overall repigmentation in patients (%)			
90%–100%	0 (0)	5 (24)	.262*
75%	2 (20)	4 (19)	
50%	5 (50)	3 (14)	
25%	1 (10)	3 (14.3)	
10%	0 (0)	3 (14.3)	
0%	2 (20)	3 (14.3)	
Total no. lesions ≥75% repigmentation			
90%–100%	2	20	.001*
75%	8	18	
Patient satisfaction			
High	2	5	.803
Moderate	2	6	
Poor	6	10	
Healing time median (range), wk	1.5 (1–2)	1.5 (1–5)	.002*
Complications	Infection (*N* = 1)	Infection (*N* = 6)	.3871

p-value < .05 is statistically significant.
**p*-value comparing patients with ≥75% repigmentation.

and the area treated (Group 1: r = 0.416, *p* = .043; Group 2: r = 0.264, *p* = .040; Group 3: r = 0.294, *p* = .004). This can be expected as the larger the area affected the more likely the larger area of vitiligo requiring therapy. No significant correlation was present between size of the lesions and repigmentation in all groups. A significant difference was seen on comparing repigmentation in different sites within the group. In Group 1, lesions over the face, elbows, and knees responded better than those over the acral skin (*p* = .02). In Group 2, lesions over the trunk, elbows, and knees responded better than those over the distal fingers and acral skin (*p* = <.001), whereas in Group 3 cases the best responding lesions were over the legs, trunk, and distal fingers (*p* ≤ .001).

Discussion

This study focused on procedural variables that could influence the surgical outcome, namely the donor and recipient sites' preparation. The authors documented no significant differences between the implemented techniques (NCECS vs ORSHFS in the donor sites and CO_2 laser resurfacing vs cryoblebbing in the recipient sites) regarding the final achieved repigmentation. However, the distal fingers were an exception, as combining NCECS and cryoblebbing showed significantly better results in comparison with NCECS and CO_2 laser resurfacing. A limitation is that none of the distal finger cases underwent ORSHFS to complete the picture.

The adopted techniques of donor tissue harvesting had no influence on either the viability or M–K ratio. The comparable M:K ratio in both suspensions is likely due to the fact that the pellet in epidermal suspension is composed of cells from the stratum basale and the lower half of the stratum spinosum, which are rich in melanocytes. There is on average, 1 basal melanocyte for every 10 basal keratinocytes in double covered buttock skin. Ultraviolet-exposed skin possess approximately twice as many basal melanocytes as covered skin[17] and some authors reported an increase in melanocyte numbers in covered epidermis some days after ultraviolet exposure of surrounding skin.[18] Most of the cases were on nbUVB phototherapy for vitiligo which may explain the elevated melanocytic count in the NCECS in this work. However, despite

© 2017 by the American Society for Dermatologic Surgery, Inc. Published by Wolters Kluwer Health, Inc. Unauthorized reproduction of this article is prohibited.

TABLE 5. Repigmentation According to Site in Different Groups

Site	*90%–100%*	*75%*	*50%*	*25%*	*10%*	*0%*	*Total*
Group 1 (donor ORSHFS, recipient: CO_2 laser)							
Distal fingers	—	—	—	—	—	—	No lesions
Acral	0	0	2	11	0	0	13
Over joints	1	3	0	4	0	0	8
Trunk/breast	—	—	—	—	—	—	No lesions
Face/neck	1	0	0	0	1	1	3
Group 2 (donor NCECS, recipient: CO_2 laser)							
Distal fingers	**0**	0	0	0	0	14	14
Acral	2	2	3	18	0	9	34
Over joints	0	3	6	0	0	0	9
Legs/arms	0	0	1	0	0	0	1
Trunk/breast	0	3	0	0	0	0	3
Face/neck	—	—	—	—	—	—	No lesions
Group 3 (donor: NCECS, recipient: cryoblebbing)							
Distal fingers	10*	0	0	0	0	0	10
Acral	3	8	8	6	8	8	41
Over joints	2	6	10	2	2	0	22
Legs/arms	3	2	0	0	0	0	5
Trunk/breast	1	2	0	0	2	3	8
Face/neck	1	0	0	1	0	1	3

p-value < .05 is statistically significant.
*Significantly better compared with CO_2 laser resurfacing.
NCECS, noncultured epidermal cell suspension; ORSHFS, outer root sheath hair follicle suspension.

the comparable M:K ratio, a larger number of melanocytes were present in the NCECS due to significantly higher cell count. The significantly higher total cell count yielded by NCECS in comparison with ORSHFS may be attributed to the larger surface area of skin used to prepare NCECS. Intriguingly, this did not influence the repigmentation outcome. The speculated cell requirement for each square centimeter (2,000 cells/cm^2)—which was exceeded by both techniques in this study—could explain the comparable achieved repigmentation, despite the difference in cell count.[5,19] The existence of a higher variety of cell populations including melanocyte stem cells in ORSHFS compared with NCECS[20,21] might represent an additional explanation for the comparable repigmentation, thus compensating for the lower cell count, that is quality versus quantity. Moreover, hair melanocytes have remarkable synthetic capacity, and a relatively small number of melanocytes can potentially produce sufficient melanin to pigment up to 1.5 m of hair shaft.[22] In agreement with Singh and colleagues,[23] the clinical parameters (VASI, start of

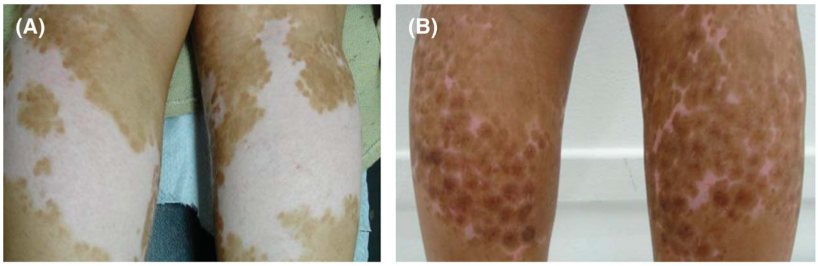

Figure 3. A female patient showing >75 repigmentation. (A) Before, (B) after 18 months of treatment. Recipient site was prepared using cryoblebs. Donor site was noncultured epidermal cell suspension.

© 2017 by the American Society for Dermatologic Surgery, Inc. Published by Wolters Kluwer Health, Inc. Unauthorized reproduction of this article is prohibited.

TABLE 6. Pros and Cons of Techniques of Tissue Preparation in Donor and Recipient Sites

Donor Sites	*NCECS*	*ORSHFS*
Advantages	Faster procedure for large areas<br>Higher cell count	Faster healing<br>No visible scar
Disadvantages	Delayed healing<br>Hyperpigmentation or scaring	Harvesting is time consuming<br>Not suitable in leukotrichia
Recipient Sites	*Cryoblebs*	*CO_2 Laser Resurfacing*
Advantages	Adequate separation of acral skin<br>Good cosmetic appearance in fingers	Faster healing<br>Homogenous repigmentation of large patches
Disadvantages	Performed 24 h before<br>Long healing time<br>Infection more common<br>Repigmentation restricted to the area of the bleb (pigmentation patchy in large lesions)	Difficult to perform on acral skin especially fingers

NCECS, noncultured epidermal cell suspension; ORSHFS, outer root sheath hair follicle suspension.

repigmentation and color match) did not show significant differences between the adopted techniques in donor site. This study is the first to compare the effect of fractional CO_2 laser and cryoblebbing on the extent of repigmentation. On analyzing the results, the distal phalanges of fingers yielded better response on combining cryoblebbing of recipient site and NCECS graft which could be attributed to the better tissue separation presented by cryoblebbing. In this study, the extent of repigmentation achieved by both NCECS and ORSHFS was lower and less evident than has been demonstrated by others[9,23,24] where 16.4% of NCECS lesions and 20.8% of ORSHFS lesions showed successful repigmentation (≥75%) which could be attributed to the fact that 61.5% of the lesions were acral with reportedly less favorable response.[24] Better selection of lesions to be treated might yield higher extent of repigmentation as suggested by Vinay and colleagues,[9] and Benzekri and colleagues.[25]

The rate of infection that was significantly higher with cryoblebbing is probably due to the moist nature of the cryoblebs and the longer healing duration. The pros and cons of each technique are illustrated in Table 6. Interestingly, infected lesions demonstrated faster repigmentation. This may be due to the upregulation of the matrix metalloproteinases (MMPs) known to occur with infections.[26] Increased MMP-2 and MMP-9 activity has been shown to increase the migration of melanocyte precursors (melanoblasts) from the outer root sheath of hair follicles, or the migration of melanocytes from the border of vitiligo lesions into the depigmented epidermis.[27] Several cytokines released during inflammatory reaction to infection also have melanocyte-stimulating properties such as leukotrienes (LT-C4 and LT-D4), prostaglandins E2 and D2, thromboxane-2, interleukin (IL)-1, IL-6, tumor necrosis factor-α, and epidermal growth factor.[28]

Different surgical procedures do not have a significant influence on the resultant repigmentation. In donor sites, NCECS showed higher cell count and ORSHFS showed better healing. Regarding recipient sites, CO_2 laser resurfacing showed faster healing and more homogenous pigmentation than cryoblebbing. The distal fingers were an exception showing significantly better results on combining NCECS with cryoblebbing.

Acknowledgments The authors thank Prof. W. Mostafa for her help in final editing of the manuscript; Dr. A. Zaghloul for his help in follow-up of some of the cases, Ms. S. Khabbar; Rheotic and Composition Instructor, The Academy of Liberal Arts, The American University in Cairo; for her kind

© 2017 by the American Society for Dermatologic Surgery, Inc. Published by Wolters Kluwer Health, Inc. Unauthorized reproduction of this article is prohibited.

help in reviewing this manuscript, Mrs. N. Mohamed for her help in viability and cell count assessment, and Ms. A. Ahmed for her help in preparation of the immunocytochemical slides.

References

1. Gauthier Y, Surleve-Bazeille JE. Autologous grafting with noncultured melanocytes: a simplified method for treatment of depigmented lesions. J Am Acad Dermatol 1992;26:191–4.
2. Olsson MJ, Juhlin L. Leucoderma treated by transplantation of a basal cell layer enriched suspension. Br J Dermatol 1998;138:644–8.
3. Olsson MJ, Juhlin L. Long-term follow-up of leucoderma patients treated with transplants of autologous cultured melanocytes, ultrathin epidermal sheets and basal cell layer suspension. Br J Dermatol 2002; 147:893–904.
4. Mulekar SV. Long-term follow-up study of segmental and focal vitiligo treated by autologous, noncultured melanocyte-keratinocyte cell transplantation. Arch Dermatol 2004;140:1211–5.
5. Mohanty S, Kumar A, Dhawan J, Sreenivas V, et al. Noncultured extracted hair follicle outer root sheath cell suspension for transplantation in vitiligo. Br J Dermatol 2011;164:1241–6.
6. Czajkowski R, Placek W, Drewa T, Kowaliszyn B, et al. Autologous cultured melanocytes in vitiligo treatment. Dermatol Surg 2007;33: 1027–36: discussion 35–6.
7. Chen YF, Yang PY, Hu DN, Kuo FS, et al. Treatment of vitiligo by transplantation of cultured pure melanocyte suspension: analysis of 120 cases. J Am Acad Dermatol 2004;51:68–74.
8. van Geel N, Wallaeys E, Goh BK, De Mil M, et al. Long-term results of noncultured epidermal cellular grafting in vitiligo, halo naevi, piebaldism and naevus depigmentosus. Br J Dermatol 2010;163:1186–93.
9. Vinay K, Dogra S, Parsad D, Kanwar AJ, et al. Clinical and treatment characteristics determining therapeutic outcome in patients undergoing autologous non-cultured outer root sheath hair follicle cell suspension for treatment of stable vitiligo. J Eur Acad Dermatol Venereol 2015;29:31–7.
10. Taieb A, Picardo M. The definition and assessment of vitiligo: a consensus report of the Vitiligo European Task Force. Pigment Cell Res 2007;20:27–35.
11. Hamzavi I, Jain H, McLean D, Shapiro J, et al. Parametric modeling of narrowband UVB phototherapy for vitiligo using a novel quantitative tool: the Vitiligo Area Scoring Index. Arch Dermatol 2004;140:677–83.
12. Bhor U, Pande S. Scoring systems in dermatology. Indian J Dermatol Venereol Leprol 2006;72:315–21.
13. Aydin F, Senturk N, Sahin B, Bek Y, et al. A practical method for the estimation of vitiligo surface area: a comparison between the point counting and digital planimetry techniques. Eur J Dermatol 2007;17:30–2.
14. van Geel N, Ongenae K, De Mil M, Naeyaert JM. Modified technique of autologous noncultured epidermal cell transplantation for repigmenting vitiligo: a pilot study. Dermatol Surg 2001;27: 873–6.
15. Pandya V, Parmar KS, Shah BJ, Bilimoria FE. A study of autologous melanocyte transfer in treatment of stable vitiligo. Indian J Dermatol Venereol Leprol 2005;71:393–7.
16. El-Zawahry BM, Zaki NS, Bassiouny DA, Sobhi RM, et al. Autologous melanocyte-keratinocyte suspension in the treatment of vitiligo. J Eur Acad Dermatol Venereol 2011;25:215–20.
17. Fallowfield ME, Curley RK, Cook MG. Melanocytic lesions and melanocyte populations in human epidermis. Br J Dermatol 1991;124:130–13.
18. Stierner U, Rosdahl I, Augustsson A, Kagedal B. UVB irradiation induces melanocyte increase in both exposed and shielded human skin. J Invest Dermatol 1989;92:561–4.
19. Tegta GR, Parsad D, Majumdar S, Kumar B. Efficacy of autologous transplantation of noncultured epidermal suspension in two different dilutions in the treatment of vitiligo. Int J Dermatol 2006; 45:106–10.
20. Tobin DJ, Paus R. Graying: gerontobiology of the hair follicle pigmentary unit. Exp Gerontol 2001;36:29–54.
21. Gho CG, Braun JE, Tilli CM, Neumann HA, et al. Human follicular stem cells: their presence in plucked hair and follicular cell culture. Br J Dermatol 2004;150:860–8.
22. Legue E, Sequeira I, Nicolas JF. Hair follicle renewal: authentic morphogenesis that depends on a complex progression of stem cell lineages. Development 2010;137:569–77.
23. Singh C, Parsad D, Kanwar AJ, Dogra S, et al. Comparison between autologous noncultured extracted hair follicle outer root sheath cell suspension and autologous noncultured epidermal cell suspension in the treatment of stable vitiligo: a randomized study. Br J Dermatol 2013;169:287–93.
24. Holla AP, Sahni K, Kumar R, Kanwar A, et al. Repigmentation of leukotrichia due to retrograde migration of melanocytes after noncultured epidermal suspension transplantation. Dermatol Surg 2014;40:169–75.
25. Benzekri L, Gauthier Y, Hamada S, Hassam B. Clinical features and histological findings are potential indicators of activity in lesions of common vitiligo. Br J Dermatol 2013;168:265–71.
26. Elkington PT, O'Kane CM, Friedland JS. The paradox of matrix metalloproteinases in infectious disease. Clin Exp Immunol 2005;142:12–20.
27. Kumar R, Parsad D, Kanwar AJ, Kaul D. Altered levels of Ets-1 transcription factor and matrix metalloproteinases in melanocytes from patients with vitiligo. Br J Dermatol 2011;165:285–91.
28. Davis EC, Callender VD. Postinflammatory hyperpigmentation: a review of the epidemiology, clinical features, and treatment options in skin of color. J Clin Aesthet Dermatol 2010;3:20–31.

Address correspondence and reprint requests to: Dalia Bassiouny, MD, 51b Damascus Street, Mohandessien, Cairo, Egypt 11214, or e-mail: daliabas73@yahoo.com

© 2017 by the American Society for Dermatologic Surgery, Inc. Published by Wolters Kluwer Health, Inc. Unauthorized reproduction of this article is prohibited.
