# Supplementary material for: Therapeutic efficacy of cell-based therapy in vitiligo: a research letter systematically reviewed using meta-analysis
Source: Arch Dermatol Res. 2024 May 22;316(5):198. doi: 10.1007/s00403-024-02920-6 (PMC11111487; doi:10.1007/s00403-024-02920-6)
Supplement: Supplementary file 1 — Supplementary file1 (ZIP 24195 KB) [file 403_2024_2920_MOESM1_ESM.zip › Studies were included/Gunaabalaji, D. R.2020.pdf]

International Journal of Dermatology

Dermatologic surgery

# Comparison of efficacy of noncultured hair follicle cell suspension and noncultured epidermal cell suspension in repigmentation of leukotrichia and skin patch in vitiligo: a randomized trial

**D. R. Gunaabalaji[1], MD, Rashi Pangti[1], MD, Apoorva Challa[1], MSc, Suman Chauhan[1], MSc, Kanika Sahni[1], MD, Sudheer Kumar Arava[2], MD, Gomathy Sethuraman[1], MD, Sreenivas Vishnubhatla[3], MD, Vinod Kumar Sharma[1], MD and Somesh Gupta[1], MD**

[1]Department of Dermatology and Venereology, All India Institute of Medical Sciences, New Delhi, [2]Department of Pathology, All India Institute of Medical Sciences, New Delhi, and [3]Department of Biostatistics, All India Institute of Medical Sciences, New Delhi, India

**Correspondence**
Somesh Gupta, MD
Department of Dermatology & Venereology
All India Institute of Medical Science
New Delhi
India
E-mail: someshgupta@hotmail.com

Conflict of interest: None.

Funding source: Indian Council of Medical Research.

Clinical Trial Registry of India (CTRI): CTRI/2017/11/010338.

doi: 10.1111/ijd.15188

## Abstract

**Background** Vitiligo manifests as hypo- to de-pigmented macules, which are sometimes associated with leukotrichia. For complete cosmetic improvement, the repigmentation of leukotrichia is an important component.

**Methods** This randomized controlled trial included patients with stable vitiligo with leukotrichia. Two vitiligo patches in each patient were randomized to receive either of the two procedures. The patients were followed up for 9 months posttransplantation. The efficacy of hair follicle cell suspension (HFCS) with epidermal cell suspension (ECS) in repigmentation of leukotrichia and skin in vitiligo was compared.

**Results** A total of 20 patients underwent the procedure, and 19 completed the follow-up. The area of the vitiligo patch and the number of leukotrichia in the patches were comparable between the two groups. There was a significant difference in the mean ± S.D. number of cells transplanted between the two groups ($5.06 \times 10^5$ in HFCS vs. $39.8 \times 10^5$ in ECS, $P < 0.0001$). The percentage viability of cells and proportion of melanocytes were comparable between the two groups. A total of 10 patients in HFCS and eight patients in ECS had repigmentation of leukotrichia. The mean ± S.D. percentages of depigmented hair showing repigmentation at nine months were 7.42 ± 11.62% in HFCS and 11.42 ± 17.90% in ECS ($P = 0.4195$), whereas the mean ± S.D. percentage repigmentation of vitiligo patches was 61.58 ± 42.68% in HFCS and 78.68 ± 30.03% in ECS ($P = 0.1618$).

**Conclusions** The mean number of cells transplanted in the HFCS group was about eight times less than those in ECS. ECS was better than HFCS in repigmentation of leukotrichia and vitiligo, although the difference was not statistically significant.

## Introduction

Vitiligo is a disease with considerable psychosocial morbidity, especially in people with skin of color. Various therapeutic options, including medical and surgical, are available for patients with vitiligo. Hair follicles are an important source of melanocytes for vitiligo repigmentation as demonstrated by the perifollicular repigmentation occurring after NBUVB therapy.[1] Leukotrichia tends to occur during early stages in segmental vitiligo and late stages in other forms. Leukotrichia had been observed in frequencies of 9.2[2] to 32.7%[3] in studies comprised of all types of vitiligo, whereas it was noted in all patients with segmental vitiligo.[4] The presence of leukotrichia indicates a loss of melanocyte reservoir and is considered a marker of poor response to medical treatment.[4] In patients with stable vitiligo, surgery can be considered, which works on the principle of transferring the intact melanocytes from a pigmented site to a depigmented site, which is immunologically stable enough to enable the transplanted melanocytes to survive and produce pigment. The noncultured epidermal cell suspension (ECS) transplantation technique was introduced by Gauthier and Surleve-Bazeille in 1992.[5] Vanscheidt and Hunziker, in a small case series, used the single-cell suspension of plucked hair follicles in the treatment of vitiligo in 2009,[6] which was further refined by Mohanty *et al.* in 2011 by extracting the hair follicle and using it as a source of melanocytes.[7]

Persistence of leukotrichia after achieving complete repigmentation of skin with treatment is associated with both cosmetic concerns and psychosocial burden.[8] There are no prospective



© 2020 the International Society of Dermatology 

studies of repigmentation of leukotrichia with any of the surgical transplantation techniques except follicular unit transplantation. Epidermis and hair follicle are two available sources of melanocytes for transplantation in vitiligo. We report the results of a randomized controlled trial of two cell-based therapies, ECS and hair follicle cell suspension (HFCS), for repigmentation of leukotrichia.

## Materials and methods

### Study design and participants

This single-center randomized controlled study (open-label) was conducted between 2017 and 2019 in the All India Institute of Medical Sciences, New Delhi, India. All participants underwent a single-day procedure with nine months for the follow-up period. All patients 18 years of age and older with a minimum of one vitiligo patch, a minimum of 15 leukotrichia hairs, and with the stability of at least one year were included in the study. Patients with a history of koebnerization, keloid formation, bleeding diathesis, active infection at either donor or recipient site, and those with hair disorders were excluded from the study. Written informed consent was obtained from all patients participating in the study. The study was approved by the Ethics Committee of the All India Institute of Medical Sciences. At baseline, the boundary of the vitiligo patch was marked on a transparent sheet for area calculation. Two patches with comparable areas were included in the study, or one large patch was divided into two equal halves. High-definition clinical photographs were taken, and leukotrichia was mapped in the zoomed-in picture, and this was repeated subsequently on every follow-up visit.

### Sample size calculation

A previous retrospective study on repigmentation of leukotrichia reported the percentage of study population achieving some repigmentation of leukotrichia with ECS as 45.23%.[9] With 95% confidence levels and 80% power, presuming that there will be 50% more repigmentation with HFCS, the recommended sample size was calculated to be 70 patches in 35 patients. However, it was decided to keep a sample size of 40 patches in 20 patients given the limited study duration (Fig. 1).

### Randomization

In the case of two patches, upper/right vitiligo lesion was designated as number 1 and lower/left one was designated as number 2. In the case of a single lesion, the lesion was divided into two, perpendicular to the long axis of the lesion, and numbered (upper 1 and lower 2 if it was vertically oriented, and right as 1 and left as 2 if it was horizontally oriented). Block randomization (with a block of 2) by computer-generated sequence was done.

### Procedure

For autologous ECS, a thin split thickness skin graft was taken from the donor site (thigh) with the help of a blade held in Kocher's forceps under strict aseptic precautions after topical anesthesia with a eutectic mixture of lidocaine and prilocaine. The donor site was dressed with chlorhexidine impregnated gauze and sterile cotton pads. The skin graft was transferred to Trypsin-EDTA (ethylenediamine tetraacetic acid) solution (0.25% trypsin and 0.05% EDTA, Gibco BRL) in a petri dish, incubated at 37°C for 40 minutes, the trypsin–EDTA solution removed after 2 hours, and trypsin inhibitor was added, then Ringer's lactate added and the tissue teased with sterile forceps to separate the cells. The suspension made was centrifuged at 2,500 rpm for 5 minutes and the pellet obtained.

For HFCS, follicular unit extraction was done from the occipital scalp. Few hairs were delineated in this area in a square of approximately 10*10 cm and trimmed to 2 mm. Field block anesthesia using 2% lignocaine with adrenaline was given encircling the delineated donor area. With injection of normal saline, tumescence was achieved in the donor area. With the help of 0.7 mm motorized punch, intact hair follicle units were extracted *in toto*. These follicles were washed three times in Ringer's lactate solution and incubated with trypsin–EDTA solution at 37°C for 90 minutes. The hair follicles were placed in a new test-tube of trypsin–EDTA every 30 minutes and the reaction in the previous tube terminated by adding trypsin inhibitor (Sigma-Aldrich). The cell suspensions of all three tubes were then put in a single tube and filtered through a 70 μm cell strainer (Becton Dickinson, Sunnyvale, CA, U.S.A.). The cell suspension was then centrifuged for 10 minutes at 2,500 rpm to obtain a cell pellet.

The pellet was resuspended in 250–500 microlitres of DMEM depending on the size of the recipient patch and transplanted onto the recipient site.[10]

### Transplantation procedure

Local anesthesia with 2% lignocaine was given to the area to be treated. Recipient site was dermabraded with motorized dermabrader until tiny bleeding points appeared.

The cell suspension was evenly spread over the recipient site using a pipette followed by application of dressing composed of dry collagen sheet (NeuSkin-F®, Eucare, Chennai, India), surgical cotton gauze/pad, thin transparent dressing film (Tegaderm®), and an elastic adhesive bandage from inside out.

Systemic antibiotics were prescribed for 5–7 days. The dressing was removed on the 7th postoperative day. Topical tacrolimus 0.1% ointment and sun exposure daily for 10 minutes was started on the 14th day postoperatively. The patients received no other adjuvant topical, systemic, or phototherapy.

### Laboratory evaluation

#### *Viability and cell count*

Viability was tested adding trypan blue on a hemacytometer and examining and counting under the microscope.

 © 2020 *the International Society of Dermatology*

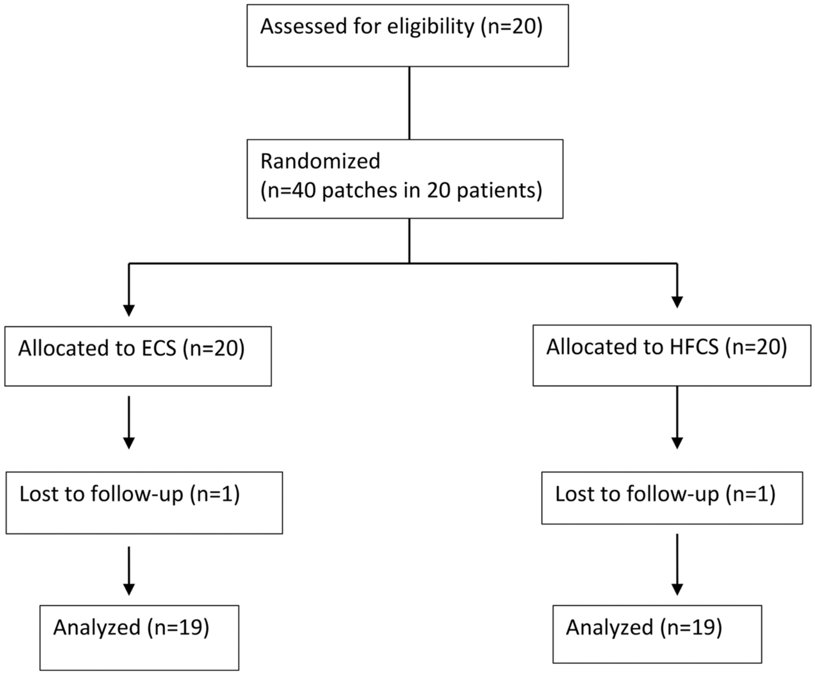


**Figure 1** Flowchart showing the methodology of the study

### *Immunohistochemistry*

For quantifying melanocytes, 50 microliters of samples from both suspensions were taken, and smear on a slide was done on the same day using cytospin chamber at 1,100 rpm for 3 minutes and fixed with 95% ethyl alcohol. The samples were stained using standard procedures for immunohistochemistry with HMB-45 and S100 as primary antibodies, horseradish peroxidase (HRP) as the enzyme, and Dab as substrate. Each slide was examined under microscope, and the number of stained and unstained cells were counted for calculating the percentages of melanocytes in the suspension.

### Outcome measures

Patients were followed up at 1, 3, 5, 7, and 9 months (Fig. 1). Clinical evaluation of repigmentation of leukotrichia hair and vitiligo patches was done at each follow-up visit. The boundary of the vitiligo patch was marked on a transparent sheet, and area calculation was done to assess repigmentation. Percentage repigmentation of the vitiligo patch was done and the number of repigmented leukotrichia hair was counted on zoomed-in photographs by a single observer. On a scale of 0–10, patients were asked to score the degree of repigmentation of both vitiligo patch and leukotrichia.

### Statistical analysis

The baseline characteristics including the area of vitiligo patch, number of leukotrichia, and characteristics of suspensions prepared including the size of the donor area, number of hair follicles extracted, number of viable cells, percentage viability of the cells, and percentage of melanocytes were tabulated and compared between the two groups using the Student's *t* test/ Mann-Whitney U test. Similarly, outcome parameters including repigmentation of leukotrichia, vitiligo patches, and color match were compared using Student's *t*-test/Mann-Whitney U test. The results were considered significant at a 5% level of significance. The data were tabulated for both groups, and the statistical analysis was done using Stata version 15.0.

## Results

Of 20 patients, 13 were male and seven were female; 15 had segmental vitiligo, two had nonsegmental, and three had mixed vitiligo. The surgical site of vitiligo was head and neck in 17 patients, whereas it was lower limb in two patients and trunk in one patient.

The mean age of patients was 23.9 $\pm$ 6.13 years (range 18–47). The mean duration of disease was 9.05 $\pm$ 5.22 years (range: 1.5–22), and the mean duration of stability was 5.225 $\pm$ 5.07 years (range: 1–12). The baseline characteristics of the patients are summarized in Table 1.

## Immunohistochemistry

The percentage of HMB-45 stained cells ranged from 5.55 to 53.33% (mean $\pm$ S.D. 16.66 $\pm$ 11.8) in ECS and from 3.84 to 50% (mean $\pm$ S.D. 14.62 $\pm$ 11.3) in HFCS. S100 stain also showed similar results, ranging from 6.35 to 55% (mean $\pm$ S.D. 17 $\pm$ 11.35) in ECS and 6.12 to 41.67% (mean $\pm$ S.D.

© 2020 *the International Society of Dermatology* 

13.7 ± 7.9) in HFCS. The difference between the percentage of melanocytes in the suspension between the two groups was not statistically significant.

## Repigmentation of leukotrichia

A total of 10 of 19 patches in HFCS and 8 of 19 patches in ECS had some repigmentation of leukotrichia at their final visit at 9 months (Fig. 2). Time taken to leukotrichia repigmentation ranged from 3 to 7 months in ECS and from 1 to 9 months in HFCS.

The average repigmentation of leukotrichia at each visit did not show any statistically significant difference between the two groups (Fig. 3). Mean ± S.D. percentage repigmentation in leukotrichia in HFCS and ECS groups was 7.42 ± 11.62 and 11.42 ± 17.90, respectively ($P = 0.4195$).

## Repigmentation of vitiligo

Among the 20 patients, 18 had repigmentation of vitiligo in the ECS group, and 15 patients had repigmentation in the HFCS group (Fig. 2). The average repigmentation of vitiligo patch is shown in Figure 3. At 9 months, mean ± S.D. percentage repigmentation in vitiligo patch in HFCS and ECS groups was 61.58 ± 42.68 and 78.68 ± 30.03, respectively ($P = 0.1618$). All the patients who had repigmentation, showed repigmentation at the first month itself. In ECS, 14 of 20 (70%) patients had >75% repigmentation. In HFCS, 10 of 20 (50%) patients had >75% repigmentation of vitiligo at their last visit.

## Adverse effects

Pain at donor site for HFCS was noted by three patients which resolved by the 1month follow-up visit. Discharge at recipient site for both HFCS and ECS was noted by two patients which resolved within one week with oral antibiotics.

## Patient preference

Of the 18 patients who had repigmentation in at least one patch of vitiligo, only two patients preferred HFCS as the procedure of choice, and 16 patients preferred ECS.

## Discussion

Vitiligo is a common depigmenting disorder with significant social and psychological morbidity. Even when the vitiligo patch gets repigmented, the persistence of leukotrichia causes distress to the patient; hence, the repigmentation of leukotrichia is an important goal of treatment. Leukotrichia in vitiligo is considered to be a poor prognostic sign.[8] The presence of leukotrichia indicates that the reservoir of melanocytes for repigmentation for medical therapies has been depleted since the repigmentation from the peripheral normally pigmented skin is very minimal.[11]

In this study, there was a greater number of cells transplanted in ECS than in HFCS; however, there was no statistically significant difference in repigmentation of leukotrichia or vitiligo patch between both the groups. Although the number of cells transplanted in the HFCS group was about eight times less than in the ECS group, we found comparable repigmentation in both groups. There is a recognized niche for melanocyte stem cells in hair follicles but not in the epidermis. We hypothesize that these stem cells also contributed to the repigmentation of the epidermis and leukotrichia.

There are some retrospective studies reporting repigmentation of leukotrichia with ECS and other surgical techniques (Table 2), however, no reports on repigmentation of leukotrichia with HFCS are available. Hair follicles act as an important reservoir for both melanocytes and melanocyte stem cells.[12] Other surgical methods where repigmentation of leukotrichia is reported in the literature are follicular unit extraction, suction blister grafting, minigrafting, thin split-thickness skin grafting, and electroepilation (Table 2).

**Table 1** Baseline characteristics of patients

| Parameters | Procedure | Mean ± SD | *P*-value |
|---|---|---|---|
| Area of patches (in sq. cm) | HFCS | 9.24 ± 3.56 | 0.9274 |
| | ECS | 9.68 ± 3.14 | |
| Leukotrichia in patches (number) | HFCS | 112.6 ± 87.86 | 0.9934 |
| | ECS | 112.8 ± 100.44 | |
| Live cells (number per microliter) | HFCS | $5.07 \times 10^5 \pm 3.19 \times 10^5$ | <0.0001 |
| | ECS | $39.81 \times 10^5 \pm 20.77 \times 10^5$ | |
| Viability (%) | HFCS | 82.37 ± 6.7 | 0.3397 |
| | ECS | 84.47 ± 7.0 | |
| Cell yield | HFCS | $6.76 \times 10^5 \pm 4.12 \times 10^5$ | <0.0001 |
| | ECS | $46.89 \times 10^5 \pm 24.20 \times 10^5$ | |
| No. of cells per unit | HFCS per hair follicle | $0.12 \times 10^5 \pm 0.084 \times 10^5$ | <0.0001 |
| | ECS per sq. cm of donor graft | $5.80 \times 10^5 \pm 3.58 \times 10^5$ | |

HFCS, hair follicle cell suspension; ECS, epidermal cell suspension.


© 2020 *the International Society of Dermatology*

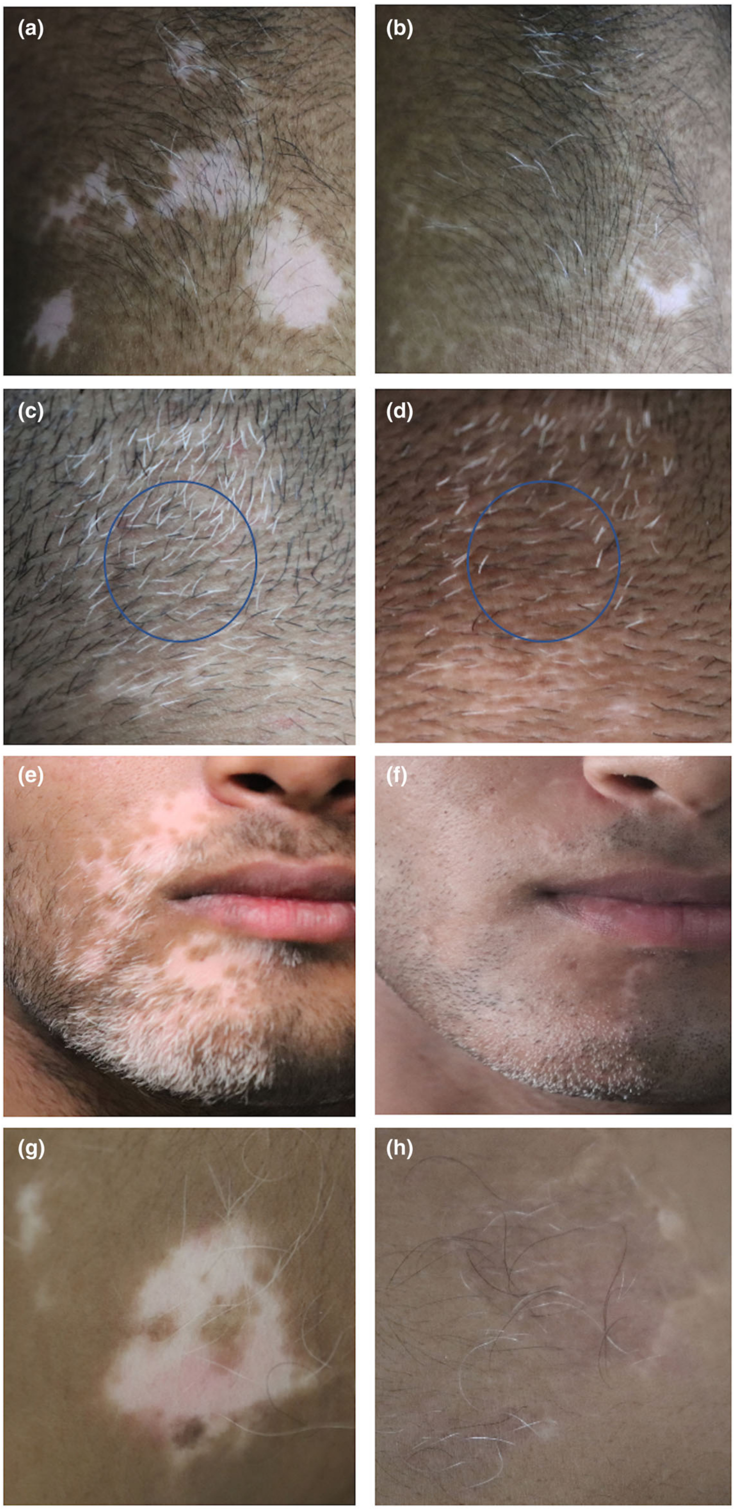


**Figure 2** (a) Vitiligo patch with leukotrichia treated with hair follicle cell suspension. (b) Good epidermal repigmentation with minimal leukotrichia improvement. (c) Vitiligo patch with leukotrichia treated with hair follicle cell suspension. (d) Good repigmentation of leukotrichia as well as vitiligo patch. (e) Vitiligo patch with leukotrichia treated with epidermal cell suspension. (f) Excellent repigmentation of vitiligo patch but partial repigmentation of leukotrichia. (g) Vitiligo patch with leukotrichia treated with epidermal cell suspension. (h) Good repigmentation of vitiligo patch and partial repigmentation of depigmented hair

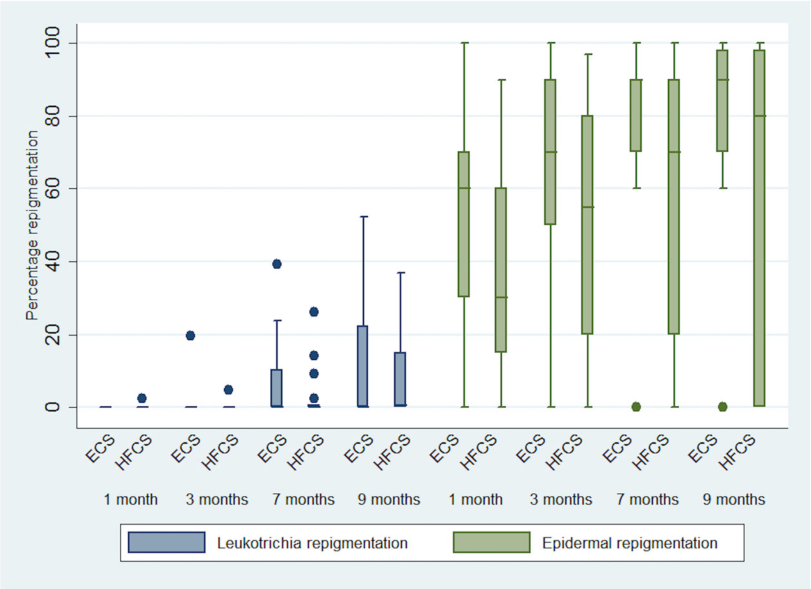


**Figure 3** Boxplot showing the mean percentage repigmentation of leukotrichia and vitiligo patch at each follow-up visit. The whiskers represent maximum and minimum values; the box edges represent the third and first quartiles; the horizontal line within the box depicts median value, and the dots denote outliers

**Table 2** Review of studies showing repigmentation of leukotrichia after various surgical methods

| S.no. | Author | Study type | Year | Number of patients/ lesions treated | Surgical procedure done | No. of patients/ lesions (percentage) showing repigmentation of leukotrichia | Duration of follow-up |
|---|---|---|---|---|---|---|---|
| 1. | Menon *et al.*[16] | Case reports | 2016 | 3 | Follicular unit extraction and transplant | 2 (66.67%) | 12 weeks |
| 2. | Chatterjee *et al.*[17] | Prospective | 2016 | 15 | Follicular unit extraction and transplant | 14 (93.33%) | 6 months |
| 3. | Thakur *et al.*[18] | Prospective | 2015 | 46 lesions | Follicular unit extraction and transplant | 11 lesions (23.9%) | 6 months |
| 4. | Holla *et al.*[9] | Retrospective | 2013 | 42 lesions | Noncultured epidermal cell suspension | 37 lesions (88.09%) | 6 months to 1 year |
| 5. | Al Jasser *et al.*[19] | Case reports | 2013 | 4 | Noncultured melanocyte-keratinocyte transplantation | 4 (100%) | 3–7 months |
| 6. | Gan *et al.*[20] | Retrospective | 2011 | 13 | Noncultured epidermal cell suspension | 9 (69.23%) | 9–12 months |
| 7. | Laxmisha *et al.*[21] | Case report | 2006 | 1 | Follicular unit extraction | 1 (100%) | 3 months |
| 8. | Malakar *et al.*[22] | Case reports | 1998 | 3 | Mini-grafting | 3 (100%) | 16 weeks |
| 9. | Bose[23] | Case reports | 1997 | 2 | Electro-epilation | 2 (100%) | 6 months, 1 year |
| 10. | Agarwal*et al.*[24] | Case series | 1995 | 7 | Thin split-thickness skin grafting | 7 (100%) | 2–6 years |
| 11. | Hann *et al.*[25] | Case reports | 1992 | 3 | Suction blister epidermal grafting | 3 (100%) | 7–10 weeks |

The mean repigmentation of leukotrichia obtained in our study was 7.42% in the HFCS group and 11.42% in the ECS group. The repigmentation in leukotrichia is slow to occur. Hair follicles show a cyclical growth, and melanocytes are incorporated in hair follicles only during the early anagen phase. Therefore, the hair repigmentation will be delayed depending on the stage of the hair follicle cycle.[9]

We followed up patients only for 9 months. The follow-up duration in previous retrospective studies was also short, ranging from 6 months to 1 year. In a previous retrospective study,

 © 2020 *the International Society of Dermatology*

with ECS the repigmentation of leukotrichia was noted in 37 of 42 (88.1%) treated lesions.[9] The improvement in pigmentation was faster in body hair than on other sites. The period of follow-up varied from 6 months to 1 year in that study. In our study, with a 9-month follow-up period, 50% of patients had repigmentation of leukotrichia in ECS-treated sites and 40% had repigmentation in HFCS-treated sites. Only a few studies report the proportion of patients showing some repigmentation of leukotrichia; however, none of them reported the percentage of leukotrichia showing repigmentation.

Repigmentation of leukotrichia was theorized to be because of retrograde migration of melanocytes from the repigmented vitiligo patch into the depigmented hair follicle.[9] The reason behind the relatively poor repigmentation of leukotrichia after transplantation may be permanent loss of melanocyte stem cells in the bulge region of the hair follicle, which a cell suspension cannot replenish. The hair follicle pigmentation depends on a complex interplay between melanotic melanocytes, amelanotic melanocytes, and their precursors in the hair follicle bulge, which is much more difficult to restore as compared to the epidermis, which has only differentiated melanotic melanocytes.[13] The melanocyte stem cells in the hair follicle are regulated by keratinocytes and dermal papilla cells as well as extrafollicular signals resulting in a much more complex multi-scale regulatory mechanism as compared to epidermis repigmentation.[14] The hair follicle melanocyte stem cells have several important functions in addition to their role in maintaining hair color.[15]

None of the long-term follow-up studies on transplantation in vitiligo has commented on repigmentation of leukotrichia. If leukotrichia persists after long-term follow-up post cell suspension transplantation, the only option that remains is the extraction of depigmented hair follicles with or without its replacement with pigmented hair follicles from the scalp.[8]

## Conclusions

Both ECS and HFCS led to good repigmentation of skin but only minimal repigmentation of leukotrichia. The difference between both the procedures was not statistically significant, however ECS resulted in slightly better repigmentation of vitiligo and leukotrichia. The mean number of cells transplanted in HFCS was significantly less, which may explain delayed repigmentation in HFCS-treated patches.

## References

1 Parsad D, Pandhi R, Dogra S, *et al.* Clinical study of repigmentation patterns with different treatment modalities and their correlation with speed and stability of repigmentation in 352 vitiliginous patches. *J Am Acad Dermatol* 2004; **50**: 63–67.
2 Begum R, Shajil E, Agrawal D, *et al.* Vitiligo: clinical profiles in Vadodara, Gujarat. *Indian J Dermatol* 2006; **51**: 100–104.
3 Mahajan VK, Vashist S, Chauhan PS, *et al.* Clinico-epidemiological profile of patients with vitiligo: a retrospective study from a tertiary care center of North India. *Indian Dermatol Online J* 2019; **10**: 38–44.
4 Lee D-Y, Kim C-R, Park J-H, *et al.* The incidence of leukotrichia in segmental vitiligo: implication of poor response to medical treatment: the incidence of leukotrichia in segmental vitiligo. *Int J Dermatol* 2011; **50**: 925–927.
5 Gauthier Y, Surleve-Bazeille J-E. Autologous grafting with noncultured melanocytes: a simplified method for treatment of depigmented lesions. *J Am Acad Dermatol* 1992; **26**: 191–194.
6 Vanscheidt W, Hunziker T. Repigmentation by outer-root-sheath-derived melanocytes: proof of concept in vitiligo and leucoderma. *Dermatology* 2009; **218**: 342–343.
7 Mohanty S, Kumar A, Dhawan J, *et al.* Noncultured extracted hair follicle outer root sheath cell suspension for transplantation in vitiligo: ORS cell transplantation in vitiligo. *Br J Dermatol* 2011; **164**: 1241–1246.
8 Kumar A. Surgical management of leucotrichia. In: Gupta S, Olsson MJ, Parsad D, *et al.*, *Vitiligo- Medical and surgical management*, 1st edn. OxfordWiley Blackwell, 2018: 401–405.
9 Holla AP, Sahni K, Kumar R, *et al.* Repigmentation of leukotrichia due to retrograde migration of melanocytes after noncultured epidermal suspension transplantation. *Dermatol Surg* 2014; **40**: 169–175.
10 Pangti R, Challa A, Chauhan S, *et al.* Comparison of cell suspension transplantation prepared from plucked hair shafts, excised hair follicles, and epidermal shave biopsies in vitiligo patients: a randomized study [published online ahead of print, 2020 Jan 31]. *Dermatol Surg* 2020. https://doi.org/10.1097/DSS.0000000000002337
11 Oh SH, Hann SK. Classification and clinical features of vitiligo. In: Gupta S, Olsson MJ, Parsad D, *et al.*, *Vitiligo- Medical and surgical management*, 1st edn. OxfordWiley Blackwell, 2018: 35–47.
12 Vashisht KR, Arava SK, Tembhre MK, *et al.* A randomized pilot study to compare hair follicle cell suspensions prepared using trypsin alone versus trypsin in combination with collagenase type I for transplantation in vitiligo. *Clin Exp Dermatol* 2020; **45**: 172–179.
13 Nishimura EK. Melanocyte stem cells: a melanocyte reservoir in hair follicles for hair and skin pigmentation. *Pigment Cell Melanoma Res* 2011; **24**: 401–410.
14 Qiu W, Chuong CM, Lei M. Regulation of melanocyte stem cells in the pigmentation of skin and its appendages: biological patterning and therapeutic potentials. *Exp Dermatol* 2019; **28**: 395–405.
15 Chou WC, Takeo M, Rabbani P, *et al.* Direct migration of follicular melanocyte stem cells to the epidermis after wounding or UVB irradiation is dependent on Mc1r signaling. *Nat Med* 2013; **19**: 924–929.
16 Menon SM, Sharma YK, Bansal P, *et al.* Restoration of pigmentation by follicular unit extraction transplant in three cases of focal vitiligo recalcitrant to therapy including with previous nonculture melanocyte-keratinocyte transplant. *Int J Trichology* 2016; **8**: 87–88.
17 Chatterjee M, Neema S, Vasudevan B, *et al.* Eyelash transplantation for the treatment of vitiligo associated eyelash leucotrichia. *J Cutan Aesthet Surg* 2016; **9**: 97–100.
18 Thakur P, Sacchidanand S, Nataraj HV, *et al.* A study of hair follicular transplantation as a treatment option for vitiligo. *J Cutan Aesthet Surg* 2015; **8**: 211–217.
19 Al Jasser MI, Ghwish B, Al Issa A, *et al.* Repigmentation of vitiligo-associated leukotrichia after autologous, non-cultured melanocyte-keratinocyte transplantation. *Int J Dermatol* 2013; **52**: 1383–1386.

20 Gan EY, van Geel N, Goh BK. Repigmentation of leucotrichia in vitiligo with noncultured cellular grafting. *Br J Dermatol* 2012; **166**: 196–199.
21 Laxmisha C, Kumari R, Thappa DM. Surgical repigmentation of leukotrichia in localized vitiligo. *Dermatol Surg* 2006; **32**: 981–982.
22 Malakar S, Dhar S. Repigmentation of leukotrichia over vitiligo patches after punch grafting. *Indian J Dermatol Venereol Leprol* 1998; **64**: 252–253.
23 Bose SK. Is there any treatment of leukotrichia in stable vitiligo? *J Dermatol* 1997; **24**: 615–617.
24 Agrawal K, Agrawal A. Vitiligo: surgical repigmentation of leukotrichia. *Dermatol Surg* 1995; **21**: 711–715.
25 Hann SK, Im S, Park YK, *et al*. Repigmentation of leukotrichia by epidermal grafting and systemic psoralen plus UV-A. *Arch Dermatol* 1992; **128**: 998–999.

 © 2020 *the International Society of Dermatology*
