# Supplementary material for: Therapeutic efficacy of cell-based therapy in vitiligo: a research letter systematically reviewed using meta-analysis
Source: Arch Dermatol Res. 2024 May 22;316(5):198. doi: 10.1007/s00403-024-02920-6 (PMC11111487; doi:10.1007/s00403-024-02920-6)
Supplement: Supplementary file 1 — Supplementary file1 (ZIP 24195 KB) [file 403_2024_2920_MOESM1_ESM.zip › Studies were included/Hamouda 2017.pdf]

Egypt, J. Plast. Reconstr. Surg., Vol. 41, No. 2, July: 199-210, 2017

Autologous Cultured and Noncultured Melanocyte Transplantation Versus Dermabrasion with Split Thickness Skin Grafting in Cases of Non Progressive Vitiligo and Post Burn Leukoderma

WESAM I.A. HAMOUDA, M.Sc.*; SAMY A. SHEHABELDIN, M.D.**; OMAR O. SHOUMAN, M.D**; SAMAR A. ASKER, M.D.*** and MOHAMMED R. EL-HADIDY, M.D.**

The Departement of Plastic Surgery, Benha Teaching Hospital & Faculty of Medicine, Mansoura University** and The Department of Histology & Cytology, Faculty of Medicine, Mansoura University****

ABSTRACT

Background: Vitiligo and post burn leucoderma are an acquired skin disorders characterized by white and depigmented patches of variable shape and sizes due to disappearance of functioning melanocytes. Transplantation of autologous cultured or non cultured melanocyte is one of the available surgical approaches used in the treatment of leucoderma in general beside ultra thin split thickness skin grafting.

Objectives: To evaluate the efficacy of cultured/non cultured melanocytes versus split thickness skin grafting in induction of repigmentation in stable vitiligo and post burn leucoderma.

Patients and Methods: Thirty patients with stable leucoderma. Divided into three groups. Pretreatment punch biobsy was taken. Preparation of cultured/non cultured melanocytes and split thickness skin graft was performed and applied to dermabraded skin. After 6-12 monthes patient response evaluated clinically and by another punch biobsy.

Results: In the first group in the regard of repigmentation between test site and control sites. It was excellent at (5) (50%) of the test sites. It was good at (2) (20%) of the test sites. Fair repigmentation was encountered among (2) (20%) of the test sites. Poor repigmentation was encountered among (1) (10%) of the test sites. Versus excellent at (3) (30%), good at (2) (20%), fair (3) (30%), Poor repigmentation (2) (20%) In the second group, and excellent at (6) (60%), good at (2) (20%), fair repigmentation (1) (10%), Poor repigmentation (1) (10%) in the third group.

Conclusion: Autologous cultured/non cultured melanocytes could be effective method for treatment of stable vitiligo as well as post burn leucoderma. Non cultured is simpler, less costy, shorter time for the method. Selection of the patient is crucial to the success of the outcome.

Key Words: *Melanocyte – Dermabrasion – Vitiligo – Post burn leukoderma.*

INTRODUCTION

Leucoderma had been a term used to describe disorders in which the skin had been turned white in some areas due to lack of melanin in the epidermis due to absence or destruction of melanocytes **[1]**. The nomination 'vitiligo' per-haps had come from Latin word 'Vitilus', that denote 'calf', and had been introduced by Roman physician, Celsus in the first century A.D. **[2]**. Vitiligo had been identified as an acquired skin disorder which had been characterized by sharply demarcated depigmented lesions of variable size and shape, with tendency to increase in size during lifetime. It had affected approximately 0.5-2% of the general population worldwide and it had appeared any time from shortly after birth to late adulthood. The average age of onset had been approximately 20 years **[3]**. Deep burns had often resulted in hypopigmentation, referred to as post burn leucoderma which had a similar psychological impact on patient as that of vitiligo **[4]**. The scar tissue laid down after healing by secondary intention had provided a barrier not only to the transfer of melanin by the dendritic processes but also to melanocyte migration, which had occurred in a favourable environment **[4]**. Post-burn leukoderma had been resistant to UV based therapies like UV-B and psoralen plus UV-A **[5]**. There had been numerous treatment strategies for leucoderma throught the years. Psoralen plus ultraviolet A (PUVA) therapy and topical corticosteroids, Narrow band UVB light theraby **[1]**. Despite availability of various types of medical treatments for vitiligo, a significant number of patients had failed to respond with a satisfactory degree of repigmentation. Transplantation of autologous melanocytes cultured or non cultured had been one of the available surgical approaches that had offerred apotential solution for patient with vitiligo who had failed to respond to medical treatments **[6]**.

The colour of the skin:

Melanin had been the most important chromophore that had determined the normal skin colour. The number, shape, size, distribution and degradation of melanin laden organelles (melanosomes) that had been produced by melanocytes and had been transferred to the surrounding epidermal keratinocytes had determined racial and ethenic differences in the skin colour. In humans two pigmention types had occurred. The first had been constitutive skin colour, which had been determined by the amount of melanin pigmentation that had been genetically determined in the absence of sun exposure and other influences. The second facultative (inducible) skin color or tan, which had resulted from sun exposure, increased pigmentation, and had also been due to endocrine, paracrine & autocrine factors [7].

Function of cutaneous melanocytes:

The melanocyte had been the primary melanin-producing cell of the epidermis [8]. The major differentiated function of the melanocytes had been to synthetize melanin in specialized organelles within the melanocytes (melanosomes) and to transfer them to neighboring keratinocytes [9]. through there dendrites, and hence function as key component of the pigmentary system [10]. Two melanocytic populations had been found in the skin, (secretory melanocytes) had cytocrine activity it had behaved as unicellular glands by melanosomes production and it's transferring to surrounding epidermal keratinocytes through network of dendrites they form in the basal layer of the epidermis. (Non-secretory melanocytes) (melanophores) had not transfered their melanosomes, but had redistributed them from the perinuclear zone into the dendrites and then back again [9].

Types of melanin:

There had been two major types of melanin, the eumelanins and pheomelanins. Eumelanins had been brown and black pigments while pheomelanins had been red and yellow pigment that is initialy synthesized just like eumelanins more photoprotective, [11] Phaeomelanin had been the major type in red hair and also had predominated in the epidermis of skin types I and II. Eumelanins, on the other hand, had been present diffusely in peoples with dark skin and hair [12].

Function of melanin:

Pigment had provided significant protection to the underlying tissues from being damaged (photoaging, photocarcinogenesis) by ultraviolet radiation besides providing wide range of cosmetic coloration to the skin, hair and eyes. It had acted also as an antioxidant and a radical scavenger, and thus it had played important roles in protecting cells from damage [13].

Morphology of cutaneous melanocytes:

Melanocytic cell body, had round to oval nucleus, numerous mitochondria and vesicles, microfilaments, intermediate filaments, abundant smooth endoplasmic reticulae, rough endoplasmic reticulae and golgi apparatuses and microtubules in the cytoplasm, sits on a specialized region of basal lamina with its dendrites had come into contact with keratinocytes as far away as the mid stratum spinosum. Thus a melanocyte had association with approximately 30-40 surrounding keratinocytes transferring melanosomes to them, this had been named the epidermal melanin unit [14].

Melanogenesis:

Melanin pigment had been large biopolymers derived from the progressive oxidation of the amino acid tyrosine in the absence of sulfhydryl groups from cysteine producing black-brown (eumelanin) or in the presence of sulfhydryl groups at cysteine producing yellow-red (phaomelanin) compounds [15]. Melanosomes that had been the specialized intracellular organelles of pigmented cells specified to the melanin pigments biosynthesis, storage and transport. It had been responsible for most visible pigmentation in mammals and other vertebrate [16].

Melanosomal Transportation to the dendrites and transfer to keratinocytes:

Studies had suggested several ways for melanosomal transfer, which had included cytophagocytosis, fusion of plasma membranes, exocytosis, and transfer by membrane vesicles [17].

Melanocytic culture:

Since 1957 isolation and subsequent culture of human epidermal melanocytes had been attempted. Only since 1982 pure normal human melanocyte cultures had been reproducibly established to yield cells in sufficient quantity for biological, biochemical, and molecular analyses [18]. Melanocytes fail to grow, and usually die, in culture media used for skin fibroblasts or keratinocytes. Melanocytes did not produce any of the growth factors that had been known to - stimulate them in contrast to many other cell types [19,20].

Melanocytic differentiation markers:

Commonly used melanocytic differentiation markers had been: S100, HMB-45 (gp100), tyrosinase and MART-1 [21].

1- *S100:*

First isolated from glial cells as acidic calcium-binding protein. Its name had been due to its solubility in 100% saturated ammonium sulfate solution **[22]**. Its specificity for melanocytic lesions had been limited(75-87%) **[23,24]**.

2- *HMB45:*

(Human Melanoma Black 45) a cytoplasmic premelanosomal glycoprotein marker (gp 100). It had been less sensitive than S100 but it had greater specificity **[25]**. The reported sensitivity of HMB45 for melanoma ranges from 69% to 93%, expression is maximal in primary melanoma specimens (77-100%) and less in metastases (58-83%) **[26]**.

3- *Melanoma antigen recognized by T cells (MART1):*

An antigen that had first detected in melanoma metastases by two different antibodies: Melan A and A-103. As in S-100 protein, most benign and malignant melanocytes, had expressed this marker. 27These antibodies had shown (75-92%) sensitivity and (95-100%) specificity **[28]**.

4- *Tyrosinase:*

An enzyme that hydroxylates tyrosine as the first step in melanin biosynthesis. It had been seen as fine granular cytoplasmic staining **[29]**. Positive staining had been tended to be strong and diffuse **[30]**. The tyrosinase sensitivity for melanoma (84-94%). Sensitivity had decreased (79-93%) with increase of the clinical stage and in metastatic lesions **[31]**. The tyrosinase specificity for melanoma is 97-100% **[32]**.

Clinical features:

Patients had been presented with one to several amelanotic macules which had appeared as chalk or milky white in color. Inflammation had been seen at the advancing margin of the lesions; however, there had been no epidermal change or erythema. Lesions had been often symmetric and usually enlarge centrifugally in size with time. The increase in size had corresponded with a substantial loss of functioning epidermal and, sometimes, hair follicle melanocytes **[33]**. Lesions had been typically well demarcated, but the edges may be scalloped **[33]**.

Histopathologic findings of vitiligo:

Melanocytic and melanin loss in the white patches, with inconstant lymphonuclear infiltrate in the advancing peripheries of lesions **[34]**. Absence of melanocytes in vitiliginous areas, Extracellular granular material, and Vacular keratinocytic degeneration in the normally pigmented epidermis of vitiligo patients **[34]**.

Perilesional skin:

Melanocytes and melanin granules had been located in the basal layer of peripheral epidermis, with reduced number of melanocytes and with some alterations such as an increase in size and with long dendritic processes **[34]**. Keratinocytes from perilesional skin had shown the features of damaged cells **[35]**. Focal gaps in perilesional skin basal membrane had been seen with lymphocytes infiltrating between basal membrane cells **[36]**.

Vitiligenous skin:

Histochemical procedures for the identification of melanocytes (DOPA-reaction) had given negative result, even if abnormal DOPA-positive cells had been seen, that represent inactivated melanocytes. Also, residual activity of the melanocyte-specific enzyme tyrosinase had been seen in lesional vitiligo patches that represent an index of the presence of residual melanocytes **[37]**. Keratinocytes' abnormalities had been seen at the edges of the patches. It had been reported a "ballooning" degeneration (vacuolization) in the basal dermal keratinocytes associated to spongiosis and the presence of a mononuclear and lymphohistiocytic infiltrate **[35]**. The upper dermis had shown lymphocytic infiltration, especially in a perivascular area **[38]**. Degenerative changes in cutaneous nerves and adnexal structures had been also reported in Long-standing lesions **[39]**.

Surgical treatment of vitiligo:

Repigmentation had been successfully induced in previously failed graft cases by NB-UVB phototherapy of 311nm **[40]**. In general; autologous transplantation methods had been only indicated after medical treatment had failed. These methods can be used in combination with medical and/or irradiation therapies. Transplantation may also be considered as first option to treat patients with stable and/or focal (segmental) vitiligo. Even after a successful grafting, depigmentation of the grafts may still occur when reactivation of the disease takes place. Sugical autologous transplantation could be tissue or cellular grafting **[41]**. The popular surgical methods among tissue grafts include Thin Thiersch's graft, Suction blister graft, punch grafting and mini punch grafting (MPG) **[42]**.

Surgical autologous transplantation methods:

(A) Tissue grafting:

1- *Minigrafting:*

Autologous mini punch grafting (MPG) had been a safe, effective and easy technique that had been performed on any site with minimal side effects and good cosmetic results **[42]**. The proce-

dure had involved the transfer of circular pieces or punches of skin tissue 1-2mm from the normally pigmented donor areas (such as the hip, buttocks, and outer thigh) into similar shaped pits spaced 5-10mm that had been made on the recipient skin **[43]**.

2- *Thin split-thickness skin grafting:*

Ultra-thin grafts (STSG-UT) of 3–6 thousandth of an inch (0.003–0.006in. or 0.08–0.15mm) had also been used for resurfacing **[44]**.

3- *Grafting of epidermal blisters:*

A suction blister apparatus that had been capable of exerting 200mm Hg to 300mm Hg negative pressure for 2-3 hours had been essential to separate the epidermis from the dermis at the normally pigmented donor skin. Two days before transplantation, blistering of the depigmented lesion had been induced using liquid nitrogen. After blister formation, the depigmented epithelium had been removed and the roofs of the pigmented donor blister had been grafted to the denuded lesional areas **[45]**.

(B) Cellular grafting:

1- *Grafting of cultured autologous melanocytes:*

Autologous melanocytes had been expanded by in vitro culturing techniques and had been transplanted into a previously denuded achromic skin area **[46]**. This is an expensive technique that had required special laboratory expertise. However, it had represented an adequate method to repigment larger vitiliginous skin areas in the future. There had been several methods to obtain cultured autologous melanocytes, Grafting of pure melanocytes and Grafting of melanocytes mixed with keratinocytes **[47]**.

2- *Grafting of non-cultured melanocytes suspension:*

More simplified methods of grafting of fresh epidermal cell suspensions bearing both melanocytes and keratinocytes (non-cultured epidermal cellular grafting) which had been an innovative surgical technique that had been used for the treatment of stabilized leucoderma, including vitiligo **[48]**.

3- *Grafting of follicular melanocytes:*

Based on the concept that the existence of a pool of undifferentiated melanocyte stem cells in the hair that can replenish the pool of differentiated melanocytes. These stem cells had been maintained in the niche microenvironment, which had been thought to be located in the "bulge" area, where arrector pili muscle attaches with the hair **[49]**.

PATIENTS AND METHODS

Thirty patients were included with stable forms of leukoderma (non-progressive vitiligo or and post-burn). It was conducted at the Plastic, Reconstructive and Burn Surgical Center, Mansoura Faculty of Medicine and Benha Teaching Hospital.

Inclusion criteria:

Patients with stable disease without increase in the size of the existing lesions or eruption of new lesions for at least 2 years.

Exclusion criteria:

1- Cases of active disease. Disease activity was determined by the presence of at least one of the following criteria: (a) Increase in lesion's size both in width or depth, (b) Freshly erupted lesion, (c) Grouping of small lesions together, and (d) Eruption of new lesions away from the primary one.
2- Cases with vitiligo affecting more than 70% body surface area, as the extent as well as with the activity of vitiligo were correlated to the level of anti-melanocytic antibodies.
3- Cases with associated autoimmune diseases particularly autoimmune thyroiditis, diabetes mellitus or pernicious anemia.

Patients were subjected to the following: (a) History taking, (b) Physical examination, (c) Explanation of the procedure, (d) Baseline investigations including complete blood count and coagulation profile, HIV, Hepatitis B and C virus serology, (e) Baseline and serial post-treatment photography, (f) All patients will be subjected to pre-treatment and post-treatment skin punch biopsy that will be examined histologicaly by immunohistochemical stain HMB45 & S100.

Informed consent was taken from all cases included in the study that was approved by the Research Ethics Committee, Faculty of Medicine Mansoura University.

The thirty patients of the study were subdivided equally into three groups.

Under general anesthesia, cleaning and sterilization the donor and the recipient areas with povidone-iodine, followed by 70% alcohol were done.Dermabration of the recipient (vitiliginous) area until uniform pin-point capillary bleeding was seen. Denuded areas were covered by gauze moistened with Saline solution until transplantation.

Very thin split-thickness skin graft (0.1-0.15mm thick) was obtained from the donor normally pigmented area; usually the inner aspect of the thigh, with size was justified according to the depigmented area size. The graft was moistened after removal with sterile saline solution, to insure that the graft did not become dry before application. The slightly bleeding doner area was covered with tulle gras and covered with adhesive bandage.

The first group (10 patients) was subjected to dermabrasion and thin split- thickness skin grafting. Gentle application of the graft on the dermabraded achromic region, where the surface was flattened and stretched over the dermabraded region.Stitches were done at the borders to fix the graft and then saline moistened gauze was used to cover the recipient area. At least 4 hours rest in a hospital after the procedure was advised. Removal of the bandages was advised after 1-2 weeks. Then patients were encouraged to expose themselves moderately to the sun in a slowly increasing dose. Follow-up every 2 weeks for one and half year.

The second group (10 patients) the patients in this group were subjected to autologous grafting with non-cultured melanocyte suspension prepared in MERC.

1- A shave biopsy skin sample of up to one-tenth the size of the recipient area is incubated.
2- The cells are mechanically separated by using trypsin EDTA solution.
3- The material is centrifuged to prepare a suspension.

Placement of the skin sample, with epidermis upward, immersed in 8ml of trypsin 0.25%, in a petri dish with EDTA and incubated at 37ºc for 30 minutes, then trypsin solution was discarded and the sample rinsed with nutrient mixture. Mechanical separation of The dermis from epidermis was done and dermis was discarded. The epidermis then transferred to 15ml centrifuge tube and centrifuged for 6 minutes to separate the melanocytes and the keratinocytes, which sink to the bottom, from other epidermal cells which float. The cell suspension formed of keratinocytes and melanocytes is applied to the dermabraded depigmented skin area.

The third group (10 patients) the patients in this group were treated by transplantation of cultured epidermal cells mainly melanocytes prepared in MERC. Placement of the skin sample, with epidermis upward, immersed in 8ml The cell suspension formed of keratinocytes and melanocytes is transferred to culture media (DMEM and HBSS media) for 2-3 weeks, then applied to the dermabraded achromic skin.The patients of all groups were evaluated at baseline and at post-treatment at 1st, 6th, and one year. Several photos were taken at each visit to compare the effect of different treatment.

RESULTS

Demographic characteristics:

Sex: 53.3% of the studied patients were males (16 patients) & 46.7% were females (14 patients) with male to female ratio 1.14 : 1. In 1st group, 60% were males (6 patients) & 40% were females (4 patients) with ratio 1.5: 1. In 2nd and 3rd groups 50% were males (5 patients) 50% were females (5 patients) with male to female ratio 1: 1.

Age: Ranged between 8 and 43 years with mean of 23.8±9.01 years. Age group below and equal 20 years includes 15 patients (50%) while age group above 20 years includes 15 patients (50%) of the patients. In 1st group ranged between 8 and 43 years with the mean of 23.50±10.32 years. Age group below or equal 20 years includes 5 patients (50%) while age group above 20 years includes 5 patients (50%) of patients. In 2nd group ranged between 13 and 43 years with the mean of 23.90±9.12 years, age group below or equal 20 years include 5 patients (50%) while age group above 20 years includes 5 patients (50%) of patients. In 3rd group ranged between 12 and 40 years with the mean age of 23.90±8.46 years. Age group below or equal 20 years include 5 patients (50%) while age group above 20 years includes 5 patients (50%) of patients.

Family history: The majority of the studied patients (86.7%) reported negative family history for vitiligo, however a positive family history of the disease was encountered among (13.3%) of the studied patients. In 1st group (90%) reported negative family history, (10%) positive family history of the disease. In 2nd group 8 patients (80%) reported negative family history (20%) positive family history. In 3rd group (90%) reported negative family history, (10%) positive family history.

Type of skin: Fifty per cent of the patients are skin type IV (15 patients), 20% of the patients are skin type III (6 patients) and 30% of the patients are type II (9 patients). In 1st group Sixty per cent of patients are skin type IV (6 patients), 10% of patients are skin type III (1 patients) and 30% of patients are type II (3 patients). In 2nd group Fifty per cent of the patients are skin type IV (5 patients), 20% of the patients are skin type III (2 patients) and 30% of the patients are type II (3 patients). In

3rd group Forty per cent of the patients are skin type IV (4 patients), 30% of the patients are skin type III (3 patients) and 30% of the patients are type II (3 patients).

Clinical characteristics:

Duration of the disease: Ranged between 3 and 12 years with a mean disease duration of 7.2±2.82. Between 3 and 11 years with a mean of 6.8±3.22 in 1st group. In 2nd group ranged between 5 and 12 years with mean of 8.00±2.67. Between 4 and 11 years with mean of 6.80±2.66 years in 3rd group.

Duration of the disease stability: Ranged between 2 and 10 years with a mean of 5.17±2.46. In 1st group ranged between 2 and 10 years with a mean of 4.9±2.73. In 2nd group between 2 and 9 years with a mean of 5.70±2.31. In 3rd group ranged between 3 and 10 years with a mean of 4.90±2.51 years.

Size of the treated lesion: The distribution of the included patients according to the size of the treated lesion was as following Table (1).

Table (1): Size and number of treated lesions.

Size of the treated lesion (cm^2)	No.	%
1	2	6.7
5	5	16.7
6	2	6.7
7	2	6.7
8	6	20
9	2	6.7
10	4	13.3
14	1	3.3
15	3	10
18	1	3.3
20	2	6.7
1st group:		
7	1	10
8	1	10
9	2	20
10	2	20
15	1	10
18	1	10
20	2	20
2nd group:		
1	2	20
5	3	30
6	2	20
8	3	30
3rd group:		
5	2	20
7	1	10
8	2	20
10	2	20
14	1	10
15	2	20

Table (2): Comparison between all groups according to different parameters with range, mean standered deviation.

All groups	Range	Mean ± S.D	F. test	*p*.value
Age:				
Group 1	8-43	23.50±10.32		
Group 2	13-43	23.90±9.12	0.006	0.994
Group 3	12-40	23.90±8.46		
Duration of disease:				
Group 1	3-11	6.80±3.22		
Group 2	5-12	8.00±2.67	0.586	0.564
Group 3	4-11	6.80±2.66		
Duration of stability:				
Group 1	2-10	4.90±2.73		
Group 2	2-9	5.70±2.31	0.335	0.718
Group 3	3-10	4.90±2.51		
Size of treated lesion:				
Group 1	7-20	12.60±5.13		
Group 2	1-8	5.30±2.58	8.511	0.001
Group 3	5-15	9.70±3.83		
Total cell count:				
Group 1	0-0	0±0		
Group 2	0.19-0.92	0.68±0.27	20.461	0.001
Group 3	0.75-2.3	1.56±0.55		
Cell count:				
Group 1	0-0	0±0		
Group 2	1.13-2	1.40±0.31	4.158	0.056
Group 3	1.47-1.88	1.62±0.14		
Size of donor:				
Group 1	7-20	12.60±5.13		
Group 2	0.1-0.8	0.53±0.26	53.084	0.001
Group 3	0.5-1.5	0.97±0.38		
Time of repigmenation:				
Group 1	7-10	8.10±1.20		
Group 2	14-21	16.80±2.78	115.459	0.001
Group 3	21-30	25.50±3.24		

Intervention technical procedure:

In 1st group Size of the graft: Split thickness skin graft was taken in ratio of 1:1 in relation to the treated area ranging in size from $7cm^2$ to 20 cm^2 with a mean of 12.6±5.13. In the 2nd group the total cell count (x 10^6) / (1ml) ranged between 0.19 and 0.92 (x 10^6) cells with a mean of 0.68 ±0.27 (x 10^6) cells. And Transplanted cell count per mm^2 : ranged between 1.13 x 10^3 and 2 x 10^3 cells with a mean cell count of 1.40±0.31 cells. In 3rd group the total cell count (x 10^6) (1ml) ranged between 0.75 and 2.3 (x 10^6) cells with a mean of

1.56±0.55 (x 10^6) cells. And transplanted cell count per mm^2: ranged between $1.47x10^3$ and $1.88x10^3$ cells with a mean cell count of 1.62±0.14 cells. In 2nd and 3rd groups donor area is 1:10 of the treated lesion ranges from 0.1-0.8cm^2 with mean of 0.53±0.26cm^2 and from 0.5-1.5cm^2 with mean of 0.97±0.38cm^2 respectively.

Efficacy outcome:

The percentage of area of repigmentation in the treated lesions relative to the baseline depigmented surface area was the main parameter to evaluate the efficacy of the intervention. Patients' response had been evaluated after 6 months, according to the extent of pigmentation. Repigmentation in the treated and the control areas around the treated areas was graded as follow excellent with 90-100% pigmentation, good with 50-89%, fair with 20-49%, and poor with 0-20% of the treated lesion. Repigmentation was evaluated of by comparing of pre and postreatment photographs of the recipient site. In the 1st group the repigmentation at 5 (50%) of the treated sites was excellent. It was good in 2 (20%). Fair repigmentation was encountered among 2 (20%) of the treated sites. Poor repigmentation was encountered at 1 (10%) of the treated sites.in the 2nd group the repigmentation at 3 (30%) of the treated sites was excellent. It was good in 2 (20%). Fair repigmentation was encountered among 3 (30%) of the treated sites. Poor repigmentation was encountered at 2 (20%) of treated sites. In the 3rd group the repigmentation at 6 (60%) of the treated sites was excellent. It was good in 2 (20%). Fair repigmentation was encountered among 1 (10%) of the treated sites. Poor repigmentationwas encountered at 1 (10%) of treated sites.On the other hand, none of the control areas showed excellent or even good repigmentation. While repigmentation was fair in nearly 40% and poor for 60% of the control areas.

Time of initial repigmentation: Occurred at time ranged between 7 and 10 days with a mean, 8.10±1.20 days. 14 and 21 days with a mean of 16.80±2.78 days. 21 and 30 days with mean of 25.50±3.24 days in the 1st, 2nd 3rd groups respectively.

Color matching of the treated area: In the 1st group the majority of patients showed darker color in comparison to that of the surrounding normally pigmented skin. 3 patients (30%) showed the same color while one patient showed somewhat lighter color in the treated area than surrounding normal skin. In the 2nd group the majority of the patients showed similar color to that of the surrounding normally pigmented skin. One patient (10%) showed hyperpigmented color while 3 patients showed somewhat lighter color in the treated area than the surrounding normal skin. In the 3rd group Four patients showed the same color as that of the surrounding normally pigmented skin. Two patients (20%) showed hyperpigmented color while 4 patients showed somewhat lighter color in treated area than the surrounding normal skin.

Side effects: In first group (90%) had side effects following grafting. Among adverse reactions at the treated sites after the intervention, scar at the donor area in 3 (30%), hyperpigmentation at the donor area in 5 patients (50%) and infection at the recepient site in 1 patient (10%). In the 2nd group (60%) of the studied patients had no side effects following the intervention. Side effects following grafting occurred in the second group (40%). Among adverse reactions at the treated sites after the intervention, scar at donor area in 3 patients (30%), hyperpigmentation at donor area in 1 patients (10%) and infection at the recepient site in no patient (0%). In the 3rd group Five patients (50%) had no side effects following the intervention. Side effects following grafting occurred in the third group (50%). Among adverse reactions at the treated sites after the intervention, scar at donor area in 2 (20%), hyperpigmentation at the donor area in 1 patients (10%) and infection at the recepient site in 2 patients (20%).

Patient satisfaction: Patient satisfaction as regard to repigmentation and donor area scaring is graded into good in 6 patients (60%), fair in 2 patients (20%) and poor in 2 patients (20%) in the 1st group. In the 2nd group good in 5 patients (50%), fair in 3 patients (30%) and poor in 2 patients (20%). In the 3rd group good in 7 patients (70%), fair in 2 patients (20%) and poor in 1 patient (10%).

Histological examination of skin biopsy: Examination of pre-treatment & six months and one year post-treatment, punch biopsies by H&E sections and HMB45 showed pretreatment negative results in all patients, post-treatment positive results for 9 patients (90%) and post-treatment negative results for 1 patients (10%) in the 1st group. In the 2nd group post-treatment positive results for 7 patients (70 %) and post-treatment negative results for patients 3 (30%). In the 3rd group post-treatment positive results for 8 patients (80%) and post-treatment negative results for 2 patients (20%) while S100 shows false positive results in all groups due to cross staining of langerhans cells.

Effect of patients characters and treatment variables on the outcome response:

Patient variables: In the patient variables, the age (Chi square test=3.200, p=0.362), type of skin (Chi square test=7.333, p=0.291) and duration of disease stability (Chi square test=0.114, p=0.949) of the studied patients had no significant positive correlation with the percentage of repigmentation of the treated areas. In the 2nd group, the age (x^2=0.667, p=0.881), type of skin (x^2=10.278, p=0.113) and duration of disease stability (x^2 =0.775, p=0.549) of the studied patients had/hadn't a significant positive correlation with percentage of repigmentation of the treated areas. In the 3rd group, the age (x^2=4.000, p=0.261), type of skin (x^2=4.861, p=0.562) and duration of disease stability (x^2=0.626, p=0.624) of the studied patients had a significant positive correlation with percentage of repigmentation of the treated areas. In the 1st group other quantitative variables as sex (x^2= 2.500, p=0.475) family history (x^2=1.111, p=0.774), duration of vitiligo (x^2=0.364 , p=0.782). As regatrds the size of treated lesion, In the 2nd group sex (x^2=0.667, p=0.881) family history (x^2= 2.708, p=0.439), duration of vitiligo (x^2=1.310, p=0.355), had no statistically significant correlation with percentage of repigmentation in treated areas. In the 3rd group sex (x^2=4.667, p=0.198) family history (x^2=0.741, p=0.864), duration of vitiligo (x^2=0.378, p=0.773), had no statistically significant correlation with percentage of repigmentation in treated areas. As regatrds the size of the treated lesion, it hadn’t significant effect the result of repigmentation.

Treatment Variable: In the 1st group the size of the graft was 1:1 donor area which had no significant positive correlation with percentage of repigmentation of the treated areas. Also in the 2nd group the total cell count/ ml suspension and cell count/mm^2 donner area had no significant positive correlation with percentage of repigmentation of the treated areas. While in the 3rd group the total cell count/ml suspension and the cell count/mm^2 donor area had a significant positive correlation with the percentage of repigmentation of the treated areas. Regarding to the comparison between the three groups shows statistically significant correlation as regard to the size of the treated lesion (p=0.001*), the total cell count (p=0.001*), the size of the donor (p=0.001*) & the time of repigmenation (p=0.001*).

As regard to the degree of improvement in the three groups, there was a statistically significant correlation as regard to satisfaction (p=0.024*).

Table (3): Comparison between the three group according to relation between distribution of different parameters and degree of improvement.

All groups	Range	Mean ± S.D	F. test	*p*.value
Age:				
Poor	20-40	28.00±8.64		
Fair	18-29	23.83±5.04	0.334	0.801
Good	17-43	23.17±10.17		
Excellent	8-43	22.79±10.37		
Duration of disease:				
Poor	5-12	8.25±3.30		
Fair	3-9	6.50±2.35	0.299	0.826
Good	4-11	7.00±2.37		
Excellent	3-12	7.29±3.22		
Duration of stability:				
Poor	4-8	6.00±2.31	0.338	0.798
Fair	2-7	4.50±2.17		
Good	3-8	4.83±1.83		
Excellent	2-10	5.36±2.95		
Size of treated lesion:				
Poor	8-15	9.75±3.50		
Fair	1-10	6.83±3.31	0.646	0.593
Good	5-20	10.67±6.74		
Excellent	1-20	9.43±5.05		
Total cell count:				
Poor	0.9-2.3	1.37±0.81		
Fair	0.19-1.3	0.78±0.46	0.637	0.602
Good	0.72-1.8	1.04±0.51		
Excellent	0.2-2.2	1.22±0.69		
Cell count:				
Poor	1.13-1.53	1.27±0.23		
Fair	1.15-1.9	1.51±0.32	1.177	0.350
Good	1.37-1.8	1.55±0.19		
Excellent	1.24-2	1.58±0.25		
Size of donor:				
Poor	0.8-8	2.78±3.50		
Fair	0.1-10	3.53±4.64	0.377	0.770
Good	0.5-20	6.77±9.50		
Excellent	0.1-20	4.86±6.39		
Time of repigmenation:				
Poor	9-30	17.75±8.85		
Fair	7-24	15.00±6.99	0.166	0.919
Good	8-30	18.00±8.60		
Excellent	7-28	16.79±7.90		

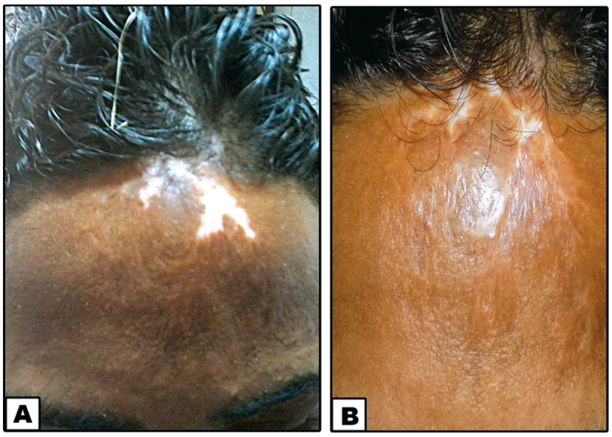

Photo (1): Male patient with post burn leucoderma at hair line at the left Photo (A) with six months after treatment with non-cultured melanocyte transplantation at the right Photo (B).

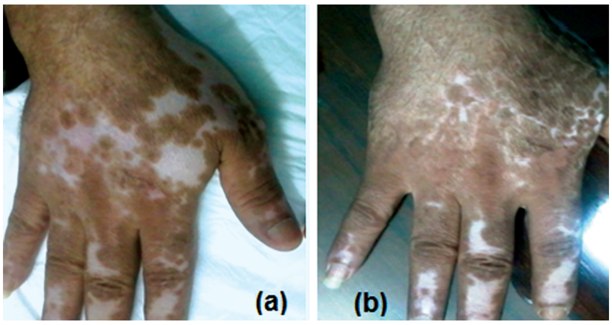

Photo (2): Male patient with vitiligo on the dorsum of the hand at the left Photo (A) with six months after treatment with cultured melanocyte transplantation at the right Photo (B).

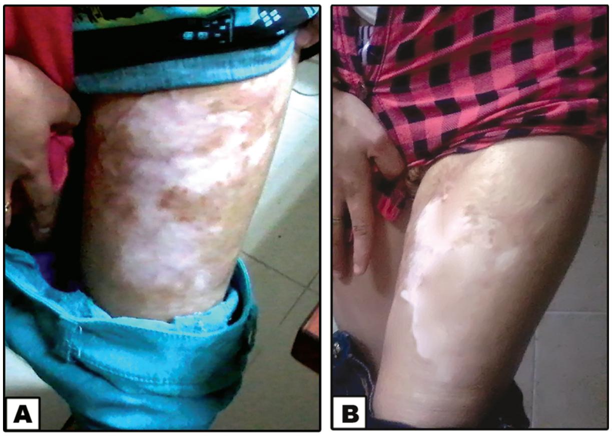

Photo (3): Female patient with post burn leucoderma on the ventral aspect of the thigh at the left Photo (A) with six months after treatment with cultured melanocyte transplantation at the righ Photo (B).

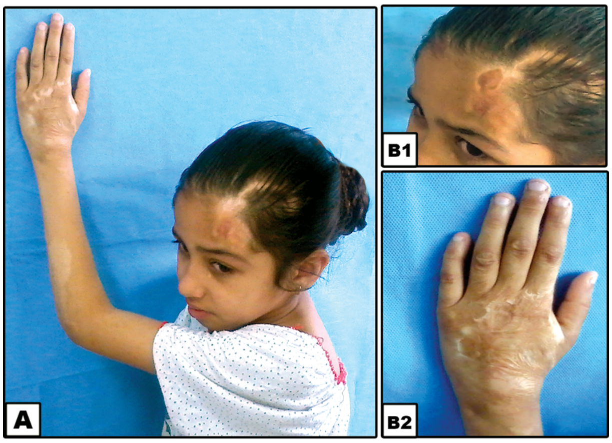

Photo (4): Female child patient with post burn leukoderma on the dorsal aspect of the hand and at hair lins at the right Photo (A) with six months after treatment with cultured melanocyte transplantation at the left Photo (B1,2).

DISCUSSION

In this study, a statistically non-significant difference was detected in the grades of repigmentation between the treated and the control sites (chi X^2=2.500, p=0.868). With overall result of excellent in 14 (46%), good in 6 (20%) and fair in 6 (20%), while poor repigmentation was encountered in 4 (14%) of them. The present results are almost similar or slightly better than those reported by Gauthier and Surleve-Bazeille **[50]** who put the melanocytes suspension obtained from the hairy scalp full-thickness graft in the bullae induced in the depigmented areas with 31% of cases showing >75% repigmentation. This better percent of improvement noticed in the present study may be due to the use of modified Gauthier and Surleve-Bazeille **[50]** technique achieved by hyaluronic acid addition to prevent spillage and performing a gluteal region donor skin grafts which are expected to have greater melanocyte density **[51]**. The results of the current study are also better than those reported by El-Zawahry et al. **[52]** who reported that among 22 patients treated, 5 patients (23%) showed excellent response, 7 patients (32%) good response, 6 patients (27%) fair response and 4 patients (18%) showed poor response. They explained the small percentage of excellent results in their study by low melanocytic density in the suspension they used and that most of their cases were having acral vitiligo and the. The results of the present study are also better than a study in the United States done by Huggins et al. **[53]** on 23 vitiligo patients that showed excellent repigmentation in just 17%, and good repigmentation in 31%. Fair and poor repigmentations were achieved in 10% and 41% of patients. However, The present

results are slightly less than those reported by Mohammed et al. [54], who had injected suspension of melanocytes that had been obtained from a graft from inner aspect of the thigh to the bullae induced at vitiliginous lesions (group II patients) or pouring melanocytes suspention on dermabraded vitiliginous lesions (group III patients) and had found that excellent to good results had been shown in 7 out of 10 patients (70%) of each group and poor results in 3 patients. This better percent of improvement noticed in their study may be due to that the donor area was 1/10 - 1/4 of the size of the recipient area, the skin samples were immersed in 0.25% trypsin solution then incubated at 40°C for 18 hours in CO_2 incubator thus the treatment was done in two steps on two successive days and the long follow-up period for one & half year. The results of our study are also less than that was reported by Mulekar (2005) [55] who found that in 142 patients with vitiligo vulgaris, 80 patients (56%) showed excellent results, 15 patients (11%) showed good results, 13 patients (9%) showed fair results and 34 patients (24%) showed poor repigmentation. This difference may be due to long follow-up period (6 years) and higher number of patients in their study [55]. Pandya et al. [56]. Reported an excellent results in 52.17% of patients treated by autologous melanocyte rich cell suspension (AMRCS) technique and in 50% of patients treated by melanocyte culture (MC) technique. The present results are also less than those reported by Elramly et al. [57] in 20 vitiligo patients, who showed that repigmentation was excellent at 7 (35%) of the treated sites and good for more than half of the treated sites (55.0%). While fair repigmentation was meet among only 2 (10.0%) of the treated areas and none of the treated areas showed poor repigmentation. This could be explained by the fact that their study included only focal and segmental types of vitiligo and the highest proportion of the studied patients (60.0%) was in the age group less than 15 years.

REFERENCES

1- Olsson M.J. and Juhlin L.: Long-term follow-up of leucoderma patients treated with transplants of autologous cultured melanocytes, ultrathin epidermal sheets and basal cell layer suspension. Br. J. Dermatol., 147 (5): 893-904, 2002.

2- Piotrowska A., Wierzbicka J. and Żmijewski M.A.: Vitamin D in the skin physiology and pathology. ABP, 63 (1): 17-29, 2016.

3- Ortonne J.P. and Passeron T.: Vitiligo and other disorders of hypopigmentation. In : Bolognia Text Book of Dermatology; Bolognia J.L., Jorizzo J.L. & Schaffer J.V. (eds); Elseveier Publishing; 3rd edition; Chapter 66, Pp 1023-1048, 2012.

4- Mulekar S.V., Issa A.A. and Eisa A.A.: Treatment of post-burn leucoderma with non-cultured melanocyte-keratinocyte transplantation (MKTP). Burns, 37 (3): 448-452, 2011.

5- Henderson M.D., Huggins R.H., Mulekar S.V., Ozog D., Lim H.W. and Hamzavi I.H.: Autologous noncultured melanocyte-keratinocyte transplantation procedure in an African American man with postburn leukoderma. Arch. Dermatol., 147 (9): 1025-1028, 2011.

6- Chen Y.F., Yang P.Y., Hu D.N., Kuo F.S., Hung C.S. and Hung C.M.: Treatment of vitiligo by transplantation of cultured pure melanocyte suspension: Analysis of 120 cases. J. Am. Acad. Dermatol., 51 (1): 68-74, 2004.

7- Abdel-malek Z. and Kadekaro A.L.: Human pigmentation: Its regulation by ultraviolet light and by endocrine, paracrine, and autocrine factors. In: The Pigmentary System: Physiology and Pathophysiology. Nordlund J.J., Boissy R.E., Hearing V.J., King R.A., Oetting W.S. & Ortonne J.P. (eds); Oxford: Blackwell Publishing, 2nd edn.; Pp 410-420, 2006.

8- James W.D., Berger T.G. and Elston D.M.: Skin basic structure and function. In: Andrews' Diseases of the Skin. Saunders Elsevier, 10th edition; Vol 1; Chapter I; Pp 1-4, 2006.

9- Cichorek M., Wachulska M., Stasiewicz A. and Tymińska A.: Skin melanocytes: Biology and development. Postepy Dermatol. Alergol., 30 (1): 30-41, 2013.

10- Tsatmali M., Ancans J. and Thody A.J.: Melanocyte function and its control by melanocortin peptides. J. Histochem. Cytochem., 50 (2): 125-133, 2002.

11- Slominski A., Tobin D.J., Shibahara S. and Wortsman J.: Melanin pigmentation in mammalian skin and its hormonal regulation. Physiol. Rev., 84 (4): 1155-1228, 2004.

12- Nielsen K.: Malignant melanoma-Risk factors and the CDKN2A mutation in relation to phenotypes and other cancers. Lund University, Faculty of Medicine, p 15, 2009.

13- Hearing V.J.: Biogenesis of pigment granules: A sensitive way to regulate melanocyte function. J. Dermatol. Science, 37 (1): 3-14, 2005.

14- Bolognia J.L. and Orlow S.J.: Melanocyte biology. In : Dermatology; Bolognia J.L., Jorizzo J.L. & Rapini R.P. (eds). Mosby Elsevier; Second edition; Section 10; Chapter 65; Pp 935-945, 2008.

15- Prota G., D'ischia M. and Napolitano A.: The chemistry of melanins and related metabolites. In: The pigmentary system; Nordlund J.J., Boissy R.E. & Hearing V.J. (eds). New York: Oxford University Press, Inc., Pp 307-332, 1998.

16- Schiaffino M.V.: Signaling pathways in melanosome biogenesis and pathology. Int. J. Biochem. Cell Biol., 42 (7): 1094-1104, 2010.

17- Van Den B.K., Naeyaert J.M. and Lambert J.: The quest for the mechanism of melanin transfer. Traffic, 7 (7): 769-778, 2006.

18- Hsu M.Y., Li L. and Herlyn M.: Cultivation of normal human epidermal melanocytes in the absence of phorbol esters. Methods Mol. Med., 107: 13-28, 2005.

19- Halaban R.: The regulation of normal melanocytes proliferation. Pigment Cell Res., 13 (1) : 4-14, 2000.

20- Hirobe T.: Role of leukaemia inhibitory factor in the regulation and differentiation of neonatal mouse epidermal melanocytes in culture. J. Cell Physiol., 192 (3): 315-326, 2002.

21- De W.N.J., Van M.G.N. and Ruiter D.J.: Immunohistochemistry in melanocytic proliferative lesions. Histopathology, 44 (6): 517-541, 2004.

22- Ohsie S.J., Sarantopoulos G.P., Cochran A.J. and Binder S.W.: Immunohistochemical characteristics of melanoma. J. Cutan Pathol., 35 (5): 433-444, 2008.

23- Cochran A.J. and Wen D.R.: S-100 protein as a marker for melanocytic and other tumours. Pathology, 17 (2): 340-345, 1985.

24- Trefzer U., Rietz N., Chen Y., Chen Y., Audring H., Siegel P., Reinke S., Koniger P., Wu S., Ma J., Liu Y., Wang H., Sterry W. and Guo Y.: SM5-1 : A new monoclonal antibody which is highly sensitive and specific for melanocytic lesions. Arch. Dermatol. Res., 292 (12): 583-589, 2000.

25- Fernando S.S., Johnson S. and Bäte J.: Immunohistochemical analysis of cutaneous malignant melanoma: Comparison of S-100 protein, HMB-45 monoclonal antibody and NKI/C3 monoclonal antibody. Pathology, 26 (1): 16-19, 1994.

26- Sundram U., Harvell J.D., Rouse R.V. and Natkunam Y.: Expression of the B-cell proliferation marker MUM1 by melanocytic lesions and comparison with S100, gp100 (HMB45) and Melan A. Mod. Pathol., 16 (8): 802-810, 2003.

27- Prieto V.G. and Shea C.R.: Immunohistochemistry of melanocytic proliferations. Arch. Pathol. Lab. Med., 135 (7): 853-859, 2011.

28- Granter S.R., Weilbaecher K.N., Quigley C. and Fisher D.E.: Role for micropthalmia transcription factor in the diagnosis of metastatic malignant melanoma. Appl Immunohistochem. Mol. Morphol., 10 (1): 47-51, 2002.

29- Hafbauer G.F., Kamarashev J., Geertsen R., Boni R. and Dummer R.: Tyrosinase immunoreactivity in formalin-fixed, paraffin-embedded primary and metastatic melanoma: Frequency and distribution. J. Cutan. Pathol., 25 (4): 204-209, 1998.

30- Clarkson K.S., Sturdgess I.C. and Molyneux A.J.: The usefulness of tyrosinase in the immunohistochemical assessment of melanocytic lesions: A comparison of the novel T311 antibody (anti-tyrosinase) with S100, HMB45, and A103 (anti-melan-A). J. Clin. Pathol., 54 (3): 196-200, 2001.

31- Busam K.J., Kucukgol D., Sato E., Frosina D., Teruya-feldstein J. and Jungbluth A.A.: Immunohistochemical analysis of novel monoclonal antibody PNL2 and comparison with other melanocyte differentiation markers. Am. J. Surg. Pathol., 29 (3): 400-406, 2005.

32- Miettinen M., Fernandez M., Franssila K., Gatalica Z., Lasota J. and Sarlomo-Rikala M.: Micropthalmia transcription factor in the immunohistochemical diagnosis of metastatic melanoma: Comparison with four other melanoma markers. Am. J. Surg. Pathol., 25 (2): 205-211, 2001.

33- Halder R.M. and Chappell J.L.: Vitiligo update. Semin Cutan Med. Surg., 28 (2): 86-92, 2009.

34- Torello L., Alessia G., Zanieri F., Colucci R. and Moretti S.: Vitiligo: New and emerging treatments. Dermatol. Ther., 21: 110-117, 2008.

35- Prignano F., Pescitelli L., Becatti M., Di Gennaro P., Fiorillo C., Taddei N. and Lotti T.: Ultrastructural and functional alterations of mitochondria in perilesional vitiligo skin. J. Dermatol. Sci., 54 (3): 157-167, 2009.

36- Buggiani G., Prignano F., Krysenka A., Pescitelli L. and Lotti T.: Vitiligo: A review about causes, clinical aspects, and therapies. J. Clin. Dermatol., 164 (3): 645-647, 2011.

37- Massi D.: Histopathological and ultrastructural features of vitiligo. Basic and Clinical Dermatology, 29: 145-158, 2004.

38- Alikhan A., Felsten L.M., Daly M. and Petronic-Rosic V.: Vitiligo: A comprehensive overview Part 1. Introduction, epidemiology, quality of life, diagnosis, differential diagnosis, associations, histopathology, etiology, and work-up. J. Am. Acad. Dermatol., 65 (3): 473-491, 2011.

39- Elder D.E., Elenitsas R., Johnson B.L. Jr., Murphy G.F. and Xu X.: Lever's Histopathology of the Skin; 10th edn; Lippincott Williams & Wilkins, New York, Pp 694 – 695,2009.

40- Lahiri K., Malakar S., Sarma N. and Banerjee U.: Inducing repigmentation by regrafting and phototherapy (311nm) in punch grafting failure cases of lip vitiligo: A pilot study. Indian J. Dermatol. Venereol. Leprol., 70 (3): 156-158, 2004.

41- Mutalik S. and Ginzburg A.: Surgical management of stable vitiligo: A review with personal experience. Dermatol. Surg., 26 (3): 248-254, 2000.

42- Chandrashekar B.S., Madura C. and Varsha D.: Autologous mini punch grafting: An experience of using motorized power punch in 10 patients. J. Cutan. Aesthet. Surg., 7 (1): 42- 45, 2014.

43- Majid I.: Grafting in vitiligo: How to get better results and how to avoid complications. J. Cutan. Aesthet. Surg., 6 (2): 83-89, 2013.

44- Gupta D.K.: Thin and ultra thin split thickness skin grafts (STSG-UT, STSG-T). In: Microskin Grafting for Vitiligo, Chapter (5); Pp15-18; Springer Science, 2009.

45- Gupta D.K.: Surgical therapies; In: Microskin Grafting for Vitiligo, Chapter (1); Pp 3 - 6; Springer Science, 2009.

46- Falabella R.: Surgical approaches for stable vitiligo. Dermatol. Surg., 31 (10): 1277-1284, 2005.

47- Plott R.T., Brysk M.M., Newton R.C., Raimer S.S. and Rajaraman S.: A surgical treatment for vitiligo: Autologous cultured-epithelial grafts. J. Dermatol. Surg. Oncol., 15 (11): 1161-1166, 1989.

48- Van Geel N., Goh B.K., Wallaeys E., DeKeyser S. and Lambert J.: A review of non-cultured epidermal cellular grafting in vitiligo. J. Cutan. Aesthet. Surg., 4 (I): l7-22, 2011.

49- Malakar S. and Dhar S.: Repigmentation of vitiligo patches by transplantation of hair follicles. Int. J. Dermatol., 38 (3): 237-238, 1999.

50- Gauthier Y. and Surleve-bazeille J.E.: Autologous grafting with non-cultured melanocytes: A simplified method for treatment of depigmented lesions. J. Am. Acad. Dermatol., 26 (2Pt 1): 191-194, 1992.

51- Tega G.R., Parsad D., Majumdar S. and Kumar B.: Efficacy of autologous transplantation of noncultured epidermal suspension in two different dilutions in the treatment of vitiligo. Int. J. Dermatol., 45 (2): 106-110, 2006.

52- El-Zawahry B.M., Zaki N.S., Bassiouny D.A., Sobhi R.M., Zaghloul A., Khorshied M.M. and Gouda H.M.: Autologous melanocyte-keratinocyte suspension in the treatment of vitiligo. J. Acad. Dermatol. Venereol., 25 (2): 215-220, 2011.

53- Huggins R.H., Henderson M.D., Mulekar S.V., Ozog D.M., Kerr H.A., Jabobsen G., Lim H.W. and Hamzavi I.H.: Melanocyte-keratinocyte transplantation procedure in the treatment of vitiligo: The experience of an academic medical center in the United States. J. Am. Acad. Dermatol., 66 (5): 785-793, 2012.

54- Mohammed N., Elbeheiry A., Elramly M., Saber H., Kamel L. and Soror O.: Transplantation of non cultured melanocytes versus dermabration and split thickness grafting in cases of non progressive vitiligo and stable forms of leucoderma. MD. thesis Alexandria Dermatology Department, 2002.

55- Mulekar S.V.: Long-term follow-up study of 142 patients with vitiligo vulgaris treated by autologous, non-cultured melanocyte-keratinocyte cell transplantation. Int. J. Dermatol., 44 (10): 841-845, 2005.

56- Pandya V., Parmar K.S., Shah B.J. and Bilmoria F.E.: A study of autologous melanocyte transfer in treatment of stable vitiligo. Indian J. Dermatol., 6: 393-397, 2005.

57- Elramly M., Nagat S.M. and Ali A.A.: Assessment of transplantation of autologous non cultured melanocytes-keratinocytes in treatment of segmental and focal vitiligo. Master Thesis submitted to the Faculty of Medicine, Alexandria University, 2011.
