# Supplementary material for: Therapeutic efficacy of cell-based therapy in vitiligo: a research letter systematically reviewed using meta-analysis
Source: Arch Dermatol Res. 2024 May 22;316(5):198. doi: 10.1007/s00403-024-02920-6 (PMC11111487; doi:10.1007/s00403-024-02920-6)
Supplement: Supplementary file 1 — Supplementary file1 (ZIP 24195 KB) [file 403_2024_2920_MOESM1_ESM.zip › Studies were included/Khodadadi 2010.pdf]

Arch Dermatol Res (2010) 302:593–599
DOI 10.1007/s00403-010-1034-7

ORIGINAL PAPER

Intraepidermal injection of dissociated epidermal cell suspension improves vitiligo

Laleh Khodadadi · Saeed Shafieyan · Masoud Sotoudeh · Ahmad Vosough Dizaj · Abdolhossein Shahverdi · Nasser Aghdami · Hossein Baharvand

Received: 13 November 2009 / Revised: 21 January 2010 / Accepted: 25 January 2010 / Published online: 4 April 2010
© Springer-Verlag 2010

Abstract This study was initiated to evaluate the safety and effectiveness of intraepidermal injection of dissociated epidermal cells into the lesions of stable vitiligo patients. Autologous dissociated epidermal cell suspensions were injected intraepidermally into 10 stable vitiligo patients. None of the patients received adjuvant therapy. The response was evaluated as: marked (76–100%), moderate (51–75%), mild (26–50%) and minimal repigmentation (0–25%). Transmission electron microscopy was used to evaluate the transplanted cells and immunohistochemical staining with HMB-45 was performed to assess the repigmentation in vivo. In all cases, repigmentation started during the 4-week period after transplantation. Six months after transplantation, a marked repigmentation in four (40%), moderate repigmentation in two (20%) and mild repigmentation in two (20%) patients were observed. Two (20%) patients with white patches on their lids showed minimal repigmentation. No side effects were observed in any patients. Interestingly, repigmentation of gray hair in one patient, 4 months post transplantation was observed. Analysis of the ultrastructure of transplanted cells showed 1.5% of the cells had melanocyte morphology. HMB-45 positive cells were observed after cell transplantation. This method is an effective, simple and safe therapeutic option for stable vitiligo lesions.

Keywords Intraepidermal injection · Single epidermal cells · Transplantation · Vitiligo

The authors L. Khodadadi and S. Shafieyan contributed equally to this study.

L. Khodadadi · S. Shafieyan · A. V. Dizaj · A. Shahverdi · N. Aghdami (✉) · H. Baharvand
Department of Regenerative Medicine, Royan Institute for Stem Cell Biology and Technology, ACECR, P.O. Box 19395-4644, Tehran, Iran
e-mail: Nasser.Aghdami@RoyanInstitute.org

M. Sotoudeh
Department of Pathology and Laboratory Medicine, Shariati Hospital, Tehran University of Medical Sciences, Tehran, Iran

H. Baharvand (✉)
Department of Stem Cells and Developmental Biology, Royan Institute for Stem Cell Biology and Technology, ACECR, P.O. Box 19395-4644, Tehran, Iran
e-mail: Baharvand@RoyanInstitute.org

H. Baharvand
Department of Developmental Biology, University of Science and Culture, ACECR, Tehran, Iran

Introduction

Vitiligo depigmentation is considered to be a consequence of melanocyte disappearance [11, 12] mediated by the immune system, toxic materials [6, 14, 15], oxidative stress [6, 19], functional impairment of epidermal cells [6, 15], environmental factors and genotype [16, 20, 22]. These observations suggest that vitiligo affects the entire keratinocyte Langerhans cell–melanocyte unit (KLM) [15]. Besides its etiology, vitiligo has two physical and psychologic aspects, of which the psychological aspect of the disease is often undervalued or neglected [17].

There are many modalities for the treatment of vitiligo, but there is still a need for a treatment that is promptly effective. Replenishing melanocytes selectively within vitiligenous macules by autologous melanocytes is a promising treatment. In these cases, the melanocytes are prepared as noncultured melanocyte rich cell suspensions [8, 13] or cultured melanocyte suspensions [3]. Gauthier and

Surleve-Bazeille [8] first introduced the use of a noncultured melanocyte and keratinocyte suspension injected into blisters that are produced over depigmented lesions. The cell suspension is poured evenly from the pipette to the denuded surface, which is then covered with a collagen dressing after superficial dermabrasion [13] or induction of a blister by suction or cryosurgery [8]. However, the transplantation of a cell suspension due to its high fluidity, especially on curved areas, at the desirable recipient site is difficult. On the other hand, suction blister induction has some limitations in a few predilection sites of the body such as bony prominences, eyelids and lips. Also, leakage of the suspension out of the blisters can occur. A few disadvantages of cryosurgery are: lack of accuracy, difficult to endure for some patients and the possibility of hypopigmentation around the recipient area due to peripheral melanocyte damage [21].

Therefore, for repigmentation in vitiligo patients, we employed a simple intraepidermal transplantation of a noncultured dissociated epidermal cell suspension.

Materials and methods

Ten patients (4 male, 6 female) with focal or generalized vitiligo aged 17–52 years (mean 29.6 years) were recruited for this study. Duration of vitiligo varied from 2.5 to 23 years (mean 8.6 years). Patient characteristics are listed in Table 1.

These patients, based on clinical diagnosis, had all vitiligo types with a history of no response or recurrence after conventional therapies. The selection criterion included: vitiligo lesions stable for a minimum of 1 year prior to surgery, patients with no active infectious disease and patients over 12 years of age. The exclusion criteria comprised actively spreading vitiligo (unstable disease), positive Köbner phenomenon, history of hypertrophic scars or kleoid formation, treatment with immunosuppressive or cytotoxic medication within the past year, UV therapy in previous 6 months, pregnant patients and patients aged less than 12 years. All patients were observed up to 6 months post surgery.

Patients were briefed in detail about the treatment and informed consent was obtained from all patients. The study was conducted with the approval of the Institutional Review Board at Royan Institute. The trial was registered at NIH clinical trials (www.clinicaltrials.gov) with the identifier: NCT00631865.

In all patients, clinical photographs were taken before and after therapy by a Canon Powershot S5IS with the same iso and macro view. A square lattice grid on tracing paper was used for the assessment of size of the donor and transplanted areas, and the extent of spread of repigmentation.

The degree of repigmentation was assessed as follows: no response or less than 25%, minimal repigmentation; 26–50%, mild repigmentation; 51–75%, moderate repigmentation; >75%, marked repigmentation. Less than 25% repigmentation seen at the end of 3 months was labeled as treatment failure. Compared with the surrounding skin, the color of repigmentation was graded as follows: somewhat darker; somewhat lighter; the same.

Cell separation

About one-third to one-seventh of the recipient area as a partial thickness skin graft was obtained from the thigh–buttock junction under local epidermal anesthesia using 1.5–3 ml lidocaine (Fig. 1a).

The thigh–buttock junction has a low risk of scar formation after partial thickness graft and less cosmetic

Table 1 Characteristics and disease parameters of the patients

Patient no.	Sex	Age	Duration of disease (years)	History of AD	Type of AD	Stable disease duration	Type of vitiligo
1	Male	44	2.5	Yes	Alopecia areata (Beard area)	1	Focal
2	Female	52	3	No	–	2	Focal
3	Female	22	12	Yes	Hypothyroidism	3	Focal
4	Male	17	4	No	–	2	Generalized
5	Male	40	4	Yes	Alopecia areata	4	Generalized
6	Female	30	9	Yes	Thyroid dysfunction	1	Generalized
7	Female	17	12	No	–	3	Generalized
8	Female	31	23	Yes	Hypothyroidism	1	Generalized
9	Female	20	3	No	–	3	Focal
10	Male	23	14	Yes	Hyperthyroidism	3	Generalized

AD Autoimmune disease

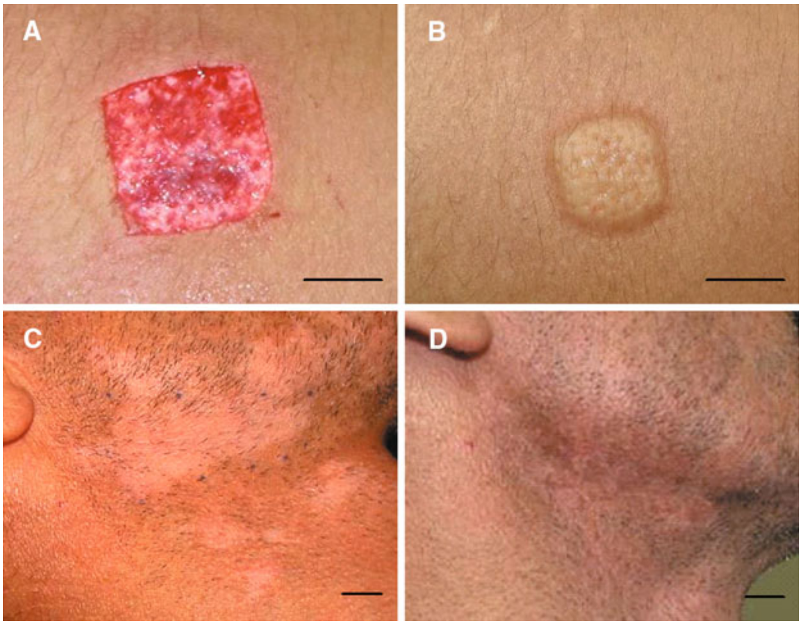

Fig. 1 **a** Partial thickness graft. **b** Intraepidermal injection of cell suspension into vitiligo patches. **c** Patient suffering from stable vitiligo before transplantation: at the right side of the face and neck, stable localized depigmented patches bearing white hairs. **d** Six months post transplantation: the repigmented area was indistinguishable from healthy skin. The formerly achromic hairs were repigmented. *Scale bar* 1 cm

significance. Moreover, it contains a relatively high concentration of melanocytes (1,900 ± 178 melanocytes/mm^2) and is considered to be a non-predilection location [7]. This partial thickness skin graft was immediately immersed in a test tube containing 10 ml transporting medium (Ca^{2+} and Mg^{2+}-free HBSS, Gibco, 14185) and transported to the laboratory.

In the laboratory, the skin specimen was soaked in 70% ethanol for 30 s to reduce contamination. Then, it was washed with HBSS twice, in a Petri dish and cut into pieces (approximately 2 × 3 mm^2) using a surgical scalpel blade with one-motion cuts. These skin pieces were incubated for 15–18 h at 4°C with dispase II, 1.2 U/ml (Gibco, 17105-041) solution in an inverted position. After incubation, the dispase solution was discarded and the tissues were washed with HBSS. The epidermis (thin translucent layer, yellowish brown color) was separated from the dermis (thick opaque layer, white color) using forceps and a scalpel blade. Subsequently, the dermis was immediately discarded in order to avoid fibroblast contamination. The collected epidermal sheets were transferred from the Petri dish to a centrifuge tube containing 0.25% trypsin/EDTA solution (Gibco, 15400) and incubated at 37°C for 30 min. The enzyme was inhibited by dilution with melanocyte growth medium (MGM-M2) (Promo Cell, C-39420) and the tube was centrifuged for 10 min at 1,000 rpm at room temperature. The supernatant was aspirated, which might contain remaining stratum corneum and the pellet was washed with HBSS and centrifuged for 10 min at 1,000 rpm.

Cell viability was checked by the dye exclusion test and in each sample, a total count of the epidermal suspension prepared for transplantation was performed using a hemocytometer. The cells at a density of 110–2000 cells/mm^2 of the recipient surface area were suspended depending on the transplanted surface area in 1–2 ml MGM-M2 for injection. The number and viability of isolated cells are given in Table 2.

Transplantation procedure

The recipient site which varied from 2.5 to 25 cm^2 (mean 9.6 cm^2) was cleaned, painted and draped with 10% povidone iodine, 70% ethanol and washed thoroughly with normal saline. The site was anesthetized with an intraepidermal injection of 1% lidocaine, a rapid anesthetic which caused the epidermal layers to bulge. Transplantation was safely performed by intraepidermal injection by an expert dermatologist using a very fine needle (30G, Soha Co., Tehran, Iran). There was no need for dermabrasion or induction of a suction blister (Fig. 1b). Injection was performed (0.05–0.1 ml/point) and the distance between the points was 0.5 cm. In this manner, the tumescent points would attach together to make an uniform filling of the recipient area (Fig. 1b). The time required to complete a transplantation procedure was approximately 5–10 min depending upon the number of patches, anatomical location(s) and total area of involvement. Following cell injection, no pressure was applied at the recipient site in order to avoid leakage. The area was covered with a light sterile dressing for only a few seconds and the patient was subsequently allowed to go home. Transplantation was done only once. The number of injected dissociated epidermal cells was (220–1,000 × 10^3; mean 571 × 10^3 cells) from donated surface (0.5–4 cm^2 ; mean 1.975 cm^2) areas (Table 2).

Table 2 The number and viability of isolated cells and surface sites of donation and injection

Patient no.	Donor surface area (cm^2)	No. of epidermal cells	Viability of cells (%)	Recipient surface area (cm^2)	Site of lesion
1	3	220×10^3	98	20	Right mandibular region
2	1	420×10^3	100	3.75	Back of the hand
3	0.5	500×10^3	99	2.5	Left side of the chin
4	1	700×10^3	96	5	Lids
5	3	500×10^3	100	12	Left cheek
6	3	650×10^3	98	9	Neck
7	2.25	700×10^3	100	7	Lids
8	1	500×10^3	100	7	Right breast
9	1	520×10^3	100	5	Left lateral side of the neck
10	4	1×10^6	100	25	Right foot

As a control in five patients with more than one lesion, only the vehicle (acellular medium) was injected in the same manner. These control lesioned areas were selected as same size and far enough to avoid any paracrine effects of transplanted cells.

Histological studies

We performed histological examination of two (2.5 mm) punch biopsies both before and 6 months after autologous transplantation in order to evaluate the existence and status of melanocytes post transplantation by immunohistochemical (IHC) staining for HMB-45. Briefly, after fixation in 10% neutral buffered formalin, the biopsy samples were processed following standard procedures and embedded in paraffin. Four to five micrometer sections were prepared from the paraffin blocks. One section of each block was stained by the hematoxylin and eosin method and examined by light microscope for sample integrity and quality. Then deparaffinized and rehydrated sections were subjected to hydrogen peroxide (one part 3% H_2O_2 in nine parts methanol) for 20 min to block the endogenous peroxidase. Antigen retrieval was performed by heating the sections in 10 mM citrate buffer (pH 6) in a microwave oven for two 20-min periods. Slides were cooled to room temperature for 20 min and rinsed in TBS for 5 min. Protein blocking was performed with 1/25 normal swine serum in TBS (Dako, X0901). Sections were incubated with ready to use mouse anti-human HMB45 (Dako, N1545) for 1 h at room temperature, washed in TBS and treated with the Dako REAL Envision detection system (Dako, K5007) following the kit's instructions. The slides were washed in tap water for 10 min, stained by hematoxyline for 30 s, dehydrated in gradual ethanol, treated with three changes of xylene and mounted by Entallen (Merck, 1.o7961.0500) in preparation for examination under a light microscope.

Electron microscopy processing

To analyze the melanocytes and keratinocytes of dissociated epidermal cells, we used transmission electron microscopy (TEM). The pellet of centrifuged cells was fixed by 2% glutaraldehyde in 0.1 M PBS (pH 7.4) for 2 h. After washing with PBS, the pellet was embedded in 1% agar, postfixed with 1% osmium tetraoxide for 1.5 h before being washed again in PBS, dehydrated in an acetone series and then embedded in epoxy resin. After resin polymerization, sections were cut and double stained with uranyl acetate and lead citrate. Electron micrographs were taken using a Zeiss EM 900 TEM (Zeiss, Oberkochen, Germany).

Results

Repigmentation started to occur after 4 weeks with hyperpigmentation and redness. At the end of 6-month follow-up, a marked repigmentation in four (40%), moderate repigmentation in two (20%) and mild repigmentation in two (20%) patients were observed. In the control lesioned areas, which received only vehicle, no repigmentation was observed.

At the end of 6-month follow-up, repigmentation was ongoing and expanding in patients with moderate repigmentation. Two (20%) patients with white patches on their lids showed minimal repigmentation. Interestingly, the formerly achromatic hairs in one patient, after 4 months post transplantation, were fully repigmented (Fig 1c, d). One patient with a white patch on her hand initially had excellent repigmentation after transplantation but due to a burn injury at the recipient site at month 4, the repigmentation was lost. The repigmentation, however, restarted at the same site after a lapse of 2 months. A temporarily slight color mismatch was observed in all patients but this improved to the same color in four (40%) patients with

marked repigmentation 6 months after the procedure. A summary of results is given in Table 3.

Three forms of repigmentation: uniform, punctuate and marginal occurred in our transplanted patients with no relation to personal and disease variables. We did not observe any correlations between start of repigmentation and total number of transplanted cells/mm^2, start of repigmentation and duration of the disease, history of autoimmune disease and familial history of vitiligo.

Immunohistochemical staining with HMB-45 (Fig. 2) revealed the absence or marked sparsity of melanosomes in the basal layer of the epidermis before transplantation. However, HMB-45 positive cells were found in the basal layer of biopsies after autologous melanocyte transfer. We performed ultrastructural analysis of the transplanted cells to confirm and distinguish melanocytes from keratinocytes. Analysis of the ultrastructure of transplanted cells showed 1.5% (3:200) of the cells with melanocyte morphology. The other cells (98.5%) were heavily pigmented keratinocytes (Fig. 3). The cytoplasm of keratinocytes contained numerous bundles of filaments that form tonofibrils. Many of these pass into the cytoplasmic process and terminate in the desmosomes. Whereas the melanosomes of melanocytes occur singly, those of keratinocytes are found in clusters of varying sizes, enclosed by a membrane.

Discussion

Here we introduced an approach to replenish melanocytes by injection of an autologous dissociated epidermal cell suspension (110–2,000 cells/mm^2 with approximately 1.5% melanocytes). Some changes in comparison to the previous techniques were made: (1) in the isolation technique, to dissociate epidermal cell suspension, we used dispase to separate the epidermal and dermal layers. Since dispase splits the epidermis from the dermis along the basement membrane, probably epidermal stem cells located in the basal layer can be isolated with less loss [2]. (2) To avoid any side effects of the transplantation technique, such as scar formation and Köbner phenomenon induction, we intraepidermally injected cells from the cell suspension. (3) To evaluate cell therapy effect, no adjuvant therapy, such as UV therapy was added after grafting. In four (40%) patients, a remarkable repigmentation of greater than 85% was obtained, while in two patients (20%) with white patches on their lids, minimal repigmentation was observed. This seemed to be due to the varied thickness of the epidermis in different parts of the body. The epidermis is thickest on the palms and soles, measuring approximately 1.5 mm; while it is very thin on the eyelids, measuring less than 0.1 mm [10]. Probably, the injection of cells on the eyelids of our patients with no pigmentation leaked to the dermal layer and the cells were not located in the right position.

It has been suggested that a decreased concentration of melanocyte growth factor(s) could play a role in the pathogenesis of vitiligo [18]. The keratinocytes produce soluble factors that regulate melanocyte proliferation, dendricity, melanization and also produce growth factors that are mitogenic for melanocytes (e.g. bFGF and TGF-α). In consideration of the keratinocyte defects in vitiligo patients that affect melanocytes, we transplanted the epidermal cell suspension in MGM-M2, not only to compensate for the growth defect, but also to substitute impaired epidermal cells with normal ones. MGM-M2 is a highly buffered medium made by mixing equal quantities of DMEM/F12 + HEPES:buffering system containing 5 ng/ml recombinant human bFGF and is a specific medium for the melanocyte population [5]. Therefore, we assumed that usage of MGM-M2 for a cell suspension

Table 3 Summary of results after cell transplantation in vitiligo patients

No. of patients	Response grading	Color matching
4/10	Marked repigmentation	Same
2/10	Moderate repigmentation	Lighter
2/10	Mild repigmentation	Lighter
2/10	Minimal repigmentation	No

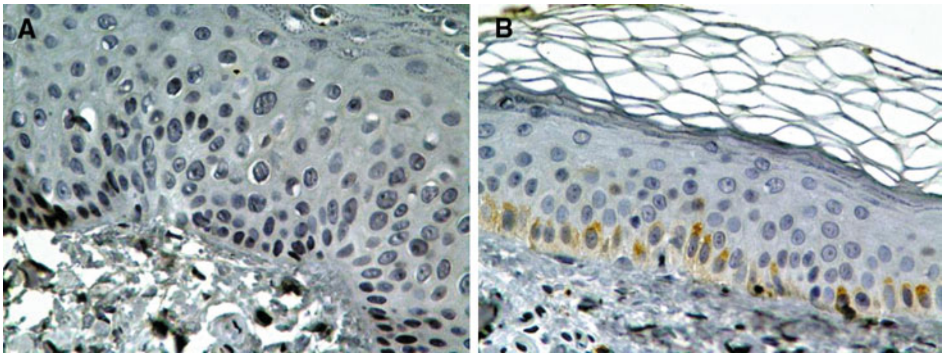

Fig. 2 Immunohistochemistry of epidermal cells of skin biopsy. Sections of skin before (**a**) and after (**b**) transplantation stained with HMB 45 reveal regularly distributed cells positive for HMB-45 in the basal layer of the epidermis after transplantation (magnification, ×40)

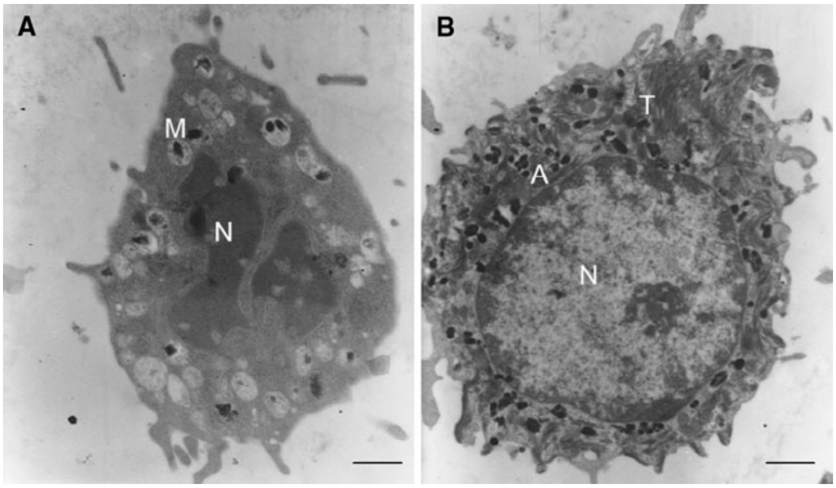

Fig. 3 Ultrastructure characteristics of dissociated epidermal cells. TEM micrograph of a melanocyte (**a**) and a heavily pigmented keratinocyte (**b**). The cytoplasm contains numerous bundles of filaments that form tonofibrils. The melanosomes of melanocytes occur singly; those of keratinocytes are found in clusters of varying sizes enclosed by a membrane. *N*: nucleus, *T*: tonofilament bundle, *M*: melanosome. *Scale bar* 2 μm

injection might enhance the multiplication of melanocytes or the recruitment of melanocytes to the lesion area.

Beause, a subpopulation of residual epidermal melanocytes persists apart from the duration of the disease, repigmentation could occur after specific stimulation [1]. It may be that the technique of intraepidermal injection is the mentioned stimulation. The transplanted melanocytes themselves or recruitment of melanocytes in hair follicles, by factors derived from transplanted cells is another explanation for this phenomenon [4].

Stable vitiligo and lesions with depigmented hairs indicating the depletion of the melanocyte reservoir in the hair follicle, mostly fail to repigment by conservative therapies. Hartmann et al. [9] presented for the first time, the transplantation of cultured melanocyte suspensions embedded in a physiological fibrin matrix, leading to a complete and stable repigmentation of both skin and hairs in one patient. We also observed the repigmentation of gray hair in one patient after 4 months post transplantation.

This method to treat stable vitiligo is simple, effective and can be carried out as an outpatient treatment. Patients with generalized and focal vitiligo can look forward to complete pigmentation of affected areas, without scarring or a cobblestone appearance. Repigmentation is likely to remain for a prolonged period. The time required to complete a transplantation procedure is approximately 5–10 min depending upon the number of patches, anatomical location(s) and total area of involvement. There is no need for any hospitalization after grafting and patients could resume their normal daily activities with no post transplant pain. It is notable that no recurrence was observed in any of the patients after 6 months follow-up. In our series, it was seen that only one patient did not improve due to burning of the recipient site after transplantation. However, after 2 months, the same site was repigmented.

It should be emphasized that this method replenishes missing melanocytes and in the long-run should prove to be a cosmetically acceptable, fast and cost-effective therapy. The use of this technique does not require special laboratory equipment. The procedure of transplantation, in comparison with other techniques, is a less expensive and time-consuming procedure. In spite of this, the technique seems to have limitations in terms of area, which can be treated in one session and the variable number of extracted cells per same location and surface area. Further attention should be paid to variables such as: cell concentration, donation site, relation to skin type and transplantation technique. To confirm our results and to evaluate the permanency of repigmentation, we have started a long-term follow up.

Acknowledgments We are grateful to Mr. Piryaei, for technical support. This study was supported by a grant from Royan Institute.

Conflict of interest statement None.

References

1. Akama K, Tatsuno R, Otsu M et al (2008) Proteomic identification of differentially expressed genes in mouse neural stem cells and neurons differentiated from embryonic stem cells in vitro. Biochim Biophys Acta 1784:773–782
2. Alonso L, Fuchs E (2003) Stem cells of the skin epithelium. Proc Natl Acad Sci USA 100(Suppl 1):11830–11835
3. Chen YF, Chang JS, Yang PY, Hung CM, Huang MH, Hu DN (2000) Transplant of cultured autologous pure melanocytes after laser-abrasion for the treatment of segmental vitiligo. J Dermatol 27:434–439
4. Czajkowski R (2004) Comparison of melanocytes transplantation methods for the treatment of vitiligo. Dermatol Surg 30:1400–1405
5. Czajkowski R, Placek W, Drewa T, Olszewska-Slonina D, Sir J, Kowaliszyn B, Weiss M (2006) Establishing melanocyte cultures

in a serum-free system for transplantation in vitiligo patients. Med Sci Monit 12:CR63–CR69
6. Dell'anna ML, Picardo M (2006) A review and a new hypothesis for non-immunological pathogenetic mechanisms in vitiligo. Pigment Cell Res 19:406–411
7. Fitzpatrick TB, Eisen AZ, Wolff K, Freedberg IM, Austen K (1993) Dermatology in general medicine. McGraw-Hill, NewYork
8. Gauthier Y, Surleve-Bazeille JE (1992) Autologous grafting with noncultured melanocytes: a simplified method for treatment of depigmented lesions. J Am Acad Dermatol 26:191–194
9. Hartmann A, Broecker EB, Hamm H (2008) Repigmentation of skin and hairs in stable vitiligo by transplantation of autologous melanocytes in fibrin suspension. J Eur Acad Dermatol Venereol 22:624–626
10. James W, Berger TG, Elston DM (2006) Andrews diseases of the skin clinical dermatology. Saunders, Philadelphia
11. Le Poole IC, van den Wijngaard RM, Westerhof W, Das PK (1997) Tenascin is overexpressed in vitiligo lesional skin and inhibits melanocyte adhesion. Br J Dermatol 137:171–178
12. Le Poole IC, van den Wijngaard RM, Westerhof W, Dutrieux RP, Das PK (1993) Presence or absence of melanocytes in vitiligo lesions: an immunohistochemical investigation. J Invest Dermatol 100:816–822
13. Olsson MJ, Juhlin L (1998) Leucoderma treated by transplantation of a basal cell layer enriched suspension. Br J Dermatol 138:644–648
14. Ongenae K, Van Geel N, Naeyaert JM (2003) Evidence for an autoimmune pathogenesis of vitiligo. Pigment Cell Res 16:90–100
15. Ortonne JP, Bose SK (1993) Vitiligo: where do we stand? Pigment Cell Res 6:61–72
16. Passeron T, Ortonne JP (2005) Physiopathology and genetics of vitiligo. J Autoimmun 25(Suppl):63–68
17. Pianigiani E, Andreassi A, Andreassi L (2005) Autografts and cultured epidermis in the treatment of vitiligo. Clin Dermatol 23:424–429
18. Ramaiah A, Puri N, Mojamdar M (1989) Etiology of vitiligo. A new hypothesis. Acta Derm Venereol 69:323–326
19. Schallreuter KU, Chavan B, Rokos H, Hibberts N, Panske A, Wood JM (2005) Decreased phenylalanine uptake and turnover in patients with vitiligo. Mol Genet Metab 86(Suppl 1):S27–S33
20. Spritz RA (2006) The genetics of generalized vitiligo and associated autoimmune diseases. J Dermatol Sci 41:3–10
21. van Geel N, Ongenae K, De Mil M, Naeyaert JM (2001) Modified technique of autologous noncultured epidermal cell transplantation for repigmenting vitiligo: a pilot study. Dermatol Surg 27:873–876
22. Zhang XJ, Chen JJ, Liu JB (2005) The genetic concept of vitiligo. J Dermatol Sci 39:137–146
