# Supplementary material for: Therapeutic efficacy of cell-based therapy in vitiligo: a research letter systematically reviewed using meta-analysis
Source: Arch Dermatol Res. 2024 May 22;316(5):198. doi: 10.1007/s00403-024-02920-6 (PMC11111487; doi:10.1007/s00403-024-02920-6)
Supplement: Supplementary file 1 — Supplementary file1 (ZIP 24195 KB) [file 403_2024_2920_MOESM1_ESM.zip › Studies were included/Lee 2010.pdf]

The superior ophthalmic vein drains the orbital and facial veins into a cavernous sinus. To our knowledge, this is the first case of superior ophthalmic vein thrombosis (SOVT) reported in streptococcal facial cellulitis. SOVT may lead to cavernous sinus thrombosis (CST), a serious event, with a mortality rate of about 20%.[1] Early diagnosis of SOVT, usually by MR angiography, may prevent this complication.

CST occurs most frequently in staphylococcal infections of the mid face. Staphylococci produce coagulase, which can induce microthrombosis formation, subsequent embolization in facial and ophthalmic veins, and finally a cavernous sinus. Streptococci do not produce coagulase, and in fact thromboses are rarely described in streptococcal infections. The pathophysiological mechanism of the thrombosis in our patient was probably vascular stagnation induced by the collection of pus and formation of a septic thrombus.

The clinical features of SOVT are chemosis, oedema of the superior and inferior eyelids, ophthalmoplegia, and nasogenian and internal canthal swelling, without neurological sign or loss of vision. Our patient's visual loss could not be explained by the ophthalmological examination; however, severe oedema could inflict ischaemic optic-nerve damage due to formation of reactive oxygen species, which may sometimes be irreversible.[2] In this way, pus collection in the upper eyelid could have been responsible for venous stasis and SOVT and ultimately for compression injury to the optic nerve. Other causes of sudden blindness during facial cellulitis are central retinal artery or vein occlusion, CST, intraocular abscess, and diffusion of facial infection by contiguity into the eyeball.

The place of anticoagulation in SOVT and in CST is unclear. Some authors believe that it can prevent dissemination of septic emboli and thrombosis. Others believe that it can induce haemorrhagic lesions in the orbit or brain, whereas clotting would limit the spread of infection.[3]

Systematic eye examination should be considered in severe soft tissue infections of the face. Ophthalmic vein thrombosis as reported here is rare, but could represent the first stage of cavernous sinus thrombophlebitis.

A. Caudron, A. Dadban, C. Brochart,* V. Viseux, T. Nguyen,* G. Baglin† and C. Lok

*Departments of Dermatology, *Radiology and †Ophthalmology, Amiens University Hospital, F-80054 Amiens, Cedex 1, France*

E-mail: alidadban@gmail.com

Conflict of interest: none declared.

Accepted for publication 22 September 2008

References

1 Schmitt NJ, Beatty RL, Kennerdell JS. Superior ophthalmic vein thrombosis in a patient with dacryocystitis-induced orbital cellulitis. *Ophthal Plast Reconstr Surg* 2005; **21**: 387–9.

2 Babovic S, Im MJ, Angel MF, Manson PN. Role of reactive oxygen species in optic nerve compression injury: a preliminary study. *Ann Plast Surg* 1998; **40**: 156–9.

3 Ogundiya DA, Keith DA, Mirowski J. Cavernous sinus thrombosis and blindness as a complication of an odontogenic infection: report of a case and review of literature. *J Oral Maxillofac Surg* 1989; **47**: 1317–21.

Epidermal grafting for vitiligo: a comparison of cultured and noncultured grafts

doi: 10.1111/j.1365-2230.2009.03429.x

Many patients with vitiligo and piebaldism have unsatisfactory responses to medical treatments such as topical corticosteroids and ultraviolet (UV) phototherapy. Surgical treatments such as epidermal grafting using suction blisters can be used when medical treatments fail.[1] Recently, autologous epidermal cultures containing melanocytes have been developed as an alternative to conventional surgical methods.[2] The main advantage to this new method is that a large amount of tissue can be produced from a small specimen.

In this study, we investigated the effectiveness of epidermal grafting, comparing the cultured and noncultured methods, in nine patients with stable vitiligo and one with piebaldism. Informed consent was obtained from each patient and our institutional human research review committee approved the study protocol.

In total, 10–12 suction blisters were obtained from all patients as described previously.[3] All patients received epidermal grafting immediately after the suction blisters were obtained.

One blister roof was used for making cultured epidermis. The cultured epidermis was prepared by coculturing of the keratinocytes and melanocytes as described previously,[4] and grafted within 3 weeks after the suction blisters (Fig. 1). In patients with generalized vitiligo, adjacent

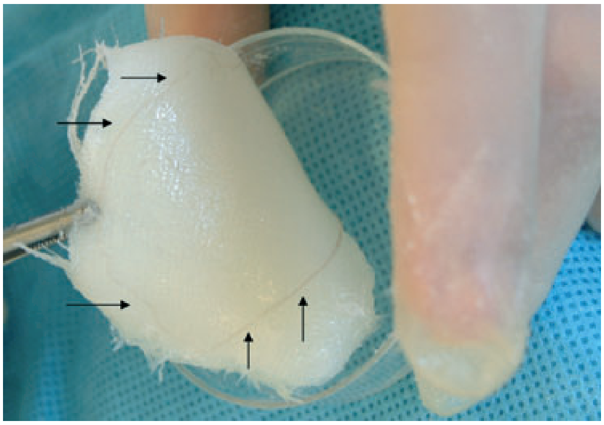

Figure 1 Cultured epidermal graft on petroleum jelly gauze immediately before grafting. Margins of the cultured epidermal graft are indicated by arrows.

Table 1 Clinical data of the patients.

Patient	Gender/Age	Clinical type	Treatment site	Outcome (after 1 year)	
				Noncultured	Cultured
1	F/24	Piebaldism	Abdomen	Excellent	Excellent
2	M/23	Segmental	Face	Excellent	Excellent
3	F/19	Segmental	Arm	Excellent	Excellent
4	F/53	Generalized	Back	Excellent	Excellent
5	F/43	Generalized	Abdomen	Good	Good
6	M/38	Generalized	Abdomen	Poor	Poor
7	M/36	Generalized	Abdomen	Poor	Poor
8	M/22	Generalized	Abdomen	Poor	Poor
9	F/39	Generalized	Abdomen	Poor	Poor
10	M/27	Generalized	Abdomen	Poor	Poor

Extent of repigmentation: excellent, 90–100%; good, 50–90%; fair, 20–50%; poor, 0–20%.

areas were grafted because the treatment effect is significantly different between lesion sites.

Two weeks after cultured epidermal grafting, topical psoralen UVA (PUVA) or narrowband UVB (NB-UVB) therapy was performed twice a week for 2–6 months depending on an outcome. PUVA was started at 0.1 J/cm^2, with subsequent increments of 0.1 J/cm^2 at alternate visits. NB-UVB was started at 250 mJ/cm^2, with subsequent increments of 15%. The dose was adjusted according to the sensitivity and tolerance of the individual patient. There was a limitation to the study, in that cultured and noncultured grafts might be of different ages when irradiated. The outcome of the treatment was evaluated every 3 months and the patients were followed up for > 12 months. The results obtained with the cultured epidermal grafts were very similar to those with the noncultured epidermal grafts (Table 1). For patients with segmental vitiligo and piebaldism, both noncultured and cultured methods were effective, but in five of the seven patients with generalized vitiligo, neither method was effective.

This study differs from previous studies on cultured epidermal grafts in that we compared cultured epidermal grafts with noncultured epidermal grafts in the same patients. The results showed that the cultured epidermal grafts were as effective as the noncultured epidermal grafts, and suggest that patients with a satisfactory response to noncultured epidermal grafts can be effectively treated with cultured epidermal grafts. However, the establishment of the cultures for cultured epidermal grafts may not always be successful in all patients. Thus, before a cultured epidermal graft, noncultured epidermal grafts may be useful to predict the effectiveness of a cultured graft.

D.-Y. Lee and J.-H. Lee

Department of Dermatology, Samsung Medical Center, Sungkyunkwan University School of Medicine, Seoul, Korea

E-mail: dylee@skku.edu

Conflict of interest: none declared.

Accepted for publication 4 February 2009

References

1 Falabella R. Epidermal grafting: an original technique and its application in achromic and granulating areas. *Arch Dermatol* 1971; **104**: 592–600.

2 Guerra L, Primavera G, Raskovic D *et al.* Erbium:YAG laser and cultured epidermis in the surgical therapy of stable vitiligo. *Arch Dermatol* 2003; **139**: 1303–10.

3 Lee DY, Choi YL, Kim MG *et al.* The effect of epidermal graft on a suction blistered donor wound. *Dermatol Surg* 2006; **32**: 1305–6.

4 Guerra L, Capurro S, Melchi F *et al.* Treatment of 'stable' vitiligo by timed surgery and transplantation of cultured epidermal autografts. *Arch Dermatol* 2000; **136**: 1380–9.

Lentigines following narrow-band ultraviolet B phototherapy for mycosis fungoides

doi: 10.1111/j.1365-2230.2009.03502.x

Mycosis fungoides (MF) is the most common form of cutaneous T-cell lymphoma. Early MF presents as erythematous patches or plaques without internal involvement. Narrowband ultraviolet B (NB-UVB) phototherapy is effective for early-stage MF, and carries a low risk of adverse events. We present a unique case of lentigines confined to the site of resolved MF plaques after NB-UVB phototherapy.

A 49-year-old Chinese man (Fitzpatrick phototype III) presented with a 1-year history of recurrent itchy erythematous patches and plaques. Physical examination revealed erythematous plaques on the flexor areas of the right thigh, and erythematous patches on both arms (Fig. 1a).

Histological examination of a biopsy taken from the plaque found intraepidermal Pautrier microabscesses and an intense infiltrate of mononuclear cells containing atypical lymphocytes in the upper dermis (Fig. 2a). Immunohistochemistry showed that most of the infiltrating cells were CD3 and CD4 positive. Results of blood cell count,

© 2009 The Author(s)
