# Supplementary material for: Therapeutic efficacy of cell-based therapy in vitiligo: a research letter systematically reviewed using meta-analysis
Source: Arch Dermatol Res. 2024 May 22;316(5):198. doi: 10.1007/s00403-024-02920-6 (PMC11111487; doi:10.1007/s00403-024-02920-6)
Supplement: Supplementary file 1 — Supplementary file1 (ZIP 24195 KB) [file 403_2024_2920_MOESM1_ESM.zip › Studies were included/Lommerts 2017.pdf]

BJD
British Journal of Dermatology

Autologous cell suspension grafting in segmental vitiligo and piebaldism: a randomized controlled trial comparing full surface and fractional CO_2 laser recipient-site preparations

J.E. Lommerts,[1] A.A. Meesters,[1] L. Komen,[1] M.W. Bekkenk,[1] M.A. de Rie,[1,2] R.M. Luiten[1] and A. Wolkerstorfer[1]

[1]Netherlands Institute for Pigment Disorders, Department of Dermatology, Academic Medical Centre, University of Amsterdam, Amsterdam, the Netherlands
[2]Department of Dermatology, VU Medical Centre, VU University, Amsterdam, the Netherlands

Correspondence
J.E. Lommerts.
E-mail: j.e.lommerts@amc.nl

Accepted for publication
31 March 2017

Funding sources
The harvesting devices were provided by Avita Medical.

Conflicts of interest
A.W. received a research grant from Avita Medical.

DOI 10.1111/bjd.15569

Summary

Background Autologous noncultured cell suspension transplantation is an effective treatment for repigmentation in segmental vitiligo and piebaldism. Full surface laser ablation is frequently used to prepare the recipient site before cell suspension transplantation, even though the optimal laser settings and ablation depth are unknown.

Objectives To assess the efficacy and safety of less invasive recipient-site preparations.

Methods In a randomized, observer-blinded, controlled trial we compared different recipient-site preparations before cell suspension transplantation in segmental vitiligo and piebaldism. In each patient, we randomly allocated three CO_2 laser recipient-site preparations (209 and 144 μm full surface, and fractional) and a control (no treatment) to four depigmentations. After 6 months we assessed repigmentation and side-effects.

Results We included 10 patients with vitiligo ($n = 3$) and piebaldism ($n = 7$). Compared with the control site, we found more repigmentation after full surface ablation at 209 μm (median 68·7%, $P = 0·01$) and 144 μm (median 58·3%, $P = 0·007$), but no repigmentation after fractional ablation (median 0·0%, $P = 0·14$).

Conclusions Superficial full surface ablation with a depth of 144 μm is an effective recipient-site preparation before cell suspension transplantation, while fractional CO_2 laser is not.

What's already known about this topic?

- The optimal ablation depth of recipient sites before cell suspension transplantation is unknown.

What does this study add?

- Superficial full surface ablation is an effective recipient-site preparation, while fractional CO_2 laser is not.

Vitiligo and piebaldism are depigmenting skin disorders that can significantly alter physical appearance and impair quality of life.[1,2] Autologous noncultured cell suspension transplantation (CST) is an effective treatment for repigmentation in segmental vitiligo and piebaldism.[3,4] The CST technique involves the transplantation of autologous epidermal cells, suspended in a fluid medium, from pigmented skin to depigmented recipient sites. Recipient-site preparation before CST is

© 2017 The Authors. British Journal of Dermatology
published by John Wiley & Sons Ltd on behalf of British Association of Dermatologists.
This is an open access article under the terms of the Creative Commons Attribution-NonCommercial License, which permits use, distribution and reproduction in any medium, provided the original work is properly cited and is not used for commercial purposes.

required to allow access to the underlying structures necessary for melanocyte adherence.[5] Full surface ablation is generally used as a recipient-site preparation in CST. Unfortunately, this technique can be uncomfortable and may result in persistent side-effects such as scarring and erythema.[6] Furthermore, the optimal depth of full surface ablation for melanocyte adherence is unclear.

In a previous study, we found 78% repigmentation after full surface CO_2 laser ablation to an estimated depth of 209 μm.[7] However, if less invasive recipient-site preparations are at least as effective in repigmentation, patients could be effectively treated more comfortably and with potentially fewer side-effects. Less invasive techniques include ablative fractional laser and superficial full surface ablation. In ablative fractional pretreatment, an array of microscopic ablation channels is created.[8] Less invasive techniques may result in minimized side-effects, such as persisting erythema and scars, and provide faster healing.[8–10]

The aim of this study was to assess the efficacy and safety of fractional and superficial full surface CO_2 laser ablation of the recipient sites before CST in segmental vitiligo and piebaldism.

Materials and methods

This prospective, randomized, observer-blinded, within-subject, controlled trial was approved by the medical ethical committee of the Academic Medical Centre, Amsterdam (#NL49720.018.14). This study is registered at the U.S. National Institutes of Health (ClinicalTrials.gov) as trial NCT02458417. We recruited and treated the patients at the Netherlands Institute for Pigment Disorders between May 2015 and February 2016. Eligible patients were aged 18 years or older, diagnosed with either stable segmental vitiligo or piebaldism, and able to give written informed consent. Stable segmental vitiligo was defined as the absence of new lesions or enlargement of existing lesions for 12 months without treatment. Furthermore, eligible patients had at least four depigmented lesions of 3 × 3 cm or one large lesion of at least 12 × 3 cm on the trunk or extremities. Patients with Fitzpatrick skin type I, recurrent herpes simplex virus infections, hypertrophic scarring, keloids, cardiac insufficiency, history of ultraviolet hypersensitivity, allergy for local anaesthetics and/or clarithromycin, or medical history with (non)melanoma skin cancer or atypical naevi, and patients who were breastfeeding or pregnant were excluded from participation.

Interventions

Cell suspension preparation

We infiltrated the donor area with lidocaine 2% and harvested a split-thickness skin sample of approximately 4 cm^2 with a depth of 0·2 mm from the hip with an electric dermatome (D42 Dermatome; Humeca, Beverwijk, the Netherlands). The skin sample was then placed in a cell harvesting device (ReCell®; Avita Medical, London, U.K.). Following previous studies[7,11–13] and the manufacturer's instructions, the skin sample was placed for approximately 15–20 min in a heated well containing an enzyme solution. Subsequently, the sample was rinsed in a sodium lactate solution, and disaggregation of cells from the epidermis and dermis was performed using a scalpel. Lastly, the suspension was drawn up in a syringe.

Recipient-site preparation

For recipient-site preparation, we used a 10 600 nm ablative CO_2 laser (Ultrapulse; Lumenis, Santa Clara, CA, U.S.A.) with a scanner handpiece suitable for full surface ablation (ActiveFx handpiece) and a handpiece for fractional ablation (DeepFx handpiece). In each patient, we randomly allocated four depigmented lesions of 4 cm^2 on the trunk or extremities to one of the following recipient-site preparations: (i) full surface ablation with an estimated depth of 209 μm (ActiveFx, one pass at 200 mJ, 60 W, density 3); (ii) full surface ablation with an estimated depth of 144 μm (ActiveFx, one pass at 150 mJ, 60 W, density 3); (iii) fractional laser ablation with an estimated depth of 225 μm (DeepFx, one pass at 7·5 mJ per microbeam, 20% density, 120 μm diameter) or (iv) untreated control site (no recipient-site preparation and no CST).

After infiltration of the treatment sites with lidocaine 2%, the recipient sites were prepared and the necrotic debris on the skin was removed with saline-soaked gauze. The cell suspension was applied on the recipient sites, with exemption of the untreated control site. We used a ratio of one to four or five (donor to recipient site), and the cell suspension was also applied to the donor site. The recipient-site preparations and CST procedures were performed by the same physicians.

Postoperative care

We covered the recipient and donor sites with a nonadherent, small-pore dressing (Telfa Clear; Covidien, Dublin, Ireland) and a secondary dressing with paraffin gauzes (Jelonet; Smith and Nephew, Hamburg, Germany) for 7 days. Furthermore, patients were advised to take clarithromycin 500 mg once daily for 7 days after CST to prevent infections. To make the repigmentation more visible, patients were advised to start with ultraviolet A treatment on all treatment sites twice weekly with a facial tanner (GB9212; Eurosolar, Bonn, Germany) from 4 weeks to 6 months after the CST.

Randomization and allocation concealment

Randomization was based on a digitally generated randomization list not known to any of the involved investigators (Graph Pad Software Inc., La Jolla, CA, U.S.A.). After inclusion, treatment regions were assigned, and numbered, opaque, sealed envelopes containing cards with the allocation were opened in ascending order. The randomization and allocation were concealed for the blinded assessor. The patient

© 2017 The Authors. *British Journal of Dermatology* published by John Wiley & Sons Ltd on behalf of British Association of Dermatologists.

and the treating physician were not blinded as this was practically impossible.

Outcomes

Primary outcome

The primary outcome of our study was the percentage of repigmentation per treatment site. This was measured by comparing surface areas of repigmentation copied on transparent sheets (baseline vs. 6 months) using a digital image analysis system (Image J; https://imagej.nih.gov/ij/).

Secondary outcomes

One week after CST, re-epithelialization (0–100%) and pain (on a visual analogue scale) per treatment site were assessed separately by the physician and patient. Six months after CST, a blinded independent physician assessed the repigmentation and side-effects (i.e. erythema, hyperpigmentation, hypopigmentation, scarring) per treatment site. Furthermore, colour matching with normal skin was measured using a reflectance meter (DermaSpectrometer; Cortex Technology ApS, Hadsund, Denmark). Patients and physicians were asked to evaluate the global outcome per treatment site measured on a four-point scale (poor, moderate, good or excellent).

Statistical analyses

Statistical analyses were performed using SPSS (version 23; IBM Corp., Armonk, NY, U.S.A.). The Wilcoxon signed-rank test was used to compare the repigmentation percentages between recipient sites, and the significance level was set at $\alpha = 0{\cdot}05$. All patients were included in the statistical analyses. Non-normally distributed data are presented as medians with their interquartile ranges (IQRs).

Results

We included 10 patients with either segmental vitiligo ($n = 3$) or piebaldism ($n = 7$). The median age was 22·5 years and the ratio male to female was 4 : 6. None of the patients was lost to follow-up. The baseline characteristics are presented in Table 1.

Repigmentation

Repigmentation percentages per recipient site are presented in Figure 1. The median repigmentation after 209-µm (68·7%, IQR 12·7–100·0, $P = 0{\cdot}011$) and 144-µm (58·3%, IQR 14·3–100·1, $P = 0{\cdot}007$) full surface ablation were significantly higher than at the control site (0%, IQR 0–0). No significant differences between fractional recipient-site preparation (median 0%, IQR 0–11·9) and the control site were found ($P = 0{\cdot}14$). Repigmentation was significantly higher for 209-µm ($P = 0{\cdot}012$) and 144-µm ($P = 0{\cdot}008$) full surface ablation than for fractional ablation. Furthermore, no significant difference in repigmentation was found between 209-µm and 144-µm full surface ablation ($P = 0{\cdot}89$).

More than 75% repigmentation was found in 50% and 40% of the 209-µm and 144-µm full surface ablated recipient sites, respectively. The global assessment of repigmentation corresponded with the repigmentation percentages measured on transparent sheets in 31 of 40 recipient sites. The outcome after 209-µm full surface, 144-µm full surface and fractional ablation was scored by the patients as good to excellent in 60%, 50% and 0% of recipient sites, respectively. The outcomes of all control sites were scored by the patients as poor. The independent physician scored the outcome of the CST as good to excellent in 50% of the full surface ablation and in 10% of the fractional ablated recipient sites. Figure 2 presents a case with excellent responses in the full surface ablation sites.

Side-effects

One week after the CST procedure, 100% re-epithelialization was reached in 30% (209 µm), 60% (144 µm), 100% (fractional) and 100% (control) of the lesions. None of the donor sites was 100% re-epithelialized after 1 week (median 5%, IQR 0–50). Pain 1 week post-treatment was scored as low to absent in all recipient sites.

Table 1 Patient characteristics

Patient	Sex	Age (years)	Diagnosis	Disease duration	Treatment location
1	Female	20	P	> 10 years	Lower leg (proximal)
2	Male	40	P	> 10 years	Knee
3	Female	42	SV	> 10 years	Upper arm (proximal)
4	Female	18	P	> 10 years	Lower leg (proximal)
5	Male	18	P	> 10 years	Lower leg (distal)
6	Male	62	P	> 10 years	Lower abdomen
7	Female	24	P	> 10 years	Knee
8	Male	19	SV	5–10 years	Upper arm (proximal)
9	Female	21	SV	> 10 years	Lower abdomen/side
10	Female	35	P	> 10 years	Lower leg (proximal)

P, piebaldism; SV, segmental vitiligo.

© 2017 The Authors. British Journal of Dermatology
published by John Wiley & Sons Ltd on behalf of British Association of Dermatologists.

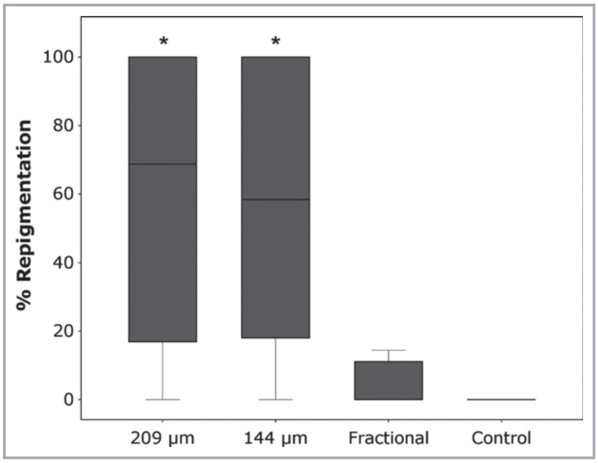

Fig 1. Box plots with percentage of repigmentation per recipient-site preparation. *$P < 0{\cdot}05$ vs. control recipient site evaluated using the Wilcoxon signed-rank test.

After 6 months, mild-to-moderate persisting erythema was more frequent in lesions pretreated with full surface ablation with a depth of 209 µm (70%) vs. 144 µm (50%). Mild hyperpigmentation, scored by the blinded physician, was seen in 10% of all full surface ablated lesions and not in any fractional or control recipient sites. Objective measurement of the hyperpigmentation with a reflectance meter showed that 10% of the full surface (209 and 144 µm) and 0% of both fractional and control sites were darker (> 10% difference in melanin index) than surrounding healthy skin. No hypopigmentation or scarring of recipient sites was observed. Mild hypertrophic scarring of the donor site occurred in two patients.

Discussion

We investigated different depths and types of ablation of the recipient site before cell suspension transplantation in segmental vitiligo and piebaldism. The results of our study suggest that more superficial CO_2 laser full surface ablation is effective, while fractional CO_2 laser ablation with the settings used in this study is not.

Previous clinical studies of noncultured CST show varying repigmentation results.[3,14] The reasons for this variation remain unclear, but recipient-site preparation is one potential determinant. The optimal depth of ablation is not known and most studies do not report the depth of ablation. Most techniques for recipient-site preparation, such as dermabrasion, microneedling and ablation with liquid nitrogen, are difficult to standardize and are not suitable for large or concave surfaces.[5,6,15] Laser settings are easy to standardize and therefore the use of ablative lasers for recipient-site preparation in CST is preferable.[6]

Repigmentation after full surface CO_2 laser ablation with a depth of approximately 209 µm was similar to that in other studies using the same depth of ablation.[6,7] In our current study, we found comparable repigmentation percentages after superficial and deeper full surface ablation (144 vs. 209 µm ablation). The superficial full surface ablation resulted in faster re-epithelialization and less persistent erythema than deeper full surface ablation. Therefore, superficial ablation seems a better option for recipient-site preparation than deeper full surface ablation. Furthermore, superficial ablation may require only topical rather than infiltration anaesthesia, which is much more comfortable for patients.

To our knowledge, this was the first study to assess superficial CO_2 laser and fractional CO_2 laser as recipient-site preparations in CST. A recent prospective study by Silpa-Archa *et al.* compared dermabrasion and full surface CO_2 laser ablation as recipient-site preparations.[6] Silpa-Archa *et al.* classified their CO_2 laser settings as fractional. However, we consider that their settings are more accurately classified as nonfractional, as the high density (82%) of the ablation channels effectively leads to a full surface ablation.[16] In our study, the recipient sites pretreated with fractional ablation did not show satisfactory repigmentation. This is contradictory to preclinical data suggesting that fractional CO_2 laser results in adequate penetration and adherence of cells.[5,17] On the other hand, fractional CO_2 lasers are known to produce a necrotic eschar that

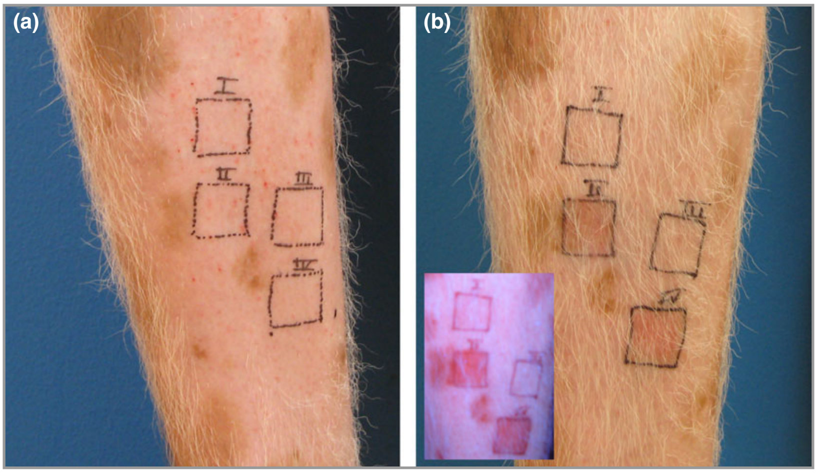

毛茸茸

Fig 2. Images of a patient with excellent responses in the full surface ablation sites. (a) before and (b) after 6 months after treatment. I, fractional laser; II, 209-µm full surface ablation; III, control site; IV, 144-µm full surface ablation. The full surface ablation sites show 100% repigmentation and persistent erythema. After Wood's lamp examination the excellent (100%) repigmentation was confirmed.

© 2017 The Authors. *British Journal of Dermatology* published by John Wiley & Sons Ltd on behalf of British Association of Dermatologists.

surrounds each ablation channel. Due to this eschar, oozing of exudate is minimized, but it may theoretically also impair permeation and adherence of the cell suspension.

As this is the first clinical study investigating ablative fractional laser therapy as a recipient-site preparation for CST, the optimal settings are still unknown. The fractional laser pulse energy settings in the current study were chosen based on expected ablation depth. The optimal depth of ablation is not known. Hypothetically, other CO_2 laser settings (e.g. pulse energy, density, depth and diameter of ablation channels) or the use of a different type of fractional laser may be more effective. Fractional erbium-doped yttrium aluminium garnet lasers usually create less necrotic eschar, and microneedling creates none, hypothetically leading to better permeation of the cell suspension.[18] However, they are also associated with more exudate and bleeding, which possibly leads to washing out the cell suspension before melanocyte adherence occurs.

Previous studies in nonsegmental vitiligo have shown repigmentation after laser ablation alone without cellular grafting.[19,20] However, in piebaldism and segmental vitiligo residual follicular melanocytes are generally lacking. This is the reason why unspecific induction of repigmentation is very unlikely in segmental vitiligo and piebaldism. Furthermore, we previously reported lack of repigmentation after CO_2 laser alone in a similar population.[7] Therefore, we assume that the contribution of laser ablation on the repigmentation is minimal in our study.

A limitation of this study is the small number of patients who were treated. Furthermore, treatment with CST was limited to the trunk and extremities, while other body parts may react differently. The study is also limited by including both piebaldism and segmental vitiligo, which are different depigmenting skin disorders. On the other hand, both have the same natural stable course without a persisting autoimmunity against melanocytes, such as in nonsegmental vitiligo. Various steps in the cell suspension transplantation process, such as harvesting of the skin sample, disaggregation of the epidermal cells and application of the cell suspension, are performed manually. This might impose another source of variation, possibly explaining the differences in repigmentation between individual patients. Another limitation is that some core outcome domains for vitiligo, such as maintenance of repigmentation and tolerability with treatment, were not measured.[21] No consensus is available on which measurement instruments should be used for target lesion assessment in vitiligo. We used the transparent sheet method that was used in our previous study and that was previously validated by Van Geel *et al.*[7,22]

In conclusion, we recommend that the recipient site before CST is prepared with full surface ablation with a depth of approximately 144 μm. Fractional CO_2 laser with the settings used in this study is not as effective for recipient-site preparation. Further research on different laser types and optimal settings is needed.

Acknowledgments

We would like to thank the patients for participating in the study.

References

1 Ongenae K, Beelaert L, van Geel N *et al.* Psychosocial effects of vitiligo. *J Eur Acad Dermatol Venereol* 2006; **20**:1–8.
2 Linthorst Homan MW, Spuls PI, de Korte J *et al.* The burden of vitiligo: patient characteristics associated with quality of life. *J Am Acad Dermatol* 2009; **61**:411–20.
3 Mulekar SV, Isedeh P. Surgical interventions for vitiligo: an evidence-based review. *Br J Dermatol* 2013; **169**(Suppl. 3):57–66.
4 van Geel N, Wallaeys E, Goh BK *et al.* Long-term results of noncultured epidermal cellular grafting in vitiligo, halo naevi, piebaldism and naevus depigmentosus. *Br J Dermatol* 2010; **163**:1186–93.
5 Al-Hadidi N, Griffith JL, Al-Jamal MS *et al.* Role of recipient-site preparation techniques and post-operative wound dressing in the surgical management of vitiligo. *J Cutan Aesthet Surg* 2015; **8**:79–87.
6 Silpa-Archa N, Griffith JL, Williams MS *et al.* Prospective comparison of recipient-site preparation with fractional carbon dioxide laser vs. dermabrasion and recipient-site dressing composition in melanocyte-keratinocyte transplantation procedure in vitiligo: a preliminary study. *Br J Dermatol* 2016; **174**:895–7.
7 Komen L, Vrijman C, Tjin EP *et al.* Autologous cell suspension transplantation using a cell extraction device in segmental vitiligo and piebaldism patients: a randomized controlled pilot study. *J Am Acad Dermatol* 2015; **73**:170–2.
8 Manstein D, Herron GS, Sink RK *et al.* Fractional photothermolysis: a new concept for cutaneous remodeling using microscopic patterns of thermal injury. *Lasers Surg Med* 2004; **34**:426–38.
9 Hunzeker CM, Weiss ET, Geronemus RG. Fractionated CO_2 laser resurfacing: our experience with more than 2000 treatments. *Aesthet Surg J* 2009; **29**:317–22.
10 Brightman LA, Brauer JA, Anolik R *et al.* Ablative and fractional ablative lasers. *Dermatol Clin* 2009; **27**:479–89.
11 Mulekar SV, Ghwish B, Al Issa A *et al.* Treatment of vitiligo lesions by ReCell vs. conventional melanocyte-keratinocyte transplantation: a pilot study. *Br J Dermatol* 2008; **158**:45–9.
12 Cervelli V, De Angelis B, Balzani A *et al.* Treatment of stable vitiligo by ReCell system. *Acta Dermatovenerol Croat* 2009; **17**:273–8.
13 Cervelli V, Spallone D, Lucarini L *et al.* Treatment of stable vitiligo hands by ReCell system: a preliminary report. *Eur Rev Med Pharmacol Sci* 2010; **14**:691–4.
14 Whitton ME, Pinart M, Batchelor J *et al.* Interventions for vitiligo. *Cochrane Database Syst Rev* 2015; **2**:CD003263.
15 Busch KH, Bender R, Walezko N *et al.* Combination of medical needling and non-cultured autologous skin cell transplantation (ReNovaCell) for repigmentation of hypopigmented burn scars. *Burns* 2016; **42**:1556–66.
16 Farkas JP, Richardson JA, Brown SA *et al.* TUNEL assay to characterize acute histopathological injury following treatment with the active and deep FX fractional short-pulse CO_2 devices. *Aesthet Surg J* 2010; **30**:603–13.
17 Oni G, Lequeux C, Cho MJ *et al.* Transdermal delivery of adipocyte-derived stem cells using a fractional ablative laser. *Aesthet Surg J* 2013; **33**:109–16.
18 Paasch U, Haedersdal M. Laser systems for ablative fractional resurfacing. *Expert Rev Med Devices* 2011; **8**:67–83.
19 Shin J, Lee JS, Hann SK *et al.* Combination treatment by 10 600 nm ablative fractional carbon dioxide laser and narrowband

© 2017 The Authors. *British Journal of Dermatology* published by John Wiley & Sons Ltd on behalf of British Association of Dermatologists.

ultraviolet B in refractory nonsegmental vitiligo: a prospective, randomized half-body comparative study. *Br J Dermatol* 2012; **166**:658–61.

20 Bayoumi W, Fontas E, Sillard L *et al*. Effect of a preceding laser dermabrasion on the outcome of combined therapy with narrowband ultraviolet B and potent topical steroids for treating nonsegmental vitiligo in resistant localizations. *Br J Dermatol* 2012; **166**:208–11.

21 Eleftheriadou V, Thomas K, van Geel N *et al*. Developing core outcome set for vitiligo clinical trials: international e-Delphi consensus. *Pigment Cell Melanoma Res* 2015; **28**:363–9.

22 Van Geel N, Vander Haeghen Y, Ongenae K *et al*. A new digital image analysis system useful for surface assessment of vitiligo lesions in transplantation studies. *Eur J Dermatol* 2004; **14**:150–5.

© 2017 The Authors. *British Journal of Dermatology* published by John Wiley & Sons Ltd on behalf of British Association of Dermatologists.
