# Supplementary material for: Therapeutic efficacy of cell-based therapy in vitiligo: a research letter systematically reviewed using meta-analysis
Source: Arch Dermatol Res. 2024 May 22;316(5):198. doi: 10.1007/s00403-024-02920-6 (PMC11111487; doi:10.1007/s00403-024-02920-6)
Supplement: Supplementary file 1 — Supplementary file1 (ZIP 24195 KB) [file 403_2024_2920_MOESM1_ESM.zip › Studies were included/Mitra 2017.pdf]

## Vitiligo International Symposium 2016

### Genetic studies of autoimmune vitiligo identify 23 new risk loci, highlight key pathways, and identify regulatory and structural variants that underlie vitiligo risk

R. Spritz

Human Medical Genetics and Genomics Program, University of Colorado School of Medicine, Denver, CO United States

Vitiligo is an autoimmune disease in which depigmented skin results from destruction of melanocytes, with epidemiologic association with other autoimmune diseases. In previous linkage and genome-wide association studies (GWAS1, GWAS2), we identified 27 vitiligo susceptibility loci in patients of European (EUR) ancestry. We now report a third GWAS (GWAS3) in EUR subjects, with augmented GWAS1 and GWAS2 controls, genome-wide imputation, and meta-analysis of all three GWAS, followed by an independent replication. The combined analyses, with 4680 cases and 39 586 controls, identified 23 new vitiligo loci and 7 suggestive loci. Most of the corresponding vitiligo susceptibility genes encode immune and apoptotic regulators, several of which are also associated with other autoimmune diseases, as well as several melanocyte regulators. Bioinformatic and fine-mapping analyses indicate a predominance of causal regulatory variation, some corresponding to expression quantitative trait loci (eQTL) at these loci, which together highlight the principal genetic architecture that underlies vitiligo risk. The identified vitiligo susceptibility genes clarify relationships to other autoimmune diseases and melanoma, and provide a pathobiological framework for pathways melanocyte damage, autoimmune triggering and propagation, and melanocyte destruction, and offer potential new targets for vitiligo treatment.

### HSP70 enhances the production of interferon-alpha by plasmacytoid dendritic cells: relevance for vitiligo pathogenesis

C. Jacquemin[1], R. Jérôme[2], G. Stéphanie[3], E. Khaled[4], T. Alain[5], B. Katia[1], S. Julien[1]

[1]Immuno-Dermatology, INSERM U1035, Bordeaux, France; [2]Immuno-Dermatology, Hôpital Saint-André, Bordeaux, France; [3]Department of Dermatology and Pediatric Dermatology, INSERM U1035, Bordeaux, France; [4]Department of Dermatology, Hôpital Henri-Mondor, Créteil, France; [5]Department of Dermatology, INSERM U1035, Bordeaux, France

Human plasmacytoid dendritic cells (pDCs) are able to produce large amount of interferon alpha (IFN$\alpha$) under appropriate activation and have been involved in a wide range of cutaneous disorders, including vitiligo and cutaneous lupus erythematosus (CLE). Heat-shock proteins (HSP) are molecular chaperones essential for maintaining cellular functions and can be released extracellularly upon cellular injury or necrosis, acting as a danger signal as shown in vitiligo. Therefore, we sought to characterize the interplay between HSP70, pDCs and IFN$\alpha$ production in vitiligo. We confirmed a significant increased production of HSP70 in the epidermis of progressive vitiligo. Importantly, pDCs were primarily localized in the epidermis, in close proximity to keratinocytes expressing HSP70. Additional in vitro experiments revealed that pDCs were able to aggregate HSP70. pDCs cultured with exogenous HSP70 underwent activation by expression of the costimulatory molecules CD80, CD86 and increased the uptake of exogenous DNA by pDCs. Interestingly, HSP70 strongly potentiated the production of IFN$\alpha$ induced by DNA in pDCs. All together these data demonstrate that the interaction between HSP70 and pDCs in vitiligo is a prerequisite for the enhancement of IFN$\alpha$ production, and could be an interesting target for future therapy in this disease that lacks specific treatment.

### A role for the unfolded protein response in the pathogenesis of vitiligo

P. Manga, S. J. Orlow, O. A. Arowojolu

The Ronald O. Perelman Department of Dermatology, New York University School of Medicine, NY United States

The mechanisms that initiate vitiligo are poorly understood. Vitiligo triggers, such as monobenzone (MB) exposure, induce stress. Understanding the survival responses that combat this stress is key to determining why melanocytes become immune targets. MB induces oxidative and endoplasmic reticulum (ER) stress, which activates the unfolded protein response (UPR). PERK, a UPR initiator, phosphorylates eIF2$\alpha$ and master antioxidant regulator, NRF2. Here, we investigated the impact of PERK-eIF2$\alpha$/-NRF2 activation on sensitivity to MB. Basal phospho-eIF2$\alpha$ and NRF2 levels are higher in melanocytes compared to fibroblasts or keratinocytes. PERK down-regulation significantly reduced melanocyte viability (implicated in several autoimmune disorders) may link exposure to vitiligo-inducing triggers with onset of autoimmunity.

### A CXCR3 depleting antibody removes autoreactive T cells and reverses depigmentation in a mouse model of vitiligo

J. Harris[1], J. Richmond[2], E. Masterjohn[3], R. Chu[4], J. Tedstone[4], M. Youd[4]

[1]Dermatology, UMass Medical School, Worcester, MA United States; [2]Dermatology, Genzyme/Sanofi, MA United States; [3]UMass Medical School, Worcester, MA United States; [4]Genzyme/Sanofi, MA United States

Vitiligo results from autoimmune destruction of melanocytes and is characterized by disfiguring patchy depigmentation of the skin, however there are no FDA-approved treatments. We previously found that the progression and maintenance of vitiligo depended on CD8+ T cell recruitment to the skin through the chemokine receptor CXCR3. Therefore, we tested a variety of blocking and depleting antibodies against CXCR3 as a new treatment strategy in our mouse model of vitiligo. We found that a CXCR3-depleting antibody reduced autoreactive T cell numbers and reversed disease while having minimal impact on other compartments of the immune system, and the repigmentation induced by this treatment outperformed others we have tested. A depleting antibody may have greater clinical efficacy and durability than chemokine neutralizing antibodies by removing autoreactive cells rather than blocking their migration. However this approach may be more aggressive, and safety must be carefully considered. Future studies in patients will help to determine the applicability of this treatment strategy for vitiligo.

© 2017 John Wiley & Sons A/S. Published by John Wiley & Sons Ltd 

## Non-lesional vitiligo skin: dermal influences

D. Kovacs, E. Bastonini, M. Ottaviani, E. Migliano, C. Cota, M. L. Dell'Anna, M. Picardo

San Gallicano Dermatologic Institute, Rome, Italy

There is an increasing interest in normal appearing skin in vitiligo. An altered expression of the adhesion molecule E-cadherin has been recently highlighted in melanocytes of pigmented areas as well as an unbalanced intracellular redox status that, when persistent, may promote the acquisition of a stress-induced pre-senescent phenotype. Due to the important contribution of the dermal components in regulating melanocyte biology, in this study we evaluated the features of non lesional vitiligo fibroblasts to investigate differences respect to normal cells and to highlight possible altered biological activities which may favour melanocyte defects. We employed non lesional cells matched with normal fibroblasts for age, gender and anatomic site of biopsy to analyze: (i) the expression of a panel of growth factors and proteins specifically involved in controlling melanocyte homeostasis and pigmentation; (ii) the effects of the treatment with conditioned medium obtained from normal and non lesional vitiligo fibroblasts on melanocyte functions. The results demonstrate a deregulation in the expression and release of several messengers involved in influencing melanocyte adhesion, growth and pigmentation, indicating the presence of an altered cross-talk among dermal and epidermal compartments, which may contribute to affect melanocytes, even in normally pigmented skin areas.

## The mitochondrial metabolic aspects in vitiligo

M. L. Dell'Anna[1], D. Kovacs[1], S. Mirabile[2], M. R. Ricciardi[2], M. Ottaviani[1], E. Bastonini[1], B. Bellei[1], C. Cota[3], A. Tafuri[2], M. Picardo[1]

[1]Cutaneous Physiopathology, San Gallicano Dermatologic Institute, Rome, Italy; [2]Department of Cell Biotechnology and Hematology, Sapienza University, Rome, Italy; [3]Dermopathology Unit, San Gallicano Dermatologic Institute, Rome, Italy

Vitiligo is characterized by loss/functional defects of epidermal melanocytes, mitochondria-driven pre-senescent phenotype and low capability to cope with stress. We look for the functional value of the mitochondrial energetic metabolism.

Vitiligo melanocytes (VHM) showed increased mitochondrial mass (+40%), expression of the glycolitic enzymes HKII (MFI 669 versus 614), PDHK1 (MFI 514 versus 481), PKM2 (MFI 828 versus 790), and lower ATP production. L-glutamine intensified the ATP production and reduced the HKII and PKM2 expression in normal melanocytes but not in VHM, indicating their inability to gain from alternative supply. SeahorseXF assay showed significant proton leak in VHM indicating inability to finalize the ETC activity.

VHM showed higher uptake of glucose, suggesting that they, beside the glycolitic boost, are unable to properly maximize the ATP production.

CREB, as well as FAKy397 (MFI $650 \pm 32$ versus $600 \pm 18$, $P < 0.05$) and FAKs910 (MFI $428 \pm 25$ versus $355 \pm 17$, $P < 0.05$), is higher phosphorylated and PGC1a expression, sustained by PPARg, is higher (MFI $650 \pm 23$ versus $540 \pm 18$; $P < 0.05$) in VHM.

In conclusion, the inappropriate mitochondrial function (inadequate ATP level) could activate the nucleus/mitochondria cross-talk to compensate and revert this metabolic impairment. However, the vitiligo inability to cope further energetic demand provide the metabolic basis for the pre-senescent progress.

## MIA (melanoma inhibitory activity) is able to induce vitiligo in an in-vivo mice model by direct injection in the tail

M. Bordignon[1], R. Luisetto[2], M. Valente[3], M. Fedrigo[3], C. Castellani[3], A. Angelini[3]

[1]Research and Development Unit, MB Skin Research, Padua, Italy; [2]Surgical, Oncological and Gastroenterological Sciences Department, University of Padua, Italy; [3]Pathology Unit, University of Padua, Italy

We previously demonstrated that a protein called MIA (Melanoma Inhibitory Activity) is present in human non-segmental vitiligo skin and seemed to cause the detachment of melanocytes, leading to the formation of achromic patches. In this study we investigated the possibility to create an animal model of vitiligo, based on the sole action of the MIA protein, verifying its direct pathogenetic role. A group of pigmented mice was injected into the tail with saline (control group) or with saline + MIA protein. The injections were repeated every 2 weeks for 3 months. Histology and immunohistochemistry were performed in all tails. All the mice did not show any side effect. The control group did not show any sign of depigmentation. The treated group showed, instead, clear zones of complete depigmentation in the injected area. Histological features showed in all groups the absence of any inflammatory cell infiltrate. MIA protein causes a complete vitiligo-like depigmentation with its direct injection in the tail of mice by a totally non-immunological mechanism. These data confirm the key role played by this protein in the vitiligo disease and open the doors to the development of drugs able to inhibit the action of MIA for curing this disease.

## Rhododendrol-induced leukoderma analyzed with a model mouse

T. Suzuki[1], Y. Abe[1], K. Okamura[1], Y. Hozumi[1], T. Kunisada[2], S. Ito[3], K. Wakamatsu[3]

[1]Dermatology, Faculty of Medicine, Yamagata University, Japan; [2]Tissue and Organ Development, Graduate School of Medicine, Gifu University, Japan; [3]Chemistry, Fujita Health University, School of Health Sciences, Toyoake, Japan

Rhododendrol (Rhododenol: RD) is a naturally occurring phenolic compound, was developed as a tyrosinase inhibitor for skin-lightening/whitening cosmetics. In 2013, skin depigmentation, which is a kind of chemical vitiligo, was reported in consumers using RD-containing cosmetics. In order to assess the depigmentation potential of RD, we developed a new mouse model of leukoderma by topically applying RD. Hairless hk14-SCF Tg mice with melanocytes distributed in the epidermis were used for this study. Immunohistological, biochemical, and electron microscopic analyses were performed on biopsy samples taken from these mice. And also, we observed time course of the leukoderma after stopping the cosmetics, and tried to find effective treatments for this condition. Biochemical analyses showed that the eumelanin content decreased in the RD-treated sites and metabolites of RD-quinone, i.e., non-protein thiol adducts and protein-SH adducts, were produced. Electron microscopic analyses revealed double-membrane-walled structures containing electron-dense material, which might be typical for melanin-containing autophagosomes and a dilated endoplasmic reticulum (ER), which would indicate ER stress. Finally, we observed re-pigmentation of the depigmented area, which were withdrawn from RD after 42 days. Furthermore, we found UV radiation plus topically applying VitD3 could increase re-pigmentation.

 © 2017 John Wiley & Sons A/S. Published by John Wiley & Sons Ltd

### Melanocyte NADPH oxidase activation induced by keratinocyte-derived ROS

V. Barygina[1], A. Mannucci[1], R. Colucci[2], N. Taddei[1], T. Lotti[3], S. Moretti[1], C. Fiorillo[1]

[1]Department of Experimental and Clinical Biomedical Sciences 'Mario Serio', University of Florence, Italy; [2]Section of Dermatology, Department of Surgery and Translational Medicine, University of Florence, Italy; [3]Chair of Dermatology, University of Rome 'G. Marconi', Italy

Perilesional keratinocytes obtained from the skin of vitiligo patients are characterized by a prominent redox imbalance. Recent reports indicate that Reactive Oxygen Species (ROS) play a major role in the regulation of human pigmentation by affecting melanin synthesis and melanosome transfer (1). In the present study we used a melanocyte-keratinocyte co-culture protocol that allows testing the effect of human HACAT keratinocyte-derived ROS on human HEMa-LP melanocytes. After oxidative stimulation by 2,2′-Azobis(2-amidinopropane) dihydrochloride (AAPH), HACAT cells were co-cultured with HEMa-LP for 48 h. Then, ROS production and NADPH oxidase activity in HEMa-LP cells were monitored by confocal microscopy and luminometric assay, respectively. Co-culture with AAPH-treated HACAT cells significantly increased melanocyte ROS production (by 30 $\pm$ 2% versus co-culture with untreated HACAT cells) and melanocyte NADPH oxidase activity (by 19 $\pm$ 3% versus co-culture with untreated HACAT cells). Our data indicate that ROS over-production by keratinocytes enhances NADPH oxidase activity and induces consequent redox imbalance in melanocytes in co-culture.

Reference1. Tang L1, Li J, Lin X, Wu W, Kang K, Fu W. Oxidation levels differentially impact melanocytes: low versus high concentration of hydrogen peroxide promotes melanin synthesis and melanosome transfer. Dermatology. 2012;224 (2):145–53.

### Autologous melanocyte-keratinocyte suspension in NSV; is supplementation of the suspension medium beneficial

D. Bassiouny, B. ElZawahry, S. Esmat, M. Saleh, D. Abdelhalim, R. Hegazy, H. Gawdat

Dermatology, Cairo University, Egypt

Autologous melanocyte-keratinocyte suspension transplantation is a relatively simple technique with good results. Supplementation of the suspension medium by several compounds was reported by some authors.

Comparison of response to autologous melanocyte-keratinocyte suspension transplantation in cases of non-segmental vitiligo (NSV) with and without supplementation of the suspension medium.

18 cases with NSV were randomly divided into 2 groups. Group A: The cellular pellet was suspended in Ham F12 medium and group B: The cellular pellet was suspended in Ham F12 supplemented with basic fibroblast growth factor and other compounds which induce melanocyte differentiation and melanogenesis. All cases received nbUVB therapy twice weekly 1 month after the procedure. Percentage of repigmentation was assessed after 3 and 6 months. Any complications were recorded.

In Group A: 90–100% repigmentation occurred in 5/9 cases; 75% in one case; 50% in one case and 10% repigmentation in 2 cases. In group B: 90–100% repigmentation occurred in 7/9 cases while 10% repigmentation occurred in the remaining 2 cases (P value: 0.77). Marginal halo was seen in 5 cases in group A and 6 in group B.

Supplementation of suspension medium does not seem to improve the outcome in cases of NSV.

### Effect of fractional carbon dioxide laser on repigmentation of acral vitiligo after non cultured epidermal cell suspension (NCES) grafting; an immunohistochemical study

S. Esmat[1], M. Assaf[2], D. Kadry[3], A. Marwa[4], G. El Hanafy[4], H. Saad[5], M. El Hawary[4]

[1]Dermatology, Faculty of Medicine, Cairo University; [2]Pathology, Cairo University, Giza, Egypt; [3]Dermatology, Zagazig University, Egypt; [4]Dermatology, Cairo University, Giza, Egypt; [5]Dermatology, National Research Centre, Cairo, Egypt

Defective matrix metalloproteinases (MMPs) and cadherins in vitiligo can affect melanocyte motility and adhesiveness respectively. $CO_2$ laser resurfacing increases expression of both molecules and thus might enhance better response to melanocyte grafting in acral vitiligo.

Evaluate effect of fractional $CO_2$ laser on MMP2, E-cadherin and repigmentation in acral vitiligo after (NCES) grafting followed by NBUVB.

This randomized prospective comparative study included twenty stable, acral vitiligo patients. In each, two symmetrical lesions were treated by NCES grafting followed by NBUVB. One side received in addition, fractional $CO_2$ laser resurfacing biweekly up to six months. Biopsy was taken from the edge of both lesions before grafting and after repigmentation and stained immunohistochemically for E-cadherin and MMP2.

Repigmentation appeared in eight cases (40%). Increased expression of E-cadherin as well as both epidermal and dermal MMP2 arose after treatment and was associated with repigmentation. No significant differences were detected in percent or onset of repigmentation or the immnunohistochemical changes between both sides.

NCES grafting followed by NBUVB increases expression of E-cadherin and MMP2 which probably helps the motility and adhesiveness of cultured melanocytes. Fractional $CO_2$ laser couldn't enhance those molecular changes or improve repigmentation of acral vitiligo.

### Insight into the molecular pathways of a PGE2 analogue in regulating skin pigmentation

E. Flori[1], E. Bastonini[1], I. Coletta[2], A. Alisi[2], L. Polenzani[2], M. Picardo[1]

[1]San Gallicano Dermatologic Institute, Rome, Italy; [2]Angelini Research Center, S. Palomba-Pomezia, Rome, Italy

Prostaglandins (PG) are lipid inflammatory mediators produced by keratinocytes and melanocytes in response to UV and play a role in skin pigmentation acting on different receptors. Topical PGE2 was used with promising results in the treatment of localized stable vitiligo. The aim of this study was to deepen the knowledge of the effects of PGE2 in melanocyte. The treatment with the PGE2 analogue Alprostadil stimulated melanocyte proliferation, increased the mRNA levels of the master regulator of pigmentation MITF, improved tyrosinase protein expression and activity and secretion of melanins, synergizing the effect of $\alpha$-MSH. Moreover, the molecule promoted an increased induction of pro-melanogenic paracrine factors in keratinocytes in response to UV and increased the mRNA of the peroxisome proliferator-activated receptors (PPARs) alpha

© 2017 John Wiley & Sons A/S. Published by John Wiley & Sons Ltd 

and gamma. The inhibitor of the microsomal prostaglandin E synthase-1 AF3485, interfering with PGE synthesis, decreased tyrosinase protein expression and activity and secretion of melanins, independently of a MITF-mediated transcriptional control. Moreover, it counteracted the pigmentation effects induced by α-MSH and the release of pro-melanogenic paracrine factors in keratinocytes in response to UV, confirming the pro-melanogenic role of PGE2. The results offer a new perspective for increasing pigmentation in hypopigmented and/or depigmented disorders, e.g. vitiligo.

## Assessment of aquaporin 3 expression in different anatomical sites in vitiligo: an immunohistochemical study

D. Hafez[1], S. Esmat[1], O. Abuzeid[1], M. Assaf[2], M. Tolba[1], M. AbdEllatif[1], V. Mladenova[1]

[1]Dermatology, Cairo University, Giza, Egypt; [2]Pathology, Zagazig University, Egypt

Aquaporin 3 (AQP3) is a glycerol water channel that is expressed in epithelial tissues. AQP3 is involved in the regulation of keratinocyte proliferation, cell migration, and tumorigenesis.
ObjectiveTo assess keratinocytes AQP3 expression in vitiliginous skin in different anatomical sites to detect the possible role of AQP3 in the pathogenesis of vitiligo.
The study included 15 patients with both acral and non-acral vitiligo lesions, 15 subjects as control. Six punch biopsies (2 mm) were taken from acral, and non-acral skin. Two (2 mm) biopsies were taken from each control subject. AQP3 tissue expression was analyzed immune-histochemically.
In basal layer, AQP3 expression was significantly lower in lesional non-acral and perilesional (acral and non-acral) biopsies when compared to controls ($P = 0.021$), ($P = 0.017$) and ($P = 0.011$) respectively. In stratum spinosum, a statistically significant less AQP3 expression was observed in perilesional biopsies compared to lesional biopsies ($P = 0.050$). Marked gradual fading of AQP3 expression was noticed in vitiliginous skin, opposite to the uniform staining in controls ($P = 0.050$).
Decreased AQP3 expression might play a role in the pathogenesis of vitiligo and in resistance of acral lesions to treatment.

## Mast cell activation promotes possible transient hypermelanosis of the perilesional skin in rhododendonol induced-leukoderma

I. Katayama, A. Takahashi, F. Yang, L. Yang, N. Arase, A. Tanemura, M. Kaneda

Dermatology, Osaka University, Suita, Japan

It has been reported that IL-17(+) mast cells are found at higher densities than IL-17(+) T cells in psoriasis which prompted us to analyze IL17 (+) infiltrating cells in the lesional skin of vitiligo (n = 5), rododendonol induced-leukoderma (n = 5) and controls (n = 5), because we preported that Th17 cell-related cytokines down regulated melanogenesis in vitiligo. Following antibodies are used in this experiment. Anti-IL17 (AF-317-NA; specificità: IL17A,B,C,D, E,F) anti-IL17A(AHP455G; specificity:IL17A). We observed IL17A (+) T cells infiltrated to the lesional skin of vitiligo (20/HPF) and lesser extent in rhododendrol induced-leukoderma (10/HPF) and normal (2/HPF) as reported. Surprisingly, prominent degranulated mast cells were observed both in these skin lesions (vitiligo. 30%, rhododendrol 48%, normal 12%). These mast cells did not show extracellular trap forming pattern of positive IL17A staining. Electron microscopic analysis demonstrated that piecemeal pattern, but not anaphylactoid degranulation was prominent. These results might suggest that mast cells infiltrate to the vitiliginous skin where increased keratinocyte expression of c-kit ligand (SCF) was reported previously. In addition, mast cell derived histamine or other substances might contribute hypermelanosis of surrounding skin of vitiliginous skin as reported. These novel observation might provide new therapeutic strategy for treatment of hypermelanotic skin lesions frequently observed in rhododendrol induced leukoderma.

## Fibroblast derived clusterin inhibits melanogenesis via TGF-β signaling

J. Lee[1], M. Kim[1], H. Y. Kang[1], T. J. Park[2]

[1]Dermatology, Ajou University, School of Medicine, Suwon, South Korea; [2]Department of Biochemistry, Ajou University, School of Medicine, Suwon, South Korea

Clusterin has been known as a biomarker of aging and is highly induced in stressed and senescent cells. In this study, RNA sequencing analysis showed transcripts for clusterin were highly expressed in UV irradiated fibroblasts. A considerable increase of clusterin was detected in the culture medium. We found that the clusterin associates with solar elastosis of melasma and solar lentigo and is overexpressed in the acutely UV-irradiated skin. To investigate the effect of clusterin on melanogenesis, normal human melanocytes were treated with conditioned media of fibroblasts infected with clusterin-lentivirus or sh-clusterin. It was found that clusterin inhibits melanogenesis through MITF/ tyrosinase downregulation via TGF-β signaling. The findings suggest that clusterin inhibits melanogenesis and that it plays a role in controlling UV-induced pigmentary changes.

## Light-independent pro-inflammatory and pro-oxidant effects of purified human hair melanins on keratinocyte cell cultures

S. Lembo[1], A. Napolitano[2], R. Di Caprio[3], L. Panzella[2], R. Micillo[3], A. Balato[3], G. Monfrecola[3]

[1]Department of Clinical Medicine, Surgery and Dentistry 'Scuola Medica Salernitana', University of Salerno, Italy; [2]Department of Organic Chemistry and Biochemistry, University of Naples 'Federico II', Italy; [3]Dermatology Unit, Department of Clinical Medicine and Surgery, University of Naples 'Federico II', Italy

The purpose of this study was to test the ability of red human hair pheomelanin (RHP) to induce oxidative damage and/or direct inflammatory response on cultured keratinocytes, independently from light exposure. HaCaT cells were incubated, in the dark, for 24 h with RHP or black human hair eumelanin (BHE), previously purified from red and black hair. Proteins from white human hair (WHP) were used as negative control. Toxicity of RHP, BHE and WHP was assessed through cell viability and proliferation/ apoptosis assessment. Thereafter the possible pro-inflammatory effect was explored analyzing IL-1β, IL-6 and TNF-α. Moreover, determination of cellular antioxidants (GSH and NADPH) and lipid peroxidation markers (TBARS) was performed. Exposure to RHP or BHE moderately decreased keratinocyte viability. RHP, and to a minor extent BHE, promoted expression of pro-inflammatory interleukins and oxidative damage in the dark. Gene expression increase was dose related and highly significant for TNF-α and IL-1 β, whereas IL-6 protein secretion resulted more enhanced than TNF-α or IL-1 β. Cellular depletion of GSH and NADPH was registered, as well as TBARS increase, after exposure to RHP and BHE. Our results indicate that RHP works as a direct pro-inflammatory and pro-oxidant agent in keratinocyte cultures, independently from light exposure.

 © 2017 John Wiley & Sons A/S. Published by John Wiley & Sons Ltd

## Role of the decreased release of keratinocyte growth factor in the pathogenesis of vitiligo hypo-pigmentation

F. Persechino[1], M. Nanni[2], F. Belleudi[2], A. Tammaro[3], S. Persechino[3], M. R. Torrisi[2]

[1]Dermatology Unit, University of Modena and Reggio Emilia, Italy; [2]Clinical and Molecular Medicine, 'Sapienza' University of Rome, Italy; [3]NESMOS, Dermatology Unit, 'Sapienza' University of Rome, Italy

The molecular mechanisms involved in cutaneous pigmentation as well as the role played by the paracrine crosstalk between the epidermal keratinocytes and dermal fibroblasts are just starting to be elucidated. We have previously demonstrated that the keratinocyte growth factor (KGF/FGF7), released from the fibroblasts, triggers melanosome uptake in human keratinocytes through promotion of the phagocytic process induced by activation of the keratinocyte growth factor receptor (KGFR/FGFR2b) and its downstream phospholipase C$\gamma$ (PLC$\gamma$) signaling. Even if the loss of melanocytes is considered the main factor leading to skin color impairment in hypopigmentary disorders, our recent results suggested that a deficient melanosome transfer, consequent to reduced expression and secretion of KGF, could be an additional pathogenic mechanism involved in vitiligo. Taking advantage of the in vitro model of fluorescent latex bead uptake, widely used to study the phagocytosis in epidermal keratinocytes, here we investigated the ability of supernatants (SNs) collected from primary cultures of dermal fibroblasts, isolated by vitiligo skin samples or from healthy skin, in promoting the phagocytic process. Our observations indicate that the reduced release of KGF from lesional fibroblasts and the decreased receptor activation on the keratinocytes are crucial in determining impairment of the phagocytosis required for proper pigmentation.

## A shorter life span and its extension by PAPLAL, nanoparticles of platinum and palladium, of cultured melanocytes obtained from uninvolved skin of a vitiligo patient

H. Ando[1], S. Yoshimoto[1], M. Yoshida[1], T. Shibata[2], M. Ichihashi[3]

[1]Department of Applied Chemistry and Biotechnology, Okayama University of Science, Japan; [2]Shibata Hifu-ka Clinic, Niigata, Japan; [3]SAISEI MIRAI Clinic, Kobe, Japan

Several studies suggest that loss of cellular redox state may play a pivotal role in the pathogenic mechanism in vitiligo. PAPLAL, platinum (Pt) and palladium (Pd) nanoparticles (Toyokosei Seiyakusyo Co., Tokyo, Japan) are reported to have a strong catalase-like activity and may be effective in the treatment of vitiligo. We studied whether PAPLAL can extend life span of cultured melanocytes obtained from epidermis of uninvolved skin taken by suction blister technique of a Japanese woman who developed non-segmental vitiligo. Melanocytes cultured in vitro showed an extremely short-lived life span compared to commercially available normal human melanocytes, characterized with gradual appearance of tiny ladder-like cytoplasmic compartments (LLCC) during a few months culture. The addition of PAPLAL solution to the culture medium prevented the appearance of LLCC in the vitiligo-derived melanocytes and prolonged the culture life span significantly. Further, the addition of PAPLAL showed attenuation of hydrogen peroxide-induced cytotoxicity for normal human melanocytes. These results reinforced the notion that the melanocytes obtained from the uninvolved site of vitiligo patient are potentially fragile against reactive oxygen species that can be rescued by antioxidants and radical scavengers, and other agents which increase the expression of antioxidant phase II system.

## Clinical significance of serum soluble CD molecules to assess disease activity in vitiligo

R. Speeckaert, N. van Geel

Dermatology, Ghent University Hospital, Belgium

It is difficult to determine disease activity in vitiligo due to the absence of inflammatory signs such as erythema or scaling. A biomarker which could confirm active disease and predict future disease progression would therefore be of considerable value.
To investigate if soluble CD27 (sCD27), CD25 (sCD25) or CD40L (sCD40L) could be valuable biomarkers to determine disease activity in vitiligo and predict future progression. Participants: 93 vitiligo patients were enrolled, including 83 patients with non-segmental vitiligo and 10 patients with segmental vitiligo.
Both sCD27 ($P = 0.006$) and sCD25 ($P = 0.002$) were strongly associated with active disease. Moreover, a link with disease progression after 3–6 months was found for sCD27 ($P = 0.021$) and to a lesser extent also for sCD25 ($P = 0.053$). Further in vitro experiments showed a correlation between sCD25 and IFN-$\gamma$, IL-10 and sCD27 secretion. No strong associations were found for sCD40L.
This study demonstrates increased values of sCD27 and sCD25 in active vitiligo patients. Moreover, our results provide for the first time evidence that these markers have a predictive capacity on future disease progression. This supports their role as attractive biomarkers in vitiligo.

## Serum CXCL 10 as activity marker for vitiligo

M. Abdallah[1], M. El Mofty[2], T. Anbar[3], S. Esmat[4], D. Bassiouny[4], D. Abdel-Halim[4], M. Ibrahim[1]

[1]Dermatology, Venereology and Andrology Department, Ain ShaUniversity, Cairo, Egypt; [2]Dermatology Department, Minya University, Egypt; [3]Dermatology, Venereology and Andrology Department, Cairo University, Giza, Egypt; [4]Dermatology Department, Cairo University, Giza, Egypt

The need for a trusted activity marker in generalized vitiligo is important before surgical correction. We aimed to evaluate serum CXCL 10, and compare it with other serum and tissue activity markers. The study included 55 vitiligo patients (30 active and 25 stable) and 30 healthy matched controls. Serum CXCL 10 was compared to IFN-$\gamma$, IL-6 and IL-17 in addition to immunohistochemical evaluation of CD8+ cytotoxic T-cells and CXCL10. Serum levels of CXCL10, IL-17 and IL-6 were elevated in all vitiligo patients compared to controls (p-17 ($P = 0.008$). In addition, a significant +ve correlation was noted between tissue CXCL10 and serum IL-6 ($P = 0.000$) and serum CXCL10 ($P = 0.000$).
Serum CXCL-10 is a reliable activity marker.

## A pilot study of CXCL9/10 levels in vitiligo lesions undergoing treatment

J. A. Hinojosa, A. G. Pandya

Department of Dermatology, University of Texas, Southwestern Medical Center, Dallas, TX United States

HypothesisDisease activity in vitiligo is unpredictable and no serum biomarker currently exists. We hypothesized that CXCL9 and CXCL10 levels would decrease in vitiligo lesions as well as in the serum of patients undergoing treatment.
Four treatment naïve patients with active vitiligo manifesting as confetti-like depigmentation were recruited. Epidermal blisters were created in lesional and non-lesional skin at baseline and then again after 4 weeks of treatment with 4 mg

© 2017 John Wiley & Sons A/S. Published by John Wiley & Sons Ltd 

dexamethasone on two consecutive days per week, NB-UVB phototherapy and clobetasol cream once daily, 5 days per week. Serum samples from blood were also taken at each visit. Levels of CXCL9 and CXCL10 within blister fluid and serum were measured in duplicate using ELISA.
There was a decrease in levels of CXCL9 in lesional skin after treatment when compared to non-lesional skin. Serum levels of CXCL9 also decrease after treatment. Changes in CXCL 10 were variable.
CXCL9 levels in both the skin and serum of patients with active vitiligo decrease after just 4 weeks of treatment. CXCL10 levels did not show a difference with treatment. CXCL9 is a potential biomarker to measure disease activity.

## Blistering lesional skin in vitiligo patients reveals sensitive and specific biomarkers of disease activity

J. Harris[1], J. Strassner[1], M. Rashighi[1], M. A. Refat[1], J. Richmond[1]

[1]Dermatology, UMass Medical School, Worcester, MA United States

Vitiligo has limited treatment options, but based on recent studies promising new treatments should soon enter clinical trials. There is an urgent need to identify and validate biomarkers of disease activity to support potentially expensive and technically challenging clinical studies. Here we sampled the lesional and non-lesional skin of vitiligo subjects with active disease via a modified suction blister technique. Markers were first defined in a group of eight active vitiligo subjects and controls and then validated in a second group of seven active and 4 stable vitiligo subjects. We found that CD8+ T cell number and CXCL9 protein concentration were significantly elevated in active lesional compared to non-lesional skin with good sensitivity and specificity for active lesional skin. One subject showed reduction of CD8+ T cells and elimination of CXCL9 protein post-treatment, suggesting these may be early markers of treatment response. Phenotyping of the T cell infiltrate revealed CXCR3 expression on T cells, supporting our previous studies. In conclusion, these lesional biomarkers could be used in small adaptive clinical trials as an early marker of treatment response.

## Deciphering the role of skin effector and resident memory T cells in vitiligo

K. Boniface[1], N. Boukhedouni[1], C. Jacquemin[1], J. Rambert[2], A. Taïeb[3], K. Ezzedine[4], J. Seneschal[1]

[1]Immunodermatology, INSERM U1035, Université de Bordeaux, France; [2]Aquiderm, Bordeaux, France; [3]Dermatologie, INSERM U1035, Université de Bordeaux, France; [4]Dermatology, Hôpital Henri-Mondor, Créteil, France

The pathological hallmark of vitiligo is the loss of melanocytes associated with autoimmune components involving both CD4+ and CD8+ T cells. However, the phenotype and contribution of effector memory T (TEM) cell subsets remain to date unclear and controversial due to the lack of extensive analysis in human and animal models reproducing the complexity of the disease. Therefore, we sought to decipher the phenotype and function of circulating and skin TEM in human vitiligo. We observed a decreased frequency of circulating CD4 and CD8 TEM expressing CXCR3, compared to healthy controls. This was associated with elevated frequency of these cell subsets in vitiligo perilesional skin, with the presence of a significant population of CD69+ CD103+ skin resident memory T cells. Multiparametric analysis of the cytokine secretion profile of these skin TEM subsets readily identified IFNg and TNFa as inflammatory cytokines involved in vitiligo pathogenesis. These two cytokines act in synergy to inhibit expression of genes involved in melanocyte function, while upregulating the expression inflammatory associated genes and proteins. Altogether our data suggest that vitiligo could be viewed as a memory skin disease and highlight new pathogenic mechanism to explain melanocyte disappearance, identifying new therapeutic strategies for vitiligo.

## Dysregulation of circulating T cells anergy might be involved in autoimmune disorder of vitiligo

L. Yang[1], A. Tanemura[2], T. Morita[3], K. Kato[1], A. Tanaka[4], S. Sakaguchi[4], I. Katayama[1]

[1]Department of Dermatology, Osaka University, Suita, Japan; [2]Department of Dermatology, Immunology Frontier Research Center, Osaka University, Suita, Japan; [3]Experimental Immunology, Osaka University, Suita, Japan; [4]Experimental Immunology, Immunology Frontier Research Center, Osaka University, Suita, Japan

Vitiligo is the most common pigmentation disease. Whereas various pathogenetic mechanisms have been suggested, the exact mechanism is still unclear. Recently, the presence of melanocyte-specific T lymphocytes with naïve characteristics in HLA-A2 healthy donors was reported. Furthermore, high frequency of circulating Melan-A-specific, HLA-A*0201* positive cytotoxic T lymphocytes was observed in patients with vitiligo, these cells was also observed with high levels of the skin homing receptor, cutaneous lymphocyte-associated antigen. To further clarify the mechanism in vitiligo by evaluating the phenotype of peripheral melanocyte-specific T cells and the anergy status in patients with vitiligo. 13 HLA-A*0201* vitiligo patients and 10 genotype-matched healthy donors were randomly selected for this study. PBMCs were separated by centrifugation over Ficoll-Paque, and then purified CD14+ APCs, CD14−CD8+ lymphocytes and CD8−CD25+ Treg cells were cultured and expanded over 10 days under Melan-A-peptide stimulation. Finally, melanocyte-specific T cells were detected using fluorescent HLA-A*02:01 tetramers containing immunodominant Melan-A (ELAGIGILTV) antigen, and immunophenotyping and function of both the cytotoxic T cells and Treg cells were analyzed by flow cytometry. Compared to healthy donors, high frequency of melanocyte-specific T cells and disturbing phenotype of Treg cells were observed in vitiligo patients, suggesting a certain contribution of T cell anergy in autoimmune vitiligo.

## Functional rnomics of MIR-2909 in human non-segmental vitiligo skin

H. Kaushik[1], D. Parsad[1], D. Kaul[2]

[1]Dermatology, Postgraduate Institute of Medical Education and Research, Chandigarh, India; [2]Experimental Medicine and Biotechnology, Postgraduate Institute of Medical Education and Research, Chandigarh, India

This study explores a molecular link between a novel micro-RNA, miR-2909, and immunomodulatory genes responsible for the initiation of non-segmental vitiligo (NSV) using qPCR, Western Blotting and Immunohistochemistry. Expression of miR-2909 increased in NSV patients. KLF-4 downregulated, CCL-5 and pro-inflammatory cytokines upregulated. This miR-2909 RNomics involving KLF-4, CCL-5, IL-17, and IFN-$\gamma$ had the inherent capacity to tailor an autoimmune condition that could initiate NSV. Thus, we propose that miR-2909 indirectly controls the infiltration of

 © 2017 John Wiley & Sons A/S. Published by John Wiley & Sons Ltd

CD8+ T-cells subsequently destroying melanocytes by modulating KLF-4 mediated CCL-5 regulation. Moreover, since NF-ĸB (via CYLD regulated by KLF-4) induces miR-2909, we propose that miR-2909 ensures its own expression by downregulating KLF-4, thereby starting a vicious cycle of unresolved inflammation responsible for increased pro-inflammatory cytokines associated with NSV pathogenesis. Interesting to note is increased expression of IL-17 despite various factors responsible for its suppression. Probably, GAPDH, IL-6 or its source of secretion could be responsible. In nut shell, we propose a miR-2909 RNomics pathway that might be responsible in orchestrating the immunopathogenesis of NSV. This study holds significance as it is first to highlight the role of miR-2909, KLF4 and CCL-5 in the pathogenesis of NSV which might prove useful in designing better therapeutics/diagnostic strategies in future.

## The innate immune receptor CD91 and levels of erythrocyte malonyldialdehyde (MDA) as activity markers of vitiligo

J. Piquero-Casals[1], L. Martinez-Martinez[2], E. Rozas[1], M. Lozano-Rabella[2], L. Puig[1], C. Juárez[2], A. Alomar[1]

[1]Dermatology, Hospital de la Santa Creu i Sant Pau, Barcelona, Spain; [2]Inmunology, Hospital de la Santa Creu i Sant Pau, Barcelona, Spain

Vitiligo is an autoimmune disease in which melanocytes are destroyed by antigen-specific T-cell. Adaptative immunity plays an important role in progression of the disease and the initiating factors are incompletely understand. Melanocytes in vitiligo patients are more susceptible to oxidative damage than melanocytes from unaffected individuals. The gap between cellular stress and adaptive immunity in vitiligo requires a better understanding. CD91 is an innate-immune-receptor involved in receptor-mediated endocytosis. This receptor is stimulated by various stimuli including head-shock-proteins among others, inducing the secretion of proinflammatory cytokines. The former of this event has the potential to trigger the chronic inflammation in vitiligo.
Serum levels of malonyldialdehyde, as a marker of oxidative stress, and CD91 were evaluated regarding their role in the pathogenesis of vitiligo, as well as their relationship with clinical presentation and disease severity and comparing with those of healthy controls.
The study included 20 patients with vitiligo and 10 healthy volunteers as the control group.
MDA and CD91 were significantly higher in patients with vitiligo than in healthy controls.
CD91 and MDA could be used as an indicator for activity status in vitiligo. Further studies are required to confirm the mechanism underlying this effect.

## Exploring vitiligo risk factors in Kazakh patients

A. Aimoldina, G. Batpenova, T. Algazina

Department of Dermatology and Venereology, Astana Medical University, Kazakhstan

Topicality. According to modern scientific trend, vitiligo is considered as multifactorial disease which is caused by a combination of exogenous and endogenous factors. GST gene family encodes enzymes, which take part in in phase 2 detoxification and neutralize DNA peroxides and products of lipid peroxidation. Purpose. Assessment of predictive significance of GSTP1 gene polymorphism and clinical manifestations of vitiligo in Kazakh patients. Materials and methods. The survey questionnaire was conducted among 100 Kazakhs patients with vitiligo and 100 Kazakh volunteers. GSTP1 polymorphism the genotyping of DNA samples was performed. Results. In most cases the disease onset occurred in spring or summer. We found a new association Ala114Val-GSTP1 polymorphism with vitiligo. Segmental form of the disease is more common when presenting genotype T/T, vulgar form is more common when presenting genotype T/C. Medicosocial factors of vitiligo development were revealed. The most informative of them were used for the development of predictive table of risk factors. Conclusion. The primary lesions of depigmentation usually occur in the spring or summer and are located on exposed skin in Kazakh patients with vitiligo. The developed predictive table of vitiligo risk factors including practically significant genotypes of GSTP allows identification of individual vitiligo risk.

## Serum level and tissue expression of syntenin 2 receptor in vitiligo patients and their correlation with disease pattern, activity and severity

M. Atwa[1], R. Elgenedy[2], H. Mohamed[3], N. Ismail[1], S. Mohamed[4], N. Kamel[5]

[1]Dermatology and Venereology, Faculty of Medicine, Suez Canal University, Ismailia, Egypt; [2]Dermatology and Venereology, Elantra Gharb Hospital; [3]Dermatology, Faculty of Medicine, Suez Canal University, Ismailia, Egypt; [4]Pathology, Faculty of Medicine, Suez Canal University, Ismailia, Egypt; [5]Clinical Pathology, Faculty of Medicine, Suez Canal University, Ismailia, Egypt

Interleukin-33 signals via syntenin (ST) 2 and they play a role in the pathogenesis of many autoimmune diseases.
Our aim was to assess the serum level and tissue expression of ST2 receptor in vitiligo patients and to assess their association with disease pattern, activity or severity.
Serum level and tissue expression of ST2 receptor were assessed in 40 vitiligo patients and 40 healthy controls using ELISA and immunohistochemistry respectively and correlated with pattern, severity using (VASI) score and activity using (VIDA) score.
Mean serum ST2 level was significantly higher in patients compared to controls ($19.46 \pm 6.09$ ng/ml and $13.96 \pm 9.48$ ng/ml respectively, $P = 0.001$). There was a statistically significant correlation between serum ST2 level and activity of the disease. Fourteen (35%) of patients showed mild tissue expression of ST2 receptor, 18 (45%) of them showed moderate expression and only 8 (20%) of patients showed heavy expression. There was no significant correlation between serum ST2 and tissue expression of ST2 receptor or severity of the disease.
Our results showed increased serum level and tissue expression of ST2 receptor among vitiligo patients, which may highlight a functional role of this receptor and its ligand (IL-33) in the pathogenesis of vitiligo.

## Melatonin concentration in the blood and peculiarities of cytokine status in patients with vitiligo and stress in anamnesis

N. Tsiskarishvili Jr, A. Katsitadze, N. Tsiskarishvili, T. Tsiskarishvili

Department of Dermatology and Venereology, Tbilisi State Medical University, ‘Vitiligo Association of Georgia’

Clinical and experimental studies indicate that the hormone melatonin, which is one of the links in a stress defense

© 2017 John Wiley & Sons A/S. Published by John Wiley & Sons Ltd 

mechanism of the body, has antioxidant and immunomodulatory properties. There is a growing evidence that the basis of autoimmune pathology are disorders having place in immune regulation due to violation of cytokine production. The aim of our study was to investigate the content of some proinflammatory cytokines (IL-2, IL-6) in the serum and to determine plasma level of melatonin in the blood of vitiligo patients (with stress in anamnesis) depending on the clinical form and duration of the disease. The cytokines in the blood serum of vitiligo patients were determined by ELISA using the corresponding monoclonal antibodies. The level of melatonin in the blood plasma was determined by ELISA (IBL – international – reagent). In patients with nonsegmental form of vitiligo lowering the level of melatonin is associated with a significant decrease in the content of cytokines. We must assume that the significant reduction of melatonin and cytokines in blood, observed in some patients with segmental form of vitiligo has a poor prognostic sign, indicating the possibility of a transition segmental form of vitiligo to nonsegmental form.

## Expression of stem cell factor (SCF) and its receptor c-kit in vitiligo epidermis

D. Wilamowska-Kokoszko[1], K. Lukasik[2], J. Szepietowski[3], D. J. Skarżyński[4], W. Placek[1]

[1]Department of Dermatology, Sexually Transmitted Diseases and Clinical Immunology, University of Warmia and Mazury in Olsztyn, Poland; [2]Department of Reproductive Immunology and Pathology, Wroclaw Medical University, Poland; [3]Department of Dermatology, Venerology and Allergology, Institute of Animal Reproduction and Food Research of Polish Academy of Sciences, Olsztyn, Poland; [4]Department of Reproductive Immunology and Pathology, Institute of Animal Reproduction and Food Research of Polish Academy of Sciences, Olsztyn, Poland

IntroductionVitiligo, an idiopathic, systemic disorder, caused by the loss of melanocytes, affects approximately 0.5–4% of the world population. In the epidermal melanin unit fibroblasts and keratinocytes secrets a number of signal molecules targeting not only melanocytes but also skin immunological system. Stem cell factor (SCF) and its receptor c-kit are important for the proper process of melanogenesis.
The aim of the study is to compare expression of stem cell factor (SCF) and its receptor c-kit in the vitiligo epidermis and healthy skin surrounding the vitiligo lesions.
The study involved 36 patients with vitiligo for Dermatology Outpatient Clinic in Olsztyn. Analyzed at the gene was done using real time PCR (Applied Biosystems 7900TH).
mSCF expression significantly increased in vitiliginous epidermis than in normal skin. However sSCF did not show noticeable changes. Expression of c-Kit was significantly lower in vitiliginous epidermis than in normal skin.
Excessive production of m SCF by keratinocytes within a vitiligo pathes confirimpact of mSCF in the stimulation of melanogenesis in response to injury. Reduction of c-KIT protein expression by melanocytes within a vitiligo patches may be associated with dysfunction and/or loss of melanocytes.

## Dynamic visualization of dendritic cells in the skin from patients with vitiligo or rhododendrol-induced leukoderma

F. Yang, L. Yang, A. Tanemura, M. Wataya-Kaneda, I. Katayama

Department of Dermatology, Course of Integrated Medicine, Graduate School of Medicine, Osaka University, Suita, Japan

Vitiligo and rhododendrol-induced leukoderma are known as acquired hypomelanotic diseases and histochemically demonstrated by an absence or a marked reduction in the number of dopa-positive melanocytes. Previously, some reports indicated that melanocytes disappeared from vitiliginous skin and were replaced in their basal-layer position by cells indistinguishable from high-level clear cells or Langerhans cells. Furthermore, some other reports also revealed that melanosome/Birbeck's granule double negative cells called as alpha-dendritic existed in the skin basal layer. In this study, we investigated the existence of these cells in skin from patients with vitiligo and rhododenrol-induced leukoderma. Immunohistochemical staining and immuno-electron miscroscopy analyses for Langerhans or premelanosomes in lesional skin, leading edge, and non-involved lesion were performed. Skin biopsy specimens were obtained from 6 nonsegmental vitiligo patients in progressive state, 2 patients with rhododendrol-induced leukoderma. The increased population of langerhans cells and the premelanosome/Birbeck's granule double negative cells were noticeably detected. Interestingly, premelanosome/Birbeck's granule double positive cells also can be observed. The existence of increased Langerhans cells and premelanosome/Birbeck's granule double negative/positive cells in basal or upper-basal layer of vitiliginous skin was confirmed, suggest that the dynamic alterations of dendritic cells might be involved in the pathogenesis of the absence of melanocytes.

## Responsiveness of the vitiligo extent score (VES) to vitiligo changes and the need for a VESplus to assess repigmentation

N. van Geel[1], J. E. Lommerts[2], M. Bekkenk[2], C. A. C. Prinsen[3], V. Eleftheriadou[4], A. Taieb[5], M. Picardo[6], K. Ezzedine[7,8], R. Speeckaert[1]

[1]Department of Dermatology, Ghent University Hospital, Ghent, Belgium; [2]Department of Dermatology, University of Amsterdam and Netherlands, Institute for Pigment Disorders, Amsterdam, The Netherlands; [3]VU University Medical Center, Department of Epidemiology and Biostatistics, EMGO Institute for Health and Care Research, Amsterdam, The Netherlands; [4]Centre of Evidence Based Dermatology, University of Nottingham, Nottingham, UK; [5]National Reference Center for Rare Skin Diseases, Hôpital St-André, University Hospital Center of Bordeaux, Bordeaux, France; [6]San Gallicano Dermatology Institute, Roma, Italy; [7]EA EpiDermE Epidémiologie en Dermatologie et Evaluation des Thérapeutiques, UPE-Université Paris-Est, Service de Dermatologie, Hôpital Henri Mondor, Paris, France; [8]Pigment Disorders, Amsterdam, The Netherlands

In our original report we demonstrated the validity and reliability of the Vitiligo Extent Score (VES) to assess the extent of vitiligo. The purpose of the current study was to test the responsiveness of the VES during treatment periods. The second aim was to evaluate the need for an additional scale (VESplus) to evaluate treatment responses over time. The responsiveness of 3 methods were tested: (I) VES only (II) VES with an additional

 © 2017 John Wiley & Sons A/S. Published by John Wiley & Sons Ltd

option of a follicular repigmentation scale (III) VES combined with a global evolution score after treatment. Three raters used these 3 different methods on a randomized series of paired pictures (baseline versus follow up) of 58 vitiligo patients under treatment during 3 scoring rounds. Results were compared with the information recorded in the patients' medical file. Furthermore, we compared the proportional changes of the VES and its variants to the changes of an 'expert' equivalent score. Two expert raters determined the evolution of the lesions using a 5 point likert scale and a physician global assessment of the percentage of repigmentation. User friendliness and timing of the three methods were also recorded.
The analyses of this study are currently ongoing and can be presented at the meeting.

### Validation of the vitiligo disease activity index

J. Coias, A. Pandya
Department of Dermatology, University of Texas, Southwestern Medical Center, Dallas, TX United States

To determine if patients are accurate in reporting their disease activity as measured by Vitiligo Disease Activity VIDA scoring.
Retrospective cohort review of all patients enrolled in the Dallas Vitiligo Registry. The Vitiligo Activity Severity Index (VASI) and VIDA scores were compared over six weeks to six months. The total VASI, Vitiligo-Specific Quality of Life (VitiQoL), and Fitzpatrick skin type categories were used to determine if accuracy of VIDA scoring changed according to body surface involvement, quality of life or skin phototype.
After evaluation and application of the inclusion and exclusion criteria, 43 sets of scores were retrieved for evaluation. The total number of accurate scores was 25 (58%). The hypothesis that the total number of accurate follow-up VIDA scores are similar to those that would occur by chance was not rejected (P = 0.360). There was no significant change in accuracy of VIDA scoring based on body surface involvement (P = 0.132), VitiQoL scores (P = 0.543) or skin phototypes 1/2, 3/4 and 5/6 (P = 0.411).
The ability of vitiligo patients to determine activity of vitiligo using the VIDA is similar to that which would occur by chance.

### Classification of non-segmental facial vitiligo: a cluster analysis of 473 patients

J. M. Bae[1], Y. S. J. Jung[1], H. Lee[1], J. H. Park[2], S.-K. Hann[2]
[1]Dermatology, St. Vincent's Hospital, College of Medicine, The Catholic University of Korea, Suwon, South Korea; [2]Dermatology, Korea Institute of Vitiligo Research and Drs Woo and Hann's Skin Center, Seoul, Korea

Vitiligo has substantial negative impacts on quality of life in affected patients, especially with involvement of face. Thus, special attention should be paid to facial vitiligo.
To classify facial vitiligo.
A cluster analysis was performed with Ward's linkage of the Euclidean distance, based on the face topography. A total of 473 patients with non-segmental facial vitiligo were enrolled. Whole face was divided into 8 compartments including forehead, hairline, temple, right and left cheeks, periorbital area, nose, and perioral area. In addition, age of onset, involvement of other body parts, and treatment response were compared among the clusters.
Four clusters were identified: Cluster 1 with involvement of almost all compartments (panfacial vitiligo, n = 85); Cluster 2 with involvement of mainly periorbital area, nose, and perioral area, called (centrofacial vitiligo, n = 277); Cluster 3 with involvement of hairline exclusively (hairline vitiligo, n = 43); and Cluster 4 with mixed involvement (mixed type, n = 68). The centrofacial vitiligo was associated with the earliest onset age and the highest response rate, while the hairline vitiligo showed the oldest onset age and the lowest response rate.
ConclusionOur classification would help to identify distinctive subtypes of facial vitiligo with different etiologies.

### Chemotactic signals and autoimmunity in segmental and non-segmental vitiligo

V. Alexeev[1], A. Rezk[2], J. Uitto[3], D. Kemp[1], O. Igoucheva[1]
[1]Dermatology and Cutaneous Biology, Thomas Jefferson University, Philadelphia, PA United States; [2]Dermatology, Thomas Jefferson University, Philadelphia, PA United States; [3]Dermatology and Cutaneous Biology, Al-Minia University, Minia, Egypt

Autoimmunity was associated with progression of generalized non-segmental vitiligo (NSV) and, to some extent, with segmental vitiligo (SV). As cutaneous autoimmunity coincides with deregulated recruitment of leukocytes to the skin, altered chemotactic signals were investigated in association with vitiligo. Initial analysis demonstrated up-regulation of several chemotactic molecules including CCL5 and CXCL12 in early vitiligo lesions in association with melanocytes and infiltrating dendritic cells (DC). Furthermore, proteome analysis revealed substantial deregulation of chemotactic signals within lesional and perilesional NSV and SV samples with higher levels of CCL27, CCL11, CCL24, CXCL1, CXCL10, CCL22, CCL5, CCL17 and CXCL12. Elevated levels of deregulated chemokines were associated with the prominent infiltration of the skin with CD11c+ DC and T cells that often extent to the epidermis. In early lesions, the majority of skin-infiltrating DC express CD86 maturation marker and co-localized with the infiltrating T cells. Collectively, these findings suggest that in early SV and NSV, DC and T cells could be recruited to the cutaneous tissue by skin/melanocyte-derived chemokines resulting in acquisition of melanocytic antigens, intracutaneous T cells priming/activation and induction of melanocytes-specific CTL that remain local in SV cases and extends to systemic in NSV.

### Changes in in matrix metalloproteinases during NB-UVB induced pigmentation in cases of active vitiligo

S. Shalaby[1], T. Anbar[2], M. El Mofty[3], S. Esmat[2], W. Mostafa[2], M. Abdalah[4], H. Rasheed[2]
[1]Dermatology, Faculty of Medicine, Cairo University, Giza, Egypt; [2]Dermatology, Cairo University, Giza, Egypt; [3]Dermatology, Minia University, Egypt; [4]Dermatology, Ain ShaUniversity, Cairo, Egypt

Repigmentation in vitiligo occurs in form of either follicular pigmentation from vertical migration of outer root sheath melanoblasts, or marginal pigmentation from horizontal migration of melanocytes at lesion margins. Combined pattern result when both vertical and horizontal migration occur. Matrix metalloproteinases enable proper migration of melanocytes through extracellular matrix degradation.
To study changes of MMP2 and MMP9 during NB-UVB induced repigmentation and their correlation with different repigmentation patterns.
Skin biopsies were obtained from 21 cases of active vitiligo (VIDA: 3–4) from lesional and peri-lesional before treatment, and first site of repigmentation after NB-UVB therapy (3 sessions/week for 3 months). Immunohistochemical analysis of MMP2 and MMP9 expression was done.

© 2017 John Wiley & Sons A/S. Published by John Wiley & Sons Ltd 

MMP2 and MMP9 levels were significantly higher in pigmented points (after NB-UVB therapy) compared to lesional skin (before therapy) (P = 0.000). Also, MMP9 levels were significantly higher in pigmented points compared to peri-lesional skin (P < 0.001). The repigmentation patterns were: follicular (18%), marginal (23%), combined follicular and marginal pigmentation (59%). Correlation between different pattern of repigmentation and MMPs expression changes were not significant.
NB-UVB induced repigmentation in vitiligo is associated with MMP2 and MMP9 elevation, whether due to vertical or horizontal migration of melanocytes.

## Immunological profile of inflammatory cells in the skin of patients with vitiligo undergoing treatment

A. Pandya[1], E. J. Wright[2], J. Hinojosa[2], J. Strassner[3], J. Harris[3]
[1]Dermatology, University of Texas Southwestern, Dallas, TX United States; [2]University of Texas Southwestern, Dallas, TX United States; [3]University of Massachusetts, MA United States

Vitiligo is a characterized by CD8+ T cell-mediated melanocyte destruction in actively spreading lesions. We hypothesized that after treatment, fewer CD8+ T cells would be found in vitiligo lesions compared to normal skin.
Four consenting vitiligo patients with active vitiligo characterized by confetti-like lesions were studied. Lesional and non-lesional skin was sampled using suction blister technique before and after 4 weeks of treatment. All patients were treated with oral dexamethasone, 4 mg, on two consecutive days per week, narrowband UVB phototherapy three times per week and clobetasol cream once daily five consecutive days per week. Blister fluid was collected and subjected to fluorescence activated cell sorting to determine the characteristics of the lymphocytes in the fluid.
There was a statistically significant difference in CD8+ T cell counts between involved and uninvolved skin. In addition, treatment resulted in a reduction of CD8+ T cell counts.
CD8+ T cells are increased in vitiligo lesions and decrease with a combination of topical and systemic treatment along with phototherapy.

## The effective potential of reflectance confocal microscopy in the diagnosis of hypopigmented lesions of the skin

M. Ardigò, P. Iacovelli, A. Pacifico, M. Agozzino, M. Picardo
Clinical Dermatology, San Gallicano Dermatologic Institute, Rome, Italy

Differential diagnosis of pigmentary disorders conditions can be difficult based only on the clinical history and presentation and with the support of the most common non invasive methods as Wood lamp. Reflectance confocal microscopy is a relatively novel, in vivo method that has been successfully used for the evaluation of both hyper and hypo-pigmentary disorders accordingly with its high sensibility in the melanin pigment detection and the evaluation of pigmented cells morphology and distribution in the different skin layers. Several studies on confocal microscopy for vitiligo evaluation has been proposed in literature underlying its possibility of diagnosis and therapeutic follow-up. Moreover, accordingly to the quasi-histology resolution of this microscopic, non invasive methodology, inflammatory cells and dilated vessels can be easily detected on confocal microscopy as expression of inflammatory skin conditions.
The different microscopic features of vitiligo and other hypopigmented conditions (i.e. post-inflammatory hypopigmentation and pytiriasis alba) has been evaluated in a group of patients using confocal microscopy focusing on the pigment and pigmented cells distribution and characterization in order to evaluate the effective potential of this method in the differential diagnosis and its possible application in clinical routine.

## Predictive factors of thyroid diseases in vitiligo

M. A. S. Hamada, Y. Ouidan, B. Hassam, L. Benzekri
Dermatology, Venereology Hôpital Ibn Sina, Service de Dermatologie, Rabat, Morocco

Vitiligo is a common skin disease. The objective of this study was to evaluate the frequency of vitiligo with thyroid diseases in our patients and search the risk factors.
A cross-sectional prospective study was conducted in patients with vitiligo attending our department, between 2007 and 2014. Only the patients who have made the investigations in search of associated autoimmune diseases specially thyroid diseases were selected.
253 patients were included. The average age was 35.8 years old, with a female predominance. 145 patients had at least one disease associated. One or more autoimmune diseases were associated with vitiligo in 111 patients, the thyroid diseases were found in 81 patients. In multivariate analysis, risk factors associated with autoimmune diseases was the beginning of vitiligo after the age of 20 years old (P = 0.005), the same result for the thyroid autoimmune diseases (P = 0.002). The female had appeared as a risk factor for autoimmune thyroid disease (P = 0.032).
The autoimmune diseases associated with vitiligo are frequent, particularly thyroid disorders. The prevalence of female sex and the beginning of vitiligo post puberty that has appeared as risk factor for thyroid autoimmune diseases. The thyroid status is a relevant consideration in any patient with vitiligo.

## Quantifying vitiligo severity through digital image analysis

L. Gionfrida[1], A. W. K. Kong[2], A. Nurhudatiana[3], S. T. T. Guan[4]
[1]School of Computer Science and Engineering, Nanyang Technological University, Singapore; [2]School of Computer Science and Engineering, Procter & Gamble, Singapore; [3]Computer Scientist, Nanyang Technological University, Singapore; [4]Head of the Pigment Clinic, Singapore National Skin Centre, Singapore

Vitiligo is a pigmentation disorder of the skin that affects and destroys melanocytes, cells that respond to the pigment. As result of this skin condition some part of the subject skin loses colour in a process called depigmentation, characterized by circumscribed macules and patches. While there are treatments available for vitiligo, there are no standardized techniques for assigning severity to a vitiligo case, no quantitative or reproducible tool to evaluate the degree of depigmentation. Many scoring system used for the assessment of vitiligo are no comprehensive, they require physicians to evaluate the area of depigmentation affected producing results affected by discrepancy and inter-observer variation. This problem of lack in standardization generates inaccurate response of the treatment outcomes that cannot be uniformed so effectiveness of diverse treatment modalities cannot be compared in meta-analysis. Our aim is to provide an automated standardized tool that generates a precise estimation of the skin depigmentation during the treatment. The developed algorithm automatically identifies vitiligo lesion from normal skin

 © 2017 John Wiley & Sons A/S. Published by John Wiley & Sons Ltd

and calculate the percentage of vitiligo to evaluate the effectiveness of treatment. The algorithm is then used in a real-scenario for a clinical validation to evaluate computer scoring and compare them to human scoring of the disease.

### Novel imaging and quantification methods for the evaluation of disease severity in vitiligo and chemical leukoderma

M. Hayashi[1], K. Okamura[1], Y. Araki[1], Y. Abe[1], Y. Hozumi[1], M. Inoie[2], T. Suzuki[1]

[1]Dermatology, Yamagata University, Faculty of Medicine, Japan; [2]Japan Tissue Engineering Co., Ltd, Gamagori, Japan

Acquired skin hypopigmentation is caused by many etiologies. Among them, vitiligo is an autoimmune disorder targeting skin melanocytes, appearing as multiple irregular white patches on the skin. Rhododendrol (4-(4-hydroroxyphenol)-2-butanol)-induced leukoderma is caused by rhododendrol-containing cosmetics. It is clinically important to distinguish the lesion from normally pigmented skin to precisely assess disease severity, however, no gold standard assessment method been reported.

We aimed to investigate if digital imaging devices can be useful tools for assessing vitiligo/leukoderma disease severity by measuring the area and quantifying the color.

We compared two methods for area measurement, three-dimensional imaging analysis by the device VECTRA®H1 and the border-tracing method as manual measurement. Subsequently, we investigated whether the digital instruments Mexameter® MX18 and CM-700d spectrophotometer can quantitatively distinguish vitiligo/leukoderma from normally pigmented skin with melanin index. CM-700d was also used to quantify the color of vitiligo/leukoderma lesions and surrounding normally pigmented skin in L*a*b* color spaces.

Three-dimensional imaging analysis systems could measure the area consistently, particularly the area with complete depigmented lesions. Portable spectrophotometers can consistently distinguish vitiligo/leukoderma in normally pigmented skin.

These methods are useful and time-saving in clinical setting for assessing vitiligo/leukoderma lesions and can even determine disease severity.

### Analysis of symmetricity in the three different (sagittal, transverse and frontal) planes in generalized non segmental vitiligo

R. Hegazy[1], T. Anbar[2], K. Ezzedine[3], R. A. Hay[1], S. Esmat[1], H. Diab, M. Anbar[5]

[1]Dermatology Department, Kasr AlAiny Hospital, Cairo University, Giza, Egypt; [2]Dermatology and Venereolgy Department, BORDEAUX, France; [3]Dermatology Department, AlMinya University, Egypt; [4]Dermatology Department, Ain ShaUniversity, Cairo, Egypt; [5]Dermatology Department, Fayoum University, Cairo, Egypt

Non segmental vitiligo (NSV) has been defined as being 'often symmetrical', however, no work has tackled how valid it is to depend upon the concept of symmetricity in generalized NSV.

ObjectiveTo investigate the symmetricity of vitiligo lesions, taking into account the sites of predilection. Clinical characteristics of patients were studied in relation to this concept, in a trial to take one further step in de-mystifying this intriguing disease.

Three models were drawn for each patient, in which sagittal, transverse and frontal planes were drawn to divide the body into right/left, upper/lower and anterior/posterior halves respectively. Patients were examined and the lesions were documented by wood's light and then drawn in the three models and analyzed according to symmetricity.

This multicenteric study included 712 NSV patients with 2876 examined lesions. Bilateral affection was present in 78%, this % was significant ($P < 0.001$). Studying similarity of clinical affection in the upper and lower body parts revealed that it was present in 38%, with significant positive association in some areas. Studying the clinical similarity in the antero-posterior distribution pattern revealed a significant positive association in 11%.

The current work offered a unique insight in foreseeing the possible events in the unpredictable NSV. It highlights the concept of early intervention, not only to the affected areas, but also to the anticipated ones.

### The utility of performing routine blood investigations for associated autoimmune disease in patients with non-segmental vitiligo in South Africa

S. Kannenberg

Dermatology, Stellenbosch University, Tyger Valley, South Africa

The prevalence of vitiligo in South Africa is not known, but the significant impact on quality of life, particularly in dark-skinned individuals, is clear. Uncertainty prevails on the viability of autoimmune screening in asymptomatic non-segmental vitiligo (NSV) patients.

The aim of this study was to ascertain whether routine investigations in patients with NSV yielded any clinically relevant abnormalities.

Over a one-year period we performed full-blood count, iron, ferritin, vitamin B12, thyroid stimulating hormone (with T4 if abnormal), antinuclear antibodies, anti-thyroid peroxidase (TPO) antibodies, thyroglobulin antibodies and a random blood glucose on all NSV patients seen at the Dermatology Clinic, Tygerberg Hospital, Cape Town.

110 patients (mean age 34.0 years, 74 females) were seen. 41 (37.3%) had significant abnormalities. In total, 35 patients (31.8%) were diagnosed with clinically significant related diseases at the time of presentation or within 12 months follow up. These included iron-deficiency anaemia ($n = 16$), autoimmune thyroid diseases ($n = 11$) and type 1 diabetes mellitus ($n = 3$). After 1 year, 4 patients with anti-TPO antibodies and 2 patients with antinuclear antibodies remained free of any systemic involvement.

Almost a third of patients with NSV had an associated clinically significant systemic disease, suggesting that 'routine' screening remains relevant.

### May the plasmatic antioxidant capacity associated with dermoscopic observation be a useful parameter to value the repigmentation in patients with vitiligo

M. La Vecchia, G. Pistone, M. R. Bongiorno

Dermatology, University of Palermo, Italy

Several studies suggest that an imbalance of intracellular redox status with a significant depletion of enzymatic and non enzymatic antioxidants may be the starting event leading to destruction of melanocytes in vitiligo patients. Low catalase and glutathione levels along with increased superoxide dismutase and xanthine oxidase levels have been found in the peripheral blood of patients. Our objective was to assess the repigmentation of patients subjected to photherapy by means

© 2017 John Wiley & Sons A/S. Published by John Wiley & Sons Ltd 

of biochemical blood parameters and dermoscopic observation of the skin lesions. The enzymatic activity of catalase, the superoxide dismutasis enzimatic activity, the amount of produced radical species were valued on plasma and PMBC of 27 Dermatology U.O.C. patients in Palermo. The total antioxidant capacity was valued only on plasma and the lesions in patients presenting vitiligo were subjected to dermoscopic observation. Our results show that the enzymatic activity of both proteins is connected only to PMBC. 17 patients with the disease to repigmentation stage had low ROS levels and high total antioxidant capacity and showed repigmentation at dermoscopic analysis. In accordance with the results of our research, we think that the total antioxidant capacity, connected with the dermoscopic observation, may rapresent a useful parameter to monitor the stage of the disease.

## Effect of different types of therapeutic trauma on vitiligo lesions

H. Mashaly, M. El Mofty, S. Essmat, N. Hunter, S. Ibrahim, O. Shaker

Dermatology, Faculty of Medicine, Cairo University, Giza, Egypt

New treatment modalities for vitiligo acting by changing certain cytokines and metalloproteinases are newly emerging.
To assess the efficacy of TCA chemical peel, dermapen and fractional $CO_2$ laser in treatment of stable non-segmental vitiligo and to detect their effects on IL-17 and MMP-9 levels.
Thirty patients with stable vitiligo were recruited in a randomized controlled study. They were randomly categorized into three equal groups. Group 1: TCA peel, Group 2: dermapen machine and Group 3: Fractional $CO_2$ Laser. Skin biopsies were taken from treated areas and from control areas for which MMP-9 and IL-17 tissue levels were measured using ELISA.
The thirty vitiligo patients had low basal tissue MMP-9 levels and high baseline IL-17 tissue levels. As regards the three different used modalities, all of them caused rise in MMP-9 as well as IL-17 levels and almost their levels were much more elevated with repetition of the previously mentioned traumatic procedures. Conclusion. TCA 25% peel proved to be the most effective modality both clinically and laboratory and it can be used prior or with other conventional therapies in the treatment of vitiligo.

## The vitiligo web data base

J. Seneschal[1], K. Ezzedine[2], N. Van-Geel[3], M. Picardo[4], A. Taieb[1]

[1]Dermatology, University of Bordeaux, France; [2]Dermatology, University of Ghent, Belgium; [3]Dermatology, APHP; [4]Dermatology, San Gallicano Dermatology Institute, Rome, Italy

Important improvements have been made in the description, classification and management of vitiligo. Therefore there is a need to have access to an instrument allowing the possibility to access data from several international centres. This software is based on the Vitiligo European Task Force (VETF) questionnaire to obtain demographic, clinical characteristics and comorbidities of the patient at each consultation. A link to the Vitiligo Extent Score (VES) calculator is included to perform the VES score. Clinical pictures of the patient can be uploaded on the software at each consultation. This web base is a useful tool for patients' follow-up, evaluation of response, tolerance to therapies, and for future clinical trials in vitiligo disease. Data obtained will remain the property of each centres. Projects using data generated will be submitted to a scientific committee. Legal data protection rules will be taken into account for all the different steps.

## Development and validation of a disease activity score for vitiligo

N. van Geel[1], L. Mertens[1], A. Wolkerstorfer[2], J. E. Lommerts[2], C. A. C. Prinsen[3], V. Eleftheriadou[4], A. Taieb[5], M. Picardo[6], K. Ezzedine[7], M. Bekkenk[2], R. Speeckaert[1]

[1]Department of Dermatology, Ghent University Hospital, Belgium; [2]Department of Dermatology, University of Amsterdam and Netherlands, Institute for Pigment Disorders, Amsterdam, The Netherlands; [3]VU University Medical Center, Department of Epidemiology and Biostatistics, EMGO Institute for Health and Care Research, Amsterdam, The Netherlands; [4]Centre of Evidence Based Dermatology, University of Nottingham, UK; [5]National Reference Center for Rare Skin Diseases, Hôpital St-André, University Hospital Center of Bordeaux, France; [6]San Gallicano Dermatology Institute, Roma, Italy; [7]EA EpiDermE Epidémiologie en Dermatologie et Evaluation des Thérapeutiques, UPE-Université Paris-Est, Service de Dermatologie, Hôpital Henri Mondor, Paris, France

So far, there is no firmly established or validated instrument for measuring disease activity in vitiligo. Currently the Vitiligo Disease Activity (VIDA) score is favoured in use but the wording of the questions included in the original version can be confusing for the patient and clinicians. To address these issues, the international Vitiligo Score Working group first investigated the possible limitations of the current VIDA score by evaluating its interpretation, possible confusion and ease in use by a patients questionnaire. Based on the information obtained in the first part, 2 new scores were developed including a Vitiligo Disease Activity Score (VDAS) with focus on timing of disease activity and a Vitiligo Activity Index (VAI) including the number of active lesions. Patients were asked to score their vitiligo activity twice with an interval of 1 week using these VDAS and VAI scores (test-retest). In addition, the user friendliness of the tools was investigated. The patients' self assessment scores of disease activity were compared with the physicians' evaluation as recorded in the patients' medical file. The analyses of this study are currently ongoing and can be presented at the meeting.

## Motor tics in patients diagnosed with vitiligo: relationship to depressive and anxiety symptoms

T. Anbar[1], A. Abdel-Rahman[1], N. Abdel-Fadeel[2], A. El-Sherbiny[2], I. Kharboush[3], A. Sanad[4]

[1]Department of Dermatology and Andrology, Faculty of Medicine, AL-Minya University, Egypt; [2]Department of Neuropsychiatry, Faculty of Medicine, AL-Minya University, Egypt; [3]Department of Maternal and Child Health, High Institute of Public Health, Alexandria University, Egypt; [4]Department of Dermatology, Malawy General Hospital, Al-Minya, Egypt

Vitiligo is known to be associated with psychiatric co-morbidity. Tics are sudden, abrupt, brief, involuntary and repetitive movements with or without vocal utterances. In this work, we are studying association between vitiligo and anxiety and depressive symptoms and tics.
Our sample included 150 vitiligo patients, 30 depression patients and 30 healthy subjects. First, we compared the whole vitiligo sample to healthy subjects and depression patients. Second, we compared vitiligo patients with severe depressive symptoms (N = 30) to healthy subjects and depression patients regarding associated tics and different psychological scales including YGTSSS, PUTS, HDRS, ZARS, SPIN and CAS.

 © 2017 John Wiley & Sons A/S. Published by John Wiley & Sons Ltd

Results The main types of tics in vitiligo patients were hiding the lesion (27.3%) and rubbing the lesion (21.3%). Vitiligo patients with tics had more anxiety and depressive symptoms than vitiligo patients without tics as measured by HDRS, ZARS, SPIN and CAS (P = 0.0001). Vitiligo patients had higher scores than healthy subjects on all scales including those measuring tics YGTSSS (P = 0.0001) and PUTS (P = 0.001). Vitiligo patients with severe depressive symptoms had higher scores than healthy subjects and depression patients on YGTSSS (P = 0.01) and PUTS (P = 0.0001).

Conclusion Vitiligo patients have tendency to develop tics than healthy subjects and depression patients regardless of associated depressive symptoms.

## Quality of life impairment in children and adults with vitiligo: a cross-sectional study based on dermatology-specific and disease-specific quality of life instruments

T. F. Cestari, N. Giongo, P. Machado, R. Horn, A. Fabbrin

Dermatology, Universidade Federal do Rio Grande do Sul, Porto Alegre, Brasil

Vitiligo can negatively affect patient's quality of life (QoL). A specific questionnaire has been developed: the vitiligo-specific quality-of-life instrument (VitiQoL). The instrument was validated into Brazilian Portuguese (VitiQoL-PB).

The aim of this study was to access the QoL in adult and pediatric patients with vitiligo.

The QoL of pediatric patients was evaluated using the CDLQI questionnaire. In adult patients we used the VitiQoL – PB and the DLQI.

A strong correlation between the scores of the total VitiQoL and DLQI was observed (r = 0.81; P. There are groups of patients that are more vulnerable, like women, patients with psychiatric diseases and adolescents.

## Development and validation of a patient reported outcome measure (PROM) in vitiligo: the self-assessment vitiligo extent score (SA-VES)

N. van Geel[1], J. E. Lommerts[2], M. Bekkenk[2], C. A. C. Prinsen[3], V. Eleftheriadou[4], A. Taieb[5], M. Picardo[6], K. Ezzedine[7], A. Wolkerstorfer[2], R. Speeckaert[1]

[1]Department of Dermatology, Ghent University Hospital, Belgium; [2]Department of Dermatology, University of Amsterdam and Netherlands, Institute for Pigment Disorders, Amsterdam, The Netherlands; [3]VU University Medical Center, Department of Epidemiology and Biostatistics, EMGO Institute for Health and Care Research, Amsterdam, The Netherlands; [4]Centre of Evidence Based Dermatology, University of Nottingham, UK; [5]National Reference Center for Rare Skin Diseases, Hôpital St-André, University Hospital, Center of Bordeaux, France; [6]San Gallicano Dermatology Institute, Roma, Italy; [7]EA EpiDermE Epidémiologie en Dermatologie et Evaluation des Thérapeutiques, UPE-Université Paris-Est, Service de Dermatologie, Hôpital Henri Mondor, Paris, France

The Vitiligo Extent Score (VES) has recently been introduced as a physicians' score for the clinical assessment of vitiligo extent. We validated a simplified version of this structured instrument as a patient reported outcome measure (PROM). After promising results obtained in the pilot testing (n = 98), 50 additional patients completed the form twice to assess its reliability. The SA-VES demonstrated a very good test-retest agreement [ICC=0.948 (95% CI: 0.911–0.970)] that was not affected by age, resemblance of the patients' vitiligo distribution with the template or skin type. According to patients, this evaluation method was easy in use (22% very easy; 49% easy; 29% normal) and required less than 5 min in the majority of patients (73%: <5 min; 24%: 5–10 min; 2%: 10–15 min). Moreover, comparison of the SA-VES and VES demonstrated an excellent agreement (r = 0.986, P < 0.001). In conclusion, the results of this study demonstrate an excellent reliability of the SA-VES and an excellent correlation with its investigator-reported counterpart (VES). This patient oriented evaluation method provides a useful tool for the assessment of the extent of vitiligo and may be useful in daily practice and epidemiological studies.

## Practical tips in vitiligo

A. Alissa

Dermatology, Derma Clinics/National Center for Treatment of Vitiligo and Psoriasis, Light Clinic, Riyadh, Saudi Arabia

Vitiligo is a common acquired idiopathic disease characterized by one or more patches of depigmented caused by the loss of cutaneous melanocyte. At the National Center of Vitiligo in Saudi Arabia, we have a large number of patients who are referred for various vitiligo treatments, such as topical therapy, phototherapy, melanocyte transplant as well as depigmentation. Vitiligo has become a social stigma especially in dark-skinned patients and affects negatively their quality of life index, both socially and psychologically. In my presentation, I will demonstrate difficult vitiligo cases and their treatment modalities. In addition, I will present practical tips to enhance the outcome of therapy. We have a broad experience in autologous melanocyte transplant, as well as, skin depigmentation that I will be sharing with the audience, in addition to practical tips when choosing these treatment modalities.

## Evaluation of factors influencing objective assessment of skin color by colorimetry

A. Eid[1], T. Anbar[2], M. Anbar[3]

[1]Department of Dermatology, Venereology and Andrology, Faculty of Medicine, Alexandria University, Egypt; [2]Department of Dermatology and Andrology, Fayoum University Hospital, Cairo, Egypt; [3]Department of Dermatology, Faculty of Medicine, AL-Minya University, Egypt

Objective evaluation of skin color is an essential requirement in the management of vitiligo. Colorimeters are non-invasive instruments that are available for measurement of skin pigmentation. Several factors have been shown to bias their readings, such as ambient temperature, vasodilation/constriction and hair density.

To study other factors that may represent a potential bias to skin color determination using a colorimeter. These factors include hair color, skin wrinkling and incomplete contact of the device with the skin in certain anatomical locations.

Dermacatch® was used to determine the influence of hair color in 30 vitiligo patients, and of wrinkles and incomplete contact of the device with the skin in 30 healthy volunteers on melanin index.

Vitiligo lesions with darkly pigmented hair recorded significantly higher readings than lesions with white hair. Skin wrinkling over extended elbows resulted in significantly higher readings than skin over flexed elbows. Areas necessitating incomplete contact of the device with the skin yielded significantly higher readings than areas allowing complete contact with the skin.

Hair color, skin wrinkling and degree of contact of the device with the tested area are points that should be considered while using colorimeters in evaluating skin color.

© 2017 John Wiley & Sons A/S. Published by John Wiley & Sons Ltd 

## The role of S100B in the pathogenesis of vitiligo

R. Speeckaert, N. van geel

Dermatology, Ghent University Hospital, Belgium

S100B is a damage associated molecular pattern protein expressed by melanocytes. While its role as a biomarker in melanoma is well established, S100B has not yet been studied in vitiligo.
We investigated the serum values of S100B in 101 vitiligo patients. In vitro experiments were performed on normal and vitiligo melanocytes using repeated freeze-thaw cycli.
Significantly elevated S100B values were found in patients with clearly active non-segmental vitiligo based on follow-up pictures (P = 0.001). Additionally, patient-reported disease activity during the last 6 months was also linked to higher S100B levels (P = 0.005). Interestingly, S100B levels correlated significantly with the affected BSA (P = 0.001). Additional in vitro experiments demonstrated a massive release of S100B after melanocyte necrosis and intracellular upregulation after freeze-thaw procedures. Importantly, the annexin V+/live death-melanocytes showed a strong release of S100B in the environment indicating that S100B is already released from early necrotic melanocytes.
In conclusion, this is the first report that demonstrates the association of S100B with disease activity in vitiligo. These data suggest that this protein could play a substantial role in the pathogenesis of vitiligo and may be a potential new target for treatment.

## A randomised control trial to study of efficacy of cultured versus non cultured melanocyte transfer in the surgical management of stable vitiligo

D. Mitra

Dermatology, Indian Armed Forces College, Pune, India

Replenishing melanocytes by autologous melanocytes selectively in vitiliginous macules is a novel and promising treatment. With expertise in culturing autologous melanocytes, it has become possible to treat larger recipient areas with smaller skin samples.
To determine the modality of choice in the management of stable vitiligo and to formulate guidelines for the management of stable vitiligo by cellular grafting.
The melanocytes were harvested as an autologous melanocyte rich cell suspension from a donor split thickness graft. Cultured or non cultured melanocytes were then transplanted to the recipient area that had been superficially dermabraded. 100 patches of vitiligo in patients reporting to the hospital were randomly allocated into 2 groups to receive either of the interventions.
An excellent response was seen in 62.17% cases with the melanocyte rich cell suspension technique and in 52% with the melanocyte culture technique.
Autologous melanocyte transplantation can be an effective form of surgical treatment in stable but recalcitrant lesions of vitiligo. Large areas of skin can be covered with a smaller donor skin using melanocyte culture technique; however culture method is more time consuming, and a labour intensive process, requiring state of the art equipments with a sterile lab setup.

## Efficacy of continuous versus cyclic on-off excimer laser treatment for vitiligo: a prospective randomized controlled non-inferiority trial

J. M. Sung[1], J. M. Bae[1], H. Y. Kang[2]

[1]Department of Dermatology, Ajou University, School of Medicine, Suwon, South Korea; [2]Department of Dermatology, St. Vincent's Hospital, College of Medicine, The Catholic University of Korea, Suwon, South Korea

The 308-nm excimer laser (EL) has been considered the treatment of choice for localized vitiligo. However, there was no report on the cyclic excimer laser treatment for vitiligo, yet.
To compare the efficacy of continuous versus cyclic on-off EL treatment for vitiligo.
The paired vitiliginous lesions were randomized to continuous EL treatment (group A) or cyclic EL treatment (group B). All lesions were treated twice weekly by the EL for a total of 9-month: continuously in group A, or cyclically with 2-month treatment and 1-month intermission in group B. The degree of repigmentation was assessed with repigmentation rate (%) from baseline by using an image analysis software. The non-inferiority margin was set at 10%.
A total of 12 patients were enrolled. Thirty two paired lesions were assigned to continuous EL treatment (group A, n = 16) or cyclic EL treatment (group B, n = 16). The mean of continuous EL group minus cyclic EL group was 2.194% and the 95% confidence interval (−4.982 to 9.370%) of this difference was lower than the non-inferiority margin (10%). Interestingly, repigmentation was observed during the intermission period in group B.
Cyclic excimer laser treatment was as effective as continuous treatment.

## Association of genital vitiligo with sexual quality of life

M. Abdallah[1], S. E.-S. Youssef[1], M. El-Habiby[2], K. A. Mohsen[1]

[1]Dermatology, Venereology and Andrology Department, Ain ShaUniversity, Cairo, Egypt; [2]Psychiatry Department, Ain ShaUniversity, Cairo, Egypt

Since little is known about the effect of genital affection in vitiligo on psychiatric well-being and sexual QOL (SQOL), we aimed to determine the association of genital vitiligo with QOL, SQOL and sexual health. A questionnaire-based study was conducted on 219 [100 males (M) and 119 matched females (F)] vitiligo patients. Patients with genital affection (GV) were compared with those with vitiligo on exposed areas (EV) using the Dermatology Life Quality Index (DLQI), General Health Questionnaire (GHQ), International Index of Erectile function (IIEF) for males and Female sexual Function Index (FSFI) for females. We found a non-significant effect of genital vitiligo on the DLQI in M and F. Sexual quality of life was significantly affected in females (F-GV and F-EV) compared to males with effect on function (P = 0.001 and 0.0001), orgasm (P = 0.001 and 0.026) and overall sexual life (P = 0.002 and 0.019 respectively). Within each gender, there was no difference between patients with GV and patients with EV as regards the distress, embarrassment, DLQI, GHQ and the overall SQOL, however male patients with genital vitiligo showed significant distress (P = 0.025).
Genital vitiligo has no effect on patients' psyche, general or SQOL. However, females with vitiligo have a worse SQOL compared to males.



© 2017 John Wiley & Sons A/S. Published by John Wiley & Sons Ltd

## Approaching a vitiligo patient

A. Alomar

Dermatology, Institut Universitari Quiron Dexeus, Barcelona, Spain

When evaluating vitiligo, the dermatologist must bear in mind that although not a serious health risk, it does have a great psychological impact, which is why he must be proactive and be motivated to convince the patient to carry out the treatment. As with any dermatological process which does not guarantee a total cure and with a long-term yet simple treatment perspective which despite being uncomfortable has very rare side effects, the vitiligo-dedicated dermatologist must know how to transmit to the patient the three following virtues: Faith, Hope and Charity. Personally this is the focus that I use with my patients that usually come to me looking for an expert. My treatment can be prescribed by any dermatologist but it could be difficult to obtain nice results without following the recommendations mentioned previously. I think that all vitiligo specialized dermatologists must be able to stimulate patients to accept the treatment but not also this, It could be important to teach general dermatologists to avoid the normal negative input that is the common sentence 'this is only an aesthetic disturbance with no treatment' Taking as a starting point that treatments must suit the patient and not only the illness.

## The treatment of vitiligo patients and new treatment approaches

S. Z. B. Gey

Medical Student, Medical University of Pleven Bulgaria

Vitiligo is a pigmentation disorder in the skin. It is also known as Leucoderma. This disorder is characterized by the destruction of melanocytes. These cells are responsible for skin colour. It affects 2% of the general population and appear with equal frequency in women and men. However the number of female patients admitted to clinic is more. Vitiligo can be seen at any age. Vitiligo has a significant impact on patients of their psychological health and their social functions particularly interpersonal relationships are affected significantly. The actual cause is not known but researches are suggested that its due to autoimmunity, genetic, neural and viral. the reason for the existence of Vitiligo, the classical theory and many new theories have tried to identify the disease although the etiology of vitiligo is still a subject of discussion. Treatment of vitiligo varies depending on the lesions of penetration, localization, age and the response of previous treatments. In this compilation, Vitiligo treatment, traditional treatment, new treatment options, surgical treatment, experimental treatments are discussed under the headings of other treatment options and it has been evaluated in accordance with the current literature.

Vitiligo, traditional treatment, new treatment, experimental treatment, surgical treatment.

## Repigmentation of vitiligo lesions is associated with improved quality of life

J. A. Hinojosa, A. G. Pandya

Dermatology, University of Texas, Southwestern Medical Center, Dallas, TX United States

Vitiligo has profound effects on quality of life (QOL). We hypothesized that improvement in vitiligo severity is associated with improved QOL.

We performed a retrospective cohort analysis of patients enrolled in the Dallas Vitiligo Registry who had a body surface area involvement measured using the Vitiligo Area Scoring Index (VASI) and QOL measured using the short form 36 (SF-36v2) at their initial visit and after at least 6 months of therapy.

19 participants met inclusion criteria and had a mean baseline VASI score of 6.8 and mean follow-up VASI of 4.26. Decrease in VASI scores correlated with improved QOL, reflected by an increased in the mental health score ($r = -0.45$, $R^2 = 0.20$) and mental component summary score ($r = -0.57$, $R^2 = 0.32$). In addition, patients with >25% improvement in VASI had significantly better mental component summary scores compared to those with <25% improvement ($P = 0.045$).

ConclusionImprovement in vitiligo disease severity is associated with improved mental health scores using the SF-36. Lower SF-36 scores have been associated with risk for depression in some studies suggesting that repigmentation of vitiligo lesions in affected patients may decrease the risk of developing depression.

## Quality of life in patients with vitiligo according to gender and age of onset

J. A. Hinojosa, E. Miller, A. G. Pandya

Department of Dermatology, University of Texas, Southwestern Medical Center, Dallas, TX United States

Vitiligo patients experience significant impairment in quality of life (QOL); however, it is still unclear what underlying factors contribute to this impairment. We hypothesized that women have a worse QOL than men and those diagnosed at a younger age are better at coping with their disease and thus have a better QOL than patients diagnosed at an older age.

The QOL of 201 patients enrolled in the Dallas Vitiligo Registry was measured using the Vitiligo Quality of Life Index (VitiQoL). Patients were grouped by gender and age groups based on the Erikson's stages of psychosocial development. VitiQoL scores were analyzed and comparisons were made between study groups by descriptive statistical analysis.

Women had a significantly worse QOL than men, particularly in the stigma and behavior domains of the VitiQoL (P-values: <0.0001 and 0.0053, respectively). We also found that patients who were diagnosed between ages 20 and 65 have a worse QOL than those diagnosed at a younger age.

Women with vitiligo have worse QOL than men. Patients diagnosed in childhood have better QOL than those diagnosed in adulthood.

## Trials and results of various novel therapies for intractable cases of vitiligo vulgaris

T. Shibata

Shibata Clinic of Dermatology, Osaka, Japan

For a long time no effective strategy for the therapy for intractable vitiligo have never been found. Recently I hit upon and carried out several therapies for intractable vitiligo. My basic hypothesis is removal of toxic materials (X-OOH, now re-examined) which may exclude normal melanocytes' immigration from surrounding normal skin. Dipending on this hypothesis I'm carrying out next therapies to activate macrophages by stimulating transient receptor potential (TRP) or removing superperoxides. These therapies are (1) heat sensation (over 43°C) by far infrared ray irradiation, (2) various laser irradiation ($CO_2$ laser ablation, YAG laser, Fractional laser), (3) 30% capsaicin ointment application, (4) 1% L-menthol cream application, (5) 1% oregano cream application, (6) platinum-palladium nano colloids mixed cream application (for the purpose

© 2017 John Wiley & Sons A/S. Published by John Wiley & Sons Ltd 

of removal of superoxides, which has pseudo-catalase activity). Each therapies shows better repigmentation comparison with excimer lamp irradiation only. And finally I report the effect of 311 nm Titan-silicon diode laser irradiation on intractable vitiligo vulgaris. This machine was made in Korea by very novel technology.

## Identifying key components for a psychological intervention for people with vitiligo – outcome of two focus groups

L. Steed[1], A. Ahmed[2], E. Burden-Teh[3], R. Shah[4], S. Sanyal[5], S. Dowey[3], M. Whitton[6], J. Batchelor[3], A. Bewley[4]

[1]Centre for Public Health and Primary Care, Queen Mary University of London, UK; [2]Watford General Hospital, UK; [3]Centre of Evidence Based Dermatology, University of Nottingham, UK; [4]Barts Health NHS Trust, London, UK; [5]Sandwell and West Birmingham NHS Trust, West Midlands, UK; [6]Cochrane Skin Group, University of Nottingham, UK

Vitiligo has important psychological and psychosocial impacts, but interventions to address these have been sparse. Our prior survey of people with vitiligo (PWV) and healthcare professionals (HCPs) identified that interventions focusing on acceptance and managing social impact are a priority.

We conducted focus groups to explore issues identified by the survey and obtain feedback on intervention content, with specific reference to psychologically-based interventions; namely cognitive behavioral therapy (CBT) and acceptance commitment therapy (ACT).

The study was funded by the UK Dermatology Clinical Trials Network. Ethical approval was obtained from Queen Mary University of London. Two focus groups were conducted with PWV (N = 11). Topics included impact of vitiligo, reflection on survey outcomes, importance of psychosocial interventions, and whether CBT or ACT approaches would be preferable. Analysis utilized a thematic framework approach.

Key themes included the impact of vitiligo on multiple areas of life, especially exercise behavior and self-esteem. The role of acceptance and it's relation to behaviors, such as using camouflage, was significant. HCPs were deemed to offer insufficient support to PWV.

There is demand for psychosocial interventions for PWV. Basic self-management/psychoeducational approaches would be beneficial, with more complex interventions relevant where needs are more challenging.

## Surgical approaches to vitiligo treatment: literature update and experience with in the period 1991–2014 of the dermatology unit of Siena

L. Tognetti, E. Pianigiani, G. Mariotti, P. Taddeucci, F. Ierardi, P. Rubegni, M. Fimiani

Dermatology Unit, Department of Medical, Surgical and Neuro-Sciences, University of Siena, Italy

There is no fully effective treatment for the treatment of vitiligo to date, and the absence of randomized clinical trial does not allow to formulate strict recommendations for clinical practice; in general, combination therapies have proved to be superior compared to mono therapies. In this context, the surgical treatment of vitiligo represents the only alternative for those patients with stable vitiligo refractory to nb-UVB light and/or medical therapies (both topical and systemic). The quote of patient that can be defined candidate to surgical therapy is actually limited. Thus, only sporadic experiences of small groups of patients are reported in medical literature: between these, various different surgical techniques have been described, with not omogenous methods and difficult to compare results. We here review and examine all currently available surgical techniques for the treatment of stable vitiligo. In particular, we describe the experience of the Dermatology Clinic – Laboratory of Cell Culture of the University of Siena, including: autografting of autologous skin in vitro cultured (used until 2000) and autologous epidermal cells suspension (used until 2014). We also evaluate the clinical results observed 15 years after treatment with grafting of autologous cultured epidermal cells.

## An increased risk of comorbid rheumatic disorders in vitiligo patients: a nation wide population-based study

C. W. Choi[1], S. H. Eun[2], K. H. Choi[3], J. M. Bae[3]

[1]Department of Dermatology, Seoul National University Hospital, South Korea; [2]Department of Dermatology, Veterans Health Service Medical Center, Seoul, South Korea; [3]Department of Dermatology, St. Vincent's Hospital, College of Medicine, The Catholic University of Korea, Suwon, South Korea

The previous studies on the comorbidity in the patients of vitiligo showed significant associations with autoimmune disorders. However, large scale epidemiologic study focused on the comorbid rheumatic disorders was not performed.

To clarify the association of vitiligo with rheumatic disorders in a nationwide population study.

We performed cross-sectional study using data from national health insurance system in South Korea. We enrolled all patients with vitiligo and collected age and gender matched controls. Multivariate logistic regression analysis was performed to clarify the association between vitiligo and rheumatic disorders.

From 2009 to 2013, a total of 86 210 vitiligo patients and 172 420 controls were enrolled for the study. Vitiligo patients were at increased risks of systemic lupus erythematosus, systemic sclerosis, Sjögren's syndrome, and rheumatoid arthritis. However, no statistically significant association was found in dermatomyositis/polymyositis, Behçet's disease, and ankylosing spondylitis. Subgroup analysis showed an increased risk of dermatomyositis/polymyositis in male, while ankylosing spondylitis in female vitiligo patient. In young vitiligo patients, increased risks of dermatomyositis/polymyositis and ankylosing spondylitis were found.

Our study confirmed the significant association between vitiligo and rheumatic disorders. In addition, differences in the comorbid rheumatic disorders according to the age or gender suggest patient specific approaches.

## Vitiligo treatment recommendations: where are we now?

A. Stanimirović[1,2,3], M. Kovacevic[3,4], M. Šitum[4]

[1]University of Applied Health Sciences, Mlinarska 38, Zagreb, Croatia; [2]Private Dermatology Office, Badalićeva 26, Zagreb, Croatia; [3]Croatian Vitiligo Association, Badalićeva 26, Zagreb, Croatia; [4]Department of Dermatology and Venereology, University Hospital Center 'Sestre milosrdnice', Zagreb, Croatia

Vitiligo is an acquired depigmentation disease with a large impact on psychosocial life of patients, due its, quite often impressive, clinical presentation. Because of the fact that vitiligo is not contagious or life-threatening disease, patients are often confronted with difficulties in receiving suitable therapy. Numerous successful therapeutic options are available for treatment of vitiligo, but physicians usually do not recognize

 © 2017 John Wiley & Sons A/S. Published by John Wiley & Sons Ltd

patients' problems and consider vitiligo as only a cosmetic problem, which should be treated only by camouflage. Also, because of the lack of the accurate information for patients, a widely open market for different kind of alternative questionable therapies occurs, so patients are often experimenting with different types of unproven medications. The need for widely accepted consensus concerning vitiligo treatment and establishment of the therapeutic guidelines exists worldwide.

Here we present a review of therapy for vitiligo regarding to various vitiligo types and severity of lesions, and as well existing therapeutic algorithms constituted from different worldwide experts and organizations UK, European Vitiligo Task Force, USA, Japan.

Croatian Vitiligo Working Group considered all available topical, systemic and surgical therapeutic options and introduced in 2014 for the first time vitiligo therapy guidelines in Republic of Croatia, based on the fully accepted vitiligo therapy world recommendations and our own experience.

Our proposal for vitiligo treatment and selection of particular therapeutic options in several therapeutic lines are based on international recommendations in accordance with evidence based medicine. Our suggestion of therapeutic guidelines is also adjusted to current situation in public health and health economics in Croatia. Our intention is to improve approach to therapy for patients with vitiligo and as well social component of patient's life through rising awareness of this condition which affects over 35 million people worldwide.

## Spontaneous autoimmune vitiligo in the Smyth chicken: relevant intermediated model for pre-clinical research

G. Erf, D. Falcon

Poultry Science, University of Arkansas-Fayetteville, AK United States

Autoimmune vitiligo, like other autoimmune diseases, is a multifactorial, non-communicable disorder where development of the disease in an individual involves interplay of genetic, environmental, metabolic, and immune system components. While rodent models are considered the most convenient experimental animal for biomedical research, no mouse models exhibiting truly spontaneous development of autoimmune vitiligo exist. A variety of murine models with induced autoimmune vitiligo have recently been developed and play an important role in dissecting immune mechanisms involved in melanocyte loss and in the development of targeted medical treatments. Before clinical trials, it is however important to also conduct treatment testing in animals with spontaneously occurring autoimmune vitiligo. The spontaneously developing vitiligo observed in the Smyth line (SL) of chickens has long been characterized as autoimmune and multifactorial in nature, and many parallels to human autoimmune vitiligo have been established over more than 25 years of research. Recent studies in SL chickens focusing on melanocyte-specific T cell responses, the role of environmental factors in vitiligo onset, melanocyte responses to cellular stress, and development of the target tissue as a skin test-site, further confirm the suitability, importance, and relevance of this model for translational research and inclusion in pre-clinical studies.

## Protective effects of platinum and palladium nanoparticles on human keratinocytes via ahr and NRF2 activation: implication in vitiligo therapy

M. Ichihashi[1], G. Tsuji[2], T. Shibata[3], H. Ando[4], Y. Yamamoto[5], Y. Minamiyama[6], M. Furue[7]

[1]Department of Dermatology, Saisei Mirai Clinic, Kobe, Japan; [2]Department of Dermatology, Shibata Clinic of Dermatology, Osaka, Japan; [3]Kyushu University, Fukuoka, Japan; [4]Department of Applied Chemistry and Biotechnology, Okayama University of Science, Japan; [5]School of Bioscience and Biotechnology, Tokyo University of Technology, Japan; [6]Food Hygiene and Environmental Health, Division of Applied Life Science Kyoto Prefectural University, Japan; [7]Department of Dermatology, Kyushu University, Fukuoka, Japan

PAPLAL, novel metal nanoparticles of platinum (Pt) and palladium (Pd) are reported to have the potential to be an antioxidant agent. We have already reported an efficacy of topical use of PAPLAL for repigmentation of vitiligo, though the mechanism are unclear. Based on the evidence that PAPLAL exhibit antioxidant properties, we hypothesized that PAPLAL may activate the aryl hydrocarbon receptor (AHR)–nuclear factor-erythroid-2-related 2 (NRF2) axis, a redox machinery against oxidative stress, in human keratinocytes. To demonstrate this, we investigated the effects of PAPLAL on the activation of AHR and NRF2 in normal human epidermal keratinocytes (NHEKs). We have found that PAPLAL exhibit dual effects on AHR and NRF2 pathways. The AHR activation inhibits IFN-$\gamma$-induced (C-X-C motif) ligand (CXCL) 10 production in NHEKs, one of key factors in the development of vitiligo. Furthermore, the NRF2 activation enhances the antioxidant NAD(P)H quinone dehydrogenase (NQO) 1 expression in NHEKs, preventing cell damages caused by IFN-$\gamma$-producing T-cells. Further, we found that PAPLAL extended longevity of cultured melanocytes from vitiligo patient. These data suggest an important role of reactive oxygen species in the development of vitiligo and the therapeutic potential of PAPLAL in vitiligo

## Histopathological comparison with anti-PD-1 antibody-induced leukoderma, vitiligo and rhododenol-induced leukoderma

Y. Kotobuki, A. Tanemura, N. Arase, F. Yang, L. Yang, M. Wataya-Kaneda, I. Katayama

Dermatology, Graduate School of Medicine, Osaka University, Suita, Japan

Anti-PD-1 antibody, nivolumab, is the most relevant immune checkpoint inhibitor and was approved for advanced melanoma and in 2014 followed by non-small-cell lung cancer (NSCLC) in Japan. We administered nivolumab to 18 advanced melanoma patients and noticed vitiliginous macules on 7 patients so far. Surprisingly, 6 out of 7 with vitiliginous lesions had showed significant clinical response of 'complete remission' or 'partial response'. Since anti-PD-1 antibody-induced leukoderma has not been occurred in NSCLC patients, anti-PD-1 antibody-induced leukoderma in melanoma might be involved in melanoma and/or melanocyte specific immune response. In 2016, Freeman et al. reported that there are statistically significant OS differences in patients with leukoderma compared with non-leukoderma. These results indicate that the occurrence of anti-PD-1 antibody-induced leukoderma might be an estimated effective predictor of anti-PD-1 antibody. To reveal a detail immune cellular response to melanocyte in the skin, vitiliginous macules were biopsied and used for immunohistochemical analysis of residual melanocytes

© 2017 John Wiley & Sons A/S. Published by John Wiley & Sons Ltd 

and immune-competent cells. In this study, we will discuss the mechanisms of anti-PD-1 antibody-induced leukoderma in comparison with vitiligo and Rhododenol-induced leukoderma.

## Epidermal cell suspension prepared inside suction blisters for transplantation in vitiligo

N. Raboobee, M. Raboobee

Westville Hospital, South Africa

A novel technique for the preparation of a non-cultured epidermal cell suspension for transplantation in stable vitiligo was recently described by Gupta et al.

Two patients were recently treated with this procedure, in Durban, South Africa and early results seem promising, with new pigment noted in the vitiligo skin within one month of the procedure in the first patient. This is the first time that this procedure has been performed in South Africa.

Suction blisters are produced on the outer thigh. The fluid in the blisters is removed and replaced with trypsin while the blisters are left intact. After 45 min, the fluid containing trypsinised epidermal cells is aspirated and collected in a petri dish. The suction blister roofs are excised and placed in the same dish. The dermal sides of the blister roofs are scraped to release any loosely attached epidermal cells. The roofs are used as a biological dressing to cover the denuded suction blisters.

Vitiligo areas are prepared with a dermabrader to remove the epidermis. The trypsinised suspension is applied onto the denuded recipient areas, covered with collagen sheets, dressed and left in place to repigment the vitiliginous skin.

This technique differs from traditional non-cultured melanocyte grafting in the following ways:

- An incubator is not required – the fluid is prepared inside intact blisters and the body acts as an incubator.
- A centrifuge is not used.
- PBS solution is not used – the small amounts of trypsin used in this procedure are neutralized quickly by the body and have no effect on the dermis.

The technique is simple, easy to learn, inexpensive, requires minimum handling of tissue outside the body and requires very basic equipment.

Somesh Gupta, Kanika Sahni, Manoj Kumar Tembhre, Sandeep Mathur, and Vinod Kumar Sharma. A novel point-of-care in vivo technique for preparation of epidermal cell suspension for transplantation in vitiligo. Am Acad Dermatol 2015;72:e65–6.

## First transplantation of basal cell layer suspension using a dermarolling system in vitiligo: a preliminary clinical study of 5 cases

L. Benzekri, Y. Gauthier

Dermatology, Mohammed V University, Rabat, Morocco

Actually the methods for melanocyte delivery are invasive and often sophisticated. The dermarolling system with needles causing tiny microinjuries in the epidermis could offer a minimally invasive and painless method of melanocyte transplantation.

To develop a new and simple method for transepidermally delivering keratinocytes and melanocytes into vitiligo skin.

5 patients with stable vitiligo (4 segmental vitiligo of the face and 1 achromia post halo nevus) were recruited. The cell suspension obtained by cold trypsinisation was applied on the surface of the skin, enabling the cells to penetrate into the resulting skin defects. Rollers with 540 microneedles, 0.2 mm in length, were repeatedly passed over the skin. A Nb-UVB phototherapy was immediately performed three times a week.

After 4 months, the repigmentation was shown in all patients by the clinical digital photography. The repigmentation was excellent (3 cases/5) or moderate (2 cases/5).

Small defects created in the surface epithelium allow a separated implantation of cells into the epidermis without inflammatory reaction but with some problems of migration of melanocytes.

It is a simple, non invasive, safe and non expensive technique for melanocytes delivery.

## The role of phototherapy in the surgical treatment of vitiligo: a systematic review

A. Lommerts[1], M. Bekkenk[1], M. de Rie[2], A. Wolkerstorfer[1]

[1]Netherlands Institute of Pigment Disorders, Academic Medical Center, Amsterdam, The Netherlads; [2]Dermatology, Academic Medical Center, Amsterdam, The Netherlads

Surgical treatment of vitiligo is frequently combined with subsequent phototherapy. There is no consensus whether the addition of phototherapy is advantageous. The objective of this review was to summarize and review the evidence of phototherapy in the surgical treatment of vitiligo.

We systematically searched electronic databases (EMBASE, MEDLINE, CENTRAL) and selected eligible studies. We critically appraised the studies with the Cochrane Risk of bias assessment. From each study pre-specified data were extracted and documented.

We identified 31 eligible articles which assessed PUVA (13 studies), NB-UVB (5 studies), UVA (1 study), active sunlight exposure (7 studies) or different phototherapies (5 studies) before or after surgical treatment of vitiligo. We identified 12 RCTs, 1 clinical trial and 18 case-series with a total of 1455 vitiligo cases. The quality of all studies was rather poor and heterogeneity was high. In 2 comparative studies, the administration of either NB-UVB or monochromatic excimer light after autologous melanocyte transplantation resulted in 12–26% more repigmentation than no phototherapy. No differences between phototherapy modalities were seen.

Phototherapy is an effective adjuvant therapy in the surgical treatment of vitiligo. Further research on the role of phototherapy in the surgical treatment of vitiligo is needed.

## Vitiligoid lesions occurring under anti-PD-1 therapies are clinically and biologically distinct from vitiligo

J. Seneschal[1], K. Boniface[2], C. Jacquemin[2], J. Rambert[3], A. Taïeb[1], C. Dutriaux[4], S. Prey[4]

[1]Dermatology, INSERM U1035, Université de Bordeaux, France; [2]Immunodermatology, Université de Bordeaux, France; [3]Aquiderm, Bordeaux, France; [4]Dermatology, CHU de Bordeaux, France

Immunotherapies targeting programmed cell death 1 (PD-1), a major checkpoint in the effector phase of cytotoxic T cells, have shown remarkable clinical results in the treatment of cancers, such as metastatic melanoma. Nonetheless, these anti-PD-1 therapies are associated with development of immune-related adverse effects. Among them, the occurrence of vitiligoid lesions is of particular interest in the context of melanoma, because this side effect seems associated with increased survival. Therefore, we sought to characterize clinically and biologically vitiligoid lesions occurring under anti-PD-1 therapies in patients with

 © 2017 John Wiley & Sons A/S. Published by John Wiley & Sons Ltd

metastatic tumors. All 8 patients included in this study developed vitiligoid lesions localized on photoexposed areas with a specific depigmentation pattern consisting of multiple flecked lesions without Koebner's phenomenon. In contrast to vitiligo, patients developing vitiligoid lesions under anti-PD-1 did not report personal or familial histories of vitiligo, thyroiditis or other autoimmune disorders. Analysis of blood and skin samples revealed increased CXCL10 levels in serum of patients developing vitiligoid lesions, associated with skin infiltration of CD8+ T cells expressing CXCR3 and producing elevated levels of IFNg and TNF-a. In conclusion, clinical and biological patterns of vitiligoid lesions occurring under anti-PD-1 differ from vitiligo, suggesting a different mechanism leading to the loss of melanocytes.

## Novel autofluorescence biomarkers of vitiligo

H. Lui, J. Zhao, S. Kalia, M. Al Jasser, S. Zandi, N. Kollias, V. Richer, H. Zeng

Department of Dermatology and Skin Science, University of British Columbia and Photomedicine Institute, Vancouver Coastal Health Research Institute, Vancouver, Canada

Skin fluorescence depends on the intensity of the exciting light, the absorption properties of the constituent molecules, and the efficiency with which the absorbed photons are converted to emission signals. Cutaneous autofluorescence has been evaluated in a number of physiological and pathological processes such as aging, photoaging, psoriasis and skin carcinoma, and shown to exhibit characteristic spectral features. The objective of this in vivo study was to systematically measure and characterize the autofluorescence properties of vitiligo as compared to adjacent normal appearing skin.
Cutaneous autofluorescence were measured using fluorescence excitation-emission (EEM) spectroscopy. The excitation and emission wavelengths were 260–450 nm and 300–700 nm, respectively. Thirty-five vitiligo patients participated in this study (mean age 47, range 24–69); there were 19 males and 16 females. Vitiligo lesions were distributed on head/neck (9 cases), trunk (5), upper extremities (14), and lower extremities (7).
As expected, the most pronounced difference between spectra obtained from vitiliginous skin compared to adjacent normal skin was the higher overall epidermal and dermal fluorescence in vitiligo which is largely related to the characteristic spectral absorption of epidermal melanin. However, detailed comparison of the fluorescence spectra from vitiligo to normal skin revealed three distinctive excitation spectral bands centered around 280–295 nm, 310 nm and 335 nm. The 280–295 nm band may be related to inflammation and/or epidermal proliferation as vitiligo is an autoimmune disease, whereas the 335 nm band may arise from collagen crosslinks, which may be related to aging and photoaging. The source of the 310 signal is uncertain, but it may be possibly be related to reactive oxygen species which others have reported within vitiligo.
Vitiligo exhibits three fluorescence excitation bands around 280–295, 310, and 335 nm that may potentially serve as disease biomarkers.

## Success of excimer light in the treatment of vitiligo lesions which stopped improvement while on NBUVB therapy

M. Anbar[1], T. Anbar[2]

[1]Department of Dermatology, Fayoum University Hospital, Giza, Egypt; [2]Department of Dermatology and Andrology, Faculty of Medicine, AL-Minya University, Egypt

Narrowband UVB (NBUVB) is considered the mainstay of vitiligo treatment. Nevertheless, some cases show initial response which ceases to improve after a number of sessions. In such cases the improvement of vitiligo is beneath the requirements of both the patient and the doctor. Excimer light is another documented modality in the treatment of this disease. In the current work we report a series of ten cases of NSV who stopped improvement during the last 3 months of NBUVB therapy. The cases were shifted to excimer light which they received twice weekly, and were meticulously followed up every 8 sessions. Seven of these cases (70%) showed marked improvement after 3 months, while the remaining three cases (30%) showed poor response. Excimer light may be a hopeful modality in the improvement of the final results of NSV under treatment with NBUVB.

## Comparison of 311-nm Ti:Sapphire laser versus 308-nm excimer laser treatment for vitiligo: a prospective randomized controlled non-inferiority trial

J. M. Bae, S. H. Eun, H. Lee, J. H. Lee, G. M. Kim

Dermatology, St. Vincent's Hospital, College of Medicine, The Catholic University of Korea, Suwon, South Korea

The 308-nm excimer laser (EL) has been widely used for localized vitiligo. Recently, the 311-nm Ti:Sapphire laser (TSL) was developed to treat patients with vitiligo.
ObjectivesTo compare the efficacy of TSL versus EL in the treatment of vitiligo.
A randomized controlled non-inferiority trial based on split-body was performed. The paired symmetric vitiliginous lesions were randomized to TSL or EL treatment groups. All lesions were treated twice weekly for a total of 12-week period. The degree of repigmentation was assessed as % from baseline by using a computer program every 4 weeks, and the non-inferiority margin was set at 10%.
A total of 16 patients, aged 24 to 79 years, were enrolled. Fifty-two paired lesions were assigned to EL group (n = 26) or TSL group (n = 26). Until now, 22 paired lesions complete the 12-week trial, and the interim analysis of the last observation was conducted. The mean difference between two groups (EL minus TSL) was 2.263%, and the 95% confidence interval (−4.485 to 9.011) was lower than the non-inferiority margin (10%).
The present study demonstrated that 311-nm TSL was as effective as 308-nm EL in the treatment of vitiligo.

## Evaluating insulin resistance and the risk factors for cardiovascular disease in adult patients with vitiligo: a controlled study

T. F. Cestari[1], C. C. Martins[2], N. P. Giongo[3], R. Horn[4], A. R. Fabbrin[4], R. G. Machado[4], J. C. Boza[1]

[1]Dermatology Department, Hospital de Clínicas de Porto Alegre, Brazil; [2]Dermatology Department, Universidade Federal do Rio Grande do Sul, Porto Alegre, Brazil; [3]Medical Scholar, Hospital de Clínicas de Porto Alegre, Brazil; [4]Medical Scholar, Universidade Federal do Rio Grande do Sul, Porto Alegre, Brazil

© 2017 John Wiley & Sons A/S. Published by John Wiley & Sons Ltd 

While the relationship between cardiovascular disease, insulin resistance (IR) and vitiligo has been investigated, there is no consensus on the issue.
To evaluate the relationship between IR and vitiligo using the Homeostasis Model Assessment of Insulin Resistance (HOMA-IR) and the Homeostasis Model Assessment of β-Cell Function (HOMAβ), and also the prevalence of risk factors for cardiovascular disease in adults with vitiligo, compared with a control group.
Controlled cross-sectional study. A convenience sample of individuals aged 14 years or older was used. Individuals with psoriasis or diabetes were excluded. Participants underwent laboratory tests and anthropometric measurements. The lipid accumulation product (LAP), HOMA-IR and HOMAβ indexes were calculated.
130 individuals were included, 73 with vitiligo and 57 controls. There was no significant difference between the groups when the LAP, HOMAIR and HOMAβ values were assessed. Among the risk factors for cardiovascular disease only systolic blood pressure (BP) was significantly higher in vitiligo group.
No higher prevalence of IR was found in individuals with vitiligo. Regarding the risk factors for cardiovascular disease, only systolic BP was higher in this group. Further studies are needed to elucidate the prevalence of IR and cardiovascular risk factors in patients with vitiligo.

## Evaluation of choroidal thickness in the eye of vitiligo patients

S. Demirkan[1], Z. Onaran[2], O. Gunduz[1], F. Ozkal[2], G. Samav[1], A. A. Karabulut[1]
[1]Dermatology and Venerology, Kirikkale University, Faculty of Medicine, Turkey; [2]Ophtalmology, Kirikkale University, Faculty of Medicine, Turkey

Destruction of pigment cells in vitiligo may not remain limited to the skin; the eyelashes, iris, ciliary body, choroid, retinal pigment epithelium and meninges may also be affected. The purpose of this study was to evaluate the thickness of the choroid layer, where melanocytes are present densely, in vitiligo through thickness measurement using optical coherence tomography and determining the correlation between choroidal thickness and disease severity.
While the foveal and parafoveal choroidal thickness was determined by measuring manually the region between the outer border of the retinal pigment epithelium layer and the sclero-choroidal interface, measurements in the peripapillary area were carried out automatically with the instrument.
In those with higher VASI value, periorbital involvement was significantly more frequent. It was observed that the frequency of periorbital involvement increased with the duration of the disease.
The melanocyte amount in the choroidal layer in vitiligo should be studied in other postmortem and in vivo studies.

## A new simple method for recipient site preparation using electro-dissection during epidermal graft procedure in acral vitiligo surgical treatment

H. El-Fakahany, W. Medhat, H. Abdel-Raouf
Minia Dermatology and Venereology Minia, Faculty of Medicine

Acral vitiligo is considered one of the challenging dermatoses facing dermato-surgeons. Although several surgical procedures are used for treating such cases, yet they need special experience to avoid graft rejection. These procedures also are time consuming and need special and expensive equipment for preparing recipient site.
Creation of a simple, easy, effective, time saving and less costly method for preparation of recipient site in epidermal graft procedures.
Epidermal graft was obtained by cupping of normal skin in the thigh area, served as donor site, of twenty four patients suffering from stable acral vitiligo. Electro dissection equipment was used for dermo-epidermal separation at recipient areas. Follow up of patients continued for 6 months post-procedure.
Repigmentation was obtained in twenty one cases successfully. Preparation of recipient sites was done effectively and successfully in all cases without graft rejection.
Electrodissection may provide a new, simple and effective method for preparing recipient site in epidermal grafts treating acral vitiligo.

## Treatment of stable vitiligo with combination of UVB and cell technologies

K. Karpenko, V. Tsepkolenko
Dermatology, UIPS Virtus, Odessa, Ukraine

Vitiligo is an acquired skin pigmentation disorder characterized by loss of epidermal melanocytes, whose aetiopatogenesis is still under debate. Several medical and surgical therapeutic options aimed at reversing melanocyte loss and repigmenting the affected skin, are available for the treatment of the disease. Among these surgical approaches, cultured melanocyte transplantation represents to be the most effective procedure. Patient – is a 28-year-old female with stable segmental vitiligo, genital localization. Local treatment and a course of NB UVB therapy weren't effective. It was suggested to use cultured melanocyte transplantation for treatment. Two 3-mm punches were taken on the buttocks after pigmentation induction. In 3 weeks 28 million cells in platelets rich plasma were administered intradermally in vitiligo area. In 2 days course of local NB UVB has begun. The outcome in 3 month is 85% repigmentation. So traditional vitiligo therapy includes NB-UVB in combination with topical calcineurin inhibitors, topical corticosteroids, antioxidants. But if first-line traditional therapy isn't effective – it's time to use surgical techniques. Combination of cultured melanocyte transplantation and NB-UVB treatment have aimed to synergistically improve the efficacy and shorten the duration and total dosage of light therapy with earlier and greater total repigmentation.

## Vitiligo 50 shades of gray come to an end! The emergence of Pseudocatalase

M. A. Malek
Aman, Jordan

Vitiligo is a multifactorial disease with an unknown etiology, it has different clinical presentations. There are several treatment modalities for vitiligo. Research demonstrated that Pseudocatalase with climatotherapy or Pseudocatalase with NB-UVB are observed to be the best treatment options for vitiligo. According to extensive research and clinical observation, the use of activated Pseudocatalase treatment significantly improved patient's well being and quality of life.

 © 2017 John Wiley & Sons A/S. Published by John Wiley & Sons Ltd

## Audiological abnormality and vitiligo

G. Iannella[1], D. Didona[2], L. Guerra[3], B. Didona[2], G. Magliulo[1]
[1]Dipartimento 'Organi di Senso', Università 'Sapienza' di Roma; [2]I Divisione Dermatologica, Istituto Dermopatico dell'Immacolata-IRCCS; [3]Laboratorio di biologia cellulare e molecolare, Istituto Dermopatico dell'Immacolata-IRCCS

Vitiligo is a common chronic acquired disease of pigmentation. It is characterized by destruction of melanocytes in the skin, that causes hypopigmented and asymptomatic macules with sharply demarcated margins. Recent clinical and experimental studies found out a systemic destruction of melanocytes, especially in the mucous membranes, eyes, and the membranous labyrinth of the inner ear. Indeed, several authors reported an association between vitiligo, ocular abnormality, hearing loss and autoimmune diseases. The membranous labyrinth of the inner ear contains melanocytes. Furthermore, pigmented cells are present in the scala vestibuli. Although the specific functions of otic melanocytes are still unclear, it has been suggested that melanin had semi-conductive properties, responding to acoustic and electrical stimulations. Several authors studied the association between vitiligo and hearing loss, detected in a range from 4 to 20%. No differences in the hearing loss were described among all clinical types of vitiligo, although hypoacusis prevalence in vitiligo vulgaris patients was reported. Inner ear involvements are frequently detected in vitiligo. Sensorineural hearing loss is the most frequently reported symptom and should always be investigated. In our opinion, hearing loss should be evaluated by pure tone audiometry at vitiligo diagnosis and at least annually thereafter.

## T regulatory cells follow chemokine signals in vitiligo to co-localize with autoreactive T cells and control their numbers in the skin

J. Harris, K. Essien, J. Richmond, M. Rashighi
Dermatology, UMass Medical School, Worcester, MA United States

Vitiligo is an autoimmune disease of the skin that results from dysregulation of immune tolerance. Melanocyte-reactive T cells are present in healthy individuals, and previous studies reported that T regulatory cells (Tregs) play an important role in preventing initiation or controlling the progression of disease in the skin. We found that Tregs play a critical role is controlling vitiligo progression by limiting the number of CD8+ T cell effectors (Teffs) in the skin and must traffic to the epidermis to efficiently control disease. Tregs accumulate in the skin during the progression of vitiligo in a pattern that mirrors the infiltration of autoreactive Teffs. Confocal imaging of mouse lesional skin and immunohistochemistry of human lesional skin revealed that Tregs and Teffs directly interact within the skin during vitiligo, suggesting that cell contact is important for Treg suppression of disease and efficient Treg homing is important for their function. Like Teffs in vitiligo, Tregs express CXCR3 and localize to areas of chemokine expression within the skin. In summary, our data support previous studies that Tregs control Teffs in vitiligo, and suggest that they follow similar chemokine signals within the skin in order to colocalize with Teffs and suppress through cell contact-dependent mechanisms.

## Platelet rich plasma in the treatment of vitiligo; a comparative clinical study

M. El Hawary, E. Shaarawy, R. Aguizy
Dermatology Department, Cairo University, Giza, Egypt

Treatment of stable vitiligo is still a challenge. Autologous platelet rich plasma (PRP) contains various growth factors and cytokines with the potential of enhancing melanocyte proliferation and migration.
To evaluate the efficacy of PRP as a monotherapy and as an adjuvant to NB-UVB phototherapy, in the treatment of stable vitiligo.
30 patients with generalized bilateral non-segmental stable vitiligo were enrolled. In each patient, three vitiligo patches were randomly assigned to treatment by NB-UVB alone, PRP alone or by combination of NB-UVB and PRP. The duration of the study was 12 weeks. The time at which initial repigmentation occurred was determined for each group, and clinical assessment was done at the middle and end of the study by blinded investigator.
The combined group showed significantly better initial, mid and final responses ($P = 0.001$) than the other 2 groups, followed by the group received NB-UVB alone, while the group received PRP monotherapy showed the least response. As for other modalities, the location markedly influenced the response.
Intradermal injection of autologous PRP is a potentially effective therapy for stable vitiligo, with a promising NB-UVB enhancing effect.
NB-UVB, platelet rich plasma, vitiligo.

## First randomised controlled trial to compare laser (755 nm) and cryotherapy as depigmentation treatments for vitiligo

N. van Geel, E. Van Hoeylandt, H. Lapeere, B. Boone, E. Verhaeghe, S. De Schepper, R. Speeckaert

Remaining pigmented patches in extensive vitiligo can be removed by using topical bleaching creams, laser therapy or cryotherapy. We performed an observer-blinded randomized controlled trial comparing cryo and laser therapy as this was lacking in literature. Twenty-eight test regions (trunk and leg) in 4 vitiligo patient were exposed at random to either 1, 2 or 3 treatments (repeated with 1 month interval) of cryotherapy or 755 nm laser therapy respectively, while 1 test area remained untreated (control). Image J analyses demonstrated no significant difference in the induced depigmentation between cryo and laser treated areas. However, the percentage of depigmentation was significantly different according to the total number of treatments repeated on the same area ($P = 0.053$). Side effects were restricted to hyperpigmentation, which was significantly higher at the cryotherapy treated areas ($P = 0.023$) compared to laser treated areas. To our knowledge, this is the first randomised controlled trial comparing head to head depigmentation strategies intra- and interindividually. We could demonstrate that in general laser and cryotherapy are equally effective to induce depigmentations in vitiligo patients. The most effective depigmentation strategy was achieved after repeating the treatment 3 times on the same area with an interval of 1 month.

© 2017 John Wiley & Sons A/S. Published by John Wiley & Sons Ltd 

## UVA 1 laser in the treatment of vitiligo

G. Babino, E. Del Duca, M. Esposito, A. Giunta, R. Saraceno, L. Bianchi, S. Nisticò

Dermatology Department, Department of SysteMedicine, University of Rome Tor Vergata, Italy

Phototherapy, as monotherapy or in combination regimens, is considered the 'gold standard' treatment of vitiligo, being effective in 50–75% of patients with recent onset of the disease. Broadband and narrow-band UV phototherapy has been proposed as an effective therapeutic option and in this view, a monochromatic laser source emitting in the UVA1 range (355 nm) could achieve high energy density, lowering the exposition time and achieve clinical results in a short time period. The purpose of this study was to evaluate the clinical efficacy and safety of a monochromatic 355 nm ultraviolet (UVA) laser in the treatment of vitiligo.

Seventeen consecutive, unselected patients were enrolled in an open-label, prospective study and treated twice weekly for 8 weeks at a fixed dose of 80–140 J/cm$^2$. Follow-up was 12 weeks.

Clinical repigmentation was observed in 15/17 patients (88.23%), with limited side effects (mild post-treatment erythema and itching). Results were maintained during the 12 week phototherapy-free follow-up period.

UVA1 wavelengths penetrate the skin more deeply than UVB, reaching the deep dermis and the subcutis. Our report suggests that the UVA1 monochromatic laser 355 nm can represent a valid alternative therapeutic approach in the management of vitiligo.

## Combined treatment of narrow band UVB (NB UVB) phototherapy and oral polypodium leucotomos (PL) extract versus NB UVB phototherapy alone in the treatment of patients with vitiligo

A. Pacifico[1], M. Ardigò[2], P. Iacovelli[1], A. P. Vidolin[1], G. Leone[3]

[1]Phototherapy Unit, San Gallicano Dermatological Institute, Rome, Italy; [2]Clinical Dermatology, San Gallicano Dermatological Institute, Rome, Italy; [3]Photodermatology Rare Disease and Porphyrias Centre, San Gallicano Dermatological Institute, Rome, Italy

Vitiligo is a skin disease characterized by loss of normal pigmentation in the skin. Low levels of antioxidants enzymes have been demonstrated in the epidermis of vitiligo patients. Clinical trials with antioxidants as an adjunct to UVB phototherapy have shown encouraging results. The aim of our study was to investigate if the addition of the oral plant extract PL to NB UVB may improve NB UVB induced repigmentation. Fifty-seven patients with generalized vitiligo were enorolled in this randomized, prospective, placebo controlled study. Twenty-nine patients were randomly selected to receive combined therapy with 480 mg oral PL given twice daily while twenty-eight patients received NB UVB alone. All subjects were treated with NB UVB on a twice weekly schedule. Treatment was continued for up to six months. Our results demonstrated that the oral intake of an extract of PL to NB UVB improved the extent of repigmentation as well as increased the response rate compared with patients treated with NB UVB alone (47.8% versus 22%). In conclusion, our study suggests that oral suppplementation of Polypodium leucotomos to NB UVB might enhance the extent of repigmentation.

## Possibile benefit of tocotrienols in vitiligo treatment

S. Briganti[1], M. Gentile[2]

[1]Laboratory of Cutaneous Physiopathology and CIRM, San Gallicano Dermatologic Institute, Rome, Italy; [2]Logofarma S.r.L, Milan, Italy

Oxidative stress may be involved in the pathophysiology of vitiligo. Reactive oxygen species are capable of bleaching constitutional melanin and causing membrane lysis through lipid peroxidation. An imbalance in the antioxidant system and free radical-mediated damage has been reported as the initial pathogenetic events in melanocyte degeneration in vitiligo. Narrow band ultraviolet B phototherapy (NB-UVB) is the most widely and effective therapeutic option in vitiligo. Antioxidants are able to halt the depigmentation and to reduce the number of NB-UVB treatment sessions. Among antioxidants, tocotrienols have superior activity than tocopherols and they exert skin photo-protection by increasing NB-UVB MED and endogenous antioxidant capacity and by reducing UV-induced inflammation. Recently, highly purified tocototrienols have been associated with essential fatty acids (EFAs) and taurine in oral gel caps and topical monodosis treatment. Both EFAs and taurine can significantly improve tocotrienol photoprotection. EFAs are able to reduce UV-induced immunosuppression and production of pro-inflammatory eicosanoids and may increase tocotrienol bioavailability. Taurine is a strong antioxidant and anti-inflammatory molecules and can contribute to tocotrienol stability. Therefore, the combination of these molecules may be useful to reduce exposure to and side effects of photo-therapy, as well as promote survival and migration of melanocytes in vitiligo patients.

## Treating stable acral vitiligo by automated microneedling and topical 5-flurouracil solution

H. El-Fakahany, W. Medhat, H. Abdel-Raouf

Dermatology and Venereology, Faculty of Medicine, Al-Minya University, Egypt

Acral vitiligo is considered one of the most distressing dermatological disorders. Although surgical treatment is considered the treatment of choice in these patients after disease stability, yet it is time, cost and effort consuming. It also needs skillful experience. Microneedling was used with little success for treating acral vitiligo either solely or combined with excimer laser.

An automated microneedling device has been tried to induce re-pigemntation in stable acral vitiligo patches. 5-Flurouracil is used topically during the procedure.

Twenty patients with stable acral vitiligo were treated with an automated microneedling device in combination with topical 5-flurouracil solution. Each patient received sessions every 2 weeks.

Repigementation was noticed in sixteen patients after variable number of sessions.

Automated microneedling with topical 5-flurouracil solution may provide a simple, effective method for treating resistant and stable cases of acral vitiligo.

 © 2017 John Wiley & Sons A/S. Published by John Wiley & Sons Ltd

## Keratinocytes drive the progression of vitiligo through chemokine production and positioning of autoreactive T cells in the epidermis

J. Harris[1], J. Richmond[2], M. Youd[3], K. Essien[1], S. Currimbhoy[4], A. Luster[5], A. Pandya[4]

[1]Dermatology, UMass Medical School, Worcester, MA United States; [2]Dermatology, Genzyme/Sanofi; [3]UMass Medical School, Worcester, MA United States; [4]Dermatology, UT Southwestern Medical School, Dallas, TX United States; [5]Medicine, Massachusetts General Hospital, Boston, MA United States

The pathogenesis of vitiligo depends on IFN-γ and IFN-γ-induced chemokines to promote T cell recruitment to the epidermis where melanocytes reside. The skin is a complex organ, with a variety of resident cell types. We sought to better define the microenvironment and distinct cellular contributions during autoimmunity in vitiligo, and found that the epidermis is a chemokine-high niche in both a mouse model and human vitiligo. Analysis of chemokine expression in mouse skin revealed that CXCL9 and CXCL10 expression strongly correlate with disease activity, whereas CXCL10 alone correlates with severity, supporting them as potential biomarkers for following disease progression. Further studies in both our mouse model and human patients revealed that keratinocytes were the major chemokine-producers throughout the course of disease, and functional studies revealed that IFN-γ signaling in keratinocytes was critical for disease progression and proper autoreactive T cell homing to the epidermis. In contrast, epidermal immune cell populations including endogenous T cells, Langerhans cells, and γδ T cells were not required. These results have important clinical implications, as topical therapies that target IFN-γ signaling in keratinocytes could be safe and effective new treatments, and skin expression of these chemokines could be used to monitor disease activity and treatment responses.

## Autologous cell suspension grafting in segmental vitiligo and piebaldism: a randomized controlled trial on the recipient site preparation

A. Lommerts[1], A. Meesters[2], L. Komen[2], M. Bekkenk[1], M. de Rie[2], R. Luiten[1], A. Wolkerstorfer[1]

[1]Netherlands Institute for Pigment Disorders, Academic Medical Center, Amsterdam, The Netherlands; [2]Dermatology, Academic Medical Center, Amsterdam, The Netherlands

Autologous non-cultured cell suspension transplantation is an effective treatment for repigmentation in segmental vitiligo and piebaldism. The optimal depth of ablation of the recipient-site before cell suspension transplantation is unknown. The objective of this study was to assess the efficacy and safety of less invasive recipient-site preparations.

In a randomized, observer-blinded, within subject, controlled trial we compared different recipient-site preparations before cell suspension transplantation in segmental vitiligo and piebaldism. In each patient, we randomly allocated four $CO_2$-laser recipient-site preparations (i.e. standard, superficial, fractional and control site) to four depigmented lesions. After six months we assessed repigmentation and side-effects.

We included 10 patients with vitiligo (n = 3) and piebaldism (n = 7). Compared to the control site we found significant higher repigmentation in standard (median 68.7%, P = 0.011) and superficial (median 58.3%, P = 0.007) full surface $CO_2$-laser ablation, but not for fractional (median 0.0%, P = 0.144) $CO_2$-laser ablation.

Superficial full surface $CO_2$-laser ablation is an effective recipient-site preparation before cell suspension transplantation in segmental vitiligo and piebaldism. Fractional $CO_2$-laser recipient-site preparation with the settings used in this study was not effective.

## Chemical peeling with trichloroacetic acid 25% for the treatment of vitiligo: a randomized controlled clinical trial

H. Mashaly, M. El Mofty, W. Mostafa, R. Hegazy, M. El Hawary

Dermatology, Faculty of Medicine, Cairo University, Giza, Egypt

Trauma in vitiligo is a double edged weapon, where indeed köebnerization (KP) remains the most alarming outcome, yet several studies succeeded to demonstrate occurrence of pigmentation after trauma to vitiliginous skin.

Evaluate combining chemical peeling using TCA 25% with NB-UVB phototherapy in treatment of non-segmental vitiligo (NSV).

Patients and methods15 patients with bilateral NSV, and grade '0' KP were included in the current randomized, controlled, single-blinded clinical trial. They underwent chemical peeling using TCA 25% for one side (Side A), and after resolution of inflammation both sides were exposed to NB-UVB phototherapy, thrice weekly for 16 weeks (48 sessions). VASI scoring and photography of both sides were performed.

Side A showed a significantly earlier initial response (P = 0.003) and a significantly lower VASI at week 8 (24 sessions) (P = 0.021). However, no significant difference was detected between both sides regarding the final clinical response (48 sessions). Facial lesions showed best response with repigmentation noted in side A even before UV exposure.

This novel therapeutic protocol combining TCA 25% with NB-UVB for treatment of stable NSV appears to be a promising tool with early initial response.

## A case series report of association between bilateral segmental vitiligo and lichen striatus: an expression of mosaicism?

C. S. de Castro[1], S. L. Dumas[2]

[1]Dermatology, Pontifícia Universidade Católica do Paraná, Prado Velho, Brazil; [2]Medicine, Pontifícia Universidade Católica do Paraná, Prado Velho, Brazil

Segmental vitiligo is characterized by one or more achromic maculae generally involving a unilateral body segment, being also recognized by its early age of onset. There are evidences that immune-mediated cellular responses including CD8+ T lymphocytes are involved in the early stages of this vitiligo subtype, moreover, clinical similarity with cases of mosaic diseases involving melanocytes supports the hypothesis that cutaneous mosaicism may be involved in its pathogenesis. Lichen striatus is a linear, benign, self-limited unilateral dermatosis that generally affects children, characterized by papules arranged in bands, usually following the Blaschko lines, the disease most often affects the limbs; immunohistochemical findings support a cellular immune response. This case series study reports the association between bilateral segmental vitiligo and lichen striatus in three female children. Two of them, have monozygotic twins with no signs of vitiligo or lichen striatus. This, probably is the first small case series report containing the association between bilateral segmental vitiligo and lichen striatus, two diseases with strong evidence of an autoimmune attack against a mosaic area. The association presented here is important not only because of the similarity of the mosaic origin, but also because of the similar etiopathology mechanism in the same patient.

© 2017 John Wiley & Sons A/S. Published by John Wiley & Sons Ltd 

## Valsartan/hydrochlorothiazide compound drug-induced photo-leuko melanoderma developed in vitiligo: comparative immuno-hisitochemical study with special reference to IL17A

I. Katayama, N. Shimada, E. Ono, A. Takahashi, A. Tanaka, A. Tanemura

Dermatology, Osaka University, Suita, Japan

A 67 male developed aberrant leukomelanoderma on the face and bilateral forearms after improvement of 3 years-preceded vitiligo by local vitamin D3 ointment and sun exposure. On consultation, he stated to have taken daily use of valsartan/hydrochlorothiazide compound drug for the treatment of hypertension 2 years before and noticed asymptomatic erythema on the sun-exposed lesions followed by irregular hypo- and hyperpigmented macules on the face. According to a diagnosis of photoleukomelanoderma induced by hydrochlorothiazide in the compound drug, we instructed the patient to discontinue the compound drug and avoid sun exposure. Thereafter, the leukomelanoderma lesions were improved without any treatment, whereas the preceded vitiligo lesions remained unchanged. The photochallenge test with the compound drug was positive. Although there have been some case reports of leukomelanoderma phototoxic to hydrochlorothiazide, this is the first patient who exhibited both photo-leuko-melanoderma and non-segmental vitiligo vulgaris. We herein present a rare vitiligo vulgaris case combined with photoleukomelanoderma and discuss the differences in the histological and immunohistochemical findings between vitiligo and leukomelanoderma lesions.

## Camouflage an underutilised option in the management of pigmentary disorders

M. Gupta

Dermatology, The Skin Hospital, Sydney, Australia

Pigmentary disorders have a high impact on the QoL for the affected person and sometimes for other family members. Most pigmentary disorders are slow to respond to medical treatments, which can be a source of frustration for both the patient and the treating physician. Patients referred to a tertiary referral centre come with high hopes and are disappointed at the slow efficacy of treatment(s) offered to them. For some patients with certain Fitzpatrick colour types, medical treatment may not be successful. Other patients may have a pressing social engagement where they want to look presentable. In such scenarios, camouflage may be a suitable and gratifying option. I would like to discuss the experience of a nurse led 'camouflage Clinic' being run under the umbrella of a 'Pigmentary Disorders Clinic' under a doctor's supervision.

## The presence of depigmented rim around the successful grafting of vitiligo lesions: a case series

T. Anbar[1], A. Alissa[2], M. Anbar[3]

[1]Department of Dermatology and Andrology, Faculty of Medicine, AL-Minya University, Egypt; [2]Fayoum University Hospital, Giza, Egypt; [3]Department of Dermatology, Dermatology National Center for Vitiligo and Psoriasis, Riyadh, Kingdom of Saudi Arabia

Repigmentation and colour matching are two important goals in the treatment of vitiligo. In the past few decades surgical treatments have gained much ground in the management of stable vitiligo. In this work we report a case series of 12 stable vitiligo cases after punch grafting, epidermal grafting and melanocyte suspension grafting. These cases developed repigmentation in all the treated lesions except for a rim at the periphery, which diminished the cosmetic outcome. The cause behind this observation is not yet clear.

## Two cases of vitiligo like depigmentation (VLD) associated with malignant melanoma MM: could distinctive clinical and histological characters be isolated?

L. Benzekri, Y. Gauthier

Dermatology, Mohammed V University, Rabat, Morocco

The VLD usually appears after metastasis in most cases of malignant melanoma (MM) patients. Its occurence is believed to be a positive prognostic factor.

Aim of the studyTo distinguish a proper clinical and histological spectrum to VLD.

A clinical analysis of two cases was performed considering the appearance, the distribution, the onset date of VDL lesions with regard to MM detection and the survival. Histologically, the sections were immunostained with HMB45, CD8 and E-Cadherin antibodies.

The lesions were hypomelanotic, located preferentially on the forehead, trunk and hands, occured in metastazing MM with a survival ranging from one month to more than 15 months. Histologically, an incomplete melanocyte loss with an unusual protrusion of the melanocytes into the dermis, a low expression of E-Cadherin and an important CD8+ T lymphocyte infiltrate were found.

VLD have a different clinical and histological pattern than vitiligo: variable asymmetric distribution, limited extent and lack of progression and histologically, a spongiosis, a melanocyte protrusion into the dermis and a greater density of CD8+ lymphocytes in the dermis and epidermis.

Further studies are needed to differentiate VLD from vitiligo by relying on the clinical and histological clues.

## New trends in Ratokderm methodology for treatment of vitiligo

F. Marina, C. Anna

Centro Dermatologico Ratokderm, Clinica Cimarosa, Milan, Italy

The combination of therapies aims at improving the treatment effect and or to reduce side effects of the single components. In this study the authors for the first time compare the efficacy of a particular source of ultraviolet ray of light with a generator capable of producing a mono and multipuntiform UVB broad band irradiation with a 200 mW/cm squared fluency which is called Ratokderm Equipment used alone for 20 years for selective treatment of Vitiligo and the new combination in the last two years of this kind of Microphototherapy with local application of low percent of psoralen solution or Piperina cream to reach remission of Vitiligo more efficently with a distribution and thus reduction of the various kinds of risk categories.

## Epidermal skin grafting in vitiligo

A. Janowska, V. Dini, T. Oranges, S. Panduri, M. Romanelli

Dermatology, University of Pisa, Italy

Vitiligo is a multifactorial acquired dermatosis characterised by achromic or hypochromic macules and by the absence of

 © 2017 John Wiley & Sons A/S. Published by John Wiley & Sons Ltd

functioning melanocytes. Treatment depends on the extent of the affected areas and on disease activity. Surgical techniques have proven to be effective in stable cases but can be time-consuming and, in some cases, aesthetically unsatisfying or painful for the patients. The aim of the study was to assess the clinical safety and effectiveness of a new automatic epidermal skin harvesting device in patients with stable localised vitiligo over a minimum 12-month period. This new system is a commercially available epidermal skin harvesting system that can be used without local anaesthesia or other pre-treatments and has been shown to have low rates of donor site morbidity. Epidermal skin grafts can used in patients with acute and hard to heal chronic wounds, burns and stable vitiligo. The use of advanced therapies may improve the quality of life, have cost benefits and accelerate re-pigmentation of patients with vitiligo. In our study, this system was seen to be a safe and efficacious means of harvesting epidermal micrografts containing melanocytes for use in patients with stable vitiligo unresponsive to standard therapies.

## Retrospective portrait of the patient with vitiligo in the Uzbek population

E. Karaev

Dermatology, Republican Specialized Scientific and Practical Medical Center of Dermatology and Venereology, Tashkent, Uzbekistan

Retrospective analysis of the clinical-amnestic data of the patients with vitiligo.

MaterialsMaterials included medical histories and ambulatory cards of the patients with vitiligo referred for the medical aid in 2016.

There were studied medical histories of 115 patients with vitiligo at the age of 6 to 43 years, of which males were 36 and females 79. Localized form of vitiligo occurred in 91 patients. Subjectively 72 patients complained on pruritus in the focuses of lesions, in 43 patients the disease was not accompanied by subjective signs. 82 patients noted appearance of the pathological process after previously having nervous stress, 27 patients had no causes for disease onset, 6 patients showed role of traumatic effect on the skin. 16 noted the worsened inherited anamnesis with regard to this pathology. 68 patients suffered from vitiligo during a year, 42 patients – from 1 to 5 years and in 5 patients this dermatosis lasted more than 5 years. 44 referred for the medical aid for the first time, 71 patients are under the observation of dermatovenerologists. Thus, vitiligo has been found mostly often among the females of young age, expresses in localized form and is accompanied with pruritus.

## Efficacy of microneedling with topical vitamin C in treatment of melasma

E. Nada, W. Abdel-Magid, E. Lsmail

Dermatology, Venereology, and Andrology, Sohag University, Dokki, Egypt

Melasma is a common dyschromia that often motivates the search for dermatological care as it results in cosmetic disfigurement that impairs the patient's quality of life.

To evaluate the effect of microneedling with addition of topical vitamin c in treatment of melasma.

This is a prospective study that included 34 adult females with 4 dropouts. All had epidermal melasma. The thirty cases who completed the study received 6 sessions of microneedling with topical vitamin C 20% at 2 week interval. All came for follow up after 3 months of the last session. Patients were evaluated by digital photographs taken at each visit and Melasma Area Severity Index (MASI scoring).

The percentage improvement of the studied cases showed gradual increase from the first session (mean = 0) to the last session (mean = 36.87 ± 19.85) and this was highly significant (P value $<$0.0001). After 3 months of the last session, 5 cases (16.67%) showed recurrence and 25 cases (83.33%) showed no recurrence. There were no major adverse events observed.

On the basis of these results, microneedling with topical vitamin C is a promising treatment option for epidermal melasma especially in fair skinned patients.

## Cerebrosides role and content of the skin in patients with vitiligo

B. Saatov

Dermatology, Republican Specialized Research-Practical Medical Center of Dermatology and Venerology, Ministry of Health of the Republic of Uzbekistan

Cerebrosides are the major constituents of the skin lipids, where they are required for lamellar body formation of the keratic layer of the epidermis and are the main barrier for water permeability in the skin.

Comparative study of the contents of cerebrosides in the skin of healthy persons and of patients with vitiligo.

This investigation was performed on the skin samples of 12 healthy persons and 25 patients of vitiligo.

According to our data in the content of the skin of healthy persons the number of cerebrosides accounted for, on the average, 283.5 ± 12.8 μg, and in the sites of the skin without lesions – 265.5 ± 11.9 μg of the cerebroside per 1 g of tissue.

1. The contents of cerebrosides was studied, for the first time, in the human skin both in norm and vitiligo. 2. It was established the rather big lowering of the contents of cerebrosides both in the depigmented and not damaged skin sites in the patients with vitiligo.

## Disturbance of melanogenesis and melanosome transfer on the leukoderma lesion in extra-mammary Pagets disease

A. Tanemura[1], A. Tanaka[1], S. F. Yang[1], M.-W. Kaneda[1], I. Katayama[1], N. Oiso[2]

[1]Dermatology, Osaka University, Suita, Japan, Graduate School of Medicine; [2]Dermatology, Kinki University, Faculty of Medicine, Higashiosaka, Japan

We frequently encounter characteristic color variation including hypopigmentation, hyperpigmentation, and erythema in extra-mammary Paget's disease (EMPD) lesions. Owing to unclear hypopigmentation, the lesional border of EMPD can be poorly defined and it is likely insufficient to perform its complete resection. Although the existence of Toker's cells and lack of lesional bFGF production have been reported to cause hypopigmentation inside of EMPD lesions, exact mechanisms of hypopigmentation in EMPD is not fully explored. We experienced three EMPD patients with obviously hypopigmented EMPD macules and histopathologically confirmed a reduced number of melanocytes on the hypopigmented macules and their loss on the erythematous plaques or nodules. An ultrastructual analysis on the hypopigmented lesions revealed disturbance of melanosome maturation and melanosome transfer to the adherent Pagets' cell on the basal layer. No Paget's cells even adhered to remaining

© 2017 John Wiley & Sons A/S. Published by John Wiley & Sons Ltd 

melanocytes with dendrites contained matured melanosome and few number of matured melanosome complex were observed in basal keratinocytes. In the present study, we hypothesize that severe disturbance of not only melanogenesis but also melanosome transfer to surrounding Paget's cells and basal keratinocytes may cause characteristic hypopigmentation in EMPD. Future bioanalysis would reveal molecular mechanisms for hypopigmentation in EMPD.

## Use of a new kit for epidermal cell suspension grafting, a multicentric placebo controlled study: preliminary results

A. Pacifico[1], A. P. Vidolin[1], G. Leone[2]

[1]Phototherapy Unit, San Gallicano Dermatological Institute; [2]Photodermatology Rare Disease and Porphyrias Centre, San Gallicano Dermatological Institute, Rome, Italy

Epidermal cell suspension grafting is a technique introduced for surgical treatment of segmental vitiligo and of limited and stable non segmental lesions. The possibility to perform the technique using commercial kits further simplifies the procedure. Six patients were enrolled in this multicentric study: one had segmental vitiligo and 5 non segmental vitiligo. Stable lesions were symmetric and were selected to receive active or placebo. In the case of segmental lesion the area was divided in two half, one was treated with active and the other one with placebo. Dermabrasion of the receiving site was obtained using $CO_2$ laser; skin samples from the donor site were shaved on the buttocks by means of a dermatome, then immersed in a trypsin solution for 20 min; the sample was then processed in order to obtain an epidermal cells solution added with hyaluronic acid 2%. The selected areas were covered with the solution. Patients were evaluated every 12 weeks up to 1 year. Preliminary data demonstrated that the best results were obtained in patients with segmental vitiligo. The epidermal cell suspension grafting may be the procedure of choice to treat cases that do not respond to phototherapy and in particular to treat segmental vitiligo.

## An atypical case of vitiligo

S. Calvieri, E. Moliterni, S. Giustini

Department of Dermatology, Sapienza University of Rome, Italy

A 60 year-old female patient came to our attention for suspected vitiligo. Her skin type was III, according to Fitzpatrick scale.
Skin examination revealed on axillary cables, forearms, back of hands, bilaterally, and on abdominal mesogastrium, multiple achromic patches. Such lesions were irregularly oval shaped, with one end slightly pointed, and had maximum diameter between 1 cm and 5 cm. The spots had clean limits and rough edges.
The patient reported that the onset of the clinical picture was in summer. No other skin lesions were observed. The anamnestic data revealed that the patient is suffering from a rare disease: lymphangioleiomyomatosis (LAM).

 © 2017 John Wiley & Sons A/S. Published by John Wiley & Sons Ltd
