# Supplementary material for: Therapeutic efficacy of cell-based therapy in vitiligo: a research letter systematically reviewed using meta-analysis
Source: Arch Dermatol Res. 2024 May 22;316(5):198. doi: 10.1007/s00403-024-02920-6 (PMC11111487; doi:10.1007/s00403-024-02920-6)
Supplement: Supplementary file 1 — Supplementary file1 (ZIP 24195 KB) [file 403_2024_2920_MOESM1_ESM.zip › Studies were included/Pandya 2005.pdf]

Original Article

A study of autologous melanocyte transfer in treatment of stable vitiligo

Vishvabhavan Pandya, Kirti S. Parmar, Bela J. Shah, F. E. Bilimoria
Department of Dermatology, Civil Hospital and BJ Medical College, Ahmedabad, India.

Address for correspondence: Dr. Vishvabhavan Pandya, S/o S K Pandya, #337, Laxmipura ward, Near Kadam ka kua, Post & Distt Sagar, Madhya Pradesh, India – 470002. E-mail: vbpandya@rediffmail.com

ABSTRACT

Background: Replenishing melanocytes selectively in vitiliginous macules by autologous melanocytes is a promising treatment. With expertise in culturing melanocytes, it has now become possible to treat larger recipient areas with smaller skin samples. **Aim:** To study the extent of repigmentation after autologous melanocyte transplantation in patients with stable vitiligo. **Methods:** The melanocytes were harvested as an autologous melanocyte rich cell suspension from a donor split thickness graft. Melanocyte culture was performed in selected cases where the melanocyte cell count was insufficient to meet the requirement of the recipient area. These cells were then transplanted to the recipient area that had been superficially dermabraded. **Results:** An excellent response was seen in 52.17% cases with the autologous melanocyte rich cell suspension (AMRCS) technique and in 50% with the melanocyte culture (MC) technique. **Conclusion:** Autologous melanocyte transplantation can be an effective form of surgical treatment in stable but recalcitrant lesions of vitiligo.

Key Words: Autologous melanocyte transplantation, Melanocyte culture, Melanocyte rich cell suspension

INTRODUCTION

There are many modalities for the treatment of vitiligo but there is still a need for a treatment that is promptly effective. Replenishing melanocytes selectively within vitiliginous macules by autologous melanocytes is a promising treatment.[1] Moreover, with expertise in culturing melanocytes, it has now become possible to treat larger recipient areas with smaller skin samples.[1] There are a few techniques for seeding autologous melanocytes. To study the extent of repigmentation after autologous melanocyte transplantation in patients with stable vitiligo, we employed two methods, melanocyte rich cell suspension and cultured melanocytes for replenishing melanocytes.

METHODS

Patients with stable vitiligo were selected for the study, the criteria for stability being no increase in the size of the lesion for at least 2 years and no new lesions since the last 2 years. Exclusion criteria were patients with active disease, infection at the recipient site, age below 8 yrs, evidence of Köebner response in the past, bleeding diathesis, keloidal tendencies and poor general condition. The study was conducted at Department of Dermatology, Civil Hospital, BJ Medical College, Ahmedabad in a span of 3 years. Patients were chosen randomly after they met the selection criteria. Out of the 27 patients recruited, a single vitiliginous lesion was taken as a control in 20 cases. The control patch

How to cite this article: Pandya V, Parmar KS, Shah BJ, Bilimoria FE. A study of autologous melanocyte transfer in treatment of stable vitiligo. Indian J Dermatol Venereol Leprol 2005;71:393-7.

Received: November, 2004. **Accepted:** April, 2005. **Source of Support:** Nil. **Conflict of interest:** None declared.

was only superficially dermabraded and subsequently dressed.

Pre-operative work-up consisted of informed consent, a clinical photograph, screening for HIV and *Hepatitis B virus* infection and charting of the area to be grafted. A prophylactic course of an antibiotic, usually oral erythromycin 500 mg thrice daily, was started 1 day before the procedure and alprazolam 0.5 mg was administered orally on the previous night.

Two techniques were employed, the autologous melanocyte rich cell suspension (non-cultured) technique[1-3] and the cultured melanocyte technique.[4-6] Both these techniques share a common principle of selective replenishment of melanocytes at the recipient stable vitiligo macules. The culture technique was used when the harvested melanocytes were less than required, when counted with Neubauer's chamber.

Donor site

About one-tenth the size of the recipient area was selected as the donor site, usually on non-cosmetically important sites like the thighs, buttocks or waist. It was cleaned with povidone iodine (Betadine©) and 70% ethanol, and draped. The site was anesthetized with 1% lidocaine (Xylocaine©) infiltrated in the subcutis. The skin was stretched and a very superficial sample was obtained with Silver's skin grafting knife or a sterile razor blade on a straight hemostat forceps. The superficial wound was then dressed with Sofra-tulle©.

Laboratory procedure for cell separation[3]

The skin graft was immediately transferred to 6 ml of 0.25% trypsin-EDTA solution in a petri dish. This sample of skin graft inside the petri dish was turned back and forth to ensure complete contact with trypsin-EDTA solution. This mixture of skin sample with trypsin-EDTA solution was incubated at 37°C and 5% CO_2 for 50 minutes. Three ml of trypsin inhibitor (soya protein solution; Sigma Chemicals, St. Louis, Mo, USA) was then added to neutralize the action of trypsin. The epidermis was separated from the dermis with the help of a pair of forceps. The epidermis was cut into tiny pieces and transferred to a 15 ml test tube with a pipette. Three ml of MK medium (Melanocyte-keratinocyte culture medium; Sigma Chemicals, St. Louis, Mo, USA) was added to it. The contents of MK Medium were DMEM (Dulbecco's Modified Eagle's Medium), insulin (25 μg), adenine (0.38 nmol), basic fibroblast growth factor (b-FGF),[7-10] human albumin [or fetal calf serum (10%)], hydrocortisone (0.1 μg/ml), streptomycin (0.1 mg/ml) and penicillin (100 u/ml). All materials used were tissue culture grade (endotoxin level <0.1EU/ml chrome-LAL test). The test tube was then centrifuged at 2000 rpm for 10-15 minutes. A pellet was formed at the bottom. The supernatant was discarded and the pellet, containing cells from the stratum basale and lower half of the stratum spinosum that were rich in melanocytes, was taken. The melanocytes were stained with trypan blue and counted simultaneously with Neubauer's chamber under the light microscope; this would also identify whether the suspension was viable as the dead cells pick up blue stain. Around 1000-1500 cells/mm^2 of recipient area were required;[8] when the separated cells were less than 1000 cells/mm^2 they were subjected to cell culture.

Recipient site

It was cleaned, painted and draped with Betadine©, 70% ethanol and washed thoroughly with normal saline. It was anesthetized with use of EMLA (Prilox©) or 1% lignocaine.

Transplantation[1]

The recipient area was abraded with a high-speed motor dermabrader fitted with a diamond fraises wheel until tiny pinpoint bleeding spots were seen, which implied that the dermoepidermal junction had been reached. The denuded area was covered with a saline moistened gauze piece. The suspension was poured evenly from the pipette to the denuded surface, which was then covered with a collagen dressing (Kollagen© 5x5). This was then covered by a small gauze piece moistened in MK medium. The dressing was kept in place by a Tegaderm© dressing.

The patient was allowed to go home 30 minutes later. The dressing was removed at their first follow-up visit after a week to the hospital.

Preparation of autologous culture of melanocytes[4]

The graft was harvested in a similar manner as above. The tissue was transferred to a Petri dish containing 6

ml of 0.25% trypsin-EDTA solution with the help of a pair of forceps and incubated for 50-60 min at 37°C and 5% CO_2. Later, a few milliliters of trypsin inhibitor were added to neutralize the action of trypsin. The epidermis was separated and cut into tiny pieces by the help of a small surgical knife and the entire content was transferred into a centrifuge tube and 3 ml of MK culture medium was added. The tube was then centrifuged at 2000 rpm for 15 minutes. The supernatant was removed and the pellet was taken out with a pipette. It was again re-suspended in 1 ml of MK medium and 0.5 ml of the solution inoculated in a T-25 tissue culture flask. Another 5 ml of MK medium was added to the flask, which was kept in the incubator for incubation with 5% CO_2 and temperature of 37°C for a period of maximum of 3 weeks.[4] The medium in the flask was changed daily carefully and incubation continued. Separation of melanocytes from the flask of melanocytes was done with trypsin EDTA solution after the end of 2-3 weeks. Trypsin-EDTA solution was again replaced with MK medium for a few minutes. This solution was further incubated for 5 minutes and then another 5 ml of MK medium was added. The entire content was collected in a pipette and poured into a centrifuge tube. It was again centrifuged at 2000 rpm for 10-15 minutes. The supernatant was discarded and the contents were re-suspended in 0.5 ml of the culture medium. A microscopic preparation of this was made and stained with Trypan Blue to check the viability of melanocytes. Viable melanocytes did not take up the stain and dead cells appeared blue in color [Figure 1 and 2]. The melanocytes were counted and the desired number of cells in the form of a certain quantity of suspension (cells/ml) was taken as per the area of recipient site. Around 1000-1500 melanocytes/mm^2 were spread uniformly over the recipient surface after superficial dermabrasion. However, in certain areas like the areola, the number of melanocytes implanted was more than 2000 cells/mm^2.

Erythromycin and nimesulide 100 mg daily (if required) were continued for 7 days following the transplantation. The dressings at the donor and recipient site were removed on day 7. A light dressing was applied on the recipient area for the next 7 days, if found necessary. The patient was called after 1 month, 3 months and 6 months to assess the extent of repigmentation. Photographs were taken and the observations were tabulated. The response was graded according to the extent of repigmentation in transplanted areas as follows: excellent, >90% repigmentation; good, 65% to 89% repigmentation; fair, 25% to 64% repigmentation and poor, below 25% repigmentation.

RESULTS

Of the total 27 patients with vitiligo, 12 (44.45%) patients were male and 15 (55.55%) female. Majority (16, 59%) of the patients were in the age group of 21 to 30 years. Vitiligo was of the vulgaris type in 25 and segmental type in 2 patients. Table 1 shows details of duration of disease in our patients.

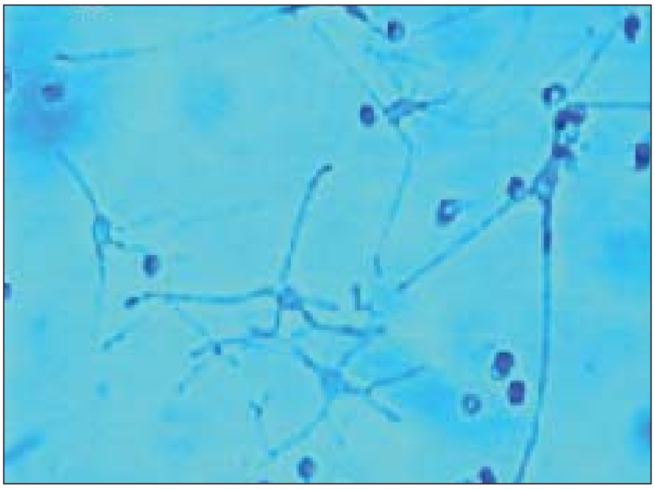

Figure 1: Melanocytes in culture (day-3): Note the developing dendrites (blue filter used in the surface phase contrast microscopy)

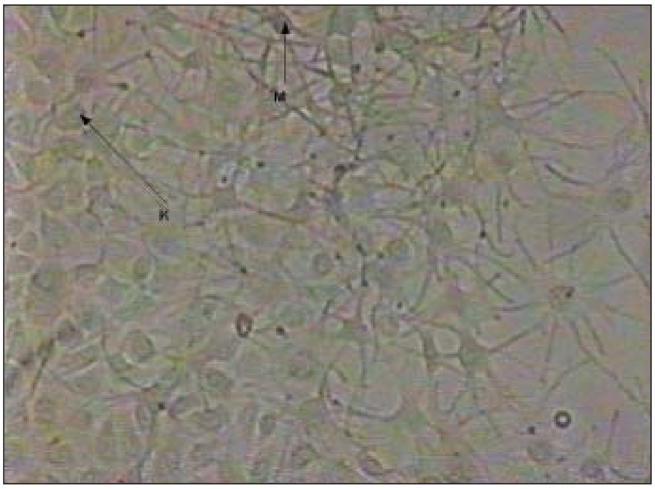

Figure 2: Melanocytes in established culture (surface phase contrast microscopy)

At first follow-up, soon after the removal of dressing the treated area appeared bright pink. Repigmentation was first seen after 2-3 weeks after the procedure and was completed in up to 6 months. It was almost of a uniform color. In a few cases, there was initial hyperpigmentation that subsequently faded to match the normal skin color.[1] This hyperpigmentation may be caused by hyperactivity of transplanted cells from the culture or oversupply of growth factors and melanogenic peptides such as â-FGF during wound healing.[1,7] In most patients we observed pigmentary islands irrespective of leukotrichia or paucity of hair follicles.

Table 1: Duration of vitiligo

Duration in yrs	Patients			
	Male	Female	Total	Percent
Less than 5	1	4	5	18.51%
5-7	2	5	7	25.9%
8-10	4	4	8	29.62%
More than 10	5	2	7	25.9%
Total	12	15	27	100%

Mean ± SD = 8 ± 3.74

Table 2: Results in different regions

	Total transplants				Response (in patients)			
Area	Sites		Patients		E	G	F	P
	N	%	N	%				
Hands	5	9.8	2	7.4	-	-	-	2
Forearm	1	1.9	1	3.7	1	-	-	-
Elbow	1	1.9	1	3.7	-	-	-	1
Feet	23	45.1	10	37	4	2	0	2
Legs	15	29.4	8	30	5	3	2	-
Knee	2	3.9	1	3.7	1	-	-	-
Thigh	1	1.9	1	3.7	-	-	1	-
Trunk	1	1.9	1	3.7	-	1	-	-
Face	2	3.9	2	7.4	2	-	-	-
Total	51	100	27	100	13	6	3	5

(E=excellent, G=good, F=fair, P=poor, n=number of subjects)

Table 3: Correlation of response with the number of implanted cells

Cells/ mm^2	Excellent	Good	Fair	Poor
Below 500	0	0	0	1
501-1000	0	0	3	2
1001-1500	6	5	0	2
1501-2000	6	0	0	0
Above 2000	2	0	0	0

Table 4: AMRCS vs Melanocyte culture

Technique	E	G	F	P
**Melanocyte culture** *(n=4)*	50%	0%	0%	50%
**AMRCS** *(n=23)*	52.20%	18.50%	11%	11%

(E=excellent, G=good, F=fair, P=poor, n=number of subjects)

The optimum time for successful culture was 1-3 weeks. At the end of 3 weeks, the cell count was raised 50-100 folds after primary culture and subculture. The melanocyte content of these cultures was 95%.

Fifty-one sites in 27 patients were chosen for autologous melanocyte transplantation. The most common sites were the feet (45.1%), legs (29.4%), hands (9.8%), knees (3.9%) and face (3.9%) [Table 2]. The results were most favorable on the legs, feet, face and forearms, and poor on the elbows and acral areas of the hand. Repigmentation was not observed in any of the control patches.

The repigmentation was best when there were 1500 cells/mm^2 or more. In 27 patients, the cells were cultured because of the large recipient area or because the number of cells separated was less than 1000 cells/ sq cm of recipient area [Table 3].

An excellent response was seen in 12 (52.2%) and 2 (50.0%) patients with AMRCS and melanocyte culture respectively [Table 4].

Some minor complications were observed. Strikingly, there was no milia formation or scarring. Two (7.4%) patients had infection at the donor area and three (11.1%) developed infection at the recipient surface. Only one patient developed Köebner response at the donor area.

DISCUSSION

Both, the autologous melanocyte rich cell suspension (non-cultured) technique and the cultured melanocyte technique, are essentially based on the principle of seeding of melanocytes. Melanocyte culture is a state of the art procedure that requires expertise.

Superficial split thickness thin skin grafts (STG) yielded more melanocytes than did punch biopsy samples chiefly because of the larger size of skin harvested and because separation of cells was easier with thinner samples of skin. Moreover, the STG donor sites healed better. The average number of cells kept was 1000-1500 cells/mm^2 irrespective of the technique employed.

The outcome was optimal when more than 1000 cells form AMRCS/mm^2 were kept but was proportionately less when the number was less. The autologous melanocyte rich cell suspension (AMRCS) technique of autologous melanocyte transfer was equally effective for smaller lesions but for a larger recipient area, melanocyte culture (MC) was found more suitable, although the technique of AMRCS was simple and efficient.[11]

Overall, an excellent response was seen in 14 patients (51.8%), a good response in 5 (18.5%), and a fair response in 3 (11.1%). Five patients (18.5%) had a poor response. Response to both techniques was comparable, with an excellent response in 52.2% cases of the AMRCS technique and 50% with melanocyte culture. Repigmentation was generally first observed at 2-3 weeks and was complete by 6 months. It was seen as multiple islands of pigmentation that later coalesced to a uniform color. The location of the recipient site was the major determinant of the outcome; acral parts including the dorsal aspects of the hands and feet, and skin over the joints were less responsive, as 2 patients each with lesions on the hands and feet, and 1 patient with lesions on the elbow had a poor response.[1]

The response was comparable to studies done by Mulekar.[9] In a study in 27 patients, Lontz et al reported excellent response in 40.7%, good response in 7.4%, and moderate response in 51.8%.[1] Lontz et al emphasize that the anatomical location is the major factor that determines the response.[1] The fingers, knuckles and elbows were the most difficult areas to repigment, in part because of the relative uncertainty in controlling the depth of dermabrasion of such heavily cornified areas and also because of the high mobility of the skin covering these joints. Olsson and Juhlin have also made a similar observation.[4]

Both these techniques had some minor complications. Infection at the donor area was seen in 7.4% of patients and at the recipient site in 11.1%. Infection occurred probably because patients did not comply with instructions to avoid unnecessary movements of the neighboring joint and subsequently the dressing slipped. Only 1 patient developed Koebner response at the donor site. None of our patients had milia formation or scarring.

In patients with stable vitiligo, autologous melanocyte transfer is a simple and effective technique to produce homogeneous pigmentation quickly. It has an advantage over conventional split thickness grafting as it requires very little donor skin (usually only one tenth of the recipient site).[9] Patients were generally satisfied with the results as the quality of repigmentation was superior. Further large scale patient studies are required, especially with melanocyte culture methods, to confirm the efficacy of autologous melanocyte transfer techniques.

REFERENCES

1. Lontz W, Olsson MJ, Moellmann G, Lerner AB. Pigment cell transplantation for the treatment of vitiligo: a progress report. J Am Acad Dermatol 1994;30:591-7.
2. Gauthier Y, Surleve-Brazeille JE. Autologous grafting with non-cultured melanocytes: a simplified method for treatment of depigmentary lesions. J Am Acad Dermatol 1992;26:191-4.
3. Olsson MJ, Juhlin L. Leukoderma treated by transplantation of basal cell layer enriched suspension. Br J Dermatol 1998;138:644-8.
4. Olsson MJ, Juhlin L. Repigmentation of vitiligo by transplantation of cultured autologous melanocytes. Acta Derm Venereal (Stockholm) 1973;73:49-51.
5. Lerner AB, Halaban R, Leffell D. Melanocyte culture in patients with disorders of hypopigmentation. In: Programme abstracts of 14th International Pigment Cell Conference. Japan: The International Pigment Cell Soc; 1990. p. 100.
6. Chen YF, Chang JS, Yang PY, Hung CM, Huang MH, Hu DN. Transplantation of cultured autologous pure melanocytes after laser-abrasion for the treatment of segmental vitiligo. J Dermatol 2000;27:434-9.
7. Halaban R, Langdon R, Birchall N, Cuono C, Baird A, Scott G, et al. Basic fibroblast growth factor from human keratinocytes is a natural mitogen for melanocytes. J Cell Biol 1988;107:1611-9.
8. Brysk MM, Newton RM, Rajamaran S, Plott T, Barlow E, Bell T, et al. Repigmentation of Vitiliginous skin by cultured cell. Pigment Cell Res 1989;2:202-7.
9. Mulekar SV. Melanocyte-kertinocyte cell transplantation for the treatment for stable vitiligo. Int J Dermatol 2003;42:132-6.
10. Falabella R. Repigmentation of segmental vitiligo by autologous minigraft. J Am Acad Dermatol 1983;9:514-21.
11. Van Geel N, Ongenae K, De Mil M, Naeyaert JM. Modified technique of autologous noncultured epidermal cell transplantation for repigmenting vitiligo: a pilot study. Dermatol Surg 2001;27:873-6.
12. Issa CM, Rehder J, Taube MB. Melanocyte transplantation for the treatment of vitiligo: effects of different surgical techniques. Eur J Dermatol 2003;13:34-9.
