# Supplementary material for: Therapeutic efficacy of cell-based therapy in vitiligo: a research letter systematically reviewed using meta-analysis
Source: Arch Dermatol Res. 2024 May 22;316(5):198. doi: 10.1007/s00403-024-02920-6 (PMC11111487; doi:10.1007/s00403-024-02920-6)
Supplement: Supplementary file 1 — Supplementary file1 (ZIP 24195 KB) [file 403_2024_2920_MOESM1_ESM.zip › Studies were included/Razmi 2014.pdf]

 doi: 10.1111/pcmr.12292

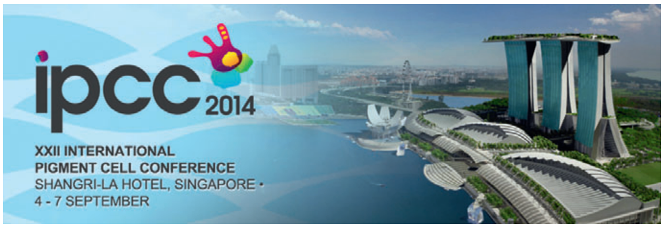

XXII International Pigment Cell Conference (IPCC) 'Bringing Colours to Life: Advances in Pigment Cell Research and Translation into Clinical Practice' Organised by the Asian Society for Pigment Cell Research (ASPCR), in partnership with the Dermatological Society of Singapore (DSS)

4th to 7th September 2014
Shangri-La Hotel, Singapore

ASIAN SOCIETY FOR PIGMENT CELL RESEARCH

DERMATOLOGICAL SOCIETY OF SINGAPORE

International Federation of Pigment Cell Societies

Local Organising Committee

Honorary Presidents:
Chee-Leok Goh, Prasad Kumarasinghe

President:
Boon Kee Goh

Vice-Presidents:
Steven Thng, Su-Ni Wong

Local Secretariat:
Alice Chew, Anjali Jhingan, Emily Gan, Lee-Foon Tang

Local Advisory Committee:
Birgit Lane, Roy Chan

ASPCR Advisory Committee:
Davinder Parsad, Cheng-Che Lan, Leihong Flora Xiang, Kyoung-Chan Park, Rashmi Sarkar, Sanjeev Mulekar

International Scientific Committee:
Zalfa Abdel-Malek, Hideya Ando, Robert Ballotti, Boris Bastian, Dorothy C Bennett, Markus Bohm, David Fisher, Jose Carlos Garcia-Borron, Nikolas K. Haass, Nick Hayward, Thomas J. Hornyak, Takahiro Kunisada, Lionel Larue, Caroline Le Poole, Mu-Hyoung Lee, Yuin-Han Loh, Lluis Montoliu, Chikako Nishigori, Emi K. Nishimura, Thierry Passeron, Mauro Picardo, Graca Raposo, Ze'ev Ronai, Shigeki Shibahara, John D. Simon, Andrzej Slominski, Richard A. Spritz, Tamio Suzuki, Alain Taïeb, Desmond J. Tobin, Nanja Van Geel, Kazumasa Wakamatsu

Melanoma Day Committee:
Boris Bastian, Dorothy C Bennett, David Fisher, Nikolas K. Haass, Nicholas Hayward, Lionel Larue, Chikako Nishigori, Ze'ev Ronai

Abstract Review Committee:
Zalfa Abdel-Malek, Hideya Ando, Robert Ballotti, Dorothy C Bennett, Markus Böhm, John Common, Sandipan Dhar, José Carlos García-Borrón, Chee Leok Goh, Somesh Gupta, Iltefat Hamzavi, Nikolas K. Haass, Nicholas Hayward, Peter Hersey, Thomas Hornyak, Ian Jackson, Hee Young Kang, Prasad Kumarasinghe, Cheng-Che Lan, Lionel Larue, Car-

© 2014 John Wiley & Sons A/S. Published by John Wiley & Sons Ltd

oline Le Poole, Lluis Montoliu, Alessandra Napolitano, Chikako Nishigori, Emi Nishimura, Frances Noonan, Amit Pandya, Kyoung Chan Park, Davinder Parsad, Thierry Passeron, Ralf Paus, Graca Raposo, Ze'ev Ronai, Rashmi Sarkar, Andrzej Slominski, Marisol Soengas, Lukas Sommer, Richard Sturm, Alain Taieb, Steven Thng, Desmond Tobin, Nanja Van Geel, Michele Verschoore, Kazumasa Wakamatsu, Leihong Flora Xiang, Hiroaki Yamamoto

Travel Awards Committee:
Mauro Picardo (Chairperson)
Andrzej Slominski (PASPCR)
Lionel Larue (ESPCR)
Lluis Montoliu (IFPCS)
Takahiro Kunisada (JSPCR)
Prasad Kumarasinghe (ASPCR)

Poster Prize Committee:
Davinder Parsad (Chairperson; ASPCR)
Lionel Larue (ESPCR)
Prashiela Manga (PASPCR)
Genji Imokawa (JSCPR)

Sponsors List (as of 11th July 2014)

Platinum Sponsors
Galderma
L'Oreal

Gold Sponsors
SK-II/ OLAY (P&G)
Roche

Silver Sponsors
Avène/Color Play Enterprise Pte Ltd
NeoAsia

Bronze Sponsors
Laboratoires Genévrier
Sinclair IS Pharma
Skin M.D

Exhibitors
Apex Pharma Marketing
Asia Healthcare Associates
Cambert (Bio-Oil)
Crystal Tomato
GSK (Stiefel)
General Topics SRL
IFC Group
Innomed Pte Ltd
Janssen Pharmaceutical
Johnson & Johnson
Laboratoire Dermatologique Bioderma
Leo Pharma
Lumenis
MSD Pharma
Medica Group
Menarini
Niks Professional
Ushio Asia Pacific Pte Ltd
Zoe Nissi

Awards and Grants
Amway
Clinuvel

General Sponsor
Curel

SCIENTIFIC PROGRAMME

Wednesday, 3rd September 2014

Pre-Congress Symposia

**07:15–08:00**	**Registration (Mezzanine Foyer, outside of Azalea Room)**
**08:00–10:30**	**Vitiligo Global Issues Consensus Conference (Pink/White Azalea Room)**<br>Chairs: K. Ezzedine - A. Taieb - M. Picardo
**10:30–11:00**	**Tea Break (Mezzanine Foyer)**
**11:00–13:00**	**Vitiligo: A Clinically Oriented Symposium (Pink/White Azalea Room)**<br>Chairs: A. Taieb - M. Picardo<br>**K. Ezzedine** (France): Web-Based Vitiligo Registry<br>**K. Ezzedine** (France): Skin Phototype May Influence the Burden of Vitiligo<br>**A. Pandya** (USA): Confetti Depigmentation as a Disease Marker in Patients with Generalized Vitiligo<br>**B. Khaitan** (India): A Study to Validate the Proposed Clinical Criteria for Diagnosis of Segmental Vitiligo<br>**S. Thng** (Singapore): Comparison of the 308 Excimer Lamp Versus Home Phototherapy in the Treatment of Focal Vitiligo<br>**T. Anbar** (Egypt): Partial Thickness Epidermal Cuts Versus Suction Blister Roofs as Sources of Preparing Non-cultured Epidermal Suspension for Vitiligo Treatment: A Comparative Study<br>**S. Gupta** (India): A point-of-Care, In-vivo Method of Preparation of Epidermal Strata Basal and Spinosum Cell Suspension for Transplantation in Vitiligo<br>**B. Laila** (Morocco): Two Atypical Cases of Mixed Vitiligo: Initial Non-segmental Vitiligo can Precede the Onset of Segmental Vitiligo<br>**C. Le Poole** (USA): Emerging Opportunities to Halt Depigmentation Using CCL22
**13:00–14:00**	**Lunch Symposium by Laboratoires Genevrier (Pink/White Azalea Room)**

© 2014 John Wiley & Sons A/S. Published by John Wiley & Sons Ltd

Ancillary Meeting 1:

14:00–17:00 **Vitiligo and Melasma in China – Integrated Medicine View (Red Azalea Room)**

W. Xiang (People's Republic of China): Real-time in vivo Confocal Laser Scanning Microscopy of Melanin-containing Cells and the Differences of Hypopigmented Macules: A Preliminary Study

S. Wang (People's Republic of China): Autologous Cultured Melanocyte Transplantation for Vitiligo Treatment: An Efficacy and Mechanism Study

Z. Pan (People's Republic of China): Confocal Laser Scanning Microscopy in the Differential Diagnosis of Hyperpigmentation Disorders

L. F. Xiang (People's Republic of China): The Role and Mechanism of RNASET2 in the Pathogenesis of Vitiligo

C. Zhang (People's Republic of China): Autophagy and Melanogenesis

D. Wang (People's Republic of China): Modulation of the Major Basement Membrane Components Control the Migration of Melanocytes in Hair Follicle

X. Chen (People's Republic of China): The Traditional Chinese Medicine in the Treatment of Vitiligo

Ancillary Meeting 2:

14:00–18:00 **Pigmentary Disorders Society: Common & Uncommon Pigmentary Disorders in Dark Skinned Patients (Pink/White Azalea Room)**

S. Gupta (India): Potential Using Epidermal Stem Cells in Vitiligo

D. Parsad (India): Newer Sources of Melanocytes: New Hope for Vitiligo

S. Dogra (India): Managing Complications and Optimizing Results in Cellular Grafting in Vitiligo

S. Mulekar (India): Segmental Vitiligo

N. Gokhale (India): Photoprotection in Dark Skinned Patients

R. Sarkar (India): Melasma - The Asian Scenario

S. Barua (India): Physical Therapies in Melasma

K. Godse (India): Ochronosis

S. Kumaran (India): Efficacy of Low Dose Oral Isotretinoin in the Treatment of Lichen Planus Pigmentosus – A Pilot Study

S. Sonthalia (India): Phenol Based Peels on Refractory Lichen Planus- A New Hope!

N. Sarma (India): Some Light on Periorbital Darkness

R. Arora (India): What's New in Pigmentary Disorders

L. Arya (India): Newer Insights in Etiopathogenesis and Management of Macular Amyloidosis

B. Swarnakar (India): Unusual Cases of Vitiligo

S. Bansal (India): Unusual Cases of Vitiligo

S. Amladi (India): Treatment of Periorbital Hyperpigmentation

J. Sakhiya (India): Combination of Various Molecules and Devices with Excimer Light in Management of Vitiligo

S. Aurangabadkar (India): Complication of Q-switched Nd-Yag Laser for Pigmentary Disorder in Dark Skinned Patients

17:00–18:00 **Regional Societies Council Meetings**

ASCPR (Red Azalea Room)

ESPCR (Yellow Orchid Room)

JSPCR (Jasmine Room)

PASPCR (White Gardenia Room)

18:00–19:00 **IFPCS 1st Council Meeting (Green Orchid Room)**

Thursday, 4th September 2014

07:00–08:15 **Registration (Foyer of Island Ballroom)**

08:15–08:35 **Opening Addresses: M. Picardo (IFPCS), D. Parsad (ASPCR), B. K. Goh (IPCC)**

08:35–09:55 **Plenary Session 1: Opening Lectures (Tower Ballroom – Casuarina/Acacia/Banyan)**

Chairs: D.Parsad - C. Le Poole - E.Nishimura

08:35–09:00 **Special Lecture 1** (**SL1**) **IFPCS Presidential Lecture** – **M. Picardo** (Italy): Vitiligo: An Update on Our Viewpoint

09:00–09:25 **SL2 Aaron Lerner Lecture** – **W. Pavan** (USA): Genetic and Genomic Analyses of Pigment Cell Development and Disease

09:25–09:55 **Guest Lecture 1** (**GL1**) – **Y. Barrandon** (Switzerland): Regenerative Medicine of Skin

09:55–10:25 **Tea Break & Exhibition Viewing (Island Ballroom/Azalea Rooms)**

10:25–12:00 **Plenary Session 2: Melanocyte UV Response & DNA Repair (Tower Ballroom – Casuarina/Acacia/Banyan)**

Chairs: Z. Abdel-Malek - K.C. Park - S. Ito

© 2014 John Wiley & Sons A/S. Published by John Wiley & Sons Ltd

10:25–10:55 **GL2 D. L. Mitchell** (USA): The Photobiology of Melanoma in Fish Models: The Importance of UV Wavelength, DNA Damage and Gender on Melanoma Etiology

10:55–11:15 **Invited Lecture 1** (**IL1**) – Z. Abdel-Malek (USA): Paracrine Factors as Guardians of Genomic Stability of Melanocytes

11:15–11:35 **IL2 C. Nishigori** (Japan): UV and Melanoma: Insights from Clinical Viewpoints

11:35–11:47 **CS1 K. Yin** (Australia), K. Jagirdar, A. Smith: Exploring Signalling Pathways Co-ordinating the Melanocyte Ultraviolet Response: Identification of a Novel Role for NR4a Orphan Nuclear Receptors in Cellular Stress Response

11:47–11:59 **CS2 V. Swope** (USA), R. Starner, L. Koikov, N. Hone, D. Mehta, A. Luisa Kadekaro, Z. Abdel-Malek: Skin Cancer Chemoprevention Strategy Using Melanocortin 1 Receptor-selective Small α-MSH Analogs

12:00–12:30 **Poster Viewing (Azalea Room)**

12:30–13:30 **Round Table Discussion by Sinclair IS Pharma (by Invitation only)**

12:30–13:30 **Concurrent Lunch Symposia**
Lunch Symposium by L'Oréal (Banyan Ballroom)
Lunch Symposium by Galderma (Casuarina Ballroom)

13:30–15:00 **Concurrent Sessions 1, 2, 3 (Tower Ballroom – Casuarina/Acacia/Banyan)**

Concurrent Session 1: Developmental Biology (Casuarina Ballroom)

Chairs: L. Sommer - T. Hirobe - E. Steingrimsson

13:30–13:50 **IL3 T. Hirobe** (Japan): Regulation of the Development of Mouse Melanocytes by Coat Color Genes

13:50–14:10 **IL4 L. Sommer** (Switzerland): Common Mechanisms Regulating Neural Crest Development and Melanoma Formation

14:10–14:22 **CS3 A. Saldana-Caboverde** (USA), D. E. Watkins-Chow, W. J. Pavan, L. Kos: ETS1 and SOX10 Act Synergistically During Early Melanocyte Development

14:22–14:34 **CS4 E. Steingrimsson** (Iceland), T. Zhang, Q. Zhou, M. H. Ogmundsdottir, R. Siddaway, M.I Singh, S. W. Kong, C. R. Goding, L. Larue, A. Palsson, F. Pignoni**:** MIT Factors as Master Regulators of the V-type H+ ATPase: Conservation of Coordinate Transcriptional Regulation of Subunit Genes in Fly and Vertebrates

14:34–14:46 **CS5 R. N. Kelsh** (UK), A. Lapedriza, H. Schwetlick: Modelling the Melanocyte Gene Regulatory Network – Quantitation, Kit and Manipulation

14:46–14:58 **CS6 H. Aoki** (Japan), T. Kunisada: Conditional Deletion of Kit Induces White Spotting Phenotype Through Cell-autonomous Requirement of Kit Signaling

Concurrent Session 2: Melanin Biophysics & Chemistry (Banyan Ballroom)

Chairs: K. Wakamatsu - A. Napolitano - P. Meredith

13:30–13:50 **IL5 P. Meredith** (Australia): The Optical and Electrical Properties of Melanins: Archetypal Bioelectronic Materials?

13:50–14:10 **IL6 K. Wakamatsu** (Japan): Elaboration of Approach to Structure of Neuromelanin Present in Various Brain Regions as Studied by Chemical Degradative Methods

14:10–14:22 **CS7 A. Napolitano** (Italy), L. Panzella, L. Leone, R. Micillo, G. Monfrecola: Human Red Hair Pheomelanin is a Potent Pro-oxidant Mediating UV-independent Mechanisms of Oxidative Stress in Red Hair Phenotypes

14:22–14:34 **CS8 S. Ito** (Japan), M. Ojika, T. Yamashita, K. Wakamatsu: Tyrosinase-catalyzed Oxidation of Rhododendrol Produces 2-methylchromane-6,7-dione, the Ultimate Toxic Metabolite: Implications for Melanocyte Toxicity

14:34–14:46 **CS9 T. Jan Sarna** (Poland), M. Zareba, J. M. Burke, C. M. Skumatz, M. Sarna, A. K. Pilat: The Modulatory Effect of Melanin on Susceptibility of Retinal Pigment Epithelial Cells to Photic Stress

14:46–14:58 **CS10 Y. Niki** (Japan), M. Fukata, Y. Fukata, S. Oku, C. Makino-Okamura, S. Takeuchi, K. Wakamatsu, S. Ito, C. Nishigori, L. Declercq, D. B. Yarosh, N. Saito: Palmitoylation of Tyrosinase Regulates Melanin Synthesis through Ubiquitination

Concurrent Session 3: Vitiligo Research (Acacia Ballroom)

Chairs: K. Ezzedine - P. Manga - A. Mar

13:30–13:50 **IL7 C. Le Poole** (USA): Experimental Basis for HSP70iQ435A Therapy to Treat Vitiligo

13:50–14:10 **IL8 Q. S. Mi** (USA): Increased Circulating Th17 Cells and Decreased Circulating iNKT Cells in Human Non-segmental Vitiligo

14:10–14:30 **IL9 X. J. Zhang** (People's Republic of China): The Susceptibility Genes of Vitiligo

14:30–14:40 **CS11 P. Manga** (USA), S. J. Orlow, S. Toosi: A Role for the Unfolded Protein Response in the Etiology of Vitiligo

 © 2014 John Wiley & Sons A/S. Published by John Wiley & Sons Ltd

14:40–14:50 **CS12 M. I. Nishimura** (USA), S. Chatterjee, J. Eby, M. Soloshchenko, S. Mehrotra, C. Le Poole: A Quantitative Increase in Regulatory T Cells Control Development of Vitiligo

14:50–15:00 **CS13 L. Komen** (The Netherlands), G. E. van der Kraaij, W. J. van der Veen, M. A. de Rie, A. Wolkerstorfer: The Validity, Reliability and Acceptability of the SAVASI: A Newly Developed Self-assessment Score in Vitiligo

15:00–15:30 Tea Break & Exhibition Viewing (Island Ballroom/Azalea Rooms)

15:30–17:00 Concurrent Sessions 4, 5, 6 (Tower Ballroom – Casuarina/Acacia/Banyan)

Concurrent Session 4: Regulation of Pigmentation (Casuarina Ballroom)

Chairs: J.C. Garcia-Borron - T. Akiyama - L. Kos

15:30–15:50 **IL10 D. Tobin** (UK): So Many Roads to Melanin – How Important are Bone Morphogenetic Proteins in Regulating Human Cutaneous Pigmentation?

15:50–16:10 **IL11 J. C. Garcia-Borron** (Spain): Pathway-specific Regulation of Human Melanocortin-1 Receptor Signaling

16:10–16:22 **CS14 F. Rambow** (France), A. Bechadergue, G. Saintigny, F. Morizot, C. Mahe, L. Larue: Regulation of Pigmentation by MIR-330-5P Targeting Tyrosinase

16:22–16:34 **CS15 J. Pino** (USA), S. Ito, K. Wakamatsu, L. Kos: Regulation of Murine Pigment Production by Endothelin-3

16:34–16:46 **CS16 T. Fukumoto** (Japan), M. Oka, T. Iwasaki, M. Sakaguchi, Y. Fukami, C. Nishigori: Ultraviolet B-induced Up-regulation of MCL-1L Mediated by the MEK–ERK1/2-STAT3 Signaling Pathway Protects Melanocytes from Apoptosis

Concurrent Session 5: Melanosome Biogenesis & Transfer (Banyan Ballroom)

Chairs: G. Raposo - H. Ando - J. S. Hwang

15:30–15:50 **IL12 G. Raposo** (France): The Biogenesis of Melanosomes and Intercellular Communication in the Skin

15:50–16:10 **IL13 H. Ando** (Japan): A Role of Multiple Melanosome-containing Globules in Melanosome Transfer and a Possible Contribution of Fibroblasts to Dermal Melanin Deposition

16:10–16:22 **CS17 M. Ishida** (Japan), S. P. Arai, N. Ohbayashi, M. Fukuda: Mechanism of the Melanosome Transport Inhibition Induced by a Constitutive Active RAB27a Mutant in Melanocytes: Development of a Novel Melanosome-targeting Tag and Its Applications

16:22–16:34 **CS18 S. Koike** (Japan), K. Yamasaki, T. Yamauchi, M. Inoue, K. Tsuchiyama, S. Aiba: TRIF and MAVS Pathway is Essential to Induce RAB27a and Melanosome Transportation by TLR3 Agonist Poly(I:C) in Human Epidermal Melanocytes

16:34–16:46 **CS19 C. Delevoye** (France), F. Gilles-Marsens, M. Dennis, M. S. Marks, G. Raposo: BLOC-1 Controls the Formation of Recycling Endosomes Required for Melanosome Biogenesis

16:46–16:58 **CS20 A. Hachiya** (Japan), D. Murase, R. Hicks, S. Moriwaki, T. Hase: Cooperation of Endothelin-1 Signaling with Melanosomes Plays a Role in Developing and/or Maintaining Hyperpigmentation

Concurrent Session 6: Repigmenting Vitiligo – Of Art & Science (Acacia Ballroom)

Chairs: T. Lotti - I. Hamzavi - B. Khaitan

15:30–15:50 **IL14 I. Hamzavi** (USA): Update on Afamelanotide and NBUVB for Vitiligo

15:50–16:10 **IL15 C. C. E. Lan** (Taiwan ROC): UVB Phototherapy in Vitiligo Treatment: Immune Regulation Versus Biostimulation

16:10–16:30 **IL16 T. Passeron** (France): Management of Refractory Vitiligo

16:30–16:40 **CS21 K. Ezzedine** (France), V. Eleftheriadou, J. Seneschal, M. Picardo, A. Taieb: How to Define 'Repigmentation/Maintenance of Repigmentation' in Vitiligo – VGICC Consensus Position

16:40–16:50 **CS22 S. Birlea** (USA), D. Norris, N. Goldstein, M. Koster, L. Hoaglin, S. Robinson, W. Robinson, D. Roop: Mobilization of Melanocyte Populations from Stem Cells in the Hair Follicle by Ultraviolet Light Treatment of Human Vitiligo

16:50–17:00 **CS23 T. Passeron** (France), M. Cavalié, K. Ezzedine, E. Fontas, H. Montaudié, E. Castela, P. Bahadoran, J. Lacour: Proactive Treatment of Non-segmental Vitiligo with 0.1% Tacrolimus Ointment: Double-blind Placebo Controlled Study

17:15–19:30 **Welcome Reception (Island Ballroom)**

Friday, 5th September, 2014

07:45–08:30 Breakfast Symposium by Avène/Color Play Enterprise Pte Ltd (Tower Ballroom – Casuarina/Acacia/Banyan)

08:30–10:00 Plenary Session 3: Genetics of Pigmentation (Tower Ballroom – Casuarina/Acacia/Banyan)

Chairs: L. Montoliu - P. Kumarasinghe - T. Suzuki

© 2014 John Wiley & Sons A/S. Published by John Wiley & Sons Ltd

08:30–08:55 **Seiji Memorial Lecture**
SL3 R. Sturm (Australia): Painting by Numbers -Genetics of Human Pigmentation and Ancestry
08:55–09:15 **IL17 G. Barsh** (USA): Genetics of Stripes and Spots
09:15–09:35 **IL18 R. Spritz** (USA): The Genetics of Vitiligo and Associated Autoimmune Diseases
09:35–09:47 **CS24 N. Box** (USA), N. Asdigian, A. Baron, J. Aalborg, V. Gonzalez, J. Dunn, R. Dellavalle, T. Terzian, M. Berwick, S. Mokrohisky, J. Morelli, L. Crane: Identifying Genes that Interact with UVR to Influence Nevi and Freckles
09:47–09:59 **CS25 J. D. Andersen** (Denmark), C. Børsting, M. M. Andersen, N. Morling: Searching for New Markers Associated with Eye Colour by Sequencing the Human OCA2-HERC2 Region

10:00–10:30 Tea Break & Exhibition Viewing (Island Ballroom/Azalea Rooms)

10:30–12:05 Plenary Session 4: Colours of Life (Tower Ballroom – Casuarina/Acacia/Banyan)
Chairs: B. K. Goh - G. Barsh - A. Taieb
10:30–11:00 **GL3 R. T. Hanlon** (USA): Ocean Colours Exemplified: Rapid Adaptive Skin Colouration and Patterning in Cephalopods
11:00–11:30 **GL4 N. Jablonski** (USA): Natural Selection and the Evolution of Skin Color Phenotypes
11:30–11:50 **IL19 J. E. Harris** (USA): Losing Color – Keratinocytes Drive Autoimmunity in Vitiligo Through CXCL10
11:50–12:02 **CS26 K. McGowan** (USA), F. Imsland, C. Henegar, S. Fard, Carl-Johan Rubin, D. Schwochow, E. Sundström, G. Barsh, L. Andersson: A Horse of a Different Color: The DUN Locus Reveals a Novel Mechanism Underlying Camouflage Pigmentation

12:05–13:05 IFPCS 2nd Council Meeting (Green Orchid Room)

12:05–12:30 Poster Viewing (Azalea Rooms)
12:30–13:30 Concurrent Lunch Symposia
Lunch Symposium by Procter & Gamble (Banyan Ballroom)
Lunch Symposium by Roche (Casuarina Ballroom)
13:30–15:00 Concurrent Sessions 7, 8, 9 (Tower Ballroom- Casuarina/Acacia/Banyan)

Concurrent Session 7: Melanocyte & Stem Cell Biology (Casuarina Ballroom)
Chairs: T. Hornyak - T. Kunisada - E. Nishimura
13:30–13:50 **IL20 T. Hornyak** (USA): Functional Properties of Subsets of Melanocyte Stem Cells
13:50–14:10 **IL21 T.Kunisada** (Japan): Developmental Control of Dermal Melanocytes Progenitors
14:10–14:22 **CS27 T. Yamauchi** (Japan), K. Yamasaki, K. Tsuchiyama, S. Koike, M. Dezawa, S. Aiba: Induction of Melanocytes from Multilineage-differentiating Stress-enduring (Muse) Cells Derived from Human Adipose Tissue
14:22–14:34 **CS28 M. Osawa** (Japan), M. Matsumoto, D. Lee: Phenotype-based Gene Function Assessment Using a Melanocyte-specific Transgenesis Approach
14:34–14:46 **CS29 C. Ceol** (USA), S. Iyengar: Dual Mechanisms Combine to Mediate Regeneration of Zebrafish Melanocytes Following Injury
14:46–14:58 **CS30 S. Aras** (USA), I. de la Serna: To Investigate the Role of BAF60a in Melanocyte Differentiation

Concurrent Session 8: Metabolic and Intracellular Signaling (Banyan Ballroom)
Chairs: Z. Ronai - R.Ballotti - G.Imokawa
13:30–13:50 **IL22 Z. Ronai** (USA): Rewired Signalling in Melanoma – A Journey along the PDK1 Axis
13:50–14:10 **IL23 E. Gottlieb** (UK): Metabolic Vulnerabilities of Melanoma
14:10–14:30 **IL24 L. Marrot** (France): Pigmentation, Oxidative Stress and Specific Epidermal Detoxification
14:30–14:42 **CS31 R. J. Perera** (USA), J. Mazar, A. D. Richardson, F. Qi, B. Lee, J. Marchica, A. Duran, Y. Tao, S. S. Govindarajan, J. Shelley, L. M. Brill, J. Li, X. Han: MIR-211 Functions as a Metabolic Switch in Human Melanoma Cells
14:42–14:54 **CS32 A. Smith** (Australia), R. Kim, A. Soogrim, M. Fane, R. Sturm: Exploring the Role of IRF4 in Melanocyte Responses to Interferon-gamma Signalling

Concurrent Session 9: Advanced Surgical Interventions in Vitiligo (Acacia Ballroom)
Chairs: D. Parsad - N. Van Geel - C. F. Zhang
13:30–13:50 **IL25 N. Van Geel** (Belgium): Non-cultured Cellular Grafting in Vitiligo: Pearls and Pitfalls
13:50–14:10 **IL26 S. Mulekar** (Saudi Arabia): Surgical Treatment in Difficult Sites
14:10–14:30 **IL27 S. Gupta** (India): Treasure Hunt – Hair Follicle as a Rich Source of Melanocytes and Stem Cells for Transplantation in Vitiligo
14:30–14:40 **CS33 D. Bassiouny** (Egypt), B. M. El-Zawahry, S. Esmat, N. Sameh, R. Sobhi, D. Abdel-Halim, M. Adly, M. safwat, R. Hegazy, H. Gawdat, N. Samir, M. Khorshied, H. Gouda, Z. El-Maadawi: Effect of Procedural-

© 2014 John Wiley & Sons A/S. Published by John Wiley & Sons Ltd

related Variables on Melanocyte-Keratinocye Suspension Transplantation in Stable Vitiligo: A Clinical and Immunohistochemical Study

14:40–14:50 **CS34 M. T. Razmi,** D. Parsad, S. M. Kumaran (India): Transplantation of Autologous Non-cultured Extracted Hair Follicle Outer Root Sheath Cell and Autologous Non-cultured Epidermal Cell Suspension in Combination as a Novel Method in Vitiligo Surgery Cell Suspension for Treatment of Stable Vitiligo

14:50–15:00 **CS35 E. Y. Gan** (Singapore), Y. Ling Kong, S. Thng, B. K. Goh: A 5-year Review of Non-cultured Cellular Grafting for Vitiligo

15:00–15:30 Tea Break & Exhibition Viewing (Island Ballroom/Azalea Rooms)

15:30–17:00 Concurrent Sessions 10, 11, 12 (Tower Ballroom – Casuarina/Acacia/Banyan)

Concurrent Session 10: Model Systems for Pigment Cell Biology & Disease (Casuarina Ballroom)

Chairs: I. Jackson - H. Yamamoto - L. Benzekri

15:30–15:50 **IL28 I. Jackson** (UK): How Do Melanocytes Find Their Way?

15:50–16:10 **IL29 H. Yamamoto** (Japan): Functional Divergence of Mammalian Melanocytes

16:10–16:22 **CS36 M. Cario-André** (France): Fibroblasts Regulate Both Physiological and Pathological Pigmentation of Skin in vitro and in vivo

16:22–16:34 **CS37 J. Qiu** (People's Republic of China), J. Liu, X. Huang, J. Chen, A. Pena, Z. Cai: Investigating the Modulation of Cutaneous Pigmentation in an Asian Reconstructed Pigmented Epidermis Using Multiphoton Microscopy

16:34–16:46 **CS38 V. Delmas** (France), R. Wagner, F. Luciani, M. Cario-Andre, A. Rubod, V. Petit, L. Benzekri, K. Ezzedine, S. Lepreux, A. Taeib, Y. Gauthier, L. Larue: Altered E-cadherin Levels and Distribution in Melanocytes Precedes Clinical Manifestations of Vitiligo

16:46–16:58 **CS39 O. A. Arowojolu** (USA), S. J. Orlow, P. Manga: Activation of Melanocyte Antioxidant Response Pathways Following Exposure to Vitiligo-inducing Phenols: Implications for Vitiligo Pathogenesis

Concurrent Session 11: Neuroendocrinology and Regulatory Controls of Pigmentation (Banyan Ballroom)

Chairs: M. Böhm - T. Kawakami

15:30–15:50 **IL31 M. Böhm** (Germany): Prohormone Convertases and Melanoma – Emerging Players in Basic and Translational Research

15:50–16:02 **CS40 S. Choi** (Republic of Korea), H. Il Cho, J. Min Jung, M. Woo Lee, J. Ho Choi, S. Eun Chang: Dopamine D4 Receptor Antagonist Inhibits a-MSH-stimulated Induced Melanogenesis Through the Downregulation of MITF via Acceleration of ERK Activation

16:02–16:14 **CS41 T. Kawakami** (Japan), A. Ohgushi, T. Hirobe, Y. Soma: Retinoic Acid Triggers Difference of Melanogenesis on Human Epidermal Melanocytes and Melanoblasts

16:14–16:26 **CS42 T. Passeron** (France), L. Duteil, N. Cardot-Leccia, C. Queille-Roussel, Y. Maubert, D. Ambrosetti, J. Lacour: Differences in Visible Light Induced Pigmentation According to Wavelengths: A Clinical and Histological Study in Comparison with UVB Exposure

Concurrent Session 12: Pigmentary Challenges – Panel Discussions (Acacia Ballroom)

15:30–16:15 **Panel Discussion 1**: Nosologic Controversy of Lichen Planus Pigmentosus
Moderator: **D. Parsad** (India);
Panelists: **R. Schwartz** (USA), **P. Kumarasinghe** (Australia), **N. Al-Mutairi** (Kuwait), **S. H. Tan** (Singapore)

16:15–17:00 **Panel Discussion 2**: Acquired Hypopigmentary Disorders in Children
Moderator: **D. Parikh** (India)
Panelists: **S. Dhar** (India), **A. Taieb** (France), **Y. K. Tay** (Singapore)

18:30–21:30 Speakers' Dinner at Altimate, 1-Altitude

Shuttle departs from Shangri-la Hotel at 18:00. Return buses to Shangri-La Hotel will leave from 21:30. The last bus will leave at 22:00.

Saturday, 6th September 2014 (Melanoma Day & DSS ASM)

07:45–08:30 Breakfast Symposium by Procter & Gamble (Tower Ballroom – Casuarina/Acacia/Banyan)

08:30–10:05 Plenary Session 5: Melanoma Biology (Tower Ballroom – Casuarina/Acacia/Banyan)

Chairs: L. Larue - N. Hayward - M.Takata

08:30–08:50 **IL32 B. Bastian** (USA) – The Genetic Evolution of Melanocytic Neoplasms

08:50–09:10 **IL33 C. R. Goding** (UK): The Role and Regulation of MITF in Melanoma Heterogeneity

09:10–09:30 **IL34 N. K. Haass** (Australia): Defining The Mode of Melanoma Heterogeneity by Real-time Cell Cycle Imaging

© 2014 John Wiley & Sons A/S. Published by John Wiley & Sons Ltd

09:30–09:50 **IL35 R. Marais** (UK): BRAF and RAS Signalling: Basic Biology and Therapeutic Options

09:50–10:02 **CS43 T. Bald** (Germany), J. Landsberg, D. A. Lopez-Ramos, M. Renn, N. Glodde, P. Jansen, E. Gaffal, J. Steitz, R. Tolba, U. Kalinke, G. Jönsson, M. Hölzel, T. Tüting: Targeted Activation of the Type I Interferon System in Combination with PD-1 Blockade is a Rational Strategy to Expose Immune Cell-poor HGF-CDK4 Mouse Melanomas to Cellular Immune Surveillance

10:05–10:35 **Tea Break & Exhibition Viewing (Island Ballroom/Azalea Rooms)**

10:35–12:00 **Plenary Session 6: Targeted Therapies and Drug Resistance (Tower Ballroom – Casuarina/Acacia/Banyan)**

Chairs: D. Norris - P. Soyer - V. Setaluri

10:35–10:55 **IL36 P. Hersey** (Australia): Targeting Resistance Pathways in Melanoma with Epigenetic Regulators

10:55–11:15 **IL37 T. Tüeting** (Germany): Inflammation-induced Phenotypic Plasticity of Melanoma in Therapy Resistance and Tumor Progression

11:15–11:35 **IL38 M. S. Soengas** (Spain): Non-invasive Imaging of Neo-lymphangiogenesis for the Identification of Metastatic Niches and Anticancer Agents in Melanoma

11:35–11:47 **CS44 H. Cheng** (USA)**,** M. Terai, K. Kageyama, S. Ozaki, T. Sato, A. Aplin: Paracrine Effect of Neuregulin 1 and Hepatocyte Growth Factor Drives Resistance to Mitogen-activated Protein Kinase Kinase Inhibitors in Metastatic Uveal Melanoma

11:47–11:59 **CS45 Y. Shellman** (USA)**,** N. Mukherjee, S. Reuland, Y. Lu, Y. Luo, M. Fujita, D. Norris: Targeting Melanoma Bulk and Cancer Initiating Cells with a Combination of the Retinoid Derivative Fenretinide and a BCL-2 Inhibitor

12:00–13:00 **Regional Society Assemblies**

ASPCR (VIP Room, Next to Jurong Ballroom)

ESPCR (Yellow Orchid Room)

JSPCR (Green Orchid Room)

PASPCR (White Gardenia Room)

12:00–12:30 **Poster Viewing (Azalea Rooms)**

12:30–13:30 **Concurrent Lunch Symposia**

Lunch Symposium by NeoAsia (Banyan Ballroom)

Lunch Symposium by Skin M.D. (Casuarina Ballroom)

13:30–15:00 **Concurrent Sessions 13, 14, 15 (Tower Ballroom- Casuarina/Acacia/Banyan)**

Concurrent Session 13: Senescence Pathways and Melanoma (Casuarina Ballroom)

Chairs: D. C. Bennett - H. Schaider

13:30–13:50 **IL39 D. C. Bennett** (UK): Melanocyte Senescence Pathways: The Impact of p16 Defects

13:50–14:10 **IL40 R. Balloti** (France) Senescence in Melanoma: A Stab in the Back?

14:10–14:22 **CS46 C. F. Zhang** (Austria & People's Republic of China)**,** F. Gruber, C. Ni, M. Mildner, U. Koenig, S. Karner,C. Barresi, H. Rossiter, L. Larue, D. Tobin, L. Eckhart, E. Tschachler: Suppression of Autophagy Dysregulates the Antioxidant Response and Causes Premature Senescence of Melanocytes

14:22–14:34 **CS47 X.Q. Wang** (USA)**,** A. Velez, J. Lu, A. S. Paller: Inhibition of HDAC3 Accelerates Autophagy to Induce Melanoma Cell Death

14:34–14:46 **CS48 M. Villareal** (Japan), H. Isoda: Diterpenoid Hirsein-A Activate Nuclear P38 Through GADD45b Upregulation: Possible Route for Induction of Melanoma Cell Senescence

14:46–14:58 **CS49 J. Teh** (USA), A. Aplin: CDK4/6 as a Therapeutic Target in Malignant Melanoma

Concurrent Session 14: Genetics and Genomics of Melanoma (Banyan Ballroom)

Chairs: N. K. Haass - A. Bosserhoff - W. Pavan

13:30–13:50 **IL41 N. Hayward** (Australia): The Genetic Architecture of Melanoma Predisposition

13:50–14:10 **IL42 A. Bosserhoff** (Germany): Role and Processing of miRs in Melanoma

14:10–14:22 **CS50 P. Laurette** (France), T. Strub, D. Koludrovic, M. Ennen, G. Mengus, I. Davidson**:** Functional Characterisation of the Microphthalmia-associated Transcription Factor (MITF) Interactome Identifies Novel Cofactors Essential for Melanoma Cell Growth

14:22–14:34 **CS51 P. F. Cheng** (Switzerland), O. Shahkova, D. S. Widmer, D. Zingg, B. Belloni, M. M. Raaijmakers, O. M. Eichhoff, S. M. Goldinger, Silvio Hemmi, Keith S. Hoek, L. Sommer, R. Dummer, M. P. Levesque: Methylation Dependent SOX9 Expression Mediates Invasion in Human Melanoma Cells and is a Negative Prognostic Factor in Advanced Melanoma

14:34–14:46 **CS52 R. J. Perera** (USA) Wei Zhao, Joseph Mazar, Bongyong Lee, John Shelley, Subramaniam S. Govindarajan, Marcel E. Dinger: The Molecular Function of the Long Non-coding RNA SPRY4-LT1 in Human Melanocytes

14:46–14:58 **CS53 H. Peter Soyer** (Australia), K. Jagirdar, N. Abbott, P. McClenahan, E. McEniery, D. Duffy, R. Sturm: Genetic Association of Clinical and Dermoscopic Nevus Patterns in a Queensland Case–Control Study of Melanoma

© 2014 John Wiley & Sons A/S. Published by John Wiley & Sons Ltd

Concurrent Session 15: Melasma – New Findings, New Approaches (Acacia Ballroom)
Chairs: C. C. E. Lan - A. Pandya - J. Lim

13:30–13:50 **IL43 ASPCR John Pawelek Lecture** – **H. Y. Kang** (Republic of Korea): Vascular Characteristics of Melasma and Therapeutic Implications
13:50–14:10 **IL44 A. Y. Lee** (Republic of Korea): Reduced H19 RNA in Melasma: Role of miR-675 Through Its Direct Target
14:10–14:30 **IL45 P. Wattanakrai** (Thailand): Laser Treatment of Melasma in Asians
14:30–14:40 **CS54 T. Passeron** (France), F. Boukari, E. Jourdan, E. Fontas, H. Montaudié, E. Castela, Jean-Philippe Lacour: Prevention of Melasma Relapses with Sunscreen Combining Protection Against UV and Short Wavelengths of Visible Light: A Prospective Randomized Comparative Trial
14:40–14:50 **CS55 S. E. Chang** (Republic of Korea), S. Choi B. Y. Chung, J. E. Kim, M. W. Lee, J. H. Choi,: Gene Express on Profiling of Melasma in Korean Women
14:50–15:00 **CS56 M. Hermawan** (Indonesia), I. Bernadette, T. N. A. Jacoeb: Telangiectases in Melasma – Could it be a Potential Clinical Severity Marker to Add Treatment Options

15:00–15.30 **Tea Break & Exhibition Viewing (Island Ballroom/Azalea Rooms)**

15:30–17:00 **Concurrent Sessions 16, 17, 18 (Tower Ballrooms – Casuarina/Acacia/Banyan)**

Concurrent Session 16: UV & Non-UV Pathways to Melanoma (Casuarina Ballroom)
Chairs: F. P. Noonan - T. Tüeting - P. Asawanonda

15:30–15:50 **IL46 F. P. Noonan** (USA): Interaction Between UVA and Eumelanin but not Phaeomelanin Results in Melanoma
15:50–16:10 **IL47 T. Bald** (Germany): UV-irradiation Promotes Melanoma Metastasis
16:10–16:22 **CS57 H. C. Murray** (Australia), Vicki E. Maltby, Nikola A. Bowden: Nucleotide Excision Repair in Response to UVA is Deficient in Melanoma
16:22–16:34 **CS58 A. P.Benaduce** (USA), D. Batista, G. Grilo, K. Jorge, D. Cardero, C. Milikowski, L. Kos: XPC Deficiency Enhances Melanoma Photocarcinogenesis in Transgenic K5-EDN3 Mice
16:34–16:46 **CS59 A. K Indra** (USA): Retinoid-X-Receptors (A/B) in Melanocytes Modulate Innate Immune Responses and Differentially Regulate Cell Survival Following UV Irradiation
16:46–16:58 **CS60 F. Liu-Smith** (USA), F. L. Meyskens: The NRF2 and NOX1 Mediated Redox Balance in UV-induced Melanoma Development

Concurrent Session 17: Developmental Biology & Melanoma Animal Models (Banyan Ballroom)
Chairs: L. Larue - M. Ziman - T. Yamashita

15:30–15:50 **IL48 L. Larue** (France): Beta-Catenin Signalling Pathway in Melanoblasts and Melanoma
15:50–16:02 **CS61 M. Hoelzel** (Germany), N. Glodde, D. van den Boorn-Konijnenberg, T. Bald, T, Tüeting: A CRISPR/CAS9-based 'Modular' Murine Melanoma Model to Study Determinants of Multimodal Immunotherapeutic Regimens
16:02–16:14 **CS62 J. Mahon** (USA)**,** J. Mosenson, J. O'Sullivan, J. Eby, M. Nishimura, S. Mehrotra, J. Guevara-Patino, C. Le Poole: HSP70iQ435a Mediates Anti-melanoma Responses in Mice
16:14–16:26 **CS63 C. C. Yang** (Taiwan ROC), L. F. Shyur: Oxylipin Metabolomics Study of Melanoma Brain Metastasis and the Drug Effect of Liposomal Doxorubicin in Syngeneic Mice
16:26–16:38 **CS64 M Fane** (Australia), A. G. Smith: Investigating the Role of MITF-BRN2 Expression Axis in Metastatic Melanoma

Concurrent Session 18: Pigmentary Challenges in Skin of Colour (Acacia Ballroom)
Chairs: S. Thng - R. Schwartz - M. Akiyama

15:30–15:50 **IL50 B. K. Goh** (Singapore): Pigmentary Challenges in Asian Skin
15:50–16:10 **IL51 A. Pandya** (USA): Evaluation and Management of Post-inflammatory Hyperpigmentation
16:10–16:30 **IL52 L. F. Xiang** (People's Republic of China): Challenges in Dermal Pigmentation
16:30–16:40 **CS65 A. Kaur** (India), M. S. Kumaran, D. De, D. Parsad, S. Handa: Lichenoid Photocontact Dermatitis vs Lichen Planus Pigmentosus: A Diagnostic and Management Dilemma in Asian Skin
16:40–16:50 **CS66 S. A. Poojary** (India), M. Khoja: Erythema Dyschromicum Perstans vs Lichen Planus Pigmentosus: A Comparative Clinico-histopathological Study
16:50–17:00 **CS67 M. Kono** (Japan), K. Sugiura, M. Suganuma, M. Hayashi, H. Takama, T. Suzuki, K. Matsunaga, Y. Tomita, M. Akiyama: Reticulate Acropigmentation of Kitamura and Dowling-degos Disease are Genetically Independent Disorders Distinct from each other

17:00–18:00 **IFPCS General Assembly (Green Orchid Room)**

18:00–21:30 **Gala Dinner & Awards Ceremony** *(Tickets required)* Seafood Paradise at Singapore Flyer. First Shuttle Departs from Shangri-la Hotel at 17:45, Last Shuttle to Leave at 18:15

© 2014 John Wiley & Sons A/S. Published by John Wiley & Sons Ltd

Sunday, 7th September 2014 (DSS ASM)

08:30–09:30 **Plenary Session 7: Clinical & Translational Dermatology (Tower Ballroom – Casuarina/Acacia/Banyan)**
Chairs: B. Lane - S. N. Wong
08:30–09:00 **GL5 J. Krutmann** (Germany): Environmentally-induced Skin Aging: Insights into New Molecular Mechanisms
09:00–09:30 **GL6 DSS Prof Chan Heng Leong Memorial Lecture – H. Y. Chang** (USA): Patterning the Skin Epigenome

09:30–10:40 **Plenary Session 8: Women Leaders in Pigment Cell Research (Tower Ballroom – Casuarina/Acacia/Banyan)**
Chairs: M. Galibert - C. Nishigori - R. Sarkar
9:30–10:00 **IL53 Prof Masako Mizoguchi Memorial Lecture – Z. Abdel-Malek** (USA): Prof Mizoguchi, a Bright Star that Shines in the East and West, and will not Fade
10:00–10:20 **IL54 P. Grimes** (USA): Skin Pigmentation in Health & Disease: Novel and Seasoned Players
10:20–10:40 **IL55 E. Nishimura** (Japan): Coupling of the Stress Sensitivity of Melanocyte Stem Cells to Their Dormancy During a Hair Cycle

10:40–11:10 **Tea Break & Exhibition Viewing (Island Ballroom/Azalea Rooms)**

11:10–12:40 **Concurrent Sessions 19, 20, 21 (Tower Ballroom – Casuarina/Acacia/Banyan)**

Concurrent Session 19: Hair Biology & Pigmentation (Casuarina Ballroom)
Chairs: D. Tobin - N. Sarma - J. Lee
11:10–11:30 **IL56 D. J. Tobin** (UK): Neuroendocrine Control of Human Hair Pigmentation – An Update
11:30–11:50 **IL57 S. Commo** (France): Study of Human Hair Pigmentation: Insights into Hair Graying and Beyond
11:50–12:02 **CS68 S. S. Joshi** (USA) J. M. Huang, T. J. Hornyak: Distinct Developmental Potentials of Melanocyte Progenitors from the Hair Follicle Bulge and Secondary Hair Germ
12:02–12:14 **CS69 M. Yuriguch**i (Japan), H. Aoki, N. Taguchi, T. Kunisada: Pigmentation of the Regenerated Hair Follicles after Wounding
12:14–12:26 **CS70 V. Mendiratta** (India), S. Rana, A. Rao, R. Chander: Premature Graying of Hair – Paving the Way to Treatment

Concurrent Session 20: Colourful Skin Optics (Banyan Ballroom)
Chairs: N. Kollias - D. Y. Lee
11:10–11:30 **IL58 N. Kollias** (USA): Human Pigmentation – The Colours of Skin
11:30–11:42 **CS71 E. Tancrède-Bohin** (France), T. Baldeweck, E. Decenciere, S. Brizion, S. Koudoro, A. Pena: In vivo 3-D Quantification of Melanin in Human Skin Through Multiphoton Microscopy and Image Processing
11:42–11:54 **CS72 P. L. Tong** (Australia), B. Roediger, R. Jain, W. Weninger: The Use of Transgenic Reporter Mice and Intravital Multiphoton Microscopy to Study Mast Cell Development in vivo
11:54–12:06 **CS73 P. Bhardwaj** (France), S. Brizion, E. Tancrede-Bohin, A. Potter, T. Baldeweck, A.-M. Pena, E. Huguet: Linking Skin Physiology Measurements to Skin Color by a Numerical Skin Color Model
12:06–12:18 **CS74 S. Thng** (Singapore), Y. F. Liang, Z. P. Lin, W. Ser, F. Lin, E. Gan, E. Tay: Automated Scoring of Melasma Using Computerized Digital Image Analysis of Clinical Photographs – A Pilot Study
12:18–12:30 **CS75 H. Liu** (People's Republic of China), F. Zhang: The Role of Reflectance Confocal Microscopy in the Evaluation of Hypopigmented Skin Disorders
12:30–12:42 **CS76 P. Singh** (India), P. Singh: Dermoscopy – A New Tool to Predict Stability in Vitiligo

Concurrent Session 21: Lasers and Light Devices for Pigmentary Conditions (Acacia Ballroom)
Chairs: C. L. Goh - T.Passeron - L. F. Xiang
11:10–11:30 **IL59 W. Manuskiatti** (Thailand): Laser Management of Common Pigmentary Problems in Asia
11:30–11:50 **IL60 C. L. Goh** (Singapore): Recent Advances on Laser Tattoo Removal
11:50–12:00 **CS77 H. S. Song** (Republic of Korea), M. Kim, W. J. Choi, H. Y. Kang: In vivo Time Sequential Histological Evaluation of Pigment and Melanocytes after Q-switched Alexandrite Laser Irradiation: Implication in Post-inflammatory Pigmentary Changes
12:00–12:10 **CS78 T. Shibata** (Japan): The Results and Side Effect of Q-switch Yag Laser Therapy for Various Skin Pigmentary Disorders
12:10–12:20 **CS79 H. S. Kim** (Republic of Korea), H. J. Jun, S. M. Kim, S. H. Cho, J. D. Lee: A Split-face Comparison of Erbium: YAG Micropeel vs. Q-Switched Nd:YAG Laser for the Treatment of Light Freckles and Lentigines in Asian Skin
12:20–12:30 **CS80 H. J. Kim** (Republic of Korea), J. H. Kim: Treatment of Periorbital Eczema Using Copper Bromide Laser in Atopic Dermatitis Patients
12:30–12:40 **CS81 J. M. Bae** (Republic of Korea), H. Kim, H. M. Jung, J. H. Lee, G. M. Kim: Nevus Depigmentosus Treated by the 308-nm Excimer Laser: A Retrospective Single-center Case Series

12:40–14:00 **Lunch Buffet (Island Ballroom)**

14:00–15:30 **Concurrent Sessions 22, 23, 24 (Tower Ballroom- Casuarina/Acacia/Banyan)**

© 2014 John Wiley & Sons A/S. Published by John Wiley & Sons Ltd

Concurrent Session 22: Hypopigmentary Disorders – Oculocutaneous Albinism & Others (Casuarina Ballroom)
Chairs: L. Montoliu - Y. Funasaka - S.Prihianti

14:00–14:20 **IL61 L. Montoliu** (Spain): Increasing the Complexity: New Genes and New Types of Albinism
14:20–14:40 **IL62 W. Li** (People's Republic of China): Mutation of SLC24A5 Causes OCA6
14:40–14:52 **CS82 K. Okamura** (Japan), J. Yoshizawa, Y. Abe, K. Hanaoka, N. Higashi, Y. Togawa, S. Nakagawa, N. Kambe, Y. Funasaka, K. Ohko, M. Kono, Y. Chinen, Y. Hozumi, T. Suzuki: Oculocutaneous Albinism in Japanese Patients: Seven Novel Mutations and a Case of OCA3
14:52–15:04 **CS83 M. Nishioka** (Japan), A. Tanemura, A. Tanaka, N. Arase, K. Teshima, H. Nishikawa, I. Katayama, S. Sakaguchi: Analysis for Regulatory T Cells Fraction and CCR4-expressing T Cells and Their Potential Function in Patients with Rhododenol-induced Leukoderma
15:04–15:16 **CS84 C. H. Ariyaratne** (Sri Lanka), K Satgurunathan, S. A. N. T. Sooriyaarachchi: Idiopathic Guttate Hypomelanosis: A Clinical and Histopathological Spectrum from National Hospital, Sri Lanka

Concurrent Session 23: Perspectives of Skin Colour (Banyan Ballroom)
Chairs: M. Verschoore - P. Grimes - W. K. Cheong

14:00–14:20 **IL63 R. Sarkar** (India): Impact of Pigmentary Disorders in Indian Patients
14:20–14:40 **IL64 F. Ly** (Senegal): Skin Bleaching in Sub Saharan African People: The Difficult Choice Between Beauty and Health
14:40–15:00 **IL65 A. Morita** (Japan): Environmental Factor and Pigmentation
15:00–15:10 **CS85 J. Na** (Republic of Korea): J. W. Shin, C. H, Huh, K. C. Park: Different Responses to UV According to Different Skin Color and Body Sites
15:10–15:20 **CS86 G. Cow** (Singapore), M. Sachdev, C.-J. Loy: Facial Skin Conditions among Southern Indian Women
15:20–15:30 **CS87 D. Murase** (USA), A. Hachiya, R. Hicks, S. Moriwaki, T. Hase, P. Manga: Variation in HSP70 Expression Contributes to Skin Color Diversity via Regulation of Melanogenesis

Concurrent Session 24: Strategies in Skin Lightening (Acacia Ballroom)
Chairs: K. C. Park - G. Lopez - H. Foong

14:00–14:20 **IL66 K. C. Park** (Republic of Korea): New Aspects of Melasma and Treatment
14:20–14:40 **IL67 K. Sato** (Japan): The Mechanisms of Skin Lightening Ingredients for Cosmetics
14:40–15:00 **IL68 E. Handog** (Philippines): Skin Lightening Agents: The Evidence and My Experience
15:00–15:10 **CS88 A. Damodaran** (India), N. Nair, R. Shariff, A. Damle, G. Chandramowli, P. Joshi, Krishnamuthy H, S. Vora: Multiple Modes of Action of Niacinamide in Regulating Skin Pigmentation
15:10–15:20 **CS89 E. Lazo** (Singapore), C. J. Loy: Hexyl Resorcinol for Spots and Skin Tone Lightening in Asian and Caucasian Skin
15:20–15:30 **CS90 C. C. Huang** (USA), K. Martinson, E. Butterbrodt, A. Monte: Identification of A11 as a Safer Skin Whitening Reagent Using Zebrafish Embryo

15:30–15:45 **Tea Break (Foyer of Tower Ballroom)**

15:45–17:00 **Plenary Session 9: Close of IPCC2014 (Acacia Ballroom)**
Chairs: M. Picardo - F. Meyskens

15:45–16:10 **SL4 Fitzpatrick Lecture – F. V. Filipp** (USA): Isozyme Shift in Cancer Metabolism
16:10–16:22 **CS91 V. Eleftheriadou** (UK), K. Thomas, A, Taieb, M. Picardo, K. Ezzedine: International Consensus on Core Outcomes Set for Vitiligo

16:22–16:42 **VGICC Report: A. Taieb**

16:42–17:00 **Closing Remarks & Announcing IPCC2017 – B. K. Goh & D. Norris**

Poster Listing

P001–P003: Developmental Biology

P001 T. Akiyama, T. Adachi, A. Shinomiya, K. Kinoshita, M. Nakano, M. Mizutani, Y. Matsuda (Japan): Functional Differentiation of the Endothelin 3-the Receptor Signal Transduction System in Pigmentation of Chicken
P002 A. Mishra, N. Srivastava (India): Epigenetic Stable Reprograming in Early Mammalian Development for the Disease Free Life
P003 H. Tabata, D. Nishihara, A. Koinui, H. Yamamoto (Japan): How is Regionalization of the Chicken Developing Eye Primordium Regulated?

P004–P009: Melanin Biophysics & Chemistry

P004 S. Choi, S. H. Bang, W. J. Lee, M. W. Lee, J. H. Choi, S. E. Chang (Republic of Korea.) N,N,N-trimethylphytosphingosine-iodide Decreases Melanin Synthesis Through ERK Activation in Human Melanocytes
P005 W. J. Choi, M. S. Kim, H. Y. Kang (Republic of Korea.) Pleiotrophin Inhibits Melanogenesis via ERK1/2-MITF Signaling in Normal Human Melanocytes

© 2014 John Wiley & Sons A/S. Published by John Wiley & Sons Ltd

P006 K. Ito, S. Ito, K. Wakamatsu (Japan) Weakly Acidic pH Suppresses Eumelanogenesis but Promotes Pheomelanogenesis

P007 R. Kishida, A. G. Saputro, H. Nakanishi, H. Kasai (Japan) Atomic-scale Understanding of Catalytic Behavior of Cu(Ii) Ion for Dopachrome Conversion

P008 K. Tabuchi, H. Tanaka, M. Ojika, F. A. Zucca, L. Zecca, S. Ito, K. Wakamatsu (Japan) Convenient Synthetic Method of Putative Degradative Markers to Identify Various Catecholic Metabolites in Addition to Dopamine and Norepinephrine Incorporated in Neuromelanin

P009 A. C. Zadlo, A. K. Pilat, M. W. Sarna, A. A. Broniec, T. J. Sarna (Poland) The Influence of Selected Transition Metal Ions on the Ability of Melanin to Decompose Hydrogen Peroxide

P010–P015: Melanosome Biogenesis & Transfer

P010 T. Adachi, A. Shinomiya, K. Kinoshita, M. Nakano, M. Mizutani, Y. Matsuda, T. Akiyama (Japan) A Retroviral Insertion in the Tyrosinase Gene (Recessive White; C/C) Induces White Plumage but not Effects on the Dermal Pigmentation in White Silky Chickens

P011 S. Choi, S. Y. Jo, J. E. Kim, M. W. Lee, J. H. Choi, S. E. Chang (Republic of Korea) Ep-2 Inhibits Melanin Synthesis and Melanosome Transfer Mediated by Protease-activated Receptor-2 in Keratinocyts

P012 H. Fujita, J. C. Menezes, S. M. Santos, S. Yokota, S. P. Kamat, J. A. Cavaleiro, T. Motokawa, T. Kato, M. Mochizuki, Y. Fujii, T. Fujiwara (Japan) Inulavosin and Its Benzo-derivatives Affect on Copper-loading Mechanism to Tyrosinase, A Key Enzyme of Melanin Synthesis in Melanocytes

P013 T.-C. Lei (People's Republic of China): UVA-induced Oxidative Stress Promotes the Degradation of Melanosomes in vitro

P014 C. H. Myung, J. Park, K. R. Lee, E. J. Oh, J. S. Hwang (Republic of Korea) Effect of Melanosome Transprot Inhibitors on Skin Pigmentation

P015 J. Pawelek, G. Diwakar, V. Klump, R. Lazova (USA) First Evidence for Glycosylation as a Regulator of the Pigmentary System: Key Roles of Sialyl(Alpha2,6)Gal/Galnac-terminated Glycans in Melanin Synthesis and Transfer

P016–P020: Intracellular & Metabolic Signaling

P016 M. Abrisqueta, C. Olivares, J. Sirés, J. C. García-Borrón, C. Jiménez-Cervantes (Spain) Human Melanocortin 1 Receptor-Mediated Ubiquitylation and Proteolysis of Nonvisual Arrestins

P017 T.-K. Kim, Z. Lin, W. Li, A. T. Slominski (USA) N1-Acetyl-5-methoxykynuramine (AMK) is Produced in The Epidermis from Melatonin and has Anti-proliferative Effects on Human Keratinocytes and Normal and Malignant Melanocytes

P018 L. Marrot, L. Denat, M. Dutordoir, Y. Phalente, C. Jones (France) Differences in Expression of Genes that Control Metabolism of Co-cultured Human Melanocytes and Keratinocytes. Modulation by Exposure to UV or Hydrogen Peroxide

P019 M. Randhawa, V. Sangar, S. Tucker-Samaras, M. Southall (Singapore) Metabolic Signature of Sun Exposed Skin Suggests Catabolic Pathways Overweighs Anabolic Pathways

P020 S. Terazawa, H. Nakajima, G. Imokawa (Japan) Withaferin a Abolishes the SCF-stimulated Pigmentation of Human Epidermal Equivalents by Interrupting C-Kit Autophosphorylation Through Dithiothreitol-suppressive Michael Addition Thioalkylation Reactions in Human Melanocytes

P021–P035: Regulation of Pigmentation

P021 N. Ando, R. Singh, S. Kaul, N. Nigam, C. Mahe, R. Wadhwa (Japan) Molecular Characterization of the Whitening Effect of Tranexamic Acid Cetyl Ester (TXC) Using Human Melanoma and Primary Melanocytes

P022 J. Sirés, R. Yousaf, S. Riazuddin, C. Jiménez-Cervantes, Z. M. Ahmed, J. C. García-Borrón (Spain) Functional Characterization of a Natural Human Melanocortin 1 Receptor Hypomorphic Mutant with Premature Truncation of the C-terminal Cytosolic Domain

P023 T. Hakozaki, T. Laughlin, S. Zhao, D. Deng, B. Jewellmotz, L. Moulton (USA) Myo-inositol Suppresses Melanogenesis Via Elevation of Cell Energy in Keratinocytes

P024 Y. Higashi, M. Yagi, M. Ichihashi, H. Ando (Japan) Evaluation of Melanogenesis Inhibitory Agent by a Novel Human Melanocyte-Keratinocyte Co-culture System

P025 M. J. Kang, J. W. Byun, H. W. Park, H. B. Lee, E. K. Kim, J. Shin (Republic of Korea) A Study on the Mechanism of Stimulating Effect of Cryptanthus on Mouse Melanoblast

P026 M. Kawaguchi, Y. Hozumi, T. Suzuki (Japan) Adam Protease Inhibitor Modulates Melanogenesis in Human Melanocytes

P027 D.-S. Kim, H. Li, M. C. Balcos, J. S. Shin, H.-Y. Yun, K. J. Baek, N. S. Kwon, K.-C. Park (Republic of Korea) Myriocin, a Serine Palmitoyltransferase Inhibitor, Increases Melanin Synthesis in Mel-Ab Cells and a Skin Equivalent Model

P028 D.-S. Kim, H. Li, J. Kim, H.-G. Hahn, J. Yun, H.-S. Jeong, H.-Y. Yun, K. J. Baek, N. S. Kwon, K.-C. Park (Republic of Korea) KHG26792 Inhibits Melanin Synthesis in Mel-Ab Cells and a Skin Equivalent Model

P029 T. Mann, W. Gerwat, H. Wenck, K.-H. Röhm, L. Kolbe (Germany) Mode of Action of Rhododendrol, Raspberry Ketone, 4-Butylresorcinol and Other Inhibitors of Human Tyrosinase

P030 A. P. W. W. Kumarasinghe, S. P. W. Kumarasinghe (Australia) Pintoid Dyschromia of Yaws: A Rare Presentation of a Chronic Infectious Disease

P031 T. Motokawa, T. Miwa, M. Mochizuki, M. Itou, K. Yokoyama (Japan) Novel Role of Adrenomedullin in Melanocyte Dendrite Branching

P032 D. J. Tobin, A. Richharia (UK) Expression of Clock Proteins Clock and Per1 in Human Skin Melanocytes, and the Impact of Alternate Light and Dark Cycles In Vitro

P033 R. Wadhwa, N. Widodo, N. Shah, N. Nigam, T. Yaguchi, N. Ando, C. Mahe, S. Kaul (Japan) Integrated Cell Based Shrna Screening and Bioinformatics Approaches Identified Mitochondrial Stress Chaperones Hsp60 and Mortalin as New Regulators of Melanogenesis

© 2014 John Wiley & Sons A/S. Published by John Wiley & Sons Ltd

P034 C. Wong, R. Minocha, N. Sharma, R. Dunn, C. Grills, N. Grills (Australia) The Prevalence of Skin Whitener Usage in Northern India and Its Related Dermatological Complications

P035 R. Minocha, C. Wong, N. Sharma, R. Dunn, C. Grills, N. Grills (Australia) The Overuse of Systemic and Topical Corticosteroid for Dermatological Conditions in India: A Case Series and Review of the Literature

P036–P040: Melanocyte UV Response & DNA Repair

P036 S. A. Ainger, H. X. Yong, J. H. Leonard, R. A. Sturm (Australia) UVR and ROS Damage Protection Mediated by DCT Expression in Human Melanocytes

P037 A. Banjar, B. S. Feltis, P. F. Wright, G. M. Boyle, T. J. Piva (Australia) The Effect of Antioxidants on UV–IR-radiated Melanocyte-derived Cells

P038 T. Budden, N. Bowden (Australia) Melanoma Exhibits Defective Nucleotide Excision Repair of UVB-induced DNA Photoproducts

P039 M. D. Galibert (France) The Usf1 and P53 Transcription Factors Coordinate the Skin Response to UV-induced DNA-damage

P040 Y. Won, C.-J. Loy, T. Oddos, H.-K. Wong, S. Kaur, M. Randhawa, M. Southall (Singapore) 4-Hexylresorcinol Improves Skin Hyperpigmentation in a Double-blinded, Placebo- and Randomized-controlled Clinical Trial

P041–P043: Genetics of Pigmentation

P041 V. Laville, S. Le Clerc, K. Ezzedine, R. Jdid, L. Taing, T. Labib, C. Coulonges, D. Ulveling, P. Rucart, W. Carpentier, I. Berlin, P. Galan, S. Hercberg, C. Guinot, F. Morizot, J. Latreille, E. Tschachler, J.-F. Zagury (France) A Genome Wide Association Study in a Caucasian Cohort Reveals a Genetic Association Between the Hla-C*0701 Allele and Solar Lentigines

P042 Y. Zhuang, V. Hariharan, J. Lyga, U. Santhanam (USA) Gene Array Analyses of Age Spot Uncovered New Bio-functions

P043 H. Shibuya, H. Yamamoto (Japan) Do Melanocytes Contribute to the Structure of Their Niches?

P044–P048: Human Skin Colour and Skin Optics

P044 C. Marionnet, S. Nouveau, V. Hourblin, K. Pillai, M. Manco, S. Del Bino, P. Bastien, C. Tran, C. Tricaud, O. de Lacharrière, F. Bernerd (France) UVA1 Exposure Leads to Darken Human Skins of Different Constitutive Pigmentation Together with a Molecular Biological Impact

P045 S. Kaul, R. Gao, N. Nigam, L. Li, R. Wadhwa (Japan) Ashwagandha-derived Phytochemicals Possess Skin Whitening Potential: Evidence from Human Cell Based Assays

P046 E. C. Murzaku, S. Hayan, B. K. Rao (USA) Methods and Rates of Dermoscopy Usage: A Cross-Sectional Survey of United States Dermatologists Stratified by Years in Practice

P047 B. Rao, E. Murzaku (USA) Confocal Microscopy for Managing Pigment Cell Disorder and Neoplasms

P048 T. Yamaoka, Y. Nagata, A. Tanemura, E. Ono, A. Tanaka, K. Kato, M. Yamada, I. Katayama (Japan) Color Assessment of Progressive Nonsegmental Vitiligo under Short-term Intravenous Methylprednisolone Pulse Therapy

P049: Melanocyte & Stem Cell Biology

P049 T. Kawakami, A. Ohgushi, T. Baba, T. Hirobe, Y. Soma (Japan) Effects of 1, 25-dihydroxyvitamin D3 on Human Epidermal Melanocytes and Melanoblasts

P050–P051: Hair Biology & Pigmentation

P050 H. Shin, H. H. Ryu, M. Choi, O. Kwon, S. J. Jo (Republic of Korea) Risk Factors for Premature Hair Graying: Family History, Smoking, and Obesity

P051 S. Tripathy (India) Psychosocial Impact of Premature Canities in a Tertiary Care Centre

P052–P053: Model Systems for Pigment Cell Biology & Disease

P052 M. Salducci, N. André, C. Guéré, M. Martin, R. Fitoussi, K. Vié, M. Cario-André (France) Cistus Monspeliensis Bio Extract Reduce Solar Lentigo Induction by Factors Secreted by Irradiated Aged Fibroblasts, in Pigmented Reconstructed Epidermis

P053 T. Kuramoto, M. Yokoe (Japan) Genetic Fine Mapping of a Rat Dominant Ventral Spotting Gene, Downunder (Du)

P054: Translational Skin Biology

P054 E. C. Eksteen, P. F. Coetzee, M. J. Bester (South Africa) Melanocytes and Melanotropin in Skin Wound Healing and Keloid Pathogenesis

P055–P071: Melanoma Biology

P055 K. Beaumont, W. Weninger, N. Haass (Australia) RAB27a as a Novel Therapeutic Target for Melanoma

P056 H. Breiteneder, D. Pucciarelli, S. Kontsekova, M. Takáčová, F. Juliano, N. Lengger, L. Csaderova, S. Pastorekova, C. Hafner (Slovakia) Response of Melanoma Cell Lines to BRAF V600 Inhibitor Treatment under Hypoxic Conditions is Cell Line Specific

P057 H.-Y. Cheng, C.-E. Wu, J.-J. Hsieh, Y.-C. Shen, C.-H. Hsieh, M.-M. Hou, W.-K. Huang, T.-T. Kuo, Y.-F. Lo, T. Hsu, J. W.-C. Chang (Taiwan ROC) Identification of Novel Prognostic and/or Therapeutic Targets in Acral Lentiginous Melanoma by Microarray Analysis

P058 Y.-L. Cheng, C.-H. Hsieh, L.-Y. Lee, C.-E. Wu, W.-K. Huang, Y.-F. Lo, J.-J. Hsieh, H.-Y. Cheng, Y.-C. Shen, Y.-C. Lin, J. W.-C. Chang (Taiwan ROC) Concomitant Blockade of BRAF and MTOR Pathway might be Required for Effective Inhibition of Malignant Melanoma

P059 A. Koch, A. Bosserhoff, C. Hellerbrand (Germany) Glucose Transporter Isoform 1 (Glut1) Expression Determines Tumorigenicity of Melanoma Cells and Their Potential to Form Hepatic Metastasis

© 2014 John Wiley & Sons A/S. Published by John Wiley & Sons Ltd

P060 T. Hida, K. Wakamatsu, T. Yamashita (Japan) Detection of Circulating Melanoma Cells in Melanoma Patients

P061 C.-H. Hsieh, Y.-L. Cheng, L.-Y. Lee, C.-E. Wu, W.-K. Huang, Y.-F. Lo, J.-J. Hsieh, H.-Y. Cheng, Y.-C. Shen, Y.-C. Lin, J. W.-C. Chang (Taiwan ROC) The Correlation Between Survival and Expression Status of BRAF, Targets of mtor, Cyclin-dependent Kinase 4 and Cyclin D1 in Patients with Acral Lentiginous Melanoma in Taiwan

P062 J.-J. Hsieh, J. W.-C. Chang, H.-Y. Cheng, Y.-C. Shen, C.-E. Wu, C.-H. Hsieh, W.-K. Huang, T. Hsu (Taiwan ROC) LKB1 Gene Alterations in Acral Lentiginous Melanoma

P063 O. Kodet, J. Kucera, L. Lacina, B. Dvorankova, K. Smetana (Czech Republic) Cancer Associated Fibroblasts Influence Tumor Microenvironment of Melanoma

P064 J. Kučera, O. Kodet, L. Lacina, B. Dvorankova, M. Kolář, H. Strnad, K. Smetana (Czech Republic) Tumor Melanocytes are Responsible for Pseudohyperplasia of Epidermis in Nodular Melanoma: In Vivo and In Vitro Study

P065 D. V. Nikolic, A. T. Nikolic, V. V. Stanimirovic, J. Gacic (Serbia) Benefit of Using Dermoscopy in Revealing Thin Melanomas in Southestern Europe

P066 D. V. Nikolic, A. T. Nikolic, V. V. Stanimirovic, J. Gacic (Serbia) World Wide Web E-melanoma Register: Myth or Reality

P067 M. Sarna, A. Zadlo, K. Burda, T. Sarna (Poland) Cell Elasticity is an Important Indicator of the Invasive Abilities of Pigmented Melanoma Cells

P068 N. Maddodi, J. Berlyn, T. Shekhani, S. Rajguru, J. B. Longley, M. R. Albertini, W. Huang, M. A. Newton, V. Setaluri (USA) Prognostic Significance of MIRNA-211 in Cutaneous Melanoma

P069 Y.-C. Shen, J. W.-C. Chang, J.-J. Hsieh, H.-Y. Cheng, M.-M. Hou, C.-H. Hsieh, W.-K. Huang, C.-E. Wu, T. Hsu (Taiwan ROC) Incidence of BRAF and C-Kit Mutations in Taiwanese Melanoma

P070 P. Szabo, B. Dvorankova, K. Smetana, H. Strnad, M. Kolar (Czech Republic) Tumor Type Unspecific Biological Activity of Cancer Associated Fibroblast on Malignant Epithelial Cells

P071 T.-L. Wu, W.-K. Huang, J. W.-C. Chang, J.-J. Hsieh, H.-Y. Cheng, C.-H. Hsieh, C.-E. Wu, T.-T. Kuo, K.-Y. Yeh (Taiwan ROC) Comparison of Immunohistochemical Analysis and DNA Sequencing in the Detection of the BRAF V600e Mutation among Asian Patients with Primary and Metastatic Melanoma

P072: Senescence Pathways to Melanoma

P072 R. Hsieh, C. M. Coutinho-Camillo, J. D. Fernandes, M. M. S. Nico, S. V. Lourenco (Brazil) NRAS and BRAF Mutation and Protein Expression Analysis in a Series of Primary Oral Mucosal Melanoma

P073–P074: UV & Non-UV Pathways to Melanoma

P073 F. L. Jr Meyskens, F. Liu-Smith, S. Yang (USA) Source of Reactive Oxygen Species and Their Roles in Melanoma Etiology

P074 K. Sato, T. Nishio, T. Mito, K. Matsui, J. N. Sato, H. Watanabe (Japan) The Mechanism of Blue Light-Induced Cell Death in Melanoma Cells

P075: Genetics & Genomics of Melanoma

P075 A. Golovko, J. M. Völker, L. Andersson (Sweden) SNP Analysis of Kit Gene in Melanoma of Grey Horses

P076–P085: Melanoma Therapeutics

P076 P. Cassidy, M. Honeggar, S. Leachman, P. Moos (USA) The Roles of Thioredoxin Reductase 1 in Melanomagenesis

P077 J. W.-C. Chang, C.-E. Wu, C.-H. Hsieh, W.-K. Huang, C.-J. Chang, J.-T. Yeh, C.-H. Yang, Y.-F. Lo, K.-J. Lin, J.-J. Hsieh, T.-T. Kuo (Taiwan ROC) In-transit Sentinel Lymph Node for Melanoma Patients in Taiwan

P078 H. Cheng, C.-H. Hsieh, J. Wen-Cheng Chang, S.-P. Yeh, L.-Y. Ou, H.-Y. Cheng, J.-J. Hsieh, Y.-F. Lo (Taiwan ROC) Result of Efficacy and Safety of Dabrafenib in BRAF V600 Mutated Melanoma in Taiwan

P079 H. Fathan, I. Widyasari, A. U. Hasanah, H. Cipto, A. S. Dahlan Suriadiredja, I. A. Krisanti, L. Paramita, A. T. Sampurna (Indonesia) Nodular Type of Malignant Melanoma, Nodes and Lung Metastasis with Good Medical Status

P080 W.-K. Huang, C.-E. Wu, C.-H. Hsieh, J.-H. Liu, C.-C. Lin, C.-Y. Lin, T.-C. Liu, W.-C. Su, Y.-M. Yeh, J.-J. Hsieh, J. W.-C. Chang (Taiwan ROC) The Safety and Efficacy of Ipilimumab in Patients with Advanced Melanoma in a Taiwanese Cohort of the Ipilimumab Expanded Access Program

P081 S. Kontsekova, D. Pucciarelli, N. L. Lengger, C. Hafner, H. Breiteneder (Slovakia) Anti-Cspg4-specific Antibodies Enhance the Effects of the BRAF Inhibitor Plx4032 on Melanoma Cells

P082 F.-J. Lai, C.-C. Hsieh, R.-K. Lin, C.-L. Cheng, N.-S. Chang, C.-C. Lin (Taiwan ROC) Novel Copper (Ii)-based Photosensitisers Induce Phototoxicity Towards Melanoma Cells in vitro

P083 D. R. Menon, C. Krepler, A. Vultur, B. Rinner, G. Zhang, R. Somasundaram, G. Hoefler, M. Herlyn, P. H. Soyer, B. Gabrielli, H. Schaider (Australia) An Early Innate Response Resulting in Melanoma Cancer Stemness Precedes Acquired Drug Resistance

P084 C.-E. Wu, C.-H. Hsieh, W.-K. Huang, C.-J. Chang, J.-T. Yeh, T.-T. Kuo, Y.-F. Lo, C.-H. Yang, K.-J. Lin, J.-J. Hsieh, H.-Y. Cheng, J. W.-C. Chang (Taiwan ROC) Sentinel Lymph Node Biopsy might Improve Survival Outcomes Patients with Clinically Node-negative Cutaneous Melanoma in Taiwan

P085 M. Ziman, M. Millward, D. Klinac, J. Freeman, A. Reid, S. Bowyer, E. Gray (Australia) Monitoring Changes in Circulating Tumour Cells as a Prognostic Indicator of Overall Survival and Treatment Response in Patients with Metastatic Melanoma

P086–P100: Hyperpigmentary Disorders

P086 U. Alagappan, B. K. Goh (Singapore). Pigmentary Mosaicism (checkerboard) of a Becker's Naevus

P087 H. M. Cheng, S. P. Kumarasinghe (Australia). Amlodipine-induced Acquired Hyperpigmentation: A Case Report

© 2014 John Wiley & Sons A/S. Published by John Wiley & Sons Ltd

P088 S. Chua, M. M. F. Chan, H. Y. Lee (Singapore) Ashy Dermatosis (Erythema Dyschromicum Perstans) Induced by Omeprazole: A Report of 3 Cases

P089 S. Y. Chuah, H. H. Ghorbel, J. P. Lacour, T. Passeron (Singapore) Acquired Dermal Melanocytosis of the Nose – Five Unsual Cases

P090 K. Debbarman, S. Sethi, V. K. Garg (India) A Case of Hyperpigmentation along Blaschkoid Lines: Case Report with a Brief Review of Literature

P091 V. Piffaut, S. Nouveau, C. Duval, P. Bastien, B. Chalmond, F. Bernerd, O. de Lacharrière (France) Semiology of Actinic Lentigines: An Intrinsic Heterogeneity Observed by Dermatoscopy and Image Analysis

P092 K. Karunaratne, M. Dissanayake, J. Liyanage (Sri Lanka) Palmar Pigmentation; a Study of 3 Cases with Clinical Variation

P093 J. E. Kim, E. J. Kim, H. Hur, J. Y. Ko, Y. S. Ro (Republic of Korea) Treatment of Café Au Lait Macules Using 1064 Nm Q-switched Nd:Yag Laser : What is the Golden Parameter?

P094 E. G. Lee, H. J. Lee, D. H. Kim, M. S. Yoon (Republic of Korea) Hypohidrotic Ectodermal Dysplasia with Acquired Dermal Melanosis

P095 C. C. Oh, W. Y. Tham, H. Y. Koh (Singapore) Hyperpigmentation with Systemic Symptoms in an Asian Female

P096 T. Shibata, M. Ichihashi (Japan) The Evaluation of Various Therapies for Congenital and Aquired Skin Hypopigmentary Disorders

P097 A. W. Tan, J. Common, B. Lane, J. S. Lee, B. K. Goh (Singapore) Galli–Galli Disease Associated with a Frameshift Mutation in Keratin 5 Gene

P098 A. Trivedi, I. de la Serna (USA) The Role of BRD4 in Melanogenesis

P099 M. T. Truchuelo, N. Jiménez, M. Vitale (Spain) Assessment of the Efficacy and Tolerance of a New Combination of Retinoids and Depigmenting Agents in the Treatment of Melasma

P100 S. S. Yang, H. Jaffar, D. C. W. Aw (Singapore) A Purpuric Variant of Acute Graft Versus Host Disease

P101–P110: Melasma

P101 M. Abdallah, A. Alhefnawy, D. Altantawi (Egypt) Comparative Study of Trichloroacetic Acid (15%) Versus Fractional Er:Yag (2940 Nm) in the Treatment of Epidermal Melasma

P102 S. Bansal, R. Sarkar, V. K. Garg (India) Glycolic Acid and Salicylic Acid Peels in Melasma

P103 S. Dayal (India) Clinical Evaluation of Efficacy of Lactic Acid Peeling in Combination with a Topical Regimen in the Treatment of Melasma

P104 S. Mangal, D. Parsad (India) Tranexamic Acid Microinjections in Melasma- A Pilot Study

P105 J. I. Na, J. W. Shin, H. R. Choi, K. C. Park (Republic of Korea) The Efficacy of 4N-butyl Resorcinol and Resveratrol Containing Cream on Melasma: Comparison with Various Whitening Agents Using Ratio of Lesional/Non-lesional Melanin Index

P106 N. Puri (India) Comparative Study of 70% Glycolic Acid Versus 35% TCA Versus 1% Tretinoin Peel for the Treatment of Melasma

P107 S. Sethi, R. Sarkar, V. K. Garg (India) Combination Phytic Peels Versus Glycolic Acid Peels in the Treatment of Melasma in Dark-skinned Patients

P108 M. K. Shin, M.-Il Bae, M.-H. Lee (Republic of Korea) The Impact of Different Fluence for Intense Pulsed Light in the Treatment of Melasma

P109 M. Song, J.-M. Park, H.-S. Kim, H.-C. Ko, B.-S. Kim, M.-B. Kim (Republic of Korea) Tranexamic Acid for Treatment of Melasma in Korean Patients: A Preliminary Clinical Trial

P110 V. Vachiramon, S. Sahawatwong, P. Sirithanabadeekul (Thailand) Treatment of Melasma in Men with Low-fluence Q-switched Neodymium-doped Yttrium Aluminum Garnet Laser vs. Combined Laser and Glycolic Acid Peeling

P111–P114: Hypopigmentary Disorders

P111 Q. Chen, S. S. J. Lee, T. G. S. Thng, S. H. Chua (Singapore) A Case of Generalized Hypopigmentation and Paradoxically Darkening of Hori's Nevus in a Patient on Imatinib

P112 T. Yamashita, M. Okura, Y. Ishii-Osai, M. Yoshikawa, Y. Sumikawa, K. Wakamatsu, S. Ito (Japan) Production of Reactive Oxygen Species and Anti-oxidative Responses in Rhododendrol- treated Human Melanocytes

P113 L. Yang, M. Wataya-Kaneda, F. Yang, A. Tanemura, N. Arase, I. Katayama (Japan) 4-(4-hydroroxyphenyl)-2-butanol (Rhododendrol) Activates Autophagy-lysosome Pathway in Melanocytes: A Potential Mechanism for Skin Depigmentation Disorder

P114 Y. W. Yeo, B. K. Goh (Singapore) Hypochromic Pityriasis Lichenoides Chronica and Its Association with Mycosis Fungoides

P115–P134: Vitiligo Research

P115 E. Bastonini, D. Kovacs, M. Ottaviani, M. L. Dell'Anna, M. Picardo (Italy) Vitiligo: Focusing on the Involvement of the Dermal Compartment

P116 L. Benzekri, Y. Gauthier (Morocco) Evidence for a Decreased Adhesiveness of Melanocytes in Vitiligo Skin: A Preliminary and Experimental Assessment

P117 L. Chandrashekar, M. Rajappa, K. S. Rajendiran, M. Munisamy, D. M. Thappa, P. H. Ananthanarayanan (India) Aquaporin-3 in Patients with Non-segmental Vitiligo and Its Association with Oxidative Stress and Disease Activity

P118 K. Ezzedine, J. Seneschal, C. Salzes, A. Taieb, C. Taieb (France) Developing a Questionnaire to Evaluate the Burden of Vitiligo

P119 K. Ezzedine, N. van Geel, M. Picardo, A. Taieb (France) Web-based Vitiligo Registry

P120 M. Inoue, K. Yamasaki, T. Yamauchi, S. Koike, A. Watabe, K. Kikuchi, S. Aiba (Japan) Comparison of Vitiligo Vulgaris and Rhododenol-induced Vitiligo by Multiband Digital Camera with Multiple Linear Regression Analysis

P121 S. Jadeja, M. S. Mansuri, M. Singh, A. Yeola, N. C. Laddha, M. Dwivedi, R. Begum (India) Association of LMP7 and TAP1 Polymorphisms with Vitiligo Susceptibility in Gujarat Population

© 2014 John Wiley & Sons A/S. Published by John Wiley & Sons Ltd

P122 H. Khurrum, K. Alghamdi (Saudi Arabia) A Pilot Trial Evaluating the Treatment of Focal Vitiligo with Intralesional Bee Venom
P123 H. Khurrum, K. Alghamdi (Saudi Arabia) Screening for Glaucoma Prevalence in Vitiligo Patients as Compared to Healthy Control Group
P124 L. Komen, V. da Graca, A. Wolkerstorfer, C. B. Terwee, M. A. de Rie, W. J. van der Veen (The Netherland) The VASI and the VETF Assessment: Reliable and Responsive Instruments to Measure the Degree of Depigmentation in Vitiligo
P125 R. Kumar, D. Parsad (India) Senescence in Keratinocytes and Fibroblasts from Perilesional Skin of Unstable Vitiligo Patients
P126 T.-C. Lei (People's Republic of China): Depigmented Epidermis in Vitiligo Patients Shows an Aberrant Ultrastructure of the Stratum Corneum Lipid Lamellar Membranes
P127 M. S. Mansuri, M. Singh, N. C. Laddha, M. Dwivedi, S. R. Bhalara, Y. S. Marfatia, R. Begum (India) Identification of Potential Micrornas Responsible for Pathogenesis of Vitiligo
P128 J. H. Shin, H. Y. Kang, K. H. Kim, C. J. Park, S. H. Oh, S. C. Lee, S. H. Lee, G. S. Choi, S. K. Hann (Republic of Korea) An Immunohistochemical Study on Segmental Vitiligo; A Joint Study of the Korean Vitiligo Association
P129 A. Singh, R. Chander, V. Mendiratta, R. Singh, A. Sharma (India) Vitiligo and Metabolic Syndrome: A Case Control Study
P130 A. Singh, R. Chander, V. Mendiratta, R. Singh, A. Sharma (India) Are Vitiligo Patients at a Higher Risk of Developing Atherosclerosis? – A Pilot Study
P131 M. Singh, M. S. Mansuri, N. P. Raval, N. C. Laddha, M. Dwivedi, R. Begum (India) Association of Interleukin 6 -572 G/C and -174 G/C Promoter Polymorphisms with Vitiligo Susceptibility in Gujarat Population
P132 S. Tanwar, D. Parsad, A. Bhatia (India) Altered Gene Expression, Oxidative Stress and Melanocytorrhagy in Vitiligo Pathogenesis
P133 A. M. Tawdy, S. M. Tahlawy, S. O. Tawfeek, N. A. Bedair (Egypt) Corticotrophin Releasing Hormone (Crh) and Its Receptor (Crhr1) Gene Expression as a Component of Skin Hpa Axis in Response to Stress in Vitiligo
P134 M. K. Tembhre, V. K. Sharma, S. Gupta, A. Sharma, A. S. Parihar (India) Regulatory T Cell (Treg) and T Cell Immunoglobulin and Mucin Domain (Tim) Mediated Immune Regulation in Active Generalized Vitiligo

P135–P143: Vitiligo: Clinical

P135 S. Aminpoojary, J. Bagadia (India) Vitiligo in a Case of Systemic Lupus Erythematosus: A Concurrent Course
P136 R. F. Dwiyana, D. Theodesia, E. Sutedja (Indonesia) The Correlation Between Serum Level of 25-hydroxyvitamin D and Clinical Improvement in Vitiligo Patients after NB-UVB 311 nm Phototherapy
P137 K. Ezzedine, J. Seneschal, C. Salzes, A. Taieb, C. Taieb (France) Skin Phototype may Influence the Burden of Vitiligo
P138 B. K. Khaitan (India) A Study to Validate the Proposed Clinical Criteria for Diagnosis of Segmental Vitiligo
P139 H. Khurrum, K. Alghamdi (Saudi Arabia) Is there a Real Relationship Between Serum Level of Homocysteine and Vitiligo?
P140 M.-J. Gwak, E.-J. Shin, J.-H. Choi, K.-H. Jeong, M. K. Shin, M.-H. Lee (Republic of Korea) A Case of Vitiligo Associated with Meniere's Disease
P141 A. Pandya, J. J. Sosa, S. Currimbhoy (USA) Confetti Depigmentation as a Disease Marker in Patients with Generalized Vitiligo
P142 A. Pandya, S. Currimbhoy, V. Zhu (USA) Patient Satisfaction with Treatment Modalities for Vitiligo
P143 D. Patel (India) Epidemiology of Vitiligo in Surat, GJ, India

P144–P150: Vitiligo Therapeutics

P144 M. Araujo, P. Avila, P. Avila, M. F. Araujo, P. F. Avila (Brazil) The Relation Between Vit B12 Levels and Vitiligo's Repigmentation
P145 J. M. Bae, H. Kim, C. Y. Won, J. H. Lee, G. M. Kim (Republic of Korea) The Effectiveness of 308-Nm Excimer Laser for Segmental Vitiligo: Retrospective Study of 159 Patients
P146 K. Chouhan, A. Kumar (India) Role of Hair Transplantation in Vitiligo Treatment
P147 A. Jha (India) Devicing an Intervention Module to Reduce Psychosocial Stress in Patients with Vitiligo
P148 G. Khullar, A. J. Kanwar, S. Singh, D. Parsad (India) Comparison of Efficacy and Safety Profile of Topical Calcipotriol Ointment in Combination with Nb-Uvb Versus Nb-Uvb Alone in the Treatment of Vitiligo: A 24-week Prospective Right-left Comparative Clinical Trial
P149 T. Maekawa, E. Maehara, N. Wada, H. Takamatsu, K. Harada, K. Urabe (Japan) Depigmentation Therapy for Normal Skin in Vitiligo Universalis with Topical Application of Tacalcitol and 4-N-butylresorcinol
P150 S. T. G. Thng, A. Chang (Singapore) Comparison of the 308 Excimer Lamp Versus Home Phototherapy in the Treatment of Focal Vitiligo

P151–P165: Vitiligo Surgery

P151 M. Abdallah, H. Diab, N. N. El-Din (Egypt) Efficacy of Er:Yag Laser, Nb-Uvb and Topical Corticosteroids in Resistant Vitiligo Patches
P152 T. Anbar, T. El-Ammawy, Y. El-Sherbeny, E. Saad, S. Salah (Egypt) Partial Thickness Epidermal Cuts Versus Suction Blister Roofs as Sources of Preparing Non-cultured Epidermal Suspension for Vitiligo Treatment: A Comparative Study
P153 A. Budania, N. Khunger (India) Non Cultured Epidermal Cell Suspension for Stable Vitiligo from Lab to Dermatologist's Clinic: An Oversimplification or a Real Possibility
P154 D. H. Ghia, S. Mulekar (India) Vitiligo Surgeries – Modifications to Simplify the Technique
P155 D. H. Ghia, S. Mulekar (India) Uncommon Responses of Segmental Vitiligo to Melanocyte Keratinocyte Transplantation Procedure
P156 V. Keshavamurthy, S. Dogra, D. Parsad (India) Clinical and Treatment Characteristics Determining Therapeutic Outcome in Patients Undergoing Autologous Non Cultured Outer Root Sheath Hair Follicle Cell Suspension for Treatment of Stable Vitiligo
P157 L. Komen, C. Vrijman, R. M. Luiten, E. P. Tjin, M. A. de Rie, W. J. van der Veen, A. Wolkerstorfer (The Netherlands) Autologous Cell Suspension Transplantation Using Recell in Segmental Vitiligo and Piebaldism Patients: A Randomised Controlled Pilot Study

 © 2014 John Wiley & Sons A/S. Published by John Wiley & Sons Ltd

P158 I. Majid, V. Mysore, T. Salim, K. Lahiri, S. Talwar, N. Khunger, S. Burua, Y. N. Sacchidanand (India) Is Lesional Stability more Important than Disease Stability for Performing Grafting Procedures in Vitiligo? Results from a Multi-centric Study
P159 I. Majid (India) Ultra-thin Skin Grafting in Resistant Stable Vitiligo: Factors Affecting the Treatment Outcome
P160 I. Majid (India) Smash Grafting as an Alternative to Non-culture Epidermal Cell Suspension (NCES) Technique in Resource Poor Settings
P161 A. A. J. Merli, E. Serra, V. Montenegro (Argentina) Treatment in Segmental Vitiligo with Autologous Skin Micrografting
P162 M. Pascal, H. Maillard, M. Ghasemi, R. Ghasemi, K. Ezzedine (France) Innovative Autologous Non-cultured Epidermal Cellular Graft for Segmental and Non-segmental Vitiligo
P163 M. Pascal, M. Ghasemi, R. Ghasemi, K. Ezzedine (France) Treatment of Difficult-to-treat Vitiligo Lesions Using a New Autologous Non-cultured Epidermal Cellular Grafting Kit
P164 G. Verma, H. K. Kar, R. Rani (India) A Comparative Study of Repigmentation in Stable Vitiligo after Autologous Cultured Melanocytes Transplantation and Non-cultured Epidermal Suspension Transplantation
P165 D. Wang, B. K. Goh (Singapore) Correction of Cobblestoning Due to Punch Grafting by Non-cultured Cellular Grafting

P166–P167: Albinism & Related

P166 V. Dewi, R. Danarti, C. C. T. Rosari, S. Radiono (Indonesia) Oculocutaneous Albinism and Gorlin Syndrome: A Coincidence?
P167 M. Shimanuki, G. Tamiya, Y. Abe, Y. Hozumi, T. Suzuki (Japan) Positive Selection with Diversity in Oculocutaneous Albinisms Type 2 Gene (Oca2) among Japanese

P168–P176: Skin Lightening Therapies

P168 H. Choi, Y.-A. Kang, H. Lee, K. Park (Republic of Korea) Disulfanyl Peptide Decreases Melanin Synthesis via Receptor-mediated ERK Activation and the Subsequent Down-regulation of MITF and Tyrosinase
P169 E. Flori, E. Bastonini, S. Briganti, D. Kovacs, G. de Paoli Ambrosi (Italy) An Innovative and Safe Therapy for Hyperpigmentary Disorders
P170 J. Eby, I. Wirjan, K. Willenborg, C. Le Poole (USA) Melanoproteins Mediating the Cytotoxic Activity of Bleaching Phenols
P171 H. Nojiri, Y. Kuo, Y. Han, W. Chen, K. Ishida, T. Nishizaka, T. Hisateru, G. Imokawa (Japan) Efficacy of Intense Pulsed Light for Treating Several Types of Pigmented Spots on the Face in Combination with an Inhibitor of Melanogenic Intracellular Signaling
P172 Z. Draelos, S. Raab, M. Yatskayer, N. Chen, Y. Krol, C. Oresajo (USA) Maintenance of 4% Hydroquinone + 0.025% Tretinoin Clinical Results with a Novel Cosmeceutical Formulation During the Summer Months
P173 K.-C. Park, S.-H. Kwon, S.-Y. Byun, C.-H. Huh, J.-I. Na, H.-O. Kim, C. Galzote (Republic of Korea) Efficacy and Safety of a Soy Extract and Niacinamide Cosmetic Serum to Improve Skin Hyperpigmentation in Korean Women
P174 M. Randhawa, J. Fantasia, J. Thakrar, S. T. Samaras (Singapore) Sustained Anti-hyperpigmentation Benefits with Daily Application of Stabilized 0.1% Retinol Formula
P175 E. Tancrède-Bohin, A.-M. Pena, N. Parent, S. Brizion, S. Victorin, E. Decencière, M. Bagot, T. Baldeweck (France) Non Invasive and Short Term Assessment of Retinoids Effects on Melanin Content Using in vivo Multiphoton Microscopy
P176 E. Cestone, F. Marzatico, V. Nobile, M. Vitale (Spain) Placebo-controlled Study of the Efficacy of a Dietary Supplement to Reduce Skin Dark Spots and to Create more Even Complexion in Asian Women
P177 J. Waugh, S. Tan (USA) Mitochondrial Depletion via Nonactivating MSH Conjugate Leads to Reduction in Pigment

P178: Paediatric Pigmentary Disorders

P178 Y. Kaku, H. Takeshita, T. Ito, E. Itou, M. Furue, K. Urabe (Japan) Combined Plaque-type Blue Nevus on Café-Au-Lait Spot

P179: Lasers and Light Interventions

P179 D. V. Nikolic, A. T. Nikolic, V. V. Stanimirovic, S. K. Vranic (Serbia) Use of Polarised Light Therapy after Skin Tumor Surgery

P180–P187: Photoprotection & Photocarcinogenesis

P180 C. Ding, Y. Liu, M. Hong, R. Wang, E. Planel, Z. Cai (People's Republic of China): Daily Ultraviolet Radiation Induced Gene Expression Modulation in a Reconstructed Asian Skin Model
P181 E. Flori, A. Mastrofrancesco, D. Kovacs, S. Briganti, V. Maresca, G. Cardinali, M. Picardo (Italy) Nuclear Receptor Activation Affords a Complete Photoprotection Effect
P182 C. L. Goh, S. Gonzalez (USA) New Developments in Photoprotection and Prevention of Photocarcinogenesis
P183 S. X. Lee, X. T. Lauw, W. S. Chong, E. Tian, W. Choo, W. Li, G. Chua, W. Chen, C. Tan, H. Oon (Singapore) Photoprotection: The Importance of a Dedicated Sun Protection Counselling Service
P184 M. Okura, Y. Ishii-Osai, T. Hida, T. Yamashita (Japan) Rapid Diagnosis of Xeroderma Pigmentosum by Using a Set of Recombinant Adenoviruses
P185 R. Halder, I. Rodney, M. Munhutu, P. Foltis, C. Battie, C. Oresajo (USA) Evaluation and Effectiveness of a Photoprotection Composition (Sunscreen) on Subjects of Skin of Color
P186 K. Qasem (Kuwait) Skin Photoaging in Kuwait: A Cross Sectional Study
P187 A. A. Vaidya, B. Sharma, N. Surendra, R. Rajagopal, C. Doraiswamy, V. Gadgil (India) To Assess the Relative Impact of UVA and Uvb Protection on Anti-darkening Efficacy During Day-to-day Sun Exposure

© 2014 John Wiley & Sons A/S. Published by John Wiley & Sons Ltd

Abstracts of Special and Named Lectures

SL1/IFPCS Presidential Lecture

Vitiligo: an update on our viewpoint

M. Picardo

San Gallicano Dermatologic Institute, Rome, Italy

Our research interest in vitiligo started more than 20 yr ago. We worked according to the idea that the immunological deregulation, described in vitiligo, could be the consequence of primary melanocyte damage. We point on the significance and role of the oxidative-driven damage in determining the functional disappearance of the melanocytes associated with the immune surveillance failure.

Starting from the demonstration of loss of the correct redox balance together with increased susceptibility to oxidative insult, we focus on the possible intracellular basis for this functional defect.

Functional in vitro studies suggested that mitochondria could be the source for the uncontrolled free radicals production giving rise to membrane-associated and/or intracellular proteins modifications able in turn to generate or unmask antigenic epitopes. Further evidence for an initial metabolic defect was provided by the study of dermal fibroblasts, characterized by similar functional defects. Considering that the mitochondrial ROS production is the inevitable charge to energy production, usually devoted to intracellular signaling and only when excessive become detrimental, we designated the energy-related metabolism as the possible primitive alteration. The metabolic impairment, characterized by low ATP production and different expression of some key enzymes for aerobic glycolysis, will produce a sufficient energy level in steady-state condition but unable to cover further cellular needs.

Possibly the defective energetic metabolism renders the vitiligo melanocytes prone to senescent process, as testified by the presence of specific markers. An epigenetic study provides further evidences for the central role of the metabolic failure. Moreover, the redox modification of the lipidic membrane structures, affecting in particular the cholesterol, may activate the peripheral dendritic cells triggering and maintaining the immune response. Ex vivo study confirmed the pro-senescent profile in clinically non affected areas further supporting that the metabolic alteration is the initiator of the degenerative process and subsequent immune response.

SL2/PASPCR Aaron Lerner Lecture

Genetic and genomic analyses of pigment cell development and disease

W. Pavan

National Institutes of Health, USA

The use of mouse pigmentation mutants to identify pathways and dissect disease etiology has been a foundation of basic and medical research since the early 1900s, leading to transformative discoveries in quantitative genetics, genome mapping and human disease modeling. Our lab continues this line of study, using genomic tools and genetic manipulation of model systems to unravel genome function and to dissect gene regulatory pathways in development and diseases associated with melanocytes. My talk will include our ongoing efforts to understand the roles of SOX10 during neural crest development, and also postnatally in melanocyte stem cells and in melanoma. One of our approaches uses whole genome in vivo modifier screens to identify pathways that exacerbate the effects of SOX10 function. A second approach defines SOX10's function at the chromatin level by identifying genomic elements and chromatin modifications in the melanocyte lineage, with the goal of correlating pathways governing developmental processes with those that impact melanoma tumor progression and the efficacy of therapeutic interventions.

SL3/Seiji Memorial Lecture

Painting by numbers – genetics of human pigmentation and ancestry

R. A. Sturm[1,2]

[1]Melanogenix Group, Institute for Molecular Bioscience, The University of Queensland; [2]Dermatology Research Centre, School of Medicine, Translational Research Institute, The University of Queensland, Brisbane, Qld, Australia

Environmental changes have had a large impact on selection of pigmentation genes as the human population has expanded into different regions of the world. A combination of approaches have been used in recent years to identify these genes, including comparative genomics of candidate genes and identification of parts of the human genome under positive selection, together with genome-wide and specific allele association studies of skin, hair and eye colour. Genes of major influence include the enzymes encoded by *TYR*, *TYRP1* and *DCT*, the P-protein (*OCA2*) and the melanocortin-1 receptor (*MC1R*). Variant alleles of the *MC1R* gene resulting from a range of amino acid substitutions have been associated with red hair, fair skin, a high degree of freckling as well as increased incidence of melanoma. A non-coding SNP located in a regulatory region upstream of the *OCA2* locus is a major determinant of blue-brown eye colour. Similarly, an intronic SNP in a regulatory region of the *IRF4* gene in Europeans has been implicated in pigmentation phenotypes through altered transcriptional expression of *TYR*. Population genetic studies have revealed specific polymorphisms within the MATP (*SLC45A2*) and NCKX5 (*SLC24A5*) protein coding regions associated with the degree of skin pigmentation, with some alleles going to near fixation in European populations. Our approach to understanding human melanogenesis takes advantage of ongoing parallel genetic and cellular studies. Firstly, by determining the genetic association of variant alleles with pigmentation phenotypes in a collection of adolescent twins and melanoma patients. Secondly, through characterisation of cultures of human primary melanocytes derived from donor skin tissue selected based on pigmentation genotype. Direct testing of a range of clonal melanocyte cultures characterised for causal SNPs within *TYR*, *OCA2*, *MC1R*, *SLC45A2*, *SLC24A5* and *IRF4* have assessed their impact on melanin content and tyrosinase enzyme activity. We propose that additive genotypes of common *TYR* polymorphisms are an important modifier of other pigmentation gene alleles in predictive models of human skin, hair and eye colour.

SL4/Fitzpatrick Lecture

Isozyme shift in cancer metabolism

F. V. Filipp

University of California Merced, Merced, CA, USA

Cancer cells undergo a dramatic rewiring of their metabolism to accommodate energy production as well as generation of

 © 2014 John Wiley & Sons A/S. Published by John Wiley & Sons Ltd

biosynthetic precursors including nucleotides, lipids, organic and amino acids. Using a multi-omics strategy in melanoma research we unraveled the ubiquitous theme of an *isozyme shift in cancer metabolism*. An isozyme shift occurs between redundant enzymes when their function is separated by somatic mutation, mutual exclusive splicing, regulation of gene expression, posttranslational modification, oligomerization, compartmentalization, or co-factor specialization (or any other form of genomic or structural modification regulating enzymatic function). Isozymes of pyruvate kinase, isocitrate dehydrogenase, and serine hydroxymethyltransferase comprise tightly regulated systems and display examples of the most dramatic switches observed in melanoma. By tracing key substrates as well as associated enzymes, today, melanoma research is in a position to re-interpret the Warburg effect at a molecular level. Multi-level control of isozyme activity tracks with cancer progression and provides proliferating cells with a robust, perturbation resistant growth phenotype.

Abstracts of Guest Lectures (GL)

GL2

The photobiology of melanoma in fish models: the importance of UV wavelength, DNA damage and gender on melanoma etiology

D. L. Mitchell

MD Anderson Cancer Center, The University of Texas, USA

We examined the wavelength dependence of ultraviolet radiation (UVR)-induced melanoma in a *Xiphophorus* backcross hybrid model previously reported to be susceptible to melanoma induction by ultraviolet-A (UVA) and visible light. Whereas ultraviolet-B (UVB) irradiation of neonates yielded high frequencies of melanomas in pigmented fish, ultraviolet-A (UVA) irradiation resulted in melanoma frequencies that were not significantly different from non-irradiated control fish. Spontaneous and UV-induced melanoma frequencies correlated with the degree of pigmentation, as expected from previous studies. The results support the conclusion that direct absorption of UVB wavelengths by DNA and the resulting formation of pyrimidine dimers are essential for inducing melanomas in this animal model. We also show a strong male bias for UVB-induced melanoma, consistent with that seen in the human population. To examine underlying factors, we exposed adult X. couchianus fish to a single, sublethal dose of UVB and measured circulating sex steroid hormones and expression of associated hormone receptor genes over a 24-h period. We found that a single exposure had profound effects on circulating levels of steroid hormones with significant decreases for all free sex steroids at 6 and 24 h and increases in conjugated 2-estradiol and 11-ketotestosterone at 6 and 24 h, respectively. Whereas ARα expression increased in male and female skin, neither ARβ nor either of the ERs showed significant responses to UVB in either sex. The rapid response of male androgens and their receptors in the skin after UVB irradiation implicates hormones in the male bias of skin cancer and suggests that the photoendocrine response immediately after UV exposure may be relevant to melanomagenesis.

GL3

Ocean colours exemplified: rapid adaptive skin colouration and patterning in cephalopods

R. T. Hanlon

Marine Biological Laboratory and Brown University, Woods Hole, MA, USA

Nature has evolved elegant solutions for manipulating ambient light within the skin to produce dramatic and colorful animal behavior. Nowhere is the diversity and speed of change in visual appearance better developed than in squid, octopus, and cuttlefish, all of which use rapid adaptive coloration to fight, attract mates, confuse prey and avoid predators. I will present new discoveries and some simplifying principles of how these refined biological systems operate. First, I will illustrate many of these complex visual behaviors with field video. I will present laboratory experimental data showing how cuttlefish visually perceive complex backgrounds and swiftly (1/4 s) produce an appropriate camouflage pattern. I will then describe details of the biophotonic skin structures and their control mechanisms that enable such remarkable visual diversity. Emphasis will be on the neuromuscular chromatophores that produce dynamic pigmentary coloration, the iridophores that produce slower-changing structural coloration via neurochemical control, and the passive leucophores that produce efficient diffuse whiteness. It is the interaction between pigmentary and structural coloration elements in the skin that enable this adaptive coloration system to operate so swiftly and with such optical variety, thus producing rapid phenotypic plasticity.

GL4

Natural selection and the evolution of skin color phenotypes

N. Jablonski

The Pennsylvania State University, Pennsylvania, PA, USA

The human lineage emerged, and *Homo sapiens* originated, under conditions of high UVR near the equator in Africa. At various points in the past, some ancient hominin and *Homo sapiens* lineages dispersed into Eurasia and regions of low and/or highly seasonal UVB. These dispersals were accompanied by biological changes, including the evolution of novel skin pigmentation phenotypes. Our knowledge of these processes in *Homo sapiens* has progressed greatly in the last decade on because of advances both in physiological and genomic studies. A range of in vitro and in vivo studies of skin function have further illuminated the range of responses of human skin to UVR challenges, and now permit clearer understanding of the role of eumelanin in creating a ‘permissive barrier’ regulating photoprotection and vitamin D photosynthesis. Complementing these efforts have been functional genomic studies aimed at studying the strength of selection operating on specific genetic loci regulating pigmentation. The emerging story, not surprisingly, is complex. Rapid biological changes accompanied the dispersal of *Homo sapiens* into Eurasia approximately 60 000 yr ago, driven by the combined influences of natural selection and genetic drift. Parallel evolution in human skin pigmentation phenotypes was common. This has been most thoroughly documented for depigmented phenotypes in Europe, but there is also ample evidence of parallel evolution in tanning phenotypes. In many high latitude populations, depigmentation and consumption of vitamin-D-rich foods have produced biocultural compromises conducive to maintenance of healthy levels of vitamin D under extreme environmental conditions. In

© 2014 John Wiley & Sons A/S. Published by John Wiley & Sons Ltd

the last 500 yr, increased urbanization combined with rapid, long-distance migrations have led to significant mismatches between skin pigmentation and environmental conditions, with predictable negative consequences for health.

GL5

Environmentally-induced skin aging: insights into new molecular mechanisms

J.Krutmann

IUF – Leibniz Research Institute for Environmental Medicine, Düsseldorf, Germany

Previous studies on the pathogenesis of extrinsic or environmentally-induced skin aging have primarily focused on solar ultraviolet (UV) radiation and tobacco smoke as the main environmental stressors. Both factors were shown to be able to induce acute stress responses in human skin which ultimately lead to increased MMP-1 activity, subsequent degradation of collagen fibers in the dermal matrix and thereby to wrinkle formation. Recently we have shown that in addition to solar radiation and tobacco smoke, exposure of human skin to traffic related particulate matter, in particular to soot, causes extrinsic skin aging i.e. wrinkle and, in particular, pigment spot formation. Mechanistic studies show that all three environmental noxae, i.e. UV radiation, tobacco smoke and traffic related PM activate the arylhydrocarbon receptor (AhR) in human skin, and that activation of this signaling pathway leads to collagen degradation and melanocyte proliferation/melanin synthesis. The AhR is thus a novel molecular target for the prevention of extrinsic skin aging and we have shown that topical application of a newly synthesized AhR antagonist is highly effective in preventing extrinsic skin aging.

GL6/DSS Prof Chan Heng Leong Memorial Lecture

Patterning the skin epigenome

H. Y. Chang

Program in Epithelial Biology and HHMI, Stanford University School of Medicine, Stanford, CA, USA

In biology as in real estate, location is a cardinal organizational principle that dictates the accessibility and flow of informational traffic. An essential question in nuclear organization is the nature of the address code—how objects are placed and later searched for and retrieved. Long noncoding RNAs (lncRNAs) have emerged as key components of the address code, allowing protein complexes, genes, and chromosomes to be trafficked to appropriate locations and subject to proper activation and deactivation. I will discuss lncRNA-based mechanisms that control cell fates during development, and show their dysregulation underlies some human disorders caused by chromosomal deletions and translocations. I will describe a new technology based on DNA transposition that reveals the epigenomic profiles of multiple purified cell types from a single clinical biopsy of solid organs. Together these insights are enabling the personal navigation of the gene regulatory landscape in health and disease.

Abstracts of Invited Lectures (IL)

IL1

Paracrine factors as guardians of genomic stability of melanocytes

Z. Abdel-Malek[1], V. Swope[1], R. Starner[1], A. Von Koschembahr[1], A. L. Kadekaro[1], N. Hone[1], D. Supp[1], P. Cassidy[2], S. Leachman[2]

[1]University of Cincinnati, Cincinnati, OH; [2]Oregon Health Sciences university, Portland, OR, USA

In human epidermis, melanocyte homeostasis is maintained to a large extent by a network of paracrine factors, including α-melanocyte stimulating hormone (α-MSH), endothelin-1 (ET-1), and 1,25 $(OH)_2$ vitamin D3 (vit D3), that regulate the survival, proliferation and function of human melanocytes (HM). Many of these factors are increased in expression upon UV exposure and up regulate the expression of their cognate receptors. We have reported on the effects of α-MSH, the melanocortin 1 receptor (MC1R) agonist, on the DNA damage response of HM to UV. We found that treatment of cultured HM with α-MSH enhances the repair of UV-induced DNA photoproducts, reduces oxidative stress, and promotes survival. These effects are mediated by activation of the MC1R, and are absent in HM expressing 2 loss-of-function alleles of the *MC1R* that render the receptor refractory to α-MSH. In UV-irradiated HM, activation of the MC1R results in the phosphorylation of the DNA damage sensors ATR and ATM, leading to increased γH2AX, which facilitates the recruitment of DNA repair proteins to the site of DNA damage. Also, activation of the MC1R activates the stress-induced MAP kinases p38 and JNK, as well as their known targets the transcription factors p53 and ATF-2. Collectively, these events are expected to participate in enhancing repair of UV-induced DNA damage. Endothelin-1 reduces the extent of DNA damage, and also reduces H_2O_2 generation and apoptosis in UV-irradiated HM. ET-1 results in robust phosphorylation of p38, JNK and ATF-2, and enhances their phosphorylation by UV. These effects are mediated by the ETB receptor signaling pathway, mainly Ca^{+2} mobilizations. Activation of both p38 and JNK is critical for the ET-1 effect on DNA photoproducts, and JNK is critical for the survival effect of ET-1. Previously we reported that both α-MSH and ET-1, the synthesis of which is increased in the epidermis upon UV exposure, up regulate the expression of the MC1R, suggesting that these factors increase or sustain the responsiveness of HM to α-MSH. 1,25 $(OH)_2$ vitamin D3 is synthesized in keratinocytes upon UV exposure. Human melanocytes express functional vitamin D receptor, which is increased upon treatment with its ligand vit D3. Treatment of UV-irradiated HM with vit D3 enhances the repair of DNA photoproducts, reduces the generation of H_2O_2, and inhibits apoptosis. These results underscore the role of paracrine factors in counteracting the genotoxic effects of UV on HM, thereby reducing mutagenesis and malignant transformation.

IL2

UV and melanoma: insights from clinical viewpoints

C. Nishigori[1], Y. Funasaka[2]

[1]Division of Dermatology, Graduate school of Medicine, Kobe University; [2]Department of Dermatology, Nippon Medical School

Although the incidence of melanoma in a Japanese population is 2/100 000, 5 to 10 times lower than that in a Caucasian population, it is rising steadily in Asian countries as well as in Western countries. A recent increase in melanoma incidence in Japan is attributed to the increase in the superficial spreading

 © 2014 John Wiley & Sons A/S. Published by John Wiley & Sons Ltd

type melanoma, which account for approximately 30% of the total melanoma incidence. The risk of UV on the development of melanoma is not so solid as non-melanoma skin cancers. Although whole genome sequence analysis provided a good tool for melanoma gene hunting, there are not so many melanoma-related genes, showing UV-signature mutation. Concerning causative wavelength in melanomagenesis, recent studies indicate UVB but not UVA induce melanoma in both mice and fish. On the other hand epidemiological study shows the possible involvement of UVA in melanoma development and WHO highlighted the sunbed use poses a risk of skin cancer, especially melanoma. In this session we will briefly present our work: the effect of UV on melanoma-prone conditional mice, which express the ectopic expression of metabotropic glutamate receptor-subtype 1 in melanocytes. UV irradiated mice developed melanoma earlier and accelerated the rate of melanoma development than un-irradiated mice. We also found yellow mice are more susceptible to melanomagenesis. Our in vivo studies will be discussed in relation to the following points: (1) UV induced DNA damage as initiation, 2) UV damage and melanocyte repair (3) UV induced inflammation as promotion, (4) the difference in color-eumelanin/pheomelanin as a modifying factor and (5) difference in conformation of gene due to the different types of DNA damage.

IL3

Regulation of the development of mouse melanocytes by coat color genes

T. Hirobe

Fukushima Project Headquarters, National Institute of Radiological Sciences, Chiba, Japan

Melanocytes, pigment-producing cells, are originally derived from neural crest cells in embryonic skin. Neural crest cells migrate dorsoventrally and localize all over the body. Melanoblasts, precursor of melanocytes, differentiate from the neural crest cells, proliferate, and colonize the epidermis. Melanocyte differentiation initiates around the time of birth and migrate to hair follicles to form pigmented hair. These processes of melanocyte development are regulated by numerous epigenetic and genetic factors. The epigenetic factors from tissue environment, especially keratinocytes, are important. Keratinocytes produces many paracrine factors including hormones, growth factors, and cytokines to stimulate melanocyte development through the interaction with their receptors. Among the genetic factors, the coat color mutant genes are very important. In mice, more than 300 genes control melanocyte development; about half of them have been cloned and their functions are clarified. Coat colors are determined by eumelanin and pheomelanin which are synthesized in melanocytes and accumulate in specialized organelles, melanosomes, which upon maturation are transferred to keratinocytes. Melanoblast migration, proliferation, and differentiation are also regulated by many coat color genes otherwise known for their ability to regulate melanosome formation and maturation, pigment switching (from eumelanin to pheomelanin and vice versa), melanosome distribution and transfer. Melanocyte differentiation is also regulated by a-melanocyte stimulating hormone (MSH) and its receptor, Mc1r. The mutation in the *Mc1r* gene is regulated by one of the classical coat color genes, extension (*e/Mc1r^{e}*). Alpha-MSH in the mouse, however, is likely carried through the blood stream and not produced locally in keratinocytes. Thus, melanocyte development is not only regulated by genes encoding typical growth factors and their receptors but also by genes classically known for their role in pigment formation.

IL4

Common mechanisms regulating neural crest development and melanoma formation

L. Sommer

Institute of Anatomy, University of Zurich, Zurich, Switzerland

Melanoma arises from the melanocyte lineage, which originates during embryonic development from the neural crest. Intriguingly, many factors known to regulate neural crest and melanocyte development also appear to be active and functionally important during melanoma formation. Interfering with mechanisms normally regulating ‘stemness’ in neural crest cells influences tumorigenesis both in genetic melanoma mouse models in vivo and in human melanoma cells. For instance, the transcription factor SOX10 regulates both maintenance of neural crest stem cells as well as melanoma formation and expansion. In contrast, the related factor SOX9 –which regulates cell cycle exit and differentiation in neural crest cells– counteracts SOX10 activity and prevents tumor initiation. Likewise, signaling pathways such as TGFb family factors that normally regulate SOX10 vs. SOX9 expression in neural crest development also control melanoma progression. Finally, epigenetic regulators that we have shown to control neural crest cell fates during embryonic development are functionally involved in melanoma formation and metastasis in vivo. Thus, we might learn from developmental biology to identify key players in melanoma formation.

IL5

The optical and electrical properties of melanins: archetypal bioelectronic materials?

P. Meredith

Centre for Organic Photonics and Electronics, School of Mathematics and Physics, University of Queensland, Brisbane, Qld, Australia

The melanins possess an intriguing set of physical and chemical properties including electrical and photo-conductance, broad band optical absorption and almost unity non-radiative conversion of absorbed light [1]. We now understand these properties within the general framework of the chemical disorder and hybrid ion-electron conduction models [2].

Based upon this new structure-property understanding there have been recent suggestions that melanin-like materials could be used as a functional interface between conventional electronic read and control systems and biological entities [2]. This is one of the central themes of the emerging field of bioelectronics – in particular, functional elements are required which are biocompatible and can transduce at high fidelity between ionic and electronic signals [3]. In my talk I will review the pertinent optical and electrical properties of melanins. I will discuss how these properties can be harnessed in bioelectronics and demonstrate prototype electrical devices capable of directly transducing between ion and electronic signals.

© 2014 John Wiley & Sons A/S. Published by John Wiley & Sons Ltd

IL6

Elaboration of approach to structure of neuromelanin present in various brain regions as studied by chemical degradative methods

K. Wakamatsu

Department of Chemistry, Fujita Health University School of Health Sciences, Toyoake, Japan

There are two chemically distinct types of peripheral melanins, black to brown pigments called eumelanin, and yellow to reddish-brown pigments called pheomelanin. Eumelanin is derived from the oxidative polymerization of 5,6-dihydroxyindole (DHI) and 5,6-dihydroxyindole-2-carboxylic acid (DHICA), while pheomelanin consists of benzothiazine and benzothiazole units arising from cysteinyl-3,4-dihydroxyphenylalanine (Cys-DOPA) isomers. On the other hand, neuromelanin (NM) consists of black to brown pigments mainly found in neurons of the substantia nigra (SN) and locus coeruleus (LC) in the central nervous system of human and other mammalian species. Previous studies from our group and other groups have shown that NM consists of complex polymers derived from eumelanic DHI and pheomelanic benzothiazine units along with additional aliphatic and proteic components. NM accumulates during normal aging in neurons of different brain areas but, interestingly, SN and LC that the main target regions of Parkinson's disease (PD) are those with the highest pigment concentrations. NM is formed by the oxidation of catecholamines, dopamine (DA) and norepinephrine (NE), in the presence of cysteine (Cys). The synthesis of NMs in the various regions of the human brain is an important protective process because the melanic components are generated through the removal of reactive or toxic *o*-quinones that would otherwise cause neurotoxicity. However, there are still unknown aspects in the chemical structure of NM from SN (SN-NM) and NM of LC (LC-NM). In order to elucidate the structure of SN-NM and LC-NM, the chemical degradative methods are thought to be more powerful tools due to the rigid covalent bond in NM. We recently designed a new method to synthesize *o*-aminophenol compounds as putative degradation products of catecholamines and their metabolites which may be incorporated into SN-NM and LC-NM. These *o*-aminophenol compounds were synthesized by the nitration of phenol derivatives followed by reduction with hydroiodic acid (HI), and they could be identified by HPLC in HI hydrolysates of SN-NM and LC-NM. This degradative approach by HI hydrolysis allows the identification of catecholic precursors unique to SN-NM and LC-NM, which are present in catecholaminergic neurons. As I mentioned above, it is known that NM accumulates during normal aging in neurons of different brain areas. Thus, it is very important to elucidate in detailed the aging process of NM in connection of the etiology of PD. I also would like to report the structural conversion in NM obtained from subjects with various ages.

IL7

Experimental basis for HSP70iQ435A therapy to treat vitiligo

J. Mosenson[1], J. Eby[2], J. Mahon[2], R. Boissy[3], M.Nishimura[2], A.Salzman[4], A. Zloza[2], P. Srivastava[5], J. Guevara-Patino[2], C. Le Poole[2]

[1]Loyola University Chicago; [2]Oncology Research Institute, Loyola University Chicago, Maywood, IL; [3]Dermatology, University of Cincinnati, Cincinnati, OH; [4]Radikal Therapeutics, Beverly, MA; [5]Immunology and Medicine, University of Connecticut, Farmington, CT, USA

Inducible heat shock protein 70 (HSP70i) chaperones proteins and peptides to protect cells from stress-induced apoptosis. Upregulated in affected vitiligo skin, HSP70i will activate dendritic cells when released by stressed or dying cells by enhancing uptake, processing and presentation of chaperoned antigens. We reasoned that stress will induce the release of HSP70i-melanosomal peptide complexes to elicit melanocyte-reactive T cell infiltration to the skin, and that blocking this process can interfere with vitiligo development. To address this, we measured release of the protein from control and vitiligo melanocytes. We assessed the contribution of HSP70i to depigmentation and to T cell responses in mouse gene gun vaccination studies. Site-directed mutagenesis served to pinpoint residues of importance to HSP70i immune function, and we studied resulting dendritic cell activation in vitro and *ex vivo*, including transcriptome analysis to further our understanding of resulting DC physiology. DNA encoding mutant $HSP70i_{Q435A}$ was used to measure its effect on depigmentation in vitiligo-prone mice. The insert was subcloned into a vector acceptable for clinical application and studies were initiated to compare the impact of mutant and wildtype *HSP70i* in this setting. Interestingly, we found that stress imposed by MBEH exposure drives HSP70i to melanosomes, and the heat shock protein is more abundantly secreted by vitiligo melanocytes in response to MBEH. HSP70iencoding DNA alone is enough to induce depigmentation in vitiligo prone mice, and mice lacking HSP70i do not depigment. Transcriptome analysis revealed CD1A, TNF, IFN-gamma, IL17A, and GLYCAM were upregulated by HSP70i, and CD14, IDO1, CCL1, ICAM2 and TSLP were among many transcripts upregulated by $HSP70i_{Q435A}$ protein exposed DCs. DNA encoding $HSP70i_{Q435A}$ markedly inhibited depigmentation in TCR transgenic mouse models of vitiligo. Initial data support that $HSP70i_{Q435A}$in pUMVC3, a.k.a. 'black magic' has equal or greater activity than the gene expressed from a TOPO vector. Together with data presented separately to show its effects on anti-tumor responses, 'black magic' shows promise for the treatment of vitiligo.

IL8

Increased circulating Th17 cells and decreased circulating iNKT cells in human non-segmental vitiligo

L. Zhou[1,2,3], Y. L. Shi[1,2,4], K. Li[1,2,5], I. Hamzavi[1,2], T. W. Gao[5], R. H. Huggins[2], H. W. Lim[1,2], Q. S. Mi[1,2,3]

[1]Henry Ford Immunology Program; [2]Department of Dermatology, Vitiligo Treatment and Research Center; [3]Department of Internal Medicine, Henry Ford Health System, Detroit, MI, USA; [4]Department of Dermatology, Shanghai Tenth People's Hospital, Tongji University School of Medicine, Shanghai; [5]Department of Dermatology, Xijing Hospital, Fourth Military Medical University, Xian, People's Republic of China

Although it is widely believed that non-segmental vitiligo (NSV) results from the autoimmune destruction of melanocytes, a clear understanding of defects in immune tolerance that mediate this uncontrolled self-reactivity is still lacking. We systemically evaluated circulating regulatory T (Treg) cells, including $CD4^{+}CD25^{+}FoxP3^{+}$ Treg cells and invariant natural killer T (*i*NKT) cells, naïve and memory $CD4^{+}$ and $CD8^{+}$ T cells, as well as Th1, Th2, Th17 cells, in a cohort of 43 progressive NSV patients with race-, gender- and age-matched healthy controls. We found that the general immunophenotypes of $CD4^{+}$ and $CD8^{+}$ T cells, the frequencies of $CD4^{+}CD25^{+}FoxP3^{+}$ Tregs, IFN-$\gamma^{+}CD4^{+}$, and $IL4^{+}CD4^{+}$ cells were comparable between NSV and healthy controls (HC). However, the frequency of peripheral *i*NKT

 © 2014 John Wiley & Sons A/S. Published by John Wiley & Sons Ltd

cells was significantly decreased, while the frequency of peripheral Th17 cells and related cytokines in serum, including IL-17A, TGF-□1 and IL-21 levels, were significantly increased in NSV patients compared to that in HC. Furthermore, the increased Th17 cell frequencies are positively correlated with serum TGF-□1 level, and the body surface area of lesions are positively correlated with elevated serum TGF-□1 and IL-21 levels and Th17 cell frequencies. These results suggest that the elevated serum TGF-□1 and IL-21 could contribute to enhanced Th17 cell differentiation in NSV, and dysregulated Th17 cells and iNKT cells may be involved in the pathogenesis of NSV.

IL9

The susceptibility genes of vitiligo

X. J. Zhang

Anhui Medical University, People's Republic of China

Vitiligo is an acquired disease characterized principally by patchy depigmentation of skin and overlying hair, with diverse prevalence rates in different geographic regions and ethnic groups. Clinical and epidemiological investigations indicated that vitiligo might follow a pattern of polygenetic or multifactorial inheritance. Genetic analyses of vitiligo for several decades, the goal was to understand the biological mechanisms and elucidating pathways that underlie the disease. A number of genetic susceptibility factors have been identified only a few genes/loci through linkage and association studies. Early candidate gene association studies yielded little successes, but most such reports now appear to be false-positives. Subsequent genome wide linkage studies of multiplex vitiligo families identified linkage signals on chromosomes 7, 8, 9, 11, 13, 17, 19, and 22. SNPs spanning the chromosome 17p, 22q linkage interval identified the corresponding gene as *NALP1* and *XBP*, apparent true vitiligo susceptibility genes involved in immune regulation.

Notably, recent several genome-wide association studies (GWASs) of vitiligo have identified a number of susceptibility genes/loci, such as 1p13.2 (*PTPN22*), 1p36.23 (*RERE*), 2q24.2 (*IFIH1*), 3p13 (*FOXP1*), 3q13.33 (*CD80*), 3q28 (*LPP*), 6p21.3 (*HLA-A*, *HLA-B*, *HLA-C*), 6q27 (*CCR6*), 10q22.3 (*ZMIZ1*), 10p15.1 (*IL2RA*), 11p13 (*CD44*), 11q14.3 (*TYR*), 12q24.12 (*SH2B3*), 12q13.2 (*IKZF4*), 14q12 (*GZMB*), 16q24.3 (*MC1R*), 21q22.3 (*UBASH3A*), 22q13.1 (*C1QTNF6*) and 22q13.2 (*TOB2*) in Caucasian and Chinese populations. Most of these loci encode immune-related proteins, and several ones have been associated with other autoimmune diseases, highlighting common immune pathogenetic pathways underlying these different disorders. In addition, some variants in *MHC* region are associated with clinical features of vitiligo (age of onset and clinical types) by genotype-phenotype analysis, which might provide an important theory for disease diagnosis. Together, these genes and pathways provide insights into underlying pathogenetic mechanisms and possible triggers of vitiligo, and provide insights into both disease pathogenesis and potential new targets for both treatment and even prevention of vitiligo and other autoimmune diseases in genetically susceptible individuals. These researches will better illustrate the comprehensive genetic basis of vitiligo in the future.

IL10

So many roads to melanin – how important are bone morphogenetic proteins in regulating human cutaneous pigmentation?

D. J. Tobin

Centre for Skin Sciences, School of Life Sciences, University of Bradford, Bradford, UK

Skin and hair pigmentation are two of the most apparent survival-associated phenotypes in mammals, especially in those non-nocturnal mammals with physiologies that have evolved in tandem with the demands of a UVR-drenched terrestrial planet. It is not surprising therefore that pathways for both the production/distribution of cutaneous melanin have developed into a veritable anastomotic contingency system, presumably because nature has demanded backups (and indeed backup for the backups) for this essential phenotypic survival trait in the wild. There has been much research and discussion on the hierarchy of particular signaling pathways regulating both constitutive and facultative human pigmentation. While there has long been a MC1R-centric view, the significance of other (often interactive) signaling pathways has become appreciated, including c-KIT/SCF, EDN1/EDNRB and others. Here I will focus on the role of the multi-functional secreted bone morphogenetic proteins (BMPs) family in pigmentation.

While it is known that BMP2/4 downregulates tyrosinase expression/activity to reduce pigmentation in epidermal melanocytes (MC), we recently examined the role of BMP6 in both melanogenesis and melanin transfer. BMP4, BMP6, and their natural antagonists Noggin & Sclerostin were variably expressed in human skin and hair follicles (incl. MC). While BMP4 downregulated melanogenesis as expected, by contrast BMP6 markedly stimulated MC melanogenesis. BMP6 also induced MC filopodia formation and Myosin-X expression, which are used in melanosome transfer from MC to keratinocytes (KC). These results were confirmed by siRNA knockdown of BMP receptor (BMPRII) or Myosin X, and by exposure of MC to Noggin and Sclerostin. These data indicate complex regulation of MC by different members of the BMP family suggesting that further differential control of skin pigmentation may be exerted by presence and relative concentration of different BMPs and their antagonists.

IL11

Pathway-specific regulation of human melanocortin-1 receptor signaling

J. C. Garcia-Borron

Department of Biochemistry and Molecular Biology, School of Medicine, University of Murcia, Murcia, Spain

The functional effects of melanocortin 1 receptor (MC1R) signaling are pleitropic, extending beyond pigmentation to involve activation of DNA damage repair and antioxidant defenses. Binding of melanocortin agonists to MC1R triggers the sequential activation of the Gs protein and adenylate cyclase to increase cAMP formation. Accordingly, MC1R is primarily considered a G protein-coupled receptor positively coupled to the cAMP pathway. However, the signaling module leading to the extracellular signal-regulated kinases (ERK) ERK1 and ERK2 is also activated downstream of MC1R. In human melanocytic cells, cAMP is neither sufficient nor necessary to achieve functional coupling of MC1R to the ERKs. On the other hand, pharmacological interference, siRNA studies, expression profiles and functional reconstitution experiments show that

© 2014 John Wiley & Sons A/S. Published by John Wiley & Sons Ltd

melanocortin peptides achieve ERK activation by transactivation of cKIT or another related receptor tyrosine kinase. Moreover ERKs are efficiently activated downstream of MC1R mutants with hypomorphic cAMP signaling. In particular, the major red hair color-associated (RHC) variants R151C, R160W and D294H are comparable to the wild type MC1R in activating ERK in spite of major impairment in triggering the cAMP pathway. Thus, RHC variants are best described as imbalanced signaling mutants rather than loss-of-function forms. In addition, functional coupling to the ERK and cAMP pathways is differentially regulated by the availability of activatory ligands and by the cytosolic β-arrestins, which downregulate signaling to cAMP with minor effects on ERK activation. Accordingly, activation of the cAMP and ERK signaling cascades are independent events that can be dissociated by specific mutations in MC1R and most likely involve different intracellular effectors. Differential responses to genetic variation, melanocortin availability and regulatory proteins might afford flexibility to fine-tune the balance of MC1R signaling to the cAMP and ERK pathways.

Supported by grants SAF2012-32134 (to JCGB) from the MINECO, Spain, FEDER funds (European Community).

IL12

The biogenesis of melanosomes and intercellular communication in the skin

G. Raposo

CNRS-UMR144, Institut Curie Paris, Paris, France

Melanosomes, the lysosome-related organelles (LROs) of pigment cells synthesize and store melanins fated for transfer to keratinocytes. Our studies focus on the biogenesis of melanosomes, a process that requires trafficking of melanosomal proteins within distinct endosomal subdomains. During early melanogenesis, sorting of the protein PMEL to intraluminal vesicles of multivesicular bodies (MVBs) precursors of melanosomes is concomitant with its cleavage and formation of PMEL-driven amyloid fibrils. Sorting of PMEL and fibril formation requires its interaction with the Tetraspanin CD63 (van Niel et al., Dev Cell, 2011) and we have recently shown that the protease BACE-2 processes PMEL to form the physiological amyloid (Rochin et al., PNAS, 2013). Late melanogenesis involves the transfer of melanogenic enzymes from early recycling endosomes to melanosomes. Proteins bearing mutations in the Hermansky Pudlak syndrome regulate endosomal trafficking and late stages of melanogenesis. We have also shown that the kinesin motor KIF13A interacts with the adaptor AP-1 to coordinate sorting and positioning of peripheral endosomal domains close to melanosomes (Delevoye et al., JCB, 2009). Our studies reveal that KIF13A is essential for endosome tubulogenesis and we have identified Rab11 as a KIF13A effector that recruits the motor to Transferrin-positive recycling endosomes (Delevoye et al., Cell Rep., 2014). The interplay between KIF13A and endocytic Rabs appears as an essential regulatory mechanism controlling functional plasticity of the endosomal recycling system for the biogenesis of melanosomes and maybe other LROS (Marks et al., Curr Op? Cell Biol., 2013). Our current studies focus on how endosome dynamics, melanosome biogenesis and consequently melanosome transfer to keratinocytes is regulated by direct interactions with keratinocytes or whether secreted vesicles called exosomes modulate the pigmentation status of the melanocytes.

IL13

A role of multiple melanosome-containing globules in melanosome transfer and a possible contribution of fibroblasts to dermal melanin deposition

H. Ando[1], Y. Higashi[1], K. Akiyama[2], M. Yagi[3], M. Ichihashi[4]

[1]Department of Applied Chemistry and Biotechnology, Okayama University of Science, Okayama; [2]Hanaichi Ultrastructure Research Institute Co., Okazaki; [3]Rosette Co., Tokyo; [4]SAISEI MIRAI Clinic Kobe, Kobe, Japan

Melanocyte dendrites penetrated through 1 μm-pore membrane filter have a potency to generate multiple melanosome-containing globules (MMCG) at various sites along the surface of the dendrites. The average diameter of the globules is larger than the pore size of the filter, indicating that the globules are constructed after elongation of the dendrites, presumably via pasting up melanosomes through filopodia. The MMCG released into the extracellular space from the dendrites are captured by the microvilli of normal human keratinocytes, and the melanosomes are distributed primarily in the perinuclear area of the keratinocytes. In the dermis, macrophages that ingest melanosomes are known to be involved in dermal melanin deposition. Our recent studies revealed that both macrophages and fibroblasts uptake the MMCG, especially fibroblasts prefers to incorporate MMCG rather than individual melanosomes. Further, the comparative study of degradation pattern of the MMCG incorporated into the cytoplasm of macrophages and fibroblasts showed that the globules incorporated in macrophages were severely degraded, while the globules incorporated in fibroblasts were not altered appreciably, leading to the characteristic in vivo behavior of dermal melanin in melanophages residing for long years. These results suggest that MMCG play a role in the melanosome transfer from melanocytes to keratinocytes in the epidermis for constructing melanin cap to prevent cellular nuclei against solar ultraviolet radiation, and fibroblasts in addition to macrophages in the dermis cooperate in melanosome processing to clean up toxic melanin in the dermal matrix by their phagocytotic activity.

IL15

UVB phototherapy in vitiligo treatment: immune regulation versus biostimulation

C. C. E. Lan

Department of Dermatology, Kaohsiung Municipal Ta-Tung Hospital, Kaohsiung Medical University, Kaohsiung, Taiwan ROC

Phototherapy is an essential part of dermatology practice for treating various skin disorders. Lights of different spectrum, ranging from ultraviolet (UV), visible, to infrared regions, all have been used for managing various skin conditions. The proposed mechanisms responsible for phototherapy, particularly those in the UV region, mostly focused on the immune suppressive effects of lights. Recently, the clinical applications of UV phototherapy have become more diversified. The immune suppressive effects of UV radiation have lead to advancements in photoimmunology including elucidating the immune regulatory roles for Langerhans cells, dermal dendritic cells, and mast cells. The biostimulatory effects of UV radiation can be exemplified by vitiligo repigmentation. Vitiligo is a depigmentary disorder resulting from disappearance of functional melanocytes. Vitiligo repigmentation depends on the activation, migration, and functional development of primitive melanoblast cells. The process of vitiligo repigmentation presents a perfect model for studying the regenerative effect of UV therapy. We have recently

 © 2014 John Wiley & Sons A/S. Published by John Wiley & Sons Ltd

demonstrated that the capacity of UVB irradiation to induce functional development of melanoblast depends on the irradiance (W/cm^2) of the radiation source. The high irradiance UVB induces stronger activation of aryl hydrocarbon receptor pathway, stimulates more prominent epidermal growth factor receptor nuclear translocation, and initiates tyrosinase transcription and translation while equivalent fluence (J/cm^2) delivered by low irradiance UVB failed to do so. Therefore, irradiance is an important parameter to consider when using UV phototherapy for regenerative purposes.

Dermatologists routinely use phototherapy for treatment of different conditions. However, our understanding regarding the effects of light, even the most commonly encountered UV radiation, remained far from complete. Delineation of the photobiological effects of different lights, including immune regulation and biostimulation, will not only enhance the therapeutic efficacy of phototherapy, but will also promote better health since human beings will continue to have intimate interactions with different spectrums of light.

IL16

Management of refractory vitiligo

T. Passeron

University of Nice Sophia Antipolis, Nice Cedex 02, France

Vitiligo is a challenging disorder to treat. To date no treatment provides truly satisfactory results. However, from molecules that target the immune system, to therapies that stimulate the proliferation and differentiation of melanocytes and/or melanocyte progenitors and others that fight against radical species or bring new melanocytes to the affected areas, we have now many strategies to repigment vitiligo patches. These treatments allow complete or near complete repigmentation in some patients, especially on some sensitive areas such as the face. At the opposite, hands and feet, and to a lesser extent, bony prominences, are extremely difficult to repigment. However, several types of combination approaches have provided significant improvement in the management of these refractory areas. When a repigmentation is finally achieved, the risk of redepigmentation is high and is frustrating for the patient and the clinician. Recent data supporting for the first time, treatment decreasing the relapses occurring after repigmentation will be discussed. Finally, in widespread vitiligo that failed to respond to all those approaches, a depigmentation with topical agents or lasers can afford a significant esthetical improvement of the lesions by removing the remaining pigmented areas.

IL18

The genetics of vitiligo and associated autoimmune diseases

R. A. Spritz

Human Medical Genetics and Genomics Program, University of Colorado School of Medicine, Aurora, CO, USA

Vitiligo is a common, complex disease in which patchy depigmentation of skin and overlying hair results from autoimmune loss of melanocytes. The causation of vitiligo is polygenic and multifactorial, involving many genes and as-yet unknown environmental triggers. We have previously carried out both genomewide genetic linkage and genomewide association studies in several different world populations, identifying at least 32 different confirmed vitiligo susceptibility loci in European-derived Caucasians. Recently, we carried out a third genomewide association study in this population, thereby detecting a number of additional vitiligo susceptibility loci. For a number of these loci the corresponding gene, causal gene variants, and mechanism of disease pathogenesis have been identified.

Most known vitiligo susceptibility genes encode immunoregulatory proteins, many also implicated in other autoimmune diseases with which vitiligo is epidemiologically associated, supporting the long-held belief that these disorders involve shared causal factors. Moreover, some vitiligo susceptibility genes (and even underlying causal variants) are shared among different world populations, while others are relatively population-specific, implying that the pathogenic mechanisms of vitiligo have both similarities and differences between different populations.

Particularly among Caucasians, some vitiligo susceptibility loci encode proteins with key functions in the melanocyte, which likely mediate immune cell programming, immune triggering, and autoimmune targeting of melanocytes. Surprisingly, these same genes have been implicated in susceptibility to melanoma, with opposite genetic specificity, suggesting these proteins may modulate immune surveillance to melanoma, and that vitiligo might result from dysregulation of melanocyte-specific immune surveillance.

The discovery of vitiligo susceptibility genes and causal variants highlights biological pathways that present targets for novel therapies, both for vitiligo and for other autoimmune diseases, validating the hopes of early investigators who first attempted to comprehend the genetic basis of vitiligo.

IL19

Losing color - keratinocytes drive autoimmunity in vitiligo through CXCL10

J. M. Richmond[1], K. Essien[1], J. R. Groom[2], A. D. Luster[3], J. E. Harris[1]

[1]University of Massachusetts Medical School, Worcester, MA, USA; [2]Walter and Eliza Hall Institute of Medical Research & University of Melbourne, Melbourne, Vic., Australia; [3]Massachusetts General Hospital, Harvard Medical School, Boston, MA, USA

Vitiligo is a disfiguring autoimmune disease of the skin caused by T cell-mediated destruction of melanocytes. We recently discovered that the IFN-γ-CXCL10-CXCR3 chemokine axis is active in both human vitiligo and in a vitiligo mouse model that we developed, and that CXCL10 is critical for both the progression and maintenance of vitiligo, implicating this pathway as a potential treatment target. In order to focus our treatment strategy, we sought to identify which cell types produce CXCL10 during vitiligo. We found that there are multiple sources, including Langerhans cells, dermal dendritic cells, $\gamma\delta$ T cells, endothelial cells, fibroblasts, and keratinocytes. To determine which cellular source was functionally required to drive depigmentation in vitiligo, we used genetically modified strains that were deficient in each cell type, or lacked the ability to respond to IFN-γ signaling (STAT1-deficient). While mice that lacked Langerin$^+$ dermal dendritic cells and $\gamma\delta$ T cells developed normal depigmentation, Langerhans cell-deficient mice developed worse disease. Vitiligo in mice with STAT1-deficient keratinocytes was largely abrogated, revealing that keratinocytes promote vitiligo, while Langerhans cells suppress disease. Therefore, suppressing IFN-γ signaling in KCs through topical treatments may be an effective targeted treatment strategy for vitiligo.

© 2014 John Wiley & Sons A/S. Published by John Wiley & Sons Ltd

IL20

Functional properties of subsets of melanocyte stem cells

T. Hornyak
University of Maryland, Baltimore, MD, USA

Mammalian coat pigmentation is maintained by melanocyte stem cells (MSCs), which generate differentiated melanocytes during the hair follicle (HF) cycle. Pigment produced by differentiated melanocytes in the base of the growing (anagen) HF is transferred to follicular keratinocytes to form the elongating, pigmented hair shaft. Prior studies described a quiescent population of MSCs in the lower region of the HF where quiescent follicular epithelial stem cells also reside.

Using transgenic mice that express GFP inducibly in melanocytic cells, we find that two distinct sets of melanocyte progenitor cells, one located in the bulge/lower permanent portion (LPP) of the HF and the other restricted to the secondary hair germ (SHG) region, can be defined and separated based upon expression of the membrane protein CD34. Analysis of FACS-separated cells using quantitative RT-PCR demonstrates that both CD34+GFP+ bulge/LPP and CD34-GFP+ SHG cells express high levels of Dct and low levels of Krt14. We also find that melanocyte differentiation genes are expressed more highly in CD34-GFP+ SHG cells, suggesting that these cells exist at a more advanced state of melanocyte differentiation.

To explore the distinction between these cells further, we tested their ability to grow as non-adherent clusters, or spheroids, under cell culture conditions favoring growth of either primary neural crest cells or differentiated melanocytes. CD34+GFP+ bulge/LPP spheroids grow preferentially in neural crest medium, whereas CD34-GFP+ SHG cell growth is best supported by melanocyte medium. When analyzed after in vitro expansion CD34-GFP+ derived cells consist principally of Tyrp1+ differentiated melanocytes, whereas CD34+GFP+ derived cells variably express cell markers associated with other neural crest-derived cell types.

Continued use of CD34 to separate bulge/LPP and SHG HF melanocyte progenitors should facilitate understanding their interrelationship and assist in dissecting their functional properties.

IL21

Developmental control of dermal melanocytes progenitors

T. Kunisada, H. Aoki
Tissue and Organ Development, Gifu University, Gifu, Japan

Unlike the thoroughly investigated melanocyte population in the hair follicle of the epidermis, the growth and differentiation requirements of the melanocytes in the eye, harderian gland and inner ear – the so-called non-cutaneous melanocytes – remain unclear. We investigated the in vitro and in vivo effects of the factors that regulate melanocyte development on the stem cells or the precursors of these non-cutaneous melanocytes. A reduction in Kit receptor tyrosine kinase signalling is known to lead disordered melanocyte development. However, melanocytes in the eye, ear and harderian gland were revealed to be less sensitive to Kit signalling than cutaneous melanocytes. Instead, melanocytes in the eye and harderian gland were stimulated more effectively by endothelin 3 (ET3) or hepatocyte growth factor (HGF) signals than by Kit signalling, and the precursors of these melanocytes expressed the lowest amount of Kit. Dermal melanocytes are hard to adequately separate from those in epidermis, however, in transgenic mice induced to express ET3 or HGF in their skin, survival and differentiation of dermal melanocytes but not epidermal melanocytes were enhanced, suggesting that dermal melanocytes share their character with non-cutaneous melanocytes. These results provide a molecular basis for the clear discrimination between non-cutaneous or dermal melanocytes and epidermal melanocytes, a difference that might be important in the pathogenesis of melanocyte-related diseases and melanomas.

IL22

Rewired signalling in melanoma – a journey along the PDK1 axis

Z. Ronai
Sanford-Burnham Medical Research Institute, La Jolla, CA, USA

Key players in melanoma signalling are mutated oncogenes along the MAPK pathway, with BRAF and NRAS that are most prevalent, mutated in over 60% of these tumors. The amplification of the MAPK signalling pathway constitutes the basis for re-wiring of signal transduction pathways, which drive melanoma development and progression. Among the affected kinases and downstream pathway components are additional protein kinases, including PDK1 (a master regulator of AGC kinases) and transcription factors, including the AP1 (C-Jun/ATF2) complex. We demonstrate the importance of PDK1 for melanoma development and progression, using genetic melanoma models in which PDK1 has been inactivated. We further demonstrate the possible consideration of pharmacological inhibitors that target components along the PDK1 pathway, as new therapeutic modalities for melanoma.

IL23

Metabolism – metabolic vulnerabilities of melanoma

E. Gottlieb[1], J. Kaplom[2], B. Chaneton[1], L. Zheng[1], D. Peeper[2]
[1]Cancer Research UK, Beatson Institute, Glasgow, Scotland, UK; [2]The Netherlands Cancer Institute, Amsterdam, The Netherlands

Despite the vast accumulation of knowledge on the molecular bases of cancer, it remains the second-leading cause of death in the developed world. Hence, more effective approaches for cancer treatment are needed. The search for new, disease-related vulnerabilities of tumours has stimulated a resurgence of interest in understanding metabolic reprogramming in cancer cells, one of the earliest biological hallmarks noted in malignant tissue. Interest in cancer metabolism has been renewed recently since it was appreciated that metabolic alterations in cancer goes beyond bioenergetics demands and include many pathways, in principle greatly expanding the number of clinically relevant metabolic targets. Furthermore, most, if not all, tumour suppressors and oncogenes regulate metabolism, such that tumour-promoting mutations in these genes elicit alterations of metabolic activities and dependencies.

Several tumour-suppressing mechanisms have evolved to avert the hazard of malignant transformation sparked by oncogenic events. One program is the cessation of cell proliferation, termed Oncogene-Induced Senescence (OIS). In recent years, a large body of evidence has shown that OIS acts as a pathophysiologic mechanism suppressing cancer in model organisms and humans alike. Indeed, senescence biomarkers have been reported for a plethora of pre-cancerous lesions including melanocytic nevi. The senescence response typically manifests after an initial phase of cell proliferation triggered by an oncogenic incident, thereby halting further expansion. Progression towards malignancy can

 © 2014 John Wiley & Sons A/S. Published by John Wiley & Sons Ltd

occur only in the context of additional tumorigenic alterations, which overcome senescence.
Numerous studies have shown that changes in metabolism are linked to the proliferative capacity of cells. To support a high rate of proliferation, cancer cells commonly shift their metabolism towards biosynthesis, thereby providing the building blocks necessary for tumour expansion. Although it is generally assumed that senescent cells remain metabolically active, surprisingly few aspects of this process have been investigated in detail. Therefore, we have performed a comprehensive study on the role and regulation of metabolism in OIS. We used metabolic flux profiling to screen for metabolic changes in central carbon and energy metabolism in OIS evoked by mutant BRAF, a common driver of senescence in benign lesions. We demonstrated that pyruvate oxidation in the mitochondria via pyruvate dehydrogenase (PDH) is necessary and sufficient for OIS. We also studied the functional biological consequences, for OIS and tumorigenesis, of perturbing the balance between glycolysis and oxidative phosphorylation and further identified pyruvate dehydrogenase kinase 1 (PDK1), the inhibitor of PDH, as a vulnerable target in BRAF mutant melanoma cells. The inhibition of PDK1 (and the consequent activation of PDH) induced senescence in established BRAF-driven melanomas and had a profound effect on tumour growth in vivo.

IL24

Pigmentation, oxidative stress and specific epidermal detoxification

L. Marrot
L'OREAL R&I, Aulnay-sous-Bois, France

Epidermal cells are exposed to various environmental insults likely to impact skin homeostasis: sunlight, pollution, exogenous chemicals... Pigmentation is one of the cutaneous responses aiming at increasing protection. However, melanin neosynthesis can sometimes be deleterious to melanocytes when performed upon UV exposure, and phaeomelanin is suspected to increase risk of melanomagenesis. In fact, a large number of data, including ours, have shown that stimulation of pigmentation could potentiate UVA genotoxicity within melanocytes. In order to maintain redox homeostasis, the transcription factor Nrf2 controls various enzymatic activities involved in antioxidant status and phase II detoxification, and we were among the first who demonstrated the importance of its modulation in pigment cells. Moreover, using normal human keratinocytes/melanocytes from same donors in coculture, we also compared basal expression of about 200 genes controlling phase I or phase II metabolism. Half of genes were equally expressed, but some significant differences were observed particularly regarding protection against prooxidant species. For instance melanocytes displayed a higher expression of genes encoding quinone-oxidoreductases or catalase. In addition, impact of various stressors (daily UV, hydrogen peroxide, hydroquinone) on gene expression was assessed. Melanocytes were not as sensitive as keratinocytes to cytotoxicity, and gene induction profiles significantly differed according to cell type. These results show that melanocytes and keratinocytes sharing the same genetic background and growing in the same culture medium displayed differences in basal and stress-induced expression of genes controlling metabolism and antioxidant defenses.

IL25

Non-cultured cellular grafting in vitiligo: pearls and pitfalls

N. Van Geel
Ghent University, Ghent, Belgium

Surgical methods have been proposed as a therapeutic option in patients with stable vitiligo (e.g. segmental vitiligo). Different surgical techniques for repigmenting vitiligo have been devised over the years and include tissue grafts (full-thickness punch grafts, split thickness grafts, suction blister grafts) and cellular grafts (cultured melanocytes, cultured epithelial sheet grafts and non-cultured epidermal cellular grafts).
Non-cultured epidermal cell transplantation is an innovative surgical technique that provides the possibility to repigment vitiliginous skin manifold larger than the donor skin and can be completed in several hours (without culturing the cells). Since its introduction, several modifications and simplifications of the original technique have been described.
Differences in patient selection criteria and variation in the treatment procedure may explain the variety in treatment outcome or even total failure of the intervention. The most important selection criterion is the stability of the disease. Furthermore, treatment localization may influence repigmentation rates intra-individually. Variation of the technical procedure can exist at different levels: 1. Donor skin (e.g. technique and body location used to obtain autologous donor skin; thickness of the graft; ratio donor skin – recipient area). 2. Recipient area (e.g. dermabrasion technique used; depth of abrasion); 3. Preparation cellular suspension (e.g. trypsinisation time; use of reagents/media); 4.Application of cell suspension (e.g. use of cell carrier; dressing; fixation); 5. Post- operative care (e.g. immobilization treated area; time period bandage; use of adjuvant cream or UV treatment).
There is still ongoing research to simplify the procedure of non-cultured cellular grafting which may further increase the success rates and accessibility of this technique.

IL26

Surgical treatment in difficult sites

S. Mulekar[1,2]
[1]National Centre of Vitiligo and Psoriasis, Riyadh, Saudi Arabia; [2]Mulekar Clinic, Mumbai, India

Surgical treatment of vitiligo is indicated for patches refractory to medical treatment. The techniques are divided into tissue grafting and cellular grafting.
Tissue grafting methods have limitations that treated areas require immobilization, smaller areas can be treated in one operative session, and they are time consuming. Pediatric patients are not considered suitable for surgical treatment, most probably, due to these reasons. Certain other locations, such as eyelids, joints, male genitals are considered as difficult to treat sites
Cellular grafting methods do not have these limitations. Larger areas are treated in shorter time period, and immobilization of treated part is not required. There are no specific precautions to treat any anatomical site, including, eyelids, male genitals and joints.
The concept of difficult to treat sites is a relative term and depends upon the technique used. Cellular transplantation does not require any specific precautions, enabling treatment of all the anatomic sites easier than tissue grafting methods.

© 2014 John Wiley & Sons A/S. Published by John Wiley & Sons Ltd

IL27

Treasure hunt- hair follicle as a rich source of melanocytes and stem cells for transplantation in vitiligo

S. Gupta
All India Institute of Medical Sciences, India

Hair follicles play a role of melanocyte reservoir in repigmenting therapies in vitiligo. Melanocyte stem cell Niche is recognized in hair follicle but not in epidermis. Therefore, hair follicle is an attractive source of melanocytes for transplantation in vitiligo.
We used Follicular Unit Extraction (FUE) technique to harvest hair follicles from donor area. It has several advantages, such as it does not cause visible scarring, and unlike plucked hair, it retains all the ORS and stem cell populations. The follicles were then incubated with 0.25% trypsin–0.05% EDTA at 37°C for 90 min to prepare the single cell suspension. In epidermis, melanocytes are distributed only in basal layer, but they are distributed in the full thickness of outer root sheath of hair follicle. Therefore melanocyte yield is more in hair follicle.
There are several advantages of using hair follicle melanocytes for transplantation in vitiligo. It is hypothesized that hair follicle melanocytes express some antigen associated with alopecia areata but not those associated with vitiligo. This is evident from the fact that leukotrichia is a late event in vitiligo and gray hair follicle spared in alopecia areata. Epidermal melanocytes are a homogeneous population of highly dendritic and weakly pigmented cells. Hair follicle has three distinct populations of melanocytes; highly pigmented, dendritic bulbar melanocytes, less-differentiated tripolar cells, and undifferentiated amelanotic cells. The later ones are supposed to be less susceptible to autoimmune damage.
This procedure is still evolving and attempts are being made to refine it further.

IL28

How do melanocytes find their way?

I. J. Jackson[1,2], K. J. Painter[3], C. A. Yates[4], M. Keighren[1], O. Harrison[1], M. Ford[1], R. L. Mort[1]
[1]MRC Human Genetics Unit, Institute of Genetics and Molecular Medicine, University of Edinburgh; [2]Roslin Institute, University of Edinburgh, Edinburgh; [3]Mathematics Institute, University of Oxford, Oxford; [4]School of Mathematical and Computer Sciences, Heriot-Watt University, Edinburgh, UK

From their origins as migratory neural crest cells, melanoblasts find their way to several sites in the body. Many years ago we visualised migrating melanoblasts by in situ hybridisation, and could find them on several pathways, including towards the stria vascularis of the inner ear. Subsequent generation of lacZ reporter transgenic lines facilitated visualisation thoughout development and in the adult. Our ultimate goal of live-imaging of melanoblasts was achieved using the Tyrosinase-Cre transgene (from Lionel Larue) activating YFP at the ROSA26 locus and an embryonic skin explant culture to enable the generation of time-lapse videos.
We can measure the migration speed (or diffusion coefficient) and proliferation rate of the melanoblasts and use these parameters in mathematical models that explain the interactions between growth of the embryonic trunk, proliferation and migration of melanoblasts, and use iteration of modelling and experiment to refine our understanding of melanoblast localisation. We have used drugs and genetic mutations to alter signalling pathways, observed how these alterations affect the behaviour of the cells in the culture system, modelled the effect of these changes in silico, and compared the outcomes with that observed in mutant mice in vivo. We can thus explain the origin of the white belly-spot seen in Kit mutant mice in terms of the effect of Kit on melanoblast behaviour.
Using 2-dimensional models we find that our simulations are able to recapitulate the coat colour patterns seen in chimaeric mice, where there is a fairly balanced contribution of melanocytes of two pigmentation phenotypes, and those patterns seen in mosaics, in which there is a labelled melanocyte population deriving from a single cell during development.
We have also developed a new cell-cycle reporter mouse, based on the FUCCI system, that gives a robust readout of cell cycle parameters and which will be invaluable for further analysis of the cell cycle during embryogenesis.

IL29

Functional divergence of mammalian Melanocytes

H. Yamamoto
Nagahama Institute of Bio-Science and Technology, Nagahama, Japan

Melanocytes originate from the neural crest, a vertebrate-specific embryonic cell population, and migrate to a variety of tissues and organs, including the skin, choroid, inner ear, heart, brain, adipose tissue, lung, etc. Although we are under the impression that we understand the functions of melanocytes in the skin, their localization over extensive skin areas with local skin microenvironments suggests that they have new functions waiting to be uncovered. Meanwhile, the functions of melanocytes in the skin are relatively well known compared with the functions of noncutaneous melanocytes distributed elsewhere in the body, where only a very small fraction of light illuminates their existence. Here I would like to introduce melanocytes in the inner ear that are indispensable for hearing ability and those in the choroid of mice. I will discuss the contribution of noncutaneous melanocytes to the structures of their habitats.

IL31

Prohormone convertases and melanoma – emerging players in basic and translational research

M. Böhm
Department of Dermatology, Laboratory for Neuroendocrinology of the Skin and Interdisciplinary Endocrinology, University of Münster, Münster, Germany

Prohormone convertases (PCs) are well known for their proteolytic processing of various precursor hormones like proopiomelanocortin which are also autonomously expressed in the skin. However, PCs are capable of processing and activating other substrates than prohormones such as transcription factors, growth factors, growth factor receptors and matrix metalloproteinases. Recently, PCs have been implicated in the pathogenesis of non-melanoma skin cancer and melanoma. In this presentation the putative role of two distinct members of the PC family, subtilisin-kexin isozyme-1 (SKI-1) and paired basic amino-acid-cleaving enzyme 4 (PACE4), is highlighted for pigment cell biology and melanoma development. SKI-1 is one of the newest members of the PC family. It is constitutively expressed (and secreted) by normal human melanocytes and melanoma cell lines. Importantly, secreted SKI-1 enzyme activity is significantly elevated in the majority of human melanoma cell lines compared to normal melanocytes. Pharmacological

 © 2014 John Wiley & Sons A/S. Published by John Wiley & Sons Ltd

inhibition of SKI-1 by small peptide-based inhibitor not only induces apoptosis of melanoma cells in vitro but also reduced the vivo the growth of tumors established by s. c. injection of melanoma cells into immunodeficient mice. With regard to PACE4 we demonstrate that ectopic expression of this PC increases invasiveness and cell migration in vitro while having little impact on proliferation and melanogenesis. When injected s. c. into immunodeficient mice tumors arising from PACE4-transfected cells grow significantly faster than those from vector-alone-transfected cells. The relevance of these findings is further supported by the finding that the majority human melanoma cell lines express higher levels of both PACE4 RNA and protein. Moreover, PACE4 immunoreactivity is present in most advanced but not early primary melanomas. In summary, these findings indicate an emerging role of distinct members of the PC family, e. g. SKI-1 and PACE4, whose pharmacological inhibition may become a novel approach for the treatment of melanoma.

IL33

The role and regulation of MITF in melanoma heterogeneity

C. R. Goding

Ludwig Cancer Research, University of Oxford, Oxford, UK

One of the major barriers to effective anti-cancer therapy is tumour heterogeneity. In addition to genetic heterogeneity, sub-populations of cancer cells display phenotypic heterogeneity with some subpopulations of cells exhibiting non-genetic therapeutic resistance. In melanoma, phenotypic identity is regulated by the microphthalmia-associated transcription factor MITF that also plays a key role in the development of the melanocyte lineage where it promotes melanoblast survival and activates genes involved in pigmentation. MITF also plays a key role in regulation of proliferation. Low levels of MITF activity are characteristic of invasive cells, while MITF expression drives either proliferation or differentiation. Since phenotypic identity must be underpinned by differential gene expression, understanding how MITF targets different sets of genes in response to environmental cues to drive phenotypic identity will facilitate the development of more effective anti-melanoma therapies. Here our current understanding of how regulation of MITF effects the proliferation to differentiation switch will be discussed.

IL34

Defining the mode of melanoma heterogeneity by real-time cell cycle imaging

C. A. Tonnessen[1], K. A. Beaumont[2], D. S. Hill[2], S. Daignault[1], A. Anfosso[2], R. J. Jurek[3], W. Weninger[2,4,5], N. K. Haass[1,2,4,5]

[1]The University of Queensland Diamantina Institute, Translational Research Institute, Brisbane, QLD; [2]Centenary Institute; [3]CSIRO Astronomy & Space Sciences; [4]University of Sydney; [5]Royal Prince Alfred Hospital, Sydney, NSW, Australia

Melanoma drug resistance may be, in part, due to tumor heterogeneity. Heterogeneity is the occurrence of different sub-populations of cancer cells within a tumor, resulting in multiple cellular phenotypes within a single site. These populations can be proliferating or arrested, invading or quiescent. As these cancer cells exhibit variable behaviors, they also respond to therapies uniquely. Understanding the molecular signature influencing cancer cell activity within tumors is therefore crucial to design the most effective therapeutic regimen.

To better understand tumor heterogeneity within melanoma, cutting edge imaging technology and the fluorescence ubiquitination cell cycle indicator (FUCCI) system were employed to observe different phases of the cell cycle in real-time. Interestingly, it was found that tumor xenografts grown from melanoma cells produced two cohorts. One that contained distinct clusters of arrested or proliferating cells, and another that displayed a more homogenous dispersion. It was then determined these two phenotypes could be separated by microphthalmia-associated transcription factor (MITF) expression, with high MITF levels correlating with uniform cellular distribution. Furthermore, in WM164 cells, which normally give rise to homogenous tumor xenografts, knockdown of MITF by shRNA converted the phenotype to become clustered. Cells that express MITF were grown into 3D tumor spheroids embedded in collagen, and stained for MITF. MITF expression was found predominantly in the periphery of the spheroid, which also had high Slug and Vimentin expression, with a decrease in E-cadherin, indicative of an epithelial to mesenchymal transition (EMT). This area also corresponds with the region of highly proliferative cells. Additionally, serum starvation, resulting in cell cycle arrest, resulted in decreased MITF levels, and knockdown of MITF by shRNA gave rise to more cells arrested in G1.

These data outline how MITF and tumor heterogeneity are tightly intertwined within tumor architecture, making it an important marker for therapy design.

IL35

BRAF and RAS signalling: basic biology and therapeutic options

R. Marais

The CR-UK Manchester Institute, Manchester, UK

NRAS is a small G-protein and BRAF is a protein kinase that is activated downstream of NRAS. These proteins are components of the RAS/RAF/MEK/ERK pathway, and they regulate cell proliferation and survival. Critically, NRAS is mutated in ~20% of melanomas and BRAF is mutated in further ~40% of cases. Drugs that target BRAF or MEK can improve progression-free and overall survival in melanoma patients whose tumours carry mutations in BRAF, but these responses are generally short-lived and most patients relapse after a relatively short period of disease control. Additionally, ~20% of patients present with primary/intrinsic resistance and do not respond to BRAF/MEK inhibitors despite the presence of a BRAF mutation. Several mechanisms of resistance have been described and the majority involve reactivation of the MEK/ERK pathway driven by hyper-activation of receptor tyrosine kinases or the acquisition of mutations in RAS. Also, BRAF inhibitors drive a curious paradox, because although they inhibit MEK/ERK signalling in BRAF mutant cells, they activate the pathway in RAS mutant cells. We have developed mouse models of melanoma driven by oncogenic NRAS and oncogenic BRAF and used these to mimic the consequences of paradoxical activation of MEK/ERK signalling. We show that kinase-dead BRAF cooperates with oncogenic NRAS to drive melanomagenesis and also to stimulate melanoma cell invasion and metastasis, but these effects are suppressed when MEK is inhibited. These studies show that BRAF/MEK inhibitor combinations are more effective than BRAF inhibition alone at suppressing the on-target side-effects of BRAF drugs.

© 2014 John Wiley & Sons A/S. Published by John Wiley & Sons Ltd

IL36

Targeting resistance pathways in melanoma with epigenetic regulators

P. Hersey, S. Gallagher, J. Tiffen

Kolling Institute, University of Sydney, Sydney, NSW, Australia

In previous studies we have shown that pan-HDAC inhibitors can induce apoptosis in melanoma that is associated with upregulation of BIM, BAX and BIK and down regulation of Bcl-XL and XIAP (5, 18, 19). Such inhibitors were strongly synergistic with selective BRAF inhibitors in induction of apoptosis of melanoma (20, 21)and provide evidence that the oncogenetic process in melanoma is acting to inhibit apoptosis by preventing upregulation of Bim.We have extended these studies to the inhibitors of a highly conserved class of 'reader' proteins referred to as bromodomain and extraterminal (BET) proteins. The BET family, which consists of BRD2, BRD3, BRD4 and the testis specific BRDT, have two bromodomains in the N terminal region which bind to acetylated lysines in histones and a C-terminal (CT) region which binds to transcription elongation factors (TEFs) (24). The bromodomains act to target protein complexes to particular chromosomal regions involved in gene transcription and act as coregulators of transcription (25, 26).

We found that the inhibitor I-BET 151, had several important effects on melanoma cells that included cell cycle arrest in G1, induction of apoptosis (Gallagher et al 2014) and suppression of NF-kB activation (Gallagher et al 2014). Induction of apoptosis was cell line dependent but all lines had upregulation of BIM, the BH3 only pro apoptotic protein. Expression arrays showed variable reduction in anti apoptotic proteins which may account for the cell line dependency. The finding that both HDAC inhibitors and BET protein inhibitors upregulate BIM suggests that acetylation either at the level of BIM itself or transcription factors that regulate BIM are involved. Initial studies have shown strong synergistic effects when both agents are combined which may suggest BET proteins are inhibitory for expression of BIM.

The EZH2 (enhancer of zeste homolog 2) protein is part of the polycomb repressive complex 2 (PRC2) that is conserved across organisms from plants to humans (28) and mediates its effect by tri- methylation of lysine 27 on H3 histones (H3K27me3) to repress transcription. We have used the GSK126 inhibitor to explore the role of EZH2 in melanoma biology including 4 melanoma lines that have activating mutations in EZH2. The mutated lines were relatively sensitive to the drug in apoptosis assays as were about a third of lines with wild type EZH2. The levels of trimethylation on K27 appeared to be one of the markers of responsiveness suggesting that EZH2 may be suppressing proapoptotic genes. These results suggest drugs that regulate epigenetic processes have potential as therapeutic agents in treatment of melanoma and are potentially targetable to particular melanoma in combination with other agents such as immunotherapy.

IL37

Inflammation-induced phenotypic plasticity of melanoma in therapy resistance and tumor progression

T.Tüting

Laboratory of Experimental Dermatology, Department of Dermatology and Allergy, University of Bonn, Bonn, Germany

These results suggest drugs that regulate epigenetic processes have potential as therapeutic agents in treatment of melanoma and are potentially targetable to particular melanoma in combination with other agents such as immunotherapy.

Genetically engineered mouse melanoma models enable new insights into pathogenesis and treatment of melanoma. We established the genetically engineered HGF-CDK4(R24C) mouse strain in our laboratory, where invasively growing primary melanomas fail to spontaneously activate the type I IFN system in the tumor microenvironment and consequently do not effectively stimulate a tumor-specific T cell response. These mouse melanomas morphologically imitate human melanomas without tumor-infiltrating lymphocytes that occur in a subgroup of patients and are associated with a poor prognosis. One approach to enforce T cell immunosurveillance of immune cell-poor HGF-CDK4(R24C) mouse melanomas consisted of adoptive cell transfer therapies (ACT) using pmel-1 TCRtg T cells that specifically recognize the melanocytic differentiation antigen gp100. This experimental model faithfully recapitulated tumor regression, remission and relapse following ACT as seen in patients. Hypotheses explaining the acquired resistance to ACTs include the immunoselection of tumor cell variants with persistent genetic loss of the antigen, as predicted by the immunoediting theory, and the suppression of T-cell effector functions in the tumor microenvironment. Our experimental observations indicated that tumor relapse after initially successful T-cell immunotherapy also involved a principally reversible adaptive process of dedifferentiation driven by proinflammatory mediators such as TNF secreted by tumor-infiltrating immune cells in the tumor microenvironment. A second approach to expose immune cell-poor HGF-CDK4(R24C) mouse melanomas to immune surveillance consisted of targeted activation of type I IFNs with polyinosinic:polycytidylic acid (pI:C). However, IFNs upregulate the expression of PDL1 receptors on melanoma cells which allows them to engage immune-inhibitory PD1 receptors on infiltrating T and NK cells. Treatment with PD1 blocking mAbs further promoted the anti-tumor efficacy of immunostimulatory RNA. Taken together these observations contribute to the growing body of evidence which shows that melanoma cells exist in a variety of dynamically interconvertible phenotypic states. This phenotypic plasticity includes but is not limited to differentiated and dedifferentiated subpopulations and allows melanoma cells to rapidly adapt inflammatory and hypoxic environments during tumor progression and in response to therapeutic intervention.

IL38

Non-invasive imaging of neo-lymphangiogenesis for the identification of metastatic niches and anticancer agents in melanoma

X. Cerezo-Wallis[1], T. G. Calvo[1], E.-R. Falkenbach[1], S. Ortega[2], M. S. Soengas[1]

[1]Melanoma Laboratory, Molecular Pathology Programme, Spanish National Cancer Research Centre (CNIO); [2]Transgenic Mice Unit, Biotechnology Programme, Spanish National Cancer Research Centre (CNIO), Madrid, Spain

Metastatic dissemination of cancer cells is a complex process invariably associated with neo-vascularization. Intriguingly, while the presence of malignant cells in lymph nodes is a defining criteria in tumor staging, the specific contribution of lymphangiogenesis to tumor progression and drug response remains largely unknown. This is mainly due to the lack of markers and amenable models for the imaging and analysis of lymphangiogenesis by non invasive methods in vivo. We have generated a new series of 'lymphoreporter' mouse strains whereby a GFP-luciferase fusion cassette was inserted by knock in technologies at the 3′ UTR region of Flt4 (VEGFR3), a

© 2014 John Wiley & Sons A/S. Published by John Wiley & Sons Ltd

classical marker of lymphangiogenesis. Here we exploit immunocompetent and immunodeficient lymphoreporter animals in the context of malignant melanoma, the most lethal form of skin cancer. We will show how non-invasive monitoring of VEGFR3 activation can reveal pre-metastatic niches activated before the onset tumor cell colonization. Moreover, the lymphoreporter mice was used as a platform to identify novel antimetastatic agents and define their mode of action. We will present dsRNA-based nanoparticles with a potent anti-melanoma activity in vivo derived from a novel dual mode of action: (i) direct tumor-self killing by autophagy, (ii) selective inactivation of the lymphangiogenic vasculature. Together, our data validate the VEGFR3-GFP-Luc reporter as a new cost-effective system for gene discovery and drug efficacy assessment in physiologically-relevant cancer models.

IL39

Melanocyte senescence pathways: the impact of p16 defects

D. C. Bennett[1], C. J. Cairney[2], J. S. Kohli[1], D. M. Kallenberg[1], W. N. Keith[2]

[1]St George's, University of London, London; [2]University of Glasgow and CRUK Beatson Laboratories, Glasgow, UK

Cell senescence is a permanent arrest of cell division, which follows excessive proliferation, oncogene activation and some kinds of cellular stress. The genetic changes seen in both familial and sporadic human melanoma include a number of common changes all of which have the effect of disrupting cell senescence and leading towards cell immortality (where cells can divide for ever). These include specific defects in the genes *CDKN2A, CDK4, PTEN* and *MITF*, and activating promoter mutations in *TERT*. These and other findings imply that cell senescence is a powerful mechanism for the suppression of potential melanoma after oncogene activation in a melanocyte, yielding benign naevi instead.

We are working to understand the mechanisms of action of *CDKN2A*. This is the most commonly affected locus in familial melanoma, and encodes the protein p16, which is also defective or silenced in around 80% of sporadic melanomas. p16 is an inhibitor of CDK4 (cyclin-dependent kinase 4), which is needed for S-phase of the cell cycle. p16-deficient human melanocytes, or melanocytes following p16 knockdown, have a greatly extended lifespan (they divide many more times than normal before senescing), showing p16 is needed for melanocyte senescence on schedule. Nonetheless these melanocytes do still senesce.

Microarray analyses of gene expression when normal and p16-deficient melanocytes senesce have supported the idea that additional cell cycle functions for CDK4 – transit of G2 and M – are also important and inhibited in cell senescence. Some specific molecular changes among these have been validated by PCR and at the protein level, and are providing potential new markers of normal and delayed senescence, for in-vivo testing as melanoma/naevus markers.

IL40

Senescence in melanoma: a stab in the back?

R. Ballotti

INSERM, Rouen, France

Together with apoptosis, senescence is a cellular failsafe program that counteracts cancer cell development. Chemotherapy-induced reduction in tumor load mainly functions through apoptotic cell death. However, the effectiveness of these pro-apoptotic therapies is compromised by alterations in the apoptotic programs. This is especially true for melanoma, which is known for its notorious resistance to apoptosis. Senescence has been considered as an alternative potent tumor-suppressor mechanism firmly halting tumor progression, as illustrated for nevi that usually remain unchanged for decades. Therefore, the induction of senescence may be exploited as an anti-melanoma therapeutic strategy. The transcription factor MITF is the master gene of the melanocyte lineage. We showed that the sustained inhibition of MITF triggers senescence of melanoma cells. Further, MITF can overcome the program of senescence and can favor melanoma development. We recently identified a gain of function mutation of MITF that predisposes to melanoma ($MITF^{E318K}$). We demonstrated in vitro that $\mathrm{MITF}^{\mathrm{E318K}}$ increases the tumorigenic properties of melanocyte cells. In vitro and in mouse model, $\mathrm{MITF}^{\mathrm{E318K}}$ is more potent than wild type MITF to bypass the oncogene-induced senescence program elicited by $\mathrm{BRAF}^{\mathrm{V600E}}$. These data suggest that re-activation of the senescence program in melanoma might by a useful therapeutic strategy. However, in addition to these beneficial effects, senescence might have deleterious consequences. Indeed, we showed that senescent melanoma cells produced a pro-inflammatory secretome with pro-tumorigenic properties that increases the number of melanoma initiating cells. This non-autonomous cell process might play a key role in tumor development, metastasis dissemination, as well as secondary drug resistances and relapses.

IL41

The genetic architecture of melanoma predisposition

N. Hayward

QIMR Berghofer Medical Research Institute, Brisbane, Qld, Australia

Family and twin studies indicate that melanoma susceptibility has a strong genetic component. Very rarely, melanoma runs in families in which there is an inherited mutation in a single 'high penetrance' gene, but in the general population melanoma susceptibility is thought to be governed by variation in a combination of 'low penetrance' genes. To date, genome-wide association studies (GWAS) have identified 17 low penetrance loci associated with melanoma susceptibility in the general population. The majority of these loci are related to the well characterised melanoma risk phenotypes of light pigmentation and high numbers of melanocytic naevi. They include variants in or near pigmentation genes: MC1R, TYR, SLC45A2, IRF4 and TYRP1; and genes associated with variation in naevus count: MTAP/CDKN2A, PLA2G6, IRF4 and TERT/CLPTM1L. Seven additional melanoma susceptibility loci not associated with either of the melanoma risk phenotypes of pigmentation or naevi have been identified. These lie in or near ATM, CASP8, CCND1, MX2, FTO, PARP1 and SETDB1, suggesting a number of other pathways are involved in melanoma susceptibility. Determining the mechanisms by which the other low penetrance loci confer risk is an area of active investigation and should provide significant new insights into the underlying aetiology of melanoma. The ATM and PARP1 loci allude to involvement of DNA repair pathways, while CCND1 and CASP1 point to regulation of cell proliferation or death respectively. Meta-analysis of all existing melanoma GWAS datasets is currently underway to identify further melanoma risk alleles affecting the population at large.

© 2014 John Wiley & Sons A/S. Published by John Wiley & Sons Ltd

Germline mutations in the high penetrance melanoma susceptibility genes CDKN2A and CDK4 account for melanoma development in ~40% of highly case-dense families. Additionally, rare mutations in BAP1 have been associated with uveal and cutaneous melanoma predisposition. Most recently, germline gain-of-function mutations in the promoter of TERT, as well as inactivating mutations in the shelterin component POT1, implicate telomere dysregulation as a novel risk pathway in familial melanoma. To identify other high penetrance melanoma susceptibility genes we carried out whole-exome sequencing of DNA from 105 cases from 51 multi-case melanoma families. This led to the identification of a variant in MITF, the lineage specific transcription factor and oncogene for melanoma, which was 2.33 times more prevalent in melanoma cases than population controls, indicating it is a medium-penetrance melanoma susceptibility variant. Other candidate familial melanoma genes are currently undergoing functional assessment.

IL42

Role and processing of miRNAs in melanoma

A. Bosserhoff

University of Regensburg, Regensburg, Germany

MicroRNAs (miRNAs) are small non-coding RNA molecules which regulate gene expression on post-transcriptional level. Recent research not only revealed their strong impact on almost every regulatory pathway exerted in eukaryotic cells but also clearly demonstrated their involvement in the tumorigenesis of a variety of human cancers. Studies from our own group revealed that the expression of miRNAs like let-7a, miR-125b and miR-196a is deregulated in malignant melanoma cells and that deregulated miRNA expression is undoubtedly linked to important processes affecting melanoma formation and progression. Moreover, we demonstrated regulation of the transcriptional regulator c-Jun by miR-125b on protein but not on mRNA level.

Processing of microRNAs (miRNAs) is a multistep and highly controlled process. Commonly, the deregulated expression of miRNAs was attributed to chromosomal aberration or promoter regulation but changes in the miRNA processing enzymes have not been analyzed in detail. In melanoma, we revealed strong reduction of Argonaute2 (AGO2, *EIF2C2*) protein expression, a key player of the RNA-induced silencing complex (RISC), compared to primary melanocytes. Interestingly, this change was not observed in other kinds of cancer. We could show that re-expression of AGO2 in melanoma cell lines leads to increased functionality of siRNAs and shRNAs and revealed strong improvement of regulatory effects. Changes in AGO2 expression result in modulated functionality of regulatory RNAs and have consequences for therapeutic applications of short interfering RNAs (siRNAs), short hairpin RNAs (shRNAs) and miRNAs.

IL43 ASPCR John Pawelek Lecture

Vascular characteristics of melasma and therapeutic implications

H. Y. Kang

Department of Dermatology, Ajou University School of Medicine, Suwon, Republic of Korea

Melasma is a common hyperpigmentary disorder of the face. A major clinical feature of melasma is the hyperpigmented patches, but melasma patients have additional distinguishing features like pronounced telangiectatic erythema confined to the melasma lesional skin. It was shown that melasma lesions have more vascularization as compared to the perilesional normal skin. The erythema index was significantly higher in the lesional skin than in the perilesional normal skin. The melanin and erythema index showed a positive correlation. It was suggested that 'erythema' in melasma reflect the severity of disease. Targeting blood vessels along with the melanin pigment would be beneficial for the treatment of melasma. The effects of pulsed dye laser and oral tranexamic acid in the treatment of melasma underline the potential interest of targeting the vascular component for treating melasma. The biological role of cutaneous blood vessels in the regulation of melanogenesis remains an interesting topic for future studies.

IL44

Reduced H19 RNA in melasma: role of miR-675 through its direct target

A. Y. Lee

Department of Dermatology, Dongguk University Ilsan Hospital, Graduate School of Medicine, Republic of Korea

Significant clinical correlation was identified with downregulation of the H19 gene on microarray analysis of hyperpigmented and normally pigmented skin from melasma patients. H19 non-coding RNA downregulation stimulates melanogenesis and melanosome transfer in melasma patients. Because methylation of regulatory factor, maternal imprinting control region, was not involved in the H19 downregulation, the potential role of a H19 microRNA, miR-675, in melanogenesis was examined. Real time PCR using cultured normal human skin keratinocytes, melanocytes, and fibroblasts with or without H19 knockdown showed accompanying changes between expression levels of H19 and those of miR-675 in keratinocytes. MiR-675 was also detected in concentrated culture supernatants and showed expression levels parallel with those of cell lysates. In addition to RNase resistance, FACS analysis showed anti-CD63-positive exosomes in culture supernatants, suggesting miR-675 could be released extracellularly and delivered to neighbouring cells without degradation. In western blot analysis, the miR-675 mimic reduced the expression of MITF and phosphorylation of CREB, ERK and AKT, whereas these expressions were increased by the miR-675 inhibitor. Although H19 was not a miR-675 target, luciferase reporter assay showed a direct binding of miR-675 to 3′-UTR of MITF. In addition, localized in vivo miR-675 overexpression in mouse using a cationic polymer transfection reagent showed reduced mRNA expression levels of MITF, tyrosinase, Trp-1, and Trp-2. Collectively, the results suggest that miR-675 derived from keratinocytes could be involved in H19-stimulated melanogenesis using MITF as a target of miR-675.

IL45

Laser treatment of melasma in Asians

P. Wattanakrai

Mahidol University, Bangkok, Thailand

Laser treatment of melasma in Asians with low fluence 1064-nm Q-switched neodymium-doped yttrium aluminum garnet (QS-Nd: YAG) laser has recently gained increased popularity. My recent studies demonstrated that after 5 Q-switched Nd:YAG laser treatments produced significant and rapid results. There was also an additional photorejuvenation effect. However the results were not curative and recurrence of melasma was the rule. The complications of the treatments included

© 2014 John Wiley & Sons A/S. Published by John Wiley & Sons Ltd

postinflammatory hyperpigmentation, mottled hypopigmentation, rebound hyperpigmentation and melasma recurrence. To avoid these serious complications, we caution the use of too many or too frequent QSNd:YAG laser sessions and emphasize the careful surveillance of development of hypopigmentation or leucoderma, which should contraindicate further QS-Nd:YAG laser treatments. Current research, pearls and pitfalls of this laser treatment for melasma will be discussed.

IL46

Interaction between UVA and eumelanin but not phaeomelanin results in melanoma

F. P. Noonan

George Washington University, Washington, DC, USA

The development of mouse models has facilitated investigations into the role of UV radiation in mammalian melanoma. We have used precise spectrally defined UVA (320–400 nm) or UVB (280–320 nm) at biologically relevant doses in a mouse model to identify two UV wavelength-dependent pathways for induction of CMM. Mice transgenic for the multifunctional growth factor hepatocyte growth factor (HGF/SF) show black hyperpigmentation on the C57BL/6 genetic background resulting from extra-follicular melanocytes at the epidermal/dermal junction and develop melanomas recapitulating human disease after UV exposure as neonates. Induction of melanoma by UVA was dependent on the presence of melanin and was associated with melanin-dependent oxidative DNA damage in melanocytes. Skin pigment was exclusively eumelanin and was largely confined to melanocytes with sparse protective epidermal melanin, resulting in direct exposure of melanocytes to UV. Initiation of melanoma by UVB was associated with direct UVB-type DNA damage, independent of melanin. Recessive yellow mice (C57BL/6e/e), with an inactivating mutation in the melanocortin-1 receptor (Mc1r), produce more phaeomelanin than eumelanin and exhibit yellow pigmentation. Yellow C57BL/6e/e-HGF mice, however, produced no melanomas either spontaneously or in response to UV, although the HGF transgene and its receptor, Met, were expressed. Melanin hyperpigmentation was not observed in the yellow C57BL/6e/e-HGF mice and there were fewer extra-follicular melanocytes than in black C57BL/6-HGF animals. In heterozygous C57BL/6e/+-HGF mice, however, the HGF transgenic black phenotype was restored and the number of extra-follicular melanocytes and levels of black melanin in the skin were equivalent to the parent C57BL/6-HGF. Significantly, statistically fewer UV and spontaneous melanomas were produced. Thus a previously unsuspected, melanin-independent, interaction between the Mc1r and HGF signalling pathways is required for HGF-dependent UV melanoma. Notably, HGF has recently been shown to be important in treatment failure and conferring resistance to B-RAF inhibitors. We thus propose that this previously unrecognized MC1r/MET interaction may have a role in human melanoma and that understanding this interaction is important in view of the potential use of MET inhibitors in the treatment of human disease.

IL47

UV-irradiation promotes melanoma metastasis

T. Bald

University of Bonn, Bonn, Germany

Intermittent intense ultraviolet (UV) exposure represents an important etiologic factor in the development of malignant melanoma. The ability of UV radiation to cause tumour-initiating DNA mutations in melanocytes is now firmly established. It has been hypothesised that the effects of UV radiation on epidermal keratinocytes and immune cells additionally promote melanoma development by stimulating the survival, proliferation and migration of DNA-damaged melanocytes. How these microenvironmental effects of UV radiation influence melanoma pathogenesis is incompletely understood. Here we report that repetitive UV exposure of primary cutaneous melanomas in a genetically engineered mouse model selectively promoted systemic metastatic disease progression independent of its tumour-initiating effects. Specifically, UV irradiation enhanced the expansion of tumour cells along blood vessel surfaces in a pericyte-like manner and increased the number of lung metastases. This effect depended on toll like receptor (TLR) 4 driven recruitment and activation of neutrophil leukocytes initiated by the release of high-mobility group box 1 (HMGB1) from UV-damaged epidermal keratinocytes. TLR4-driven neutrophilic inflammation stimulated angiogenesis and promoted the ability of mouse melanoma cells to migrate towards endothelial cells. Furthermore, endothelial cells possess selective cues to promote melanoma cell motility on their surfaces. Our results reveal how UV radiation is sensed by epidermal keratinocytes and show that the resulting neutrophilic inflammatory response catalyses reciprocal melanoma-endothelial interactions and drives perivascular melanoma cell invasion, a phenomenon originally described as angiotropic growth by histopathologists in human melanomas6. Angiotropism represents a hitherto underappreciated mechanism of metastasis7, that also increases the likelihood of intravasation and dissemination via the blood. Consistent with our findings, neutrophil infiltration and reactive angiogenesis in ulcerated primary melanomas is associated with angiotropism and metastatic progression in this patient subgroup. A better understanding of inflammation-induced interactions between tumour and endothelial cells will reveal new treatment approaches to prevent the metastatic progression of high-risk primary melanomas.

IL 48

Beta-catenin signalling pathway in melanoblasts and melanoma

L. Larue

Institut Curie, Normal and Pathological Development of Melanocytes, CNRS, UMR3347, INSERM U1021, Orsay, France

Melanocyte transformation is a combinatorial multi-step, multi-etiological event that results in one of the most lethal types of skin cancer, melanoma, with the average 5 years' survival prognosis of patients with metastatic melanoma less than 5%. Recent evidence has solidified the involvement of several signalling pathways with altered functions in melanoma, MAPK, PI3K and Wnt/beta-catenin. The modulation of beta-catenin expression affects not only proper development and maintenance of melanocytes, but also melanomagenesis. Unlike its positive role in stimulating proliferation, migration and invasion of carcinoma cells, beta-catenin signalling decreased proliferation and migration of melanocytes and melanoma cell lines. In vivo, beta-catenin signalling in melanoblasts reduced the proliferation and migration of these cells, causing a white belly-spot phenotype. The inhibition by beta-catenin of migration was dependent on MITF-M, a key transcription factor of the melanocyte lineage, and CSK, an Src-inhibitor. Moreover, beta-catenin may induce the bypass of senescence in vitro and in

© 2014 John Wiley & Sons A/S. Published by John Wiley & Sons Ltd

vivo after repressing p16INK4A. Interestingly, beta-catenin activity is regulated by PTEN through Cav1 and not through AKT/GSK3, as expected. In conclusion, beta-catenin has apparent conflicting roles during melanomagenesis. These results highlight that melanoma initiation and progression, including invasion and metastasis formation require a series of successful cellular processes, any one of which may not be optimally efficient.

IL50

Pigmentary challenges in Asian skin

B. K. Goh
Skin Physicians @ Mount Elizabeth, Singapore

Pigmentary disorders in Asian skin can be a challenge to diagnose and treat, as they are not well characterized in standard dermatological textbooks. In Oriental skin, melasma co-exists with Hori's naevi, solar lentigines and freckles, and differentiating them is important as the therapeutic interventions differ. Often misdiagnosed as melasma, lichen planus pigmentosus, ochronosis and drug-induced facial pigmentation are important differential diagnoses to highlight in Asian skin. Besides these conditions, there are disorders that may only manifest in or are preponderant in the skin of colour; they include the guttate leukoderma of Darier's disease, amyloidosis cutis dyschromica, and achromic pityriasis lichenoides. To the untrained eyes, these unusual pigmentary conditions are diagnostic challenges. Their features and diagnostic approaches will be presented in this lecture, together with other 'dermatological gems' such as Dowling-Degos disease, Galli-Galli disease and prurigo pigmentosus.

IL51

Evaluation and management of post-inflammatory hyperpigmentation

A. Pandya
USA

Postinflammatory hyperpigmentation (PIH) is a common, acquired, transient excess of skin pigment that follows inflammation or injury to the skin. Those with Fitzpatrick skin types IV-VI are the most susceptible. PIH typically manifests as macules or patches at the site of previous trauma or inflammation. Lesions range from light brown dark gray depending on whether the excess melanin resides in the epidermis or dermis. Inflammatory mediators, such as IL-1α, endothelin-1, and reactive oxygen species, increase the synthesis of melanin after inflammation. Hyperpigmentation is particularly difficult to treat with follicular, basal layer, or basement membrane involvement. Pigment incontinence leads to dermal melanophages which are isolated from the effects of treatment, helping to explain the long time course of persistent lesions. The most important component of management is eliminating all inflammation from affected areas by using a treatment tailored to the underlying condition. Hydroquinone, topical retinoids, azelaic acid, kojic acid, glycolic acid peels, and combination cream with hydroquinone, tretinoin, and fluocinolone have been successfully used. The treatment course is often prolonged and may result in no benefit. Sun avoidance and use of broad-spectrum sunscreens are essential, especially in individuals with higher skin phototypes who may not be aware of the darkening effects of UV radiation. Evidence for the various available treatment modalities for PIH is not strong, and is underpowered by small population size, poor study design, and lack of validated outcome measures. Few studies focus on dark-skinned patients. The pathogenesis, evaluation and treatment of PIH will be reviewed during this presentation.

IL54

Skin pigmentation in health & disease: novel and seasoned players

P. Grimes
Vitiligo and Pigmentation Institute of Southern California, Los Angeles, CA, USA

In health, the mammalian pigmentary system and its purveyor of physiology and function, the melanocyte and melanin are responsible for a variety of cutaneous events, including skin and hair color and protection of the skin from the deleterious effects of ultraviolet light. Melanocytes, derived from neural crest cells, migrate to the basal layer of the epidermis and hair follicles. The migration and proliferation of these cells and the synthesis of melanin is indeed a complex process. Innate or constitutive pigmentation is influenced by multiple extrinsic and intrinsic factors. Extrinsic factors include ultraviolet light and drugs, whereas intrinsic factors include myriad genes, proteins, hormones and growth factors. Some of the key players in pigmentation include tyrosinase, TRP1, TRP2, melanocortin 1 receptor and its agonists, α-MSH, micropthalmia-associated transcription factor (MITF) and Wnt glycoproteins. In diseased states, disruption of melanin pathways may result in pigmentary disorders characterized by hyperpigmentation, hypopigmentation or depigmentation. Our enhanced knowledge of the mechanisms involved in melanocyte and melanogenesis dysregulation is the investigative canvas for novel therapeutic interventions. While seasoned players such as hydroquinone, kojic acid, arbutin, and retinoids work for hyperpigmentation, there is an intense interest and quest for novel players with enhanced efficacy. Novel agents for hyperpigmentation include MITF antagonists, β-adrenergic receptor and α-MSH receptor antagonists. Novel agents for vitiligo include afamelanotide, a synthetic analogue of α-MSH and agents reducing oxidative stress.

IL55

Coupling of the stress sensitivity of melanocyte stem cells to their dormancy during a hair cycle

E. Nishimura
Deparment of Stem Cell Biology, Medical Research Institute, Tokyo Medical and Dental University, Tokyo, Japan

Current stem cell studies have revealed that stem cells are more radiosensitive than mature cells. As somatic stem cells are mostly kept in a quiescent state, this conflicts with Bergonié and Tribondeau's law that actively mitotic cells are the most radiosensitive. In this study, we focused on hair graying to understand the stress-resistance of melanocyte stem cells (McSCs). We used Dct-H2B-GFP transgenic mice which enable the stable visualization of McSCs and an anti-Kit monoclonal antibody which selectively eradicate amplifying McSCs and found that quiescent McSCs are rather radiosensitive but the coexistence of quiescent and non-quiescent McSCs provide the stem cell pool with radioresistance. The irradiated quiescent McSCs prematurely differentiate in the niche upon their activation without sufficiently renewing themselves nor providing mature melanocytes to the hair bulb for hair pigmentation. These data indicate that tissue radiosensitivity is largely dependent on the state of somatic stem cells under their

 © 2014 John Wiley & Sons A/S. Published by John Wiley & Sons Ltd

local microenvironment. Furthermore, the coexistence of non-quiescent McSCs in the niche ensures the resistance of the McSC pool to different kinds of stresses to prevent hair graying.

IL56

Neuroendocrine control of human hair pigmentation – an update

D. J. Tobin
Centre for Skin Science, School of Life Sciences, University of Bradford, Bradford, UK

The establishment of the sub-field of cutaneous neuroendocrinology has significantly increased our understanding of skin function both at the organ and organismal levels. In particular, there is an emerging appreciation of the role of the recently-described hypothalamus-pituitary-adrenal axis equivalent and the hypothalamus-pituitary-thyroid axis equivalent in skin health and disease, as well as the wider skin-brain axis. While the involvement of the proopiomelanocortin-derived peptides alpha-melanocyte stimulating hormone (a-MSH) and adrenocorticotropin hormone (ACTH) in regulating hair follicle melanogenesis is well known, this now extends to beta endorphin (b-end), corticotropin releasing factor (CRF), thyrotropin-releasing hormone and beyond. Not surprisingly perhaps these mediators not only impact on hair pigmentation but also on other hair follicle functions including hair growth and keratinization and hair follicle immune status etc. This so called 'self-similarity' of the melanocortin and thyroid hormones in this mammal-specific mini-organ (ie the hair follicle, akin to their expression at systemic i.e. CNS levels) is quite remarkable and points to an even greater roles for this skin appendage in stress sensing at the periphery. This presentation will provide a timely update of the hair follicle as both a source of and a target for an increasingly diverse range of neuro-endocrine mediators, many of which are produced by and target the hair follicle pigmentation system.

IL58

Human pigmentation – the colours of skin

N. Kollias
Watertown, MA, USA and University of British Columbia, Vancouver, BC, Canada

The perception of visual information, color in an image, is an inference; in documenting skin color we measure color coordinates or concentration and distribution of chromophores successfully yet we try to separate the measurements from emotion and symbolism that are integral parts of color in art. In grading the perceived pigment in skin we use the rods and cones of the retina and the L*, a*, b* or the R, G, B parameters instrumentally. Then classification of the pigment level of human subjects should be an easy task based on these measures yet our eyes tell of a more complex situation. The classification of ethnicities may not be accomplished based on optical measurements alone. The constitutive pigment eumelanin presents spectral qualities that require a multicomponent system in order to describe spectral measurements and the range of colors of facultative pigmentation requires a further expansion of what may be considered as the fundamental constituents.

The pigment responses of skin due to any specific waveband may not be predicted based on the resident pigment and the responses are often due to multiple chromophores over their course of development. Analysis of skin color in terms of chromophores, inadequate as it is, does provide insights on the sequence of events in the evolution of facultative pigmentation and of the mixture of chromophores necessary. Study of pigmentation at the microscopic level provides only weak links with the observable colors and provides evidence for a mosaic structure for the epidermis that needs better definition. Induction of pigmentation at a microscopic scale produces events that are both familiar and curious deserving further study. Finally utilization of 3D instrumentation together with the development of software allows documentation of pigment disorders as vitiligo.

IL60

Recent advances on laser tattoo removal

C. L. Goh
National Skin Centre, Singapore City, Singapore

In the past tattoo removal involves tissue-destructive techniques including excision/grafting, dermabrasion, cryosurgery and electrosurgery. Although effective at removing the tissue pigments, these techniques often cause scaring, textural changes, keloids and permanent dyschromia. When Anderson and Parrish's introduced the theory of selective photothermolysis in 1981, pigment specific lasers were developed and this revolutionized the tattoo removal process. By preferentially targeting specific tattoo pigment with specific wavelengths and pulse durations of laser light, the tattoo pigments were selectively disrupted while adjacent structures are undamaged. At present, the nanosecond pulse duration lasers e.g. Q-switched ruby (694 nm), Nd:YAG (1064 and 532 nm), alexandrite (755 nm) lasers are lasers of choice for the removal of most tattoos. Recently a R20 treatment regimen was introduced to enhance tattoo removal. But this is a consuming procedure. Topical perfluorodecalin was recently reported to resolve immediate whitening reactions and allows rapid effective multiple pass treatment of tattoos. Another significant recently development in laser tattoo removal is the introduction of the 755 nm picosecond alexandrite laser was introduced to enhance laser tattoo removal.

The common complications include scaring, skin textural changes, incomplete removal of pigment and pigment darkening. Pigment darkening is often seen upon treatment of flesh-tone, red-brown, or off-white tattoo colors with the Quality-switched lasers. Chemical reduction of iron and titanium is thought to be responsible for the potentially permanent darkening of tattoos5. Paradoxical darkening after treatment with QSL systems is most likely thought to be pulse duration, wavelength, and fluence dependent. Repeated Q-switched laser treatments of up to 10–30 sessions can lighten these laser-darkened tattoos, other non-selective modalities are often required to shorten the treatment course. These include ablative and nonablative fractionated lasers. Fractionated photothermolysis uses microbeams to damage an array of microscopic columns of skin. This technique has recently been reported to be effective in removing cosmetic tattoos. Fractionated photothermolysis damage the dermal-epidermal junction and epidermis and allows egress of dermal tattoo pigments. The surrounding tissue is not involved which allows for fast epidermal repair. Combination of lasers may be necessary to effectively treat the broad spectrum of colors within cosmetic tattoos.

© 2014 John Wiley & Sons A/S. Published by John Wiley & Sons Ltd

IL61

Increasing the complexity: new genes and new types of albinism

L. Montoliu, M. Martínez-García, A. Fernández

Molecular and Cellular Biology, CNB-CSIC and CIBERER-ISCIII, Madrid, Spain

Albinism is a rare genetic condition affecting 1:17000 individuals in Europe and North-America although different frequencies have been reported in Asia and Africa. Albinism is globally characterized by severe and handicapping deficits in the visual system in association with a most variable hypopigmentation phenotype. Visual deficits include foveal hypoplasia, reduced pigmentation of retinal pigment epithelium cells, misrouting of the optic nerves at the chiasm, reduced pigmentation in the iris, photophobia and nystagmus. The hypopigmentation phenotype might affect the eyes, skin and hair (oculocutaneous albinism, OCA), or only the eyes (ocular albinism, OA). In addition, there are several syndromic forms of albinism (e.g., Hermansky-Pudlak and Chediak-Higashi syndromes, HPS and CHS, respectively) in which the described hypopigmented and visual phenotypes coexist with more severe pathological alterations. To date, mutations in up to 18 genes have been associated with different types of albinism. Recent uncovered genes include a locus that has been mapped to the 4q24 human chromosomal region associated with OCA, termed OCA5, while the gene itself has not yet been fully identified. Two additional new genes have been identified as causing OCA when mutated: SLC24A5 and C10orf11, and hence designated as OCA6 and OCA7, respectively. Finally, a large collaborative team recently reported mutations in the SLC38A8 locus causing foveal hypoplasia and optic nerve misrouting defects in the absence of obvious pigment alterations, a new syndrome termed FHONDA (abbreviation of foveal hypoplasia, optic nerve decussation defects and anterior segment dysgenesis) whose parallelism with albinism phenotype is challenging and has been discussed (Montoliu and Kelsh, 2014). This recent report about FHONDA suggests that mutations in albinism-associated genes might not necessarily all display pigment alterations, and instead the visual alterations should perhaps be the common and defining trait for this group of rare diseases. This presentation will summarize a recently published consensus review, in Pigment Cell & Melanoma Research (Montoliu et al. 2014), involving all laboratories that have reported these new genes, aiming to update and agree upon the current gene nomenclature and types of albinism, while providing additional insights from the function of these new genes in pigment cells

Acknowledgements: The associations in support of people with albinism (i.e. ALBA in Spain, Genespoir in France, . . .) are greatly acknowledged for their continuous support. This work has received the support of the Spanish Rare Disease Initiative (CIBERERISCIII), the local Madrid Autonomous Government and the Spanish Ministry of Economy and Competitiveness (MINECO).

IL 62

Mutation of SLC24A5 causes OCA6

A. Wei[1,2], Z. Zhang[1], W. Li[1]

[1]State Key Laboratory of Molecular Developmental Biology, Institute of Genetics & Developmental Biology, Chinese Academy of Sciences, Beijing, People's Republic of China; [2]Department of Dermatology, Beijing Tongren Affiliated Hospital of Capital Medical University, Beijing, People's Republic of China

Oculocutaneous albinism (OCA) is a heterogeneous and autosomal recessive disorder with hypopigmentation in eye, hair and skin color. Four genes, TYR, OCA2, TYRP1 and SLC45A2, have been identified as causative genes for non-syndromic OCA1-4 respectively. Additional unknown OCA genes may exist as at least 5% of OCA patients have not been characterized during mutational screening in several populations. We here used exome sequencing with a family-based recessive mutation model to determine that SLC24A5 is a previously unreported causative gene for non-syndromic OCA, which we designate as OCA6. Two deleterious mutations in this patient, c.591G>A and c.1361insT, were identified. We found apparent increase of immature melanosomes and less mature melanosomes in the patient's skin melanocytes. However, no defects in the platelet dense granules were observed, excluding it from typical Hermansky-Pudlak syndrome (HPS), a well-known syndromic OCA. Moreover, the SLC24A5 protein was reduced in steady-state levels in mouse HPS mutants with deficiencies in BLOC-1 and BLOC-2, suggesting that the HPS proteins may be involved in the melanosomal targeting of SLC24A5. It remains unknown why loss of SLC24A5 causes the defects in pigment production. We will discuss the possible pathogenesis of OCA6.

IL64

Skin bleaching in sub Saharan African people: the difficult choice between beauty and health

F. Ly

University Cheikh Anta Diop of Dakar, Dakar, Senegal

The pursuit of beauty is a major issue for people in the age of globalization, whatever the phototype: UV cabins or skin bleaching. This latter is a public health concern in sub Saharan Africa, about 67% of women in some area use high potent corticosteroids, hydroquinone, acid fruits and mercury in cosmetic purpose. Recently, a widespread use of systemic skin whitening agent is reported: GSH; it's used alone or in various combinations, both as oral and intravenous formulations. All these products had an effect on melanogenesis by inhibition or destruction of melanin. This practice is associated with medical complications such as cutaneous infections (cellulitis, mycoses, and scabies), squamous cell carcinoma and dyschromia.

Despite being aware of the medical complication, the majority of women keep on this practice for 10 yr and even more. The main reason advanced by the women is aesthetic and for women who refuse skin bleaching sometimes ambivalence is reflected. The majority of women consider that men prefer women with clear complexion and competition appears between women especially in a context of polygamy.

We develop medical, socio-anthropological aspects of Skin bleaching in sub-Saharan Africa through quantitative and qualitative studies conducted in Senegal to illustrate the difficult choice between beauty and Health.

 © 2014 John Wiley & Sons A/S. Published by John Wiley & Sons Ltd

IL65

Environmental factor and pigmentation

A. Morita

Departments of Geriatric and Environmental Dermatology, Nagoya City University Graduate School of Medical Sciences, Nagoya, Japan

Large epidemiologic studies suggest a clear link between environmental factors and extrinsic skin aging. Not only ultraviolet but also other environmental factors including tobacco smoke and air pollution have been identified. We compared aging between Asian and Caucasian women and observed that Asian women develop pigment spots much earlier than age-matched Caucasian (German) women, whereas the opposite is true for the formation of coarse wrinkles. Based on the epidemiologic study, we found an association of smoking with an increase of spots on the back of hands in German women and wrinkles on upper lip in Japanese women.

Several studies have demonstrated that tobacco smoke causes premature skin aging (e.g., smoker's face). Tobacco smoke causes pigmentation of the face, oral mucosa, and dorsal hands. To our knowledge, however, no studies have examined the mechanisms underlying smoking-induced pigmentation. We investigated whether tobacco smoke enhances skin pigmentation by culturing normal human epidermal melanocytes with tobacco smoke extract. When cultured with tobacco extract, melanocytes grew quite large and produced more melanin. Culturing the cells with tobacco smoke extract combined with UVB irradiation significantly increased microphthalmia-associated transcription factor (MITF) expression in a dose-dependent manner. Immunocytochemical studies revealed that these activated melanocytes actively expressed aryl hydrocarbon receptors (AhR) around the nuclear membrane. The tobacco smoke-induced MITF activation was inhibited by RNA silencing of AhR. This study provides the first evidence that tobacco smoke enhances sun-induced pigmentation by AhR-mediated mechanisms inside melanocytes. Previous reports revealed that the role of melanin and melanocytes is not only photo protection, but also the rejection of free radicals. This reaction of melanocytes to tobacco smoke might be an important biologic defense mechanism against environmental insults.

IL66

New aspects of melasma and treatment

K. C. Park, H. R.Choi, J. I. Na

Department of Dermatology, Bundang, Seoul National University College of Medicine, Seoul, Republic of Korea

Melasma is difficult to treat and is often refractory to multiple treatment modalities. Histologically, melasma is characterized by increased melanin in epidermis but melasma skin often has more free melanin and melanophages in the dermis. Increased production and accumulation of melanin is a hallmark of melasma and a huge number of agents is claimed to be effective for reducing pigmentation. Recently, pendulous melanocytes have been reported to be a characteristic feature of melasma which is related with reduced expression of type IV collagen along the basement membrane. These findings suggest that melasma is not only a disease of melanocytes but also a disease of surrounding environment. Now, different approaches are used to inhibit melanogenesis by their working mechanisms. Most whitening or lightening agents act specifically to reduce the function of tyrosinase by means of several mechanisms: (i) before melanin synthesis (interfering its transcription and/or glycosylation), (ii) during melanin synthesis (tyrosinase inhibition, peroxidase inhibition, and reduction of by-products), and (iii) after melanin synthesis (tyrosinase degradation, inhibition of melanosome transfer, skin turnover acceleration). In addition, basement membrane will provide niches for the epidermal stem cells and microenvironment changes will affect epidermal stem cells. Thus, strategies for manipulating cell to extracellular matrix interactions hold promise in preventing aging effects or controlling skin disease such as melasma. In this presentation, new therapeutic agents or approaches will be discussed in terms of signaling pathway of melanogenesis and epidermal stem cells.

IL67

The mechanisms of skin lightening ingredients for cosmetics

K. Sato

Shiseido Co., Ltd., Shiseido, Japan

Skin lightening products which give lighter skin tone or an even skin complexion are very popular especially in Asian countries. In Japan, skin lightening products are categorized in quasi-drugs which are defined as cosmetic products that have moderate pharmacological activity and contain active ingredients approved by Japanese Ministry of Health, Labor and Welfare. Now approximately twenty ingredients are used as active skin lightening ingredients in Japanese cosmetic market.

The most common target of skin lightening ingredients is tyrosinase in melanocytes. Arbutin is known as a tyrosinase inhibitor. It inhibits tyrosinase by competing with L-tyrosine, which is a substrate of tyrosinase, at the active biding site of tyrosinase. Kojic acid, which is also known as a tyrosinase inhibitor, chelates copper needed for tyrosinase to exert its activity. Magnolignan controls tyrosinase activity inside melanocytes in a different way. It inhibits the maturation of tyrosinase and reduces the amount of tyrosinase in melanosomes.

Tranexamic acid and chamomilla extract are the different type of skin lightening ingredients. Melanogenesis in melanocytes is much influenced by mediators released from keratinocytes. The ingredients reduce melanogenesis by controlling keratinocyte-derived mediators to stimulate melanogenesis.

Usually active ingredients are small molecules. But we can find that a lot of plant extract are used in skin lightening products. Though almost all of them are not approved as active ingredients, they show wide variety of mechanisms. To overview the skin lightening ingredients used in the cosmetic field, some plant extracts having unique mechanisms are picked up and are introduced.

IL68

Skin lightening agents: the evidence and my experience

E. Handog

Asian Hospital and Medical Center, Makati, Philippines

Coming from a tropical country where brown skin is actually beautiful, majority of my Filipina patients prefer to have fairer skin. For a multitude of reasons, one's interest in commercial skin lightening products becomes a hype and therefore hope comes easily as these commodities proliferate in the market and drugstores. They come in the form of creams, pills, soaps or lotions, many of which are bought because of tri-media advertisement or high profile endorsements. Unfortunately,

© 2014 John Wiley & Sons A/S. Published by John Wiley & Sons Ltd

some of these skin lightening agents don't really work or don't have sufficient studies to guarantee efficacy and safety.
Standard skin lightening agents include hydroquinone, kojic, licorice, azelaic and while alternative treatment for hyperpigmentation includes multiple herbal compounds, the discussion on combination therapy will be relevant.
A few of the trials on depigmenting agents done at the Research Institute of Tropical Medicine will be shared.
A. TOPICALS, to include tetrahydrocurcumin, indomethacin
B. Skin lightening agents thru enhanced delivery system, to include tranexamic acid solution via iontophoresis technology, glutathione via iontophoresis andglutathione via mesotherapy
C. ORAL, to include Procyanidin and Glutathione

Abstracts of Sponsored Breakfast Symposia Breakfast Symposium by Avène/Color Play Enterprise Pte Ltd

Safety assessment and protective efficacy of a large spectral range sunscreen formulation against DNA damage and oxidative stress induced by solar exposure

H. Duplan
Pierre Fabre Dermo-cosmétique(PFDC), France

Sunlight radiation is a human carcinogen contributing to photoaging and aggravating hyperpigmentation disorders (lentigo, melasma). The harmful effects of solar radiation are mainly related to direct DNA damage and oxidative stress induced by the ultraviolets (*UV*) and more recently the visible and infrared (*VIS/IR*) radiations. Sunscreens are becoming widely used to prevent sunburn by using broad spectrum UVB+A filters and antioxidants to limit free radical production. The aim of our research was to develop a large spectral range UVB+A and VIS/IR photoprotective system (*a patented association of 4 filters and tocopheryl glucoside (TG), a vitamin E precursor*), and to assess its safety and efficacy against solar light.
The $SPF50^+$ sunscreen was developed by Pierre Fabre and validated according to the COLIPA and International SPF test methods. Bioavailability of the 4 sunfilters was assayed by HPLC analysis in intact and damaged/irradiated skin explants, and showed no filter presence in receptor fluid after a 24 hr application.
Solar-simulated radiation (*SSR*) induced significant tissue damage and apoptosis 24hr after acute irradiation of a reconstructed human epidermis (sunburn cell, caspase-3). Liquid chromatography/mass spectrometry revealed the generation of cyclobutane pyrimidine dimers after irradiation (*TT>TC>CT>CC*). None of the above cellular responses was observed in non irradiated epidermis. The topical application of sunscreen 1 hour before SSR exposure afforded an almost complete photoprotection.
The VIS/IR irradiation of ex vivo porcine ear skin showed a significant radical formation in this solar wavelength range by electron paramagnetic resonance spectroscopy (*EPR technique*). Sunscreen revealed a radical protection factor (*RPF*) value of 45×10^{14} radicals/mg and had the highest antioxidant properties as compared to placebo without photoprotective system and TG or formula with TG only (*RPF 10 and 15* $\times$ 10^{14}*, respectively*). It also presented high scattering properties and decreased the radical production in the skin after VIS/IR irradiation (*41% versus 23% for placebo*).
Thus, the new sunscreen formulation provides skin protection against DNA damage and oxidative stress covering the entire solar spectrum from UV to IR, suggesting that it might be used to prevent hyperpigmentation disorders.

Abstracts of Sponsored Lunch Symposia Laboratoires Genevrier

Surgical techniques in vitiligo: introduction

N.van Geel, K. Ezzedine
Ghent University, Ghent, Belgium

Surgical methods can be a therapeutic option in patients with stable vitiligo/leukoderma (e.g. segmental vitiligo, piebaldism). These surgical techniques are based on a common principle: to transplant autologous melanocytes from a normal pigmented donor skin to depigmented area. Many surgical techniques have been devised over the years and can broadly be divided into tissue and cellular grafting. Tissue grafts include full-thickness punch grafts, thin dermo-epidermal grafts and suction blister grafts. With these tissue grafts a limited surface area can be treated per treatment session. Cellular grafts include cultured pure melanocytes suspension and non-cultured epidermal cellular suspensions (mixture of melanocytes and keratinocytes). These epidermal cells can also be co-cultured to epithelial sheet grafts. The major advantage of these suspension and culturing techniques is that they permit treatment of affected skin manifold larger than the donor area. An additional advantage of the non cultured epidermal cellular grafting technique is that the procedure can be completed in several hours on an outpatient basis.

Micro holes for delivering melanocytes in vitiligo skin. An ex vivo approach as compared to laser-assisted dermabrasion

C. Regazzetti[1], T. Passeron[1,2]
[1]INSERM U1065, team 12, C3M; [2]Department of Dermatology, University hospital of Nice, Nice, France

Epidermal suspension is an effective approach for treating segmental vitiligo, or stable and localized vitiligo. Laser-assisted dermabrasion is considered as the gold standard procedure for preparing the graft recipient site. However, the technique is relatively painful and requires technical equipment and a specific training for the procedure that strongly limit the use of this approach among dermatologists and plastic surgeons. Micro holes performed using micro needles, or erbium or CO2 fractional ablative lasers have been shown to enhance the penetration of topical agents, such as methyl ALA used in photodynamic therapy. These techniques are easy to perform (especially micro needles) and are painless when the targeted depth is the epidermis or the superficial dermis. Using these techniques for delivering melanocytes in vitiligo skin could be an attractive approach.
In a first step we have made GFP normal human melanocytes (GFP-NHM). Then, on skin taken from abdominoplasty, we performed micro holes using micro needles, ablative fractional erbium laser and ablative fractional CO2 laser. In the same time, we also performed erbium laser-assisted dermabrasion. GFP-NHM were delivered onto the skin samples and cultured ex-vivo for 4 days. Skin samples were then fixed and histological analysis was performed to compare the relative efficacy of the techniques by assessing the presence of GFP-

© 2014 John Wiley & Sons A/S. Published by John Wiley & Sons Ltd

NHM on the basal membrane. The results and the perspectives will be discussed during the symposium.

Viticell®, single-use device for preparation of an autologous cell suspension dedicated to skin repigmentation

Viticell® is a single-use, stand-alone device for performing a cell suspension. Viticell® is a kit comprised of reagents and attached instruments: enzymatic solutions, sterile application solutions and devices. The kit also includes a portable instant heat pack containing an aqueous solution saturated with sodium acetate, which provides heat through crystallisation.
Starting with a thin-skin biopsy, this device enables, through disintegration of the graft, a cell suspension made up of a mixed population, primarily of keratinocyte basal cells but also containing Langerhans cells, melanocytes and fibroblasts.
Viticell® is a device that enables cell suspension and, in particular, it can be dedicated to skin repigmentation, for example as part of non-progressive vitiligo or hypochromatic post-traumatic scars.
For more information visit: www.viticell.com

A multicenter double-blind placebo-controlled trial of non-cultured epidermal cellular grafting versus hyaluronic acid for repigmenting stable leukoderma (vitiligo and piebaldism)

Several studies have suggested that non cultured epidermal cells may be efficient to treat depigmented lesions in leukoderma. However, most of these studies are a limited sample size or the absence of double blind.
The purpose of this study is to assess the efficacy of autologous epidermal cells suspension grafting using a newly developed device named VITICELL® compared to placebo in segmental, non-segmental vitiligo and piebaldism.
The secondary objective is to evaluate safety of VITICELL® and global satisfaction of patient and investigator.
ClinicalTrials.gov Identifier: NCT02156427

Lunch Symposium by L'Oréal

Skin color diversity: from clinical assessment towards melanin chemical phenotype

S. Del Bino[1], S. Ito[2], K. Wakamatsu[2], F. Bernerd[1]
[1]L'Oréal Research & Innovation, Aulnay-sous-Bois, France; [2]Fujita Health University School of Health Sciences, Toyoake, Japan

Constitutive pigmentation of the skin is largely responsible for the individual sensitivity to UV exposure. It can be objectively classified through the Individual Typology Angle (°ITA) based upon colorimetric parameters of the L*a*b* system. We assessed the physiological relevance of this classification on 3500 women living in different geographical areas which gave an overall overview of the wide diversity of constitutive pigmentation especially among Asian countries. Experimentally, we showed a correlation between ITA values and biological markers of UV-induced erythema. However, quantitative and qualitative analysis of melanin in human skin is lacking. In particular the relative proportion between brown-black eumelanin and yellow-red pheomelanin in a large number of skins with broad distribution of pigmentation intensities has not been described. We therefore analyzed melanin in 35 skin samples of different constitutive pigmentations corresponding to the six ITA skin color groups by i) quantification by image analysis of Fontana Masson stained sections ii) quantitative analysis by spectrophotometry following solubilization with Soluene-350, iii) quantitative and qualitative analysis of specific degradation products using high-performance liquid chromatography (HPLC). These included pyrrole-2,3,5-tricarboxylic acid (PTCA) and thiazole-2,4,5-tricarboxylic acid (TTCA) obtained after hydrogen peroxide oxidation of DHICA moiety of eumelanin and benzothiazole-type pheomelanin respectively, or 4-amino-3-hydroxyphenylalanine (4-AHP) obtained after hydrolysis of benzothiazine-type pheomelanin with hydroiodic acid. Results from image analysis showed significant correlations between ITA and melanin amount in whole epidermis or within the epidermal basal layer. Chemical analysis led to significant correlations between ITA and total melanin amount assessed either by spectrophotometry or HPLC. Furthermore a good correlation between ITA and PTCA, a specific product of eumelanin degradation was found. Altogether, our results contribute to a better characterization of the 'chemical' melanin phenotype in skin of different constitutive pigmentations. They also confirm the low content of photoprotective eumelanin among lighter skin types, thereby explaining their higher sensitivity towards UV rays.

Common facial cosmetic pigmentary disorders in Asians

C. L. Goh
National Skin Center, Singapore City, Singapore

There are many causes of pigmentation disorders. Some are acquired and some are congenital/familial, some are due to epidermal, some dermal and others mixed melanin or other pigment deposition. Often a single patient has multiple pigmentation disorders. Recognition of type disorders and the location of pigmentation are important as treatment regimens and prognosis of different pigmentation disorders varies. Recent advances in lasers and light devices have enabled some of these pigmentary disorders to be removed very effectively. However, many of these pigmentary disorders recur or are recalcitrant to laser and light treatment. Topical treatment remains the cornerstone for the prevention and maintenance treatment of most acquired cosmetic pigmentary disorders. These topical treatments include prescriptive skin whitening agents and cosmeceuticals.
There are three major causes of acquired cosmetic facial pigmentary disorders viz. lentigenes/ephilides, dermal melanocytosis (Hori's naevus) and melasma (superficial and deep). A patient can have a combination of two or more of the disorders. Lentigenes are readily removed by pigment lasers or IPL after single or few treatments, Hori naevi and naevus of Ota can be removed with long wavelength Q switch lasers but require multiple treatments. Melasma usually does not respond to any treatment and is best treated with sunlight avoidance, sunscreens and topical bleaching cream. Becker's naevus is unresponsive to treatment and café-au-lait respond transiently most of the time. Other pigmentary disorders seen in Asians include post inflammatory hyperpigmentation, lichen planus pigmentosus, pigmented contact dermatitis. Based on the predictable treatment response, patients with the various predominant skin pigmentation disorders can be advised on their prognosis.

© 2014 John Wiley & Sons A/S. Published by John Wiley & Sons Ltd

Actinic Lentigines in Japanese and European populations: new insights from genomic profiling

C. Duval[1], E. Warrick[1], S. Nouveau[1], C. M. Benoit-Jay[1], P. Bastien[1], S. Deret[2], A. Morita[3], O.de Lacharrière[1], F. Bernerd[1]
[1]L'Oréal R&I, Clichy; [2]Galderma R&D, Sophia-Antipolis, France; [3]Nagoya City University Graduate School of Medical Sciences, Nagoya, Japan

Age spots or Actinic Lentigines (AL), are hyperpigmented lesions associated with age and chronic sun exposure and therefore represent a hallmark of photoaged skin. Despite the high prevalence of pigmented spots in elderly people, especially in Asian populations where they develop earlier, the biological events underlying the development of these lesions remain ill-defined. In this study, we analyzed the morphology and gene expression profiles of AL from 12 Japanese women (aged 50–70, phototype III-IV) and compared data with that previously obtained from 15 European volunteers to better understand the pathophysiological mechanisms of AL in both populations. AL from the dorsal side of hands were selected through the same epiluminescence criteria (elongated honeycomb pattern) as in the European study. AL and adjacent non lesional (NL) skin biopsies were processed for histology, melanogenesis and transcriptomic analysis. Histological sections of Japanese AL versus NL skin revealed a drastic deformation of the dermal epidermal junction with deep epidermal invaginations. Fontana Masson staining confirmed a higher amount of melanin in the lesional skin and an accumulation of melanin in the depth of epidermal rete ridges. The whole genome transcriptomic study showed that 245 genes were differentially expressed in AL versus NL skin. Modulated genes were associated with the different skin compartments and involved in various biological processes, such as epidermal homeostasis, oxidative stress, extracellular matrix organization and inflammation. Strikingly, among the canonical genes known to be involved in melanogenesis, none was significantly modulated in AL compared to NL skin. Comparison of modulated genes in Japanese and European AL showed that most genes (> 80%) were modulated in the same way in both populations. We identified a strong signature of 138 genes that recapitulates the overall biological alterations of AL lesions, regardless of the studied population. We also selected a set of similarly modulated inflammation markers attesting that a proinflammatory microenvironment does exist in actinic lentigines.
Altogether, the results clearly showed that Japanese and European AL's alterations do not only concern melanocytes but more widely include disorganization of the whole skin structure and alteration of multiple biological functions, thus emphasizing the interest of a multi-targeted strategy for an efficient age spots treatment.

Lunch Symposium by Procter & Gamble

The role of TLRs-signaling in melanogenesis

F. Xiang[1], Q. Chen[1], X. Yan[2]
[1]Department of Dermatology, Huanshan Hospital, Fudan University, Shanghai, People's Republic of China; [2]P&G Innovation Godo Kaisha, Kobe, Japan

The hyperpigmented macules on facial skin was a big concern, especially for Asian women. Multiple causes were considered to be associated to hyperpigmented spots, however, their mechanism were not fully understood.
It was well established that keratinocytes play a crucial role in the regulation of skin pigmentation by producing Proopiomelanocortin (POMC) and other cytokines related to melanocytes proliferation and survival. It was also reported that human keratinocytes expressed a panel of Toll-like receptors (TLRs). Recent studies suggested that TLRs expressed by keratinocytes constitute part of the innate immune system of the skin and could have significant role in the pathogenesis of inflammatory skin disorders. The bacterial floras of human skin could produce TLRs agonists, and result in post-inflammatory hyperpigmentation. However, the roles of TLRs-signaling induced hyperpigmentation required further investigation.
We used cell wall components of P. acne, including peptidoglycans (PGN) and lipoteichoic acids (LTA) as TLR2 triggers in HaCaT cells. In addition, Lipopolysaccharide (LPS) was used as TLR4 agonist. The level of IL-8 and POMC was evaluated with or without those TLRs stimulants. In addition, we evaluated the impact of GFF (Galactomyces Ferment Filtrate) and Niasinimide (nicotinic acid amide) in the TLRs-signaling.
In conclusion, our results showed that TLRs-signaling potentially induce hyperpigmentation following skin inflammation. This indicated a novel cause of hyperpigmented spots, which was a highly concerned skin issue. Furthermore, our results showed that GFF and Niacinamide down-regulate TLRs-signaling; this indicated that those two materials possibly inhibited potential hyperpigmented spots.

Lunch Symposium by Roche

Transform the treatment of aBCC: the role of an oral Hedgehog pathway inhibitor in dermatology

S. M. Dinehart
Arkansas Skin Cancer Center, USA

Vismodegib is a novel oral Hedgehog pathway inhibitor that has recently been approved in the US and EU for the treatment of selected patients with advanced basal cell carcinoma.
Dr. Scott Dinehart is a renowned Mohs and Dermatologic surgeon who practices at the Arkansas Skin Cancer Center in Arkansas (US). Dr. Dinehart will deliver a talk during the lunch symposium at the International Pigment Cell Conference (IPCC) in Singapore on 5 September 2014.
Dr. Dinehart's talk will focus on the role of Vismodegib as an option in the treatment of advanced basal cell carcinoma. He will present clinical data that led to the approval of Vismodegib by the US FDA and will also share his clinical experience with this novel drug.
Vismodegib is currently not approved in Singapore.

Lunch Symposium by NeoAsia

A combined strategy to improve the efficacy of skin lightening therapy

E. Flori[1], E. Bastonini[1], S. Briganti[1], D. Kovacs[1], G. de Paoli Ambrosi[2]
[1]San Gallicano Dermatologic Institute IFO IRCCS, Rome, Italy; [2]GENERAL TOPICS s.r.l., San Felice del Benaco (BS), Italy

Increased production and accumulation of melanins characterize a large number of skin diseases, including acquired hyperpigmentation, such as melasma, postinflammatory

 © 2014 John Wiley & Sons A/S. Published by John Wiley & Sons Ltd

melanoderma and solar lentigo, with a considerable impact on the quality of life of affected individuals. Acquired hyperpigmentation is a complex process, involving the regulation of tyrosinase and related melanogenic enzymes, the transfer of melanosomes into recipient keratinocytes and the pigment processing and degradation. Many of the commonly used whitening agents act as competitive inhibitors of tyrosinase, which is the main enzyme involved in melanin synthesis. Due to the incomplete effectiveness and the possible side effects of high dosage of these compounds, recent strategies for the development of skin lightening products include the combination of ingredients acting upon different steps of the pigmentation pathway. An innovative formulation has been recently developed, acting by the specific inhibition of tyrosinase activity through three key steps.

The active ingredients of THIOSPOT® cream are: phenyl ethyl resorcinol, an inhibitor of tyrosinase catalytic activity; acetylglucosamine, which possesses a glycosidase activity of proteins; ethyl linoleate, which increases proteasomal degradation of proteins and promotes epidermal turn-over.

We evaluated the effect of the compounds on tyrosinase activity and expression as well as on the inhibition of melanin production in both murine and human melanocytes, exposed or not to alpha-Melanocyte Stimulating Hormone (alpha-MSH). The single compounds significantly reduced the amount of intra-cellular melanin and the expression and activity of tyrosinase by acting in different steps of its regulation. The combined treatment showed a greater inhibition of melanogenesis than single ingredients, indicating an additive effect. Importantly, the inhibition of pigmentation was significantly higher towards alpha-MSH induced melanogenesis. Clinical data confirmed the effectiveness of the formulation. In conclusion, the combination of the three ingredients contained in THIOSPOT® cream is able to significantly interfere with melanin synthesis by reducing the amount of tyrosinase, increasing its proteolysis and inhibiting its catalytic activity, supporting this formulation as an innovative and safe therapy for acquired hyperpigmentation.

New developments in photoprotection and prevention of photocarcinogenesis

C. L. Goh

National Skin Centre, Singapore City, Singapore

Photoprotection is essential to prevent the deleterious effects of ultraviolet (UV) light, including skin cancer, photoaging, hyperpigmentation, and immunosupression. The use of botanic supplements endowed with substantial antioxidant activities has generated wide interest to decrease the risk of skin disease induced by UV radiation-dependent oxidative stress. Oral sun screening agents can provide a degree of protection by blocking, at least partially, the abnormal generation of ROS that underlies most of these diseases. In this context, a hydrophilic extract obtained from the aerial parts of the fern Polypodium leucotomos (PL, Fernblock®) exhibits strong photoprotective properties, reducing UV-induced inflammation and pigmentation. We will discuss the evidence on the cellular and molecular mechanisms underlying its photoprotective effect. PL is a natural mixture of phytochemicals endowed with powerful antioxidant properties. Its short-term effects include inhibition of reactive oxygen species production induced by UV radiation, hyperpigmentation and related disorders such as melasma, DNA damage, and isomerization and decomposition of *trans*-urocanic acid, preservation of Langerhans cells population, prevention of UV-mediated apoptosis and necrosis, as well as degradative matrix remodeling, which is the main cause of photoaging. These short-term effects translate into long-term prevention of photoaging and photocarcinogenesis.

© 2014 John Wiley & Sons A/S. Published by John Wiley & Sons Ltd

Abstracts of Oral Communications CS01–CS91

Plenary Session: Melanocyte UV Response & DNA Repair

CS01

Exploring signalling pathways co-ordinating the melanocyte ultraviolet response: identification of a novel role for NR4A orphan nuclear receptors in cellular stress response

K. Yin[1], K. Jagirdar[2,3], A. Smith[1]

[1]School of Biomedical Sciences; [2]Institute for Molecular Bioscience, The University of Queensland; [3]Dermatology Research Centre, Princess Alexandra Hospital, Brisbane, Qld, Australia

Ultraviolet radiation (UVR) is the most common mutagen which melanocytes are exposed to. UVR causes a diverse range of DNA photolesions contributing to genomic instability and promotes melanoma and non-melanoma development. In melanocytes, a number of signalling pathways have evolved to counter the damaging effects of UVR exposure. Amongst them is the G-protein coupled receptor called melanocortin-1-receptor (MC1R) that signals via cAMP induction when stimulated by its ligand, α-MSH. The MC1R signalling pathway has been directly linked to melanogenesis, enhanced cytoprotection against UV damage and augmented DNA repair responses. We have previously identified the rapid and transient induction of the NR4A family of orphan nuclear receptors upon α-MSH stimulation in melanocytes. Using immunofluorescence imaging, we revealed that upon exposure to cellular stress, NR4A nuclear receptors are recruited to distinct nuclear foci by a mechanism requiring p38-mediated phosphorylation and poly (ADP-ribose) polymerase activity. Furthermore, NR4A2 foci co-localise and interact with proteins involved in the recognition and repair of DNA. NR4A2 thus appears to be crucial for the ability of the MC1R signalling pathway in enhancing DNA repair capacity following UVR. While the roles of NR4A1 and NR4A3 have not been characterised beyond the observation of foci, we hypothesise that NR4A3 maybe involved in RNA processing. Based on current data, we propose that NR4A nuclear receptors may represent different repair components contributing to genomic maintenance and cyto-protection following UVR exposure. Interestingly, a recent study has found that NR4A2 expression confers resistance to BRAF inhibitors. Accordingly, the NR4A nuclear receptors represent interesting drug candidates for addressing melanoma drug resistance and for chemo-prevention of UV induced carcinogenesis. Current efforts using targeted mutagenesis of the NR4A receptors are being employed to elucidate the role of these proteins in both UV cyto-protection and melanoma drug resistance.

CS02

Skin cancer chemoprevention strategy using melanocortin 1 receptor-selective small α-MSH analogs

V. Swope[1], R. Starner[1], L. Koikov[2], N. Hone[1], D. Mehta[2], A. L. Kadekaro[1], Z. Abdel-Malek[1]

[1]University of Cincinnati; [2]Dermatology, University of Cincinnati, Cincinnati, OH, USA

A paradigm-shifting discovery in our laboratory was that in addition to the well-known function of α-melanocyte stimulating hormone (α-MSH) in stimulating eumelanin synthesis, it counteracts the damaging effects of UV by enhancing repair of DNA damage and inhibiting oxidative stress in human melanocytes (HM). These effects are expected to restore genomic stability, and thereby prevent mutagenesis and malignant transformation to melanoma. These findings led us to design tetrapeptide and tripeptide analogs of α-MSH, and test their efficacy on cultured HM, human skin explants (HSE) and melanocyte-containing cultured skin substitutes (CSS). A major milestone was the design of tetra- and tripeptide α-MSH analogs that are highly selective for the melanocortin 1 receptor (MC1R), which circumvents the off-target effects resutling from activation of other MCRs. We tested these analogs, the tetrapeptides LK 184, the lead peptide, and LK 467, as well as the tripeptides LK 511, LK 513, and LK 514 on cultured HM. We found that LK 184 and LK 467 were equally potent in activating cAMP formation, the main second messenger of the MC1R, and in stimulating tyrosinase activity, and at least 100 fold more potent than α-MSH in both assays (having a minimal effective dose between 0.1 and 1 pM, compared to 0.1 nM for α-MSH). Both tetrapeptides at 1 nM had an equal effect on increasing repair of DNA photoproducts as 10 nM α-MSH. The tripeptides LK 511, LK 513, and LK 514 retained full melanotropic activity, but were 10 fold less potent that α-MSH in stimulating cAMP formation and tyrosinase activity. The tripeptides at 100 nM had a comparable effect as 10 nM α-MSH in increasing DNA repair. To validate the effects obtained using cultured HM, we tested the tetrapeptide LK 467 on HSE that was maintained in organotypic culture. Pretreatment of HSE with 1 nM LK 467 for 3 days prior to, and immediately after UV irradiation significantly reduced the induction of DNA photoproducts 2 h post UV, and enhanced the repair of DNA damage 48 h after irradiation in melanocytes and the entire epidermis. We also tested the effects of LK 184 on CSS that we engineered in our laboratory. The CSS can be maintained for longer time periods (weeks) than the HSE, which lose their viability within a few days. Treatment with 1 nM LK 184 for 3 days prior to, and 24 h post UV resulted in reduction of DNA photoproducts in melanocytes as well as in the epidermis. No significant change in melanin was observed in either HSE or CSS after this short term treatment with LK 467 or LK 184, respectively, as determined by Fontana-Masson stain. This suggests that reduction in UV-induced DNA damage was due to enhancement of DNA repair pathways rather than increased melanin. Treatment of CSS with 1 nM LK 184 for 10 days resulted in an increase in melanin. Currently, we are testing the effects of the tripeptides LK 513 and LK 514 on HSE and CSS. Collectively, these results strongly suggest that the tetrapeptides LK 184 and LK 467 are highly efficacious in protecting HM and the skin as an organ from the genotoxic effects of UV. We propose that these analogs can be utilized in a chemoprevention strategy against skin cancer, including melanoma, in high risk individuals, including those heterozygous for loss-of-function *MC1R* variants.

 © 2014 John Wiley & Sons A/S. Published by John Wiley & Sons Ltd

Concurrent session: Developmental Biology

CS03

Ets1 and SOX10 act synergistically during early melanocyte development

A. Saldana-Caboverde[1], D. E. Watkins-Chow[2], W. J. Pavan[2], L. Kos[1]

[1]Biological Sciences, Florida International University, Miami, FL; [2]National Human Genome Research Institute, National Institutes of Health, Bethesda, MD, USA

Melanocytes differentiate from the neural crest (NC), a pluripotent population of cells that delaminate from the dorsal aspect of the neural tube during embryonic development. Many of the genes required for the specification of melanocytes from the NC have been identified through the study of mouse pigmentation mutants. Mice carrying a deletion of the transcription factor *Ets1* exhibit hypopigmentation, hinting at a potential role for this transcription factor in melanocyte development. In the mouse embryo, *Ets1* is widely expressed in developing organs and tissues, including the NC, and is required for the endogenous expression of the melanocyte-specific transcription factor *SOX10* in the chick cranial NC. We aim to establish the temporal requirement and role of *Ets1* and to examine potential genetic interactions between *Ets1* and melanocyte-specific transcription factors, including *SOX10*. To this end, embryos from crosses between $Ets1^{+/-}$ and *Dct-LacZ* transgenic mice, where *LacZ* is expressed in melanocyte precursors (melanoblasts), were stained between embryonic days (E)10.75–15.5. $Ets1^{-/-}$ embryos have fewer melanoblasts compared to $Ets1^{+/-}$ and $Ets1^{+/+}$ littermates. Cell survival assays showed increased melanoblast cell death in E10.75 $Ets1^{-/-}$ embryos. Similarly, double-label immunofluorescence staining against SOX10 and cleaved Caspase-3 showed increased NC cell death in E10.25 $Ets1^{-/-}$ embryos. To establish whether *Ets1* interacts with *SOX10*, $Ets1^{+/-}$ mice were crossed to $SOX10^{+/LacZ}$ mice, in which *LacZ* is inserted into the *SOX10* locus, and the hypopigmentation phenotypes of the progeny were compared. The incidence of hypopigmentation in $Ets1^{+/-}$::$SOX10^{+/LacZ}$ mice was significantly higher than that of $Ets1^{+/-}$ and $SOX10^{+/LacZ}$ mice. Additionally, the areas of hypopigmentation of $Ets1^{+/-}$:: $SOX10^{+/LacZ}$ mice were significantly greater than the sum of the areas of hypopigmentation of $Ets1^{+/-}$ and $SOX10^{+/LacZ}$ mice, suggesting that a synergistic interaction exists between *Ets1* and *SOX10*. To characterize the nature of this interaction, the ability of Ets1 to activate the *SOX10* MCS4 enhancer, which was previously shown to direct *SOX10* expression in melanoblasts and melanocytes, was analyzed via Luciferase reporter assay. In the mouse melanoma cell line B16, Ets1 was able to activate the *SOX10* MCS4 enhancer, suggesting a possible role for *Ets1* in the regulation of *SOX10* expression. Additional evidence for this potential role, comes from an apparent decrease in *SOX10* expression, examined via LacZ staining, at E11.5 and E12.5 in 50% of $Ets1^{-/-}$ embryos compared to $Ets1^{+/+}$ littermates. Consistent with *Ets1* function in melanocyte development, whole exome sequence analysis of the spontaneous mouse mutant *Variable Spotting* at the NIH Sequencing Center identified a G-to-A nucleotide transition in exon 3 of the *Ets1* gene, which predicts a G102E missense mutation. We determined that this results in a hypomorphic mutation as in vitro Luciferase reporter assay demonstrated a 2-fold decrease in the activation of the SOX10 MCS4 enhancer compared to wild type *Ets1*. Together, our results suggest that *Ets1* is required for proper melanocyte development on or before E10.75 and regulates melanoblast survival at this stage. Furthermore, *Ets1* interacts synergistically with *SOX10*, and may be important in the regulation of *SOX10* expression.

CS04

Mit factors as master regulators of the V-type H+ Atpase: conservation of coordinate transcriptional regulation of subunit genes in fly and vertebrates

T. Zhang[1], Q. Zhou[1], M. H. Ogmundsdottir[2], R. Siddaway[3], M. Singh[3], S. W. Kong[4], C. R. Goding[3], L. Larue[5], A. Palsson[6], E. Steingrimsson[7], F. Pignoni[1]

[1]Department of Opthalmology, Biochemistry and Molecular Biology, Upstate Medical University, Syracuse, NY, USA; [2]Biochemistry and Molecular Biology, Faculty of Medicine, University of Iceland, Reykjavik, Iceland; [3]Ludwig Institute for Cancer Research, University of Oxford, Oxford, UK; [4]Informatics Program, Children's Hospital Boston; [5]Normal and Pathological Development of Melanocytes, Institut Curie, Paris, France; [6]Life and Environmental Sciences, University of Iceland; [7]Biochemistry and Molecular Biology, Faculty of Medicine, University of Iceland, Reykjavík, Iceland

The membrane-bound vacuolar-ATPase (V-ATPase) is a multi-subunit, ATP-dependent proton pump that is required for a wide variety of biological processes in almost every eukaryotic cell. For instance, it drives acidification of subcellular compartments and transport of metabolites across membranes. However, despite these and many other vital functions, it is still not understood how expression of the multiple genes that encode its many subunits is regulated. Here we show that the sole *Drosophila* member of the *MiT* family of transcription factors (which includes mammalian TFEB, TFE3, TFEC and MITF) coordinately regulates the expression of genes encoding its 15 subunits. Through the V-ATPase and other key genes, fly MiT controls the endosomal–autophagic–lysosomal system; whereas, through TORC1, the V-ATPase restricts nuclear entry of MiT. These results suggest that MiT, TORC1 and V-ATPase form a negative feedback loop that likely contributes to homeostasis under varying environmental conditions. Remarkably, the coordinated regulation of V-ATPase subunit genes by an MiT factor is conserved in the melanocyte lineage of mammals. This suggests that this enzyme complex is a major target of MITF in this cell type and may also be an important target in other cell types requiring MITF, including osteoclasts. We propose that the regulation of the V-ATPase by MiT factors is a fundamental function of an ancestral TOR/MiT/V-ATPase network centered around the single MiT gene in *Drosophila* but one or more family members in vertebrates, based on cell type.

CS05

Modelling the melanocyte gene regulatory network – quantitation, kit and manipulation

R. N. Kelsh[1], A. Lapedriza[1], H. Schwetlick[2]

[1]Biology & Biochemistry, University of Bath; [2]Mathematics, University of Bath, Bath, UK

Neural crest stem cells generate a wide variety of derivatives, including melanocytes; like all stem cell development, these differentiation events are controlled by an intricate gene regulatory network (GRN). As stem cells become specified to individual fates, and these differentiate, the GRN becomes reconfigured to different (transient or stable) states. We aim for a quantitative understanding of the GRN driving melanocyte differentiation in zebrafish embryos which would allow us to predict the phenotypic consequences of mutation of key components. We are building on

© 2014 John Wiley & Sons A/S. Published by John Wiley & Sons Ltd

an initial core GRN and its qualitative mathematical modelling (Greenhill et al., 2011 PLoS Genetics, 7, e1002265). We have now developed a simulation tool to aid in visualisation of the shifting flux through different parts of the GRN. We explore the possibility that Kit signalling contributes to stabilised differentiation of melanocytes, because this addresses a major feature proposed by our modelling. Surprisingly, we show that initial expression of *kit* is SOX10-dependent, but Mitfa-independent, although as expected maintenance of *kit* expression is Mitfa-dependent. We investigate Kit signalling-dependent feedback on Mitfa. However, quantitative effects are troublesome to judge by in situ hybridisation, so we have developed a method of estimating gene expression levels in single melanocytes using a combination of in situ hybridisation and quantitative RT-PCR. These quantitative data will then be used to develop a quantitative version of our mathematical model of the melanocyte core GRN. The identification and characterisation of a temperature sensitive *kit* allele by Rawls and Johnson (2003; Dev. Biol. 262, 152–161) allows precise control of the timing and levels of Kit activity in melanocytes. We will present data comparing the model's predicted changes in melanocyte gene expression when Kit signalling is inactivated at different stages in development with quantitative RT-PCR data on temperature-shifted *kit* mutant embryos in order to assess the extent to which the model satisfactorily predicts the behaviour of the GRN under *kit* inactivation scenarios.

CS06

Conditional deletion of kit induces white spotting phenotype through cell-autonomous requirement of kit signaling

H. Aoki, T. Kunisada

Tissue and Organ Development, Gifu University, Gifu, Japan

Kit signaling is required for the growth, differentiation, survival, migration or fate determination of melanocytes. Formation of a berry white spot in *W/+* results from loss of these functions by the reduced amount of Kit signaling. A fundamental question not to be answered yet is whether the white spotting is the direct and cell autonomous effect of Kit signaling or the effect by non-cell autonomous Kit signaling since Kit dependent neuronal, hematopoietic, mesenchymal cells may indirectly affect the melanocyte development via some growth factors they produced.

Here we tried to evaluate the possible non-cell autonomous effect of *Kit* mutation for the melanocyte development. Trans-membrane region of the *Kit* was flanked by loxP to excise the *Kit* floxed allele by Cre recombinase. To take advantage of melanocyte lineage specific induction of Cre, the *Kit*$^{floxed/+}$ mice were crossed with *Tyr-Cre* mice expressing Cre directed by the melanocyte lineage specific tyrosinase promoter/enhancer sequence. The resultant *Kit*$^{floxed/+}$; *Tyr-Cre+* mice generated the white spotting phenotype, indicating that our melanocyte lineage specific *Kit* conditional KO heterozygous mice are equivalent to the *Kit* deficient heterozygous mice such as *W/+* mutant. These results clearly indicate that melanocyte lineage restricted reduction of Kit signaling precisely controlled by the melanocyte specific induction of *Kit* loss of function mutation is just enough to induce white spotting phenotype.

To investigate whether the generated white spot is rescued by the overexpression of Kitl or not, we crossed *Kit*$^{floxed/+}$*; Tyr-Cre+* mice with the previously established mice generated by the construct containing *Kitl* cDNA connected with human cytokeratin 14 promoter. The resultant *Kit*$^{floxed/+}$*; Tyr-Cre+; hk14-Kitl+* mice showed similar coat pigmentation pattern with that of *Kit*$^{floxed/+}$*; Tyr-Cre+* mice, indicating that the increase of the environmental Kitl from the skin keratinocytes is not enough to recover the white spotting phenotype of *Kit*$^{floxed/+}$*; Tyr-Cre+* mice.

We previously clarified that the cutaneous melanocytes can be classified into two types, one is the epidermal melanocyte which is highly dependent on Kit signaling, and the other is the dermal melanocyte which is much more dependent on endothelin 3 (ET3) and hepatocyte growth factor (HGF) signalings. As the complete lack of Kit signaling leads to the loss of ET3 or HGF dependent dermal melanocytes, some amount of Kit signaling is also indispensable for the survival of dermal melanocytes. We tested the effect of ET3 and HGF under the melanocyte specific reduction of Kit signaling. Both *Kit*$^{floxed/+}$; *Tyr-Cre+; hk14-ET3+* and *Kit*$^{floxed/+}$; *Tyr-Cre+; hk14-HGF+* mice were found to form white spotting, indicating that the increase of the environmental ET3 or HGF from the skin keratinocytes is also not enough to recover the white spotting phenotype of *Kit*$^{floxed/+}$*; Tyr-Cre+* mice. Unexpectedly, these mice contained unpigmented inter-follicular skin. Considering the fact that dominant negative *Kit V620A* mutation (*hk14-ET3+;Kit*$^{V620A/+}$ *or hk14-HGF+;Kit* $^{V620A/+}$) results uniformly pigmented dermis, *Tyr-Cre* induced more stringent Kit signaling conditions reduce the survival or maintenance of ET3 or HGF dependent dermal melanocyte stem cells.

Our findings confirmed the previously not asked significance of melanocyte-autonomous requirement of Kit signaling in vivo and also highlighted the complex nature of Kit signaling activated by the environmental Kitl and interacting with other signaling molecules such as ET3 and HGF.

Concurrent session: Melanin Biophysics

CS07

Human red hair pheomelanin is a potent pro-oxidant mediating UV- independent mechanisms of oxidative stress in red hair phenotypes

A. Napolitano[1], L. Panzella[1], L. Leone[1], R. Micillo[1,2], G. Monfrecola[2]

[1]Department of Chemical Sciences, University of Naples Federico II; [2]Department of Clinical Medicine and Surgery, University of Naples Federico II, Naples, Italy

Traditionally, the positive correlation between red hair and melanoma has been attributed to both the poor antioxidant and photoprotective properties of pheomelanins compared to the dark eumelanins, and the capacity of pheomelanin to act as photosensitizer inducing generation of reactive oxygen species upon UV irradiation. Recently, the occurrence of UV-independent pathways of carcinogenesis was provided by a series of experiments showing that induction of an activating mutation of BRAF into red mice resulted in a high incidence of invasive melanomas in the absence of UV stimulation. In addition, the skin of pheomelanic mice contained higher levels of oxidative DNA and lipid damage with respect to albino-*Mc1r*$^{e/e}$ mice (Mitra *et al*, *Nature* 2012, *491*, 449–453). These data clearly showed that the pheomelanin pathway could mediate oxidative stress and melanomagenesis; however the mechanisms have not so far addressed at molecular level.

To get an insight into these processes we investigated the reactivity of pheomelanin pigment isolated from human red hair (RHP) and synthetic pheomelanins from cysteinyldopa (CD-Mel) toward critical cellular antioxidants such as GSH and NAD(P)H. Data showed a remarkable increase in the oxidation rate of both

 © 2014 John Wiley & Sons A/S. Published by John Wiley & Sons Ltd

compounds in the presence of RHP compared to the controls, whereas much less marked variations were noticed with the eumelanin extracted from black hair. HPLC analysis consistently indicated a rise in GSH disulfide (GSSG) levels with GSH decrease, confirming a redox reaction. Formation of $NAD(P)^+$ was similarly observed in the reaction mixture of NAD(P)H with RHP. In the absence of oxygen, GSH and NAD(P)H depletion was not observed while the presence of enzymes as superoxide dismutase and catalase did not modify the effect of pheomelanin suggesting a ROS independent mechanism. Similar effects were obtained with CD-Mel confirming that the prooxidant effects are due to the pheomelanic component. The mechanism of GSH oxidation by RHP was investigated by EPR spectroscopy.

Besides inducing antioxidant depletion, RHP, like CD-Mel, proved also capable of promoting autoxidation of melanin precursors, such as 5-*S*-cysteinyldopa and dopa, under conditions of exclusion of light, with formation of pheomelanin or eumelanin pigments. This effect is oxidative in character, since it depends on oxygen, revealing a remarkable prooxidant capacity of the pigment serving as a biocatalyst for non-enzymatic melanogenesis (Panzella *et al.*, *PCMR* 2014, *27*,244–252; Greco *et al. Chem Comm.* 2011,*47*, 10308–10310).

A mechanism has been proposed to account for the observed effects in which GSH, NAD(P)H and melanin precursors oxidation is not mediated by ROS, but is the result of a direct redox exchange with oxidized 1,4-benzothiazine units within the pigment scaffold. Re-oxidation of reduced pheomelanin by oxygen with formation of ROS would ensure shuttling of pheomelanin between the redox states.

Overall these results provide a unifying chemical framework into which to explain the mechanisms underlying the relationships between pheomelanin pigmentation and oxidative stress.

CS08

Tyrosinase-catalyzed oxidation of rhododendrol produces 2-methylchromane-6,7-dione, the ultimate toxic metabolite: implications for melanocyte toxicity

S. Ito[1], M. Ojika[2], T. Yamashita[3], K. Wakamatsu[1]

[1]Department of Chemistry, Fujita Health University School of Health Sciences, Toyoake; [2]Department of Applied Molecular Biosciences, Nagoya University Graduate School of Bioagricultural Sciences, Nagoya; [3]Department of Dermatology, Sapporo Medical University School of Medicine, Sapporo, Japan

RS-4-(4-Hydroxyphenyl)-2-butanol (rhododendrol or rhododenol, RD) had been used as a skin whitening agent until the report of RD-induced leukoderma in July, 2013. RD reduces production of melanin in the skin whose mechanism was believed to be a competitive inhibition of tyrosinase. However, its 4-alkylphenol structure suggests that RD can be a good substrate for tyrosinase and the position of the alcoholic hydroxy group renders a facile cyclization. To explore the mechanism of its melanocyte toxicity, we performed detailed analysis of tyrosinase-catalyzed oxidation of RD by spectrophotometry and HPLC. Oxidation of RD with mushroom tyrosinase rapidly produced RD-quinone which within several minutes was converted to 2-methylchromane-6,7-dione (RD-cyclic quinone) through an intramolecular addition of the hydroxy group. These quinones were identified as RD-catechol and RD-cyclic catechol after $NaBH_4$ reduction, respectively. RD-cyclic quinone was gradually transformed through addition of water molecule to RD-hydroxy-*p*-quinone. These quinones decayed gradually to form ill-characterized products, indicating a very complex metabolic pathway. Autoxidation of RD-cyclic catechol was found to produce superoxide radical. RD-quinone and RD-cyclic quinone effectively bound thiols such as cysteine and GSH; the thiol addition was confirmed as *N*-acetylcysteine adducts. These results suggest that the melanocyte toxicity of RD is caused by the tyrosinase-catalyzed oxidation through production of RD-cyclic quinone which depletes cytosolic GSH and then bind essential cellular proteins through SH group. Production of ROS through autoxidation of RD-cyclic catechol may augment the toxicity. The present biochemical study will facilitate in vitro and in vivo studies to confirm involvement of RD-cyclic quinone in the tyrosinase-dependent RD toxicity to melanocytes.

CS09

The modulatory effect of melanin on susceptibility of retinal pigment epithelial cells to photic stress

M. Zareba[1], J. M. Burke[1], C. M. Skumatz[1], M. Sarna[2], A. K. Pilat[2], T. J. Sarna[2]

[1]Ophthalmology, Medical College of Wisconsin, Milwaukee, WI, USA; [2]Biophysics, Jagiellonian University, Krakow, Poland

Although photoprotective and antioxidant action of melanin has been clearly demonstrated in model systems of different complexity, showing the protective effects of melanin against photic and oxidative stress in cells has presented an experimental challenge. Using sensitive dynamic analyses, we were able to observe that phagocytosed melanosomes protected ARPE-19 cells exposed to hydrogen peroxide by acting as an antioxidant, and that experimental photoaging diminished the stress resistance conferred by the pigment granules. The difficulty in demonstrating clear photoprotection by melanin in cellular systems may in part result from intrinsic photoreactivity of this pigment, which upon irradiation with UV or even short-wavelength visible light generates potentially cytotoxic superoxide anion and hydrogen peroxide. In this study, we analyzed the modulatory effects of phagocytosed melanin granules, in comparison with control black latex beads, on photic injury in primary cultures of human RPE cells and in ARPE-19 cells induced by the endogenous age pigment lipofuscin. Cell death during lethal irradiation with blue light was quantified by dynamic imaging of the onset of nuclear propidium iodide fluorescence. Sub-lethal effects of blue light irradiation were measured by real-time imaging of the motility of lipofuscin and melanosome granules within the same RPE cells. Sub-lethal effects of lipofuscin-dependent phototoxicity were also monitored by atomic force microscopy (AFM), working in either ac mode or force spectroscopy mode, and by standard immunofluorescence staining. Lethal doses of blue light irradiation induced cell death that occurred earlier in cells containing larger amount of lipofuscin granules. Phagocytosed melanosomes, unlike black latex beads, slowed down the onset of the photoinduced cell death in a number of the granules dependent manner. Sub-lethal irradiation of cells with blue light resulted in a greater impairment of movement of lipofuscin granules than that of melanosomes within the same individual human RPE cells. AFM examination of sub-lethally treated APRE-19 cells with blue light revealed that lipofuscin-mediated photic stress induced significant decrease in the formation of actin stress fibers. Such cells exhibited substantially modified nanomechanical properties. Fluorescence imaging confirmed that early changes associated with photic stress comprised disorganization of the architecture of the cell cytoskeleton. The results clearly demonstrate melanosome protection in human RPE cells against lipofuscin-dependent phototoxicity. Endogenous lipofuscin mediates photo-killing of RPE cells, but

© 2014 John Wiley & Sons A/S. Published by John Wiley & Sons Ltd

the effect is cell autonomous and modulated by coincident melanosome content. Importantly, the protection is not due to simple optical screening.

Supported by National Science Center (grant Maestro-2013/08/A/NZ1/00194) and by NEI-NIH (grants RO1EY019664 and P30EY01931).

CS10

Palmitoylation of tyrosinase regulates melanin synthesis through ubiquitination

Y. Niki[1], M. Fukata[2], Y. Fukata[2], S. Oku[2], C. Makino-Okamura[1], S. Takeuchi[1,3], K. Wakamatsu[4], S. Ito[4], C. Nishigori[3], L. Declercq[5], D. B. Yarosh[5], N. Saito[1]

[1]Kobe Skin Research Department, Kobe University, Kobe; [2]Division of Membrane Physiology, National Institute for Physiological Science, Okazaki; [3]Division of Dermatology Graduate School of Medicine, Kobe University, Kobe; [4]Department of Chemistry, Fujita Health University School of Health Sciences, Toyoake, Japan; [5]Basic Science Research, Estée Lauder Companies, Melville, NY, USA

S-palmitoylation (palmitoylation) is one of the most frequent posttranslational lipid modifications of proteins which consists in the attachment of a saturated C16 fatty acid chain to the specific cysteine residue through a thioester bond and significantly impacts protein function, trafficking and localization mainly via modulating membrane affinity and protein stability. Palmitoylation modifies numerous soluble and integral membrane proteins, including signaling proteins, enzymes, scaffolding proteins and ion channels. Recent large-scale profiling studies indicate that several hundreds of proteins may be palmitoylated in mammals. However the role of palmitoylation in the regulation of melanin synthesis little is known. Recently, we reported that tyrosinase, a key enzyme of melanin synthesis pathway, is modified by palmitoylation in normal human epidermal melanocyte (NHEM) and that Cys500 is responsible for the palmitoylation site. In mammalian cells, the palmitoylation is an enzymatic reaction and mediated by a family of cyltransferases containing DHHC (Asp-His-His-Cys) motif which there are 23 in humans. Systematic screening for palmitoyl transferase activity of the DHHC proteins using DHHCs and tyrosinase transfected HEK293 cells and siRNA for DHHCs using MM3KO human melanoma cells showed that DHHC-2, -3, -7 and -15 are involved in the tyrosinase palmitoylation. RT-PCR showed that these DHHCs are expressed in NHEM and human melanoma cells. We expressed DHHC-2, -3, -7 and -15 in NHEM as *N*-terminary-His6-Myc tagged proteins and investigated an intracellular localization of these DHHCs by immunocytochemistry. All four DHHCs were expressed in the Golgi apparatus, and DHHC-2 and -15 were expressed in plasma membrane. Only DHHC-2 was expressed in the melanosomes. And these DHHCs co-localized with tyrosinase at the Golgi apparatus and DHHC-2 in the melanosomes. Depletion of DHHC-2, -3, -7 and -15 decreased the ratio of palmitoylated tyrosinase but increased both the total tyrosinase level and melanin synthesis in MM3KO cells. A palmitoylation inhibitor, 2-bromopalmitate (2-BP), showed similar results in a dose-dependent manner in NHEM and MM3KO cells. Depletion of these DHHCs and 2-BP treatment did not affect the tyrosinase mRNA expression levels. The increase in melanin contents by 2-BP was also observed in a reconstructed human skin. Reversely, in these DHHCs over-expressed MM3KO cells, tyrosinase protein levels and melanin contents were decreased. These results indicated that the palmitoylation of tyrosinase leads to a down-regulation of melanin synthesis through the decrease of tyrosinase protein level at posttranslational event. 2-BP treatment suppressed the tyrosinase degradation in NHEM and MM3KO cells. Moreover, the degradation rate of mutated tyrosinase Cys500Ala which cannot be palmitoylated was significantly slower than that of wild type tyrosinase. The immunoprecipitation and Western blot analysis revealed that the non-palmitoylated C500A tyrosinase was not ubiquitinated and 2-BP also suppressed the ubiquitination of tyrosinase. This result raises the possibility that the palmitoylation of tyrosinase induces the ubiquitination and the palmitoylated tyrosinase is preferentially degraded via the ubiquitin proteasome system. Taken together, in this work, we demonstrated that the palmitoylation of tyrosinase may play an important role in the melanogenesis regulation via tyrosinase degradation through the ubiquitination.

Concurrent session: Vitiligo Research

CS11

A role for the unfolded protein response in the etiology of vitiligo

P. Manga, S. J. Orlow, S. Toosi

The Ronald O. Perelman Department of Dermatology, New York University School of Medicine, New York, NY, USA

Vitiligo is characterized by depigmented skin patches due to localized loss of melanocytes. The etiology of vitiligo is not fully understood, particularly the mechanisms that initially trigger depigmentation. Induction of oxidative stress is thought to be a common attribute of trigger events in vitiligo, while autoimmunity contributes to disease progression. In this study we sought to identify mechanisms that link disease triggers and the spread of lesions. A hallmark of melanocytes at the periphery of vitiligo lesions, even when cultured in vitro, is dilation of the endoplasmic reticulum (ER). We hypothesized that oxidative stress caused by trigger events extends to the ER where redox reactions that facilitate protein folding are disrupted and that the subsequent accumulation of misfolded peptides activates the unfolded protein response (UPR). The UPR initially signals a reduction in global protein synthesis, while promoting expression of folding chaperones in order to restore homeostasis. Sustained UPR activation has been implicated in several autoimmune disorders and may play a role in vitiligo. We used 4-tertiary butyl phenol (4-TBP) and monobenzyl ether of hydroquinone (MBEH), phenols known to trigger occupational vitiligo, to study pathways that contribute to melanocyte loss. Expression of key UPR components was increased following exposure of human melanocytes to the phenols. In addition, we observed UPR-mediated increases in pro-inflammatory interleukin-6 (IL6) and IL8, which have been shown to be increased in the skin of patients with vitiligo. We next investigated UPR-regulated pathways that may contribute to cytokine production and determined that cross-talk between the UPR and the nuclear factor-kappa B (NFkB) pathway contributes to the increase in expression of IL6 and IL8 following phenol exposure. Identification of the pathways that link exposure to vitiligo-inducing triggers and onset of autoimmunity may allow for the development of more effective therapies for this disfiguring disease.

 © 2014 John Wiley & Sons A/S. Published by John Wiley & Sons Ltd

CS12

A quantitative increase in regulatory T cells control development of vitiligo

S. Chatterjee[1], J. Eby[2], M. Soloshchenko[1], M. I. Nishimura[3], C. I. LePoole[2], S. Mehrotra[1]

[1]Surgery, Medical University of South Caroline, Charleston, SC; [2]Pathology, Loyola University; [3]Surgery, Loyola University, Chicago, IL, USA

T cell cytolytic activity targeting epidermal melanocyte is shown to cause progressive depigmentation and autoimmune vitiligo. Using the recently developed transgenic mice h3TA2 that carry T cell with a HLA-A2 restricted human tyrosinase reactive TCR and develop spontaneous vitiligo from an early age, we addressed the mechanism regulating autoimmune vitiligo. Depigmentation was significantly impaired only in IFN-γ knockout h3TA2 mice but not in TNF-α or perforin knockout h3TA2 mouse strains, confirming a central role for IFN-γ in vitiligo development. Additionally, the regulatory T cells (Treg) were relatively abundant in h3TA2-IFN-$\gamma^{-/-}$ mice, and depletion of Treg employing anti-CD25 antibody fully restored the depigmentation phenotype in h3TA2-IFN-$\gamma^{-/-}$ mice mediated in part through upregulation of pro-inflammatory cytokines as IL-17 and IL-22. Adoptively transferring purified Treg or using rapamycin evaluated further therapeutic potential of Treg abundance in preventing progressive depigmentation. Both adoptive transfer of Treg and rapamycin induced lasting remission of vitiligo in mice treated at the onset of disease, or in mice with established disease. We thus propose that promoting regulatory responses at the expense of effector responses can overcome immune activation and halt the progression of vitiligo. Overall this study thus leads us to conclude that reduced regulatory responses are pivotal to the development of vitiligo in disease-prone mice, and that a quantitative increase in the Treg population may be therapeutic for vitiligo patients with active disease.

CS13

The validity, reliability and acceptability of the SAVASI; a newly developed self-assessment score in vitiligo

L. Komen[1], G. E.van der Kraaij[1], W. J.van der Veen[1,2], M. A.de Rie[1,3], A. Wolkerstorfer[1]

[1]Department of Dermatology, Academic Medical Centre, Netherlands Institute for Pigment Disorders (SNIP), Amsterdam; [2]Department of Dermatology, MC Haaglanden, Den Haag; [3]Department of Dermatology, Academic Medical Centre, Amsterdam, The Netherlands

Vitiligo is a common skin disorder characterized by depigmented macules that can influence the quality of life of patients. It is important for patients to be involved in their disease progress and treatment, and patient reported outcomes are becoming more important. This has led to the development of self-assessment tools like the SA-PASI for psoriasis and the PO-SCORAD for atopic dermatitis. Until now, there is no vitiligo self-assessment tool available to assess the degree of depigmentation. Therefore, we developed the SAVASI, a self-assessment tool to assess the degree of depigmentation in vitiligo.
To assess the validity, reliability and acceptability of the SAVASI and to evaluate the correlation between the patient's ability to assess the extent of their vitiligo with the quality of life.
Until now, we included 55 vitiligo patients who visited the Netherlands Institute for Pigment Disorders (SNIP). To assess the validity, the correlation between the VASI the SAVASI was calculated. The VASI is a well described clinician reported outcome that is used to assess the degree of depigmentation. To assess the intra-rater reliability, the correlation between the first SAVASI and the SAVASI after 2 weeks was calculated. To assess the acceptability we assessed the time it took patients to complete the SAVASI, and the difficulty of the questionnaire was assessed by the patient on a five-point scale. The Skindex-29 was used to determine the quality of life. Correlations were calculated with the Intraclass Correlation Coefficient (ICC). ICCs of >0.70 were considered adequate.
Preliminary results show a high correlation between the VASI and SAVASI (ICC 0.97, 95% CI: 0.95 – 0.98) in 55 patients. An adequate intra-observer reliability for the SAVASI (ICC 0.73, 95% CI 0.42- 0.89) was found in 19 patients. Forty-six (83.4%) of the patients completed the questionnaire within 10 min and only four (7.3%) patients considered the SAVASI hard. We found no correlation between over-or underestimation of the SAVASI score and the Skindex-29 score (ICC = 0.05).
The SAVASI is a valid, reliable and acceptable self-assessment tool to measure the degree of depigmentation in vitiligo. With the SAVASI the degree of depigmentation can be reliably be assessed by the patients itself which can be useful in (epidemiological) studies. Furthermore this could contribute to the patient's disease insight and therapy loyalty.

Concurrent session: Regulation of Pigmentation

CS14

Regulation of pigmentation by Mir-330-5P targeting tyrosinase

F. Rambow[1], A. Bechadergue[1], G. Saintigny[2], F. Morizot[2], C. Mahe[2], L. Larue[1]

[1]Developmental Genetics of Melanocytes, Institut Curie, CNRS UMR3347, INSERM U1021, Orsay; [2]Parfums Beauté, CHANEL, Neuilly/Seine, France

Increasing evidence suggests an important role for miRNAs regulating melanogenesis. Many different proteins whose mRNAs are potentially targeted by miRNAs orchestrate this multistep process. MicroRNAs (miRNAs) are small noncoding RNAs that extensively regulate gene expression. In general, miRNAs inhibit protein synthesis by either repressing translation and/or destabilizing/degrading mRNAs by imperfect base pairing to the 'seed match' region (2-8nt sequence) in the 3′UTR, CDS or 5′UTR of the mRNA. Today, 1872 precursor miRNAs have been identified in humans, giving rise to 2578 mature miRNAs (mirbase release 20). Melanogenesis includes the synthesis of melanin and its distribution by melanosomes to surrounding keratinocytes. Recently, different miRNAs such as miR-145, miR-203, miR-125b, and miR-675-3p for humans, and miR-434-5p for mice have been shown to interfere in the melanogenic process. These observations identify miRNAs as promising future modalities to treat pigmentory disorders. However, different pigmentation genes are more suitable targets than others when thinking about a miRNA-based strategy. For example, the expression of MITF-M, which controls the expression of various genes involved in melanogenesis, is essential to melanocytes and hence should be avoided as a miRNA target. To this purpose, we decided to identify miRNAs that may act on pigmentation levels without affecting the survival/proliferation of melanocytes. As major miRNA target, we chose tyrosinase

© 2014 John Wiley & Sons A/S. Published by John Wiley & Sons Ltd

(TYR), which is the key and rate-limiting enzyme for melanin production. We applied an *in silico* sequence-based prediction approach (mirDIP) which aimed to identify miRNAs targeting most likely TYR but not MITF. From this approach, miR-330-5p turned out to be the most attractive candidate, as it targets TYR and not MITF. To this respect, we tested if miR-330-5p was able to target TYR and act on melanin levels. To do so, we overexpressed miR-330-5p using mimics in highly pigmented human melanocytes (MNT-1) and normal human epidermal melanocytes (NHEM) for a period of twelve days. Melanin levels of MNT-1 cells were significantly reduced upon treatment with miR-330-5p mimics as shown by spectrometry. Overexpression of miR-330-5p in MNT-1, 501mel and T1 cells resulted in a significant reduction of tyrosinase mRNA and protein levels, as predicted. The effect of miR-330-5p on TYR was also observed in NHEM. Using BrdU incorporation and Celltiterglo cell viability assays we showed that miR-330-5p mimic treatment did not influence neither proliferation nor cell survival. Mechanistically, miR-330-5p is predicted to bind to the position 68–75 of the TYR 3′UTR (seed match). To test this prediction, we cloned the predicted seed match region into a reporter vector and observed a significant reduction of Luciferase activity upon miR-330-5p overexpression. After mutating the seed match region, we could rescue the luciferase activity, suggesting that the predicted binding region is valid. In summary, we showed that miR-330-5p is a potent negative regulator of TYR in pigmented melanoma cells and normal melanocytes, and sustained overexpression of miR-330-5p induces depigmentation. Our data further imply that miR-330-5p acts specifically on TYR whereas related pigmentation genes such as TYRP1, DCT, MLANA and most importantly MITF stay intact. Cells of the melanocyte lineage treated with miR-330-5p mimics do not show any morphological changes or alterations in proliferation/survival, qualifying miR-330-5p as an ideal candidate to treat hyperpigmentation-related disorders.

CS15

Regulation of murine pigment production by Endothelin 3

J. Pino[1], S. Ito[2], K. Wakamatsu[2], L. Kos[1]

[1]Department of Biological Sciences, Florida International University, Miami, FL, USA; [2]Department of Chemistry, Fujita Health University School of Health Sciences, Toyoake, Japan

The production of pigment involves various signaling molecules that are essential for the proper development and function of melanocytes. α-Melanocyte Stimulating Hormone (α-MSH) regulates the switch between the production of both pigments, eumelanin (black/brown) and pheomelanin (yellow/red), while Endothelin 3 (Edn3) is required during melanocyte development. Doxycycline (dox) inducible transgenic mice that express Edn3 under the *Keratin 5* (*K5*) promoter showed hyperpigmentation of both the skin and coat. The goal of this study is to understand the role of *Edn3* in pigment production. We hypothesize that *Edn3* plays a role in the production of pigments by increasing melanin production. *Lethal yellow* mice (A^y) have a non-functional α-MSH signaling pathway leading to the production of yellow pigment in the hair. A^y mice carrying the *K5-Edn3* transgene were considerably darker than A^y littermates. In order to test if continuous *Edn3* transgenic expression is required to maintain a dark pigmentation phenotype in A^y mice, dox was administered to 1-day old pups, deactivating the transgene. After 6 weeks of dox treatment, the coat color of A^y;*K5-Edn3* mice became lighter and was similar to those of A^y littermates. In order to determine if transgenic *Edn3* expression has an effect on melanin concentrations in mouse hair, dorsal hairs were collected and melanin contents in the hair were determined by comparative analysis of melanin content using high performance liquid chromatography of A^y, A^y;*K5-Edn3* and wild type mice. Results showed that transgenic *Edn3* expression significantly increases both the amount of eumelanin ($P < 0.001$) and pheomelanin ($P = 0.005$) in A^y mice. To determine if the higher levels of melanin are due to an increased number of melanocytes in the hair follicles, dorsal mouse skins were collected and stained with TRP1 antibody. Preliminary results suggest that there is no difference in the number of hair follicle melanocytes between *K5-Edn3* and wild type littermates. These results indicate that the paracrine expression of *Edn3* from keratinocytes is capable of generating and maintaining a dark coat color in the absence of a functional α-MSH pathway. Different than α-MSH signaling, Edn3 does not appear to regulate a switch between eumelanin and pheomelanin production, but can up-regulate both simultaneously. The elucidation of the network of pathways involved will allow for the complete understanding of how color patterns in all organisms are formed and the possible development of more effective therapies for skin cancers, as well as, hypopigmentary and hyperpigmentary disorders in humans.

CS16

Ultraviolet B-induced up-regulation of Mcl-1L mediated by the Mek–ERK1/2-STAT3 signaling pathway protects melanocytes from apoptosis

T. Fukumoto[1], M. Oka[1], T. Iwasaki[2], M. Sakaguchi[1], Y. Fukami[2], C. Nishigori[1]

[1]Dermatology, Kobe University Graduate School of Medicine; [2]Research Center for Environmental Genomics, Organization of Advanced Science and Technology, Kobe University, Kobe, Japan

The role and regulation of myeloid cell leukemia-1 (Mcl-1) in ultraviolet (UV) B-induced apoptosis in melanocytes were examined. Melanocytes expressed two Mcl-1 isoforms, Mcl-1L and Mcl-1S, and signal transducer and activator of transcription 3 (STAT3) phosphorylated at Ser727 (pS-STAT3) but not STAT3 phosphorylated at Tyr705 (pY-STAT3). UVB irradiation at a dose of 10 mJ/cm^2 induced long-term up-regulation of Mcl-1L protein, which was detected as early as 24 h and lasted by 72 h after irradiation, and did not induce apoptosis in the cells. The expression of Mcl-1S and pY-STAT3 was not affected by UVB irradiation. UVB irradiation induced an increase in Mcl-1L mRNA, extracellular signal-regulated kinase (ERK) 1/2 phosphorylation, Ser727 phosphorylation on STAT3, and nuclear translocation of STAT3. All of these responses were inhibited by ERK1/2 kinase (MEK) inhibitor U0126, concomitant with a decrease in UVB-induced up-regulation of Mcl-1L protein. Furthermore, U0126 induced apoptosis in melanocytes irradiated with UVB 24 h after irradiation. Electrophoretic mobility shift assay using WM39 melanoma cells, which express pS-STAT3 but not pY-STAT3, revealed that pS-STAT3 binds to a putative binding site in the promoter region of the *Mcl-1* gene. These results demonstrate that UVB-induced up-regulation of Mcl-1L protein is mediated by the MEK–ERK1/2 signaling pathway with pS-STAT3 as a transcription factor and has a protective role in UVB-induced apoptosis in melanocytes. We have previously suggested that pS-STAT3, but not pY-STAT3, plays a crucial role in melanocytes. Our present study clearly shows that pS-STAT3 has its own physiological role in melanocytes as a transcription factor of Mcl-1.

 © 2014 John Wiley & Sons A/S. Published by John Wiley & Sons Ltd

Concurrent session: Melanosome Biogenesis & Transfer

CS17

Mechanism of the melanosome transport inhibition induced by a constitutive active RAB27a mutant in melanocytes: development of a novel melanosome-targeting tag and its applications

M. Ishida, S. P. Arai, N. Ohbayashi, M. Fukuda

Laboratory of Membrane Trafficking Mechanisms, Department of Developmental Biology and Neurosciences, Tohoku University, Sendai, Japan

Small GTPase Rab proteins are universal regulators of intracellular vesicle transport in all eukaryotes. RAB27a, whose functional defects are known to cause human Griscelli syndrome type 2, is a crucial component of actin-dependent melanosome transport in melanocytes. Although constitutive active mutants of Rab proteins are generally thought to promote specific vesicle transport events, a constitutive active RAB27a(Q78L) mutant has been shown to inhibit melanosome transport and to induce perinuclear aggregation of melanosomes; however, the molecular mechanism by which RAB27a(Q78L) inhibits melanosome transport remained unclear. In this study, we attempted to elucidate why RAB27a(Q78L) causes perinuclear melanosome aggregation at the molecular level. We demonstrated that RAB27a(Q78L) is not able to be present on melanosomes and that its inhibitory effect on melanosome transport is completely dependent on its binding to the RAB27a effector Slac2-a/melanophilin, indicating that RAB27a(Q78L) traps Slac2-a in the cytosol of melanocytes. When we forcibly targeted RAB27a(Q78L) to mature melanosomes by using a novel melanosome-targeting (MST) tag that we developed here, the MST-RAB27a(Q78L) fusion protein functioned the same as wild-type RAB27a (i.e. it localized on melanosomes without inducing perinuclear melanosome aggregation and restored normal peripheral melanosome distribution in RAB27a-deficient melanocytes). Our findings indicate that the GTPase cycle of RAB27a is necessary for its melanosome localization, but is not necessary for melanosome transport (Ishida, M. et al., *J. Biol. Chem.* in press). Furthermore, we will introduce several examples of applications of MST-tag for analysis of the melanosome transport machinery.

CS18

TRIF and MAVS pathway is essential to induce RAB27a and melanosome transportation by TLR3 agonist poly(I:C) in human epidermal melanocytes

S. Koike, K. Yamasaki, T. Yamauchi, M. Inoue, K. Tsuchiyama, S. Aiba

Department of Dermatology, Tohoku University Graduate School of Medicine, Miyagi, Japan

Innate immune stimuli such as microbe molecules restlessly influence epidermis where human melanocytes reside. We have examined how innate immunity affects pigmentation and observed that TLR3 agonist poly(I:C) increased melanin release from melanocytes. We also revealed poly(I:C) increased mRNA and protein expression and accumulation of RAB27a, which facilitate melanosome transportation to cell membrane. SiRNA for RAB27a (siRAB27a) abrogated melanosome transfer to neighboring keratinocytes by poly(I:C).

Since MITF is known to increase RAB27a transcription and poly(I:C) increased MITF mRNA about 2-fold, we examined if MITF is involved in RAB27a induction by poly(I:C). We treated melanocytes with siRNA for MITF (siMITF) and then stimulated melanocytes with poly(I:C). Pretreatment of siMITF did not suppress RAB27a increase, the perimembranous localization of Gp100 and RAB27a, and extracellular melanin release by poly(I:C). Thus poly(I:C) induces RAB27a and melanosome transportation independent of MITF function.

To examine further how poly(I:C) induces RAB27a expression and melanosome transportation, we focused the downstream pathways of TLR3, TRIF pathway and MAVS pathway. TRIF is involved in induction of IL-1, IL-6 and TNF-α by TLR3 recognition of viral RNA, and MAVS, the membrane protein which localizes on mitochondrial outer membrane, also activated by virus RNA and induces type I interferon which leads antiviral action. Knockdown of TRIF as well as MAVS suppress RAB27a induction and melanosome transport to neighboring keratinocytes by poly(I:C) stimulation. These indicated that TLR3 agonist poly(I:C) activates melanosome transport by increase RAB27a expression through TRIF and MAVS pathway but MITF has little affect on RAB27a induction by TLR3 activation. TLR3 has unique cascades in melanocyte to enhance melanocyte transportation to keratinocytes.

CS19

BLOC-1 controls the formation of recycling endosomes required for melanosome biogenesis

C. Delevoye[1], F. Gilles-Marsens[1], M. Dennis[2], M. S. Marks[2], G. Raposo[1]

[1]CNRS-UMR144, Institut Curie, Paris, France; [2]Department of Pathology & Laboratory Medicine, Children's Hospital of Philadelphia and University of Pennsylvania, PA, USA

Specialized cell types, such as skin melanocytes, exploit endosomal trafficking pathways to deliver cargoes to Lysosome-Related Organelles (LROs), but how endosomes are specified for this function is not known. Melanosomes are LRO generated by pigment cells in which the melanin pigment is synthesized and stored.

Genetic diseases affecting LRO biogenesis and function, such as the Hermansky-Pudlak Syndrome (HPS), have highlighted several components required for the proper trafficking of melanosomal proteins from endosomes to melanosomes and for skin pigmentation. Among them, the Biogenesis of LRO Complex-1 (BLOC-1) plays a critical role.

We have demonstrated that the kinesin microtubule motor KIF13A creates recycling endosomal subdomains required for cargo delivery to maturing melanosomes (Delevoye et al, 2009; Delevoye et al, 2014). Our biochemical and light and electron microscopic studies now highlight that BLOC-1 and KIF13A function within the same endosome-melanosome trafficking pathway. BLOC-1 modulates locally the dynamic of the actin cytoskeleton at the endosomal level and cooperates functionally with KIF13A to generate recycling endosomes required for melanosome maturation and melanocyte pigmentation.

All together, our results show that BLOC-1 coordinates the actin- and microtubule-associated machineries to couple the endosomal sorting of melanogenic cargoes to the formation and trafficking of endocytic carriers towards melanosomes.

Delevoye et al., AP-1 and KIF13A coordinate endosomal sorting and positioning during melanosome biogenesis. J. Cell Biol (2009). 187, 247–264.

Delevoye et al., Recycling Endosome Tubule Morphogenesis from Sorting Endosomes Requires the Kinesin Motor KIF13A, Cell Reports (2014), http://dx.doi.org/10.1016/j.celrep.2014.01.002

© 2014 John Wiley & Sons A/S. Published by John Wiley & Sons Ltd

CS20

Cooperation of endothelin-1 signaling with melanosomes plays a role in developing and/or maintaining hyperpigmentation

A. Hachiya[1], D. Murase[2], R. Hicks[2], S. Moriwaki[1], T. Hase[1]

[1]Biological Science Laboratories, Kao Corporation, Haga-gun, Japan; [2]Biological Science Americas Laboratories, Kao Corporation, Cincinnati, OH, USA

The increased synthesis and deposition of melanin characterizes skin hyperpigmentation which can cause significant psychosocial and psychological distress. Although some cytokine-receptor signaling cascades have been shown to be involved in the formation and/or maintenance of ultraviolet B (UVB)-induced skin hyperpigmentation as well as hyperpigmented spots such as lentigo senilis, it has never been clearly addressed which signaling cascades substantially contribute to those processes. Given that our continuous studies using more than 30 samples of hyperpigmentation, including lentigo senilis as well as unevenly pigmented skin, demonstrated the consistent augmentation of endothelin-1 and endothelin B receptor signaling in those conditions, the potential functions of endothelin-1 signaling in regulating melanin synthesis and accumulation in their development and/or maintenance were characterized. In agreement with previous reports, endothelin-1 significantly induced melanogenesis followed by an increase in melanosome transfer to keratinocytes. Consistently, the expression of RAB27a, which plays a critical role in melanosome transfer in melanocytes, was also stimulated in the presence of endothelin-1 and endothelin-1 expression in keratinocytes was conversely increased following treatment with isolated melanosomes. Furthermore, consistent with the previous proposal that the formation of autophagosomes rather than melanosomes is stimulated according to conditions of stress, starvation or defective melanosome production, endothelin-1 induced a remarkably augmented expression of Pmel17, a marker of the initiation of the early stage of melanosome formation, implying the counteraction against autophagy-targeting melanosome degradation in melanocytes. Taken together, our data suggest for the first time that endothelin-1 plays a substantial role in the development and maintenance of skin hyperpigmentation in reciprocal cooperation with the increased production of melanosomes.

Concurrent session: Repigmenting Vitiligo: Of Art & Science

CS21

How to define repigmentation/maintenance of repigmentation in vitiligo; VGICC consensus position

K. Ezzedine[1], V. Eleftheriadou[2], J. Seneschal[1], M. Picardo[3], A. Taieb[1], On behalf of the Vitiligo Global Issues Consensus Conference

[1]National Reference Center for Rare Skin Disorders and Deprtment of Dermatology and Pediatric Dermatology, CHU Bordeaux, Bordeaux, France; [2]Centre of Evidence Based Dermatology, University of Nottingham, Nottingham, UK; [3]San Gallicano institute, San Gallicano Institute, Roma, Italy

To achieve international consensus over the core outcome domains to be captured in future vitiligo trials, the Vitiligo Global Issues Consensus Conference group has recently set-up a three stage, web-based, international Delphi (e-Delphi) exercise involving dermatologists and researchers with interest in vitiligo, patients with vitiligo (and their carers), representatives of regulatory agencies, and journal editors. In completion of the consensus exercise, three domains were deemed essential: repigmentation, side effects and harms of treatment, maintenance of gained repigmentation. Five domains were deemed recommended: cosmetic acceptability of the results, quality of life, cessation of spreading of vitiligo, tolerability/burden of treatment.

To prepare a position or consensus paper on behalf of the VGICC delineating the major identified outcome items ‘repigmentation/ maintenance of repigmentation’.

A questionnaire was sent to the seven regional working groups that includes the following questions.

1. How will you define ‘repigmentation’ in Vitiligo. The following item are listed and photograph illustrations for each type are needed. With this regard, participants may send their photographs.
2. What are in your opinion the set of minimal tools for the assessment of ‘repigmentation’.
3. What are the relevant endpoints for assessing extent and speed of repigmentation for RCTs
4. How will you define ‘maintenance of gained repigmentation’ in vitiligo
5. What are in your opinion the best qualitative and quantitative methods to assess ‘maintenance of gained repigmentation’

The group discussed each of these five items to reach consensus before the Denver meeting that has been held in March 2014.

A total of 45 participants responded to the questionnaire.

The items for patterns of repigmentation were commented in terms of pathophysiology and prognosis i.e. durability/ maintenance of gained pigmentation. The proposal of a ‘mixed pattern’ was agreed upon. The need of an illustrative atlas with photos before and after treatment to illustrate the defined repigmentation patterns was highlighted.

For question 2, the major point was the need to separate systemic intervention vs local intervention. It was decided to send an additional question to the group for exploring systemic intervention and overall cutaneous assessment, since most of the discussion focused on the assessment of target lesions.

For question 3, the first debated problem was the distinction between acral and non acral lesions. Another general issue discussed was the need to include stabilized patients versus the selection of active/progressive disease in studies with systemic intervention.

For question 4, the majority opts for up to 20% of loss of gained pigmentation over 1 yr (from 0 to 10–20%) as a definition of success. However, considering the cost of long-term observation in RCTs, the majority was in favour to limit at 6 month the period of observation and to maintain the same definition of success (up to 20% of loss of gained pigmentation).

For question 5, the addition of patient-oriented outcomes (PROs) should be considered. The localisation of relapses and the subtype of pattern of repigmentation vs persistence of repigmentation need to be analysed.

The VGICC group agreed to draft a manuscript that should be validated at the next IPCC in Singapore after the final round of discussion.

© 2014 John Wiley & Sons A/S. Published by John Wiley & Sons Ltd

CS22

Mobilization of melanocyte populations from stem cells in the hair follicle by ultraviolet light treatment of human vitiligo

D. Norris[1], N. Goldstein[1], M. Koster[1], L. Hoaglin[1], S. Robinson[2], W. Robinson[2], D. Roop[1], S. Birlea[1]

[1]Department of Dermatology, University of Colorado; [2]Division of Oncology, University of Colorado, Colorado, CO, USA

In human vitiligo, the autoimmune destruction of pigment cells in interfollicular epidermis (IE) produces white spots that can be repigmented by melanocyte precursors in the hair follicles, following stimulation with ultraviolet (UV) light. We have found that this repigmentation process provides a powerful model to study the emergence of the entire melanocyte lineage in adult human skin from precursor cells in the bulge and infundibulum. We examined by immunofluorescence the distribution of melanocyte markers (c-KIT, DCT, PAX3 and TYR) coupled with markers of proliferation (KI-67) and migration [melanoma cell adhesion molecule (MCAM)] in the hair follicles and IE, using untreated and narrow band UVB (NBUVB)-treated human vitiligo skin. We have found that the human hair follicle in untreated vitiligo contains melanocyte stem cells (DCT+/c-KIT−/TYR−) and melanoblasts (DCT+/c-KIT+/TYR−) in the bulge, but rare melanoblasts or melanocytes in the regions proximal to the bulge and infundibulum. During NBUVB-induced repigmentation, we observed expansion of DCT+/c-KIT+ melanoblasts in the bulge and infundibulum, as well as the emergence of differentiated melanocytes (DCT+/c-KIT+/TYR+) in large numbers in the infundibulum and IE. A majority of these TYR+ cells were potentially migratory (MCAM+) and a subset (about one tenth), were proliferative (KI-67+). We also identified a subpopulation of DCT-/c-KIT+/TYR− precursors in the infundibulum and IE which could represent a secondary melanocyte germ, in addition to the primary melanocyte germ in the bulge. There were also small subpopulations of migrating and proliferating undifferentiated melanocytes in the bulge, and infundibulum, which may be migrating and proliferating cells emanating from the stem cells in the bulge, that repopulate the infundibulum and IE. This model will facilitate better characterization of the subpopulations mobilized by UV light in vitiligo and may lead to better approaches to induce repigmentation.

CS23

Proactive treatment of non-segmental vitiligo with 0.1% tacrolimus ointment: double blind placebo controlled study

M. Cavalié[1], K. Ezzedine[2], E. Fontas[3], H. Montaudié[1], E. Castela[1], P. Bahadoran[1], J.-P. Lacour[1], T. Passeron[1,4]

[1]Department of Dermatology, University Hospital of Nice, Nice; [2]Department of Dermatology, University Hospital of Bordeaux, Bordeaux; [3]Department of Research and Innovation, University hospital of Nice, Nice; [4]INSERM U1065, team 12, C3M, Nice, France

Although vitiligo does not lead to severe physical illness, it can be a psychologically devastating disease, especially in darker skinned individuals and children. At the moment, treatment for vitiligo has mainly aimed to induce repigmentation. However after repigmentation, relapses occur in more than 40% of cases. To date no treatment is available for preventing these relapses contrarily to other diseases such as atopic dermatitis that have been shown to benefit from such an approach.

The objective of this study was to assess the efficacy of a twice weekly application of 0.1% tacrolimus ointment to prevent depigmentation in patients who achieved a complete or almost complete repigmentation after a treatment.

We conducted a double blind placebo controlled study in the departments of dermatology of Nice and Bordeaux University hospitals from December 2011 to October 2013. Patients with non-segmental vitiligo who achieved rates of repigmentation ≥75% after any type of treatments were proposed to participate in the study. After central randomization, patients were instructed to apply 0.1% tacrolimus ointment or placebo ointment, twice a week for 6 months on the area that had previously repigmented. For each patient, 1 to 4 lesions were treated. The main outome was the absence/presence of depigmentation at 6 months in the treated lesion. The evaluation was performed on standardized pictures by two independent physicians blinded to the treatment received.

Thirty five patients with 67 lesions were included. In per protocol analysis, 46% of lesions showed some depigmentation in the placebo group versus 9% in the tacrolimus group (P = 0.001%). In the intention-to-treat analysis (using the *maximum bias* bound approach), 46% of lesions showed some depigmentation in the placebo group versus 17% in tacrolimus group (P = 0.03%). Both treatments were well tolerated.

These results show that 0.1% tacrolimus ointment, used as a proactive treatment twice a week, significantly reduces the relapse in previously repigmented vitiligo lesions. This argues for a new approach in vitiligo management with a very practical impact in the management of the disease.

Twice weekly applications of 0.1% tacrolimus ointment is the first treatment for preventing relapses in vitiligo patients and remain easy to use in daily practice.

Plenary Session: Genetics of Pigmentation

CS24

Identifying genes That interact with UV to influence nevi and freckles

N. Box[1], N. Asdigian[2], A. Baron[3], J. Aalborg[2], V. Gonzalez[1], J. Dunn[1], R. Dellavalle[1], T. Terzian[1], M. Berwick[4], S. Mokrohisky[1], J. Morelli[1], L. Crane[2]

[1]Dermatology, University of Colorado Denver; [2]Preventive Medicine and Biometrics, University of Colorado Denver; [3]Biostatistics and Informatics, University of Colorado Denver, Aurora, CO; [4]Division of Epidemiology, University of New Mexico, Albuquerque, NM, USA

Ultraviolet radiation (UVR) in sunlight and genetic and phenotypic risk factors are strongly correlated with melanoma incidence. Nevi and freckles are two important melanoma risk phenotypes that are influenced by UV exposure. However, the putative interactions between sun exposure and genetic make-up in relation to these two melanoma risk phenotypes have not been elucidated. To examine these gene-environment interactions, we performed a first-of-its-kind longitudinal study of nevus and freckle development in a cohort of 1200 children, monitored from ages 6 to 16. These children had different sun exposure behaviors and were classified according to their pigmentation, nevus and melanoma risk genotypes at 15 major loci including *IRF4*, *MTAP*, *PLA2G6*, *OCA2*, *TYR*, *TYRP1*, *MC1R*, *ASIP* and *SLC45A2*. Using linear mixed analysis methods, we showed that all measures of sun exposure (chronic, sunburns and waterside vacations) interacted to predict total nevus counts. Moreover, we

© 2014 John Wiley & Sons A/S. Published by John Wiley & Sons Ltd

showed that waterside vacations and sunburns interacted with SNPs in some of these genes to increase total nevus counts, numbers of nevi ≥2 mm, and facial freckling scores. We demonstrate that nevus and freckle genes may be categorized into those that interact with UV and those that do not. Our findings emphasize the importance of implementing sun-protective behavior to at childhood regardless of genetic make-up; while children with particular genetic variants may need to be more specifically targeted for preventive measures to counteract their inherent risk of melanoma. Moreover, we demonstrate, for the first time, the power of the longitudinal studies as a tool to uncover gene-environment interactions as determinants of cancer risk.

CS25

Searching for new markers associated with eye colour by sequencing the human OCA2-HERC2 region

J. D. Andersen[1], C. Børsting[1], M. M. Andersen[2], N. Morling[1]

[1]Section of Forensic Genetics, Department of Forensic Medicine, University of Copenhagen, Copenhagen; [2]Department of Mathematical Sciences, Aalborg, Denmark

The human blue and brown eye colours are mainly explained by the single nucleotide polymorphim (SNP) rs12913832 that is located in the RLD2 (*HERC2*) gene that overlaps with the promotor region of gene. It was shown that the rs12913832 region functions as an enhancer regulating the oculocutaneous albinism type II (*OCA2*) transcription (Visser et al. 2012). The genotype rs12913832 GG predicts blue/light eye colour while rs12913832 GA/AA predicts brown/dark eye colour. However, a dominant-recessive gene model is too simple and several examples of eye colours that do not fit with the expected eye colour based on the rs12913832 genotype have been observed. Other SNPs located in the *OCA2-HERC2* region, including rs1800407 (missense mutation), were suggested to further modify the eye colours. A better understanding of the genetics underlying eye colours is important for several reasons. For example, prediction of physical characteristics including eye colours can be an important investigative tool for police investigators in criminal cases or disaster victim identification cases. A better understanding of the genetics underlying the different shades of eye colours may also sheet light to pigmentary gene regulation including *OCA2* regulation.

In this study, we sequenced a 500 kb region on chromosome 15 encompassing *OCA2* and *HERC2* in individuals with eye colours that did not match the expected eye colour based on the rs12913832 genotype. The individuals included subjects with light eye colours and the genotype rs12913832 GA/AA and subjects with dark eye colours and the genotype rs12913832 GG. The sequencing were carried out on a Illumina platform using the Haloplex Target Enrichment technology (Agilent Technologies). Combinations of observed variations in the *OCA2-HERC2* region were compared to the various shades of eye colours by means of the pixel index of the eye (PIE-score) (Andersen et al. 2013).

Plenary Session: Colours of Life

CS26

A horse of a different color: the DUN locus reveals a novel mechanism underlying camouflage pigmentation

K. McGowan[1,2], F. Imsland[3], C. Henegar[2], S. Fard[4], C.-J. Rubin[5], D. Schwochow[6], E. Sundström[5], G. Barsh[1,2], L. Andersson[5,6]

[1]Genetics, Stanford University, Stanford, CA; [2]HudsonAlpha Genomics Institute, Huntsville, AL, USA; [3]Department of Medical Biochemistry and Microbiology, Uppsala University; [4]Department of Neuroscience, Uppsala University; [5]Medical Biochemistry and Microbiology, Uppsala University; [6]Animal Breeding and Genetics, Uppsala University, Uppsala, Sweden

Agouti banding is a prototype for mammalian hair color pattern, in which pigment type varies as a function of hair length. During the course of studying the wild type color of horses, Dun, we have discovered a new aspect of hair color pattern that varies along the radial rather than the longitudinal axis. The Dun phenotype—pale body hair, a dark dorsal stripe, and zebra-like striping most commonly seen on the distal extremities —is characteristic of Wild Asses, Quaggas and the Prezwalski's horse. Dun is important for camouflage but a darker derivative phenotype, non-Dun, has been selected for during horse domestication, and behaves as a single gene that is recessive to Dun. Histological studies reveal that the dilute color on the flank of Dun horses actually reflects asymmetric deposition of pigment granules in a semicircular pattern with the 'outer' half of the hair pigmented; this asymmetry is lost in non-Dun horses. Positional cloning using Dun and non-Dun horses from a large number of breeds identified the causative mutation in a gene not previously associated with pigmentation. The gene is expressed in a unique population of cells in the hair follicle germinal matrix that is situated asymmetrically, as well as in the inner root sheath. RT-PCR, RNA-Seq, and immunohistochemistry studies reveal that in animals bearing the derivative allele (*non-Dun*), expression is extinguished specifically in the germinal matrix cells. Additional studies using markers for melanocytes, melanogenesis and melanoblast migration and survival point to a new and intriguing pathway for hair follicle pigment patterning, and establish a model that enlightens our understanding of coat color patterning in mammals.

Concurrent session: Melanocyte Stem Cells

CS27

Induction of Melanocytes From Multilineage-Differentiating Stress-Enduring (Muse) cells derived from human adipose tissue

T. Yamauchi[1], K. Yamasaki[1], K. Tsuchiyama[1], S. Koike[1], M. Dezawa[2], S. Aiba[1]

[1]Dermatology, Tohoku University Graduate School of Medicine; [2]Stem Cell Biology and Histology, Tohoku University Graduate School of Medicine, Miyagi, Japan

Multilineage-differentiating stress-enduring (Muse) cell is a multipotent somatic stem cell existing mesenchymal tissues such as dermis. Muse cells have unique characters; (i) having low or no telomerase activity, (ii) having pluripotency to differentiate into representative cells of all three germ layers, (iii) not developing tumors, (iv) exhibiting self-renewal ability, (v) expressing unique glycosphingolipid, stage specific embryonic antigen 3 (SSEA3). In terms of isolating directly from living

 © 2014 John Wiley & Sons A/S. Published by John Wiley & Sons Ltd

tissues, Muse cells are distinct from iPS cells and embryonic stem cells (ES cells), which require complicated manipulations. We have isolated SSEA3-positive Muse cells from cultured human fibroblasts and generated melanocytes by stimulating Muse cells with 10 factors including Wnt3a (JID 2013). Muse cell-derived melanocytes (Muse-MC) expressed melanocyte markers MITF, tyrosinase and gp100 and showed tyrosinase activity. Using skin reconstitute culture model, we confirmed that Muse-MC localized at the basal layer and expressed MITF and tyrosinase. Thus we succeeded to differentiate Muse cells into functional melanocytes.

We further aimed to clinical application and examined if Muse cells can be isolated from autologous tissues and differentiate into melanocytes. We collected human adipose-derived stem cells (hASC) from human adipose tissues obtained from surgical specimens. We identified 2.6 $\pm$ 0.28% of SSEA3-positive cells in hASC (hASC-Muse). hASC-Muse expressed alkaline phosphatase, smooth muscle actin, neurofilament, α-fetoprotein, indicating hASC-Muse possessed pluripotency. Using 10 factors for 6 weeks, hASC-Muse, but not from non-Muse hASC cells, induced melanocyte-relating molecules such as tyrosinase de novo and tyrosinase activity. Using skin reconstitute culture model, we confirmed hASC-Muse-derived melanocytes (hASC-Muse-MC) migrate into the basal layer of reconstituted epidermis. hASC-Muse-MC have lower telomerase activity than human primary melanocytes. These showed that we could generate functional melanocytes from human tissue-derived hASC-Muse without any external gene transduction. Now we are improving our procedures to make hASC-Muse-MC produce melanine efficiently. Our preliminary data suggested that hASC-Muse-MC showed greater L-DOPA activity and melanin contents when hASC-Muse-MC cultured in conditional medium containing bovine pituitary extract (BPE) or α-MSH. However, melanocyte induction was inhibited when hASC-Muse was cultured with 10 factor and α-MSH. Thus proper sequential stimulation of Muse cells and Muse-MC is essential to induce melanocytes and to enhance melanin production after melanocyte differentiation. Our methods will provide more insights for the process of melanocyte differentiation and migration.

CS28

Phenotype-based gene function assessment using a melanocyte-specific transgenesis approach

M. Osawa, M. Matsumoto, D. Lee

Graduate School of Medicine, Gifu University, Gifu, Japan

Stem cells hold great promise to revolutionalize regenerative medicine. To realize safe and effective stem cell-based therapies, it is essential to understand the basic mechanism of stem cell regulation. However, despite extensive progress in stem cell research over the past decade, molecular understanding of stem cell regulations is still challenging due to technical difficulties in studying stem cell biology. The melanocyte stem cell (MSC) offers an advantageous model for studying molecular basis of stem cell regulation through molecular genetics approaches, as genetic alterations involved in the MSC regulation results in a readily identifiable pigmentary phenotype in animals. Traditionally, such gene alterations have been achieved by spontaneous or induced mutagenesis or gene engineering in the mouse. However, despite a number of recent methodological advances, the generation of genetically altered mice is still laborious and time-consuming, which largely hampers in vivo gene function assignment. Here, we have developed a melanocyte-specific transgenesis system, by which rapid gene functional assessment is allowed in the mouse. To establish this system, we have combined the following three technological features: (i) derivation of an ES cell line from *Tyr-Cre* transgenic mice, enabling Cre-regulatable melanocyte-specific genetic modification and resulting phenotype-based assessment mice; (ii) application of the 'ES-mouse' generation methodology, by which mice fully derived from genetically modified ES cells are generated in the F0 generation, permitting immediate assessment of the pigmentary phenotype resulting from the genetic alteration; (iii) use of recombinase-mediated cassette exchange (RMCE)-based and Cas9/CRISPR-based approaches in the ES cells to allow stable and melanocyte-specific gene manipulation in genetically modified mice. A proof-of-concept study confirmed the broad applicability of this approach to define key molecules involved in melanocyte-related diverse biological phenomena, such as hair graying and hypopigmentation. Hence, these results clearly indicate the robustness of the transgenesis system in delineating gene function in the melanocyte lineage in vivo. Overall, our study highlights the usefulness of the transgenesis system for understanding the molecular basis of melanocyte-related diverse biological phenomena, including stem cell regulation.

CS29

Dual mechanisms combine to mediate regeneration of zebrafish melanocytes following injury

C. Ceol, S. Iyengar

Program in Molecular Medicine, University of Massachusetts Medical School, Worcester, MA, USA

Melanocytes, which can be lost during hair graying, injury and disease-related depigmentation, are replenished in mammals by resident stem cells. To gain insight into melanocyte regeneration we set out to identify whether melanocyte stem cells are present in adult zebrafish and how such cells might reconstitute the pigment pattern following injury. Using a targeted cell ablation approach we determined that *mitfa* is expressed not only in differentiated melanocytes but also in the cells that mediate melanocyte regeneration. When *mitfa*-positive cells are selectively ablated no melanocyte regeneration occurs. However, when ablation is performed in a *p53*-deficient background, melanocyte regeneration occurs, suggesting that death of the cells that mediate regeneration is dependent on *p53*. We then used *mitfa*-positivity to perform lineage-tracing experiments and assay whether unpigmented *mitfa*-expressing cells have stem cell properties. During regeneration, *mitfa*-positive cells can divide asymmetrically with one daughter cell differentiating and the other daughter remaining uncommitted; these are melanocyte stem cell divisions. In addition, some *mitfa*-positive cells directly differentiate during regeneration. Taken together, these data indicate that multiple mechanisms are used to re-establish pigmentation following injury and enable regeneration following subsequent rounds of ablation. We have used reporter assays and drug studies to assess whether pathways important for melanocyte development are also involved in regeneration. We found that Wnt signaling gets turned on during melanocyte regeneration and that Wnt inhibition after ablation of differentiated melanocytes delays regeneration. These studies have established a system by which regeneration can be traced with single-cell resolution and perturbations to regeneration analyzed in exquisite detail.

© 2014 John Wiley & Sons A/S. Published by John Wiley & Sons Ltd

CS30

To investigate the role of BAF60A in melanocyte differentiation

S. Aras, I.de la Serna

Biochemistry and Cancer Biology, The University of Toledo, Toledo, OH, USA

Transcriptional activation of melanocyte specific pigmentation genes involves a crosstalk between tissue specific transcriptional activators and SWI/SNF chromatin remodeling complexes which induce chromatin modifications permissive for transcription. SWI/SNF enzymes are multi-subunit chromatin remodeling complexes composed of an ATPase subunit which is BRG1 or BRM as well as 8–11 associated factors (BAFs). The BAF60 variants are important subunits of both the BRG1 and BRM complexes. The three mutually exclusive BAF60 variants, BAF60A, BAF60B, and BAF60C, are thought to mediate direct interactions with gene specific transcription factors. Melanocyte differentiation is an important process in maintaining melanocyte biology and the role of variant BAF60A in this process has not yet been investigated. In this study, we used an immortalized melanoblast cell line Melb-a which were induced to differentiate into melanocytes with alpha-MSH to investigate the role of the BAF60A in the activation of MITF target genes which regulate melanocyte differentiation and pigmentation. Our results indicate that differentiation of melanoblasts with alpha MSH led to an increase in the expression of MITF target genes leading to melanin synthesis. However, this increase in the expression was abrogated significantly both at RNA and Protein levels upon siRNA – mediated downregulation of BAF60A variant. Furthermore, Chromatin Immunoprecipitation (ChIP) Assays showed an increase in the enrichment of BAF60A on the promoters of MITF target genes upon alpha-MSH treatment suggesting its role in melanocyte differentiation. Moreover, BAF60A also co-immunoprecipitated with MITF, suggesting a physical interaction between them. Thus, based on these observations, we hypothesize that BAF60A variant mediates SWI/SNF interactions with MITF to activate melanocyte specific pigmentation genes. Abnormalities in melanocyte differentiation increase the risk of various pigmentary disorders as well as melanoma. Understanding the molecular mechanisms behind regulation of melanocyte differentiation would provide us novel therapeutic targets for treatment of these disorders.

Concurrent session: Metabolic and Intracellular signaling

CS31

Mir-211 functions as a metabolic switch in human melanoma cells

J. Mazar[1], A. D. Richardson[2], F. Qi[1], B. Lee[1], J. Marchica[1], A. Duran[2], Y. Tao[2], S. S. Govindarajan[1], J. Shelley[1], L. M. Brill[2], J.-L. Li[1], X. Han[1], R. J. Perera[1]

[1]Sanford-Burnham Medical Research Institute, Orlando, FL; [2]Sanford-Burnham Medical Research Institute, La Jolla, CA, USA

A hallmark of cancer cells is their ability to re-route major pathways of energy provision and consumption to support the energy demands associated with growth and survival. This is an emerging area of intense research, although much remains to be discovered. Melanoma is the leading cause of skin cancer deaths in the United States. Because melanoma is resistant to many existing therapies, there is an urgent need to better understand the gene- and protein-regulatory pathways that contribute to melanomagenesis and metastatic potential. Metabolic rewiring is known to occur in melanoma, but this has not been systematically characterized until recently.

In a search for critical microRNAs (miRNAs) that drive the development and progression of melanoma, we and others have identified miR-211 as the most differentially expressed miRNA in human melanomas when compared to those expressed in normal melanocytes. The expression of miR-211 is significantly lower in non-pigmented melanoma cells and in clinical melanoma samples than in normal melanocytes, and forced re-expression of miR-211 inhibited the growth and invasion of melanoma cells suggesting that miR-211 may act as a tumor suppressor in melanocytes.

Here, we report that melanoma cells with forced expression of *miR-211* increase signature pattern of metabolic re-programming that includes increased oxygen consumption, glutamine uptake, and fatty acid synthesis and downregulate the transcription factor hypoxia inducible factor 1α (HIF-1α) and undergo cell death when exposed to hypoxia. We have also identified a subset of novel *miR-211* target genes involved in these metabolic changes, including pyruvate dehydrogenase kinase 4 (PDK4), which inhibits the pyruvate dehydrogenase complex, a critical step in the entry of glucose and lactate-derived pyruvate into the Tricarboxylic Acid cycle (TCA) in both normal and cancer cells. These results provide important mechanistic insights into the function of *miR-211* in the metabolic re-programming of melanoma development in human.

CS32

Exploring the role of IRF4 in melanocyte responses to interferon- gamma signalling

A. Smith[1], R. Kim[1], A. Soogrim[1], M. Fane[1], R. Sturm[2]

[1]School of Biomedical Sciences; [2]Institute fro Molecular Bioscience/School of Medicine, University of Queensland, Brisbane, Qld, Australia

Total nevus number and exposure to UVR represent the greatest risk factors in the development of cutaneous melanoma. Recent GWAS studies have revealed the presence of a SNP rs12203592*C/T within the fourth intron of the Interferon Regulatory Factor 4 gene (IRF4) that is associated with pigmentation, nevus count, UVR sensitivity and melanoma susceptibility. While this association has been identified in numerous independent GWAS, the functional effect of the SNP and role of the IRF4 transcription factor in melanocyte cell function is unknown. IRF4 and other members of the IRF family have been recognised as key mediators of cellular differentiation and gene regulation in other systems. They are crucial regulators of interferon responses in these biological contexts. Interestingly, an Interferon gamma (IFNγ) -dependent crosstalk between melanocytes and macrophages has recently been reported that allows UVR-irradiated melanocytes to evade immune-surveillance, which ultimately contributes to melanomagenesis. Using primary human melanocyte cultures we have demonstrated differential expression of IRF4 between individuals homozygous for the T and C allele, with C/C individuals having significantly higher IRF4 expression. Analysis of the cis-regulatory activity of the IRF4 intron 4 suggests the SNP directly effects the transcriptional regulation of the IRF4 gene. To gain further insight into the function of IRF4 in melanocytes, transcriptome profiling of primary human melanocytes following siRNA-mediated IRF4 knock-down was performed, in the presence or absence of IFNγ. Validation of a

 © 2014 John Wiley & Sons A/S. Published by John Wiley & Sons Ltd

number of candidate genes by real time PCR and examination of the expression of these genes in melanocytes of known IRF4 genotype provides evidence for a role for IRF4 in mediating melanocytic interferon responses. Current investigations are aimed at further understanding the role of IRF4 in melanocyte function and exploring the link between differential IRF4 expression and the response of melanocytes to IFNγ previously linked to melanomagenesis.

Concurrent session: Advanced Surgical Interventions in Vitiligo

CS33

Effect of procedural-related variables on melanocyte-keratinocye suspension transplantation in stable vitiligo: a clinical and immunohistochemical study

D. Bassiouny[1], B. M. El-Zawahry[1], S. Esmat[1], N. Sameh[1], R. Sobhi[1], D. Abdel-Halim[1], M. Adly[1], M. safwat[1], R. Hegazy[1], H. Gawdat[1], N. Samir[1], M. Khorshied[2], H. Gouda[2], Z. El-Maadawi[3]
[1]Dermatology; [2]Clinical and Chemical Pathology; [3]Histology, Cairo University, Cairo, Egypt

Surgical treatment of stable vitiligo by melanocyte-keratinocye suspension (M-K susp) is gaining popularity among dermatologists due to its simplicity and efficacy. Several studies addressed the issue of patient-related variables such as duration of stability, extent and type of vitiligo. Despite variations in the technique described in literature no assessment of effect of procedural-related variation was done.
Assessment of the effect of different procedural-related variables (donor tissue, preparation of recipient site) and cytological and immune-histochemical composition of the suspension on the percentage of repigmentation in cases of stable vitiligo.
Forty cases of stable non-segmental vitiligo were treated surgically by M-K susp. Donor site was thierch graft in 34 cases and Hair follicle units (HFU) from the scalp in 6 cases. Recipient site was prepared by cryo-cautery in 24 cases versus CO2 laser resurfacing in 16 cases. Cell viability, count and immunohistochemical staining for melanocytes (Melan-1) and Keratinocytes (cytokeratin) in M-K susp were examined in 15 cases. All cases received nb-UVB phototherapy twice weekly following the procedure. Digital photography was done before and monthly after treatment. Assessment of repigmentation of each vitiligo lesion was done 3–17 months after the procedure using a simple modification of the VASI scoring. Complications and downtime in days following therapy were also assessed.
Recepient site preparation: 22 of 24 cases done using cryo cautery returned for follow up. Five patients showed 90–100% repigmentation, four 75%, three 50%, three 25%, four 10% and three 0% repigmentation (41% of cases achieved >75% repigmentation). Mean downtime till full healing of cryo blebs was 15 days. 14 of 16 cases done using CO2 laser resufacing returned for follow up. Three showed 90–100% repigmentation, three 75%, five 50%, two 25% and one 0% repigmentation (43% of cases achieved>75% repigmentation). Mean downtime was 7 days. CO2 laser resurfacing of the recipient site produced better colour matching and homogenecity.
Donor site: 30 cases done by thiersh grafting returned for follow up. Seven patients showed 90–100% repigmentation, six 75%, seven 50%, three 25%, four 10% repigmentation and three none (0%) (43% of cases achieved>75% repigmentation). Mean time of healing of thierch graft site was 10 days. Of the 6 cases done using HFU one showed 90–100% repigmentation, one 75%, one 50%, two 25% and one 10% repigmentation (33% achieved >75% repigmentation). HFU harvesting was more difficult and time consuming than thierch grafts. Scalp needed a mean time of 5 days to heal.
Cell viability was slightly higher in HFU samples. Cell count and percentage of keratinocytes was significantly higher in M-K susp obtained from thierch grafts and positively affected repigmentation.
Although comparable percentage of repigmentation was obtained with both techniques applied to recipient site, CO2 resurfacing had a beneficial effect in terms of better homogenecity of repigmentation and less downtime. Obtaining donor sample in the form of thierch graft produced higher concentration of cells with better outcome but the small sample size of HFU donors in this study is a limitation. However HFU harvesting produced no visible scar and faster healing than thiersh grafts.

CS34

Transplantation of autologous noncultured extracted hair follicle outer root sheath cell and autologus noncultured epidermal cell suspension in combination as a novel method in vitiligo surgery

M. T. Razmi, D. Parsad, S. M. Kumaran
Dermatology, PGIMER, Chandigarh, India

Autologous non cultured epidermal cell suspension (NCES) and autologus non cultured extracted hair follicle outer root sheath cell suspension (FCS) are established surgical modalities for treatment of stable vitiligo. However there is a need for refinement of these surgical techniques for better outcome, especially over resistant sites.
To study the efficacy of transplanting combination of NCES and FCS (NCES+FCS) as a novel technique in vitiligo surgery.
Thirty stable vitiligo patients, with bilaterally symmetrical patches or multiple patches in the same anatomical region, were recruited for the study. NCES+FCS is obtained by mixing equal proportion of NCES and FCS. Using random number tables the matched patches were treated either with NCES+FCS or NCES alone in the same patient. They were followed up at 4, 8 and 16 week intervals by a blinded observer and extent of repigmentation, color match, pattern of repigmentation and complications were noted. Patient satisfaction was assessed with patient global assessment (PGA) questionnaire at 16 week visit.
Total 30 patients underwent surgery, of these, 13 patients (three males, 10 females), with 16 paired patches, completed 16 weeks follow up till now, who will be denoted as study population. The mean age of the study population was 22.63 ± 6.5 yr and mean lesional stability was 3.43 ± 3.72 yr. Site of surgery in 75% patients were acral or bony area. The mean surface area treated in NCES+FCS (Group A) was 8.563 and NCES (Group B) was 8.406 (P = 0.923). At week 16, the extent of repigmentation was excellent (showing 90–100% repigmentation) in 56.3% in Group A compared to 12.5% in Group B (P = 0.475). Good repigmentation (>75% repigmentation was seen in 81.3% in Group A and 50% in Group B (P = 0.200). Repigmentation was also early in Group A, in which good repigmentation was seen in 43.75% as early as 4 weeks compared to 12.5% in Group B (P = 0.094). Eleven patches were of difficult type of vitiligo (vitiligo vulgaris and acrofacial vitiligo), in which repigmentation was good, 72.7% in

© 2014 John Wiley & Sons A/S. Published by John Wiley & Sons Ltd

Group A and 54.5% in Group B (P = 0.061). PGA score was 24.6 and 20.5 in Group A and Group B respectively (P = 0.026). Surgery of choice in future was NCES+FCS in 68.8%, NCES in 12.5% and both in 18.8% (P = 0.095). Color matching was seen in 75% in Group A and 62.5% in Group B (P = 0.003). Complications were minimal in both groups.
NCES+FCS showed better results in terms of extent of repigmentation, early achievement of good repigmentation and patient satisfaction than NCES. This novel method may suffice quench for a refined surgical technique to get early result and an apt technique for acral vitiligo.

CS35

A 5-year review of noncultured cellular grafting for vitiligo

E. Y. Gan, Y. L. Kong, S. T. G. Thng, B. K. Goh
National Skin Centre, Singapore City, Singapore

Noncultured cellular grafting is an advanced surgical technique for vitiligo. In this study, we evaluated our centre's 5-yr follow-up results.
All patients with stable vitiligo, who had undergone noncultured cellular grafting from March 2006 to December 2012, were included. Standard grafting procedures were performed for all. Hyaluronic acid dressings were switched to collagen dressings in April 2008. Clinico-epidemiological characteristics and repigmentation data were reviewed. The primary outcome was degree of repigmentation at 12 months. Secondary outcome measures included repigmentation rates up to 60 months post-grafting and proportion with loss of initially achieved repigmentation. Vitiligo repigmentation was graded as 'poor', 'fair', 'good' and 'excellent', corresponding to 25% intervals on a scale of 0–100% repigmentation.
There were 177 patients, with a mean age of 34 yr and majority (86%) of Fitzpatrick skin type IV. Mean duration of vitiligo prior to grafting was 99 months. Ninety-eight patients (55%) had non-segmental, 77 patients (43%) had segmental and two had mixed vitiligo. Out of 197 grafting sessions, 132 were primary, 29 were repeated and 36 were staged. The most commonly grafted sites were the face/neck (60%) and trunk (17%). Of those on active follow-up, good-to-excellent repigmentation was present in 75% at 3 months, 77% at 12 months and 82% at 60 months. At 12 months, 88% (n = 52) with segmental vitiligo achieved good-to-excellent repigmentation compared to 71% (n = 55) with non-segmental and 33% (n = 1) with mixed vitiligo ($P < 0.05$). Patients without a history of koebnerisation or active disease achieved better final repigmentation outcome ($P < 0.05$). A larger proportion of patients on collagen dressings (82%) achieved good-to-excellent repigmentation compared to those on hyaluronic acid dressings (63%) ($P < 0.05$). Anatomical site of lesions and use of post-grafting phototherapy did not significantly affect final repigmentation outcome. Loss of initially achieved repigmentation occurred in 11% of grafting procedures, due to active disease. There was no significant scarring or adverse reactions. The study was limited by its retrospective nature and gradual loss to follow up of patients after 12 months.
This study is to our knowledge, the largest published cohort of patients undergoing noncultured cellular grafting at a single centre. Noncultured cellular grafting is safe, efficacious and durable. Most patients achieve maximal repigmentation by 12 months, lasting till at least 60 months. Better repigmentation occurred in segmental vitiligo and in those who received collagen dressings. Post-grafting phototherapy did not affect final repigmentation outcome. Careful patient selection also allows good-to-excellent results in non-segmental vitiligo (71%) and difficult areas such as acral sites.

Concurrent sessions: Model Systems for Pigment cell Biology & Disease

CS36

Fibroblasts regulate both physiological and pathological pigmentation of skin in vitro and in vivo

M. Cario-André[1,2]
[1]INSERM 1035, University Bordeaux; [2]Department of Dermatology, University Hospital, Bordeaux, France

We have previously described that white human skin or white human epidermal reconstructs xenografted onto nude mice could become black or totally white. These modifications of pigmentary pattern were associated with changes in densities of dermal fibroblasts. In vitro, we have compared reconstructs made on DDD colonized or not with various concentrations of normal fibroblasts and we have reproduced this phenomenon of increase or decrease of pigmentation. Using media conditioned by UVA-irradiated fibroblasts on epidermal reconstructs we were able to reproduce the main features of senile lentigo (increased pigmentation, increased rete ridges and impaired skin barrier) confirming the role of fibroblasts in pigmentation. Furthermore, since in systemic scleroderma, a disease characterized by an excessive secretion of collagen by fibroblasts, pigmentation changes are often marked, we have reconstructed epidermis on DDD colonized by fibroblasts of systemic scleroderma (SSc) patients and demonstrated that for a same seeding density SSc fibroblasts, cells can either stimulate or inhibit pigmentation according to patients. Since fibroblast dermal density in physiological conditions, or status of fibroblasts in pathological or stressed conditions regulate pigmentation, we looked at possible fibroblast-derived molecules responsible for this effect. Fibroblasts secreted FGF-2 is a well-known pigment enhancer and a good candidate in this setting. By immunohistochemistry, western-blot or ELISA we measured the level of epidermal FGF-2 in mouse xenografts and SSc patients. The level of FGF2 was low in hypopigmented xenografts and high in hyperpigmented xenografts, showing an inverse correlation with the number of fibroblasts. Increase in FGF-2 was associated with an increase in keratin 5, the keratin associated with a genetic hyperpigmentary disorder: Dowling Degos disease. In patients with SSc, FGF-2 was also differentially expressed. In vitro treatment of reconstructed epidermis without fibroblasts with FGF2 increased epidermal FGF2 and cytokeratin 5 expressions. Using our model of reconstructs on DDD colonized by increasing densities of FGF2 overexpressing fibroblasts we observed, as in mouse xenografts, that pigmentation and epidermal FGF-2 were not increased in presence of low or high number of FGF-2 transduced fibroblasts. Since low level of FGF-2 was associated with depigmentation we looked at the expression of FGF-2 in skin biopsies of non lesional, perilesional and lesional vitiligo/NSV patients. We measured a significant decrease in FGF-2 in peri-lesional skin associated with an increased number of dermal vimentin positive cells. In vitiligo/NSV, the pattern of expression of FGF-2 is similar to that of one of its target molecule CCN3. CCN3 is a matricellular protein, which regulates melanocyte adhesion to the basal membrane via DDR1-collagen IV. CCN3 and CCN2 are associated with fibrosis. In systemic scleroderma CCN2 is increased and SSc fibroblasts in culture secrete more

 © 2014 John Wiley & Sons A/S. Published by John Wiley & Sons Ltd

CCN3. Thus we measured CCN3 in mouse xenografts and SSc patients. Similar to NSV, CCN3 expression followed that of FGF-2. In conclusion, our data associated with clinical observations in pigmentary disorders support the hypothesis that fibroblasts modulate pigmentation by paracrine factors especially the FGF2-CCN3 axis. CCN3 activity in skin should be investigated as possible target for the treatment of SSc patients with pigmentary troubles.

CS37

Investigating the modulation of cutaneous pigmentation in an Asian reconstructed pigmented epidermis using multiphoton microscopy

J. Qiu[1], J. Liu[1], X. Huang[1], J. Chen[1], A.-M. Pena[2], Z. Cai[1]

[1]L'OREAL Research and Innovation, Shanghai, People's Republic of China; [2]L'OREAL Research and Innovation, Aulnay-sous-bois, France

Assessing the amount, distribution and transfer of melanin in the epidermis is essential for medical and cosmetic applications. Reconstructed 3D human skin equivalents, showing morphological and functional characteristics similar to in vivo human skin, are now recognized as relevant in vitro models for studying skin biology and functional mechanisms. Multiphoton microscopy has been shown to be an appropriate tool for in situ 3D characterization of melanin, since it provides a direct 3D noninvasive visualization of tissue architecture and cell morphology without the need of exogenous contrasting agents. This study aimed at evaluating Asian skin pigmentation and its modulation by constructing and using an organotypic epidermis model of Asian origin. The Asian pigmented epidermis model was developed using normal human melanocytes and keratinocytes from Chinese origin. This model presents well-stratified histological structures, with basal layer, spinous layer, granular layer, and horny layer, similar to those found in vivo. The melanocytes are well-integrated into the basal layer and exhibit a good dendritic morphology. Using in vitro multiphoton microscopy, the 3D distribution of melanin in the Asian pigmented epidermis and its modulation by whitening compounds have been characterized. About a hundred 3D stacks of multiphoton images of reconstructed skin samples were acquired by the Nikon A1RMP/FN1 microscope, taking advantage of intrinsic multiphoton signals from keratin, NAD(P)H, flavins, melanin. Based on these images, we developed a new melanin quantification method to allow the acquisition of quantitative parameters in each epidermal stack, thus the melanin in melanocytes and keratinocytes can be analyzed respectively. The total melanin content in the reconstructed epidermis and the transferred melanin in keratinocytes can then be separately quantified, thereby providing a new way to analyze the modulation of pigmentation. Our study demonstrates that the application of multiphoton microscopy with specific image analysis on in vitro Asian pigmented epidermis model can be a powerful tool to assess the mechanisms of Asian skin pigmentation and skin response to various modulators.

CS38

Altered E-cadherin levels and distribution in melanocytes precedes clinical manifestations of vitiligo

V. Delmas[1], R. Wagner[1], F. Luciani[1], M. Cario-Andre[2], A. Rubod[1], V. Petit[1], L. Benzekri[3], K. ezzedine[2], S. Lepreux[2], A. Taeib[4], Y. Gauthier[2], L. Larue[1]

[1]UMR 3347 CNRS-U1021 INSERM, Institut Curie, Orsay; [2]Service de dermatologie, Hopital Saint Andre, Bordeaux; [3]Ibn Sina, Univeristy Hospital, Rabat, Morocco; [4]Service de dermatologie, Institut Curie, Orsay, France

Vitiligo is a frequent depigmenting disorder resulting from the loss of melanocytes in the basal layer of the epidermis. The pathogenesis of the disease is likely multifactorial and involves autoimmune causes as well as oxidative and mechanical stress. It is important to identify early events in vitiligo, to clarify pathogenesis, improve diagnosis and inform therapy. Here, we show that E-cadherin, which mediates the adhesion between melanocytes and keratinocytes in the epidermis, is absent from, or discontinuously distributed across melanocyte membranes of vitiligo patients long before clinical lesions appear. The abnormal distribution of E-cadherin correlated with lower melanocyte numbers in the basal epidermal layer and higher melanocyte numbers in the suprabasal layer. Using reconstructed human epidermis and mouse models with defective E-cadherin expression in melanocytes, we show that E-cadherin is required for melanocyte adhesiveness to the basal layer under oxidative and mechanical stress. In stressed murine melanocytes lacking E-cadherin and in vitiligo epidermis the detached melanocytes are not adequately replaced in the basal layer, possibly due to an exhaustion of melanocyte renewal. We suggest that altered E-cadherin distribution in melanocytes precedes vitiligo pathogenesis but remains silent until melanocyte renewal becomes a limiting factor for normal pigmentation of the skin. These observations establish a link between pre-clinical, cell-autonomous defects in vitiligo melanocytes and known environmental stressors accelerating disease expression. Our results implicate a primary predisposing skin defect affecting melanocyte adhesiveness, which under stress conditions, leads to disappearance of melanocytes and clinical vitiligo. Melanocyte adhesiveness is thus a potential target for therapy aiming at disease stabilization.

CS39

Activation of melanocyte antioxidant response pathways following exposure to vitiligo-inducing phenols: implications for vitiligo pathogenesis

O. A. Arowojolu, S. J. Orlow, P. Manga

Dermatology, New York University School of Medicine, New York, NY, USA

Vitiligo is a common disorder characterized by progressive melanocyte death. Vitiligo can be induced in an occupational setting by exposure to vitiligo-inducing phenols (VIPs) such as 4 tert-butyl phenol (4TBP) and monobenzyl ether of hydroquinone (MBEH). These VIPs are believed to specifically target melanocytes due to their structural similarity to tyrosine and competition for binding to tyrosinase, the rate-limiting enzyme for melanin synthesis. By exposing normal melanocytes to VIPs and using microarray analysis and bioinformatics approaches for gene expression profiling, we identified key signaling pathways that are involved in the melanocyte response to VIP exposure. In particular, we hypothesized that exposure of primary human

© 2014 John Wiley & Sons A/S. Published by John Wiley & Sons Ltd

melanocytes to VIPs would result in oxidative stress that triggers antioxidant responses in order to protect melanocytes from cell death. Following melanocyte exposure to VIPs, HO-1 was upregulated (4TBP exposure: 5.49-fold; MBEH exposure: 25.98-fold). HO-1 is a direct target of Nrf2, a key regulator of the Nrf2-ARE antioxidant response. Activation of Nrf2 and its targets, HO-1 and NQO1, was confirmed by conventional Western blot analysis and quantitative RT-PCR. We are now characterizing the mechanisms that regulate Nrf2 activation in response to VIPs. Additional antioxidants including SOD2 (4TBP: 10.5-fold; MBEH: 38.5-fold), peroxiredoxin 6 (4TBP: 2.44-fold; MBEH: 15.18-fold), and Nrf2 binding partners, MafK (4TBP: 2.12-fold; MBEH: 2.50-fold) and MafF (4TBP: 2.32-fold; MBEH: 3.16-fold) were also upregulated with VIP exposure and are being investigated further. Several studies have revealed dysfunctional antioxidant responses in melanocytes from patients with vitiligo, however the underlying mechanisms that reduce their efficacy are yet to be determined. We hypothesize that investigation of VIP-induced pathways may lead to the characterization of these mechanisms and provide opportunities for development of targeted therapeutics for the treatment of vitiligo.

Concurrent sessions: Neurofocrinology and Regulatory Controls of Pigmentation

CS40

Dopamine D4 receptor antagonist inhibits a-MSH-stimulated induced melanogenesis through the downregulation of MITF via acceleration of ERK activation

S. Choi, H. I. Cho, J. M. Jung, M. W. Lee, J. H. Choi, S. E. Chang
Department of Dermatology, Asan Medical Center, University of Ulsan College of Medicine, Seoul, Republic of Korea

Dopamine actions are mediated through five specific cell surface receptors coupled to G proteins and belonging to two main families (D1-like and D2-like receptors). Although these receptors have been shown to participate in the regulation of melanin synthesis process, there have been no report or study concerning the relation of dopamine receptor D4(DRD4) and melanogenesis. Hence, we examined the effects of DRD4 antagonist on melanogenesis and its related signaling pathways using Mel-ab mouse melanocyte cells and B16F10 mouse melanoma cells. Present results showed that melanin content was significantly reduced in both cells after DRD4 antagonist treatment in a concentration-dependent manner. We also investigated changes in the phosphorylation of AKT, which is related to melanin regulation. These results indicated that DRD4 antagonist treatment led to the phosphorylation of AKT. To better understand the molecular mechanisms of the change, we examined regulation of PI3K/AKT pathways using LY294002, a selective inhibitor of PI3K. LY294002 treatment significantly increased melanin production with DRD4 antagonist treatment, suggesting that DRD4 antagonist's antimelanogenic effect was regulated by AKT phosphorylation.
Additionally, DRD4 antagonist treatment reduced both microphthalmia-associated transcription factor (MITF) and tyrosinase protein levels. These results suggest that the hypopigmentary action of DRD4 antagonist is due to MITF and tyrosinase downregulation by AKT activation.

CS41

Retinoic acid triggers difference of melanogenesis on human epidermal melanocytes and melanoblasts

T. Kawakami[1], A. Ohgushi[1], T. Hirobe[2], Y. Soma[1]
[1]Department of Dermatology, St. Marianna University School of Medicine, Kawasaki; [2]Fukushima Project Headquarters, National Institute of Radiological Sciences, Chiba, Japan

Pigmentary disorders such as senile pigment freckle and melisma are difficult to treat; topical retinoid acid is one choice. However, the basic mechanisms underlying retinoid acid treatment remain unknown. We previously showed that retinoic acid inhibited the proliferation of mouse melanoblast and induced apoptosis.
We investigated the effects of retinoic acid on the proliferation and differentiation of melanocyte using human melanocytes and melanoblasts when various concentrations of retinoic acid were added to the culture.
Normal human adult epidermal melanocytes (HEMa-LP cells) were purchased and human epidermal undifferentiated melanoblasts were cultured as described previously. We used the Alamar blue assay for cell proliferation analysis, and examined melanogenesis, performed electron microscopy, western blot analysis, and RT-PCR method.
Addition of retinoic acid decreased cell proliferation of both human melanocyte and melanoblast significantly in a dose-dependent manner. Addition of retinoic acid decreased tyrosinase activity of human melanocyte significantly. In contrast, the addition did not affect tyrosinase activity of human melanoblasts. Retinoic acid addition decreased endothelin B receptor protein expression using Western blot analysis. Addition of retinoic acid decreased tyrosinase, Microphthalmia-associated transcription factor, and tyrosinase-related protein 1 on melanocyte using RT-PCR analysis.
The results revealed that topical retinoic acid could trigger difference of melanogenesis on human epidermal melanocytes and melanoblasts. These findings were consistent with the clinical facts that topical retinoids are effective for pigmentary disorders including senile pigment freckle.

CS42

Differences in visible light induced pigmentation according to wavelengths: a clinical and histological study in comparison with UVB exposure

L. Duteil[1], N. Cardot-Leccia[2], C. Queille-Roussel[1], Y. Maubert[1], D. Ambrosetti[2], J.-P. Lacour[3], T. Passeron[3,4]
[1]Centre de Pharmacologie Clinique Appliquée à la Dermatologie (CPCAD); [2]Pathology, University Hospital of Nice; [3]Dermatology, University Hospital of Nice; [4]U1065 team 12, C3M, INSERM, Nice, France

The role of visible light in pigmentation has been recently emphasized. However, it can be hypothesized that all the wavelengths between 400 and 700 nm do not induce the same photobiological effects on pigmentation.
To assess the potential pro-pigmenting effects of two single wavelengths located at both extremities of the visible spectrum: the blue/violet line (l = 415 nm), and the red line (l = 630 nm), as compared to UVB.
Twelve healthy male subjects (mean age 27 ± 5 yr) were recruited for the study (six with skin type III and 6 with skin type IV). Colorimetric, clinical, and histological assessments were

 © 2014 John Wiley & Sons A/S. Published by John Wiley & Sons Ltd

used to evaluate the pigmentation. In a first step of the study increasing doses of 415 and 630 nm light were given using Light Emitting Diode to assess the Minimal Pigmentary Dose of each wavelength. MPD was defined as the first dose which induced a coloration difference of $\Delta L^* = -2$ compared to the non-exposed skin. In a second step, we compared these irradiations (at the MPD or at 150 J/cm² if the MPD could not be determined), to non-exposed and UVB exposed skin (1.5 MED). Morphologic examination was done on Hematoxylin and Eosin and Fontana Masson stained sections. Immunostaining was performed using the primary antibodies against MITF, p53, Ki67, and Oxoguanine 8. A semi quantitative evaluation (from 0 to +++) was performed by the pathologist who was blinded to the irradiation received on the skin.

The colorimetric and clinical assessments showed a clear dose-effect with the 415 nm irradiation, in both skin type III and IV subjects, whereas the 630 nm did not induce hyperpigmentation. When compared to UVB irradiation, the blue-violet light induced a significantly more pronounced hyperpigmentation that lasted up to 3 months. Histological examinations showed a significant increase of keratinocyte necrosis and p53 positive staining with UVB, as compared to 415 and 630 nm exposures.

This study demonstrates that various wavelengths of the visible part of the solar spectrum have different effects on the skin pigmentation. Although inducing a potent and long-lasting hyperpigmentation, the 415 nm light does not lead to a significant increase of p53, suggesting that other pathways are involved in this induced melanogenesis. The doses of blue-violet light that stimulate the pigmentation are achieved in <2 h of sun exposure, suggesting that those wavelengths could play a key role in the worsening of some pigmentary disorders after sun exposure, despite the use of sunscreens with a broad and efficient UVB and UVA protection.

Plenary Session: Melanoma Biology

CS43

Targeted activation of the type I IFN system in combination with PD-1 blockade is a rational strategy to expose immune cell-poor HGF-CDK4 mouse melanomas to cellular immune surveillance

T. Bald[1], J. Landsberg[1], D. A. Lopez-Ramos[1], M. Renn[1], N. Glodde[1], P. Jansen[1], E. Gaffal[1], J. Steitz[2], R. Tolba[2], U. Kalinke[3], G. Jönsson[4], M. Hölzel[5], T. Tüting[1]

[1]Department of Dermatology and Allergy, University of Bonn/ Experimental Dermatology, Bonn; [2]Institute for Laboratory Animal Science, University Hospital Aachen, Aachen; [3]Twincore, Centre for Experimental and Clinical Infection Research, Hannover, Germany; [4]Department of Oncology, Clinical Sciences, Lund University, Lund, Sweden; [5]Department of Clinical Chemistry and Clinical Pharmacology, University of Bonn/Unit for RNA Biology, Bonn, Germany

Infiltration of human primary melanomas with cytotoxic immune cells correlates with the spontaneous activation of the type I interferon (IFN) system and a favorable prognosis. Therapeutic antibody mediated blockade of immune inhibitory receptors in patients with pre-existing lymphocytic infiltrates prolongs survival. However, new complementary strategies are needed to efficiently activate innate and adaptive anti-tumor immunity in immune cell-poor human melanomas. In this study we experimentally demonstrated that primary DMBA-induced cutaneous melanomas in Hgf-Cdk4(R24C) mice, which imitate a sub-group of human immune cell-poor melanomas with a low type I IFN response signature, escape type I IFN-induced immune surveillance as well as immunoediting. Peritumoral injections of the prototypic immunostimulatory RNA polyinosinic: polycytidylic acid (polyI:C) initiated a cytotoxic inflammatory response in the tumor microenvironment and significantly impaired tumor growth of primary as well as transplanted Hgf-Cdk4(R24C) melanomas. This critically required the coordinated activation of the type I IFN system by dendritic, myeloid, NK and T cells. However, targeted activation of the type I IFN system led to an up regulation of PD-L1 on melanoma cells and increased the number of PD1+CD8+ circulating T cells in the peripheral blood of melanoma bearing mice treated with poly(I:C). Importantly, combination of type I IFN activation with antibody-mediated blockade of the immune-inhibitory receptor PD-1 significantly prolonged survival of melanoma bearing mice. These results highlight important interconnections between the type I IFN system and immune-inhibitory receptor signaling in melanoma pathogenesis which serve as targets for combination immunotherapies.

Plenary Session: Targeted Therapies and Drug Resistance

CS44

Paracrine effect of neuregulin 1 and hepatocyte growth factor drives resistance to mitogen-activated protein kinase kinase inhibitors in metastatic uveal melanoma

H. Cheng[1], M. Terai[2], K. Kageyama[2], S. Ozaki[2], T. Sato[2], A. Aplin[1,3]

[1]Cancer Biology, Thomas Jefferson University; [2]Medical Oncology, Thomas Jefferson University; [3]Dermatology and Cutaneous Biology, Thomas Jefferson University, Philadelphia, PA, USA

Metastatic uveal melanoma (UM) patients usually die within 1 yr, emphasizing an urgent need to develop new treatment strategies. Mitogen-activated protein kinase kinase (MEK) small molecule inhibitors improve survival in cutaneous melanoma patients but show only modest efficacy in UM patients. In this study, we identified compensatory mechanisms that provide resistance to MEK inhibition in metastatic UM. We screened for growth factors that were able to induce resistance in three newly characterized metastatic UM cell lines to MEK inhibitors, trametinib and selumetinib, with colony formation assay and viability assay. Cellular signaling pathway analysis was carried out to determine the molecular mechanisms of resistance to MEK inhibition in metastatic UM cells. Clinical-grade antibody and inhibitors targeting these pathways were used to determine their capability to overcome the resistance mediated by these growth factors. Conditioned medium collected from fibroblasts were used to evaluate whether stromal effect from fibroblasts could drive resistance to metastatic UM cells. We demonstrate that Neuregulin 1 (NRG1) and hepatocyte growth factor (HGF) provide resistance to MEK inhibition. Mechanistically, trametinib enhances the responsiveness to NRG1, whereas HGF induces sustained activation of AKT in the presence of trametinib. Individually targeting ERBB3 and cMET, the receptors for NRG1 and HGF, overcomes resistance to trametinib provided by these growth factors and by conditioned medium from fibroblasts, which produce NRG1 and HGF. Analysis of 10 metastatic liver biopsies showed that ERBB2, which is the co-

© 2014 John Wiley & Sons A/S. Published by John Wiley & Sons Ltd

receptor for NRG1, and cMET were phosphorylated in some of these clinical specimens. Our data suggest that combination of MEK inhibitors with ERBB3 and cMET targeting agents may provide novel and effective treatment strategies in metastatic UM.

CS45

Targeting melanoma bulk and cancer initiating cells with a combination of the retinoid derivative fenretinide and a BCL-2 inhibitor

Y. Shellman, N. Mukherjee, S. Reuland, Y. Lu, Y. Luo, M. Fujita, D. Norris

Dermatology, School of Medicine, University of Colorado Denver, Aurora, CO, USA

Developing effective treatments for melanoma is still a pressing issue in medicine. Multiple laboratories have recently verified the existence of cancer initiating cells in melanoma (MICs), which contribute to melanoma's resistance to treatments. Recent approved drugs show exciting short term success, but many patients relapse or do not respond probably due to the failure of these drugs to target the MICs. Thus, developing other treatment options targeting melanoma including MICs is still urgent. ABT-737/ABT-263, small molecule inhibitors against Bcl-2/Bcl-XL/Bcl-w, has shown promising results in treating various cancers. However, it has not been very successful in treating melanoma as a single agent, and inhibiting anti-apoptotic protein Mcl-1 can strongly sensitize the melanoma cells to ABT-737. 4-HPR, a synthetic retinoid, acts as a Mcl-1 inhibitor and is currently in clinical trials for treating breast cancers. In this study, we tested the effects of combining ABT-737 and 4-HPR in melanomas, both the bulk and the MICs, in both melanoma cell lines and patient samples. Our results indicated that the combination synergistically decreased cell viability and increased cell death in multiple melanoma cells lines but not in melanocytes. In addition, the combination treatment disrupted the primary spheres and decreased the percentage of $ALDH^+$ cells in multiple melanoma cell lines and in patient samples. Moreover, the secondary sphere-forming assays with multiple cell lines indicated that only the combination significantly inhibited self-renewability of MICs. In summary, results here indicated that only the combination of ABT-737 plus 4-HPR, but not the single drug treatments, can both de-bulk the melanoma cells and inhibit the self-renewability of MICs. Preliminary in vivo studies also indicated that the combination inhibits tumor growth more than single drug treatment. Thus this combination may be a promising strategy for treating melanoma, and results here provide scientific basis for taking this combination into clinical trial.

Concurrent session: Senescence Pathways and Melanoma

CS46

Suppression of autophagy dysregulates the antioxidant response and causes premature senescence of melanocytes

C. F. Zhang[1,2], F. Gruber[1,3], C. Ni[1,2], M. Mildner[1], U. Koenig[1], S. Karner[1], C. Barresi[1], H. Rossiter[1], L. Larue[4,5,6], D. Tobin[7], L. Eckhart[1], E. Tschachler[1]

[1]Research Division of Biology and Pathobiology of the Skin, Department of Dermatology, Medical University of Vienna, Vienna, Austria; [2]Department of Dermatology, Huashan Hospital, Fu Dan University, Shanghai, People's Republic of China; [3]Christian Doppler Laboratory for Biotechnology of Skin Aging, Department of Dermatology, Medical University of Vienna, Vienna, Austria; [4]Institut Curie, Centre de Recherche, Developmental Genetics of Melanocytes; [5]CNRS UMR3347; [6]INSERM U1021, Orsay, France; [7]University of Bradford, Centre for Skin Sciences, Bradford, West Yorkshire, UK

Autophagy is the central cellular mechanism for delivering organelles and cytoplasm to lysosomes for degradation and recycling of their molecular components. To determine the contribution of autophagy to melanocyte (MC) biology, we knocked out specifically in melanocytes the essential autophagy gene *Atg7* using the Cre-loxP system. Mice carrying a floxed Atg7 gene were crossed with mice expressing the Cre recombinase under the control of the tyrosinase promoter which is active in neural crest–derived pigment cells. This gene deletion efficiently suppressed a key step in autophagy, lipidation of microtubule-associated protein 1 light chain 3 beta (LC3), in MC and induced slight hypopigmentation of epidermis in mice. The melanin content of hair was decreased by 10–15% in mice with autophagy-deficient MC as compared to control animals. When cultured in vitro, MC from mutant and control mice produced equal amounts of melanin per cell. However, *Atg7*-deficient MC went into premature growth arrest, accumulated ubiquitinated proteins and the multi-functional adaptor protein SQSTM1/p62. Moreover, nuclear factor erythroid 2–related factor 2 (Nrf2)-dependent expression of NAD(P)H dehydrogenase, quinone 1 (Nqo1) and glutathione S-transferase Mu 1 (Gstm1) was increased, indicating a contribution of autophagy to redox homeostasis in MC. In summary, the results of our study suggest that Atg7-dependent autophagy is dispensable for melanogenesis but necessary for achieving the full proliferative capacity of MCs.

CS47

Inhibition of HDAC3 accelerates autophagy to induce melanoma cell death

X. Wang[1], A. Velez[1], J.-Y. Lu[2], A. S. Paller[1]

[1]Dermatology, Northwestern University's Feinberg School of Medicine, Chicago, IL, USA; [2]Dermatology, The Third Xiangya Hospital, Zhongnan University, Changsha, People's Republic of China

Although non-specific HDAC inhibitors have shown early promise in clinical trials in preventing tumor progression, little is known about the role of histone deacetylases (HDACs) in melanomas. We have previously found that specific inhibition of HDAC3 reduces ganglioside GM3 deacetylation to prevent activation of PI3K/Akt and mTOR/p70S6 kinase signaling, thereby inducing cell death and inhibiting melanoma growth. We hypothesized that HDAC3

© 2014 John Wiley & Sons A/S. Published by John Wiley & Sons Ltd

inhibition induces melanoma death through both caspase-dependent apoptosis and caspase-independent autophagy. Using immunohistochemistry, qRT-PCR and western blot, we found that increased expression and cellular distribution of HDAC3 correlates with melanoma metastasis both in vivo and in vitro. When HDAC3 function was suppressed by a small molecule inhibitor (MS-275, specific inhibitor of the class I HDACs), the percentage of 'dead cells' was increased by 4-fold, correlating with a 10-fold increase in caspase-3 activation ($P < 0.001$) and a 4-fold reduction in Bcl-2 expression ($P < 0.01$). Importantly, targeted reduction of HDAC3 expression, rather than other class I HDACs (HDAC1/2), also led to a 5-fold increase in expression of beclin1 ($P < 0.001$) and a 3-fold increase in the expression of LC3 ($P < 0.01$), leading to melanoma cell autophagy. Inhibition of autophagy with chloroquine or caspase-3 with Z-DEVD-FMK reduced the death of metastatic melanoma cells induced by HDAC3 inhibition ($P < 0.01$), demonstrating that both caspase-independent autophagy and caspase-dependent apoptosis participate in HDAC3 inhibition induced melanoma cell death. Although autophagy is essential for normal cells survival in a stress environment, autophagy's impact on cancer progression and on the efficacy of cancer therapeutics is uncertain. Results from our study indicate that specific inhibition of HADC3, a promising target for treating metastatic melanoma, leads to cell death via induction of both autophagy and apoptosis.

CS48

Diterpenoid hirsein a activate nuclear P38 through GADD45B upregulation: possible route for induction of melanoma cell senescence

M. Villareal, H. Isoda

Faculty of Life and Environmental Sciences, University of Tsukuba, Tsukuba, Japan

DNA microarray analysis has revealed that the diterpenoid Hirsein A can inhibit the MITF gene and at the same time upregulate GADD45B, a gene associated with DNA damage, in B16 cells. We initially proposed that this compound may be used as a tyrosinase inhibitor for melanomas can increase the toxicity of anti-cancer drugs. Here, we explore the possibility that the increased GADD45B expression may induce cell senescence through MAPK p38 activation in human melanoma cells. Real-time PCR was used to validate the expression of GADD45B and determine the expressions of NFKB, melanoma-associated oncogenes (MITF, BRAF, and NRAS), while western blotting was performed to determine the expression of MAPK P38 in the nuclear and cytoplasmic fractions of SK-MEL 28 and SK-MEL5 human melanoma cells. Cell count was determined using flow cytometry. Results showed that GADD45B expression in SK-MEL 28 and SK-MEL5 cells was increased in B16, SK-MEL28 and SK-MEL5 cells. Determination of the expression of NFKB, which lies in the promoter region of GADD45B, revealed a significant increase in its expression. The expressions of oncogenes MITF and NRAS were decreased but not BRAF. Western blotting results showed that the increase in GADD45B expression resulted to the activation of P38 in the nuclear fraction of the cells but not in the cytoplasmic fractions. Treatment with 1 μM hirsein A for 48 h decreased the number of viable cells which was not observed in hirsein A-treated human epidermal melanocytes. GADD45B is known to activate MAPK p38 activation which has been reported to induce cell senescence in endothelial progenitor cells wherein activated p38 increase glucose-induced senescence. Previously obtained unpublished metabolomics analysis data revealed that hirsein A-treated cells glycolytic activity is increased in B16 melanoma cells. Here we present the diterpenoid hirsein A as a possible anti-melanoma agent through its ability to induce P38 activation resulting from GADD45B upregulation. Further research to understand the relationship between the inhibition of MITF and NRAS with the increase in GADD45B expression will be done, as well as to evaluate the overall effect of hirsein A on cell senescence-related signal transduction.

CS49

CDK4/6 as a therapeutic target in malignant melanoma

J. Teh, A. Aplin

Cancer Biology, Thomas Jefferson University, Philadelphia, PA, USA

Melanoma is the most dangerous form of skin cancer and FDA-approved therapies such as vemurafenib and ipilimumab are associated with short-term responses and/or severe toxicities for patients. Recent favorable clinical data with cyclin-dependent kinase (CDK) 4/6 inhibitor, palbociclib (PD'991) in ER+ breast cancer has rekindled an interest in targeting cell cycle progression to stop aberrant cell growth. Thus, we set out to analyze the potential use of CDK4/6 inhibitors in melanoma. Due to the inability of CDK4/6 inhibitors to induce apoptosis as a single agent, we explored viable combinatorial strategies that will trigger both pro-senescent and pro-apoptotic pathways that may lead to a more effective treatment modality for melanoma patients. Here, we demonstrate the benefit of combining a MEK inhibitor, trametinib (GSK'212) with palbociclib (PD'991) utilizing a panel of human melanoma cell lines. We did not detect any additive effects of combining both GSK'212 and PD'991 in cells wild-type for BRAF and NRAS or an RB-null BRAF mutant line. However, B-RAF and N-RAS mutant cells exhibited an enhanced response to the combinatorial agents as compared to single agents. Interestingly, we observed a dramatic increase in apoptotic cells in two cell lines, A375 (B-RAF mutant) and Sbcl2 (N-RAS) when treated simultaneously with GSK'212 and PD'991. We are currently elucidating the mechanism of response to MEK and CDK4/6 inhibition. Finally, because it is important to understand how resistance develops, we have generated a novel E2F reporter system to be utilized in vivo to quantitatively and temporally measure the efficacy of CDK4/6 inhibitors and possible re-activation of the pathway during acquired drug resistance.

Concurrent session: Genetics and Genomics of Melanoma

CS50

Functional characterisation of the Microphthalmia-Associated Transcription Factor (MITF) interactome identifies novel cofactors essential for melanoma cell growth

P. Laurette[1], T. Strub[2], D. Koludrovic[1], M. Ennen[1], G. Mengus[1], I. Davidson[1]

[1]Functional Genomics and Cancer, Institute of Genetics and Molecular and Cellular Biology (IGBMC), Illkirch, France; [2]Mount Sinai School of Medicine, New York City, NY, USA

Microphthalmia-associated transcription factor (MITF) is the master regulator determining the identity and properties of the melanocyte lineage. MITF drives melanoma cell proliferation by

© 2014 John Wiley & Sons A/S. Published by John Wiley & Sons Ltd

activating genes involved in DNA replication, mitosis, but represses genes involved in invasion. The molecular mechanisms by which MITF regulates transcription remain however poorly characterised.
By tandem affinity purification and mass spectrometry, we present a comprehensive characterisation of the MITF interactome identifying known partners such as the BRG1 chromatin-remodelling complex and multiple novel cofactors involved in transcription regulation, chromatin organisation and DNA replication and repair. We show that BRG1 is essential in melanoma cells and melanocytes in vitro and for development of mouse melanocytes in vivo as part of a specific remodeling complexe recruited by MITF to activate target gene expression. We identify the ubiquitin E3 ligase HERC2 and the ubiquitin-specific protease USP11 as novel MITF interacting proteins playing essential roles in melanoma cells and melanocytes. Comparative analysis of transcriptional regulatory processes involving MITF and his major partners provides new avenues to understand the diverse functions of MITF and its central role in melanoma cell physiology thus validating our biochemical approach coupled to functional genomics as a means of identifying novel and relevant cofactors in melanoma. Integration of these studies that we have initiated in melanoma cells with functional studies in mouse genetic models should provide a better understanding of their role in melanocyte development and melanoma.

CS51

Methylation dependent SOX9 expression mediates invasion in human melanoma cells and is a negative prognostic factor in advanced melanoma

P. F. Cheng[1], O. Shahkova[2], D. S. Widmer[1], D. Zingg[2], B. Belloni[1], M. M. Raaijmakers[1], O. M. Eichhoff[1], S. M. Goldinger[1], S. Hemmi[3], K. S. Hoek[1], L. Sommer[2], R. Dummer[1], M. P. Levesque[1]

[1]Dermatology Clinic, University Hospital Zurich; [2]Institute of Anatomy, University of Zurich; [3]Institute of Molecular Life Sciences, University of Zurich, Zurich, Switzerland

Malignant melanoma is the most fatal skin cancer with a high degree of genetic and epigenetic aberrations. To investigate the role of DNA methylation on melanoma heterogeneity, we performed methylated DNA immunoprecipitation (MeDIP) microarray analysis of 10 primary melanoma cell cultures. Integrative analysis of methylation and gene expression datasets revealed two distinct clusters. One cluster exhibited high expression of melanocytic genes like MITF, TYR, and SOX10 and the cells had a high proliferative capacity and low invasive capacity. The other cluster exhibited high expression of mesenchymal genes like SOX9, TGFB, and WNT5A and the cells had a low proliferative capacity and high invasive capacity. SOX9 was one of the targets of DNA methylation and was not expressed in proliferative melanoma cell cultures but was expressed in invasive melanoma cell cultures. Over expression of SOX9 in proliferative melanoma cell cultures increases invasion, while knockdown of SOX9 in invasive melanoma cell cultures decreases invasion. Microarray analysis of SOX9-overexpressing melanoma cells and validation with the TCGA melanoma dataset revealed enrichment in epithelial to mesenchymal transition (EMT) pathways suggesting SOX9 could be a driver of EMT in melanoma. Higher SOX9 levels were seen in patients with primary tumours and regional metastases. Survival analysis of the TCGA melanoma dataset showed that patients with high levels of SOX9 have significantly worse survival rates than patients with low levels of SOX9.

CS52

The molecular function of the long noncoding RNA SPRY4-IT1 in human melanocytes

W. Zhao[1], J. Mazar[1], B. Lee[1], J. Shelley[1], S. S. Govindarajan[1], M. E. Dinger[2], R. J. Perera[1]

[1]Sanford-Burnham Medical Research Institute, Orlando, FL, USA; [2]Garvan Institute of Medical Research, Darlinghurst, NSW, Australia

The long noncoding RNA (lncRNA) *SPRY4-IT1,* which lies within the intronic region of the *Sprouty4* gene is upregulated in human melanomas compared to melanocytes, and increases cellular proliferation and motility. siRNA knockdown of *SPRY4-IT1* blocks melanoma cell invasion and proliferation, and increases apoptosis. In order to better understand the role of *SPRY4-IT1* in human melanocytes, we ectopically expressed *SPRY4-IT1* in human melanocytes using lenti-viral stable transfection and observed increased cellular proliferation and multinucleated dendritic-like phenotype in engineered cells. Deep-sequencing and Mass Spec analysis of *SPRY4-IT1 e*xpressing melanocytes demonstrated that *SPRY4-IT1* modulates the expression of subsets of genes involved in DNA packaging, chromosome organization, chromatin architecture, DNA damage and cell cycle regulation. Notably, enrichment for chromatin-related gene expression explains multi-nucleated character of *SPRY4-IT1*-overexpressing cells, and suggests that *SPRY4-IT1* may have a role in inducing DNA replication and nuclear division. *SPRY4-IT1* ectopic expression is also results in the downregulation of the tumor suppressor gene dipeptidyl peptidase IV (DPPIV), and upregulation of the cellular proliferation marker Ki67 in engineered cells. Our results demonstrate that long noncoding RNA *SPRY4-IT1* plays an important role in melanocyte biology, and may be involved in cell proliferation and senescence. This is the first report to demonstrate the importance of a long noncoding RNA in melanocyte biology.

Khaitan D, Dinger ME, Mazar J, Crawford J, Smith MA, Mattick JS, Perera RJ. 'The melanoma-upregulated long noncoding RNA *SPRY4-IT1* modulates apoptosis and invasion'. *Cancer Res.* 2011;71(11):3852–62.
Mazar J, Zhao W, Khalil AM, Lee B, Shelley J, Govindarajan SS, Yamamoto F, Ratnam M, Aftab MN, Collins S, Finck BN, Han X, Mattick JS, Dinger ME, and Perera RJ. 'The Functional Characterization of Long Noncoding RNA SPRY4-IT1 in Human Melanoma Cells'. *Oncotarget.* 2014.

CS53

Genetic association of clinical and dermoscopic nevus patterns in a Queensland case–control study of melanoma

H. Peter Soyer[1], K. Jagirdar[1,2], N. Abbott[1], P. McClenahan[1], E. McEniery[1], D. Duffy[3], R. Sturm[1,2]

[1]Dermatology Research Centre, School of Medicine, The University of Queensland; [2]Institute for Molecular Bioscience, University of Queensland; [3]Queensland Institute of Medical Research, Brisbane, Qld, Australia

Total number of nevi is recognized as the most important risk factor for melanoma, however our understanding of this relationship for the general population is still superficial and minimal in relation to mole morphology. We have initiated a study of nevi in volunteers from Queensland to determine the

 © 2014 John Wiley & Sons A/S. Published by John Wiley & Sons Ltd

pigmentation and nevus phenotype and also the dermoscopic nevus subtypes to combine this data set with genotypic comparisons of these individuals. We aim to have 600 individuals recruited at completion of the study equally divided into 300 melanoma patients and 300 matched controls. DNA prepared from saliva samples from each volunteer has been subject to complete *MC1R* gene sequencing and SNP genotyping at a set of 31 candidate polymorphisms, including nevogenic genes *MTAP*, *PLA2G6* and *IRF4*. Analysis of the dermoscopic examination patterns of 168 melanoma cases showed that the dominant nevus pattern was reticular (69%), then nonspecific (28%), with a small minority having a globular pattern (3%). There was a significant association between the dominant dermoscopic pattern and *MC1R* genotype (P = 0.004), *MC1R* RHC allele carriers were nonspecific rather reticular in type. We have also found 6 patients in our cohort who carry the SUMOylation deficient *MITF* E318K mutation that has recently been described as a medium-penetrance melanoma gene. The phenotype of these individuals showed a commonality of fair skin, total nevi count ranged from 46 to 430, all were multiple primary melanoma patients. The frequency of globular nevi in carriers does not suggest that the *MITF* E318K mutation will be acting to force the continuous growth of nevi. There was a high incidence of amelanotic melanomas found within the group, with 3 of 5 melanomas from one patient suggesting a genetic interaction between the *MITF* E318K allele and *MC1R* RHC homozygous genotype.

Concurrent session: Melasma: New Findings, New Approaches

CS54

Prevention of melasma relapses with sunscreen combining protection against UV and short wavelengths of visible light: a prospective randomized comparative trial

F. Boukari[1], E. Jourdan[2], E. Fontas[3], H. Montaudié[1], E. Castela[1], J.-P. Lacour[1], T. Passeron[1,4]

[1]Dermatology, University hospital of Nice, Nice; [2]Research Department, Bioderma, Lyon; [3]Department for Clinical Research and Innovation, University hospital of Nice; [4]U1065 team 12, C3M, INSERM, Nice, France

Traditional sunscreens protecting only against UV do not prevent from worsening during the summer. Short wavelengths of visible light may play a role in melasma.

To evaluate the protective properties against melasma relapses of a broad UVA and UVB sunscreen containing additive filters against the shorter wavelengths of the visible light compared to a sunscreen with exactly the same protection against UVA and UVB but devoid of visible light protection.

Single-center, open-label, 1:1 randomized study in real-life settings during the summer season performed in the Department of Dermatology of Nice (South of France). After a calculation of the sample size, 40 patients with facial melasma applied tinted (Formula A) or untinted (Formula B) sunscreen over a period of 6 months. Both formulas contained the same filters against UV. In addition, Formula A contained iron oxides. The principal clinical assessments comprised the MASI score, patient questionnaire and safety. Assessments were made by 2 independent evaluators blinded to the type of cream applied. To compare differences between both evaluations, MASI scores were normalized by calculating the percentage of improvement relative to basal conditions. If the difference between the results was more than 2 points on the scale, a third assessment by a third evaluator was made.

One patient in group A was excluded as she also had post inflammatory hyperpigmentation. No patient was lost in follow-up. No statistically significant difference between both groups for the quantity of sunscreen used during the 6 months of the trial was observed. After 6 months of summer period, the MASI had increased by 0.45 (0.0–1.65) with Formula A and by 2.43 (0.45–3.68) with Formula B (P = 0.027). Eighteen patients out of 19 having used Formula A said that the use of the sunscreen limited the worsening of their melasma compared to 15 out of 20 for Formula B. Both products were safe.

The use of a specific tinted sunscreen protecting against the shorter wavelengths of the visible spectrum provides better protection against melasma relapses. For the first time, this study demonstrates the impact of visible light in the pathogenesis of melasma and fosters new fields of research for preventing melasma relapses.

CS55

Gene expression profiling in melasma in Korean women

S. Choi[1], B. Y. Chung[1], J. E. Kim[2], M. W. Lee[1], J. H. Choi[1], S. E. Chang[1]

[1]Department of Dermatology, Asan Medical Center, University of Ulsan College of Medicine; [2]Department of Dermatology, Hanyang University College of Medicine, Seoul, Republic of Korea

To date, there is a paucity of data about the difference in the degree of mRNA transcription of the genes between the malasma lesional skin and its normal adjacent one. We conducted this study to identify novel genes involved in the pathogenesis of melasma using gene expression profiling with microarray.

In this study, we performed large-scale gene expression profiling using a microarray analysis and confirmed the results on the quantitative real-time polymerase chain reaction (qRT-PCR) in five Korean women with melasma.

There were 334 genes whose degree of expression showed a significant difference between the melasma lesional skin and its normal adjacent one. Of these, five genes were confirmed on both the microarray analysis and qRT-PCR. In the melasma lesional skin as compared with its normal adjacent one, there was down-regulation (>2 fold) of genes involved in the PPAR signaling pathway [*adiponectin, C1Q and collagen domain-containing* (*ADIPOQ*)] and up-regulation of *guanine deaminase* (*GDA*) (9 fold), those involved in the functions of stratum corneum barrier [*S100 calcium-binding protein A8* (*S100A8*), *small proline-rich protein 2A* (*SPRR2A*), *small proline-rich protein 2B* (*SPRR2B*) and *kallikrein-related peptidase 6* (*KLK6*)] (>2 fold), *NAD*(*P*)*H dehydrogenase, quinine 1* (*NQO1*) (>2 fold) and those involved in the tyrosine metabolism, the activity of testosterone 17-b-dehydrogenase or the arachidonic acid metabolism.

In conclusion, our results indicate that the pathogenesis of melasma is associated with the up-regulation and down-regulation of novel genes involved in the PPAR signaling pathway, neuronal component and the functions of stratum corneum barrier in Korean women.

© 2014 John Wiley & Sons A/S. Published by John Wiley & Sons Ltd

CS56

Telangiectases in melasma: could it be a potential clinical severity marker to add treatment options

M. Hermawan, I. Bernadette, T. N. A. Jacoeb

[1]Dermatovenereology, University Indonesia, Jakarta, Indonesia

Melasma is a common acquired symmetrical hypermelanosis characterized by irregular light to dark brown macules and patches on sun-exposed areas of the skin. This disease is often found in Indonesian people, whose mostly have darker skin types. The pathogenesis of melasma is still poorly understood. Recently, interaction between skin vascularity and melanocytes has been proposed to have influence in melasma pigmentation. We investigated whether vascular factor that represented by telangiectases score, influences pigmentation severity in facial melasma skin.

A total of 48 woman with melasma were included in this cross-sectional observational study. They were selected based of consecutive sampling method. The face of each subject were examined and divided into 4 regions: forehead, left malar, right malar, and chin area. Pigmentation severity of facial melasma skin was evaluated using the pigmentation score of Melasma Area and Severity Index (MASI). Telangictases score was assessed using a validated 5-point dermoscopic scale with the aid of a hand-held non-contact polarized dermoscope. Statistical analyses were performed to assess the association between pigmentation severity and telangiectases score.

Of the women enrolled, 6.2% (n = 3/48) presented phototype III, 87.5% (n = 42/48) phototype IV, and 6.2% (n = 3/48) phototype V. Based on phenotype, 45.8% (n = 22/48) presented a centrofacial melasma, 52.1% (n = 25/48) a malar, and 2.1% (n = 1/48) a mandibular type. About 66.7% (n = 32/48) developed melasma after thirty, with mean age at onset was 32.06 yr. The overall women with family history of melasma was 45.8% (n = 22/48). Most of the women reported hormonal contraception as a triggering factor and sun exposure as an aggravating factor. Using dermoscope, we found telangiectases with various severity in 35.4% (n = 17/48) of the subjects. Of the total 192 facial areas examined, 124 (64.5%) of which have melasma, pigmentation. Of these, 29 (23.3%) pigmentation areas had telangiectases. There was significant relationship between telangiectases and pigmentation in melasma, as increased pigmentation was correlated modestly with telangiectases score ($r^2 = 0.474$, $P < 0.0001$).

This study showed that telangiectases can be found as one of clinical finding in some lesions of melasma. Increased pigmentation severity was positively associated with the presence and severity of telangiectases. It might be one of the factors that influence the severity of melasma. These results emphasize the feasibility to apply vascular-targeted therapy in addition to the main treatment in treating melasma, especially in those patients with marked telangiectases.

Concurrent session: UV & non-UV Pathways to Melanoma

CS57

Nucleotide excision repair in response to UVA is deficient in melanoma

H. C. Murray[1,2], V. E. Maltby[2,3], N. A. Bowden[1,2]

[1]Centre for Information Based Medicine, Hunter Medical Research Institute; [2]School of Biomedical Sciences and Pharmacy, Faculty of Health, University of Newcastle; [3]Department of Paediatric Endocrinology and Diabetes, John Hunter Children's Hospital, Newcastle, NSW, Australia

Despite the known causative association between UV light exposure and melanoma development, the exact mechanistic relationship remains incompletely characterised. Both UVA and UVB components of sunlight are implicated in melanomagenesis, however the majority of studies have focused on the effects of UVB and UVC light. Melanoma tumour sequencing has revealed an overrepresentation of point mutations signature of unrepaired UVA-induced DNA damage, with a particularly high mutation load in non-transcribed regions of the genome; suggestive of a lack of repair. Repair of UVA induced DNA damage occurs primarily through the Nucleotide Excision Repair (NER) pathway; yet whether this pathway retains functionality in melanoma cells is unknown. The NER pathway recognises and repairs DNA damage either coupled to transcription (Transcription-Coupled Repair; TCR), or through global genome scanning (Global Genome Repair; GGR).

One normal melanocyte cell line and four melanoma cell lines were used in this study. Cells were UVA irradiated, and DNA damage levels were determined by immunodetection of Cyclobutane Pyrimidine Dimer (CPD) and (6–4) Photoproduct (6-4PP) lesions. Expression of the NER pathway components following UVA treatment was quantified by real time PCR and western blot.

UVA did not induce detectable induction of 6-4PP lesions, consistent with previous studies. Repair of CPD lesions induced by UVA occurred majorly after 4 h in melanocytes, corresponding with the induction of GGR transcripts at 4 h following UVA exposure. In contrast, melanoma cells displayed a delayed and attenuated repair response, with the majority of repair occurring after 24 h and induction of GGR transcripts not occurring until 24 h following UVA exposure.

These findings confirm that NER activity is reduced in melanoma cells compared to normal melanocytes due to deficient GGR. These results warrant further investigation into NER and UVA in the development of melanoma, and have implications for melanoma treatment.

CS58

XPC deficiency enhances melanoma photocarcinogenesis in transgenic K5-EDN3 mice

A. P. Benaduce[1], D. Batista[1], G. Grilo[1], K. Jorge[1], D. Cardero[1], C. Milikowski[2], L. Kos[1]

[1]Department of Biological Sciences, Florida International University; [2]Department of Pathology, University of Miami, Miami, FL, USA

Ultraviolet (UV) radiation is considered to be the major etiological factor of skin cancer; it can lead to mutations in critical genes that encode for proteins related to DNA repair, apoptosis and cell cycle control. Photoproducts induced by UV radiation are, in normal cells, repaired by the nucleotide excision repair (NER)

 © 2014 John Wiley & Sons A/S. Published by John Wiley & Sons Ltd

pathway. The prominent role of NER in cancer resistance is well exemplified by patients with Xeroderma Pigmentosum (XP). This disease results from mutations in the components of the NER pathway, such as XPC. In humans, NER pathway disruption leads to the development of skin cancers, including melanoma. Similar to humans afflicted with XP, *Xpc* deficient mice show high sensitivity to UV light, leading to basal and squamous cell carcinomas development. However, *Xpc* deficient mice do not develop melanoma even upon UV exposure. There is some evidence that Endothelin receptor b (Ednrb) and its ligands Endothelin 1 (Edn1) and 3 (Edn3) pathway is also involved in melanomagenesis. In vitro data indicates activation of Ednrb by Edn1 and Edn3 in melanoma cell lines alters the expression of cell adhesion proteins and metalloproteinases that are associated with the disruption of normal host-tumor interactions and progression of cutaneous melanoma. Edn3 is also essential for proper melanocyte development and is responsible for the proliferation, survival, and migration of precursor cells. Our laboratory generated transgenic mice that over-express *Edn3* by epidermal keratinocytes throughout development (K5-*Edn3*), leading to a hyperpigmented phenotype due to the accumulation of large numbers of melanocytes in the skin of adult mice where they are not normally found. The purpose of this study was the development a UV induced melanoma mouse model that combines *Xpc* deficiency with the over-activation of the Edn3 pathway. To this end, transgenic mice over-expressing *Edn3* under the control of the keratin 5 promoter (K5-*Edn3*) and carrying a targeted mutation in *Xpc* were exposed to a single suberythemal neonatal dose of UV radiation. A subgroup of mice, at 6 weeks of age, was additionally exposed to a second dose of UV radiation. Animals were monitored weekly for skin lesion development during the period of 18 months. Histomorphology and immunostaining were used to confirm the melanocytic origin of primary skin tumors. Melanoma was only found in animals with the K5-*Edn3* transgene. Higher penetrance was observed in animals exposed to one dose of UV radiation that were *Xpc* null (33%, n = 9) when compared to *Xpc* heterozygous (17%, n = 18) or *Xpc* wild type (14%, n = 14). Melanoma development latency was reduced in the subgroup of mice exposed to a second dose of UV radiation. These results indicate that UV radiation exposure, together with over-activation of the Edn3 pathway is sufficient to lead to melanomagenesis in mice and that *Xpc* deficiency enhances its development. In the future, this model may serve as a potential tool to evaluate the efficacy of therapeutic agents.

CS59

Retinoid-X-receptors (A/B) in melanocytes modulate innate immune responses and differentially regulate cell survival following UV irradiation

A. K. Indra[1,2,3]

[1]Pharmaceutical Sciences, Dermatology, College of Pharmacy, Corvallis, Portland; [2]Dermatology, OHSU; [3]Knight Cancer Institute, Oregon Health & Science University, Portland, OR, USA

Understanding the molecular mechanisms of ultraviolet (UV) induced melanoma formation is becoming crucial with more reported cases each year. Expression of type II nuclear receptor Retinoid-X-Receptor α (RXRα) is lost during melanoma progression in humans. Here, we observed that in mice with melanocyte-specific ablation of RXRα and RXRβ, melanocytes attract fewer IFN-γ secreting immune cells than in wild-type mice following acute UVR exposure; via altered expression of several chemoattractive and chemorepulsive chemokines/cytokines. Reduced IFN-γ in the microenvironment alters UVR-induced apoptosis, and due to this the survival of surrounding dermal fibroblasts is significantly decreased in mice lacking RXRα/β. Interestingly, post-UVR survival of the melanocytes themselves is enhanced in the absence of RXRα/β. Loss of RXRs α/β specifically in the melanocytes results in an endogenous shift in homeostasis of pro- and anti-apoptotic genes in these cells and enhances their survival compared to the wild type melanocytes. Therefore RXRs modulate post-UVR survival of dermal fibroblasts in a 'non-cell autonomous' manner, underscoring their role in immune surveillance; while independently mediating post-UVR melanocyte survival in a 'cell autonomous' manner. Our results emphasize a novel immunomodulatory role of melanocytes in controlling survival of neighboring cell types besides controlling their own; and identifies RXRs as potential targets for therapy against UV induced melanoma.

CS60

The NRF2 and NOX1 mediated redox balance in UV-induced melanoma development

F. Liu-Smith[1,2,3], F. L. Meyskens[1,2,3]

[1]Medicine, University of California Irvine School of Medicine; [2]Chao Family Cancer Center, University of California Irvine School of Medicine; [3]Epidemiology, University of California Irvine School of Medicine, Irvine, CA, USA

The NADPH oxidase (NOX) family enzymes have emerged as a major generator of cellular reactive oxygen species (ROS), which are important effectors for UV radiation during melanoma development. When balanced and maintained in the appropriate range, ROS can serve as proliferation signals. The master transcriptional factor for redox balance is nuclear factor (erythroid-derived 2)-like 2, also known as *NFE2L2* or Nrf2. Both Nrf2 and Nox1 are over-expressed in all melanoma cell lines examined as well as melanoma tumor samples as compared to cultured normal human melanocytes or benign nevi, suggesting a co-evolution of oxidant and anti-oxidatant systems in tumors to maintain cell proliferation and tumor growth. Upon oxidative stress, Nrf2 binds to and activates transcription of a plethora of cellular antioxidant system including glutathione synthase and thioredoxine reductase. We found that Nrf2 also serves as a transcriptional repressor to repress Nox1 promoter activity. However, while UV induced Nrf2 protein accumulation, it also induced total NOX activity and Nox1 protein (as well as Nox1 activator Rac1). Hence during a period of time after UVR, Nrf2 and Nox1 exhibit a complex regulation against or for each other. UVA induced higher level of NOX activity than UVB in WM3211 melanoma cells and immortalized human melanocytes PIG cells, confirming that the UVA-induced NOX activity is a major cause for UVA-induced oxidative stress. Rac1 signal is also dramatically enhanced by both UVA and UVB (examined by immunofluorescence) in PIG cells, confirming recent discovery of Rac1 as a melanoma oncogene. Taken together Nox1 and Nrf2 seem to play antagonistic roles to maintain a redox balance after UVR. Only when this balance is disrupted (e.g., the stress signal is too strong to push cells to high oxidative status), melanoma may occur. Targeting the NOX enzymes using specific NOX inhibitors may be effective for prevention of melanoma.

© 2014 John Wiley & Sons A/S. Published by John Wiley & Sons Ltd

Concurrent session: Developmental Biology & Melanoma Animal Models

CS61

A CRISPR/CAS9-based Modular murine melanoma model to study determinants of multimodal immunotherapeutic regimens

N. Glodde[1], D.van den Boorn-Konijnenberg[2], T. Bald[1], T. Tüeting[1], M. Hoelzel[2]
[1]Experimental Dermatology, University Hospital Bonn; [2]Institute of Clinical Chemistry and Clinical Pharmacology, University Hospital Bonn, Bonn, Germany

Metastatic malignant melanoma is a highly aggressive and chemoresistant skin cancer with poor clinical outcome. Significant breakthroughs in the treatment of this devastating disease have been made in the recent years. Small molecule inhibitors against the mutated BRAF or MEK kinases and antibody-mediated blockade of negative immune checkpoint molecules like CTLA-4 and PD1/PD-L1 prolong overall survival rates, but acquired resistance to these targeted therapies is the major obstacle for long-term remissions despite profound initial responses. Hence, malignant melanoma is a paradigm disease to exploit the synergisms of combined tumor biological and tumor immunological multimodal treatment approaches to achieve durable responses. Recent studies including ours identified diverse mechanisms of resistance to signaling inhibitors and immunotherapies in melanoma. We found that phenotypic plasticity of melanoma cells caused by a proinflammatory tumor microenvironment represents a critical route to resist a targeted T-cell immunotherapy directed against melanocytic antigens through reversible dedifferentiation without the need for acquired secondary hardwired genetic aberrations (Landsberg, ..., Hölzel, Tüting. *Nature* 2012). Therefore we conceptualized scenarios how genetic and non-genetic sources of tumor heterogeneity could account for therapy resistance in part through reciprocal interactions (Hölzel, Bovier and Tüting. Nat Rev Cancer 2013). To address this emerging field in therapy resistance we exploited MET tyrosine kinase oncogene addiction in our HgfxCdk4R24C murine melanoma model and implemented CRISPR/Cas9 genome editing to establish a rapid pipeline to probe clinically relevant determinants of responsiveness to multimodal immunotherapeutic regimens.

CS62

HSP70i$_{Q435A}$ mediates anti-melanoma responses in mice

J. Mahon[1], J. Mosenson[1], J. O'Sullivan[1], J. Eby[1], M. Nishimura[1], S. Mehrotra[2], J. Guevara-Patino[1], C. Le Poole[1]
[1]Oncology Research Institute, Loyola University, Chicago, IL; [2]Oncologic and Endocrine Surgery, Medical University of South Carolina, Charleston, SC, USA

Modified inducible HSP70 (HSP70i$_{Q435A}$) has been shown to protect TCR transgenic mice against T cell-mediated depigmentation in a mouse model of vitiligo. Bringing this development to the clinic involves demonstrating a favorable risk to benefit ratio, in that HSP70i$_{Q435A}$ may impair more beneficial immune responses as well. We assessed whether anti-melanoma responses remain intact following HSP70i$_{Q435A}$ application. Splenocytes from vitiligo-prone mice gene gun vaccinated with HSP70i isoforms were adoptively transferred to tumor challenged, immunedeficient recipients, measuring tumor growth and T cell persistence. SCID/beige mice showed persistence of adoptively transferred, tyrosinase reactive T cells for >6 weeks following transfer, and tumor growth was inhibited particularly in response to mutant HSP70i. We also vaccinated C57BL/6 mice measuring B16 tumor growth, and assessed tumor immune infiltrates. In C57BL/6 mice, exposure to the HSP70i molecule alone was sufficient to induce significant anti-tumor responses that were superior in HSP70i$_{Q435A}$ treated mice, showing a 38% decrease in average tumor mass following vaccination. Vaccinated mice also displayed marked humoral anti-tumor responses to HSP70i. Next, mice knockouts for CD4 or CD8 co-receptors were vaccinated with DNA encoding HSP70i isoforms with or without TRP-2 encoding DNA followed by B16 tumor challenge. Tumors grew unopposed in CD4 knockout mice and in CD8 knockout animals, mutant HSP70i vaccination provided superior anti-tumor protection over the unadulterated molecule. Finally, we sorted DCs, B cells and T cells from vaccinated C57BL/6 mice and introduced them to tumor bearing hosts. Only adoptive B cell transfer from HSP70i$_{Q435A}$ vaccinated mice conveyed protection from tumor growth. These data strongly suggest that besides the therapeutic efficacy towards vitiligo, vaccination with HSP70i$_{Q435A}$ offers anti-melanoma protection in mice.

CS63

Oxylipin metabolomics study of melanoma brain metastasis and the drug effect of liposomal doxorubicin in syngeneic mice

C.-C. Yang[1], L.-F. Shyur[2]
[1]Ph.D. Program for Cancer Biology and Drug Discovery, College of Medical Science, Taipei Medical University and Academia Sinica; [2]Agricultural Biotechnology Research Center, Academia Sinica, Taipei, Taiwan ROC

Brain metastases (BM) are the most common brain tumors diagnosed in adults, and melanoma represents the third most common brain metastatic tumors and the life span of patients is often measured in months. Current conventional therapies for BM include surgical operation, radiotherapy, chemotherapy, and biochemical target agent, but the outcome remains poor. Pegylated liposomal doxorubicin (Lipo-Dox) has been proved with long term stabilization in recurrent high-grade glioma patients in phase II clinical trial; however, whether Lipo-Dox is effective to melanoma BM (MBM) is not clear. In this study, we established an allograft MBM mouse model using intracarotid injection of B16 cell carrying a COX-2 promoter driven-luciferase reporter gene (B16$^{COX\text{-}Lux}$) to evaluate in vivo drug efficacy of Lipo-Dox. The metastatic melanoma tumors were successfully observed in mice brains detected by bioluminescence imaging system, which was significantly reduced in mice treated with *i.v.* intervention of Lipo-DOX. Tumor growth factor β (TGF-β), a marker involved in promoting melanogenesis and cancer invasion ability was found up-regulated in microglia (Iba-1+ cells) in mouse brain with melanoma metastasis. Oxylipins, by acting as small molecule lipid mediators, play a central role in inflammation and are implied in cancer. We have established oxylipin metabolomics platform using UPLC/TSQ triple-quadrupole mass spectrometry hyphenated partial least squares discriminant analysis (PLS-DA), to investigate the role of oxylipins involved in BMB in mice. The effect of Lip-Dox on several biomarkers related to brain metastasis of melanoma and on modulating specific oxylipins were evaluated.

 © 2014 John Wiley & Sons A/S. Published by John Wiley & Sons Ltd

CS64

Investigating the role of the MITF-BRN2 expression axis in metastatic melanoma

M. Fane, A. G. Smith
SBMS, University of Queensland, Brisbane, Qld, Australia

Metastatic melanoma is a complex and heterogeneous disease, which is highly resistant to conventional chemotherapies and radiation. Recent development of small molecular weight inhibitors for the mutant BRAF kinase (present in approximately 50% of melanomas), induce dramatic tumour regression in many patients, however tumour resistance to these agents develops in the majority of patients within 6 to 12 months. Accordingly, a deeper understanding of the cellular programs that underpin melanoma growth, invasion and metastasis is required to identify novel targets to treat this disease. The transition from radial to vertical growth phase is a pivotal event associated with the metastatic transformation of melanoma cells. The basis of the molecular switch that occurs and confers the ability of tumour cells to migrate is unknown, however accumulating evidence suggests that these cells undergo epigenetic changes driven by specific expression programmes imposed by the cellular microenvironment that allows switching between a proliferative or invasive phenotype. Recent findings have now shown that both in vitro and in vivo models of metastatic melanoma contain two distinct subpopulations characterised by an inverse expression profile between the MITF and BRN2 transcription factors. Importantly, intravital imaging in real time in vivo tumours has revealed that BRN2 expressing populations are highly motile and capable of exiting the primary tumour and entering the vasculature, however resulting lung metastases are primarily made up of MITF expressing populations, thus implicating the importance of phenotype switching and the MITF-BRN2 expression axis in metastasis. While BRN2 clearly plays a role in increasing invasiveness and tumorigenicity of tumour cell populations, there is very little known about downstream signalling targets that BRN2 regulates to allow such a phenotype. Using both BRN2 gain and loss of function models in-vitro, we have found that BRN2 regulates NOTCH, IRF4, and NFIB signalling, all of which may play a role in increasing the migratory and tumorigenic potential of these BRN2 expressing populations. We have also developed a genetic model of invasive melanoma in zebra fish using transient melanocyte specific expression of oncogenic H-RAS in which nodular tumours are only seen in combination with BRN2 over-expression. Current efforts are aimed at identifying BRN2 downstream effector genes with the hope of providing novel therapeutic targets against cellular populations capable of initiating metastasis.

Concurrent session: Pigmenary Challenges in Skin of Colour

CS65

Lichenoid photocontact dermatitis versus lichen planus pigmentosus: a diagnostic dilemma in Asian skin

A. Kaur, M. S. Kumaran, D. De, D. Parsad, S. Handa
Dermatology, PGIMER, Chandigarh, India

Lichen planus pigmentosus (LPP) is a fairly common cause of facial melanosis in Asians. A definite etiology is still ambiguous, although paraphenylenediamine (PPD) was suggested as a precipitating factor at the time of its origin. Most studies have not further investigated its role and largely considered LPP as just a variant of lichen planus. The aim of this study was to evaluate the role of photocontact dermatitis with PPD in causing lichenoid facial pigmentation simulating LPP.
Patients presenting between June 2013 and December 2013, with history suggestive of contact dermatitis to hair dye and subsequently demonstrating facial lichenoid pigmentation clinically mimicking LPP, were assessed. A complete history, clinical examination, skin biopsy and photopatch test with Indian standard series and 'as is' with patient's own products were performed in all patients.
Among 25 patients screened during this period, seven patients presented to us with shades of dermal pigmentation varying from slate grey to blue-black. Face and neck were the commonest affected areas, predominately involving the frontal hairline, temporal areas and nape of neck, in accordance with the photo-distributed pattern. Severity of prior eczematous phase varied from subtle pruritus to frank vesiculation and its onset prior to pigmentation varied from as early as 6 weeks to 8 yr.
Histopathological examination revealed necrotic keratinocytes and spongiosis in the epidermis, with basal layer degeneration. Dermis showed pigment incontinence and a perivascular lymphocytic infiltrate, thereby confirming the diagnosis of lichenoid dermatitis in all 7 patients. Photopatch test showed photoaggravation with PPD at 96 h in four patients, only contact allergy to PPD in 2 patients and 1 patient demonstrated blue-black pigmentation at PPD test site at 4 weeks, which was clinically and histologically similar to the affected area. All patients were managed with topical calcineurin inhibitors and sunscreens with discontinuation of hair dye. The progression of the disease was halted, although pigmentation showed only mild improvement at 1 yr follow-up. Q-switched Nd-YAG laser (1064 nm) was performed in two patients at monthly intervals, which showed good improvement at 1 yr follow up.
Our findings clearly indicate hair dye induced photocontact dermatitis as a causative factor for lichenoid facial pigmentation, clinically mimicking LPP. A modified photopatch testing with delayed readings might enlighten the pathogenesis of this facial melanosis. Whether lichenoid photocontact dermatitis represents a separate entity or PPD seems to trigger this autoimmune disease needs to be further evaluated. Differentiating the two conditions holds a significant implication in the treatment of this recalcitrant hyperpigmentary disorder.

CS66

Erythema dyschromicum perstans versus lichen planus pigmentosus: a comparative clinico-histopathological study

S. AminPoojary, M. Khoja
Department of Dermatology, K.J. Somaiya Medical College, Mumbai, Mumbai, India

Erythema dyschromicum perstans and lichen planus pigmentosus are dermal pigmentary disorders with several overlapping clinical and histopathological features. This has led to several queries; Are they part of the same spectrum of dermal melanosis? Or are they entirely different entities? To answer these questions, it is essential to carefully delineate their clinical and histological features.
To define and differentiate the clinical and histological features of Erythema dyschromicum perstans (EDP) and lichen planus pigmentosus (LPP)

© 2014 John Wiley & Sons A/S. Published by John Wiley & Sons Ltd

Materials and methodsAn observational comparative prospective study was performed on 29 patients (20 patients of lichen planus pigmentosus and 9 patients of Erythema dyschromicum perstans). The clinical features and histopathological findings of these patients were analysed and compared.
There was a female preponderance in both LPP and EDP. The average age of onset was higher in LPP as compared to EDP.
Patchy pattern of distribution was predominant in both LPP (85%) and EDP (100%). However, EDP was characterised by well defined oval brownish- black to slate grey macules, while in LPP, the patches were irregular and tended to coalesce. Other patterns including diffuse, perifollicular, linear and reticulate patterns were also observed in LPP, but were absent in EDP. Patients with LPP had associated symptoms such as pruritus and photosensitivity, while EDP was largely asymptomatic. Coexisting lichen planus lesions were observed in only 2 patients with LPP. None of the patients with EDP showed an erythematous border around the patches.
Histologically epidermal atrophy, basal cell vacuolisation, dermal melanophages and superficial perivascular lymphocytic infiltrate were common to both, while lichenoid infiltrate was observed only in LPP.
A few unusual features, hitherto not commonly described were observed: increased basal melanin in both LPP and EDP. Fifty percentage of patients with LPP showed significant perifollicular infiltrate and follicular basal cell vacuolisation. Also acanthosis was observed in 4 patients of LPP instead of the commonly observed feature of epidermal atrophy
This study delineates the significant clinical and histopathological differences of LPP and EDP, thereby differentiating them into distinct entities. This study also brings forth a few unusual histopathological features of LPP and EDP.

CS67

Reticulate acropigmentation of Kitamura and Dowling-Degos Disease are genetically independent disorders distinct from each other; further confirmation

M. Kono[1], K. Sugiura[1], M. Suganuma[1], M. Hayashi[2], H. Takama[3], T. Suzuki[2], K. Matsunaga[4], Y. Tomita[1], M. Akiyama[1]
[1]Dermatology, Nagoya University Graduate School of Medicine, Nagoya; [2]Dermatology, Yamagata University School of Medicine, Yamagata; [3]Takama Dermatology Clinic, Kasugai; [4]Dermatology, Fujita Health University School of Medicine, Toyoake, Japan

Reticulate acropigmentation of Kitamura (RAK) [MIM# 615537] is a rare genetic disorder of cutaneous pigmentation with an autosomal dominant pattern of inheritance and a high penetration rate, which was first reported in Japanese by Kitamura and Akamatsu in 1943 and described in English by Kitamura et al. in 1953. The characteristic skin lesions are reticulate, slightly depressed brown macules mainly affecting the dorsa of the hands and feet, which first appear before puberty and subsequently expand to the proximal limb and the trunk. On the other hand, Dowling-Degos disease (DDD) show the similar skin manifestation of reticulate, slightly depressed brown macules on flexore region. There is controversy over whether RAK and DDD are two distinct clinical entities or just on a spectrum of a single disease. We have reported that *ADAM10* mutations caused RAK in 2013 (Kono et al, Hum Mol Genet 2013). In our previous report, we performed exome sequencing of 4 family members in a Japanese pedigree with RAK. Fifty-three SNV/Indels were considered as candidate mutations after some condition narrowing. We confirmed the mutation status in each candidate gene of 4 other members in the same pedigree to find the gene that matched the mutation status and phenotype of each member. A heterozygous mutation, [c.415C>T + c.424–425insCAGAG] (p.Pro139Ser + p.Arg142fsX43) in *ADAM10* encoding a zinc metalloprotease, a disintegrin and metalloprotease domain-containing protein 10 (ADAM10) was identified in the RAK family. ADAM10 is known to be involved in the ectodomain shedding of various substrates in the skin. Sanger sequencing of *ADAM10* in five more patients from four unrelated Japanese families identified four different mutations, including a nonsense mutation c.429T>A (p.Tyr143X), an 1-bp deletion mutation c.1264delA (p.Thr422fsX19) and a splice site mutation c.1511G>A as well as a missense mutation c.1571G>A (p.Cys524Tyr). For these data, we concluded that mutations in *ADAM10* are a cause of RAK. We searched for mutations in the *KRT5* gene, a causative gene for the similar pigmentation disorder DDD, in all the patients and found no *KRT5* mutation. Recently *POFUT1* gene was reported as another causative gene of Dowling-Degos disease. Thus we performed mutation search for *KRT5* and *POFUT1* in one DDD patient we have experienced. In this paper, we report that a *POFUT1* mutation but no *ADAM10* mutation was detected in the patient, which makes further confirmation that RAK and DDD are genetically independent disorders distinct from each other.

Concurrent session: Hair Biology & Pigmentation

CS68

Distinct developmental potentials of melanocyte progenitors from the hair follicle bulge and secondary hair germ

S. S. Joshi[1], J. M. Huang[1], T. J. Hornyak[2,3]
[1]Department of Biochemistry & Molecular Biology, University of Maryland School of Medicine; [2]Department of Dermatology and Biochemistry & Molecular Biology, University of Maryland School of Medicine; [3]VA Maryland Health Care System, Baltimore, MA, USA

Melanocyte stem cells (MSCs) are the relatively undifferentiated melanocytic cells of the mammalian hair follicle (HF) responsible for recurrent generation of a large number of differentiated melanocytes during each HF cycle. In previous work, we have shown that HF MSCs residing in the bulge/lower permanent portion (LPP) and the secondary hair germ (SHG) can be distinguished by their mutually exclusive expression of the membrane proteins CD34 and Cdh3/P-cadherin, respectively.
Using *Dct*-H2BGFP mice, we can separate bulge/LPP and SHG MSCs using FACS with GFP and anti-CD34. We find that CD34+GFP+ bulge/LPP cells express high levels of *Cd34*, whereas CD34-GFP+ SHG cells express high levels of *Cdh3*, using quantitative RT-PCR, confirming that their expression in the HF environment is transcriptionally regulated. Melanocyte differentiation genes *Tyr*, *Tyrp1*, and *Pmel17* are expressed more highly in CD34-GFP+ SHG cells, whereas growth and marker analysis of cultured CD34+GFP+ bulge/LPP cells suggest that they possess an expanded developmental potential compared to CD34−GFP+ MSCs. CD34+GFP+ bulge/LPP MSCs express markers of other neural crest derivatives such as glia, neurons, and myofibroblasts in neural crest cell differentiation condition whereas CD34-GFP+ MSCs do not. However, cultured in melanocyte medium, only CD34−GFP+ cells demonstrate visible melanization. Consistent with the

 © 2014 John Wiley & Sons A/S. Published by John Wiley & Sons Ltd

notion that CD34+GFP+ bulge cells represent a more primitive and multipotent subset of MSCs, these cells exhibit the ability to express myelin basic protein in a neuronal distribution when co-cultured with dorsal root ganglion explants. The use of CD34 to separate MSCs into distinct subsets should continue to be helpful to expand understanding of their functional properties.

CS69

Pigmentation of the regenerated hair follicles after wounding

M. Yuriguchi, H. Aoki, N. Taguchi, T. Kunisada
Gifu university graduate school of medicine, Gifu, Japan

Hair follicle stem cells and melanocyte stem cells share their niche at the lower permanent portion of the bulge to regenerate pigmented hairs during in close coordination with hair cycle. However, recently observed hair regeneration after large wound healing is lacking pigments in the hair keratinocytes, suggesting the loss of coordination with these two stem cells during the wound healing associated hair regeneration process. As reported facts, regenerating hair follicles after wounding are likely to be started from keratinocyte stem cells migrated from inter follicular epidermis and melanocyte stem cells usually do not present in the inter follicular epidermis but immigrate to inter follicular epidermis from the hair bulge after the wounding. These two phenomena were reported to be related with the activation of Wnt signaling in hair follicle stem cells and melanocyte stem cells, as also reported on hair regeneration during normal hair cycles, however, the reason why only the unpigmented hairs are regenerated during wound healing process is still not known. Here, we show that regeneration of the pigmented hairs occur after wounding the skin but depends on the timing of wound execution during the hair cycle. We found that anagen stage is the requisite for the secure regeneration of pigmented hairs and the significant increase of Wnt mRNA expression was confirmed at the anagen skin compared with the telogen. As expected, mice containing activated melanocyte stem cells in their epidermal skin were easy to regenerate pigmented hairs even after wounding executed during the anagen skin. Based on these findings, we propose that bulge melanocyte stem cells are likely to be stimulated by the Wnt signaling and melanocyte stem cells migrated to inter follicular epidermis are necessary to develop fully pigmented hairs in the regenerating hair follicle after wounding. Our data provide a new insight to the intimate regulation process between two stem cell systems, hair follicle stem cell and melanocyte stem cell during de novo hair regeneration after wounding.

CS70

Premature graying of hair- paving the way to treatment

V. Mendiratta, S. Rana, A. rao, R. chander
Dermatology, lady Harginge Medical College, New Delhi, India

Premature graying of hair is an enigmatic condition whose exact etiology and treatment are unknown. It is also suggested that hair graying is a risk marker for coronary artery disease independent of age and other traditional risk factors. Premature graying is an important cause of low self-esteem, often interfering with socio-cultural adjustment. The onset and progression of graying or canities is intimately related with chronological aging, and occurs in varying degrees in all individuals eventually, regardless of gender or race. Premature canities may occur alone as an autosomal dominant condition or in association with various autoimmune or premature aging syndromes.. Reduction in melanogenically active melanocytes in the hair bulb of gray anagen hair follicles with resultant pigment loss is central to the pathogenesis of graying. Defective melanosomal transfers to cortical keratinocytes and melanin incontinence due to melanocyte degeneration are also believed to contribute to this. Graying of hair is usually progressive and permanent, but there are occasional reports of spontaneous repigmentation of gray hair. Recently oxidative stress and cytokine mediated injury have been proposed to play a role in its causation.

Study DesignCross sectional study

MethodologyFifty patients who complained of early graying of hair and diagnosed as premature graying i.e (before 25 yr of age) were taken up for the study. Patients belonged to either sex. Demographic details, clinical history for age of onset, family history, associated complaints like vitiligo, atopic dermatitis, symptoms suggestive of malabsorption, site of scalp involvement were recorded. Investigations were done to evaluate hemoglobin, serum ferritin, vitamin B12. Thyroid function tests, blood sugar estimation, and antinuclear antibody assay were also done. Results were pooled and analyzed.

ObservationsThere were 20 (40%) males and 30 (60%) females The mean age of patients was 8.5 yr. Majority of patients (34%) were between 14 and 20 yr of age. Thirty-two percentage had onset before 6 yr. Family history was positive in 20%. Frontal scalp was the commonest involved area. Associated conditions were – Anemia 12 (24%), Vitligo 4 (8%), Vitamin B12 deficiency in 9 (18%), atopic dermatitis 6 (12%), low serum ferritin in 6 (12%), thyroid dysfunction in 4 (8%), diabetes mellitus in 2 (4%) and malabsorption in 3 (6%) patients respectively. Antinuclear antibody test was negative in all.

Premature graying is a multifactorial condition. It may be autosomal dominant and seen in family members. Role of nutritional factors like serum ferritin, vitamin B12 and micronutrients needs further study in order to work upon management of this progressive condition. Premature graying of hair is not only a cosmetic concern but can also be a surrogate marker of underlying nutritional deficiencies, autoimmune diseases and coronary artery disease. Correction of modifiable factors may help in its management.

Concurrent session: Colourful Skin Optics

CS71

In vivo 3-D quantification of melanin in human skin through multiphoton microscopy and image processing

T. Baldeweck[1], E. Decenciere[2], S. Brizion[1], S. Koudoro[2], E. Tancrède-Bohin[1,3], A.-M. Pena[1]
[1]Research and Innovation, L'Oréal, Aulnay Sous Bois; [2]Centre de Morphologie Mathématique – Mathématiques et Systèmes, MINES ParisTech, Fontainebleau; [3]Department of Dermatology, St Louis Hospital, Paris, France

The amount and distribution of cutaneous melanin are important criteria in both medical and cosmetic applications. These parameters are usually recorded through 2D white light imaging associated with histological staining, a procedure that is inappropriate for in vivo applications.

An alternative approach consists in using multiphoton microscopy that offers the possibility to image the skin in vivo in a non invasive way [1]. For example, the melanin present in the epidermal basal layer is often highly concentrated, leading to a

© 2014 John Wiley & Sons A/S. Published by John Wiley & Sons Ltd

fluorescence intensity stronger than that emitted by other endogenous fluorophores. However, methods based upon fluorescence intensity are not always satisfactory since i) the signal may be disrupted by other fluorophores of a strong fluorescence intensity (e.g. keratin in the *stratum corneum*), and ii) it does not take into account pixels with low melanin concentration with a fluorescence intensity comparable to that of other endogenous fluorophores.

A more specific method takes into account the melanin fluorescence lifetime (Fluorescent Life Time Imaging, FLIM). However, classical FLIM imaging is a too long process for obtaining 3D data, and is in practice limited to selected 2D slices, obtained at a depth chosen by the operator.

In the present work, we propose a new quantitative method based on 'reduced' FLIM data acquisition (4 time channels) for each pixel of the 3D-image. The acquisition time of this 'Fast-FLIM' protocol is compatible with in vivo investigations. Using image processing methods including a 3D automatic segmentation method [2], quantitative parameters such as the global 3D melanin content in the whole epidermis can be extracted. In addition, normalized layers within the 3D-delimited epidermis are defined in depth and a specific algorithm leads to the normalized profile of melanin as a function of normalized depth, from the Dermal Epidermal Junction (DEJ) up to the *stratum corneum* (skin surface).

This new method was applied in a clinical trial on 15 Caucasian women (aged 70–75y) focusing on the ventral and dorsal sides of their forearms, and recorded data were compared to histological results. Additionally, results obtained from a clinical study including 45 women (18–55 yr) with ITA grade values ranging I to V on the ventral side of the forearm will be shown.

In conclusion, the method based upon multiphoton microscopy coupled with Fast-FLIM seems a promising strategy for detecting and quantifying the 3D distribution of melanin within the human skin in vivo.

1. H. Ait El Madani, E. Tancrède-Bohin, A. Bensussan, A. Colonna, A. Dupuy, M. Bagot, and A.-M. Pena, 'In vivo multiphoton imaging of human skin: assessment of topical corticosteroid-induced epidermis atrophy and depigmentation', *Journal of Biomedical Optics* 17(2), 026009-1-026009-8 (2012).

2. E. Decencière, E. Tancrède-Bohin, P. Dokladal, S. Koudoro, A. M. Pena, and T. Baldeweck, 'Automatic 3D segmentation of multiphoton images: A key step for the quantification of human skin', *Skin Research and Technology* 19, 115–124 (2013).

CS72

The use of transgenic reporter mice and intravital multiphoton microscopy to study mast cell development in vivo

P. L. Tong[1,2,3], B. Roediger[1], R. Jain[1], W. Weninger[1,2,3]

[1]Immune Imaging, Centenary Institute, Newtown; [2]Discipline of Dermatology, University of Sydney; [3]Department of Dermatology, Royal Prince Alfred Hospital, Camperdown, NSW, Australia

Mast cells develop from a bone marrow-derived population of the myeloid lineage and are distributed strategically at environmental interfaces including the dermis of the skin whereby they are thought to play a critical role in the innate immune response to a range of pathogens in particular parasites. They are however also implicated in inflammatory and neoplastic conditions whereby mast cell numbers are increased in the skin. The mechanisms underlying this accumulation of mast cells is unclear and specifically, it is unknown to what extent local or reactive mastocytosis is mediated by in situ mast cell proliferation versus recruitment of mast cell progenitors. We have developed a novel imaging and flow cytometry-based platform that allows the characterization of the dynamics of mast cell proliferation within the skin at the single cell level in vivo. Using *Kit*-eGFP mast cell reporter mice, we have quantified the spatio-temporal proliferation of connective tissue mast cell re-population in ear skin following inflammation. While skin mast cells are self-renewing and radioresistant during steady-state, mast cell re-population following inflammation was mediated by bone marrow-derived progenitors. Following their differentiation, these donor-derived mast cells proliferated within the skin and mast cell density was restored to that of wild type levels within 6 weeks. We propose a model of homing of inflammation-responsive mast cell progenitor recruitment that has implications for the pathogenesis of cutaneous mastocytosis.

CS73

Linking skin physiology measurements to skin color by a numerical skin color model

P. Bhardwaj[1], S. Brizion[1], E. Tancrede-Bohin[1,2], A. Potter[1], T. Baldeweck[1], A.-M. Pena[1], E. Huguet[1]

[1]Research and Innovation, L'Oréal, Aulnay Sous Bois; [2]Department of Dermatology, St Louis Hospital, Paris, France

One current challenge in cosmetic science deals with understanding the link(s) between skin physiology and skin color, for which several numerical skin color models have been previously developed. However, their accuracy and precision are limited due to the simplified description of the skin structure or its optical properties used in these models.

The present work aimed at improving the existing numerical skin color models by studying how physiological alterations of the skin may impact its color. Accordingly, we developed a new model that uses a more detailed skin description based upon experimental in vitro and in vivo measurements as well as literature data. The data included in the model were the following:

1. Epidermal thickness measured in vivo on cheek and forearm by Optical Coherence Tomography (OCT) [1];
2. Information about skin morphology obtained in vivo on forearm (thickness of different epidermal layers, undulation of the dermal-epidermal junction), as well as the density and distribution of melanin in each epidermal layer by multiphoton microscopy [2,3];
3. Published data on optical properties of melanin and hemoglobin;
4. Experimental light diffusion spectra in the visible range measured in vitro on reconstructed skin samples;
5. Colorimetric data (diffused reflectance spectra obtained by Chromasphere®) measured in vivo on cheeks of 500 women of different ethnic origins (Caucasian, Afro-American, Asian and Hispanic), and different skin color (i.e. Individual Typological Angle (ITA) grade value ranging from II-*light skin* to VI-*very dark skin*).

Our model used standard laws of light propagation in skin to estimate in vivo skin color [4] and optical properties of each skin layer described by absorption spectra (obtained from literature) and diffusion spectra (measured in vitro). Due to the lack of experimental data on cheek for melanin distribution, we made

 © 2014 John Wiley & Sons A/S. Published by John Wiley & Sons Ltd

the hypothesis that this melanin distribution is the same for cheek and forearm, but we took into account the thickness variation thanks to the OCT measurements.
Our results show that this improved numerical skin color model reproduces with great precision the diversity of skin colors as measured in vivo while integrating all major physiological constraints. This model was applied to test the hypothesis of partial elimination of melanin within the *stratum corneum* and to visualize the resultant skin color. The results are in agreement with the experimental multiphoton microscopy and colorimetric data.
In conclusion, we propose a new, highly reliable numerical skin color model that could be helpful in studying skin color changes possibly induced by physiological alterations related to various factors such as UV, disease or treatment, and predicting the impact of pharmaceutical or cosmetic products on skin color.

1. Querleux et al. 'Skin from various ethnic origins and aging: an in vivo cross-sectional multimodality imaging study', *Skin Res Technol*, 15, 306–313 (2009).
2. Ait El Madani et al., 'In vivo multiphoton imaging of human skin: assessment of topical corticosteroid-induced epidermis atrophy and depigmentation', *J Biomed Opt* 17(2), 026009-1-026009-8 (2012)
3. Decencière, et al., 'Automatic 3D segmentation of multiphoton images: A key step for the quantification of human skin', *Skin Res Technol* 19, 115–124 (2013).
4. Yang et al., 'Revised Kubelka–Munk theory. I. Theory and application', *J Op. Soc Am A* 21, 1933–1941 (2004).

CS74

Automated scoring of melasma using computerized digital image analysis of clinical photographs. a pilot study

Y. Liang[1], Z. Lin[1], W. Ser[1], F. Lin[1], E. Gan[2], E. Tay[2], S. T. G. Thng[2]
[1]Nanyang Technological University; [2]National Skin Centre, Singapore City, Singapore

Melasma is a common and persistent disorder of hyperpigmentation, affecting up to 40% of the Southeast Asian population. Objective quantitative outcome measures for melasma severity are essential for the evaluation of results of treatment. The Melasma Area and Severity Index (MASI) and modified MASI score are currently the only two reliable and validated scoring system for melasma severity. However, individual components of the mMASI are still problematic, requiring doctors to estimate the percentage areas involved as well as the darkness component. This estimation of percentage area involved as well as degree of darkness is subjected to human error and usually results in inter and intra-assessor variability resulting in inconsistent scoring for melasma.
The aim of this study is to develop and validate an algorithm for automated calculation of mMASI using computer image analysis of whole-face photographs, so as to enable clinicians around the globe to use this tool to standardize melasma severity scoring and outcome measurements in an easy and reproducible manner, with little or minimal inter-assessor variability.
MethodologyThere are 2 phases in this study. The first is the design and development of an algorithm for digital image analysis. The development of the proposed scoring algorithm involved three main steps:

1. Image segmentation, using a combined method of adaptive and global threshold segmentation;
2. Calculation of statistics of regions affected by melasma (foreground) and other regions (background), including the areas and darkness of the skin in each region;
3. Calculation of scores based on computer-generated statistics with reference to mMASI.

The second phase is the validation of the developed algorithm. Twenty four patients with melasma were recruited into the study after the creation of the software. They were reviewed at baseline and week 12 with clinical assessment by independent dermatologist and whole-face standardized digital photographs using VISIA system. The clinical mMASI scores from the clinical assessment by dermatologist were then compared with the digital scores derived via computer image analysis. A sample size of 24 was selected to detect a correlation of 0.6 between two sets of scores at 5% significance level with a power of 90%.
The digitally derived mMASI score from computer image analysis showed good reliability and correlated well with the clinical mMASI scored by independent dermatologist assessing the patients. There were some specific problems in dark skin patients but overall, the developed algorithm proved to be a reliable measure of treatment outcomes in melasma patients.
This developed algorithm proposes a novel image segmentation approach by combining adaptive and global tresholding methods in melasma digital image analysis. It is simple, easy to use, and the algorithm can be downloaded via internet to analyse any standardised digital image. With further fine-tuning, this developed algorithm for scoring of melasma severity via digital image analysis shows good promise as there is no need for training and the algorithm will generate consistent mMASI scores all the time with no inter-assessor variability. This algorithm would enable clinicians around the globe to standardize melasma severity scoring and outcome measurements in an easy and reproducible manner.

CS75

The role of reflectance confocal microscopy in the evaluation of hypopigmented skin disorders

H. Liu, F. Zhang
Shandong Provincial Institute of Dermatology and Venereology, Jinan, People's Republic of China

The hypopigmented skin disorders are very common in dermatology clinics, among which the propotion of vitiligo is just about 60 percent according to the Japanese guidelins of vitiligo. Sometimes the differential diagnosis is difficult to make just by naked eyes, and the compliance of histology analysis is decreasing owing to its invasive nature. The imaging modalities are essential in the diagnosis and evaluation of those skin disorders.
The reflectance confocal microscopy(RCM) could image the epidermis and superficial dermis in cellular leval resolution in its natural conditions, which provides the dermatology clinic a useful tool in the evaluation of skin disorders.
We evaluated the hypopigmented skin disorders with RCM by comparing the melanin changes of the lesion and perilesional normal skin, or by folowing up the melanin changes of the same lesion in different stages, and in some conditions we compare

© 2014 John Wiley & Sons A/S. Published by John Wiley & Sons Ltd

the RCM imaging with histology analysis results. Based on the imaging and following up results of thousands of cases every year from 2006 to 2013, we have some certain findings with RCM: (1)The RCM could differentiate the vitiligo and other hypopigmented skin disoders based on the melanin changes in basal cell layer; (2) The RCM could distinguish whether the vitligo lesion condition were progressive or stable by comparing the pigment ring of the lesional margin; (3) The RCM could observe the melanin changes of the same lesion by imaging in different stages, which is very important in melanin evaluation and follow-up of vitiligo treatment.

CS76

Dermoscopy – a new tool to predict stability in vitiligo

N. khunger, P. Singh

Dermatology and STD, VMMC and Safdarjung Hospital, New Delhi, India

To characterize dermoscopic features of stable and unstable vitiligo and to correlate them clinically and histologically to be able to predict stability of vitiligo.

Sixty-six untreated patients were included in this cross-sectional study and divided into two groups: stable group (SG) and unstable group (UG) based on history of inactivity of atleast 1 yr. Further history, clinical, histological (from a representative lesion) and dermoscopic findings were analysed appropriately.

Clinically ill-defined border was seen in 58.3% of cases in UG while sharp border was seen in 70% cases of SG and the respective associations were significant (P-value: 0.027). Koebnerisation was seen in 55.6% of UG and 16.7% of SG (P-value: 0.002). The dermoscopic features including perilesional focal hypopigmentation (PFH) (P value: 0.004), pseudopodia (P-value: 0.011), perifollicular hypopigmentation (PH) (P-value: 0.001), Comet tail pattern (CT) (P-value: 0.0342), Nebular pattern (P-value: 0.006) were significantly associated with UG. While, residual perifollicular pigmentation (RPP), polka dot pattern (PD), starburst and amoeboid patterns were more commonly seen with UG, however, the association was not significant. Sharp border (SB) (P-value: 0.022), sharply defined perifollicular pigmentation (SPP) (P-value: 0.026) and reticular pattern within vitiligo macule (RVM) (P-value < 0.0001) were significantly associated with stable vitiligo. In clinical examination, ill-defined border was concordant with pseudopodia of dermoscopy (P-value: 0.0043). Histological findings included epidermal lymphocytes, spongiosis, basal layer vacuolization, >100 dermal lymphocytes and melanophages. Histology was labeled as inflammatory if atleast one feature of both epidermal and dermal changes were present. Among dermoscopic findings, pseudopodia was significantly concordant with inflammatory histology (P-value: 0.0003).

The knowledge of activity of vitiligo is of utmost importance regarding choice of therapeutic modality and prognostication. This study characterizes dermoscopic features of stable and unstable vitiligo and establishes direct correlation of clinical, histopathological and dermoscopic features with activity of vitiligo. Clinically, ill-defined margin and presence of koebnerisation were found to be significantly associated with activity. Dermoscopic examination revealed several features viz. PFH, pseudopodia, PH, CT, nebular pattern, RPP, PD, starburst, amoeboid patterns, SB, SPP and RVM, most of them are self-explanatory. Out of these, PFH, pseudopodia, PH, CT and nebular patterns were significantly associated with activity while SB, SPP and RVM were significantly associated with stability of vitiligo. On correlating clinical features with dermoscopic findings, ill-defined margins significantly concorded with pseudopodia in dermoscopy. Moreover, presence of pseudopodia in dermoscopic examination significantly concorded with inflammatory histology. We propose that clinically ill-defined margin and presence of pseudopodia strongly indicate active vitiligo.

History, clinical features and dermoscopic findings altogether may provide adequate clue towards activity of vitiligo. Further randomized controlled studies are required to standardize the dermoscopic terminologies in vitiligo and to work out a scoring system based on history, clinical and dermoscopic examination to predict stability or activity of vitiligo.

Concurrent session: Lasers and Light Devices for Pigmentary Conditions

CS77

In vivo time sequential histological evaluation of pigment and melanocytes after Q-switched alexandrite laser irradiation: implication in post-inflammatory pigmentary changes

H. S. Song, M. Kim, W. J. Choi, H. Y. Kang

Dermatology, Ajou University School of Medicine, Suwon, Republic of Korea

Q-switched alexandrite laser (QSAL) has been used in treating hyperpigmentary disorders. Postinflammatory pigmentary changes after laser treatment are challenging.

This study aimed to characterize the dynamic alterations involved in laser-assisted pigment removal and melanocyte change. The possible factors implicating melanocyte activation after irradiation were also investigated.

The back skin of four healthy volunteer was irradiated with QSAL. Time-sequential skin biopsies were performed, i.e. baseline, immediate, day 2, day 7, and day 28. The pigmentary change was observed with Fontana-Masson staining and melanocytes were visualized with melanocyte-specific markers such as MITF, tyrosinase and gp100. Immunohistochemical studies such as TNF-α, α-MSH, ET-1, and SCF were performed.

QSAL produced successful removal of pigments and most melanocytes after irradiation. However, the numerous activated melanocytes were recognized on day 7 which restored the pigmentation. After laser irradiation, TNF-α expression was found to be decreased and returned to near normal levels at day 28. None of other well-known melanogenic factors such as α-MSH, ET-1, and SCF showed significant changes.

Although QSAL causes removal of pigmentation, irradiated melanocytes seemed to be activated on day 7 which may implicate in the pigmentary changes after laser treatment. Further studies are needed to identify regulators of melanocyte activation after laser irradiation.

CS78

The results and side effect of Q-switch YAG laser therapy for various skin pigmentary disorders

T. Shibata

Shibata Clinic of Dermatology, Osaka, Japan

I report the clinical results and side effects of Laser Toning (Q-switch YAG laser 1064 nm, Hoya ConBio Revlite's MedLite C6)

 © 2014 John Wiley & Sons A/S. Published by John Wiley & Sons Ltd

therapy for various skin pigmentary disorders in my clinic for 2 yr. The combination of urtla short pulse and proper wave length makes laser light selectively absorbed in targets (melanosomes). And then only targets are broken without any extra damages on surrounding normal tissues. These broken targets are removed by phagocytes. The most cases are melasma, acquired dermal melanosis (ADM), solar lentigo, post inflammatory pigmentation, skin amyloidosis and ephelides. The availavility of Laser Toning therapy after over six times irradiations was 512/589 (87%) and unavailability was 77/589 (13%). This therapy was very much effective especially for melasma, ephelides, skin amyloidosis and post inflamatory pigmentation. And over 10times irradiations with step by step increasing energy was also effective for ADM. The ratio of side effects was 225/789 (30.5%). The essencial side effects were redness (after irradiation, short duration) and temporary pain and remaining side effects were papules (16.8%), petechen (4.8%), macular depigmentation (1.1%), hyperpigmentation (0.7%), wheal (0.7%) and crust (0.3%). These side effects were reduced or disappeared according to the treatment progress. Until now no severe side effects were found in 30 000 times irradiations. The total satisfactory ratio of Laser Toning therapy was 512/589 (87%) and remarkable satisfactory ratio was 130/589 (22%). From the above results and other improvements of skin conditions that are expansion of pores, dullness, wrinkles, acne scars and dark rings, I cocluded that Laser Toning therapy for skin pigmentary disorders was very safe and effective.

CS79

A split-face comparison of Erbium: YAG micropeel versus Q-switched Nd:YAG laser for the treatment of light freckles and lentigines in Asian skin

H. J. Jun, S. M. Kim, S. H. Cho, J. D. Lee, H. S. Kim
Department of Dermatology, Incheon St. Mary's Hospital, The Catholic University of Korea, Seoul, Republic of Korea

Asians are prone to develop epidermal pigmentary lesions as a result of photoaging. Solar lentigines, especially those which are light in color, show somewhat limited response to pigment lasers and intense pulsed light sources.
We sought to compare the early effects as well as the side effects of Q-switched Nd: YAG and Er: YAG micropeel in treating light solar lentigines in Asians.
This was a split-face, evaluator-blind, randomized controlled study. A single session of treatment was performed on Asian patients with light facial lentigines. Q-switched Nd: YAG laser was allocated to one half of the face, and Er: YAG micropeel to the other half. The response to therapy was evaluated by two independent dermatologists with standardized photographs taken 2 weeks and 1 month after the laser treatment. Patients' satisfaction and preference in treatment were also assessed.
Fifteen patients completed the study and were analyzed. A reduction in pigment was observed with both lasers during the study period. The degree of pigment reduction in the Q-switched Nd: YAG treated side of the face was significantly higher than that of the Er: YAG micropeel treated side at 2-week follow-up ($P < 0.001$). The degree of pigment reduction between the Q-switched Nd: YAG treated side and the Er: YAG micropeel treated side was similar at 1 month follow-up ($P = 0.110$).
While there is no perfect therapy for light solar lentigines, a single session of Q-switched Nd: YAG laser and Er: YAG micropeel was shown to reduce pigmentation. The immediate effects (2-week follow-up) were better with the Q-switched Nd: YAG laser but there was no great difference between the two laser types at 1 month follow-up due to the greater degree of post-inflammatory hyperpigmentation following Q-switched Nd: YAG. Both the laser types could be applied either singly in turns, or in combination for maximal efficacy in future.

CS80

Treatment of periorbital eczema using copper bromide laser in atopic dermatitis patients

H. J. Kim[1,2], J. H. Kim[3]
[1]Dermatology, Seoul Medical Center, Seoul, Republic of Korea; [2]Reserch, National Skin Center, Singapore City, Singapore; [3]Dermatology, Yonsei University College of Medicine, Seoul, Republic of Korea

Atopic dermatitis (AD) is a common chronic inflammatory disease that presents a relapsing form of inflammation due to skin barrier interruption and allergic sensitization [1]. Histologic findings show dilated and tortuous vessels on the dermal papilla, which aggravate the inflammation by recruiting a number of immunoreactive factors then cause a vicious itching cycle [1]. Recently, vasoactive agents including vascular endothelial growth factor (VEGF), which lead to vessel dilatations and proliferations, are overexpressed in the lesions of AD [2, 3]. A therapeutic target to the vessel, thus, becomes an important method for stopping the chronic inflammations in AD [3]. We encountered two patients who have lichenifications on the periorbital areas caused by chronic AD and treated the areas using a copper bromide laser which is a new type of vascular laser with an output of 578-nm yellow light.
A 26-yr-old Korean male patient visited our clinic with a 2-yr AD history of scaly erythematous patches over his whole body. A second 23-yr-old Korean male patient presented to our clinic with chronic AD. He had previously been treated with topical corticosteroids, topical pimecrolimus, antihistamines and moisturizers for 2 yr. However, inflammations and lichenifications on the periorbital area of both patients, referred to Dennie-Morgan folds, cannot be well controlled by the regular use of topical corticosteroids or calcineurin inhibitors combined with oral antihistamines and corticosteroids .
Two sessions of treatment using a 578-nm copper bromide laser (Plus Yellow™; Norseld, Adelaide, SA, Australia) at 6-week intervals were performed for both patients. Two passes of treatment were delivered to the bilateral periorbital area, with settings of 578-nm yellow light, 25J/cm^2 and rapid pulse mode using a 1-mm hand-piece. The periorbital lesions of both patients were resolved by laser treatment and remained in an improved state one month after the final treatments In conclusion, 578-nm copper bromide laser treatments using yellow light are a good alternative option for the treatment of periorbital eczema in AD. However, further studies are required to understand the precise mechanisms of vascular laser treatment on AD.

CS81

Nevus depigmentosus treated by the 308-Nm exicimer laser: a retrospective single-center case series

J. M. Bae[1], H. Kim[2], H. M. Jung[1], J. H. Lee[1], G. M. Kim[1]
[1]Department of Dermatology, College of Medicine, The Catholic University of Korea; [2]Sosom Dermatologic Clinic, Seoul, Republic of Korea

Nevus depigmentosus is a congenital, nonprogressive, hypomelanotic disorder that is usually stable throughout life. Its pathophysiology is probably associated with a defect in the transfer of melanosomes from melanocytes to keratinocytes.

© 2014 John Wiley & Sons A/S. Published by John Wiley & Sons Ltd

Various treatment modalities have been attempted to induce pigmentation with variable results, and definite therapeutic modalities to treat nevus depigmentosus are not yet established. The aim of this study was to evaluate the effectiveness of 308-nm excimer laser in nevus depigmentosus, and to research into the long-term outcome after treatment.
A retrospective chart and photographic review was performed on nine patients with nevus depigmentosus (mean age: 10.3 $\pm$ 8.3, females: 44.4%) who had undergone 308-nm excimer laser treatment, from July 2008 to December 2012.
Six patients (66.7%) showed complete repigmentation after 8.3 months (4.2–13.6) of mean treatment duration. Maximum and cumulative dosages were 1316.7 mJ/cm^2 (range 1000.0–1600.0) and 43561.8 mJ/cm^2 (range 27150.0–72875.0), respectively. The remaining three patients showed 95%, 70%, and 8% of repigmentation, respectively. Interestingly, all of four patients who had been closely observed after the end of treatment revealed loss of pigmentation in their previous lesions.
The 308-nm excimer laser could be used to induce pigmentation in nevus depigmentosus. The 308-nm wavelength light might induce the upregulation and activation of some molecules which increase the transfer of melanosomes. However, it could also be expected to lose the pigmentation again when to discontinue the treatment.

Concurrent session: Hypopigmentary Disorders: Oculocutaneous Albinism & Others

CS82

Oculocutaneous Albinism (OCA) in Japanese patients: seven novel mutations and a case of OCA3

K. Okamura[1], J. Yoshizawa[1], Y. Abe[1], K. Hanaoka[2], N. Higashi[3], Y. Togawa[4], S. Nakagawa[4], N. Kambe[4], Y. Funasaka[5], K. Ohko[6], M. Kono[7], Y. Chinen[8], Y. Hozumi[1], T. Suzuki[1]

[1]Dermatology, Yamagata University Faculty of Medicine, Yamagata; [2]Dermatology, Tsuchiya General Hospital, Hiroshima; [3]Dermatology, Nippon Medical School Tamanagayama Hospital, Tokyo; [4]Dermatology, Chiba University Graduate School of Medicine, Chiba; [5]Dermatology, Nippon Medical School, Tokyo; [6]Dermatology, Kochi Medical School, Kochi University, Kochi; [7]Dermatology, Nagoya University Graduate School of Medicine, Nagoya; [8]Pediatrics, University of the Ryukyus Faculty of Medicine, Nishihara, Japan

Oculocutaneous albinism (OCA) is a rare genetic disorders characterized by a reduction or deficiency of melanin in the eyes, skin, and hair. OCA is classified into two groups: non-syndromic and syndromic. The former is caused by a reduction in melanogenesis, resulting in hypopigmentation and amblyopia, whereas the latter has a number of additional features, such as bleeding diathesis, immunodeficiency, and neurological dysfunction. To date, there are seven types of non-syndromic OCA: OCA type 1 (OCA1) that is caused by mutations in tyrosinase gene (*TYR*), OCA type 2 (OCA2) that is caused by mutations in *P* gene, OCA type 3 (OCA3) that is caused by mutations in tyrosinase-related protein 1 gene (*TYRP1*), OCA type 4 (OCA4) that is caused by mutations in solute carrier family 45 member 2 gene (*SLC45A2*), and OCA types 5, 6, and 7. The latter three types were reported very recently, and are rare.
We genetically examined seven Japanese patients who were clinically diagnosed with OCA. Then, we detected seven novel mutations. These mutations included six missense mutations; two in *TYR* (c.131G>A, p.S44N; c.283T>C, p.F95L), one in *P* (IVS17-3C>A), three in *SLC45A2* (c.157G>C, p.A53P; c.170C>T, p.T57I; c.233C>T, p.P78L), and one deletion mutation in *SLC45A2* (c.192delT, p.G64fsX112).
In addition, we identified a case of OCA3 in Japanese. This type is rare worldwide, especially in the East Asian area. To date, only one Japanese and two Chinese patients have been reported. So this was the fourth case from East Asia. The patient was a 5-yr-old Japanese boy. Physical examination showed blond hair and eyebrows, dark brown eyelashes and irises, lighter skin than that of the parents, and several pigmented nevi on the face. He had normal visual acuity with no nystagmus but had mild photophobia. Mutation screening revealed a homozygous mutation at c.1100delG, p.G367fsX384 in *TYRP1* for OCA3. The patient had an apparent clinically tyrosinase-positive OCA despite the homozygous frameshift mutation. This fact might support the suggestion that TYRP1 protein is not the rate-limiting factor in melanogenesis.
In conclusion, we detected seven novel mutations in Japanese patients with OCA and identified a fourth case of OCA3 in East Asia.

CS83

Analysis for regulatory T cells fraction and CCR4-expressing T cells and their potential function in patients with rhododenol-induced leukoderma

M. Nishioka[1,2], A. Tanemura[1], A. Tanaka[1], N. Arase[1], K. Teshima[2], H. Nishikawa[2], I. Katayama[1], S. Sakaguchi[2]

[1]Dermatology, Osaka University; [2]Experimental Immunology, Immunology Frontier Research Center, Osaka University, Suita, Japan

Rhododenol (4-(4-hydroxyphenyl)-2-butanol) is a skin-whitening agent potentially acting as a competitive inhibitor of tyrosinase. Rhododenol-induced leukoderma is hypopigmentary disorder induced by the cosmetics containing a whitening ingredient, rhododenol, and the patient number is estimated to be 16 864 in Japan. The hypopigmented lesions involved diverse incomplete to complete mainly on the bilateral face, neck, and dorsal hand. The biological pathogenesis may be complex including direct melanocyte toxicity of rhododenol or its metabolite and higher susceptibility to rhododenol. Moreover, it is also suggested that indirect effect through up regulation of immune-reactivity to melanocyte is influenced in disease onset and exacerbation.
To elucidate whether rhododenol-induced leukoderma patients show alteration in systemic and/or cutaneous immune milieu.
We investigated and compared naïve and effector regulatory T cells in the peripheral blood of rhododenol-induced leukoderma patients with those of vitiligo patients and normal individuals by FACS method. In parallel, we performed immunohistochemical analysis for skin-homing chemokines and their receptors in the skin.
In rhododenol-induced leukoderma patients, CD4 positive T cells and effector Tregs tended to be increased compared to those of healthy controls. Examining the expression of CCR4, a known skin homing receptor, on effector T cells, we observed significantly higher CCR4 expression on CD8+ T cells not CD4+ T cells in rhododenol-induced leukoderma patients than that of healthy control. In another analysis of sequentially harvested peripheral T cells, we found that CCR4 expression of all CD4+ effector cells, regulatory T cells and CD8+ T cells were decreased in most of the patients with time after withdrawal of rhododenol.

© 2014 John Wiley & Sons A/S. Published by John Wiley & Sons Ltd

We also detected CCL22, one of ligands for CCR4, expressing inflammatory cells in the lesional skin of rhododenol-induced leukoderma.
These results suggest that increased number and chemoattractant migration of $CCR4^{+}CD8$ T cells to the skin may play a part role in the induction of rhododenol-induced leukoderma.

CS84

Idiopathic guttate hypomelanosis: a clinical and histopathological spectrum from National Hospital, Sri Lanka

C. H. Ariyaratne[1], K. Satgurunathan[1], S. A. N. T. Sooriyaarachchi[2]
[1]Dermatology Unit, National Hospital Sri Lanka; [2]Pathology Unit, National Hospital, Colombo, Sri Lanka

Idiopathic guttate hypomelanosis (IGH) is a common, leukodermic dermatosis, typically presenting as sharply defined, asymptomatic porcelain white macules. The etiology remains uncertain.
Evaluate the clinical patterns of IGH with regard to morphological types, distribution, histological characteristics and evaluate the perilesional skin in IGH for evidence of solar damage.
A clinical survey was carried out on patients presenting to the skin clinic, over a period of one month. A total of 46 subjects with features of IGH were enrolled in the study while subjects with vitiligo and postinflammatory hypopigmentation were excluded. The skin type, distribution and characteristics of the hypopigmented macules, and other associated dermatoses were noted. Histopathological analysis of the lesions and the perilesional skin was performed with H and E and Massons-Fontana (MF) staining.
A female preponderance noted with a prevalence of 89.1%. Mean age of the sample was 53y. Mean age of onset was 39y. Anterolateral aspect of the lower limbs were most commonly affected (96%) followed by, photo exposed area of the forearms and the thighs (52%), trunk and arms (26%).
Majority of our patients (52%) found to have coexisting, hypopigmented sharply marginated oval or stellate shaped macules with typical porcelain white macules of idiopathic guttate hypomelanosis.
Well-circumscribed, slightly raised, smooth, flat topped, 3–4 mm sized, hypopigmented papules with preserved dermatoglyphics were noted in 9 (20%) patients along with the above lesions. Dermatoscopy ruled out seborrhoic kerarosis.
Porcelain white macules were more common in the fair skin type (68%) and hypopigmented macules were more in darker skin (83%).
Histology from both the typical porcelain white macules and the hypopigmented macules showed basket weave hyperkeratosis, epidermal atrophy with flattened rete ridges and reduced pigment and melanocytes in the basal layer which was confirmed with MF stain. Of the 10 biopsies performed on the hypopigmented flat topped papules, all had hyperkeratosis with basket weave appearance in 70%. Epidermal atrophy was less compared to the typical IGH in 80%. All showed reduced pigment in the basal layer confirmed with MF stain. Melanocyte count was reduced in 60%.
Only 16% of perilesional skin showed solar elastosis.
Xerosis and seborrhoic keratosis were noted in 46% and 41% respectively.
We observed three morphological variations with histopathological features similar to idiopathic guttate hypomelanosis. In addition to the typical porcelain white macules of IGH, majority of our patients had coexisting hypopigmented macules with angulated margins but with a lesser reduction in the melanin pigment in the basal layer. The third morphological variant was the well-defined, hypopigmented, flat topped papules with smooth surface and preserved dermatoglyphics. The histology was comparable with IGH but with lesser epidermal atrophy.
Thus IGH could have a spectrum of clinical and pathological types varying from porcelain white depigmented macules to hypopigmented macules and flat topped papules.
Only 16% biopsies revealed solar elastosis thus actinic influence as a major cause of IGH is less likely. Interestingly 6 of the patients with lowerlimb IGH were cooking with a wood hearth kept on the ground. Thus prolonged exposure to fire and high temperature could also be a risk factor. Higher percentage of coexisting seborrhoic keratosis and xerosis could imply aging as a causative factor. However a controlled study is needed.

Concurrent session: Perspectives of Skin Colour

CS85

Different responses to UV according to different skin color and body sites

J. Na, J. Shin, C. Huh, K. Park
Dermatology, Seoul National University Bundang Hospital, Seongnam, Republic of Korea

Erythema and pigmentation are major skin responses after UV irradiation. The degree of erythemal response is known to be dependent on Fitzpatrick skin type, age, anatomical site, UV wavelength, dose of radiation and skin thickness. However, it is difficult to predict responses. This study was performed to analyze the factors which are involved in UV responses. Healthy adults (six volunteer) were enrolled and melanin index (MI) and increment of erythema index (△EI) were measured. In one individual, twelve different body sites were selected and a total of 36 spots were irradiated with a single shot of monochromatic excimer laser (350 mJ/cm^2, XTRAC® Excimer Laser). Site was categorized into three groups (UZ: unexposed zones IEZ: intermittently exposed zones, FEZ: frequently exposed zones). The sun exposure indexes (SEI) were also calculated. △EI of UZ was significantly higher than that of FEZ, but lower than that of IEZ. In general, a significant relationship was found between △EI and MI (R^2 = 0.135). However, IEZ did not show significant relationships. Surprisingly, a more strong relationship was found between △EI and SEI (R^2 = 0.344). All of regression square was significantly higher by analyzing with SEI [0.541(UZ), 0.281(IEZ), and 0.228(FEZ)] than with MI [0.311 (UZ), 0.011 (IEZ), and 0.073 (FEZ)]. These findings showed that SEI is strongly correlated with UV sensitivity and 'induced level of pigmentation above constitutive level' is a better indicator for UV sensitivity. During the study, it was found that there were significant variations in pigmentation after UV irradiation. We planned to observe pigmentation changes after 308 nm excimer laser irradiation according to different skin color and body sites. Healthy adults (10 volunteers) were included. Irradiated sites were divided as UZ, IEZ and FEZ. Total 24 spots were irradiated with single shot of 300 mJ/cm^2 dose of excimer laser. The MI measurement was done before the irradiation, right after the irradiation, 1 day, 3 days, 1, 2, and 3 weeks after irradiation. MI decreased 1 day after irradiation and this trend was more obvious in light skin

© 2014 John Wiley & Sons A/S. Published by John Wiley & Sons Ltd

color. MI started to increase from day 3, and kept increasing until 3 weeks in light skin group whereas showed peak value at 1 week then started to decrease in dark skin group. In total, MI was significantly decreased 1 day after irradiation and started to increase 3 days after irradiation in all group. However, the peak MI value was shown at 1 week in IEZ and FEZ, whereas was shown at 2 week in UZ. These findings suggested that melanin index decrease especially in light skin before the development of delayed tanning after UV irradiation. Clinically, it will be important to know there are variations in UV responses according to different skin color (induced) or body sites.

CS86

Facial skin conditions among Southern Indian Women

G. Cow[1], M. Sachdev[2], C.-J. Loy[1]

[1]Johnson & Johnson Asia Pacific, Singapore City, Singapore; [2]MS Skin Centre, Bangalore, India

Skin hyperpigmentation is a common and distressing global skin condition. Among darker skin toned populations such as India, the manifestation and presentation of skin hyperpigmentation is variously perceived and not well described. To shed more light on self-perceived skin issues, a comprehensive study was conducted on 200 healthy female, age 13–50 yr old in Bangalore, South India to gain deeper insights into Indian skin pigmentation conditions. The current study involves dermatologist and self assessment, as well as non-invasive instrumental measurements with chromameter, mexameter, and diffuse reflectance spectroscopy (DRS). Our results indicated that the average skin color darkens with increase in age, as indicated by decrease in L-value and increase in melanin index in both the forehead and cheek regions. In addition, the forehead region consistently shows darker tone compared with the cheek region. It is interesting to note that 50% of the study population was dissatisfied with their current skin tone and aspired to have lighter skin tones. Furthermore, our data suggests that study subjects have limited knowledge on the type of facial skin discolorations that they currently experience and the importance of daily sunscreen use in their skincare regimen. Undereye dark circles appear to be a concern as evaluated by the dermatologist and by self-assessment. In conclusion, these findings provide a better understanding and characterization of facial skin conditions among Southern Indian women, especially as they relate to skin pigmentation conditions.

CS87

Variation in HSP70 expression contributes to skin color diversity via regulation of melanogenesis

D. Murase[1], A. Hachiya[2], R. Hicks[1], S. Moriwaki[2], T. Hase[2], P. Manga[3]

[1]Biological Science Americas Laboratories, Kao Corporation, Cincinnati, OH, USA; [2]Biological Science Laboratories, Kao Corporation, Haga-gun, Japan; [3]The Ronald O. Perelman Department of Dermatology, New York University School of Medicine, New York, NY, USA

Differences in epidermal melanin levels result in the wide variation in color associated with ethnic skin diversity. Ethnic differences result from variance in melanogenesis, melanin transfer, and melanosome degradation in the epidermis, but the mechanisms underlying these differences, and their contribution to the regulation of skin color, are not fully understood. In this study, we explored proteins which were differentially expressed between Caucasian- and African American donor skin- derived melanocytes using two-dimensional gel electrophoresis and mass spectroscopy. We consequently identified an important role for Heat Shock Protein 70 (Hsp70 encoded by *HSPA1A*) in skin color determination. In contrast to a previous report suggesting negative regulation of melanogenesis, Hsp70 was found to be more abundant in melanocytes from African American donors compared to those from Caucasian donors, which also reflected protein expression in the skin. In particular, inhibition of Hsp70, using either a specific inhibitor or siRNA targeting, significantly decreased melanin content of African American donor melanocytes, consistent with previous reports that Hsp70 is an Endoplasmic Reticulum (ER) chaperone involved in post-translational modification of tyrosinase. Indeed, inhibition of Hsp70 caused a decrease in tyrosinase protein levels by affecting its maturation. Taken together, our data reveal that Hsp70 plays an essential role in regulation of melanogenesis and thereby contributes to the ethnic diversity of skin color.

Concurrent session: Strategies in Skin Lightening

CS88

Multiple modes of action of niacinamide in regulating skin pigmentation

A. Damodaran[1], N. Nair[1], R. Shariff[1], A. Damle[1], G. Chandramowli[1], P. Joshi[2], H. Krishnamuthy[3], S. Vora[1]

[1]Unilever R&D, Bangalore; [2]Department of Biophysics, National Institute of Mental Health and Neuro Sciences; [3]Centre for Cellular and Molecular Platforms, NCBS-TIFR, Bangalore, India

Unilever pioneered the use of Niacinamide for skin lightening. We continued to dissect out the cellular and molecular mechanisms underlying the skin lightening potential of Niacinamide.

Niacinamide was found to reduce size of melanin granules in surface corneocytes of niacinamide-treated human skin. In co cultures of primary human melanocytes and HaCaT keratinocytes, we demonstrated a time-dependent increase in transfer by flow cytometry using dual staining, which was significantly down regulated by Niacinamide. Both phagocytosis and contact transfer has been suggested as possible modes of transfer of melanosomes to keratinocytes. In this study we demonstrate that Niacinamide inhibits contact-mediated transfer. Treatment of keratinocytes with niacinamide rendered keratinocytes incapable of accepting melanosomes from melanocytes in co cultures. Such melanocyte-keratinocyte interactions were found to be very specific as addition of melanocyte plasma membrane fraction on keratinocytes elicited intracellular calcium signals (Ca_i) in the latter, due to its release from intracellular stores, a process which was also inhibited by Niacinamide.

Besides contact-mediated transfer, the possibility of niacinamide modulating phagocytic uptake of melanosomes was also evaluated. Keratinocytes were pretreated with niacinamide or Soybean Trypsin Inhibitor (a known inhibitor of phagocytosis) and evaluated for uptake of fluorescent beads. While STI, significantly decreased bead uptake, niacinamide could not modulate this process. This was further corroborated by experiments which demonstrated that only STI and not niacinamide downregulated expression of PAR-2, receptors on keratinocytes known to modulate phagocytosis.

In order to examine the possibility of other modes of action of niacinamide on melanocytes and keratinocytes, we carried out

 © 2014 John Wiley & Sons A/S. Published by John Wiley & Sons Ltd

global gene expression profiling studies on co-cultures of primary human keratinocytes and melanocytes treated with niacinamide. Down regulation of key inflammatory cytokines and pigmentation gene transcripts such as IL1α, SOX9 and endothelin-1 was observed. This was substantiated in a UV-induced keratinocyte model wherein secretion of IL-1α was found to be down regulated by niacinamide. We had earlier shown the role of UV induced IL-1α in skin pigmentation in vitro. This phenomenon was verified in vivo through a modified SDS-induced inflammation model to assess Post-inflammatory Hyperpigmentation (PIH) where niacinamide significantly decreased SDS-induced PIH.
Taken together, the study throws light on the molecular and cellular mechanisms underlying the skin lightening benefits of niacinamide. It also expands our understanding of how niacinamide influences inflammation and thereby finds safe use in cosmetic formulations aimed at treating inflammation-induced PIH.

CS89

Hexyl resorcinol for spots and skin tone lightening in Asian and Caucasian skin

E. Lazo, C.-J. Loy
Johnson & Johnson Asia Pacific, Singapore City, Singapore

Skin, whether Asian or Caucasian, is naturally fair, tan, dark, prone to fine lines and wrinkles in some, prone to brown spots and pigmentation in others. Despite these physiological variations and aesthetic needs of the individual, treating different skin-types with a suitable cosmetic active to address skin pigmentation needs is still possible with active ingredients like Hexyl Resorcinol (HR).
HR is a high efficacy inhibitor of tyrosinase enzyme and UV-induced melanogenesis. It has been shown to be efficacious in reducing melanin production in cell culture and in 3-dimensional pigmented skin equivalent model. An 8-week clinical study with female Caucasian subjects aged 40–65 yr old have shown that a cosmetic formula containing HR provides significant improvements in mottled pigmentation and age spots versus baseline and placebo ($P < 0.05$) within 4–8 weeks based on dermatological assessment and around 65% of the subjects perceived improvements, also in mottled pigmentation and age spots after 8 weeks. Similarly, a 4-week study conducted with female Asian subjects aged 30–55 yr old showed that HR provides significant improvement versus baseline on spot and skin lightening and are both better than placebo after 4 weeks based on instrumental, dermatological, and self-assessment grading. In addition, another 8-week study with female Asian subjects aged 30–45 yr old showed that HR provided significant improvements versus baseline ($P < 0.05$) in terms of spot related attributes like reduction in contrast between spot and surrounding skin after 8 weeks.
These studies, all double-blind, placebo-controlled, full-face clinicals comparing twice-daily application of the test products and conducted separately in the United States and Asia show that HR provides significant clinical and self-perceived skin benefits regardless of skin-type, whether Asian or Caucasian.

CS90

Identification of A11 as a safer skin whitening reagent using zebrafish embryo

C.-C. Huang[1], K. Martinson[1], E. Butterbrodt[1], A. Monte[2]
[1]Biology, University of Wisconsin-River Falls, River Falls, WI; [2]Chemistry & Biochemistry, University of Wisconsin-La Crosse, La Crosse, WI, USA

Throughout the cosmetic industry skin whitening has become a major practice because of its ability to correct uneven skin pigmentation, lighten natural skin tones, and treat pigment disorders. However, many chemicals presently used in skin whitening exhibit adverse health effects including skin sensitivity, and cancers such as leukemia. The goal of this project is to explore new skin whitening chemicals that act in different mechanistic pathways than the present therapeutic options in hopes of developing safer skin-whitening alternatives. Several current human skin whitening agents, including Arbutin, Niacinamide, Tretinoin, Kojic acid, Gallic acid, Haginin and a known melanin synthesis inhibitor, PTU (phenylthiourea) were compared with our newly discovered skin whitening reagents, A11 and MEK-I using zebrafish embryos. To test the melanin synthesis inhibition, the zebrafish embryos were treated with the drugs from 24 to 76 h post-fertilization (hpf). The embryos were monitored and imaged to track the development of melanin development. As expected, PTU produced embryos with ~0% pigmentation. While most of the human reagents produced embryos with between 5% and 80% pigmentation reduction, A11 produced >90% reduction and ~40% by MEK-I. To test the effect of these reagents on existing melanin, the embryos were treated with the chemicals at 48 hpf, after pigment had developed. Interestingly, we found that most tested agents had little effect on the existing pigment but A11 and PTU seemed able to remove distinguishable amount of existing pigment. Additionally, when treatment was washed away, all embryos exhibit melanin recovery quickly, except those treated with A11 which recovered slowly and only partially, suggesting the effects of A11 may be longer lasting. Furthermore, even though Tretinoin and Haginin show potent skin whitening effect, they caused severe toxicity in the body axis and developing heart of the embryos, respectively. In contrast, A11 caused no detectable toxicity. Our results suggest that A11 may be used as a safer skin whitening reagent.

Plenary Session: Close of IPCC 2014

CS91

International consensus on core outcomes set for vitiligo

V. Eleftheriadou[1], K. Thomas[1], A. Taieb[2], M. Picardo[3], K. Ezzedine[2]
[1]Centre of Evidence Based Dermatology, University of Nottingham, Nottingham, UK; [2]Dermatology, University of Bordeaux National Reference Center for rare skin diseases Hôpital St-André, Bordeaux, France; [3]Dermatology, Dermatological Institute San Gallicano IRCCS, Rome, Italy

Vitiligo is the most common depigmentation disorder of the skin affecting around 0.5% of the population worldwide. The Cochrane systematic review of randomised controlled trials (RCTs) for the treatment of vitiligo was unable to perform meta-analyses due to heterogeneity of the included trials. A

© 2014 John Wiley & Sons A/S. Published by John Wiley & Sons Ltd

subsequent systematic review of outcomes measures used in published vitiligo RCTs, and a survey amongst patients and clinicians concluded that there is no unified scale to measure repigmentation and that patient input in outcomes assessment is limited. Twenty five different outcomes were measured in 54 RCTs. From patients' and clinicians' perspective, cosmetically acceptable repigmentation rather than percentage of repigmentation was the most desirable outcome; however this has not been reflected in the outcome measures used in RCTs to date, and there is currently no core outcome set for use in vitiligo trials.
To achieve international consensus over the core outcome domains to be captured in future vitiligo trials.
This was a three stage, web-based, international Delphi (e-Delphi) exercise involving dermatologists and researchers with interest in vitiligo, patients with vitiligo (and their carers), representatives of regulatory agencies, and journal editors. Participants were identified through the International Federation of Pigment Cell Societies (IFPCS). The study took place from January 2013 to September 2013). The initial survey included nine outcome domains that had been previously identified as being important to capture in the systematic review of outcome measures. In round 1, participants were able to add additional outcomes for consideration in the subsequent round. In the first two rounds, participant were asked to rate the importance of the individual outcome measures by using a five-point Likert scale (very important, important, less important, not important, not sure). Consensus was pre-defined as being achieved if >75% of participants in two stakeholder groups agreed for an individual response category. In round 3, participants were asked to rank the outcomes that had reached consensus in round 2 as being essential (must be relevant to all interventions for vitiligo and reported in all clinical trials) or recommended (should be reported if relevant to the intervention being tested and trial design used). Statistical analysis was performed using the 'Survey Monkey' online tool to determine the modal answer for each question.
Overall 101 participants took part in the e-Delphi process and 81/101 (80.2%) completed all three rounds. Participants included 51 dermatologists, 32 patients, 14 Journal editors, 2 representative of regulatory body, 1 nurse and 1 researcher), representing 25 countries. In completion of the consensus exercise, three domains were deemed essential: repigmentation, side effects and harms of treatment, maintenance of gained repigmentation. Five domains were deemed recommended: cosmetic acceptability of the results, quality of life, cessation of spreading of vitiligo, tolerability/burden of treatment.
Core outcomes domains were identified with a high degree of consensus amongst different stakeholders groups internationally. The core set of outcomes is the minimum set of domains to be assessed in future vitiligo trials. More research is needed to define which scale should be used to measure each of these domains.

Abstracts of Posters P1 – P187

P001 – P003: Developmental Biology

P001

Functional differentiation of the Endothelin 3-the receptor signal transduction system in pigmentation of chicken

T. Akiyama[1], T. Adachi[1], A. Shinomiya[2], K. Kinoshita[3], M. Nakano[3], M. Mizutani[3], Y. Matsuda[3]

[1]Biology, Keio University, Yokohama; [2]Photobiology, National Institute for Basic Biology, Okazaki; [3]Grad. School of Agricultural Sciences, Nagoya University, Nagoya, Japan

Endothelin 3 (EDN3) induces active proliferation and differentiation in pigment cells during early embryogenesis. In human and mouse, mutations of the genes of EDN3 and the receptor, endothelin receptor B (EDNRB), cause the severe defect such as white spotting, agangliotic megacolon or lethal in homozygous mutant mouse. In Aves, there has been no report on 'loss of the function' of EDN3 by genetic mutation but we have report on 'gain of the function' of EDN3 by duplication of the region containing *EDN3* gene in Silky chicken. In the Silky, many inner organs display hyperpigmentation by lots number of the melanocytes and the phenotype is called '*Fibromelanosis; Fm*'. We disclosed that the responsible gene for *Fm* locates on the duplication region with 130 kb containing *EDN3* gene in chromosome 20 by linkage mapping (Genetics 2012, 190, 627–638). Furthermore, we isolated recently the *EDNRB2* mutants from the several Japanese domestic chicken lines (PLOS ONE 2014, 9, e86361). These mutant chickens show whole white plumage with mo^w and white spotting with black feather with *mo* mutation in *EDBRB2.* They have normal *EDNRA* and *B* and grew up to adult with neither health problems such as megacolon nor abnormal behavior to the sound or light. To examine the effects of the *EDNRB2* mutation on the *Fm* expression, firstly, we made hybrid progeny between mutants with homozygous mo^w (mo^w/mo^w, *fm/fm*) and GSP (wild type chicken line for *EDNRB2; Mo/Mo, fm/fm*) and the F_1 progeny (*Mo*/mo^w, *fm/fm*) was crossed with Silky (*Mo/Mo*, *Fm/Fm*). Then the effect of the *EDNRB2* mutation on the phenotype of *Fm* in the final progeny (*Mo/Mo* or *Mo*/mo^w, *Fm/fm*) was analyzed. Although all the final progeny had similar outlook such as almost black-brown plumage, black eye and gray colored shank, some of these showed small variation with white feather and yellow toes. To evaluate the effects on the dermal hyperpigmentation (*Fm* phenotype), we had to consider the involvement of *Id* (*dermal melanin inhibitor*, a gene locates on z chromosome; undetermined) or *E* (*extension; Mc1r*) in the expression of *Fm* phenotype because these genes are believed to interfere the dermal pigmentation. As the useful samples, we selected a family (C-30, 31 individuals) with black feather and gray shank and toes in the obtained 3 families with 137 individuals. Since it was possible to presume the existence of *Id* from the yellow or white shank color, we could select the family that the male (z/z) didn't show the white or yellow shank (*Id/Id* or *Id/id*). From the results, all the progeny shows black plumage color in major part but the less pigmentation in the inner organs of the mutant chicken (*Mo*/mo^w, *Fm/fm*) was found than the wild type (*Mo/Mo*, *Fm/fm*). The cell number of the melanoblasts from neural crest in mutant significantly decreased from wild type in both single cell culture and organ culture. Then, it is suggested that the proliferation of the melanoblasts in *EDNRB2* mutants was blocked at the neural crest before migration. These results demonstrate that the

 © 2014 John Wiley & Sons A/S. Published by John Wiley & Sons Ltd

mutation of *EDNRB2* clearly suppressed the *Fm* phenotype expression. Therefore, it is strongly suggested that the high dose of the EDN3 signal produced by the duplication of *EDN3* region is necessarily to be fully received with normal EDNRB2. The EDN3-EDNRB2 signaling is essential for melanocyte development in chicken but *EDNRB2* gene was lost in the mammals. The function of EDN3-EDNRB and -B2 signaling in chicken was integrated into EDN3-EDNRB in mammal in evolutional process.

P002

Epigenetic stable reprograming in early mammalian development for the disease free life

A. Mishra, N. Srivastava

Pharmaceutical Chemistry, Aibs, Kanpur, India

Epigenetic modifications of the gene play a significant role in the elaboration of the genetic code as established at fertilisation. These modifications affect early growth and development through their influence on gene expression especially on imprinted genes. Gene-wide epigenetic reprogramming is essential in order to reset the specific marking of imprinted genes, but may have a more general role in the restoration of totipotency in the early embryo. DNA methylation is one of the best-studied epigenetic modifications of DNA in all unicellular and multicellular organisms. Maternal chromatin is organized such that DNA methylation and chromatin modifications are already abundant at fertilization. These include both nucleohistone modifications and chromatin proteins associated with active and repressive configurations. In particular, different modifications (e.g. acetyl or methyl) of the same amino acid residues are detectable, but these occur in different parts of the genome. Preimplantation phase is characterised by its replication dependency (passive demethylation) and results as a consequence of the exclusion of DNA methyltransferase 1 (Dnmt1) from the nucleus. This enzyme is the primary maintenance DNA methyltransferase and normally restores DNA methylation to symmetrical dinucleotides in a semi-conservative manner during or shortly after DNA replication. Hence this type of genetic reprogramming will used for the any type of desired changes for the fourth coming life of that mammalian to obtain a totally disease free life.

P003

How is regionalization of the chicken developing eye primordium regulated?

H. Tabata, D. Nishihara, A. Koinui, H. Yamamoto

Nagahma institute of Bio-science and technology, Nagahama, Japan

The retinal pigment epithelium (RPE) is a monolayer of melanin-producing cells and is an important component of the vertebrate eye. The optic vesicle (OV), which is derived from the lateral wall of the forebrain, develops both the RPE and the neural retina (NR) primordia. The inductive interactions between the OV and the surface ectoderm (the future lens and cornea) induce the invagination of the OV to form the bilayered optic cup (OC), in which the outer and inner layers are specified into the RPE and NR, respectively. Unlike the monolayered RPE, the NR develops multiple cell layers, which contains photoreceptors, retinal ganglion cells (RGC), horizontal cells, amacrine cells, bipolar cells and Müller glia cells.
The developmental event of the RPE and NR from the OV is called regionalization, which is promoted by several transcription factors. The characteristics of the presumptive RPE regions are induced by *Orthodenticle homeobox 1 and 2 (Otx1 and 2), Microphthalmia-associated transcription factor (MITF)* and others. On the other hand, the induction of NR differentiation is promoted by several other transcription factors and by *Fibroblast growth factor 8 (Fgf8)*, etc. Still, it is not known whether *Paired box 6 (Pax6)*, a key gene for eye development, is critically involved in this regionalization at early developmental stages of the eye. The expression patterns of Pax6 in both the RPE and NR primordia at these stages make their functional involvement obscure. Still, we think that this transcription factor is critically involved in the regionalization. We will show the expression patterns of several other related factors and will discuss the possible role of Pax6 in this developmental step.

P004 – P009: Melanin Biophysics & Chemistry

P004

N,N,N-trimethylphytosphingosine-iodide decreases melanin synthesis through ERK activation in human melanocytes

S. Choi, S. H. Bang, W. J. Lee, M. W. Lee, J. H. Choi, S. E. Chang

Department of Dermatology, Asan Medical Center, University of Ulsan College of Medicine, Seoul, Republic of Korea

Melanin is produced by specialized organelles named melanosomes in melanocytes. In melanosomes, melanin pigment are synthesized by an enzymatic cascade that is controlled by tyrosinase, tyrosinase related protein 1 (TRP-1) and tyrosinase related protein 2 (TRP-2).
N,N,N-trimethylphytosphingosine-iodide (TMP) was recently prepared as an antitumor agent. Although phytosphingosine is an important molecule in many signaling pathways, the effects of TMP on melanogenesis have not been evaluated. Here, we examined the effects of TMP on melanogenesis and its related signaling pathways using human melanocytes.
Liposomal preparation of TMP was elucidated to reduce cytotoxicity of TMP in cell viability test. Present results showed that melanin content and tyrosinase activity were significantly reduced in human melanocytes after TMP treatment in a dose-dependent manner. However, TMP did not affect mushroom tyrosinase activity. TMP treatment also reduced both microphthalmia-associated transcription factor (MITF) and tyrosinase protein levels. Additionally, We also investigated changes in the phosphorylation of extracellular signal-regulated protein kinase (ERK), which is related to melanin regulation. These results indicated that TMP treatment led to the phosphorylation of ERK and a specific ERK pathway inhibitor, PD98059 restored the TMP-induced reduction of tyrosinase activity and pigmentation. These results suggest that the hypopigmentary action of TMP is due to MITF and tyrosinase downregulation by ERK activation.

P005

Pleiotrophin inhibits melanogenesis via ERK1/2-MITF signaling in normal human melanocytes

W. J. Choi, M. S. Kim, H. Y. Kang

Dermatology, Ajou University School of Medicine, Suwon, Republic of Korea

Epidermal melanocytes synthesize melanin pigments in specialized cellular organelles called melanosomes, which are

© 2014 John Wiley & Sons A/S. Published by John Wiley & Sons Ltd

transported from melanocytes to adjacent keratinocytes. Interactions between melanocytes and neighboring skin cells, such as keratinocytes and fibroblasts, are important in the regulation of the melanocyte function and in the consequent cutaneous pigmentation process. For example, keratinocytes secrete many paracrine factors, such as α-MSH and endothelin-1, which induce pigmentation and the tanning response upon UV irradiation

Pleiotrophin (PTN) is a secreted heparin-binding protein that is involved in various biological functions of cell growth and differentiation processes. Little is known about the effects of PTN on the melanocyte function and skin pigmentation. In this study, we investigate the expression and functional role of PTN in normal human melanocytes. PTN is expressed in the melanocytes and fibroblasts of human skin. The upregulation of PTN in melanocytes significantly reduces melanogenesis. Consistent with the inhibitory effect of PTN, PTN shRNA induces melanogenesis. The fibroblast-derived PTN also inhibits melanin synthesis in melanocytes. Moreover, exogenous PTN decreases pigmentation in *ex vivo* cultured skin. The inhibitory action of PTN in pigmentation is associated with MITF degradation *via* ERK1/2 activation in melanocytes. These findings suggest that PTN is expressed in human skin and that PTN inhibits melanogenesis in normal human melanocytes through autocrine and paracrine processes.

P006

Weakly acidic pH suppresses eumelanogenesis but promotes pheomelanogenesis

K. Ito, S. Ito, K. Wakamatsu

Department of Chemistry, Fujita Health University School of Health Sciences, Toyoake, Japan

The diversity of pigmentation in the skin, hair, and eyes of humans has been largely attributed to the diversity of pH in melanosomes with acidic pH being proposed to suppress melanin production, especially eumelanogenesis. Human tyrosinase has an optimum pH of 7.4 and its activity is suppressed greatly at lower pH values. Recently we showed that an acidic pH greatly suppresses the late stages of eumelanogenesis after the dopachrome stage (Ito et al., PCMR, 2013). When tyrosine is oxidized by tyrosinase in the presence of cysteine, cysteinyldopa isomers are formed, which are further oxidized to give rise to pheomelanin via benzothiazine intermediates. However, how these steps are controlled by pH is not known. In this study, we examined the effects of pH (5.3–7.3) on the oxidation of tyrosine or dopa (1 mM) in the presence/absence of cysteine (1 mM). The oxidation was carried out with mushroom tyrosinase at 37°C for 4 h. Mushroom tyrosinase has a broad pH optimum with a maximum activity at pH 6.8 using L-tyrosine as a substrate (at pH 5.3, the activity was 75%). Melanins produced were analyzed by Soluene-350 solubilization (A500, total melanin marker), alkaline hydrogen peroxide oxidation to give pyrrole-2,3,5-tricarboxylic acid (PTCA, eumelanin marker), and hydroiodic acid hydrolysis to give 4-amino-3-hydroxyphenylalanine (4-AHP, pheomelanin marker). As expected, eumelanin production from tyrosine was suppressed at acidic pH; at pH 5.8, A500 and PTCA were 77% and 48% those at pH 7.3. On the contrary, pheomelanin production from tyrosine and cysteine was not suppressed, but rather promoted, at acidic pH with plateau around pH 5.8–6.3. At pH 6.3, A500 and 4-AHP were 172% and 177% those at pH 7.3. Taken together, these results indicate that pheomelanin production at acidic pH of 5.8–6.3 is 2–3-fold favored over eumelanin production at pH 7.3. Production of melanins from dopa (plus cysteine) gave similar trends of data. In conclusion, pheomelanogenesis is promoted by weakly acidic pH. Further studies will be needed to elucidate which stage of pheomelanogenesis is promoted by weakly acidic pH.

P007

Atomic-scale understanding of catalytic behavior of Cu (II) ion for dopachrome conversion

R. Kishida[1], A. G. Saputro[1], H. Nakanishi[1], H. Kasai[1,2]

[1]Department of Applied Physics, Osaka University, Suita, Japan; [2]Center for Atomic and Molecular Technologies, Osaka University, Suita, Japan

Dopachrome conversion to 5,6-dihydroxyindole (DHI) and 5,6-dihydroxyindole-2-carboxylic acid (DHICA), the second regulatory step in eumelanogenesis, affects the chemical phenotype of generated eumelanin. Previous experimental studies have revealed that inclusion of DHICA unit in eumelanin is necessary to exert its antioxidation activity, and the rate of dopachrome conversion for DHICA can be catalyzed by a certain kind of metal ions such as Cu(II), Ni(II), or Co(II) in the solution. However, despite its importance in understanding control of melanogenesis, the effect of metal ions on the mechanism of dopachrome conversion remains to be elucidated at the atomic level.

Considering experimental difficulties in the measurement of dynamical atomic behaviors, we performed first-principles calculations to evaluate appropriate reaction scheme and to clarify the effect of metal ions on the conversion.

Our results showed that Cu(II) coordination to 5,6-oxygens of dopachrome greatly reduces the activation barriers for α,β-deprotonation and decarboxylation from the Cu(II)-dopachrome, corresponding to the catalytic behavior of Cu(II) that was examined in the previous experiment. In addition, we found that proton rearrangements to Cu(II) coordinated 5/6-oxygen, which are the critical steps to form DHI, is not energetically preferable. Especially, O5/O6-protonation to carboxyl dissociated structures with Cu(II) coordination exhibited significant energy rise.

These findings indicate that the predominant formation of DHICA in the presence of Cu(II) is not achieved by selective acceleration of α-deprotonation but rather achieved by inhibition of the proton rearrangement to 5/6-oxygen.

On the basis of above understanding at the atomic level, we point that inhibition of O5/O6-protonation is important to obtain DHICA-melanin.

P008

Convenient synthetic method of putative degradative markers to identify various catecholic metabolites in addition to dopamine and norepinephrine incorporated in neuromelanin

K. Tabuchi[1], H. Tanaka[1], M. Ojika[2], F. A. Zucca[3], L. Zecca[3], S. Ito[1], K. Wakamatsu[1]

[1]Department of Chemistry, Fujita Health University School of Health Sciences, Toyoake; [2]Department of Allied Molecular Biosciences, Nagoya University Graduate School of Bioagricultural Sciences, Nagoya, Japan; [3]Institute of Biomedical Technologies, National Research Council of Italy, Milano, Italy

Neuromelanin (NM), black to brown pigment, is produced mainly in the substantia nigra (SN) and locus coeruleus (LC) in the

 © 2014 John Wiley & Sons A/S. Published by John Wiley & Sons Ltd

central nervous system of human and mammalian species. NM is formed by the oxidation of catecholamines, dopamine (DA) and norepinephrine (NE), in the presence of cysteine (Cys). Biosynthesis of NM, oxidative polymerization of catecholamines in addition to DA and NE, suggests a possibility that various catecholic metabolites would also participate in the NM genesis. Thus, in addition to DA and NE, 3,4-dihydroxyphenylacetic acid (DOPAC), 3,4-dihydroxymandelic acid (DOMA), 3,4-dihydroxyphenylethanol (DOPE), and 3,4-dihydroxyphenylethyleneglycol (DOPEG) can be incorporated into NM. Hydroiodic acid (HI) hydrolysis of NM derived from the above catechol derivatives is expected to afford the corresponding *o*-aminophenol derivatives, aminohydroxyphenylethylamine isomer (AHPEAs), aminohydroxyphenylacetic acid isomer (AHPAAs), and aminohydroxyethylbenzene isomer (AHEBs). We newly synthesized these *o*-aminophenol derivatives as putative degradative markers of NM. They were synthesized by the nitration of the corresponding phenol derivatives followed by reduction with HI. The reductive HI hydrolysis of NM isolated from natural SN and LC in the human midbrains was performed, and the degradation products were identified with the synthesized *o*-aminophenol compounds in HPLC determination. In this study we could detect AHEBs for the first time in NM from SN. In NM from LC, we could newly detect AHPAAs and AHEBs together with AHPEAs. These results suggest that NM in SN and LC is derived not only from DA and NE, but also from DOPAC, DOMA, DOPE, and DOPEG, because AHPEAs, AHPAAs, and AHEBs are expected to arise from DA or NE, DOPAC or DOMA, DOPE or DOPEG, respectively. This elaborated degradative approach would help identify catecholic precursors unique to SN-NM and LC-NM which are present in catecholaminergic neurons.

P009

The influence of selected transition metal ions on the ability of melanin to decompose hydrogen peroxide

A. C. Zadlo[1], A. K. Pilat[1], M. W. Sarna[1,2], A. A. Broniec[1], T. J. Sarna[1]

[1]Biophysics, Faculty of Biochemistry, Biophysics and Biotechnology, Jagiellonian University; [2]Medical Physics and Biophysics, Faculty of Physics and Applied Computer Science, AGH University of Science and Technology, Krakow, Poland

Melanin is believed to be a photoprotective pigment and numerous studies have shown that in model systems melanin can also act as an efficient antioxidant. Although some reports suggest that melanin is able to decompose hydrogen peroxide, the mechanism of this process remains unknown. All natural melanins contain transition metal ions, which may significantly affect physicochemical properties of the pigments. In this study, we analyzed the effect of zinc, cupric, ferric and ferrous ions on the ability of melanosomes, isolated from bovine retinal pigment epithelium (BMs), and synthetic melanins to decompose hydrogen peroxide. Electron paramagnetic resonance (EPR) spectroscopy, EPR oximetry, electrochemical methods, dynamic light scattering (DLS), atomic force microscopy (AFM) and spectrophotometry were employed. We have shown that decomposition of H_2O_2 by BMs is accompanied by generation of oxygen. While enrichment of melanosomes with iron or copper ions accelerated this process, it was inhibited by proteinase K in combination with EDTA. Synthetic melanins obtained by DOPA autooxidation (DMa) or by tyrosinase-catalyzed DOPA oxidation (DMt) were unable to decompose hydrogen peroxide. After incubation of DMt with 1% (w/w) Zn^{2+}, Cu^{2+} or Fe^{3+} such a melanin was able to decompose H_2O_2 and the effect increased with incubation time. However, even after 15 days, the observable decomposition rate was rather slow and the effect of different ions was comparable. Although enrichment of DMa with Fe^{2+} significantly accelerated the decomposition rate of H_2O_2, the process was not accompanied by a substantial release of oxygen. On the other hand DMt incubated for one week with Fe^{2+} decomposed H_2O_2 at least five times faster than DMt incubated with Zn^{2+} with concurrent production of oxygen. Corresponding changes in redox state of iron were monitored by direct and indirect EPR spectroscopy; by measuring EPR spectra of ferric complexes with melanin, and of melanin radicals and power saturation of the melanin signals. Such EPR analysis and spectrophotometric measurements of supernatant from DMt indicated that slow, time-dependent binding of iron ions by melanin occurred and that the ferrous ions were bound faster that the ferric ions. DLS and AFM analyses suggested that DMa could aggregate in different way depending on the type of metal ions. Oxygen evolution that accompanied decomposition of hydrogen peroxide by melanin incubated with iron for one week suggested catalytic mechanism of this process rather than a Fenton-type reaction. The catalytic process was apparent only after binding of most of the iron ions by melanin and probably involved contribution of both ferrous and ferric ions. Elucidation of the predominant mechanism of decomposition of hydrogen peroxide by melanin under physiologically relevant conditions may be of particular importance in the case of retinal pigment epithelium (RPE) melanin, which undergoes very little, if any, metabolic turnover. This is because possible oxidative modifications of RPE melanin, expected in Fenton-type chemistry, could significantly reduce antioxidant properties of the melanin and even induce its pro-oxidant action. It can be postulated that such changes, accompanying aging, may contribute to pathogenesis of age-related macular degeneration. Supported by National Science Center (grant 2012/07/D/ST4/02211)

P010 – P015: Melanosome Biogenesis & Transfer

P010

A retroviral insertion in the tyrosinase gene (Recessive White; C/C) induces white plumage but not effects on the dermal pigmentation in white silky chickens

T. Adachi[1], A. Shinomiya[2], K. Kinoshita[3], M. Nakano[3], M. Mizutani[3], Y. Matsuda[3], T. Akiyama[1]

[1]Biology, Keio University, Yokohama; [2]Photobiology, National Institute for Basic Biology, Okazaki; [3]Grad. School of Agricultural Sciences, Nagoya University, Nagoya, Japan

Melanosome transfer from melanocytes to keratinocytes is one of the interesting topics in human dermatology. After melanosome maturation in melanocytes, the pigment-loaded melanosomes are translocated from the original position in the perinuclear cytoplasm towards the melanocyte dendrite tips using microtubule-based and actin-based motor proteins. For instance, Rab27 is known to be involved in recognition of the matured melanosomes, binding to the melanosomes, and transport of it to the end of the dendrites. Then melanosomes in melanocytes are passed into the neighboring keratinocytes. When the melanosomes are not transferred to keratinocytes by mutation or blockade by inhibitors, the skin expresses very light

© 2014 John Wiley & Sons A/S. Published by John Wiley & Sons Ltd

color according to the small amount of melanin in the surficial keratinocytes. In human, it is notified as useful phenomenon to get white skin with cosmetics. In Aves, the individuals with the defect of melanosome transfer reveal white plumage since the feather color is determined by melanin transferred to feather keratinocytes. So far, the four chicken lines having white plumage have been reported; (i) dominant white (*I; Pmel17*); (ii) albino (white-red eye; c^a/c^a) (Tobita-Teramoto *et al.*, 2000); (iii) recessive white (*c/c*) (Chung *et al.*, 2006) and (iv) mo^w (*EDNRB2*) (Kinoshita *et al*, 2014). Tyrosinase, the key enzyme in melanin synthesis, is known as a responsible gene for an albino mutation and we reported previously that a 6 bp-deletion was found in the tyrosinase. Moreover, it has been reported that the insertion of a complete avian retroviral sequence of the Avian Leukosis Virus (ALV) family in the tyrosinase gene causes the recessive white mutation in chicken (*c/c*).

Silky chicken, which show a huge number of melanocytes in its dermal tissues and inner organs, is known to have two color variants. White Silky (WS) and Black Silky (BS) reveal white and black plumage, respectively. But both display similar hyperpigmentation in their inner organs and show dermal melanization in dark gray skin. We initially genotyped the insertion of the ALV using albino, white Plymous Rock, a recessive white strain which is established from Tosa-jidori and Shokoku which express white plumage and Black Minorca as an example of wild-type. Results from these strains show that all homozygous carriers of this insertion display a white plumage. The insertion was not found in any of the population showing colored chickens. In Silky, all of the WS have the insertion but not in BS. Interestingly, BS derived chickens with white plumage and dark gray skin also had the insertion. These data show that the *c/c* mutation could affect only feather color but not in dermal tissue. Therefore, the comparative study using WS and BS is a valuable tool to examine the pigmentation in integumental and dermal tissues. From comparison of the pigmentation between theses tissues in WS, we disclosed that (i) there is no keratinocytes around inner organs, (ii) melanosomes are retained in melanocytes and (iii) the melanosome maturation is seemed to be defect in the *c/c* mutant chickens. From these observations, we suppose that the *c/c* mutation cause disorganization of pigment in melanosomes and induce the defect of transfer to the feather keratinocytes. In WS, there is no pigmentation in the feather keratinocytes although hyperpigmentation of internal organs. From this result, it is suggested that unmatured melanosomes in WS loading *c/c* are suppressed the transportation into the keratinocytes.

P011

Ep-2 inhibits melanin synthesis and melanosome transfer mediated by protease-activated receptor-2 in keratinocytes

S. Choi[1], S. Y. Jo[1], J. E. Kim[2], M. W. Lee[1], J. H. Choi[1], S. E. Chang[1]

[1]Department of Dermatology, Asan Medical Center, University of Ulsan College of Medicine; [2]Department of Dermatology, Hanyang University College of Medicine, Seoul, Republic of Korea

Melanin is produced by specialized organelles named melanosomes in melanocytes and the process is called as melanogenesis. The melanogenesis is regulated by many extrinsic and intrinsic factors including UV radiation, melanocyte stimulating hormone, endothelin 1, and a wide variety of growth factors and cytokines. Tyrosinase is the key enzyme in melanogenesis, initiating a cascade of reactions that regulates the rate-limiting steps. This study was conducted to understand the effect of natural extract (called as EP-2) from yeast, Saccharomyces cerevisiae, on melanogenesis and its underlying mechanism. In this study, EP-2 was not a direct tyrosinase inhibitor, while when EP-2 was applied to the B16F10 melanoma cell culture media, the intracellular tyrosinase activity was decreased. However, EP-2 has no effect on the expression of melanogenic proteins such as tyrosinase and microphthalmia-associated transcription factor. EP-2 was found that inhibit melanogenesis and melanosome transfer when it was treated to co-cultured melanocyte and keratinocyte. Protease-activated receptor 2 (PAR-2), a key protein associated with melanosome transfer from melanocyte to keratinocyte, was downregulated when EP-2 was treated to co-cultured melanocyte and keratinocyte. In conclusion, the findings herine indicate that EP-2 is a potent inhibitor of melanogenesis and its hypomelanogenic effect may be related to the inhibition of intracellular activity of tyrosinase and transfer of melanosome to keratinocyte.

P012

Inulavosin and its benzo-derivatives affect on copper-loading mechanism to tyrosinase, a key enzyme of melanin synthesis in melanocytes

H. Fujita[1], J. C. Menezes[2], S. M. Santos[3], S. Yokota[1], S. P. Kamat[4], J. A. Cavaleiro[2], T. Motokawa[5], T. Kato[5], M. Mochizuki[5], Y. Fujii[1], T. Fujiwara[1]

[1]Faculty of Pharmaceutical Sciences, Nagasaki International University/Faculty of Pharmaceutical Sciences, Sasebo, Japan; [2]Department of Chemistry and QOPNA; [3]Department of Chemistry and CICECO, University of Aveiro, Aveiro, Portugal; [4]Department of Chemistry, Goa University, Goa, India; [5]Cutaneous Drug Research Department, POLA Chemical Industries, Inc., Yokohama, Japan

In the recent cosmetic market, the identification and development of safe and natural skin-lightening compounds has attracted much attention. While large numbers of tyrosinase inhibitors have been reported, there is limited information on the intermolecular interactions between inhibitors and tyrosinase.

Inulavosin [2-(2′-hydroxy)-2,4′,4,4,7-penta methyl flavan] inhibits melanogenesis by enhancing a degradation of tyrosinase in lysosomes. However, the mechanism by which inulavosin redirects tyrosinase to lysosomes is not yet known. The analyses of structure-activity relationship of inulavosin and its benzo-derivatives, [2-(2,4,4-trimethyl-3,4-dihydro-2*H*-benzo[*h*]chromen-2-yl)-1-naphthol & 3-(2,4,4-trimethyl-3,4-dihydro-2*H*-benzo[*g*]chromen-2-yl)-2-naphthol], reveal that the hydroxyl and the methyl groups play a critical role in their inhibitory activity. Intriguingly, the hypopigmentation efficacy depends on the docking efficacy by measuring the cellular melanin contents using inulavosin and its benzo-derivatives in B16 melanoma cells. The theoretical docking studies to tyrosinase suggest that the inulavosin and its benzo-derivatives bind to the cavity of tyrosinase below which the copper binding sites are located. This cavity is proposed to be required for the association with 'Caddie protein', a chaperon that assists in copper loading to tyrosinase in *Streptomyces antibioticus*. We predict that inulavosin and its benzo-derivatives may compete with the mammalian homologue of caddie protein (mCaddie) and result in a lysosomal mistargeting of apo-tyrosinase that has a conformational defect. The docking study also suggest that inulavosin and its benzo-derivatives do not directly inhibit the

© 2014 John Wiley & Sons A/S. Published by John Wiley & Sons Ltd

tyrosinase activity, since the distance from the compounds to copper ions located in the catalytic center of tyrosinase is too large to inhibit substrate accessibility. This assumption is consistent with the results of in vitro assay of tyrosinase activity using the cell lysate prepared from the B16 cells cultured without any compounds. Neither inulavosin nor one of its benzo-derivatives reduced tyrosinase activities in vitro, since tyrosinase in the cell lysate was already loaded with copper. Identification and characterization of mCaddie protein in melanocyte is on going in order to conclude the mechanism of inulavosin and its benzo-derivatives. This is being carried out by an affinity purification of mCaddie as a binding protein of tyroisnase with anti-tyrosinase antibody. Inulavosin is used as specific competitors that specifically dissociate caddie protein from tyrosinase in vitro.

P013

UVA-induced oxidative stress promotes the degradation of melanosomes in vitro

T.-C. Lei

Dermatology, Wuhan University, Renmin Hospital, Wuhan, People's Republic of China

Melanin is produced inside small membrane-bound packages called melanosomes and is usually considered as a photoprotective pigment and antioxidant agent in epidermal cells. However, it is unclear as to how melanosomes transferred from donor melanocytes protect recipient keratinocytes against oxidative stress induced by UV radiation, particularly in proliferating basal and differentiated suprabasal keratinocytes, which has a critical implication for better understanding photocarcinogenesis. We rationalized that such membrane-bound organelle melanosomes need to release melanin through unrecognized degradation mechanism(s) because of the limited efficiency of melanin encysted in melanosome granules to scavenge and quench reactive oxygen species. To address these issues, we first analyzed the effects of UVA radiation, a known risk factor for skin cancer, on ultra-microstructural changes of naked melanosomes purified from MNT-1 cells using a transmission electron microscopy technique.

(i) Highly pigmented MNT-1 human melanoma cell line was generously provided by Dr. Vince Hearing (Pigment Cell Biology Section, NCI, NIH, USA); (ii) Each individual naked melanosome (stages III-IV) sample was prepared from cultured MNT-1 cells using a purification protocol; (iii) The naked melanosomes were resuspended in PBS and then exposed to 3J/cm^2 UVA radiation. In parallel, the melanosomes were also incubated with fresh medium containing 10 μM hydroquinone (HQ), 100 μM deoxy-arbutin, and 100 μM H_2O_2 at 37°C for 30 m, as well as treating with freeze/thaw cycles and manual grinding, serving as positive control. 4) The treated melanosomes were pelleted by centrifugation, washed with D-PBS, and fixed with 2% glutaraldehyde for cutting ultrathin section. The section was then stained with uranyl acetate and lead citrate and was examined with an electron microscope.

(i) The ultra-microstructural examination illustrated that typical stage III and IV melanosomes were visualized in purified melanosome fraction, of which has intact and well-organized structure of plasma membrane in untreated melanosome, whereas, the aberrant membrane structures such as fragmentation and even disintegration was clearly seen in UVA-irradiated melanosome; (ii) Similar membrane structural destructions were also observed in the melanosomes treated with 10 μM hydroquinone and 100 μM hydrogen peroxide (H_2O_2), Melanosome degradation intensity indicated that oxidative insults (UVA, HQ, and H_2O_2) is more efficacious than physical methods such as freeze/thaw cycles plus manual grinding to induce the destruction of melanosome membrane structure; (iii) 100 μM deoxy-arbutin barely destroys the membrane structural integrity of melanosomes, as compared with its parent compound HQ.

Oxidative stress might be an important factor in promoting melanosome degradation in keratinocytes under physiological conditions, which releases melanin pigment from melanosomes to scavenge and quench reactive oxygen species. Accelerated melanosome degradation in keratinocytes could also open new strategies to develop novel skin whitening agents. (This work was supported by grants from the National Natural Science Foundation of China (No. 81371717).

Key wordsmelanosome; degradation; UVR

P014

Effect of melanosome transport inhibitors on skin pigmentation

C. H. Myung[1], J. Park[1], K. R. Lee[1], E. J. Oh[1], J. S. Hwang[1,2]

[1]Graduate School of Biotechnology, Kyung Hee University, Yongin-si; [2]Skin Biotechnology Center, Kyung Hee University, Suwon, Republic of Korea

Melanocytes produce specific melanin-containing intracellular organelles called melanosomes. The movement of melanosome was mediated by the formation of a RAB27a-Melanophilin-MyosinVa protein complex. Melanosomes are transported from the perinuclear area of melanocytes toward the plasma membrane as they become more melanized and transferred to keratinocytes. The genetic defects of RAB27a, Melanophilin and Myosin Va resulted in melanosome aggregation and abnormal skin pigmentation. In this study we are trying to find chemical regulators on melanosome transport and know the effects of these chemicals on skin pigmentation. One of the strategies was to find novel compounds which inhibit binding interface between RAB27a and melanophilin. A pharmacophore model was built based on a modeled 3D structure of the protein complex that describes the essential binding residues in the intermolecular interaction. A pharmacophore model was employed to screen a chemical library database. Finally, 25 virtual hits were selected for biological evaluations. The biological activities of 11 analogues were evaluated in a second assay. Two compounds were identified as having concentration-dependent inhibitory activity. By analyzing structure-activity relationships of derivatives of BMD-20, two hydroxyl functional groups were found to be critical for blocking the intermolecular binding between RAB27a and melanophilin. We also screened natural compounds which inhibit melanosome transport using cell-based assay. we found that 2-methyl-naphtho [1, 2, 3-de]quinolin-8-one (MNQO) induced melanosome aggregation around the nucleus in Melan-a melanocytes and Melan-a melanocytes/SP-1 keratinocytes co-cultures without toxicity or altering melanin contents. In an attempt to elucidate the inhibitory mechanism of MNQO on melanosome transport, effect of MNQO on protein and mRNA expression of RAB27a, Melanophilin and MyosinVa were measured. The protein expression level of RAB27a, Melanophilin and MyosinVa were decreased by MNQO on Melan-a melanocytes. Also mRNA expression level of RAB27a, Melanophilin and MyosinVa were decreased. To know the effect of MNQO on skin pigmentation, reconstituted human epidermis model was used. Treatment of MNQO (0.001%) reduced skin

© 2014 John Wiley & Sons A/S. Published by John Wiley & Sons Ltd

pigmentation compared with control. These results showed that regulation of melanosome transport can be a good target for searching depigmenting agent.

P015

First evidence for glycosylation as a regulator of the pigmentary system: key roles of sialyl(alpha2,6)gal/galnac-terminated glycans in melanin synthesis and transfer

J. Pawelek[1], G. Diwakar[2], V. Klump[1], R. Lazova[1]

[1]Dermatology, Yale University School of Medicine, New Haven, CT; [2]Research, The Amway Corporation, Ada, MI, USA

Most proteins in melanogenesis and melanosome transfer to keratinocytes are glycoproteins and many studies have implicated glycosylation in the function of tyrosinase. But little is known of roles for glycosylation in other areas of the pigmentary system. We used a panel of 20 lectins as markers for specific oligosaccharides to survey binding patterns in human skin biopsies. Notably, Elderberry Bark Lectin (EBL) stained melanocytes and highlighted dendrites in both skin biopsies and melanocyte-keratinocyte co-cultures. EBL binds the sialylated sequence Neu5Ac(alpha2,6)Gal/GalNAc- at the termini of oligosaccharide antennae on some glycoproteins. We tested agents to inhibit the synthesis and/or binding function of this sequence to assess potential effects on pigmentation.

Cell culture, lectin histochemistry and assays for dopa oxidase and melanin were carried out by standard techniques

Cytidine, a sialyltransferase inhibitor, inhibited EBL binding and markedly reduced melanin production and melanosome transfer from melanocytes to keratinocytes. Similar effects were produced by 6′-sialyllactose, a short homolog and potential competitive inhibitor of the sequence in question. Unexpectedly, 3′-sialyllactose, chosen as a negative control because it is not recognized by EBL, also inhibited melanin production and transfer. Combinations of these agents showed Bliss additivity or in some cases synergism compared to the single agents ($P = \leq 0.01$ to $P = 0.06$). Interestingly, though strong inhibitors of melanization, none of the agents affected tyrosinase/dopa oxidase activity, indicating previously unrecognized post-tyrosinase regulation of melanin production.

We report for the first time that the Neu5Ac(alpha2,66)Gall/GaNAc-terminated oligosaccharides play a key role in melanogenesis and melanosome transfer. Inhibitors of this sequence should be useful tools for further studies in this new area of melanocyte biology as well as for treating disorders of hyperpigmentation.

P016 – P020: Intracellular & Metabolic Signaling

P016

Human melanocortin 1 receptor-mediated ubiquitylation and proteolysis of nonvisual arrestins

M. Abrisqueta, C. Olivares, J. Sirés, J. C. García-Borrón, C. Jiménez-Cervantes

University of Murcia, Murcia, Spain

The melanocortin 1 receptor (MC1R) is a major determinant of human skin pigmentation, hair color and skin cancer susceptibility. MC1R is a plasma membrane, Gs protein-coupled receptor (GPCR) responsible for the activation of the cAMP pathway by the melanocortin peptides αMSH and ACTH. Increased cAMP levels downstream of MC1R promote the synthesis of photoprotective eumelanins within cutaneous melanocytes. MC1R signaling to cAMP is regulated by intracellular proteins acting on most GPCRs, in particular the β-arrestins (ARRBs) and by more specific partners such as the E3-ubiquitin ligase mahogunin ring finger 1 (MGRN1). ARRBs are cytosolic proteins normally recruited to intracellular GPCRs domains upon receptor activation. Receptor-bound ARRBs uncouple active receptors from signal transducing G proteins (desensitization) and interact with clathrin and AP2 to trigger the sequestration of GPCRs away from the cell surface. MC1R undergoes homologous desensitization in melanoma and heterologous cells. Normal human melanocytes and melanoma cells were found to express ARRB1 and ARRB2, and both isoforms were able to bind MC1R even in the absence of agonists, in a competitive and mutually exclusive manner. However, the functional effects of ARRB binding to MC1R were isoform-specific, in that ARRB1 did not show any uncoupling activity whereas ARRB2 inhibited agonist-dependent cAMP production and promoted receptor internalization. Internalization of GPCRs depends on ubiquitylation of ARRBs that then efficiently attract endocytic cellular components. Expression of MC1R and ARRBs in HEK293T cells led to a sustained, αMSH-independent, ARRB1 and ARRB2 ubiquitylation. These results are in agreement with the constitutive activity reported for MC1R. High molecular weight ARRB forms were also detected in human melanoma cells, and their concentration strongly decreased upon si-RNA mediated silencing of MC1R expression. The native and active conformation of MC1R in the plasma membrane was necessary for ARRBs ubiquitylation, since hypomorphic MC1R variants and C-terminal deleted MC1R mutants with impaired forward trafficking were less active in promoting ARRB conjugation with ubiquitin. We also found that the MGRN1 interacts with the ARRBs. This interaction was strongly enhanced by co-expression with MC1R, suggesting occurrence of ternary complexes. Consistently, silencing of MGRN1 by si-RNA-mediated knockdown decreased ARRB ubiquitylation. Human ARRBs contain over thirty Lysine residues. We tackled the screening of potential MC1R-dependent ARRB2 ubiquitylation sites by site-directed mutagenesis. Analysis of the electrophoretic pattern of selected single Lys→Arg mutants and truncated forms suggests the occurrence of more than one ubiquitylation target. Finally, we show that although ARRBs are stable proteins, interaction ARRBs-MC1R specifically promotes differential regulated proteolysis of their N- and C-terminal ends, most probably compatible with a dominant-negative behavior of the resulting ARRB2 fragments, which could modulate the endocytosis process. In summary, we report that MC1R interacts with the major ARRB isoforms. ARRB2, but not ARRB1, actively participate in the internalization of the receptor upon multiple ubiquitylation, at least partially conducted by MGRN1. This modification leads to a regulated proteolysis of ARRB2, with the occurrence of fragments lacking short N-terminal or C-terminal peptides, whose physiological functions are under study.

Supported by grant SAF2012-32134 from MINECO, Spain and FEDER funds (European Community).

 © 2014 John Wiley & Sons A/S. Published by John Wiley & Sons Ltd

P017

N1-Acetyl-5-Methoxykynuramine (AMK) is produced in the epidermis from melatonin and has anti-proliferative effects on human keratinocytes and normal and malignant melanocytes

T.-K. Kim[1], Z. Lin[2], W. Li[2], A. T. Slominski[1]

[1]Departments of Pathology and Laboratory Medicine, University of Tennessee Health Science Center; [2]Pharmaceutical Sciences, University of Tennessee Health Science Center, Memphis, TN, USA

Our recent study has shown that cultured in vitro skin cells are able to metabolize melatonin into 6-hydroxymelatonin, N^1-acetyl-N^2-formyl-5-methoxykynuramine (AFMK) and 5-methoxytryptamine. In this study, we determined that AMK is endogenously produced in the human epidermis from melatonin through kynuric pathway. Its epidermal content (average from 13 subjects) is of 0.99 ± 0.21 ng/mg protein being significantly higher in African Americans (1.50 ± 0.36 ng/mg protein) than Caucasians ($.0.56 \pm 0.09$ ng/mg protein). It is especially high in young African Americans. However, the production does not significantly differ between males and females. In vitro testing using HaCaT keratinocytes has shown that exogenously added melatonin is metabolized to AMK in a dose dependent manner having V_{max} = 388 pg/million cells and K_m = 185 μM. The AMK production is higher in melanized than in amelanotic melanoma cells. Testing of DNA incorporation shows that AMK has anti-proliferative effects in HaCaT and SKMEL-188 cells (non-pigmented and pigmented) having, respective, EC_{50} values of 7.38×10^{-10}, 4.24×10^{-12} and 8.72×10^{-10} M. AMK also inhibits growth of normal melanocytes but has no significant effect on melanogenesis. These findings indicate that anti-proliferative effects of AMK are not related to melanin pigmentation. In summary, AMK is produced endogenously in the epidermis, being higher in pigmented individuals, and is a good candidates for further testing as therapeutics of hyper-proliferative disorders of the skin.

P018

Differences in expression of genes that control metabolism of co-cultured human melanocytes and keratinocytes. Modulation by exposure to UV or hydrogen peroxide

L. Marrot, L. Denat, M. Dutordoir, Y. Phalente, C. Jones

L'OREAL Research & Innovation, Aulnay sous bois, France

Skin is particularly exposed to oxidative stress and environmental insults such as sunlight and/or pollution known to impact metabolic homeostasis. In this study, the metabolic status of normal human keratinocytes and melanocytes from same donors was assessed in co-cultures at the transcriptional level. The basal expression of about 200 genes encoding phase I or phase II metabolism was compared in both cell types. Half of genes were equally expressed, although some significant differences were observed. For instance, for what concerns Nrf2 downstream genes, melanocytes displayed a higher expression of antioxidant genes such as NQO1 (quinone oxydoreductase), HO1 (heme oxygenase), Ferritin, whereas GPX (glutathione peroxidase) gene family was mainly expressed in keratinocytes. When cells were exposed to simulated solar UV, HO1 was induced in both cell types, but clear inductions (over twofold) were mostly observed in keratinocytes (e.g. GPX2, inflammation genes such as PSTG2, phase I genes such as CYP1A1, CYP1B1). Similar experiments were performed using hydrogen peroxide (H_2O_2) (100 or 200 μM): HO1 and Thioredoxin Reductase were induced in both cell types, but most overexpressions were noticed in keratinocytes (GCLC, NQO1, Ferritin, CYP1B1...). The kinetics back to basal expression level appeared faster in melanocytes than in keratinocytes. These results show that epidermal cells sharing the same genetic background and growing in the same culture medium display differences in basal and stress-induced expression of genes controlling metabolism. Melanocytes seem to be less sensitive to stress than keratinocytes. Even if pathways like Nrf2-ARE, AhR or inflammation are shared in response to H_2O_2 or solar UV, some modulations of stress genes appear specific to melanocytes and keratinocytes.

P019

Metabolic signature of sun exposed skin suggests catabolic pathways overweighs anabolic pathways

M. Randhawa[1], V. Sangar[2], S. Tucker-Samaras[1], M. Southall[1]

[1]Johnson & Johnson Skin Research Center, Johnson & Johnson Consumer Companies, Inc., Skillman; [2]Institute for Systems Biology, Seatle, WA, USA

Skin exposed to sun is susceptible to photooxidative damage along with long term detrimental effects like photoaging, which is characterized by wrinkles, loss of skin tone, and resilience. Photoaged skin displays prominent alterations in the cellular level and the extracellular matrix and has been studied very well through genomics and proteomic analysis. In contrast to genomics, metabolites, the end product are generated as a result of biochemical reactions are often studied as a culmination of complex interplay of gene and protein expression, have not been well studies in Photoaged skin. In this study we used a metabolomic approach to determine the differences in metabolome from full thickness skin biopsies extracted from sun exposed and sun protected sites from 25 human subjects. Biochemical pathway analysis revealed energy metabolism in photo exposed skin is predominantly anaerobic and the skin resides under high oxidative stress. Chains of reactions leading to production of these metabolites are inclined toward catabolism rather than anabolism, which persuade the skin cells to generate metabolites through the salvage pathway instead of *de novo* synthesis pathways. Metabolomic profile suggests the catabolic pathways and generation of reactive oxygen species operate in a feed forward fashion to alter the biology of sun exposed skin. Biochemical analysis also revealed altered energy metabolism in photoexposed skin with a shift from aerobic to anaerobic pathways, which are less energy efficient, hence resulting in energy deprived skin. The anaerobic environment of photoexposed skin may further contribute to the manifestation of photodamage and photoaging. Taken together, metabolomic investigations can provide a much greater representation of skin physiology beyond genomics and proteomics analysis.

© 2014 John Wiley & Sons A/S. Published by John Wiley & Sons Ltd

P020

Withaferin a abolishes the SCF-stimulated pigmentation of human epidermal equivalents by interrupting C-KIT autophosphorylation through dithiothreitol-suppressive michael addition thioalkylation reactions in human melanocytes

S. Terazawa[1], H. Nakajima[2], G. Imokawa[1]

[1]Research Institute for Biological Functions, Chubu University, Aichi; [2]School of Bioscience and Technology, Tokyo University of Technology, Tokyo, Japan

Withaferin A (WFA) is a bioactive steroidal lactone produced by a medicinal plant, *Ashwagandha*. Since an extract of *Ashwagandha* was found to elicit a potent inhibitory effect on stem cell factor (SCF)-stimulated MAPK signaling and on pigmentation in normal human melanocytes (NHMs) and of human epidermal equivalents (HEEs), respectively, we have now examined the mechanism(s) of the abrogating effect of WFA on the SCF-stimulated pigmentation of HEEs. When HEEs were cultured in DMEM medium supplemented with SCF (at 5 nM) and were treated with or without WFA (at 12.5, 25 and 50 nM) for 14 days, the addition of WFA (at least at 50 nM) elicited a marked abrogating effect on the SCF-stimulated pigmentation of HEEs after 14 days of culture. That effect was accompanied by a diminished visible pigmentation and decreased eumelanin (PTCA) content but not by a cytotoxic effect assessed by the MTT assay compared with control HEEs treated with SCF only. Real-time RT-PCR and western blotting analyses revealed that increased gene and protein expression levels of tyrosinase, tyrosinase-related protein-1, dopachrome tautomerase, PMEL17, c-KIT and their targeted transcription factor, microphthalmia-associated transcription factor (MITF) were significantly reversed at days 7 and 10, respectively, by treatment with WFA (at 50 nM). Enzymatic analysis of SCF-stimulated NHMs revealed that WFA has no direct inhibitory effect on tyrosinase activity in vitro, but pretreatment with WFA abolished the SCF-stimulation of tyrosinase activity in NHMs. Western blotting of potential intracellular signaling intermediates revealed that in WFA-treated NHMs, there was a marked deficiency in the SCF-stimulated series of phosphorylations of c-KIT, Shc, Raf-1, MEK, ERK, MITF and CREB compared with untreated NHMs, which indicates that WFA interrupts the initial activation of the c-KIT receptor. Treatment with dithiothreitol (DTT) significantly abolished the suppressive effect of WFA on the SCF stimulated phosphorylations of c-KIT, MITF and CREB in NHMs. On the other hand, even after incubation at 4°C for 2 h with 5 nM SCF, followed by the removal of free unbound SCF by washing and then raising the temperature to 37°C to start the signaling reaction, c-KIT was distinctly phosphorylated by incubation for 5 min at 37°C, followed by the phosphorylation of MITF and CREB to a similar extent by treatment with SCF only or SCF+WFA, which suggests that WFA does not interrupt the binding of SCF to the c-KIT receptor. These findings indicate that WFA attenuates the SCF binding-induced activation of c-KIT in NHMs by directly inhibiting the auto-phosphorylation of c-KIT tyrosine kinase through DTT-suppressible Michael addition thioalkylation reactions. That effect abrogates the SCF-stimulated expression of melanocyte-specific proteins, including MITF, which abrogates the SCF-stimulated pigmentation of HEEs.

P021 – P035: Regulation of Pigmentation

P021

Molecular characterization of the whitening effect of Tranexamic Acid Cetyl Ester (TXC) using human melanoma and primary melanocytes

N. Ando[1], R. Singh[2], S. Kaul[2], N. Nigam[2], C. Mahe[1], R. Wadhwa[2]

[1]KK Chanel Research and Technology Development Laboratory, Funabashi-Chiba; [2]Cell Proliferation Research Group and DBT-AIST International Laboratory for Advanced Biomedicine, National Institute of Advanced Industrial Science & Technology (AIST), Tsukuba, Japan

Understanding and manipulation of melanogenesis process in specialized skin cells, melanocytes, are extremely important aspects of functional cosmetics that aims to improve skin texture, coloration, and safety from harsh environmental conditions and toxins. Several whitening ingredients have been commercialized that work through manipulation of tyrosinase, a key enzyme involved in the process of melanogenesis. We have developed Tranexamic acid (TXA) derivative, trans-4-(Aminomethyl) cyclohexanecarboxylic acid hexadecyl ester hydrochloride (TXC) as a quasi-drug substance. It possesses hydrophobic characteristics. TXC was shown to inhibit melanin synthesis in vitro and in vivo and was effective in reducing UV-induced pigmentation. However, its precise mechanism of action under stressed and unstressed conditions has not been elucidated. In the present study, we have investigated the effect of TXC on melanogenesis in human primary melanocytes and melanoma that retain the capacity of melanin synthesis. Using OAG (diacylglycerol (DAG) 1 -oleoyl-2-acetyl-sn-glycerol)-induced melanogenesis as an assay system, we found that the cells treated with TXC show reduced level of OAG-induced melanin. Such reduction in melanin was associated with reduction in tyrosinase enzyme, DNA damage response protein gammaH2AX and oxidative stress marker, ROS. Primary melanocytes when treated with TXC showed clear protection against OAG-induced pigmentation, DNA and oxidative stress, suggesting the potency of TXC as a 'Treatment × Care' reagent.

P022

Functional characterization of a natural human melanocortin 1 receptor hypomorphic mutant with premature truncation of the C-terminal cytosolic domain

J. Sirés[1], R. Yousaf[2], S. Riazuddin[2], C. Jiménez-Cervantes[1], Z. M. Ahmed[2], J. C. García-Borrón[1]

[1]University of Murcia, Murcia, Spain; [2]Cincinnati Children's Hospital Research Foundation, Cincinnati, OH, USA

The melanocortin 1 receptor (MC1R) is a Gs protein-coupled receptor (GPCR) expressed on the cell surface of melanocytes and crucial for the regulation of proliferation and function of this cell type. Upon binding melanocortins, MC1R activates the cAMP pathway, ultimately leading to synthesis of photoprotective eumelanins. Conversely, impaired cAMP synthesis is associated with pheomelanogenesis. Thus, differences in MC1R signaling potential resulting from *MC1R* gene polymorphisms are a major source of normal variation of human skin pigmentation, hair color and skin cancer susceptibility. The MC1R is a 317 amino acid integral transmembrane protein with the canonical GPCR structure consisting of seven transmembrane fragments, a glycosylated

 © 2014 John Wiley & Sons A/S. Published by John Wiley & Sons Ltd

extracellular N-terminus, and a cytosolic C-terminal extension. The C-terminal domain may play an important role in receptor trafficking, functional coupling and regulation of signaling. A natural p.Arg306* mutation with premature truncation of the C-terminal tail has been found in domestic dogs, with homozygous animals showing pheomelanic coats. However, comparable mutations have not yet been described in humans. Here we report the functional characterization of a similar nonsense mutation of human MC1R causing the premature truncation of the C-terminal nonadecapeptide. This mutation was found in the course of the molecular screening of a large consanguineous family with hypopigmentation of the skin and hair, performed by whole exome enrichment and massive parallel sequencing, followed by segregation analysis. In the mutant protein (p.Tyr298*) deletion of the complete cytosolic extension abolished functional coupling to the cAMP pathway, with residual activity lower than observed for most hypomorphic natural alleles, including p.Asp294His. The electrophoretic pattern of the p.Tyr298* and wildtype (WT) proteins was similar, indicative of comparable post-translational glycosylation and oligomerization. However, the steady state levels of the mutant protein in transiently transfected cells was slightly lower than WT and its intracellular half-life was also shorter, suggesting sensitivity to proteolytic digestion and a faster turnover. Co-immunoprecipitation experiments performed with differentially labeled WT MC1R and p.Tyr298* indicated the presence of heterodimeric WT-p.Tyr298* species. Unexpectedly, expression of the p.Tyr298* variant on the cell surface was detected by ELISA in non-permeabilized heterologous cells, using an antibody directed against the extracellular N-terminus of the receptor. Significant plasma membrane localization of p.Tyr298* was confirmed by laser scanning confocal microscopy, thus challenging the current view of the role of the MC1R C-terminus in forward trafficking through the biosynthetic-secretory pathway.

Supported by grants SAF2012-32134 (to JCGB) from the MINECO, Spain and FEDER funds (European Community), and RPB funds (ZMA). JSC holds a fellowship from the MINECO, Spain.

P023

Myo-inositol suppresses melanogenesis via elevation of cell energy in keratinocytes

T. Hakozaki[1], T. Laughlin[1], S. Zhao[1], D. Deng[2], B. Jewellmotz[1], L. Moulton[1]

[1]The Procter & Gamble Company, Cincinnati, OH, USA; [2]The Procter & Gamble Company, Singapore City, Singapore

Myo-inositol (MI), sometimes referred to as vitamin B8, is prevalent in many plants. It plays key roles in human body such as secondary messengers and as a component of structural lipids such as phosphatidylinositol. It is involved in various biological processes such as insulin signal transduction, cell membrane maintenance, breakdown of fats and reducing blood cholesterol, but no reports are available for the effect on skin pigmentation.

We investigated the effect of MI on melanogenesis by utilizing melanocyte and keratinocyte cell culture models. The treatment of MI on melanocyte cell culture did not affect melanin production. However, gene expression profiling analysis with MI treatment on human keratinocytes indicated that MI stimulated lipid utilization and activated fatty acid beta-oxidation pathway, leading to increased ATP (Adenosine triphosphate) production in keratinocytes. Addition of MI in the human keratinocyte cell culture model significantly elevated ATP level, confirming the results of gene expression profiling analysis.

We then evaluated the impact of ATP modulation on the release of melanogenic cytokines from keratinocyte. Addition of phenformin or AICAR (5-Aminoimidazole-4-carboxamide ribonucleotide), which are known AMP kinase activators (increase ATP levels), significantly reduced the release of endothelin-1 (ET-1) by 13% measured by ELISA kit. On the other hand, addition of 6-[4-(2-piperidin-1-ylethoxy)phenyl]-3-pyridin-4-ylpyrazolol[1,5-a]pyrimidine (AMP kinase inhibitor) increased ET-1 release by 22%. Addition of MI also reduced ET-1 release by 19% in keratinocyte, confirming the potential relationship between ATP production and ET-1 release in keratinocyte. Seven days treatment of MI in a human skin explant model also confirmed suppression of gene expression of ET-1, together with the suppression of TRP1 and DCT genes.

Lastly, we evaluated the effect of MI-containing formula on facial hyperpigmented spots among Chinese females in a randomized, double-blinded clinical study. After 8 weeks of treatment, the MI-containing formula significantly reduced the appearance of facial spots versus matched control ($P = 0.038$). These data indicated MI is effective in reducing the appearance of facial hyperpigmentation, and one of its mechanisms of action can be attributed to reducing ET-1 release via upregulation of ATP in keratinocyte.

P024

Evaluation of melanogenesis inhibitory agent by a novel human melanocyte-keratinocyte co-culture system

Y. Higashi[1], M. Yagi[2], M. Ichihashi[3], H. Ando[1]

[1]Okayama University of Science, Okayama; [2]Rosette Co., Tokyo; [3]SAISEI MIRAI Clinic Kobe, Kobe, Japan

The co-culture system composed of normal human melanocytes and keratinocytes is recently reported to be useful to study the effect of keratinocyte-derived cytokines on melanogenesis via paracrine fashion. Since the alteration of melanin synthesis in co-culture system using human melanocytes is not so appreciable compared to murine melanoma cells, it is requested to detect a small difference of melanin content precisely without interference of the mixed keratinocytes. Here we developed a novel human melanocyte-keratinocyte co-culture system separated by a microporous membrane filtered trans-well that enables the isolation of melanocytes from the co-cultured keratinocytes. We evaluated the efficacy and sensitivity of our novel co-culture system in melanogenic activity using the rose fruit-extract which is already known to have anti-melanogenic activity of murine melanoma cells. After 6 days co-culture with medium containing the rose fruit-extract at 10 μg/ml, melanocytes were harvested without contamination of keratinocytes. Intracellular melanin content was measured by spectrophotometric absorbance and tyrosinase amount was detected by Western blotting analysis. The rose fruit-extract decreased melanin content to around 2/3, and tyrosinase amount to around 1/3 of the control, respectively. However, in the melanocytes in mono-culture incubated with rose fruit-extracts, neither intracellular melanin content nor tyrosinase amount was decreased to the level of statistical difference. These results indicate that our novel trans-well separated co-culture system of normal human melanocytes and keratinocytes is a sensitive and useful method for the quantitative evaluation of melanogenesis regulatory factors which mostly operate in keratinocytes and regulate melanogenesis of melanocytes in paracrine fashion.

© 2014 John Wiley & Sons A/S. Published by John Wiley & Sons Ltd

P025

A study on the mechanism of stimulating effect of *Cryptanthus* on mouse melanoblast

M. J. Kang[1], J. W. Byun[1], H. W. Park[2], H. B. Lee[2], E. K. Kim[2], J. Shin[1]

[1]Department of dermatology, Inha University School of Medicine; [2]Department of Biological Engineering, Inha University, Incheon, Republic of Korea

Vitiligo is an acquired hypomelanotic disorder characterized by hypomelanotic macules of various sizes which is caused by the loss of melanocytes. Current treatment modalities are directed towards restoring of pigmentation by stimulating melanocytes, however melanocytes are missing in the vitiligo lesions which attributes to the unsatisfactory treatment results. However, melanoblasts are present in the outer root sheath of hair follicles and these cells may serve as melanocyte reservoir and provide sources for repigmentation. In a screening experiment to find potent melanogenetic herbal medicine, extract of *Cryptanthus* was found to be effective in inducing melanogenesis and migration in melanocytes and melanoma cells. In this study, different concentrations of extract of *Cryptanthus* were added to melb-a mouse melanoblast to study effect on differentiation, migration and viability. Melanin content and cell viability were measured and a transwell migration assay was done. The results were compared to that of negative control treated with 10.0 μg/l of DMSO and positive control treated with 10.0 μg/l of α-MSH. Also, in order to elucidate the signaling pathway, phosphorylation and expression of extracellular signal-regulated kinase(ERK) and cAMP response element binding protein(CREB) were evaluated using western blotting analysis. Melanin content in *Cryptanthus* treated melb-a mouse melanoblast was increased in a dose-dependent manner and 130% increase compared to α-MSH treated cells without affecting cell viability. Cell migration showed 203% increase after treatment with *Cryptanthus* compared to α-MSH. Western blot revealed that phosphorylation of CREB and ERK were increased after treatment with *Cryptanthus* extract. In conclusion, *Cryptanthus* seems to induce differentiation of mouse melanoblast through activation of ERK and CREB pathway and it may be a good candidate for vitiligo treatment.

P026

Adam protease inhibitor modulates melanogenesis in human melanocytes

M. Kawaguchi, Y. Hozumi, T. Suzuki

Department of Dermatology, Yamagata University, Fuculty of medicine, Yamagata, Japan

The ADAMs (a disintegrin and metalloprotease) are the family of proteases involved in ectodomain shedding. These molecules are involved in various biological processes such as cell adhesion, cell migration, and membrane protein shedding. ADAM17 plays a critical role in the ectodomain shedding of many soluble proteins, including TNF-α, TNF receptor, epidermal growth factor receptor ligands, KIT ligand (KITL), and its receptor KIT. KITL/KIT play important roles in melanocyte development, survival, proliferation, and melanogenesis. Recently, *Adam17* knockout mice revealed a disorganized distribution and structure of hair follicles, which contain hairs with irregular pigment deposition. In humans, *ADAM17* is also known as candidate gene that regulates pigmentation in East Asians. ADAM10 is involved in the ectodomain shedding of various substrates, including adhesion molecules such as CD44, E-cadherin, and N-cadherin. Recently, mutations in *ADAM10* were identified as a cause of reticulate acropigmentation of Kitamura characterized by reticulate, slightly depressed pigmented macules mainly affecting the dorsa of the hands and feet. We therefore examined the effect of ADAM protease inhibitor on the modulation of melanogenesis in normal human epidermal melanocytes (NHEM). In NHEM, melanin content was reduced by the treatment with ADAM protease inhibitor. The inhibitor was not involved in the protein expression of tyrosinase, Melan A, or MITF. To investigate the possible effect of the inhibitor on cAMP-induced melanogenesis, we treated B16F10 melanoma cells with α-MSH in the presence of ADAM protease inhibitor. Pretreatment of cells with the ADAM protease inhibitor diminished the α-MSH-induced increase of melanin content. Electron microscopic examination revealed that the number of melanosomes was significantly reduced by the treatment with ADAM protease inhibitor. In addition, NHEM treated with ADAM protease inhibitor harbored vacuolar compartments filled with dense unstructured aggregates, and demonstrated a dramatic impairment of structural parallel sheets, with a decrease in the number of fibrillar melanosome. These result indicated that ADAM protease inhibitor contributed to the formation of fibril and their assembly into sheet in melanosomes. We therefore investigated the processing of pmel17. Pmel17 is a melanosomal glycoprotein that forms a fibrillar matrix, on which the melanin gets deposited. Proteolytic processing of pmel17 is required to form functional fibrils during melanogenesis. In the cells treated with ADAM protease inhibitor, C-terminal fragment of pmel17 accumulated and N-terminal fragment of pmel17 was decreased. These results suggest that processing of pmel17 is affected by the ADAM protease inhibitor.

P027

Myriocin, a serine palmitoyltransferase inhibitor, increases melanin synthesis in Mel-Ab cells and a skin equivalent model

D.-S. Kim[1], H. Li[1], M. C. Balcos[1], J. S. Shin[1], H.-Y. Yun[1], K. J. Baek[1], N. S. Kwon[1], K.-C. Park[2]

[1]Department of Biochemistry, Chung-Ang University College of Medicine, Seoul; [2]Department of Dermatology, Seoul National University Bundang Hospital, Seongnam, Republic of Korea

The purpose of this study was to investigate effects of myriocin, an inhibitor of serine palmitoyltransferase, on melanogenesis. Myriocin had no cytotoxic effect on Mel-Ab cells at concentrations <10 μM. It was found that myriocin increased melanin synthesis in a concentration-dependent manner. Moreover, myriocin upregulated microphthalmia-associated transcription factor (MITF) and tyrosinase expression via phosphorylation of CREB, but it did not directly activate tyrosinase, a rate-limiting melanogenic enzyme. Importantly, only phospho-CREB was increased time-dependently, while Akt, GSK-3β, and p-38 were not phosphorylated and β-catenin level was not changed. Furthermore, we demonstrated increased melanin synthesis with myriocin on a pigmented skin equivalent model established using *Cervi cornus Colla* (deer antler glue). One and 5 μM of myriocin darkened the color of the skin equivalent. Moreover, the skin equivalent section was stained by Fontana-Masson to detect melanin. Melanin pigment was detected at a greater level in the myriocin-treated skin equivalent than in the untreated control. In conclusion, myriocin promoted melanogenesis through phosphorylation of CREB and increased MITF and tyrosinase levels. Furthermore, the pigment-inducing effect of myriocin was confirmed in a skin equivalent

© 2014 John Wiley & Sons A/S. Published by John Wiley & Sons Ltd

model containing Mel-Ab cells. Therefore, we suggest that myriocin could be used for the treatment of vitiligo or sunless tanning. This study was supported by a grant of the Korea Healthcare Technology R&D Project, Ministry of Health and Welfare, Republic of Korea. (Grant No. A103017).

P028

I KHG26792 inhibits melanin synthesis in Mel-Ab cells and a skin equivalent model

D.-S. Kim[1], H. Li[1], J. Kim[1], H.-G. Hahn[2], J. Yun[2], H.-S. Jeong[1], H.-Y. Yun[1], K. J. Baek[1], N. S. Kwon[1], K.-C. Park[3]

[1]Department of Biochemistry, Chung-Ang University College of Medicine; [2]Organic Chemistry Laboratory, Korea Institute of Science & Technology, Seoul; [3]Department of Dermatology, Seoul National University Bundang Hospital, Seongnam, Republic of Korea

The purpose of this study is to characterize the effects of KHG26792 (3-(naphthalen-2-yl(propoxy)methyl)azetidine hydrochloride), a potential skin whitening agent, on melanin synthesis and identify the underlying mechanism of action. Our data showed that KHG26792 significantly reduced melanin synthesis in a dose-dependent manner. Tyrosinase activity was measured in a cell-free system after the addition of KHG26792. However, KHG26792 did not block tyrosinase directly. Instead, KHG26792 downregulated microphthalmia-associated transcription factor (MITF) and tyrosinase, the rate-limiting enzyme in melanogenesis. KHG26792 activated extracellular signal-regulated kinase(ERK), whereas an ERK pathway inhibitor, PD98059, rescued KHG26792-induced hypopigmentation. These results suggest that KHG26792 decreases melanin production via ERK activation. Moreover, the hypopigmentary effects of KHG26792 were confirmed in a pigmented skin equivalent model using *Cervi cornus Colla* (deer antler glue), in which the color of the pigmented artificial skin became lighter after treatment with KHG26792. In conclusion, KHG26792 exhibited skin whitening effects both in a monolayer cell culture model and a pigmented skin equivalent model, most likely through the activation of ERK. Moreover, the pigmented skin equivalents developed in this study could be used as a general model for testing skin whitening agents. This study was supported by a grant of the Korea Healthcare Technology R&D Project, Ministry of Health and Welfare, Republic of Korea. (Grant No. A103017).

P029

Mode of action of rhododendrol, raspberry ketone, 4-butylresorcinol and other inhibitors of human tyrosinase

T. Mann[1], W. Gerwat[1], H. Wenck[1], K.-H. Röhm[2], L. Kolbe[1]

[1]R&D Frontend Innovation, Beiersdorf AG, Hamburg; [2]Institute of Physiological Chemistry, Philipps University, Marburg, Germany

Melasma, actinic lentigines and post-inflammatory hyperpigmentation are major cosmetic concerns. Various strategies have been proposed to reduce unwanted melanin production in human skin. The most prominent target for inhibitors of hyperpigmentation is tyrosinase, the key enzyme of melanin production. In addition, inhibitors of melanocyte activity, compounds interfering with the regulation of tyrosinase expression, and agents reducing melanosome transfer to keratinocytes were also described as effective drugs for the treatment of hyperpigmentary disorders. Still, due to their immediate and reversible mode of action, selective tyrosinase inhibitors are considered not only most effective but also very safe reducers of hyperpigmentation.

Recently, cosmetic products containing the natural compound rhododendrol [4-HBP; 4-(p-hydroxyphenyl)butan-2-ol] were recalled because of induction of severe leucoderma in many customers. To analyze potential reasons for the side effects of rhododendrol and its oxidized derivative raspberry ketone [4-(p-hydroxyphenyl)butan-2-one], we compared the in vitro effects of these compounds on purified human tyrosinase and on melanoDerm skin models with those of 4-butylresorcinol and other known tyrosinase inhibitors. To our surprise, rhododendrol only weakly inhibited human tyrosinase (IC_{50} > 1200 μM) while raspberry ketone was essentially ineffective with maximum inhibition of <20% at 1 mg/ml. In contrast, 4-butylresorcinol was a very effective competitive inhibitor of human tyrosinase with an IC_{50} of 21 μM. In melanoDerm skin models, rhodendrol only marginally reduced melanin production with maximum effects of around 20% at 50 μM. Raspberry ketone was even less active with maximum inhibition of about 10% at 50 μM. Again, 4-butylresorcinol turned out to be a potent inhibitor of in vivo melanin production with an IC_{50} of 13.5 μM.

In silico modeling studies of ligand binding to homology models of human tyrosinase also predicted that both rhododendrol and raspberry ketone should be very poor inhibitors of the enzyme with binding affinities at least 100-fold lower than those of 4-butylresorcinol and other high-affinity resorcinol derivatives. From these results we conclude, that the unwanted effects of rhododendrol and raspberry ketone are not mediated by direct inhibition of the human tyrosinase protein.

P030

Pintoid dyschromia of yaws: a rare presentation of a chronic infectious disease

A. P. W. W. Kumarasinghe[1], S. P. W. Kumarasinghe[2]

[1]General Medicine, Sir Charles Gairdner Hospital, Nedlands; [2]Dermatology, Royal Perth h Hospital, Perth, WA, Australia

Yaws is a rare condition today in most countries. Rarely sequelae of previous yaws infections are noted in patients with a history of yaws. We describe a 53 old man who is originally from the West Indies, presenting with multiple ill defined asymptomatic hypopigmented, normosensitive macules on the on the body, developing gradually over a few years. He did not have any recent history of preceding inflammation or pruritus in these areas. Hypopigmented macules were several centimeters in diametre; they were most prominent on his scalp, upper limbs and upper trunk. There were keratotic plaques on both feet. He had a history of increased titres of syphilis serological tests, he had been treated on suspicion of having had syphilis. There was no history of syphilitic chancres or any clinical features of secondary syphilis. On direct questioning he admitted that he had yaws as a child when he was living in Jamaica, the West Indies, where yaws was endemic at that time. It had been treated with home remedies according to him. There was no history of pinta. A skin biopsy was done from a hyperkeratotic lesion on a foot. It showed non specific hyperkeratosis without any evidence of psoriasis or eczema, it was not diagnostic but consistent with hyperkeratotic lesions of yaws. Biopsies from the hypopigmented macules did not show any evidence of leprosy, vitiligo, hypopigmented mycosis fungoides or eczema, it showed non specific perivascular and perifolliciular lymphocytic infiltration. Serological tests for treponemal infection (Treponema pallidum particle agglutination Assay 4+ and negative rapid plasma reagin test) were positive. As there is no method to

© 2014 John Wiley & Sons A/S. Published by John Wiley & Sons Ltd

distinguish between syphilis and previous yaws, he was treated again with a full course of penicillin injections as for syphilis. A diagnosis of pintoid dyschromia of yaws was made. As he was mostly concerned about the pigmentary changes of skin, narrowband ultraviolet B therapy was tried for 3 months. He had only a very slight improvement of the macules. As such NBUVB treatment was discontinued.

We present this case to highlight that yaws should still be considered in hypopigmented macules in a patient with a past history of yaws, or is from an area previously endemic for yaws, with a positive TPPA and sequelae consistent with yaws.

P031

Novel role of adrenomedullin in melanocyte dendrite branching

T. Motokawa[1], T. Miwa[1], M. Mochizuki[1], M. Itou[1], K. Yokoyama[1]
[1]Pola Chemical Industries, Inc., Yokohama, Japan

The shape of melanocytes and number of dendrites, as well as melanin synthesis, are thought to be critical for melanin accumulation in human skin. A melanocyte has several dendrites, which allows one melanocyte to distribute melanin to many keratinocytes. Although previous studies demonstrated that paracrine factors such as prostaglandin E2 increased dendrite numbers, the effect seemed to be insufficient for melanocytes to make contact with a large numbers of cells. We thought there are yet undiscovered factors that influence dendrite branching and some might play a pivotal role in melanin accumulation.

The aim of this study was to identify a novel dendrite-branching factor and to demonstrate its importance in melanin accumulation in vitro and in vivo.

HypothesisWe hypothesized that novel dendrite branching factors could be identified in the ultraviolet B (UVB)-irradiated human skin model (consisting of human keratinocytes and melanocytes) because cells with branching dendrites have been observed in this model.

Methods and Results

1. In vitro study: First, we developed a human skin co-culture model that is irradiated twice with UVB to increase the number of dendrites of cells. We carried out a microarray analysis using this model to detect candidate genes. Among the candidates, we identified adrenomedullin (ADM) as a novel dendrite-branching factor using a recombinant peptide assay. This effect was confirmed by knockdown studies with *ADM* siRNA. A relatively higher expression of *ADM* mRNA and ADM peptide were found in UVB-irradiated keratinocytes and in their culture supernatant, respectively. We also found that media containing ADM peptide not only increased the number of dendrites of melanocytes by two-fold, but also increased melanin synthesis by two-fold compared with the effects of the control medium.
2. In vivo study: We performed immunostaining studies of pigmented lesions and UV-induced pigmentation in human skin. ADM was present in the epidermis and was increased in pigmented lesions compared with perilesional skin. ADM protein was highly expressed in UV-irradiated human skin in areas of erythema (after 2 days of UVB irradiation) and pigmentation (after 5 days of UVB irradiation).

Discussion and Conclusion We identified adrenomedullin as a novel dendrite-branching factor using an UVB-irradiated human skin model. ADM is known as a hypotensive factor that exists in numerous organs including the skin, but its association with melanin accumulation has not been reported. In vitro and in vivo studies suggested that ADM is a paracrine factor secreted by keratinocytes.

Since ADM induced dendrite branching and melanin synthesis, and higher expression of ADM was observed in both pigmented lesions and UVB-irradiated areas in human skin, ADM is thought to play a pivotal role in melanin accumulation in human skin.

P032

Expression of clock proteins clock and PER1 in human skin melanocytes, and the impact of alternate light and dark cycles in vitro

D. J. Tobin[1], A. Richharia[1]
[1]Centre for Skin Sciences, University of Bradford, Bradford, UK

Circadian rhythms are endogenously-driven, oscillating cycles (physiological and behavioural) with a periodicity of ~24 h. Given the strategic location of the skin and its appendages at the interface of the body's internal and external environments, it is perhaps not surprising that the skin also transcribes core clock genes, whose expression may be regulated by a most striking of circadian cues - sunlight. Both CLOCK and Per1 are part of a transcription-translation negative feedback mechanism that creates a cell-autonomous molecular clock. In this preliminary study we examined the in situ expression of the circadian proteins CLOCK (Circadian Locomotor Output Cycles Kaput, positive limb) and PER1 (Period circadian protein homolog 1, negative limb) in cutaneous melanocytes, and studied the effect of alternating light and dark cycles on the expression of CLOCK and PER-1 proteins in normal healthy human epidermal melanocytes in vitro.

Human haired scalp was obtained from five young and old healthy donors, while epidermal melanocytes were isolated and cultured from the epidermis of a healthy young donor. The effect of light and dark on the expression of CLOCK and PER1 proteins in epidermal melanocytes was determined by incubating melanocytes in either constant darkness or constant light (from LED light source) for 50 h before immunostaining under light or dark conditions.

CLOCK and PER1 were variably expressed in melanocytes located in the epidermis and hair bulb. Under standard culture conditions a predominantly nuclear expression of PER1 was observed in cultured epidermal melanocytes. A more mixed patter pattern of cytoplasmic/nucler expression was observed for CLOCK. However, while melanocytes exposed to constant darkness expressed CLOCK immunoreactivity in a cytoplasmic manner, this positive-limb protein translocated to the nucleus when the melanocytes were switched to constant light incubation conditions. By contrast, the expression of the negative limb circadian protein PER1 was exactly opposite to that of CLOCK under these alternating conditions. Thus, under dark conditions PER1 was found predominantly in the melanocyte nucleus but was expressed in the cytoplasm when incubated under conditions of light. It is known that a shift in the light/dark cycle evokes a proportional shift of Per1 gene expression in the suprachiasmatic nucleus of the brain (main body pacemaker) and that the timing of Per1 gene expression is sensitive to light. Thus, these preliminary data may suggest that light is an important cue in the circadian oscillations in pigment cells, and that disturbance in the light/dark cycle may have implications for the functioning of the epidermal and hair follicle melanin units.

 © 2014 John Wiley & Sons A/S. Published by John Wiley & Sons Ltd

P033

Integrated cell based shrna screening and bioinformatics approaches identified mitochondrial stress chaperones HSP60 and mortalin as new regulators of melanogenesis

R. Wadhwa[1], N. Widodo[1], N. Shah[1], N. Nigam[1], T. Yaguchi[1], N. Ando[2], C. Mahe[2], S. Kaul[1]

[1]Cell Proliferation Research Group and DBT-AIST International Laboratory for Advanced Biomedicine, National Institute of Advanced Industrial Science & Technology AIST, Tsukuba; [2]KK Chanel Research and Technology Development Laboratory, Funabashi-Chiba, Japan

Melanogenesis is the process through which melanocytes (constitute about 10% of cells in the basal layer of epidermis) produce melanin pigment that determines skin coloration from fair to dark. Besides it relevance in cosmetics, melanin signaling is important to protect skin from adverse environmental conditions. Once synthesized, it is stored in special structures called melanosomes and shipped to the top layer of keratinocytes by the arm-like structures called dendrites. Since individuals with low level of endogenous pigmentation are known to have high incidence of skin cancers, agents that induce melanogenesis hold potential as skin-protection against UV and carcinogenesis. On the other hand, agents that induce depigmentation are valuable for (i) treatment of moles, dark spots that appear on aging skin and (ii) skin whitening as desired in cosmetics.
Tyrosinase enzyme is an established key regulator in production of melanin from tyrosine; the process is highly influenced by hormones, intra- and extra-cellular environment by complex signaling cross talks in which the role of cellular factors has not been clearly established. In order to identify the cellular factors involved in human melanogenesis, we used shRNA-mediated loss-of-function screening in conjunction with induction of melanogenesis by OAG (diacylglycerol (DAG) 1 -oleoyl-2-acetyl-sn-glycerol) in human melanoma G361 cells. Cells were transfected with shRNA library (2044 gene targets) and assayed for induction of melanogenesis by multi-dimensional approaches involving quantitative biochemical and visual determination of the melanin content and tyrosinase activity. Gene targets of the shRNAs that led to the loss of OAG-induced melanogenesis were considered as candidate cellular factors crucial for melanogenesis. By four rounds of screenings, we identified 40 gene targets. Bioinformatics and pathway analyses revealed that these gene targets are involved in the regulation of cell proliferation, apoptosis, stress response and mitochondrial functions. Based on these data, the role of mitochondrial stress proteins in melanogenesis is discovered. We demonstrate that the mitochondrial stress chaperones, Hsp60 and mortalin/mtHsp70, are strong regulators of melanogenesis and hence could be the targets for cosmetics and therapeutic manipulation.

P034

The prevalence of skin whitener usage in Northern India and its related dermatological complications

C. Wong[1], R. Minocha[2], N. Sharma[3], R. Dunn[1,4], C. Grills[1], N. Grills[5]

[1]Skin and Cancer Foundation Inc, Melbourne, Vic.; [2]Dermatology Department, Westmead Hospital, Westmead, NSW; [3]Dermatology registrar, Monash Health; [4]Consultant, Royal Melbourne Hospital; [5]The Nossal Institute, University of Melbourne, Melbourne, Vic., Australia

Within India, the social, cultural and anthropological importance of fair skin date back thousands of years where prosperity and beauty were relayed by a light complexion. Our literature review on the use and impact of skin lightening products revealed they account for 61% of the dermatology cosmetic market within India.[i]
We surveyed 92 women who resided within villages in Northern India about their extent of skin whitener usage, route of use, and the observed cutaneous and systemic side effects. 17% (16/92) of women that we surveyed used skin whiteners. All of whom commented that their extent of use increased prior to a social event. All women reported using ‘Fair and lovely’ as the skin whitener of choice. One also used a ‘Garnier’ whitening product. We analysed these results in relation to the demographic data collected from the participants, inclusive of their age, residence, education, caste and monthly income.
Even this limited data collection from India demonstrated that skin lightener utilisation is associated with cutaneous side effects of paradoxical hyperpigmentation, contact dermatitis and ephilides. The financial burden created by these products is also remarkable.
The high prevalence of use and significant side effects indicates the need for further research, which is currently being developed by our team.

[i] Verma SB. Obsession with light skin – Shedding some light on us of skin lightening products in India (Commentry). Int J Derm. 2010; 49:464–465.

P035

The overuse of systemic and topical corticosteroid for dermatological conditions in India: a case series and review of the literature

R. Minocha[1], C. Wong[2], N. Sharma[3], R. Dunn[2,4], C. Grills[2], N. Grills[5]

[1]Dermatology Department, Westmead hospital, NSW; [2]Skin and Cancer Foundation Inc; [3]Dermatology registrar, Monash Health; [4]Consultant, Royal Melbourne Hospital; [5]The Nossal Institute, University of Melbourne, Melbourne, Vic., Australia

Corticosteroid overuse is prevalent worldwide, especially in developing countries, such as India where high potency preparations are readily available over-the-counter without prescription and at cost. Our literature review on the extent and purpose of steroid use in India demonstrated that non-physician recommended use of topical steroids in a sample population of 433 was 59.3%, the majority of those being potent or super potent.[I]
We present literature outlining the issue and four cases from Northern India where there is an extensive use of corticosteroid preparations for the treatment of dermatological conditions. The patients assessed had been using over the counter steroid

© 2014 John Wiley & Sons A/S. Published by John Wiley & Sons Ltd

preparations for the treatment of melasma, lichen planus and urticaria.
All patients had taken advice to use these products by unqualified 'Doctors' within their own village or from a close friend or relative. The dispensing of high potency topical and systemic medications such as Clobitasol Propionate and Prednisolone respectively by unqualified health care workers in villages and pharmacies was witnessed as being common practice. One patient had taken strong doses of oral prednisolone for 18 months resulting in cutaneous side effects of striae and cushingoid signs.
Further data collection is required to gauge the prevalence of such misuse and there is an undeniable need for public health intervention to educate the communities within India regarding the harmfulness of steroid misuse. In addition, Governments need to create more stringent laws regarding the restriction of dispensing such medication.
[I] Saraswat A, Lahiri K, Chatterjee M, et al. Topical Corticosteroid abuse on the face: A prospective, mutlticentre study of dermatology outpatients. Indian J Dermatol Venereol Leprol. 2011; 77:160–166.

Melanocyte UV Response & DNA Repair

P036

UVR and ros damage protection mediated by DCT expression in human melanocytes

S. A. Ainger[1], H. X. Yong[1], J. H. Leonard[2], R. A. Sturm[1,3]
[1]Institute of Molecular Bioscience, University of Queensland; [2]Queensland Institute of Medical Research, QIMR Berghofer Medical Research Institute; [3]Dermatology Research Centre University of Queensland School of Medicine, Translational Research Institute, Brisbane, Qld, Australia

The melanogenic enzyme Dopachrome Tautomerase (DCT) is involved in the formation of the photoprotective skin pigment eumelanin, and has also been shown to have a role in response to apoptotic stimuli and oxidative stress. To examine the effect of DCT on UVB DNA damage responses and survival pathways in melanocytic cells, knockdown experiments using melanoma cells, MC1R locus genotyped primary melanoblasts (MB) in monoculture and in co-culture with primary keratinocytes were carried out. Transduction with lentiviral vectors was used for DCT ablation and overexpression, and included MC1R WT and RHC homozygous strains known to be deficient in DCT. We found survival was reduced by DCT ablation and by UVB over time, whereas increased DCT protein levels enhanced cellular survival. DCT ablation reduced p53/pp53 proteins in most cases, while RHC MB cells displayed unchanged or decreased pp53. Overexpression of DCT in MB cells resulted in increased or unchanged p53/pp53 levels. Knockdown of DCT in melanoma cell lines and WT MC1R primary MB cells reduces the cell's ability to survive after UVB exposure, and alters DNA damage response protein expression, which reduces the cell's ability to repair UV-induced DNA damage. When compared to WT MC1R MB cells, RHC MC1R variant cells display vastly reduced expression of melanogenic proteins such as DCT, affecting sensitivity to UVB radiation and DNA repair pathways. When comparing co-cultures of WT MC1R-expressing MB cells with monocultures of MB cells only, a protective effect seems to be conferred by the keratinocytes to the MB, shown by increased cell survival when exposed to UVB.

P037

The effect of antioxidants on UV-irradiated melanocyte-derived cells

A. Banjar[1,2], B. S. Feltis[1], P. F. Wright[1], G. M. Boyle[3], T. J. Piva[1]
[1]School of Medical Sciences, RMIT University, Melbourne, Vic., Australia; [2]Department of Medical Technology, Faculty of Applied Sciences, King Abdulaziz University, Jeddah, Saudi Arabia; [3]Queensland Institute of Medical Research, Herston, Qld, Australia

Ultraviolet radiation (UV) plays an important a role in melanomagenesis. This may occur in part by inducing reactive oxygen species (ROS) formation in melanocytes however, antioxidants may stabilise these levels post-UV exposure. It is unknown what role melanin may play in this process. UV can also induce mutations of cell signalling pathway intermediates, which may also contribute to melanomagenesis. Mitogen-activated protein kinases (MAPK) include p-38 and c-Jun N-terminal kinases (JNK) are known to be activated by UV radiation. In this study, we investigated the effect antioxidants (Vitamin C and E) had on cell viability, melanin content and phosphorylation levels of (BRAF, p-38, JNK and ERK) on human melanoma and melanocytes exposed to UV radiation. In this study, lightly pigmented MM418-C1 (C1), darkly pigmented MM418-C5 (C5) melanoma cells and epidermal melanocytes were treated with either vitamin C (1 mM) or the vitamin E analogue trolox (0.1 mM). Cells were exposed to either UVA and/or UVB radiation, and viability was measured after 24 h using the MTS assay. Melanin content was determined spectrophotometrically, while intracellular ROS levels (DCF assay), and mitochondrial superoxide (MSO) levels (MitoSOX assay) were measured using flow cytometry. The effect of antioxidants on the phosphorylated BRAF and MAPK including (p38, JNK and ERK) were investigated over the first two hours post-UV exposure using Western blots. C5 cells were more sensitive to UVAB radiation than C1 cells (50% cell viability: C1 0.4 kJ/m^2 UVB and 8 kJ/m^2 UVA; C5 0.33 kJ/m^2 UVB and 6.15 kJ/m^2 UVA). Only Vitamin C conferred a protective effect to C1 but not C5 cells exposed to either UVB or UVAB radiation. The melanin content of C1 but not C5 cells increased following exposure to UVB or UVAB radiation. Neither Vitamin C nor trolox had an effect on intracellular melanin levels. Intracellular ROS levels were increased in C1 but not C5 cells, following exposure to UVB and UVAB radiation. In irradiated C1 cells trolox reduced ROS formation. Of interest was that in C5 but not C1 cells UVB and UVAB radiation increased MSO levels. Vitamin C reduced MSO levels in the UVB and UVAB irradiated C5 cells. Intracellular p-BRAF and p-ERK levels did not change following exposure to UV radiation unlike that seen for p-p38 and p-JNK post-UVB and -UVAB exposure. Neither vitamin C nor trolox treatment affected the expression of these phosphorylated proteins post-UV exposure. The implications of these finding will be discussed.

P038

Melanoma exhibits defective nucleotide excision repair of UVB-induced DNA photoproducts

T. Budden[1], N. Bowden[1]
[1]University of Newcastle, Newcastle, NSW, Australia

UVB exposure leads to DNA damage in the form of pyrimidine dimers, which when unrepaired can induce C>T transitions. These signature mutations are found throughout the genome of melanoma and more specifically in genes frequently mutated in melanoma, thereby supporting a role for UVB-induced DNA

 © 2014 John Wiley & Sons A/S. Published by John Wiley & Sons Ltd

damage in melanoma development. The nucleotide excision repair (NER) pathway is responsible for repairing UV-induced DNA damage. The global genome repair (GGR) branch of NER is responsible for primarily repairing damage across non-transcribed and silent regions of the genome. Melanoma displays reduced expression of GGR transcripts in response to treatment with the chemotherapy agent cisplatin, and the UV mutation spectrum seen in the melanoma genome shows a higher prevalence of mutations in the non-transcribed regions of the genome, indicative of reduced GGR function. Altogether this suggests a possible role for NER, particularly GGR, in melanoma development as a result of UV exposure. This study aimed to examine the relationship between UVB, NER and melanoma. Quantitative real time PCR was used to measure and compare the relative expression of NER transcripts in melanocytes and melanoma cell lines before and after treatment with UVB. Repair capacity was measured by quantifying the levels of UVB-induced DNA damage after UVB treatment by flow cytometry. Expression and induction of GGR transcripts *XPC*, *DDB1* and *DDB2* was significantly lower in melanoma than melanocytes after UVB treatment, indicating that GGR gene expression fails to respond to UVB in melanoma. Melanoma also showed significantly reduced repair of DNA damage when compared to melanocytes, with the most noticeable difference in the S phase of the cell cycle. Reduced GGR in melanoma provides a possible explanation of the UV mutation spectrum of the melanoma genome and data from this study adds further to the growing evidence of the link between UV, NER and melanoma.

P039

The USF1 and P53 transcription factors coordinate the skin response to UV-induced DNA-damage

M. D. Galibert[1]

[1]CNRS UMR6290 – Université de Rennes 1, IGDR Institute of Genetics and Development of Rennes, Rennes, France

Solar irradiation elicits at the skin level a very complex cellular response that is dependent on the UV-dose and the skin phototype. Dedicated sensors are thus required to detect and drive appropriate and coordinated cell response.

To date, the tumor suppressor p53 is the most important sensor, playing a pivotal role in regulating cell growth and cell death in response to UV-induced DNA-damage. More recently, several lines of evidence support a critical role of the Upstream Stimulatory Factor 1 (USF1), an ubiquitous transcription factor of the basic-helix-loop-helix leucine-zipper family to operate as a stress sensor. USF1 is a direct target of the p38 stress-activated kinase and genetic studies demonstrate that USF1 is a transcriptional rheostat for the UV-induced stress response.

The USF1 and p53 pathways both have pivotal roles in the response to UV-irradiation, where they participate in the immediate molecular and cellular responses, including pigmentation and DNA-repair pathways. They tightly regulate common biological processes to mitigate deleterious effects. Both pathways have been studied in details, but little is known about any crosstalk between them.

Using a combination of in vivo and in vitro assays, we demonstrate that p53 requires USF1 to direct appropriate cell fate decision. We identified the underpinning mechanism, showing that USF1 stabilize the p53 protein in the presence of UV-induced DNA-damage at the skin level.

Together these findings underscore the new role of USF1 and gives new clues of how p53 loss of function can occur in any cell types. Finally, these findings are of clinical relevance because they provide new therapeutic prospects in stabilizing and reactivating the p53 pathway.

P040

4-Hexylresorcinol improves skin hyperpigmentation in a double-blinded, placebo- and randomized-controlled clinical trial

Y. Won[1], C.-J. Loy[1], T. Oddos[2], H.-K. Wong[2], S. Kaur[3], M. Randhawa[3], M. Southall[3]

[1]Johnson & Johnson Asia Pacific, Singapore City, Singapore; [2]Pharmacology Department, Johnson & Johnson Sante Beaute France, Val de Reuil, France; [3]The Johnson & Johnson Skin Research Center, Johnson & Johnson Consumer Companies, Inc., Skillman, NJ, USA

Photodamage is a major cause of skin hyperpigmentation disorders, especially in melano-competent skin types such as Asian skin. Various topical pharmacological agents that modulate the pigmentation process have been used clinically to mitigate the condition. The most widely used remedy to treat hyperpigmentation is perhaps the topical application of hydroquinone. It has been used clinically for the treatment of various hyperpigmentation disorders for more than 50 yrs. Hydroquinone can induce skin irritation, thus there is the need for safe and effective alternatives to treat hyperpigmentation. The Objective of the current study is to evaluate the de-pigmenting efficacy and photoprotection of 4-hexylresorcinol in vitro and its ability to reduce skin hyperpigmentation in a double-blinded, placebo- and randomized-controlled clinical study. Our data showed that 4-hexylresorcinol significantly reduced melanogenesis in primary human melanocytes, murine melanoma cells and pigmented human epidermal equivalents. We demonstrated that 4-hexylresorcinol inhibited melanogenesis via the inhibition of tyrosinase enzyme activity and protein expression, while no effect on tyrosinase gene transcription was detected. Further investigation revealed that the inhibitory effect of 4-hexylresorcinol on melanogenesis is reversible and not associated with melanotoxicity, a concern associated with use of hydroquinone. In addition, 4-hexylresorcinol protected skin cells from UV induced DNA damages by its capacity to counterbalance the NF-kB induced decreased of DNA repair. A double-blinded, placebo- and randomized-controlled clinical study was conducted to investigate the efficacy of a topical formulation containing 4-hexylresorcinol. The study assessed efficacy and safety in Chinese women over 12-weeks. Significant improvement was observed in the group using 4-hexylresorcinol on dermatologists-graded parameters such as overall skin lightening, appearance of spots on the cheeks, overall contrast between spots and surrounding skin, and overall pigmentation size compared to placebo after 12-weeks of treatment. Furthermore, no product-related adverse event was reported. In Conclusion, 4-hexylresorcinol is a safe and effective topical agent which can significantly improve hyperpigmentation and is well-tolerated after 12-week topical application.

© 2014 John Wiley & Sons A/S. Published by John Wiley & Sons Ltd

Genetics of Pigmentation

P041

A genome wide association study in a Caucasian cohort reveals a genetic association between The HLA-C*0701 allele and solar lentigines

V. Laville[1], S. L. Clerc[1], K. Ezzedine[2], R. Jdid[3], L. Taing[1], T. Labib[1], C. Coulonges[1], D. Ulveling[1], P. Rucart[1], W. Carpentier[4], I. Berlin[3], P. Galan[5], S. Hercberg[5], C. Guinot[6], F. Morizot[3], J. Latreille[3], E. Tschachler[7], J.-F. Zagury[1]

[1]Équipe Génomique, Bioinformatique et Applications, Chaire de Bioinformatique, Conservatoire National des Arts et Métiers, Paris; [2]Department of Dermatology, Hôpital Saint-André, Bordeaux; [3]Department of Skin Knowledge & Women Beauty, Chanel R&T, Pantin; [4]Plate-forme Post-Génomique P3S, Hôpital Pitié-Salpêtrière, Paris; [5]Equipe de Recherche en Epidémiologie Nutritionnelle EREN UMR U1153 Inserm/U1125 Inra/Cnam/Univ Paris 13, Centre de Recherche en Epidémiologies et Biostatistiques Sorbonne Paris Cité, UFR SMBH, Bobigny; [6]CE.R.I.E.S., Neuilly sur Seine, France; [7]Department of Dermatology, University of Vienna Medical School, Vienna, Austria

Solar lentigines are dark spots that appear on photoexposed areas of middle-aged and elderly people. Although lentigines are thought to be an inherited trait, no specific study has aimed to identify specific genes associated with lentigines. We have performed a genome wide analysis with the aim to identify potential genes associated with lentigines in a cohort of 520 middle-aged French women. Severity of lentigines was evaluated using a photographic severity scales on the forehead and cheeks. In addition, a global facial score of lentigines severity was generated as a linear combination of the two latter scores. After quality control, 795 063 Single Nucleotide Polymorphisms (SNPs) were analyzed and 502 unrelated Caucasian women were included in the study. We then computed the P-values associated with each SNP using linear regressions adjusted for age, lifetime sun exposition, body mass index, smoking habits, hormonal status and population stratification computed from the EIGENSOFT package. Imputation of the HLA class 1 and class 2 alleles were done using the SNP2HLA software.

A group of nine Single Nucleotide Polymorphisms (SNPs), located on chromosome 6, exhibited a False Discovery Rate below 25% when testing the association with the global facial lentigine score. These SNPs gathered into two distinct blocks. One of these two blocks was within the *HLA* region. The minor alleles of these SNPs were associated with a lower global facial lentigine score and with a decrease of *HLA-C* expression. Interestingly, these SNPs were also in high linkage desequilibrium with the *HLA-C*0701* allele ($r^2 = 0.95$). The other block, corresponded to intergenic SNPs and was 11 megabases apart from the *HLA* region. These latter SNPs exhibited an association with the evaluation of lentigines on the forehead* that passed the genome-wide significance threshold ($P = 1.37 \times 10^{-8}$). Importantly, these two signals located on chromosome six were independent from each other since their P values remained unchanged when putting the other SNPs as covariates.

Our study points towards an association of lentigines to HLA and immunity. Interestingly, the presence of the *HLA-C*0701* allele has been associated with a better lymphocytes CD8+ response to melanoma. Consequently, this allele could play a protective role in the development of lentigines. However, the decrease of the *HLA-C* gene expression suggests a more complex mechanism. Yet, these results have to be confirmed by replication studies and more specific molecular analysis or search for genetic interactions should be conducted to better understand the relationship between immunity and development of lentigines.

P042

Gene array analyses of age spot uncovered new bio-functions

Y. Zhuang[1], V. Hariharan[1], J. Lyga[1], U. Santhanam[2]

[1]Bioscience, AVON Products Inc.; [2]CBIVT, AVON Products Inc., Suffern, NY, USA

Senile lentigo (or age spot) are hyperpigmented macules with irregular shape on the skin. They mostly occur on UV-exposed areas such as forearm, back of the hand and face and are also associated with age since they normally occur after age of 40. The mechanism of how the age spots are formed is still not clear. Histological studies have shown that, compared to peri-lesion area, they show hyperpigmented basal layer, increased amount of melanocytes and melanosomal protein, elongated rete ridges with increased melanin at the tips, altered keratinocyte differentiation and increased inflammatory response. In this study, we utilized gene array approach to gain a more systematic view of changes in the area of age spots. Twelve Caucasian women aged from 55 to 75 were recruited for the study. Biopsies were taken from lesional and peri-lesional area of the skin, as well as sun-protected area. We identified differentially expressed genes by comparing gene expression profiles between lesional and peri-lesional area, as well as gene expression profiles between lesional and sun-protected area. Using 1.3 fold as a cutoff, 524 genes showed differential expression between lesional and sun-protected area whereas 43 genes showed differential expression between lesional and peri-lesional area. Besides melanogenesis- and inflammation-related genes that have been reported previously, we also uncovered many novel biological functions and pathways such as cellular assembly and organization, cellular development, cell morphology and tissue development.

P043

Do melanocytes contribute to the structure of their niches?

H. Shibuya[1], H. Yamamoto[1]

[1]Nagahama Institute of Bio-Science and Technology, Nagahama, Japan

Melanocytes differentiate from the vertebrate embryo-specific neural crest, migrate to and settle in various organs, including not only the skin and hair follicles but also extracutaneous locations such as the inner ear, choroid, brain, heart, adipose tissue, lung, etc. What are these extracutaneous melanocytes doing in those sun-protected habitats, still producing melanin?

Among those extracutaneous melanocytes, cochlear melanocytes are mainly localized in the stria vascularis. The stria vascularis consists of three types of cell layers (from the endolymph side: marginal, intermediate and basal) and rich capillaries. Among those cells, the intermediate cells are believed to be melanocytes. Cochlear melanocytes are known to be essential for hearing ability. Strial intermediate cells are required for the generation of endolymph-mediated action potentials that are necessary for normal hearing acuity. In addition, melanocytes in the stria vascularis ‘specifically’ express *Gsta4* and may play a critical role

© 2014 John Wiley & Sons A/S. Published by John Wiley & Sons Ltd

in reducing oxidative stress in the cochlea. But what are the function(s) of other types of extracutaneous melanocytes?
In order to study whether those other melanocytes scattered all over the body functionally contribute to their niches, we used a mouse *MITF* mutant allele, $MITF^{mi\text{-}bw}$, to focus on the structures of their habitats without melanocytes. $MITF^{mi\text{-}bw}$ homozygous mice have black eyes and a white coat color. This recessive allele comprises an insertion of a 7.2 kb novel L1 element into the intron sequence located between exons 3 and 4, that abolishes expression of the MITF-M isoform which is indispensable for melanocyte development, thus those mice lack mature melanocytes all over the body.
We will discuss such phenotypes in the inner ear in detail.

Human Skin Colour and Skin Optics

P044

UVA1 exposure leads to darken human skins of different constitutive pigmentation together with a molecular biological impact

C. Marionnet[1], S. Nouveau[1], V. Hourblin[1], K. Pillai[2], M. Manco[2], S. D. Bino[1], P. Bastien[1], C. Tran[1], C. Tricaud[3], O. de Lacharrière[1], F. Bernerd[1]
[1]L'Oréal Research & Innovation, Aulnay sous bois, France; [2]L'Oréal Research & Innovation, Clark, NJ, USA; [3]L'Oréal Research & Innovation, Chevilly Larue, France

Human skin is daily exposed to solar UV rays. Among the range of UV that reach the Earth ground, longwave UVA (UVA1, 340–400 nm) can represent up to 80% of total UV and show high penetration properties, reaching deep dermis. An increasing body of evidences indicates that longwave UVA1 can have a significant contribution in long-term clinical consequences of solar UV's. This work aimed at studying in vivo the clinical pigment darkening response as well as the biological response following UVA1 exposure of volunteers exhibiting various constitutive pigmentations. Accordingly, in vivo controlled studies were conducted in populations with light or more highly pigmented skin color including 1) European descent volunteers with phototype II to IV and individual typologic angle, ITA, from 10° to 45° or 2) Indian descent volunteers with phototype IV toV and ITA from 15° to 30°. At different time points after a single UVA1 exposure, darkening response was followed using visual scoring and colorimetric measurements (L*, a*, b * parameters assessed by chromametry). In addition, 6 h post UVA1 exposure, punch biopsies were performed to study gene expression levels using quantitative PCR. The expression of genes related to different functional families such as oxidative stress response and inflammation was analyzed. Visual scoring revealed an increased pigmentation in both populations following UVA1 exposure together with a decrease in corresponding chromametric measurements such as luminance (L*) or the yellow component (b*). Results also showed that 6 h post UVA1 exposure, the expression of genes related to oxidative stress response or to inflammation was modulated. Altogether, these data revealed that, in different skin color phenotypes, the clinical darkening of the skin observed after UVA1 exposure is associated with a biological impact at the molecular level.

P045

Ashwagandha-derived phytochemicals possess skin whitening potential: evidence from human cell based assays

S. Kaul[1], R. Gao[1], N. Nigam[1,2], L. Li[1,2], R. Wadhwa[1]
[1]Cell Proliferation Research Group and DBT-AIST International Laboratory for Advanced Biomedicine, National Institute of Advanced Industrial Science & Technology AIST; [2]Graduate School of Life and Environmental Sciences, University of Tsukuba, Tsukuba, Japan

Skin coloration is regulated by the process of melanogenesis in specialized cells called melanocytes that constitute about 10% of cells in the basal layer of epidermis. Skin color is associated with many other characteristics of skin and its functional aspects such as tolerance to stress and environmental conditions. Compared to white skin, dark skin has been established to possess high tolerance to supra-optimal temperature, UV and oxidative stress. Furthermore, several strategies including traditional home medicine and modern drugs have been in use to manipulate the stress tolerance of skin and to increase its outlook for safety and cosmetics, respectively. Ashwagandha extracts have been in use for a variety of activities ranging from immunostimulation, anti-stress and anti-aging. We have earlier demonstrated that the alcoholic and water extracts of Ashwagandha leaves possess selective cancer cell killing activity that operates through activation of tumor suppressor p53 and oxidative stress pathways. On the contrary, low doses of the extract and its purified components protect normal human cells against a variety of oxidative stress. In the present study, we examined the effect of Ashwagandha (a popular Ayurvedic herb) leaf extracts on skin-derived cells by using OAG (diacylglycerol (DAG) 1 -oleoyl-2-acetyl-sn-glycerol)-induced melanogenesis as a model system. We report that Ashwagandha leaf extracts have capability to reduce OAG-induced upregulation of melanin in human primary melanocytes and melanoma cells. These reagents could be used for cosmetics and therapeutic manipulation of skin color and other characteristics regulating stress tolerance and pathologies.

P046

Methods and rates of dermoscopy usage: a cross-sectional survey of United States dermatologists stratified by years in practice

E. C. Murzaku[1], S. Hayan[2], B. K. Rao[3]
[1]Rutgers Robert Wood Johnson Medical School, Piscataway, NJ; [2]State University of New York at Stony Brook, Stony Brook, NY; [3]Dermatology, Rutgers Robert Wood Johnson Medical School, Somerset, USA

Dermoscopy is a non-invasive imaging technique that allows for the in vivo visualization of skin surface and subsurface structures, increases the sensitivity for melanoma detection, and decreases the rate of biopsy of benign skin lesions. Despite the demonstrated efficacy of dermoscopy and its widespread use in Europe and Australia, dermoscopy remains underutilized in the United States. Our aims in this cross-sectional study were to evaluate rates of dermoscopy use and to analyze dermoscopy training, utilization practices, and diagnostic methods employed by US dermatologists as a function of the number of years in dermatology practice.
Our survey consisted of questions focusing on years in dermatology practice, use of and training in dermoscopy, selection of skin lesions for dermoscopic exam, utilization of

© 2014 John Wiley & Sons A/S. Published by John Wiley & Sons Ltd

sequential dermoscopic imaging of individual lesions, and self-estimated malignant to benign ratio of biopsied melanocytic lesions. We invited 500 practicing US dermatologists randomly selected from an American Academy of Dermatology member list to participate in our study. We also distributed paper surveys to 47 dermatologists attending national and regional dermatology conferences between May and December 2013.
A total of 277 dermatologists participated in our study for an overall response rate of 50.6%. Of the 277 dermatologists who completed the survey, 264 reported their use of dermoscopy. We found that, overall, 80.7% of survey respondents reported dermoscopy use and 83.4% were trained in dermoscopy. When stratified by years in dermatology practice (≤5 yrs, 6–10 yrs, 11–15 yrs, 16–20 yrs, and >20 yrs), a statistically significant difference was observed between groups both in use of ($P = 0.030$) and training in ($P = 0.013$) dermoscopy. Notably, among dermatologists in practice for ≤5 yrs, 97.8% used dermoscopy and 100% were trained in dermoscopy. When methods of dermoscopy utilization were examined among respondents, only 31.3% reported performing dermoscopy on all pigmented lesions and 49.3% used sequential dermoscopic imaging in order to follow changes in individual lesions, with no significant differences noted among groups as a function of years in dermatology practice. No differences in the self-estimated malignant to benign ratio of biopsied melanocytic lesions were observed between groups.
We found that 80.7% of surveyed dermatologists utilized dermoscopy, a rate notably higher than that reported in previous studies. This apparent increase in dermoscopy use may be due to improved and expanded dermoscopy instruction in dermatology residency training programs, as supported by our finding that 97.8% of dermatologists with ≤5 yrs in dermatology practice used dermoscopy and 100% had training in dermoscopy. Only 31.3% of respondents performed dermoscopy on all pigmented lesions, a diagnostic technique that has been shown to detect early, clinically inconspicuous melanomas in frequently overlooked locations such as the limbs. Sequential dermoscopic imaging to follow changes in individual lesions was also used by a minority of respondents (49.3%), despite its well-established capability to detect thinner melanomas with a low rate of excision of benign lesions. These indicate areas of potential future growth among dermoscopy users.

P047

Confocal microscopy for managing pigment cell disorder and neoplasms

B. Rao[1], E. Murzaku[2]
[1]Rao Dermatology, New York, NY; [2]RWJMS, New Brunswick, NJ, USA

Biopsy and histological evaluation of tissue is currently the gold standard for diagnosing cutaneous diseases. Discomfort and scarring which result from biopsies can be justified as a means to differentiate neoplastic and benign conditions, however newer, non-invasive diagnostic tools are preferable in many instances. Confocal microscopy is a novel technology that uses low-power laser to emit near-infrared light and illuminate tissue, allowing visualization of the epidermis and dermis on a cellular level comparable to routine histology. Although it is limited in depth of penetration, confocal microscopy has proved to be useful for diagnosing pigment cell disorders and neoplasms, including melanoma. Confocal microscopy can also play a role in managing melasma and other pigmentary disorders. It can quickly and non-invasively determine the level of pigment in melasma (i.e. epidermal, dermal or mixed) and also reveal other significant findings, such as telangiectasias. In addition to its use as a diagnostic tool, confocal can also be used to monitor response to various treatments without the adverse effects associated with standard biopsy technique. In this presentation, we will discuss data regarding diagnostic accuracy of confocal microscopy for melanoma, nevi and other skin tumors. We will also present how confocal microscopy is helpful in management and diagnosing inflammatory skin conditions. This will include a thorough literature review as well as a presentation of our clinical experiences and cases.

P048

Color assessment of progressive nonsegmental Vitiligo under short-term intravenous methylprednisolone pulse therapy

T. Yamaoka[1], Y. Nagata[1], A. Tanemura[1], E. Ono[1], A. Tanaka[1], K. Kato[1], M. Yamada[1], I. Katayama[1]
[1]Dermatology, Osaka University Graduate School of Medicine, Suita, Japan

The recommended treatment for nonsegmental vitiligo vulgaris includes topical ointments of steroids and tacrolimus, ultraviolet phototherapy, systemic steroids and surgery according to international guidelines. Ultraviolet light therapy such as narrow band UVB has been considered to be the most common modalities in treating cases of disseminated vitiligo. However, narrow band UVB irradiation requires consultations once or twice a week and is mostly refractory in advanced and/or long-lasting cases. Steroid therapy for advanced vitiligo recommended as a class C1 treatment in one set of guidelines is known as fairly effective and has two routes of administration, namely, oral and intravenous. Although oral steroid therapy suggested by Radakovic, Kim, and Pasricha, unacceptable side effects (moon face, exacerbation of diabetes mellitus and hypertension, and so on) were observed in 50% or more of patients due to the long-term administration period. Meanwhile, with respect to intravenous steroid pulse therapy, Seiter obtained disease improvements in pathology in 90% of cases with temporary and reversible side effects. Although the administration of systemic steroid for nonsegmental vitiligo in the progressive stage is a recommended treatment according to guidelines, the clinical efficacy of this regimen has not been fully established.
To evaluate the clinical efficacy of minidose systemic steroid treatment and stratify the evidence regarding its usefulness in progressive vitiligo patients.
Minidose steroid pulse therapy (500 mg/day of methylprednisolone for three sequential days) was administered intravenously three times monthly in five vitiligo patients. The visual changes in vitiligo lesions were evaluated on photographs and quantified using a spectrophotometer.
All patients completed three cycles of treatment without severe adverse events. Three of the five patients achieved disease arrest with decrease in white contrast.
Short-term minidose steroid therapy is well tolerated and effective for achieving disease arrest in progressive nonsegmental vitiligo. The whiteness assessed by a spectrophotometer is possibly associated with therapeutic response to steroid therapy.

© 2014 John Wiley & Sons A/S. Published by John Wiley & Sons Ltd

Melanocyte & Stem Cell Biology

P049

Effects of 1, 25-dihydroxyvitamin D3 on human epidermal melanocytes and melanoblasts

T. Kawakami[1], A. Ohgushi[1], T. Baba[1], T. Hirobe[2], Y. Soma[1]
[1]Department of Dermatology, St. Marianna University School of Medicine, Kawasaki; [2]Fukushima Project Headquarters, National Institute of Radiological Sciences, Chiba, Japan

Vitamin D_3 analogs have been used as topical therapeutic agents in vitiligo. We previously reported that 1,25-dihydroxyvitamin D3 ($1,25(OH)_2D_3$), which is the active metabolite of vitamin D3, affects the proliferation and differentiation of mouse melanoblasts via the endothelin B receptor.
We examined the effects of $1,25(OH)_2D_3$ on human epidermal melanocytes and human epidermal undifferentiated melanoblasts upon addition of various concentrations of $1,25(OH)_2D_3$ to the culture.
Normal human adult epidermal melanocytes (HEMa-LP cells) were purchased and human epidermal undifferentiated melanoblasts were cultured as described previously. We used the Alamar blue assay for cell proliferation analysis, and examined tyrosinase activity, DOPA reaction, and performed electron microscopy, and western blot analysis.
Tyrosinase activity of melanocytes increased in a dose-dependent manner upon addition of $1,25(OH)_2D_3$. In contrast, $1,25(OH)_2D_3$ addition led to a slight increase in tyrosinase activity in melanoblasts, with significance reached at concentrations higher than $10^{-7\ M}$. DOPA reaction increased upon addition of $1,25(OH)_2D_3$ at concentrations higher than $10^{-5\ M}$. Electron microscopic analysis following the addition of 10−5 M $1,25(OH)_2D_3$ demonstrated the presence of melanosomes at more advanced stages than in untreated cells. Western blot analysis was performed on the endothelin B receptor of human melanoblasts following treatment with $1,25(OH)_2D_3$. Endothelin B receptor protein expression of human melanocyte decreased in a concentration-dependent manner following the addition of $1,25(OH)_2D_3$.
We propose that $1,25(OH)_2D_3$ addition could be a trigger for differentiation and increase tyrosinase activity in human melanoblasts. These results provide important clues to the mechanisms involved in topical vitamin D3 treatment for vitiligo. We also believe that the endothelin B receptor could be the key molecule involved in understanding the roles and working mechanisms of topical vitamin D3 analogs in effective treatment of melanocyte-associated disorders.

Hair Biology & Pigmentation

P050

Risk factors for premature hair graying: family history, smoking, and obesity

H. Shin[1], H. H. Ryu[2], M. Choi[2], O. Kwon[2], S. J. Jo[2]
[1]Department of Dermatology, Dongguk University Ilsan Hospital, Goyang; [2]Department of Dermatology, Seoul National University College of Medicine, Seoul, Republic of Korea

Hair graying is a natural part of the aging processes, but premature hair graying (PHG) causes low self-esteem in young people. However, the risk factors for PHG itself have not been the focus of research and studies of such risk factors are rare.
This study aimed to identify risk factors for PHG in healthy young Korean males.
We conducted a cross-sectional study using questionnaires in males younger than 30 yrs. After a pilot study that included 1069 males, we surveyed 6390 males about their gray hair status and various socio-clinical characteristics.
In the pilot study, body mass index, family history of PHG, and emotional stress were associated with PHG. The age of participants in the main survey was 20.2 $\pm$ 1.3 yrs (mean $\pm$ standard deviation). Of the 6390 participants, 1618 (25.3%) presented with PHG. Family history of PHG (odds ratio [OR], 12.82), obesity (OR, 2.61), and >5 pack-years history of smoking (OR, 1.61) were significantly associated with the development of PHG. A paternal history of PHG (OR, 14.84; CI, 10.10–21.81) ($P < 0.001$) had a greater association with PHG than a maternal history (OR, 2.92; CI, 1.57–5.43) ($P = 0.001$). Medical history of admission or operation, chronic disease, androgenic alopecia, current medication, drinking, exercise, diet, educational Background, and scholarly achievement did not show significant correlations with PHG ($P > 0.05$). In the multivariate analysis, family history of PHG (OR, 2.63) and obesity (OR, 2.22) correlated with the severity of PHG. Owing to the use of questionnaires, the possibility of recall bias exists. Females were not evaluated in this study.
Family history of PHG, obesity, and smoking were significantly associated with the incidence of PHG. In particular, family history and obesity showed reproducible significance in both the pilot and main studies and were correlated with the severity of PHG. Smoking and obesity are independently associated with systemic oxidative stress. Our findings imply that hair graying is affected by oxidative stress, which may provide an approach for health education against obesity and smoking. We also hope that this study helps further research on the etiology, prevention, and treatment of PHG.

P051

Psychosocial impact of premature canities in a tertiary care centre

S. Tripathy[1]
[1]IMS &SUM Hospital, Bhubaneswar, India

Premature canities or premature graying of hair in Indian patients is defined as the manifestation of gray hair before 25 yrs of age. Hair length, colour and style play an important role in peoples physical appearance, self perception and contribute immensely in human communication. Hence premature canites has significant adverse effects on the appearance, self esteem and socio-cultural acceptance of the affected individual. Being a bothersome and disfiguring condition it causes significant interference with social adjustment and acceptance. Lack of established, effective long lasting treatment modalities adds on to the patients psychosocial trauma.
AimsOur study aims to determine the aspects of the disease, the beliefs about causation, medical and psychosocial needs of the patient, expectation from the treating physician, affects of the disease and treatment modality on the patient's quality of life.
The study was carried out in the dermatology department of a tertiary care hospital of a teaching institute in eastern India over a period of 2 yrs from February 2012–2014.

© 2014 John Wiley & Sons A/S. Published by John Wiley & Sons Ltd

190 subject were enrolled in the study by purposive sampling and interviewed in privacy in the department of Psychiatry of the same Institute. Purposive sampling was used as the mode of selection of the subjects due to the low prevalence rate of premature canities in a general OPD. The subjects enrolled comprised of a wide range of patients less than 25 yrs of age.The study aims at evaluating the effects of premature canities on the quality of life.(QOL) Psychiatric morbidity manifests as anxiety, depression and sleep disturbances which leads to QOL impairment, hence mode of standardization of the enrolled subjects involved the use of HAMILTON ANXIETY (HAM-A) SCALE, HAMILTON DEPRESSION (HAM-D) SCALE and preconstructed questionnaires.
Written inform consent to participate in the study was obtained from the patients and parents of children with pre mature canities. Any individual hesitant to talk about the disease or refraining to impart information about their concerns were excluded from the study. The interviewer recorded the information from the patients which was later evaluated by a psychiatrist and grouped under the headings of anxiety, depression and sleep disturbances.
A total of 190 patients were interviewed and based on the information obtained from the interviews, they were divided in to 3 groups i.e anxiety, depression and sleep disturbances. The obtained data was then analyzed using the chi-square Test of association, which allows the comparison of two attributes in a sample of data to determine if there is any relationship between them. It was noticed that a wide variety of concerns were expressed with respect to the disease, like apprehensive attitude to face a social gathering, loss of interest in work, preoccupation with the concern about the disease, feeling of weakness, irritability, restlessness, unsteady voice, difficulty in falling sleep, feelings of guilt, fear of being left alone and fatigue on waking up from sleep. The obtained data was then analyzed in detail by a qualified psychiatrist and an association between premature canities and QOL obtained.
The study has established a correlation between premature canities and QOL. The association obtained shows impairment of QOL which will create a bench mark among dermatologist to approach a patients of premature canities and counsel them to modify their QOL.

Model Systems for Pigment cell Biology & Disease

P052

Cistus monspeliensis bio extract reduce solar lentigo induction by factors secreted by irradiated aged fibroblasts, in pigmented reconstructed epidermis

M. Salducci[1], N. André[1], C. Guéré[1], M. Martin[1], R. Fitoussi[1], K. Vié[1], M. Cario-André[2]
[1]Laboratoires Clarins, Pontoise; [2]INSERM 1035, Université Bordeaux, Bordeaux, France

We developed a new in vitro model which reproduces the main solar lentigo characteristics *i.e.* increases the dermo-epidermal junction's length and the melanocytes number, but also impairment in terminal differentiation, using factors secreted by UVA-stress aged fibroblasts in pigmented reconstructed epidermis. To confirm our model functionality, we tested an active ingredient, the *Cistus monspeliensis* bio extract, in order to reduce factors secretion by UVA-stress aged fibroblasts and at the same time, the appearance of solar lentigo.
We prepared conditioned media produced by fibroblasts intrinsically (from old donors) and artificially aged (by successive passages in vitro), submitted or not to UVA-irradiation, and treated or not with different doses of *Cistus monspeliensis* bio extract (0.01% or 0.05%). Reconstructed pigmented epidermis were incubated with these conditioned media during differentiation.. Sections of paraffin embedded reconstructed epidermis were stained with collagen IV to measure the length of dermo-epidermal junction and melanA to quantify the number of melanocytes, by immunohistochemistry staining.
Our results show that treatment of reconstructed epidermis with conditioned media produced by UVA-stress aged fibroblasts treated with *Cistus monspeliensis* bio extract, reduce the appearance of the two solar lentigo main characteristics. Indeed the use of conditioned media produced by aged fibroblasts induced an increase of the dermo-epidermal junction's length and of the melanocytes number. The addition of *Cistus monspeliensis* bio extract, during conditioned media preparation, reduces the increase of these both characteristics respectively by −15% ($P < 0.05$) and −40% ($P < 0.001$), in a dose-dependent manner.
The aim of this study was to evaluate the ability of an active ingredient, the *Cistus monspeliensis* bio extract, to reduce the appearance of solar lentigo, using our new in vitro model. Our results, by decreasing both dermo-epidermal junction's length and melanocytes number, show that the *Cistus monspeliensis* bio extract is a good candidate to reduce the solar lentigo appearance, probably by reducing factors secretion by UVA-stress aged fibroblasts, and that his efficiency is dose-dependent.

P053

Genetic fine mapping of a rat dominant ventral spotting gene, downunder (*Du*)

T. Kuramoto[1], M. Yokoe[1]
[1]Kyoto University, Kyoto, Japan

The downunder (*Du*) mutation has been discovered in an Australian fancy stock and it displays ventral spotting (Pigment Cell Res 17:451. 2004). Inheritance of the ventral spotting is dominant and the *Du*/*Du* embryos are lethal. The *Du* locus locates on the rat chromosome 3 (Pigment Cell Melanoma Res 24:820, 2011). The *Du* is thought to be a novel spotting gene in rats as well as mice and identification of *Du* will provide us valuable information about melanocyte differentiation and development. Here, we carry out genetic fine mapping of the *Du* locus.
We maintain the *Du* mutation using F344.Cg-*Du* congenic rats. To narrow down the *Du*, we analyzed haplotypes of congenic rats that were produced by successive backcross with F344. Eight single nucleotide polymorphism (SNP) markers were used to make haplotypes of each congenic rat.
Du is mapped to ~1.2-Mb region between *D3Got18* (24.2 Mb) and *D3-Du-SNP10* (25.64 Mb). Within the *Du*, three gene, *Arhgap15*, *Gtdc1*, and *Zeb2* were mapped. The *Zeb2* encodes transcription factor and is the causative gene for Mawat-Wilson syndrome, in which patients show Hirschsprung's disease with mental retardation. Because some mouse models of Hirschspring's disease show white spotting, the *Zeb2* was thought to be good candidate for the *Du* mutation. We

 © 2014 John Wiley & Sons A/S. Published by John Wiley & Sons Ltd

sequenced all exons of *Zeb2* of F344.Cg-*Du*, but could not find any non-synonymous mutation in them. Thus, The *Du* mutation would locate in *Arhgap15*, *Gtdc1*, or *Zeb2* regulatory region.

Translational Skin Biology

P054

Melanocytes and melanotropin in skin wound healing and keloid pathogenesis

E. C. Eksteen[1], P. F. Coetzee[1], M. J. Bester[2]

[1]Department of Surgery, University of Pretoria; [2]Department of Anatomy, University of Pretoria, Pretoria, South Africa

Skin wound healing in humans are typically described as the overlapping stages of hemostasis,inflammation,proliferation and then remodelling.The melanocyte is,however,absent from wound healing descriptions we found in the literature.
It is a fact that keratinocytes require melanin from melanocytes to protect them from the sun's harmful ultra-violet rays.In the process of transferring melanosomal contents from melanocytes to keratinocytes,not only melanin are supplied.The precursor to melanotropin(alphaMSH),known as pro-opiomelanocortin (POMC), is also present within melanosomes,as well as the enzymes prohormone convertases 1 and 2.
We propose that an anti-inflammatory stage is always present in physiologic wound healing.It involves primarily melanocytes and keratinocytes producing alphaMSH.The alphaMSH act via the melanocortin-1-receptor on immune cells to prevent Nuclear Factor kappa light-chain enhancer of B-cells(NFkB)-mediated transcription of proinflammatory cytokines.This stage follows immediately after epithelialization.
The continued production of melanotropin by melanocytes and keratinocytes after a skin wound had epithelialized is also anti-fibrotic and helps to regulate the proliferative phase of healing.
This study received approval from the Main Ethics Committee of the University of Pretoria,Faculty of Health Sciences,in June 2009.
Determine if a close association exists between fibroblasts and melanocytes in normal and keloid skin in vivo an in vitro.
Materials & MethodsWith informed consent,skin specimens were taken from patients with keloids and normal skin during routine surgery from Sept 2009 to August 2012 at the Steve Biko Academic Hospital in Pretoria,South Africa.Samples were then used mainly to perform light microscopy,polarized light microscopy and transmission electron microscopy. Immunohistochemistry and cell cultures were also performed.
Thirty three patient samples were included.Normal skin epidermal-dermal junction anatomy was compared to that of keloids.Melanocytes were photographed in very close association with fibroblasts in both keloid and normal skin.Melanocyte dendrites morphologically appeared restricted in keloid samples.Melanosomes in all stages of development were identified in keloid and its transferral to keratinocytes visualized.We photographed both keloid fibroblasts and keloid mast cells phagocytosing collagen.
Melanocytes associated with the basal layer of the epidermis and made contact with multiple keratinocytes.They were often found very close to/in contact with fibroblasts.These findings,combined with the fact that melanotropin(alphaMSH) is produced by both melanocytes and keratinocytes especially under conditions of stress,provide enough evidence that the melanocyte is involved in wound healing.
In keloid, we hypothesize that melanocortin-1-receptor (MC1R) polymorphism result in an abnormally prolonged inflammatory stage after epithelialization of the wound,at the time when melanocytes start producing melanin (which indicates alphaMSH activity).The alphaMSH then cannot provide the necessary anti-inflammatory signals to mast cells,macrophages and endothelial cells within the granulation tissue, immunomodulatory changes to T-lymphocytes, and the antifibrotic signals to fibroblasts because of dysfunctional MC1Rs.

Melanoma Biology

P055

RAB27a as a novel therapeutic target for melanoma

K. Beaumont[1], W. Weninger[1], N. Haass[1,2]

[1]Centenary Institute, Sydney, NSW; [2]University of Queensland Diamantina Institute, TRI, Brisbane, Qld, Australia

Metastatic melanoma is notoriously difficult to treat. Although treatment options have improved with the introduction of new targeted therapies and immunotherapies, more therapeutic targets/strategies are needed. RAB27a (member of the Rab GTPase protein trafficking family) has increased expression in melanoma cells compared to benign nevi and has been identified as a potential driver gene in a study of chromosomal copy number and gene expression in melanoma. In melanocytes RAB27a regulates pigment granule trafficking. The function of RAB27a in melanoma cell biology is not well known. Rabs are overexpressed in several cancers and aberrant Rab expression is known to alter cancer cell biology via altered secretion, recycling or localization of growth factor receptors, adhesion molecules or other factors important for melanoma growth, survival and movement.
Hypothesis: Melanoma cells that express high levels of RAB27a are dependent on this protein for survival, proliferation, migration or invasion.
shRNA knockdown of RAB27a in Rab27a-high cell lines was performed to determine the function of RAB27a in melanoma cell biology. We also treated melanoma cells with a non-specific inhibitor of small GTPase function – simvastatin. We performed 2D in vitro proliferation, cell death, morphology and migration assays, as well as 3D spheroid assays which more accurately mimic the tumour microenvironment. Intracellular localisation of RAB27a in melanoma cells was also investigated using Rab27a-GFP.
RAB27a co-localised with pigment granules in melanoma cells, but was also found at other vesicular structures at the cell periphery, indicating RAB27a has a function independent of granule trafficking in melanoma. Loss of RAB27a in melanoma cell lines inhibited melanoma proliferation and invasion. Preliminary data indicate that treatment of melanoma cells with a non-specific inhibitor of Rab function (simvastatin) also inhibits invasion, and that invasion is more substantially inhibited in Rab27a-high cell lines.
Our data indicate that RAB27a plays a central role in proliferation and invasion in Rab27a-high melanoma cells, although the molecular mechanisms are still unclear. RAB27a expression is confined to melanocytic cells as well as some other specialised cell types making RAB27a a novel potential therapeutic target. Although no specific Rab inhibitors are available, statins, which are commonly used to treat hypercholesterolaemia, are known to

© 2014 John Wiley & Sons A/S. Published by John Wiley & Sons Ltd

inhibit the function of small GTPases such as Rabs. At clinically relevant low concentrations, statins are able to reduce melanoma invasion. Our data indicate that loss of Rab function may be at least partially responsible for this effect. Statins may thus be a safe therapy in early stage patients for preventing melanoma metastasis.

P056

Response of melanoma cell lines to BRAF V600 inhibitor treatment under hypoxic conditions is cell line specific

H. Breiteneder[1], D. Pucciarelli[1], S. Kontsekova[1], M. Takáčová[2], F. Juliano[2], N. Lengger[1], L. Csaderova[2], S. Pastorekova[2], C. Hafner[1,3]

[1]Depatment of Pathophysiology and Allergy Research, Medical University of Vienna, Vienna, Austria; [2]Institute of Virology, Slovak Academy of Sciences, Bratislava, Slovakia; [3]Karl Landsteiner Institute of Dermatological Research, St.Poelten, Austria

Hypoxia, an important phenomenon of the tumor microenvironment, is often associated with pathological processes necessary for tumor progression which leads to drug resistance and poor prognosis. As other cancers, melanoma tumors include regions of hypoxia and anoxia caused by an imbalance in both oxygen supply and consumption. Tumor hypoxia is known to reduce the sensitivity of solid tumors to radiation therapy and can negatively influence treatment outcome and patient survival in a number of cancer types. Advanced melanoma is often characterized by a BRAF V600 mutation. The development of specific BRAF V600 inhibitors has shown encouraging results in the treatment of melanoma. However these benefits are mainly temporary.

In this study, which was supported by a Marie Curie ITN-Network for Initial Training (EngCaBra, FP7-PEOPLE-2010-ITN, GA 264417), we aimed to analyze the influence of hypoxia on the effects of the BRAF V600 inhibitor PLX4032 on cell proliferation, cell motility and invasive capacity in three different melanoma cell lines.

Melanoma cell lines M14, 518A2 and A375 were grown in normoxia (21% pO2) and hypoxia (2% pO2) with and without 1 mM PLX4032. Antiproliferative effects were evaluated in a real-time setting in the impedance-based x-CELLigence® system. To evaluate the effects of hypoxia and PLX4032 on cell motility 2D migration and Matrigel® invasion assays were performed.

Under hypoxic conditions M14 and A375 cells showed a reduced proliferation rate (−20% for M14 cells and −60% for A375 cells) compared to normoxic conditions. In contrast, 518A2 cells were not susceptible to hypoxic conditions and the proliferation rate did not change. After addition of PLX4032, hypoxic M14 and 518A2 cells reduced cell growth by additional 20% and 38% compared to normoxic, PLX4032-treated M14 and 518A2 cells, respectively. Surprisingly, hypoxic PLX4032-treated A375 cells showed an enhanced cell proliferation rate (+34%) when compared to normoxic, PLX4032-treated A375 melanoma cells. Motility of hypoxic, PLX4032-treated A375 cells was enhanced (+45%) when compared to normoxic, PLX4032-treated A375 cells, whereas the invasion capacity of hypoxic, PLX4032-treated 518A2 cells was enhanced (+22%) when compared to normoxic, PLX4032-treated 518A2 cells.

Our data confirm that the effect of PLX4032 on melanoma cell lines is significantly modified by hypoxia in a cell-type specific manner. Hypoxic conditions in the tumor microenvironment might significantly alter the therapeutic benefit of this drug in melanoma patients.

P057

Identification of novel prognostic and/or therapeutic targets in acral lentiginous melanoma by microarray analysis

H.-Y. Cheng[1], C.-E. Wu[1], J.-J. Hsieh[1,2], Y.-C. Shen[1,2], C.-H. Hsieh[1], M.-M. Hou[1], W.-K. Huang[1], T.-T. Kuo[3], Y.-F. Lo[4], T. Hsu[2], J. W.-C. Chang[1]

[1]Division of Hematology-Oncology, Chang Gung Memorial Hospital, Taoyuan; [2]Institute of Bioscience and Biotechnology and Center of Excellence for Marine Bioenvironment and Biotechnology, National Taiwan Ocean University, Keelung; [3]Department of Pathology, Chang Gung Memorial Hospital; [4]Department of General Surgery, Chang Gung Memorial Hospital, Taoyuan, Taiwan ROC

Acral lentiginous melanoma (ALM) is the most common type of malignant melanoma in Taiwan; however studies on the tumor biology of ALM are limited. Therefore, the aim of this study was to investigate novel prognostic and/or therapeutic targeted genes via microarray analysis to explore the mechanism of tumor metastasis.

We used an array comparative genomic hybridization (arrayCGH) to compare the differential gene expressions between tumor cells in the primary site and the metastatic sentinel lymph node (SLN) in a patient with ALM. The expressions of target genes in paired paraffin-embedded ALM tissues were analyzed by reverse transcription-PCR (RT-PCR) and immunohistochemical (IHC) staining.

Using the microarray, the top 10 genes which were higher in the SLN than in the primary tumor ($P < 0.05$) were *MGP, IGFBP3, PMEPA1, TGFA, RPS6KA2, CREB5, SALL1, APOD, ITGA9* and *INADL*. In contrast, the top 10 genes which were higher in the primary tumor than the SLN ($P < 0.005$) were *CTAG1A/CTAG1B, WFDC1, CTAG2, SERPINF1, CXorf48, CHST9, LOC285401, LOC283352, CRIM1,* and *C9orf93*. The expression of *MGP* gene was 100-fold higher in the tumor cells in the SLN than in the primary site. Therefore, we have analyzed the expression of MGP gene by RT-PCR and IHC staining in 16 paired paraffin-embedded tissues of ALM. The results will be presented at the meeting.

MGP could be a candidate target to investigate the mechanisms of tumor invasion and metastasis in patients with ALM.

P058

Concomitant blockade of BRAF and mTOR pathway might be required for effective inhibition of malignant melanoma

Y.-L. Cheng[1], C.-H. Hsieh[1], L.-Y. Lee[2], C.-E. Wu[1], W.-K. Huang[1], Y.-F. Lo[3], J.-J. Hsieh[1], H.-Y. Cheng[1], Y.-C. Shen[1], Y.-C. Lin[1], J. W.-C. Chang[1]

[1]Division of Hematology-Oncology, Chang Gung Memorial Hospital; [2]Department of Pathology, Chang Gung Memorial Hospital; [3]Department of General Surgery, Chang Gung Memorial Hospital, Taoyuan, Taiwan ROC

Metastatic melanoma is the most aggressive type of skin cancer for which many new drugs have been approved in recent years following advances in the understanding of melanoma biology and tumor immunology. More than 50% of cases of the *BRAF* mutation have been found in patients with melanoma, which constitutively activates downstream signal transduction in the

 © 2014 John Wiley & Sons A/S. Published by John Wiley & Sons Ltd

MAPK pathway. However, emerging evidence suggests that the mTOR pathway may contribute to resistance to *BRAF* therapy. In addition, only a few Asian studies have addressed this issue to date. This study aimed to elucidate the correlation between *BRAF* mutations and target expressions in the MAPK and mTOR pathways in Taiwanese patients with melanoma. The hypothesis of dual blockade strategy (simultaneously blockade both the MAPK and mTOR pathways) is considered to be a rationale for new therapy with better efficacy and response of duration.
Three cell lines including A375, MS-1 and MC-2 were used in this study. The latter 2 cell lines from Taiwanese patients with acral lentiginous melanoma (ALM) and nodular melanoma (NM), respectively, were successfully cultured at Chang Gung Memorial Hospital at Linkou. *BRAF* (exon 15), *NRAS* (exon 1, 2), *MEK* (exon 3, 6) and *PI3K3CA* (exon 9, 20) mutations were analyzed by direct sequencing. Western blotting was performed for both the MAPK and mTOR pathways in the 3 cell lines. Rapamycin, sorafenib and dabrafenib were applied for 24 h to observe the efficacy of tumor inhibition in single drug or combined regimens.
In mutational analysis, the $BRAF^{V600E}$ mutation was detected in the A375 and MC-2 cell lines. However, no known mutation were found in the *NRAS*, *MEK* and *PI3KCA* genes. Western blotting analysis showed a marked activation of the MAPK pathway in the MC-2 cell line compared with the A375 ($BRAF^{V600E}$ and $PTEN^{WT}$) and MS-1 ($BRAF^{WT}$ and $PTEN^{WT}$) cell lines. We also found that the expression and activation of the mTOR pathway was obviously elevated in MC-2 cells harboring $BRAF^{V600E}$ and $PTEN^{loss}$. The most promising inhibition effects were when rapamycin and dabrafenib were combined compared to rapamycin and sorafenib.
In Western (A375) and Asian melanoma cell lines (MC-2 and MS-1), dual blockade inhibition may be a promising therapeutic strategy. Further prospective clinical trials to elucidate the efficacy and toxicity in melanoma treatment are warranted.

P059

Glucose transporter isoform 1 (GLUT1) expression determines tumorigenicity of melanoma cells and their potential to form hepatic metastasis

A. Koch[1], A. Bosserhoff[1], C. Hellerbrand[1]
[1]University Hospital Regensburg, Regensburg, Germany

Approximately 15% of patients with malignant melanoma either present with metastatic disease or develop metastases during the course of their illness. Metastatic malignant melanoma remains a challenge with limited treatment options and median overall less than 9 months. The liver is one of the primary sites of metastasis and high portal glucose levels may be one of the factors supporting tumor growth in the liver. The facilitative glucose transporter isoform 1 (GLUT1) is known to be the key rate-limiting factor in glucose transport into cancer cells, and herewith, to promote tumor growth. Data on GLUT1 expression in malignant melanoma have been sparse and the role of GLUT1 in (hepatic) metastasis was not known.
The aim of this study was to analyze whether GLUT1 expression and a high capacity for glucose uptake, respectively, do affect hepatic metastasis of malignant melanoma.
Methods and ResultsWhile GLUT1 expression in hepatocytes and normal liver tissue was below the detection limit, GLUT1 expression was enhanced in melanoma cell lines compared to primary melanocytes, as well as in melanoma compared to naevi. Furthermore, immunohistochemical analysis of a tissue micro array consisting of 140 human melanoma tissues showed that GLUT1 expression was significantly enhanced in metastasis compared to primary tumors. GLUT1 expression in primary tumors correlated with tumor staging, and most importantly, with progression- and overall-survival, which are known to be determined by metastasis in this tumor. To determine the role of GLUT1 in melanoma metastasis, GLUT1 expression was suppressed in the murine melanoma cell line B16 (i) by stable transfection with shRNA and (ii) by using the selective GLUT1-Inhibitor WZB117. GLUT1 suppression caused decreased anaerobic glycolysis and lactate secretion, and inhibited proliferation and migration of B16 cells. Moreover, GLUT1 suppression lowered apoptosis resistance of melanoma cells. Next, B16 cell clones with and without GLUT1 suppression were subjected to an established model of hepatic metastasis, in which tumor cells were injected into the spleen of syngeneic mice, from where they metastasize into the liver *via* the portal circulation. GLUT1 suppressed cells formed significantly less metastases than mock-transfected controls. Furthermore, hepatic metastases derived from GLUT1 suppressed B16 cells revealed less immune-cell infiltration and more apoptosis as assessed by CD3-immunohistochemistry and TUNEL staining.
Our data promote the hypothesis that high glucose levels in the portal circulation and the liver, and the capacity to utilize those, respectively, promote hepatic metastasis of malignant melanoma. Our data indicate enhanced apoptosis resistance of tumor cells and known immunomodulatory effects of lactate as potential underlying mechanisms of this phenomenon. GLUT1, which is almost selectively expressed in malignant cells but not in healthy liver or other non-malignant tissues, appears as an attractive therapeutic target for hepatic metastasis.

P060

Detection of circulating melanoma cells in melanoma patients

T. Hida[1], K. Wakamatsu[2], T. Yamashita[1]
[1]Department of Dermatology, Sapporo Medical University School of Medicine, Sapporo; [2]Department of Chemistry, Fujita Health University School of Health Sciences, Toyoake, Japan

Metastasis or recurrence is frequently observed in melanoma patients after the initial surgical treatment. Serum biomarkers have been employed to detect melanoma metastasis and to estimate the prognosis but most do not respond until the tumor burden becomes high. The existence of circulating melanoma cells (CMCs) that are released by a tumor into the blood indicate systemic subclinical disease. Detection of CMCs may be of benefit for detecting early melanoma metastasis, monitoring treatment failure of certain therapeutics and predicting the prognosis.
To examine the validity of existence of CMCs in the clinical assessment of melanoma patients.
Sixteen melanoma patients with various clinical stages who had undergone the initial surgical treatment were prospectively enrolled. Peripheral blood was collected and analyzed by CellSerch system with CellTracks Circulating Melanoma Cell kit (Veridex) according to the manufacturer's instructions. Briefly, CD146-positive cells were magnetically separated from the bulk of other cells in the blood and stained with antibodies against high molecular weight melanoma-associated antigen (HMW-MAA), CD34 and CD45. HMW-HAA+, CD34- and CD45- cells were regarded as CMCs and enumerated. Serum 5-S-cysteinyldopa (5-S-CD) was simultaneously measured. The cut-

© 2014 John Wiley & Sons A/S. Published by John Wiley & Sons Ltd

off values of 2 cells and 10 nmol/l for CMCs and 5-S-CD were applied, respectively.
Among six patients with clinically detectable distant metastasis, one had 11 CMCs without 5-S-CD elevation, one had 3 CMCs with elevated 5-S-CD and two had elevated 5-S-CD without CMC detection. The sensitivity of the CMC detection and the serum 5-S-CD was 33% and 50%, respectively. When the CMC detection and the serum 5-S-CD were combined, the sensitivity was 67%. Among these patients, the mean disease-specific survival of CMC-positive and -negative patients was 5 and 10 months, respectively. Among the other ten melanoma patients who had no detectable metastasis, one had elevated 5-S-CD but none had any CMCs.
This study revealed that the CMC detection has potential to be a useful marker for detecting melanoma metastasis. However, the combination of the CMC detection and the serum 5-S-CD was more reliable for detecting metastasis because the sensitivity of CMC was relatively low. Additionally, the fact that CMC-positive patients tended to have shorter disease-specific survival indicates that CMC detection may also have the potential to estimate the prognosis of late-stage melanoma patients. One limitation of this study is its small sample size. Further study is needed to validate the clinical efficacy of CMC.

P061

The correlation between survival and expression status of BRAF, targets of mTOR, cyclin-dependent kinase 4 and cyclin d1 in patients with acral lentiginous melanoma in Taiwan

C.-H. Hsieh[1], Y.-L. Cheng[1], L.-Y. Lee[2], C.-E. Wu[1], W.-K. Huang[1], Y.-F. Lo[3], J.-J. Hsieh[1], H.-Y. Cheng[1], Y.-C. Shen[1], Y.-C. Lin[1], J. W.-C. Chang[1]

[1]Division of Hematology-Oncology, Chang Gung Memorial Hospital; [2]Department of Pathology, Chang Gung Memorial Hospital; [3]Department of General Surgery, Chang Gung Memorial Hospital, Taoyuan, Taiwan ROC

Metastatic melanoma is the most aggressive type of skin cancer for which many new drugs have been approved in recent years following advances in the understanding of melanoma biology and tumor immunology. More than 50% of cases of the *BRAF* mutation have been found in patients with melanoma, which constitutively activates downstream signal transduction in the MAPK pathway. However, emerging evidence suggests that the mTOR pathway may contribute to resistance to *BRAF* therapy. In addition, only a few Asian studies have addressed this issue to date. This study aimed to elucidate the correlation between *BRAF* mutations and target expressions in the MAPK and mTOR pathways in Taiwanese patients with melanoma.
Seventy-seven patients with histologically proven melanoma treated at Chang Gung Memorial Hospital (Linkou) from June 2001 to October 2010 were retrieved for *BRAF* mutational analysis. Thirty patients with acral lentiginous melanoma (ALM) were selected for analysis of the expression and activation of the mTOR pathway by immunohistochemical staining. Western blotting was performed to determine the expression and activation of both the MAPK and mTOR pathways in three cell lines harboring different genetic Backgrounds: A375 (Western, $BRAF^{V600E}$ and $PTEN^{WT}$), MC-2 (Asian, $BRAF^{V600E}$ and *PTEN* unknown) and MS-1 (Asian, $BRAF^{WT}$ and *PTEN* unknown).
BRAF gene mutations were observed in 12 (15.6%) of the 77 cases, including 2 $BRAF^{V600E}$ in 40 cases of ALM, 1 $BRAF^{V600E}$ in 15 MM[MH1], 5 $BRAF^{V600E}$ and 1 $BRAF^{V600K}$ in 11 NM[MH2], 2 $BRAF^{V600E}$ in 5 SSM, [MH3] and 1 $BRAF^{V600E}$ in 6 unknown types of melanoma. In total, 11 (14.3%) $BRAF^{V600E}$ and 1 (1.3%) $BRAF^{V600K}$ mutations were found in the 77 Taiwanese patients with melanoma. *BRAF* and targets of mTOR pathway expression status were prognostic. Using immunohistochemical staining, the expression of mTOR was observed in 14 (87.5%) of 16 patients with ALM. The activation of mTOR downstream pS6 was observed in 15 (93.8%) of 16 patients with ALM. Expressions of cyclin-dependent kinase 4 (CDK4) and cyclin D1 (CCND1), downstream components of the RAS-BRAF pathway, were observed in 14 (87.5%) and 16 (100%) of 16 patients with ALM, respectively. A high intensity of CDK4 expression was correlated to a better prognosis in disease-free survival and overall survival, whereas a high percentage of the expression of CDK4 was associated with a poor prognosis both in disease-free survival and overall survival. Similarly, a high percentage of CCND1 expression was associated with a poor prognosis.
Expressions of the MAPK and mTOR pathways were highly correlated to the status of *BRAF* mutation and *PTEN*. These findings may help in the development of treatment strategies targeting CCND1 and CDK4 in Asian patients with melanoma.

P062

LKB1 gene alterations in acral lentiginous melanoma

J.-J. Hsieh[1,2], J. W.-C. Chang[2], H.-Y. Cheng[2], Y.-C. Shen[1,2], C.-E. Wu[2], C.-H. Hsieh[2], W.-K. Huang[2], T. Hsu[1]

[1]Institute of Bioscience and Biotechnology and Center of Excellence for Marine Bioenvironment and Biotechnology, National Taiwan Ocean University, Keelung; [2]Division of Hematology-Oncology, Chang Gung Memorial Hospital, Taoyuan, Taiwan ROC

LKB1 has been demonstrated to be the underlying control element in Peutz-Jeghers syndrome, a proliferative melanocytic genetically dominant disorder. It controls certain pathways and as a result can be considered as a candidate in the development and progression of melanoma. LKB1 mutations have been reported to occur in 10% of cutaneous melanomas. Somatic alterations in LKB1 have not been identified in acral lentiginous melanoma (ALM) which is the most common type of malignant melanoma in Taiwan. The aim of this study was to investigate LKB1 gene alterations in patients with ALM.
The mutational hot spots of the LKB1 gene in patients with ALM were examined. Exons 3, 4, and 5 of the LKB1 gene, exon 15 of the BRAF gene, and exons 2 and 3 of the NRAS gene were sequenced in 3 melanoma cell lines and tumor samples from 30 Taiwanese patients with ALM.
In 13 evaluable ALM, 10 patients had LKB1 gene alterations (77%), including 3 heterozygous and 7 homozygous 7-bp duplications in intron 3. None of the 10 patients with an LKB1 alteration showed either a BRAF or NRAS mutation. No LKB1 mutations in exon 3, 4, and 5 were observed in any of the patients. One melanoma cell line (MS-1) had LKB1 gene alterations with homozygous 7-bp duplication in intron 3 and wild type BRAF and NRAS genes. The other 2 cell lines had no gene alterations in LKB1, BRAF, and NRAS genes.
This study demonstrated the high percentage of the 7-bp duplication polymorphism in intron 3 of LKB1 gene in Taiwanese patients with ALM.

 © 2014 John Wiley & Sons A/S. Published by John Wiley & Sons Ltd

P063

Cancer associated fibroblasts influence tumor microenvironment of melanoma

O. Kodet[1,2], J. Kucera[1,2], L. Lacina[3], B. Dvorankova[1], K. Smetana[1]

[1]Institute of Anatomy, 1st Faculty of Medicine, Charles University; [2]Department of Dermatology and Venerology, 1st Faculty of Medicine, Charles University, Prague, Czech Republic; [3]Institute of Medical Biology, A*STAR, Singapore City, Singapore

The tumor microenvironment plays an essential role in the biology and behavior of tumors, which is already well documented in different types of tumors. Cancer associated fibroblasts (CAFs) are component of the tumor stroma. The function of CAFs we already well documented in tumors derivatived from squamous epithelium from head and neck and skin. Similar mechanisms can be also expected in melanoma.

We isolated the CAFs from skin metastasis of melanoma and we tasted the biological effect to the two similar melanoma lines, which were negative for typical melanocytic markers (HMB45, melan-A/MART-1 and tyrosinase), but there were positivity only for S100. We used this cell lines in indirect culture with aim to monitor changes at the level of differentiation markers in melanoma lines. On the other hand, the biological activity of CAFs we can documented in model with normal keratinocytes. CAFs from melanoma are able to induce the expression of keratin 19, keratin 14 and keratin 8 in normal keratinocytes. This biological function is practically uniform in compared with the function of CAFs from other types of tumors such as squamous cell carcinoma of the oral mucosa. We also demonstrated the influence of CAFs from melanoma in long-term culture with 3T3 fibroblasts. CAFs are able to induce expression of multipotent stem cells markers such Oct4 and Nanog in 3T3 fibroblasts, like CAFs from basal cell carcinoma of the skin.

The result of this study demonstrated the biological function of CAFs and their possible influence to the expression of melanocytic markers (HMB45, melan-A/MART-1 and tyrosinase) and also their effect to the normal keratinocytes. We also demonstrated biological effect of CAFs to induction of stem cells markers in 3T3 fibroblasts.

The biological effect of CAFs is very similar in CAFs derivated from different types of tumors and their role in cancer progression despite melanoma may by crucial to keep the tumor microenvironment.

Supported by the Grant Agency of the Czech Republic No. 304/12/1333 and by the Charles University project PRVOUK 27 and Biotechnology and Biomedicine Center of the Academy of Science and Charles University in Vestec.

P064

Tumor melanocytes are responsible for pseudohyperplasia of epidermis in nodular melanoma: in vivo and in vitro study

J. Kučera[1], O. Kodet[1], L. Lacina[1,2], B. Dvorankova[1], M. Kolář[3], H. Strnad[3], K. Smetana[1]

[1]Anatomický ústav 1.LF UK, Praha, Czech Republic; [2]Institute of Medical Biology, A*STAR - Agency for Science, Technology and Research, Singapore City, Singapore; [3]Ústav molekulární genetiky AV CR, Praha, Czech Republic

Nodular melanoma (NM) is one of the most life threatening tumors with frequently poor therapeutic outcome. Similarly to the other tumors, the intercellular interactions may be of crucial importance for biological properties of melanoma and course of the disease. Interaction of tumor melanocytes and stromal components play an important role in microenvironment of melanoma, but data about the interaction of the tumor melanocytes and keratinocytes are limited. Some studies demonstrated the dependence of the tumor melanocytes to the surrounding keratinocytes during vertical growth phase. This study is focused on the influence of the tumor melanocytes on differentiation pattern of normal keratinocytes.

100 samples of NM removed during radical melanoma resection were analyzed by routine histology and immunohistochemistry. The data were compared with in vitro model. For in vitro study of intercellular crosstalk we co-cultured in transwell system melanoma cells, neonatal melanocytes and neural crest stem cells (as precursors of melanocytes) respectively with human normal keratinocytes. Transcriptional analysis of these models was carried out on the whole genome level by using Illumina microarrays.

Pseudohyperplastic change of the epithelium showed significantly four and three times thicker of the epidermal layer in the periphery of the tumors compared to the surgical margin. Pseudohyperplastic character of the epithelium was supported by the aberrant suprabasal expression of keratin 14 (K14) and K10. In vitro models revealed that melanoma cells were able to influence expression of K14, K19, K8 and also vimentin in co-cultured keratinocytes. This model was compared with the neural crest stem cells, which were able to very similarly influence the normal keratinocytes, but neonatal melanocytes did not. We detected cytokines and growth factors such as FGF2, CXCL1, IL8 and VEGFA, which seem to participate in this activity of the melanoma cell to the keratinocytes, by transcriptional analysis.

Melanoma cells are able to modify the landscape of tissue surrounding the proliferating malignant clone. Melanoma-epidermal interactions result in maintenance of low differentiation status of keratinocytes in vivo as well as in vitro. This interaction further highlights the role of intercellular interactions in melanoma, but the functional significance for melanoma biology requires further analysis.

This study was supported by the Grant Agency of the Czech Republic, project no 304/121/333 and by Charles University, projects PRVOUK 27-1, UNCE 204013, SVV 264510 and project BIOCEV.

Prof.MUDr.Karel Smetana,DrSc.

Anatomicky ustav 1.LF UK: U nemocnice 3, 128 00 Praha 2, Tel.: 222 496 5873, E-mail: ksmet@lf1.cuni.cz.

© 2014 John Wiley & Sons A/S. Published by John Wiley & Sons Ltd

P065

Benefit of using dermoscopy in revealing thin melanomas in Southestern Europe

D. V. Nikolic[1], A. T. Nikolic[2,2], V. V. Stanimirovic[3], J. Gacic[4]

[1]Oncosurgery, Medical Faculty Belgrade, UMC Bezanijska kosa; [2]Internal Medicine, Medical Faculty Belgrade, UMC Bezanijska kosa; [3]ALIMS, UMC Bezanijska kosa; [4]Oncosurgery, UMC Bezanijska kosa, Belgrade, Serbia

Melanoma is one the most malignant tumors of the human body. Incidence is rising every year, from 60.000 new cases in 2005 to 200.000 cases in 2008.The majority of them will die in the first five years of the diagnosis, specially if the intial diagnosis of melanoma is deeper then 2 mm, according to Breslow classification.Melanoma represents arround 5% of skin camcers but is responsible for the 65% of deaths. It is the most incereasing malignancy in men, and second increasing malignancy in women.

The majority of pigmented skin lesions can be diagnosed correctly on the basis of clinical criteria; however, there remain a surprisingly high number of small pigmented lesions in which the distinction between melanocytic and non-melanocytic and benign and malignant lesions, and thus between melanoma and non-melanoma, is difficult or impossible to make with the naked eye. Dermoscopy is a non-invasive technique that, by use of oil immersion, makes sub-surface structures of skin accessible for in vivo microscopic examination and thus provides additional criteria for the diagnosis of pigmented lesions.

Patients and MethodsDuring the period of ten years, 1999–2009, 648 patients with melanoma were revealed in the Melanoma Center of Serbia, University Medical Center Bezanijska Kosa.

During the period of ten years, we examined more then 30.000 patients with the total number of more then 500.000 images with *MoleMax II,* an integrated system for digital epiluminescence microscopy based on a new concept of illumination. This system combines an easy to use microscope with excellent optical quality and modern computer technology for efficient data storage and retrieval. This system can store thousands of images and they are immediately available for comparison. Therefore, simultaneous dermatologist/patient on screen observation of potential mole alteration is made possible. The integrated system has capability to recall and display stored images.

From the total number of melanoma patients for the last ten years, 37% (241) patients were found with thin melanomas, melanomas that are thinner then 2 mm, according to Breslow classification. From the total number of 241 thin melanomas, 6% (40) of them were melanoma in situ.

This result of revealed thin melanoms talks about the improvement of diagnose of melanocytic tumors of the skin with dermoscopy, in significant percent comparing with earlier statistics of thin melanomas versus thick melanoms (less then 2 mm versus more then 2 mm), when thin melanomas were present in only less then 20%, and melanoma in situ were present in about less then 3%. It is good to say that melanoma campaign that is world wide spread last years rise up the thinking of early diagnostic of melanomas, specially with dermoscopy, as a good too,l that can cure in a big percent, patients with melanomas, if they are revealed on time-when melanomas are less then 1 mm or in the stage of melanoma in situ.

P066

World Wide Web e melanoma register: myth or reality

D. V. Nikolic[1,2], A. T. Nikolic[3], V. V. Stanimirovic[4], J. Gacic[5]

[1]Oncosurgery, Medical Faculty Belgrade, UMC Bezanijska kosa; [2]Oncosurgery, Faculty of Medicine, UMC Bezanijska kosa; [3]Faculty of Medicine, ICVD, Noninvasive Department, UMC Bezanijska kosa; [4]ALIMS, UMC Bezanijska kosa; [5]Oncosurgery, UMC Bezanijska kosa, Belgrade, Serbia

Cutaneous melanoma is often lethal disease and its incidence world wide has increased rapidly over the past two decades. Early detection and treatment of melanoma might reduce mortality, while early detection and treatment of nonmelanoma skin cancer might prevent major disfigurement.Current recommendations from different professional medical associations regarding screening and therapy for melanoma vary.

Patients and MethodsWe analyzed data that were possible to find using internet browsers and different medical libriries like PubMed. We made e melanom register with all data included (like complete HP data of melanoma) for the patients for whole districts in Republic of Serbia, population of seven million.

Different studies were done based on different population-based cancer registries, where examining all-ages and age-truncated standardized incidence rates of melanoma, estimating the annual percentage change and incidence rate ratios from age-period-cohort models were done. Incidence rates of melanoma continue to rise in most European countries (primarily Southern and Eastern Europe), especially in rural part of countries, whereas in Australia, New Zealand, the U.S., Canada, Israel and Norway, rates have become rather stable in recent years. Indications of stabilization or decreasing trend were observed mainly in the youngest age group (25–44 yrs).

Disscusion:Together with melanoma campaign and Euromelanoma day every year, it will be good to standardize all items and try to arrange for the whole world e melanoma register like we did it in Serbia, and in that way it will be worth to have all patients on one web site where differrent users and professionals will be able to have all datas concerning melanomas. Having such large informations we will be able to precise all about melanomas in the whole world what will be very usefull in revealing early melanomas.

P067

Cell elasticity is an important indicator of the invasive abilities of pigmented melanoma cells

M. Sarna[1,2], A. Zadlo[1], K. Burda[2], T. Sarna[1]

[1]Department of Biophysics, Jagiellonian University; [2]Department of Medical Physics and Biophysics, AGH University of Science and Technology, Krakow, Poland

The relationship between melanin pigmentation and metastatic phenotype of melanoma cells is an intricate issue, which needs to be unambiguously elucidated to fully understand the process of metastasis of malignant melanoma. In spite of significant research efforts to solve this long lasting puzzle, the outcomes are not satisfying. Importantly, none of the proposed explanations take into consideration mechanical effects of melanin presence on the invasive abilities of melanoma cells. In recent years, different biomechanical assays demonstrated that cell elasticity plays an important role in the process of metastasis of cancer cells, particularly during their invasion. Recently, we have shown that melanin granules dramatically modify the elastic properties of pigmented melanoma cells (1, 2).

 © 2014 John Wiley & Sons A/S. Published by John Wiley & Sons Ltd

This observation prompted us to examine mechanical effects of melanin granules on the invasive abilities of melanoma cells. Using an array of advanced biophysical techniques such as atomic force microscopy (AFM), electron paramagnetic resonance (EPR), laser scanning confocal microscopy (LSCM) and complementary cell biology techniques, we have shown that melanin granules inhibit the invasive abilities of melanoma cells under in vitro conditions in a number of granules dependant manner. Moreover, we showed that the inhibitory effect of melanosomes was mechanical in nature due to dramatic modification of the cells elastic properties. Our findings demonstrate that cell elasticity may be an important indicator of the metastatic phenotype of melanoma cells in vivo. Furthermore, the obtained results may lead to better understanding of the process of metastasis of malignant melanoma.

This work was supported in part by Poland Ministry of Science and Higher Education (Statutory Activity of the Faculty of Biochemistry, Biophysics and Biotechnology, Jagiellonian University – DS 16).

References

1. Sarna, M. *et al.* Melanin modifies nanomechanical properties of pigmented melanoma cells. In: *The melanocyte and Its Environment*. 23–27 (Medimond, 2012).
2. Sarna, M. *et al.* Nanomechanical analysis of pigmented human melanoma cells. *Pig. Cell Mel. Res.* 26: 727–730 (2013).

P068

Prognostic significance of mi-RNA211 in cutaneous melanoma

N. Maddodi[1], J. Berlyn[1], T. Shekhani[1], S. Rajguru[2], J. B. Longley[1], M. R. Albertini[3], W. Huang[4], M. A. Newton[5], V. Setaluri[1]

[1]Dermatology, University of Wisconsin-Madison; [2]Medicine-Clinical Oncology, University of Wisconsin-Madison; [3]Medicine, University of Wisconsin-Madison; [4]Pathology & Laboratory Medicine, University of Wisconsin-Madison; [5]Statistics, University of Wisconsin-Madison, Madison, WI, USA

Micro RNA-211 is among the most specifically and highly expressed miRNAs in cutaneous melanocytes. miR-211 is present within an intron of an MITF-regulated gene transient receptor potential, melastatin 1 (TRPM1). There is evidence that miR-211 expression is strictly correlated with TRPM1 expression and regulated by MITF. Several studies have documented that miR-211 expression is down-regulated or absent in malignant melanoma implicating a tumor suppressor role for miR-211. However, there are contradictory reports on the relative expression levels of miR-211 in normal melanocytes and melanoma cell lines and malignant melanocytic lesions. Although expression of TRPM1, hence miR-211, is known to be inversely correlated with aggressiveness, the prognostic significance of miRNA-211 in cutaneous primary melanoma has not been performed. We investigated whether miR-211 is regulated independently from the host gene promoter and examined the prognostic value of miR-211 expression in primary cutaneous malignant lesions. Here, we report that a1.4 kb genomic DNA fragment of *TRPM1* intron 6 containing miRNA-211, cloned into a luciferase reporter plasmid, is sufficient to drive expression of miRNA-211 (and the downstream luciferase) in melanoma cells. These data suggest presence of a putative promoter in the intron 6 of TRPM1 that can regulate miR-211 expression of its host gene. To investigate the relationship of miR-211 to early stages in melanoma progression, we constructed a tissue microarray consisting of >150 primary melanoma specimens and performed quantitative in situ hybridization analysis for miR-211 expression. Using stringent criteria for miR-211 expression (percent cells positive as well as signal intensity), we show that miR-211 expression is inversely correlated with Breslow thickness. Kaplan-Meier survival analysis of primary melanoma patients with disease recurrence showed that miR-211 expression is associated with better prognosis. Our results show that miR-211 can be regulated independent of its host gene and serve as a useful prognostic indicator of aggressiveness of primary melanoma.

P069

Incidence of BRAF and C-KIT mutations in Taiwanese melanoma

Y.-C. Shen[1,2], J. W.-C. Chang[1], J.-J. Hsieh[1,2], H.-Y. Cheng[1], M.-M. Hou[1], C.-H. Hsieh[1], W.-K. Huang[1], C.-E. Wu[1], T. Hsu[2]

[1]Division of Hematology-Oncology, Chang Gung Memorial Hospital, Taoyuan; [2]Institute of Bioscience and Biotechnology and Center of Excellence for Marine Bioenvironment and Biotechnology, National Taiwan Ocean University, Keelung, Taiwan ROC

Recent studies have shown that novel agents targeting on BRAF and c-KIT appears to be effective in treating melanoma. However, only a few reports were presented in Asian countries. A report from China demonstrated c-KIT mutations are common in mucosal and acral melanomas, which comprise greater than 70% of all melanomas in Asia. Since pathological type and sun-exposure are associated with genetic alterations in melanoma, we investigate the incidence rate in Taiwan.

To analyze the incidence of BRAF and c-KIT mutation in Taiwanese melanoma, we sequenced exon 15 of BRAF gene and exons 9, 11, 13, 17, and 18 of c-KIT gene in 108 and 50 melanomas, respectively by Sanger sequencing and peptide nucleic acid - locked nucleic acid (PNA-LNA) clamping, using archived paraffin-embedded tissue. We also categorized BRAF and c-KIT mutations by tumor type, age and gender of patients. In all melanoma. BRAF and c-KIT mutation rates were 18.5% and 8%, respectively. The BRAF mutation rates in acral, mucosal, and cutaneous melanomas were 6.4%, 4.4%, and 46.4%, respectively. The BRAF V600E comprised 95% of all BRAF mutations. The c-KIT mutation rates in acral, mucosal, and cutaneous melanomas were 15%, 0%, and 0%, respectively. In 4 melanomas with c-KIT mutation, 2 expressed silence mutation (L862L and P574P), one expressed L576P point mutation, and one expressed a double mutation (V560G and G487V).

The incidence rate of BRAF and c-KIT mutations is low in Taiwan. Novel therapy targeting on other genes are needed.

P070

Tumor type unspecific biological activity of cancer associated fibroblast on malignant epithelial cells

P. Szabo[1], B. Dvorankova[1], K. Smetana[1], H. Strnad[2], M. Kolar[2]

[1]Institute of Anatomy, First Faculty of Medicine, Charles University; [2]Institute of Molecular Genetics, Academy of Science of Czech Republic, Prague, Czech Republic

Many results demostrate that cancer cells need for their growth and spread through organism a specific microenvironment-the tumor stroma. Carcinomas represent highly complex tissue composed from cancer cells and stroma including fibroblasts producing extracellular matrix and bioactive substances,

© 2014 John Wiley & Sons A/S. Published by John Wiley & Sons Ltd

inflamantory cells and blood vessels. We focused our research on most abundant cell component of cancer stroma on cancer associated fibroblasts. We isolated stromal fibroblasts from the squamous epithelium such as basal and squamous cell carcinoma, melanoma and skin metastasis of breast cancer. We expanded them and in vitro evaluated their biological effect on normal keratinocytes and breast cancer keratinocytes (EMG-3). The results were compared with control experiments using normal human dermal fibroblasts, 3T3 mouse fibroblasts and 3T3 fibroblasts influenced by the fibroblasts prepared from the basal cell carcinoma. Our results demostrated that expression of luminal marker keratin eight influenced only by cancer associated fibroblasts prepared from any tested tumors. In contrast, all tested types of fibroblasts showed a strong stimulatory effect on the expression of basal/myoepithelial marker keratin 14. Since keratin 14 is a marker of basal myoepithelial cells and keratin eight is a marker of luminal cells, these double positive cells can be considered for precursor cells with properties close to stem cells. Their presence in clinical samples indeed signals very poor prognosis in cancer suffering patients. In Conclusion, our data indicate that cancer associated fibroblasts are able to influence the phenotype of breast cancer cell line and this effect is based on a tumor type-unspecific mechanism.

P071

Comparison of immunohistochemical analysis and DNA sequencing in the detection of the BRAF V600E mutation among Asian patients with primary and metastatic melanoma

T.-L. Wu[1], W.-K. Huang[2], J. W.-C. Chang[2], J.-J. Hsieh[2], H.-Y. Cheng[2], C.-H. Hsieh[2], C.-E. Wu[2], T.-T. Kuo[3], K.-Y. Yeh[1]

[1]Division of Hematology-Oncology, Chang Gung Memorial Hospital, Keelung; [2]Division of Hematology-Oncology, Chang Gung Memorial Hospital; [3]Department of Pathology, Chang Gung Memorial Hospital, Taoyuan, Taiwan ROC

To determine the sensitivity and specificity of immunohistochemistry using the VE1 antibody compared with DNA sequencing in Taiwanese patients with melanoma harboring the BRAF V600E mutation.

Patients with a pathological diagnosis of melanoma from 2011 to 2013 were enrolled. All immunostained slides were evaluated by a senior pathologist blinded to all clinical and genetic data. Immunoreaction was scored positive when viable tumor cells showed non-ambiguous cytoplasmic staining for VE1. Faint diffuse staining, any type of isolated nuclear staining, weak staining of single interspersed cells or staining of monocytes/macrophages was scored negative. Paraffin-embedded, formalin-fixed melanoma biopsies were analyzed for the BRAF mutation using both conventional DNA molecular techniques including direct sequencing and the PNA-LNA clamping technique.

Among the 38 patients with melanoma tissues, 14 (37%) had acral lentiginous melanoma. DNA analysis revealed that 13 of the 38 (35%) cases with melanoma tissues possessed the BRAF V600E mutation, and 1 (2.6%) had the BRAF V600K mutation. In immunohistochemical analysis, cytoplasmic positivity with anti-BRAF was noted in 17 of the 38 (44%) cases. Statistical analysis of the data showed that the sensitivity and specificity of the immunohistochemistry were 100% and 89%, respectively.

This study demonstrated the potential use of immunohistochemistry using the VE1 antibody as an ancillary screening tool to assess the BRAF V600E mutation status in Asian patients with melanoma.

Senescence Pathways to Melanoma

P072

NRAS and BRAF mutation and protein expression analysis in a series of primary oral mucosal melanoma

R. Hsieh[1,2], C. M. Coutinho-Camillo[3], J. D. Fernandes[4], M. M. S. Nico[1,5], S. V. Lourenco[1,2]

[1]LIM06, Institute of Tropical Medicine; [2]Dental School, University of Sao Paulo; [3]Centro Internacional de Pesquisa, AC Camargo Cancer Center, Sao Paulo; [4]Medical School, Federal University of Bahia, Salvador; [5]Medical School, University of Sao Paulo, Sao Paulo, Brazil

Backround: Primary Oral Mucosal Melanoma is an extremely rare and aggressive tumor arising from melanocytes located in the mucosal epithelium of the oral cavity. Although malignant melanoma of oral mucosa shares some clinical features with its cutaneous counterpart, it has a poorer prognosis and the etiology and pathogenesis are still poorly understood and there is no influence of UV radiation in the development of this neoplasm. It is known that the MAPK pathway mediates cellular responses to growth signals and its the activation is an important phenomenon in melanoma and thus a possible target for future therapies. Method: point mutation of NRAS (codons 12, 13 and 61) and BRAF (codon 600) were screened by pyrosequencing method in a serie of primary oral mucosal melanoma, and its results were associated to the protein expression of RAS and BRAF performed by immunohistochemistry, data found in a previous study. Results: we observed mutation in BRAF 600 (3/14); NRAS codon 12 and 13 (2/14) and NRAS codon 61 (2/8). One case showed positive RAS protein expression, but no mutation was observed. Twelve in fourteen cases showed positive BRAF protein expression: three cases showed BRAF mutation; two cases showed NRAS codon 61 mutation; two cases showed NRAS codon 12 and 13 mutation but no simultaneously. Discussion: Tumor cells intensively expressed BRAF, but not for RAS protein in these cases. Additionally, low NRAS and BRAf mutation was observed in the cases studied and they were mutually exclusive, so the correlation between NRAS and BRAF protein expressions and mutations was not possible. Conclusion: Although NRAS and BRAF mutation frequency and RAS protein expression are low, BRAF protein expression was intense, probably NRAS and BRAF mutation are independent events and alternative molecular mechanisms in the primary oral mucosal melanoma tumorigenesis.

UV & Non-UV Pathways to Melanoma

P073

Source of reactive oxygen species and their roles in melanoma etiology

F. L. J. Meyskens[1], F. Liu-Smith[1,2], S. Yang[1]

[1]Medicine, UCIrvine; [2]Epidemiology, UCIrvine, Irvine, CA, USA

Reactive oxygen species (ROS) play crucial roles in all aspects of melanoma development, however, the source of ROS is not well defined. The cellular ROS pool in melanocytes can be derived from mitochondria, melanosomes, NADPH oxidase (NOX) and

 © 2014 John Wiley & Sons A/S. Published by John Wiley & Sons Ltd

nitric oxide synthase (NOS) enzymes. While evidence for mitochondria-generated ROS in melanoma etiology are limited, emerging evidence suggests that melanosomes, NADPH oxidase (NOX) and nitric oxide synthase (NOS) are crucial in melanoma etiology. Our recent results demonstrated that Nox1, Nox4 and nNOS are expressed in melanocytic lineage, among which Nox1 and nNOS were UV-inducible. Both Nox1 and nNOS are over-expressed in all melanoma cell lines examined, however there is no difference in Nox1 expression levels in primary and metastatic melanoma tissues, suggesting that these ROS/RNS generating enzymes may play important roles in melanoma etiology. NOX-generating superoxide can interact with NOS-generating NO and form ONOO- radicals which are extremely active and can be an important DNA-damaging agent in melanoma development. To make the situation worse, melanins in melanosomes, especially pheomelanin from pheomelanosome, may be leaking out of the deformed melanosomes in the melanocytes (the deformation may be caused by NOX/NOS-generated ROS or direct UV radiation), thus increase ROS to a high level to cause severe oxidative stress and DNA damage. Our study shows that eumelanin can interact with metals and become pro-oxidants which at least partly explains the high incidence rate of melanoma in metal-on-metal hip replacement patients. Furthermore, inhibiting nNOS by specific nNOS inhibitors reduced both invasion rate in vitro and tumor growth in vivo, validating the nNOS as a potential target for melanoma therapy. Taken together, our data suggest that NOX, NOS and melanins are all effectors for UV radiation and play crucial roles in melanomagenesis, therefore can be served as prevention targets for melanoma.

P074

The mechanism of blue light-induced cell death in melanoma cells

K. Sato[1], T. Nishio[1], T. Mito[1], K. Matsui[1], J. N. Sato[1], H. Watanabe[1,2]

[1]Department of Life Science, Tamagawa University; [2]Biosystems and Biofunctions Research Center, Tamagawa University Research Institute, Tokyo, Japan

Many studies have investigated the biological effects of UV on animal species and cells. It is well known that UV radiation induces cell cycle arrest and cell death, and increases melanin synthesis. However, little is known about the effect of visible light on the metabolism of animal cells. Recently, we reported that blue light induces enhancement of superoxide anion production and depolarization of mitochondrial membrane potential ($\Delta\Psi$m) in B16F1 melanoma cells. $\Delta\Psi$m depolarization is a key event of early apoptosis. Therefore, we hypothesized that blue light can promote oxidative stress and subsequent cell death. In this study, we attempted to elucidate the effect of blue light on the cell cycle and the cell death cascade in B16F1 melanoma cells. We also investigated the possibility of blue light as a melanogenic inducer. We found that blue light significantly inhibited cell proliferation at 50 W/m^2 upon only 30 min of irradiation. Blue light also induced enhancement of the intracellular ROS level and caspase-3 activation. These results indicate that blue light acts as a cell death inducer. It is well known that UV induces melanogenesis via up-regulation of the expression of melanogenic genes, such as tyrosinase, tyrosinase-related protein and microphthalmia-associated transcription factor (MITF). In addition, we performed RT-PCR analysis to elucidate the effect of blue light on MITF mRNA transcription. The results suggest that blue light enhances MITF transcription level in B16F1 melanoma cells. Accordingly, our findings indicate that blue light acts as a potential apoptosis inducer, and visible light possibly induces melanin synthesis.

Genetics & Genomics of melanoma

P075

Snp analysis of KIT gene in melanoma of grey horses

A. Golovko[1], J. M. Völker[1], L. Andersson[1,2]

[1]Science for Life Laboratory, Department Medical Biochemistry and Microbiology, Uppsala University; [2]Department of Animal Breeding and Genetics, Swedish University of Agricultural Sciences, Uppsala, Sweden

Grey horses exhibit a fascinating pigment cell disorder phenotype manifested by gradual loss of coat pigmentation, vitiligo-like skin depigmentation and a high incidence of melanoma. It is estimated that ~80% of Grey horses older than 15 yrs have melanomas, while this is a rare condition in horses with other coat colors. Although most of the melanomas have a long initially benign growth period, up to 66% of these tumors may become malignant with metastases formation in other organs. The tumors arise in the dermis of the glabrous skin under the tail, in the perianal and genital regions, lips and eyelids, but could also occur internally. We have previously screened Grey horse melanomas for activating mutations in BRAF, RAS and GNAQ/GNA11 genes and found none. The pattern of melanoma occurrence in grey horses, with the prevalence in the mucosal sites, may be suggestive of the involvement of mutations in the gene encoding the receptor tyrosine kinase KIT, as found in up to 40% of human mucosal melanomas. To address this issue, we have screened five mucosal and seven non-mucosal tumors from grey horses for mutations in exon 9–21 of KIT gene. We found no mutations that would alter the amino acid sequence of the protein in any of the exons. However, we found 4 synonymous SNPs in exon 14, 15, 19 and 20, respectively. We did not find higher representation of these SNPs in mucosal sites compared to the non-mucosal sites. Another SNP was detected at 3′ of exon 11, potentially affecting the splicing site, which could lid to exon skipping/frame shifting. Interestingly, this SNP was found to have a higher allelic frequency in grey horses compared to non-grey horses. However, we detected no mRNA sequence difference between cell lines with and without the SNP, arguing against its involvement in the RNA splicing. Further analysis, including expansion of the sample size, will help in clarifying the involvement of KIT in melanoma of grey horses.

Melanoma Therapeutics

P076

The roles of thioredoxin reductase 1 in melanomagenesis

P. Cassidy[1], M. Honeggar[2], S. Leachman[1], P. Moos[3]

[1]Dermatology, Oregon Health and Science University; [2]Oregon Health and Science University, Portland; [3]Pharmacology and Toxicology, University of Utah, Salt Lake City, UT, USA

Thioredoxin reductase 1 (TR1) is an antioxidant protein that is important for many diverse cellular processes ranging from the biosynthesis of deoxyribonucleotides to the metabolism of reactive oxygen species. We have found that treatment of

© 2014 John Wiley & Sons A/S. Published by John Wiley & Sons Ltd

human melanocytes with the chemoprevention agent sulforaphane potently upregulates the expression of TR1 and protects melanocytes from UV-induced apoptosis. Remarkably, we have also found that TR1 expression increases in melanocytic lesions during the progression from nevus, to radial and vertical growth phase and metastatic melanoma. Expression levels of TR1 even discriminate between thick and thin primary melanomas. Our insights into the diverse and seemingly contradictory roles played by melanoma in melanocytic cells have led us to the discover that TR1 inhibition sensitizes melanoma to inhibition of glycolytic metabolism, a strategy that holds the promise of a new therapeutic treatment that could quickly be translated into the clinic for treatment of advanced melanoma. In this new work we explore the mechanisms of this therapeutic synergy with the goal of broadening our understanding the role of thioredoxin reductase 1 in melanoma progression. We believe that this insight will help us design more effective prevention agents, and improve upon the performance agents of recently-introduced agents, primarily directed at immune modulation or oncogene addiction, that have have thus far suffered from either limited clinical efficacy or the development of resistance tumor resistance.

P077

In-transit sentinel lymph node for melanoma patients in Taiwan

J. W.-C. Chang[1], C.-E. Wu[1], C.-H. Hsieh[1], W.-K. Huang[1], C.-J. Chang[2], J.-T. Yeh[3], C.-H. Yang[4], Y.-F. Lo[5], K.-J. Lin[6], J.-J. Hsieh[1], T.-T. Kuo[7]

[1]Division of Hematology-Oncology, Chang Gung Memorial Hospital; [2]Department of Plastic Surgery, Chang Gung Memorial Hospital; [3]Department of Plastic and Reconstructive Surgery, Chang Gung Memorial Hospital; [4]Department of Dermatology, Chang Gung Memorial Hospital; [5]Department of General Surgery, Chang Gung Memorial Hospital; [6]Department of Medical Imaging and Radiological Sciences, Chang Gung Memorial Hospital; [7]Department of Pathology, Chang Gung Memorial Hospital, Taoyuan, Taiwan ROC

In-transit sentinel lymph node (SLN) is defined as a lymph node in the area between the primary melanoma and the first regional lymph node basin. However, the clinical significance of in-transit SLN is still unknown. This study aimed to evaluate the role of in-transit SLNs in melanoma patients in Taiwan.

From January 2000 to December 2011, a total of 127 patients underwent successful SLN biopsies at Linkou Chang Gung Memorial Hospital. We retrospectively analyzed the predictors of in-transit SLNs and evaluated the survival outcomes in these patients.

Among the 127 patients, 28 (22%) had in-transit SLNs on lymphoscintigraphy. Twenty-four patients had 1, 3 patients had 2, and 1 patient had 3 in-transit SLNs. In-transit SLNs could not be identified in 8 patients during surgery, so only 20 of the 28 patients underwent excision of the in-transit SLN. Six (30%) of these 20 patients experienced metastatic in-transit SLN, and the remaining 14 had no metastatic SLN. There were no significant differences in clinicopathological features between the patients with and without in-transit SLNs, except that more male patients had in-transit SLNs (67.9% versus 44.1%, $P = 0.03$), and there was a trend of a higher rate of in-transit SLNs in the extremities (100% versus 86.9%, $P = 0.07$). As of July 2013 the median follow-up time was 43.5 months. The disease-free survival (DFS) and overall survival (OS) did not differ between the patients with and without in-transit SLNs (DFS 38.5 versus 54.1 months, $P = 0.983$; OS 112.4 versus 90.9 months, $P = 0.993$), the patients having in-transit SLN with and without excision (DFS, 38.5 versus 24.4 $P = 0.686$; OS 112.4 versus 36.9 months, $P = 0.232$), and the patients with and without metastatic SLN (DFS not reached versus 36.2 months, $P = 0.269$; OS 112.4 versus not reached, $P = 0.419$).

In-transit SLNs did not impact the prognosis of the Taiwanese melanoma patients in this study. The positive in-transit SLN rate of 30% provides evidence that patients should undergo excision of in-transit SLNs even though there was no survival difference between the patients having in-transit SLNs with and without excision.

P078

Result of efficacy and safety of dabrafenib in BRAF V600 mutated melanoma in Taiwan

H. Cheng[1], C.-H. Hsieh[1], J. W.-C. Chang[1], S.-P. Yeh[2], L.-Y. Ou[1], H.-Y. Cheng[1], J.-J. Hsieh[1], Y.-F. Lo[3]

[1]Division of Hematology/Oncology, Department of Internal Medicine, Chang Gung Memorial Hospital, Linkou Branch, Tao Yuan; [2]Division of Hematology/Oncology, Department of Internal Medicine, People's Republic of China Medical University Hospital, Taichung; [3]Department of General Surgery, Chang Gung Memorial Hospital, Linkou Branch, Tao Yuan, Taiwan ROC

Dabrafenib is a potent inhibitor of BRAF kinase activity against BRAF V600 mutated metastatic melanoma. A phase III study of dabrafenib showed significantly improved progression free survival as compared with conventional chemotherapy. We provide a program to supply dabrafenib on compassionate use basis for BRAF V600 mutated melanoma in Taiwan.

We analyzed the efficacy and safety of dabrafenib in patients with BRAF V600 mutated metastatic or unresectable melanoma in Taiwan. These patients all participated in the compassionate access program which had been approved by department of health in Taiwan. The dosage of dabrafenib is 150 mg twice daily, orally.

Approximately 18 patients were diagnosed with metastatic or unresectable BRAF V600 mutated melanoma in Taiwan between Nov, 2011 to Dec, 2013. Unfortunately, five of them had died before the drug is dispensed. Four patients were enrolled into other ongoing clinical trials during initial screening. After reviewing by the GSK medical adviser, one patient with BRAF T500K mutated melanoma dropped out the study due to ineligibility. A total of eight patients had been dosed with dabrafenib. The median progression-free survival (PFS) was 5.3 months. Six patients (75%) achieved partial response (PR) and two patients (25%) have complete response (CR). The rate of total disease control was 100% in the patients eligible for evaluation. Dabrafenib was well tolerated overall, however one patient experienced serious adverse event (SAE) of liver failure and death due to hepatitis C flare up after taking the drug for two weeks. The most common adverse events (AEs) were cutaneous hyperkeratosis, palmar-plantar erythrodysaesthesia, pyrexia, fatigue, headache, and arthralgia.

From the compassionate use program experience, dabrafenib demonstrated significant activity in BRAF V600 mutated melanoma in Taiwan.

 © 2014 John Wiley & Sons A/S. Published by John Wiley & Sons Ltd

P079

Nodular type of malignant melanoma, nodes and lung metastasis with good medical status

H. Fathan[1], I. Widyasari[1], A. U. Hasanah[1], H. Cipto[1], A. S. Dahlan Suriadiredja[1], I. A. Krisanti[1], L. Paramita[1], A. T. Sampurna[1]

[1]Dermatovenereology, Faculty of Medicine, University of Indonesia, Cipto Mangunkusumo Hospital, Jakarta, Indonesia

Nodular type of malignant melanoma is the second most common type found (15–30%) following superficial type (70%). Lesions present as red blue (amelanotic) or bluish-black papules to nodes, growing rapidly in a matter of weeks to months. Clinically, nodular type of malignant melanoma can mimic other diseases. Early detection with dermoscope greatly improves the prognosis. In advanced cases with metastases stated as poor prognosis with a low number of 5 yrs survival rate.

A 57 yrs old woman come with a lump of pain in the right lower limb since 2 yrs ago. Originally arise in the feet, black, easy to wound and bleed and spread proximally in the form of skin-colored bumps, multiple. Previous trauma is undeniable, but often in contact with the ground barefoot. History of weight loss is denied. Physical examination showed a good medical status, vital signs and other general status within normal limits. There are multiple, skin-colored to hyperpigmented nodes, linear, discrete, some with erosion and pus, on the plantar region and right lower limb. There are skin-colored to hyperpigmented plaques, verrucous, on the third middle of the right lower limb. There are multiple palpable bilateral inguinal lymphadenopathy. Biopsy and immunohistochemistry using HMB45 and S100 figured a nodular type malignant melanoma. Results of X-rays showed metastases in the lungs.

Malignant melanoma cases rarely show a pattern of chronic disease. In this case the slow course of the disease is found with a good medical status, so that at first there are sporotrichosis, chromomycosis, atypical *Mycobacterium* infections and metastatic malignant melanoma as differential diagnosis. Advanced stage of malignant melanoma found in patients supposed to have a poor prognosis, but it was not found in patients. Other patients with slow disease course and good medical status has also been reported, with a primary lesion in the buccal mucosa.

Keywordsmalignant melanoma, nodular type, metastasis, chronic, good medical status.

P080

The safety and efficacy of ipilimumab in patients with advanced melanoma in a Taiwanese cohort of the ipilimumab expanded access program

W.-K. Huang[1], C.-E. Wu[1], C.-H. Hsieh[1], J.-H. Liu[2], C.-C. Lin[3], C.-Y. Lin[4], T.-C. Liu[5], W.-C. Su[6], Y.-M. Yeh[6], J.-J. Hsieh[1], J. W.-C. Chang[1]

[1]Division of Hematology-Oncology, Chang Gung Memorial Hospital, Taoyuan; [2]Division of Hematology-Oncology, Taipei Veterans General Hospital; [3]Division of Hematology-Oncology, National Taiwan University Hospital, Taipei; [4]Division of Hematology-Oncology, China Medical University Hospital, Taichung; [5]Division of Hematology-Oncology, Kaohsiung Medical University Chung-Ho Memorial Hospital, Kaohsiung; [6]Division of Hematology-Oncology, National Cheng Kung University Hospital, Tainan, Taiwan ROC

The prognosis of patients with metastatic melanoma in Taiwan is poor. Biochemotherapy with low-dose interleukin-2 plus carmustine, cisplatin, dacarbazine and tamoxifen has demonstrated a 5-year survival rate of 12.5% in patients with metastatic melanoma. Ipilimumab has been approved for the treatment of advanced melanoma in Western Countries, however the efficacy and safety is not fully understood in Asian populations. We have conducted an expanded access program in Taiwan since September 2013.

Patients with advanced melanoma who failed at least one systemic treatment were eligible. The patients were treated with ipilimumab 3 mg/kg every 3 weeks for a total of four doses. Re-induction with the same dose scheduled at the time of progression was allowed for the patients with a response or durable stable disease to prior induction treatment. Dose reduction or modification was not allowed. Skipped doses due to severe immune-related adverse events (irAE) can be delayed up to 6 weeks. The response according to immune-related response evaluation was assessed at 12 weeks and every 3 months after the treatment course. Safety was assessed at every visit according to irAE and CTCAE [MH1] criteria.

Among the 34 screened patients from 6 hospitals, 18 were enrolled. Fourteen patients (78%) were male, and the median age was 59.5 (range: 29–82) years. The most common metastatic sites were regional lymph nodes (56%) and the lung (44%). LDH [MH2] level was elevated in 10 (56%) patients. Half of the enrolled patients received two or more prior systemic treatments. A performance status of 0 and 1 were noted in 17% and 67% of the patients, respectively. Of 8 evaluable patients, one patient (12.5%) achieved a partial response, four patients (50%) had stable disease, and three patients (37.5%) had disease progression. Regarding irAE, three patients (17%) had grade 1 diarrhea and six patients (33%) had grade 1 and 2 skin rashes. One patient (6%) had grade 1 hepatic toxicity. Due to the limited follow-up duration, the median progression-free and overall survival were not reached.

The preliminary results of this ipilimumab expanded access program study in Taiwan demonstrated encouraging efficacy with limited toxicity in a Taiwanese population with refractory metastatic melanoma. Although melanoma is rare in Asia, immune therapy and targeted drugs are urgently needed in this area.

P081

Anti-CSPG4-specific antibodies enhance the effects of the BRAF inhibitor PLX4032 on melanoma cells

S. Kontsekova[1], D. Pucciarelli[1], N. L. Lengger[1], C. Hafner[1,2], H. Breiteneder[1]

[1]Depatment of Pathophysiology and Allergy Research, Medical University of Vienna, Vienna; [2]Karl Landsteiner Institute of Dermatological Research, St.Poelten, Austria

Malignant melanoma is one of the most common forms of fatal skin cancer. Despite the progress in understanding of the biology of melanoma progression, it still remains a significant clinical problem. Advanced melanoma is often characterized by the BRAF V600 mutation which is responsible for constitutive activation in MAPK pathways and increased proliferative and migratory potential of tumor cells. Several small-molecule BRAF V600 inhibitors such as PLX4032 (vemurafenib) have proven highly effective at inhibiting the V600 mutation. However, resistant-associated secondary mutation led to reactivation of the MAPK pathway together with activation of alternative survival pathways. Therefore, single-target treatment seems to be unsuccessful in curing such a complex disease.

© 2014 John Wiley & Sons A/S. Published by John Wiley & Sons Ltd

To overcome the potential mechanisms of resistance and to increase the duration of the initial response to PLX4032 we aimed to characterize the effects of anti-225D9+-TT chondroitin sulfate proteoglycan polyclonal Abs on $BRAF^{V600E}$ mutant melanoma cell lines expressing CSPG4 ($melanoma^{BRAF(V600E)/CSPG4+}$ cells). The anti-225D9+-TT Abs are polyclonal rabbit Abs directed against the integral membrane chondroitin sulfate proteoglycan 4 (CSPG4) expressed by melanoma cells. These Abs were obtained after immunization with a mimotope vaccine.
Material and MethodsMelanoma cell lines M14 and 518A2 which both harbor $BRAF^{V600E}$ mutation, but only 518A2 cells also express CSPG4, were incubated with PLX4032 with or without anti-225D9+-TT Abs. Proliferation assays and Western blot analyses were performed.
Data demonstrated that the treatment with PLX4032 and the anti-225D9+-TT Abs together was more effective in inhibiting the growth of $melanoma^{BRAF\ (V600E)/CSPG4+}$ cells than either agent alone. This enhanced inhibition was both $BRAF^{V600E}$ mutation- and CSPG4-dependent as it was not observed in $melanoma^{BRAF\ (V600E)/CSPG4-}$ cells. While treatment of $518A2^{BRAF(V600E)/CSPG4+}$ cells with PLX4032 or anti-225D9+-TT Abs alone showed only slightly decreased levels of pAKT and pERK compared to the untreated control, treatment with combination of both agents completely blocked pAKT and pERK expression. This effect was not detected on $M14^{BRAF(V600E)+/CSPG4-}$ cells.
This study, which was supported by a Marie Curie ITN-Network for Initial Training (EngCaBra, FP7-PEOPLE-2010-ITN, GA 264417), provides a foundation for future investigations to improve BRAF inhibitor effectiveness for treating melanoma in combination with CSPG4-specific antibodies. Our findings suggest that CSPG4 can serve as a novel target for enhancing the anti-tumor effects of BRAF inhibitors.

P082

Novel copper (Li)-based photosensitisers induce phototoxicity towards melanoma cells in vitro

F.-J. Lai[1], C.-C. Hsieh[1], R.-k. Lin[2], C.-L. Cheng[3], N.-S. Chang[4], C.-C. Lin[2]

[1]Department of Dermatology, Chi Mei Medical Center, Tainan; [2]Department of Chemistry, National Chung Hsing University, Taichung; [3]National Tainan Institute of Nursing, National Cheng Kung University Medical College; [4]Institute of Molecular Medicine, National Cheng Kung University Medical College, Tainan, Taiwan ROC

Photodynamic therapy is a treatment involving light and a chemical substance (a photosensitiser), used in conjunction with molecular oxygen to elicit cell death. Photodynamic therapy is a clinically approved, minimally invasive therapeutic procedure that can exert a selective cytotoxic activity toward malignant cells. The aim of the present study is to investigate new synthesis copper (II) complexes (ghn-12, C53H39ClCuN8O5; e-22, Cu(L4)dppn(ClO4),) as potent photocytotoxic agents. Photoinduced DNA cleavage activity, cell cytotoxicity, cell cycle distribution and apoptosis assay are used to investigate the effect of copper (II)-based photosensitisers on therapeutic applications. Ghn-12 induced supercoiled pUC19 DNA cleavage from supercoiled to nicked circular form at a low-power monochromatic UV-A light of 365 nm. Flow cytometry indicated that photoinduced ghn-12 and e-22 sensitized melanoma cells (B16-F10) in the G0/G1 and S-G2/M phases with a concomitant significantly increased sub-G1 fraction, indicating cell death by apoptosis. This photoinduced apoptosis process was accompanied by activation of caspase-3 expression and FITC Annexin V staining after melanoma cells treated with ghn-12 or e-22. Our data suggest that new photosensitizing compounds, ghn-12 and e-22, exhibiting light-induced cleavage of double-stranded DNA and causing apoptosis of Melanoma cells. A variety of second generation photosensitisers have been developed and evaluated against a range of clinical applications. These novel copper (II)-based photosensitisers, ghn-12 and e-22, seem to be potential for therapeutic applications.

P083

An early innate response resulting in melanoma cancer stemness precedes acquired drug resistance

D. R. Menon[1], C. Krepler[2], A. Vultur[2], B. Rinner[3], G. Zhang[2], R. Somasundaram[2], G. Hoefler[4], M. Herlyn[2], P. H. Soyer[1], B. Gabrielli[5], H. Schaider[1]

[1]School of Medicine, The University of Queensland, Brisbane, Qld, Australia; [2]The Wistar Institute, Philadelphia, PA, USA; [3]Center for Medical Research, Medical University of Graz; [4]Institute of Pathology, Medical University of Graz, Graz, Austria; [5]The University of Queensland Diamantina Institute, The University of Queensland, Brisbane, Qld, Australia

Acquired drug resistance (ADR) in melanoma is a major challenge for effective therapy including targeted therapies. Two commonly accepted models have so far been elaborated as main contributors to the emergence of ADR. One describes a process of clonal selection of few cells which gradually acquire random mutations or epigenetic changes during drug exposure. Another model favors the existence of different preexisting small subpopulations displaying characteristics of cancer stem like cells. For both models, however, it is largely unknown how ADR is ultimately evolving. Our study delivers evidence for the induction of stemness as an early primary survival mechanism of melanoma cells independent of a certain subpopulation when exposed to persistent drug treatment with molecular targeted inhibitors like BRAF or MEK inhibitors. The early transition is characterized by gain of a multi-drug tolerant state along with increased expression of the mesenchymal stem cell marker CD271 (p75NTR) and elevated ALDH activity leading to highly angiogenic and tumorigenic cells. The cells also exhibit activation of multiple RTKs resulting in persistent AKT-MAPK signaling activation enabling the cells to undergo continuous signaling maneuver against many therapeutic interventions. Upon discontinuation of the drug a reversal of the multi-drug tolerant state to a parent like drug sensitive state is accomplished. We propose a model of combining a primary drug with distinct secondary drugs like CD271, HDAC or PI3-K inhibitors targeting the transition from a parent state to a stem like state before the acquisition of ADR. Employing these combinations together with drug holidays may prevent or delay ADR.

 © 2014 John Wiley & Sons A/S. Published by John Wiley & Sons Ltd

P084

Sentinel lymph node biopsy might improve survival outcomes patients with clinically node-negative cutaneous melanoma in Taiwan

C.-E. Wu[1], C.-H. Hsieh[1], W.-K. Huang[1], C.-J. Chang[2], J.-T. Yeh[3], T.-T. Kuo[4], Y.-F. Lo[5], C.-H. Yang[6], K.-J. Lin[7], J.-J. Hsieh[1], H.-Y. Cheng[1], J. W.-C. Chang[1]

[1]Division of Hematology-Oncology, Chang Gung Memorial Hospital; [2]Department of Plastic Surgery, Chang Gung Memorial Hospital; [3]Department of Plastic and Reconstructive Surgery, Chang Gung Memorial Hospital; [4]Department of Pathology, Chang Gung Memorial Hospital; [5]Department of General Surgery, Chang Gung Memorial Hospital; [6]Department of Dermatology, Chang Gung Memorial Hospital; [7]Department of Medical Imaging and Radiological Sciences, Chang Gung Memorial Hospital, Taoyuan, Taiwan ROC

Sentinel lymph node biopsy (SLNB) is the standard management for clinically node-negative cutaneous melanoma patients. The aim of this study was to evaluate the role of SLNB in melanoma patients, and in particular Taiwanese patients with acral lentiginous melanoma (ALM).

A total of 465 cutaneous melanoma patients underwent surgery from January 2000 to December 2011 at Linkou Chang Gung Memorial Hospital. We retrospectively analyzed the clinicopathological characteristics and survival outcomes of these patients who underwent primary surgery followed by either SLNB or observation.

Among the 465 patients, 127 underwent SLNB and 51 were observed only after primary surgery. There were no significant differences in clinicopathological features between the two groups except that patients who underwent SLNB were older and had a higher rate of ALM than those under observation. The median follow-up time was 43.5 months (to July 2013). The patients who underwent SLNB had significantly better disease-free survival (DFS) (57.1 versus 18.7 months, $P = 0.01$) and overall survival (OS) (112.4 versus 45.2 months, $P < 0.01$) than those under observation. Improved DFS (hazard ratio [HR]: 0.508, $P < 0.01$) and OS (HR: 0.602, $P = 0.03$) were observed even after adjusting for age and disease pathology. Among the patients with ALM, those who underwent SLNB ($n = 90$) had a significantly better DFS (62.6 versus 19.3 months, $P < 0.01$) and a favorable trend of OS (not reached versus 45.2 months, $P = 0.05$) compared to those under observation ($n = 24$). However, among the patients without ALM, DFS (16.7 versus 16 months, $P = 0.381$) and OS (51.4 versus 41.3 months, $P = 0.114$) did not differ between the patients who underwent SLNB ($n = 37$) and those under observation ($n = 27$).

SLNB might improve outcomes in patients with clinically node-negative cutaneous melanoma, particularly in Taiwanese patients with ALM.

P085

Monitoring changes in circulating tumour cells as a prognostic indicator of overall survival and treatment response in patients with metastatic melanoma

M. Ziman[1], M. Millward[2], D. Klinac[1], J. Freeman[1], A. Reid[1], S. Bowyer[3], E. Gray[1]

[1]School of Medical Sciences, Edith Cowan University; [2]Department of Medicine, University of Western Australia; [3]Department of Medical Oncology, Sir Charles Gairdner Hospital, Perth, WA, Australia

New effective treatments for metastatic melanoma greatly improve survival in a proportion of patients. However biomarkers to identify patients that are more likely to benefit from a particular treatment are needed. We previously reported on a multimarker approach for the detection of heterogenous melanoma circulating tumour cells (CTCs). Here we evaluated the prognostic value of this multimarker quantification of CTCs and investigated whether changes in CTC levels during therapy can be used as a biomarker of treatment response and survival outcomes.

CTCs were captured by targeting the melanoma associated markers MCSP and MCAM as well as the melanoma stem cell markers ABCB5 and CD271. CTCs were quantified in 27 metastatic melanoma patients treated by surgery or with vemurafenib, ipilimumab or dacarbazine. Patients were enrolled prospectively and CTC counts performed at baseline (prior to treatment), during and after treatment.

Baseline CTC numbers were not found to be prognostic of overall survival nor of progression free survival. However, a low baseline CTC number was associated with a rapid response to vemurafenib therapy. A decrease in CTCs after treatment initiation was associated with response to treatment and prolonged overall survival particularly in vemurafenib treated patients.

Measuring changes in CTC numbers during treatment is useful for monitoring therapy response in melanoma patients and for providing prognostic information relating to overall survival. Further studies with larger sample sizes are required to confirm the utility of CTC quantification as a companion diagnostic for metastatic melanoma treatment.

Hyperpigmentary Disorders

P086

Pigmentary mosaicism (checkerboard) of a Beckers naevus

U. Alagappan[1], B. K. Goh[1]

[1]Dermatology, National Skin Centre, Singapore City, Singapore

Becker's nevus (or Becker's melanosis) is an epidermal melanosis, considered to be a cutaneous hamartoma, It is characterized by an asymptomatic, irregular, well defined, discrete or confluent macules usually with hypertrichosis on the shoulder, anterior chest and scapular region. It is five times more frequent in males, and usually involves the upper trunk. Although it is a common finding, affecting ~0.5% of young men, multiple lesions are rare. Here, we report a rare form of the disorder, manifesting as multiple lesions with a checkerboard pattern of cutaneous mosaicism.

© 2014 John Wiley & Sons A/S. Published by John Wiley & Sons Ltd

25- year- old female presented with an asymptomatic hyperpigmented hairy patches on the abdomen and the left thigh for more than 15 yrs. The patient had noticed an increase in hair growth and darkening of colour in these patches, over the last 10 yrs. On clinical examination, there was a well-defined, dark brown patch on the left abdomen with a midline demarcation and hypertrichosis. The other hyperpigmented patch on the left thigh had increased thick, coarse, black hairs. There was no underlying swelling or mass palpable. There was no asymmetry of the thighs or the abdomen. Physical examination did not reveal any neurologic or musculoskeletal defect. A clinical diagnosis of Becker's nevus in a checkerboard pattern was made made.
S.W. Becker first described Becker's nevus in 1949 as ''concurrent melanosis and hypertrichosis in the distribution of nevus unius lateris'. It is more frequent in men and develops in the peripubertal period. The finding of multiple Becker's nevi in one patient is extremely rare, and an extensive literature search revealed less than 15 patients with multiple lesions. Especially interesting is the distribution pattern of the lesions in our patient, which revealed a checkerboard mosaic pattern.
Collectively, together with reports on Becker's nevi in a Blaschko line configuration, our case strengthens the notion that Becker's nevus can follow one of the mosaic patterns, and may prompt detailed physical examination in patients presenting with a single lesion.

P087

Amlodipine-induced acquired hyperpigmentation: a case report

H. M. Cheng[1], S. P. Kumarasinghe[1,2,3]
[1]Dermatology, Royal Perth Hospital; [2]University of Western Australia; [3]Western Diagnostics, Perth, WA, Australia

Slowly progressive acquired pigmentation can be due to a varied aetiology including hormonal, drug induced and metabolic causes.
A 53 yrs old woman of Sri Lankan descent presented with slowly progressive pigmentation for 2 yr. The pigmentation was subtle and was more pronounced on neck and limbs. There was no preceding pruritus or inflammation. She had been on amlodipine for 8 yrs for hypertension. She was not any other relevant medications on a regular basis. There were no known drug allergies. There was mild hyperpigmentation on her neck, posterior trunk and the limbs with indistinct borders. There were no round patches of pigmentation. There was no pigmentation of the mouth, palms, soles or the nails. There was no acanthosis nigricans, lichen planus or eczema. The skin biopsy taken from the left arm and right leg showed a normal epidermis with moderate pigmentary incontinence in the superficial dermis. Melanization of epidermal keratinocytes was prominent but pigmentation was evenly distributed. There was no interphase dermatitis. Adnexae appeared unremarkable. Blood tests did not reveal any other causes of hyperpigmentation. The possibility of amlodipine induced hyperpigmentation was entertained with a differential diagnosis of ashy dermatosis. Amlodipine 10 mg once daily was changed to irbesartan 300 mg once daily. Examination after 6 months of stopping amlodipine showed that the hyperpigmentation has faded significantly.Only one other case of amlodipine induced hyperpigmentation has been reported so far in the medical literature. Exact mechanism of hyperpigmentation is not known. We believe that amlodipine induced hyperpigmentation may go unrecognised as it is subtle and very slowly progressive.

P088

Ashy dermatosis (erythema dyschromicum perstans) induced by omeprazole: a report of 3 cases

S. Chua[1], M. M. F. Chan[2], H. Y. Lee[1,3,4]
[1]Duke NUS Graduate Medical School; [2]Department of Pathology, National University of Singapore; [3]Dermatology Unit, Singapore General Hospital, National University of Singapore; [4]National University of Singapore, Singapore City, Singapore

Ashy dermatosis is a disorder of pigmentation, characterized by asymptomatic symmetric ashy gray-colored macules. It is noted to have a higher prevalence in the pediatric population. It has been reported to be induced by ethambutol treatment, pesticide exposure (Chlorothalonil),cobalt allergy, ammonium nitrate consumption, Herbal consumption(i.e. Tokishakuyakusa), fluoxetine, HIV serology conversion and intestinal parasite infection. There has only been one case of ashy dermatosis induced by Omeprazole.
To describe the clinical and histological features of three adult patients with ashy dermatosis induced by Omeprazole.
A retrospective study of three patients with ashy dermatosis induced by Omeprazole attended our adult hospital between 2012 and 2014 was performed. The clinical and histopathological features were reviewed.
Two males and one female between the ages of 47–57 were included in this study. The patients did not have any significant medical or dermatological history. The patients had omeprazole started for gastritis for duration of 9–12 months. No other new medications were started in the same period.
The patients had hyperpigmented grey patches that began initially on the trunk before extending to the neck and extremities. The lesions were completely asymptomatic and had been stable for several months. The palms, soles, or mucous membranes were not involved. Biopsy showed pigmentary incontinence and moderate numbers of melanophages. A clinical diagnosis of ashy dermatosis induced by omeprazole was provided. Omeprazole was stopped with no further progression of the dermatoses.
We describe a series of patients with features that were suggestive of Ashy dermatosis. Omeprazole was deemed a likely trigger based on the temporal relationship and lack of progression on drug cessation. Recognition of this association is important due to the widespread use of omeprazole.

P089

Acquired dermal melanocytosis of the nose - five unusual cases

S. Y. Chuah[1], H. H. Ghorbel[2], J. P. Lacour[3], T. Passeron[4]
[1]Dermatology, National Skin Centre, Singapore City, Singapore; [2]Department of Dermatology, Habib Thameur Hospital, Montfleury, Tunis, Tunisia; [3]Department of Dermatology, University Hospital of Nice; [4]Department of Dermatology and INSERM U1065 Team 12, C3M University Hospital of Nice, Nice, France

Acquired dermal melanocytosis (ADM) usually appears in Asian women during adult life. It is uncommon amongst the Caucasian but not rare. The condition may presents symmetrically as blue-brown or slate-grey macules known as acquired bilateral nevus of Ota-like macules (ABNOM), also named Hori's nevus. Localized and asymmetrical ADM have been also reported on the face or other part of the body. Histopathological examination reveals

© 2014 John Wiley & Sons A/S. Published by John Wiley & Sons Ltd

irregularly-shaped, bipolar melanocytes in the upper and middle dermis without disturbance of the normal skin architecture.

We report five cases of ADM with an unusual and misleading presentation. All the lesions were acquired and appeared at the mean age of 34 (21–57). They were present for a mean of 6 yrs (3–14) before the diagnosis. They presented as blue-brown macules located initially only on the nose before involving other areas on the face for two out of the five patients. Interestingly, three out of these five cases presented with blue-brown macules that remained localized only on the nose (nasal alae and nasal root) with up to 14 yrs of evolution. This misleading presentation of an acquired pigmentation located on the nose has led to a delayed diagnosis in all the patients. No history of contactants, prior inflammation, trauma or significant sun exposure was found. Skin biopsies were performed in two of the three lesions localized only on the nose and confirmed an ADM. Only one of those patients asked for a treatment. The lesion improved for more than 75% after 2 sessions of 755 nm Q-switched laser. No relapse was observed after 6 months follow-up.

When ADM is presented as a widespread hyperpigmention of the face, it should be differentiated from bilateral nevus of Ota, Riehl's melanosis, melasma and post-inflammatory hyperpigmentation (PIHP). Unlike naevus of Ota, the pigmented lesions are not observed in the conjunctiva or mucous membranes of the mouth or nose. Naevus of Ota usually present at birth or develops within 1 yr of life or in adolescence. Riehl's melanosis usually involves the cheeks and is not located only on the nose. Mild erythema, edema, and pruritus is often reported. Melasma has irregular border, spares the nose, and worsens during the summer period. PIHP is probably the main differential diagnosis of ADM of the nose. History of contactants, prior inflammation, trauma or repeated frictions is usually found. Moreover, the lesions progressively fade with time, and do not remain stable for years or slowly spread as it is the case for ADM. Histologically, only dermal melanin and melanophages but no dermal melanocytes are found in the lesions of Riehl's melanosis, melasma and post-inflammatory hyperpigmentation. Finally, if ADM presented only localized to the nose, the main differential diagnosis to exclude would be melanoma. In this case, the lesion is not symmetrical, with irregular borders and pigmentation. Dermoscopy and histological examination should be performed if there is any doubt.

To our knowledge, localized ADM of the nose has not been published yet in the literature. We hope that reporting this unique clinical phenotype of ADM will be of educational value to increase awareness of such presentation, and offer possible options of management for the patients.

P090

A case of hyperpigmentation along blaschkoid lines: case report with a brief review of literature

K. Debbarman[1], S. sethi[1], V. K. Garg[1]

[1]Dermatology &VD, Maulana Azad Medical College, New Delhi, India

The lines of Blaschko represent a pattern followed by many cutaneous disorders. Various inherited and acquired disorders with hyperpigmentation have been described along these lines, they include 'Linear and whorled nevoid hypermelanosis' (LWNH), 'Progressive cribriform and zosteriform hyperpigmentation' (PCZH), 'Zebra-like hyperpigmentation in whorls and streaks' and 'Reticulate hyperpigmentation a zosteriform pattern'. Presenting an adult onset case of hypermelanosis along the blaschkoid lines with involvement of palm and no systemic abnormalities.

CaseA 18 yrs old male presented with uniformly pigmented dark brown macules of in a linear distribution along the lines of Blaschko extending from the xiphi-sternum to palmar aspect of the right thumb in an inverted U pattern, forming an arc on upper chest and a V pattern on back. These macules were 0.1–1 cm in size with irregular and well defined margins. There is no other abnormality. Histopathology, revealed increased melanization of the basal layer in the absence of dermal melanophages and pigment incontinence.

Pigmentation in this case is a linear without formation of whorls or streaks and extended to involve the palmar surface of right hand, which to the best of our knowledge has never been described in a case of PCZH or LWNH. The eruption was bilateral, asymmetric and differed from the diffuse widespread involvement of LWNH and localized unilateral zosteriform involvement in PCZH. The lesions are not distributed along dermatomes. It manifested as a purely cutaneous eruption without any systemic involvement. Our case of *Blaschkoid hypermelanosis* –is a borderline manifestation between the clinical spectrum of PMZH and LWNH. Old terms like LWNH and PMZH which do not encompass the entire continuum of clinical manifestations should be discarded and a revised nomenclature and classification of these pigmentary anomalies be adopted.

P091

Semiology of actinic lentigines: an intrinsic heterogeneity observed by dermatoscopy and image analysis

V. Piffaut[1], S. Nouveau[1], C. Duval[1], P. Bastien[1], B. Chalmond[2], F. Bernerd[1], O. de Lacharrière[1]

[1]L'Oréal Research & Innovation, Aulnay-sous-bois; [2]CMLA, ENS, Cachan, France

Although actinic lentigines (AL's) are very frequent benign hyperpigmented disorders associated with age and chronic sun exposure, their etiology remains unclear. Both clinical and precise instrumental evaluations may thus provide new insights in terms of knowledge and characterization of such lesions. Using both confocal microscopy and dermatoscopy, AL's have revealed various features, based upon different patterns related to the organization of the dermal epidermal junction (DEJ) which may be indicative of their severity. We have also shown that in a given AL, the clinical patterns are heterogeneous, comprising both round and elongated honeycomb network structures. It has been suggested that the latter corresponded to an 'advanced' stage of AL.

To better characterize an AL lesion, we developed a dedicated software for grading AL observed by dermatoscopy (images ×70 magnification, Fotofinder dermoscope®, Teachscreen, Bad Birnbach, Germany). By combining image and statistical analyses, the software, developed on Matlab® (Software SQA) defines the respective probabilities of a round or elongated pattern. The analyses used mathematical parameters such as curvature, length, scattering orientation of the pigmented network. Accordingly, analyzing more than 50 AL's from the dorsal side of hands from different clinical studies allowed us to validate the software by comparing with histological features. The results showed that the software quantification was consistent with the degree of deformation of the dermal epidermal junction observed on histological sections. The Objective and specific phenotypic criteria extracted from this

© 2014 John Wiley & Sons A/S. Published by John Wiley & Sons Ltd

software analysis more precisely define the characteristics of a given AL. Important applications for such a non invasive method are numerous, from predicting the stage of the lesion, selecting an homogeneous set of lesions to be included in a research clinical study, up to the choice of the most appropriate treatment.

P092

Palmar pigmentation: a study of 3 cases with clinical variation

K. Karunaratne[1], M. Dissanayake[1], J. Liyanage[1]
[1]Teaching Hospital, Kandy, Sri Lanka

Palmar pigmentation is not a common presentation to the dermatology clinic, but may represent potentially serious diagnoses. Causes of palmar pigmentation include Addison disease, hyperthyroidism, vitamin B12 deficiency, drugs including bleomycin, zidovudine and cyclophosphamide. Rarer causes include dyskeratosis congenita, Acropigmentation of Dohi, and Fanconi anaemia.
Different morphological patterns of palmar pigmentation have been described. These include diffuse palmar pigmentation, pigmentation of creases, mottled hypo and hyperpigmentation and reticulate pigmentation. The morphological pattern helps in clinically diagnosing the aetiology.
Case 1A 67 yrs old female who had presented with faintishness was referred to the skin clinic due to hyperpigmentation of buccal mucosa and palms. She also had numbness of feet for 2 yrs and was under investigation for anaemia. She had poor intake of fish and meat in her diet.
On examination she had diffuse pigmentation of palms especially inbetween skin creases, with sparing of the creases. She was also found to have pallor, peripheral neuropathy and bilateral optic atrophy. Investigations showed pancytopaenia with evidence of early marrow fibrosis seen in the blood picture. Endoscopy and gastric biopsy showed gastric atrophy.
The patient was diagnosed with vitamin B12 deficiency and treated with intramuscular vitamin B12. Skin pigmentation showed gradual reduction with treatment.
Case 2An 8 yrs old boy, the product of a consanguineous marriage, presented with a 4 yrs history of altered pigmentation of palms, soles and axillae. No other family members were affected.
Examination showed diffuse pigmentation of the palms with superimposed guttate macular hypopigmentation. The changes did not extend beyond the wrist and the dorsum of the hands were normal. There was no nail dystrophy. Similar changes were seen on the soles and axillae. A large café-au-lait macule with geographic margins and midline demarcation was found on the back. The patient was clinically pale, otherwise the oral cavity was normal. There were no dysmorphic features or structural abnormalities. There was no history of easy bruising or frequent infections.
Investigation found low platelets and low white cell count. Bone marrow biopsy showed a hypoplastic marrow. Due to a change of residence the patient was lost to follow up during investigation.
Case 3A 58 yrs old female presented complaining of generalized pigmentation, mainly affecting face and arms. She also complained of malaise with on and off dizziness.
On examination there was generalized pigmentation with accentuation over sun-exposed areas and flexures. The oral cavity was pigmented. On the palms there was pigmentation limited to the creases. She was also pale and found to have a postural drop in blood pressure.
Preliminary investigations showed low serum sodium, normal serum potassium and low random blood sugar. The Short Synacthen test was positive. The patient was referred to the endocrinology unit for further evaluation.
Palmar pigmentation can be classified according to the clinical pattern. Unilateral pigmentation is most likely due to localized causes such as tinea nigra. Bilateral pigmentation most likely represents an underlying systemic cause such as endocrine or nutritional disease or drug therapy. The presence of a combination of hypo and hyperpigmentation (dyschromatosis) indicates rarer diagnoses such as dyskeratosis congenita, Fanconi anaemia and Acropigmentation of Dohi.

P093

Treatment of café au lait macules using 1064 nm Q-Switched Nd:YAG laser: what is the golden parameter?

J. E. Kim[1], E. J. Kim[1], H. Hur[2], J. Y. Ko[1], Y. S. Ro[1]
[1]Department of Dermatology, Hanyang University Hospital, Seoul; [2]Chois Skin Clinic, Gyeonggi-do, Republic of Korea

Café au lait macules (CALMs) are common, benign epidermal pigmented lesions, which can be solitary or associated with neurocutaneous syndromes. Since the concept of subcellular photothermolysis has been introduced and used in the treatment of various pigmentary diseases, there have been anecdotal cases on the treatment of CALMs with low energy 1064 nm Q-switched (QS) Nd:YAG laser. However, there has been lack of systematic study and established parameter of the treatment method. ObjectivesTo evaluate the efficacy and safety of 106 nm QS Nd:YAG laser treatment and find the golden parameter in the treatment of solitary CALM in Asian patients.
A 32 patients with solitary CALMs were enrolled in this study. Patients received 1 pass of 1064 nm QS Nd:YAG laser treatment (7 mm spot size, 2.4J/cm^2, 10 Hz) every week. Lesions on face were treated up to 30 sessions and off-face lesions were treated up to 50 sessions. Clinical responses were assessed by the blinded dermatologists 3 months after the treatment and follow-up period were up to 24 months.
All patients responded to the laser treatment and showed excellent cosmetic results. The treatment was well tolerated, with no post-treatment bleeding or crust formation, and the postinflammatory hyperpigmentation. Recurrence was not noted in all subjects during follow up periods.
Our study showed high efficacy and safety in removing CALMs with 1064 nm QS Nd:YAG laser and we propose the parameter we used as the 'golden parameter' in the treatment of solitary CALMs.

P094

Hypohidrotic ectodermal dysplasia with acquired dermal melanosis

E. G. Lee[1], H. J. Lee[1], D. H. Kim[1], M. S. Yoon[1]
[1]Department of Dermatology, CHA Bundang Medical Center, CHA University, Seongnam, Republic of Korea

Ectodermal dysplasia is a rare inherited disorder which is characterized by defects of more than two organs originated from the ectoderm, and usually involves hair, teeth, nails and sweat glands. Ectodermal dysplasia is categorized into two subsets, hypohidrotic and hidrotic, and hypohidrotic ectodermal dysplasia shows hypohidrosis/anhidrosis, anodontia, hypotrichia

 © 2014 John Wiley & Sons A/S. Published by John Wiley & Sons Ltd

and characteristic facial morphology. In addition to the typical characteristics, patients often develop periocular hyperpigmentation, but usually in ring shapes or limited to lower eyelids. Diagnosis can be easily made from the clinical manifestation.
A 26-year-old man presented with bluish patch on the glabella and both cheeks in the shape of letter x which persisted more than 1 yr. The patient denied any trauma history except fot tattooing both eyebrows and extremities. He was bold because he shaved all his hair to mask hypotrichia. He suffered from anhidrosis since infancy and had marked heat intolerance. There was periorbital hyperpigmentation over the dry and wrinkled skin. Oligodontia was masked by the implants but he had only 7 real teeth of his own. Lips were thick and everted. No abnormal findings were observed upon the examining the nails, eyes, and ears, and he has normal intelligence. His grandfather had similar cutaneous features. A clinical diagnosis of hypohidrotic ectodermal dysplasia was made. A punch biopsy of the bluish patch on the left cheek revealed incontinence of melanin in the upper dermis and hypoplasia of skin appendages. Whether this acquired dermal melanosis is related to ectodermal dysplasia or tattooing history on other body sites is not clear. Laser treatment for the facial pigmentation is ongoing.
We herein report a case of atypical-appearing facial dermal melanosis in a patient diagnosed with hypohidrotic ectodermal dysplasia.

P095

Hyperpigmentation with systemic symptoms in an Asian female

C. C. Oh[1], W. Y. Tham[1], H. Y. Koh[1]
[1]Dermatology, Singapore General Hospital, Singapore city, Singapore

We present a case of an asian middle age Chinese female who presented with sudden onset of hyperpigmented macules on her oral mucosal surfaces, palms and soles, in association with hypoguesia and loss of weight. This was found to be associated with underlying severe vitamin B12 deficiency secondary to pernicious anaemia on subsequent workup. She was started on intramuscular cyanocobalamin injections at three times a week for one week, followed by once a week for a month. She would subsequently receive lifelong monthly cyanocobalamin injections. On review 2 months later, she had regained her taste sensation, gained weight, with pigmentation fading off. In a patient who presents with hyperpigmentation, the clinical approach would be to conduct a relevant history and examination to determine the underlying cause of the hyperpigmentation. The history should focus on family history to ascertain if the condition may be inherited, drug history, and systemic review of symptoms to look for secondary causes of hyperpigmentation. Physical examination should include the distribution of the hyperpigmented lesions, blood pressure measurement, and signs of thyroid disease. B12 deficiency is an important but often forgotten cause of hyperpigmentation. The exact pathophysiology of hyperpigmentation in vitamin B12 deficiency is not well understood at present. The diagnostic test for vitamin B12 deficiency is simple, and treatment is readily available. We will also discuss the various causes of adult onset hyperpigmentation, and suggest an algorithm.

P096

The evaluation of various therapies for congenital and aquired skin hypopigmentary disorders

T. Shibata[1], M. Ichihashi[2]
[1]Shibata Clinic of Dermatology, Osaka; [2]Saisei Mirai Clinic Kobe, Kobe, Japan

I examined about 7000 patients of vitiligo vulgaris every year in our clinic. This time I'll present results of various therapyies for about 1000 patients of vitiligo vulgaris, pigmentary mosaicism, nevus depigmentosus and acuired skin hypopigmentation induced by Rhododenol. They visited my clinic in last 7 yrs. And I'll present methods, risks and effects of narrow-band UVB (DERMARAY-400), excimer lamp (VTRAC & 308 excimer system) therapies. Futhermore I'll analyze the effect of various ointments, for example vitaminD3, tretinointocoferil, tacrolimus, prostaglandin and platinum-palladium colloids mixture (paplal). The number of valid patients was 449. We found 91.5% patients (411/449) who were treated in my clinic could get repigmentation. And 13.6% patients (61/449) showed over 50% remarkable repigmentation. The effectiveness depends upon not age but site (ex. face) and period from onset. So I propose the 4 conditions of better prognosis for skin hypopigmentary disorders before therapies. Face (FA),child(C), early phase(E) and smaller lesions(S)=(FACES) are the 4 key-words.
Additionary I'll report a case of hyperpigmentary mosaicism. This patient congenitally has philloid type hyperpigmentary macules only on her right leg. According to her growth, some of these macules spontaneously disappeared and others slowly changed into hypopigmented macules. Recently, these macules present hypertrichosis and dyskeratosis with surrounding hyperpigmentation and follicular pigmentation without using a steroid ointment. This rare case cannot be described by the stereotype concept of pigmentary mosaicism. Therapies for this case are irradiation of excimer lamp (VTRAC), after applying activated vitamin D3 ointment and prostaglandin ointment and several times suction blister epidermal grafting therapy (SBT) was carried out. She gets good repigmentation of lesions.
Furthermore I present the cases of aquired hypopigmentation induced by Rhododenol including cosmetics and consider the course of this deseas. And two new therapies are carried out and successful in treatment of refractory vitiligo for example on back of hands. One is Papural (platina and palladium nano colloids mixture) cream. This therapy is based on the removal of peroxides in vitiligo skin. 18/30(60%) patients are getting new repigmentation. Other one is extreme infrared radiation therapy on refractory vitiligo on back of hands. This therapy is based on the activation of macrophages in lesional skin and were started only one month ago. After 3–4times irradiation,4/17(24%) patients are getting new repigmentation.
Images:

P097

Galli-galli disease associated with a frameshift mutation in keratin 5 gene

A. W. Tan[1], J. Common[2], B. Lane[2], J. S. Lee[1], B. K. Goh[1]
[1]Dermatology, National Skin Centre; [2]Institute of Medical Biology, Singapore City, Singapore

Keratin mutations underlie a diverse group of hereditary disorders, affecting skin, hair, nails and mucosa. In addition to mechanical skin fragility and abnormal keratinisation, these mutations may also affect cutaneous pigmentation.

© 2014 John Wiley & Sons A/S. Published by John Wiley & Sons Ltd

We present a 32-year-old Chinese man with Galli-Galli disease, associated with a frameshift mutation in exon 1 of keratin 5 (KRT5). This man presented with asymptomatic reticulate hyperpigmentation over the face and neck, and forearms papules since his teens. His mother also reported similar pigmentation over the neck.

Histological examination was consistent with Galli-Galli disease. The stratum corneum showed mild hyperkeratosis with focal parakeratosis in a follicular plug. The epidermis showed suprabasal acantholysis, and characteristic downward delving, filiform epidermal projections interconnected by thin epithelial strands. Patchy melanin pigmentation was present in the basal layer, with melanophages seen in the papillary dermis. Immunohistochemistry revealed normal expression pattern of keratin markers, indicating normal epidermal differentiation. Electron microscopy confirmed that the keratin intermediate filament network was intact, with no obvious differences in melanocytes or keratinocytes. Genetic analysis revealed a 2 base-pair deletion mutation (c.442delAG), which resulted in a premature termination codon in the V1 domain of keratin 5 (p.S148fsX30). This was previously identified in a Spanish family and this study confirms the disease association with this mutation.

In Conclusion, Galli-Galli disease is a rare genodermatosis, with a clinical presentation which can be similar to Dowling-Degos disease, but the presence of acantholysis on histopathology is thought to distinguish the two disorders. KRT5 mutations underlie both disorders.

P098

The role of BRD4 in melanogenesis

A. Trivedi[1], I. de la Serna[1]

[1]Biochemistry and Cancer Biology, The University of Toledo, Toledo, OH, USA

Hyperpigmentation is a common condition in which patches of skin become darker in color than the surrounding skin. Melanocytes are cells located in the basal layer of the epidermis that produce the pigment melanin. Disruption of normal melanocyte function can lead to skin cancer and other skin diseases that affect the level of pigmentation. Bromodomain and extraterminal domain (BET) proteins are epigenome readers that play a key role in gene expression by acting at the interface between chromatin remodeling and transcriptional regulation. BRD4, a BET protein, is a transcriptional coactivator implicated in cellular differentiation and cancer development. However, the role of BRD4 in melanocyte differentiation has not been investigated. Immortalized murine melanoblasts (melb-a cells) can be differentiated in vitro by treatment with alpha-MSH. Alpha-MSH activates the Microphthalmia-Associated Transcription Factor (MITF), the master regulator of melanocyte differentiation and melanoma oncogene. MITF in turn activates genes required for melanin production, proliferation, and survival. Treating melb-a cells with the BET inhibitor, JQ1, compromised MITF target gene expression, melanin synthesis, and reduced proliferation of melb-a cells as well as normal human melanocytes and melanoma cells. Furthermore, we determined that BRD4 occupied the promoters of MITF target genes. Treatment with JQ1 inhibited the binding of both BRD4 and MITF to these promoters. The effects on gene expression were recapitulated by siRNA mediated silencing of BRD4. Thus, we hypothesize that BRD4 plays a crucial role in melanogenesis by interacting with MITF and can be a potential novel target for treatment of hyperpigmentation disorders.

P099

Assessment of the efficacy and tolerance of a new combination of retinoids and depigmenting agents in the treatment of melasma

M. T. Truchuelo[1], N. Jiménez[1], M. Vitale[2]

[1]Department of Dermatology, University Hospital Ramón y Cajal; [2]Medical Department, IFC, Madrid, Spain

Melasma is a common hyperpigmentation disorder which mainly affects women with a high phototype and often remains active for several years, being associated with a considerable psychological impact. The exact aetiology is unknown but several factors have been suggested to promote it, such as genetics, ultraviolet and visible radiation, and hormone alterations. The management of melasma is often challenging, because it is very resistant to treatments and requires long-term therapeutic protocols. Conventional treatment includes depigmenting products, such as hydroquinone (HQ), retinoids, mequinol, azelaic acid, arbutin, kojic acid, etc., which exert their action at different stages of melanogenesis. In addition, avoidance of aggravating factors is strongly indicated.

Combination therapy is usually prescribed, with a typical association of HQ and retinoic acid. However, HQ's safety has been questioned in certain cases, and prolonged use may lead to adverse effects such as depigmentation and ochronosis, so it cannot be considered as a golden standard.

In the search for safer alternatives, a new product based on RetinSphere® technology has been designed. It incorporates two topical retinoids: retinol glycospheres and hydroxypinacolone retinoate, which directly interacts with the retinoic acid receptor but does not induce irritation. Additionally, the product includes other depigmenting active ingredients, such as N-acetylglucosamine, kojic acid and niacinamide, among others, and photoprotective ingredients. The Objective has been creating a product which may reduce or even substitute the use of HQ in the management of melasma patients.

To determine the efficacy and safety of a new combination of retinoids in the improvement of melasma.

Patients/Methods Prospective, double-blind, vehicle-controlled and randomized study in 30 adult patients with melasma, and without any concomitant diseases or treatments. The product was applied twice daily during 3 months on one side of the face, and vehicle on the other. The observation period was 3 months, organized into three visits at baseline (T0), one and a half months (T1) and three months (T2). Standardized photographs were taken using Reveal® System with RBX technology in each visit. The main variable to determine the efficacy was the improvement of the hemifacial Melasma Area Severity Index (MASI).

Twenty eight patients (27 females and 1 male; mean age: 39 yrs) completed the study. The photographic analysis demonstrated that 89% of the patients showed some degree of improvement in the side treated with the product, compared to only 56% on the vehicle side (P = 0.005). The reduction of the MASI score at 3 months of treatment on the side treated with the product was 74% compared to 55% on the side that received vehicle (P = 0.009). No remarkable side effects were detected, in spite of a significant percentage (67%) of patients included in the study citing a history that could be compatible with sensitive skin.

This new combination of retinoids and depigmenting agents proved to be effective in reducing melasma hyperpigmentation, while being safe and not yielding any side effects after 3 months of treatment.

 © 2014 John Wiley & Sons A/S. Published by John Wiley & Sons Ltd

P100

A purpuric variant of acute graft versus host disease

S. S. Yang[1], H. Jaffar[1], D. C. W. Aw[1]

[1]General Medicine, National University Hospital Singapore, Singapore City, Singapore

A 31-year-old Indonesian woman was recently diagnosed with acute myelomonocytic leukemia presenting with a left breast lump. Due to the aggressive nature of her disease, she failed first line induction chemotherapy and required salvage chemotherapy which was complicated by pancytopenia and severe neutropenic sepsis. Consequently, she underwent a myeloablative matched umbilical cord haematopoietic progenitor cell transplant.

3 weeks post-transplantation, she developed an intensely pruritic eruption over her palms which progressively worsened over a 48 h period.

On examination, there were multiple purpuric maculopapules and hemorrhagic vesicles on both palms and fingers ranging from a size of 1–3 mm. The morphological appearance was most reminiscent of pompholyx, and she was thus treated with diproprionate 0.05% cream twice daily.

The other differentials for this acute palmar eruption, given the clinical context also included acute graft-versus-host disease (GVHD) and leukemia cutis.

Skin biopsy demonstrated acanthosis, focal parakeratosis, exocytosis, spongiosis, scattered apoptotic keratinocytes and intra-epidermal red blood cells in the overlying epidermis.

There were also histiocytic and lymphocytic infiltrates around the superficial dermal vessels with papillary oedema and vacuolar alteration at the dermoepidermal junction, with extravasation of red blood cells, in the absence of features of vasculitis.

This was compatible with acute GVHD, of a grade 1 histologic stage, presenting as a purpuric variant, which is probably contributed by thrombocytopenia in this case.

She was started on MMF 500 mg BD then changed to prednisolone 50 mg daily at 1 mg/kg. Fortunately, her condition remained limited to her palms, less than 25% of body surface area involvement and improved over a two week period with subsequent resolution.

Consistent with the clinical features of this case, acute GVHD usually occurs 10–30 days from transplantation, and is attributed to the T-cell response when immunologically competent cells are introduced into an immune-incompetent host.

Cutaneous presentations usually affect the palms or soles first, and then develop a widespread maculopapular rash, desquamation, erythroderma and blistering should the condition become more severe. Chronic GVHD on the other hand, develops one hundred days post transplantation, and tends to present as lichenoid papules or sclerodermatous changes. This purpuric variant of acute GVHD is not commonly seen.

The approach to hemorrhagic blistering eruptions is important to clinical dermatology, where the common considerations would include infective processes such as herpes zoster, bullous impetigo, acute eczema, pompholyx, contact dermatitis, and autoimmune causes such as pemphigus vulgaris.

This case also highlights the importance of interpreting clinical features in the appropriate clinical context. Although the morphological appearance is most reminiscent of pompholyx, one must also consider malignant causes such as leukemia cutis which may herald recurrent disease in her case.

Other diagnostic considerations should include engraftment syndrome, drug eruptions due to the complex medical regimes these patients are usually on, such as palmoplantar hyperpigmentation secondary to chemotherapy such as cyclophosphamide, and the infective causes such as zoster in the otherwise immunocompromised host. This host of conditions can also have similar histopathological findings – hence the paramount importance of clinical correlation.

P101

Comparative study of trichloroacetic acid (15%) versus fractional Er:YAG(2940 nm) in the treatment of epidermal melasma

M. Abdallah[1], A. Alhefnawy[1], D. Altantawi[2]

[1]Dermatology & Venereology, Ain Shams University; [2]Dermatology, Kobry Alqobba Military Hospital, Cairo, Egypt

Trichloro-acetic acid (TCA15%) is one of the effective tools in the treatment of melasma. The efficacy of fractional Erbium:YAG (Er: YAG) laser has not been evaluated.

Patients & MethodsTCA 15% peeling (Group T, 2–3 coats) was compared to ablative fractional Er:YAG (group E, 800 mJ, 5 cm spot size, short pulse) in the treatment of 20 females (10 cases in each group) with epidermal melasma for three sessions. Patient used sunscreen and emollients, but no priming or additive bleaching agents. Results were evaluated by MASI score, photography, digital image analysis and patients' self-evaluation before treatment, 4 and 12 weeks after treatment.

For the Er:YAG group, the mean MASI score decreased from 14 ± 8.92, to 11.5 ± 7.1 at week 4 (representing 17.86%, P = 0.01) decrease to 10.6 ± 6.2 at week 12 (24.29%, P = 0.021). The average area changed from 49.35 ± 24.1 cm^2 to 43.23 ± 21.07 cm^2 at week 4 (12.41%, P = 0.024) to 44.85 ± 22.19 cm^2 at week 12 (9.12%, P = 0.014). The average darkness decreased from 2.71 ± 0.89, to 2.39 ± 0.72 at 4 weeks (11.81%, P = 0.017) and to 2.28 ± 0.77 at 12 weeks (15.87%, P = 0.011).

For the TCA group, the mean MASI score decreased from 17.8 ± 6.7 to 14.97 ± 6.6 at 4 weeks (15.9%, P = 0.04). Further decrease in MASI score was observed at week 12, 12.6 ± 5.3, (29.22%, P = 0.029). The area decreased from 65.37 ± 20.5 cm^2 to 54.97 ± 16 cm^2 (15.91%, P = 0.015) at 4 weeks to 53.99 ± 19.42 cm^2 at 12 weeks (17.41%, P = 0.04). For the average darkness, before treatment was 3.05 ± 0.42, whereas 4 weeks after treatment 2.7 ± 0.55 (11.48%, P = 0.025) to 2.55 ± 0.7 at 12 weeks (16.4%, P = 0.037).

On comparing Group T to E, there was no significant difference between fractional Er:YAG and TCA 15% cases as regards MASI before treatment, after treatment and at end of follow up. Also no significant difference was found between E & T cases regarding the change in MASI level from baseline MASI to after treatment MASI and to the end of follow up. The same applied for area and average darkness. No rebound hyperpigmentation was observed in both groups.

Both TCA (15%) and ablative fractional Er:YAG (2940 nm) laser were found to be beneficial and gave comparably close results in the treatment of epidermal melasma. Therefore, TCA 15% is recommended due to effectiveness and less cost.

© 2014 John Wiley & Sons A/S. Published by John Wiley & Sons Ltd

P102

Glycolic acid and salicylic acid peels in melasma

S. Bansal[1], R. Sarkar[1], V. K. Garg[1]
[1]Dermatology, Maulana Azad Medical College, New Delhi, India

Melasma is acquired symmetric hypermelanosis characterized by light-to-deep brown pigmentation over cheeks, forehead, upper lip, and nose. Treatment of this condition is difficult and associated with high recurrence rates. Chemical peels have become a popular modality in the treatment of melasma.
This study was designed to compare the therapeutic response of melasma in Indian patients to glycolic acid (GA 20–35%) versus salicylic mandelic (SM) acid for chemical peeling.
Thirty Indian patients diagnosed as melasma were included. These patients were assigned in two groups of 15 patients each. Both groups were primed with 4% hydroquinone cream for 4 weeks before treatment. After a detailed history and clinical examination under natural light, melasma area severity index (MASI) was calculated and color photographs were taken of all the patients. They were then treated with graded concentrations of 20–35% glycolic acid facial peel in GA group and salicylic mandelic acid peel in the SM group. Chemical peeling is done after every 15 days in both the groups till 12 weeks. Both the groups were also advised to apply topical 4% hydroquinone cream during treatment period.
Significant response was noted in both the groups. Objective response to treatment evaluated by reduction in MASI scoring after 12 weeks was by 67% reduction in GA group and by 65.8% reduction in SM group. Nevertheless, there was no statistically significant difference between the two groups. (P = .917). Side effects were minimal. Few patients in GA group complained of erythema.
It was concluded that a serial glycolic acid and salicylic mandelic acid peel along with 4% hydroquinone proved to be an equally effective treatment modality for melasma.

P103

Clinical evaluation of efficacy of lactic acid peeling in combination with a topical regimen in the treatment of melasma

Dr. S. Dayal[1]
[1]Pt. B.D. Sharma PGIMS, Rohtak, Rohtak, India

Lactic acid is a mild peeling agent with slower penetration due to its large molecular weight producing gradual improvement in melasma. Lactic acid peel has not been combined with topical regimen (hydroquinone plus tretinoin plus topical steroid) before in the literature for enhancing its treatment efficacy in melasma.
Evaluation of clinical efficacy and safety of lactic acid peeling in combination with a topical regimen containing 2% hydroquinone, 0.025% tretinoin and 0.1% mometasone furoate in treatment of melasma.
Forty patients with Fitzpatrick's skin type III-V with epidermal melasma attending the OPD of Dermatology Department of PGIMS, Rohtak, India were included in the study. Detailed history, complete examination including Wood's Lamp examination was done in all patients. Severity of melasma was assessed by melasma area severity index (MASI) score. The patients were then randomly divided into two groups of twenty each. Group I (combination group) received 92% lactic acid (pH 3.5) peels plus topical regimen (2% Hydroquinone plus 0.025% tretinoin plus 0.1% mometasone furoate). Group 2 (control group) received only 92% lactic acid (pH 3.5) peel alone. In both groups peeling was done at every 3 weeks interval for 18 weeks (total six peeling sessions). MASI scoring was calculated at baseline and then after each successive peeling session. Paired *t*-test was used to statistically analyse the data.
Majority patients were in group of 21–40 yrs with female preponderance in both groups. Wood's lamp examination showed increased contrast in all patients in both groups indicating epidermal melasma. Mean percentage decrease in MASI score from baseline to the end of therapy in combination group (52.05% ± 11.13%) was significantly higher ($P < 0.0001$) than control group (40.20% ± 5.95%). No severe side effects were observed in either group.
Combination of lactic acid peels with topical regimen (2% Hydroquinone plus 0.025% tretinoin plus 0.1% mometasone furoate) enhances the efficacy of lactic acid peel and is a safe, well tolerated and highly effective treatment strategy for melasma.

P104

Tranexamic acid microinjections in melasma- a pilot study

S. Mangal[1], D. Parsad[2]
[1]Dermatology, PGIMER; [2]Dermatology, PGI, Chandigarh, India

Melasma is a common, acquired cause of facial hyperpigmentation with significant psychological impact. Although several treatment modalities are available, none is satisfactory.
Recently, oral tranexamic(TA) acid has gained wide attention for its role in melasma.
This pilot study was carried out to assess the efficacy and safety of intralesional localized microinjection of tranexamic acid for the treatment of melasma in Indian women.
Materials & MethodsA total of 50 women with melasma, after written informed consent, were enrolled for a prospective open pilot study of 12 weeks. 0.05 ml TA (4 mg/ml) was injected intradermally into the melasma lesion at 1 cm intervals by using a 1 ml insulin syringe with a 30-gauge needle after application of topical anaesthesia for 60 min. The procedure was repeated at weekly intervals for 12 weeks. The results were evaluated using the Melasma Area and Severity Index (MASI) scoring at baseline, 4, 8, and 12 weeks. The patient satisfaction questionnaire was documented at 12 weeks.. Safety evaluations were performed at each follow-up visit.
All the patients completed the study. A significant reduction in the MASI scoring was observed from baseline to 12 weeks (11.32 ± 6.05–6.03 ± 4.04); $P < .05$). The patients' self-assessment of melasma improvement was as follows: Four of 50 patients (8%) rated as good (51–75% lightening), 39 patients (78%) as fair (26–50% lightening), and seven patients (14%) as poor (0–25% lightening).No major adverse events were observed.
Based on the above mentioned results, we suggest that the intralesional localized microinjection of TA acid can be used as a prosimimg, safe and effective therapeutic modality for the treatment of melasma.

© 2014 John Wiley & Sons A/S. Published by John Wiley & Sons Ltd

P105

The efficacy of 4n-butyl resorcinol and resveratrol containing cream on melasma: comparison with various whitening agents using ratio of lesional/non-lesional melanin index

J. I. Na[1], J. W. Shin[1], H. R. Choi[1], K. C. Park[1]

[1]Dermatology, Seoul National University Bundang Hospital, Seongnam-si, Republic of Korea

The efficacy evaluation of whitening agent is difficult, because the general skin tone changes with season. Skin tone becomes darker during summer and lighter in winter, therefore the effect of whitening agent may be underestimated in summer and vice versa. To compensate the seasonal effects, we suggest a new parameter: a ratio of lesion/non-lesional melanin index (MI ratio). In this study we evaluated the efficacy of 4n-butyl resorcinol and resveratrol containing cream using MI ratio and compared the effects with other whitening agents.

Twenty one female subjects with melasma were enrolled. Subjects used 4n-butyl resorcinol and resveratrol containing cream for 4 weeks. Skin color of the lesional and preauricular non-lesional skin was measured with a Mexameter® (Courage and Khazaka, Cologne, Germany) at baseline, week 2 and week 4, and MI ratio was obtained. To compare the efficacy with other whitening agents for melasma, MI ratio was obtained from the data of seven other clinical trials performed in Seoul National University Bundang Hospital during 2003–2013.

Mean MI of lesional skin decreased from 201.1 to 189.4 at week2 and 182.8 at week 4, and the difference was statistically significant from week 2. MI ratio decreased from 155.9% at baseline to 142.5% at week 4 (−13.4%). This result was compared with that of seven other clinical trials on whitening agents. Among eight different whitening agents, 4n-butyl resorcinol and resveratrol containing cream showed the biggest decrease of MI ratio at week 4. Combination of oral and topical tranexamic acid containing agent showed the second biggest decrease (−13.3%), and ascorbic acid-2-glucoside and adenosine containing cream was the third (−11.4%). Other whitening agents showed +2.0% to −6.5% change of MI ratio.

4n-butyl resorcinol and resveratrol containing cream has fast and potent whitening effect on melasma. MI ratio can be used as a simple and reliable indicator for evaluation of whitening agents.

P106

Comparative study of 70% glycolic acid versus 35% tca versus 1% tretinoin peel for the treatment of melasma

N. Puri[1]

[1]Dermatology, Punjab Health Systems Corporation, Ludhiana, Punjab, India

Chemical peels are used in melasma to create an injury of a specific skin depth with the goal of stimulating new skin growth and improving surface texture and appearance. The exfoliative effect of chemical peels stimulates new epidermal growth and collagen with more evenly distributed melanin.

AimsThe aim of our study was to compare the efficacy and side effects of 70% glycolic acid versus 35% TCA versus 1% tretinoin peel for the treatment of melama in sixty Indian female patients. We selected 60 female patients of melasma for the study. The patients were divided into three groups of 20 patients each. In Group I patients 70% Glycolic acid peel was used, in Group II patients 35% TCA peel was used and in Group III patients 1% tretinoin peel was used. Both glycolic acid and TCA peels were used for a period of 3 min only where as the tretinoin peel was left on the skin of the patient for 4 h without disruption.

In group I (Glycolic acid group) patients, 65% reduction of MASI score was seen, in group II (TCA group) 59% reduction of MASI score was seen, whereas in group III (Tretinoin group) patients 50% reduction in MASI score was seen. Duration of melasma ranged between 1 and 8 yrs with a mean of 3.8 ± 4.3 yrs. Subjective response, as graded by the patient, showed good or very good response in 70% in GA group and 60% in TCA group and 50% in the tretinoin group. There was no statistically significant difference in reduction of MASI scores at the end of six peels after 12 weeks in all the three groups ($P > 0.05$). Relapse was seen in only one case after a follow up of 6 months in GA group, three patients in TCA group and one patient in the tretinoin group. Regarding side effects of peels, erythema was seen in three patients in TCA group and three patient each from glycolic acid group and tretinoin group.Itching was seen in three patients in TCA group, two patients in glycolic acid group and in one patient with tretinoin peel. Postpeel crackening was seen only in two patients in the TCA group andwas not seen in any other group.Post inflammatory hyperpigmentation was seen in two patients with TCA peel and in none of the other two groups. Considerable reduction of MASI scores was achieved in all the three groups with variable side effects with all the three peels.

P107

Combination phytic peels versus glycolic acid peels in the treatment of melasma in dark-skinned patients

S. Sethi[1], R. sarkar[2], V. K. garg[1]

[1]Dermatology, Maulana Azad Medical College; [2]Maulana Azad Medical College, New Delhi, India

Melasma is a common cause of facial hyperpigmentation which has a significant impact on the quality of life. It is characterized by pigmented macules distributed symmetrically on the sun-exposed parts of the body and seen predominantly in Fitzpatrick skin types IV-VI, especially among Hispanics, African Americans, Africans and Asians. Many treatment modalities are available, but none is satisfactory.

This study was designed to compare the therapeutic response of melasma in Indians to glycolic acid (GA) versus combination phytic acid for chemical peeling.

We selected 30 participants of melasma aged between 20 and 60 yrs from the dermatology outpatient department. After a detailed history and clinical examination under natural light and woods light, MASI was calculated and color photographs were taken of all the patients. Priming was done with hydroquinone containing triple combination which was continued between the chemical peel treatment sessions. We treated equal numbers with glycolic acid and combination phytic acid.

A significant decrease in the Melasma Area Severity Index (MASI) score from baseline to 12 weeks was observed in both groups ($P < .001$). Reduction in MASI scoring after 12 weeks was by 67% in GA group and by 56% in phytic group. Patients with epidermal-type melasma showed a better response to treatment than those with mixed-type melasma ($P < 0.05$). There was no significant difference in the treatment efficacy of phytic and glycolic peels for treating melasma in Indian patients. Based on the results of this study, GA and combination phytic peels for melasma therapy were efficacious, but the phytic peel was found to be less irritating and was better tolerated.

© 2014 John Wiley & Sons A/S. Published by John Wiley & Sons Ltd

P108

The impact of different fluence for intense pulsed light in the treatment of melasma

M. K. Shin[1], M.-I. Bae[1], M.-H. Lee[1]
[1]Department of Dermatology, Kyung Hee University, Seoul, Republic of Korea

Melasma is a common acquired symmetric brownish macules with irregular borders, often coalescing in a reticular pattern patches. Various treatment protocols for melasma have been suggested in the previous literature, but the treatment efficacy and safety varies according to the reports.
This study was aimed to investigate selective photothermolysis effect of fluence dependent IPL for the treatment of melasma.
Korean adults with bilateral melasma was enrolled our study. Randomly assigned two grouped and treated with 10 J or 13 J fluence of IPL weekly during 6 weeks. Evaluations were done at baseline and weekly during 6 weeks and after 3 weeks the final IPL treatment. The outcome assessments included subjective and investigator' assessment about visual improvement of melasma. Melanin and erythema index was scored using a Spectrophotometer.
10J and 13J IPL treatment groups decreased melanin index after 3 weeks. Effect of IPL in melasma is slightly more effect in subjects treated with 13J of IPL than 10J during 9 weeks. 10J and 13 of IPL increased erythema index transiently during 1–3 weeks, but after 4 weeks erythema index decreased than baseline.
In investigator's assessment, 55% of subjects of 10J treatments groups showed improvement than control group after 6 weeks. 65% of subjects of 13J treatments groups showed improvement than control group during after 6 weeks. In patient's subjective assessment, 50% of subjects of 10J treatments groups showed improvement and 60% of subjects of 13J treatments groups showed improvement after 6 weeks.
We suggest that low fluence IPL treatment for melasma could be more effective protocol with minimal side effects.

P109

Tranexamic acid for treatment of melasma in Korean patients: a preliminary clinical trial

M. Song[1], J.-M. Park[1], H.-S. Kim[1], H.-C. Ko[1], B.-S. Kim[1], M.-B. Kim[1]
[1]Dermatology, Pusan National University, Pusan, Republic of Korea

New treatment modality for melasma is still in demand, for its common recurrence and inconsistant outcome. Tranexamic acid is known as an antifibrinotic agent but recently, it has been shown to interferes melanocytic-keratinocytic interaction.
To evaluate the effectiveness and tolerability of tranexamic acid for treatment of melasma in Korean women.
A prospective open-label study was conducted in 15 patients with melasma. The patients received oral administration of tranexamic acid 1500 mg daily for period of 6 months. Effectiveness and tolerability was monitored and evaluated at baseline and at 1, 2, 3, and 6 months afterwards. Pigment and erythema were measured by Mexameter® and clinical improvement was evaluated by Melama Area and Severity Index(MASI), melasma quality of life scale(MELASQoL), and patient- and physician-rated improvement scale.
Obvious or moderate improvement was observed in 66.7% patients in patients-rated improvement scale and 55.5% in physician-rated improvement scale. MASI decreased from 8.5 to 4.2 ($P < 0.05$) and level of erythema and pigmentation also showed improvement ($P < 0.05$). MELASQoL documented improvement in quality of life from 2 months after oral intake of tranexamic acid. Tanexamic acid was well tolerated but 1 patient discontinued the study due to headache.
Oral tranexamc acid monotherapy could be effective and safe alternative adjuvant therapy for melasma treatment. Although, controlled, blind, large scale studies are required for the confirmation of response rate and safety of oral tranexamic acid.

P110

Treatment of melasma in men with low-fluence Q-switched neodymium-doped yttrium aluminum garnet laser versus combined laser and glycolic acid peeling

V. Vachiramon[1], S. Sahawatwong[1], P. Sirithanabadeekul[1]
[1]Dermatology, Ramathibodi Hospital, Mahidol University, Bangkok, Thailand

Treatment of melasma in men is challenging. Men need a fast, effective, and less complex method, compared to women. Low-fluence Q-switched neodymium-doped yttrium-aluminum-garnet 1064-nm laser (LFQS) and glycolic acid (GA) peeling have been shown to deliver good results in melasma patients. However, there are limited data on their efficacy in men.
To compare the efficacy and safety of LFQS monotherapy, with combined LFQS and 30% GA peeling in the treatment of melasma in men.
Fifteen male patients with mixed type melasma were randomized to receive LFQS on one side of the face and combined LFQS plus 30% GA peeling on the contralateral side of the face for five sessions at one-week interval. Participants were followed for 12 weeks afterward. The efficacy in each treatment arm was evaluated using the relative lightness index (RL*I), modified Melasma Area Severity and Index (mMASI) score, and patient satisfaction.
Twelve patients completed the study. The mean RL*I of the combined treatment side was consistently lower than LFQS monotherapy throughout the study period ($P = 0.023$ at 4th week). Patient self-assessment is favorable in the combined treatment side. A transient hyperpigmentation was observed on both sides at 2nd week after treatment initiation but improved after subsequent treatment. The overall adverse events observed in both treatment sides were mild. Two subjects with Fitzpatrick skin phototype 5 developed dyspigmentation after the last treatment.
LFQS combined with 30% GA peeling is a simple, efficacious, and fast method in clearing melasma. Therefore, it is suitable for male patients. However, the risk of dyspigmentation should also be considered in dark complexions.

Hypopigmentary Disorders

P111

A case of generalized hypopigmentation and paradoxically darkening of horis nevus in a patient on imatinib

Q. Chen[1], S. S. J. Lee[1], T. G. S. Thng[1], S. H. Chua[1]
[1]Medicine, National Skin Centre, Singapore City, Singapore

Imatinib is an orally available tyrosine kinase inhibitor used to treat chronic myeloid leukemia (CML), gastrointestinal stromal tumors

© 2014 John Wiley & Sons A/S. Published by John Wiley & Sons Ltd

(GIST) and dermatofibrosarcoma protuberans. It causes a variety of mucocutaneous pigmentary abnormalities. Generalized and gradual skin hypopigmentation is most frequently reported with majority of cases are from Asian and dark skin individuals. However there are also rare reports of hyperpigmentation mainly affecting gingivae, palate, teeth and nails. There are also contradictory reports of either imatinib inducing premature graying hair or repigmentation of gray hair. The mechanism of dyspigmentation by imatinib is likely due to either inhibition or activation of c-kit and therefore dysregulation of melanogenesis pathway.

We report here a case of 61 yr-old Chinese female who presented with generalized hypopigmentation a few months after the initiation of imatinib for her underlying CML. Her hypopigmentation worsened with the continuation of imatinib treatment. In addition, she also has preexisting Hori's nevus since young; and she noted her Hori's nevus got much darker and more widespread on her face ~1 yr after the beginning of imatinib treatment.

It is very interesting that despite the generalized hypopigmentation of skin, her Hori's nevus did not lighten as in other reported cases of either disappearance of lentigines or more disseminated vitiligo depigmentation on imatinib treatment. Instead her Hori's nevus became more prominent. Although the paradoxical hyperpigmentation could be explained by the contrast with overall hypopigmentation of the rest part of skin, it could also be true worsening of hyperpigmentation in the Hori's nevus site as patient indeed believed the dark pigmentation was not only much more prominent in color, but also more widespread.

There are numbers of reports in pigmentary alteration caused by imatinib in Chinese ethnicity; however there has been no report from Singapore so far. Therefore it is very important to highlight this condition to local doctors and emphasis the importance of sun protection in this group of patients.

In addition, we hypothesize that the co-existing opposite effects of hypopigmenation and hyperpigmentation in our patient could be target-dependent effect of imatinib to melanogenesis and it will be interesting to carry out further pathogenesis study for better understanding.

P112

Production of reactive oxygen species and anti-oxidative responses in rhododendrol- treated human melanocytes

T. Yamashita[1], M. Okura[1], Y. Ishii-Osai[1], M. Yoshikawa[1], Y. Sumikawa[1], K. Wakamatsu[2], S. Ito[2]

[1]Department of Dermatology, Sapporo Medical University School of Medicine, Sapporo; [2]Department of Chemistry, Fujita Health University School of Health Sciences, Toyoake, Japan

RS-4-(4-Hydroxyphenyl)-2-butanol (rhododendrol or rhododenol, RD) had been used as a skin-whitening agent in Japan until RD-containing cosmetics were reported to be associated with RD-induced leukoderma. Because biopsied samples taken from depigmented lesions of the patients contained few or no detectable epidermal melanocytes, it was suggested that RD exhibited a cytotoxic effect on them. In order to elucidate the molecular mechanism of cytotoxicity of RD, we have analyzed effects of RD on the expression and enzymatic activity of tyrosinase, growth and viability of melanocytic cells and production of reactive oxygen species (ROS) and expression of anti-oxidative proteins in them. RD as well as hydroquinone suppressed oxidative activity of mushroom tyrosinase in a dose-dependent manner (ID50 of RD: 89.7 μM). Expression levels of tyrosinase and tyrosinase-related protein 1 in human melanocytes detected by RT-PCR and Western blotting were not apparently affected in the presence of RD (10–80 μM). Cellular extracts were prepared from melanocytes cultured in the RD-containing media and their DOPA oxidation activitie were measured. As a result, the cellular extracts showed decreases of DOPA oxidation as concentration of RD increased. However, growth of cultured human melanocytes was not affected for at least seven days in the presence of 10–200 μM RD. By using a DCF reagent and flow cytometry, we detected ROS production in the primary melanocytes and pigmented human melanoma cells cultured with 50 μM RD. In addition, real-time PCR detected a remarkable induction of heme oxygenase-1 in melanocytes cultured in the RD-containing medium. From these results, it was suggested that RD could degrade melanocytic cells at least partially by producing ROS in spite of the induction of heme oxygenase-1.

P113

4-(4-hydroroxyphenyl)-2-butanol (rhododendrol) activates autophagy-lysosome pathway in melanocytes: a potential mechanism for skin depigmentation disorder

L. Yang[1], M. Wataya-Kaneda[1], F. Yang[1], A. Tanemura[1], N. Arase[1,2], I. Katayama[1]

[1]Department of Dermatology, Course of Integrated Medicine Graduate School of Medicine, Osaka University; [2]Laboratory of Immunochemistry, World Premier International WPI Immunology Frontier Research Center, Osaka, Japan

4-(4-hydroroxyphenyl)-2-butanol (rhododendrol) (4-HPB) has been demonstrated as a useful compound for skin whitening in the cosmetic industry. The inhibitory mechanism of 4-HPB on melanin synthesis was shown to be due to its competitive inhibition of tyrosinase activity. Recently, a kanebo product containing rhododendrol has been banned to use because it caused unexpected depigmentation disorder. To find out whether other unknown mechanism was involved, in the present study, we investigated the effect of rhododendrol on human primary epidermal melanocytes in vitro.

Cultured normal human epidermal melanocytes were treated with rhododendrol solution (300 μM, 600 μM, 900 μM) which were prepared by dissolving in dimethyl sulfoxide (DMSO), or treated with DMSO as control. Melanin contents were determined spectrophotometrically. The granules in melanocytes including melanosomes, autophagosomes and lysosomes were examined by electron miscroscopy. The expression of melanogenetic factors and autophagy-lysosome-related proteins were assessed by western blot and immunoflurosence staining analyses.

Rhododendrol-treated melanocytes demonstrated a marked decrease in melanin contents. By electron miscroscopy analysis, melanosomes were found decreased and the autophagosome and lysosome were found increased markedly in rhododendrol-treated melanocytes. Furthermore, the decrease in TRP-1, Melan-A expression, and the increase in P62, LC3, LAMP1 expression were also confirmed in rhododendrol-treated melanocytes, as assessed by western blot and immunoflurosence staining analyses.

These findings suggest a possible role of autophagy-lysosome pathway might be involved in rhododendrol-induced depigmentation disorder.

© 2014 John Wiley & Sons A/S. Published by John Wiley & Sons Ltd

P114

Hypochromic pityriasis lichenoides chronica and its association with mycosis fungoides

Y. W. Yeo[1], B. K. Goh[2]

[1]Dermatology Unit, Singapore General Hospital; [2]National Skin Centre, Singapore City, Singapore

Pityriasis lichenoides is an idiopathic acquired dermatosis characterized by recurrent crops of scaly papules which resolve spontaneously over weeks to months or may follow a more chronic course. Hypochromic pityriasis lichenoides chronica is a rarely described variant of pityriasis lichenoides which presents more commonly in the paediatric population as widespread hypopigmented macules, often associated with a few typical erythematous papules with a mica scale. Its association with mycosis fungoides is contentious with rare reports of malignant transformation in the literature. Previous studies on T cell gene rearrangements have suggested that pityriasis lichenoides chronica may be a clonal T cell disease. More recently, cases of pityriasis lichenoides-like mycosis fungoides have been reported, thus strengthening the possible link between pityriasis lichenoides and mycosis fungoides. Here we report two cases of hypochromic pityriasis lichenoides chronica, both with a similar presentation but one with a concominant mycosis fungoides. Both patients were children aged 8 and 13 and presented with erythematous papules with a mica scale associated with widespread hypopigmented macules. Clinicians should thus be mindful that a particular subset of pityriasis lichenoides chronica, in particular the hypochromic variant of pityriasis lichenoides chronica, could possibly co-exist with or evolve into pityriasis lichenoides chronica-like mycosis fungoides. Thus, close follow up is warranted.

Vitiligo Research

P115

Vitiligo: focusing on the involvement of the dermal compartment

E. Bastonini[1], D. Kovacs[1], M. Ottaviani[1], M. L. Dell'Anna[1], M. Picardo[1]

[1]San Gallicano Dermatologic Institute IRCCS, Rome, Italy

Vitiligo is an acquired depigmented skin disorder, whose etiopathogenesis is still under debate. Increasing evidences underline the key modulatory action exerted by the dermis in the control of melanocyte homeostasis. To date, few studies have focused on the possible involvement of the dermis in vitiligo. Among them, an up-modulation of the extracellular matrix protein tenascin, possibly linked to the reduced melanocyte adhesion, has been demonstrated in lesional skin. An increased expression of the fibroblast-derived factor dickkopf1 (DKK1), known to be involved in the physiological hypopigmentation of soles and palms, was also reported. In our study, we investigated the features of fibroblasts derived from non lesional vitiligo skin to evaluate the possible involvement of mesenchymal cells in inducing and/or maintaining melanocyte dysregulation associated to vitiligo. In all the experiments, the fibroblasts collected from vitiligo patients and healthy subjects were matched for age, gender and anatomical sites. We first analyzed the expression of the pro-melanogenic mesenchymal growth factors keratinocyte growth factor (KGF), hepatocyte growth factor (HGF) and stem cell factor (SCF) at mRNA and protein levels. In vitiligo fibroblasts, a significant up-modulation of KGF and HGF but not of SCF was detected. We previously demonstrated an induction of these growth factors in stress-induced premature senescent fibroblasts. We have also reported that non lesional vitiligo melanocytes are subjected to a persistent intracellular oxidative stress which, in turn, can render cells more susceptible to exogenous damages and inclined to senescence. We therefore analyzed the basal level of reactive oxygen species (ROS) in vitiligo fibroblasts and an increased spontaneous ROS production was found. We then analyzed the same senescence-associated parameters previously demonstrated to be deregulated in melanocytes. Vitiligo fibroblasts showed an higher membrane cholesterol content and an up-modulation of the markers p53, GADD45, IGFBP3, MMP3, suggesting an activation/remodeling state of the dermal compartment. Therefore, to better characterize the phenotypical features of these cells, we analyzed the expression of alpha-smooth muscle actin (α-SMA), a cellular transdifferentiation/activation marker related to both ROS and growth factors production and a significant induction of the protein was detected. The modifications observed in mesenchymal cells may be taken into account of: (i) the evidence of a broad cutaneous alteration in vitiligo, involving not only melanocytes but also dermal fibroblasts; (ii) a compensatory and protective response of the microenvironment surrounding melanocytes with the attempt to counteract their impairment through the release of paracrine mediators able to positively regulate their functions and survival. Based on these results, therapeutic approaches to be effective should act at multiple levels, modulating also the mesenchymal-derived influences.

P116

Evidence for a decreased adhesiveness of melanocytes in vitiligo skin: a preliminary and experimental assessment

L. Benzekri[1], Y. Gauthier[2]

[11]Dermatologie, Universite Mohammed V Souissi Chu Ibn Sina, Rabat, Morocco; [2]Dermatologie, Hopital Saint André, Bordeaux, France

In vitiligo, precipitating factors including mechanical traumas and oxidative stress could successively induce melanocytes damage in genetically susceptible individuals, apoptosis and induction of melanocyte-directed autoimmunity. The melanocytes involved in vitiligo may have inherent aberrations that make them vulnerable to extracellular injury. The Koebner's phenomenon generated by minor traumas may represent an example of such vulnerability. In vitiligo skin, a decreased adhesiveness of melanocytes was suggested by histological, clinical, biological (1) and genetical findings and was supported by melanocytorrhagy hypothesis (2, 3).

Aim of our studyFor the first time, using two original and different methods of assessment, we would like to investigate « ex vivo » the decreased adhesivity of melanocytes previously reported in vitiligo skin. The defects of adhesivity related to the adhesion molecules (E-Cadherin) will be studied.

1. *« Ex vivo » study of the melanocyte adhesion to the keratinocytes after centrifugation of skin samples.* Centrifugal strength was used for testing the quality of melanocyte attachment in vitiligo skin after mechanical injury. Ten skin samples from vitiligo patients and ten healthy skin samples from controls were imme-

 © 2014 John Wiley & Sons A/S. Published by John Wiley & Sons Ltd

diately centrifuged after the biopsy for 15 min with a speed about 600 tr/min. After centrifugation, the skin samples were processed under routine techniques for histological study: Hematein-Eosin (HES), HMB45 and E- Cadherin (E-Cadh) immunostainings.

2. « Ex vivo » *assessment of melanocytes adhesivity to the basement membrane after incubation of skin samples in ethylenediaminetetraacetic acid (EDTA) solution*. EDTA solution (20 nM), after variable times of incubation at 4°C, yielded excellent separation of the epidermis from the basement membrane (4). Ten skin samples from vitiligo patients and ten healthy skin samples were incubated in EDTA solution for 9, 10 and 12 h. After the incubation, the skin samples were routinely processed for histological study: HES, HMB45 and E- Cadh immunostainings.

After centrifugation, in 8/10 sections from vitiligo skin, several detached melanocytes (from 5 to 12/section) were seen in mid layers of the epidermis, whereas in 10 control skin samples, all the melanocytes were remaining in the basal layer.
After incubation in EDTA solution: In vitiligo skin samples, an early detachment of epidermis from the basement membrane was observed at 9 h (2 cases) and at 10 h (Eight cases). In control skin samples, detachment of the epidermis was observed later in four cases at 12 h and the other six samples after 12 h.
Our experimental studies could suggest primary defects in melanocyte adhesion both to the neighbouring keratinocytes and to the basement membrane. In current practice, we have to take into account the decreased adhesivity of melanocytes in vitiligo skin. This inherent aberration makes them vulnerable to extracellular injury such as mechanical traumas.

1. Delmas V et al. *J Invest Dermatol 2012;(132):S44–49*
2. Gauthier Y et al. *Pigment Cell Res* 2003;16 (4): 322–32
3. Kumar R et al. *Br J Dermatol* 2011;164 (1):187–91
4. Baker KW et al. *J.Invest Dermatol* 1983;80 (2) :104–7

P117

Aquaporin-3 in patients with non-segmental vitiligo and its association with oxidative stress and disease activity

L. Chandrashekar[1], M. Rajappa[2], K. S. Rajendiran[2], M. Munisamy[1], D. M. Thappa[1], P. H. Ananthanarayanan[2]
[1]Dermatology, Jawaharlal Institute of Postgraduate Medical Education and Research; [2]Biochemistry, Jawaharlal Institute of Postgraduate Medical Education and Research, Puducherry, India

Vitiligo is a pigmentation disorder where depigmentation occurs due to death of melanocytes in the skin. Vitiligo is associated with systemic and local oxidative stress mediated by H_2O_2. Recent reports have suggested that aquaporin-3 expression is decreased in depigmented epidermis in patients with vitiligo. We undertook to study the circulating levels of aquaporin-3 and markers of oxidative stress (malondialdehyde and total anti-oxidant status) and identify their association with disease activity. 36 non-segmental vitiligo patients and 36 age and gender matched healthy controls were included in the study. Disease activity was graded according to vitiligo index of disease activity (VIDA) scoring in all patients with vitiligo. Aquaporin-3 levels were assayed by commercially available ELISA kit. Malondialdehyde (MDA) and total antioxidant status (TAS) were assayed using standard methods in all study subjects.
Mean MDA levels were elevated, whilst TAS and aquaporin-3 were lowered in non-segmental vitiligo patients as compared to controls ($P < 0.001$). Oxidant parameters (MDA) showed a significant positive correlation with disease activity, whilst TAS and aquaporin-3 showed a negative correlation with VIDA. Aquaporin-3 levels also showed a positive correlation with TAS and negative correlation with lipid peroxidation (MDA).
Our results indicate that aquaporin-3 has a role in the pathogenesis of vitiligo and this needs to be explored further to reveal the pathomechanisms involved.

P118

Developing a questionnaire to evaluate the burden of vitiligo

K. Ezzedine[1], J. Seneschal[2], C. Salzes[1], A. Taieb[1], C. Taieb[3]
[1]Department of Dermatology, National Reference Center for Rare Skin Diseases; [2]Department of Dermatology, Natinal Reference Center for Rare Skin Diseases, CHU Bordeaux, Bordeaux; [3]Epidemiology Health Services Research, Pierre Fabre, Paris, France

Vitiligo is the most common cause of depigmentation and affects nearly 0.5% of the population. However, as the disease is not life-threatening, vitiligo is still considered by some healthcare professionals as an esthetic or cosmetic condition. Recently, a Cochrane review on interventions in viti:ligo has highlighted the need for psychological interventions in the treatment of vitiligo and there is now a consensus that treatment of vitiligo patients should take into account the psychological impact of this highly disfiguring disease. However, although several publications have reported the condition's significant impact on the patient's quality of life, there has been no study evaluating the global burden and patient reported outcomes of the disease, i.e. economic effects, work, relationships with others, sex life, daily activities.
The Objectives of this study was to create a specific questionnaire that will facilitate the evaluation of this burden in order to help healthcare practitioners to manage the difficulties encountered by patients affected with vitiligo. The questionnaire was developed as recommended in 3 phases: exploration, development and validation. Patients & Methodology Each of these phases followed its own strict methodological process, involving a multi-disciplineary team involving dermatologist, patients' associations, and experts in 'patient reported outcomes'. *Exploration phase:* A literature review revealed that the methods used to assess vitiligo are generally not specific to the condition and give very heterogeneous results, which served to confirm the necessity for creating this questionnaire. During the course of semi-structured interviews and discussions, it became clear that it would be relevant to include phototype as a parameter in the questionnaire. Following qualitative interviews, the primary effects reported by patients were: feeling of discouragement du to the disease, importance of physical disfigurement, discrimination at work, difficulty initiating intimate relationships with others, a general feeling of unease, and financial difficulties linked to treatment costs. At this stage, 56 items were generated and then reorganized according to their structure, forming 41 questions. *Development phase:* Possible responses to the questions were created in the classic method (seven items) allowing easy calibration of responses. A preliminary evaluation helped to limit redundant items and group questions according to type. Questions that did not give

© 2014 John Wiley & Sons A/S. Published by John Wiley & Sons Ltd

clear answers were also removed. The pilot version of the questionnaire comprised 25 questions, which made it suitable for the use in large-scale longitudinal studies. Finally, each question was subject to cognitive debriefing for patients' ease of comprehension and acceptability, carried out by a specialist. This stage resulted in no major modifications of the wording of the questions. In order for the questionnaire to be used in studies, it needs to undergo psychometric validation (internal and external). Factorial analysis is also needed to establish the underlying features that the questionnaire is intended to measure, and identify items that are very or too correlated with them. Finally, the answers will be scored on a fixed scale in order to ensure easy interpretation of the large-scale effects of vitiligo on patients' daily lives.

P119

Web-based vitiligo registry

K. Ezzedine[1,2], N.van Geel[3], M. Picardo[4], A. Taieb[1,2], On behalf of the VETF group

[1]Department of Dermatology and Pediatric Dermatology, National Centre for Rare Skin Disorders, Hôpital Pellegrin; [2]INSERM U1035, Bordeaux Segalen University, Bordeaux, France; [3] Department of Dermatology, Ghent University Hospital, Ghent, Belgium; [4]Laboratorio di Fisiopatologia Cutanea, Istituto Dermatologico San Gallicano, IFO, Rome, Italy

Vitiligo is the most common depigmenting disorder. It is an acquired disease characterized by a progressive loss of melanocytes. In Caucasian populations, its estimated prevalence is around 0.5% to 1%. Clinically, vitiligo can be classified into three major forms, namely segmental vitiligo (SV), non-segmental vitiligo (NSV) and mixed vitiligo (MV). Vitiligo generally begins during early or late childhood and approximately half of the patients have a disease onset before the age of twenty.

The relation between vitiligo and melanoma has long been a major topic of interest in the Dermatology community. Indeed, autoimmune leukoderma occurrence during melanoma treatment, especially interferon immunotherapy, is considered to be a good prognosis factor. In addition, the role of the cutaneous immune system in vitiligo to check and eliminate malignant melanocytes has been recently revisited based on genome wide association studies (GWAS). GWAS data suggest that better auto-immunization to melanocytic antigens may be facilitated in the context of HLA class I restriction and may help clearing abnormal as well as normal melanocytes in the context of vitiligo. The tyrosinase gene susceptibility variant identified in vitiligo GWAS is the major (Arg) allele of rs1126809, a common non-synonymous (Arg402Gln) tyrosinase polymorphism that has a minor allele frequency of 0.22–0.40 in European-derived white individuals. In contrast, the minor (Gln) allele, which is protective with respect to vitiligo, is associated with susceptibility to malignant melanoma in European-derived white individuals and represents an inverse relationship between vitiligo and melanoma.

However, these GWAS studies have been conducted without taking into account the clinical type of vitiligo and it can be suspected that some clinical features associated with vitiligo, such as the age of onset of vitiligo, halo nevi or premature hair greying, may be clinical markers of immune surveillance in vitiligo.

Yet, only a few studies searching for the association between melanoma and vitiligo have been conducted. These studies have not been taken into account the clinical spectrum of vitiligo when conducting statistical analysis. Thus, refining the relation between vitiligo and melanoma in further studies should take into account the clinical Background of patients. This may help to identify group(s) of vitiligo patients with greater protection towards melanoma. In addition, there is preliminary evidence that non melanoma skin cancer risk is also reduced in vitiligo patients.

We propose under the auspices of the Vitiligo European Task Force and with the support of ARIV, to set up an international web-based cohort study using an updated version of the 2007 original VETF form which gives a detailed phenotypic description of cases in addition to skin cancer related items (melanoma and NMSK). The principal aim of this study is to set-up an international web-based vitiligo cohort. This cohort will help to identify factors associated with disease severity/aggravation and to estimate the prevalence of skin cancer in a cohort of vitiligo patients.

P120

Comparison of vitiligo vulgaris and rhododenol-induced vitiligo by multiband digital camera with multiple linear regression analysis

M. Inoue[1], K. Yamasaki[1], T. Yamauchi[1], S. Koike[1], A. Watabe[1], K. Kikuchi[1], S. Aiba[1]

[1]Dermatology, Tohoku University Graduate School of Medicine, Sendai, Japan

Rhododenol is derived from a substance in white birch tree and inhibits tyrosinase activity and melanin synthesis. Rhododenol-containing cosmetics have been sold in Asian countries including Japan, Singapore, Thai, Malaysia, Indonesia, Filipinas, Vietnam, Myanmar, Korea, Taiwan and Hong Kong since March 2010 to July 2013. Since autumn 2012, some dermatologists observed vitiligo patients who have used the rhododenol-containing cosmetics. In March 2014, more than 18 000 were registered to as individuals with rhododenol-induced vitiligo in Japan.

The skin lesion of the rhododenol-induced vitiligo showed decrease in melanocytes and melanin contents. The differential diagnosis between rhododenol-induced vitiligo and vitiligo vulgaris is extremely difficult in some occasion because both show similar clinical manifestations of skin lesions. To clarify the clinical manifestation of rhododenol-induced vitiligo and to made accurate differential diagnosis from vitiligo vulgaris, we examined the photo-spectrometric character of rhododenol-induced vitiligo. We used the multiband filtered digital camera to take images of vitiligo patients. The multiple linear regression analysis of images from the multiband filtered camera allows us to obtain the information of the skin color as the spectrum of the absorbance between 370 and 780 nm. We examined 15 rhododenol-induced vitiligo (15 females and 0 males, 24–71 yrs old), 9 vitiligo vulgaris (Seven females and two males, 9–64 yrs old), and four individuals without skin lesions (two females and two males, 29–50 yrs old). All images were taken in the dark room with a flashlight, and the distance from the objects to the lens was exact a meter.

As we expected from the wide range of absorbance spectrum of melanin, we observed that vitiligo vulgaris lesions decrease the absorbance throughout the spectrum between 370 and 780 nm comparing to the non-lesional skins. However, the apparent reduction was observed in the absorbance between 410 and 500 nm in vitiligo vulgaris and rhododenol-induced vitiligo. We compared the maximum absorbance between 420 and 460 nm among three groups, and observed that the maximum absorbance of the groups of vitiligo vulgaris and rhododenol-induced vitiligo are significantly less than the non-lesional skins.

 © 2014 John Wiley & Sons A/S. Published by John Wiley & Sons Ltd

Moreover, the maximum absorbance of rhododenol-induced vitiligo group is significantly more than vitiligo vulgaris. Although the exact absorbance spectrum of melanin in vivo is not clear yet, the result from the multiband digital camera with multiple linear regression analysis illuminated that the skin melanin may preferentially absorb the wavelength between 420 and 460 nm.

P121

Association of *LMP7* and *TAP1* polymorphisms with vitiligo susceptibility in Gujarat population

S. Jadeja[1], M. S. Mansuri[1], M. Singh[1], A. Yeola[1], N. C. Laddha[1], M. Dwivedi[1], R. Begum[1]

[1]Department of Biochemistry, Faculty of Science, The Maharaja Sayajirao University of Baroda, Vadodara, Gujarat, India

Vitiligo is an acquired, idiopathic, progressive dermatological disorder characterized by circumscribed milky white patches on the skin affecting about 1–2% of the population worldwide. In India, the incidence is 0.5–2.5% and the states of Gujarat and Rajasthan have high incidence rate of 8.8%. Major hypotheses which could explain the pathogenesis of vitiligo are oxidative stress, autoimmune, genetic and neurochemical hypotheses. The Low Molecular weight Polypeptides (LMP) and Transporter associated with Antigen Processing (TAP) genes located in MHC class II region on chromosome 6 play important role in the regulation of MHC class I antigen processing and presentation and are reported to be involved in the pathogenesis of several autoimmune disorders. The aim of present study was to assess the role of autoimmunity in vitiligo pathogenesis by exploring the two different gene polymorphisms: *LMP7* (rs2071543) intron6 G/T and *TAP1* (rs1135216) exon10 G/A, and determining the expression levels of *LMP7* and *TAP1* in vitiligo patients as compared to controls. We have investigated *LMP7* gene polymorphism by a case-control study involving 172 patients and 139 healthy age matched controls; and *TAP1* gene polymorphism involving 134 patients and 103 healthy age matched controls using PCR-RFLP technique. Our results showed that genotype and allele frequencies of *LMP7* intron6 G/T were not significantly different ($P = 0.1344$, $P = 0.055$ respectively) between vitiligo patients and unaffected controls suggesting the non-association of *LMP7* intron 6 G/T with vitiligo. However, the genotype and allele frequencies for *TAP1* exon 10 G/A was significantly different ($P < 0.001$, $P < 0.0015$ respectively) between vitiligo patients and controls suggesting a significant association of this SNP with vitiligo susceptibility. The haplotype analysis revealed that the susceptible TA haplotype was more frequent in patients as compared to controls. Expression of *LMP7* ($P = 0.0002$) and *TAP1* ($P = 0.0152$) mRNA showed a significant increase in patients as compared to controls. In Conclusion, the present study shows significant association of *TAP1* exon10 G/T polymorphism and significant increase in expression of *LMP7* and *TAP1* mRNAs in vitiligo patients thus, suggesting the crucial role of *LMP7* and *TAP1* in vitiligo pathogenesis.

P122

A pilot trial evaluating the treatment of focal vitiligo with intralesional bee venom

H. Khurrum[1], K. Alghamdi[2]

[11]Dermatology, Vitiligo Research Chair, King Saud University; [2]Dermatology, King Saud University, Riyadh, Saudi Arabia

Vitiligo is characterized by depigmentation of skin.[1]Pigmentation of skin may result from melanocyte proliferation, melanogenesis, migration or increase in dendricity (Korean study) [2.]
Apitherapy, or ‘bee therapy’ (api is from the Latin apis meaning ‘bee’ and therapy from the Greek word meaning ‘caring service’) is the medicinal use of products made by honeybees. This therapy includes Bee Sting Therapy, also known as Bee Venom Therapy (BVT).[3]
People and physicians have used bees and bee products for healing for thousands of years. Hippocrates wrote about using bee stings and Alexander the Great was successfully treated for gout with bee stings. Beekeepers have always known about the health benefits of being stung by bees.[8]
Bee venom therapy (BVT), as a kind of herbal acupuncture, exerts not only pharmacological actions from the bioactive compounds isolated from bee venom but also a mechanical function from acupuncture stimulation.[9]
Propolis is a natural compound made by honeybees to coat the inside of their hives. Some of its ingredients have shown antioxidant and antitumor properties in early laboratory and animal studies, but it has not been tested in people.[10,11]
Melittin is a main component of bee venom. It has anti-inflammatory properties. Scientists in Australia have changed the structure of the melittin molecule by removing the part that causes allergic reactions in some patients, keeping its cell-killing ability, and combining the molecule with an antibody to target cancer cells.[12] Using this approach, they have been able to show some anti-cancer activity in studies using mice. Studies in people have not been reported in the available medical literature.
BVT is growing in popularity, especially in Korea, and is used primarily for pain relief in many kinds of diseases. Few studies demonstrated the effect of phospholipase A2 (PLA2) on melanogenesis and melanocytic proliferation.[13,14] There is one article published in Experimental and Molecular Medicine, where they reported that secreted PLA2 known as a component of bee venom (BV), stimulates melanocyte dendricity and pigmentation.[2]
The results of this study showed that BV increased the number of melanocytes dose and time dependently. 1.25–20 μg/ml of BV was treated in human melanocytes for 5 days. The level of cAMP was also increased by BV treatment. Moreover, BV induced melanogenesis through increased tyrosinase expression. Furthermore, BV induced melanocyte dendricity and migration through PLA2 activation. Overall, this study demonstrated that BV may have an effect on the melanocyte proliferation, melanogenesis, dendricity and migration through complex signaling pathways in vitro, responsible for inducing pigmentation. However, more clinical trials are needed to prove that.
Side effects of BV included systemic and local reactions. Systemic reactions are urticaria, pruritus, anxiety and nausea. More severe allergy can lead to dyspnea, asthma, angioedema, dizziness, numbness and syncope. Local reactions comprised of local swelling at injection site. Unusual reactions reported are serum sickness and generalized vasculitis.[15] Side effects can be minimized by administering histamine.

© 2014 John Wiley & Sons A/S. Published by John Wiley & Sons Ltd

P123

Screening for glaucoma prevalence in vitiligo patients as compared to healthy control group

H. Khurrum[1], K. Alghamdi[1]

[1]Dermatology, King Saud University, Riyadh, Saudi Arabia

Vitiligo is a common, often distressing condition. There are many clinical coincidences between vitiligo and open angle glaucoma (OAG). One of the basic coincidences is that both diseases are chronic, progressive and irreversible. Vitiligo is an acquired idiopathic disease, and so is POAG.

To investigate and estimate the prevalence of glaucoma in vitiligo patients, as compared to healthy population. And to determine whether there is an association between periorbital topical corticosteroid use and the incidence of OAG.

A prospective, cross-sectional study, ninety vitiligo patients were recruited from the dermatologic outpatient clinic. All vitiligo patients were checked for visual field, visual acuity, intraocular pressure assessment and fundoscopy by using specified ophthalmological tools. This group was compared with ninety age and gender matched healthy control group.

Patients with any medical illness, including allergies, systemic corticosteroid use, diabetes mellitus, and asthma were excluded. 18–55 yrs old patients were also excluded.

Detailed eye history, including cataracts, glaucoma, other eye diseases and infections, eye trauma, and corrective lenses was also taken. Detailed history about vitiligo disease including location of vitiligo, body surface area of vitiligo, duration of vitiligo, age of onset, leucotrichia, Koebner's phenomenon was noted. Family history of glaucoma, use of toxic drugs or systemic steroids, autoimmune/vascular disease or use of topical steroids around eye were noted.

Descriptive analysis was used to summarize patients' characteristics in SPSS, version 16.0 Statistical comparison was done by use of $\chi 2$-test and Mann-Whitney test. $P < 0.05$ was considered statistically significant.

The sample studied consisted of 90 patients with vitiligo. The ninety age and gender- matched controls were also recruited. Both cases and controls had no difference in term of presence of co-morbidities or age. Only two of our vitiligo patients. Because age is a recognized independent risk factor for cataract development, the relationship between vitiligo and cataract was re-examined by restricting the comparison to patients less than 50 yrs of age. We did not find significant relationship for cataracts with vitiligo. Similarly, there was no significant relationship between cataracts of any type and topical steroid use. 19.3% use topical steroids around eye but did not develop glaucoma. 2 glaucoma patients used steroids around eyes.

In our study, no glaucoma findings were noted in vitiligo patients. Moreover, patients treated for vitiligo with topical steroids in periorbital area were also not found to have any ophthalmologic problem. The present study is methodologically strong as it had large sample size and included a well-matched control group. Therefore, this clinical observation and our Conclusions did not support the hypothesis that vitiligo is associated with glaucoma.

P124

The VASI and the VETF assessment: reliable and responsive instruments to measure the degree of depigmentation in vitiligo

L. Komen[1], Vd. Graca[1], A. Wolkerstorfer[1], C. B. Terwee[2], M. A.de Rie[1,3], W. J.van der Veen[1,4]

[1]Netherlands Institute for Pigment Disorders SNIP, Department of Dermatology, Academic Medical Centre; [2]Department of Epidemiology and Biostatistics, EMGO Institute for Health and Care Research, VU University Medical Centre; [3]Department of Dermatology, Academic Medical Centre, Amsterdam; [4]Department of Dermatology, MC Haaglanden, Den Haag, The Netherlands

Vitiligo is a common skin disorder causing depigmented macules that can impair a patient's quality of life. Currently, there are no standardised outcome measures to assess the degree of depigmentation. Moreover, there is limited knowledge on the measurement properties of outcome measures in vitiligo. Therefore, the aim of our prospective study was to assess the reliability and responsiveness of the VASI and the VETFa, two well described clinician reported outcomes that assess the degree of depigmentation.

We included three groups of non-segmental vitiligo patients treated in the Netherlands Institute for Pigment Disorders. The study was designed according to COSMIN quality criteria for studies on measurement properties. To assess the inter-observer reliability, three observers, dermatologists and/or experienced vitiligo physicians, independently assessed the degree of depigmentation with the VASI and the VETFa in 31 patients. To assess the intra-observer reliability, one of the physicians repeated the measures two weeks after the first measurement, in 27 patients. The inter-rater and intra-rater reliability and the correlation in the responsiveness analyses were calculated with the Intraclass Correlation Coefficient (ICC). ICCs of >0.70 were considered adequate. To assess the responsiveness the repigmentation rate was calculated after six months in 33 patients. This repigmentation rate was tested against hypotheses.

The inter-observer reliability of the three observers was high for the VASI (ICC 0.93, 95% CI 0.88–0.96) and the VETFa depigmentation (ICC 0.88, 95% CI 0.79–0.94). The intra-observer reliability was high for the VASI (ICC 0.93, 95%CI 0.86–0.97) and the VETFa depigmentation (ICC 0.97, 95%CI 0.94–0.99). The smallest detectable change (SDC) was 7.1% and 10.4% for the inter-observer reliability and 4.7% and 2.9% for the intra-observer reliability in the VASI and VETFa depigmentation, respectively. All four responsiveness hypotheses formulated a priori were confirmed.

The VASI and the VETFa are reliable and responsive instruments to assess the degree of depigmentation in vitiligo research. Based on our results it is not possible to recommend one of these instruments specifically. Both instruments are easy and quick in use and can be used in widespread vitiligo. The VASI and the VETFa for depigmentation are potential instruments for vitiligo research in the future. However, for the use in individual patient care, caution is needed when interpreting change scores in individual patients because of the relatively large SDC.

 © 2014 John Wiley & Sons A/S. Published by John Wiley & Sons Ltd

P125

Senescence in keratinocytes and fibroblasts from perilesional skin of unstable vitiligo patients

R. Kumar[1], D. Parsad[2,2]

[1]Zoology, Panjab University; [2]Dermatology, PGIMER, Chandigarh, India

Vitiligo is a depigmenting disease of uncertain aetiopathogenesis characterized by depigmented patches due to destruction of melanocytes. Keratinocytes play an important role in melanocyte homeostasis and keratinocyte alteration may play a role in melanocyte dysfunction in vitiligo. The purpose of this study was to check the unstable vitiligo patients keratinocytes and fibroblasts for any defect as compared to normal skin of healthy control.

Fifteen unstable vitiligo patients with no ongoing treatment for last 8 weeks were selected at the outpatient clinic of the Department of Dermatology, Post Graduate Institute of Medical Education and Research, Chandigarh with their written informed consent and this part of the study was approved by the Institute Ethics Committee. Keratinocytes were isolated and cultured from perilesional skin of vitiligo patients and normal skin of healthy control. Fibroblasts were grown as explants from the dermis of perilesional skin. We examined the morphology and checked the senescence markers in the keratinocytes and fibroblasts and compared them with the controls.

Keratinocytes and fibroblasts from perilesional skin of unstable patients were found to be bigger in size, more vaculated as compared to the healthy control. We also found that senescent markers were significantly higher in unstable vitiligo keratinocytes and fibroblasts as compared to the controls.

Keratinocytes and fibroblasts are the main component of the epidermis and very important factor for the growth of melanocytes. We conclude that significant number of keratnocytes and fibroblasts in perilesional skin were senescent and are unable to support the growth of the melanocytes that may leads to the melanocyte death and depigmentation in the lesion of vitiligo patients.

P126

Depigmented epidermis in vitiligo patients shows an aberrant ultrastructure of the stratum corneum lipid lamellar membranes

T.-C. Lei[1]

[1]Dermatology, Wuhan University, Renmin Hospital, Wuhan, People's Republic of China

Beyond its role as natural photoprotectants, melanin has been recognized in recent years to possess extra biological functions, such as free radical scavenging, drug trapping and detoxification, as well as epidermal microenvironment acidification. However, there is no convincing evidence as yet that melanin contributes to maintaining epidermal permeability barrier although this barrier function expresses much more strongly in darker skin than lighter skin. One clinical report shows that loss of melanin pigment leads to abnormal permeability barrier function in vitiligo-involved skin (Liu J. et al. Skin Pharmacol Physiol. 2010, 23:193–200). To date, the research has not fully addressed the question whether the stratum corneum lipid lamellar membranes are disrupted in vitiliginous skin since such multilamellar lipid membranes are the crucial structural basis for permeability barrier in human epidermis, which holds important implications not only for the treatment of vitiligo but for deeply understanding the role of melanin in sustaining healthy permeability barrier. In this study, we assessed the ultrastructural changes of lipid lamellar membranes in lesional and perilesional skin of patients with vitiligo using a transmission electron microscopy technique in combination with ruthenium tetroxide (RuO_4) staining. Methods: (i) Patient recruitment: Three patients who suffered from stable vitiligo (2 male and 1 female, aged from 23 to 35 yrs old, disease duration 1–3 yrs from onset) and three age-matched normal volunteers were recruited in this study, written informed consent was obtained from all participants before enrollment. The Ethical Committee of Renmin Hospital of Wuhan University approved this study and supervised its compliance with the Declaration of Helsinki Guidelines; (i) Electron microscopy: Full skin biopsies were taken from depigmented (lesional) and normally pigmented (perilesional) areas of each patient, biopsies were minced to 0.5 mm^3, fixed in modified Karnovsky's fixative overnight, washed in 0.1 M cacodylate buffer, and postfixed in 0.25% RuO_4 in 0.1M cacodylate buffer for 45 min in the dark at room temperature. After rinsing in buffer, samples were dehydrated in graded ethanol solutions, and embedded in an Epon-epoxy mixture. Ultrathin sections were examined in an electron microscope after further contrasting with uranyl acetate-lead citrate. Results: (i) We first examined RuO_4 postfixed skin samples taken from normal skin, the EM images of normal stratum corneum (SC) clearly revealed that the multilayered lipid lamellae exists within the intercellular space of SC with a characteristic alternating electron-dense and electron-lucent repeating pattern. (ii) Normal-appearing lamellar membrane was visualized in the pigmented perilesional skin of vitiligo, but the lamellar organization was drastically disrupted in depigmented lesional skin, such as fragmentation and lamellar separation. Conclusion: Our findings provide the unambiguous evidence that the ultrastructure of SC lamellar membrane is destroyed in the depigmented epidermis in vitiligo, the molecular mechanism underlying melanin-related lipid lamellae maintenance is a key point worthy of exploration. (This study was supported by a grant from the National Natural Science Foundation of China (No. 81371717) and in part by a CMA-L'Oreal China Skin Grant (No. S2011080814).
ultrastructure; lipid lamellar membranes; vitiligo.

P127

Identification of potential micrornas responsible for pathogenesis of vitiligo

M. S. Mansuri[1], M. Singh[1], N. C. Laddha[1], M. Dwivedi[1], S. R. Bhalara[1], Y. S. Marfatia[2], R. Begum[1]

[1]Department of Biochemistry, Faculty of Science, The Maharaja Sayajirao University of Baroda; [2]Department of Skin and V.D., Faculty of Medicine, The Maharaja Sayajirao University of Baroda, Vadodara, Gujarat, India

Vitiligo is an acquired depigmentation disorder characterized by the milky white patches on the skin which occurs due to the destruction of melanocytes. The precise *modus operandi* for pathogenesis of vitiligo has remained elusive. Theories regarding loss of melanocytes are based on autoimmune, cytotoxic, oxidant-antioxidant and neural mechanisms along with various genetic factors. The onset and progression of vitiligo are thought to be driven by multiple inherited genes and environmental triggers. microRNAs (miRNAs) are small conserved non-coding RNA molecules that post-transcriptionally regulate gene expression by targeting the 3′ un-translated region (UTR) of specific mRNA/s for degradation or translational repression. The aim of present study was to detect the differentially expressed

© 2014 John Wiley & Sons A/S. Published by John Wiley & Sons Ltd

miRNAs and to explore their potential role in non-segmental vitiligo (NSV). We performed whole miRNA profiling of lesional and non-lesional skin from patients and controls using TaqMan® Low Density Array (TLDA). Further, we used customized TLDA cards to estimate the expression levels of differentially expressed skin miRNAs in whole blood from vitiligo patients and controls. The DataAssist™ Software Version 3.01 software from Applied Biosystems® was used for miRNA profiling data analysis. Our results suggest that 38 miRNAs were differentially expressed in the patients compared to controls. We identified 13 miRNAs which were significantly differentially expressed in lesional skin of patients. Further, 29 miRNAs were found to be significantly differentially expressed between non-lesional skin of patients and controls. Interestingly, three miRNAs were specifically down-regulated in the lesional skin compared to non-lesional skin from patients. In addition, validation of these 38 differentially expressed skin miRNAs in blood from vitiligo patients revealed that five miRNAs: miR-1, miR-184, miR-328, miR-383 and miR-577 contain similar pattern of expression as of skin, suggesting their potent eminence for being putative markers for vitiligo. In Conclusion, for the first time the present study suggests the crucial role of differentially expressed miRNAs in vitiligo patients.

P128

An immunohistochemical study on segmental vitiligo: a joint study of the Korean vitiligo association

J. H. Shin[1], H. Y. Kang[2], K. H. Kim[3], C. J. Park[4], S. H. Oh[5], S. C. Lee[6], S. H. Lee[7], G. S. Choi[1], S. K. Hann[8]

[1]Dermatology, Inha University Hospital, Graduate School of Medical Science, Incheon; [2]Dermatology, Ajou University School of Medicine, Suwon; [3]Dermatology, Dong-A University College of Medicine, Busan; [4]Dermatology, Bucheon St. Mary's Hospital, College of Medicine, The Catholic University of Korea, Bucheon; [5]Dermatology, Severance Hospital, Cutaneous Biology Research Institute, Yonsei University College of Medicine, Seoul; [6]Dermatology, Chonnam National University Medical School, Gwangju; [7]Dermatology, Soonchunhyang University College of Medicine, Bucheon; [8]Korea Institute of Vitiligo Research & Drs. Woo and Hann's Skin Center, Seoul, Republic of Korea

Segmental vitiligo (SV) is restricted to the dermatome and its clinical characteristics differ from those of nonsegmental vitiligo (NSV). While NSV is known as an autoimmune disease to melanocytes, the pathogenesis of SV is still uncertain. However, recent studies suggested an overlapping autoimmune mechanism between SV and NSV. Although the histopathological findings of fully developed vitiligo lesions have no melanocytes or inflammation, T-cell infiltration is observed in the margins of active lesions in NSV. However, the histopathological findings in the active margin of SV are little known.

To determine if T-cell inflammatory responses are present in the early active margin of SV using immunohistochemical staining.

Fifteen patients diagnosed with early or actively spreading SV were enrolled from different hospitals according to internal review board regulations. Disease duration or spreading was <8 weeks. For each patient, 3-mm punch biopsies were taken from the lateral inner margin of the actively spreading lesion and the normal skin on the same dermatome of the other side. After reviewing the hematoxylin and eosin-stained slides, specimens from 12 patients were stained for CD4, CD8, CD25, interferon gamma (IFN-r), stem cell factor (SCF) and nerve growth factor (NGF) in immunohistochemical studies. The staining results were quantified using a computer image analysis program and unpaired student *t*-test was performed to determine statistical significance.

Seventy percent (8/12) of patients showed T cell infiltration in the active margin of SV. CD4+ T cells infiltrated the dermis; however, CD8+ T cells were present in the epidermis or basal layer. The CD8+ T cell increase was significant ($P < 0.04$), though CD4+ or CD25+ T cells appeared increased no significantly. SCF and NGF were decreased in the lesion ($P < 0.03$, $P < 0.21$, respectively). IFN-r was not increased in the lesion.

In this study, the active margin of SV showed CD8+ T-cell inflammatory responses. This result suggests that SV also has an autoimmune response to melanocytes. SCF and NGF were decreased in the lesion, suggesting that keratinocytes and nerve factor are also involved in the pathogenesis.

P129

Vitiligo and metabolic syndrome: a case control study

A. Singh[1], R. Chander[1], V. Mendiratta[1], R. Singh[2], A. Sharma[2]

[1]Dermatology, Venereology, Leprology, Lady Hardinge Medical College; [2]Biochemistry, Lady Hardinge Medical College, New Delhi, India

Vitiligo is a chronic progressive depigmenting disease of multifactorial etiology. It is known to be associated with diabetes mellitus and insulin resistance. Vitiligo patients may have a preponderance to develop metabolic syndrome owing to the common autoimmune, proinflammatory and genetic factors. We enrolled 35 vitiligo patients of age >20 and <50 yrs with >5% body surface area involvement in our study. 30 age and sex matched healthy controls were also recruited. Body mass index (BMI), waist circumference and blood pressure were recorded for each of the subjects. Fasting plasma glucose, total cholesterol, triglycerides, high-density lipoprotein (HDL) and insulin levels were measured. These parameters were utilized for assessing the presence of metabolic syndrome using the International Diabetes Federation (IDF) criteria and the Homeostasis model assessment (HOMA-IR) method for insulin resistance. Analysis was made for the association of duration, extent, severity and activity of vitiligo with metabolic syndrome. The association of vitiligo with metabolic syndrome was found to be highly significant compared to controls ($P < 0.001$). The waist circumference and BMI of cases (96.54 ± 13.53 cm, 25.18 ± 4.59 kg/m^2) were significantly higher than in controls (86.93 ± 7.83 cm, 21.79 ± 2.51 kg/m^2; $P < 0.01$). Moreover, 40% of the vitiligo patients were found to be hypertensive in contrast to only 3.3% of the controls. The serum triglycerides and insulin level were also notably raised in cases (141.8 ± 57.1 mg/dl, $7.78 \pm 2.21 \mu$IU/l) in comparison to controls (111.13 ± 34.56 mg/dl, $5.62 \pm 2.44 \mu$IU/l; $P < 0.05$). The HDL level was low in cases as compared to controls (<50 mg/dl in females and <40 mg/dl in males; $P < 0.05$). However, there was no difference in the plasma glucose and serum cholesterol levels. Insulin resistance was not significantly associated. No association was found between the duration, extent and activity of vitiligo with metabolic syndrome. It is possible that patients with vitiligo are at a higher risk of developing metabolic syndrome and dyslipidemias. Larger, multicentric trials would be needed to further elucidate this association.

 © 2014 John Wiley & Sons A/S. Published by John Wiley & Sons Ltd

P130

Are vitiligo patients at a higher risk of developing atherosclerosis? – a pilot study

A. Singh[1], R. Chander[1], V. Mendiratta[1], R. Singh[2], A. Sharma[2]

[1]Dermatology, Venereology, Leprology, Lady Hardinge Medical College; [2]Biochemistry, Lady Hardinge Medical College, New Delhi, India

The etiopathogenesis of vitiligo is complex and involves the interplay of inflammation, autoimmunity and oxidative stress among others. Insulin resistance and raised homocysteine levels have also been found in these patients. Proinflammatory and oxidative milieu is responsible for development of early atherosclerotic plaques. Elevated homocysteine level is also an independent risk factor for atherogenesis. Besides these, polymorphism of atherosclerogenic genes endothelin-1 and angiotensin converting enzyme (ACE) has been found in vitiligo patients. Hence, it is possible that vitiligo patients maybe predisposed to atherosclerosis.

To determine the level of serological biomarkers of early subclinical atherosclerosis in vitiligo patients to establish any likely association.

Thirty five vitiligo patients and 30 age and sex matched healthy controls with no previous history of cardiocascular disease were enrolled in our study. Blood samples from each of the subjects were obtained for determination of high-sensitivity C-reactive protein (hsCRP) and oxidized low-density lipoprotein (oxLDL) by enzyme immunoassay along with total cholesterol, triglycerides, high-density lipoprotein (HDL) levels.

The difference between mean serum hsCRP and oxLDL levels in vitiligo cases (3.9 $\pm$ 2.3 mg/l, 313.5 $\pm$ 157.3 pg/dl) and controls (2.3 $\pm$ 1.4 mg/l, 172.1 $\pm$ 103.8 pg/dl) was highly significant ($P < 0.01$). The HDL level was low in cases as compared to controls (<50 mg/dl in females and <40 mg/dl in males; $P < 0.05$). Triglycerides were raised in cases than controls (141.8 $\pm$ 57.1 mg/dl, 111.13 $\pm$ 34.56 mg/dl; $P < 0.05$). There was no difference in the total cholesterol level.

We propose that vitiligo patients are at a higher risk of developing atherosclerosis and thereby, cardiovascular accidents. Timely screening of such individuals would help in early diagnosis and treatment of subclinical atherosclerosis. However, larger multicentric studies utilizing other serological and radiological markers of early atherosclerosis would assist in further establishing this association.

P131

Association of interleukin 6 -572 G/C and -174 G/C promoter polymorphisms with vitiligo susceptibility in Gujarat population

M. Singh[1], M. S. Mansuri[1], N. P. Raval[1], N. C. Laddha[1], M. Dwivedi[1], R. Begum[1]

[1]Department of Biochemistry, Faculty of Science, The Maharaja Sayajirao University of Baroda, Vadodara, Gujarat, India

Vitiligo is an acquired pigmentary disorder characterized by areas of depigmented skin resulting from loss of functional melanocytes. It affects 0.5–1% of the world population. The contribution of genetic factors in susceptibility to vitiligo is exemplified by familial clustering. Several theories have been proposed to explain the mechanism of vitiligo pathogenesis however, autoimmune hypothesis is well accepted. IL6 is a pleiotropic cytokine with a wide range of biological activities in immune regulation, hematopoiesis, inflammation and oncogenesis. IL6 plays an important role in the pathogenesis and progression of many autoimmune disorders. Increased levels of IL6 have been reported in the serum samples of vitiligo patients. IL6 −572 G/C and −174 G/C promoter polymorphisms have been studied in many autoimmune diseases like rheumatoid arthritis, type 2 diabetes, celiac disease and it has shown association with respective autoimmune diseases. The aim of present study was to explore promoter IL6 −174 G/C (rs1800795) and −572 G/C (rs1800796) single nucleotide polymorphisms (SNPs) in *IL6* and to study their associations with vitiligo susceptibility. PCR- RFLP was used for the analysis of −572 G/C and −174 G/C *IL6* promoter polymorphisms in 107 vitiligo patients and 101 unaffected age-matched controls of Gujarat population where prevalence of vitiligo is alarmingly high (i.e. 8.8%). The genotype frequency for −572 G/C SNP significantly differed between patients and controls ($P = 0.024$) however, allele frequency did not differ significantly ($P = 0.1198$). We observed only single type of genotype (GG) and allele (G) for IL6 −174 G/C promoter polymorphism (rs1800795) in both patient and control populations. In Conclusion, our results suggest that the −572 G/C promoter polymorphism of *IL6* may be a genetic risk factor for vitiligo susceptibility in Gujarat population.

P132

Altered gene expression, oxidative stress and melanocytorrhagy in vitiligo pathogenesis

S. Tanwar[1], D. Parsad[1], A. Bhatia[2]

[1]Dermatology, Post Graduate Institute of Medical Education and Research; [2]Experimental Medicine and Biotechnology, Post Graduate Institute of Medical Education and Research, Chandigarh, India

Melanocytes death pathways in vitiligo via apoptosis, melanocytorrhagy and autoimmunity can be consequences of irregulated gene expression in patients in response to stressed conditions at the depigmenting site. In past few years oxidative stress has gained a sound importance as a contributor to vitiligo pathogenesis. Probably vitiligo patients are more prone to develop oxidative stress due to weak antioxidant system genetically. Supporting this we found low expression level of catalase the typical antioxidant system gene, in patients in comparison to controls. Also we found statistically significant low antioxidant power of patient's plasma in comparison to controls suggesting higher level of oxidative stress in patients. Up regulated level of catecholamines in patient's perilesional skin biopsy estimated via HPLC as well as its metabolism regulatory genes COMT and GTPCH1expression data strongly support its role in vitiligo pathogenesis at the perilesional site. Catecholamines being a local factor in skin at depigmenting site are most probable contributor to oxidative stress generated at the perilesional site due to its toxic oxidative products accumulation. Catecholamines are known to play an important role in induction of proinflammatory cytokines. Both cytokines as well as reactive oxygen species produced during oxidative stress are quite possible stimuli to alter gene expression of a cell being part of signal transduction phenomenon. However up and down regulation of specific genes expression is a normal phenomenon in response to a signal originating external or internal to the cell. But these conditions in periphery of melanocytes can lead to apoptotic or melanocytorrhagic changes via irregulating its gene expression system. We observed significant differences in melanocytes development, proliferation, migration and adhesion as well as melanogenesis regulatory system genes

© 2014 John Wiley & Sons A/S. Published by John Wiley & Sons Ltd

expression level at the perilesional site in active patients in comparison to controls. Differences in gene expression level also observed at perilesional and normal site of same patients.

P133

Corticotrophin releasing hormone (CRH) and its receptor (CRHR1) gene expression as a component of skin hpa axis in response to stress in vitiligo

A. M. Tawdy[1], S. M. Tahlawy[1], S. O. Tawfeek[1], N. A. Bedair[1]
[1]Dermatology, Kasr EL Ainy, Cairo, Egypt

Psychological stress is known to aggravate autoimmune skin diseases such as vitiligo, psoriasis and alopecial areata by altering the cellular constituents of the immune system.Human skin is a local source of corticotropin-releasing hormone (CRH) and expresses CRH and CRH receptors (CRH-R) at mRNA and protein levels. Epidermal melanocytes respond to CRH by induction of cAMP with up-regulation of pro-opiomelanocortin gene expression and subsequent production of adrenocorticotropin hormone The skin appendages function dually as prominent target sand sources of the peripheral corticotropin-releasing hormone–proopiomelanocortin (CRH-POMC) axis.
We examined the expression level of CRH and CRHR-1 in vitiligo, a well-known stress-related autoimmune skin disease, as well as detecting psychological stress in the last 6 months prior to biopsies.
This study was conducted on patients suffering from Non Segmental Vitiligo who presented to the Dermatology outpatient clinic, Kasr El Eini School of Medicine, Cairo University, during period from 7\2011 till 3/2012 after approval of Kasr El Einy scientific ethical committee. An informed consent was obtained from each patient before enrollment in the trial.
We measured CRH and CRHR-1 mRNA expression in vitiligo lesional, nonlesional skin and healthy control by quantitative RT-PCR in vitiligo lesional, nonlesional and control skin. We also detected psychological stress using Social Readjustment Stress Scale.
There was significant increase of CRH and CRHR-1 expression in both lesional and nonlesional skin of vitiligo patients compared to normal control.Psychological stress also showed significant increase in vitiligo patients compared to normal control. Both markers were significantly correlated with psychological stress in vitiligo patients.
CRH-POMC system could have a substantial immunomodulatory role in skin as well as in nervous system and might be directly encountered in the aetiopathogenesis of vitiligo in response to psychological stress.

P134

Regulatory T Cell (Treg) and T cell immunoglobulin and mucin domain (TIM) mediated immune regulation in active generalized vitiligo

M. K. Tembhre[1], V. K. Sharma[1], S. Gupta[1], A. Sharma[2], A. S. Parihar[1]
[1]Dermatology and Venereology, All India Institute of Medical Sciences; [2]Biochemistry, All India Institute of Medical Sciences, New Delhi, India

Vitiligo is an acquired autoimmune depigmentary disease where deregulation in regulatory T cells (Tregs) has been proposed in the pathogenesis of generalized vitiligo (GV). The role of T cell immunoglobulin and mucin domain 3 (TIM-3)+ Tregs is not known in active GV (aGV).
To characterize the CD3 + CD4 + CD25 + FOXP3 + regulatory T cells (Tregs) and TIM3 + Tregs, and to determine the effect of TIM-3 blocking on Treg associated markers in the peripheral blood mononuclear cells (PBMCs) of aGV and controls.
The percentage of circulating Tregs and TIM3 + Tregs was evaluated in 50 aGV and 51 controls using multi-parameter flow cytometry. The mRNA expression levels of Foxp3, TGF-β and CTLA-4 was quantified in PBMCs after in vitro blocking with anti-TIM-3 antibody in the presence of anti-CD3 antibody in a time dependent manner in both the groups ($n = 30$ each).
The percentage of Tregs was lower ($P < 0.05$) and the percentage of TIM3+ Tregs were higher ($P < 0.05$) in peripheral blood of aGV compared to controls. When compared with percentage body surface area affected, a significant negative correlation was observed with percentage of Tregs. There was no significant difference revealed in the mRNA expression levels of FOXP3, TGF-β and CTLA-4 at 24, 48 and 72 h of treatment except for FOXP3 that was significantly increased at 48 h in aGV group compared to controls. Further, TGF-β mRNA levels were also markedly increased at 48 and 72 h post-treatment in aGV, but the difference was not significant. The percentages of Tregs and TIM3+ Tregs were analyzed using independent sample t-test and Mann–Whitney *U*-test respectively. The quantitative gene expression data was analysed using Freidman's non-parametric test. The correlation study was performed using Spearman's rank correlation test. Statistical significance was assigned to a value of $P < 0.05$.
Higher TIM3 + Treg frequency in aGV and inhibitory effect of TIM-3 on FOXP3 and/or TGF-β expression suggested the annihilating effect of TIM-3 on Treg function. Decrease in Treg frequency may occur due to enhanced engagement of TIM-3 to its ligand galectin-9 that may lead to the exhaustion and eventually apoptosis of Tregs. Further, investigation is required to delineate the involvement of TIM-3/Galectin-9 signaling pathways in regulating Treg function in aGV.

Vitiligo: Clinical

P135

Vitiligo in a case of systemic lupus erythematosus: a concurrent course

S. Aminpoojary[1], J. Bagadia[1]
[1]Department of Dermatology, K.J. Somaiya Medical College, Mumbai, India

Vitiligo has been found to be associated with several autoimmune disorders including Systemic Lupus Erythematosus (SLE). An increased incidence of vitiligo has been reported in patients of SLE due to a common susceptibility gene locus, SLEV1 on chromosome 17p13. Also, ~25% patients with an autoimmune disease have a tendency to develop additional autoimmune disorders.
Case ReportA 32 yrs old female, presented with white patches over forehead, lips and both upper extremities since a duration of 4 months. These lesions were gradually increasing in size and number. The patches showed accentuation on examination with Wood's lamp. A clinical diagnosis of vitiligo vulgaris was made.
In addition to the white patches, she complained of severe joint pains involving both knee, wrist and interphalangeal joints. She also had multiple painless ulcers in the oral cavity, Raynaud's

 © 2014 John Wiley & Sons A/S. Published by John Wiley & Sons Ltd

phenomenon and thin fragile scalp hair. There was however no significant family history suggestive of any autoimmune disease. On reviewing her medical records, she was found to have been diagnosed with SLE 9 months prior to the present episode. At that time she had complaints of malar rash associated with photosensitivity and joint pains. She also had history of recurrent spontaneous abortions in the past. Nine months back, she had been started on hydroxychloroquine and predisolone 40 mg, which had been gradually tapered to 10 mg. The tapering of prednisolone dose to 10 mg had coincided with exacerbation of SLE along with onset of vitiligo.
Her investigations revealed severe anemia, leucopenia, high ANA titers and anti-dsDNA positivity. Her thyroid profile and serum cortisol levels were normal. Her renal function tests were also within normal limits.
We restarted her on high dose oral prednisolone which resulted in improvement in her symptoms of SLE and also stabilized her vitiligo lesions. Four months later, when her steroids were tapered, she again developed an exacerbation of SLE along with multiple new lesions of vitiligo. The patient is currently on prednisolone 30 mg and topical tacrolimus. Both SLE as well as vitiligo is currently stable.
This case emphasizes the need for continued surveillance in a patient of any autoimmune disorder for development of other autoimmune diseases. Also, correlation of disease activity may be found between these autoimmune processes with simultaneous exacerbations/remissions and therapeutic management can be tailored accordingly.
An unusual feature in this case was SLE as initial clinical presentation followed by vitiligo. Commonly, in multiple autoimmune disorders associated with vitiligo, vitiligo is the first autoimmune disease to be detected followed by the other disorders.

P136

The correlation between serum level of 25-hydroxyvitamin D and clinical improvement in vitiligo patients after NB-UVB 311 nm phototherapy

R. F. Dwiyana[1], D. T. MP[1], E. Sutedja[1]
[1]Department of Dermatology and Venereology, Faculty of Medicine, Padjadjaran University/Hasan Sadikin Hospital, Bandung, Indonesia

Vitamin D deficiency recently has been found in several autoimmune diseases including vitiligo. In the pathogenesis of vitiligo, vitamin D is thought to play a role in immunoregulation and as an antioxidant to prevent melanocyte destruction. Narrowband UVB (NB-UVB) phototherapy 311 nm is the treatment of choice for vitiligo. NBUVB 311 nm is able to induce the provitamin D3 conversion in the epidermis, forming 25-(OH)D. Vitamin D status can be evaluated by measuring the 25-(OH)D serum level This study aimed to evaluate the 25-(OH)D serum level increment before and treated with NB-UVB 311 nm phototherapy, and to evaluate if there is positive correlation between the 25-(OH)D serum level in the vitiligo patients before and after receiving NB-UVB 311 nm phototherapy with clinical improvement, evaluated with vitiligo area scoring index (VASI).
This study was conducted on May-July 2013, at the Department of Dermatovenereology Outpatient Clinic, Hasan Sadikin Hospital, Bandung. This is an analytical experimental prospective clinical study with a pre-post design. Subject consisted of 15 nonsegmental vitiligo patients, collected with consecutive sampling. After history taking and physical examination, serum extractions were performed from the subjects to evaluate the 25-(OH)D level at the first week, then the subjects received twice weekly phototherapy for 8 weeks, and serum extractions were performed at the 8th week.
There was a significant increase of 25-(OH)D serum level after NB-UVB 311 nm phototherapy treatment ($Zw = -3296$; $P = 0.001$). There was a positive correlation between the increase of 25-(OH)D level increment after NB-UVB 311 nm phototherapy with clinical improvement evaluated with VASI, but it was not statistically significant ($rx = 0.154$, $P = 0.585$).
This study concluded that there was a significant increase of 25-(OH)D level after 16 sessions of NBUVB phototherapy in vitiligo patients, and there is a positive correlation between the 25-(OH) D increment with clinical improvement based on VASI, however it was not statistically significant. All of the subjects in this study had a lower vitamin D level. Study with longer duration of photherapy are needed for vitiligo patient.

P137

Skin phototype may influence the burden of vitiligo

K. Ezzedine[1], J. Seneschal[1], C. Salzes[1], A. Taieb[1], C. Taieb[2]
[1]Department of Dermatology and Pediatric Dermatology, Rare Skin Diseases Reference Centre, CHU de Bordeaux, Bordeaux; [2]Public Health, PFSA, Paris, France

Vitiligo is the most common cause of depigmention in the general population. Although the disease does not lead to severe physical illness, vitiligo can be a psychologically devastating disease. Patients suffering from vitiligo experience various degrees of psychosocial impairment and altered quality of life (QoL) although little attention has been paid to the disability it causes in the broadest sense of the term (financial, professional, interpersonal relationships, sexuality and daily activities). Despite this, as vitiligo is not a lethal disease, doctors have traditionally been dismissive of it and patients are often told that there is nothing much to be done and that they should 'accept' and live with their condition.
However, it is now widely accepted that the treatment of vitiligo must take into account the patient's distress and complaints and that management of the disease should include psychological interventions, as reported in the last Cochrane review of vitiligo (Whitton et al., 2000). To date, no study has specifically explored the burden of the disease and its relation to skin phototype in Northern countries, i.e. Europe and the USA.
The aim of this study was to determine whether vitiligo-related distress is influenced by skin phototype.
French law does not permit 'ethnic' studies, which means that it is not possible to select a group of patients on the basis of their skin colour. To overcome this obstacle, we used skin phototype as our selection criterion. We were thus able to divide our subjects into a fair to intermediate skin group (Fitzpatrick skin phototype I, II, III) versus a darker skin complexions (Fitzpatrick skin phototype IV, V, VI). The terms we used ('feeling discouraged', 'fear of passing it on to a child', 'sexuality', 'the way others look at me', 'difficulty in answering questions', 'difficulties getting a job' and vitiligo 'perceived as a disability' as the last question) were verbatim responses collected from a face-to-face interview with vitiligo patients.
123 subjects were invited to participate. Of these, 96 below to the fair complexion group (Fitzpatrick skin phototype I, II, III) and 27 to the darker complexion group (Fitzpatrick skin phototype IV, V, VI). Significantly more subjects with dark and black skin (phototypes IV to VI) considered vitiligo to be a 'disability' (50% in phototypes IV to VI versus 29.4% in phototypes I to III, $P = 0.03$).

© 2014 John Wiley & Sons A/S. Published by John Wiley & Sons Ltd

In addition, darker phototypes were significantly more frequently concerned about the impact of the treatment on their sexuality (17.9% in phototypes IV to VI versus 10.5% in phototype I to III, P = 0.014), more concerned about the perception of their physical aspect by others (64.3% in phototypes IV to VI versus 42.9% in phototype I to III, P = 0.008).Besides, both groups expressed similar opinions concerning difficulties in getting a job and the fear of passing on the disease to a child.
There is a marked difference in the burden of vitiligo experienced by patients with dark skin phototype as compared to those with lighter skin phototypes. This should be taken into account in future interventions involving psychological therapies.

P138

A study to validate the proposed clinical criteria for diagnosis of segmental vitiligo

B. K. Khaitan[1]
[1]Dermatology, All India institute of Medical Sciences, New Delhi, India

Vitiligo is traditionally divided into two distinct clinical forms: segmental and non-segmental vitiligo. Both non-segmental and segmental vitiligo can initially give the appearance of focal vitiligo, which is characterized by a localized affected area.
Early recognition of segmental vitiligo can help to reassure the patient that it usually does not spread to other parts of the body, it stabilizes early, responds very well to surgical methods with lower risk of recurrence. In early and localized cases where the diagnosis is uncertain, clinical criteria to differentiate segmental and non-segmental vitiligo are required. Based on our earlier study on 188 patients, a set of criteria was proposed:
Essential Criteria: (i) Acquired depigmented macules occurring unilaterally not crossing the midline or with minimal spill; (ii) Localized to a particular body area/site; and (iii) Patterned distribution including recognizable patterns viz. blaschkoid, dermatomal, phylloid, checkerboard, linear/oblong or any other specific type.
Additional Criteria: (i) Age at onset <15 yrs; (ii) Leucotrichia >50% over affected lesion; (iii) Leucotrichia extending beyond margin; (iv) Presence of islands of normal or hyperpigmentation within the lesion; (v) Margins >50% irregular/straight line; (vi) Stability achieved in by 1 yr.
Patients fulfilling all essential and one or more of the additional criteria were labelled as segmental vitiligo and remaining patients as non-segmental vitiligo.
To validate the proposed clinical criteria, 120 patients were recruited with 139 sites involvement. Each site was separately assessed by three experts (gold standard) and was also classified by the criteria. Out of 139 cases according to experts' consensual opinion, 73 cases (52.51%) were classified as 'segmental vitiligo' and 32 cases (23.02%) were classified as 'non-segmental vitiligo'. With criteria, 86 cases (61.87%) were diagnosed as segmental vitiligo and 39 cases (28.06%) were diagnosed as non-segmental vitiligo. Thus, on comparison, this set of clinical criteria was found to be a highly specific (100%) and sensitive (93.59%) diagnostic tool for segmental vitiligo.
The mean age at onset was 15.96 yrs (segmental vitiligo 14.5 yrs, non-segmental vitiligo 18.32 yrs). There were 13 patients (9.35%) who had family history of vitiligo: segmental-7 (8.14%) cases, non-segmental- 6 (11.32%) cases]. The lesions followed a blaschkoid pattern in 53.48% (46/86) of segmental vitiligo followed by dermatomal pattern and linear pattern in 9.3% (8/86) cases. Leucotrichia was present in 70 cases (segmental vitiligo: 65.12% and non-segmental vitiligo: 39.63% cases]. The leucotrichia was extending beyond the margin of the lesion in only segmental vitiligo group 13/50 cases (15.12%) and in no patients with non-segmental vitiligo. Hence this is a very specific feature. The margin of the lesion was predominantly irregular (rather than smooth) or sharp straight line in 72.09% cases of segmental vitiligo and predominantly smooth in 73.58% cases of non-segmental vitiligo. Islands of hyperpigmented or normopigmented macules were noted in 77 cases [57 (66.28%) in segmental vitiligo and in 29 cases (33.72%) in non-segmental vitiligo].
Hence, it can be concluded that the clinical criteria for diagnosis of segmental vitiligo is a highly sensitive and specific tool. Also, the various additional criteria described in this study, needs to be further evaluated to assess their diagnostic importance and order of preference.

P139

Is there a real relationship between serum level of homocysteine and vitiligo?

H. Khurrum[1], K. Alghamdi[2]
[1]Dermatology, Vitiligo Research Chair, King Saud University; [2]Dermatology, King Saud University, Riyadh, Saudi Arabia

Reduced folic acid and Vitamin B_{12} have been found in vitiligo patients. Folic acid and vitamin B_{12} deficiencies cause homocysteine elevations. Homocysteine may mediate melnocyte destruction and therefore play a role in the pathogenesis of vitiligo. This study was designed to determine the levels of homocysteine, folic acid and vitamin B_{12} in vitiligo patients in order to reveal possible associations in the pathogenesis of vitiligo.One hundred and fifty-three vitiligo patients of both sexes and age and sex matched controls were included in the study. After excluding factors that may affect serum homocysteine levels, blood samples were taken from patients and controls to determine the levels of homocysteine (Hcy), folic acid (FA) and vitamin B_{12}.The median serum Hcy level in cases and controls was 12.73 umol/l and 12.94 respectively (P = 0.94). The median FA level was 10.0 ng/ml in patients and 7.60 ng/ml in controls (P = 0.001). The median B12 level was 351.6 pg/ml in patients and 356.85 pg/ml in controls (P = 0.76). The median levels of Hcy, FA and vitamin B12 were not affected by the type of vitiligo and activity (P = 0.08).The results of our study revealed that there was no association between serum levels of Hcy and vitamin B_{12} and vitiligo. However, FA was higher in patients than in controls.
This case controlled study showed that Hcy, and vitamin B12 levels were not significantly different in vitiligo patients from healthy controls, while FA levels are high in vitiligo patients as compared to controls. Additional studies in larger series are needed to investigate the potential role of folic acid in vitiligo.
Homocysteine, Vitiligo, Folic Acid, Vitamin $B_{12.}$

P140

A case of vitiligo associated with Menieres disease

M.-J. Gwak[1], E.-J. Shin[1], J.-H. Choi[1], K.-H. Jeong[1], M. K. Shin[1], M.-H. Lee[1]
[1]Department of Dermatology, College of Medicine, Kyung Hee University, Seoul, Republic of Korea

Vitiligo is a pigmentary skin disorder characterized by chronic and progressive loss of melanocytes. Although the etiology of vitiligo is still unknown, several theories have been proposed to explain the pathogenesis of vitiligo including autoimmune, neural, self-destruction, oxidative stress, and genetic theories. Currently, the

 © 2014 John Wiley & Sons A/S. Published by John Wiley & Sons Ltd

most convincing of these theories invokes an interaction between genetic and unknown environmental factors, resulting in autoimmune melanocyte destruction.
Meniere's disease (MD) is a chronic multifactorial disorder, where combined environmental and genetic factors determine its development. MD is characterized by recurrent vertigo, fluctuating or progressive sensorineural hearing loss and tinnitus, and it is associated with an accumulation of endolymph in the inner ear (endolymphatic hydrops). Although its etiology is not known, genetic or epigenetic factors have a significant contribution. And recently many articles support the hypothesis that Meniere's disease is an autoimmune disorder and associated with immune-mediated disorder.
Loss of otic melanocytes may occur in patients with vitiligo and, evidences of sensorineural hearing loss in vitiligo patients have been reported over the last decade. However, there have been no reports of Meniere's disease associated with vitiligo patients.
A 15-year-old male presented with irregular depigmented maculopatches on the left pre-auricular, forehead, and lower cheek area about 3 months ago. He had been suffering from Meniere's disease from 3 yrs ago. Physical examination revealed localized well-demarcated irregular depigmented maculopatches on the left pre-auricular, forehead, and lower cheek area. We report an interesting case of vitiligo on the face with Meniere's disease.

P141
Confetti depigmentation as a disease marker in patients with generalized vitiligo

A. Pandya[1], J. J. Sosa[1], S. Currimbhoy[1]
[1]Dermatology, University of Texas Southwestern Medical Center, Dallas, TX, USA

Determination of vitiligo stability is important to determine prognosis and select the appropriate therapy for affected patients. To date, few studies have evaluated clinical patterns of depigmentation as prognostic markers.
We set out to determine if the presence of numerous, small, depigmented macules in a confetti-like pattern is a negative prognostic sign indicating rapidly progressing vitiligo in patients that are not undergoing treatment.
This was a retrospective, single center chart review of 178 patients. 15 patients with a confetti-like pattern of depigmentation were identified. Seven of these fit the inclusion criteria of at least one follow up image and lack of treatment. Photographs of 13 lesions at baseline and follow-up visits were assessed for percent depigmentation by three independent reviewers. Areas of involvement included the face, extremities, and acral sites. Individual lesions were scored using the percentage component of the Vitiligo Area Scoring Index (VASI).
The median time between baseline and follow-up images was 16 weeks. The mean percentage of depigmentation at baseline was 19% (±2) and increased to 51% (±6), a rise of 32% ($P < 0.0001$). Two patients had an additional follow-up visit at a median of 31 weeks showing mean depigmentation of 71% (±8) compared to baseline, an increase of 53% ($P < 0.0012$). A skin biopsy of a confetti lesion in one patient revealed an inflammatory infiltrate in the papillary dermis including CD 8+ T cells at the dermal-epidermal junction.
A confetti-like pattern of depigmentation may be a negative prognostic indicator for patients with rapidly progressing vitiligo. Patients identified with this pattern of depigmentation may require more aggressive treatment in order to stabilize their disease. Further, prospective studies evaluating this physical finding should be performed.

P142
Patient satisfaction with treatment modalities for vitiligo

A. Pandya[1], S. Currimbhoy[1], V. Zhu[1]
[1]Dermatology, University of Texas Southwestern Medical Center, Dallas, TX, USA

A wide variety of treatment modalities are used for patients with vitiligo. Current outcome measures typically focus on the repigmentation rate based on clinician evaluation. Recently it has been emphasized that patient satisfaction with treatment is an important outcome which is lacking in the great majority of vitiligo treatment studies. A literature review of vitiligo studies over the past 40 yrs demonstrated that only 7% of studies measure patient satisfaction with treatment. Gauging overall satisfaction with treatment has important implications on the physician-patient relationship, compliance with therapy, and development of novel treatment modalities, all of which in turn has an impact on patient quality of life.
The purpose of this study was to determine patient satisfaction with the most commonly used treatments for vitiligo: Topical corticosteroids, topical tacrolimus ointment and NBUVB phototherapy.
Patients with generalized and segmental vitiligo enrolled in the Dallas Vitiligo Registry were asked to assess their overall satisfaction with the treatments they had received, taking into account the treatment regimen, amount of repigmentation achieved, and any associated side effects. All responses were recorded using a visual analog scale completed by the patient.
There was a significant difference in overall patient satisfaction between the different therapies ($P < 0.05$, ANOVA analysis). Patients rated NBUVB phototherapy as the most satisfactory treatment, followed by topical corticosteroids and finally, topical tacrolimus. Sub-analysis comparing treatment satisfaction to disease duration and Fitzpatrick types (I–III versus IV–VI) revealed no statistical differences.
The results of this study suggest there is a difference in patient satisfaction between commonly used treatments for vitiligo. This information may be useful for dermatologists when selecting the best therapies for patients with vitiligo. Preliminary results indicate phototherapy to be the most satisfactory treatment for patients. Further, larger studies should be performed to confirm these findings.

P143
Epidemiology of vitiligo in Surat, GJ, India

D. Patel
MCI India

Epidemiology information of vitiligo was collected by home to home survey in Surat City. Gujarat. India. Overall less than 1% prevalence with Vitiligo. Females are more affected than males with vitiligo. Maximum cases of vitiligo are reported by people of age group between 15–45 yrs and associated with autoimmune disease and stressful life. In almost 40% of cases positive family history was found. New lesion develops at the site of trauma (Koebner Phenomenon). Higher number of cases are found with people living in hot and humid

© 2014 John Wiley & Sons A/S. Published by John Wiley & Sons Ltd

atmosphere. Early onset reported in segmental Vitiligo. Spontaneous improvement is reported in children. Diet does not have any role in Vitiligo surveillance.

P144

The relation between vit B12 levels and vitiligos repigmentation

M. Araujo[1], P. Avila[1], P. Avila[1], M. F. Araujo[2], P. F. Avila[3]
[1]Santhe Day Use; [2]Surgery, Santhe Day Use; [3]Dermatology, Santhe Day Use, Belo Horiznte, Brazil

Vitiligo is an acquired, idiopathic disorder caracterized by circunscribed depigmented macules. Functional melanocytes disappear from involved skin by a mechanism that has not yet been identified.Vitiligo affects approximaly 0,5–2% of the general population worldwide and it may appear anytime from shortly after birth to senescence.Most patients with vitiligo attribute the onset of their disease to specific life events(physical injury, sunburn, emotional injury, illness). With the exception of Koebner phenomen, there is no proof that these factors cause or precipitate vitiligo.Many are the treatments used for vitiligo. The aim of all of them is repigmentation and stability of vitiligo macules. Among them, we can present NB-UVB 311 nm, excimer 308 nm,psoralen plus phototherapy topically used, corticosteroids and topical immunossupressants.Some patients have some blood exams alter.Some recent articles have suggested a relation between vitB12 levels and vitiligo repigmentation. The aim of the present study is to investigate if there is, in fact, a relation between vit B12 levels and repigmentation in patients with vitiligo.Thirty three patients who were in treatment of vitiligo,in Belo Hriznte- Brazil- were monthly avaliated by one of the doctors of Santhe- Day use Hospital. These patients were using 308 nm excimer light or NB UVB 311 nm- depending n the extension of their lesions- to treat their vitiligo lesions and had their vitamin B12 levels dosed before the treatment started.
In none of them we found a relation between vitamin B12 levels (upper than 365) and better repigmentation.

P145

The effectiveness of 308-nm excimer laser for segmental vitiligo: retrospective study of 159 patients

J. M. Bae[1], H. Kim[2], C. Y. Won[1], J. H. Lee[1], G. M. Kim[1]
[1]Department of Dermatology, College of Medicine, The Catholic University of Korea; [2]Sosom Dermatologic Clinic, Seoul, Republic of Korea

Segmental vitiligo (SV) is characterized by a unilateral dermatomal distribution, earlier onset and stable disease after rapid progression, and often associated with poor response to various treatment modalities.
The aim of this study was to evaluate the effectiveness of the combination treatment with 308-nm excimer laser and 0.1% tacrolimus ointment for SV, and to search for factors associated with poor response to the treatment.
A retrospective chart and photographic review was performed on 159 SV patients (mean age: 24.5 ± 15.1, females: 58.5%) who had undergone the combination treatment for more than 3 months, from July 2008 to December 2012. Treatment success was defined as more than 75% repigmentation of whole lesions in each patient.
Overall success rate was 50.3% after 14.6 months of mean treatment duration; 13.8% and 36.5% of SV patients showed complete and nearly complete (more than 75%) repigmentation, respectively. The factors associated with poor response were adult patient, later-onset disease, disease duration of over 12 months, presence of poliosis, and plurisegmental subtype. When conducting multivariable analysis, we found the following to be independent factors with poor response: disease duration of over 12 months (OR 0.372, 95% CI 0.157–0.882, P = .018), poliosis (OR 0.175, 95% CI 0.065–0.474, P = .001), and plurisegmental subtype (OR 0.494, 95% CI 0.247–0.988, P = .046).
The combination treatment with 308-nm excimer laser and 0.1% tacrolimus ointment could be used as an effective treatment modality for SV. Disease duration of over 12 months, presence of poliosis, and plurisegmental subtype were confirmed to be independent prognostic factors of poor response for SV patients.

P146

Role of hair transplantation in vitiligo treatment

K. Chouhan[1], A. kumar[1]
[1]Dermatology, Dermaclinix, Delhi, India

Stable vitiligo has been treated with various surgical modalities including hair transplantation at vitiligo patch with variable success rates. Regardless of the mode of treatment, repigmentation in vitiligo usually begins in the perifollicular area highlighting the importance of melanocytes and stem cells present in hair follicles. Hair transplantation in vitiligo is based on the concept of existence of undifferentiated stem cells in the hair follicle, which forms a good source of melanocytes for repigmentation. These melanocytes when grafted, then spread to surrounding depigmented epidermis but the appearance of pigmentation is delayed when compared to other modalities and the colour match is much more acceptable. This can be done by FUT, FUE & Body hair transplant etc. In some cases due to phototoxicity scarring occurs in vitiligo patch with loss of hairs and it is difficult to achieve good repigmentation in these cases. Body hair transplantation helps in repigmentation by perifollicular spread of melanocyte & stem cells from the implanted follicles. In addition, the aesthetic appearance also is restored by the new hairs. This method is effective in focal vitiligo, vitiligo in non-glabrous areas and in those patches with leukotrichia.
FUT for repigmentation for vitiligo has been reported earlier.The diameter of pigment spread is 5–12 mm per hair grafted. Pigmentation starts appearing at ~6 week and continues upto 6 months or even longer. Even in cases of unresponsive or treatment-resistant vitiligo, grafted hairs retained the pigmentation. Transformation of depigmented hairs into pigmented hairs has been reported following Hair transplant. Noncultured extracted hair follicular outer root sheath (ORS) cell suspension transplantation has also been tried with 65.7% repigmentation. Body hair transplantation by follicular unit extraction (FUE) circumvents the need of tedious melanocyte suspension process.
The advantages of hair transplantation in vitiligo:

1. A single hair contains more melanocytes than normally pigmented glabrous, usually gluteal area skin. Hair follicle melanocytes also seem to be more resistant to the vitiligo process.
2. This method is advantageous for hair restoration in a non-glabrous area which is not possible with typical vitiligo surgery techniques.
3. Does not produce post-operative hyperpigmentation in the grafted sites as does autologous suction blister grafts.

© 2014 John Wiley & Sons A/S. Published by John Wiley & Sons Ltd

4. No need for dermabrasion of recipient area.
5. Cobblestone hypertrophic scar does not appear because small bored needle is used for implantation.

The advantages of body hair transplantation (by FUE) over FUT in vitiligo:

1. No visible scarring
2. Hairs does not require frequent trimming to match length with other hairs
3. Ready to implant Follicular units
4. Excellent aesthetic outcome as body hairs matches with surrounding hairs

However, this method has some limitations; body hair transplantation (BHT) by follicular unit extraction (FUE) is a time-consuming and delicate procedure requiringlots of expertise.
Body hair transplantation (BHT) by follicular unit extraction (FUE) appears to be an effective method for treating localized/ segmental vitiligo, especially on hairy parts of the skin, including the eyelids and eyebrows and for small areas of vitiligo. The best application of this method will be in vitiligo patches with leukotrichia. In cases of focal vitiligo with scarring/ loss of hairs, this procedure could be considered as procedure of choice.

P147

Devicing an intervention module to reduce psychosocial stress in patients with vitiligo

A. Jha[1]
[1]Dermatology and STD, VM Medical College and Safdarjung Hospital, New Delhi, India

Vitiligo is associated with enormous psychosocial burden. Vitiligo patients with psychosocial problems are likely to benefit from psychosocial interventions. In this study, we have attempted to develop an intervention module to relieve the psychosocial stress in vitiligo patients and tried to assess the feasibility of the developed module in general dermatology practice.
In this study, we developed an intervention module based on cognitive behaviour therapy to relieve the psychosocial stress in patients with vitiligo. Our module consisted of psycho-education, self-statements, relaxation/imagery and exposure/ desensitization. Thirteen patients, more than 12 yrs age, who presented with psychosocial distress and were willing to undertake cognitive behavior therapy were recruited in the study. They were asked to fill DLQI and Skindex-16 at baseline and a behavioural assessment of these patients was also done. Baseline clinical photographs were also taken. After that each patient was given five sessions of cognitive behavior therapy at weekly intervals for 5 weeks on weekends or as preferred by the patient. During the therapy, patients maintained their daily mood charts. At the end of 5 weeks and 7 weeks after the end of the therapy, DLQI, Skindex-16 and behavioural assessment was repeated. Clinical photographs of the lesions were also taken at the end of five sessions.
Eight patients who completed the full course of cognitive behavior therapy felt more relaxed, more confident and were less bothered about the patches. They were able to cope with feared situations lot better than before and most of them showed a continuous improvement in the mood charts during the psychosocial intervention. There was a significant reduction in the mean DLQI scores by 3.8 points at the end of five sittings as compared to baseline (2.8 versus 6.6). The reduction in Skindex-16 score was by 16 points (26 versus 42). Most of them had no change in the pigmentation of their skin lesions which indicates that the improvement in quality of life after cognitive behavior therapy occurred irrespective of the repigmentation status. The improvement in the DLQI and Skindex-16 scores in our patients were maintained at the follow up visit 7 weeks later. Thirty eight percent of our patients did not complete the psychotherapy session stating time constraints, inability to follow up every week and inappropriateness of therapy as the reasons for their noncompliance. There was no difference in the age, sex, body surface area or disease duration between the patients who completed the treatment as compared to those who did not complete their treatment.
We developed an intervention module based on cognitive behaviour therapy which was provided to 13 vitiligo patients with moderate and severe psychosocial distress. Out of these, eight patients completed the full course of cognitive behavior therapy, the remaining stating time constraints, inability to follow up every week and inappropriateness of therapy as the reasons for their noncompliance. Those who completed the sessions were more motivated from the beginning than those who did not complete the sessions.
We recommend that patients with vitiligo should be given information about the disease. Those having greater degrees of distress should be identified and these patients may be provided cognitive behaviour therapy if they are motivated and willing to attend therapy sessions regularly.

P148

Comparison of efficacy and safety profile of topical calcipotriol ointment in combination with NB-UVB versus NB-UVB alone in the treatment of vitiligo: a 24-week prospective right-left comparative clinical trial

G. Khullar[1], A. J. Kanwar[1], S. Singh[2], D. Parsad[1]
[1]Dermatology, PGIMER, Chandigarh; [2]Dermatology, AIIMS, New Delhi, India

Vitiligo is a pigmentary disorder involving destruction of melanocytes secondary to interplay of factors like genetic predisposition, autoimmunity, oxidative stress, altered calcium homeostasis and melanocytorrhagy. Narrow-band UVB (NB-UVB) is an efficacious and safe treatment option in patients with generalized vitiligo. Calcipotriol, a synthetic vitamin D3 analogue has been tried as monotherapy and in combination with phototherapy with equivocal results. We undertook this study to compare the efficacy and tolerability of topical calcipotriol (0.005%) in combination with NB-UVB versus NB-UVB alone in generalized vitiligo.
A prospective right-left comparative clinical trial including twenty-five patients of generalized vitiligo was conducted for a total duration of 24 weeks. Bilaterally symmetrical lesions, one on each side of the body were selected as reference lesions. On one side selected randomly, 0.005% calcipotriol ointment was applied twice a day, while the other side served as an intra-individual control in each patient. Both sides were exposed to NB-UVB therapy thrice a week on alternate days. Calcipotriol was applied two hours after exposure to NB-UVB. The response to treatment was assessed using change in Lund and Browder (L&B) score for percentage reduction in body surface area involved, investigator's global assessment (IGA) and patient's global assessment (PGA) of the extent of repigmentation. Mean

© 2014 John Wiley & Sons A/S. Published by John Wiley & Sons Ltd

was calculated for all quantitative variables and for measures of dispersion standard deviation and standard error were calculated. Proportions were compared using Chi square test. All statistical tests were two-sided and P value <0.05 was considered statistically significant.

The mean percentage reduction in L & B score at 24 weeks was 51.39 ± 28.10 on NB-UVB alone and 49.00 ± 24.47on NB-UVB and calcipotriol side (P = 0.557). The mean IGA score on NB-UVB side was 2.68 ± 0.47and on NB-UVB and calcipotriol side was 2.64 ± 0.42 (P = 0.821). The mean PGA score on NB-UVB side was 5.64 ± 3.36 and on NB-UVB and calcipotriol side was 5.76 ± 3.20 (P = 0.706). Perifollicular repigmentation that matched with the surrounding normal skin was observed on both sides in majority of the patients. NB-UVB was well tolerated. Topical calcipotriol produced mild local adverse effects like erythema, xerosis and folliculitis. There were no abnormalities in serum and urinary calcium and phosphorus levels.

NB-UVB therapy alone is effective in patients of generalized vitiligo. However, the addition of topical calcipotriol to NB-UVB does not enhance the efficacy of treatment in terms of extent of repigmentation and time to initiation of repigmentation. Minor side-effects limited to application site were observed with calcipotriol.

P149

Depigmentation therapy for normal skin in vitiligo universalis with topical application of tacalcitol and 4-N-Butylresorcinol

T. Maekawa[1], E. Maehara[2], N. Wada[2], H. Takamatsu[2], K. Harada[2], K. Urabe[2]

[1]Dermatology, Hiroshima Red Cross Hospital, Hiroshima; [2]Dermatology, Kyushu Medical Center, Fukuoka, Japan

Vitiligo is a common depigmentation disorder occurring in about 1% of the population worldwide. The disease causes the appearance of white patches on the skin and white patches rarely spread to most of the body. If vitigo involves most of the body, it might be easier to depigment the normal remaining skin rather than to attempt repigmentation. For depigmentation therapy, monobenzyl ether of hydroquinone (MBEH) remains the only drug that the Food and Drug Administration approved for depigmentation therapy in the United States. However, in Japan, MBEH ointment is not sold, and is not available in general. Therefore, we used 0.002% tacalcitol ointment (Bonalfa High, Teijin Parma Ltd, Japan) and 0.3% 4-n-butylresorcinol lotion (Rucinol, POLA, Japan). Topical application of vitamin D3 analogue was reported to be effective for improving the pigmentation of café au lait spot. And 0.3% 4-n-butylresorcinol is used in the clinical treatment of liver spot.

In order to examine the depigmenting effect of 0.002% tacalcitol ointment and 0.3% 4-n-Butylresorcinol lotion, we treated two patients of vitiligo universalis.

Treatment of two patients was done with 0.002% tacalcitol ointment and 0.3% 4-n-Butylresorcinol lotion. These drugs were topically applied once a day on the normal remaining skin of the patient of vitiligo universalis.

Depigmentation was obtained after one months in one patient, and almost complete bleaching was achieved in eight months. However, in the second patient, no effect was observed after eight months.

Two patients of vitiligo universalis were treated with topical tacalcitol and 4-n-butylresorcinol. Good result was obtained in one patient. These drugs may be one of candidates for depigmenting normal remaining skin of patients of vitiligo universalis.

P150

Comparion of the 308 excimer lamp versus home phototherapy in the treatment of focal vitiligo vulgaris

S. T. G. Thng[1], A. Chang[2]

[1]Medical, National Skin Centre, National University of Singapore; [2]Medical, National University of Singapore, Singapore City, Singapore

Vitiligo is a common idiopathic acquired or inherited disease with loss of normal melanin pigments and functioning melanocytes from otherwise healthy looking skin. Phototherapy, specifically NBUVB, is the treatment of choice for vitiligo with more than 50% of patients achieving good repigmentation. Phototherapy however, is time consuming as it needs to be carried out mostly in an institution or treatment facility. Home based UVB phototherapy was first introduced over 20 yrs ago for the treatment of psoriasis. While multiple case series have shown that home based UVB treatment, in a carefully selected population, yield as good a result as clinic based phototherapy for the treatment of psoriasis, there been no proper studies done to determine the effectiveness as well as safety of home based phototherapy for localized vitiligo. To our knowledge, we report a first randomized, parallel group trial to compare home based phototherapy system excimer lamp for patients with localized vitiligo.

Patients with focal vitiligo (defined as non segmental vitiligo involving less than 10% body surface area) were recruited over 1 yr from the vitiligo clinic in National Skin Centre, Singapore. Recruited patients were then randomized into two groups, with one group using home phototherapy three times per week while the other group underwent exicmer lamp treatment in National Skin Centre two times per week. All patients were assessed at recruitment and subsequently after every 12 weeks with photographs taken on each occasion of follow up. At the end of the study, the percentage of repigmented areas was examined using photographs, and were graded in the following manner: Worsening of Vitiligo (−1), No change (0), Slight repigmentation (less than 50% repigmentation) (i), Good repigmentation (more than 50% repigmentation but less than 75%) (ii), Excellent repigmentation (more than 75% repigmentation but less than complete repigmentation) (iii) and Fully repigmentated. (iv).

Forty-four patients were recruited for this study. Of these, forty completed the study with three patients from excimer lamp group defaulting treatment due to logistical reasons. One patient from excimer group had to be switched to NBUVB whole body phototherapy as he developed worsening of vitiligo with new lesions developing on other parts of body. Both excimer lamp and home based phototherapy produced significant improvement in patients with focal vitiligo vulgaris. All in all 77% of patients in home phototherapy group achieved some repigmentation as compared to 75% of patients in excimer lamp group. There is no statistical difference in rate of response in the two groups at 9 months of treatment. Looking at the rates of repigmentation for each subgroup, in the home phototherapy group, 69% and 61% of this group had more than 50% and 75% repigmentation respectively. One patient had complete repigmentation at 6 months. For the excimer lamp group, 54% and 36% had more than 50% and 75% repigmentation

 © 2014 John Wiley & Sons A/S. Published by John Wiley & Sons Ltd

respectively. One patient had complete repigmentation too. This is much less than those seen in the home phototherapy group and is statistically significant.
We have demonstrated that with a proper training program in place and with careful selection of patients, home based phototherapy can be as, if not more, effective than institutional based treatment options. Other than its efficacy,home based phototherapy is an attractive option too for patient as it saves patient time and money, freeing up patients' productive hour for work and not tied to treatment process.

Vitiligo Surgery

P151

Efficacy of Er:YAG laser, NB-UVB and topical corticosteroids in resistant vitiligo patches

M. Abdallah[1], H. Diab[1], N. N. El-Din[2]
[1]Dermatology & Venereology, Ain Shams University; [2]Dermatology & Venereology, Mattareya Teaching Hospital, Cairo, Egypt

Despite the efficacy of phototherapy in non-segmental vitiligo, some lesions do not repigment. Few studies evaluated the efficacy of epidermal ablation for vitiligo lesions in combination with conventional therapy.
Comparing the efficacy of combining ablative Erbium: YAG (Er: YAG) laser followed by narrowband ultraviolet B (NB-UVB) and/or topical corticosteroids (TCS) in recalcitrant vitiligo patches.
Six different patches in 15 patients were treated with six different combination modalities. Er:YAG was first done in patches 1, 2, 3 and 4 followed by TCS (betamethasone dipropionate 0.05% ointment) + NB-UVB, NB-UVB, TCS and Er: YAG only respectively. In patches 5 and 6, TCS and NB-UVB respectively were applied without previous laser ablation. The patients were their own control. Repigmentation was evaluated by digital image analysis of scanned transparent sheets and subjectively by two blinded observers at week 12 post-laser.
Four males and 11 females with a median age 34 yrs completed the study. Patches pretreated with Er:YAG (1–4) showed statistically significant better repigmentation response compared to those receiving TCS and NB-UVB (5&6) ($P = 0.008$). Four patches (30.8%) in group1, 3 (23.1%) in group 2, 3 (20%) in group 3 and 1 (8.3%) in group 4 showed more than 50% repigmentation. However, the difference between patches 1, 2, 3 & 4 was not significant ($P > 0.05$). Er: YAG induced Koebner phenomenon in patients with disease stability shorter than one year. Delayed healing was observed in patients receiving TCS after Er:YAG laser.
Ablative Er:YAG laser followed by NB-UVB and/or topical corticosteroids is a helpful treatment modality in stable, resistant vitiligo.

P152

Partial thickness epidermal cuts versus suction blister roofs as sources of preparing non-cultured epidermal suspension for vitiligo treatment: a comparative study

T. Anbar[1], T. El-Ammawy[1], Y. El-Sherbeny[2], E. Saad[3], S. Salah[1]
[1]Dermatology Department; [2]Department of Clinical Patholoy, Al-Minya University, Al-Minya; [3]Dermatology Department, Matay Central Hospital, Matay, Egypt

Vitiligo is an idiopathic acquired de-pigmentation disorder that usually occurs among young people. Its incidence is 1–2% worldwide without sex or skin colour predilection.
For years, treatment of vitiligo was thought to be unsatisfactory and patients were advised to seek effective cosmetic camouflage for the lesions on exposed skin. Treatment modalities included potent topical steroids, topical or systemic psoralens with UV light exposure. grafting techniques, minigrafts and autologous cultured melanocytes had been used with some success.
Non cultured epidermal suspension transplantation is a well established surgical treatment for stable vitiligo not responding to either medical treatment or phototherapy. This suspension is traditionally prepared from partial thickness epidermal cuts taken by thiersch knives or a dermatome.
In this work, we introduced the using of suction blister roof as a source of this suspension and we compared it with that obtained by the traditional method i.e. using thiersch knife. The comparison entailed the treatment outcome on recipient vitiligo lesions.
Twenty patients with localized lesions of stable vitiligo were randomized into two groups each of them was treated with non-cultured epidermal suspension transplantation. In Group 1(10 cases) the suspension was obtained from partial thickness epidermal cuts while in group 2(10 cases) it was obtained from suction blister roofs.
Repigmentation was complete (100%) in two cases, excellent (90–99%) in seven cases and no response in one case in group 1, while in group 2 it was complete in 2 cases, excellent in 5cases, very good (76–90%) in 2 cases with no response in one case.
Repigmentation results are comparable by both techniques with some pros and cons of each technique.

P153

Non cultured epidermal cell suspension for stable vitiligo from lab to dermatologists clinic: an oversimplification or a real possibility

A. Budania[1], N. Khunger[1]
[1]Dermatology, VMMC and Safdarjung Hospital, New Delhi, India

Surgical management of vitiligo is becoming a cost effective and highly efficacious tool in a dermatologist's armamentarium. Cellular transplantation methods have been found to have an edge over tissue transplantation methods of vitiligo surgery. Most recent developments have been observed in non cultured epidermal cell suspension (NCES), which is the most common cellular transplantation method. Few years ago, NCES was a tedious and very expensive method because of the requirement of a well furnished lab with incubator, melanocyte media, trypsin inhibitor, manual preparation of trypsin-EDTA solution, laminar flow chambers and high end centrifuge machines. We carried out this study to overcome all these difficulties and bringing NCES from lab to a dermatologist's clinic.
A total of 25 patients with 38 stable vitiligo lesions were selected. A split thickness skin graft was harvested from lateral side of the thigh. Graft was kept overnight in lower compartment of

© 2014 John Wiley & Sons A/S. Published by John Wiley & Sons Ltd

refrigerator for about 15 h in readymade solution of 0.25% trypsin and 0.02% EDTA. Next morning, epidermal cells were separated from the tissue graft and cellular suspension was made in phosphate buffered saline. We did not use trypsin inhibitor and melanocyte media. Then, the suspension was applied to dermabraded skin and dressing was kept for 1 week.

Patients were evaluated 16 weeks post surgery for the extent of repigmentation, pattern of repigmentation, colour match, change in Dermatology life quality index (DLQI) and patient satisfaction. The statistical analysis was carried out using Statistical Package for Social Sciences (SPSS Inc., Chicago, IL, version 17 for windows). Means were compared using student's *t*-test for outcome. Qualitative data were compared using Chi square or Fisher's exact test.

Thirty out of the 38 stable vitiligo (78.94%) lesions, showed >90% extent of repigmentation that was considered to be excellent. DLQI reduced from 12.41 before surgery to 2.32 after surgery. All the patients were highly satisfied and ready for the repeat procedure. Most common pattern of repigmentation was diffuse, that was observed in 96.7% lesions. Color match to the surrounding skin was observed in 97% of the lesions.

Apart from the storage of trypsin-EDTA solution in deep freezer at −20°C, all other requirements for NCES could be met at dermatologist's clinic. To conclude, we used the most basic, simple and economic yet efficacious methods of NCES. We don't recommend the oversimplification in the form of a kit containing all the ingredients for preparation of NCES because the trypsin-EDTA solution may lose its efficacy within 2 weeks if not stored at −20°C. So NCES at Dermatologist's office is a real possibility with few limitations.

P154

Vitiligo surgeries - modifications to simplify the technique

D. H. Ghia[1,2], S. Mulekar[1]

[1]Mulekar Vitiligo Clinic; [2]All Doctors Cliinic, Mumbai, India

Different vitiligo surgeries are performed. Often the choice for the vitiligo surgery is restricted by the facilities available at a centre.

To study the different modifications in vitiligo surgeries which make it technically simpler to perform or more cost effective.

In this study we discuss each of the standard methods of vitiligo surgery like minipunch grafting, split thickness skin grafting, suction blister grafting, smash grafting and non cultured epidermal cell suspension along with different modifications which make these procedures simpler or economically viable so as to reach out to a larger number of vitiligo patients. Minipunch grafting is modified by taking 1 mm punches which improve the cosmetic results, for hairy areas affected a hairy graft is taken which improves the results as both skin and hair melanocytes are grafted. Flip top grafting modification of skin grafting eliminates the use of biological dressings for split thickness skin grafting. Use of syringes with three ways for formation of blister, use of warmth, saline injections and adequate negative pressures in suction blister grafting makes this technique easy to perform. Using different types of smash grafts, makes vitiligo surgery easy to perform at even primary healthcare centers. Modifications in noncultured epidermal cell suspension using cold trypsinisation, hyaluronic acid derivates to make a viscous suspension, using four compartment technique eliminates requirement for centrifuge machine, phosphate buffered saline is used in place of Trypsin Inhibitor and Dubelco's Modified Eagles Medium (DMEM), and a blade with a long artery forceps can be used instead of a special grafting knife. These modifications simplify the surgery.

All patients of stable vitiligo not responding to medical line of therapy should get the option of surgical modality irrespective of place and economic status.

P155

Uncommon responses of segmental vitiligo to melanocyte keratinocyte transplantation procedure

D. H. Ghia[1], S. Mulekar[1,2,3]

[1]Mulekar Vitiligo Clinic, Mumbai, India; [2]National Center for Vitiligo & Psoriasis, Riyadh, Saudi Arabia; [3]Department of Dermatology, Multicultural Dermatology Center, Henry Ford Hospital, Detroit, MI, USA

Treatment of Segmental Vitiligo (SV) consists of topical therapies and phototherapy. If medical treatment is unsatisfactory, surgical treatment should be considered. The surgical therapies can either result in complete or partial re-pigmentation, which might in some instances warrant repeat transplant surgery.

Approximately 85% of patients with SV show greater than 95% of repigmentation as compared to Non-segmental Vitiligo (NSV) where ~55% patients show similar results when treated with melanocyte-keratinocyte transplant procedure (MKTP).

Thus, SV patients are ideal candidates for surgical treatment.

Case StudyIn this study we discuss 10 rare patients of SV who had a varied response to MKTP a deviation from the normal predictable outcome.

Case 1: Complete failure of repigmentation with MKTP and successful repigmentation with split thickness skin graft (STSG).

Case 2: Partial repigmentation with MKTP and successful repigmentation in the remaining area with STSG.

Case 3: Complete repigmentation with repeat MKTP

Cases 4 and 5: Koebnerization of donor or recipient site

Cases 6, 7 and 8: New patches in previously unaffected area, but located in the same segment

Case 9 and 10: Hypopigmented ring at margin of MKTP treated areas

It is difficult to fully explain our observations reported in these SV patients based on present concepts of pathogenesis. With further basic science and clinical research studies, these questions can one day be answered to provide better outcomes for vitiligo patients undergoing the melanocyte-keratinocyte transplant procedure.

P156

Clinical and treatment characteristics determining therapeutic outcome in patients undergoing autologous non cultured outer root sheath hair follicle cell suspension for treatment of stable vitiligo

V. Keshavamurthy[1], S. Dogra[1], D. Parsad[1]

[1]Dermatology, Venereology and Leprology, Post Graduate Institute of Medical Education and Research, Chandigarh, India

Autologous non cultured outer root sheath hair follicle cell suspension (NCORSHFS) is a recently described novel cellular graft technique for the treatment of stable vitiligo. There is lack of data about various factors determining the repigmentation rate in vitiligo patients undergoing this novel surgical therapy.

 © 2014 John Wiley & Sons A/S. Published by John Wiley & Sons Ltd

To study the clinical characteristics and treatment variables determining therapeutic outcome in patients of stable vitiligo undergoing NCORSHFS.
This was an open labelled prospective study in a tertiary care centre. Patients of stable vitiligo, defined as no fresh lesions and previous lesions not increasing in size for the last 12 months were recruited. The exclusion criteria were actively spreading disease, keloidal tendency, vitiligo patch of >100 cm^2 and patients with unrealistic expectations. NCORSHFS was prepared from anagen hairs extracted from the occipital area. The number of melanocytes and hair follicle stem cells (HFSC) in the suspension was quantified by staining with anti-HMB45 and anti-CD200 antibody respectively. In all patients, a 2 mm punch skin biopsy was taken from one of the vitiligo patch to be treated prior to surgery for assessment of histomorphological features. Post surgery patients were followed up at regular intervals for 24 weeks.
Thirty patients (21 females, 9 males), with a total of 60 target lesions were included in this study. The mean age of the study population was 21.10 ± 5.64 yrs). In the study population, vitiligo was present for a mean duration of 6.90 ± 3.92 yrs and the stability period was 3.62 ± 2.37 yrs. Seventeen patients had focal vitiligo, 11 patients had generalized vitiligo and 2 patients had segmental vitiligo. The lesions treated surgically in this study were widely distributed all over the body. Thirty one (51.67%) lesions were present over proximal extremities (leg, thigh and arm), 18 (30%) lesions over acral areas (feet and hand), 8 (13.33%) over head and neck area and 3 (5%) over trunk.
The number of melanocytes transplanted ranged from 96 to 2747 cells/cm^2 and the number of HFSC transplanted ranged from 81 to 1442 cells/cm^2. Optimum repigmentation (RP > 75%) was seen in 21 out of 60 (35%) lesions. Ten of these 21 lesions had achieved excellent repigmentation defined as repigmentation rate of more than 90% (RP > 90%).There was no statistically significant difference in the repigmentation rate among the different body sites ($P = 0.146$) or different types of vitiligo ($P = 0.21$).The number of melanocytes ($P = 0.04$) and HFSC ($P = 0.01$) transplanted was significantly higher among patients achieving optimum repigmentation (>75% repigmentation). There was a strong correlation between repigmentation at 24 week and number of melanocytes and HFSC transplanted. Number of HFSC transplanted and absence of dermal inflammation were significant predictors of achieving optimum repigmentation.
The number of melanocytes and HFSC transplanted and absence of dermal inflammation were important determents of optimal repigmentation in patients undergoing NCORSHFS for treatment of stable vitiligo. Hence refining the technique of NCORSHFS on the basis of these factors would help in achieving better surgical outcomes.

P157

Autologous cell suspension transplantation using recell in segmental vitiligo and piebaldism patients: a randomised controlled pilot study

L. Komen[1], C. Vrijman[1], R. M. Luiten[1], E. P. Tjin[1], M. A. de Rie[1,2], W. J. van der Veen[1,3], A. Wolkerstorfer[1]

[1]Department of Dermatology, Netherlands Institute for Pigment Disorders SNIP; [2]Department of Dermatology, Academic Medical Centre, Amsterdam; [3]Department of Dermatology, MC Haaglanden, Den Haag, The Netherlands

Segmental vitiligo and piebaldism are skin disorders causing depigmented macules that can impair a patient's quality of life. In stable segmental vitiligo and piebaldism depigmented macules can be repigmented by autologous cell suspension transplantation (CST). Until now, specialised laboratories were necessary to prepare the suspension for CST. Recently, the ReCell portable cell harvesting device came available, which obviates the need for specialised laboratories. The aim of our study was to evaluate the effectiveness, safety, and patients' satisfaction of CST with the ReCell method in vitiligo patients. Furthermore, analyses were done on cell type and viability of the cell suspension.
10 stable segmental vitiligo and piebaldism patients were included in this randomised intra-patient controlled pilot study. In every patient, three depigmented regions on the trunk or proximal extremities were randomly allocated to; CO2 laser abrasion +ReCell treatment, CO2 laser abrasion, or no treatment. The percentage of repigmentation, side effects and patient satisfaction were assessed after 6 months. The superfluous cell suspension was analyzed for total cell count and viability, and the melanocyte content and viability was determined in each graft by flow cytometry.
showed a median repigmentation of 78%, 0%, and 0% in the treated ReCell, laser and control group, respectively. There was a significant difference between the lesions treated with ReCell and the lesions treated with laser only and the control site. Six patients recommended the ReCell treatment. Erythema, and hyper- and hypopigmentation was seen in 30% and 40% of the patients, respectively. No adverse effects, such as scars or infections were seen. There was a significant correlation between the percentage of repigmentation of the CST-CHD site and total cell count ($P = 0.04$, correlation coefficient 0.648) and the percentage of repigmentation and the percentage of viable melanocytes ($P = 0.04$, correlation 0.811).
CST with the ReCell technique is an effective and well tolerated treatment for stable vitiligo and piebaldism patients.

P158

Is lesional stability more important than disease stability for performing grafting procedures in vitiligo? Results from a multi-centric study

I. Majid[1], V. mysore[2], T. Salim[3], K. lahiri[4], S. talwar[5], N. khunger[6], S. burua[7], S. Y. N[8]

[1]Cutis Skin Institute, Srinagar; [2]Venkat Charmalaya – Centre for Advanced Dermatology, Bangalore; [3]Cutis Centre for Advanced Dermatology, Calicut; [4]Wizderm Speciality Skin Clinic, Kolkatta; [5]Talwar Skin Centre, Lucknow; [6]Dermatology, Safdarjung Hospital, Delhi; [7]Dermatology, Assam Medical College, Dibrugarh; [8]Dermatology, Bangalore Medical College, Bangalore, India

Stability of the disease process is the prime requisite for undertaking any surgical intervention in vitiligo. However, there is no consensus regarding the minimum duration of stability for vitiligo surgery and different authors recommend different durations of disease stability ranging from 6 months to 3 yrs. There is also no consensus whether it is the lesional stability or the overall disease stability in vitiligo that needs to be considered while selecting a patient for surgical treatment.
This multi-centric study was aimed to assess the effect and relative importance of lesional and patient stability on the cosmetic results obtained in vitiligo grafting.
Patients enrolled into the study were divided into four groups on the basis of their disease and lesional stability. Group A patients included those in whom the disease stability was in the range of 6–12 months while the lesion selected for grafting was stable for

© 2014 John Wiley & Sons A/S. Published by John Wiley & Sons Ltd

1–2 yrs and Group B included patients with similar disease stability but with a lesional stability of >2 yrs. Similarly patients in Group C had disease as well as lesional stability of 1–2 yrs while in Group D, patients had an overall disease stability of >2 yrs. All the enrolled patients underwent any of the tissue or cellular grafting procedures and the repigmentation and the cosmetic results achieved were assessed and compared between the different groups. Response was graded as excellent (>90% repigmentation), fair (50–90% repigmentation) and poor (<50% repigmentation).
A total of 124 vitiligo patients were enrolled who underwent any of the tissue or cellular grafting procedures. Among these excellent results in the form of >90% repigmentation were obtained in 49 (39.5%) patients. While in Group A, 13 out of 35 patients (37.1%) achieved excellent results, the corresponding percentage in Group B, C and D was 38.1% (8/21), 36.4% (8/22) and 43.5% (20/46) respectively. Patients achieving fair and poor results were also distributed almost equally among the groups. Statistical analysis of the results obtained revealed no significant difference in repigmentation response among the four study groups. Response obtained at the recipient site was also correlated with the site of the grafted lesion as well as the grafting procedure performed. While face and neck showed the best response to vitiligo grafting, acral areas were seen to respond the worst. Among the different grafting procedures performed, split-thickness skin grafting (STSG) and ultra-thin skin grafting (UTSG) showed the best repigmentation response followed by Non-culture epidermal cell suspension (NCES) technique.
Lesional stability seems to be more relevant than the overall disease stability for selecting a patient for surgical intervention in vitiligo.

P159

Ultra-thin skin grafting in resistant stable vitiligo: factors affecting the treatment outcome

I. Majid[1]
[1]Cutis Skin Institute Srinagar Kashmir, Srinagar, Kashmir, India

Resistant vitiligo that does not respond to medical treatments can be managed by any of the grafting techniques. Ultra-thin skin grafting is one of the tissue grafting techniques with good cosmetic results in patients with vitiligo.
The study aims to assess different factors that affect the cosmetic response after ultra-thin skin grafting in vitiligo.
Over a period of 6-years, 180 patients with stable resistant vitiligo were treated at our institute with ultra-thin skin grafting over different areas of the body. The results obtained in these patients were analyzed retrospectively and an effort was made to assess the overall cosmetic response at the recipient as well as donor sites. In addition, the effect of site, size, morphological type and duration of stability of vitiligo, post-procedure treatment followed along with age and sex of the treated patient on the final results achieved was also assessed.
Results Of the 180 patients who underwent ultrathin skin grafting, excellent results with >90% repigmentation were achieved in ~61% (109/180) patients. The cosmetic acceptability of the repigmentation achieved was also excellent in all these patients. Poor results with minimal (<25%) repigmentation was seen in 9% (16/180) patients. Commonest adverse effect noted was ‘perigraft halo of depigmentation’ in 21 patients. The most important factor that correlated with the final results was the site of vitiligo with face and neck area showing the best response and acral areas exhibiting the least response to UTSG. In addition, a positive correlation of the repigmentation achieved was seen with the morphological type of vitiligo (segmental type showing the best response) and post-treatment NBUVB treatment. Age and sex of the patient were not seen to influence the treatment outcome.
Ultra-thin skin grafting provides excellent cosmetic results in stable resistant vitiligo and the cosmetic results obtained have a direct correlation with the site and morphological type of vitiligo as well as with post-treatment NBUVB phototherapy.

P160

Smash grafting as an alternative to non-culture epidermal cell suspension (NCES) technique in resource poor settings

Dr I. Majid[1]
[1]CUTIS Institute of Dermatology, Srinagar, Kashmir, India

Non-culture Epidermal cell suspension (NCES) technique is currently the most favoured cellular grafting technique employed in therapy resistant vitiligo. However, the procedure needs costly reagents and laboratory support which is not always possible in resource poor settings.
We aim to propose ‘smash grafting’ as an alternative to NCES technique in resource poor settings where cost of the procedure and laboratory support are the limiting factors.
Over a span of 1-year, 30 patients with stable vitiligo resistant to medical treatment were treated with one or more sessions of smash grafting on different parts of the body. An ultra-thin skin graft, one-fifth to one-tenth of the size of the recipient area to be treated, was taken from the anterior thigh and smashed into a uniform paste with the help of scissors or surgical blade. The ‘smashed’ graft was then applied on the dermabraded recipient skin and covered with collagen dressings or any other suitable dressing material. Post-operatively the patients were given supplementary medical treatment in the form of NBUVB or topical psoralens in diluted form with sun exposure.
All the 30 patients were available for follow up and responded partially or completely to the grafting procedure. Majority of the patients (22/30) showed excellent results in the form of >90% repigmentation on the recipient area while repigmentation was partial in the other eight patients. Perigraft halo (8/30) was the commonest adverse effect seen followed by non-uniform pigmentation (4/30). The commonest technical difficulty in performing the procedure was to achieve a ‘uniform’ spread of the smashed graft. However, large areas of the body could be treated with multiple sessions of the procedure.
Smash-grafting provides a cheap and easy alternative to NCES technique in resource poor settings. Larger areas of vitiligo can be treated with this procedure in single or multiple sessions.

P161

Treatment in segmental vitiligo with autologous skin micrografting

A. A. J. Merli[1], E. serra[1], V. Montenegro[1]
[1]Medical Group, Rosario, Argentina

Vitiligo is a pigmentary disorder manifested by achromic macules in the skin and mucous membranes as a result of the progressive destruction of the epidermal and follicular melanocytes.
Although the clinical course and treatment are a matter of discussion, we can recognize two clinical forms of vitiligo:

 © 2014 John Wiley & Sons A/S. Published by John Wiley & Sons Ltd

segmental and non-segmental. They respond differently to each therapy and are associated with different causal factors.

According to the European Dermatology consensus forum, the clinic segmental form is presented following a unilateral distribution that may affect a skin segment partially or totally.

Repigmentation process can be achieved by using different therapeutic alternatives, such as: corticosteroids, phototherapy, immunomodulators, surgical procedures, or a combination of other treatments, between others.

Repigmentation from the micrografting technique of total skin.

A 35 yrs old female patient, diagnosed with segmental vitiligo covering the suprapubic region, groin, inner thigh and inner knee anterior face following a linear path. Iniciated 15 yrs ago and stable for the last 6 months. It presents, according to Fitzpatrick classification, a skin phototype IV without leucotrychia. Previous treatments experience: local and systemic steroids, melagenine, pimecrolimus with no response. Laboratory tests with no specificities. The patient has no history of cicatrization disorders. She does not relate family history of vitiligo.

Surgical TechniqueDonor site: retroauricular, repaired with minimal scarring and as being hidden.

Procedure: antisepsis and local anesthesia. Then, it is preceded with 1.5 mm punch to take grafts of the total skin, close one to each other.

Receiving area: achromians skin area.

Procedure: antisepsis and local anesthesia. Every 5 mm distance it is preceded to the extraction of total skin achromians with 1.2 mm punch. After completing this procedure, adhesive membrane is applied. Checks every 48 h and replacement of bandage are performed according to evolution. Once obtained the cicatrization of the implants, treatment continues with sessions of narrow band phototherapy with a frequency of twice a week, starting with a dose of 0.20 joules at baseline, and with progressive increases as a minimal erythema dose.

Before applying the technique, the history of hypertrophic or keloid scars must be considered. Achromic stable lesions should be over six months.

It is important to provide a proper explanation of the techniques as well as the advantages and disadvantages. The monitoring and control of the changes occurring in the skin is very important. By controlling the evolution, perilesional progressive pigmentation can be seen, extending in circular shape around it. Over the months, the lesions show coalescence or remain unpigmented areas; however, the Dermoscopy still shows some pigmentary projections.

The stability of the pigment retention of implanted grafts should be noted. Although irregular pigmentation intensity is expressed, the evolution time tends to align, and the patient finds it more acceptable than the total absence of color.

Technique with little complexity, effective, reliable and a valid technique for use, due to its stability in response.

1. Taieb. A, Alomar, A., Bohm, M., Dell ‘Anna, ML, et al. *Guidelines for the management of vitiligo: the European Dermatology Forum consensus*. B. J. of D. 2013; 168, pp 5–19.
2. Falabella R.*Surgical treatment of vitiligo. Why, when and how?*.J. Eur. Acad. Derm. V. 2003; 17:518–20.

P162

Innovative autologous non-cultured epidermal cellular graft for segmental and non-segmental vitiligo

M. Pascal[1], H. Maillard[2], M. Ghasemi[3], R. Ghasemi[3], K. Ezzedine[4]

[1]Pigmentation Disorders Research Center; [2]Department of Dermatology General Hospital; [3]UPMC, PARIS; [4]University of Bordeaux National Reference Center for rare skin diseases, Bordeaux, France

Stable vitiligo unresponsive to conventional medical treatment are indicated for surgical intervention such as punch graft, suction blister graft, split-thickness skin graft, cultured melanocytes or non-cultured epidermal cell (NCEC) transplantation. However, to date, there are very few surgical ready-to-use devices that have been proposed for the treatment of stable vitiligo.

Evaluate the interest of a new ready-to-use device for NCEC graft (Viticell®, Laboratoires Genévrier, France) in the treatment of stable vitiligo not responding to conventional medical treatments. Eight patients (10 lesions) with resistant and stable vitiligo for at least 6 months were proposed to receive an autologous NCEC graft with a ready-to-use kit.

A healthy thin skin biopsy (0.2–0.3 mm) was taken with an electric dermatoma under local anesthesia from the gluteal area. The biopsy size was determined according to the target lesion surface area: 1 cm^2 on donor site for 5 cm^2 on vitiligo site treated.

The biopsies were treated with Viticell® to obtain, after enzymatic digestion, a melanocyte/keratinocyte epidermal cell suspension. The NCEC suspension combined with a hyaluronic acid solution (2%) was applied on the vitiliginous areas previously dermabraded under local anesthesia. A non-adhesive dressing was used to cover the treated lesions during 4 days.

One week after the graft, patients received excimer lamp or UVB TL01 twice a week for six months period. Photographs of the lesions were taken every two weeks under strict reproducible conditions. Repigmentation (calculated by IMAGE J software) and patient global satisfaction were documented.

Nine lesions belonging to eight patients (mean age 41.5) were treated. The nine targets lesions were located on forehead, face, wrist, forearm, back of the hand and abdomen.For seven lesions, a rapid initiation of repigmentation (20%) was observed on all the localisations except for the back of the hand within 30 days post-graft. And for the same localisations, six lesions showed more than 50% of repigmentation after 45 days and more than 70% of repigmentation after 60 days for six lesions.For 1 lesion, the follow up was too short, the results will be included in the final analysis.We noticed that patients with phototype >IV showed a faster repigmentation.The quality of pigmentation was homogenous.Patients reported a good degree of satisfaction using the global patient satisfaction assessment at month one.After the dermabrasion, the treated lesions were inflammatory for 2 weeks, but no side effect, no hypertrophic or keloid scar, no Koebner phenomenon and no infection were observed.

The characteristics of repigmentation (homogeneous and diffuse) observed, suggest that this procedure may enhance the restoration of the cellular interaction, especially between melanocyte and keratinocyte. In addition, as previously reported, patients with darker skin complexion (phototype > IV) seem to have faster onset of repigmentation.

NCEC using Viticell® is a safe and efficient procedure that seems to initiate repigmentation in a short term with a good success rate (above 70% for five lesions). In addition, it has the advantage

© 2014 John Wiley & Sons A/S. Published by John Wiley & Sons Ltd

of inducing a homogeneous pigmentation compared to epidermal or mini grafts methods as described in the literature. Viticell® gives the opportunity to dermatologists to realize, either at hospital or in private practice, an easy and secure autologous epidermal graft for stable vitiligo.

Disclosure of interestViticell was graciously provided by the Laboratoires Genévrier, France.

P163

Treatment of difficult-to-treat vitiligo lesions using a new autologous non-cultured epidermal cellular grafting kit

M. Pascal[1], M. Ghasemi[2], R. Ghasemi[2], K. Ezzedine[3]

[1]Pigmentation Disorders Research Center; [2]UPMC, Paris; [3]University of Bordeaux National Reference Center for rare skin diseases, Bordeaux, France

Numerous medical treatments exist for the management of vitiligo (corticotherapy, calcineurin derivatives, phototherapy, etc.) although difficult-to-treat areas such as fingers, toes, lips, areolas, elbows and genitals remain resistant to these therapeutics. Common surgical treatments such as thin dermo-epidermal grafts, blister roof graft or minipunch graft, non-cultured epidermal cell (NCEC) suspension and cultured melanocytes have been successful for repigmentating these sites.

However, optimum results depend on adequate immobilization of the lesion, quality of the dressing and use of standardized techniques to minimize the risk of hypertrophic scars.

Evaluate the interest of NCEC using Viticell® kit, a new ready-to-use device in difficult-to-treat areas.

Two patients with stable (at least 6 months) non-segmental vitiligo on wrist (1 treated lesion), forearm (1 treated lesion), and back of the hand (1 treated area), not responding to conventional treatments were proposed to perform an autologous NCEC graft using a new cellular graft technique associated with hyaluronic acid (Viticell®, Laboratoires Genévrier, France) and the use of a non-adhesive dressing.

A healthy thin skin biopsy (0.3 mm) measuring 12 cm^2 was taken with an electric dermatoma under local anesthesia from the gluteal area. The biopsy was immediately incubated in a digesting cocktail and was mechanically scraped to collect melanocytes, keratinocytes and other epidermal cells. The NCEC suspension combined with a hyaluronic acid solution (2%) was applied on the vitiliginous areas previously dermabraded with a CO_2 laser. The treated sites were covered with a silicon dressing perforated on the whole surface (MEPITEL®) and a secondary transparent adhesive dressing (HYPAFIX®); removed 4 days after. Patients received phototherapy treatment (excimer lamp or UVB TL01) one week after the graft, twice a week during 3 months. Pictures were taken each two weeks under strict reproducible conditions. Cumulative UVB dose before graft was 164 Joules on 58 sessions for patient 1 (phototype V) and was 64 Joules (wrist) and 95 joules (back of the hand and forearm) on 61 sessions for patient 2 (phototype IV).

Repigmentation rate was calculated by Image J (N.I.H, USA). Global evaluation of the patients and the quality of the repigmentation were documented.

60% of repigmentation was reached in the lesion of patient 1 (wrist) at 1 month and around 2 months for the lesions of patient two (back of hand & forearm). On the wrist, 99% of repigmentation was achieved at D95 with a cumulated UBV dose of 21,5 Joules. On the forearm, 85% was reached at D72 with cumulated UVB dose of 15.3 Joules. For the three lesions, homogenous repigmentation and normal skin texture were observed. Both patients showed global satisfaction.

The NCEC suspension's viscosity provided by the adjunction of hyaluronic acid may improve the contact between the cells and the treated sites.

Given the positive results obtained in a short-term, this technique would be interesting to reduce the cumulative dose of UVB administered and decrease substantially the treatment time, resulting in less patient drop out.

Viticell®, a ready-to-use device, shows improvement of repigmentation on difficult-to-treat vitiligo and in the treatment time. NCEC suspension combined with hyaluronic acid, used with adequate dressings contribute to achieve a satisfactory and homogenous repigmentation on challenging localizations.

Disclosure of interestViticell® was graciously provided by the Laboratoires Genévrier, France.

P164

A comparative study of repigmentation in stable vitiligo after autologous cultured melanocytes transplantation and non-cultured epidermal suspension transplantation

G. Verma[1,1], H. K. KAR[1], R. Rani[2]

[1]Department of Dermatology, Post Graduate Institute of Medical, Education & Research, Dr. Ram Manohar Lohia Hospital; [2]National Institute of Immunology, New Delhi, India

Vitiligo vulgaris is an acquired disorder of pigmentation due to loss of epidermal melanocytes. Despite the availability of medical treatment, many patients fail to respond. Surgical modalities particularly cellular grafting techniques that includes autologous non cultured epidermal suspension transplantation (NCES) and autologous cultured melanocytes transplantation (CMT) offers promising role in treatment of stable vitiligo.

Aims and ObjectivesTo compare the effectiveness of two surgical modalities NCES and CMT in treatment of stable vitiligo using following parameters: (i) Area and extent of repigmentation (ii) Pattern and colour matching of repigmentation, and (iii) Adverse events if any.

Thirty patients of stable vitiligo vulgaris having at least two target patches at comparable sites in the same patient of area ≥10 cm^2 were selected. After taking split thickness skin graft of 1/10 th size of each recipient site, the tissue graft were subjected to prepare NCES and transplanted next day after dermabrasion of the recipient site while the donor tissue subjected to culture was transplanted on the second target patch when the number of melanocytes reach 1500/mm^2. Patients were followed up till 3 months to evaluate for repigmentation. No other medical treatment for vitiligo including phototherapy were prescribed during follow up period of three months.

Mean percentage improvement in area of repigmentation after CMT was 83% while that after NCES was 30% which was calculated using graph paper. Paired *t* test was used to calculate statistical significance, which was found to be highly significant (P value = 0.0001). In terms of extent of repigmentation, excellent (>75% repigmentation i.e. only few spots of depigmentaion left) was seen in 67% of patient after CMT, 23% showed good response (pigmented area ≥depigmented area) and no patients showed poor response to CMT. While 36% and 50% of patients showed fair (<50% repigmentation) and poor response (<25% repigmentation) respectively to NCES. Chi square test was performed to compare the statistical difference

 © 2014 John Wiley & Sons A/S. Published by John Wiley & Sons Ltd

between two procedure, which was found to be significant for excellent (P = 0.0001) and poor response (P = 0.0001), whereas difference between good (P = 0.299) and fair (P = 0.03) response did not reached the level of statistical significance. No significant difference was seen in color match which was 'same as that of normal skin' in most of the patients and in pattern of repigmentation which was 'diffuse and homogeneous' after both procedures in all patients. Also, leucotrichia improvement was noted equally in both groups. Adverse effects were minimal in both techniques.
Although culturing melanocytes is more time consuming and a labour intensive process, which requires costly equipments with a sterile lab setup but it gives better repigmentation in stable vitiligo cases as compared to that of NCES even with higher recipient:donor graft ratio is used in terms of extent of repigmentation, area repigmented and color matching of the repigmented area. To best of our knowledge, this is the first study directly comparing efficacy of these two techniques in the same patients of stable vitiligo vulgaris.

P165

Correction of cobblestoning due to punch grafting by non-cultured cellular grafting

D. Wang[1], B. K. Goh[1]
[1]National Skin Centre, Singapore City, Singapore

Vitiligo is a disfiguring depigmenting dermatosis that is associated with significant psychosocial morbidity and impairment in quality of life. Punch grafting is a surgical technique for treating vitiligo. Its use can be associated with aesthetically unacceptable complications like cobblestoning.
Case ReportWe present a case of segmental vitiligo on the chin and neck of a 14-year-old boy, whose initial treatment with 2 millimetre mini-punch grafting was complicated by persistent cobblestoning and residual depigmentation. He subsequently underwent non-cultured cellular grafting with carbon dioxide laser ablation (Sharplan Co, Tel-Aviv, Israel) of the cobblestoned lesions, and achieved excellent cosmetic improvement as well as repigmentation of residual lesions.
Mini-punch grafting is an easy-to-perform and cost-effective surgical therapy for vitiligo. Its use, however, can be hampered by the formation of a cobblestoned appearance over the recipient site, which can occur in as many as 47% of treated patients. To date, the treatment of cobblestoning has been limited to electrodessication and electrofulguration. Non-cultured cellular grafting (NCCG) is a more time-consuming and expensive technique, but has a much better adverse effect profile compared to punch grafting. This case study highlights the potential use of NCCG as a 'rescue' modality for patients who have responded sub-optimally to tissue grafting, and its potential in correcting cobblestoning following punch grafting.

Albinism & Related

P166

Oculocutaneous albinism and gorlin syndrome: a coincidence?

V. Dewi[1], R. Danarti[1], C. C. T. Rosari[2], S. Radiono[1]
[1]Dermatovenereology, DR Sardjito Hospital; [2]Department of Dermatology and Venereology, Dr. Sardjito Hospital, Yogyakarta, Australia

Oculocutaneous albinism (OCA) is a genetically heterogenous congenital disorder characterized by decreased or absent pigmentation in the hair, skin, and eyes. Aside from decreased pigment in the iris and retina, there are also optic changes include decreased visual acuity, misrouting of the optic nerves at the chiasm, and nystagmus. Gorlin Syndrome (GS, Nevoid Basal Cell Carcinoma syndrome, MIM 109400) is a rare autosomal dominant disorder; caused by mutation in the patched gene found on chromosome arm 9 q, and is characterized by multiple basalioma, odontogenic keratocyst, palmar and/or plantar pits, and ectopic calcifications of the falx cerebri. This case report draws attention of the coincidence of an OCA with GS, of which we could not find other case report from the literature.Case: A 43-years-old Javanese farmer, was born with white skin and brown eyes. He; complained of wounds that never cured on his back and calf. He has hypertelorism, decreased visual acuity since childhood, nystagmus, multiple basalioma on his trunk, and palmar pits. Orthopantomogram showed multiple radioluscencies in the mandibula and maxilla. Chest radiograph did not show any significant changes. Histopathological examination from back and calf revealed basalioma. Routine blood test, liver function, renal function, CT/BT/PPT/APTT were in normal range. Giant granules were not found in perifer blood smear. We consulted to the surgical oncologist for the multiple basalioma, and gave him SPF30 sunscreen, and education to avoid sun radiation. Discussion: There is increased risk of basalioma in OCA and GS. We assumed that the diagnoses of OCA together with GS in our patient was merely a coincidence, since the pathogenesis of OCA and GS are different. Early detection is needed to educate the patient avoiding sun radiation, ocular disorder detection, and genetic counseling Kata kunci: Albinisme, Gorlin syndrome.

P167

Positive selection with diversity in oculocutaneous albinisms type 2 gene (OCA2) among Japanese

M. Shimanuki[1], G. Tamiya[2], Y. Abe[1], Y. Hozumi[1], T. Suzuki[1]
[1]Department of Dermatology, Yamagata University Faculty of Medicine, Yamagata; [2]Tohoku Medical Megabank Organization, Tohoku University, Sendai, Japan

Human skin pigmentation shows geographical variation, and has been thought as the result of environmental adaptation. The difference is also seen between and within human populations. In Japanese, polymorphisms in *OCA2* and *MC1R* may have association with pigmentary variation.
In order to investigate as if evolutional selective pressure has occurred in Japanese, we genotyped the coding region of *OCA2*. Ninety two genomic DNA collected from healthy Japanese and Japanese data of the 1000 genome project were used for statistical analyses. For neutrality testing, Tajima's D test was used, and evidence for positive selection was detected using PAML with posterior probability ≥0.9 using Bayes empirical Bayes (BEB) approach.

© 2014 John Wiley & Sons A/S. Published by John Wiley & Sons Ltd

We identified 4 synonymous and 5 non-synonymous mutations in *OCA2* of our Japanese samples. Tajima's D value was positive (D = 1.41), but did not reach the significance level of 0.95, suggesting the selection with diversity. On the other hand, three amino acid sites (P241R, T387M, A481T) were identified as being under positive selection.

OCA2 variation showed the possibility of diversifying selection. In previous study, two of three amino acid sites which were identified as under positive selection would have strong association with the mean of the melanin index in the Japanese female. Positive selection seen in this study has a possibility of being concerned with melanin levels. Keeping wide range of abilities in melanin synthesis might be needed in Japanese population.

Skin Lightening Therapies

P168

Disulfanyl peptide decreases melanin synthesis via receptor-mediated ERK activation and the subsequent down-regulation of MITF and tyrosinase

H. Choi[1], Y.-A. Kang[1], H. Lee[1], K. Park[1]

[1]Dermatology, Seoul National University Bundang Hospital, Seongnam, Republic of Korea

Emerging evidence shows that peptides participate in the regulation of cell growth, differentiation, migration and programmed cell death. Despite the extremely important roles of peptides in many biological processes, little attention has been paid to the crucial action of short peptides. In this study, we hypothesized that peptides may regulate melanogenesis via a receptor-mediated pathway. Screening an internal library of peptides permitted as to identify a peptide 2-{2-amino-3-[(2-amino-2-{[1-carboxy-3-(methylsulfanyl)propyl]carbamoyl}ethyl) disulfanyl] propanamido}-4-(methylsulfanyl) butanoic acid (disulfanyl hypopigmenting peptide, dSHP) that inhibits melanin synthesis in normal human melanocytes. dSHP significantly decreases tyrosinase activity in a concentration-dependent manner. dSHP was not effective in a direct in vitro assay and it induces the delayed activation of ERK and subsequently down-regulates levels of MITF and tyrosinase. In addition, PD98059 abolished the dSHP- induced down-regulation of MITF. These results indicate that the ERK pathway is involved in the signaling cascade affected by dSHP and that the dSHP-induced activation of ERK contributes to reduced melanin synthesis via the down-regulation of MITF. Serial fluorescent microscopic studies also showed ERK activation in dSHP-treated cells. We therefore further investigated the actions of dSHP to determine whether it can act via a receptor-mediated pathway and showed that pertussis toxin reverses the down-regulation of MITF. In turn, exact these results suggest that receptor-mediated ERK activation is involved, despite the receptor remaining role is not identified. Furthermore, down-regulation of MITF was clearly inhibited by chloroquine. In summary, our results show that the novel tetrapeptide dSHP reduces melanin synthesis by both receptor-mediated downregulation of MITF and tyrosinase and also the lysosomal degradation of MITF.

P169

An innovative and safe therapy for hyperpigmentary disorders

E. Flori[1], E. Bastonini[1], S. Briganti[1], D. Kovacs[1], G. de Paoli Ambrosi[2]

[1]San Gallicano Dermatologic Institute IFO IRCCS, Rome; [2]General Topics S.R.L., San Felice del Benaco, BS, Italy

A primary function of melanin is the protection against ultraviolet irradiation. However, an excessive accumulation of melanin causes hyperpigmentary disorders such as melasma, post-inflammatory melanoderma and solar lentigo, with a considerable impact on the quality of life of affected individuals. Many of the commonly used whitening agents act as competitive inhibitors of tyrosinase, which is the main enzyme involved in melanin synthesis. Because of the incomplete effectiveness and the possible side effects of high dosage of these compounds, recent strategies for the development of skin lightening products include the combination of ingredients acting upon different steps of the pigmentation pathway. An innovative combination of compounds has been recently developed, acting by the specific inhibition of the catalytic activity of tyrosinase by three key steps. The active ingredients of THIOSPOT® cream are: phenyl ethyl resorcinol, which inhibits tyrosinase catalytic activity on DOPA; acetylglucosamine, which perturbates tyrosinase glycosylation and ethyl linoleate, which increases proteasomal degradation of tyrosinase and promotes epidermal turn-over. We evaluated whether the formulation can exert a combined inhibition of the melanogenic process without inducing cell damage. To this end, we first treated B16-F0 murine melanoma cell line with non-cytotoxic doses of single ingredients or their combination, in presence or not of alpha-Melanocyte Stimulating Hormone (alpha-MSH), as a principal hormone involved in the stimulation of melanogenic process. The following parameters were assayed: cell viability by Neutral Red cytotoxicity test; intra-cellular melanin content and tyrosinase activity measured by spectrophotometrical analysis; tyrosinase expression by means of Western Blot and immunofluorescence evaluation. The results evidenced the ability of the single compounds to significantly reduced the amount of intra-cellular melanin as well as the expression and the activity of tyrosinase. The combined treatment showed a greater effect than single ingredients, indicating an additive action of the combination. Interestingly, the depigmenting action was most effective towards alpha-MSH-induced melanin synthesis. The analyses were also performed on primary cultures of normal human melanocytes (NHM) and we obtained similar results about melanogenesis inhibition. Moreover, in NHM, the ability of the single ingredients as well as their combination to interfere with alpha-MSH-induced activation of tyrosinase was more pronounced than that observed in B16. Clinical data confirmed the efficiency of the product. In Conclusion, our results suggest that the combination of the three ingredients contained into the THIOSPOT® cream is able to significantly interfere with melanin synthesis by reducing the amount of tyrosinase, increasing its proteolysis and inhibiting its catalytic activity, mostly under hyper stimulated melanogenesis. Our results support THIOSPOT® cream as an innovative and safe therapy for acquired hyperpigmentation.

 © 2014 John Wiley & Sons A/S. Published by John Wiley & Sons Ltd

P170

Melanoproteins mediating the cytotoxic activity of bleaching phenols

J. Eby[1], I. Wirjan[2], K. Willenborg[1], C. L. Poole[1]

[1]Oncology Research Institute, Loyola University Chicago, Maywood; [2]Biology, Illinois Mathematics and Science Academy, Aurora, IL, USA

The skin bleaching effects of phenolic agents may be associated with their structural similarity to tyrosine. We are interested in phenolic agents with selective cytotoxic activity towards melanocytes for application in vitiligo or melanoma patients. Monobenzyl ether of hydroquinone (MBEH) is the single USFDA approved agent for use in vitiligo, and induces melanocyte necrosis. We reported that heavily melanized cells are relatively protected from MBEH-induced cytotoxicity. This is in apparent contrast to the suggested enzymatic role of rate-limiting enzyme tyrosinase in MBEH induced cell death, by converting the compound to a toxic quinone. Here we addressed the role of individual melanoproteins in MBEH and 4-tertiary butylphenol (4-TBP)-mediated cytotoxicity. For these studies we cloned cDNA encoding melanoproteins tyrosinase, TRP-1, TRP-2, gp100 and MART-1 from mouse melanoma cell line B16F10. PCR products were inserted into a eukaryotic expression vector and sequence verified. Plasmids were subsequently introduced into HEK293 cells. Protein expression was verified in G418 selected cells by fluorocytometry. Resulting transfected cells and empty vector transfected, as well as B16F10 control cells were subjected to 125–250 μM MBEH and 4-TBP treatment for 24 h. Cell viability was evaluated microscopically and by MTT assays. Pigmented, tyrosinase expressing cells experienced a 2.4 -fold increased sensitivity to MBEH over Background Interestingly, TRP-1, TRP-2 and gp100 also mediated a 1.8 fold increase in cell death. The 4 melanoproteins contributed approximately equally to cell death in response to 4-TBP. MART-1 expression however, offered marked protection from cell death at lower concentrations of either agent. These experiments consolidate the role of melanogenic enzymes in bleaching agent-induced induced melanocyte loss and suggest a possible toxin sensing role of MART-1 in melanocytes.

P171

Efficacy of intense pulsed light for treating several types of pigmented spots on the face in combination with an inhibitor of melanogenic intracellular signaling

H. Nojiri[1], Y. Kuo[2], Y. Han[3], W. Chen[3], K. Ishida[1], T. Nishizaka[1], T. Hisateru[1], G. Imokawa[4]

[1]Skin Care Product Development Laboratories, Kao Corporation, Tokyo, Japan; [2]Aphrodite Beauty Clinic, Taipei; [3]Consumer Product R&D Division, Kao Taiwan Corporation, Hsinchu Hsien, Taiwan ROC; [4]Research Institute of Biological Function, Chubu University, Aichi, Japan

Several different treatment modalities exist to treat pigmented spots on the face. Cutaneous lasers, including the frequency-doubled Q-switched Nd:YAG laser, the Q-switched ruby laser (QSRL) and the 510 nm pigmented lesion dye laser, as well as Intense Pulsed Light (IPL) have been reported to improve various types of pigmented spots such as solar lentigines and ephelides. However, those laser treatments sometimes cause adverse effects including cutaneous inflammation and hyperpigmentation, depending on the laser energy applied and the duration. Hyperpigmentation, probably due to preceding cutaneous inflammation, frequently spoils the anti-pigmenting effect of cutaneous laser treatment of pigmented spots. Cutaneous inflammation as a result of tissue photothermolysis is a major strategy to remove melanin-containing keratinocytes and melanocytes, but can also serve as a stimulus for melanogenesis in melanocytes by releasing a primary inflammatory cytokine (IL-1alpha) and subsequently by secreting a potent melanogenic cytokine endothelin-1 (EDN1) in an autocrine fashion. Topical tretinoin, 4-hydroxyanisole and hydroquinone have been used to prevent inflammation-elicited hyperpigmentation in spite of their properties as primary irritants. Thus, immediately after cutaneous laser treatment, using an inhibitor of the EDN1-triggered intracellular signaling pathway that leads to stimulated epidermal pigmentation would be a good therapeutic resolution to enhance the anti-pigmenting effect of cutaneous lasers. In this study, the efficacy of a single treatment with intense pulsed light (IPL) for several types of pigmented spots on the face in combination with a whitening cream containing an EDN1 signaling inhibitor (*Chamomilla* extract) and a synthetic pseudo-ceramide was evaluated in a single-blind test using 22 volunteers who had pigmented spots on their face (melasma, freckles or solar lentigines). The results show that all subjects completed this test. During the test, no adverse effects were observed. Uncomfortable sensations after IPL surgery (e.g. stinging, burning, itchiness, pain, tightness and redness) had almost diminished 1 week after treatment with the test product, which indicates that the test product has a high tolerability. Skin hydration at three different parts of the face significantly decreased just after the IPL surgery, and continued for 2 weeks. At 6 weeks, those values had almost returned to the level before surgery. TEWL values significantly decreased at 1 and 2 weeks after surgery and returned at 6 weeks. These results indicate that the function of the stratum corneum was injured by IPL exposure and that aggressive skin care using the test products restored them. An improvement in pigmented spots was observed in 17 of the 21 subjects (about 80%) at week 2. Improvement of the pigmented lesions occurred mainly for solar lentigines and freckles, but not for melasma or nevi. At week 6, some of the improved pigmented lesions reappeared in 12 of the 17 subjects (70%), but the intensity of color of those recovered spots was still lighter than before the IPL exposure. The faint pigmented lesions on the cheek of 1 subject became obvious at week 6, but that lesion was actually melasma. Post-inflammatory hyperpigmentation (PIH) was not observed in this study. The sum of these findings suggests that the moisturizing effects of the test products are suitable for skin care and tissue recovery after IPL treatment and the whitening agent of the test products is effective to some extent in suppressing PIH.

P172

Maintenance Of 4% hydroquinone + 0.025% tretinoin clinical results with a novel cosmeceutical formulation during the summer months

Z. Draelos[1], S. Raab[2], M. Yatskayer[3], N. Chen[4], Y. Krol[5], C. Oresajo[6]

[1]Dermatology Consulting Services, High Point, NC; [2]L'Oreal USA Research & Innovation, Clark, NJ; [3]Skinceuticals, New York, NY; [4]L'Oreal USA Research& Innovation, Clark, NJ; [5]Skinceuticals, New York, NY; [6]L'Oreal USA Research & Innovation, Clark, NJ, USA

The goal of this study was to evaluate the ability of a novel cosmeceutical formulation, containing hydroxyphenoxy propionic acid, ellagic acid, yeast extract, and salicylic acid, applied for

© 2014 John Wiley & Sons A/S. Published by John Wiley & Sons Ltd

20 weeks during the summer months, in maintaining the dyspigmentation improvement attained after 12 weeks of hydroquionone/tretinion prescription therapy.
33 healthy subjects ages 25–60 yrs were enrolled in this 20-week single center study. After 12 weeks use of generic 4% hydroquinone cream in combination with generic 0.025% tretinoin cream, the subjects were instructed to discontinue the use of prescription therapy and immediately start the application of cosmeceutical formulation twice daily for the 20 week period. Evaluation assessments included dark spot (size and intensity), hyperpigmentation, smoothness (visual and tactile), skin tone (clarity and evenness), radiance, blotchiness, and overall appearance. Tolerability assessment included erythema, edema, dryness, and peeling while the subjects assessed stinging, tingling, itching, and burning. Bioinstrumentation measurements and digital photographs were obtained at weeks 12 and 20.
The cosmeceutical formulation improved skin feel and appearance while maintaining the pigment lightening results achieved with the hydroquinone/tretinoin combination, even though the prescription treatment was discontinued after week 12. This was especially noteworthy since the cosmeceutical formulation was tested during the summer months, providing the ultimate torture test for a hyperpigmentation product. Bio-instrumental analysis, skin quality improvements, and continued reduction in unwanted pigmentation all confirmed the complementary benefits offered by the cosmeceutical formula when used following the recommended 12 week use of the prescription hydroquinone/tretinoin combination. The formulation was well tolerated by all the subjects.

P173

Efficacy and safety of a soy extract and niacinamide cosmetic serum to improve skin hyperpigmentation in Korean women

K.-C. Park[1], S.-H. Kwon[1], S.-Y. Byun[1], C.-H. Huh[1], J.-I. Na[1], H.-O. Kim[2], C. Galzote[3]

[1]College of Medicine, Seoul National University Bundang Hospital, Seoul, Republic of Korea; [2]Johnson & Johnson Asia Pacific, Singapore City, Singapore; [3]Johnson & Johnson Emerging Market Innovation Center, Shanghai, People's Republic of China

Soybean extract contains soybean trypsin inhibitor that inhibits melanosome transfer by inactivating protease-activated receptor 2 (PAR2). A clinical study was performed to investigate the clinical efficacy of a cosmetic serum containing soy extract and naicinamide for facial hyperpigmentation. 33 volunteer Korean females with skin tone unevenness and facial hyperpigmentation were recruited. Subjects applied the serum twice a day for 8 weeks. Clinical efficacy was evaluated by clinical grading of overall fairness, skin tone evenness, spot color, blotchiness, smoothness, moisture and radiance. Non-invasive instrumental skin measurements were taken at baseline, 20 min, 2-, 4-, 8-weeks. Subject self-assessment via questionnaire was also taken during these visits. In addition, immunohistochemistry was performed on skin biopsies taken from five patients at baseline and week 8 to determine the clinical effects of the serum on PAR2 distribution in the epidermis. Twenty nine subjects who completed the trial showed significant improvements in all clinical grading attributes. Instrumental assessments also showed significant improvement. Surface and inner moisture continuously increased throughout the study. Overall rating showed good improvement in quality of life assessment. Immunohistochemistry revealed significantly lower expression of PAR2 after 8 weeks of serum application. No reportable skin irritation events were observed, indicating good tolerance of the test serum on Korean female facial skin. In Conclusion, the soy extract plus naicinamide serum improves skin fairness, and all radiance parameters.

P174

Sustained anti-hyperpigmentation benefits with daily application of stabilized 0.1% retinol formula

M. Randhawa[1], J. Fantasia[1], J. Thakrar[1], S. T. Samaras[1]

[1]Johnson & Johnson Consumer Companies, Inc, Skillman, NJ, USA

Regular application of retinol-based preparations have been shown to improve skin wrinkles, fine lines, pigmentation and skin tone. The majority of clinical studies designed to investigate the efficacy and tolerance of such cosmetic technologies are typically evaluated at 1, 2 and 3 months. However, researchers and experts in aesthetic dermatology recognize that further clinical benefits can be obtained when retinol is routinely used on a long-term basis.
We report the results of a 52 week, double-blinded, randomized, balanced, full face, vehicle-controlled clinical study in 60 healthy female subjects, aged between 40 and 55 yrs. All subjects had moderately photodamaged facial skin at time of recruitment, defined as Fitzpatrick Skin type I-III. The subjects applied either the active preparation, containing 0.1% stabilized retinol, or the vehicle, to their faces once daily for twelve months. Clinical evaluations were performed by investigators at 3, 6, 9 and 12 months. Self-assessment questionnaires were completed by all subjects. Determination of skin benefit was based on clinical observations, photographs, and subject self-assessment. Tolerance of the formulations was evaluated according to the incidence and severity of skin irritation and discomfort, as determined by the investigator.
The cosmeceutical formulation, containing 0.1% stabilized retinol, significantly improved parameters associated with discrete and mottled hyperpigmentation, skin tone evenness and overall photodamage all time points. Aging parameters like wrinkles and fine lines also showed improvements at all time-points throughout the study. Significantly greater benefits were observed compared to those who applied the vehicle.
The results described here from a long-term anti-aging cosmeceuticals clinical study provide clear evidence-based data that can guide doctors specializing in aesthetic medicine to offer the best recommendations for appropriate products for their patients seeking sustained improvement in the appearance of aging facial skin.

P175

Non invasive and short term assessment of retinoids effects on melanin content using in vivo multiphoton microscopy

E. Tancrède-Bohin[1,2], A.-M. Pena[1], N. Parent[1], S. Brizion[1], S. Victorin[1], E. Decencière[3], M. Bagot[2], T. Baldeweck[1]

[1]Research and Innovation, L'Oréal, Aulnay sous Bois; [2]Department of Dermatology, St Louis Hospital, Paris; [3]Centre de Morphologie Mathématique – Mathématiques et Systèmes, MINES ParisTech, Fontainebleau, France

In the past decade, multiphoton microscopy (MMP) has emerged as a very powerful tool for non invasive skin imaging, allowing the

 © 2014 John Wiley & Sons A/S. Published by John Wiley & Sons Ltd

3D structure of human skin to be characterized in vivo with a sub-cellular resolution. We recently developed 3D image processing tools for MMP images that allow us to automatically segment the different skin layers and extract several quantitative parameters characterizing the skin including the melanin density in the epidermis[1,2].
To demonstrate the usefulness of this method for non invasive kinetic studies of the modulation of melanin content induced by two retinoids, as gold standard molecules in both photo-ageing and pigmentation disorders.
Twenty healthy female caucasian volunteers, aged 50–65 yrs, with photodamaged skin were enrolled in a randomized, double-blind, monocenter comparative study. Two products, Retinol 0.3% (RO) and Retinoic acid 0.025% (RA) were applied to the dorsal side of the forearm under occlusive patches for 12 days, according to the short term screening protocol previously described[3]. A patch alone was applied to a third area as control. Evaluation of the three areas was performed at D0, D12, (end of the occlusion period), D18 and D32 using MPM and colorimetric measurements.
Main significant results ($P < .05$, with moderate to very strong effect sizes) were: (i) A thickening of the epidermis, as described with histology in this kind of protocol, and an increase in the DEJ undulation on retinoid treated areas at different times of the study versus baseline and versus control, with RO > RA at D32. Cutaneous irritation which could be responsible of edema was noted in 19 out of 20 RO-treated areas, and in 14 out of 20 RA-treated areas at D12, but resolved within a few days and could not account for the epidermal thickening observed at D32.
(ii) A decreased melanin content with RO (at D12 and D18 versus baseline and at D32 versus baseline and versus control) and with RA (at D12 versus baseline), associated with a clinically visible whitening. Results of colorimetric measurements were found either slight, either paradoxical, likely due to the colour interferences between irritation (redness) or scaling and pigmentation.
In this study, beneficial effects on epidermal morphology and pigmentation on photo-damaged skin as well as local irritation are more pronounced with RO than with RA which is probably due to the high concentration of RO used (0.3%), 12 times higher compared to that of RA (0.025%).
This study shows that such short term protocol combined with in vivo multiphoton microscopy allows the effects of retinoids upon melanin content to be detected, quantified and followed over time post treatment, non-invasively. It also shows that the specific melanin detection by multiphoton microscopy overcomes the known bias of colorimetric interferences induced by other constituents than melanin e.g. haemoglobin when evaluating cutaneous pigmentation.

References: [1]Ait el Madani *et al.*, J. Biomed. Optics 17 (2012); [2]Decencière *et al.*, Skin Res Technol. 19 (2013); [3]Watson *et al.* Br. J. Dermatol. 158 (2008).

P176

Placebo-controlled study of the efficacy of a dietary supplement to reduce skin dark spots and to create more even complexion in Asian women

E. Cestone[1], F. Marzatico[2], V. Nobile[1], M. Vitale[3]
[1]Farcoderm SRL European Network for Tests in Dermatology and Wellness; [2]Institute of Molecular Biology CNR, University of Pavia, Pavia, Italy; [3]Medical Department, IFC, Madrid, Spain

Solar radiation induces skin blotches, uneven complexion and worsens conditions involving hyperpigmentation, such as melasma and skin aging. Photoaging is a premature aging of the skin caused by repeated exposure to several environmental factors, especially sun radiation, but also artificial radiation sources. The early signs of photoaging include solar lentigines (age spots) on areas such as the face and hands.
The hyperpigmentation condition commonly affects subjects with a high phototype and often is associated with a considerable psychological impact. It is usually treated with conventional topical treatments (depigmenting products) and sunscreens are strongly indicated to prevent sun exposure. We hypothesize that the intake of an oral dietary supplement including: (i) an extract with very well-known antioxidant and photoprotective properties, namely Fernblock®; and (ii) a combination of ingredients that reduce melanogenesis, *i.e. Punica granatum*, ascorbic acid and niacinamide, could be very effective to reduce and prevent skin dyschromia.
The aim of this study has been to demonstrate the lightening, brightening and anti-aging efficacy of the dietary supplement under study in women with Asian skin.
The study was carried out in 30 women showing the clinical signs of photoaging: skin blotches, uneven complexion and skin aging. All of them applied topical sunscreen (SPF 30) and 20 subjects received Heliocare Pure White Radiance® (2capsules/day) and 10 subjects received placebo (2capsules/day).
After 12 weeks with the oral supplement treatment, the 75% of women showed a significant diminish of melanin index (12.9%). The group with the active ingredients treatment showed a significant decrease of transepidermal water loss (TEWL) ($P = 0.004$), melanin index ($P = 0.034$), wrinkles depth ($P = 0.032$) and a significant increase of skin moisturization ($P = 0.002$), gloss value ($P = 0.020$), skin elasticity ($P = 0.043$) and a significant improvement of skin firmness compared to placebo patients. Some of these effects were also statistically significant when comparing with the start of the study. No adverse events have been reported during the study.
The oral supplementation with the product under study is effective in decreasing the signs of skin photoaging and dyschromia lesions. Furthermore, the treatment has shown, through specific tests, an eutrophic skin effect (increased firmness, moisturization and decreased wrinkles and skin blotches).
Heliocare Pure White Radiance® has a clear advantage as an orally administrated depigmenting product due to the achievement of a uniform distribution and systemic photoprotective effects since Fernblock® is one of the main ingredients.

P177

Mitochondrial depletion via nonactivating MSH conjugate leads to reduction in pigment

J. Waugh[1], S. K. Tan[1]
[1]JYSK, Singapore City, Singapore

Mitochondrial activity is a key regulator of cell activity. Targeted depletion of melanocyte mitochondria was hypothesized to lead to selective decrease in activity.
Two peptides were synthesized: (i) native Alpha MSH sequence (aMSH) and (ii) a high avidity binding but non-activating MSH analogue conjugated with a mitochondrial depletion sequence (HPP2). On six guinea pigs, four discrete $1.5 \times 1.5\ cm^2$ test sites of the shaved dorsum per animal were selected and marked by a single point tattoo. Each site was randomized to one of four topical treatments: (i) aMSH (200 mcg/100 mcL) + 0.02% tretinoin in saline, (ii) HPP2 (200 mcg/100 mcL) + 0.02%

© 2014 John Wiley & Sons A/S. Published by John Wiley & Sons Ltd

tretinoin in saline, (iii) 0.9% saline, 4) 0.02% tretinoin in saline. Treatments were applied once daily for 4 weeks. H&E, Fontana-Masson, TUNEL, and PCNA stains were performed on the test sites at week 4 after weekly grading of gross pigmentation.
AlphaMSH treatment resulted in gross darkening of skin in 83% of animals, with 50% exhibiting marked darkening. Saline and tretinoin treatments did not alter pigment levels of skin or hair versus untreated areas. Treatment with HPP2 achieved a mild gross reduction in pigment in skin and marked reduction of pigment in hair in 100% of animals. Histology confirmed the gross observations. Selective apoptosis was confirmed in TUNEL stains in some HPP2 animals.
Targeted mitochondrial depletion can be used to decrease cellular activity selectively and reduce pigmentation. As in other applications, further work to establish dosage will be essential to control the balance of reduced energy versus apoptosis.

Paediatric Pigmentary Disorders

P178

Combined plaque-type blue nevus on café-au-lait spot

Y. Kaku[1,2], H. Takeshita[1], T. Ito[2], E. Itou[2], M. Furue[2], K. Urabe[3]
[1]Division of Dermatology, Japanese Red Cross Fukuoka Hospital; [2]Department of Dermatology, Graduate School of Medical Sciences, Kyushu University; [3]Department of Dermatology and Allergy, National Hospital Kyushu Medeical Center, Fukuoka, Japan

Blue nevus is a small slate-blue to blue-black macule or papule found most commonly on the extremities. Occasionally, an overlying intradermal nevus is present: such lesions are called combined nevi. Combined nevi are characterized by the presence of two or more different types of melanocytic nevi in a single lesion. We report here plaque-type blue nevus combined with café-au-lait spot on the face.
A 1-year-old Japanese woman was referred to our clinic for a second opinion of pigmented lesions, present since birth on her right preauricular area. Physical examination revealed a livid macule 43 × 16 mm in size with café-au-lait, spot-like brown patch 26 × 14 mm in size. However, there were no other findings suggestive of neurofibromatosis. Dermoscopically, the macule showed a homogenous livid in color, suggesting a substantial amount of melanin in the upper dermis. Histopathologic examination revealed a spare population of dendritic and spindle-shaped melanocytes with melanin pigments in the deep reticular dermis. Based on these findings, a diagnosis of combined blue nevus on café-au-lait spot was made.
Plaque-type blue nevus is a rare variant of blue nevus. It contains numerous blue macules and papules, with or without an intervening bluish discoloration. To our knowledge, there have been only seven reported cases of plaque-type blue nevus combined with café-au-lait spot in the English literature. We reviewed these cases and found that there were four male cases and three female cases and the patient' s ages ranged 15–38 yrs old with a mean age of 27.6. In all the cases, lesions occurred on the trunk or extremities. This is the first case of plaque-type blue nevus combined with café-au-lait spot on the face.
Blue nevus is a small slate-blue to blue-black macule or papule found most commonly on the extremities. Occasionally, an overlying intradermal nevus is present: such lesions are called combined nevi. Combined nevi are characterized by the presence of two or more different types of melanocytic nevi in a single lesion. We report here plaque-type blue nevus combined with café-au-lait spot on the face. A 1-year-old Japanese woman was referred to our clinic for a second opinion of pigmented lesions, present since birth on her right preauricular area. Physical examination revealed a livid macule 43 × 16 mm in size with café-au-lait, spot-like brown patch 26 × 14 mm in size. However, there were no other findings suggestive of neurofibromatosis. Dermoscopically, the macule showed a homogenous livid in color, suggesting a substantial amount of melanin in the upper dermis. Histopathologic examination revealed a spare population of dendritic and spindle-shaped melanocytes with melanin pigments in the deep reticular dermis. Based on these findings, a diagnosis of combined blue nevus on café-au-lait spot was made. Plaque-type blue nevus is a rare variant of blue nevus. It contains numerous blue macules and papules, with or without an intervening bluish discoloration. To our knowledge, there have been only seven reported cases of plaque-type blue nevus combined with café-au-lait spot in the English literature. We reviewed these cases and found that there were four male cases and three female cases and the patient' s ages ranged 15–38 yrs old with a mean age of 27.6. In all the cases, lesions occurred on the trunk or extremities. This is the first case of plaque-type blue nevus combined with café-au-lait spot on the face. We underwent irradiation test. Therapic effect on the café-au-lait spot and not on the blue nevus.

Lasers and Light Interventions

P179

Use of polarised light therapy after skin tumor surgery

D. V. Nikolic[1], A. T. Nikolic[2], V. V. Stanimirovic[3], S. K. Vranic[4]
[1]Oncosurgery, Medical Faculty, Melanoma Center, UMC Bezanijska Kosa; [2]Non invasive cardiology, Medical Faculty, ICVD Dedinje; [3]ALIMS, ALIMS, Belgrade, Serbia; [4]International BD Zepter Medical, Bioptron Zapter, Wollerau, Switzerland

It is well known that effect of polarized light (wave length 400–2000 nm, degree of polarization >95%, power density 40mW/cm^2, light energy 2.4J/cm^2) using Bioptron Light Therapy Device on the healing of wounds are very good.
The primary Objective of this study was to show the results of use of Bioptron light therapy in different conditions after variety of procedures on the skin.
We read papers with open access on Pubmed which describes effects of Bioptron light therapy. We found 23 articles on Pubmed with the keyword Bioptron. Dominantly in these articles use of polarized light therapy on wound healing after different procedures is described.
16 yrs ago in article in German Krankenpfl Journal it was described the good effect of Bioptron therapy on acne vulgaris in cosmetic medicine. Also there were few articles talking about good effect of light therapy in wound healing after diabetic gangrene or treatment of disorders of wound healing. Using the same light in improving healing of cutaneous wounds in nourished or undernourished Wistar rats in Brazilian Dental Journal or in pressuring ulcer healing in Vojnosanitetski Pregled, showed significant positive results on treated areas. One very good article concerning use of phototherapy without consulting a physician, when patient applied phototherapy onto the area for 30 months, talks about ignorance of light therapy and possibilities that this therapy could not cure primary cutaneous melanoma with abundant myxoid matrix. This article was published in American Journal of Pathology. There are bunch of

 © 2014 John Wiley & Sons A/S. Published by John Wiley & Sons Ltd

articles talking about very good effects of light therapy in different diseases in rheumatology.
The BIOPTRON electromagnetic spectrum does not contain UV radiation! BIOPTRON Light is a polychromatic light. It contains not only one wavelength (like laser light), but a wide range, including visible light and a portion of the infrared range. The wavelength of BIOPTRON Light ranges from 480 nm to 3400 nm. That means that UV light prolonged phototherapy can be used under the physician control when the good results for specific treatments are guaranteed Thus, personally applied phototherapy is not bared out. It is obligatory for all patients to consult physicians before decision to use phototherapy, especially for prolonged time.

Photoprotection & Photocarcinogenesis

P180

Daily ultraviolet radiation induced gene expression modulation in a reconstructed Asian skin model

C. Ding[1], Y. Liu[1], M. Hong[1], R. Wang[1], E. Planel[2], Z. CAI[1]
[1]L'Oréal Research and Innovation, Shanghai, People's Republic of China; [2]L'Oréal Research and Innovation, Aulnay-sous-Bois, France

Exposure to repetitive sub-erythemal doses of daily UV radiation (DUVR) may lead to long term clinical alterations such as photoaging and, ultimately, skin cancers. Reconstructed human skin models have been demonstrated to be useful tools for studying UV-induced biological effects, including gene expression modulation [1]. In the present study, a full-thickness skin model containing a well differentiated epidermis and a living dermis was reconstructed in vitro with skin cells from Chinese origin. Genes involved in different biological functions were selected and their modulations by daily UV exposure were investigated using Affymetrix QuantiGene RNA Multiplex assay platform. The observed gene modulations were further quantitatively confirmed by RTqPCR. The genes modulated by DUVR were identified respectively in dermal fibroblasts and epidermal keratinocytes. The approach of gene expression analysis based on reconstructed Asian skin model provides a valuable tool for studying the early events induced by DUVR and could become a unique and powerful methodology to better understand the mechanisms of cutaneous photoprotection and to evaluate the photoprotection efficacy of cosmetic actives and formulas. This methodology therefore can fully support the development of daily photoprotection products for meeting the specific needs of Asian populations in the domain of UV protection.

[1] Marionnet, C. *et al.* (2012). Modulations of gene expression induced by daily ultraviolet light can be prevented by a broad spectrum sunscreen. *J Photochem Photobiol B*. 116, 37–47.

P181

Nuclear receptor activation affords a complete photoprotection effect

E. Flori[1], A. Mastrofrancesco[1], D. Kovacs[1], S. Briganti[1], V. Maresca[1], G. Cardinali[1], M. Picardo[1]
[1]San Gallicano Dermatologic Institute IFO IRCCS, Rome, Italy

Increasing attention is addressed to identify products able to enhance skin photoprotection and to prevent skin carcinogenesis. Several studies demonstrate that alpha-MSH, acting on a functional MC1R, provides a photoprotective effect by inducing pigmentation, antioxidants and DNA repair. We recently discovered a link between alpha-MSH and the nuclear receptor Peroxisome Proliferator-Activated Receptor-gamma (PPAR-gamma), suggesting that some of the alpha-MSH protective effects may be dependent on PPAR-gamma transcriptional activity. In fact, the pharmacological activation of this nuclear receptor induces some of these defensive activities. We previously described the effects of the parrodiene octatrienoic acid (Octa), a molecule sharing some structural features with carotenoids and retinoids. In particular, we demonstrated that Octa operates as a novel promoter of melanogenesis and antioxidant defence in normal human melanocytes in situ and in vitro, through the activation of PPAR-gamma. These results were supported by the evidence that Octa increases cell antioxidant defence and counteracts senescence-like phenotype in primary cultures of human fibroblasts. Considering that keratinocytes are the major target cells of UV radiation, the aim of the present study was to evaluate the protective effect of Octa on UVA and UVB-induced damage on normal human keratinocytes (NHKs) and to define its possible mechanisms of action. Hence, cells were pre-incubated with the compound and then irradiated. Octa exerted a cytoprotective effect resulting in increased cell survival and lowered apoptosis following UVA and UVB. Moreover, Octa enhanced the removal of cyclobutane pyrimidine dimers (CPDs) and 7,8-dihydro-8-oxoguanine (8-oxodG), two major DNA damages induced by UVB and UVA. Octa-treated keratinocytes showed a reduced induction of p53, phospho-p53 and GADD45a (growth arrest and DNA damage response gene) proteins, which are involved in UV-induced DNA damage response. Consistent with these results, cells treated with Octa presented, after UVA or UVB irradiation, a decreased fraction of phosphorylated histone H2AX, whose expression is associated with the amount of DNA double strand breaks and a decreased level of phospho-ATM protein, a key DNA-damage sensor. We also evaluated the action of Octa addition soon after UVA or UVB irradiation to investigate the effects specifically due to the post-treatment. We observed a photoprotective effect similar to that obtained with the pre-treatment on cell viability, apoptosis, CPDs and 8-oxodG formation. Moreover, Octa post-incubation decreased the levels of proteins involved in UV-induced DNA damage response in UVB-irradiated keratinocytes. Time course analysis indicated that the decrease in the level of these proteins was associated with the accelerated p53, phospho-p53 and GADD45a expression at earlier post-UVB periods, thus favouring the repair of the UVB-induced DNA damage. The analysis of the possible Octa mechanism of action showed the ability of the compound to activate PPAR-gamma and to concomitantly up-regulate Nrf-2 and heme oxygenase-1, important players in the defence of cells against oxidative stress. Furthermore, in PPAR-gamma deficient cells, Octa photoprotective action was significantly less effective. In Conclusion, our results indicate that PPAR-gamma stimulation affords photoprotective effects also in NHKs, suggesting perspectives for the development of molecules, such as Octa, able to act as complete sunscreens through the activation of this nuclear receptor.

© 2014 John Wiley & Sons A/S. Published by John Wiley & Sons Ltd

P182

New developments in photoprotection and prevention of photocarcinogenesis

C. L. Goh[1], S. Gonzalez[2]

[1]National Skin Centre, Singapore City, Singapore; [2]Sloane Kettering Cancer Center, New York, NY, USA

Photoprotection is essential to prevent the deleterious effects of ultraviolet (UV) light, including skin cancer, photoaging, hyperpigmentation, and immunosupression. The use of botanic supplements endowed with substantial antioxidant activities has generated wide interest to decrease the risk of skin disease induced by UV radiation-dependent oxidative stress. Oral sun screening agents can provide a degree of protection by blocking, at least partially, the abnormal generation of ROS that underlies most of these diseases. In this context, a hydrophilic extract obtained from the aerial parts of the fern Polypodium leucotomos (PL, Fernblock®) exhibits strong photoprotective properties, reducing UV-induced inflammation and pigmentation. We will discuss the evidence on the cellular and molecular mechanisms underlying its photoprotective effect. PL is a natural mixture of phytochemicals endowed with powerful antioxidant properties. Its short-term effects include inhibition of reactive oxygen species production induced by UV radiation, hyperpigmentation and related disorders such as melasma, DNA damage, and isomerization and decomposition of *trans*-urocanic acid, preservation of Langerhans cells population, prevention of UV-mediated apoptosis and necrosis, as well as degradative matrix remodeling, which is the main cause of photoaging. These short-term effects translate into clinical prevention or treatment of UV-related disorders such as polymorphous light eruption and other photodermatoses, melasma and actinic keratosis, and long-term prevention of photoaging and photocarcinogenesis.

P183

Photoprotection: the importance of a dedicated sun protection counselling service

S. X. Lee[1], X. T. Lauw[1], W. S. Chong[1], E. Tian[1], W. Choo[1], W. Li[1], G. Chua[1], W. Chen[1], C. Tan[1], H. Oon[1]

[1]National Skin Centre, Singapore City, Singapore

Exposure to ultraviolet radiation has been linked to various dermatological conditions, including pigmentary disorders, melanoma and non-melanoma skin cancers. There is a considerable degree of inter-individual variability with regards to sun protection awareness and attitudes.

To assess if patients and allied health professionals understood the importance of photoprotection, and to evaluate the effectiveness of a dedicated counselling service with regards to improving their level of awareness.

Fifty patients who were seen at the National Skin Centre (NSC) in Singapore, as well as 50 NSC allied health staff members, were surveyed with a questionnaire designed to assess sun protection awareness, attitudes and behaviours. Each study subject then underwent a standardised counselling session by a trained pharmacist at our institution. The patients and staff members were subsequently surveyed a second time after the session to assess the efficacy of the intervention.

After the intervention, there was an improvement in sun protection awareness, as evidenced by an increase in the proportions of patients and allied health professionals who gave appropriate responses to each question in the questionnaire. Our service encountered several limitations in terms of language barriers as well as time constraints.

Our preliminary results show that the implementation of a specialised sun protection counselling service is potentially useful in the education of patients, so as to reduce the harmful effects of ultraviolet radiation on the skin.

P184

Rapid diagnosis of xeroderma pigmentosum by using a set of recombinant adenoviruses

M. Okura[1], Y. Ishii-Osai[1], T. Hida[1], T. Yamashita[1]

[1]Department of Dermatology, Sapporo Medical University School of Medicine, Sapporo, Japan

Xeroderma pigmentosum (XP) is composed of eight genetic complementation groups (XP-A to -G and XP-V). XP-A to -G are associated with defective nucleotide excision repair (NER) and XP-V with defective translesion DNA synthesis caused by mutations in the DNA polymerase eta. Since protection of the skin from UV exposure can prevent skin cancer in XP patients later in life, early diagnosis of XP is essential. To establish an easy, rapid and precise diagnostic method for XP, we constructed recombinant adenoviruses carrying cDNAs from the eight complementation groups.

Human cDNAs from each group were prepared from normal human fibroblast tig118 and inserted into the HCMV promoter downstream of the adenoviral vector. Each XP cDNA sequence was verified by sequencing all the coding regions. The fibroblasts derived from patients in each XP group were purchased from American CORIELL and other cell banks. The cells derived from each group were infected with the recombinant adenoviruses at the 10–40 pfu/cell. 24 h later cells were irradiated with UVC at 10–50 J/m^2 and aliquots of cells (10^5) were split into 96-well plates and cultured for a further five days. The subsequent number of viable cells was then measured by WST-8 assay and BrdU cell proliferation assay. We also analyzed the cell cycle by flow cytometry.

When the XP-A through XP-G cells were infected with each corresponding adenovirus and irradiated with UVC at 10–50 J/m^2, the number of viable cells clearly increased compared to cells infected with other control adenoviruses. Flow cytometric analysis also detected a decreased subG1 phase in the XP cells infected with the corresponding XP adenoviruses. For the XP-E cell, recovery from UVC irradiation by XP-E adenovirus was lower than that in other groups. However, increased recovery was observed when XPE cells were co-infected with XP-E and XP-A adenoviruses. Recovery of XP-V cells after XP-V adenovirus infection and UV-C irradiation was hardly differentiated by WST-8 assay. Consequently, rather than WST-8 assay, we examined BrdU uptake in the XP-V cells using BrdU cell proliferation assay, and the levels of BrdU uptake specifically and clearly increased and were correctly differentiated.

Our procedure using a set of recombinant adenoviruses may provide a convenient and precise method for diagnosis of the XP complementation groups and variant forms.

 © 2014 John Wiley & Sons A/S. Published by John Wiley & Sons Ltd

P185

Evaluation and effectiveness of a photoprotection composition (sunscreen) on subjects of skin of color

R. Halder[1], I. Rodney[1], M. Munhutu[1], P. Foltis[2], C. Battie[3], C. Oresajo[2]

[1]Dermatology, Howard University College of Medicine, Washington, DC; [2]R & D, L"Oreal USA, Clark, NJ, USA; [3]R & D, L'Oreal Advanced Research, Asnieres-sur-Seine, France

The majority of clinical research on sunscreens has focused on Fitzpatrick skin types I, II and III, and have reported improvements with skin aging and texture. The use of photoprotection in darker skin types has not been fully studied. Due to the paucity of this type of research in skin of color, we sought to evaluate the efficacy of commercially available sunscreens on individuals of ethnic descent (skin of color) who are non-sunscreen users and to see if there is a benefit for prevention of hyperpigmentation. Eighty-nine African-American and Hispanic subjects were prospectively enrolled in the summer months and randomized to using either SPF 30 or SPF 60 sunscreen formulations. Skin examination and patient data collection including triplicate L*A*B chromameter readings were obtained on all subjects at baseline, 4 weeks and 8 weeks. Face and hand photographs using the Canfield Visia CR photography system were obtained at the same intervals. The primary efficacy endpoint was to establish if any improvement occured in the subject's overall condition of skin and evenness compared to baseline after eight weeks of daily sunscreen usage. The secondary efficacy endpoint was to determine if subjects perceived improvements after eight weeks of daily sunscreen use compared to baseline.

Preliminary analysis of Objective quantitative measurements of skin lightening were statisically significant overall in all groups when baseline was compared to eight weeks. Pooled chromameter measurements for all subjects showed significant lightening of the hands at week four, the forehead at week eight, the cheeks at week eight and the neck at week eight with daily use of sunscreen. When stratified by racial groups, African-Americans had significant lightening of the neck and hands at week eight. Hispanics had significant lightening of the forehead, cheeks and hands at weeks four and eight.

Analysis of photographs showed lightening of normal skin and lightening of pre-existing facial and hand pigmentary anomalies with usage of both sunscreen products. Qualitative data indicated overall improved skin condition particularly with regards to dyschromias and tolerability of the product. This study is the first to Objectively evaluate the effect of regular sunscreen usage in skin of color. The results of this preliminary study indicate that regular sunscreen usage in subjects of skin of color Objectively improves dyschromias and lightens the skin in most subjects.

P186

Skin photoaging in Kuwait: a cross sectional study

K. Qasem[1]

[1]Dermatology, MAK Hospital, Jabria, Kuwait

As the population ages, many clinical skin changes characterize old skin. Among harmful environmental factors that contribute to extrinsic aging, repeated exposure to ultraviolet light (UV) is the most significant and is referred to as photoaging. Careful, quantitative and qualitative assessment of photo-aging in human skin in the middle-east in vivo will be critical to a better understanding of these processes which will yield even more clinically useful information to share with our colleagues.

A cross-sectional study of 100 randomly selected people aged 30–80 yrs, using a questionnaire, performing dermatological assessment, and taking photographs.

In this population sample we found a significant correlation of advancing age, skin phototype, outdoor work or leisure, and sunburn with moderate and severe wrinkling scores, skin laxity, and tensile strength (P-value 0.00). Apart from age, a factor predictive of premature skin aging was gender; males were six times more likely to develop wrinkles on the forehead (P-value 0.01), and twice more likely to develop facial roughness (P-value 0.02), and to lose facial skin's tensile strength (P-value 0.02), females were more likely to develop hand wrinkles (P-value 0.04), and twice more likely to develop comedones (P-value 0.03), and facial mottled pigmentation (P-value 0.04). Skin phototype and multi ethnicity we're strongly associated with pigmentation.

LimitationsSmall sample size.

Premature skin aging is common in our population, associated to some extent with factors that can be modified.

As the population ages, many clinical skin changes characterize old skin. Among harmful environmental factors that contribute to extrinsic aging, repeated exposure to ultraviolet light (UV) is the most significant and is referred to as photoaging. Careful, quantitative and qualitative assessment of photo-aging in human skin in the middle-east in vivo will be critical to a better understanding of these processes which will yield even more clinically useful information to share with our colleagues.

A cross-sectional study of 100 randomly selected people aged 30–80 yrs, using a questionnaire, performing dermatological assessment, and taking photographs.

In this population sample we found a significant correlation of advancing age, skin phototype, outdoor work or leisure, and sunburn with moderate and severe wrinkling scores, skin laxity, and tensile strength (P-value 0.00). Apart from age, a factor predictive of premature skin aging was gender; males were six times more likely to develop wrinkles on the forehead (P-value 0.01), and twice more likely to develop facial roughness (P-value 0.02), and to lose facial skin's tensile strength (P-value 0.02), females were more likely to develop hand wrinkles (P-value 0.04), and twice more likely to develop comedones (P-value 0.03), and facial mottled pigmentation (P-value 0.04). Skin phototype and multi ethnicity we're strongly associated with pigmentation.

Limitations: Small sample size.

Premature skin aging is common in our population, associated to some extent with factors that can be modified.

P187

To assess the relative impact of UVA and UVB protection on anti-darkening efficacy during day-to-day sun exposure

A. A. Vaidya[1], B. Sharma[2], N. Surendra[2], R. Rajagopal[3], C. Doraiswamy[2], V. Gadgil[4]

[1]Skin Care Group, Unilever R&D; [2]Clinical Science, Unilever R&D; [3]Enabing Science Group, Unilever R&D; [4]Strategic Science Group, Unilever R&D, Bangalore, India

Skin darkening upon sun exposure is commonly perceived by consumers. UVA radiations predominantly stimulate skin darkening [[1,2]]. Hence, skin care product need to deliver superior UVA protection, in addition to SPF. Consumers get

© 2014 John Wiley & Sons A/S. Published by John Wiley & Sons Ltd

exposed to random but cumulative sunlight dose during their routine outdoor day-to-day activities. Under such activities, relative impact of UVA and UVB protection on the long term anti-darkening efficacy is not studied so far.
To assess the relative impact of UVA and UVB protection on anti-darkening efficacy, under day-to-day sun exposure over two months time.
Skin creams containing different levels of UV-A and UV-B sunscreens were applied twice a day on the pre-designated sites on volar forearm, for 2 months. Subjects were free to continue their routine outdoor day-to-day activities. Their total sun exposure was measured using a portable UV device. Recorded average daily sun exposure was equivalent to ~5 Joule of UVA. Visual skin color change (i.e. anti-darkening) from unprotected base line was measured at the end of 2 months, using spectrophotometer and visual assessment.
Study was carried out with the creams containing two different levels of UVA sunscreen (0.4% & 1.2% of 4-t-butyl, 4′-methoxydibenzoylmethane) and UVB sunscreen (0.75% & 2.4% of Octyl methoxycinnamate). The results showed that composition containing 1.2% UVA sunscreens showed significant anti-darkening efficacy over cream containing NO sunscreens. In contrary, compositions containing 2.4% UVB sunscreens did not show significant anti-darkening efficacy.
The data shows that the skin cream containing 1.2% UVA sunscreen provides superior protection against darkening in comparison to skin cream containing 2.4% UVB sunscreen. UVA protected skin resulted as significantly lighter compared to daily sun exposed skin after 2 months.

[1] UVA Phototransduction Drives Early Melanin Synthesis in Human Melanocytes - Najera, J. A. *Current Biology* 2011, 21 (22), 1–6.
[2] UVA and endogenous photosensitizers–the detection of singlet oxygen by its luminescence Bäumler W et al. Photochem Photobiol Sci. 2012 Jan;11(1):107–17.

 © 2014 John Wiley & Sons A/S. Published by John Wiley & Sons Ltd

LOBBY LEVEL
ISLAND / TOWER BALLROOM

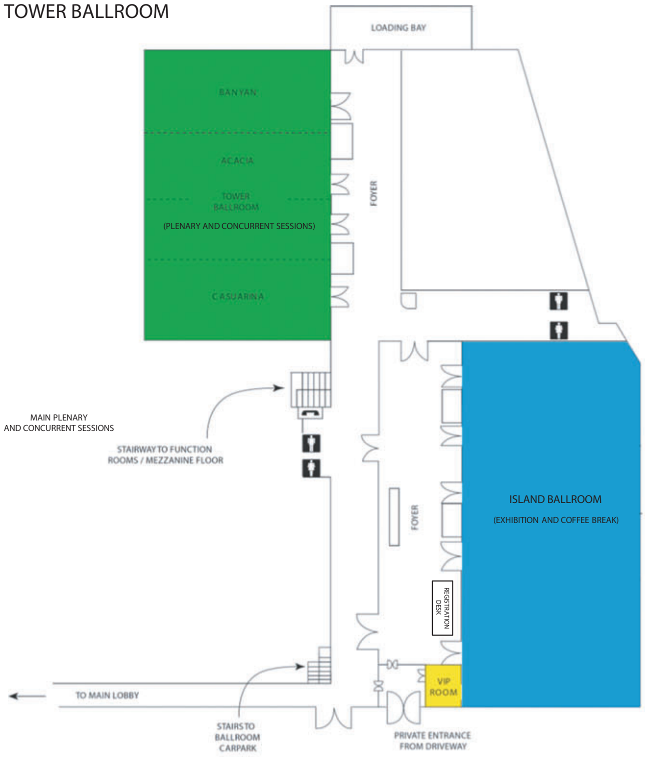

MEZZANINE LEVEL

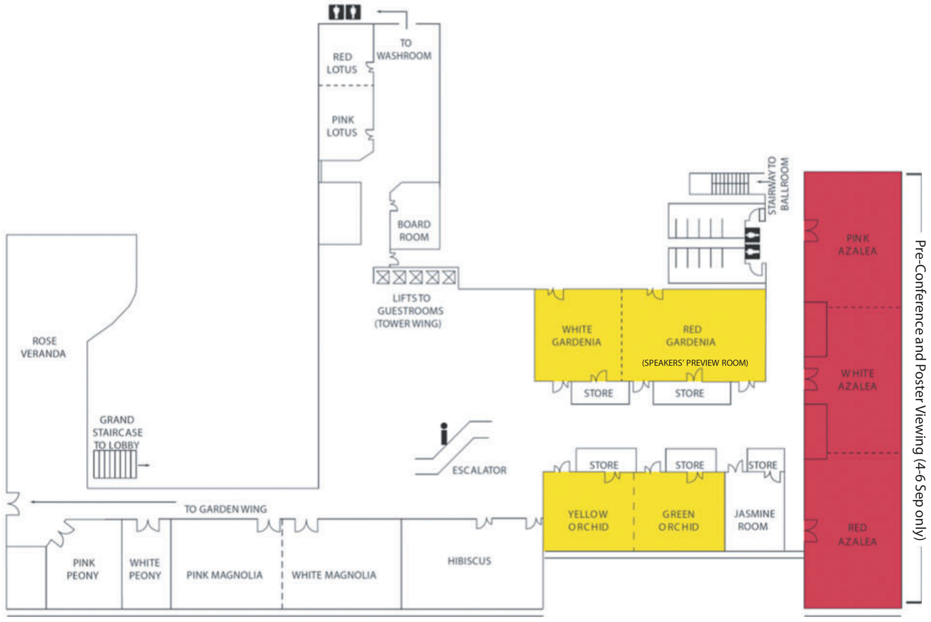

© 2014 John Wiley & Sons A/S. Published by John Wiley & Sons Ltd
