# Supplementary material for: Therapeutic efficacy of cell-based therapy in vitiligo: a research letter systematically reviewed using meta-analysis
Source: Arch Dermatol Res. 2024 May 22;316(5):198. doi: 10.1007/s00403-024-02920-6 (PMC11111487; doi:10.1007/s00403-024-02920-6)
Supplement: Supplementary file 1 — Supplementary file1 (ZIP 24195 KB) [file 403_2024_2920_MOESM1_ESM.zip › Studies were included/Razmi 2016.pdf]

Main Plenary Sessions

O01
Patterns of biological therapy use in the management of psoriasis: cohort study from the British Association of Dermatologists' Biologic Interventions Register

I.Y.K. Iskandar,[1] R. Warren,[2] I. Evans,[2] K. McElhone,[2] C.M. Owen,[3] A.D. Burden,[4] C. Smith,[5] N.J. Reynolds,[6] D.M. Ashcroft[1] and C.E.M. Griffiths[2]

[1]*Centre for Pharmacoepidemiology and Drug Safety, Manchester Pharmacy School, The University of Manchester, Manchester, U.K.;* [2]*Dermatology Centre, Salford Royal NHS Foundation Trust, The University of Manchester, Manchester Academic Health Science Centre, Manchester, U.K.;* [3]*Department of Dermatology, East Lancashire Hospitals NHS Trust, Royal Blackburn Hospital, Blackburn, U.K.;* [4]*Department of Dermatology, Western Infirmary, Glasgow, U.K.;* [5]*St. John's Institute of Dermatology, Guy's and St Thomas' NHS Foundation Trust, London, U.K. and* [6]*Dermatological Sciences, Institute of Cellular Medicine, Medical School, Newcastle University, and Department of Dermatology, Royal Victoria Infirmary, Newcastle Hospitals NHS Foundation Trust, Newcastle upon Tyne, U.K.*

Treatment modifications, including dose escalations and reductions, switches, discontinuations and restarts of biological therapies, are often necessary and important in the 'real-world' management of moderate-to-severe psoriasis. We examined the treatment utilization patterns of adalimumab, etanercept and ustekinumab among biologics-naive and non-naive patients with psoriasis enrolled in the British Association of Dermatologists' Biologic Interventions Register (BADBIR). We also assessed the patterns of use of conventional systemic therapies in combination with biological therapies in BADBIR. The cohort study included adults with chronic plaque psoriasis who were registered with BADBIR and followed up for at least 12 months. Treatment modifications were assessed during the first year of therapy. The time–trend method, comparing the annual cumulative dose the patients received with the annual recommended cumulative dose per product prescribing information based on the National Institute for Health and Care Excellence guidelines, was used to assess dosing patterns. Bridging therapy was defined as conventional systemic therapies coprescribed during the induction of the biological therapy ($\leq$ 120 days), while rescue therapy was defined as additional medication required following the induction phase (> 120 days). In total, 2980 patients with a mean age of 45·9 years were included, of whom, 79·2% were biologic naive. Data for patients on adalimumab (*n* = 1675), etanercept (*n* = 996) and ustekinumab (*n* = 309) were available. Over 12 months, 77·4% of patients continued with the biological therapy, 2·6% restarted therapy after a break of at least 90 days, 2·5% discontinued and 17·5% switched to an alternative biological therapy. Most patients (85·7%) received the recommended cumulative dose of the biological therapy, 6·2% were exposed to a lower cumulative dose and 8·1% were exposed to a higher cumulative dose. Specifically, 4·5%, 11·4% and 17·7% of adalimumab, etanercept and ustekinumab patients ($P < 0·01$), respectively, were exposed to a higher cumulative dose. A total of 749 (25·1%) patients used conventional systemic therapies in combination with biological therapy at some stage; methotrexate was the most commonly coprescribed therapy (*n* = 458; 61·2%). Of those using combination therapy, 454 (60·6%) continued the use of the conventional systemic therapy for > 120 days after the start of the biological therapy, whereas 160 (21·4%) and 152 (20·3%) used the conventional systemic therapy as bridging and rescue therapy, respectively. In summary, one-fifth of patients starting biological therapy in BADBIR experienced treatment modifications within the first year. Modifications included dose escalations and reductions; restarting treatment after a break; and discontinuing or switching to an alternative biological therapy. Conventional systemic therapies, particularly methotrexate, are commonly used concurrently with biological therapies; this should be considered when evaluating treatment response and adverse events.

O02
Medication beliefs are associated with medication adherence in patients with psoriasis using systemic therapies: findings from the Investigating Medication Adherence in Psoriasis study

R. Thorneloe, C.E.M. Griffiths, R. Emsley, D.M. Ashcroft and L. Cordingley

University of Manchester and Manchester Academic Health Science Centre, Manchester, U.K.

Medication adherence is a major barrier to optimizing the effectiveness of available therapies; however, few high-quality studies have examined adherence to systemic therapies used for psoriasis. We assessed whether patients with psoriasis can be categorized into groups with similar medication beliefs, and examined the relationship between belief group membership with adherence to systemic therapy and psychological distress. Cross-sectional data from 565 patients with moderate-to-severe psoriasis using a systemic therapy for $\leq$ 2 years were collected from 35 dermatology clinics across England: 68·5% prescribed a biologic and 31·5% prescribed a conventional systemic therapy. A questionnaire assessed patients' medication beliefs (Beliefs about Medicines Questionnaire), psychological distress (Hospital Anxiety and Depression Scale) and medication adherence [Medication Adherence Report Scale (MARS)], with a score $\leq$ 38 on the MARS indicating nonadherence. The optimal number of medication belief groups was identified using latent profile analysis (LPA). In total, 21·5% were classified as nonadherent to systemic therapy, with a

higher proportion of patients using a conventional systemic classified as nonadherent (31·3%) compared with those using a biologic (16·8%). The LPA model-fit statistics supported a three-group model; each group reported strong and positive beliefs in the necessity of their systemic therapy and perceived treatment controllability, but differed in their level of concern about systemic therapies and medicines in general; group 1 (22·0%) reported the strongest concerns, followed by group 2 (64·0%), with group 3 (14·0%) reporting the weakest concerns. The proportion of patients classified as nonadherent significantly differed by belief group ($\chi^2 = 9{\cdot}4$, $P = 0{\cdot}01$), with nonadherence highest in group 1 (28·7%), followed by group 2 (21·3%) and lowest in group 3 (10·4%). There were significant differences in level of anxiety ($H = 7{\cdot}71$, $P = 0{\cdot}02$) and depression ($H = 19{\cdot}1$, $P < 0{\cdot}01$) between the three belief groups, with group 1 reporting the highest level of anxiety [median 7·5, interquartile range (IQR) 3·25–11·0] and depression (median 6·00, IQR 2·00–9·75), compared with group 2 (median 6·00, IQR 3·25–9·00, and median 4·00, IQR 1·00–7·00, respectively) and group 3 (median 6·00, IQR 2·00–9·00, and median 3·00, IQR 1·00–7·00, respectively). Significant proportions of patients with psoriasis using systemic therapies reported nonadherence. Patients can be categorized into groups with similar medication beliefs. Patients with conflicting medication beliefs, holding strong beliefs in medication necessity *and* strong medication concerns, reported higher nonadherence and higher levels of psychological distress. Assessing medication beliefs may help identify those most likely to experience self-management difficulties and have poorer medication outcomes.

O03
The primary-care management of hidradenitis suppurativa in the U.K.: an evaluation of patient pathways and healthcare resource use using The Health Improvement Network database

A. Bewley,[1] B. Malcolm[2] and Prashanti Shah[3]
[1]*Barts Health NHS Trust, London, U.K.;* [2]*Northern Devon Healthcare Trust, Barnstaple, U.K. and* [3]*Former AbbVvie Contractor, Maidenhead, U.K.*

Hidradenitis suppurativa (HS) is a painful, debilitating skin condition that affects an estimated 1% of the U.K. population. HS can adversely affect patients' quality of life, work productivity and emotional well-being. This study aimed to describe the patient profile, treatment pathways and resource use associated with HS management in U.K. primary care. We conducted a retrospective population-based cohort study using The Health Improvement Network (THIN) database, which contains longitudinal primary-care patient records collected from U.K. general practices (GPs). Patients with an HS READ code (M25y111/M25y100) between 1 January 2009 and 31 December 2013 were identified. Data on patient demographics, primary-care visits, medications and secondary-care referrals from the first recorded skin symptom until the data extraction date (August 2015) were obtained for each patient. 'HS diagnosis' (date of first recorded HS READ code) ranged from January 1957 to September 2014. The number of patients with data available for each analysis is stated where data are missing. Of 12 524 patients identified, 75·0% were female, with a mean ± SD age of 35·9 ± 12·9 years at HS diagnosis and 41·6 ± 13·9 years at data extraction. A variety of skin conditions/symptoms were coded prior to HS diagnosis, including folliculitis, acne vulgaris, furuncles, boils, abscesses and pilonidal cysts, and there was a mean ± SD of 4·2 ± 7·1 years ($n = 9204$) from first coded skin symptom to HS diagnosis. Endocrine/metabolic disorders (20·0%), cardiovascular disease (12·7%) and depression (12·4%) were the most common comorbidities. Rates of smoking/obesity (body mass index ≥ 30 kg m^{-2}) were higher in this cohort compared with published statistics for the U.K. general population: current smokers 43·5% vs. 22·0% ($P < 0{\cdot}01$) and obesity 56·7% vs. 27·6% ($P < 0{\cdot}01$). Patients had a mean ± SD of 8·9 ± 9·8 ($n = 10\ 372$) primary-care visits in the 12 months before and 8·9 ± 10·4 ($n = 11\ 865$) in the 12 months after HS diagnosis. Few patients (3·3%; $n = 413$) had a secondary-care referral specifically coded as HS or skin symptom related. The most common medications prescribed (of those considered relevant to HS) were antibiotics (flucloxacillin 80·2%, erythromycin 55·5%, oxytetracycline 26·6%) and prednisolone (26·7%). Patients with HS had high rates of smoking and obesity and were very frequent primary-care attendees, despite being a predominantly working-age cohort. This highlights the morbidity associated with HS and the considerable burden it places on patients and the National Health Service. The range of coded skin conditions/symptoms suggests that HS is not well recognized by GPs, and many alternative diagnoses are considered before HS. This leads to significant delays in specialist referral and initiation of appropriate treatments.

O04
The Patient-Oriented Eczema Measure of eczema severity in young children: responsiveness and minimal clinically important difference

D.M. Gaunt,[1] C. Metcalfe[1] and M. Ridd[2]
[1]*Bristol Randomised Trials Collaboration, Bristol, U.K. and* [2]*University of Bristol, Bristol, U.K.*

The Patient-Oriented Eczema Measure (POEM) has been recommended as the core patient-reported outcome measure for trials of eczema treatments. This recommendation is more likely to be followed by investigators planning trials of eczema treatments if the POEM's responsiveness to change can be demonstrated, and the size of a minimal clinically important difference (MCID) can be established across a range of eczema patient populations. Our aim was to assess the responsiveness to change and determine the MCID of the POEM in young children using data from the Choice of Moisturiser for Eczema Treatment (COMET) feasibility trial. This randomized trial was designed to determine the feasibility of recruiting young children (from 1 month to < 5 years of age) with eczema from primary care into a clinical trial testing emollient treatments. Parents completed weekly POEM and monthly Parent Global Assessment (PGA) measures. Responsiveness to change by repeated administrations of the POEM was investigated in

© 2016 The Authors
BJD © 2016 British Association of Dermatologists

relation to parent-recalled change using the PGA. Five methods of determining the MCID of the POEM were employed: (i) the mean change between baseline and follow-up in POEM scores for the subgroup of children identified as minimally improved (within-patient score change); (ii) the mean difference in POEM change scores between subgroups of children identified as minimally improved and unchanged (between-patient score change); (iii) the POEM score that optimally discriminates between minimally improved and unchanged subgroups of children (sensitivity and specificity method); (iv) the change in POEM scores between baseline and follow-up, divided by the SD of the baseline scores (effect-size estimate); and (v) half of the SD of the baseline distribution of POEM scores. The minimally improved and unchanged subgroups were identified by parental response to the PGA. Successive POEM scores were found to be responsive to change in eczema severity. The MCID of the POEM change score, in relation to a slight improvement in eczema severity as recalled by parents on the PGA, estimated by the within-patient score change (4·27), the between-patient score change (2·89), and the sensitivity and specificity method (3·00), was similar to half the SD of the POEM baseline scores (2·95) and the effect-size estimate (2·50). In conclusion, the POEM as applied to young children is responsive to change and the MCID is around 3. This study will encourage the use of POEM and aid in determining sample sizes for future randomized controlled trials of treatments for eczema in young children.

O05
Doxycycline vs. prednisolone for initial treatment of bullous pemphigoid: a pragmatic noninferiority randomized controlled trial

H.C. Williams,[1] F. Wojnarowska,[2] G. Kirtschig,[3] J. Mason,[4] T. Godec,[5] E. Schmidt,[6] J.R. Chalmers,[1] M. Childs,[1] S. Walton,[7] K. Harman,[8] A. Chapman,[9] D. Whitham,[1] A. Nunn[5] and UKDCTN BLISTER Study Group[10]

[1]*University of Nottingham, Nottingham, U.K.,* [2]*University of Oxford, Oxford, U.K.,* [3]*University of Tübingen, Tübingen, Germany,* [4]*University of Warwick, Warwick, U.K.,* [5]*Medical Research Council Clinical Trials Unit at UCL, London, U.K.,* [6]*University of Lübeck, Lübeck, Germany,* [7]*Hull Royal Infirmary, Hull, U.K.,* [8]*Leicester Royal Infirmary, Leicester, U.K.,* [9]*Queen Elizabeth Hospital, Greenwich, U.K. and* [10]*U.K. Dermatology Clinical Trials Network, Nottingham, U.K.*

Bullous pemphigoid (BP) is associated with increased morbidity and mortality in the elderly, which may be related to treatment with oral prednisolone. Tetracyclines are often used for BP treatment, but they have not been evaluated in a large randomized controlled clinical trial (RCT). We hypothesized that doxycycline is noninferior to prednisolone by up to 25% for short-term blister control but superior by at least 20% for long-term safety. In this pragmatic multicentre two-armed parallel-group RCT, adults with BP (at least three blisters at two sites and positive IgG and/or C3 immunofluorescence) were allocated, using online randomization, to initial doxycycline treatment (200 mg daily) or prednisolone (0·5 mg kg^{-1} daily). Up to 30 g weekly of potent topical corticosteroids was permitted for weeks 1–3. After 6 weeks, clinicians could switch treatments or alter the prednisolone dose as normal. Primary outcomes were (i) proportion with at most three blisters at 6 weeks (investigator blinded) and (ii) proportion with severe, life-threatening and fatal treatment-related events at 52 weeks. Analysis used regression models adjusting for baseline severity, age and Karnofsky score, with missing data imputed. Secondary outcomes included blister control after 6 weeks, relapses, related adverse events and quality of life. An economic evaluation included costs and quality-adjusted life-years from a National Health Service perspective. We randomized 132 patients to doxycycline and 121 to prednisolone from 54 U.K. and seven German secondary-care centres. The mean age was 77·7 years and baseline severity was mild 31·6%, moderate 39·1% and severe 29·3%. For those starting doxycycline, 83 of 112 (74·1%) had at most three blisters at 6 weeks, compared with 92 of 101 (91·1%) for prednisolone, a difference of 18·6% in favour of prednisolone [upper limit of 90% confidence interval (CI) 26·1%, within predefined acceptable margin], using modified intention-to-treat (mITT) analysis. Per protocol and sensitivity analyses showed similar results. Related severe, life-threatening and fatal events at 52 weeks were 18·5% for those started on doxycycline and 36·6% for those on prednisolone (mITT analysis), an adjusted difference of 19·0% (90% CI 7·9–30·1%; $P < 0·01$) in favour of doxycycline. Secondary outcomes showed consistent findings. The cost-effectiveness of treatments was similar for mild and moderate disease but not for severe disease. We conclude that a strategy of starting patients with BP on doxycycline is noninferior to standard treatment with oral prednisolone for short-term blister control and considerably safer in the long term. Funding: National Institute for Health Research Health Technology Assessment Programme.

O06
Preliminary results of a randomized controlled trial of silk therapeutic clothing for the management of eczema in children (CLOTHES Trial)

K. Thomas,[1] L.E. Bradshaw,[1] J.M. Batchelor,[1] S. Lawton,[2] E. Harrison,[1] A. Ahmed,[1] R.H. Haines,[2] T. Dean,[3] N. Burrows,[4] I. Pollock,[5] H. Buckley,[6] H.C. Williams,[1] E.J. Mitchell,[1] F. Cowdell,[7] S.J. Brown,[8] T.H. Sach[9] and A.A. Montgomery[1]

[1]*University of Nottingham, Nottingham, U.K.,* [2]*Nottingham University Hospitals NHS Trust, Nottingham, U.K.,* [3]*University of Portsmouth, Portsmouth, U.K.,* [4]*Cambridge University Hospitals NHS Foundation Trust, Cambridge, U.K.,* [5]*Royal Free London NHS Foundation Trust, London, U.K.,* [6]*Portsmouth Hospitals NHS Trust, Portsmouth, U.K.,* [7]*University of Hull, Hull, U.K.,* [8]*University of Dundee, Dundee, U.K. and* [9]*University of East Anglia, Norwich, U.K.*

The role of clothing in the management of atopic eczema (AE) is poorly understood. This study evaluated the effectiveness of silk therapeutic clothing when used in addition to usual care over a period of 6 months for children with AE. This was a parallel-group randomized controlled trial of

© 2016 The Authors
BJD © 2016 British Association of Dermatologists

children (aged 1–15 years) with moderate-to-severe AE recruited from secondary and primary care in five U.K. centres (November 2013 to May 2015). Randomization (1 : 1) to usual care or usual care plus 100% sericin-free, knitted silk garments (DermaSilk™ or Dreamskin™) was done with a secure web-based system and stratified by age and recruiting centre. Three sets of garments were supplied to each participant, to be worn day and night for up to 6 months. The primary outcome was assessed at baseline and 2, 4 and 6 months by nurses blinded to treatment allocation using the Eczema Area Severity Index (EASI), which was log-transformed for analysis. Secondary outcomes included patient-reported eczema severity [Patient-Oriented Eczema Measure (POEM)] and number of skin infections. Three hundred children were randomized (42% female, mean age 5 years). EASI was assessed at least once at follow-up for 282 of 300 (94%) children ($n = 141$ in each group). The garments were worn for at least 50% of the time by 82% of children in the silk clothing group. Geometric mean EASI scores at baseline and 2, 4 and 6 months were 8·4, 6·6, 6·0 and 5·4, respectively, for usual care and 9·2, 6·4, 5·8 and 5·4, respectively, for silk clothing. There was no evidence of any difference between the groups in EASI score averaged over all follow-up visits [ratio of geometric means 0·95, 95% confidence interval (CI) 0·85–1·07]. This CI is approximately equivalent to a difference of −1·5 to 0·5 in the original EASI scale units. There were small differences in the POEM score: the difference in means averaged over all follow-up visits was −2·4 (95% CI −3·5 to −1·3). The numbers of participants reporting at least one skin infection that required antibiotic/antiviral treatment were 39 (28%) and 36 (25%) for usual care and silk clothing, respectively. These preliminary results suggest that silk clothing is unlikely to provide additional benefit to usual care. Small differences in the unblinded secondary outcome (POEM) are most likely to be the result of response bias. Trial registration: Current Controlled Trials ISRCTN77261365. Funding: National Institute for Health Research Health Technology Assessment programme (ref.: 11/65/01).

O07
Assessment of human leucocyte antigen Cw6 genotype and correlation to ustekinumab response in a large cohort of patients with moderate-to-severe psoriasis

M. Plotnick, K. Li, C. Huang, Y. Wasfi, B. Randazzo, S. Li, P. Szapary, K. Campbell and C. Brodmerkel

Janssen Research and Development, Spring House, PA, U.S.A.

The link between the human leucocyte antigen (HLA)-Cw6 allele in patients with psoriasis and improved clinical response to ustekinumab (UST) therapy was reported previously. The aim of this retrospective analysis was to determine the association of HLA-Cw6 status and response to UST in a large, well-controlled clinical-trial population. DNA was collected from approximately 600 North American participants in the UST Phoenix 1, Phoenix 2 and ACCEPT Ph3 psoriasis clinical trials. HLA-Cw6 genotype was assessed by single-nucleotide polymorphism–chip imputation then correlated to Psoriasis Area and Severity Index (PASI) and Physicians, Global Assessment responses. Association between HLA-Cw6 status and efficacy (short and long term) of UST or likelihood for dose escalation was evaluated. The prevalence of HLA-Cw6 was 44·6%. The HLA-Cw6 genotype in this combined population was associated with longer disease duration and earlier age of disease onset. Both HLA-Cw6-positive and HLA-Cw6-negative patients demonstrated relatively high response rates to UST [75% reduction in PASI (PASI 75) responses at week 24 were 86% and 76%, respectively]. A modestly higher percentage of HLA-Cw6-positive patients achieved PASI 50, PASI 75, PASI 90 and PASI 100 at weeks 4, 12, 24 and 28, respectively, vs. HLA-Cw6-negative patients. The largest delta between positive and negative patients (18%) was observed for PASI 75 response at week 12, with smaller differences noted at later time points for PASI 90 (12% at week 24) and PASI 100 (10% at week 28) response rates. HLA-Cw6-positive patients had modestly higher long-term efficacy rates than those who were HLA-Cw6 negative, with statistical significance reached for PASI 75 response rates (83% vs. 64%, respectively) at the 5-year final efficacy assessment in Phoenix 1. Lastly, a larger percentage of HLA-Cw6-negative patients underwent a shortened dosing interval and/or dose escalation through year 5 in Phoenix 2. Fifty-seven percent of HLA-Cw6-positive patients did not require dose escalation vs. 50% of HLA-Cw6-negative patients. Among patients who received initial treatment with UST 45 mg, 41% of HLA-Cw6-negative and 32% of HLA-Cw6-positive patients required both dose escalation and dose interval adjustment. While a differential response to UST is evident in HLA-Cw6-positive vs. HLA-Cw6-negative patients, the difference is modest, particularly for response rates of more complete responses (PASI 90 and PASI 100); thus, the clinical utility of this marker as a predictor of response to UST appears to be limited. Funding: Janssen R&D, LLC supported this research.

O08
Risk of serious infections in patients with psoriasis on biological therapies: a systematic review and meta-analysis

Z. Yiu,[1] L. Exton,[2] Z. Jabbar-Lopez,[3] M.F. Mohd Mustapa,[2] E. Samarasekera,[4] D. Burden,[5] R. Murphy,[6] C.M. Owen,[7] R. Parslew,[8] V. Venning,[9] D.M. Ashcroft,[10] C.E.M. Griffiths,[1] C. Smith[11] and R. Warren[1]

[1]*Dermatology Centre, Salford Royal NHS Foundation Trust, The University of Manchester, Manchester Academic Health Science Centre, Manchester, U.K.,* [2]*British Association of Dermatologists, London, U.K.,* [3]*Dermatological Sciences, Institute of Cellular Medicine, Medical School, Newcastle University, and Department of Dermatology, Royal Victoria Infirmary, Newcastle Hospitals NHS Foundation Trust, Newcastle upon Tyne, U.K.,* [4]*National Clinical Guideline Centre, Royal College of Physicians of London, London, U.K.,* [5]*Department of Dermatology, Western Infirmary, Glasgow, U.K.,* [6]*Sheffield University Teaching Hospitals and Sheffield Children's Hospitals, Sheffield, U.K.,* [7]*Department of Dermatology, East Lancashire Hospitals NHS Trust, Royal Blackburn Hospital, Blackburn, U.K.,* [8]*Department of*

© 2016 The Authors
BJD © 2016 British Association of Dermatologists

Dermatology, Royal Liverpool and Broadgreen Hospitals NHS Trust, Liverpool, U.K., [9]Department of Dermatology, Churchill Hospital, Oxford, U.K., [10]Centre for Pharmacoepidemiology and Drug Safety, Manchester Pharmacy School, The University of Manchester, Manchester, U.K. and [11]St John's Institute of Dermatology, Guy's and St Thomas' NHS Foundation Trust, London, U.K.

The risk of infections leading to significant morbidity and/or mortality in association with biological therapies for psoriasis is a key concern, but a comprehensive evaluation of this is lacking. We have performed a systematic review and meta-analysis of serious infection in people taking any licensed biological therapy for psoriasis compared with those taking placebo or nonbiological therapy. We searched, up to 29 September 2015, the PubMed, Medline, Embase and Cochrane databases for randomized clinical trials (RCTs) or prospective cohort studies involving etanercept, infliximab, adalimumab, ustekinumab or secukinumab for the treatment of psoriasis that reported on serious infection. The results were meta-analysed using RevMan 5·3, and study quality and risk of bias assessed using National Institute for Health and Care Excellence checklists and the Grading of Recommendations Assessment, Development and Evaluation criteria. Heterogeneity was assessed using the I^2 statistic. Data from 32 RCTs (13 359 participants) and one cohort study (4993 participants) were included. Only three studies clearly defined the outcome within the published report. No significant heterogeneity was found for any of the comparisons, while the individual studies ranged from having a low to a very high risk of bias. Eight RCTs (two in children) reported no serious infection in either of the study arms. In adults, low to very low-quality RCT data showed no significant difference between any biological therapy and placebo at weeks 12–16 [etanercept pooled risk ratio (RR) 0·63, 95% confidence interval (CI) 0·22–1·76; adalimumab RR 0·77, 95% CI 0·25–2·33; ustekinumab RR 0·49, 95% CI 0·14–1·80; secukinumab RR 0·64, 95% CI 0·36–1·14] and weeks 20–30 (infliximab RR 1·30, 95% CI 0·15–11·01). Cohort study data of low quality suggested that adalimumab (adjusted hazard ratio 2·52, 95% CI 1·47–4·32), but not infliximab, etanercept or ustekinumab, was associated with a significantly higher risk of serious infection compared with acitretin and/or phototherapy in adults. In summary, underpowered RCT data did not indicate any increased short-term risk of serious infection in association with any of the licensed biological therapies compared with placebo in adults with psoriasis. Observational data from one study suggests that adalimumab is associated with a higher risk of serious infections compared with acitretin and/or phototherapy in adults. Work on harmonization of outcome definition, longer head-to-head comparative RCTs, more studies involving children and adequately powered and well-designed observational studies are needed to inform the uncertainty about the risk of serious infection when biological therapies are used in patients with psoriasis. PROSPERO number: 2015:CRD42015017538.

O09
British Association of Dermatologists national audit on nonmelanoma skin cancer excision 2014

D. Keith, D. de Berker, A. Bray, S. Cheung, A. Brain and M.F. Mohd Mustapa

British Association of Dermatologists, London, U.K.

Diagnosis and management of nonmelanoma skin cancer (NMSC) represents a large part of the dermatology workload, and complete excision is a required surgical standard for treatment. We conducted an audit of the surgical practice of U.K. dermatologists in the treatment of NMSC. U.K. dermatologists collected data on 10 consecutive nonmicrographic excisions for NMSC. Data were collected on site, preoperative diagnosis, histological diagnosis, proximity to previous scars, and histological deep and peripheral margins. A total of 227 responses from 135 centres reported 2739 excisions. Excisions from the head and neck accounted for 58·3% of cases. The mean ± SD tumour diameter was 10·6 ± 6·9 mm (maximum 130·0 mm) and 96·7% of cases were primary excisions with 3·3% re-excisions. Basal cell carcinomas (BCCs) accounted for 79·1% (n = 2167) of total cases and squamous cell carcinomas (SCCs) 17·9% (n = 491). Of the suspected BCCs, 94·4% (n = 2045) were confirmed histologically; for suspected SCCs, 66·8% were confirmed histologically (n = 328). Similar proportions of BCC and SCC cases were within 10 mm of a previous excision. BCC lateral margins were clear in 98·3%; deep margins were clear in 99·2%. SCC lateral margins were clear in 98·4%. SCC deep margins were clear in 97·1%. The reported surgical complication rate in the audit was 3·4%. The majority of excisions for NMSC are for BCC and SCC. Our figures for diagnostic accuracy are at the upper range of previously published figures. Most patients were not followed up in secondary care; hence, complication rates may be under-reported.

O10
Chemoprevention of cutaneous squamous cell carcinoma with acitretin in renal transplant recipients: a case–control analysis

R. Anand,[1] A. Cronin,[2] S. Frame,[1] R. Hung,[1] K. Bramham[1] and M. Wain[3]

[1]*Guy's and St Thomas' NHS Foundation Trust, London, U.K.,* [2]*King's College London, London, U.K. and* [3]*St John's Institute of Dermatology, London, U.K.*

Organ transplant recipients are up to 200 times more likely to develop cutaneous squamous cell carcinoma (SCC) than age-matched general populations. Nonmelanoma skin cancer is now a leading cause of death in long-term renal transplant recipients (LRTRs). Systemic retinoids have shown promising secondary preventative effects against the development of SCC; however, currently there are no consensus guidelines on their use in LRTRs. There are few published data regarding whether they adversely affect transplant function, liver function or lipid profile in LRTRs. We aimed to determine the safety and efficacy of acitretin as a chemopreventative agent against the

© 2016 The Authors
BJD © 2016 British Association of Dermatologists

development of SCC in LRTRs. Retrospective data were collected from a large cohort ($n = 469$) of LRTRs (> 7 years) attending an annual review transplant clinic, of whom 23% ($n = 108$) had been diagnosed with NMSC [70 (65%) with SCCs]. We identified patients ($n = 12$) on treatment with acitretin for the prevention of SCC. We matched these patients to an equal number of controls by age, total years from transplant and Fitzpatrick skin type. We compared glomerular filtration rate (GFR), liver function tests (LFTs) and lipid profile 1 year before and 1 ($n = 12$), 3 ($n = 11$) and 5 ($n = 9$) years after commencing acitretin. We compared the total number of SCCs in the 5 years prior to starting acitretin treatment with the 5 years after starting acitretin. A Wilcoxon signed-rank test was used to compare blood chemistry values before and after acitretin treatment within cases, and a Mann–Whitney test was used to compare differences between cases and controls. Serum total cholesterol concentrations and low-density lipoprotein were significantly lower (both $P = 0{\cdot}01$) in patients prescribed acitretin at 5 years post-treatment compared with baseline measurements ($n = 9$). However, there were no other statistically significant differences in lipid profile, GFR or LFTs at baseline compared with 1, 3 and 5 years after starting treatment, within cases or comparing cases and controls. During the 5 years after starting acitretin the median number of new SCCs per patient was 2 (range 0–4), significantly lower than the median for 5 years prior to starting treatment of 6 (range 3–10; $n = 9$; $P = 0{\cdot}01$). In our cohort, treatment with acitretin did not adversely affect renal transplant function, liver function or lipid profiles, and was associated with a significant reduction in the total number of new SCCs during the 5-year follow-up. Introduction of acitretin should be considered in carefully selected LRTRs with multiple SCCs who are under regular dermatology surveillance and have adequate renal graft function.

O11

Clinical and immunological outcomes of treatment with rituximab vs. combination of rituximab and intravenous cyclophosphamide in treatment of refractory pemphigus: a pilot randomized double-blind study

U. Shraddha,[1] S. Dogra,[2] S. Handa,[2] K. Vinay,[2] B. Saikia[2] and N. Joshi[2]

[1]*Chitwan Medical College and Teaching Hospital, Chitwan, Nepal and* [2]*Post Graduate Institute of Medical Education and Research, Chandigarh, India*

Both intravenous (IV) rituximab and IV cyclophosphamide have been a part of the management of pemphigus; however, the combination of IV cyclophosphamide and IV rituximab has not been studied. This was a prospective, double-blind, randomized controlled trial (NCT01974518) in a tertiary-care centre. Twenty-two patients who fulfilled the international consensus criteria for refractory pemphigus were recruited and randomized into two groups. Patients in both groups received rituximab 1 g on days 1 and 15. Patients in group 2 were given additional IV cyclophosphamide 750 mg on days 2 and 16, whereas patients in group 1 received placebo on these days. Clinical activity was assessed by a blinded investigator on days 15 and day 30 and monthly thereafter for 9 months. Immunological evaluation was done on months 3, 6 and 9 for naive B cells, memory B cells and transitional B cells. Thirteen patients completed the study; the minimum length of follow-up was 20 weeks. No patients were lost to follow-up. Time to disease control was achieved significantly earlier in group 2 ($P = 0{\cdot}04$). End of consolidation phase, time to partial remission and time to complete remission on and off therapy were all achieved earlier for group 2, although this was not statistically significant. The mean dose of steroids at 20 weeks in group 1 was significantly higher than in group 2 ($P = 0{\cdot}01$). There was no significant difference in the incidence of adverse effects. Baseline B-cell levels were comparable in both groups. These decreased to undetectable levels 3 months after therapy. B-cell repopulation started at about 6 months in both groups. The average B-cell levels at the end of 6 and 9 months for both groups were comparable. The ratio of naive to memory B cells was 1·4 : 1 and 1·3 : 1 in groups 1 and 2, respectively, at baseline. However, at the end of 9 months, the ratio of naive to memory B cells was 8·94 : 1 for group 1 and 24·06 : 1 for group 2. Combining rituximab and IV cyclophosphamide in refractory pemphigus was safe and did not show any increase in adverse effects. Clinical improvement was observed earlier and a lower steroid dosage was required. Immunological evaluations showed a much higher ratio of naive to memory B cells with this combination. In other studies this higher ratio of naive to memory B cells is associated with favourable prognosis.

O12

Combination of follicular and epidermal cell suspension as a novel surgical approach in acral vitiligo: a randomized controlled study

M. Razmi, D. Parsad and S. Kumaran

Post Graduate Institute of Medical Education and Research, Chandigarh, India

Epidermal and follicular cell suspensions (ECS and FCS, respectively) are established surgical modalities for stable vitiligo. However, even these methods give inferior results at acral sites. FCS has the additional benefits of using various melanocyte stem cells, better melanocyte/keratinocyte ratio and melanocytes with better morphological properties. By combining ECS and FCS, we hypothesized that we would get better results by replenishing healthy keratinocytes and creating a favourable microenvironment to transplant hair follicle stem cells. Matched patches from acral areas of 30 patients with stable vitiligo were randomized to ECS plus FCS or ECS alone. We harvested skin from the lateral thigh at a donor-to-recipient ratio of 1 : 5 and trypsinized the samples to obtain ECS. Harvested hair follicles from the occipital scalp were trypsinized sequentially to get FCS. ECS plus FCS was prepared by mixing ECS and FCS in a 1 : 10 ratio and transplanted to a manually dermabraded recipient site. They were followed up at 4, 8 and 16 weeks by a blinded observer, and extent of repigmentation, colour match and complications were noted. At week 16, the extent of repigmentation was ≥ 75% in 70% of patients allocated to ECS + FCS compared with 57% of those

© 2016 The Authors
BJD © 2016 British Association of Dermatologists

allocated to ECS ($P < 0{\cdot}001$). Excellent (90–100%) repigmentation was seen in 43·3% of those receiving ECS + FCS and 23·3% of those receiving ECS alone ($P = 0{\cdot}08$). ECS + FCS was rated better in regard to patient satisfaction ($P = 0{\cdot}04$) and colour matching ($P = 0{\cdot}001$). The outcome of the study was not dependent on age, sex, total duration of illness, stability period or lesional surface area. Complications were minimal in both groups. We established ECS + FCS as a novel approach in attaining good-to-excellent repigmentation in a short time period with good colour match in acral vitiligo. Acral vitiligo lesions have a smaller melanocyte stem cell reservoir and less production of stem cell factor (SCF) (Esmat SM, El-Tawdy AM, Hafez GA *et al.* Acral lesions of vitiligo: why are they resistant to photochemotherapy? *J Eur Acad Dermatol Venereol* 2012; **26**: 1097–104). The superior repigmentation obtained in ECS + FCS might be due to keratinocyte growth factors like SCF in ECS facilitating the growth of various stem cells in FCS. Mesenchymal stem cells in FCS also have various immunomodulatory properties. Amelanotic hair follicle melanocytes, which express fewer vitiligo-related antigens, in ECS + FCS might not be affected by the ongoing epidermal melanocyte-directed antibody attack, which is not possible in ECS. Thus, ECS + FCS combines local immunomodulation and repigmentation. A short follow-up period of only 16 weeks and nonstandardization of cell suspensions are limitations of our study.

O13
Leprosy in Kiribati: understanding the challenges and progress towards eradication

E. Trowbridge,[1] A. Cunanan,[2] S. Chambers,[3] A. Tonganibeia,[4] N. Ioteba[5] and J. Green[6]

[1]*Department of Dermatology,* [3]*Department of Infectious Diseases and* [6]*Department of Microbiology, Christchurch Hospital, Christchurch, New Zealand;* [2]*Chief of Culion Sanitarium and General Hospital, Culion, the Philippines;* [4]*Department of Communicable Diseases, Kiribati Ministry of Health, Tarawa, Kiribati and* [5]*New Zealand College of Public Health, Christchurch, New Zealand*

The nation of Kiribati is among the poorest in the developing world. Made up of 33 low-lying atolls, Kiribati has gained international awareness due to its vulnerability to the effects of climate change. The associated health consequences have been recognized by the World Health Organization (WHO), and a national adaption strategy has been developed. Additionally, in 1991 the WHO classified the elimination of leprosy as a public health priority. This is of direct relevance to Kiribati, as elimination of this treatable disease caused by *Mycobacterium leprae* remains an elusive goal for the nation. A strategic plan for the elimination of leprosy in Kiribati was developed by the WHO in 2014. Despite the availability of effective treatment, implementation of this programme in a nation that has very limited infrastructure, severe overcrowding (favouring spread) and poverty is a significant challenge. The Pacific Leprosy Foundation is a New Zealand charity working towards eradication of leprosy in the South Pacific. The foundation works in seven countries including Kiribati. The charity has adopted a community-focused approach to achieve its goal, recognizing that understanding a population's culture and environment is critical to successful implementation of any public health initiative. Furthermore, this approach provides much-needed morale and support to the clinic workers in Kiribati who are at the front line of delivering the programme and have understandably been overwhelmed by the enormity of the task within such a deprived and resource-poor community. In 2015 the Pacific Leprosy Foundation launched a three-phase pilot programme in Teaoraereke village in Kiribati. The first two phases were carried out in the first half of the year with a focus on raising community awareness, training staff in the identification of leprosy and contact screening. The final phase of the programme was a 3-day skin clinic in October 2015, the aim of which was primarily to detect undiagnosed cases of leprosy. In total 450 patients attended the clinic and 10 new cases of leprosy were diagnosed. Tuberculoid cases underwent a punch biopsy. Biopsies were subsequently transported to New Zealand for confirmatory polymerase chain reaction identification of *Mycobacterium leprae*. A wide range of other dermatological conditions were also seen and treated, including pityriasis versicolor, dermatitis, palmoplantar pustulosis and lichen planus. The programme is to be implemented progressively throughout Kiribati with the goal of achieving eradication of leprosy.

© 2016 The Authors
BJD © 2016 British Association of Dermatologists
