# Supplementary material for: Therapeutic efficacy of cell-based therapy in vitiligo: a research letter systematically reviewed using meta-analysis
Source: Arch Dermatol Res. 2024 May 22;316(5):198. doi: 10.1007/s00403-024-02920-6 (PMC11111487; doi:10.1007/s00403-024-02920-6)
Supplement: Supplementary file 1 — Supplementary file1 (ZIP 24195 KB) [file 403_2024_2920_MOESM1_ESM.zip › Studies were included/Razmi 2017.pdf]

based on the assumption that increased search queries for itch, and thus interest, serves as a proxy of increased itch symptoms in the public. That SVI for pain did not correlate with temperature further validates our observations for itch.

The rise in itch SVI with increasing temperatures during the summer months is likely a result of the increased incidence of pruritic skin disorders, such as poison ivy and insect bites, during the summer seasons. In addition, although the seasonality of itch has not explicitly been previously studied, it is known that heat or warmth sensations are major factors that provoke the itch response.[5] The transient receptor potential vanilloid type 1 protein is involved in the induction of histaminergic-associated pruritus and is activated by heat.[5] The calcitonin gene-related peptide has also been implicated in heat-induced pruritus, as selective ablation of calcitonin gene-related peptide alpha primary sensory neurons in mice can attenuate histamine-induced scratch behaviors.[5] Furthermore, skin inflammation has been shown to reduce the temperature threshold for transient receptor potential vanilloid type 1 activation, allowing itch induction at lower temperatures.[5] Increased environmental heat stimuli is not only an independent trigger for itch in healthy skin, but can also exacerbate pre-existing pruritus at a lower heat threshold.

A limitation of this study is that the population is limited to only Google users and extrapolates Google queries to the prevalence of pruritus. This research letter serves as an initial investigation of the seasonal variation of itch.

Radhika Grandhi, MPH,[a] Alice He, BS,[b] Yevgeniy R. Semenov, MD, MA,[c] and Shawn G. Kwatra, MD[b]

University of Cincinnati College of Medicine, Ohio[a]; Department of Dermatology, Johns Hopkins University School of Medicine, Baltimore, Maryland[b]; and Division of Dermatology, Washington University School of Medicine, St Louis, Missouri[c]

Ms Grandhi and Ms He contributed equally to this letter.

Funding sources: None.

Conflicts of interest: None declared.

Correspondence to: Shawn G. Kwatra, MD, Johns Hopkins University School of Medicine, Cancer Research Bldg II, 1550 Orleans St, Baltimore, MD 21231

E-mail: skwatra1@jhmi.edu

REFERENCES

1. Carneiro HA, Mylonakis E. Google Trends: a Web-based tool for real-time surveillance of disease outbreaks. *Clin Infect Dis*. 2009;49(10):1557-1564.
2. NOAA. Gridded climate divisional data set (CLIMDIV). nClimDiv (climate indices). Available from: https://www.ncdc.noaa.gov/monitoring-references/maps/us-climate-divisions.php. Accessed March 25, 2016.
3. Met Office. Climate summaries. Available from: http://www.metoffice.gov.uk/climate/uk/summaries. Accessed March 25, 2016.
4. Google Note. An improvement to our geographical assignment was applied retroactively from 1/1/2011. Available from: https://support.google.com/trends/answer/1383240?hl=en. Accessed September 4, 2016.
5. Murota H, Katayama I. Evolving understanding on the etiology of thermally provoked itch. *Eur J Pain*. 2015;20(1):47-50.

http://dx.doi.org/10.1016/j.jaad.2016.09.046

Combined epidermal and follicular cell suspension as a novel surgical approach for acral vitiligo

To the Editor: Autologous noncultured epidermal cell suspension (ECS) and follicular cell suspension (FCS) are effective modalities for the surgical management of stable vitiligo. There is need for further modification of these methods as repigmentation over acral and bony areas is not satisfactory with these methods. We devised a new surgical approach by combining ECS and FCS.

We followed the method described in our previous publication[1] for preparing and transplanting ECS and FCS. ECS was mixed with FCS in a 1:5 ratio. The combined suspension was transplanted to the dermabraded vitiligo patches. No additional treatment apart from regular sun exposure was advised.

In this case series, 5 patients with 12 symmetric vitiligo patches underwent combined ECS and FCS on one side and ECS on the opposite side. All except 1 patient had acral, bony, or nonsegmental vitiligo patches (Table I). Lesions were of comparable sizes with lesional stability of more than 1 year. They were followed up at 4-, 8-, and 16-week intervals by a blinded observer and the extent of repigmentation (by visual assessment and computerized image analysis) (Fig 1), color match, patient global assessment score, and complications were noted.

Extent of repigmentation at week 16 by visual assessment was superior in combined ECS and FCS as compared with ECS whether attaining 75% or greater (7/7, 100% vs 4/5, 80%) or 90% or greater repigmentation (7/7, 100% vs 1/5, 20%) (Table I). Similar results were obtained on computerized image analysis: combined ECS and

Table I. Patient characteristics and analysis of extent of repigmentation, rapidity in repigmentation, color match, and patient satisfaction

Patient	Lesion	Age, y	Sex	Vitiligo type	Stability, y	Lesion site	Lesion area, cm^2	Surgical method	RP at wk 8	RP at wk 16	RP at wk 16 by image analysis	RP with FCS done in the past	Color match at wk 16	PGA score (maximum score = 30)
1	1	24	F	AFV	6	Dorso-lateral aspect of foot (A)	24	ECS	50%-75%	75%-90%	76%	NA	Darker	20
1	2	24	F	AFV	6	Dorso-lateral aspect of foot (A)	12	ECS+FCS	>90%	>90%	95%	NA	Good	26
2	3	33	F	GV	4	Medial malleolus (A)	16	ECS+FCS	>90%	>90%	99%	25%-50%	Good	25
2	4	33	F	GV	4	Dorso-medial aspect of foot (A)	10	ECS+FCS	>90%	>90%	100%	<25%	Good	
2	5	33	F	GV	4	Dorso-medial aspect of foot (A)	8	ECS+FCS	25%-50%	>90%	79%	<25%	Good	
2	6	33	F	GV	4	Dorso-medial aspect of foot (A)	6	ECS	<25%	75%-90%	88%	<25%	Good	23
3	7	23	M	FV	8	Forehead (NAcr)	8	ECS+FCS	75%-90%	>90%	92%	25%-50%	Good	26
3	8	23	M	FV	8	Forehead (NAcr)	6	ECS	50%-75%	>90%	91%	25%-50%	Good	26
4	9	21	F	GV	14	Shin (NAcr)	12	ECS+FCS	75%-90%	>90%	95%	NA	Good	27
4	10	21	F	GV	14	Shin (NAcr)	14	ECS	25%-50%	50%-75%	55%	NA	Good	20
5	11	25	F	GV	1	Iliac crest (B)	34	ECS+FCS	50%-75%	>90%	99%	NA	Good	27
5	12	25	F	GV	1	Iliac crest (B)	40	ECS	50%-75%	75%-90%	82%	NA	Good	24

A, Acral area; *AFV*, acrofacial vitiligo; *B*, bony area; *ECS*, epidermal cell suspension; *ECS+FCS*, combined ECS and FCS; *F*, female; *FCS*, follicular cell suspension; *FV*, focal vitiligo; *GV*, generalized vitiligo; *M*, male; *NA*, not applicable; *NAcr*, nonacral area; *PGA*, patient global assessment; *RP*, repigmentation.

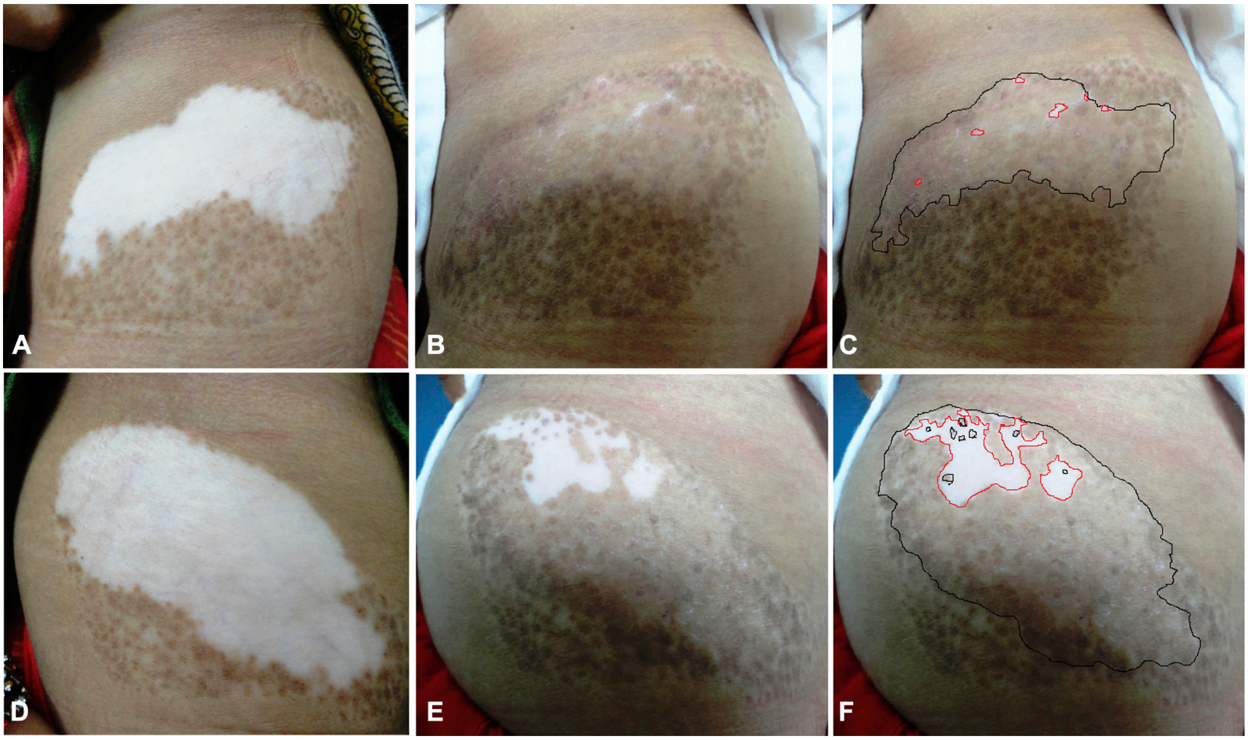

Fig 1. Depigmented patches on the left (**A**) and right (**D**) iliac crests of patient 5. After 16 weeks, combined epidermal cell suspension (ECS) and follicular cell suspension (FCS) side (**B**) attained more than 90% repigmentation while ECS (**E**) side attained only 75% to 90% repigmentation. Objective analysis of repigmentation at week 16 using software (Adobe Reader 11.0 for Windows, Adobe Systems Inc, San Jose, CA) showed 99% repigmentation over combined ECS and FCS side (**C**) and 82% repigmentation on ECS side (**F**). The area under repigmented portion (*black lines*) and nonpigmented portion (*red lines*) was measured as percentages of total lesional area using area tool in the measuring tool option of Adobe Reader 11.0 for Windows software (Adobe Systems Inc).

FCS was superior to ECS whether attaining 75% or more (7/7, 100% vs 4/5, 80%) or 90% or more repigmentation (6/7, 86% vs 1/5, 20%). Average repigmentation at week 16 on image analysis was 78% with ECS and 94% with combined ECS and FCS. Repigmentation was rapid (≥75% on visual assessment at week 8) in combined ECS and FCS compared with ECS (71.4% vs 0%). Combined ECS and FCS also showed better color matching (7/7, 100% vs 4/5, 80%). Complications were minimal in both groups. Average patient global assessment score was 26.2/30 in combined ECS and FCS and 22.6/30 in ECS. Six of 12 lesions were treated with FCS in the past with documented repigmentation of only 25% to 50% whereas these lesions showed more than 90% repigmentation with combined ECS and FCS. Thus combined ECS and FCS was found to be superior to either ECS or FCS alone in getting 90% or more repigmentation. Small sample size and short follow up-period are the limitations of the study.

Although FCS has more proliferative melanocytes and various stem cells in comparison with ECS, both had comparable repigmentation outcome in our previous study.[1] Combined ECS and FCS, in contrast to FCS, supplements healthy keratinocytes to the vitiligo lesions with damaged keratinocytes.[2] It is known that keratinocytes supply essential growth factors for melanocyte growth and proliferation.[3] ECS was chosen as a control in this study because it is the established surgical modality with a good outcome and hence we could compare any added benefit of our novel approach. The superior repigmentation obtained in combined ECS and FCS (Fig 1) might be a result of keratinocyte growth factors like stem cell factor or basic fibroblast growth factor from ECS, which facilitates the growth of various stem cells in FCS. It was shown

in a mouse study that stem cell factor and basic fibroblast growth factor should be supplied in the culture medium for the in vitro proliferation of melanocyte stem cells.[4] This new approach may be a good option in acral vitiligo where melanocyte stem cell defect is one of the reasons for treatment resistance.[5]

Muhammed Razmi T, MD, DNB, Davinder Parsad, MD, and Sendhil M. Kumaran, MD

Department of Dermatology, Venereology, and Leprology, Postgraduate Institute of Medical Education and Research, Chandigarh, India

Funding sources: None.

Conflicts of interest: None declared.

Correspondence to: Davinder Parsad, MD, Department of Dermatology, Venereology, and Leprology, Postgraduate Institute of Medical Education and Research, Sector 12, Chandigarh 160012, India

E-mail: parsad@me.com

REFERENCES

1. Singh C, Parsad D, Kanwar AJ, Dogra S, Kumar R. Comparison between autologous noncultured extracted hair follicle outer root sheath cell suspension and autologous noncultured epidermal cell suspension in the treatment of stable vitiligo: a randomized study. *Br J Dermatol*. 2013;169:287-293.
2. Bhawan J, Bhutani LK. Keratinocyte damage in vitiligo. *J Cutan Pathol*. 1983;10:207-212.
3. Gauthier Y, Surleve-Bazeille JE. Autologous grafting with noncultured melanocytes: a simplified method for treatment of depigmented lesions. *J Am Acad Dermatol*. 1992;26:191-194.
4. Nishikawa-Torikai S, Osawa M, Nishikawa S-I. Functional characterization of melanocyte stem cells in hair follicles. *J Invest Dermatol*. 2011;131:2358-2367.
5. Seleit I, Bakry OA, Abdou AG, Dawoud NM. Immunohistochemical expression of aberrant Notch-1 signaling in vitiligo: an implication for pathogenesis. *Ann Diagn Pathol*. 2014;18:117-124.

http://dx.doi.org/10.1016/j.jaad.2016.10.004

Ivermectin therapy for papulopustular rosacea and periorificial dermatitis in children: A series of 15 cases

To the Editor: Treatment of papulopustular rosacea (PPR) and periorificial dermatitis (POD) can be challenging in children. Demodex mites, although part of the normal-appearing skin fauna, are more numerous in rosacea and POD than in normal-appearing skin.[1] In 2014, we reported a case of severe oculocutaneous rosacea in a 12-year-old girl treated with a single dose of oral ivermectin[2]; the excellent response encouraged us to use it in subsequent cases. The aim of this retrospective study was to assess the benefit and tolerability of oral and topical ivermectin therapy in pediatric PPR and POD.

Eight patients with PPR and 7 with POD (mean age 9.8 ± 2.2 years) were treated with either a single dose of 200 to 250 μg/kg of oral ivermectin or a compound of 1% ivermectin in an oil-in-water base cream applied once a day for 3 months. Oral ivermectin was prescribed for 6 children with PPR and 3 with POD. Oral or topical therapy was chosen depending on the severity of the condition. No other medications were allowed.

To assess disease severity at baseline and after treatment, we applied the Investigator Global Assessment score by retrospectively reviewing clinical charts and photographs. We rated overall severity on a 0-to-4 scale as 0 = clear, 1 = almost clear, 2 = mild, 3 = moderate, and 4 = severe. We considered treatment to be successful when lesions cleared or almost cleared. Recurrence was documented when a disease flare required a further course of therapy. Table I summarizes the demographic and clinical data.

Complete or almost complete clearance (Investigator Global Assessment score 0-1) was achieved in 8 patients treated orally and in 6 children treated with topical ivermectin. One patient did not improve after oral therapy. The overall response to topical or oral ivermectin was excellent: 14 of 15 (93%) patients achieved complete or almost complete clearance of lesions (Fig 1); 3 of 14 patients experienced relapses (21%) and 11 of 14 remained disease-free for a prolonged period. Mean follow-up was 11.9 ± 7.1 (range 2-42) months. The only adverse event observed was mild, transient desquamation of the affected skin in 3 patients receiving oral ivermectin and in 2 patients using topical ivermectin.

In 2014, the US Food and Drug Administration approved 1% ivermectin cream for treatment of rosacea in adults. Oral ivermectin is licensed for treatment of filariasis in children weighing more than 15 kg and has been used off-label in refractory cases of rosacea.[2,3] Topical ivermectin may produce a burning sensation, pruritus, and dry skin in 0.7% to 1.8% of patients.[4] The only adverse event in our series was transient, mild desquamation in 5 patients. This was observed in patients receiving either topical or oral therapy. We hypothesize the desquamation may be a result of a Mazzotti-like reaction resulting from an immunologic reaction to dying mites, as observed in systemic parasitoses after ivermectin therapy.[5]
