# Supplementary material for: Therapeutic efficacy of cell-based therapy in vitiligo: a research letter systematically reviewed using meta-analysis
Source: Arch Dermatol Res. 2024 May 22;316(5):198. doi: 10.1007/s00403-024-02920-6 (PMC11111487; doi:10.1007/s00403-024-02920-6)
Supplement: Supplementary file 1 — Supplementary file1 (ZIP 24195 KB) [file 403_2024_2920_MOESM1_ESM.zip › Studies were included/Razmi 2018.pdf]

JAMA Dermatology | Original Investigation

# Combination of Follicular and Epidermal Cell Suspension as a Novel Surgical Approach in Difficult-to-Treat Vitiligo
## A Randomized Clinical Trial

Muhammed Razmi T, MD, DNB; Ravinder Kumar, PhD; Seema Rani, MSc; Sendhil M. Kumaran, MD, DNB; Sushma Tanwar, PhD; Davinder Parsad, MD

Supplemental content

**IMPORTANCE** Epidermal cell suspension (ECS) and follicular cell suspension (FCS) are successful surgical modalities for the treatment of stable vitiligo. However, repigmentation in generalized and acrofacial vitiligo and over acral or bony sites (eg, elbows, knees, iliac crests, and malleoli), which are difficult to treat, is challenging.

**OBJECTIVE** To study the efficacy of transplanting a combination of autologous, noncultured ECS and FCS (ECS + FCS) compared with ECS alone in stable vitiligo.

**DESIGN, SETTING, AND PARTICIPANTS** A prospective, observer-blinded, active-controlled, randomized clinical trial was conducted at a tertiary care hospital, with treatment administered as an outpatient procedure. Thirty participants who had stable vitiligo with symmetrical lesions were recruited between October 18, 2013, and October 28, 2016. All of the lesions were resistant to medical modalities with minimum lesional stability of 1 year. Intent-to-treat analysis was used.

**INTERVENTIONS** ECS + FCS was prepared by mixing equal amounts (in cell number) of FCS with ECS. After manual dermabrasion, ECS was applied to 1 lesion and ECS + FCS was applied to the anatomically based paired lesion of the same patient. No adjuvant treatment was given.

**MAIN OUTCOMES AND MEASURES** Patients were followed up at 4, 8, and 16 weeks by a blinded observer and extent of repigmentation, color match, pattern of repigmentation, patient satisfaction and complications were noted. Both the visual and the computerized image analysis methods were used for outcome assessment. Cell suspensions were assessed post hoc for OCT4+ stem cell counts using flow cytometry; expression of stem cell factor and basic fibroblast growth factor was evaluated using quantitative relative messenger RNA expression.

**RESULTS** Of the 30 patients included in the study, 18 (60%) were women; mean (SD) age was 23.4 (6.4) years. Seventy-four percent of the lesions (62 of 84) were difficult-to-treat vitiligo. ECS + FCS showed superior repigmentation outcomes compared with ECS: extent (76% vs 57%, $P < .001$), rapidity (48% vs 31%, $P = .001$), color match (73% vs 61%, $P < .001$), and patient satisfaction (mean [SD] patient global assessment score, 23.30 [6.89] vs 20.81 [6.61], $P = .047$). Melanocyte stem cell counts (2% in ECS + FCS vs 0.5% in ECS) as well as expression of basic fibroblast growth factor (11.8-fold) and stem cell factor (6.0-fold) were higher in ECS + FCS suspension ($P<.05$ for both).

**CONCLUSIONS AND RELEVANCE** The findings from this study establish ECS + FCS as a novel approach in vitiligo surgery for attaining good to excellent repigmentation in a short period with good color match, even in difficult-to-treat vitiligo.

**TRIAL REGISTRATION** ctri.nic.in Identifier: CTRI/2017/05/008692

*JAMA Dermatol*. 2018;154(3):301-308. doi:10.1001/jamadermatol.2017.5795
Published online January 31, 2018.

**Author Affiliations:** Department of Dermatology, Venereology and Leprology, Postgraduate Institute of Medical Education and Research, Chandigarh, India (Razmi T, Kumaran, Tanwar, Parsad); Department of Zoology, Panjab University, Chandigarh, India (Kumar, Rani).

**Corresponding Author:** Davinder Parsad, MD, Department of Dermatology, Venereology and Leprology, Postgraduate Institute of Medical Education and Research, sector 12, Chandigarh 160012, India (parsad@me.com).



© 2018 American Medical Association. All rights reserved.

Downloaded From: by a Universitätsbibliothek Leipzig User on 04/24/2018

Epidermal cell suspension (ECS) and follicular cell suspension (FCS) are established surgical methods for the repigmentation of stable vitiligo. The ultimate aim of any surgical procedure in vitiligo is complete and quick repigmentation with good cosmetic appeal. Although this target is almost achieved by ECS over the face and trunk regions and in focal and segmental vitiligo, there is a need for further improvement to attain optimal repigmentation in difficult-to-treat vitiligo. In the present study, difficult-to-treat vitiligo refers to the vitiligo lesions at acral or bony areas (elbows, knees, iliac crests, and malleoli)[1] or vitiligo types, such as generalized and acrofacial vitiligo,[2] that are inherently resistant to various repigmentation modalities.

Kim et al[3] have demonstrated better survival of melanocytes on coculturing with adipose-derived stem cells compared with keratinocyte-melanocyte coculture or melanocyte monoculture. Mesenchymal stem cells were shown to inhibit skin-homing $CD8^+$ T-cell activity in a coculture, suggesting its use as an auxiliary agent to improve transplantation efficacy in vitiligo of the autoimmune type.[4] Hair follicles are easy sources of various stem cells, including mesenchymal stem cells, and better melanocytes. By combining ECS and FCS (ECS + FCS), we tried to translate these in vitro results to the repigmentation outcome of difficult-to-treat vitiligo. A pilot study on 5 patients demonstrated the superior repigmentation outcome of ECS + FCS over either ECS or FCS alone.[5] In the present study, we evaluated the efficacy of ECS + FCS in a randomized clinical trial, with ECS as an active control. Because ECS was the established surgical modality with a good outcome, we could compare any added benefit of our novel approach.

## Key Points

**Question** Does the addition of follicular cell suspension improve the repigmentation outcome of epidermal cell suspension?

**Findings** In this randomized clinical trial that included 84 matched, stable vitiligo patches obtained from 30 patients, the repigmentation outcome of combined epidermal and follicular cell suspension was significantly superior to epidermal cell suspension alone (76% vs 57%). Significantly superior repigmentation outcome with epidermal and follicular cell suspension was observed also in treatment-resistant acral vitiligo and nonsegmental vitiligo.

**Meaning** Findings from this study establish epidermal and follicular cell suspension as a novel surgical approach in stable vitiligo with superior repigmentation outcome over epidermal cell suspension, even in inherently resistant lesions.

## Methods

This was a prospective, observer-blinded, intrapatient, active-controlled, randomized clinical trial conducted in a tertiary care center. Participants were recruited from patients attending the pigmentary clinic of our department between October 18, 2013, and October 28, 2016. Of 868 vitiligo patients screened, 63 patients satisfied the inclusion and exclusion criteria. Of these, 30 patients with bilaterally symmetrical patches or multiple patches in the same anatomic region with lesional stability of at least 1 year were recruited for the study. Children younger than 10 years, pregnant women, those with actively spreading vitiligo, and those with a history of koebnerization, hypertrophic and keloidal scars, and bleeding disorder were excluded.

The ethics committee of Postgraduate Institute of Medical Education and Research, Chandigarh, India, follows the Helsinki guidelines[6] and approved the study protocol (Supplement 1). Written informed consent was obtained from every patient; there was no financial compensation.

Matched vitiligo patches based on anatomic locations were randomized with computer-generated random number tables to ECS + FCS (group A) or ECS alone (group B). Centralized allocation concealment was done. We generated the random number table (M.R.T.) and enrolled the participants (S.M.K. and D.P.) and our laboratory staff, with little knowledge about the study, assigned the participants to the interventions based on the random numbers. At the first visit, baseline characteristics, history, and examination findings were noted. A CONSORT study flow diagram for this trial is depicted in **Figure 1**.

### Preparation of ECS + FCS

We followed the method described in a previous publication[7] with the following modifications for preparing ECS. Skin was harvested from the lateral thigh in a 1:5 ratio of donor to recipient skin. The cold method[8] of trypsinization was done to separate tissue into individual cell suspension. The FCS was prepared as proposed by Mohanty et al.[9] One hair follicle was harvested for 1 $cm^2$ of recipient area. An equal number of viable cells (measured using a hemocytometer) of FCS was added to ECS and gently mixed to prepare ECS + FCS. With the help of the trypan blue dye exclusion method, we ensured the viable melanocyte count in both suspensions (ECS + FCS or ECS) to be above 250 cells/$mm^2$ of the recipient area.

### Suspension Transplantation

The recipient area was dermabraded manually under local anesthesia until pinpoint bleeding was noted. The cell suspension was carefully transferred to the recipient site with the help of a tuberculin syringe mounted with an 18-gauge needle. A surgical dressing composed of petroleum jelly-chlorhexidine gauze, collagen (Eucare Pharmaceutical), and a sterile surgical pad was placed over the recipient site.

### Follow-up Evaluation

Follow-up examinations were done at day 8, week 4, week 8, and week 16. After removal of the dressing on day 8, no additional treatment apart from regular sun exposure was advised. Patient assessment was done by a blinded observer (S.M.K.) to compare the efficacy of both methods using primary (extent of repigmentation) and secondary (pattern of repigmentation, correlation of extent of repigmentation with the type of vitiligo and the site of surgery, color matching, patient satisfaction, and any adverse events) outcome factors.

Serial digital photography was taken at each follow-up visit in the same settings with respect to patient positioning, background, lighting, and camera settings. Repigmentation was assessed subjectively (visual analysis) as follows: less than 25% was



© 2018 American Medical Association. All rights reserved.

Downloaded From: by a Universitätsbibliothek Leipzig User on 04/24/2018

Figure 1. CONSORT Study Flow Diagram

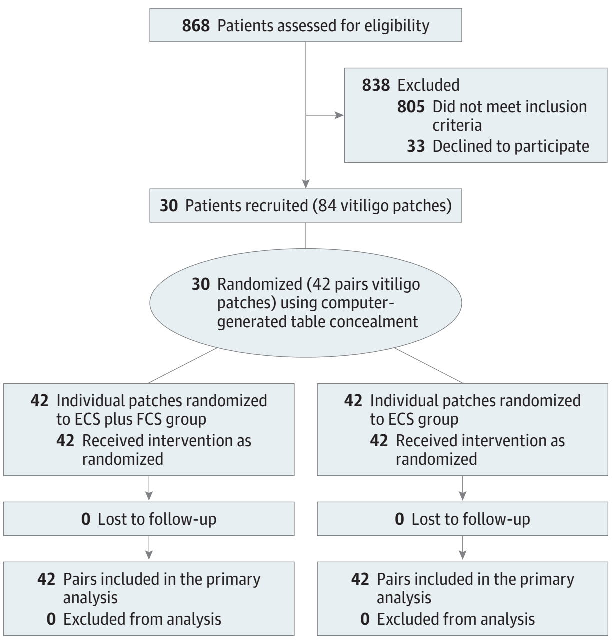


Patient enrollment and allocation. ECS indicates epidermal cell suspension; ECS + FCS, combined ECS and follicular cell suspension.

considered as minimal repigmentation (grade 0); less than 50%, poor repigmentation (grade 1); 50% to 75%, moderate repigmentation (grade 2); 75% to 90%, marked repigmentation (grade 3); and 90% to 100%, excellent repigmentation (grade 4). Extent of repigmentation was assessed objectively by computerized image analysis. Digital images of the lesions photographed at 16th week follow-up were converted to PDF format and the measuring tool of the Adobe Reader, version 11.0 for Windows software (Adobe Systems Inc) was used to assess percentage area covered through repigmentation from the baseline.[10]

The repigmentation pattern was noted as diffuse, perifollicular, or marginal. A note was also made of the color matching of the repigmented skin, as somewhat lighter than, same as, or somewhat darker than normal skin. Patient global assessment was done at week 16 with the help of a patient-satisfaction questionnaire[11] comprising 3 items: Grade the change in pigmentation in the transplanted area, Are you satisfied with the treatment?, and Do you find the treatment worthwhile? Patients were asked to answer in terms of scoring from 0 to 10.

## In Vitro Assessment of Cell Suspensions

In addition, we checked the percentage of HMB45+ melanocytes and OCT4+ stem cells in ECS, FCS, and ECS + FCS by fluorescence-activated cell sorting analysis. Fresh single-cell suspension obtained from ECS, FCS, and ECS + FCS was used for the flow cytometry. Expression of stem cell factor (SCF) and basic fibroblast growth factor (bFGF) was evaluated using quantitative relative messenger RNA expression. The gene-specific primers (eTable in Supplement 2) obtained from Sigma Aldrich, USA, were used for the evaluation. Methodology of this post hoc in vitro analysis is provided in the eAppendix in Supplement 2.

Table 1. Patient Characteristics

| Characteristic | Study Population |
|---|---|
| No. of patients | 30 |
| No. of lesions | 84 (42 pairs) |
| Age (range), y | 10-36 |
| Age, mean (SD), y | 23.37 (6.43) |
| Sex, No. (%) | |
| Women | 18 (60) |
| Men | 12 (40) |
| Duration of disease, median (IQR), y | 8 (5-13) |
| Duration of stability, median (IQR), y | 2.8 (1.1-6.0) |
| Type of vitiligo, No. (%)[a] | |
| Nonsegmental | 18 (60) |
| Segmental | 6 (20) |
| Focal | 3 (10) |
| Acrofacial | 3 (10) |
| Site of surgery (distribution of the lesions), No. (%)[b] | |
| Acral/bony areas | 50 (60) |
| Nonacral/nonbony areas | 34 (40) |
| Family history, No. (%) | |
| Acrofacial vitiligo | 1 (3) |
| Nonsegmental vitiligo | 2 (7) |
| Associated autoimmune disorder, No. (%)[c] | |
| Hypothyroidism | 3 (10) |
| Mean surface area treated, median (IQR), $cm^2$ [d] | |
| ECS + FCS | 6 (4.0-13.6) |
| ECS | 6 (3.3-12.0) |

Abbreviations: ECS, epidermal cell suspension; FCS, follicular cell suspension; IQR, interquartile range.

[a] Combining nonsegmental and acrofacial vitiligo and acral/bony areas, there were 62 of 84 (74%) patches of difficult-to-treat vitiligo.

[b] Acral areas (hands and feet, 38; fingers and toes, 4; eyelids, 2); bony areas (iliac crests, 4; malleoli, 2); nonacral/nonbony areas (arms and legs, 18; face, 12; trunk, 4).

[c] Patients had nonsegmental vitiligo.

[d] $P = .73$.

## Statistical Analysis

Sample size was estimated based on the previous study[11] assuming excellent (>90%) repigmentation in 1 group as 71% and in the other group as 27%. Our sample size determined to be 31 lesions per group at a power of 95% and CI of 95%. We decided to include extra samples for lost-to-follow-up cases. Finally, we included 42 lesions per group.

All quantitative variables are expressed using measures of central tendency (mean, median) and measures of dispersion (SD, interquartile range). Normality of data was checked by the Kolmogorov-Smirnov test. For normally distributed data, means were compared using a *t* test for outcome. For skewed data or scores, the Mann-Whitney test was used. For significance of changes within and between the groups over a period, the Wilcoxon signed-rank test was used. Proportions were compared between groups using the $\chi^2$ test or Fisher exact test when applicable. Intent-to-treat analysis was used. The Fisher exact test was used for intrapatient comparison of extent and rapidity of repigmentation (subjective assessment) as well as color match. All statistical tests were 2-sided and performed at a significance level of $P < .05$. Statistical analysis was carried out using SPSS, version 16.0 for Windows (SPSS Inc).



© 2018 American Medical Association. All rights reserved.

Downloaded From: by a Universitätsbibliothek Leipzig User on 04/24/2018

Table 2. Extent of Repigmentation, Rapidity in Repigmentation, Patient Satisfaction, Color Match, and Pattern of Repigmentation

| Variable | Outcome | No. (%) ECS + FCS | No. (%) ECS | *P* Value |
|---|---|---|---|---|
| Repigmentation at week 16 | ≥75% (good) | 32/42 (76) | 24/42 (57) | <.001 |
| | ≥90% (excellent) | 22/42 (52) | 13/42 (31) | .001 |
| Quantitative assessment of repigmentation by computerized image analysis, % (95% CI) | Median repigmentation at week 16 | 89.5 (52-91) | 76.5 (73-95) | .02 |
| Repigmentation at week 8 | ≥75% (good) | 20/42 (48) | 13/42 (31) | .001 |
| | ≥90% (excellent) | 7/42 (17) | 3/42 (7) | .003 |
| Patient global assessment, mean (SD) | Maximum score, 30.0 | 23.3 (6.9) | 20.8 (6.6) | .047 |
| Color match[a] | Good | 30/41 (73) | 25/41 (61) | <.001 |
| Pattern of repigmentation[a] | Diffuse | 21/41 (51) | 20/41 (49) | .32 |
| | Perifollicular | 19/41 (46) | 19/41 (46) | |
| | Marginal | 1/41 (2) | 2/41 (5) | |

Abbreviations: ECS, epidermal cell suspension; ECS + FCS, combined ECS and follicular cell suspension.

[a] One patient with single pair of matched lesions failed to achieve repigmentation with both methods; hence, we excluded those lesions from the analysis. The remaining 29 patients (41 matched pairs of lesions) were analyzed for color match and pattern of repigmentation.

## Results

All of the patients completed the study period of 16 weeks and were included in the final analysis. Eighty-four target lesions (42 pairs) were included in this study. Seventy-four percent of the lesions were of difficult-to-treat vitiligo. The baseline characteristics of the patients in both groups are reported in **Table 1**. There was no statistically significant difference between the lesional surface area between the groups (*P* = .73).

### Outcome of the Surgery

Outcome of the surgery was not dependent on age, sex, duration of illness, stability period, or lesional surface area. Comparison of the extent of repigmentation between both groups at each follow-up visit is expressed in eFigure 1 in Supplement 2. In the intrapatient comparison, ECS + FCS consistently showed better repigmentation scores throughout the 16-week follow-up period (*P* = .002).

The extent of repigmentation at week 16 is reported in eFigure 2 in Supplement 2. Repigmentation at week 16, quantitative assessment of repigmentation by computerized image analysis, rapidity of repigmentation at week 8, patient satisfaction, color match, and pattern recognition are reported in **Table 2**. The extent of repigmentation on visual analysis was superior in ECS + FCS whether attaining 75% or more or 90% or more repigmentation (*P* = .001). Objective assessment by computerized image analysis also revealed superior results with ECS + FCS compared with ECS (*P* = .02). Patient satisfaction (*P* = .047) as well as the color match (*P* < .001) were also significantly better with ECS + FCS.

Repigmentation outcome of ECS + FCS was found to be superior to ECS even at acral or bony sites and in generalized or acrofacial vitiligo. Objective assessment of repigmentation attained at week 16 on computerized image analysis showed superior results with ECS + FCS compared with ECS at both acral (82%; 95% CI, 59%-92.5% vs 76%; 95% CI, 43.5%-80.5%; *P* = .005) and nonacral (94%; 95% CI, 82.5%-95% vs 84%; 95% CI, 54%-92.5%; *P* = .001) areas. A similar observation was noted in generalized and acrofacial vitiligo (94%; 95% CI, 88%-95% vs 84%; 95% CI, 55%-91%; *P* = .02) as well as focal and segmental vitiligo (87%; 95% CI, 56%-94% vs 76%; 95% CI, 36%-92%; *P* < .001).

Seven patients had hyperpigmentation at the skin donor site, which was a common occurrence for both the methods. None of our patients developed koebnerization, visible scarring or milia at any donor or recipient site.

### Melanocytes and OCT4+ Stem Cells in the ECS and FCS

The melanocyte cell percentage was comparable in both groups; slightly higher in ECS + FCS compared with ECS (3.6% vs 2.7%). Results further showed that OCT4+ stem cells were present in both ECS and FCS, but the number was higher in ECS + FCS compared with ECS alone (2% vs 0.5%) (eFigure 3 in Supplement 2).

### Quantitative Real Time Expression of *SCF* and *bFGF*

Our in vitro study showed that the expression of *SCF* (NCBI 4254 and MIM 184745) was higher in ECS + FCS (6.0-fold) compared with ECS alone (eFigure 4 in Supplement 2). The expression of *bFGF* (NCBI 2247 and MIM 134920) was significantly higher in the ECS + FCS (11.8-fold) compared with ECS (eFigure 5 in Supplement 2).

## Discussion

Our study showed superior repigmentation with ECS + FCS vs ECS. The repigmentation outcome of ECS was satisfactory and was better than the results reported in the literature, considering that the majority of the treated lesions in this study were difficult-to-treat vitiligo. However, ECS + FCS showed better repigmentation compared with ECS, especially in difficult-to-treat vitiligo, in terms of rapidity and the extent of repigmentation. The cosmetic appeal of the vitiligo lesions was better with ECS + FCS owing to the superior color match and nearly complete repigmentation, resulting in better patient satisfaction.

Enhancement of repigmentation on combining FCS with ECS might be due to the improvement of melanocyte properties and the possible presence of various stem cells in FCS.



© 2018 American Medical Association. All rights reserved.

Downloaded From: by a Universitätsbibliothek Leipzig User on 04/24/2018

Figure 2. Repigmentation Outcome in Acral Vitiligo

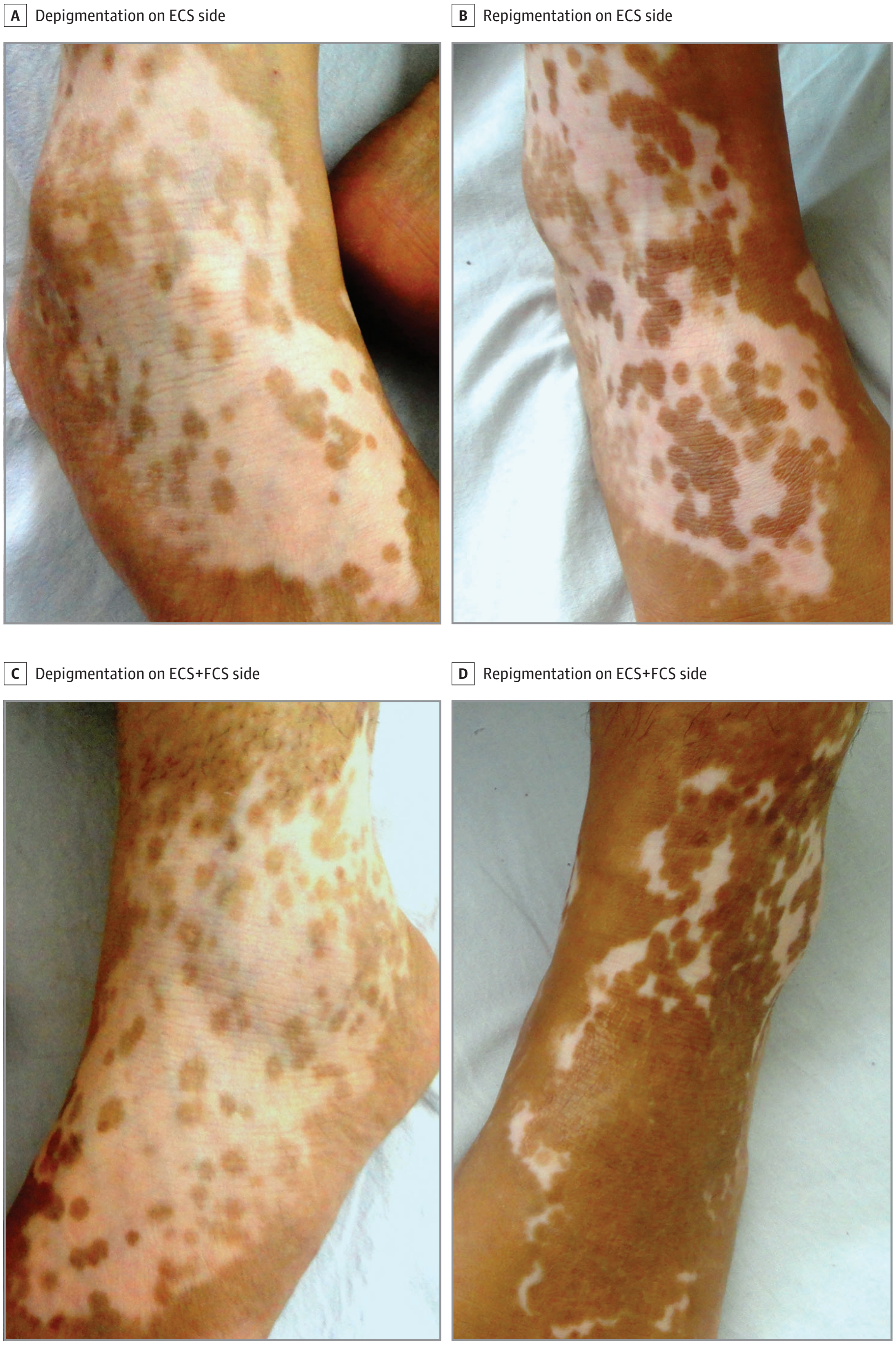


A, Depigmented patches on the dorsum of the right foot, epidermal cell suspension (ECS) side. B, At 16 weeks' follow-up, repigmentation achieved on the ECS side was only 25% to 50%. C, Depigmented patches on the dorsum of the left foot, ECS+follicular cell suspension (FCS) side. D, At 16 weeks' follow-up, repigmentation achieved on the ECS + FCS side was 75% to 90%.

In follicular melanin units, the melanocyte to keratinocyte ratio is higher (1:5) compared with skin (1:36).[12] Melanocyte stem cells, melanoblasts, and other immature melanocytes are mainly located in the hair follicle compared with the epidermis.[13] However, the repigmentation outcome of FCS was not appealing compared with ECS according to 3 studies.[7,14,15] Keratinocyte growth factors, such as SCF or bFGF in ECS, by facilitating the expression of various stem cells in FCS, might be the reason for better repigmentation of ECS + FCS in this study. Keratinocytes supply essential growth factors for melanocyte growth.[16] Keratinocyte-derived factors that help in melanogenesis include endothelin-1, SCF, bFGF, and nerve growth factor etc.[17] It was shown in a mice study that SCF and bFGF should be supplied in the culture medium for the in vitro proliferation of melanocyte stem cells.[18]

Follicular cell suspension might also enhance the repigmentation outcome of ECS when transplanted in combination through immunomodulatory properties of hair follicle



© 2018 American Medical Association. All rights reserved.

Downloaded From: by a Universitätsbibliothek Leipzig User on 04/24/2018

Table 3. Overview of Literature on ECS and FCS

| Source | Method | No. of Patients Evaluated (No. of Lesions) | Follow-up | Outcome | Comments |
|---|---|---|---|---|---|
| Gauthier et, al,[16] 1992 | ECS | 12 | 1-3 mo | 70% RP in 63% of patients | Only FV, SV; not many lesions on difficult sites; short follow-up period |
| Mulekar et al,[35] 2005 | ECS | 142 | 1-6 y | >65% RP in 67% of GV | Long-term follow-up, benefit of more time for RP; chance of additional therapy during follow-up |
| van Geel et al,[36] 2006 | ECS | 40 | 3-12 mo | 70% RP in 62% of the lesions | Phototherapy, topical medications as additional therapy after ECS |
| El-Zawahry et al,[37] 2011 | ECS | 22 | 6-17 mo | >75% RP in 27% of the patients | Inferior result in acral area even though photochemotherapy used after the procedure; long follow-up period |
| Holla et al,[38] 2013 | ECS | 36 | NR | >75% RP in 64% of the lesions | Study was retrospective |
| Mohanty et al,[9] 2011 | FCS | 14 | 1-15 mo | >75% RP in 64% of the patients | Only one-third of lesions were on acral area; even 3-mo stable patients were included; long follow-up period. |
| Singh et al,[7] 2013 | ECS vs FCS | 30 (47) | 4 mo | >75% RP in 92% in ECS vs 78% in FCS | Half of the patients had FV or SV; site of surgery not mentioned; short follow-up period |
| Vinay et al,[15] 2015 | FCS | 30 (60) | 6 mo | >75% RP in 35% of the lesions | Only one-third of lesions were on acral area |
| Donaparthi and Chopra,[14] 2016 | ECS vs FCS | 11 (60) | 6 mo | >75% RP in 90% in ECS vs 43% in FCS | Authors reported the lack of surgical skill on FCS; fewer lesions on acral areas; short follow-up period |
| Present study | ECS + FCS vs ECS | 30 (84) | 4 mo | >75% RP in 76.2% in ECS + FCS vs 57.1% in ECS | 60% of lesions were on acral area; only 26% lesions were FV or SV; same patient used as control unlike the above studies; short follow-up period |

Abbreviations: ECS, epidermal cell suspension; FCS, follicular cell suspension; FV, focal vitiligo; GV, generalized vitiligo; RP, repigmentation; SV, segmental vitiligo.

mesenchymal stem cells.[19,20] Expression of OCT4 is essential for maintaining mesenchymal stem cell properties.[21] The OCT4+ cells are pluripotent stem cells present in the skin, mostly in the stem cell niche of hair follicles, which can perceivably give rise to follicular melanoblasts.[22-24] In fact, using a 3-dimensional skin equivalent, Li et al[23]showed that OCT4+ dermal stem cells could differentiate into HMB45-positive melanocytes and migrate to the epidermis. An animal study[25] demonstrated that simultaneous grafting of melanocytes and adipose-derived stem cells resulted in more durability of transplanted melanocytes and hence increased efficacy over grafting of melanocytes alone. However, there are no data in the literature regarding OCT4+ hair follicle stem cells or bFGF and SCF in the cell suspensions used for vitiligo surgery. Our post hoc in vitro study revealed a better OCT4+ stem cell count and *bFGF* and *SCF* expression in ECS + FCS compared with ECS alone. Hence, ECS + FCS with higher amounts of OCT4+ stem cells and melanocyte growth factors possibly combine immunomodulation and repigmentation.

Repigmentation outcome of ECS + FCS was found to be superior to ECS even in generalized vitiligo and acrofacial vitiligo. In one study, autoantibodies were found to be more commonly present in patients with nonsegmental vitiligo (50%-67%) than in those with segmental vitiligo (0%-17%).[26] Cell-mediated immune attack takes place against transplanted melanocytes in generalized vitiligo following melanocyte transplantation, which results in an inferior repigmentation outcome.[4,27,28] This melanocyte-directed immune attack may also be the reason for the poor repigmentation outcome in Koebner sites, such as the bony areas or the eyelids.[29] Amelanotic hair follicle melanocytes express some antigens associated with alopecia areata, but not antigens associated with vitiligo, whereas the reverse is true for epidermal melanocytes.[30] Therefore, hair follicle melanocytes in ECS + FCS might have escaped from the ongoing epidermal melanocyte-directed antibody attack, which might not be possible for epidermal melanocytes in the ECS group.

Aberrant Notch-1 signaling and hence elimination of melanoblasts and melanocyte stem cells was attributed to be the cause of treatment resistance in acral vitiligo.[31] The lower density of melanocytes and melanocyte stem cell reservoir as well as the lower SCF production was hypothesized to be the cause of repigmentation failure at acral areas.[32] In our study, ECS + FCS with a greater amount of OCT4+ stem cells, bFGF, and SCF showed superior repigmentation compared with ECS alone at acral sites (**Figure 2**).

Another important finding is the quicker repigmentation in ECS + FCS compared with ECS. Although approximately half of the lesions in ECS + FCS arm attained greater than 75% repigmentation within 2 months, similar results were seen in only less than one-third of the lesions in the ECS arm. We have observed that this aspect is especially important when there is a demand for quick repigmentation outcome from the patients to tide over important life events, such as marriage.

Pattern of repigmentation was primarily diffuse (50%) or perifollicular (46%) in both groups. This finding is contrary to the results of previous studies[7,11] in which the diffuse pattern was considered to be the pattern of successful transplantation of melanocytes and keratinocytes. However, studies on ECS have established that transplanted melanocytes migrate retrograde to hair follicles to repigment leukotrichia.[33] This process might be the reason for the perifollicular pattern of repigmentation in our study.

A frequently reported limitation of any surgical method is the presence of color mismatch between the treated area and the surrounding skin. In our study, color match was obtained



© 2018 American Medical Association. All rights reserved.

Downloaded From: by a Universitätsbibliothek Leipzig User on 04/24/2018

in 73% in ECS + FCS and 61% in ECS sites. Initially, the patients had hyperpigmentation at the recipient site that matched the surrounding skin color by week 16. Color matching of only 20% to 50% was noted in ECS or FCS in some studies.[15,34]

On patient global assessment, ECS + FCS was found to be significantly superior to ECS in patient satisfaction. Although an extra visit for FCS treatment was needed in this study, since we utilized the cold method of trypsinization in ECS, most of the patients (60%) opted for it as the surgery of choice in the future if needed, while only 7% opted for ECS alone. The remaining patients did not find any difference between the methods.

The repigmentation outcome varies widely within different studies: the number of patients attaining more than 75% repigmentation in published studies ranges from 27% to 100%.[34] This finding might be explained by the fact that many differences exist among the studies. An overview of published studies on ECS and FCS relevant to our study is given in **Table 3**.

### Limitations

Limitations of our study were small sample size and a short follow-up period of only 16 weeks. The observed results may be different in a longer follow-up, since repigmentation after the surgery continues after 16 weeks. We cannot rule out the possible role of slightly better melanocyte counts for superior repigmentation of ECS + FCS in the present study. Although the number of melanocytes in the cell suspension determines the extent of repigmentation, the outcome enters a plateau phase after an optimum concentration of melanocytes as demonstrated in a study on cultured melanocytes by Hong et al.[39] A similar observation was noted with FCS.[15] Although there is a definite correlation between repigmentation outcome and the higher amount of stem cells and growth factors in ECS + FCS, further studies are needed for better understanding of the contribution of these factors in the repigmentation outcome.

## Conclusions

This study established ECS + FCS as a novel approach in vitiligo surgery for attaining good to excellent repigmentation within a short time with good color match, even in difficult-to-treat vitiligo. Superior repigmentation outcome with use of ECS + FCS underscores the translational message of previous animal studies that had shown better melanocyte as well as stem cell survival on their coculturing. This novel approach can be used in clinical practice for achieving optimal repigmentation in difficult-to-treat vitiligo and in scenarios with a demand for a quicker repigmentation outcome.

ARTICLE INFORMATION

**Accepted for Publication:** November 28, 2017.

**Published Online:** January 31, 2018. doi:10.1001/jamadermatol.2017.5795

**Author Contributions**: Drs Razmi T and Parsad had full access to all of the data in the study and take responsibility for the integrity of the data and the accuracy of the data analysis.
*Study concept and design:* Razmi T, Kumar, Sendhil Kumaran, Parsad.
*Acquisition, analysis, or interpretation of data*: All authors.
*Drafting of the manuscript:* Razmi T, Rani.
*Critical revision of the manuscript for important intellectual content:* Kumar, Sendhil Kumaran, Tanwar, Parsad.
*Statistical analysis:* Razmi T, Kumar, Rani.
*Administrative, technical, or material support:* Rani, Tanwar, Parsad.
*Study supervision:* Kumar, Sendhil Kumaran, Parsad.

**Conflict of Interest Disclosures**: No conflicts were reported.

**Additional Contributions:** Kusum Chopra, MSc, provided statistical analysis for the study; there was no financial compensation.

REFERENCES

1. Holla AP, Parsad D. Vitiligo surgery: its evolution as a definite treatment in the stable vitiligo. *G Ital Dermatol Venereol*. 2010;145(1):79-88.

2. Mulekar SV, Isedeh P. Surgical interventions for vitiligo: an evidence-based review. *Br J Dermatol*. 2013;169(suppl 3):57-66.

3. Kim JY, Park CD, Lee JH, Lee CH, Do BR, Lee AY. Co-culture of melanocytes with adipose-derived stem cells as a potential substitute for co-culture with keratinocytes. *Acta Derm Venereol*. 2012;92(1):16-23.

4. Zhou MN, Zhang ZQ, Wu JL, et al. Dermal mesenchymal stem cells (DMSCs) inhibit skin-homing CD8+ T cell activity, a determining factor of vitiligo patients' autologous melanocytes transplantation efficiency. *PLoS One*. 2013;8(4):e60254.

5. Razmi T M, Parsad D, Kumaran SM. Combined epidermal and follicular cell suspension as a novel surgical approach for acral vitiligo. *J Am Acad Dermatol*. 2017;76(3):564-567.

6. World Medical Association. World Medical Association Declaration of Helsinki: ethical principles for medical research involving human subjects. *JAMA*. 2013;310(20):2191-2194.

7. Singh C, Parsad D, Kanwar AJ, Dogra S, Kumar R. Comparison between autologous noncultured extracted hair follicle outer root sheath cell suspension and autologous noncultured epidermal cell suspension in the treatment of stable vitiligo: a randomized study. *Br J Dermatol*. 2013;169(2):287-293.

8. Gauthier Y, Benzekri L. Non-cultured epidermal suspension in vitiligo: from laboratory to clinic. *Indian J Dermatol Venereol Leprol*. 2012;78(1):59-63.

9. Mohanty S, Kumar A, Dhawan J, Sreenivas V, Gupta S. Noncultured extracted hair follicle outer root sheath cell suspension for transplantation in vitiligo. *Br J Dermatol*. 2011;164(6):1241-1246.

10. Verma G, Varkhande SR, Kar HK, Rani R. Evaluation of repigmentation with cultured melanocyte transplantation (CMT) compared with non-cultured epidermal cell transplantation in vitiligo at 12th week reveals better repigmentation with CMT. *J Invest Dermatol*. 2015;135(10):2533-2535.

11. Budania A, Parsad D, Kanwar AJ, Dogra S. Comparison between autologous noncultured epidermal cell suspension and suction blister epidermal grafting in stable vitiligo: a randomized study. *Br J Dermatol*. 2012;167(6):1295-1301.

12. Tobin DJ, Paus R. Graying: gerontobiology of the hair follicle pigmentary unit. *Exp Gerontol*. 2001;36(1):29-54.

13. Goldstein NB, Koster MI, Hoaglin LG, et al. Narrow band ultraviolet B treatment for human vitiligo is associated with proliferation, migration, and differentiation of melanocyte precursors. *J Invest Dermatol*. 2015;135(8):2068-2076.

14. Donaparthi N, Chopra A. Comparative study of efficacy of epidermal melanocyte transfer versus hair follicular melanocyte transfer in stable vitiligo. *Indian J Dermatol*. 2016;61(6):640-644.

15. Vinay K, Dogra S, Parsad D, et al. Clinical and treatment characteristics determining therapeutic outcome in patients undergoing autologous non-cultured outer root sheath hair follicle cell suspension for treatment of stable vitiligo. *J Eur Acad Dermatol Venereol*. 2015;29(1):31-37.

16. Gauthier Y, Surleve-Bazeille JE. Autologous grafting with noncultured melanocytes: a simplified method for treatment of depigmented lesions. *J Am Acad Dermatol*. 1992;26(2, pt 1):191-194.

17. Lee BW, Schwartz RA, Hercogová J, Valle Y, Lotti TM. Vitiligo road map. *Dermatol Ther*. 2012;25(suppl 1):S44-S56.

18. Nishikawa-Torikai S, Osawa M, Nishikawa S. Functional characterization of melanocyte stem cells in hair follicles. *J Invest Dermatol*. 2011;131(12):2358-2367.



© 2018 American Medical Association. All rights reserved.

Downloaded From: by a Universitätsbibliothek Leipzig User on 04/24/2018

19. Nishimura EK, Jordan SA, Oshima H, et al. Dominant role of the niche in melanocyte stem-cell fate determination. *Nature*. 2002;416(6883):854-860.

20. De Miguel MP, Fuentes-Julián S, Blázquez-Martínez A, et al. Immunosuppressive properties of mesenchymal stem cells: advances and applications. *Curr Mol Med*. 2012;12(5):574-591.

21. Tsai CC, Hung SC. Functional roles of pluripotency transcription factors in mesenchymal stem cells. *Cell Cycle*. 2012;11(20):3711-3712.

22. Yu H, Fang D, Kumar SM, et al. Isolation of a novel population of multipotent adult stem cells from human hair follicles. *Am J Pathol*. 2006;168(6):1879-1888.

23. Li L, Fukunaga-Kalabis M, Yu H, et al. Human dermal stem cells differentiate into functional epidermal melanocytes. *J Cell Sci*. 2010;123(Pt 6):853-860.

24. Limbourg A, Schnabel S, Lozanovski VJ, et al. Genetic reporter analysis reveals an expandable reservoir of OCT4+ cells in adult skin. *Cell Regen (Lond)*. 2014;3(1):9.

25. Lim WS, Kim CH, Kim JY, Do BR, Kim EJ, Lee AY. Adipose-derived stem cells improve efficacy of melanocyte transplantation in animal skin. *Biomol Ther (Seoul)*. 2014;22(4):328-333.

26. Pradhan V, Patwardhan M, Thakkar V, et al. Vitiligo patients from India (Mumbai) show differences in clinical, demographic and autoantibody profiles compared to patients in western countries. *J Eur Acad Dermatol Venereol*. 2013;27(3):279-286.

27. Rao A, Gupta S, Dinda AK, et al. Study of clinical, biochemical and immunological factors determining stability of disease in patients with generalized vitiligo undergoing melanocyte transplantation. *Br J Dermatol*. 2012;166(6):1230-1236.

28. Abdallah M, Abdel-Naser MB, Moussa MH, Assaf C, Orfanos CE. Sequential immunohistochemical study of depigmenting and repigmenting minigrafts in vitiligo. *Eur J Dermatol*. 2003;13(6):548-552.

29. Mulekar SV, Al Issa A, Al Eisa A. Treatment of vitiligo on difficult-to-treat sites using autologous noncultured cellular grafting. *Dermatol Surg*. 2009;35(1):66-71.

30. Tobin DJ, Bystryn JC. Different populations of melanocytes are present in hair follicles and epidermis. *Pigment Cell Res*. 1996;9(6):304-310.

31. Seleit I, Bakry OA, Abdou AG, Dawoud NM. Immunohistochemical expression of aberrant notch-1 signaling in vitiligo: an implication for pathogenesis. *Ann Diagn Pathol*. 2014;18(3):117-124.

32. Esmat SM, El-Tawdy AM, Hafez GA, et al. Acral lesions of vitiligo: why are they resistant to photochemotherapy? *J Eur Acad Dermatol Venereol*. 2012;26(9):1097-1104.

33. Holla AP, Sahni K, Kumar R, Kanwar A, Mehta S, Parsad D. Repigmentation of leukotrichia due to retrograde migration of melanocytes after noncultured epidermal suspension transplantation. *Dermatol Surg*. 2014;40(2):169-175.

34. van Geel N, Goh BK, Wallaeys E, De Keyser S, Lambert J. A review of non-cultured epidermal cellular grafting in vitiligo. *J Cutan Aesthet Surg*. 2011;4(1):17-22.

35. Mulekar SV. Long-term follow-up study of 142 patients with vitiligo vulgaris treated by autologous, non-cultured melanocyte-keratinocyte cell transplantation. *Int J Dermatol*. 2005;44(10):841-845.

36. van Geel N, Ongenae K, Vander Haeghen Y, Vervaet C, Naeyaert JM. Subjective and objective evaluation of noncultured epidermal cellular grafting for repigmenting vitiligo. *Dermatology*. 2006;213(1):23-29.

37. El-Zawahry BM, Zaki NS, Bassiouny DA, et al. Autologous melanocyte-keratinocyte suspension in the treatment of vitiligo. *J Eur Acad Dermatol Venereol*. 2011;25(2):215-220.

38. Holla AP, Sahni K, Kumar R, Parsad D, Kanwar A, Mehta SD. Acral vitiligo and lesions over joints treated with non-cultured epidermal cell suspension transplantation. *Clin Exp Dermatol*. 2013;38(4):332-337.

39. Hong WS, Hu DN, Qian GP, McCormick SA, Xu AE. Ratio of size of recipient and donor areas in treatment of vitiligo by autologous cultured melanocyte transplantation. *Br J Dermatol*. 2011;165(3):520-525.

NOTABLE NOTES

# Monroe's Mark

Tyler Marion, BS, MBA; Kevin Cao, BS; Jorge Roman, MD

For most of human history, beauty marks have not always considered to be beautiful. In Ancient Rome, the standard of beauty was fair and spotless skin. In medieval Europe, pigmented lesions were one of several cutaneous signs associated with "evil" or demonic connection, the so-called Devil's mark.[1] The attitude toward beauty marks improved in the 18th century, when moles became popular among the royal courts of Europe. Artificial beauty marks, known as *mouches*, were created from silk, velvet, or taffeta and were worn by both men and women. After a several-centuries–long period of quiescence, however, the beauty mark reemerged in the 1950s in association with the era's greatest sex symbol, Marilyn Monroe. She quickly became known for the off-center mole above her left upper lip, and with it she appealed to generations of women for many years to come.

Monroe, one of the most famous stars of classic Hollywood cinema, was a female icon who reshaped the status quo of beauty. Appearing in various movies, photographs, and through modeling, she captivated the world like nobody before her. The way Monroe carried herself and how she embraced her curvy figure made her an icon of femininity and sexuality. Beyond the tight-fitting sequined dress she wore to sing "Happy Birthday" to President John F. Kennedy, or the pink satin gown she wore when she sang "Diamonds Are a Girl's Best Friend" in *Gentlemen Prefer Blondes* (1953), the way she was comfortable in her own skin changed how millions of women wore theirs. Perhaps best embodied by her iconic beauty mark, the message she sent to girls in the 1950s was one of strength, confidence, and positive body image.

While Monroe was not the first person to inspire the use of beauty marks, she was the force behind the reemergence of the beauty mark in Western society. She opened the door for others to use beauty marks as a form of self-expression. Celebrities such as Cindy Crawford, Blake Lively, and Natalie Portman all have distinctive moles. Lady Gaga in particular uses makeup to highlight and emphasize hers, a stark contrast to the women of ancient Rome who tried to remove blemishes with ashes made from snails. At the end of Thomas Harris's book *The Silence of the Lambs*, FBI agent Clarice Starling acquired an artificial beauty mark high on her cheekbone from grains of burnt powder from the revolver of the late Jame "Buffalo Bill" Gumb.[2] Being well versed in tattoos, body symbology, and ritual mutilation, agent Jack Crawford comments on the mark at the beginning of the next book in the series, *Hannibal*.[3] Alluding to the French symbolism of the location of Starling's *mouche* he said, "They call that one 'courage.' You can wear that one. I'd keep it if I were you."[3(p58)]

**Author Affiliations:** The University of Texas Medical Branch School of Medicine, Galveston (Marion, Cao); Department of Internal Medicine, Texas Health Presbyterian Hospital, Dallas (Roman).

**Corresponding Author:** Jorge Roman, MD, Department of Internal Medicine, Texas Health Presbyterian Hospital, 8200 Walnut Hill Ln, Dallas, TX 75231 (joromanmd@gmail.com).

1. Maranda EL, Lim VM, Taneja R, Simmons BJ, Kallis PJ, Jimenez JJ. Witches and warts. *JAMA Dermatol*. 2016;152(8):877.

2. Harris T. *The Silence of the Lambs*. London, England: William Heinemann; 1989.

3. Harris T. *Hannibal*. New York, NY: Delacorte Press; 1999.



© 2018 American Medical Association. All rights reserved.

Downloaded From: by a Universitätsbibliothek Leipzig User on 04/24/2018
