# Supplementary material for: Therapeutic efficacy of cell-based therapy in vitiligo: a research letter systematically reviewed using meta-analysis
Source: Arch Dermatol Res. 2024 May 22;316(5):198. doi: 10.1007/s00403-024-02920-6 (PMC11111487; doi:10.1007/s00403-024-02920-6)
Supplement: Supplementary file 1 — Supplementary file1 (ZIP 24195 KB) [file 403_2024_2920_MOESM1_ESM.zip › Studies were included/RCT Budania 2012.pdf]

BJD
British Journal of Dermatology

Comparison between autologous noncultured epidermal cell suspension and suction blister epidermal grafting in stable vitiligo: a randomized study

A. Budania, D. Parsad, A.J. Kanwar and S. Dogra

Department of Dermatology, Postgraduate Institute of Medical Education and Research, Chandigarh 160012, India

Correspondence
Davinder Parsad.
E-mail: parsad@mac.com

Accepted for publication
9 August 2012

Funding sources
None.

Conflicts of interest
None declared.

DOI 10.1111/bjd.12007

Summary

Background Vitiligo is an acquired disorder of pigmentation due to loss of epidermal melanocytes. Autologous noncultured epidermal cell suspension (NCES; a cellular grafting technique) and suction blister epidermal grafting (SBEG; a tissue grafting technique) are important established surgical modalities for the treatment of stable vitiligo.

Objectives To compare the two techniques, NCES and SBEG, for producing repigmentation in patients with stable vitiligo.

Methods We randomized 41 patients with 54 stable vitiligo lesions into two groups. Patients in group 1 were treated with NCES, and those in group 2 with SBEG. They were evaluated 16 weeks postsurgery for the extent of repigmentation, colour match, change in Dermatology Life Quality Index (DLQI) score and patient satisfaction.

Results The extent of repigmentation was excellent (showing 90–100% repigmentation) in 71% of lesions in the NCES group and 27% of lesions in the SBEG group ($P = 0{\cdot}002$). Repigmentation $\geq 75\%$ (good repigmentation) was observed in 89% of lesions in the NCES group and 85% of lesions in the SBEG group ($P = 0{\cdot}61$). There was a significant decline in DLQI score in both the groups; the mean decline among groups differed significantly ($P = 0{\cdot}045$). No significant difference was seen in colour match and pattern of repigmentation. Adverse effects were minimal.

Conclusions NCES is significantly better than SBEG and should be the preferred treatment for patients with stable vitiligo. To best of our knowledge, this is the first study directly comparing these two techniques.

Vitiligo, commonly known as leucoderma or phulwari in India, is an acquired disorder of pigmentation. Worldwide prevalence of vitiligo is around 0·5–1%.[1] Loss of cutaneous pigmentation occurs because of loss of melanocytes from the basal layer of the epidermis. The aetiopathogenesis of vitiligo is multifactorial and polygenic including genetic, immunological, autoimmune and neurogenic factors, growth factor defects and environmental factors. Vitiligo initially starts as hypopigmented macules and patches which over a period of time become depigmented.[2] It is often associated with leucotrichia. Patients with vitiligo experience psychosocial distress and social stigmatization due to widespread prejudices, taboos and ignorance among the general population.[3]

Various treatment modalities (both medical and surgical) are available. The majority of cases are managed using medical therapies; surgical methods are reserved for lesions not responding to medical treatment and which are stable in nature. The basic principal surgical method is the transfer of melanocytes from uninvolved skin to the stable vitiligo patch in the form of either a tissue graft or a cellular graft. Among the surgical options, autologous noncultured epidermal cell suspension grafting (NCES; a cellular transplantation technique) and suction blister epidermal grafting (SBEG; a tissue transplantation technique) give promising results.

The major advantages of NCES are the smaller amount of donor skin needed to cover a large recipient area, little postoperative pain and discomfort, easier placement of the cellular graft and excellent colour match. The major advantages of SBEG are that it is a simple procedure requiring no laboratory setup and there is no scarring at the donor or recipient site as both sites heal simultaneously. On the basis of size of the recipient area, it is assumed that cellular transplantation methods

© 2012 The Authors

should be preferred when treating large areas, and tissue transplantation methods should be preferred when treating smaller areas.[4,5]

However, there is no consensus regarding situations such as (i) which surgical technique is preferred if stable vitiligo lesions are of equal size; (ii) which surgical technique will satisfy most of the patients; (iii) the effectiveness of the surgical technique at a particular site; or (iv) the surgical technique that is going to produce early repigmentation.

Therefore, this randomized study was planned to compare the outcome of these two surgical modalities in patients with stable vitiligo in terms of the extent of repigmentation, change in patients' psychosocial quality of life, speed of repigmentation and any adverse events.

Materials and methods

Subjects were recruited from the patients attending the pigmentary clinic of the Department of Dermatology, Venereology and Leprology, Postgraduate Institute of Medical Education and Research (PGIMER), Chandigarh, India between December 2010 and December 2011. A total of 41 patients with a clinical diagnosis of vitiligo that had been stable for a period of 1 year and not responding to medical therapy were recruited for the study. Patients aged < 10 years, those with actively spreading vitiligo, a history of koebnerization, hypertrophic and keloidal scars, a bleeding disorder or vitiligo patches of > 100 cm^2, and pregnant patients were excluded.

The ethical committee of PGIMER, Chandigarh approved the study and written consent was obtained from every patient. All the patients were randomly divided into two groups using a randomization table. Group 1 comprised 21 patients with 28 stable vitiligo lesions and group 2 comprised 20 patients with 26 stable vitiligo lesions. Group 1 was treated using autologous NCES and group 2 was treated with SBEG.

Technique of noncultured epidermal cell suspension grafting

About one-tenth the size of the recipient area was selected as the donor site. Hairy skin over the lateral side of the thigh was selected as the preferred donor site. The donor area was cleansed, shaved and anaesthetized with 2% lignocaine. A split-thickness skin graft was taken with the help of a shaving blade and straight artery forceps. The skin specimen was transferred under aseptic conditions to a container with normal saline and transferred to the laboratory. The graft was transferred to trypsin–ethylenediamine tetraacetic acid (EDTA) solution (0·25% trypsin and 0·02% EDTA) and incubated at 37 °C in 5% CO_2 for 1 h. Afterwards, the trypsin–EDTA solution was removed and phosphate-buffered saline (PBS) was added and pipetted well so as to separate the cells from the tissue.[6] The solid waste of tissue was removed and the suspension was centrifuged at 78 **g** for 5 min. The supernatant was then discarded and the pellet, containing cells from the stratum basale and the lower half of the stratum spinosum, which are rich in melanocytes, was taken. Around 1–3 mL of PBS according to recipient area was added to the pellet of melanocytes to make a suspension of noncultured epidermal cells. The recipient site was shaved, cleansed with Betadine® (Ayrton Saunders, Liverpool, U.K.) and surgical spirit and anaesthetized with 2% lignocaine. Dermabrasion was done with the help of a motorized dermabrader until punctate bleeding was seen. Dermabrasion was extended 5 mm beyond the margins to prevent halo phenomenon. The NCES was carefully transferred to the recipient site with the help of a tuberculin syringe and 18-gauge needle. A surgical dressing composed of Vaseline–chlorhexidine gauze, collagen and a sterile surgical pad was placed over the recipient site.

Technique of suction blister epidermal grafting

The anterolateral thigh was selected for raising blisters. The donor area was shaved and cleansed with Betadine and surgical spirit. The suction apparatus was created with the help of 20- and 50-mL syringes, a three-way cannula, small latex rubber tubes and a pressure gauge.[7] Suction syringes were placed over the stretched skin of the donor site. Suction was applied with the help of the 50-mL syringe and was monitored on the connected pressure gauge so as to create a negative pressure of about 300 mmHg. After this, the 50-mL syringe and the pressure gauge were removed and the free ends of the three-way tap were locked. A few small vesicles appeared in the suction area. After about 2 h, the small blisters coalesced to form a large blister. The recipient site was prepared and dermabraded in the same way as in the NCES technique until the appearance of punctate bleeding. The roof of the blister was cut with the help of curved scissors and transferred to a glass slide with the help of jeweller's forceps. Then the epidermal graft was transferred to the recipient site. After transplantation, the recipient site was covered with a surgical dressing as in NCES.

The patients in both the groups were asked to lie down for 1 h after procedure and then allowed to go home. The dressing was removed after 8 days at the first follow-up visit at the hospital.

Follow-up

Patients in both the groups were asked to attend for follow-up at the clinic on day 8, and weeks 4, 8, 12 and 16 after the transplantation procedure. We asked our patients to expose the area to sunlight at home starting from 5 min and up to a maximum of 30 min daily. This was much more convenient than asking the patient to attend the clinic two to three times per week for phototherapy sessions.

Repigmentation was assessed subjectively by digital photography as follows: < 50%, poor repigmentation; 50–74%, fair repigmentation; 75–89%, good repigmentation; 90–100%, excellent repigmentation. Also, the repigmentation pattern was noted as 'diffuse', 'perifollicular' or 'dotted'. A note was also made of the colour matching of the repigmented skin as

© 2012 The Authors

Table 1 Characteristics and disease parameters of the patients in the two groups

Characteristic	NCES group	SBEG group	P-value
Number of patients	21	20	
Number of lesions	28	26	
Age (years), mean ± SD (range)	21 ± 4·087 (12–27)	21·30 ± 6·001 (14–40)	0·852
Sex (F : M), n	14 : 7	12 : 8	0·658
Duration of disease (years), mean ± SD	6·05 ± 1·93	5·75 ± 2·845	0·696
Duration of stability (years), mean ± SD	2·05 ± 1·284	2·5 ± 2·039	0·398
Type of vitiligo (G/F/S), n	8/3/10	7/7/6	0·267
Size of treated area (cm^2), mean ± SD	15·25 ± 10·63	10·70 ± 7·41	0·137

NCES, noncultured epidermal cell suspension; SBEG, suction blister epidermal grafting; G, generalized; F, focal; S, segmental.

'somewhat lighter than', 'same as' or 'somewhat darker than' normal skin.

At each visit, patients were also asked about any adverse events and were asked to fill in a questionnaire about their satisfaction with the procedure results at weeks 4, 8, 12 and 16 and also a Dermatology Life Quality Index (DLQI) questionnaire at week 16. Hence, both objective and subjective evaluations of the results were carried out.

Statistical methods and data analysis procedures

The statistical analysis was carried out using Statistical Package for Social Sciences version 15.0 for Windows (SPSS Inc., Chicago, IL, U.S.A.). For normally distributed data, means were compared using Student's t-test for outcome. For skewed data or scores, the Mann–Whitney test was used. Qualitative or categorical variables were described as frequencies and proportions. Proportions were compared using chi-squared or Fisher's exact test, whichever was applicable. All statistical tests were two-sided and performed at a significance level of $\alpha = 0{\cdot}05$.

Results

All the patients completed the study period of 16 weeks and were included in the final analysis. The baseline characteristics of the patients in both groups are shown in Table 1. Patients in both groups were comparable. The size of treated area ranged from 3 to 35 cm^2 in the NCES group and from 3 to 32 cm^2 in the SBEG group. All patients had previously received various medical treatments for the management of their condition, including topical corticosteroids, topical calcineurin inhibitors, oral minipulse dexamethasone, psoralen plus ultraviolet (UV) A, psoralen with sunlight exposure (PUVASOL) and narrowband UVB. These patients had either not responded to the medical modalities of treatment, or had achieved a partial response with a few recalcitrant lesions remaining resistant to therapy.

Most patients did not report any postoperative discomfort. A few reported pain, which was easily relieved by oral analgesics. At the time of removal of the dressing, the treated area in the NCES group appeared bright pink and showed minimal erosion in some cases, which healed over a short period of time. In the SBEG group, the epidermal graft was found attached to the recipient area at the time of dressing removal. The graft was shed over the next few days and the lesion appeared erythematous. Erythema at the recipient site persisted and was gradually replaced by pigmentation. The earliest repigmentation was noticed between 4 and 6 weeks postsurgery and the pattern of repigmentation was mostly diffuse.

Repigmentation at 16 weeks postsurgery

In the NCES group, excellent repigmentation (90–100%) was observed in 20 out of 28 (71%) lesions, while in the SBEG group, excellent repigmentation was observed in only seven out of 26 (27%) lesions. This difference was statistically significant ($P = 0{\cdot}002$). Repigmentation ≥ 75% (good repigmentation) at week 16 was achieved in 25 out of the 28 (89%) lesions in the NCES group compared with 22 of the 26 (85%) lesions in the SBEG group ($P = 0{\cdot}61$) (Figs 1–7).

In 23 of the 28 lesions in the NCES group (79%) and 18 of 26 lesions in the SBEG group (69%), the colour of the repigmented area matched excellently with the normal surrounding skin. Four out of 28 lesions in the NCES group and three out of 26 lesions in the SBEG group showed somewhat darker

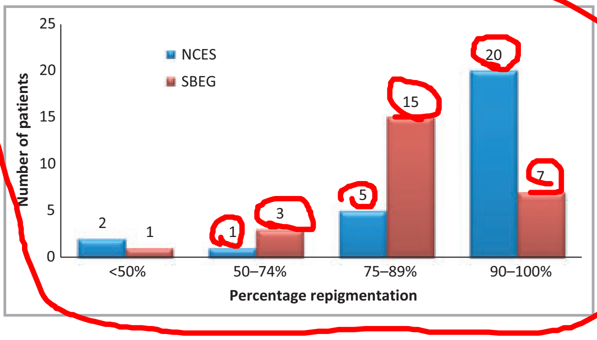

Fig 1. Extent of repigmentation at 16 weeks postsurgery. NCES, noncultured epidermal cell suspension; SBEG, suction blister epidermal grafting.

© 2012 The Authors

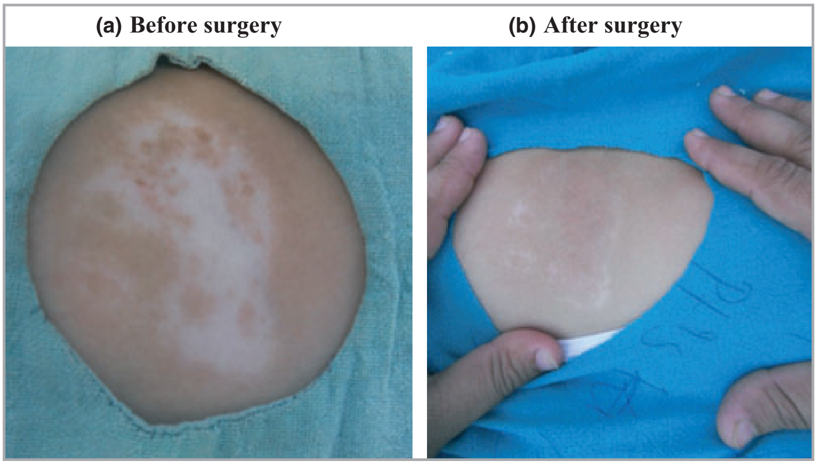

Fig 2. A patient in the noncultured epidermal cell suspension group with lesions over the trunk showing > 90% repigmentation. (a) Before surgery, (b) 16 weeks after surgery.

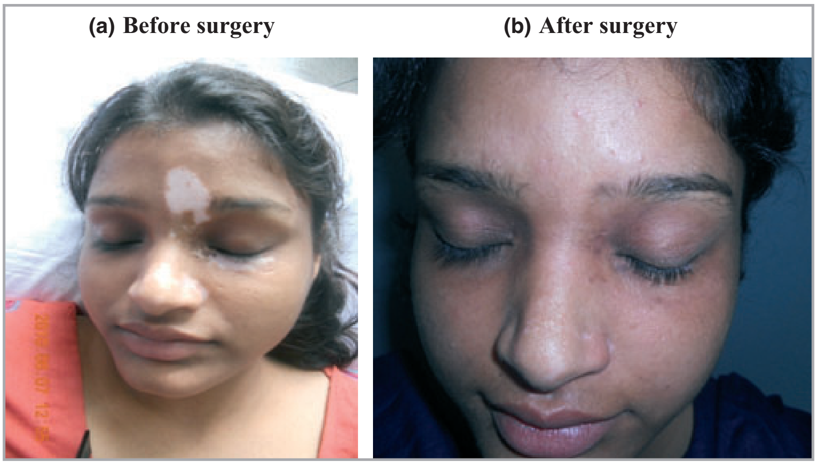

Fig 3. A patient in the noncultured epidermal cell suspension group with lesions over the face showing near 100% repigmentation. (a) Before surgery; (b) 16 weeks after surgery.

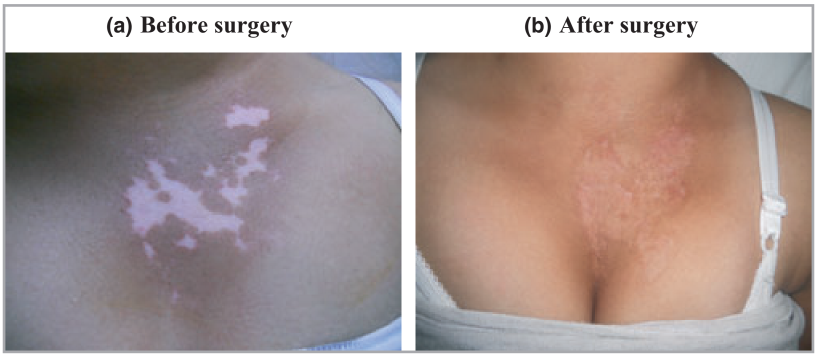

Fig 4. A patient in the noncultured epidermal cell suspension group with lesions over the trunk showing near 100% repigmentation. (a) Before surgery; (b) 16 weeks after surgery.

pigmentation and two out of 28 lesions in the NCES group and five out of 26 lesions in the SBEG group showed somewhat lighter pigmentation than normal skin, but with a tendency to match with normal skin colour over time.

There was no significant effect of age or sex of the patient, type of vitiligo, site and size of the treated area on the extent of repigmentation. Repigmentation started around 4 weeks earlier in the SBEG group than in the NCES group.

Change in Dermatology Life Quality Index and patient satisfaction

Comparing the mean change in DLQI score from before treatment to 16 weeks after surgery, we observed a highly significant ($P < 0{\cdot}001$) decline in DLQI score in both the groups, the mean reduction in DLQI being significantly greater in the NCES group than the SBEG group ($P = 0{\cdot}045$). In the NCES

© 2012 The Authors

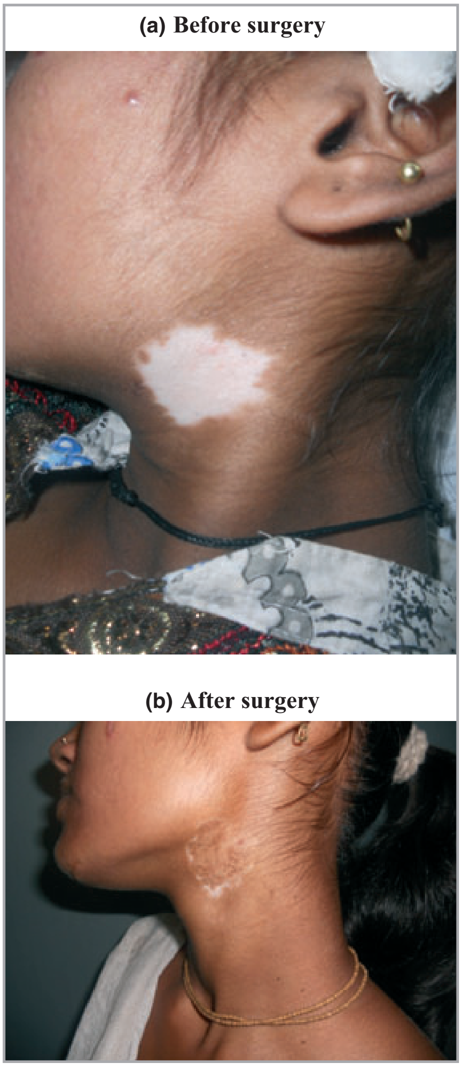

Fig 5. A patient in the suction blister epidermal grafting group with lesions over the neck showing > 90% repigmentation. (a) Before surgery; (b) 16 weeks after surgery.

group, the DLQI reduced from a mean value of 11·52 before surgery, to 2·24 after surgery, while in the SBEG group, it reduced from 9·70 before surgery to 2·90 after surgery.

Patients in both the groups were satisfied with the surgical intervention of their recalcitrant vitiligo. They were ready to opt for this treatment in future if needed. The mean score, median score and mean rank of the three patient-satisfaction questionnaires in both groups are given in Table 2. Patients in the NCES group were significantly more satisfied than the patients in SBEG group.

None of our patients in either of the groups developed infection, scarring or milia at any site – donor or recipient.

Discussion

Vitiligo is a difficult to manage condition despite the availability of several modalities of treatment. Surgical modalities appear to be the method of choice in recalcitrant stable vitiligo. Both NCES and SBEG are simpler and cheaper methods requiring minimal infrastructure.[8] The NCES method for the management of stable vitiligo was pioneered by Gauthier and Surleve-Bazeille in 1992.[9] Since then it has been used by a number of independent researchers with variable results. However, results have been inconsistent in some studies and there has been a need to refine the technique further. It is an important method of cellular transplantation in stable vitiligo, yet it has not been standardized and is still in the early phase of development. SBEG was first used as the treatment of stable vitiligo by Falabella[10] in 1971. Various researchers have described many modifications to this time-tested technique including the site of raising blisters, the method of applying pressure, and methods to increase the yield of blisters and decrease the suction blister induction time. However, no direct comparison between tissue and cellular transplant methods has been reported in the literature to date.

Some systematic review studies have been carried out. Njoo *et al.*[11] reviewed 39 studies, comprising minigrafting, split-thickness grafting, grafting of epidermal blisters, grafting of cultured melanocytes and NCES grafting. The highest success rates were achieved with split-thickness grafting [87%, 95% confidence interval (CI) 82–91%] and epidermal blister grafting (87%, 95% CI 83–90%). The lowest success rate was reported with NCES grafting (31%, 95% CI 11–59%). The reason behind this outcome can be explained by the fact that there were too few patients in the NCES group to draw a firm conclusion; also, this study was carried out in 1998, when the NCES method was still in its early stage of development.

Olsson and Juhlin[12] carried out a retrospective study to investigate the responses in vitiligo to three different trans-

Fig 6. A patient in the suction blister epidermal grafting group with lesions over the face showing > 75% repigmentation. (a) Before surgery; (b) 16 weeks after surgery.

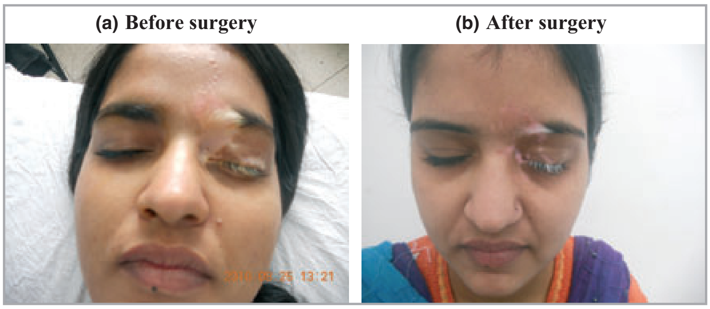

© 2012 The Authors

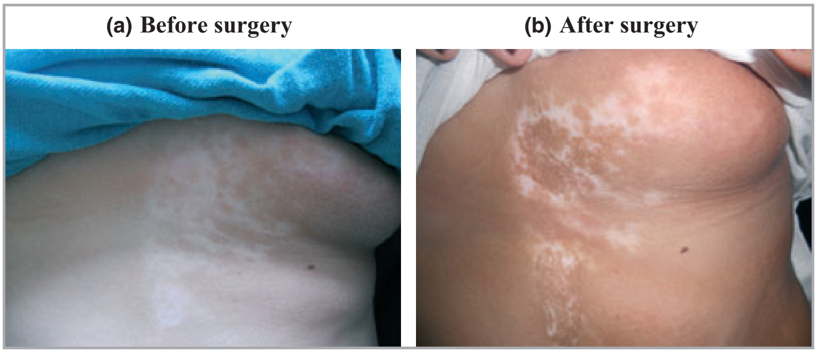

Fig 7. A patient in the suction blister epidermal grafting group with lesions over the trunk showing 50–74% repigmentation. (a) Before surgery; (b) 16 weeks after surgery.

Table 2 Measures of the three patient-satisfaction questionnaires (PSQ)

Measures	PSQ 1	PSQ 2	PSQ 3
Mean score			
NCES	8·14 ± 1·95	8·52 ± 1·96	8·57 ± 1·88
SBEG	7 ± 1·58	7·45 ± 1·66	7·5 ± 1·63
Median score			
NCES	9	9	8·85
SBEG	7·5	8	8
Mean rank			
NCES	25·93	25·93	26·26
SBEG	15·83	15·83	15·48
P-value	0·006	0·005	0·003

NCES, noncultured epidermal cell suspension; SBEG, suction blister epidermal grafting.

plantation methods: autologous cultured melanocytes, ultrathin epidermal sheets and basal cell layer suspension and concluded that (i) segmental vitiligo responded in most cases with 100% repigmentation; (ii) in vitiligo vulgaris, the ultrathin epidermal sheet method gave somewhat better results; (iii) irrespective of the method, fingers and elbows were the most difficult areas to repigment; and (iv) hyperpigmentation was common with the ultrathin epidermal sheath method.

In our study, at the end of 16 weeks, the NCES technique of vitiligo surgery was found significantly superior to the SBEG technique in producing excellent repigmentation. However, a good extent of repigmentation was obtained with both techniques. The significantly better results in terms of excellent repigmentation (90–100%) obtained with NCES over SBEG can be explained by the following hypotheses. (i) Repigmentation produced by epidermal grafts is usually confined to the area in contact and has little tendency to spread. We hypothesize that there is some difficulty or obstacle to pigment spread from the epidermal grafts in vitiligo patches. This could possibly explain the observation that vitiligo lesions in the SBEG group, although they exhibited faster early repigmentation, failed to show excellent repigmentation in significant numbers. However, good repigmentation was seen in most of the patients in the SBEG group. (ii) The average suction blister induction time in our patients was 1·5–2 h. According to Czajkowski *et al.*,[13] with increased suction time, the number of live melanocytes is decreased in the epidermis. This may be the reason why only 27% of the lesions in the SBEG group showed excellent repigmentation. (iii) Keratinocytes in the epidermal cell suspension help in growth and development of melanocytes.[14] However, in SBEG, the epidermal graft is shed 1–2 weeks after transplantation after releasing melanocytes to the recipient area. Therefore, we hypothesize that in SBEG, only melanocytes are transferred and virtually no keratinocytes, while in NCES, both melanocytes and keratinocytes are transferred, so melanocytes grow better in the presence of keratinocytes and produce excellent repigmentation. (iv) The epidermal grafts in SBEG are very thin and have a tendency to roll, split and displace from the recipient site with slight movement, resulting in a lesser extent of repigmentation. No such problems are encountered with NCES.

We used simplified methods in both the techniques of vitiligo surgery. In NCES, we applied the methods described by Holla *et al.*[6] who used PBS for making noncultured cellular suspension instead of melanocyte media. In SBEG, we followed the methods described by Gupta *et al.*[7] who used simple syringes for raising blisters.

While considering the speed of repigmentation, we observed that repigmentation was faster, and around 4 weeks earlier, in the SBEG than in the NCES group. The reason for faster repigmentation with SBEG can be explained by the fact that in SBEG we use epidermis that has a higher melanocyte concentration. Higher concentrations of melanocytes help in early repigmentation, but, on the other hand, we found that there was little spread of repigmentation achieved early in the SBEG group. According to our hypothesis, there is some resistance to pigment spread in epidermal grafts.

In the NCES technique, the concentration of melanocytes in suspension is lower than in a tissue graft and so the initial speed of repigmentation is slow. However, as the melanocytes repopulate the recipient site evenly, the final outcome is better in terms of extent of repigmentation. Keratinocytes are also transferred along with melanocytes in the NCES technique which helps in growth and survival of melanocytes.

The most commonly observed pattern of pigmentation was diffuse. This signifies that the repigmentation was due to melanocytes transplanted by NCES and SBEG. Perifollicular or marginal repigmentation is mainly induced by the melanocyte

© 2012 The Authors

reserve in the hair follicle, which is stimulated by dermabrasion or phototherapy.

We observed a significant reduction in DLQI[15] after surgery. A similar improvement in DLQI score was previously reported by van Geel *et al.*[16] In addition, the reduction in DLQI was significantly better in the NCES group compared with the SBEG group. Apart from DLQI, we also carried out patients' global assessment with the help of a patient-satisfaction questionnaire including three questions: 'Grade the change in pigmentation in the transplanted area', 'Are you satisfied with the treatment?' and 'Do you find the treatment worthwhile?' Patients were asked to answer in terms of scoring from 0 to 10. The scores obtained by this patient-satisfaction questionnaire were significantly higher in the NCES group than in the SBEG group. One particular problem faced by every patient in the SBEG group was that they had to lie in the same position for 2–3 h for suction blister induction. Patients were very uncomfortable with this situation. No such problem was faced by any of the patients in the NCES group. That is why patients in the NCES group were significantly more satisfied than those in the SBEG group.

Our study is the first of its kind in the literature to evaluate cellular transplantation techniques (NCES) in vitiligo compared with a tissue transplantation technique (SBEG). Cellular transplantation techniques have been found to be sophisticated and costly, to require laboratory input, and to be somewhat difficult to perform at a clinic. However, we used a much cheaper technique of NCES without any melanocyte culture media, trypsin inhibitor or hyaluronic acid. We did not use any sophisticated or costly suction apparatus. We just used a simple syringe-based suction apparatus.

To conclude, we compared two simplified techniques of vitiligo surgery. Our study indicates that both NCES and SBEG are safe and effective techniques in terms of good repigmentation (≥ 75% extent of repigmentation), colour matching and side-effect profile, but NCES is superior to SBEG in excellent repigmentation (90–100% extent of repigmentation), patient satisfaction and DLQI score reduction. However, further molecular level research is needed to elaborate the factors impeding pigment spread in SBEG and the factors provided by keratinocytes for the growth and survival of melanocytes in NCES.

What's already known about this topic?

- Both autologous noncultured epidermal cell suspension (NCES) and suction blister epidermal grafting (SBEG) offer promising treatments for stable vitiligo.
- With NCES (a cellular transplantation technique) a larger body surface area can be covered than that covered by SBEG (a tissue transplantation technique).

What does this study add?

- Our study is a direct comparison between NCES and SBEG in terms of extent of repigmentation, colour matching, pattern of repigmentation, patient satisfaction and Dermatology Life Quality Index (DLQI) score.
- Our study concludes that NCES has an edge over SBEG in terms of extent of repigmentation, patient satisfaction and DLQI.

References

1 Taieb A, Picardo M. The definition and assessment of vitiligo: a consensus report of the Vitiligo European Task Force. *Pigment Cell Res* 2007; **20**:27–35.
2 Njoo MD, Westerhof W. Vitiligo: pathogenesis and treatment. *Am J Clin Dermatol* 2001; **2**:167–81.
3 Thompson AR, Kent G, Smith JA. Living with vitiligo: dealing with difference. *Br J Health Psychol* 2002; **7**:213–25.
4 Mysore V, Salim T. Cellular grafts in management of leucoderma. *Indian J Dermatol* 2009; **54**:142–9.
5 Gauthier Y, Benzekri L. Non-cultured epidermal suspension in vitiligo: from laboratory to clinic. *Indian J Dermatol Venereol Leprol* 2012; **78**:59–63.
6 Holla AP, Kumar R, Parsad D, Kanwar AJ. Modified procedure of noncultured epidermal suspension transplantation: changes are the core of vitiligo surgery. *J Cutan Aesthet Surg* 2011; **4**:44–5.
7 Gupta S, Shroff S, Gupta S. Modified technique of suction blistering for epidermal grating in vitiligo. *Int J Dermatol* 1999; **38**:306–9.
8 Holla AP, Parsad D. Vitiligo surgery: its evolution as a definite treatment in the stable vitiligo. *G Ital Dermatol Venerol* 2010; **145**:79–88.
9 Gauthier Y, Surleve-Bazeille J. Autologous grafting with noncultured melanocytes: a simplified method for treatment of depigmented lesions. *J Am Acad Dermatol* 1992; **26**:191–4.
10 Falabella R. Epidermal grafting: an original technique and its application in achromic and granulating areas. *Arch Dermatol* 1971; **104**:592–600.
11 Njoo MD, Westerhof W, Bos JD, Bossuyt PM. A systematic review of autologous transplantation methods in vitiligo. *Arch Dermatol* 1998; **134**:1543–9.
12 Olsson MJ, Juhlin L. Long-term follow-up of leucoderma patients treated with transplants of autologous cultured melanocytes, ultrathin epidermal sheets and basal cell layer suspension. *Br J Dermatol* 2002; **147**:893–904.
13 Czajkowski R, Placek W, Drewa T *et al.* Autologous cultured melanocytes in vitiligo treatment. *Dermatol Surg* 2007; **33**:1027–36.
14 Guerra L, Capurro S, Melchi F *et al.* Treatment of 'stable' vitiligo by timed surgery and transplantation of cultured epidermal autografts. *Arch Dermatol* 2000; **136**:1380–9.
15 Finlay AY, Khan GK. Dermatology Life Quality Index (DLQI): a simple practical measure for routine clinical use. *Clin Exp Dermatol* 1994; **19**:210–16.
16 van Geel N, Ongenae K, De Mil M, Naeyaert JM. Modified technique of autologous noncultured epidermal cell transplantation for repigmenting vitiligo: a pilot study. *Dermatol Surg* 2001; **27**:873–6.

© 2012 The Authors
