# Supplementary material for: Therapeutic efficacy of cell-based therapy in vitiligo: a research letter systematically reviewed using meta-analysis
Source: Arch Dermatol Res. 2024 May 22;316(5):198. doi: 10.1007/s00403-024-02920-6 (PMC11111487; doi:10.1007/s00403-024-02920-6)
Supplement: Supplementary file 1 — Supplementary file1 (ZIP 24195 KB) [file 403_2024_2920_MOESM1_ESM.zip › Studies were included/RCT Czajkowski 2007.pdf]

Autologous Cultured Melanocytes in Vitiligo Treatment

Rafal Czajkowski, MD, PhD,*† Waldemar Placek, MD, PhD,* Tomasz Drewa, MD, PhD,† Bogna Kowaliszyn, MD, PhD,‡ Jan Sir, MD,§ and Wioletta Weiss, MD*

BACKGROUND Surgical treatment of vitiligo is indicated when lesions are localized in poorly responding areas.

OBJECTIVES The objectives were: (1) to establish the melanocyte culture obtained from the epidermis of vitiligo patients for future treatment; (2) to estimate the influence of selected factors on the formation of suction blisters and the results of culture; and (3) to compare the results of treatment of vitiliginous macules localized in the dorsum of the hands and lower limbs by transplantation of cultured autologous melanocytes plus psoralen and ultraviolet A (PUVA) therapy (CMP), suction blister transplantation plus PUVA therapy (SBP), cryotherapy plus PUVA-therapy (CP), and only PUVA therapy (OP).

METHODS Forty patients were qualified for the study. The roofs of the suction blisters were used as a melanocyte source for culture establishment or were directly transplanted.

RESULTS The CMP procedure was successfully performed on only 10 of 20 patients because of the difficulties in cell culture establishment. The SBP method was carried out on all 20 patients. A total lack of effectiveness was found in CP and OP methods.

CONCLUSIONS The effectiveness of culture depends on time of suction blister forming, phototype, and previous PUVA therapy. This study demonstrated the advantage of the SBP over the CMP method.

The authors have indicated no significant interest with commercial supporters.

Vitiligo is a common dermatosis characterized by patches of depigmentation and affecting approximately 0.1 to 2.0% of the world population.[1] The pathogenesis of vitiligo remains unclear, but there are several hypotheses trying to explain the etiology of the disease. Autoimmune, neural, self-destruct, biochemical, and genetic hypotheses are the most popular. The etiology of vitiligo is probably multifactorial and each of the factors plays the role in disease pathogenesis.[2] Vitiligo is a visible cosmetic defect that leads to serious emotional stress. The patients are characterized by low self-esteem, suffer more frequently from depression, and have difficulties in finding a job or starting a family.[3]

Causative treatment of vitiligo is not available, so current modalities are directed toward stopping progression and to achieving repigmentation. It is very difficult to treat vitiliginous lesions located on the back of the hands, feet, distal sections of limbs, eyelids, and genitals and around the mouth and nipples.[4] Surgical therapy is indicated in stable vitiligo when medical therapy (psoralen and ultraviolet A [PUVA] and immunomodulating therapy) fails. There are many surgical options including minigrafting, micropigmentation, split-thickness skin grafting, suction blister transplantation, transplantation of cultured autologous melanocytes, keratinocyte/melanocyte cocultures, or noncultured suspension of epidermal cells. Patients with lesions affecting more than 80% total body surface can be treated with depigmenting methods such as the application of monobenzylether of hydroquinone 20% or Q-switched ruby laser therapy.[4,5]

*Department of Dermatology and †Department of Tissue Engineering, Nicolaus Copernicus University, Ludwik Rydygier Medical College, Bydgoszcz; ‡Genetics and Fundamentals of Animal Breeding, Technical and Agricultural Academy, Bydgoszcz; §Depatment of Pathology, F. Lukaszczyk Oncology Center, Bydgoszcz, Poland

© 2007 by the American Society for Dermatologic Surgery, Inc. • Published by Blackwell Publishing •
ISSN: 1076-0512 • Dermatol Surg 2007;33:1027–1036 • DOI: 10.1111/j.1524-4725.2007.33216.x

The aims of the study were: (1) to establish the melanocyte culture obtained from the epidermis of vitiligo patients for future treatment; (2) to estimate the influence of selected factors on the formation of suction blisters and the results of culture; and (3) to compare the results of treatment of vitiliginous macules localized in the dorsum of the hands and lower limbs by transplantation of cultured autologous melanocytes plus PUVA therapy (CMP), suction blister transplantation plus PUVA therapy (SBP), cryotherapy plus PUVA therapy (CP), and only PUVA-therapy (OP).

Methods

Patients

Forty patients (25 women and 15 men) aged from 14 to 59 years (median, 30.2 years) were qualified for the study. Informed consent was obtained from each patient. The study protocol conformed to the ethical guidelines of the 1975 Declaration of Helsinki and was approved by our institutional human research review committee. On the basis of clinical tests, all the patients were diagnosed with a stable form of vitiligo with lesions on the backs of hands and feet and on the shins. The stable form of vitiligo was diagnosed in patients without appearance of new patches and without spreading of old vitiliginous lesions during the previous 6 months. The patients were randomly split into two groups of 20 patients. The first group consisted of 15 women and 5 men (mean age, 36.0 years); the second group consisted of 10 women and 10 men (mean age, 24.3 years). In the first group, the CMP procedure was performed on one of affected hands or legs and simultaneously OP was carried out on the other symmetrical limb (Table 1). Similarly, SBP and CP were performed as therapeutic procedures in the second group, each of them on one of symmetrical

TABLE 1. Clinical Data of the Patients Treated with CMP and OP Methods

Patient	*Sex*	*Age (years)*	*Fitzpatrick skin phototype*	*Localization of lesion*	*Duration of disease (years)*	*Previous PUVA therapy (weeks)*	*Number of grafts performed/ number of successful grafts with CMP method*
1	Female	54	III	Dorsum of the hands	21	2	8/8
2	Female	21	III	Dorsum of the hands	3	—	1/1
3	Female	26	III	Dorsum of the hands	11	3	7/7
4	Male	31	III	Dorsum of the hands	21	2	19/19
5	Female	27	II	Extensory surface of the lower legs	24	2	19/19
6	Female	43	III	Dorsum of the hands	6	3	8/8
7	Female	55	III	Dorsum of the hands	20	—	—
8	Male	45	III	Dorsum of the hands	15	—	—
9	Male	19	II	Dorsum of the hands	12	—	—
10	Female	35	II	Dorsum of the hands	5	—	—
11	Female	30	III	Dorsum of the feet	2	2	7/7
12	Female	26	III	Dorsum of the hands	1	2	8/8
13	Male	24	III	Dorsum of the feet	14	3	7/7
14	Female	39	III	Dorsum of the hands	5	2	3/3
15	Female	30	II	Dorsum of the hands	4	—	—
16	Female	51	II	Dorsum of the hands	34	—	—
17	Female	47	III	Dorsum of the hands	23	—	—
18	Male	29	III	Dorsum of the hands	1	—	—
19	Female	38	II	Dorsum of the hands	8	—	—
20	Female	51	II	Dorsum of the hands	34	2	—

CMP, cultured autologous melanocytes plus PUVA therapy; OP, only PUVA therapy.

TABLE 2. Clinical Data of the Patients Treated with SBP and CP Methods

Patient	Sex	Age (years)	Fitzpatrick skin phototype	Localization of lesion	Duration of disease (years)	Previous PUVA therapy	Number of grafts performed/number of successful grafts with CMP method
1	Male	23	III	Dorsum of the hands	6	—	8/8
2	Female	17	II	Dorsum of the hands	12	—	8/8
3	Female	30	II	Dorsum of the hands	14	—	2/2
4	Female	17	II	Dorsum of the hands	4	—	8/8
5	Male	22	III	Dorsum of the hands	6	—	5/3
6	Female	32	II	Dorsum of the hands	16	—	16/16
7	Male	20	III	Dorsum of the hands	8	—	8/8
8	Female	17	III	Dorsum of the feet	2	—	8/8
9	Male	47	III	Dorsum of the hands	2	—	8/8
10	Female	59	III	Dorsum of the hands	1	—	8/8
11	Male	17	III	Dorsum of the feet	1	—	5/5
12	Female	30	III	Dorsum of the hands	6	—	8/8
13	Male	15	III	Dorsum of the feet	1	—	8/8
14	Male	14	III	Dorsum of the feet	6	—	11/11
15	Female	15	II	Dorsum of the hands	5	—	5/5
16	Male	18	III	Dorsum of the feet	15	—	8/8
17	Female	19	III	Dorsum of the feet	8	—	8/8
18	Male	37	III	Dorsum of the feet	32	—	6/6
19	Female	19	III	Dorsum of the feet	10	—	8/8
20	Male	18	II	Dorsum of the feet	4	—	8/8

SBP, suction blister transplantation plus PUVA therapy; CP, cryotherapy plus PUVA therapy.

extremities (Table 2). On the basis of anamnesis, a phototype of the skin was established for all of the patients according to Fitzpatrick's classification. In the first group, 7 patients had skin phototype II and 13 had phototype III, and in the second group, 6 patients had skin phototype II and 14 had phototype III. The patients had not been subjected to phototherapy or immunotherapy during the previous 12 months, but 10 of the patients from the first group were given PUVA therapy for 2 to 3 weeks according to generally accepted principles before the roofs of blisters were taken for melanocyte cultures.

A comparison of the results of treatment by the different methods (CMP, SBP, CP, OP) was conducted after 6 months of PUVA therapy. In the case of the CMP, SBP, and CP methods, the number of successful procedures after which repigmentation was achieved was evaluated. Successful repigmentation means 100% repigmentation in the areas (each approx. 8 mm in diameter) covered with melanocytes suspension (CMP), suction blisters roofs (SBP), or treated with cryotherapy (CP). In patients treated only with PUVA therapy (OP), repigmentation in randomly selected points localized in the dorsum of the hands and lower limbs was evaluated.

Donor Site

The donor site was the arm or forearm, where, using an electric vacuum suction machine (−400 mmHg) connected with plastic plate (using elastic tube), suction blisters were formed (eight in the case of the CMP method and an appropriate number, depending on the size of the vitiliginous lesion, in the SBP method). The roof of the blister (approx. 8 mm in diameter) was delicately removed using microsurgical scissors (B. Braun Melsungen AG, Melsungen, Germany).

Recipient Site

Site Treated with CP Blisters were produced with the application of nitrous oxide (cryoapplicator 8 mm in diameter). The site for freezing was sprayed with

neomycin sulfate and provided with a dressing (Bactigras, Smith & Nephew, London, UK) for 7 days.

Site Treated with OP PUVA therapy was performed according to generally accepted principles—3 × per week, initial dose UVA 0.5 J/cm^2, 1.2 mg of 5-methoxypsoralen per kilogram of body weight.

Site Treated with Transplantation of CMP The roofs of the blisters were placed in petri dishes filled with a solution of 0.25% trypsin/EDTA (Sigma, St Louis, MO) placing the tissues with the surface of the horny layer on the bottom of the dish. The tissues were incubated for 15 minutes at an atmosphere of 5.0% CO_2 and a temperature of 37.0°C in an incubator (IG 150, Jouan, Saint-Herblain, France). Using a scalpel, the basal layer of the roofs of the blisters was scraped off and then a trypsin inhibitor (soya protein; Sigma) was added. The cell suspension was centrifuged, the supernatant was discarded, and the cells were transferred by pipette to two culture flasks with a growth surface area of 25 cm^2 each (Greiner, Kremsmunster, Austria). The culture flasks contained culture medium MGM M2 (PromoCell, Heidelberg, Germany) containing only one growth factor [recombined human basic fibroblast growth factor (rhbFGF)], supplemented with geneticin at a concentration of 100 μg/mL medium (Gibco BRL, Grand Island, NY). Geneticin was used for the first 3 days of culture to eliminate keratinocytes and fibroblasts. The melanocytes were incubated at an atmosphere of 5.0% CO_2 and a temperature of 37.0°C for the period necessary to obtain the appropriate number of pigment cells to perform the transplant. The medium was changed every 3 days. While the culture liquid was being changed, the viability and morphology of the cells were evaluated under an inverted optic microscope (TMS + F, Nikon, Tokyo, Japan). The cells were passaged when they covered approximately 80% of the growth surface area of the culture flask. Immediately before the transplantation, melanocytes were suspended in a phosphate-buffered saline solution with a calcium and magnesium content at a quantity of 5 × 10^5 cells per 1 cm^2 of skin surface. The suspension prepared in this way was transplanted into the previously formed blisters, as already described. The transplantation site was sprayed with neomycin sulfate and provided with a dressing (Bactigras, Smith & Nephew) for 7 days. Before transplantation, the viability and quantity of cells in the suspension were evaluated using 0.4% trypan blue solution (Sigma). The presence of melanocytes in the culture was confirmed on the basis of an analysis of the morphology of the cells (inverted optic microscope TMS + F, Nikon) and immunocytochemical analysis using anti-HMB-45 and anti-S100 (DakoCytomation, Glostrup, Denmark) antibodies (Figure 1). Photographic documentation of the culture was performed using a digital camera (Coolpix 4500, Nikon) in the first 24 hours of culture and before each passage of melanocytes.

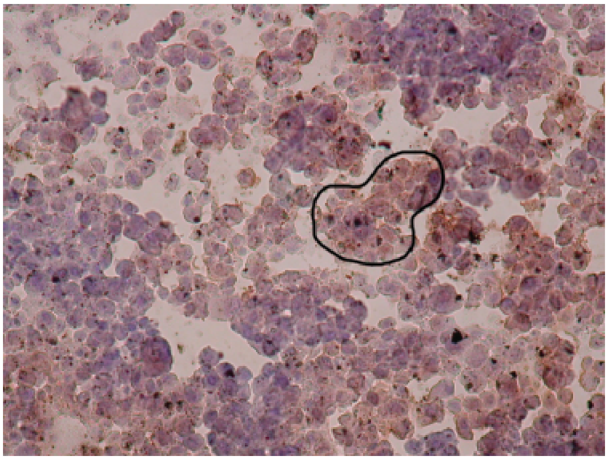

Figure 1. Cultured cells are HMB-45–positive, confirming the identity of the cells. The melanocytes containing melanosomes in early stages are brown (arrow). (EnVision + System- HRP; original magnification, × 200.)

Site Treated with SBP The removed roofs of the suction blisters were placed basal layer down on microscopic glass moistened with a drop of physiologic saline and then transplanted on to the site of the removed roofs of previously formed blisters, as already described. The transplantation site was sprayed with neomycin sulfate and provided with a dressing (Bactigras, Smith & Nephew), which was changed on the second day after transplantation and removed 7 days after transplantation.

Statistical Analysis

An analysis of the influence of the investigated factors on suction blisters creation was carried out by a multifactor variance analysis. The significance of the differences between the averages was verified using Scheffe's test on the level $p = .05$ and $p = .01$. Because of the lack of a normal distribution of the researched variables (numbers of melanocytes received from culture), a transformation was performed using a natural logarithm. To improve the readability of these data, the basic measurements were also calculated for the location in the investigated subgroups for the number of cells–median (Me). The influence of transplantation technique on repigmentation after surgical treatment was carried out by a chi-square test with Yates correction. Treatment efficacy (number of transplants performed/number of successful transplants $\times$ 100%) was carried out by Kruskal–Wallis test (lack of a normal distribution).

Results

Evaluation of the Donor Site

All of the patients were given a total of 336 blisters using the suction blister method in a time of between 45 and 100 minutes (Figure 2). The time for producing the blisters in 25 women was on average 53.0 $\pm$ 16.90 minutes, whereas in the 15 men it was on average 55.73 $\pm$ 29.52 minutes. The suction

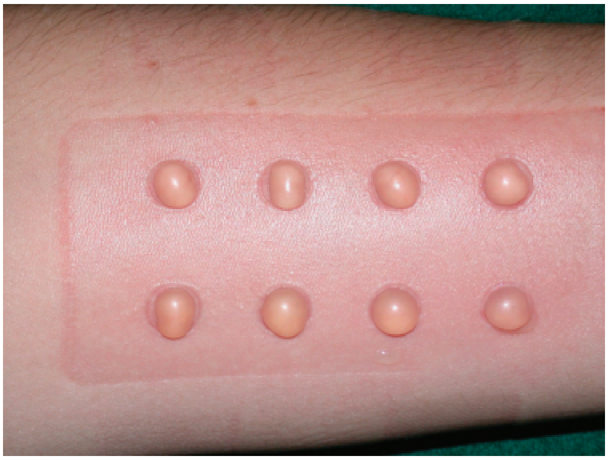

Figure 2. Suction blisters in donor site.

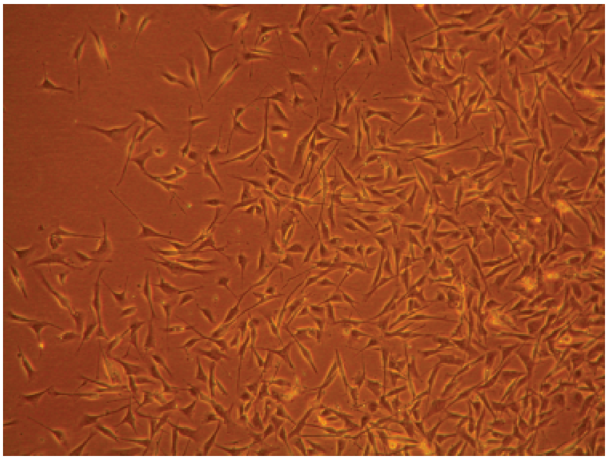

Figure 3. The culture of melanocytes observed in inverted microscope (original magnification, $\times$ 100).

blisters appeared on the arm of 20 patients in a mean time of 48.60 $\pm$ 7.17 minutes and on the forearm in 20 patients in a mean time of 59.45 $\pm$ 25.53 minutes. The time for producing the blisters in 12 patients ($\leq$19 years of age) was 54.67 $\pm$ 22.09 minutes, 19 patients (20–39 years of age) was 53.11 $\pm$ 22.64 minutes, and 9 patients ($\geq$40 years of age) was 55.11 $\pm$ 25.21 minutes. A statistically highly significant difference was found in the time for the formation of a blister depending on the sex of the patient ($p = .01$) and a highly significant difference statistically depending on the place of forming the blister ($p = .01$). A statistically significant difference was not found in the time of forming suction blisters depending on the age of the patient.

Evaluation of the Melanocyte Culture

From the roofs of eight blisters, epidermal cells were isolated and a primary culture was established for 18 patients (Figure 3). In the case of 2 patients, where the time for forming blisters was 58 and 90 minutes, respectively, it was not possible to establish a primary melanocyte culture. A highly statistically significantly greater number of melanocytes was obtained from the skin of the 10 patients' forearms (median, $1.03 \times 10^6 \pm 118.39$) than from the skin of the 10 patients' arms (median, $8.3 \times 10^4 \pm 158.15$). A sufficient quantity of melanocytes for conducting the transplantation

procedure was obtained for 10 patients (9 with skin phototype III and 1 with phototype II). A sufficient number of melanocytes was not cultivated for 10 patients (4 with phototype III and 6 with phototype II). The number of melanocytes cultivated from the roofs of blisters obtained from 13 patients with skin phototype III (median, $1.8 \times 10^6 \pm 101.90$) is statistically highly significantly greater than the number of pigment cells obtained from 7 patients with skin phototype II (median, $8.0 \times 10^4 \pm 238.34$). A highly significant difference statistically was also found for the number of cells obtained from patients who had previously been given PUVA therapy (2 weeks, median, $2.0 \times 10^6 \pm 79.40$; 3 weeks, median, $1.8 \times 10^6 \pm 6.19$) compared with patients not treated earlier with the PUVA method (median, $7.2 \times 10^4 \pm 90.99$).

Evaluation of Recipient Site

In 10 patients from the first group, a total of 87 grafts were performed with 100% success (Figure 4). Repigmentation on the second limb, which had been treated only with PUVA therapy (OP), was not observed in any patient.

In all the patients from the second group (SBP), a total of 154 grafts were performed with and repigmentation was obtained in 152 cases (98.7%; Figure 5). In the case of limbs treated with CP, no repigmentation was obtained.

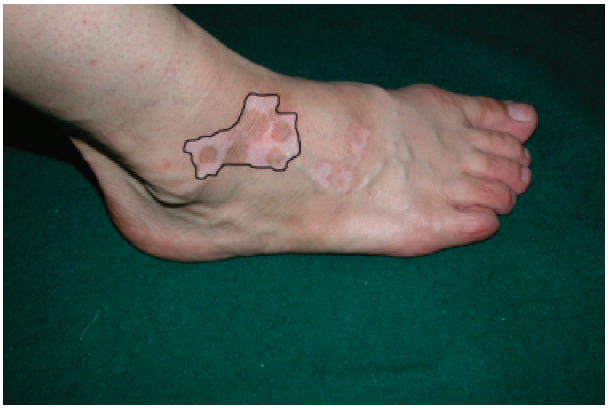

Figure 4. Six months after transplantation of cultured autologous melanocytes plus PUVA therapy (CMP). The area after transplantation is marked.

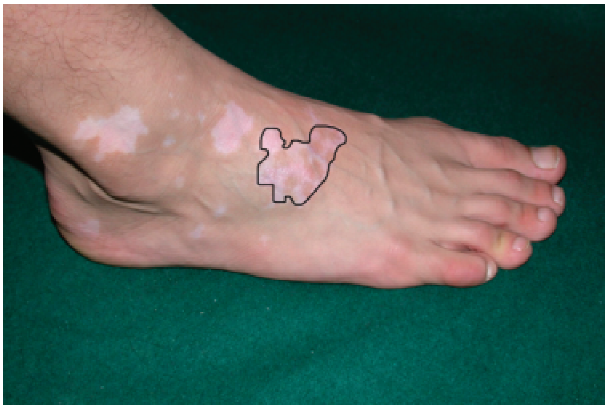

Figure 5. Six months after suction blister transplantation plus PUVA therapy (SBP). The area after transplantation is marked.

Transplantation of cultured autologous melanocytes was performed in only 10 out of 20 patients, while suction blister transplantation was performed in all 20 (a statistically highly significant difference, $p = .000793$). No significant difference was found between the number of successful grafts in both groups of patients ($p = .4795$).

Discussion

Vitiliginous lesions localized in the dorsum of the hands, distal sections of limbs, and genitals and around the mouth and nipples are characterized by a poor response to conservative therapy (pharmacologic therapy, phototherapy, photochemotherapy, immunotherapy).[4] It is possible to receive repigmentation using surgical treatment based on the transfer of autologous melanocytes to the vitiliginous lesions. Many methods for the surgical treatment of vitiligo are known which enable more or less satisfactory repigmentation to be obtained, depending on the technique chosen.[5,6] Culture methods create the possibility of obtaining a large number of cells from a small area of removed epidermis (e.g., the roofs of suction blisters). Only in some research do the authors describe the differences in the time needed for producing a suction blister depending on various factors as well as the influence of different factors on melanocyte culture.[7,8]

In this study, we have estimated the influence of selected factors (sex, age, donor site) on the formation of suction blisters and the results of melanocyte culture depending on donor site, skin phototype, and previous PUVA therapy. The outcome of treatment of vitiliginous macules localized in the dorsum of the hands and lower limbs by transplantation of CMP, SBP, CP, and OP were also compared. Owing to small areas selected, all four procedures (CMP, SBP, CP, and OP) could not be carried out in each patient.

In this research it was shown that blisters formed considerably more quickly on the arm than on the forearm and faster in women than in men. Peachey[9] obtained similar results in 1971 researching the speed of formation of blisters using the suction blister method in healthy volunteers. The suction time can be shortened by increasing the temperature of the skin. In this study, after applying a temperature of approximately 38°C, blisters formed in a mean time of 48.6 minutes on the arm and 59.45 minutes on the forearm. Peachey[10] measured the time for the formation of blisters in 24 volunteers at temperatures of 34, 37, and 40°C. Kiistala[11] conducted similar research by warming the skin of patients to 39.4°C. The results confirmed that increasing the temperature of the donor site of the epidermis shortens the suction time. With an increase in temperature, the stickiness of the serous fluid filling the blister decreases, which hastens its development.[12] An earlier theory presuming the contribution of enzymatic mechanisms in the process of separating the epidermis from the dermis is unfounded because the majority of proteolytic enzymes reach their greatest activity at a temperature of 37°C.[10]

The culture methods create the possibility to obtain a large number of cells from a small size donor site. Culture media contain different growth factors ensuring optimal conditions for the proliferation of cells. In 1982, Eisinger and Marko[13] were the first to use 12-*O*-tetradeacynol-phorbol-13-acetate (TPA) and cholera toxin in the culture of melanocytes obtained from newborn foreskin. During subsequent years, the culture technique was improved, adding or replacing growth factors in culture media. In 1987, Lerner and coworkers[14] performed the first transplantation of cultured autologous melanocytes in a patient suffering from vitiligo, obtaining a very good cosmetic effect. A suitable number of melanocytes for the transplantation was obtained using the medium elaborated by Eisinger and Marko, supplemented with 3-isobutyl-1-methylxanthine. The majority of culture media used for clinical purposes contain bovine serum. The addition of patients' serum to the medium could accelerate the proliferation of pigment cells. In accordance with the autoimmunologic theory of the pathogenesis of vitiligo, however, there exists a great risk of the melanocytes being destroyed by antibodies in the serum. After analyzing potential advantages and risks connected with the transplantation of cultured autologous melanocytes, MGM M2 was used for the study, devoid of bovine serum and TPA. The only growth factor contained in the medium is rhbFGF.

In most cases a sufficient number of melanocytes were obtained from the skin of the forearm or arm, this being a good source of pigment cells. A culture of autologous melanocytes enabling a transplant to be performed was successfully established mainly for patients who had been given PUVA therapy before the melanocytes being taken. The intensification of the proliferation and the increase in the activity of pigment cells of the epidermis after PUVA therapy could result from the action of psoralens that, under the influence of UVA radiation, activate the differentiation of melanoblasts and melanocytes and the release of α-MSH by keratinocytes. α-MSH is a mitogen for melanocytes and stimulates the production of metalloproteinase-2, which facilitates the migration of pigment cells from the hair follicle to the surrounding epidermis by loosening intercellular junctions.[15,16]

The impossibility of establishing culture in the case of two patients, in whom the suction blisters appeared after a time of more than 58 minutes, suggests that with increased suction time, the number of live melanocytes in the epidermis decreases

and remaining cells are insufficient to culture establishment. In one piece of research it was shown that the time of proliferation of melanocytes in in vitro conditions depends also on the age of the patient and the place where cells are taken for culture. Pigment cells from newborn foreskin undergo division the fastest, while cells isolated from the epidermis of the arm of adults are the slowest.[17] In this study, it was shown that melanocytes from the epidermis of the forearm proliferate faster than pigment cells isolated from the epidermis of the arm of adults. The forearm is more frequently exposed to solar radiation than arm and probably because of that the number of melanocytes in culture is higher in the case of pigment cells isolated from blister roof produced on forearm.

The color of the skin not only depends on the number of melanocytes, but it depends on activity of pigment cells and melanoblasts size.[18] The melanocytes of the patients with skin phototype III are more active than melanocytes received from the patients with skin phototype II. A higher melanocyte activity in the skin phototype III individuals makes it possible to obtain more melanocytes in culture than from epidermis of skin phototype II patients. The low proliferation activity of pigment cells obtained from some patients might also result from a defect or an insufficient number of receptors for rhbFGF (only growth factor contained in the MGM M2).

Melanocytes used for transplantation can also come from biopsy specimens of healthy skin obtained using a scalpel or dermatome and hair follicles from the occipital region of the hair-covered skin of the head or eyebrows.[19,20] The process of isolating melanocytes from skin of full thickness lasts considerably longer than when using the epidermis alone. The short time of trypsinization reduces the risk of irreversible damage to the pigment cells and increases the chances of establishing culture. The procedure of cutting off the roofs of blisters obtained using the suction blister method is safe and painless and does not leave scars, keloids, or the Koebner phenomenon. Therefore, it seems to be a good technique to obtain material for transplantation in patients who have been properly qualified for treatment. The treatment of vitiligo using the transplant of cultivated autologous melanocytes requires the ability to conduct a cell culture and to use reagents and laboratory equipment, which considerably increases the cost and lengthens the treatment process.

The method of creating suction blisters was first described by Kiistala in 1964,[21] and the first transplantation of suction blisters roofs in vitiligo patient was carried out by Falabela in 1971.[22] Until now many publications have appeared describing the effectiveness of treating vitiligo with suction blister transplantation.[23,24] This study confirmed earlier observations by dermatosurgeons concerning the effectiveness of the method and the ease of performance of the procedure.[25] Only transitional hyperpigmentation was observed as regards transplanted epidermis, lasting for approximately 2 months. This procedure is characterized by very high effectiveness, regardless of the duration of the formation of suction blisters in the donor site.

The absence of pigmentation after using CP and PUVA therapy alone testifies to the ineffectiveness of these methods of treatment in the case of vitiliginous lesions localized in the dorsum of the hands and lower limbs. The ineffectiveness of these methods should be sought in the small number of hair follicles from which melanocytes migrate during epidermization and PUVA therapy.

This study demonstrated the advantage of the suction blister transplantation method over the culture method because of the difficulties in cell culture establishment in some vitiligo patients. We have not observed depigmentation in surgically treated areas after 6 months, but final outcome of surgical techniques should be estimated after long-term observation.

Acknowledgment This work was supported by the State Committee for Scientific Research in Poland (No. 3P05B 156 23).

References

1. Alkhateeb A, Fain PR, Thody A, et al. Epidemiology of vitiligo and associated autoimmune diseases in Caucasian probands and their families. Pigment Cell Res 2003;16:1–7.
2. Grimes PE. New insights and new therapies in vitiligo. JAMA 2005;293:730–5.
3. Thompson AR, Kent G, Smith JA. Living with vitiligo: dealing with difference. Br J Health Psychol 2002;7:213–25.
4. Halder RM, Young CM. New and emerging therapies for vitiligo. Dermatol Clin 2000;18:79–89.
5. Mulekar SV. Long-term follow-up study of 142 patients with vitiligo vulgaris treated by autologous, non-cultured melanocyte-keratinocyte cell transplantation. Int J Dermatol 2005;44:841–5.
6. Njoo MD, Westerhof W, Bos JD, Bossuyt PMM. A systematic review of autologous transplantation methods in vitiligo. Arch Dermatol 1998;134:1543–9.
7. Gupta S, Kumar B. Suction blister induction time: 15 minutes or 150 minutes? Dermatol Surg 2000;26:754–6.
8. Hsu MY, Li L, Herlyn M. Cultivation of normal human epidermal melanocytes in the absence of phorbol esters. Methods Mol Med 2005;107:13–28.
9. Peachey RD. Some factors affecting the speed of suction blister formation in normal subjects. Br J Dermatol 1971;84:435–52.
10. Peachey RD. Skin temperature and blood flow in relation to the speed of suction blister formation. Br J Dermatol 1971; 84:447–52.
11. Kiistala U. Dermal-epidermal separation. II. External factors in suction blister formation with special reference to the effect of temperature. Ann Clin Res 1972;4:236–46.
12. van der Leun JC, Lowe LB, Beerens EG. The influence of skin temperature on dermal-epidermal adherence: evidence compatible with a highly viscous bond. 1974;62:42–6.
13. Eisinger M, Marko O. Selective proliferation of normal human melanocytes in vitro in the presence of phorbol ester and cholera toxin. Proc Natl Acad Sci U S A 1982;79:2018–22.
14. Lerner AB, Halaban R, Klaus SN, Moellmann GE. Transplantation of human melanocytes. J Invest Dermatol 1987;89:219–24.
15. Lei TC, Vieira WD, Hearing VJ. In vitro migration of melanoblasts requires matrix metalloproteinase-2: implications to vitiligo therapy by photochemotherapy. Pigment Cell Res 2002;15: 426–32.
16. Lee AY, Jang JH. Autologous epidermal grafting with PUVA-irradiated donor skin for the treatment of vitiligo. Int J Dermatol 1998;37:551–4.
17. Abdel-Malek ZA, Swope VB, Nordlund JJ, Medrano EE. Proliferation and propagation of human melanocytes in vitro are affected by donor age and anatomical site. Pigment Cell Res 1994;7:116–22.
18. Diamond J. Evolutionary biology: geography and skin colour. Nature 2005;435:283–4.
19. Baltaci V, Kilic A. A new application for reconstruction of areola with transplantation of cultured autologous melanocytes. Plast Reconstr Surg 1997;101:1056–9.
20. Na GY, Seo SK, Choi SK. Single hair grafting for the treatment of vitiligo. J Am Acad Dermatol 1998;38:580–4.
21. Kiistala U. In vivo separation of epidermis by production of suction blister. Lancet 1964;1:1444.
22. Falabella R. Epidermal grafting: an original technique and its application in achromic and granulating areas. Arch Dermatol 1971;104:592–600.
23. Gupta S, Kumar B. Epidermal grafting in vitiligo: influence of age, site of lesion and type of disease on outcome. J Am Acad Dermatol 2003;49:99–104.
24. Gupta S, Kumar B. Epidermal grafting for vitiligo in adolescents. Pediatr Dermatol 2002;19:159–62.
25. Mutalik S, Ginzburg A. Surgical management of stable vitiligo: a review with personal experience. Dermatol Surg 2000;26: 248–54.

Address correspondence and reprint requests to: R. Czajkowski, MD, PhD, Department of Dermatology, Nicolaus Copernicus University, Ludwik Rydygier Medical College, Ul. Kurpinskiego 5, 85-096 Bydgoszcz, Poland, or e-mail: rafal.czajkowski@poczta.pf.pl

COMMENTARY

Vitiligo is a common skin depigmenting disease resulting from the loss of melanocytes in the cutaneous epidermis. The etiology of the disorder remains obscure and curative therapies are not currently available. Several immunosuppressive treatments including corticosteroids and photochemotherapy have been used to induce repigmentation in vitiligo patients but these have given mixed and usually unsustained responses. Surgical techniques including minigrafting, suction blister transplantation, and the transplantation of autologous melanocytes have also been applied to the treatment of vitiligo but not always with satisfactory results. In the article by Czajkowski and colleagues, various surgical modalities in conjunction with photochemotherapy have been compared for the treatment of vitiligo. Most successful appeared to

be the use of suction blisters to transplant melanocytes to depigmented lesions plus concurrent treatment with psoralen and ultraviolet A radiation (PUVA). In all patients, depigmentation was not observed in the 6 months after surgery. Of course, follow-up of these individuals will be required to determine the long-term effectiveness of this mode of therapy. The technique avoids the need to culture autologous melanocytes, which is often difficult with some vitiligo patients, and the use of PUVA may suppress immune responses against the newly transplanted pigment cells.

Although, the current study demonstrates a relatively effective treatment for vitiligo, the etiology of the disease needs to be understood before completely effective and routine therapies can be established. Particularly, the immune reactions against melanocytes that may cause recurrent depigmentation must be further studied. In addition, different clinical types of vitiligo may have different etiologies, and this needs to be determined because it might affect the degree to which a certain treatment is successful. Indeed, it is already known that autologous skin grafting is less applicable to generalized (nonsegmental) vitiligo and this may be related to causative factors.

E. Helen Kemp
Sheffield, UK
