# Supplementary material for: Therapeutic efficacy of cell-based therapy in vitiligo: a research letter systematically reviewed using meta-analysis
Source: Arch Dermatol Res. 2024 May 22;316(5):198. doi: 10.1007/s00403-024-02920-6 (PMC11111487; doi:10.1007/s00403-024-02920-6)
Supplement: Supplementary file 1 — Supplementary file1 (ZIP 24195 KB) [file 403_2024_2920_MOESM1_ESM.zip › Studies were included/RCT Czajkowski 2004.pdf]

ORIGINAL ARTICLE

Comparison of Melanocytes Transplantation Methods for the Treatment of Vitiligo

RAFAL CZAJKOWSKI, MD

Department of Dermatology and Department of Medical Biology, Ludwik Rydygier Medical University, Bydgoszcz, Poland

BACKGROUND. **Surgical therapy of vitiligo is indicated when depigmented macules are localized in areas that are known to respond poorly.**

OBJECTIVE. **The objective was to compare the results of treatment of vitiliginous macules localized in the dorsum of the hands and lower limbs by transplantation of cultured autologous melanocytes plus PUVA therapy (CMP), suction blister transplantation plus PUVA therapy (SBP), cryotherapy plus PUVA therapy (CP), and only PUVA therapy (OP).**

METHODS. **Twenty patients qualified for the study. The patients were split into two groups of 10 patients. In the first group, the CMP procedure was performed on one limb and OP on the other. In the second group, SBP and CP were used, respectively.**

RESULTS. **The CMP procedure was successfully performed on only 6 of 10 patients, whereas SBP was carried out on all 10 patients. No significant difference was found between the number of successful transplants in both groups of patients. A total lack of effectiveness was found in CP and OP methods.**

CONCLUSION. **This study demonstrated the advantage of the suction blister transplantation method over the autologous cultured melanocytes transplantation method because of the difficulties in cell culture establishment in some cases.**

RAFAL CZAJKOWSKI, MD HAS INDICATED NO SIGNIFICANT INTEREST WITH COMMERCIAL SUPPORTERS.

VITILIGO IS an acquired disease of uncertain etiology, affecting approximately 0.1% to 2.0% of the world population.[1] There are several hypotheses trying to explain the pathogenesis of the disease. Autoimmune, neural, self-destruct, biochemical, and genetic hypotheses are the most popular. It seems that the etiology of vitiligo is multifactorial and each of the factors plays an important role in pathogenesis.[2] Vitiligo is a visible cosmetic defect that leads to serious emotional stress in about two-thirds of patients. Psychosocial problems include depression, low self-esteem, job discrimination, and even social rejection.[3]

Causative treatment of vitiligo is not available, so current modalities are directed toward stopping progression and to achieving repigmentation by means of surgical and nonsurgical methods. Among currently accepted nonsurgical methods of treating vitiligo, the most common are psoralen plus ultraviolet A therapy (PUVA therapy) and immunomodulating therapy. Surgical therapy is indicated in stable vitiligo when medical therapy fails and when depigmented macules are localized in such areas as lips, nipples, genitals, eyelids, and distal extremities that are known to respond poorly. There are currently many surgical options including split-thickness skin grafting, minigrafting, suction blister transplantation, micropigmentation, transplantation of cultured autologous melanocytes, keratinocyte/melanocyte cocultures, or noncultured suspension of epidermal cells. Patients with widespread lesions (more than 80% total body surface) can be treated with depigmenting methods such as the application of monobenzylether of hydroquinone 20% or Q-switched ruby laser therapy.[2,4] The cell culture methods are comparatively new options in experimental medicine. There are a low number of publications evaluating the effectiveness of vitiligo therapy by the cultured melanocytes transplantation method. In our study, we have compared the results of treatment of vitiliginous macules localized in the dorsum of the hands and lower limbs by transplantation of cultured autologous melanocytes plus PUVA therapy (CMP), suction blister transplantation plus PUVA therapy (SBP), cryotherapy plus PUVA therapy (CP), and only PUVA therapy (OP).

Materials and Methods

Patients

Twenty patients (13 women and 7 men) aged from 17 to 59 years (median, 32.0 years) qualified for the study. All had localized (focalis) or generalized (acrofacial) vitiligo with lesions localized in the dorsum of the hands or lower limbs. The patients were randomly split into two groups of 10 patients. The first group

Address correspondence and reprint requests to: Rafal Czajkowski, MD, Department of Dermatology, Ludwik Rydygier Medical University, Ul. Kurpin skiego 5, 85-096 Bydgoszcz, Poland, or e-mail: rafal.czajkowski@pf.pl.

© 2004 by the American Society for Dermatologic Surgery, Inc. • Published by Blackwell Publishing, Inc.
ISSN: 1076-0512/04/$15.00/0 • Dermatol Surg 2004;30:1400–1405

consisted of 7 women and 3 men (mean age, 35.6 years); the second group consisted of 6 women and 4 men (mean age, 28.4 years). In the first group, the CMP procedure was performed on one limb and OP on the other (Table 1). In the second group, SBP and CP were used, respectively (Table 2). All patients had previously received nonsurgical treatments with limited or no success. The mean duration of the disease was 4.2 years in the first group and 3.1 years in the second group. The patients had not had any vitiligo therapy for 12 months before the study. A comparison of the results of treatment by the different methods (CMP, SBP, CP, OP) was conducted after 6 months of PUVA therapy. In the case of the CMP, SBP, and CP methods, the number of successful procedures after which repigmentation was achieved was evaluated. Successful procedure means 100% repigmentation in the areas (each about 8 mm in diameter) covered with melanocytes suspension (CMP), suction blisters roofs (SBP), or treated with cryotherapy (CP). In patients treated only with PUVA therapy (OP), repigmentation in randomly selected points localized in the dorsum of the hands and lower limbs was evaluated.

Donor Site

The donor site was the forearm, where, using an electric vacuum suction machine (–400 mmHg) and a plastic plate, suction blisters were formed (eight in the case of the CMP method and an appropriate number, depending on the size of the vitiliginous lesion, in the SBP method). The roof of the blister (8 mm in diameter) was delicately removed using scissors.

Recipient Site

Site treated with CP

Blisters were produced with the application of nitrous oxide (cryoapplicator 8 mm in diameter). The duration of freezing lasted from 5 to 10 s. The site for freezing was sprayed with neomycin sulfate and provided with a nonadherent gauze dressing for 7 days.

Site treated with OP

Photochemotherapy PUVA was performed according to generally accepted principles—3 × per week; initial dose of UVA, 0.5 J/cm^2; and 5-methoxypsoralen, 1.2 mg/kg body weight.

Site treated with transplantation of CMP (Figure 1)

The removed roofs of the suction blisters were placed in petri dishes filled with 0.25% trypsin/EDTA solution (Sigma) so that the surface of the horny layer of the tissue was face down. The epidermis was incubated for 15 min in an atmosphere of 5% CO_2 and at a temperature of 37°C. Using a scalpel, the basal layer of the epidermis was scraped off and trypsin inhibitor

Table 1. Clinical Data of the Patients Qualified for the Study (First Group)

Patient	Sex	Age (Years)	Localization of Lesion	Clinical Type of Vitiligo	Time (min) Required for Suction Blister Formation	Transplantation of CMP: Duration (Days) of Cell Culture	Transplantation of CMP: Start (Day) of Repigmentation	Transplantation of CMP: Number of Transplants Performed/ Number of Successful Transplants	OP: Number of Randomly Chosen Points/Number of Repigmented Points
S.C.	F	54	Dorsum of the hands	Generalized (acrofacial)	45	87	16th	8/8	8/0
J.B.	F	21	Dorsum of the hands	Localized (focalis)	53	15	14th	1/1	1/0
M.N.	F	26	Dorsum of the hands	Generalized (acrofacial)	51	68	15th	7/7	7/0
S.B.	M	31	Dorsum of the hands	Generalized (acrofacial)	47	60	21st	1/1	1/0
D.S.	F	27	Extensory surface of the lower legs	Generalized (acrofacial)	50	80	13th	19/19	19/0
M.G.	F	43	Dorsum of the hands	Generalized (acrofacial)	54	65	18th	8/8	8/0
A.Z.	F	55	Dorsum of the hands	Generalized (acrofacial)	58	—	—	—	8/0
K.N.	M	45	Dorsum of the hands	Generalized (acrofacial)	90	—	—	—	8/0
N.W.	M	19	Dorsum of the hands	Generalized (acrofacial)	46	13	—	—	8/0
M.P.	F	35	Dorsum of the hands	Generalized (acrofacial)	48	10	—	—	8/0

Abbreviations: CMP, cultured autologous melanocytes plus PUVA therapy; OP, PUVA therapy only.

Table 2. Clinical Data of the Patients Qualified for the Study (Second Group)

					Transplantation of SBP			
Patient	*Sex*	*Age (Years)*	*Localization of Lesion*	*Clinical Type of Vitiligo*	*Time (min) Required for Suction Blister Formation*	*Start (Day) of Repigmentation*	*Number of Transplants Performed/ Number of Successful Transplants*	*CP: Number of Frozen Points/ Number of Repigmented Points*
P.G	M	23	Dorsum of the hands	Generalized (acrofacial)	52	12th	8/8	8/0
J.K.	F	17	Dorsum of the hands	Generalized (acrofacial)	60	10th	8/8	8/0
M.M.	F	30	Dorsum of the hands	Generalized (acrofacial)	54	16th	2/2	2/0
A.M.	F	17	Dorsum of the hands	Generalized (acrofacial)	90	14th	8/8	8/0
D.K.	M	22	Dorsum of the hands	Generalized (acrofacial)	100	20th	5/3	5/0
L.S.	F	32	Dorsum of the hands	Generalized (acrofacial)	58	13th	16/16	16/0
K.D.	M	20	Dorsum of the hands	Generalized (acrofacial)	47	17th	8/8	8/0
J.G.	F	17	Dorsum of the feet	Generalized (acrofacial)	58	15th	8/8	8/0
A.D.	M	47	Dorsum of the hands	Generalized (acrofacial)	56	14th	8/8	8/0
H.K.	F	59	Dorsum of the hands	Generalized (acrofacial)	49	11th	8/8	8/0

Abbreviations: SBP, suction blister transplantation plus PUVA therapy; CP, cryotherapy plus PUVA therapy.

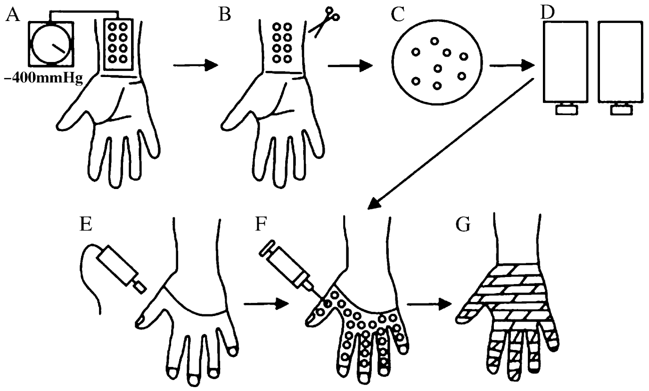

Figure 1. Therapeutic procedure of cultured autologous melanocytes transplantation in vitiligo patients. (A) Formation of suction blisters (in donor site); (B) removal of the roofs of suction blisters; (C) enzymatic bath of the epidermis; (D) establishing melanocyte culture; (E) preparation of the recipient site using a cryoapplicator (formation of blisters); (F) transplantation of the cultured melanocyte suspension under the roof of the blister using a syringe; (G) application of the dressing.

(soybean) (Sigma) was added. The cell suspension was centrifuged at a speed of 2000 rpm for 5 min.The supernatant was removed, while the sediment containing the cells was transferred to culture flasks containing melanocyte growth medium M2 (PromoCell, Heidelberg, Germany), supplemented with geneticin (100 μg/mL) (GibcoBRL, Carlsbad, CA). Geneticin was used for the first 3 days of culture to eliminate keratinocytes and fibroblasts. Melanocytes were incubated in an atmosphere of 5% CO_2 and at a temperature of 37°C for the length of time necessary to obtain a suitable number of pigment cells. The medium was changed every 3 days. Immediately before the transplantation, melanocytes were suspended in a phosphate-buffered saline solution with a calcium and magnesium content at a quantity of 5×10^5 cells per 1 cm^2 of skin surface. The suspension prepared in this way was transplanted into the previously formed blisters, as already described. The transplantation site was sprayed with neomycin sulfate and provided with a nonadherent gauze dressing for 7 days. Before transplantation, the viability and quantity of cells in the suspension were evaluated using 0.4% trypan blue solution (Sigma Chemical Co., St. Louis, MO). The presence of melanocytes in the culture was confirmed on the basis of an evaluation of cell morphology under the optic microscope (Nikon) and also immunocytochemically (antibodies S-100) (Dako, Carpinteria, CA).

Site treated with SBP (Figure 2)

The removed roofs of the suction blisters were placed basal layer down on microscopic glass moistened with a drop of physiologic saline and then transplanted on to the site of the removed roofs of previously formed blisters, as already described. The transplantation site was sprayed with neomycin sulfate and provided with a nonadherent gauze dressing, which was changed on the second day after transplantation and removed 7 days after transplantation.

The Student's t test was used for statistical analysis.

Results

In all of the patients from the first group (CMP), eight suction blisters were formed over a period of between 45 and 90 min (on average 54.2 min). Over 15 to 87 days (mean, 62.5 days), melanocytes were cultivated in six patients in sufficient quantities for transplantation. No melanocyte culture was established for two pa-

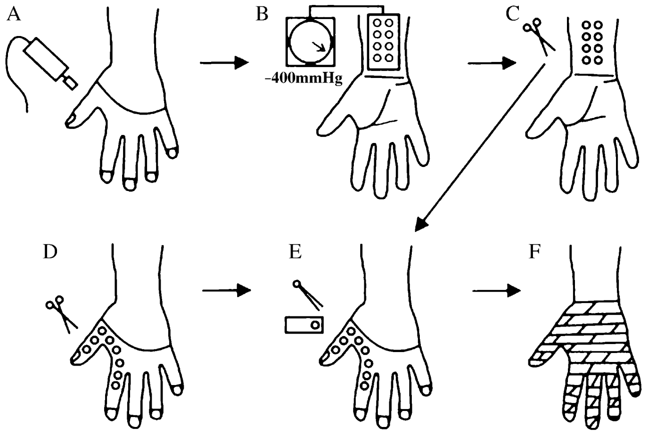

Figure 2. Therapeutic procedure of suction blister transplantation in vitiligo patients. (A) Preparation of the recipient site using a cryoapplicator (formation of blisters); (B) formation of suction blisters (in donor site); (C) removal of the roofs of suction blisters; (D) removal of the roofs of blisters in the recipient site; (E) transfer of the roofs of blisters from the donor site to the recipient site using tweezers and microscopic glass; (F) application of the dressing.

tients whose suction blisters appeared over a time period that was longer than/equal to 58 min. In addition, a suitable quantity of pigment cells was not obtained for two other patients. In the case of the transplantation performed under the roof of the blister covering the whole vitiliginous lesion, an unequal distribution of the pigment was obtained with hyperpigmentation on the periphery of the lesion. A reduction of the diameter of the blisters in the transplantation site to 8 mm enabled proper, uniform pigmentation to be obtained (Figure 3). In six patients from the first group, a total of 44 transplantations were performed with 100% success. Repigmentation began 13 to 21 days after PUVA therapy was applied (mean, 16.1 days). Repigmentation on the second limb, which had been treated with OP, was not observed in any patient.

In all the patients from the second group (SBP), depending on the size of the vitiliginous lesion, between 2 and 16 suction blisters were formed over a time period of between 47 and 100 min (mean, 62.4 min). Ten patients were given a total of 79 transplantations, and repigmentation was obtained in 77 cases (97.47%). The beginning of repigmentation was observed 10 to 20 days after PUVA therapy was applied (mean, 14.2 days) (Figure 4). In the case of limbs treated with CP no repigmentation was obtained.

Transplantation of cultured autologous melanocytes was performed in only 6 of 10 patients, whereas suction blister transplantation was performed in all 10 patients. No significant difference was found between the number of successful transplantations in both groups of patients ($p = 0.34$). Equally, no significant difference was found regarding the start of repigmentation ($p = 0.22$).

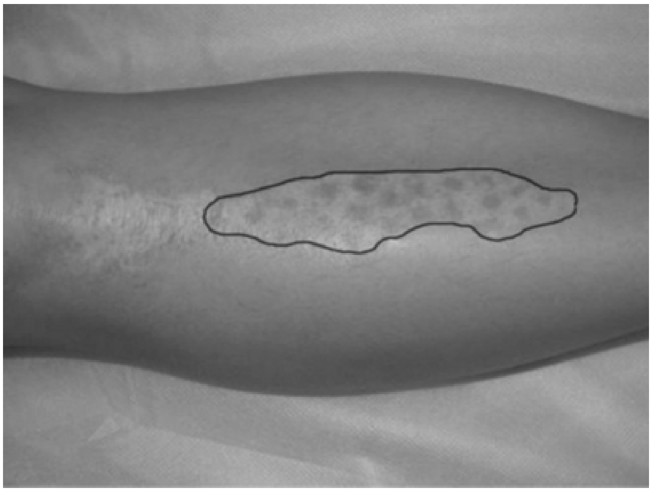

Figure 3. Patient D.S. 6 months after transplantation of CMP. The area after transplantation is marked.

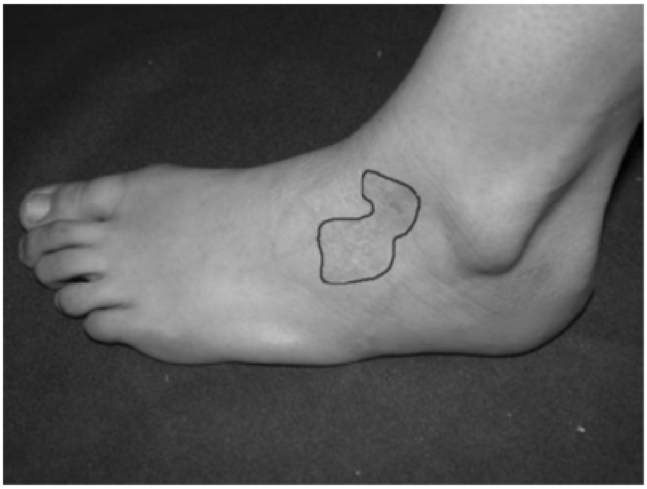

Figure 4. Patient J.G. 6 months after SBP. The area after transplantation is marked.

Discussion

The lack of diagnostic-therapeutic management standards in vitiligo confronts the dermatologist with a difficult choice regarding the method of treatment. Vitiliginous lesions localized in the dorsum of the hands and lower limbs are characterized by a very weak response to nonsurgical therapy (pharmacologic therapy, phototherapy, photochemotherapy, immunotherapy).[2] Many methods for the surgical treatment of vitiligo are known which enable more or less satisfactory repigmentation to be obtained, depending on the technique chosen.[5] In this study, we have compared the results of treatment of vitiliginous macules localized in the dorsum of the hands and lower limbs by transplantation of CMP, SBP, CP, and OP.

The culture methods create the possibility of obtaining a large number of cells from a donor site of

small size. Culture media contain different growth factors ensuring optimal conditions for the proliferation of cells. In 1982, Eisinger and Marko[6] were the first to use 12-O-tetradecanoyl-phorbol-13-acetate and cholera toxin in the culture of melanocytes obtained from newborn foreskin. During subsequent years, the culture technique was improved, adding or replacing growth factors in culture media. In 1987, Lerner et al.[7] performed the first transplantation of cultured autologous melanocytes in a patient suffering from vitiligo, obtaining a very good cosmetic effect. A suitable number of melanocytes for the transplantation was obtained using the medium elaborated by Eisinger and Marko,[7] supplemented with 3-isobutyl-1-methylxanthine. The majority of culture media used for clinical purposes contain bovine serum. Owing to the potential risk of transmitting diseases caused by prions, media devoid of serum have been elaborated. The addition of patients' serum to the medium could accelerate the proliferation of pigment cells. In accordance with the autoimmunologic theory of the pathogenesis of vitiligo, however, there exists a great risk of the melanocytes being destroyed by antibodies in the serum. Equally, a solution has not been found to the problem concerning 12-O-tetradecanoyl-phorbol-13-acetate, which is considered by many authors to be an oncogenic factor.[8] After analyzing potential advantages and risks connected with the transplantation of cultured autologous melanocytes, melanocyte growth medium M2 was used for the study, devoid of bovine serum and 12-O-tetradecanoyl-phorbol-13-acetate. The only growth factor contained in the medium is recombinant human basic fibroblast growth factor.

Melanocytes are cells that are characterized by slow proliferation. Under in vitro conditions, at least two synergistically acting mitogens are required for the rapid proliferation of pigment cells, for example, basic fibroblast growth factor, hepatocyte growth factor/scatter factor, mast/stem cell factor, endothelin-1, 12-O-tetradecanoyl-phorbol-13-acetate, cholera toxin, and 3-isobutyl-1-methylxanthine.[9] Culture medium enriched only in recombinant human basic fibroblast growth factor (a proliferation stimulator of melanocytes and melanogenesis) ensures a very slow growth of melanocytes, which significantly prolongs the culture process and sometimes renders it impossible. The low proliferation activity of pigment cells obtained from some patients might also result from a defect or an insufficient number of receptors for recombinant human basic fibroblast growth factor.

Melanocytes occur in the epidermis of humans in different densities, depending on the anatomic localization. The greatest percentage of melanocytes is found in the genital region, then in the skin of the head and neck, back, upper, and lower limbs. The smallest number of melanocytes is found in the skin of the abdomen and chest (with the exception of the nipples).[10] To establish a melanocyte culture, epidermis from the forearm was used, obtained using the suction method. In the majority of cases, a sufficient quantity of melanocytes was obtained from the skin of the forearm, which is a good source of pigment cells. The number of melanocytes obtained from the roof of a suction blister is approximately 40% to 60% lower in comparison with the quantity of melanocytes evaluated in skin biopsy specimens.[11] As the number of pigment cells obtained from the donor site falls, the chances of a successful culture being performed are reduced. The impossibility of establishing a culture in the case of two patients whose suction blisters were formed in a period of time over 58 min suggests that with increasing "suction" time, the number of melanocytes in the epidermis decreases.

Melanocytes used for transplantation can also come from biopsy specimens of healthy skin obtained using a scalpel or dermatome, from pigmented moles and hair follicles from the occipital region of the skin from a hair-covered head.[7,12,13] The process of isolation of melanocytes from full-thickness skin lasts considerably longer than when only the epidermis is used. To loosen intercellular junctions, an enzymatic bath is required (e.g., in a 0.25% solution of trypsin/EDTA), which only lasts a few minutes in the case of the epidermis. The short time for trypsinization reduces the risk of irreversible damage to the pigment cells and increases the chances of establishing culture. The procedure of removing the epidermis from the suction blister is safe and painless. It does not cause scarring or the Koebner phenomenon and that is why it should be the method of choice for the preparation of the donor site in culture techniques and the method of direct transplantation of the epidermis. Equally, we did not observe complications regarding the recipient site after cultured melanocytes transplantation. Treating vitiligo with transplantation of cultured autologous melanocytes requires the ability to perform cell culture and to use reagents and laboratory equipment, which considerably raises costs and prolongs the period of therapy. The use of "safe" growth medium devoid of 12-O-tetradecanoyl-phorbol-13-acetate and bovine serum reduces the rate of proliferation of melanocytes and thus restricts the possibility of using culture techniques in clinical practice.

The method of creating suction blisters was first described by Kiistala in 1968.[14] Until now many publications have appeared describing the effectiveness of treating vitiligo with suction blister transplantation.[5,15] This study confirmed earlier observations by dermatosurgeons concerning not only the effectiveness of the method, but also the ease of performance of the

procedure and the absence of complications in the donor and recipient sites.[16] Only transitional hyperpigmentation was observed regarding transplanted epidermis, lasting for about 2 months. This procedure is characterized by very high effectiveness, regardless of the duration of the formation of suction blisters in the donor site. It should also be emphasized that the suction blister transplantation method is a rapid and relatively cheap method.

The absence of pigmentation after using cryotherapy plus PUVA therapy and PUVA therapy alone testifies to the ineffectiveness of these methods of treatment in the case of vitiliginous lesions localized in the dorsum of the hands and lower limbs. The ineffectiveness of these methods should be sought in the small number of hair follicles from which melanocytes migrate during epidermization and PUVA therapy.

A few methods for the preparation of the recipient site for the transplantation of cultured melanocytes and the suction blister transplantation are known. The aim of all of the methods is to remove the epidermis or to separate it from the dermis, which ensures optimal growth conditions for the transplanted melanocytes. The most popular techniques are freezing with liquid nitrogen or nitrous oxide, dermabrasion, the use of lasers, diathermosurgery (Timedsurgery), chemical epilation, and ultrasonic abrasion.[17–20] In the study, freezing with nitrous oxide was used, resulting in blisters in the recipient site. In the transplantation of cultured autologous melanocytes method, an important role is played by the size of the frozen blister. In the case of lesions covering the dorsum of the hands and fingers, a better cosmetic result is obtained using the small blister method. The surface of the fingers is convex, and because of this, if one large blister is frozen over the whole lesion, there is the risk of the melanocyte suspension running to the periphery of the lesion. The unequal distribution of pigment cells leads to hyperpigmentation of the periphery and hypopigmentation of the central part of the lesion. The small blister method makes it possible to distribute the melanocytes uniformly, and the pigmentation obtained is identical to the color of healthy skin from the beginning.

This study demonstrated the advantage of the suction blister transplantation method over the autologous cultured melanocytes transplantation method because of the difficulties in cell culture establishment in some cases. Total ineffectiveness of cryotherapy plus PUVA therapy and PUVA therapy alone in the treatment of vitiliginous lesions localized in the dorsum of the hands and lower limbs was proven. Owing to the simple technique of the procedure, the low costs, the short time required for the transplantation, and the lack of complications, the suction blister transplantation method should be the method of choice in the treatment of vitiliginous lesions localized in sites where repigmentation is difficult. The lack of any significant difference between the number of successful transplantations in both groups of patients and the absence of transitional hyperpigmentation after the procedure of transplantation of cultured autologous melanocytes prompts further research into culture media that would ensure the rapid and safe growth of pigment cells.

Acknowledgments This work was supported by the State Committee for Scientific Research in Poland (No. 3P05B 156 23)

References

1. Alkhateeb A, Fain PR, Thody A, Bennett DC, Spritz RA. Epidemiology of vitiligo and associated autoimmune diseases in Caucasian probands and their families. Pigment Cell Res 2003;16:1–7.
2. Njoo MD, Westerhof W. Vitiligo pathogenesis and treatment. Am J Clin Dermatol 2001;2:167–81.
3. Thompson AR, Kent G, Smith JA. Living with vitiligo: dealing with difference. Br J Health Psychol 2002;7:213–25.
4. Halder RM, Young CM. New and emerging therapies for vitiligo. Dermatol Clin 2000;18:79–89.
5. Njoo MD, Westerhof W, Bos JD, Bossuyt PM. A systematic review of autologous transplantation methods in vitiligo. Arch Dermatol 1998;134:1543–9.
6. Eisinger M, Marko O. Selective proliferation of normal human melanocytes in vitro in the presence of phorbol ester and cholera toxin. Proc Natl Acad Sci U S A 1982;79:2018–22.
7. Lerner AB, Halaban R, Klaus SN, Moellmann GE. Transplantation of human melanocytes. J Invest Dermatol 1987;89:219–24.
8. Arita Y, Santiago-Schwarz F, Coppock DL. Survival mechanisms induced by 12-O-tetradecanoylphorbol-13-acetate in normal human melanocytes include inhibition of apoptosis and increased Bcl-2 expression. Melanoma Res 2000;10:412–20.
9. Halaban R. The regulation of normal melanocyte proliferation. Pigment Cell Res 2000;13:4–14.
10. Whiteman DC, Parsons PG, Green AC. Determinants of melanocyte density in adult human skin. Arch Dermatol Res 1999;291:511–6.
11. Lee AY, Jang JH. Autologous epidermal grafting with PUVA-irradiated donor skin for the treatment of vitiligo. Int J Dermatol 1998;37:551–4.
12. Baltaci V, Kilic A. A new application for reconstruction of areola with transplantation of cultured autologous melanocytes. Plast Reconstr Surg 1997;101:1056–9.
13. Na GY, Seo SK, Choi SK. Single hair grafting for the treatment of vitiligo. J Am Acad Dermatol 1998;38:580–4.
14. Kiistala U. In vivo separation of epidermis by production of suction blister. Lancet 1964;1:1444.
15. Gupta S, Kumar B. Epidermal grafting in vitiligo: influence of age, site of lesion and type of disease on outcome. J Am Acad Dermatol 2003;49:99–104.
16. Mutalik S, Ginzburg A. Surgical management of stable vitiligo: a review with personal experience. Dermatol Surg 2000;26:248–54.
17. Guerra L, Capurro S, Melchi F, et al. Treatment of "stable" vitiligo by timedsurgery and transplantation of cultured epidermal autografts. Arch Dermatol 2000;136:1380–9.
18. Kaufmann R, Greiner D, Kippenberger S, Bernd A. Grafting of in vitro cultured melanocytes onto laser-ablated lesions in vitiligo. Acta Derm Venerol 1998;78:136–8.
19. Kim CY, Yoon TJ, Kim TH. Epidermal grafting after chemical epilation in the treatment of vitiligo. Dermatol Surg 2001;27:855–6.
20. Tsukamoto K, Osada A, Kitamura R, et al. Approaches to repigmentation of vitiligo skin: new treatment with ultrasonic abrasion, seedgrafting and psoralen plus ultraviolet A therapy. Pigment Cell Res 2002;15:331–4.
