# Supplementary material for: Therapeutic efficacy of cell-based therapy in vitiligo: a research letter systematically reviewed using meta-analysis
Source: Arch Dermatol Res. 2024 May 22;316(5):198. doi: 10.1007/s00403-024-02920-6 (PMC11111487; doi:10.1007/s00403-024-02920-6)
Supplement: Supplementary file 1 — Supplementary file1 (ZIP 24195 KB) [file 403_2024_2920_MOESM1_ESM.zip › Studies were included/RCT Ghosh 2012.pdf]

Efficacy and Safety of Autologous Cultured Melanocytes Delivered on Poly (DL-Lactic Acid) Film: A Prospective, Open-Label, Randomized, Multicenter Study

DEEPA GHOSH, PHD,* PUSHPA KUCHROO, MS,* CHANDRA VISWANATHAN, MD, PHD,* SHAILENDRA SACHAN, MBBS,† BELA SHAH, MD,‡ DEEPA BHATT, MD,§ SHRICHAND PARASRAMANI, MD,¶ AND SATISH SAVANT, MD||

BACKGROUND Small vitiliginous patches have been treated with epidermal grafts or their cell suspensions. In an attempt to overcome some of the shortcomings of cell suspension delivery, we have delivered melanocytes on a polymeric film.

OBJECTIVES To evaluate the clinical effectiveness of a cultured graft consisting of autologous cultured melanocytes on a poly (DL-lactic acid) (PLA) film in subjects with stable vitiligo.

METHODS A prospective open-label, randomized, multicenter clinical trial was conducted with 22 patients. Each subject was treated with cultured graft and polyurethane dressing (control arm) after epidermal ablation and followed for up to 9 months. The extent of repigmentation in the treated sites was compared with that control sites at days 90, 180, and 270.

RESULTS In the treatment arm, a minimum of 70% repigmentation was observed in five subjects at day 90; nine at day 180, and 10 at day 270. In the control arm, only one subject showed repigmentation until day 270. None of the test sites reported any recurrence of vitiliginous patches by the end of the study.

CONCLUSIONS Cultured melanocytes delivered on PLA film were efficacious and safe when applied on patients with stable vitiligo.

This study was funded by Reliance Life Sciences.

Vitiligo is a common acquired disorder characterized by white patches on the skin resulting from loss of melanocytes. The incidence of vitiligo worldwide is approximately 1% to 2%.[1] There is no cure for vitiligo, but there are a number of treatment options that improve the condition. The main aim of treating vitiligo is to restore pigmentation in the affected area and improve the patient's appearance. Conventional treatment for vitiligo includes narrow-band ultraviolet B light therapy, psoralen plus ultraviolet A light (PUVA) therapy, PUVA sol therapy, local steroids, and local immunosuppressive agents such as pimecrolimus cream. Such treatments usually take a few months, and the results are not always satisfactory.

Autologous skin transplantation is an option to treat patients with stable vitiligo who do not respond to conventional treatment. The aim is to repopulate areas lacking melanocytes with functional cells taken from normally pigmented areas. Autologous epidermis transfer procedures such as thin split-thickness grafts have been performed successfully, with repigmentation ranging from 80% to 95%.[2–4]

*Tissue Engineering Group, Regenerative Medicine, Navi Mumbai, India; †Clinical Research Group, Reliance Life Sciences Pvt. Ltd., Navi Mumbai, India; ‡B. J. Medical College and Civil Hospital, Ahmedabad, India; §SCL Municipal and General Hospital, Ahmedabad, India; ¶Anisha Clinic, Khar, Mumbai, India; ||Humanitarian Clinic, Andheri (W), Mumbai, India

© 2012 by the American Society for Dermatologic Surgery, Inc. • Published by Wiley Periodicals, Inc. •
ISSN: 1076-0512 • Dermatol Surg 2012;38:1981–1990 • DOI: 10.1111/dsu.12000

Limitations of this method include donor site morbidity and inability to cover extensive areas.

Autologous epidermal cell transplantation can be another option to treat patients with stable vitiligo.[5–8] The cell suspension includes melanocytes and other skin cells. Transplantation with noncultured epidermal cell suspension gives coverage of only 3 to 4 times the biopsy specimen size, and the exact number of melanocytes in the suspension is not known. In vitro cultured melanocytes have been used for the surgical treatment of larger areas of vitiligo, wherein a pure population of melanocytes is delivered to the vitiliginous site. By using cultured melanocytes from a small biopsy specimen, a larger number of cells can be obtained, enabling coverage of large affected areas. The efficacy of cultured melanocytes to induce pigmentation has been demonstrated in patients with vitiligo.[9–11] Disadvantages of cell suspension delivery include loss of cells in the dressing and lack of retention of cells in difficult-to-apply areas such as elbow and knee.

In an attempt to overcome some of the shortcomings of cell suspension delivery, Redondo and colleagues[12] delivered cultured melanocytes on an amniotic membrane. A similar methodology was used to induce pigmentation by culturing epidermal cells on a substrate and placed on the dermabraded vitiliginous areas.[13,14] The use of polymers on which the cells are cultured and transferred would facilitate easy application of the graft. Advantages of using a transparent polymer film include microscopic observation of cells during processing and visualization of the underlying wound after its application on the débrided site.

We had earlier reported an effective system by which we can collect and transport skin biopsies under viable conditions within 96 hours from various tertiary hospitals to a central processing center, culture melanocytes, and deliver the cells on poly (DL-lactic acid) (PLA) film back to the patient.[15] We chose PLA for its biochemical and biocompatible properties. We have developed a cultured graft consisting of autologous melanocytes cultured on a PLA film for the treatment of stable vitiligo.

A trial was conducted to establish the safety and efficacy of the cultured graft. The primary objective was to compare the percentage of treatment sites repigmented after application of the cultured graft with that of a control group. The secondary objectives were to study the safety and the repigmentation pattern and recurrence of vitiliginous patches at the recipient sites.

Patients and Methods

Study Design

A prospective, open-label, randomized, multicenter clinical trial was conducted to establish the safety and efficacy of the cultured graft when applied to patients with stable segmental vitiligo. The study was conducted in compliance with the ethical principles that originate in the Declaration of Helsinki and the International Conference on Harmonization guidelines for Guidelines for Good Clinical Practice, Drug Controller General of India and Schedule Y regulations. The study was registered on Clinical trials registry-India (CTRI/2009/091/000638).

Participants

Twenty two patients aged 14 to 50 with stable vitiligo were enrolled for the trial at four centers. Stable vitiligo was defined as no new lesions or expansion of existing lesions for at least 2 years before enrollment into the trial. The patients enrolled had not responded earlier to routine therapy. The patients had a minimum of two achromic maculae of similar size at least 10 cm apart with a lesion size between 2 and 12 cm^2.

Patients with Koebner response in the past; scarring or keloidal tendencies; infection at the recipient site; or infection with human immunodeficiency virus or hepatitis B or C were excluded from the study. Patients at each study center were enrolled after obtaining the approval from the respective institutional ethics committee and written informed consent from the subjects.

Study Setting

The study was conducted in four centers in India. Data were collected from the participating centers, two of which were government-run hospitals and two private clinics. On each patient, control and test recipient areas were allocated based on the randomization schedule that the clinical research group provided using the fixed, permuted block randomization method.

Donor Site

The selected donor site (inner thigh or buttocks area) had normal pigmentation and was free from infection. The site was cleaned with 70% ethanol and washed thoroughly with normal saline. The site was anesthetized by infiltrating 1% lidocaine in the subcutis. After the donor area was anesthetized, two punch biopsies 6 mm in diameter were taken from the donor site and transferred into a transport vial containing biopsy collection medium and shipped to the central cell processing facility at Reliance Life Sciences.

PLA Film Preparation

We had earlier described the preparation of PLA films.[15] PLA granules (Reliance Life Sciences, India) were dissolved in acetone (E. Merck, Mumbai, MH, India) to make a 20% solution. This solution was then cast on stainless steel plates by spin coating and left overnight at 37°C to dry. After drying, the films were carefully peeled off the plates and cut to size. For this study, the films were cut to circles 4.5 cm in diameter and sterilized using ethylene oxide (Microtrol Sterilization Services, India).

Cell Culture

Melanocyte isolation and expansion was performed as described earlier.[15] Briefly, the biopsies were decontaminated and incubated in dispase solution (Sigma, St. Louis, MO). The epidermal cells were released from the separated epidermis using enzymatic digestion with trypsin–ethylenediaminetetraacetic acid (Invitrogen, Carlsbad, CA) and suspended in melanocyte medium (254-CF) (Cascade Biologics, Portland, OR). Melanocytes were selectively passaged on reaching 70% to 80% confluence.

Cultured Graft Preparation

Cultured grafts were prepared by seeding the melanocytes at 1 to 2 $\times$ 10^4 cells/cm^2 on PLA films (16 cm^2) and cultured for 3 to 4 days in a specially designed container (Figures 1 and 2).

Recipient Site

The recipient sites were prepared and anesthetized in the same manner as the donor site. After the area was marked, epidermal ablation was performed using a high-speed motor dermabrader.

Grafting

After preparation of the recipient site, the cultured graft was placed on the dermabraded area so that the cells were in apposition to the wound bed. The

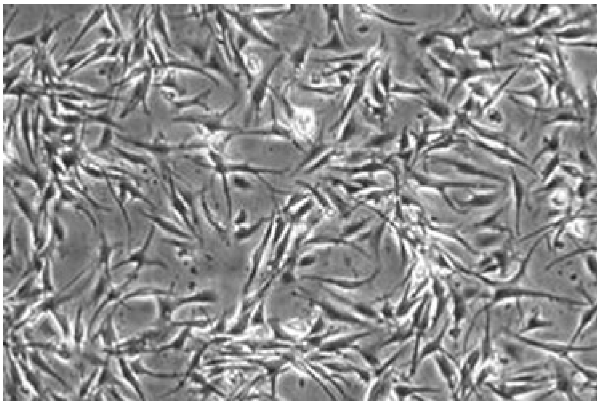

Figure 1. Melanocytes cultured on poly (DL-lactic acid) film (magnification × 4).

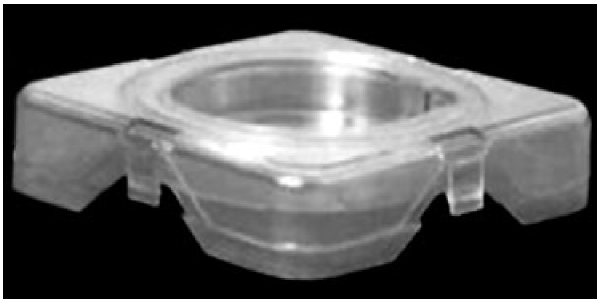

Figure 2. Shipping container used for transporting the cultured graft.

graft was held in place with a polyurethane film dressing. The control site was similarly dermabraded and covered with a similar polyurethane film dressing. After 7 to 8 days, subjects were advised to expose the control and treated areas daily to sunlight for 15 to 20 minutes until signs of pigmentation were seen. Postgrafting evaluations were performed on days 7, 15, 90, 180, and 270.

Efficacy Measurements

The primary objective was to compare the percentage of treatment sites showing repigmentation after application of the cultured graft with that of the control group. Efficacy was graded as successful when 70% or more of the treated area showed repigmentation at the end of the study.

Photographs of test and control sites were taken on day 0 (before dermabrasion and before application of the test device) and at each subsequent visit. The extent of re-pigmentation of the test and control sites was clinically and photographically evaluated using NIH-J image software.

Safety Measurement

At each study visit, the investigators assessed the study lesions for wound breakdown. Clinical laboratory tests including hematology (hemoglobin, total and differential leucocyte count, and platelet count), biochemistry (aspartate aminotransferase, alanine aminotransferase, serum bilirubin, alkaline phosphatase, serum creatinine, and blood sugar), urinalysis, and serology (human immunodeficiency virus, hepatitis B and C virus, and Venereal Disease Research Laboratory test) were done at baseline and end of study. Vital signs such as temperature, blood pressure, and pulse and weight were assessed at screening, before treatment, and at each subsequent study visit.

Statistical Methods

The differences in the proportion of repigmentation between groups were statistically evaluated using the Fisher exact two-sided test. A p-value $\leq .05$ was considered significant.

Results

In two of the 22 patients enrolled, the melanocytes could not be expanded in culture, and in one patient, the cultured graft was not implanted because the subject was found to have vitiliginous patches on more than 30% of the total body surface area (exclusion criteria). These three patients were not evaluated. Two patients who were lost to follow-up were not analyzed (Figure 3). The mean age of the patients evaluated in the study was 28.7 (range 14–50) (Table 1). The study, which was initiated in February 2008, enrolled 15 (78.9%) male and four

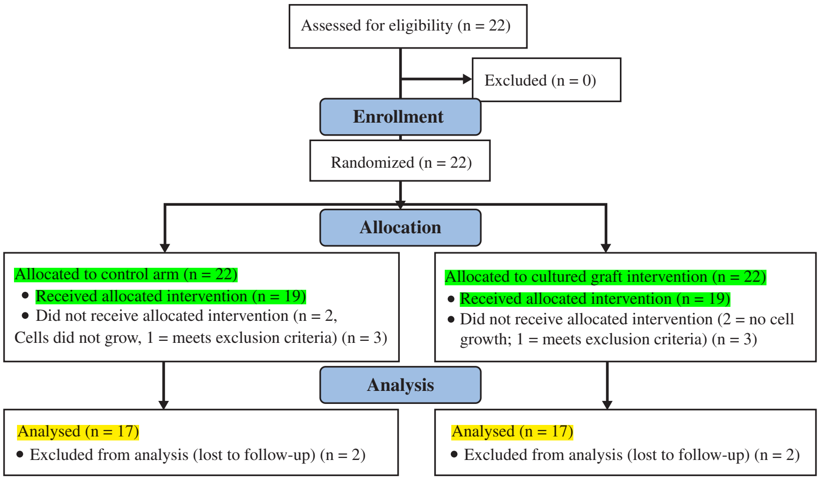

Figure 3. Study flow chart of patients recruited to the study.

TABLE 1. Demography of Evaluated Patients

Patient Number	*Age*	*Sex*	*Location of Test Site*	*Size of Test Site, cm*	*Location of Control Site*	*Size of Control Site, cm*
7101	45	Female	Right leg	2 × 2	Left leg	2 × 2
7102	23	Female	Right foot	2.5 × 2	Left foot	2 × 2
7103	19	Female	Right foot	2.5 × 2	Left foot	2 × 2
7104	15	Male	Abdomen	3 × 2	Abdomen	2 × 2
7201	29	Female	Legs	2 × 2	Legs	2 × 2
7202	19	Female	Patient withdrawn before IP application			
7203	42	Female	Ankles	2 × 2	Ankles	2 × 2
7301	20	Male	Right loin	2 × 2	Left loin	2 × 2
7303	50	Female	Left leg, below knee, over shin	3 × 6	Right leg, below knee, over shin	3 × 7
7304	19	Female	Left forearm	3 × 7	Right forearm	2 × 10
7305	26	Female	Left leg, below knee, over shin, top	2 × 3	Left leg, below knee, over shin, bottom	2 × 4
7306	27	Male	Left leg, below knee, over shin	3 × 2	Right leg, below knee, over shin	4 × 2
7307	24	Female	Left foot below medial malleolus	3 × 4	Right foot below medial malleolus	1 × 3
7308	23	Female	Left leg, below knee, over shin	2 × 2	Right leg, below knee, over shin	2 × 2
7401	48	Female	Left leg	4 × 2	Left thigh	4 × 2
7402	46	Female	Left elbow	4 × 5	Right elbow	5 × 4
7403	18	Female	Right leg on lateral aspect above lateral malleolus	2.5 × 3	Right knee	2.5 × 3
7404	18	Female	Right side of abdomen	4 × 3	Left side of abdomen	3 × 4
7405	14	Female	Left side of abdomen	5 × 5	Right side of abdomen	5 × 9
7406	39	Male	Screen failure			
7407	39	Male	Right leg below knee top half	5 × 6	Right leg below knee bottom half	3 × 6

IP, investigational product.

(21.1%) female Indian subjects who were followed up for 9 months after treatment. Test and control vitiliginous sites were identified on the same patient at two different locations.

The efficacy of the study is shown in Table 2, and representative pictures of response to the cultured graft and the control are shown in Figures 4I and 4II, respectively. A significant difference in the extent of repigmentation between treated and control lesions was observed 90 (p = .17), 180 (p = .006), and 270 days (p = .002) after implantation. Successful repigmentation in the sites treated with cultured graft was observed in 29.4% (95% confidence interval (CI) =0.07–0.48%) patients at day 90, 52.9% (95% CI = 0.27–0.73%) at day 180, and 58.8% (95% CI = 0.33, 0.79%) at day 270. In the control sites, success was noted in 5.8% (95% CI=–0.05 to 0.16%) of patients throughout the study.

Repigmentation Pattern

The pattern of repigmentation was analyzed in the patients who had successfully responded to treatment with the cultured graft. The pattern of repigmentation in the nine patients who had 70% or more repig-

TABLE 2. Repigmentation over Time

Subject Number	Test (%) Day 90	Test (%) Day 180	Test (%) Day 270	Control (%) Day 90	Control (%) Day 180	Control (%) Day 270
7101	100	90	80	0	0	0
7102	0	0	0	0	0	0
7103	100	100	100	0	0	0
7104	35	85	85	0	0	0
7201	60	85	90	40	70	80
7203	*	*	82	*	*	0
7301	100	100	100	30	30	30
7303	90	95	100	85	85	40
7304	0	0	0	0	0	0
7305	5	0	*	0	0	*
7307	55	58	60	20	23	25
7306	*	*	*	*	*	*
7308	75	95	95	25	25	25
7401	30	26	35	0	0	0
7402	40	50	52	45	50	50
7403	10	10	10	10	10	10
7404	5	80	85	10	15	18
7405	60	75	75	40	40	40
7407	50	30	30	5	5	5

*Patient absent for follow-up.

mentation at days 90 and 180 was diffuse in eight and combined in one, whereas at day 270, the pattern was diffuse in seven of the 10 successfully repigmented patients and combined in three.

Post-treatment Recurrence of Vitiliginous Patches

All patients treated with the cultured graft maintained pigmentation until day 270, whereas one patient in the control arm who had shown 85% repigmentation until day 180 had retained only 40% of the pigmentation at day 270 (Table 2).

Safety

Safety assessment of the cultured graft was based on physical examination, vital signs, laboratory tests, and any adverse events reported on the subjects enrolled in the study by the participating centers.

There were no significant changes in mean temperature, pulse rate, blood pressure, or hematology profile from screening to after application. Because the test and control sites were selected in the same patient, adverse events could not be classified in the test or control arm. A total of four adverse events were reported in three (15.8%) subjects; none of these events were related to the cultured graft application (Table 3).

Discussion

The aim of treating vitiligo using autologous melanocytes in the form of skin cell suspension or cultured melanocytes is not to cure the disease but to induce pigmentation by replacing nonfunctional or deficient melanocytes in the skin. All of the studies on melanocyte transplantation were conducted in hospitals with cell culture facilities. The establishment of a cell culture facility that meets regulatory norms is prohibitively expensive. The advantage of having a centralized cell culture facility could provide hospitals hitherto not equipped with cell culture facilities an opportunity

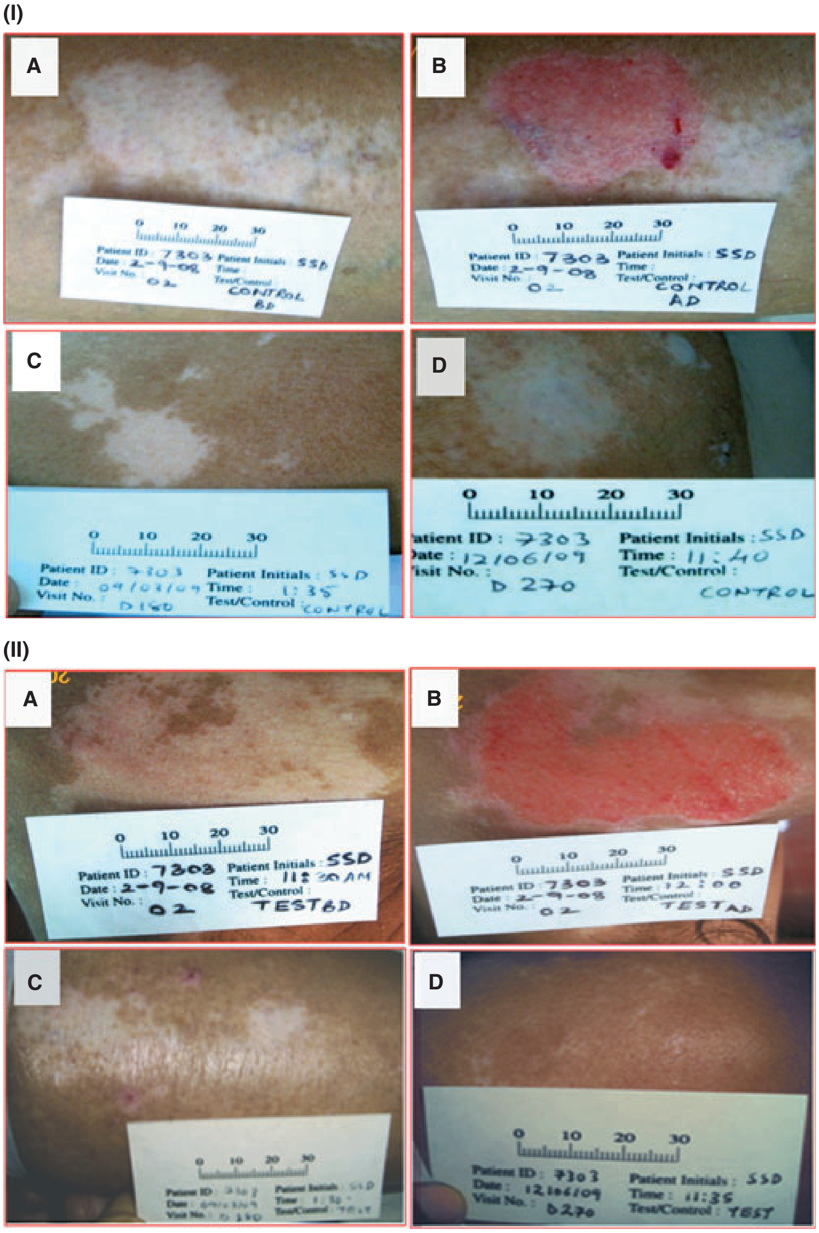

Figure 4. (I) Pigmentation pattern on control and (II) treated areas: (A) before dermabrasion, (B) after dermabrasion, day (C) 180 and (D) 270 after treatment.

to offer patients cultured melanocyte therapy at a reasonable cost.

Using culturing techniques, it was possible to isolate and propagate enough melanocytes from a 1-cm^2 biopsy specimen to cover a 500-cm^2 vitiliginous area using media that included tumor-promoting agents such as 12-O-tetradecanoyl phorbol-13-acetate, which are not recommended for transplantation.[16] Tegta and colleagues,[17] using noncultured epidermal cell suspension, had suggested that the minimum number of melanocytes required to produce satisfactory repigmentation is probably in the range of 210 to 250 cells/mm^2. Using our culture conditions, which do not include any tumor-promoting agents, we were able to obtain a 64-cm^2 graft seeded with

TABLE 3. Summary of Adverse Events

Patient Number	Adverse Event	Start Date	End Date	Serious Adverse Event	Severity	Relationship to Interventional Product	Treatment Required	Outcome
7201	Pyrexia	April 15, 2008	April 16, 2008	No	Mild	Unrelated	Medication	Recovered
	Nasopharyngitis	April, 15, 2008	April 16, 2008	No	Mild	Unrelated	Medication	Recovered
7203	Foot fracture	March 28, 2009	May 9, 2009	Yes	Moderate	Unrelated	Hospitalization	Recovered
7308	Heat rash	July 17, 2009	July 23, 2009	No	Mild	Unrelated	Medication	Recovered

250 to 300 melanocytes/mm^2 from a 1-cm^2 skin biopsy in 4 to 6 weeks. With our culture conditions, it is feasible to prepare large grafts that can cover extensive vitiliginous lesions from small skin biopsies.

Although there are no uniform and acceptable criteria for the evaluation of the outcome after melanocyte transplantation, it is difficult to compare the results of different studies using different transplantation methods. The success rates of treating stable vitiliginous lesions with melanocytes suspension vary between 22% and 72%.[18]

During dermabrasion, amelanotic (inactive) melanocytes present at the outer root sheath of the hair follicle are activated, leading to proliferation and migration of melanocytes.[19] In this study, test sites were treated with melanocytes cultured on PLA films and covered with polyurethane film dressing. The control sites should appropriately have been covered with PLA films without melanocytes, but abiding by the regulatory norms that recommend the use of a standard treatment in the control group, we used polyurethane films on the control sites. Dermabrasion, which was used as an active comparator, showed successful repigmentation in only 5.8% of patients, whereas repigmentation was achieved in 58.8% of patients in the cultured graft–treated sites. No recurrence of vitiliginous spots was seen in the test arm. Only one serious adverse event of foot fracture was reported which healed and was not related to the application of the cultured graft. The age and sex of the patient and the size and location of the lesion could not be correlated with the study outcome in the small population studied.

Selection of patients is critical for the outcome of the study, because active disease might negatively influence the results. It was difficult to verify whether the vitiliginous lesions had been stable for at least 2 years before enrollment because most subjects were not under the care of the participating clinicians.

It has been reported that optimal take of the melanocytes occurs when melanocytes are presented at the dermo-epidermal junction.[20] Kaufmann and colleagues reported successful repigmentation using an erbium-doped yttrium aluminum garnet laser, which is optimal for depth control when ablating the skin surface. In our study, the use of a dermabrader to cause epidermal ablation might have resulted in deeper dermabrasion, thus compromising the take of the cultured graft. This exploratory trial with a small subject population indicates the safety and efficacy of cultured grafts, but a better response to the cultured graft might be observed after the use of laser epidermal ablation in a larger trial involving more subjects with stable vitiligo.

Conclusion

After the reported success of treating vitiligo with cultured melanocytes, this treatment now provides an opportunity for patients with large areas of stable vitiligo, but setting up a facility for culturing melanocytes that meets regulatory requirements would be too expensive and not feasible for most centers.

We for the first time used a process by which we collected biopsies from various centers and transferred them to a centralized facility for processing and expanding the cells. The expanded melanocytes were cultured on a PLA film and delivered to the centers for implantation. This process provides an opportunity to all clinicians interested in offering this treatment option to their patients.

Using our established system, the study demonstrated that the cultured graft was safe and could be effectively used to treat stable vitiliginous patches.

Acknowledgments We would like to thank the members of Reliance Clinical Research Services for conducting this study. We gratefully acknowledge the encouragement and support of Reliance Life Sciences Pvt Ltd in carrying out the research work.

References

1. Mulekar SV. Melanocyte-keratinocyte cell transplantation for stable vitiligo. Int J Dermatol 2003;42:132–6.
2. Kahn AM, Cohen MJ. Repigmentation in vitiligo patients: melanocyte transfer via ultra-thin grafts. Dermatol Surg 1998;24:365–7.
3. Agrawal K, Agrawal A. Vitiligo: repigmentation with dermabrasion and thin split-thickness skin graft. Dermatol Surg 1995;21:295–300.
4. Hann SK, Im S, Bong HW, Park YK. Treatment of stable vitiligo with autologous epidermal grafting and PUVA. J Am Acad Dermatol 1995;32:943–8.
5. Mulekar SV. Long term follow-up study of segmental and focal vitiligo treated by autologous non-cultured melanocyte-keratinocyte cell transplantation. Arch Dermatol 2004;140: 1211–5.
6. Olsson MJ, Juhlin L. Leucoderma treated by transplantation of a basal cell layer enriched suspension. Br J Dermatol 1998;138: 644–8.
7. van Geel N, Ongenae K, De Mil M, Haeghen YV, et al. Double-blind placebo-controlled study of autologous transplanted epidermal cell suspensions for repigmenting vitiligo. Arch Dermatol 2004;140:1203–8.
8. Mulekar SV. Long-term follow-up study of 142 patients with vitiligo vulgaris treated by autologous, non-cultured melanocyte-keratinocyte cell transplantation. Int J Dermatol 2005;44:841–5.
9. Chen YF, Yang PY, Hu DN, Kuo FS, et al. Treatment of vitiligo by transplantation of cultured pure melanocytes suspension: analysis of 120 cases. J Am Acad Dermatol 2004;51:68–74.
10. Olsson MJ, Juhlin L. Long-term follow-up of leucoderma patients treated with transplants of autologous cultured melanocytes, ultrathin epidermal sheets and basal cell layer suspension. Br J Dermatol 2002;147:893–904.
11. Hong W, Hu D, Qian G, McCormick S, et al. Treatment of vitiligo in children and adolescents by autologous cultured pure melanocytes transplantation with comparison of efficacy to results in adults. J Eur Acad Dermatol Venereol 2011;25:538–43.
12. Redondo P, del Olmo J, García-Guzman M, Guembe L, et al. Repigmentation of vitiligo by transplantation of autologous melanocyte cells cultured on amniotic membrane. Br J Dermatol 2008;158:1168–71.
13. Arenberger P, Broz L, Veselý P, Havlícková B, et al. Tissue engineered skin in the treatment of vitiligo Lesions. Folia Biol (Praha) 2000;46:157–60.
14. Brysk MM, Newton RC, Rajaraman S, Plott T, et al. Repigmentation of vitiliginous skin by cultured cells. Pigment Cell Res 1989;2:202–7.

15. Ghosh D, Shenoy S, Kuchroo P. Cultured melanocytes: from skin biopsy to transplantation. Cell Transplant 2008;17:351–60.

16. Yaar M, Gilchrest BA. Vitiligo: the evolution of cultured epidermal autografts and other surgical treatment modalities. Arch Dermatol 2001;137:348–9.

17. Tegta GR, Parsad D, Majumdar S, Kumar B. Efficacy of autologous transplantation of noncultured epidermal suspension in two different dilutions in the treatment of vitiligo. Int J Dermatol 2006;45:106–10.

18. Borderé AC, Lambert J, van Geel N. Current and emerging therapy for the management of Vitiligo. Clin Cosmet Investig Dermatol 2009;2:15–25.

19. Mohammad NS, Elgoweini MF, Khadr NA. Dermatomal Vitiligo: therapeutic implication of dermabrasion. Journal of Pan-Arab League of Dermatologists 2008;19:31–44.

20. Kaufmann R, Greiner D, Kippenberger S, Bernd A. Grafting of in vitro cultured melanocyte onto laser-ablated lesions in vitiligo. Acta Derm Venereol 1998;78:136–8.

Address correspondence and reprint requests to: Deepa Ghosh, PhD, Tissue Engineering Group, Regenerative Medicine, Reliance Life Sciences Pvt Ltd., Thane Belapur Road, Navi-Mumbai, 400701, India, or e-mail: deepa_ghosh@relbio.com
