# Supplementary material for: Therapeutic efficacy of cell-based therapy in vitiligo: a research letter systematically reviewed using meta-analysis
Source: Arch Dermatol Res. 2024 May 22;316(5):198. doi: 10.1007/s00403-024-02920-6 (PMC11111487; doi:10.1007/s00403-024-02920-6)
Supplement: Supplementary file 1 — Supplementary file1 (ZIP 24195 KB) [file 403_2024_2920_MOESM1_ESM.zip › Studies were included/RCT Gimenez-Azcarate 2013.pdf]

P6113

Could idiopathic vitiligo possibly be contact vitiligo?

Jamie Johnson, Indiana University School of Medicine, Indianapolis, IN, United States; Nico Mousdicas, MD, Indiana University School of Medicine, Indianapolis, IN, United States

Contact vitiligo is a process in which melanocyte destruction and depigmentation occur after exposure to certain chemicals. While phenol and catechol derivatives are most commonly implicated, many other causative substances have been documented. This condition is also known as "occupational vitiligo," but it is not a disease limited to occupational exposures. We report a case of contact vitiligo in a hospital janitor triggered by a phenol-containing cleaning solution. We also identify compounds known to induce this disease as well as the common materials, products and manufacturing processes in which they are found or used. Our aims are to increase awareness of this condition and to facilitate the process for clinicians in identifying causative chemicals. We propose a 5-step process to potentially assist clinicians and patients more efficiently and effectively identify and hence avoid future exposure to possible candidate chemical exposures currently known to cause contact vitiligo.

Commercial support: None identified.

1: 15/30
2: 29/30
3: 0/30

P6024

Cysteamine cream as a new skin depigmenting product

Christophe Hsu, MD, Center Medical du Lignon, Geneva, Switzerland; Hussain Ali Mahdi, MD, Royal Bahrain Hospital, Manama, Bahrain; Mohammad Pourahmadi, MD, Jahrom University Medical School, Jahrom, Iran; Sara Ahmadi, MD, Jahrom University Medical School, Jahrom, Iran

Background: Cysteamine hydrochloride (beta-mercaptoethylamine hydrochloride) has been known to be a potent depigmenting molecule for over 5 decades. Despite its strong depigmenting effect, shown to be more important than hydroquinone in vivo, cysteamine was never developed into a depigmenting product because of the very offensive odor it produced in topical preparations. Recently, a new technology has become available permitting to considerably reduce the odor of cysteamine in cream conditions. Cysteamine cream made available as such, was evaluated in our studies for its depigmenting activity.

Methods: Cysteamine cream was applied to one ear, and the vehicle alone to the contralateral ears of black female guinea pigs for 10 consecutive days. Dermatoscopic, chromametric, and histologic examinations (H&E, Fontana-Masson, HMB45) were performed at the end of the study. In the human study, 30 female patients with epidermal melasma were treated daily for 6 weeks by cysteamine cream. Before and after dermatoscopic images were taken and chromametric evaluations were performed at the beginning and at the end of the trial.

Results: In contrast to the vehicle, cysteamine cream showed a potent depigmenting effect in guinea pig skin. The depigmenting effect was confirmed by dermatoscopal and chromametric evaluations as well as the histologic studies. Cysteamine cream showed a considerable efficacy in the treatment of epidermal melasma confirmed by all evaluation methods. Previous cell culture studies confirmed that cysteamine acts through melanogenesis inhibition and not melanocytotoxicity to depigment cultured melanocytes.

Conclusion: Cysteamine is a nonmelanocytotoxic and nonmutagenic molecule. Cysteamine cream, made usable with the new technology for the first time, has a strong depigmenting action in vivo.

The study was funded by Scientis Pharma.

P6991

Double-blind, randomized, intraindividually controlled clinical trial to evaluate the efficacy of autologous melanocyte cell transplant using amniotic membrane as a scaffold in the treatment of stable vitiligo

Ana Giménez-Azcárate, MD, University Clinic of Navarra, Pamplona, Spain; Isabel Bernad, MD, University Clinic of Navarra, Pamplona, Spain; Isabel Irarrazaval, MD, University Clinic of Navarra, Pamplona, Spain; Miguel Lera, MD, University Clinic of Navarra, Pamplona, Spain; Pedro Redondo, PhD, MD, University Clinic of Navarra, Pamplona, Spain

Background: Cellular therapy for stable recalcitrant vitiligo is divided into 2 main categories: grafting of melanocyte-rich tissue and grafting of melanocyte cell suspensions.

Objectives: To compare the efficacy of autologous melanocyte seeded amniotic membrane versus melanocyte cell suspension in the treatment of stable vitiligo lesions. Efficacy is evaluated in terms of short- and long-term cosmetic results.

Methods: Thirty vitiligo patients with at least 1 year of stable lesions were included. A normopigmented cutaneous biopsy was taken by shaving on the lower back. Three different lesions of each patient were depithelized using carbon dioxide laser and then randomized to receive either melanocyte cells in suspension (group 1), melanocyte cells in amniotic membrane (group 2), or no melanocyte cells (placebo, group 3). Sun exposure was recommended in the next months to enhance repigmentation. Evaluation of the response was made 3 and 6 months posttreatment. Photographs of the lesions were taken before the treatment and repeated in the monitoring visits.

Results: A significant difference between cellular grafts and placebo was observed after 3 and 6 months ($P < .05$). In groups 1 and 2, repigmentation of at least 75% of the treated area was achieved in 51% and 95% of the actively treated lesions, whereas in group 3 repigmentation of at least 50% of the treated area was not observed at any time point.

Conclusion: Transplantation resulted in repigmentation of at least 75% of the treated area in most actively treated vitiligo lesions. Repigmentation was primarily caused by the transplanted melanocytes. Our results demonstrate that transplantation of autologous melanocytes cultured using amniotic membrane as a scaffold is superior to suspension melanocytes in the treament for stable vitiligo. In placebo-treated lesions, repigmentation induced by epidermal ablation and UV therapy is extremely limited.

Commercial support: None identified.

P6251

Psychological stressors preceding vitiligo onset are associated with itch and intermittent abdominal cramping

Jonathan Silverberg, MD, PhD, private practice, New York, NY, United States; Nanette Silverberg, MD, private practice, New York, NY, United States

Vitiligo is characterized by a loss of pigmentation caused by autoimmune destruction of melanocytes. Little is known about the impact of psychological stressors preceding vitiligo onset on the extent of and symptoms associated with vitiligo. We performed a questionnaire-based study of 1541 adults with vitiligo and found that 56.6% of subjects reported at least 1 death or stressor within 2 years before vitiligo onset, including death of a loved one (17.2%) and stressful life events (51.0%). Number of deaths or stressors was not associated with body surface area (BSA), laterality or distribution of lesions (logistic regression; $P \geq .11$). 522 subjects (34.5%) reported intermittent abdominal cramping, which was associated with BSA >75% (OR = 1.65; 95% CI = 1.17-2.32; $P = .004$). Multiple stressors were associated with intermittent abdominal cramping, including premenstrual and/or menstrual cramping (OR = 1.84; 95% CI = 1.15-2.95; $P = .01$), IBS (OR = 3.29, 95% CI = 1.34-8.05; $P = .01$) and autoimmune etiologies (OR = 4.02, 95% CI = 1.27-12.80; $P = .02$). 510 subjects (35.1%) reported any itching or burning from their vitiligo lesions, which was associated with BSA >25% (OR = 1.53, 95% CI = 1.23-1.90; $P < .0001$). One or multiple stressors was also associated with itching or burning of skin (one: OR = 1.43, 95% CI = 1.12-1.82; $P = .005$; multiple: OR = 1.51, 95% CI = 1.12-2.04; $P = .007$). In conclusion, there is a high prevalence of stressful life events preceding vitiligo, which may play an important role as triggers, as well as predict the presence of intermittent abdominal cramping, and itching and/or burning of skin. These associations indicate that screening of vitiligo patients for psychological stressors, abdominal cramping, itching and/or burning of skin should be included in the routine assessment of vitiligo patients.

Commercial support: None identified.
