# Supplementary material for: Therapeutic efficacy of cell-based therapy in vitiligo: a research letter systematically reviewed using meta-analysis
Source: Arch Dermatol Res. 2024 May 22;316(5):198. doi: 10.1007/s00403-024-02920-6 (PMC11111487; doi:10.1007/s00403-024-02920-6)
Supplement: Supplementary file 1 — Supplementary file1 (ZIP 24195 KB) [file 403_2024_2920_MOESM1_ESM.zip › Studies were included/RCT Komen 2015.pdf]

Mekokishvili, Hoda Moneib, Nir Nathanshon, Boris Nedelciuc, Kari Nielsen, Judith Olah, Margaret Oliviero, Fezal Özdemir, John Paoli, Giovanni Pellacani, Ketty Peris, Ana Maria Perusquia, Domenico Piccolo, Maria Antonietta Pizzichetta, Susana Puig, Babar Rao, Ahmed Sadek, Mustafa Sahin, Thomas Roger Schopf, Alon Scope, Peter Soyer, Ignazio Stanganelli, William Stoecker, Wilhelm Stolz, Alex Stratigos, Pietro Rubegni, Lidia Rudnicka, Masaru Tanaka, Danica Tiodorovic-Zivkovic, Karin Terstappen, Luc Thomas, Antonella Tosti, Sergeev Vasily, Ingrid Wolf, Pedro Zaballos, and Iris Zalaudek.

Funding sources: None.

Conflicts of interest: None declared.

Correspondence to: Giuseppe Argenziano, MD, Dermatology Unit, Second University of Naples, Via Pansini 5 - 80131 Naples, Italy

E-mail: g.argenziano@gmail.com

REFERENCES

1. Kittler H, Guitera P, Riedl E, et al. Identification of clinically featureless incipient melanoma using sequential dermoscopy imaging. *Arch Dermatol*. 2006;142:1113-1119.
2. Salerni G, Terán T, Puig S, et al. Meta-analysis of digital dermoscopy follow-up of melanocytic skin lesions: a study on behalf of the International Dermoscopy Society. *J Eur Acad Dermatol Venereol*. 2013;27:805-814.
3. Menzies SW, Gutenev A, Avramidis M, Batrac A, McCarthy WH. Short- term digital surface microscopic monitoring of atypical or changing melanocytic lesions. *Arch Dermatol*. 2001;137:1583-1589.
4. Salerni G, Carrera C, Lovatto L, et al. Benefits of total body photography and digital dermatoscopy ("two-step method of digital follow-up") in the early diagnosis of melanoma in patients at high risk for melanoma. *J Am Acad Dermatol*. 2012;67:e17-e27.
5. Argenziano G, Mordente I, Ferrara G, et al. Dermoscopic monitoring of melanocytic skin lesions: clinical outcome and patient compliance vary according to follow-up protocols. *Br J Dermatol*. 2008;159:331-336.

http://dx.doi.org/10.1016/j.jaad.2015.03.033

Autologous cell suspension transplantation using a cell extraction device in segmental vitiligo and piebaldism patients: A randomized controlled pilot study

See related article on page 76

To the Editor: Stable vitiligo and piebaldism can be repigmented by autologous cell suspension transplantation (CST).[1] Previously, specialized laboratories were necessary for preparation of cell suspensions. A cell extraction device (CED, ReCell, Avita Medical, Cambridge, UK) obviates this need.[2] However, little reliable data on this technique are available. We performed a single-center, randomized, observer blinded, intrapatient controlled pilot study on the repigmentation ability, tolerability, patient satisfaction, and cellular suspension composition of the CST-CED method. Three depigmented lesions of 9 cm^2 were randomly allocated to receive the following treatments (Fig 1): (1) CO_2 laser ablation plus autologous cell suspension (CST-CED); (2) CO_2 laser ablation (CO_2 control); (3) no treatment (no-treatment control). A split-thickness skin biopsy of approximately 4 cm^2 and 0.2 mm thickness was harvested from the hip using an electric dermatome (D42, Humeca, Beverwijk, Netherlands). The cell suspensions were produced using the CED.[2] First, the split skin was placed in the battery heated well of the device containing trypsin enzyme solution. After 15 minutes, epidermal cells could be disaggregated from the dermis with a scalpel. The cells were rinsed in the second well of the device with buffered sodium lactate solution. The suspension was applied onto the sites in a 1:5 expansion ratio. Laser treatments were performed with a 10,600 nm CO_2 laser (Ultrapulse, Lumenis ActiveFx hand piece, Santa Clara, CA) with 1 pass of 200 mJ (estimated depth 209 μm), 60 W, density 3 (full coverage). Four weeks after treatment, UVA-treatment (924T Eurosolar Facial Tanner, Beusichem, Netherlands) was administered to the treatment and control sites.[3] The primary outcome of our study was the percentage of repigmentation 6 months after study intervention using a digital image analysis system.[4] An investigator blinded to treatment allocation assessed side effects. Patients reported satisfaction. Cell counts and viability were assessed using a microscope (Leica DM2000, Leica Microsystems, Eindhoven, Netherlands) using trypan blue solution (Sigma-Aldrich Chemie, Zwijndrecht, Netherlands), and flow cytometry (FACS Canto II, Becton Dickinson, Breda, Netherlands), with Flow Jo software (Tristar, Ashland, OR).

Five patients with stable segmental vitiligo and 5 patients with piebaldism were included. The median repigmentation in 10 patients (mean age 34, 60% male) was 78%, 0%, and 0% for the CST-CED, the CO_2 control, and the no-treatment control sites, respectively (P = .001, Friedman test, Fig 2). Sixty percent of the CST-CED sites showed greater than 75% repigmentation. Repigmentation was assessed as good or excellent by 70% of the patients in the CST-CED sites. No long-term side effects were seen in the recipient sites. Two donor sites showed mild textural change. Fig 3 shows a positive correlation between the percentage of repigmentation and the total number of all viable cells transplanted

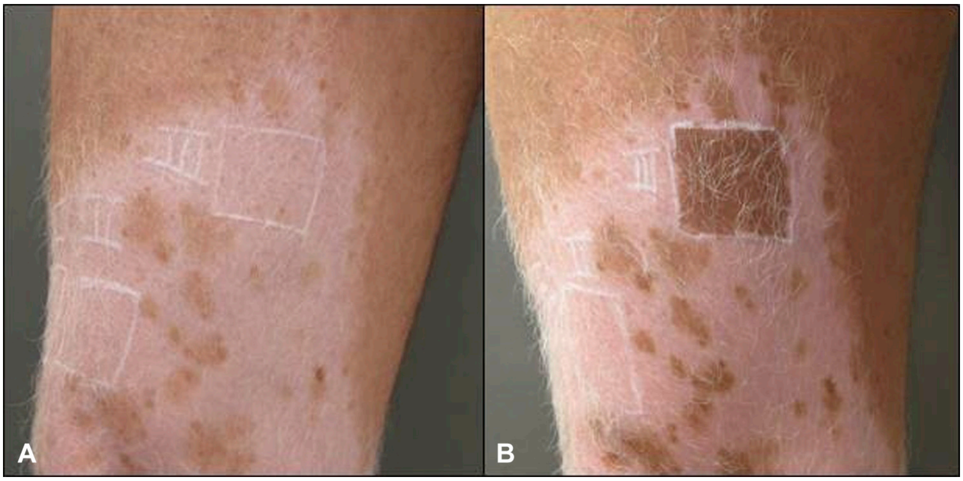

Fig 1. Autologous cell suspension transplantation for vitiligo and piebaldism. The figure shows 2 recipients sites before (**A**) and 6 months after treatment (**B**). Site III was treated with the CST-CED treatment and showed 100% repigmentation and moderate hyperpigmentation. Site II was the control site showing 0% repigmentation.

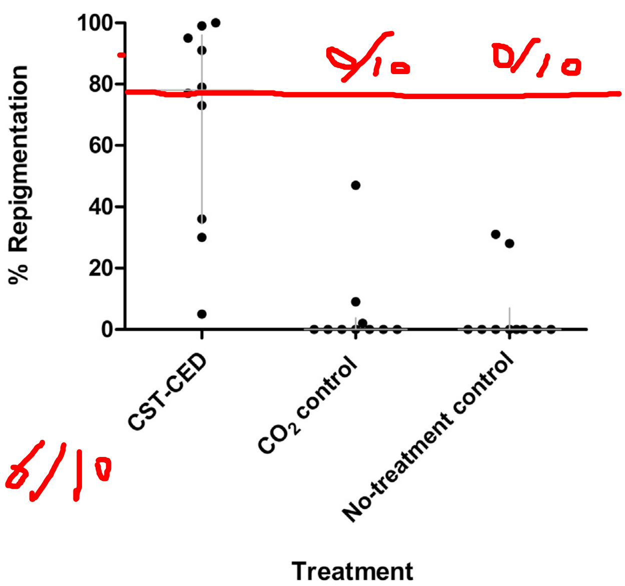

Fig 2. Autologous cell suspension transplantation for vitiligo and piebaldism. The figure shows the repigmentation percentages from CST-CED, the CO_2 control, and no-treatment control treatment. The CST-CED treated areas show a median repigmentation of 78% with a minimum to maximum range of 5% to 100%. The CO_2 control and no-treatment control sites show a median repigmentation rate of 0% (minimum to maximum range 0-47%) and 0% (0-31%), respectively. The horizontal and vertical gray lines show the medians with interquartile ranges.

(rho = 0.71) and the percentage of viable melanocytes transplanted (rho = 0.81), confirming the importance of viable melanocytes for the extent of repigmentation. The importance of other cell types remains unclear. The same CST-CED was used previously by Cervelli and colleagues[5] and also by Mulekar and colleagues,[2] who reported in an uncontrolled and small study greater than 75% repigmentation in 80% and 40% of the treated patients, respectively. Studies with laboratory-based CST reported greater than 75% repigmentation in approximately 57% to 100% of the stable patients.[1,6] Future research should focus on mechanisms underlying variation in cell counts, correlation with the repigmentation results, and optimal donor/recipient expansion ratio.

We are indebted to Avita Medical (Cambridge, UK) for donating the ReCell kits and facial tanners used for UVA treatment during the study.

Lisa Komen, MD,[a] Charlotte Vrijman, MD,[a] Esther P. M. Tjin, PhD,[a] Gabrielle Krebbers,[a] Menno A. de Rie, MD, PhD,[a,b] Rosalie M. Luiten, PhD,[a] J. P. Wietze van der Veen, MD, PhD,[a,c] and Albert Wolkerstorfer, MD, PhD[a]

Netherlands Institute for Pigment Disorders (SNIP), Department of Dermatology, Academic Medical Centre,[a] and Department of Dermatology, VU Medical Centre,[b] Amsterdam; and Department of Dermatology, MC Haaglanden, Den Haag,[c] The Netherlands

Funding: ReCell kits were provided free of charge by Avita Medical.

Conflicts of interest: None declared.

Correspondence to: Lisa Komen, MD, Netherlands Institute for Pigment Disorders (SNIP), Department of Dermatology, Academic Medical Centre,

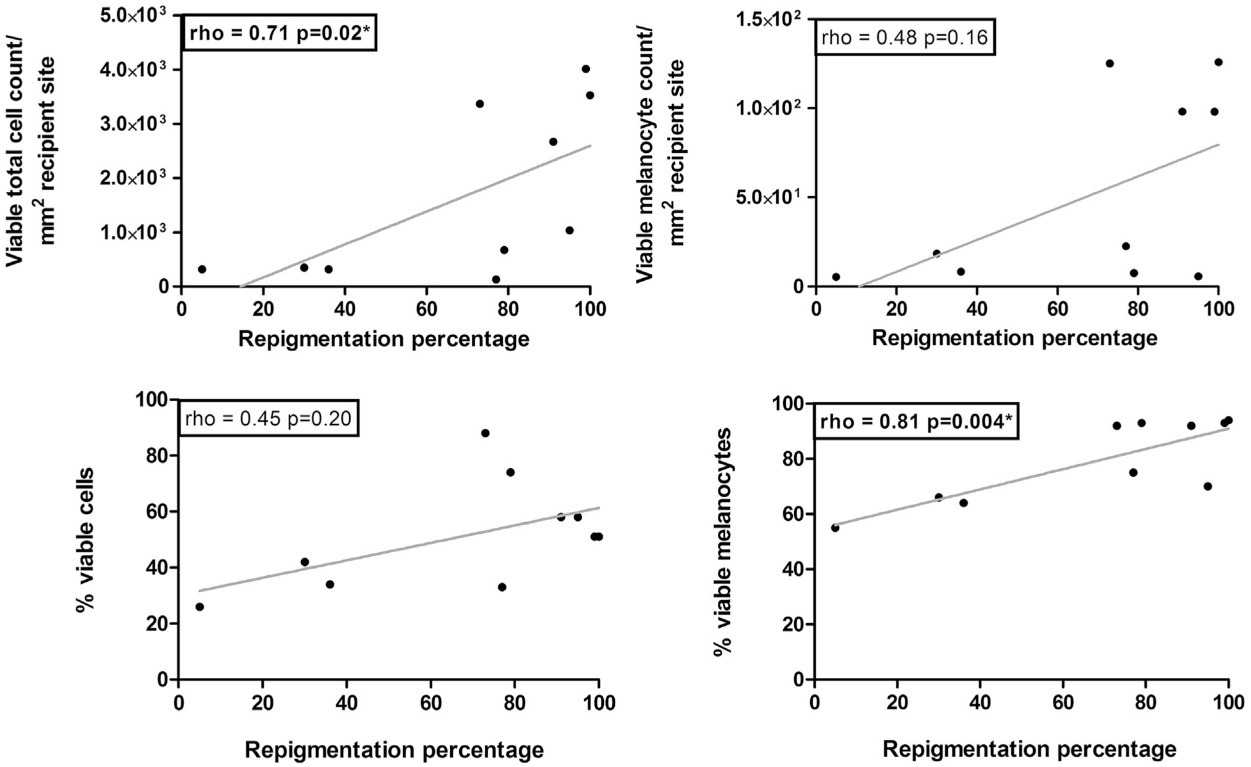

Fig 3. Autologous cell suspension transplantation for vitiligo and piebaldism. The upper graphs show the viable total cell count (all epidermal cells including melanocytes and keratinocytes, and fibroblasts) and viable melanocytes transplanted per square millimeter of recipient site in correlation with the repigmentation results. The 2 lower graphs show the correlation between the repigmentation results and the percentage of viable total cells and percentage of viable melanocytes in 1 mL superfluous suspension. The Spearman's rho test was used to calculate the correlation coefficient (rho). *Significant *P* values.

University of Amsterdam, P.O. box 22660, 1105 AZ Amsterdam, The Netherlands

E-mail: l.komen@amc.uva.nl

REFERENCES

1. van Geel N, Goh BK, Wallaeys E, De Keyser S, Lambert J. A review of non- cultured epidermal cellular grafting in vitiligo. *J Cutan Aesthet Surg*. 2011;4:17-22.
2. Mulekar SV, Ghwish B, Al Issa A, Al Eisa A. Treatment of vitiligo lesions by ReCell vs. conventional melanocyte-keratinocyte transplantation: a pilot study. *Br J Dermatol*. 2008;158:45-49.
3. Wind BS, Meesters AA, Kroon MW, et al. Punchgraft testing in vitiligo; effects of UVA, NB-UVB and 632.8 nm helium-neon laser on the outcome. *J Eur Acad Dermatol Venereol*. 2011;25:1236-1237.
4. Linthorst Homan MW, Wolkerstorfer A, Sprangers MA, van der Veen JP. Digital image analysis vs. clinical assessment to evaluate repigmentation after punch grafting in vitiligo. *J Eur Acad Dermatol Venereol*. 2013;27:e235-e238.
5. Cervelli V, De AB, Balzani A, Colicchia G, Spallone D, Grimaldi M. Treatment of stable vitiligo by ReCell system. *Acta Dermatovenerol Croat*. 2009;17:273-278.
6. Mulekar SV, Isedeh P. Surgical interventions for vitiligo: an evidence-based review. *Br J Dermatol*. 2013;169(Suppl 3):57-66.

http://dx.doi.org/10.1016/j.jaad.2015.03.036

The applicability and prognostic value of the TNM classification system for primary cutaneous lymphomas other than mycosis fungoides and Sézary syndrome in primary cutaneous NK/T-cell lymphoma patients

To the Editor: We previously reported the differences in survival outcomes and prognostic factors of cutaneous NK/T-cell lymphoma according to the primary tumor site.[1] The extent of cutaneous lesions was found to be associated with the prognosis of primary cutaneous NK/T-cell lymphoma (PCNKTL), except in nasal NK/T-cell lymphoma with cutaneous involvement.[1] A TNM classification system was developed in 2007 for primary cutaneous lymphomas other than mycosis fungoides and Sézary syndrome (Supplementary Table I; available at http://www.jaad.org).[2] The present study investigated the applicability and prognostic value of this TNM classification system for PCNKTL.

The current study included 28 patients with PCNKTL. The extent of skin lesions was evaluated using the proposed TNM classification system for
