# Supplementary material for: Therapeutic efficacy of cell-based therapy in vitiligo: a research letter systematically reviewed using meta-analysis
Source: Arch Dermatol Res. 2024 May 22;316(5):198. doi: 10.1007/s00403-024-02920-6 (PMC11111487; doi:10.1007/s00403-024-02920-6)
Supplement: Supplementary file 1 — Supplementary file1 (ZIP 24195 KB) [file 403_2024_2920_MOESM1_ESM.zip › Studies were included/RCT Singh 2013.pdf]

BJD
British Journal of Dermatology

Comparison between autologous noncultured extracted hair follicle outer root sheath cell suspension and autologous noncultured epidermal cell suspension in the treatment of stable vitiligo: a randomized study

C. Singh, D. Parsad, A.J. Kanwar, S. Dogra and R. Kumar

Department of Dermatology, Postgraduate Institute of Medical Education and Research, Chandigarh, 160012, India

Correspondence
Davinder Parsad.
E-mail: parsad@mac.com

Accepted for publication
13 March 2013

Funding sources
None.

Conflicts of interest
None declared.

DOI 10.1111/bjd.12325

Summary

Background Vitiligo is an acquired disorder of pigmentation caused by loss of epidermal melanocytes. Autologous noncultured epidermal cell suspension (NCES) and autologous noncultured extracted hair follicle outer root sheath cell suspension (NCORSHFS) are important surgical modalities for the treatment of stable vitiligo.

Objectives To compare NCES and NCORSHFS for producing repigmentation in stable vitiligo.

Methods We randomized 30 patients with 47 stable vitiligo lesions into two groups. Patients in group 1 were treated with NCES, and those in group 2 with NCORSHFS. They were evaluated 16 weeks postsurgery for the extent of repigmentation, colour match, change in Dermatology Life Quality Index (DLQI) score and patient satisfaction.

Results The extent of repigmentation was excellent (90–100% repigmentation) in 83% of lesions in the NCES group and 65% of lesions in the NCORSHFS group ($P = 0{\cdot}154$). Repigmentation $\geq$ 75% (good repigmentation) was observed in 92% of lesions in the NCES group and 78% of lesions in the NCORSHFS group ($P = 0{\cdot}425$). There was a significant improvement in DLQI score in both the groups, but the mean decrease among groups did not differ significantly ($P = 0{\cdot}244$). However, patients in the NCES group were significantly more satisfied than the patients in the NCORSHFS group. No significant difference was seen in colour match and pattern of repigmentation. Adverse effects were minimal.

Conclusions Both NCES and NCORSHFS are safe and effective techniques with comparable efficacy. To the best of our knowledge, this is the first study directly comparing two different cellular techniques.

What's already known about this topic?

- Noncultured epidermal cell suspension (NCES) is emerging as the first line of surgical management of stable vitiligo.
- Recently, noncultured extracted hair follicle outer root sheath cell suspension (NCORSHFS) has been shown to be effective in surgical treatment of vitiligo.
- As the hair follicle is an important reservoir of melanocytes and their precursor cells, this seems to be an important technique of replenishing melanocytes to depigmented patches.

What does this study add?

- This is the first randomized study to compare two cellular techniques of surgical management of vitiligo.

© 2013 The Authors
BJD © 2013 British Association of Dermatologists

- We have shown that both NCES and NCORSHFS are safe and effective techniques with comparable efficacy.

Vitiligo, commonly known as leucoderma, or phulwari in India, is an acquired disorder of pigmentation. Worldwide prevalence of vitiligo is around 0·5–1%.[1] Loss of cutaneous pigmentation occurs because of loss of melanocytes from the basal layer of the epidermis. The aetiopathogenesis of vitiligo is multifactorial and polygenic, including genetic, immunological, autoimmune and neurogenic factors, growth factor defects and environmental factors.[2] Vitiligo initially starts as hypopigmented macules and patches, which over a period of time become depigmented. It is often associated with leucotrichia. Patients with vitiligo experience psychosocial distress and social stigmatization due to widespread prejudices, taboos and ignorance among the general population.[3]

Various treatment modalities (both medical and surgical) are available. The majority of cases are managed using medical therapies; surgical methods are reserved for lesions not responding to medical treatment and that are stable in nature. The basic principal surgical method is the transfer of melanocytes from uninvolved skin to the stable vitiligo patch in the form of either a tissue graft or a cellular graft.

In cellular transplantation, cells are extracted from an unaffected skin or hair sample and transplanted as a suspension. It is assumed that cellular transplantation methods should be preferred when treating large areas. Cellular methods include transplantation of cultured pure melanocytes, cocultured melanocyte–keratinocyte cell suspension, cultured epidermis, noncultured epidermal cell suspension (NCES) or noncultured extracted hair follicle outer root sheath cell suspension (NCORSHFS). Each of these techniques has some advantages and some disadvantages, and attempts are ongoing to evolve a better technique.[4,5]

There is no comparative study in the literature between two different cellular transplantation techniques. Therefore, this randomized study was planned to compare the outcome of NCES and NCORSHFS transplantation in patients with stable vitiligo in terms of the extent of repigmentation, change in patients' psychosocial quality of life, colour matching and any adverse events.

Patients and methods

Patient selection

Subjects were recruited from the patients attending the pigmentary clinic of the Department of Dermatology, Venereology and Leprology, Postgraduate Institute of Medical Education and Research (PGIMER), Chandigarh, India between August 2011 and August 2012. Thirty patients with a clinical diagnosis of vitiligo that had been stable for 1 year and did not respond to medical therapy were recruited for the study. Patients aged < 10 years, those with actively spreading vitiligo, a history of koebnerization, hypertrophic and keloidal scars or a bleeding disorder, and pregnant patients were excluded.

The ethical committee of PGIMER approved the study, and written consent was obtained from every patient. The patients were randomly divided into two groups using a randomization table. Group 1 comprised 15 patients with 24 stable vitiligo lesions and group 2 comprised 15 patients with 23 stable vitiligo lesions. Group 1 was treated using autologous NCES and group 2 was treated with NCORSHFS.

At the first visit, a pro forma was filled in noting the baseline characteristics, history and examination findings. Informed consent was taken for the procedure. Patients were asked to complete the Dermatology Life Quality Index (DLQI) questionnaire, which was designed by Dr Finlay, and permission was received from him to use the questionnaire.

Technique of noncultured epidermal cell suspension grafting

An area of skin about one-tenth the size of the recipient area was selected as the donor site. The lateral side of the thigh was selected as the preferred donor site. The donor area was cleansed, shaved and anaesthetized with 2% lidocaine (AstraZeneca, Bangalore, India). A split-thickness skin graft was taken with the help of a shaving blade and straight artery forceps. The skin specimen was transferred under aseptic conditions to a container with normal saline and transferred to the laboratory. The graft was transferred to trypsin–ethylenediaminetetraacetic acid (EDTA) solution (0·25% trypsin and 0·02% EDTA) and incubated at 37 °C in 5% CO_2 for 1 h. Afterwards, the trypsin–EDTA solution was removed and phosphate-buffered saline (PBS) was added by pipette so as to separate the cells from the tissue.[5,6] The solid waste from the tissue was removed and the suspension was centrifuged at 78 ***g*** for 5 min. The supernatant was then discarded and the pellet was taken, containing cells from the stratum basale and the lower half of the stratum spinosum, which are rich in melanocytes. Around 1–3 mL of PBS according to recipient area was added to the pellet of melanocytes to make a suspension of noncultured epidermal cells. The recipient site was shaved, cleansed with Betadine (Win Medicare, New Delhi, India) and surgical spirit, and anaesthetized with 2% lidocaine. Dermabrasion was performed with the help of a manual dermabrader until punctate bleeding was seen. Dermabrasion was extended 5 mm beyond the margins to prevent halo phenomenon. The NCES was carefully transferred to the

© 2013 The Authors
BJD © 2013 British Association of Dermatologists

recipient site with the help of a tuberculin syringe and 18-gauge needle. A surgical dressing composed of Vaseline–chlorhexidine gauze, collagen (Eucare Pharmaceuticals, Chennai, India) and a sterile surgical pad was placed over the recipient site.

Technique of noncultured extracted hair follicle outer root sheath cell suspension

Hair samples were taken from the occipital area of the scalp. All subjects had undergone the follicular unit extraction (FUE) method for hair follicle tissue harvest. To obtain follicular units, a 1-mm punch was rotated to the mid-dermis in the direction of the hair follicle. Then the follicular unit was pulled out gently using hair-follicle-holding forceps by holding the skin surrounding the hair shaft(s). Depending on the area to be transplanted, approximately 15–25 pigmented follicles were extracted per subject and collected in normal saline. The extracted hair follicles were transferred under aseptic conditions to a container with normal saline and transferred to the laboratory and washed three times with PBS. The follicles were then incubated with 0·25% trypsin–0·05% EDTA at 37 °C for 90 min to prepare the single-cell suspension.[7] After every 30 min the hair follicles were placed in a new tube of trypsin–EDTA and the reaction in the previous tube was terminated by adding trypsin inhibitor (Sigma-Aldrich, St Louis, MO, U.S.A.). This was done to stop the proteolytic activity of trypsin in order to improve the attachment of grafted cells. After cell separation only thin keratinous shafts of the hair were left, which were discarded. The cell suspensions of all three tubes were combined in a single tube. Finally, the cell suspension was centrifuged for 5 min at 78 *g* to obtain a cell pellet, which was resuspended in a small amount of PBS and transported to the operating theatre for transplantation.[7] The transplantation procedure at the recipient site was the same as that for NCES.

The patients in both groups were asked to lie down for 1 h after the procedure and then allowed to go home. The dressing was removed after 7 days at the first follow-up visit at the hospital.

Follow-up

Patients in both groups were asked to attend for follow-up at the clinic on day 8, and weeks 4, 8, 12 and 16 after the transplantation procedure. We asked our patients to expose the area to sunlight at home starting from 5 min up to a maximum of 30 min daily.

Repigmentation was assessed subjectively by digital photography as follows: < 50%, poor repigmentation; 50–74%, fair repigmentation; 75–89%, good repigmentation; 90–100%, excellent repigmentation. Also, the repigmentation pattern was noted as 'diffuse', 'perifollicular' or 'dotted'. A note was also made of the colour matching of the repigmented skin, as 'somewhat lighter than', 'same as' or 'somewhat darker than' normal skin.

At each visit, patients were also asked about any adverse events and were asked to fill in a questionnaire about their satisfaction with the procedure results at weeks 4, 8 and 16, and also a DLQI questionnaire at week 16. Hence, both objective and subjective evaluations of the results were carried out.

Statistical methods and data analysis procedures

The statistical analysis was carried out using Statistical Package for Social Sciences (version 15.0 for Windows; SPSS Inc., Chicago, IL, U.S.A.). All quantitative variables were estimated using measures of central location (mean, median) and measures of dispersion (SD). Normality of data was checked by measures of Kolmogorov–Smirnov tests of normality. For normally distributed data, means were compared using Student's t-test for the outcome. For skewed data or scores the Mann–Whitney test was used. Qualitative or categorical variables were described as frequencies and proportions. Proportions were compared using the χ^2 or Fisher exact test, whichever was applicable. All statistical tests were two sided and performed at a significance level of $P = 0{\cdot}05$.

Results

All the patients completed the study period of 16 weeks and were included in the final analysis. The baseline characteristics of the patients in both groups are shown in Table 1. Patients in both groups were comparable. The size of the treated areas was 4–40 cm^2 in the NCES group and 4–48 cm^2 in the

Table 1 Characteristics and disease parameters of the patients in the two groups

	Group 1 (NCES)	Group 2 (NCORSHFS)	P-value
Number of patients	15	15	
Number of lesions	24	23	
Age range (years)	13–30	14–35	
Age (years)	20·67 ± 4·761	23·33 ± 4·894	0·142
Sex (female : male)	11 : 4	9 : 6	0·439
Family history, *n*	2	1	
Associated autoimmune diseases, *n*	1	2	
Duration of disease (years)	4·27 ± 1·387	5·13 ± 1·727	0·141
Duration of stability (years)	2·53 ± 2·167	2·00 ± 1·309	0·421
Type of vitiligo (G/F/S), *n*	8/2/5	7/2/6	0·865
Size of treated area (cm^2)	18·80 ± 7·803	16·20 ± 15·497	0·566

Data are mean ± SD unless otherwise stated. NCES, noncultured epidermal cell suspension; NCORSHFS, noncultured extracted hair follicle outer root sheath cell suspension; G, generalized; F, focal; S, segmental.

© 2013 The Authors
BJD © 2013 British Association of Dermatologists

NCORSHFS group. All patients had previously received various medical treatments for the management of their condition, including topical corticosteroids, topical calcineurin inhibitors, oral minipulse dexamethasone, psoralen plus ultraviolet (UV) A, psoralen with sunlight exposure, and narrowband UVB. These patients had either not responded to the medical modalities of treatment, or had achieved a partial response with a few recalcitrant lesions remaining resistant to therapy. Most patients did not report any postoperative discomfort. A few reported pain, which was easily relieved by oral analgesics. At the time of removal of the dressing, the treated area in both groups appeared bright pink and in some cases showed minimal erosion, which healed over a short period of time. The earliest repigmentation was noticed between 2 and 4 weeks postsurgery and the pattern of repigmentation was mostly diffuse.

Repigmentation at 16 weeks postsurgery

In the NCES group, excellent repigmentation (90–100%) was observed in 20/24 lesions (83%), while in the NCORSHFS group, excellent repigmentation was observed in 15/23 lesions (65%). This difference was not statistically significant ($P = 0{\cdot}154$). Repigmentation $\geq$ 75% (good repigmentation) at week 16 was achieved in 22/24 lesions (92%) in the NCES group compared with 18/23 lesions (78%) in the NCORSHFS group ($P = 0{\cdot}425$) (Figs 1–7). In 15/24 lesions in the NCES group (62%) and 15/23 lesions in the NCORSHFS group (65%), the colour of the repigmented area matched excellently with the normal surrounding skin. Six of 24 lesions in the NCES group and five of 23 lesions in the NCORSHFS group showed somewhat darker pigmentation, and three of 24 lesions in the NCES group and three of 23 lesions in the NCORSHFS group showed somewhat lighter pigmentation than normal skin, but with a tendency to match with normal

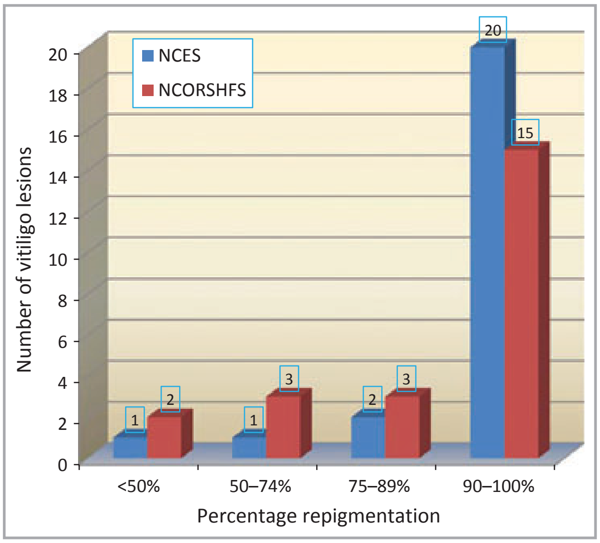

Fig 1. Repigmentation at 16 weeks postsurgery. NCES, noncultured epidermal cell suspension; NCORSHFS, noncultured extracted hair follicle outer root sheath cell suspension.

skin colour over time. The difference was not statistically significant ($P = 0{\cdot}966$). There was no significant effect of the age or sex of the patient, type of vitiligo, or site and size of the treated area on the extent of repigmentation.

Change in Dermatology Life Quality Index and patient satisfaction

Comparing the mean change in DLQI from before to 16 weeks after surgery, we observed a highly significant ($P < 0{\cdot}001$) improvement in terms of a reduction in DLQI score in both groups, and this was not significantly different between the two groups ($P = 0{\cdot}244$). In the NCES group the DLQI reduced from a mean value of 10·73 before surgery to 2·13 after surgery, while in the NCORSHFS group it reduced from a mean of 10·47 before surgery to 3·27 after surgery. Patients in both groups were satisfied with the surgical intervention of their recalcitrant vitiligo. They were ready to opt for this treatment in future if needed. The mean scores of the three patient satisfaction questionnaires in both groups are given in Table 2. Patients in the NCES group were significantly more satisfied than the patients in the NCORSHFS group.

None of our patients in either of the groups developed infection, visible scarring or milia at any site – donor or recipient.

Discussion

Several modalities are currently available for the treatment of vitiligo, but they generally do not result in complete cure of the disease. Despite the limitations and some side-effects, surgical modalities are indicated for all types of stable vitiligo, including segmental, generalized and acrofacial, that do not respond to medical therapy.

Melanocyte transplantation can be broadly classified into tissue grafts and cellular grafts. In tissue grafts intact pieces of uninvolved epidermis are used to transfer melanocytes. On the other hand, in cellular grafts cells are extracted from an unaffected skin or hair sample and transplanted as a suspension. Both NCES and NCORSHFS are simple and cheap methods of cellular grafts requiring minimal infrastructure.

The NCES method for the management of stable vitiligo was pioneered by Gauthier and Surleve-Bazeille in 1992.[8] Since then it has been used by a number of independent researchers with variable results. In the original method by Gauthier and Surleve-Bazeille, scalp was chosen as the donor site. However, it has been concluded that the transplantation of NCES is an efficacious and safe procedure. We observed excellent repigmentation (90–100%) in 83% of patients and good repigmentation (75–89%) in 8% of patients in our NCES group.

NCORSHFS is a novel surgical method of cellular transplantation for treatment of stable vitiligo and is still in the early phase of development.[7] The hair follicle is an important reservoir of melanocytes and their precursor cells.

© 2013 The Authors
BJD © 2013 British Association of Dermatologists

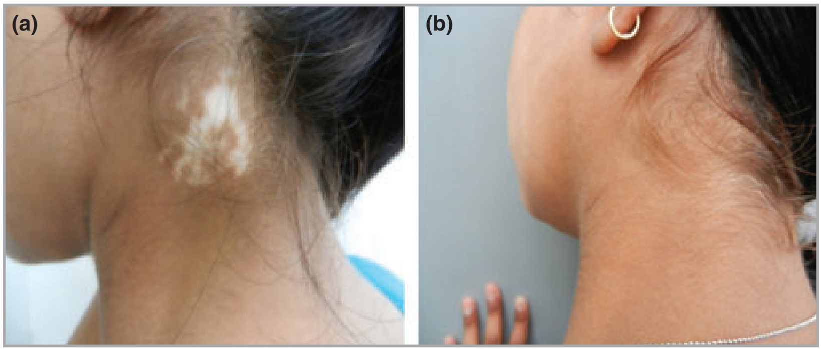

Fig 2. A patient in the noncultured epidermal cell suspension group with a lesion over the neck showing 100% repigmentation. (a) Before surgery; (b) 16 weeks after surgery.

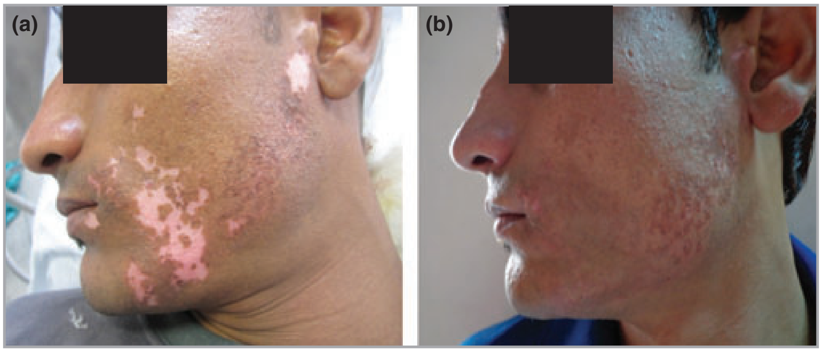

Fig 3. A patient in the noncultured extracted hair follicle outer root sheath cell suspension group with lesions of segmental vitiligo over the face showing near 100% repigmentation. (a) Before surgery; (b) 16 weeks after surgery.

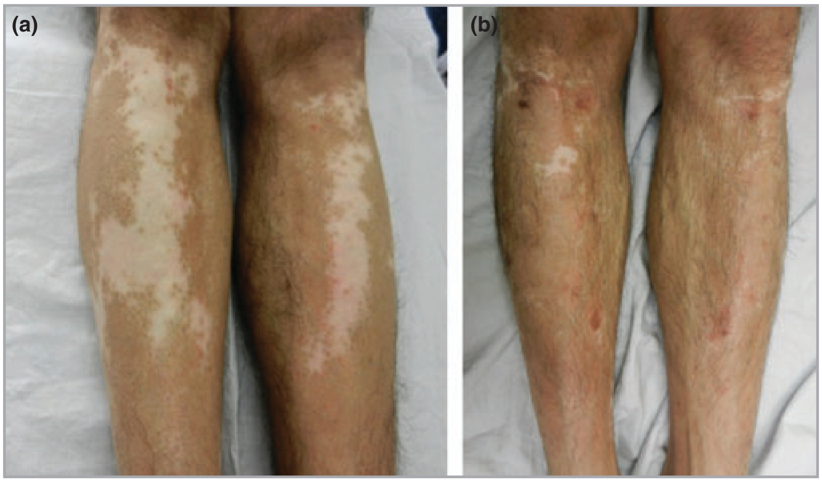

Fig 4. A patient in the noncultured extracted hair follicle outer root sheath cell suspension group with lesions of vitiligo vulgaris over the legs showing > 90% repigmentation. (a) Before surgery; (b) 16 weeks after surgery.

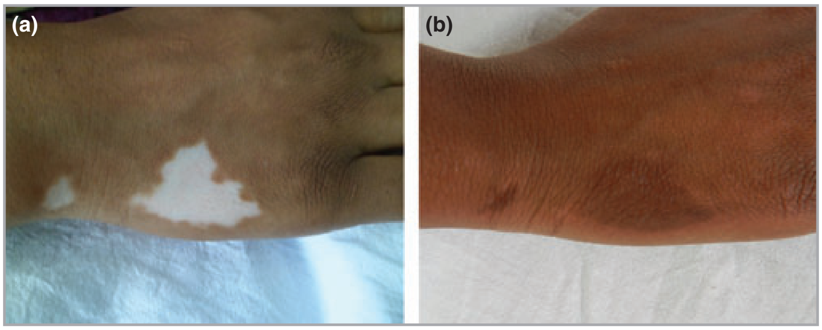

Fig 5. A patient in the noncultured epidermal cell suspension group with lesions of vitiligo over the dorsa of the hand showing 100% repigmentation. (a) Before surgery; (b) 16 weeks after surgery.

© 2013 The Authors
BJD © 2013 British Association of Dermatologists

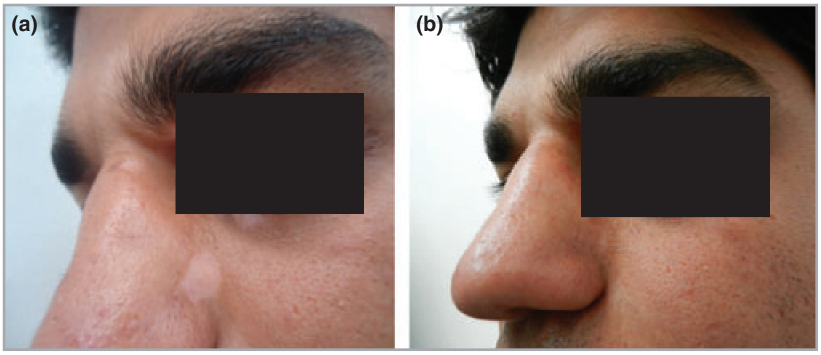

Fig 6. A patient in the noncultured extracted hair follicle outer root sheath cell suspension group with lesions of vitiligo over the face showing near 100% repigmentation. (a) Before surgery; (b) 16 weeks after surgery.

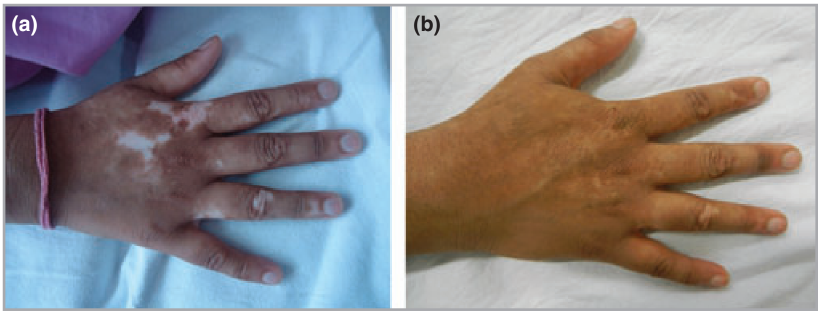

Fig 7. A patient in the noncultured epidermal cell suspension group with lesions of vitiligo over the dorsum of the hand showing near 100% repigmentation. (a) Before surgery; (b) 16 weeks after surgery.

Table 2 Mean scores of the three patient satisfaction questionnaires (PSQs) at weeks 4, 8 and 16

Measure	PSQ 1	PSQ 2	PSQ 3
Mean score			
NCES	9·13 ± 1·125	9·13 ± 1·060	9·00 ± 0·845
NCORSHFS	7·13 ± 2·416	7·27 ± 2·219	7·53 ± 2·066
P-value	0·007*	0·007*	0·017*

NCES, noncultured epidermal cell suspension; NCORSHFS, noncultured extracted hair follicle outer root sheath cell suspension. *Significant value ($P < 0·05$).

Melanocyte-lineage antigens plus cells stained with c-Kit (the receptor for stem cell factor) are localized in the outer layer of the outer root sheath of the infundibulum and mid-follicle and the matrix of the hair bulb.[9] This reservoir of melanocytes and melanocyte stem cells is important in the treatment of vitiligo, as the initial repigmentation in vitiligo patches often occurs around the hair follicles,[10] and vitiligo patches on skin lacking hair follicles, such as the palms and eyelids, are often resistant to medical therapies. There are many differences between epidermal and hair-follicle melanocytes. Epidermal melanocytes largely consist of a homogeneous population of highly dendritic and uniformly weakly pigmented cells. Cultured hair-follicle melanocytes showed at least three distinct subpopulations, including highly pigmented/dendritic bulbar melanocytes, less-differentiated tripolar cells, and an undifferentiated amelanotic bipolar subpopulation.[11,12] Follicular melanocytes appear to be more sensitive than epidermal melanocytes to ageing influence.[13] Amelanotic hair-follicle melanocytes differed from epidermal melanocytes in being less differentiated, and they expressed less mature melanosome antigens. In addition, hair-follicle melanocytes expressed some antigens associated with alopecia areata, but not antigens associated with vitiligo.[11] This could be an added advantage of repigmentation induced by NCORSHFS, and long-term follow-up is required for assessing the stability of repigmentation.

There are other populations of cells that might be used to constitute the hair follicular cell suspension.[14] These include basal cells high in α6-integrin/keratin 14 (K14) expression; suprabasal cells low in α6-integrin/K14 expression; hair germ cells expressing leucine-rich repeat-containing G protein-coupled receptor 5, P-cadherin and S100A4; and the bulge cells expressing CD34 and CD200, while a more distal population expresses MTS24.[14] The perifollicular connective tissue sheath and the papilla is a potential source for mesenchymal stem cells when using a cell suspension obtained from extracted hair follicles.[15]

The procedure involves removal of only 15–25 follicular units, which provide 25 000–50 000 cells, sufficient to treat up to 25 cm^2. From the experience gained from epidermal cell suspension transplantation, the experts recommended the desired number of cells for repigmentation as 2000 cells per cm^2.

Vanscheidt and Hunziker, in a small case series, have used a single-cell suspension of 'plucked' hair follicles in the treatment of vitiligo.[16] They found almost complete (> 90%) repigmentation in three of five patients with vitiligo, around 50% repigmentation in one patient and < 10% repigmentation in one patient. Mohanty *et al.*,[7] in their study, used a single-cell suspension of hair follicles extracted by the FUE method in the treatment of vitiligo. The mean ± SD repigmentation

© 2013 The Authors
BJD © 2013 British Association of Dermatologists

was 65·7 ± 36·7%. Overall, nine of 14 patients achieved ≥ 75% repigmentation. We observed excellent repigmentation (> 90%) in 65% of patients and good repigmentation (75–89%) in 12% of patients in the NCORSHFS group.

Excellent and good repigmentation were higher in the NCES group compared with NCORSHFS, but this was not statistically significant ($P > 0{\cdot}05$).

While considering the time taken for repigmentation, we found that repigmentation at 4 weeks was significantly higher with NCES compared with NCORSHFS ($P < 0{\cdot}001$). However, there was no significant difference in repigmentation at 8, 12 and 16 weeks ($P > 0{\cdot}05$). So it can be concluded that although final repigmentation at 16 weeks between the two groups did not differ significantly, the NCES technique produced significantly faster repigmentation than NCORSHFS.

In both techniques we did not use any postoperative phototherapy. We advised just sun exposure to all the patients. Our follow-up was 16 weeks, less than any study described in the literature. Yet we observed comparable and even somewhat better results than some studies in the literature for both of our techniques (NCES and NCORSHFS).

The most commonly observed pattern of pigmentation was diffuse. This signifies that the repigmentation was due to transplanted melanocytes by NCES and NCORSHFS. Perifollicular or marginal repigmentation is induced mainly by the melanocyte reserve in the hair follicle, which is stimulated by dermabrasion or phototherapy.

We observed a highly significant reduction in DLQI[17] after surgery in both the groups. However, there was no statistically significant difference in reduction in DLQI between NCES and NCORSHFS. Apart from DLQI, we also carried out patients' global assessment with the help of a patient satisfaction questionnaire including three questions: 'grade the change in pigmentation in the transplanted area', 'are you satisfied with the treatment?' and 'do you find the treatment worthwhile?' Patients were asked to answer in terms of scoring 0–10. The scores obtained by this patient satisfaction questionnaire were significantly higher in the NCES group than in the NCORSHFS group ($P < 0{\cdot}05$). This was due to highly significant repigmentation at 4 weeks in the NCES group compared with NCORSHFS. Although not statistically significant, overall repigmentation at 8, 12 and 16 weeks was higher in the NCES group compared with NCORSHFS.

To conclude, our study is the first of its kind in the literature to compare two different cellular transplantation techniques (NCES and NCORSHFS) in vitiligo. Our study indicates that both NCES and NCORSHFS are safe and effective techniques with comparable efficacy in terms of good repigmentation (≥ 75% extent of repigmentation), excellent repigmentation (90–100% repigmentation), colour match, DLQI reduction and side-effect profile, but NCES is superior to NCORSHFS in patient satisfaction.

References

1 Taïeb A, Picardo M. The definition and assessment of vitiligo: a consensus report of the Vitiligo European Task Force. *Pigment Cell Res* 2007; **20**:27–35.

2 Njoo MD, Westerhof W. Vitiligo: pathogenesis and treatment. *Am J Clin Dermatol* 2001; **2**:167–81.

3 Thompson AR, Kent G, Smith JA. Living with vitiligo: dealing with difference. *Br J Health Psychol* 2002; **7**:213–25.

4 Mysore V, Salim T. Cellular grafts in management of leucoderma. *Indian J Dermatol* 2009; **54**:142–9.

5 Gauthier Y, Benzekri L. Non-cultured epidermal suspension in vitiligo: from laboratory to clinic. *Indian J Dermatol Venereol Leprol* 2012; **78**:59–63.

6 Holla AP, Kumar R, Parsad D, Kanwar AJ. Modified procedure of noncultured epidermal suspension transplantation: changes are the core of vitiligo surgery. *J Cutan Aesthet Surg* 2011; **4**:44–5.

7 Mohanty S, Kumar A, Dhawan J *et al.* Non-cultured extracted hair follicle outer root sheath cell suspension for transplantation in vitiligo. *Br J Dermatol* 2011; **164**:1241–6.

8 Gauthier Y, Surleve-Bazeille J. Autologous grafting with noncultured melanocytes: a simplified method for treatment of depigmented lesions. *J Am Acad Dermatol* 1992; **26**:191–4.

9 Randall VA, Jenner TJ, Hibberts NA *et al.* Stem cell factor/c-Kit signalling in normal and androgenetic alopecia hair follicles. *J Endocrinol* 2008; **197**:11–23.

10 Lan CC, Wu CS, Chen GS, Yu HS. FK506 (topical tacrolimus) an endothelin combined treatment induces mobility of melanoblasts: new insights of follicular vitiligo repigmentation induced by topical tacrolimus on sun-exposed skin. *Br J Dermatol* 2011; **164**:490–6.

11 Kauser S, Westgate GE, Green MR, Tobin DJ. Human hair follicle and epidermal melanocytes exhibit striking differences in their aging profile which involves catalase. *J Invest Dermatol* 2011; **131**:979–82.

12 Tobin DJ. The cell biology of human hair follicle pigmentation. *Pigment Cell Melanoma Res* 2011; **24**:75–88.

13 Tobin DJ, Bystryn JC. Different populations of melanocytes are present in hair follicles and epidermis. *Pigment Cell Res* 1996; **9**:304–10.

14 Legué E, Sequeira I, Nicolas J-F. Hair follicle renewal: authentic morphogenesis that depends on a complex progression of stem cell lineages. *Development* 2010; **137**:569–77.

15 Sellheyer K, Krahl D. Skin mesenchymal stem cells: prospects for clinical dermatology. *J Am Acad Dermatol* 2010; **63**:859–65.

16 Vanscheidt W, Hunziker T. Repigmentation by outer-root-sheath-derived melanocytes: proof of concept in vitiligo and leucoderma. *Dermatology* 2009; **218**:342–3.

17 Finlay AY, Khan GK. Dermatology Life Quality Index (DLQI): a simple practical measure for routine clinical use. *Clin Exp Dermatol* 1994; **19**:210–6.

© 2013 The Authors
BJD © 2013 British Association of Dermatologists
