# Supplementary material for: Therapeutic efficacy of cell-based therapy in vitiligo: a research letter systematically reviewed using meta-analysis
Source: Arch Dermatol Res. 2024 May 22;316(5):198. doi: 10.1007/s00403-024-02920-6 (PMC11111487; doi:10.1007/s00403-024-02920-6)
Supplement: Supplementary file 1 — Supplementary file1 (ZIP 24195 KB) [file 403_2024_2920_MOESM1_ESM.zip › Studies were included/RCT Verma 2015.pdf]

Evaluation of repigmentation with Cultured Melanocytes transplantation (CMT) compared to non-cultured epidermal cells (NCES) transplantation in vitiligo at 12th week reveals better repigmentation with CMT.

Gunjan Verma[1*], Suraj RishaVarkhande[2*], Hemanta Kumar Kar[1@], Rajni Rani[2@]

1. Department of Dermatology, Post Graduate Institute of Medical Education and Research, Dr. Ram Manohar Lohia Hospital, New Delhi, India.
2. Molecular Immunogenetics Group, National Institute of Immunology (NII), New Delhi, India.

Supplementary Material

MATERIALS and METHODS

Thirty patients of stable vitiligo (24 females and 6 males with mean age 26.1±6.92 years) presenting to the Out Patient Department of Dermatology, Post Graduate Institute of Medical Education and Research, Dr. RML Hospital (PGIMER, RMLH), New Delhi, India, were recruited for this study after obtaining informed consent. The study was approved by Human Ethics Committee of PGIMER, RMLH and National Institute of Immunology following declaration of Helsinki protocols. Clinical diagnosis of vitiligo was made on the basis of depigmented macules, chalk or milk-white in colour with well demarcated margins. 26 of the patients were of vitiligo vulgaris type and 4 were segmental vitiligo cases. Cases without any new lesions for the preceding 6 months and no extension or regression of the lesions were considered stable.

Other inclusion criteria were ≥ 18 years of age, availability of at least two target patches of vitiligo vulgaris or segmental vitiligo, preferably of identical size (10 cm^2or more) present on the body of the patient. The exclusion criteria included presence of keloids and hypertrophic scars, pregnant and lactating women, patients suffering from any chronic illness like diabetes mellitus, thyroid disorders, immunocompromised patients, patients with bleeding disorders, patients on steroids or anticancer drugs and patients on any medical treatment of Vitiligo.

A detailed clinical history was taken and physical examination and routine laboratory tests were performed. All patients were also tested negative for HIV ELISA, HBs antigen and Anti-HCV RNA antibody levels.

Two target patches of vitiligo were measured by tracing them on a translucent graph paper (3-D measurement) and the photographs were also taken for later use. One of the target patches was transplanted with1500 cells/mm^2 of NCES and the other site received same number of CMT (Figure S1, S2). There was no significant difference in the average area to be treated with NCES (Mean ± S.E.M. 50.15±7.03 cm^2) and CMT (51.11±7.08 cm^2) (Figure S1a). After transplantation repigmentation in each case was analyzed using three different methods viz. visual estimation, 3-D estimation using graph paper (Figure S1b) and 2-D computerized analysis of digital photographs (Figure S1c, S3 and Figure 1). Since it took about 4 weeks to grow melanocytes *in-vitro* before transplantation and NCES were transplanted just the next day after harvesting the graft, the final 3-D and 2-D analyses were done after 16 weeks of NCES transplantation and after 12 weeks of CMT.

Harvesting of the graft

The skin graft of one-tenth size of the each recipient patches (NCES + CMT) was taken from the donor site (thighs, gluteal region or waist). Donor area was shaved, cleaned with betadine and surgical spirit and anaesthetized with mixture of 2% lignocaine. Split thickness skin graft was then taken with the help of skin grafting knife. Haemostasis was established and the area was dressed with antibiotic gauze. Suitable antibiotics and analgesics were prescribed. This tissue graft was divided equally into two pieces for NCES and CMT. In some cases 3mm-5mm punch biopsies were obtained for the preparation of NCES and CMT. The donor tissue was collected in sterile Hank's balanced salt solution (HBSS) (Gibco, USA) containing antibiotics and antifungal agents.

Autologous Non Cultured Epidermal Cell Suspension (NCES) Transplantation

All procedures were carried out under sterile conditions in laminar flow hood with negative pressure. The graft piece was washed with 70% alcohol for 1 minute and twice with HBSS to remove alcohol followed by incubation in 0.1% Trypsin / EDTA (Gibco, USA) solution overnight at 4°C. Trypsin inhibitor (Gibco, USA) was added after about 18 hours incubation. The disrupted tissue mass was then passed through a 5 ml syringe to get single cell suspension, washed with HBSS and resuspending in appropriate amount of HBSS to get 1500 cell/mm^2. In case of punch biopsies, the punches were rinsed in 70% alcohol, washed with HBSS and incubated with 0.25% Dispase II (Roche Diagnostics GmbH, USA) overnight at 4°C. Next morning the epidermis was removed from the dermis and the epidermal cells were teased out and resuspended in 3 ml of 0.1% Trypsin/EDTA and incubated for five minutes at 37°C followed by addition of M254 medium (Gibco, USA) to stop the action of trypsin. After another wash in the medium the cells were resuspended in HBSS for transplantation.

NCES Transplantation procedure:

On the second day, one out of the two target vitiligo patches was randomly selected and subjected to NCES transplantation. The skin was prepared for the surgical procedure as described earlier and after providing local anaesthesia, dermabrasion was done over the recipient vitiligo patch using an electrically operated dermabrader with diamond fraise rotating at 1500 to 2000 rotating speed per minute. Clinically, the adequacy of the level of dermabrasion was determined by the appearance of pinpoint bleeding points. The epidermal cell suspension (1500 cells/mm^2) was transplanted on the dermabraded recipient site using a pipette and spread out evenly with its tip, allowed to adhere for 5 minutes and covered with sterile paraffin gauze containing antibiotics and a thin transparent collagen film, which was kept in place with a thin

polyurethane membrane coated with a layer of an acrylic adhesive. The dressings were removed from both donor and recipient sites on the 7thday and patient was advised to refrain from direct exposure to sunlight and to avoid any local application on the treated sites after removal of dressing.

Autologous Cultured Melanocyte Transplantation

Melanocyte culture:

After rinsing with 70% ethanol and HBSS, the skin tissue was incubated with 0.25% Dispase II (Roche Diagnostics GmbH, USA) overnight (15 -18 hour) at 4°C. Next morning the epidermis was removed carefully from the dermis and the epidermal cells were teased out and resuspended in 3 ml of 0.1% Trypsin/EDTA and incubated for five minutes at 37°C followed by addition of M254 medium to stop the action of trypsin. The cell suspension was washed and resuspended in M254 medium containing PMA-free supplements and plated in a T25 flask and cultured at 37°C, in humidified 5% CO_2 atmosphere. When the cultures were confluent, cells were differentially trypsinized using 0.1% cold trypsin for 1 min to separate the melanocytes from keratinocytes. Melanocytes were propagated in M254 medium for one more passage and on subsequent passage the cells were treated with 100 µg/ml Geneticin (Gibco, USA) to remove contaminating fibroblasts.

All cultured melanocyte transplants were carried out with pure melanocytes (Figure S2) obtained from third to fifth passages of cultures when sufficient numbers of melanocytes were available, depending on the area of the lesion to be transplanted. Melanocytes were detached from tissue culture flask by trypsinization and resuspended in HBSS for transplantation.

Transplantation procedure:

For the second target patch, the patients were asked to return to the clinic after sufficient cultured melanocytes were available for transplant, which varied from 4 to 6 weeks after collection of the tissue from the donor site. The recipient site was prepared and dermabraded as described earlier and cultured melanocytes (1500 cells/mm^2) were carefully transferred to the dermabraded area and dressed as described earlier for NCES. Removal of the dressing and instructions to refrain from direct exposure to sunlight and to avoid any local application on the treated sites were the same as in case of NCES.

Follow-up and Analysis of repigmentation:

After transplantation repigmentation in each case was analyzed using three different methods viz. visual estimation, 3-D estimation using graph paper (Figure S1b) and 2-D computerized analysis of digital photographs (Figure S1c, S3 and Figure 1). Since it took about 4 weeks to grow melanocytes *in-vitro* before transplantation and NCES were transplanted just the next day after harvesting the graft, the final 3-D and 2-D analyses were done after 16 weeks of NCES transplantation and after 12 weeks of CMT. Follow-up for five cases was done for at least six months.

Visual Analysis

Follow- up was done at day 7th and 4th, 8th, 12th and 16th (for NCES) weeks after the transplantation and repigmentation was assessed and photographs were taken to score the extent of repigmentation. Visually, repigmentation was assigned the following scores depending on the

extent of repigmentation: 0-25% poor repigmentation, 25-50% fair repigmentation, 50-75% good repigmentation, 75-100% excellent repigmentation.

3-D Graphical evaluation

More objectively, to determine the area covered, the original lesional area and repigmented area were traced on a translucent graph paper to get approximate area and percent coverage was determined by the formula:

$$\frac{\text{Area repigmented}}{\text{Original area with the lesion}} \times 100$$

Also, the repigmentation pattern was noted as ‘diffused’, ‘perifollicular’ or ‘marginal’. A note was also made of the colour matching of repigmented skin as ‘somewhat lighter than’, ‘same as’ or ‘somewhat darker than’ normal skin. At each visit, patients were also asked about any adverse events and about the satisfaction with the procedure results at the end of 12 weeks.

2-D Computerized image analysis

To evaluate the percent area repigmented using photographs, they were converted to PDF format and the measuring tool of the Adobe acrobat reader XI software was used to trace the outline of the lesion and the repigmented part of the lesion (Figures 1 and S3). The measuring tool determined the area covered by the outlines. Then the total lesional area (area in red lines), the repigmented area (area in black lines), the nonpigmented (area in white lines) and the uninvolved area (area in green line) were determined and marked on the image by using measurement tool of Adobe reader XI software. Once the respective areas were drawn, the software displayed the respective measured areas. The percent repigmentation was determined by the same formula

used for 3D measurement i.e. by dividing the repigmented area by the total lesional area and multiplying by 100.

Statistical Evaluation

The statistical evaluation was carried out using Graph Pad Prism 4 software (San Diego, CA, USA) and STATA 9.2 statistical program. Paired t-test and Fisher's exact test with 2-sided p values were used to determine statistical significance. Odds ratios were calculated using Woolf's method (Woolf, 1955) with Haldane's (Haldane, 1956) modification as described earlier (Rani *et al*, 1998) since the numbers in one or the other group were less than 5. p value <0.05 was considered significant.

Table S1. Distribution of vitiligo lesions according to body site

Sites	Number of patients<br>N=30*	Percentage (%)
Abdomen	2	6.66
Arm, hands	5	16.66
Dorsum of foot	5	16.66
Leg	14	46.66
Trunk	2	6.66
Face	4	13.33

* The total number of sites for lesions is more than 30 since a few patients had lesions at two anatomical sites.

Table S2. Previous treatments taken by the patients

Treatment taken	Number of patients N=30	Percentage
Psoralen + UVA (PUVA) + Topical Steroids (TS) + Topical Calcineurin Inhibitor (TCI)	5	16.67
TCI+TS	16	53.33
PUVA + Solar UVA (PUVASOL) + TCI + TS	1	3.33
TCI+PUVA+PUVASOL	3	10
Oral minipulse of dexamethasone (OMP) + TCI + PUVA	1	3.33
TCI + Narrow band UVB (NBUVB) +TS	4	13.33

Table S3. Family history of vitiligo in 30 enrolled cases.

Positive family history	Number of patients N=30	Percentage
Absent	17	56.66
Present	13	43.33

Table S4. Visually estimated extent of repigmentation in 27 cases followed up for 4th, 8th and 12th weeks after transplantation with cultured melanocytes (CMT) and non-cultured epidermal cell suspension (NCES).

Extent of pigmentation	CMT N=27			NCES N=27			CMT Versus NCES p value# Odds Ratio@ (95% Confidence Interval)		
Follow-up at	4 weeks No. (%)	8 weeks No. (%)	12 weeks No. (%)	4 weeks No. (%)	8 weeks No. (%)	12 weeks No. (%)	4 weeks	8 weeks	12 weeks
Excellent (>75% repigmentation)	2 (7.4)	16 (59.25)	19 (70.37)	0 (0)	0 (0)	2 (7.4)	0.49 5.39 (0.61-47.7)	1.23×10^{-6} 76.52 (9.86-593.8)	3.09×10^{-6} 23.4 (7.94-68.95)
Good (51-75% repigmentation)	20 (74.07)	9 (33.33)	7 (25.92)	1 (3.7)	1 (3.7)	1 (3.7)	9.32×10^{-8} 48.29 (13.09-178.06)	0.01 9.07 (2.5-32.8)	0.05 6.46 (1.7-23.8)
Fair (26-50% repigmentation)	4 (14.8)	2 (7.4)	1 (3.7)	4 (14.8)	2 (7.4)	12 (44.44)	1.0 1.0 (0.36-2.74)	1.0 1.0 (0.27-3.66)	0.0009 0.07 (0.02-0.25)
Poor (<25%repigmention)	1 (3.7)	0 (0)	0 (0)	22 (81.48)	24 (88.88)	12 (44.44)	4.05×10^{-9} 0.01 (0.003-0.052)	4.17×10^{-12} 0.002 (0.0003-0.021)	0.0001 0.02 (0.002-0.17)

# p values calculated using Fisher's exact test, @ Odds ratios and 95% Confidence Intervals calculated using Woolf's method (Woolf, 1955) with Haldane's (Haldane, 1956) modifications since at least one of the numbers was less than 5 in each calculation.

Table S5. A comparison of percent coverage by CMT and NCES using 3-D graph paper analysis and 2-D computerized image analysis at 12thweek after CMT and 16 weeks after NCES.

Percent Repigmentation	3-D graph paper analysis N=27		CMT vs NCES	2-D computerized image analysis of pictures N=25		CMT vs NCES
	CMT No. (%)	NCES No. (%)	p value	CMT No. (%)	NCES No. (%)	p value
50-98%	27 (100%)	3 (11.11%)	$4.17x10^{-12}$	24 (96%)	3 (12%)	$1.1x10^{-9}$
>30%	27 (100%)	9 (33.33%)	$9.7x10^{-8}$	25 (100%)	9 (36%)	8.2×10^{-7}

Table S6. Color match of repigmented skin: a comparison between NCES and CMT

Color of repigmented areas	CMT N=27		NCES N=27	
	Number of patients	Percentage	Number of patients	Percentage
Same as normal skin	10	37.04	14	51.85
Somewhat lighter than normal skin	0	0	1	3.7
Somewhat darker than normal skin	17	62.96	12	44.44

Table S7. Patient satisfaction survey to evaluate their satisfaction with respect to NCES and CMT

Patient satisfaction	CMT N=27		NCES N=27		
	Number of patients	Percentage	Number of patients	Percentage	p value<br>Odds Ratio<br>(95% Confidence Interval)
Extremely satisfied	18	66.66	0	0	9.6×10^{-8}<br>107.1<br>(13.73-834.99)
Somewhat satisfied	5	18.52	9	33.33	0.17<br>0.454<br>(0.11-1.86)
Neither satisfied nor dissatisfied	4	14.81	8	29.63	0.16<br>0.439<br>(0.17-1.09)
Somewhat dissatisfied	0	0	7	25.93	0.01<br>0.04<br>(0.006-0.39)
Extremely dissatisfied	0	0	3	11.11	0.23<br>0.127<br>(0.015-1.07)

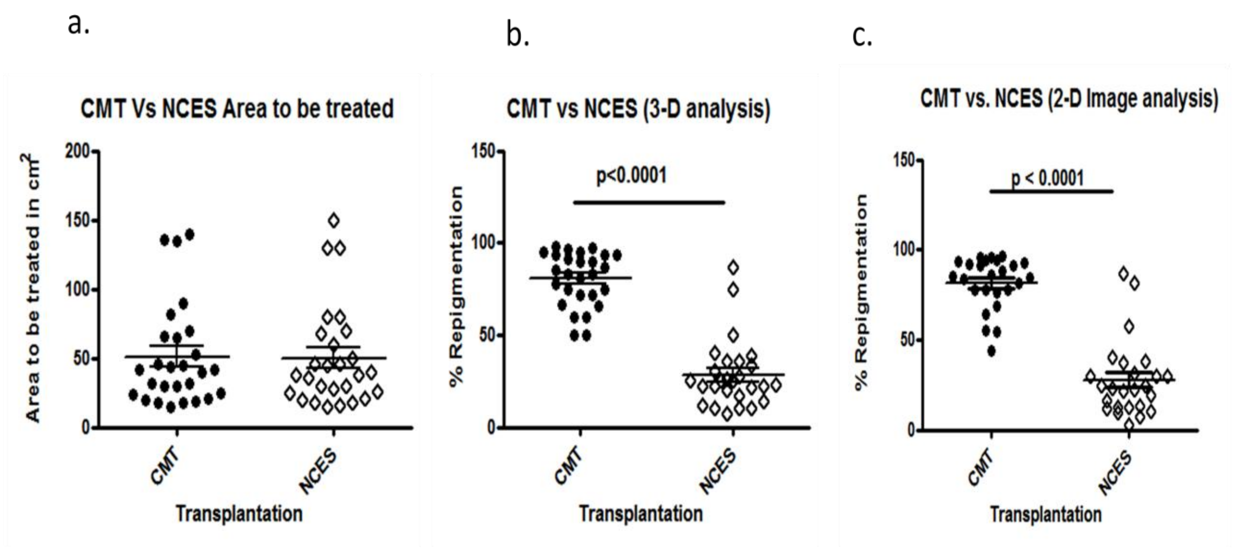

Figure S1. a. Comparison of the areas to be treated with NCES and CMT showing no significant difference in the average area to be treated with NCES (Mean ± S.E.M. 50.15±7.03 cm^2) and CMT (51.11±7.08 cm^2), b. percent coverage showing repigmentation after two treatments as determined by 3-D evaluation using graph paper (80.59 ± 2.8% with CMT and 28.39 ± 3.5% with NCES), c. percent coverage showing repigmentation after two treatments as determined by 2-D computerized evaluation (81.66 ± 2.86% with CMT and 27.73 ± 4.19% with NCES).

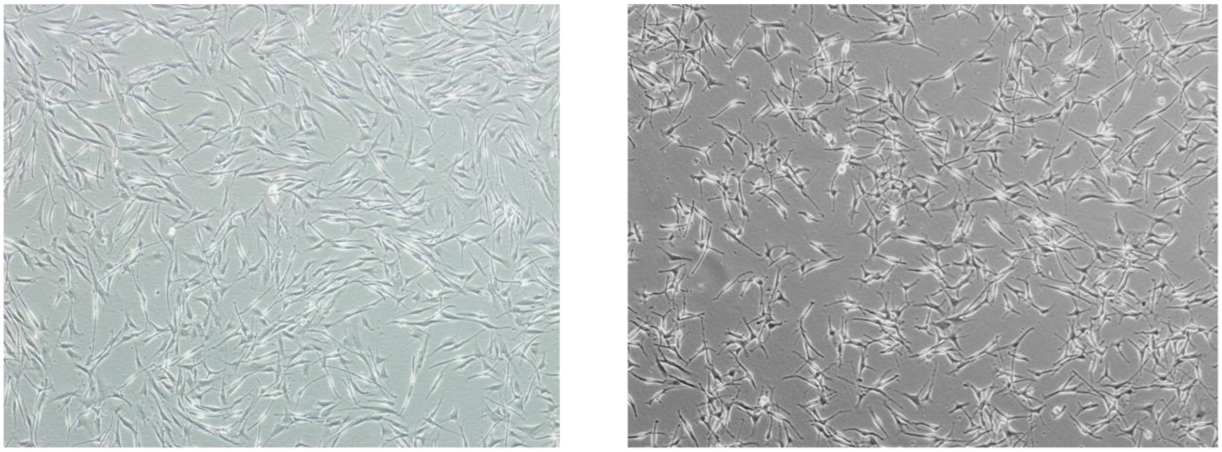

Figure S2. Representative samples of pure melanocytes in culture before transplantation of CMT (Phase contrast images).

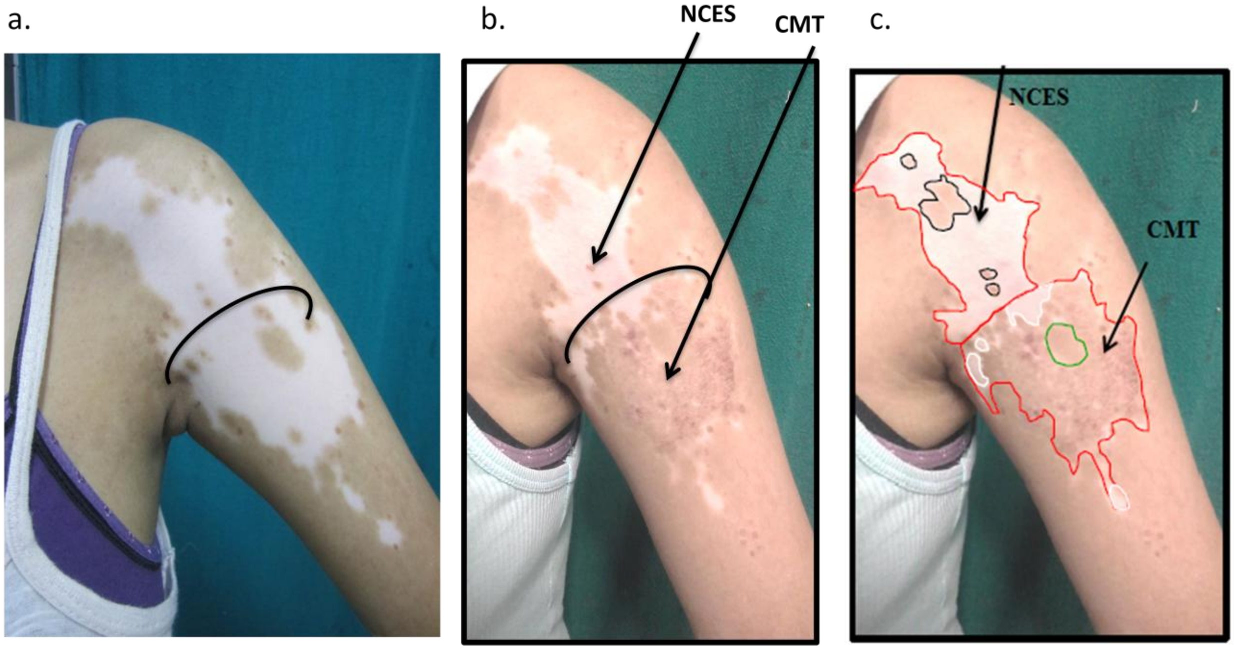

Figure S3. A segmental vitiligo case treated with NCES and CMT. a. Lesional upper arm before treatment, b. lesional area treated with NCES and CMT 16 and 12 weeks after treatment respectively, c. Measurement of lesional area and repigmented area using Adobe Acrobat Reader XI. The total lesional area (area in red lines), the repigmented area (area in black lines, the non-pigmented (area in white lines) and uninvolved area (area in green line).

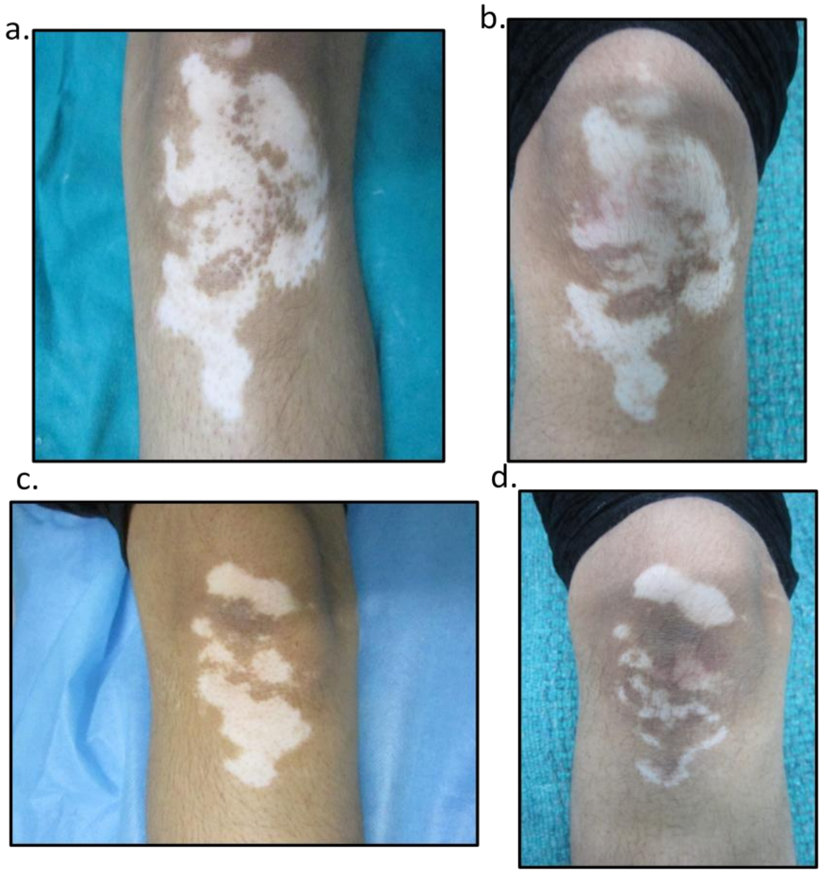

Figure S4. A vitiligo vulgaris case treated with NCES and CMT. a. left knee before treatment, b. left knee 4 months after treatment with NCES showing fair repigmentation, c. right knee before CMT, d. right knee three months after CMT showing excellent repigmentation.

.

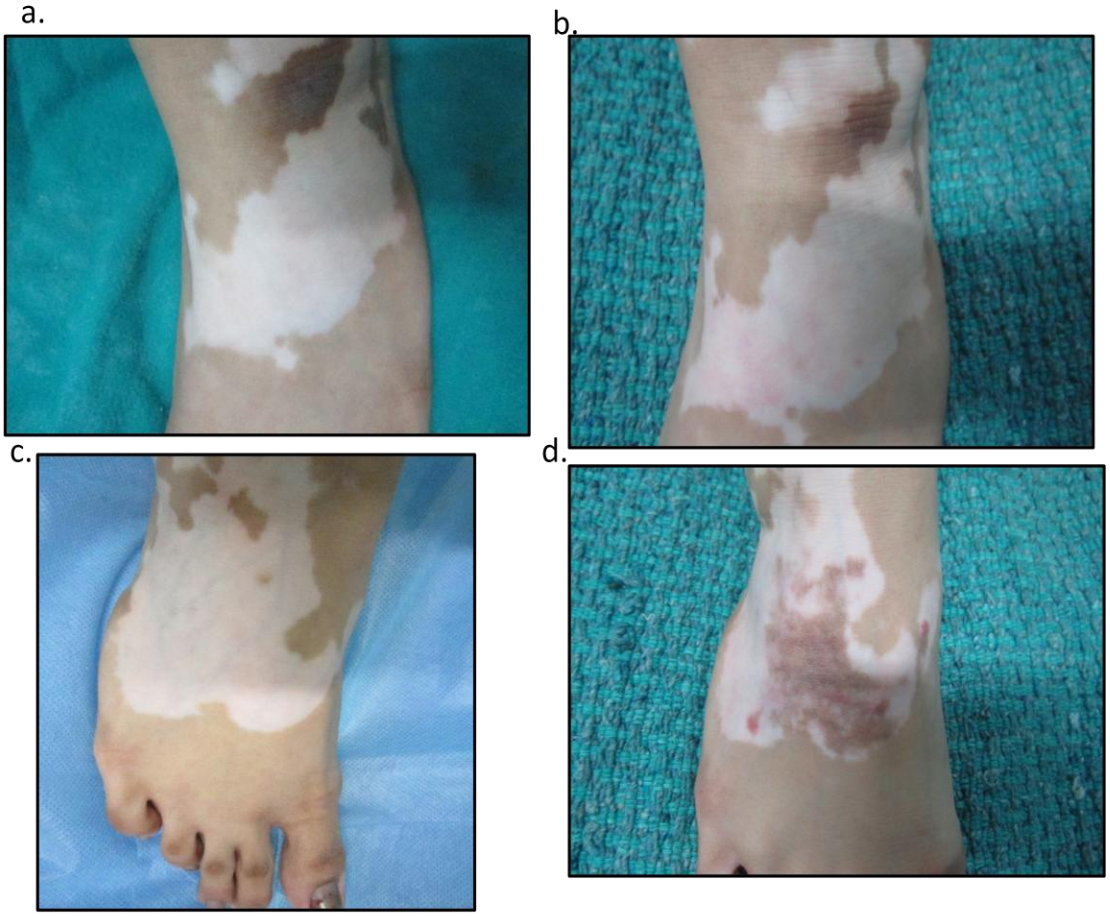

Figure S5. A vitiligo vulgaris case treated with NCES and CMT. a. left foot before treatment, b. left foot 4 months after treatment with NCES showing poor repigmentation, c. right foot before CMT, d. right foot three months after CMT showing good repigmentation.

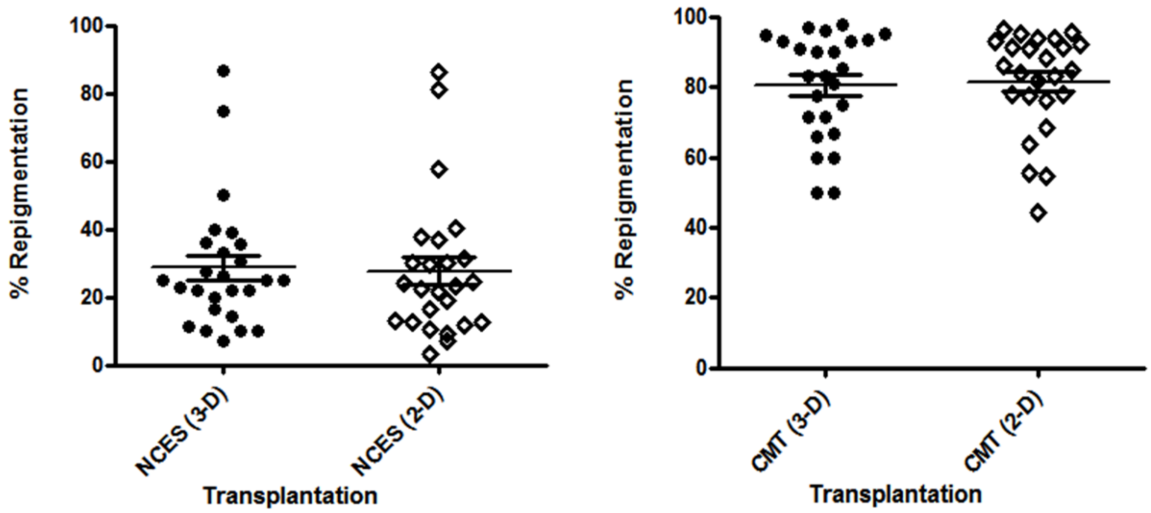

Figure S6. Comparison of 3-D and 2-D analysis shows that there was no significant difference between the two types of analysis for NCES and CMT transplantation.

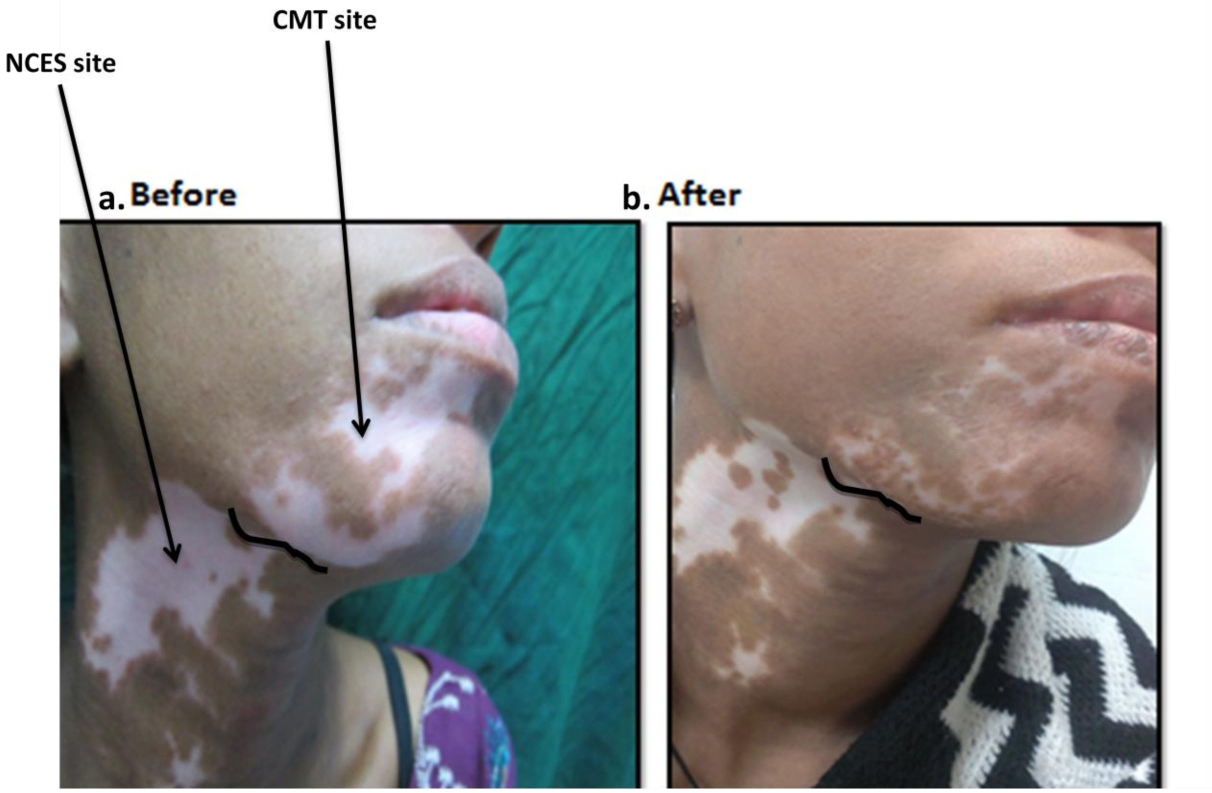

Figure S7. A segmental vitiligo case treated with NCES and CMT. a. Right side of the neck and chin before NCES and CMT treatment, b. Right side of the neck showing poor pigmentation after NCES transplantation and the chin showing excellent repigmentation three months after CMT . NCES and CMT sites are shown by arrows.

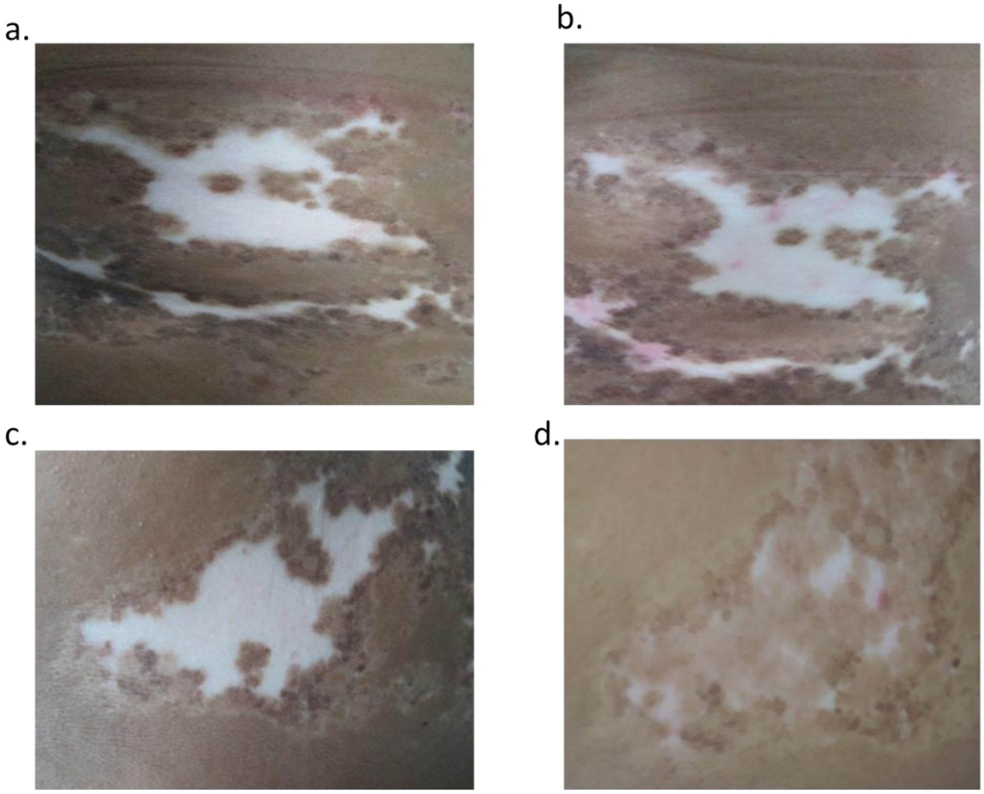

Figure S8. A segmental vitiligo case treated with NCES and CMT. a. lesion on abdomen before NCES treatment, b. lesion on abdomen showing poor pigmentation after NCES transplantation. c. Another lesion on abdomen before CMT treatment. d. Lesion shown in c three months after CMT showing excellent repigmentation.

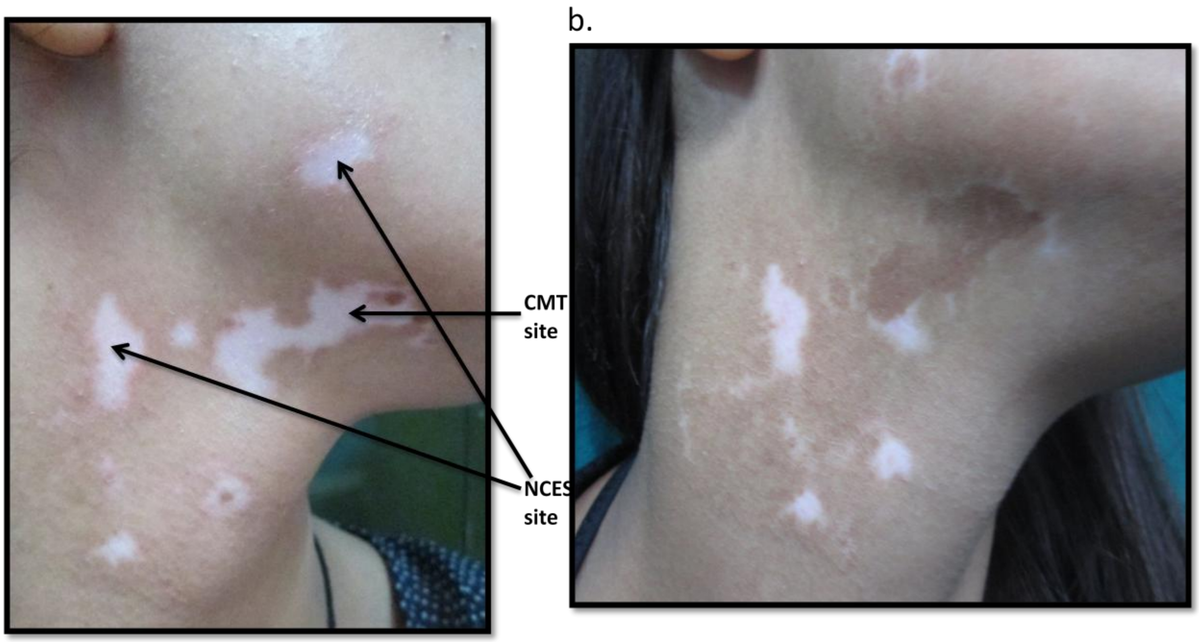

Figure S9. A segmental vitiligo case treated with NCES and CMT. a. Right side of the neck and face before NCES and CMT treatment, b. Right side of the neck showing poor pigmentation after NCES transplantation and the face showing excellent repigmentation three months after CMT. NCES and CMT sites are shown by arrows.

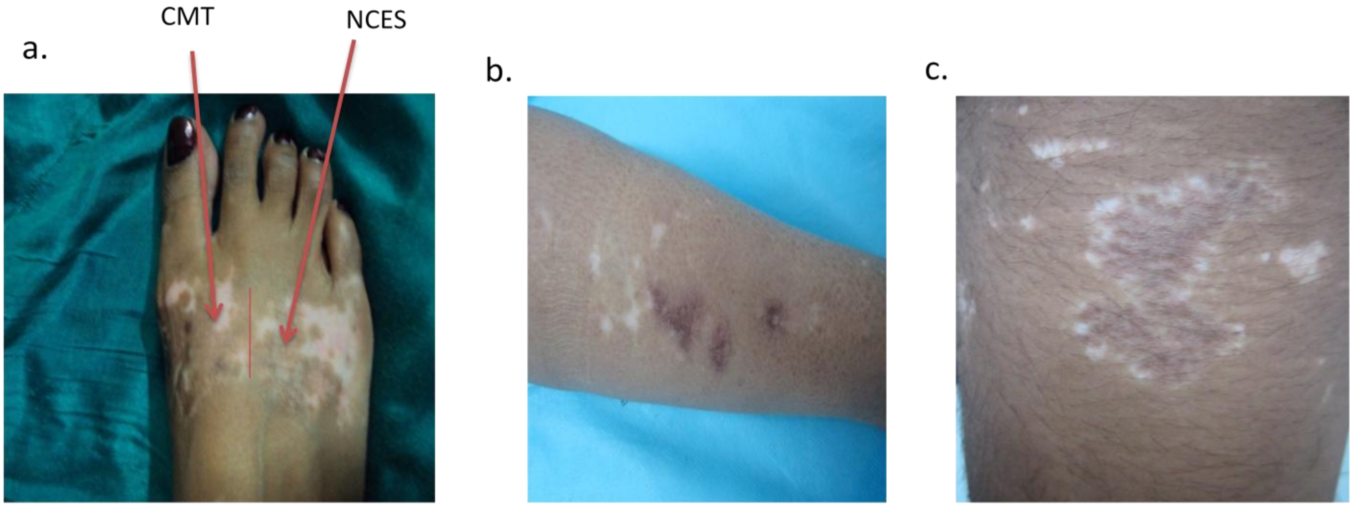

Figure S10. Three vitiligo vulgaris cases treated with NCES showing considerable good repigmentation a. one case showing 57.8% repigmentation, b. second case showing 81.25% repigmentation with NCES and c. third case showing 86.6% repigmentation after 16 weeks of NCES transplantation.

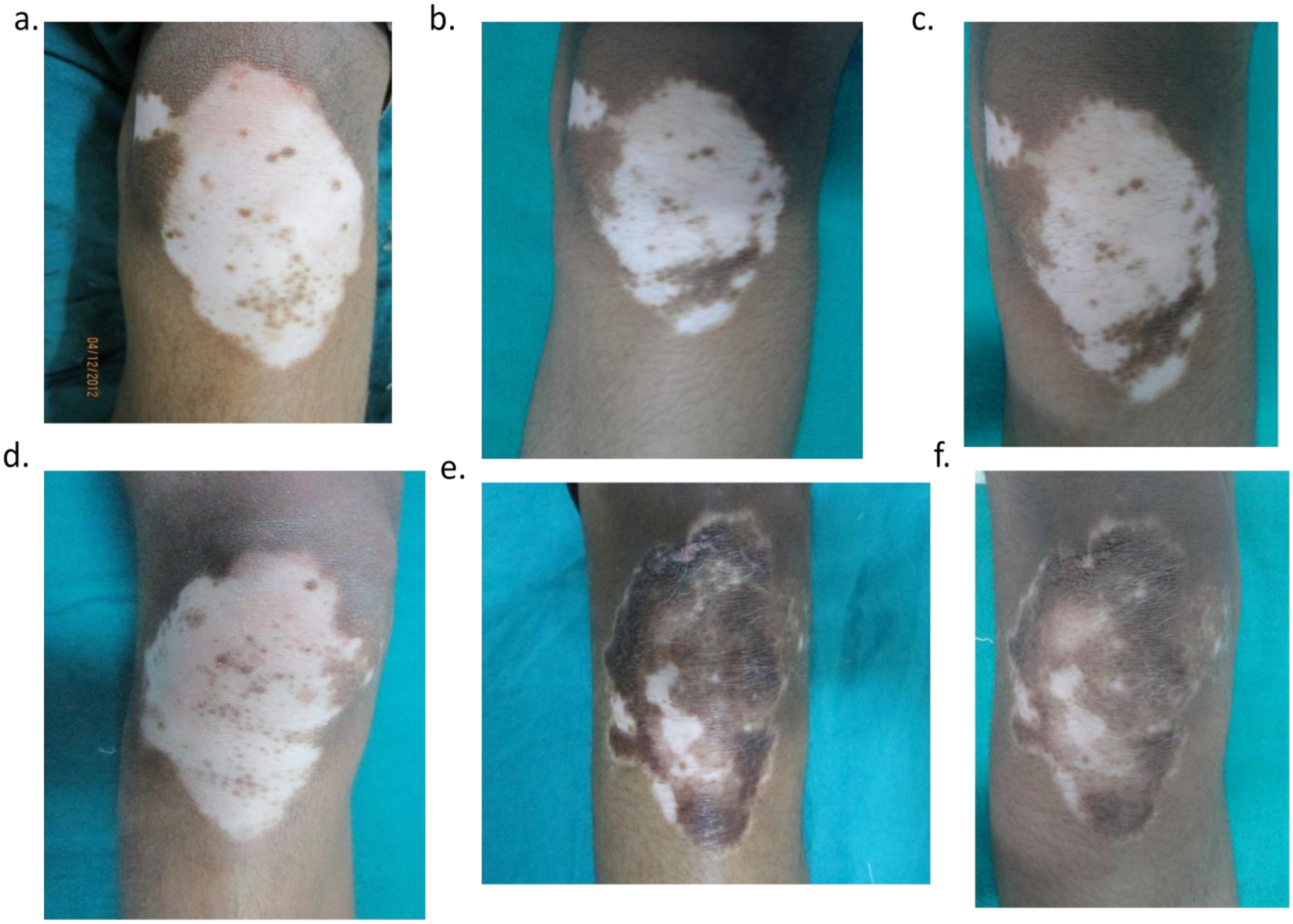

Figure S11. A vitiligo vulgaris case treated with NCES and CMT, a. left knee before treatment, b. left knee treated with NCES four months after treatment, c. left knee treated with NCES seven months after treatment, d. right knee before CMT, e. right knee three months after CMT showing 97% repigmentation which is slightly darker than the normal skin colour, f. right knee six months after CMT showing lightening of repigmented patch matching the normal skin colour

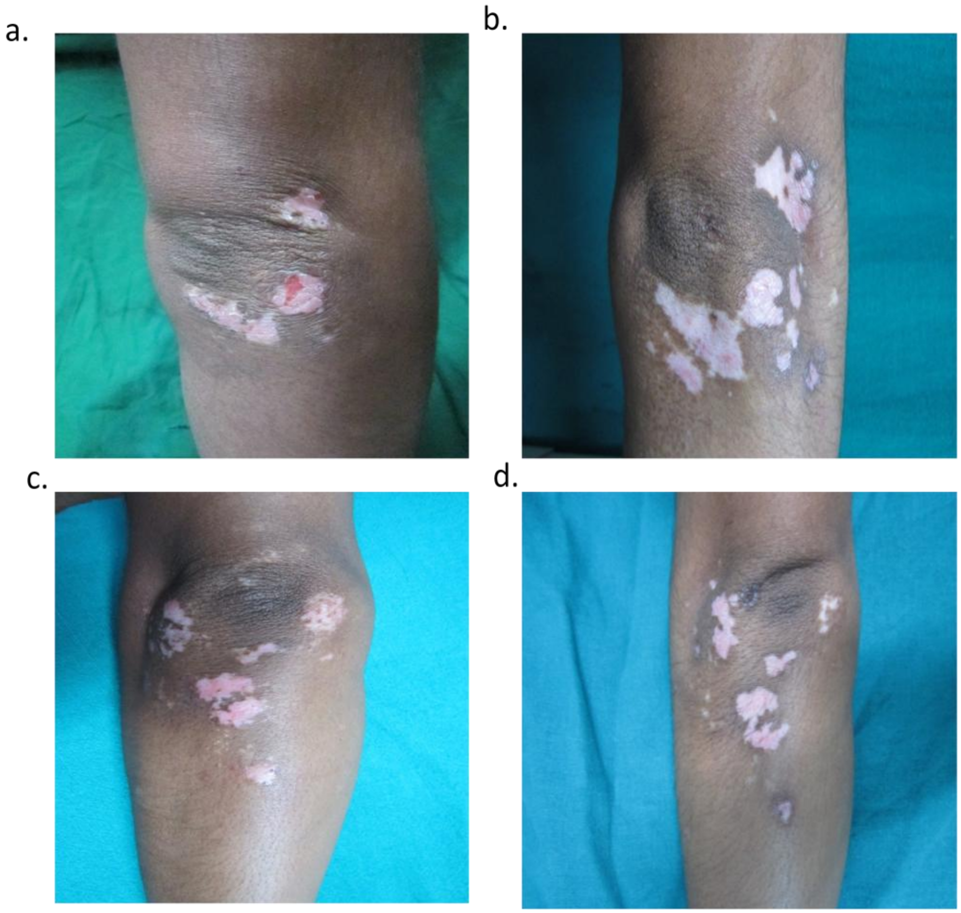

Figure S12. A vitiligo vulgaris case treated with NCES and CMT on the elbows showing difficult to treat sites with cellular grafting. a. left elbow before treatment, b. left elbow treated with NCES four months after treatment, c. right elbow before CMT, d. right elbow three months after CMT.

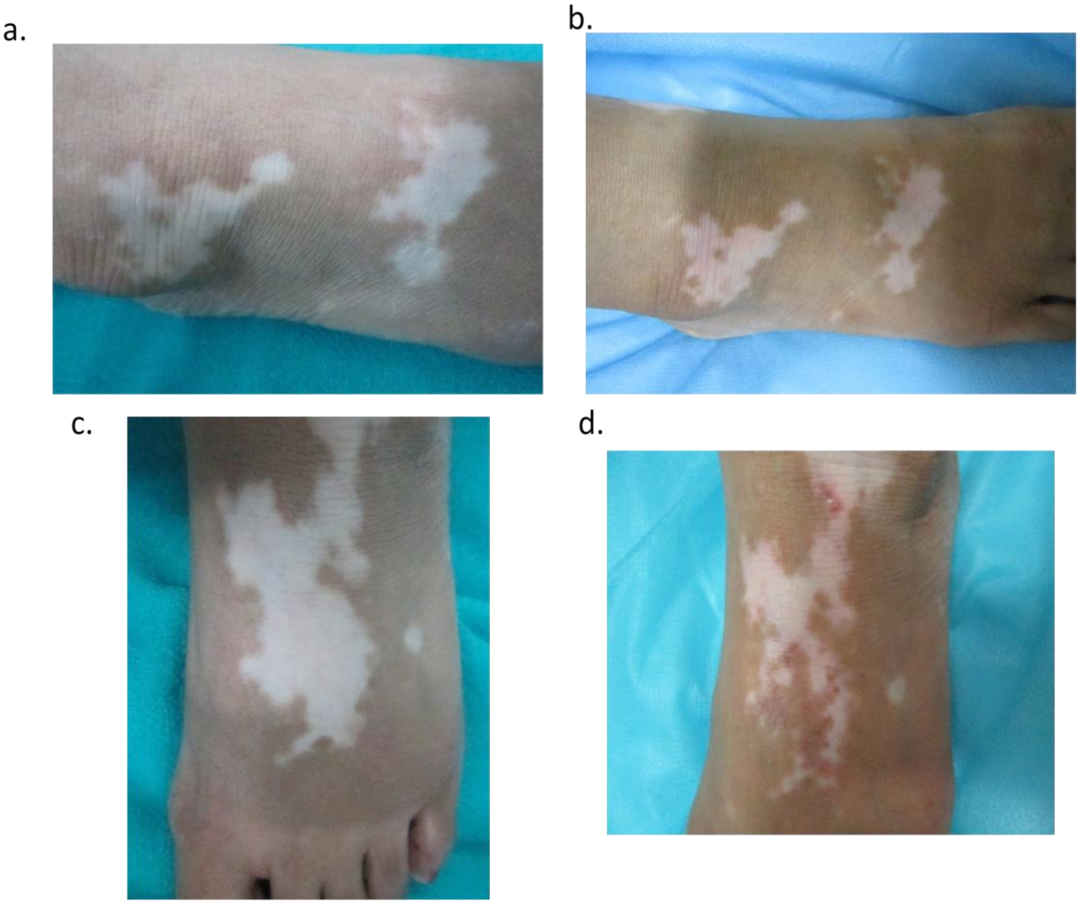

Figure S13. A vitiligo vulgaris case treated with NCES and CMT on the dorsum of the foot showing difficult to treat sites with cellular grafting. a. right foot before treatment, b. right foot treated with NCES four months after treatment, c. left foot before CMT, d. left foot three months after CMT.

References

Haldane JB. (1956). The estimation and significance of the logarithm of a ratio of frequencies. *Ann Hum Genet* 20: 309-311.

Rani R, Fernandez-Vina MA, Stastny P. (1998). Associations between HLA class II alleles in a North Indian population. *Tissue Antigens* 52: 37-43.

Woolf B. (1955). On estimating the relation between blood group and disease. *Ann Hum Genet* 19: 251-253.
