# Supplementary material for: Therapeutic efficacy of cell-based therapy in vitiligo: a research letter systematically reviewed using meta-analysis
Source: Arch Dermatol Res. 2024 May 22;316(5):198. doi: 10.1007/s00403-024-02920-6 (PMC11111487; doi:10.1007/s00403-024-02920-6)
Supplement: Supplementary file 1 — Supplementary file1 (ZIP 24195 KB) [file 403_2024_2920_MOESM1_ESM.zip › Studies were included/Talukdar K 2016.pdf]

International Journal of Medical Research and Review

Randomized control trial to compare the efficacy of split thickness transplant versus autologous melanocyte transplant in the management of stable vitiligo

Talukdar K [1], Mitra D [2]

[1]Dr Krishna Talukdar, Associate Professor & HOD, Jorhat Medical College, Jorhat, Assam, [2]Dr Debdeep Mitra, Graded Specialist, Air Force Hospital Jorhat, Assam, India

Address for Correspondence: Dr Krishna Talukdar, Email: drkrishna.talukdar@gmail.com

Abstract

Background: Stable cases of vitiligo can be managed surgically and over the years there has been a shift in the management protocol from the time tested epidermal tissue grafting techniques to the newer melanocyte or cellular transplant techniques. This randomized control trial has evaluated the relative efficacy of both the transplant techniques in the management of stable cases of vitiligo with at least one year of stability. **Methods**: Epidermal split thickness grafts were harvested from medial aspect of thigh. In the split thickness epidermal grafting technique the donor tissue was directly transplanted on the dermabraded vitiliginous areas. In the autologous melanocyte transplant technique melanocytes were harvested from a donor split thickness graft as a melanocyte rich cell suspension, which was then transplanted to the recipient area that had been superficially dermabraded. 50 patches of vitiligo in patients were randomly allocated into 2 groups to receive either of the two interventions. **Results**: An excellent response was seen in 64% cases with the melanocyte cell suspension technique and in 52% with the split thickness epidermal grafting technique. **Conclusion**: Both the surgical techniques are effective in obtaining re-pigmentation in recalcitrant but stable lesions of vitiligo. Large areas of skin can be covered with a smaller donor skin using melanocyte transfer technique; however melanocyte transplant method is more time consuming, and a labor intensive process, requiring state of the art equipments with a sterile laboratory and dermato-surgery setup.

OPEN JOURNAL SYSTEMS

Journal Help

USER

Username

Password

Remember me

Login

NOTIFICATIONS

- View
- Subscribe

JOURNAL CONTENT

Search

Search Scope

All

Search

Browse

- By Issue
- By Author
- By Title
- Other Journals

FONT SIZE

INFORMATION

- For Readers
- For Authors
- For Librarians

Keywords: Melanocyte transplantation, Vitiligo surgery, epidermal grafting, Stable vitiligo

---

Manuscript received: 18th Jan 2016**, Reviewed**: 28th Jan 2016 **Author Corrected:** 08th Feb 2016**, Accepted for Publication:** 17th Feb 2016

Introduction

Vitiligo is chronic pigmentary dermatoses that produces hypo-pigmented or de-pigmented macules which results from reduction or absence in the number of epidermal melanocytes in mucous membrane and/or skin. It leads to severe cosmetic disability and in addition to its immense socio-psychological ramifications. Treatment in vitiligo is based on decreasing the disease activity, thereby achieving stability and later inducing pigmentation. Most of the times, medical therapy in the form of immune-suppressives or phototherapy, alone is not sufficient and the vitiliginous macules may remain as it is without showing any repigmentation. Such type of patients who are stable for more than 1 year are considered fit for surgical treatment options including transfer of epidermal skin sheets or autologous melanocytes [1]. These surgical procedures basically donate some viable melanocytes to the affected area of depigmentation. These viable pigmentary cells are then stimulated by different means like phototherapy including Narrow Band Ultra-Violet B therapy or PUVA-sol therapy. These techniques are collectively known as surgical grafting procedures and they are a popular choice among dermatologists. The extent or the area of the vitiligo lesion to be treated, the age of the patient, the site of the de-pigmented lesions, patient expectations and lastly the expertise of the treating surgeon are few of the factors that help in deciding the grafting procedure to be performed. In general, grafting techniques in managing stable vitiligo are divided mainly into two groups: Tissue grafting and cellular grafting procedures. [2,3]. As the former group includes the different techniques of transferring tissue grafts as a whole to the involved recipient skin, the latter group involves further separation of these epidermal grafts into cellular components. These cellular components are then spread on to the dermabraded recipient skin either as such or after growth and multiplication in culture media. Generally, tissue grafting procedures are much easier and simpler to perform than the cellular transplantation methods. Replenishing melanocytes selectively within depigmented macules is a promising and novel treatment [2,3,4]. We undertook this study to compare the two methods of grafting procedure in stable vitiligo, namely melanocyte rich cell suspension and epidermal graft transfer for replenishing the lost melanocytes in the vitiliginous macules.

Material and Methods

This was a randomized control study wherein 25 unresponsive sites of stable vitiligo, each were operated upon by the 2 modalities, i.e. split thickness epidermal graft transfer and non cultured autologous melanocyte transfer technique leading to a total of fifty sites in all, over a period of one year. Simple random sampling method was used to randomize the 50 patches of stable vitiligo. Patients having a stable form of vitiligo (no increase in the size of the lesion for at least 1 year) and with a of body surface

area involvement of up to a maximum of 20% were included in the study. The pigmentation was compared to the pre-procedure status after 6 months post procedure based on observational analysis and comparing digital photographic records. No blinding was done in the study.

Sample size calculation was done based on the incidence of vitiligo in the society as mentioned in the various studies followed by statistical calculation. Though the calculated sample size was 22, twenty five unresponsive sites were considered for surgery by each of the modalities, leading to a total of fifty sites in all. Pre operative work-up consisted of an informed consent, clinical photographs, screening for Hepatitis B and HIV infection and charting of the area to be grafted.

Two techniques were employed: the epidermal graft transfer technique [3,4,5] and autologous melanocyte cell suspension (non cultured melanocyte) technique [6,7,8,9] . Epidermal split thickness skin grafting is claimed to be the one of the most successful amongst the tissue grafting procedures in vitiligo at present [4]. Non culture autologus melanocyte transplant on the other hand is a type of cellular grafting which involves separation of of a split-thickness skin graft into its different cellular components in a controlled environment in the presence of a nourishment media. The cellular suspension which consists of a mixture of melanocytes and epidermal keratinocytes is then applied on to a dermabraded recipient area. The procedure of melanocyte transplant offers certain key advantages over the traditional tissue grafting techniques in that a larger area of depigmented skin can be managed in a single operating session and with a much smaller size of donor skin graft.

Donor site: Usually cosmetically not so important sites like the medial aspect of thighs or the buttocks were selected for harvesting the donor tissue. About one-tenth the size of the recipient depigmented area was selected as the donor site in the melanocyte transplant technique; however the donor and the recipient size were approximately same in the epidermal graft transplant technique. It was cleaned and draped. The site was anesthetized locally and a very superficial skin graft was harvested using Silver's skin grafting knife. The raw donor area over the inner aspect of thigh, was then dressed with Sofra-tulle after achieving hemostatsis.

Laboratory procedure for melanocyte separation: The skin grafts obtained were immediately transferred to 5 ml of 0.25% trypsin-EDTA solution contained in a petridish. This mixture of trypsin-EDTA with epidermal grafts was incubated at 37 °C for one hour. The grafts were then gradually transferred onto a dish containing ten ml of melanocyte nourishment medium i.e. DMEM-(Dulbecco's modified eagle medium/F12). This media also diluted and terminated the proteolytic action of trypsin. Laminar air flow bench and strict asepsis was maintained throughout the procedure. The epidermis was scraped and teased gently from the dermis with forceps till it was clear of any dark pigment. The dermal pieces were discarded and the pigmented epidermal pieces were retained. Care was taken that the processing of the melanocytes did not take a long time as this would hamper the vitality of the melanocytes. The contents of the petridish were transferred gradually onto a centrifuge tube and centrifuged for 10 minutes at 3000 rpm. The pigmented cell pellet rich in

melanocytes and keratinocytes settled down at the bottom. The supernatant fluid was kept aside to be reused for soaking the gauze pieces for dressing, and the cell pellet, containing cells rich in melanocytes, was taken. The pellet was again re-suspended in a total volume of 0.6 ml DMEM medium and transferred gradually in steps to a syringe.

Recipient site (vitiliginous area): These areas were dermabraded down to the papillary dermis with a CO2 ablative Laser in a de-focussed mode or a diamond fraise wheel electrical dermabrader after surgical cleaning and infiltration of local anaesthesia.

The epidermal graft was placed on the dermabraded area in cases managed with epidermal sheet grafting and the cell suspension was applied evenly on the abraded area and spread uniformly with spatula in the melanocyte grafting technique. The areas were covered initially with a collagen dressing and there-after with gauze pieces soaked in DMEM/F12 and held in place by transparent Tegaderm dressing. Patient was made to lie down for 30 minutes with limb elevation if required and then discharged with the instructions to avoid vigorous physical activities and to carry out only restricted movements for next one week.

Post operative care: Analgesics and oral antibiotics were given for a total duration of 5 days in all the cases. Donor area dressing was changed on every second day and for the recipient areas, the dressing was removed after a total of one week. PUVASOL (1:10) was added for accelerating the repigmentation process and was started two weeks after the erythema subsided. The patients were followed up at one, two, three and six months after procedure for assessing re-pigmentation.

Results

A total of 20 patients were enrolled in this randomized control study, which included 25 sites of depigmentation for autologous melanocyte–keratinocyte transfer and 25 sites for epidermal graft transplant. In the melanocyte transplant group 9 patients were enrolled with 25 unresponsive depigmented sites. 6 were males (66.7%) and 3 (33.3%) were females. In the epidermal graft transplant group 11 patients with 25 unresponsive depigmented sites were enrolled. 5 (45%) were male patients and 6 (55%) were females. In the melanocyte transplant group, minimum duration of vitiligo was 2 years and maximum duration was 12 years; mean duration was 7.00 ± 3.43 years. In epidermal graft transplant group, minimum duration of vitiligo was 4 years and maximum duration was 13 years; mean duration was 7.73 ± 4.25. Demographically and statically both the groups were comparable.

Post operative evaluation: At first follow up, dried serum and crusted scabs were seen after removal of dressing from the treated recipient areas, partially attached to the skin surface leaving behind erythematous depigmented area. Infection and pus discharge was noted at one of the sites following melanocyte transplant technique and 5 sites following epidermal graft transplant technique; all of which resolved within a week of oral antibiotics without any scarring. Percentage of re-pigmentation was calculated after six months of procedure, for both the groups and the same has been highlighted in Table 1.

- In the epidermal graft transplant method, 13 (52%) patches showed good re-pigmentation, i.e. more than 70% re-pigmentation [Fig. 1], 5 (20%) patches showed fair re-pigmentation, i.e. 30–69% repigmentation, 7 (28%) patches showed a poor re-pigmentation, i.e. less than 30% re-pigmentation.
- In the melanocyte transplant method, 16 (64%) patches showed good re-pigmentation, i.e. more than 70% re-pigmentation [Fig2], 5 (20%) patches showed fair re-pigmentation, i.e. 30–69% re-pigmentation, 4(16%) patches showed a poor re-pigmentation, i.e. less than 30% re-pigmentation.

figure01 figure02

figure03 figure04

Fig. 1: Epidermal graft transplant: procedure and results

figure05 figure06

figure07 figure08

figure09 figure10

Fig. 2: Melanocyte transplant : Procedure & Results

Table-1: Percentage of repigmentation.

**Repigmentation**	**Epidermal grafting**	**Melanocyte transplant**
Poor (≤30%)	7	4
Fair (31%–70%)	5	5
Good (>70%)	13	16
**Total No of grafts**	**25**	**25**

No difference was noted in the extent re-pigmentation process in the two methods statistically. Chi-square test was used as the statistical test and p-value for the degree of re-pigmentation noted after 6 months was 0.059, which was >0.05; therefore there was no statistical association between method used to treat and the outcome that is re-pigmentation. So the final outcome was that re-pigmentation is independent of the modality of treatment used in this study. However in patients of the melanocyte transplant group, more patches showed excellent results as compared to the epidermal graft transplant group, though this was not statistically significant.

Both the methods proved that patches over lips, face, trunk and hairy parts of extremities showed good re-pigmentation however patches over bony prominences over extremities and acral areas had poor re-pigmentation.

Complications: One patient in the melanocyte transplant group with two patches and one patient in the epidermal graft transplant group with four patches, after the procedure noted a reactivation vitiligo. Both the recipient and donor areas were depigmented 1

month after the procedure. No re-pigmentation was noted even after 6 months of follow up. One patient complained of pain and one patient had erythema and pus discharge at the donor site in the melanocyte transplant group and one patient in the epidermal graft transplant group noted redness and foul smelling pus discharge at the donor site and few of the recipient sites after 7 days once the dressing was removed. The infection was controlled with one week of topical and oral antibiotics. This patient however had excellent re-pigmentation.

Discussion

There are a number of dermato-surgical techniques available to achieve repigmentation of stable vitiligo, such as suction blister grafting, follicular grafting,split-thickness punch grafting, skin grafting [10], cultured-melanocytes transplantation [11], and noncultured-melanocytes transplantation [12]. Every method has its own advantages and disadvantages. As there are no specific data or any prospective study available in literature, it is uneasy to recommend which surgical approach to vitiligo offers the best result. This randomized trial was done to compare two of the standard surgical procedures and a time tested procedure was compared to novel and upcoming procedure.

Tissue grafting techniques have traditionally been linked to various deficiencies in the form of cobblestoning, pigment mismatch, stuck on appearance, inadequate pigment cover and patient discomfort [13]. Thus, these days there is an increasing shift towards the use of advanced technology in the treatment of vitiligo and cellular transplant methods are favored over tissue transplants. Replenishing melanocytes selectively in depigmented macules by autologous melanocytes is one such promising treatment. Autologous melanocyte rich cell suspension transplant is essentially based on the principle of seeding of melanocytes i.e. transplantation of melanocytes from normal skin into a region of depigmented skin, where melanocytes are either damaged or destroyed completely [14]. The advantages of this technique over pre-existing treatment modalities are a small donor vs recipient skin ratio, no cobble-stoning, good color match and probably excellent cosmesis [15].

However, melanocyte transplantation procedure requires state of the art dermato-surgery facilities and has a steep learning curve. The procedure is expensive and is available across very few centres in the country. Epidermal sheet grafting on the other hand is a simple procedure and can be easily performed in resource poor setting with excellent patient satisfaction.

The biggest difference between the two methods was the donor and recipient skin area ratio. The donor and the recipient surface area was approximately the same in the epidermal grafting technique but about 1cm2 of normal skin could cover upto 10 cm2 of depigmented skin in the melanocyte transplant method, thus highlighting the gradual shift in the treatment strategies for surgical management of stable cases of vitiligo.

This was a comparative study of efficacy of epidermal tissue grafts versus melanocyte transplant in the management of stable vitiligo in 50 patches of stable vitiligo. Patients who satisfied the inclusion and exclusion criteria were enrolled for the study. The basic principle in both the transplant technique was replenishing normal

melanocytes to the melanocyte depleted areas. Further pigmentation was accelerated by photo-therapy. It seems likely that cytokines and growth factors released during wound healing phase also helps in the multiplication of transplanted melanocytes. Pigmentation in the treated areas gradually increases in size due to melanocyte proliferation and migration and increased melanin production under the influence of cytokines secreted by surrounding keratinocytes. Out of 50 patches operated upon, good results were seen in 64% patches by using melanocyte transplant method and 52% by using epidermal grafting method. Similarly fair results were seen in 20% patches both by epidermal split thickness transplant and melanocyte transplant method. Poor results were seen in 16% patches by melanocyte transplant method and 28% patches by epidermal transplant method.

Extensive search in literature did not yield any controlled study comparing these two standard modalities of therapy in stable vitiligo. As there were no randomized trials and to standardize the protocols for surgical management of stable vitiligo in our population this study was undertaken.

In our study there no statistical difference was found between the two groups, meaning that both the procedures were definitely beneficial to the patients. However the melanocyte transplant method did show slightly better cosmetic results.

Covering larger areas of skin with small donor tissue was the main advantage of the melanocyte transplant technique. However this method was more time consuming.

Limitations of both procedures

• Taking an epidermal graft requires expertise and has a learning curve.
• The pathogenesis of vitiligo is still poorly understood, so the stability of vitiligo and reactivation of disease activity in future after any surgical technique cannot be predicted as there are no objective markers to predict stability effectively.
• Patients with extensive vitiligo involving more than 20% of body surface area are not suited for any surgical technique.

Conclusion

In patients having stable vitiligo, surgical managemnt is an effective technique to produce homogeneous pigmentation as in these cases medical management alone is not effective. Both tissue and cellular transplant techniques are equally effective in obtaining re-pigmentation of vitiligo. Melanocyte transfer technique is time consuming and labour intensive technique which requires a sophisticated laboratory setup, though it can cover vitiliginous areas 10 times the donor area. It is therefore suitable to cover large body surface areas. It has a definite advantage over conventional epidermal split thickness grafting as it requires very little donor skin. We propose that, though the difference between the two grafting procedures was not statistically significant, the split thickness epidermal graft transfer technique method is simpler, less time consuming and requires less technical expertise. Hence epidermal graft transfer technique should be the first choice technique when it comes to not so widespread cases of vitiligo. When area involved is large, and in centers where

technical expertise and trained manpower are available, the melanocyte transplant technique can be undertaken. Patients were generally satisfied with the results to both methods, as patients had a fairly decent cosmetic outcome and both the procedures were day care OPD procedures. Further large scale randomized patient studies are required, especially with melanocyte transplant methods, to confirm the efficacy of autologous melanocyte transplant techniques as this is a novel technique and very few studies are documented in literature. The patients also need to be followed over a longer duration to note the long term complications and the status of re-pigmentation and stability of vitiligo after a period of time.

Funding: Nil, **Conflict of interest:** None initiated.
Permission from IRB: Yes

References

1. Parsad D. A new era of vitiligo research and treatment. J Cutan Aesthet Surg. 2013 Apr;6(2):63-4. doi: 10.4103/0974-2077.112664. [PubMed]

2. Löntz W, Olsson MJ, Moellmann G, Lerner AB. Pigment cell transplantation for treatment of vitiligo: a progress report. J Am Acad Dermatol. 1994 Apr;30(4):591-7. [PubMed]

3. Majid I. Grafting in vitiligo: how to get better results and how to avoid complications. J Cutan Aesthet Surg. 2013 Apr;6(2):83-9. doi: 10.4103/0974-2077.112668. [PubMed]

4. Khunger N, Kathuria SD, Ramesh V. Tissue grafts in vitiligo surgery - past, present, and future. Indian J Dermatol. 2009;54(2):150-8. doi: 10.4103/0019-5154.53196. [PubMed]

5. Majid I, Imran S. Ultrathin split-thickness skin grafting followed by narrowband UVB therapy for stable vitiligo: an effective andcosmetically satisfying treatment option. Indian J Dermatol Venereol Leprol. 2012 Mar-Apr;78(2):159-64. doi: 10.4103/0378-6323.93632.

6. Gauthier Y, Benzekri L. Non-cultured epidermal suspension in vitiligo: from laboratory to clinic. Indian J Dermatol Venereol Leprol. 2012 Jan-Feb;78(1):59-63. doi: 10.4103/0378-6323.90947. [PubMed]

7. Olsson MJ, Juhlin L. Leucoderma treated by transplantation of a basal cell layer enriched suspension. Br J Dermatol. 1998 Apr;138(4):644-8.

8. Pandya V, Parmar KS, Shah BJ, Bilimoria FE. A study of autologous melanocyte transfer in treatment of stable vitiligo. Indian J Dermatol Venereol Leprol. 2005 Nov-Dec;71(6):393-7.

9. Njoo MD, Westerhof W, Bos JD, Bossuyt PM. A systematic review of autologous transplantation methods in vitiligo. Arch Dermatol. 1998 Dec;134(12):1543-9. [PubMed]

10. Behl PN. Treatment of vitiligo with homologous thin Thiersch's graft. Curr Med Pract 1964:8:218-22. [PubMed]

11. Olsson MJ, Juhlin L. Repigmentation of vitiligo by

transplantation of cultured autologous melanocytes. Acta Derm Venereal (Stockholm) 1973;73:49-51. [PubMed]

12. Mulekar SV. Melanocyte- Keratinocyte cell transplantation for treatment of stable vitiligo. Int J Dermatol 2003;42:132-6. [PubMed]

13. Olson MJ, Juhlin L. Epidermal sheet grafts for repigments of vitiligo and piebaldism with review of surgical techniques. Acta Dermatol Venereol 1997;77:463-6. [PubMed]

14. Gauthier Y, Surleve-Bazeille JE. Autologous grafting with non-cultured Melanocytes: A simplified method for treatment of depigmented lesion. J Am Acad Dermatol 1992;26:191-4. [PubMed]

15. Lerner AB, Halaban R, Klavs SN. Transplantation of human melanocytes. J Invest Dermatol 1987;89:215-9.

How to cite this article?

Talukdar K, Mitra D. Randomized control trial to compare the efficacy of split thickness transplant versus autologous melanocyte transplant in the management of stable vitiligo. Int J Med Res Rev 2016;4(2):174-180. doi: 10.17511/ijmrr.2016.i02.009.

Refbacks

- There are currently no refbacks.
