# Supplementary material for: Therapeutic efficacy of cell-based therapy in vitiligo: a research letter systematically reviewed using meta-analysis
Source: Arch Dermatol Res. 2024 May 22;316(5):198. doi: 10.1007/s00403-024-02920-6 (PMC11111487; doi:10.1007/s00403-024-02920-6)
Supplement: Supplementary file 1 — Supplementary file1 (ZIP 24195 KB) [file 403_2024_2920_MOESM1_ESM.zip › Studies were included/van Geel 2004.pdf]

Double-blind Placebo-Controlled Study of Autologous Transplanted Epidermal Cell Suspensions for Repigmenting Vitiligo

Nanny van Geel, MD; Katia Ongenae, MD; Martine De Mil; Yves Vander Haeghen, PhD; Chris Vervaet, PhD; Jean Marie Naeyaert, MD, PhD

Objectives: To investigate the efficacy of epidermal noncultured cellular grafting in patients with vitiligo and the role of postinflammatory, spontaneous, or UV-induced pigmentation in obtaining repigmentation.

Design: A prospective, randomized, double-blind, placebo-controlled study.

Setting: Ambulatory patients in an institutional practice. Patients were followed up for 3 to 12 months.

Patients: A total of 33 paired, symmetrically distributed leukodermic lesions, all resistant to therapy, were observed in 28 patients. Nineteen patients appeared to have a stable vitiligo (group 1), whereas there was doubt about the stability of the disease in 9 patients (group 2).

Intervention: After laser ablation, a hyaluronic acid–enriched cellular graft was applied to 1 lesion while the paired lesion received placebo. Three weeks later all lesions were exposed to UV irradiation twice per week for approximately 2 months.

Main Outcome Measures: Primarily, the percentage of repigmentation was assessed after 3, 6, and 12 months using a digital image analysis system. The repigmentation pattern was also evaluated after 1 and 3 months.

Results: A strongly significant difference between cellular grafts and placebo was observed after 3, 6, and 12 months ($P<.001$, $P=.002$, and $P=.002$, respectively). In group 1, repigmentation of at least 70% of the treated area was achieved in 55%, 57%, and 77% of the actively treated lesions 3, 6, and 12 months after treatment, whereas in group 2 repigmentation of at least 70% of the treated area was not observed at any time point. The repigmentation pattern was diffuse in 94% of the responding patients.

Conclusions: After a strict preoperative selection for disease stability, transplantation resulted in repigmentation of at least 70% of the treated area in most actively treated vitiligo lesions. Repigmentation was primarily caused by the transplanted melanocytes.

Arch Dermatol. 2004;140:1203-1208

As one of the most prevalent pigment cell disorders of the skin, vitiligo is characterized by depigmented macules that may appear anywhere on the body, but mainly on sites of movement and friction such as wrists, elbows, knees, the periorificial regions, and the dorsum of hands and fingers. Approximately 1% of the world population experiences this disease, whose psychosocial impact is often underestimated.[1] Current conventional treatment consists of UV therapy (UV-B or psoralen plus UV-A [PUVA]) and local steroids. Although there are reports of some repigmentation with these treatments, the results have often been unsatisfying.[2,3] UV therapy lasts at least several months and causes physical discomfort, and the cumulative radiation dose limits its total use. Transplantation of autologous melanocytes is an additional option in patients with a stable vitiligo that no longer responds to conventional therapy. Several techniques have been tested and clinically introduced, with variable success.[4,5] Among these are split-thickness grafts, punch grafts, suction blister grafts, autologous cultured epithelial grafts, and autologous cultured melanocytes.

See also pages 1211 and 1273

In 1989, Gauthier and Surleve-Bazeille[6] introduced the use of noncultured cellular grafts, and they were followed by Ollson and Juhlin[7] a few years later. Major advantages of this technique are the possibility to treat larger skin areas with only a small piece of donor skin and the simple laboratory techniques involved. However, we found that the cellular suspension was impractical on curved areas, where it gave poorer results. In 2001 we published a pilot study describing a modified technique for transplanting noncultured

From the Department of Dermatology, Ghent University Hospital (Drs van Geel, Ongenae, Vander Haeghen, and Naeyaert and Ms De Mil), and the Laboratory of Pharmaceutical Technology (Dr Vervaet), Ghent University, Ghent, Belgium. The authors have no relevant financial interest in this article.

©2004 American Medical Association. All rights reserved.
Downloaded From: by a Trinity College Dublin User on 04/24/2018

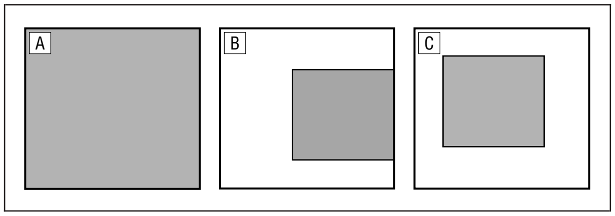

Figure 1. Zones where treatment or placebo was applied over the entire surface of a smaller lesion (A); part of a larger lesion, with lesion and treated area sharing a boundary line with normally pigmented skin (B); and part of a larger lesion, with no communication with normally pigmented skin (C).

cellular grafts.[8] The viscos ity of the cellular gaft was increased by addition of hyaluronic acid, a biodegradable cell carrier that improved adhesion of the graft to the dermabraded skin significantly. Using this method we obtained pigmentation in more than 80% of the treated area in the first 4 patients.[8] However, there are still many uncertainties about the exact cellular mechanisms that lead to repigmentation using transplantation of autologous cells to abraded skin. In 1990 Knoell and associates[9] mentioned repigmentation of small dermabraded depigmented macules that had been exposed to PUVA therapy. Several years later Özdemir and colleagues[10] reported repigmentation without additional UV therapy in 25% of lesions whose epidermis was removed by means of suction blisters. The hypothesis is that dermabrasion could be a melanocyte-stimulating trigger to a reservoir of melanocytes, ie, hair follicles and the surrounding skin. On the other hand the induced repigmentation might be postinflammatory, caused by the release of inflammatory mediators after epidermal trauma (dermabrasion, bullae, cryotherapy, or punch biopsy). Actually, these mediators could function as growth factors for melanocytes. Furthermore, cytokines can stimulate melanocyte migration, dendricity, and neomelanogenesis. We performed this first double-blind placebo-control study to judge the therapeutic value and evaluate the mechanism of action of this specific transplantation procedure.[11,12] To this end, the different parameters that may participate in the repigmentation process during and after transplantation using noncultured autologous cellular grafts were investigated.

METHODS

STUDY DESIGN AND RECRUITMENT

A randomized, double-blind, placebo-controlled study was performed at the department of Dermatology of Ghent University Hospital. Patient recruitment was performed between 1999 and 2002 and follow-up ranged from 3 to 12 months. The local ethics committee approved this project and all participants provided signed informed consent. Only patients at least 15 years old with a generalized and preferably stable vitiligo were included in the study. Stable disease was defined as no new lesions or expansion of preexisting lesions in the last 12 months (a positive regression was accepted). To investigate the influence of a possible disease activity we also included some patients whose vitiligo stability was questionable or with anamnestic indications for a Koebner phenomenon. Further analysis of these patients was performed separately (group 2). All patients had UV therapy in the past, without further improvement. Furthermore, all patients had a minimum of 2 lesions at the same anatomical localization (left vs right, or 2 separate lesions at the same anatomical location). Exceptionally, a second pair of lesions was selected at another location in the same patient. In general, for small lesions (<4 cm^2), the whole lesion was treated (**Figure 1** A), while in larger lesions (>4 cm^2), only a part of the depigmented macule was marked for treatment. Treatment was performed at the border of a vitiligo lesion and normally pigmented skin (Figure 1B), or in the center of a lesion (Figure 1C). We chose this study design to gain more insight in the pattern of repigmentation. Treatment with either a cellular suspension (melanocyte medium + hyaluronic acid + epidermal cells) or a placebo (melanocyte medium + hyaluronic acid) was randomized by a lottery system.

Neither patient nor investigator was informed about the randomization code. A nonblinded coworker (M.D.M) applied the suspension. At the time of removal of the bandage, 1 week later, the actively treated and the placebo-treated lesions could not be distinguished from each other.

TECHNICAL PROCEDURE

Donor Site

Using a hand dermatome, a shave biopsy specimen of approximately 2 cm^2 was taken from the patients' normally pigmented gluteal region under local anesthesia (lidocaine hydrochloride, 20 mg/mL, without epinephrine). The wound was covered with a transparent occlusive bandage (10 cm × 12 cm) and secured with gauze and adhesive tape.

Preparation of Cellular and Placebo Suspension

The donor skin specimen was transported to the laboratory in a medium containing Dulbecco modified Eagle medium supplemented with 40-µL/mL fetal bovine serum and 2-µL/mL RAID solution (10.000-U/mL penicillin-streptomycin, 5-mg/mL fungisone, and 40-mg/mL gentamicin) (GIBCO, Grand Island, NY). The specimen was then washed in phosphate-buffered saline solution (GIBCO) and torn to pieces. For dermo-epidermal separation the skin pieces were incubated in 0.25% trypsine and 0.08% wt/vol EDTA for 40 minutes at 37°C in a 10% carbon dioxide atmosphere. After incubation and subsequent addition of 5 mL of 10% fetal calf serum and 50 µL of 0.01-µg/mL basic fibroblast growth factor, the epidermis was separated from the dermis with fine forceps. The different pieces of skin were vortex-mixed for 45 seconds in a tube containing melanocyte medium (low calcium [0.03mM] M199 medium supplemented with 10-µg/mL insulin, 0.4-µg/mL hydrocortisone, 10 000-U/mL penicillin/streptomycin, and T/T (5-µg/mg transferrin plus 2×10^{-9}M triiodothyronine). This was centrifuged for 10 minutes at 120 g. The obtained pellet was resuspended in the melanocyte medium. Finally, hyaluronic acid (10-mg/mL hyaluronic acid [Provisc], Alcon Couvreur, Puurs, Belgium) was added in a 1:1 ratio to increase viscosity and obtain the gel.

The same quantity of melanocyte medium enriched with hyaluronic acid was used in the same ratio for the placebo suspension.

Recipient Site

On all test lesions 5-mg/g lidocaine and 25-mg/g prilocaine cream were applied for topical anesthesia (EMLA; AstraZeneca, Södertälje, Sweden) and remained under plastic occlusion for 1 hour. After disinfection with a solution of 0.5% chloramine, epidermal ablation was performed using a pulsed carbon dioxide laser set at 300 mJ and 200 pulses per second (Coherent UltraPulse; Parallax Technology Inc, Waltham, Mass). After the papillary dermis was reached the denuded lesions were treated with the active cellular suspension on one site and the placebo solution on the other by the nonblinded coworker. The lesion was covered with a sterile transparent occlusive dressing, dry gauze, and adhesive tape, and patients were asked to limit movement of the treated region for 3 to 7 days. One week later the bandage was changed and left in place for another week. Three weeks after the

©2004 American Medical Association. All rights reserved.
Downloaded From: by a Trinity College Dublin User on 04/24/2018

Patient and Disease Characteristics and Treatment Results*

Treatment No.	Patient No./Sex/ Fitzpatrick Skin Type/Age, y	Grafted Area	Size of Grafted Area, cm^2		Disease		Graft Repigmentation at 3/6/12 mo, %		Repigmentation Pattern
			Cellular	Placebo	Preoperative	Postoperative	Cellular	Placebo	
1	1/F/III/53	Trunk	1.1 (T)	2.7 (T)	S	S	63/63/93	0/0/0	D
2	2/M/III/19	Ankle	8.9 (T)	8.1 (P)	S	S	94/94/96	3/3/0	D
3	3/M/III/15	Ankle	3.9 (C)	3.4 (C)	S	S	41/53/0	0/0/0	D
4	4/F/III/53	Arms	6.4 (C)	6.8 (C)	S	S	91/87/93	45/61/12	D
5	5/M/III/58	Fingers	1.0 (P)	0.8 (P)	S	S	0/0/0	0/0/0	. . .
6	6/F/III/23	Arms	4.2 (C)	4.0 (C)	S	S	50/90/100	0/12/2	D
7	7/F/IV/31	Trunk	1.5 (T)	2.3 (T)	S	S	100/100/100	60/40/0	D
8	8/F/III/33	Hands	3.1 (C)	3.2 (C)	S	S	97/X/75	6/X/0	D
9	8/F/III/33	Legs	3.4 (P)	3.8 (P)	S	S	79/X/71	0/X/9	D
10	9/M/VI/28	Legs	1.2 (T)	1.1 (T)	S	S	X/X/X	X/X/X	. . .
11	10/F/II/17	Trunk	2.0 (P)	2.8 (P)	S	X	X/X/X	X/X/X	. . .
12	11/F/III/39	Wrists	3.5 (P)	5.2 (P)	S	S	96/100/98	3/3/0	D
13	11/F/III/39	Elbows	1.7 (T)	1.7 (T)	S	S	96/96/100	0/0/0	D
14	12/M/III/65	Trunk	2.6 (C)	3.1 (C)	S	S	0/0/0	15/0/0	. . .
15	13/F/II/38	Wrists	2.8 (P)	1.7 (P)	S	S	95/X/X	0/X/X	D
16	14/F/III/32	Trunk	1.1 (C)	1.4 (C)	S	S	96/X/X	0/X/X	D
17	15/M/III/22	Wrists	3.0 (P)	3.0 (P)	S	S	0/0/X	0/0/X	. . .
18	16/M/III/33	Feet	1.0 (T)	0.7 (T)	S	A	14/0/X	10/0/X	. . .
19	17/M/VI/44	Wrists	1.6 (C)	1.8 (P)	S	S	100/100/100	0/0/0	D
20	18/F/V/15	Legs	1.5 (T)	0.6 (T)	S	S	88/88/X	0/0/X	D
21	19/F/IV/30	Arms	1.6 (T)	1.8 (P)	S	K	0/X/X	0/X/X	. . .
22	19/F/IV/30	Legs	1.0 (P)	1.1 (P)	S	K	0/X/X	0/X/X	. . .
23	20/M/II/38	Hands	3.7 (T)	4.0 (T)	A	S	0/0/0	0/0/0	. . .
24	21/M/III/27	Feet	4.1 (T)	3.4 (T)	A	A + K	0/X/0	0/X/0	. . .
25	21/M/III/27	Hands	4.9 (P)	7.2 (P)	A	A + K	4/X/0	16/X/0	. . .
26	22/F/II/51	Legs	1.1 (T)	1.2 (T)	A	A	67/0/0	0/0/0	D
27	23/M/III/42	Fingers	0.2 (T)	0.6 (T)	A	A	0/0/0	0/0/0	. . .
28	24/M/II/17	Trunk	1.5 (P)	1.9 (P)	A	A	0/0/X	0/0/X	. . .
29	25/M/IV/50	Fingers	1.7 (T)	2.1 (T)	A	A	0/X/X	0/X/X	. . .
30	26/F/III/41	Arms	1.9 (C)	2.0 (C)	K	K	5/X/X	30/X/X	PF
31	27/M/IV/33	Elbow	6.2 (P)	5.2 (P)	K	K	0/0/0	0/0/0	. . .
32	28/F/III/39	Wrists	4.0 (P)	4.3 (P)	K	K	0/X/X	0/X/X	. . .
33	28/F/III/39	Legs	5.7 (T)	4.4 (T)	K	K	0/X/X	0/X/X	. . .

Abbreviations: A, active disease; C, center of lesion; D, diffuse; K, Koebner phenomenon; P, partial lesion; PF, perifollicular; S, stable disease; T, total lesion; X, no evaluation performed.

*Treatments 1 through 22 represent group 1 (patients with stable vitiligo and no Koebner phenomenon preoperatively); treatments 23 through 33 represent group 2 (patients with doubtful vitiligo stability and/or possible presence of Koebner phenomenon preoperatively). Ellipses indicate that no repigmentation occurred.

procedure all patients began to receive UV irradiation (UV-B [311 nm] or PUVA twice per week for approximately 2 months to stimulate melanocyte proliferation: for UV-B, starting with 0.1 J/cm^2 and increasing by 0.1 J/cm^2 each session until a slight erythema appeared; for UV-A, starting with 0.5 J/cm^2 and increasing by 0.5 J/cm^2 each session until a slight erythema appeared).

Outcome Evaluation

The main parameter to measure the efficacy of treatment was the percentage of area of repigmentation in the test lesions. This percentage was objectively assessed with an area measurement on the day of treatment and 3, 6, and 12 months after treatment.

To obtain an accurate estimation of the size of the vitiligo lesions, the contours of all test lesions were first traced on transparent sheets. This had the advantage of taking the original curvature into account, thereby avoiding possible underestimation of the lesion surface caused by the move from 3 to 2 dimensions. These sheets were then scanned at a predefined resolution, and analyzed using a digital image analysis software (Matlab; Mathworks, Inc, Natik, Mass). The reproducibility of this method (scanning, segmentation, and surface estimation) for a plane surface is very good, with a maximun error of 2% between consecutive measurements of the same area. The accuracy of the measurement is of the same order, and is suitable for the evaluation of small surfaces and lesions with well-defined borders.

Other aspects of the repigmentation process were also analyzed. Special attention was paid to the repigmentation pattern, ie, whether it was diffuse, perifollicular, or migrating from the borders. This was evaluated 1 and 3 months after treatment. The colors of the treated area and the surrounding normally pigmented skin were subjectively compared.

STATISTICAL ANALYSIS

For repigmentation we evaluated changes within groups using a Wilcoxon signed rank test, whereas differences between groups were statistically evaluated using the Mann-Whitney test. A *P* value of .05 or less indicated statistical significance. To reach statistical significance a sample size of at least 20 patients was recommended.

RESULTS

PARTICIPANTS

A total of 28 patients (14 women and 14 men) with generalized vitiligo were included. Their mean age was 35.2

©2004 American Medical Association. All rights reserved.
Downloaded From: by a Trinity College Dublin User on 04/24/2018

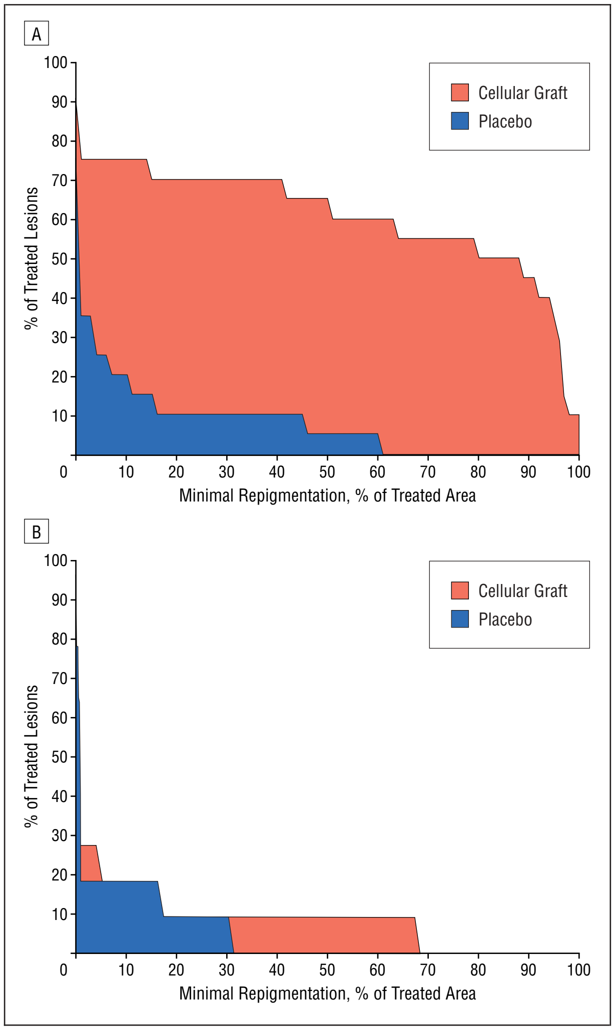

Figure 2. Area of repigmentation at 3 months in group 1 (A) and group 2 (B).

years (range, 15-65 years). In 19 patients (67%) all criteria for an anamnestically and clinically stable vitiligo were fullfilled (group 1). However, 9 patients (33%) had clinical or anamnestic indications for disease activity (new lesions or enlargement of old ones in the past year, and/or presence of the Koebner phenomenon) (group 2). Vitiligo was present during a mean of 144 months (range, 18-324 months). Twenty-five patients (89%) had Fitzpatrick skin type II, III, or IV (**Table**). The mean extension of leukoderma assessed with the rule of 9 was about 4% of the total body surface area (range, 0.2%-20.0%). In 23 patients (82%) only 1 pair of lesions was selected and in 5 patients (18%) 2 pairs were selected. In total, 66 lesions were treated. The mean surface area of the actively treated lesions was 2.8 cm^2 (range, 0.2-8.9 cm^2), while the mean surface of the placebo-treated lesions was 2.9 cm^2 (range, 0.6-8.1 cm^2). In this study lesions were often chosen in cosmetically less important body regions, which often include areas difficult to treat. Lesion distribution was as follows: 18 (27%) were at the joints (wrists, elbows, and ankles), 12 (18%) on the trunk, 12 (18%) on the legs, 10 (15%) on the feet or dorsum of the hands, 8 (12%) on the arms, and 6 (9%) on the fingers. The complete surface of the depigmented macule was selected for treatment in 26 lesions (39%), while in 40 lesions only a part of the depigmented macule was marked for treatment (at the border in 25 lesions [38%] and in the center in 15 lesions [23%]). Twenty-six patients (82%) were followed up for at least 3 months. Eighteen patients (64%) and 16 patients (57%) could only be evaluated at 6 and 12 months of follow-up, respectively. In 1 patient there was doubt about the original location of the test lesions, so that no correct evaluation of the therapy could be performed; and 1 patient left the country and was lost to follow-up.

POSTTREATMENT CLINICAL COURSE

Erythema was observed in all test lesions during the first 2 weeks, then changed to a pink color that persisted for a maximum of 1 month. The first signs of repigmentation were observed 3 weeks after transplantation. In 5 responding lesions hyperpigmentation was observed for approximately 6 months. In all other responding lesions, color matching was immediately good. No scars were observed in the recipient areas.

EFFICACY OUTCOME

At 3 observed time points (3, 6, and 12 months) there was a significant difference in repigmentation ($P<.001$, $P=.002$, and $P=.002$, respectively) between actively treated lesions and placebo-treated lesions (**Figures 2**, **3**, **4**, **5**, **6**, and **7**). At 3, 6, and 12 months the the median percentages of the repigmented areas at the actively treated site in group 1 were 84%, 88%, and 93%, respectively (means, 60%, 62%, and 71%), and repigmentation in 70% or more of the treated aeras occurred in 55%, 57%, and 77% of actively treated lesions at the same time points. In group 2 the median was 0% at all time points (means, 7%, 0%, and 0%), and no actively treated lesion achieved repigmentation in 70% or more of the treated area.

Repigmentation at the placebo-treated site was limited, as only patients 4, 7, and 26 showed repigmentation in at least 20% of the area (Table). In 2 of these 3 patients the percentage of area of repigmentation was lower than at the actively treated site, and was decreasing toward the end of the follow-up period. This finding suggests postinflammatory pigmentation. In the third patient we observed a spectacular generalized perifollicular repigmentation after initiation of UV-B therapy. Repigmentation was perifollicular and not diffuse at both the actively treated and placebo sites. In the past this patient had only received PUVA therapy, which raises the suspicion that this repigmentation was UV-B induced.

DISEASE ACTIVITY AND KOEBNER PHENOMENON AFTER TREATMENT

In 1 patient (patient 21) the Koebner phenomenon was accompanied by vitiligo activity after treatment. Of 27 patients, 6 (22%) had active disease after treatment. In all of those, except in patient 16, this was correctly assessed at the day of treatment. One of these (16%) exhibited the Koebner phenomenon. When we divide the patients in groups according to these posttreatment findings (group A, patients with stable vitiligo and no Koebner phenomenon at donor site after treatment; group B,

©2004 American Medical Association. All rights reserved.
Downloaded From: by a Trinity College Dublin User on 04/24/2018

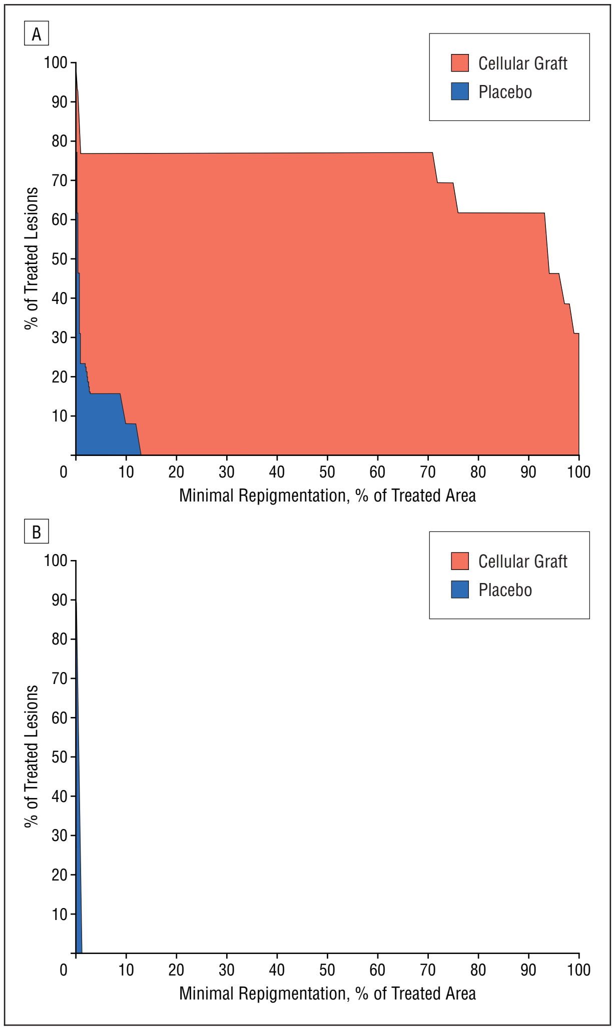

Figure 3. Area of repigmentation at 12 months in group 1 (A) and group 2 (B).

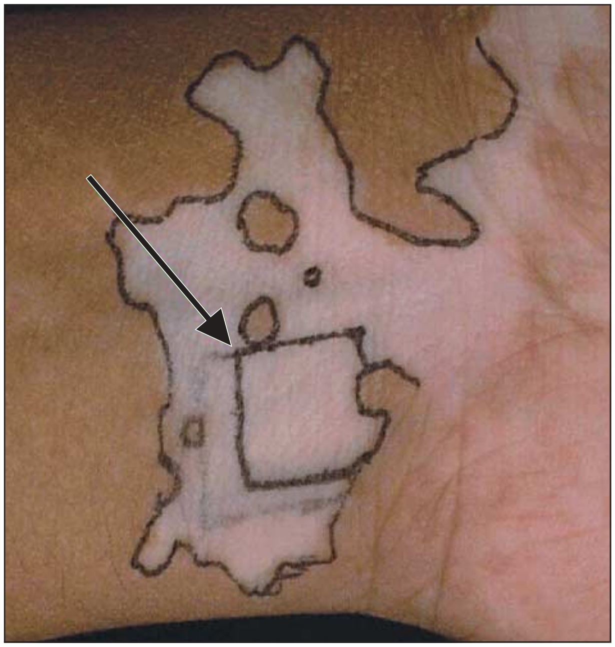

Figure 4. Test site (arrow) (left wrist) before cellular grafting.

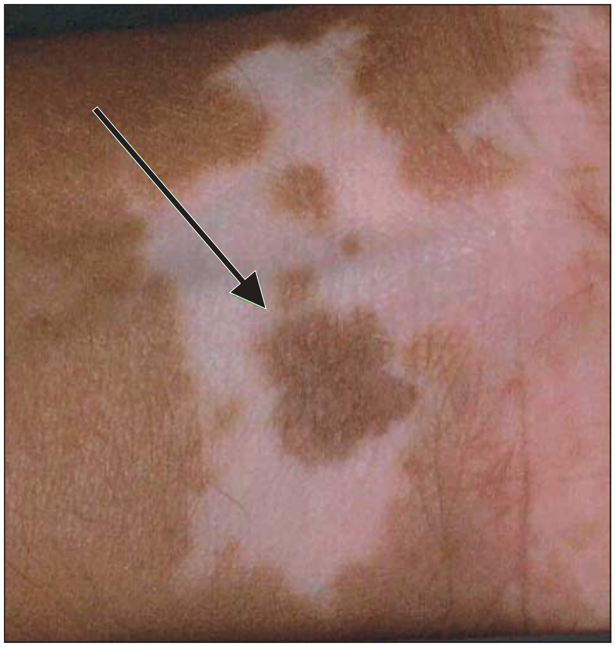

Figure 5. Test site (arrow) (left wrist) 3 months after cellular grafting.

patients with active vitiligo after treatment and/or a posttreatment Koebner phenomenon), results are as follows: the median area percentages of repigmentation at actively treated lesions in group A were 88%, 88%, and 93% (means, 62%, 62%, and 66%) 3, 6, and 12 months after treatment, respectively. In group B the median was 0% at all time points (means, 8%, 0%, and 0%).

REPIGMENTATION PATTERN

Except in 1 patient, all responding lesions showed a diffuse repigmentation pattern rather than a typically perifollicular pattern or repigmentation from the perilesional margins (Table). This interesting finding was especially obvious when looking at the hairless test lesions and lesions not bound or only partially bound by normally pigmented skin (Figures 1 B and C, 4, and 5).

COMMENT

Vitiligo has been treated using conservative therapy and/or transplantation techniques, with variable success.[13,14] Transplantation of autologous noncultured epidermal cell suspensions has the major advantage of treating larger areas (up to 10 times the donor area) without the need for cell cultures.

However, in the absence of an objective evaluation of the efficacy of this treatment, this prospective, double-blind, randomized, placebo-control study was designed to help solve this question. Thirty-three lesions were treated with an active cellular suspension enriched with hyaluronic acid, while 33 symmetrically located lesions received a placebo. An evaluation of repigmentation was objectively performed using a digital image analysis system.

A significant difference in the results of treatment with cellular grafts and placebo was observed. Results were slightly inferior to our previous findings in the actively

©2004 American Medical Association. All rights reserved.
Downloaded From: by a Trinity College Dublin User on 04/24/2018

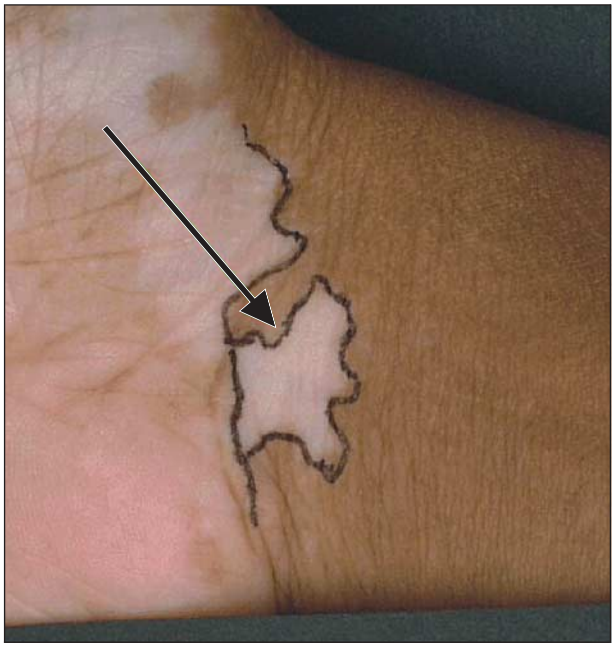

Figure 6. Test site (arrow) (right wrist) before application of placebo.

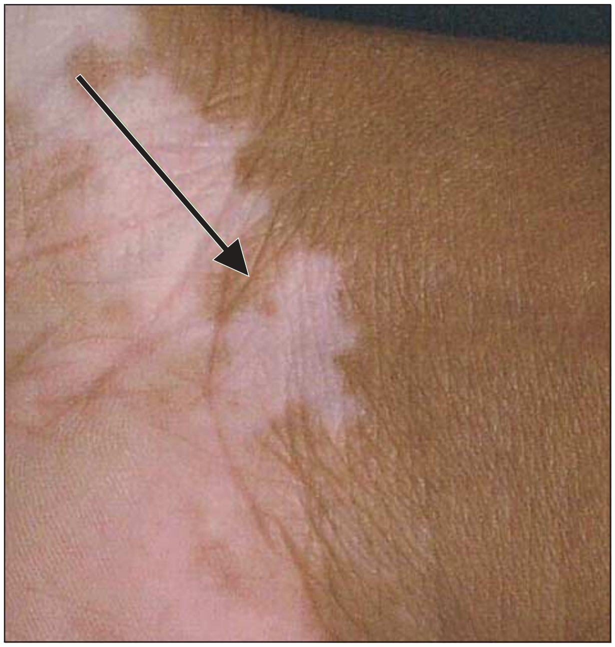

Figure 7. Test site (arrow) (right wrist) 3 months after application of placebo.

treated lesions, but because of ethical, aesthetic, and scientific reasons the lesions in this study were often chosen at sites relatively difficult to treat, eg, at finger joints, ankles, and wrists.[8]

As the posttreatment clinical course at the recipient site was only characterized by minimal signs of erythema and a temporary hyperpigmentation, the use of autologous epidermal cell suspensions may also be considered a safe therapeutic option in treating vitiligo.

To assess the mechanism of action of the repigmentation process, we looked at the repigmentation pattern after treatment. In more than 90% of cases, repigmentation occurred in a diffuse pattern. Especially in hairless lesions and lesions clinically not bound by normally pigmented skin, this pattern indicates that repigmentation originates from the transplanted cellular suspension. We also showed that in placebo-treated lesions repigmentation induced by epidermal ablation, UV therapy, and inflammatory mediators is extremely limited.

A strict selection of the patients is of major importance for the outcome, as active disease and the presence of the Koebner phenomenon negatively influence treatment results. A longer pretreatment observation time or a minigrafting test may therefore be indicated in some cases.[15]

Accepted for publication February 15, 2004.

This study was supported by Ghent University Bijzonder Onderzoeksfonds grant 01108101 (Dr van Geel) and Fonds Wetenschappelijk Onderzoek (Dr Ongenae), Ghent, Belgium.

We thank Dirk De Bacquer, PhD, for the statistical analysis of the results and Alcon Couvreur for supplying the hyaluronic acid.

Correspondence: Jean Marie Naeyaert, MD, PhD, Department of Dermatology, Ghent University Hospital, De Pintelaan 185, 9000 Ghent, Belgium (Jeanmarie.naeyaert@UGent.be).

REFERENCES

1. Kent G, al-Abadie M. Factors affecting responses on Dermatology Life Quality Index items among vitiligo sufferers. *Clin Exp Dermatol.* 1996;21:330-333.
2. Njoo MD, Spuls PI, Bos JD, Westerhof W, Bossuyt PM. Nonsurgical repigmentation therapies in vitiligo: meta-analysis of the literature. *Arch Dermatol.* 1998;134:1532-1540.
3. Taneja A. Treatment of vitiligo. *J Dermatolog Treat.* 2002;13:19-25.
4. van Geel N, Ongenae K, Naeyaert JM. Surgical techniques for vitiligo: a review. *Dermatology.* 2001;202:162-166.
5. Yaar M, Gilchrest BA. Vitiligo: the evolution of cultured epidermal autografts and other surgical treatment modalities. *Arch Dermatol.* 2001;137:348-349.
6. Gauthier Y, Surleve-Bazeille JE. Autologous grafting with noncultured melanocytes: a simplified method for treatment of depigmented lesions. *J Am Acad Dermatol.* 1992;26:191-194.
7. Olsson MJ, Juhlin L. Leucoderma treated by transplantation of a basal cell layer enriched suspension. *Br J Dermatol.* 1998;138:644-648.
8. van Geel N, Ongenae K, De Mil M, Naeyaert JM. Modified technique of autologous noncultured epidermal cell transplantation for repigmenting vitiligo: a pilot study. *Dermatol Surg.* 2001;27:873-876.
9. Knoell KA, Schreiber AJ, Milgraum S. Treatment of vitiligo with the ultrapulse carbon dioxide laser in patients concomitantly receiving oral psoralen plus UV-A therapy. *Arch Dermatol.* 1997;133:1605-1606.
10. Özdemir M, Cetinkale O, Wolf R, et al. Comparison of two surgical approaches for treating vitiligo: a preliminary study. *Int J Dermatol.* 2002;41:135-138.
11. Ongenae K, van Geel N, Naeyaert JM. Autologous cellular suspensions and sheets in the treatment of achromic disorders: the need for future controlled studies. *Dermatology.* 2001;202:158-161.
12. van Geel N, Ongenae K, De Mil M, Naeyaert JM. The first prospective double blind placebo controlled study of autologous non-cultured epidermal cell transplantation for repigmenting leukoderma [abstract]. *Pigment Cell Res.* 2002;15(suppl 9):S45.
13. Njoo MD, Westerhof W. Vitiligo: pathogenesis and treatment. *Am J Clin Dermatol.* 2001;2:167-181.
14. Falabella R. What's new in the treatment of vitiligo. *J Eur Acad Dermatol Venereol.* 2001;15:287-289.
15. Falabella R, Arrunategui A, Barona MI, Alzate A. The minigrafting test for vitiligo: detection of stable lesions for melanocyte transplantation. *J Am Acad Dermatol.* 1995;32:228-232.

©2004 American Medical Association. All rights reserved.
Downloaded From: by a Trinity College Dublin User on 04/24/2018
