# Supplementary material for: Therapeutic efficacy of cell-based therapy in vitiligo: a research letter systematically reviewed using meta-analysis
Source: Arch Dermatol Res. 2024 May 22;316(5):198. doi: 10.1007/s00403-024-02920-6 (PMC11111487; doi:10.1007/s00403-024-02920-6)
Supplement: Supplementary file 1 — Supplementary file1 (ZIP 24195 KB) [file 403_2024_2920_MOESM1_ESM.zip › Studies were included/Verma 2014.pdf]

Available online at www.sciencedirect.com

SciVerse ScienceDirect

journal homepage: www.elsevier.com/locate/mjafi

Original Article

A comparative study of efficacy of cultured versus non cultured melanocyte transfer in the management of stable vitiligo

Col Rajesh Verma [a], Brig R.S. Grewal, VSM [b], Col Manas Chatterjee [c], Lt Col Vijendran Pragasam [d], Lt Col Biju Vasudevan [d,*], Sqn Ldr Debdeep Mitra [e]

[a] Professor & Senior Advisor, Department of Dermatology, Command Hospital (Southern Command), Pune 411040, India
[b] Dy DGAFMS (Prov), O/o DGAFMS, New Delhi, India
[c] Senior Advisor (Dermatology), Command Hospital (Eastern Command), Kolkata, India
[d] Classified Specialist (Dermatology), Command Hospital (Southern Command), Pune 411040, India
[e] Resident (Dermatology), Command Hospital (Southern Command), Pune 411040, India

ARTICLE INFO

Article history:
Received 24 April 2013
Accepted 12 September 2013
Available online 20 November 2013

Keywords:
Autologous melanocyte transplantation
Melanocyte rich cell suspension
Melanocyte culture
Vitiligo

ABSTRACT

Background: Replenishing melanocytes by autologous melanocytes selectively in vitiliginous macules is a novel and promising treatment. With expertise in culturing autologous melanocytes, it has now become possible to treat larger recipient areas with smaller skin samples. To determine the relative efficacy of cultured versus non cultured melanocyte transfer in the management of stable vitiligo.

Methods: The melanocytes were harvested as an autologous melanocyte rich cell suspension from a donor split thickness graft. Cultured or non cultured melanocytes were then transplanted to the recipient area that had been superficially dermabraded. 100 patches of vitiligo in patients reporting to this hospital were randomly allocated into 2 groups to receive either of the interventions.

Results: An excellent response was seen in 62.17% cases with the autologous melanocyte rich cell suspension technique and in 52% with the melanocyte culture technique.

Conclusion: Autologous melanocyte transplantation can be an effective form of surgical treatment in stable but recalcitrant lesions of vitiligo. Large areas of skin can be covered with a smaller donor skin using melanocyte culture technique; however culture method is more time consuming, and a labour intensive process, requiring state of the art equipments with a sterile lab setup.

© 2013, Armed Forces Medical Services (AFMS). All rights reserved.

* *Corresponding author*. Tel.: +91 7798225557.
E-mail address: biju.deepa@rediffmail.com (B. Vasudevan).

0377-1237/$ – see front matter © 2013, Armed Forces Medical Services (AFMS). All rights reserved.

http://dx.doi.org/10.1016/j.mjafi.2013.09.004

Introduction

Vitiligo is a pigmentary disease of unknown cause, which is characterized by depigmented or hypopigmented macules which results from absence or reduction in the number of epidermal melanocytes in skin and/or mucous membranes. It has immense socio-psychological ramifications in addition to its cosmetic disability. Mode of therapy is based on decreasing the activity, thereby achieving stability and later inducing pigmentation. Many a times, medical therapy, alone is not helpful. The vitiliginous areas may remain static without showing any repigmentation or depigmentation. Such type of patients who are stable for more than 1 year duration are considered suitable for surgical treatment options including transfer of autologous melanocytes.[1]

Replenishing autologous melanocytes selectively within vitiliginous macules is a novel and promising treatment.[2] This can be carried out by either culture or non culture techniques, each having its advantages and disadvantages.[3,4] We undertook this study to compare the two methods of melanocyte transplantation in stable vitiligo, namely melanocyte rich cell suspension and cultured melanocyte transfer for replenishing melanocytes.

Material and methods

This was a comparative study wherein 50 unresponsive sites, each were operated upon by the 2 modalities, i.e. cultured melanocyte transfer and non cultured autologous melanocyte transfer technique leading to a total of one hundred sites in all, over a period of 1 year. The 100 patches of vitiligo were randomized based on simple random sampling method. Only cases with stable form of vitiligo (no increase in the size of the lesion for at least 1 year) and with a maximum percentage of body surface area involvement up to 30% were included in the study. The pigmentation was compared to the baseline after 6 months post procedure. No blinding was done in the study.

Sample size determination was done as follows: $(1 - \alpha) = 0.98$, $(1 - \beta) = 0.80$, $\pi1 = 0.81$, $\pi2 = 0.50$, $\delta = 0.1$and therefore $n = 36$. Though the calculated sample size was 36, 50 unresponsive sites were considered for surgery by each of the modalities, leading to a total of one hundred sites in all. Pre operative work-up consisted of an informed consent, clinical photographs, screening for HIV and Hepatitis B virus infection and charting of the area to be grafted.

Two techniques were employed, the autologous melanocyte rich cell suspension (non cultured melanocyte) technique (NCMT technique)[5–7] and the cultured melanocyte technique (CMT technique).[8–10] Both these techniques share a common principle of selective replenishment of melanocytes at the recipient stable vitiligo macules.

Donor site

About one-tenth the size of the recipient area was selected as the donor site, usually on non-cosmetically important sites like the medial aspect of thighs. It was cleaned and draped. The site was anesthetized and a very superficial sample of skin was obtained using Silver's skin grafting knife. The superficial wound was then dressed with Sofra-tulle.

Laboratory procedure for cell separation

The skin graft was immediately transferred to 6 ml of 0.25% trypsin-EDTA solution in a petridish. This mixture of skin sample with trypsin-EDTA solution was incubated at 37 °C for 50 min. The grafts were then transferred into a petridish containing 8 ml of melanocyte nourishment medium i.e. Dulbecco's modified eagle medium/F12-(DMEM). This media also acted as a diluting agent to wean off the trypsin action. All the subsequent steps were performed in a laminar air flow bench under strict aseptic conditions. The epidermis was teased gently and separated from the dermis with forceps. The dermal pieces were discarded and the epidermal pieces were retained. The epidermal pieces were scraped, so that they did not have any pigment left on their surface. The contents of the petridish were transferred into a centrifuge tube and centrifuged for 6 min at 3000 rpm. The cell pellet settled down at the bottom. The supernatant was discarded and the pellet, containing cells from the stratum basale and lower half of the stratum spinosum that were rich in melanocytes, was taken. The pellet was resuspended in a total volume of 0.8 ml DMEM medium and transferred gently in steps to a syringe.

Recipient site (vitiliginous area)

The vitiliginous areas were dermabraded down to the papillary dermis with a diamond fraise wheel after surgical cleaning and infiltration of local anaesthesia. The cell suspension was applied evenly on the denuded area and spread uniformly with spatula. The areas were covered with a collagen dressing and later with sterile gauze pieces moistened with DMEM/F12 and held in place by Tegaderm transparent dressing. Patient was made to lie down for 30 min (elevation of part if required – foot) and then allowed to leave with the instructions to avoid vigorous activities and to carry out only restricted movements for next 7 days.

Post operative care

Oral antibiotics and analgesics were given for 5 days. Dressing of donor area was changed on alternate days and for the recipient areas, it was removed after 7 days. PUVASOL (1:10) was added for accelerating the repigmentation and was started 2 weeks after the erythema subsided. The patients were followed up at 1, 3 and 6 months after procedure for assessing repigmentation.

CMT group

Patients in this group received treatment in the form of autologous, cultured, melanocyte plus keratinocyte grafting followed by topical Psoralen therapy. The initial steps were similar to the ones followed during autologous, non cultured, melanocyte plus keratinocyte grafting (NCMT group), till the formation of a cell suspension pellet. This cell suspension was cultured in tissue culture flasks along with 05 ml of M2

medium which is F12 medium supplemented with bFGF (20 ng/ml), isobutyl methyl xanthine (0.1 mM), cholera toxin (10 ng/ml), glutamine 2 mM and 10% foetal bovine serum (serum free tetradecanoylphorbol acetate). This was incubated at 37 °C in 95% air and 5% CO_2 atmosphere (using anaerobic gas chargers) in an incubator for a total of 21 days. The tissue culture fluid was replaced on a daily basis. Part of the cultured cell suspension which was obtained after 21 days was stained with Trypan blue stain and the density of melanocytes was counted under a microscope using a Neubaeur's chamber. A density of 1000–2000 melanocytes/mm^2 was to achieved before transplantation. The cultured grafts were transferred to a petridish, containing 8 ml of DMEM media and thereafter centrifuged at 3000 rpm for 6 min. The cell suspension pellet was obtained and the subsequent steps were similar to the NCMT group. The cell suspension was then applied over the dermabraded vitiliginous recipient area.

The patients were called after 1 month, 3 months and 6 months to assess the extent of repigmentation. Photographs were taken and the observations were tabulated. The response was graded according to the extent of repigmentation in transplanted areas as follows – good: >70% repigmentation, fair: 30%–69% repigmentation, poor: below 30% repigmentation.

Results

A total of 25 patients were enrolled in the study, which included 50 sites of depigmentation for autologous non culture melanocyte–keratinocyte transfer (NCMT) and 50 sites for autologous cultured melanocyte transfer (CMT). In the NCMT group 19 patients were enrolled with 50 unresponsive depigmented sites. 10 were males (52.6%) and 9 (47.4%) were females. In the CMT group 6 patients with 50 unresponsive depigmented sites were enrolled. 3 (50%) were male patients and 3 (50%) were females. In the NCMT group, minimum duration of vitiligo was 1 year and maximum duration was 14 years; mean duration was 7.00 ± 3.43 years. In CMT group, minimum duration of vitiligo was 3 years and maximum duration was 14 years; mean duration was 7.83 ± 4.45. Demographically both the groups were comparable.

Among the study groups vitiligo vulgaris was present in 17 (68%) patients, localized vitiligo in 3 (12%), segmental in 2 (8%), mucosal in 2 (8%) and 1 (4%) patient had acrofacial vitiligo.

Among the patients with vulgaris type vitiligo, NCMT was done on 11 (64.7%) patients with 34 (68%) patches and CMT was done in 6 (35.3%) patients with 50 (100%) patches.

Two (10.5%) patients with 3 (6%) patches in NCMT group had mucosal vitiligo. Localized vitiligo was present in 3 (15.8%) patients with 3 (6%) patches. Two (10.5%) patients with 7 (14%) patches in MT group had segmental vitiligo. Acrofacial vitiligo was operated upon in 1 (5.3%) with 1 (2%) patch. All (100%) the patients in the CMT group had vitiligo vulgaris.

Post operative evaluation

At first follow up, soon after removal of dressing from the treated area, crusted scabs were seen partially attached to the skin surface leaving behind erythematous achromatic area.

Infection was noted at one of the sites following NCMT technique and 5 sites following CMT technique; all of which resolved within a week of oral antibiotics without any scarring. After 6 months of procedure, percentage of repigmentation was calculated for both the groups and the same has been depicted in Table 1.

- In the NCMT method, 31 (62%) patches showed good repigmentation, i.e. more than 70% repigmentation [Figs. 1 and 2], 10 (20%) patches showed fair repigmentation, i.e. 30–69% repigmentation, 9 (18%) patches showed poor repigmentation, i.e. less than 30% repigmentation.
- In the CMT method, 26 (52%) patches showed good repigmentation, i.e. more than 70% repigmentation [Fig. 3], 5 (10%) patches showed fair repigmentation, i.e. 30–69% repigmentation, 19 (38%) patches showed poor repigmentation, i.e. less than 30% repigmentation.

The optimum time for successful culture was 3 weeks. At the end of 3 weeks, the cell count was raised 50–100 folds after primary culture and subculture. The melanocyte content of these cultures was more than 1000 cells/mm^2 and no difference was noted in the repigmentation process in the two methods. By using Chi-square test *p*-value for the degree of repigmentation noted after 6 months was 0.058, which was >0.05; therefore there was no statistical association between treatment methodology and repigmentation. So the final outcome i.e. repigmentation is independent of the modality of treatment. However in patients of the NCMT group, more patches showed good results as compared to the CMT group, though they were not statistically significant.

By both the methods, patches over face, lips, trunk and legs showed good repigmentation, however patches over acral areas and bony prominences had poor repigmentation.

Complications

One patient in the NCMT group with three patches and one patient in the CMT group with ten patches, after the procedure noted a reactivation of their disease process. Both the donor and recipient areas were depigmented after 1 month of the procedure. Even after 6 months the patches remained depigmented. One patient noted erythema and one patient noted pain, redness and pus discharge at the donor site in the NCMT group and one patient in the CMT group noted redness and pus discharge at donor site and few of the recipient sites. The infection was controlled with 1 week of oral and topical antibiotics.

The consort flow diagram based on the 2010 updated guidelines for the study is as given in Fig. 4.[11]

Table 1 – Percentage of repigmentation.

Repigmentation	NCMT	CMT	*p*-value
Poor (≤30%)	9	19	0.058
Fair (31%–70%)	10	5	
Good (>70%)	31	26	
Total	50	50	

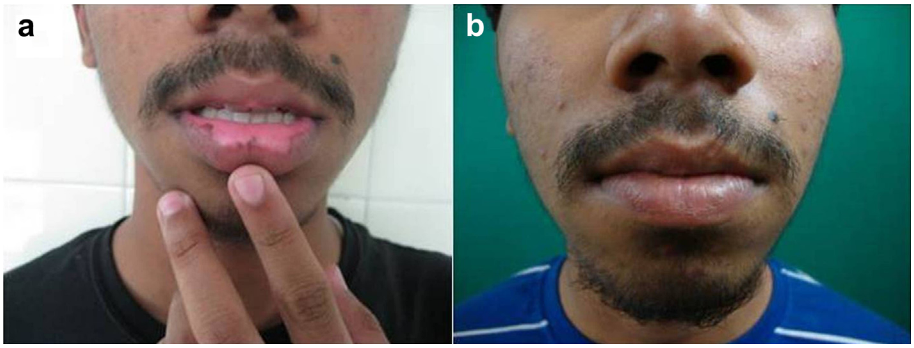

Fig. 1 – Non culture melanocyte transplant – Lip vitiligo (a) Pre procedure (b) 6 months after procedure.

Discussion

There are many existing dermatosurgical procedures for treatment of stable vitiligo. These include suction blister grafting, punch grafting, split thickness skin grafting, tattooing, needling and plain dermabrasion. However they have various deficiencies in the form of cobblestoning, pigment mismatch, stuck on appearance, inadequate pigment cover and patient discomfort. Thus, there is an increasing trend towards the use of advanced technology in the treatment of this condition. Replenishing melanocytes selectively in vitiliginous macules by autologous melanocytes is one such promising treatment. Both, the autologous melanocyte rich cell suspension (non cultured) technique and the cultured melanocyte technique, are essentially based on the principle of seeding of melanocytes i.e. introduction of melanocytes from normal skin into a region of depigmented skin. The distinctive advantages of this technique over pre-existing modalities are a wide recipient area for small donor area, no cobblestoning, good colour match and probably the best efficacy.

Melanocyte culture is a state of the art procedure that requires more expertise as compared to the NCMT technique. This was a comparative study of efficacy of cultured versus non cultured melanocyte transfer in the management of stable vitiligo in 100 patches of stable vitiligo, satisfying the inclusion and exclusion criteria.

It is an accepted fact that during the initial 7 days of healing, melanocytes and keratinocytes present in the grafted material gets entrapped in the healing recipient site tissue. These cells multiply and repigment the depigmented area. Further pigmentation can be accelerated by topical Psoralen therapy. It seems likely that growth factors and cytokines released during wound healing phase helps in the transplantation and multiplication of melanocytes.

Pigmentation in the treated areas gradually increases in size due to melanocyte proliferation and migration under the

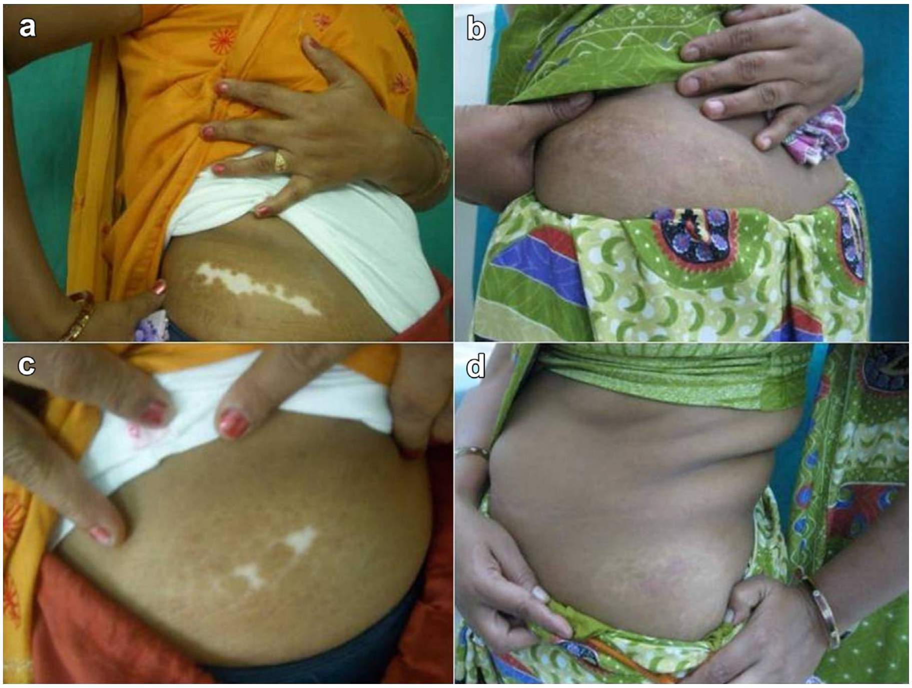

Fig. 2 – Non culture melanocyte transplant – Bilateral hip vitiligo (a) Right hip – Pre procedure (b) Right hip – 6 months after procedure (c) Left hip – Pre procedure (d) Left hip – 6 months after procedure.

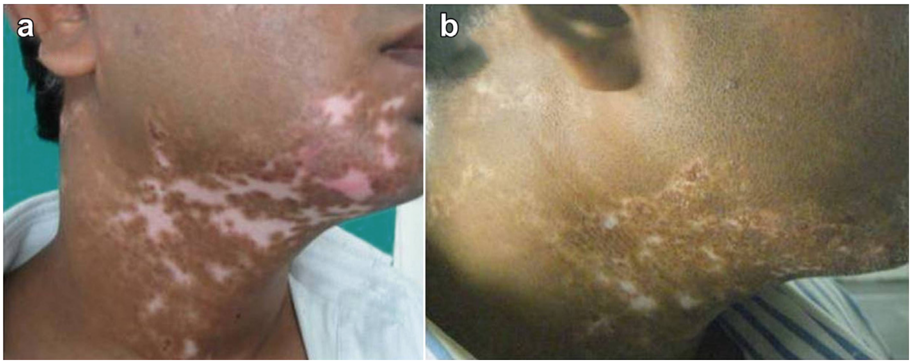

Fig. 3 – Culture melanocyte transplant – Segmental vitiligo: right side of neck (a) Pre procedure (b) 6 months after procedure.

influence of cytokines secreted by surrounding keratinocytes. Hence, melanocytes taken from a small donor area can pigment a much larger recipient area.

Out of 100 patches operated upon, good results were seen in 62% patches by using NCMT method and 52% by using CMT method. Similarly fair results were seen in 20% patches by NCMT method and 10% patches by CMT method. Poor results were seen in 18% patches by NCMT method and 38% patches by CMT method.

The only study from India comparing cultured and non cultured melanocyte transfer demonstrated 50% improvement results in both groups, with no difference between the two groups.[5] However, this study recruited very few depigmented sites. In view of the fact that this study did not show any difference between cultured and non cultured melanocyte transfer, it was necessary to rigorously study these two modalities of cellular transfer therapy of vitiligo in a larger number of areas of depigmentation to confirm or refute these

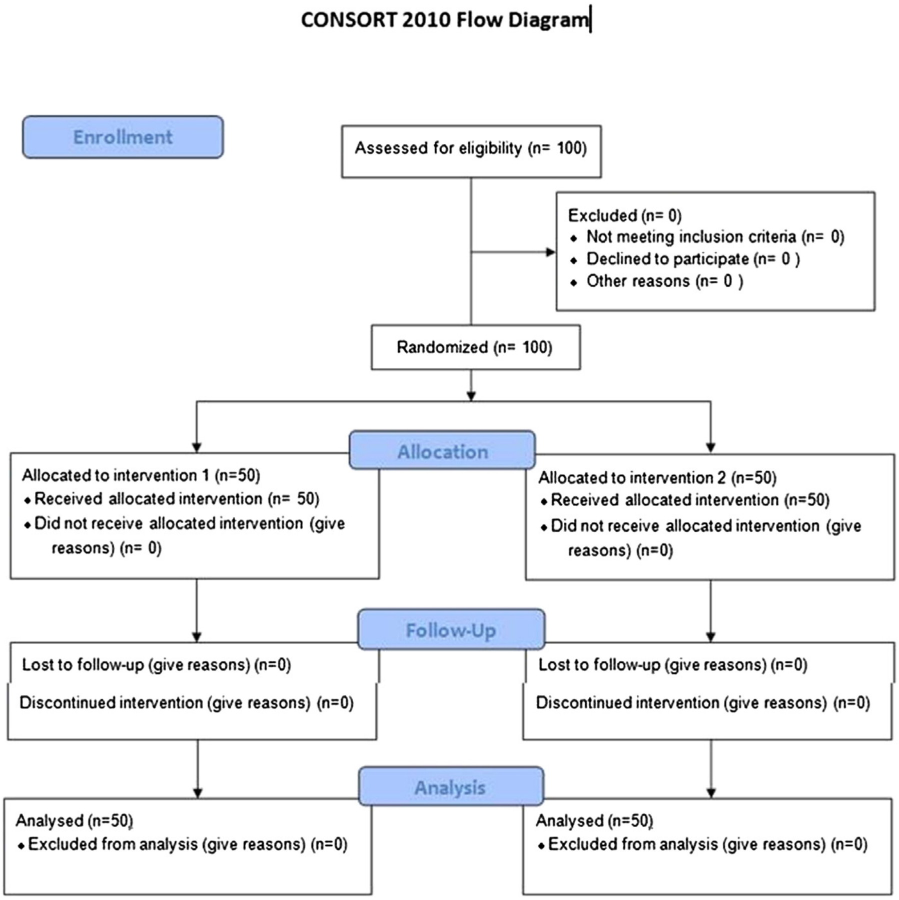

Fig. 4 – Consort statement for study.

findings, as well as to standardize the protocols for performance of these procedures in our population.

In our study there was no statistically significant difference between the two groups, meaning that both the procedures were definitely beneficial to the patients. However the NCMT method did show slightly better results. The cumbersomeness of the culture method as well as the delay in transplantation i.e. transplantation occurring 3 weeks after the donor graft was taken, could be the reasons behind the inferior results.

The donor:recipient area in the NCMT group was 1:10, i.e. 1 cm^2 of normal skin was sufficient to cover about 10 cm^2 of vitiliginous skin. The donor:recipient area in the CMT group was 1:100, i.e. 1 cm^2 of normal skin was sufficient to cover about 100 cm^2 of vitiliginous skin. This was a distinct advantage in the CMT method as compared to the NCMT method. However CMT method was more time consuming, i.e. it took about 3 weeks for culturing autologous melanocytes. Also culturing melanocytes is a labour intensive process and requires state of the art equipments and a sterile lab setup.

Limitations of NCMT and CMT

- Both techniques require an equipped laboratory setup with trained manpower.
- Taking an epidermal graft requires expertise.
- The pathogenesis of vitiligo is still poorly understood, so the stability of vitiligo and reactivation of disease activity after any surgical technique cannot be predicted.
- Autologous melanocyte transfer techniques are state of the art novel surgeries in vitiligo and the results obtained indicate that the procedures can be valuable in motivated patients, when the extent of vitiligo does not exceed 30% of the total body surface area, and when the disease is stable.

Conclusion

In patients with stable vitiligo, autologous melanocyte transfer is a simple and effective technique to produce homogeneous pigmentation quickly. Cultured melanocyte technique is time consuming and labour intensive technique which requires a sophisticated laboratory setup for cell culture, though it can cover vitiliginous areas 100 times the donor area. It is therefore suitable to cover large body surface areas. It has an advantage over conventional split thickness grafting as it requires very little donor skin. We propose that though the difference between the two procedures were not statistically significant, the non culture method is simpler, less time consuming and requires less technical expertise. Hence NCMT should be the first choice technique when it comes to not so widespread cases of vitiligo. When area involved is large, and in institutions where technical expertise and trained manpower are available, the culture method can be undertaken. Patients were generally satisfied with the results to both methods, as the quality of repigmentation was superior to other surgical techniques. Further large scale patient studies are required, especially with melanocyte culture methods, to confirm the efficacy of autologous melanocyte transfer techniques. The patients also need to be followed up for a longer duration to note the long term complications and the status of repigmentation after a period of time.

Intellectual contribution of Authors

Study concept: Col Rajesh Verma, Col Manas Chatterjee, Brig RS Grewal, VSM, Sqn Ldr Debdeep Mitra.

Drafting & manuscript revision: Col Rajesh Verma, Lt Col Biju Vasudevan, Lt Col Vijendran Pragasam, Sqn Ldr Debdeep Mitra.

Statistical analysis: Lt Col Vijendran Pragasam, Sqn Ldr Debdeep Mitra.

Study supervision: Brig RS Grewal, VSM, Col Rajesh Verma, Col Manas Chatterjee, Lt Col Biju Vasudevan.

Conflicts of interest

This study has been funded by research grants from O/o DGAFMS.

REFERENCES

1. Parsad D, Gupta S. Standard guidelines of care for vitiligo surgery. *Indian J Dermatol Venereol Leprol*. 2008;74:37–45.
2. Lontz W, Olsson MJ, Moellmann G, Lerner AB. Pigment cell transplantation for the treatment of vitiligo: a progress report. *J Am Acad Dermatol*. 1994;30:591–597.
3. Savant SS. Surgical therapy of vitiligo: current status. *Indian J Dermatol Venereol Leprol*. 2005;71:307–310.
4. Njoo MD, Westerhof W, Bos JD, Bossuyt PMM. A systemic review of autologous transplantation methods in vitiligo. *Arch Dermatol*. 1998;134:1543–1549.
5. Gauthier Y, Benzekri L. Non-cultured epidermal suspension in vitiligo: from laboratory to clinic. *Indian J Dermatol Venereol Leprol*. 2012;78:59–63.
6. Olsson MJ, Juhlin L. Leukoderma treated by transplantation of basal cell layer enriched suspension. *Br J Dermatol*. 1998;138:644–648.
7. Pandya V, Parmar KS, Shah BJ, Bilimoria FE. A study of autologous melanocyte transfer in treatment of stable vitiligo. *Indian J Dermatol Venereol Leprol*. 2005;71:393–397.
8. Olsson MJ, Juhlin L. Repigmentation of vitiligo by transplantation of cultured autologous melanocytes. *Acta Derm Venereal (Stockholm)*. 1973;73:49–51.
9. Lerner AB, Halaban R, Leffell D. Melanocyte culture in patients with disorders of hypopigmentation. In: *Programme Abstracts of 14th International Pigment Cell Conference*. Japan: The International Pigment Cell Soc; 1990:100.
10. Chen YF, Chang JS, Yang PY, Hung CM, Huang MH, Hu DN. Transplantation of cultured autologous pure melanocytes after laser-abrasion for the treatment of segmental vitiligo. *J Dermatol*. 2000;27:434–439.
11. Schulz KF, Altman DG, Moher D. CONSORT 2010 statement: updated guidelines for reporting parallel group randomised trials. *BMJ*. 2010;340:c332.
